# Targeting the *PARP10*-*BCAT2* axis disrupts branched-chain amino-acid metabolism to suppress bone metastasis in lung cancer

**DOI:** 10.1038/s41419-026-08989-3

**Published:** 2026-06-19

**Authors:** Yahan Qin, Ke Xue, Yujian Xu, Hongyu Pan, Wenjun Chai, Lei Sun, Xiaoli Liu, Jing Li, Yue Cao, Jing Li, Qian Liu, Mingxia Yan

**Affiliations:** 1 Cancer Institute, Fudan University Shanghai Cancer Center, Shanghai, China; 2 Department of Oncology, Shanghai Medical College, Fudan University, Shanghai, China; 3 Department of Oncology, Huashan Hospital Fudan University, Shanghai, China; 4 Department of Respiratory Medicine, Xinhua Hospital, School of Medicine, Shanghai Jiao Tong University, Shanghai, China

**Keywords:** Bone metastases, Cancer microenvironment, Non-small-cell lung cancer, DNA damage and repair

## Abstract

Lung cancer bone metastasis presents a major clinical challenge due to therapeutic resistance and severe morbidity. Although disrupting tumor-bone microenvironment crosstalk is a promising strategy, clinically actionable targets remain limited. Here, by analyzing bulk RNA sequencing data from bone metastatic tumors across multiple cancer types, we identified *PARP10* as a gene consistently upregulated in bone metastases. High *PARP10* expression in primary tumors was correlated with poor patient survival. Functional studies demonstrated that PARP10 promoted lung cancer growth and bone metastasis both in vitro and in vivo. Mechanistically, multi-omics integrated analyses revealed that *PARP10* deletion induced DNA damage and oxidative stress, and upregulated BCAT2 expression in a MYC-dependent manner to enhance BCAA catabolism. This metabolism exerts an adaptive compensatory effect on tumor cells via boosting mitochondrial oxidative phosphorylation, yet depletes bone microenvironmental BCAA and consequently suppresses osteoclast differentiation, thereby inhibiting bone metastasis. Importantly, pharmacological inhibition of PARP10 with OUL232 mitigated bone metastatic burden in mice without observable toxicity, demonstrating its therapeutic potential by concurrently inducing tumor cell apoptosis and disrupting the pro-metastatic niche. Our findings establish PARP10 as a central regulator of a targetable metabolic competition axis and propose its inhibition as a dual-mechanism strategy that simultaneously attacks tumor cells and disrupts the pro-metastatic niche.

## Introduction

Lung cancer ranks among the most prevalent malignancies globally and is the leading cause of cancer-related mortality worldwide, posing a significant threat to human health [1]. Lung adenocarcinoma (LUAD) is the most common subtype, accounting for approximately half of all lung cancer cases. Bone metastasis is a frequent and severe complication in advanced stages of LUAD [2]. Once bone metastasis occurs, the 5-year survival rate drops to <10% [3, 4]. Current treatments, including localized radiotherapy, surgery, systemic chemotherapy, targeted therapy, and immunotherapy, can improve patients’ quality of life to some extent, but the prognosis remains poor [5–8]. Therefore, elucidating the mechanisms of bone metastasis in LUAD and identifying novel therapeutic targets are urgent research priorities.

The development of poly (ADP-ribose) polymerase (PARP) inhibitors marks a major advance in cancer therapy. PARPs are a family of multifunctional enzymes that mediate post-translational modifications, with core roles in DNA damage repair and apoptotic regulation [9]. Among the 17-member PARP family, *PARP1*, *PARP2*, and *PARP3* are primarily involved in DNA damage repair [10], whereas others like *PARP10*, *PARP5A*, and *PARP5B* are associated with cellular metabolism and energy homeostasis [11–13]. PARP inhibitors are the first class of clinically successful drugs based on “synthetic lethality” [14]. Leveraging this mechanism, these inhibitors are particularly effective against tumor cells with pre-existing homologous recombination repair (HRR) deficiencies caused by mutations in genes such as *BRCA1/2*, *ATM*, or *PALB2* [15–18]. Conventional PARP inhibitors primarily target *PARP1/2*, and have been clinically approved for the treatment of multiple malignancies, including ovarian, breast, prostate, and pancreatic cancers. However, most lung cancers, particularly non-small cell lung cancer (NSCLC), are not driven by germline HRR gene mutations [19]. Consequently, the absence of such baseline HRR deficiencies renders the majority of lung cancers insensitive to conventional PARP inhibitor monotherapy [20].

Unlike *PARP1/2*, which catalyze poly-ADP-ribosylation, *PARP10* primarily catalyzes mono-ADP-ribosylation [21]. This distinct catalytic activity differentiates PARP10 functionally from most PARP family members. Studies show that PARP10 regulates mitochondrial function [22] and is aberrantly expressed in various cancers, promoting tumor cell proliferation, migration, and invasion, thus representing a potential biomarker and therapeutic target [23–26]. As the understanding of the biological functions of PARP10 has deepened, the iterative development of selective PARP10 inhibitors has advanced gradually [27–30]. In 2023, a highly selective PARP10 inhibitor OUL232 was developed [31], enhancing clinical translation prospects.

This study examined the potential of PARP10 inhibition in treating LUAD bone metastasis. We found that genetic or pharmacological targeting of PARP10 significantly suppressed LUAD bone metastasis. Our findings suggest a new mechanism whereby PARP10 inhibition suppresses osteoclast differentiation by remodeling branched-chain amino acid (BCAA) metabolism in the bone microenvironment, thereby highlighting a potential therapeutic strategy for managing LUAD bone metastasis.

## Materials and methods

### Patients

Seventy-two paired tumor and adjacent non-tumor tissues from patients with LUAD were included in this study. Patient characteristics are summarized in Table S1. All samples were obtained from Huashan Hospital Affiliated to Fudan University. Informed consent was obtained from all subjects, and all the samples were anonymized. This study was conducted in strict accordance with the Declaration of Helsinki, and approved by the Ethics Committee of Huashan Hospital Affiliated to Fudan University (approval No. 2022-634).

### Cell culture

The HEK293T (RRID:CVCL_0063), RAW264.7 (RRID:CVCL_0493), A549 (RRID:CVCL_0023), MC3T3 (RRID:CVCL_0D74) and PC9 (RRID:CVCL_XA18) cells were purchased from American Type Culture Collection. Osteoclast Precursor Cells (OCPs) and Bone Marrow Mesenchymal Stem Cells (BMSCs) were isolated from 6- to 8-week-old male C57BL/6 mice. The BM5 cell line was established by our laboratory. HEK293T and RAW264.7 cell lines were cultured in Dulbecco’s Modified Eagle Medium (DMEM; Cat. No. F211316, YuanPeiBio, Shanghai Yuanpei Biotechnology Co., Ltd., Shanghai, China) supplemented with 10% (v/v) fetal bovine serum (FBS; Cat. No. 40130ES76, Yeasen Biotechnology Co., Ltd., Shanghai, China) and 1% (v/v) penicillin–streptomycin solution (final concentration: 100 U/mL penicillin, 100 μg/mL streptomycin; Cat. No. S110JV, YuanPeiBio). A549, PC9, and BM5 cell lines were cultured in RPMI-1640 Medium (Cat. No. C211314, YuanPeiBio) supplemented with 10% (v/v) FBS and 1% (v/v) penicillin–streptomycin solution. MC3T3-E1 cell line, primary OCPs and BMSCs were cultured in Minimum Essential Medium Alpha (α-MEM; Cat. No. D211303, YuanPeiBio) supplemented with 10% (v/v) FBS and 1% (v/v) penicillin-streptomycin solution. All cell lines were validated by STR profiling and tested negative for mycoplasma. Cells were cultured in a humidified incubator at 37 °C and 5% CO_2_. All commercial and in-house cell lines were authenticated via short tandem repeat (STR) profiling within 6 months before the study initiation, and were routinely tested for mycoplasma contamination throughout the study using a Mycoplasma Detection Kit (Cat. No. 40612ES, Yeasen Biotechnology Co., Ltd., Shanghai, China). Only mycoplasma-negative cell lines were used for all experiments.

### Real-time quantitative PCR

Total RNA was extracted from cultured cells and clinical tissue specimens using a Total RNA Extraction Kit (Cat. No. RC112-01, Vazyme Biotech Co., Ltd., Nanjing, China) following the manufacturer’s instructions. RNA concentration and purity were assessed using a NanoPhotometer® N60 spectrophotometer (Implen GmbH, Munich, Germany). Only RNA samples with an A260/A280 ratio of 1.8–2.1 (indicating high purity) were used for subsequent reverse transcription. One microgram of qualified total RNA was reverse-transcribed into complementary DNA (cDNA) using the HiScript® III RT SuperMix for qPCR (Cat. No. R323-01, Vazyme). RT-qPCR amplification was performed on an Eppendorf Mastercycler® ep realplex Real-Time PCR System (Eppendorf AG, Hamburg, Germany) using Hieff® qPCR SYBR Green Master Mix (Cat. No. 11202ES03, Yeasen Biotechnology). The amplification reaction conditions were as follows: initial denaturation at 95 °C for 30 s, followed by 40 cycles of denaturation at 95 °C for 10 s and annealing/extension at 60 °C for 30 s. The primer sequences used are listed in Table S2. The relative expression level of each target gene was normalized to that of the endogenous reference gene ACTB (β-actin). Relative gene expression was calculated using the comparative 2^−ΔΔCt^ method. All samples were analyzed in technical triplicates.

### Lentiviral production and transduction

Recombinant lentiviruses encoding human *PARP10* (Gene ID: 84975) overexpression cassette, *PARP10*-specific short hairpin RNA (shRNA), and corresponding negative controls (empty vector for overexpression, non-targeting scrambled shRNA for knockdown) were constructed and purchased from Hongxun Biotechnology Co., Ltd. (Nanjing, China). For viral transduction, cells were seeded in six-well plates at a density of 2 × 10⁵ cells per well. At 30–50% confluence, the medium was replaced with fresh complete medium containing lentiviral particles at a multiplicity of infection (MOI) of 20, supplemented with 1 µl/mL Polybrene (Cat. No. TR-1003, Sigma-Aldrich, St. Louis, MO, USA). After 48 h of incubation, the virus-containing medium was aspirated and replaced with fresh complete medium. Cells were selected by culturing in complete medium supplemented with 1 μg/ml Puromycin dihydrochloride (Cat. No. CNSBR0089, Sigma-Aldrich) for 7 days to eliminate non-transduced cells. The knockdown or overexpression efficiency of the *PARP10* gene was confirmed at both the mRNA and protein levels. Target sequences of shRNAs used are listed in Table S3.

### Western blotting

Total protein was extracted from treated cells, mouse bone tissues and human lung tissues using Pierce™ RIPA Lysis Buffer (Cat. No. 89900, Thermo Fisher Scientific, Waltham, MA, USA) supplemented with Phosphatase Inhibitor Cocktail (Cat. No. 20109ES05, Yeasen Biotechnology Co., Ltd., Shanghai, China) and Protease Inhibitor Cocktail (Cat. No. 20123ES50, Yeasen Biotechnology). Protein concentrations were determined with the BCA Protein Assay Kit (Cat. No. BCA1, Sigma-Aldrich, St. Louis, MO, USA). Equal amounts of protein (20–40 μg per lane) were separated by SDS-PAGE (10–15% gel) and transferred onto PVDF Membrane (Cat. No. IPVH00010, Merck Millipore, Burlington, MA, USA). The membranes were blocked with 5% (w/v) non-fat skim milk (Cat. No. P0216, Beyotime Biotechnology Co., Ltd., Shanghai, China) in PBST for 1 h at room temperature and then incubated overnight at 4 °C with primary antibodies against. The details of the antibodies used are provided in Table S4. After washing, the membranes were incubated with HRP-conjugated secondary antibodies (anti-rabbit/mouse IgG; 1:5000 dilution) for 1 h at room temperature. Protein bands were visualized using a High-Sensitivity ECL Chemiluminescence Substrate (Cat. No. 7E1050I4, SunBio Biotechnology Co., Ltd., Shanghai, China). Images were acquired using the Tanon-5200 Imaging System with standard resolution settings and its accompanying software. After chemiluminescence detection of the target protein, membranes were stripped using Antibody Stripping Buffer (Cat. No. P0025, Beyotime Biotechnology) following the manufacturer’s instructions. Stripped membranes were then re-blocked, re-probed with the corresponding primary and secondary antibodies, and visualized via chemiluminescence as described above. Band intensities were quantified using ImageJ software (RRID:SCR_003070).

### Co-immunoprecipitation assay

Co-IP assays were performed to validate protein-protein interactions. Briefly, cells in logarithmic growth phase were lysed with ice-cold cell lysis buffer (Cat. No. P0013, Beyotime Biotechnology) supplemented with 1% protease and phosphatase inhibitor cocktail (Cat. No. P1045, Beyotime Biotechnology). Lysates were transferred to EP tubes and centrifuged at 12,000 rpm for 15 min at 4 °C, and the soluble supernatant was collected for total protein quantification via BCA assay. For each reaction, equal amounts of total protein were incubated with the corresponding target primary antibody or species-matched isotype normal IgG control overnight at 4 °C with constant rotation. On the following day, 30 μL of Protein A/G Magnetic Beads were added to each system, followed by an additional 3 h incubation at 4 °C to capture immune complexes. The beads were washed five times with ice-cold lysis buffer, and bound proteins were eluted by boiling in 1× SDS loading buffer at 100 °C for 10 min. Eluted samples and input controls were analyzed by western blot as described previously.

### Cell viability and cytotoxicity assay

Cell viability and the cytotoxic effects of paclitaxel (Cat. No. HY-B0015, MedChemExpress, Monmouth Junction, NJ, USA), camptothecin (Cat. No. SC0141, Beyotime Biotechnology), and the PARP10 inhibitor OUL232 (Cat. No. E1614, Selleck Chemicals, Houston, TX, USA) on A549, BM5, and PC9 cells were determined using the Cell Counting Kit-8 (CCK-8; Cat. No. C7408050, Yeasen Biotechnology) following the manufacturer’s instructions. Cells were seeded into 96-well plates at a density of 800 cells/well (for cell viability assay) or 2000 cells/well (for cytotoxicity assay) in 100 μL of complete medium, and cultured overnight to allow cell attachment. The next day, cells were treated with the drug or vehicle control. Cell viability was measured at 24 h intervals for 5 consecutive days, while cytotoxicity was measured at 24 h and 48 h post-treatment. At each detection time point, the culture medium was aspirated, and 10 μL of CCK-8 reagent plus 90 μL of fresh serum-free medium were added to each well. Plates were incubated at 37 °C for 2 h in the dark to allow formazan formation. The absorbance (optical density, OD) at 450 nm was measured using an ELx808 Microplate Reader (BioTek Instruments Inc., Winooski, VT, USA; RRID: SCR_018053). The viability of treated cells was calculated as a percentage relative to the control group (set as 100%) using the following formula:

$$\mathrm{Cell\; Viability}\,( \% )=[\mathrm{OD}(\mathrm{treatment})-\mathrm{OD}(\mathrm{blank})]/[\mathrm{OD}(\mathrm{control})-\mathrm{OD}(\mathrm{blank})]\times 100$$

Cytotoxicity was assessed by the half-maximal inhibitory concentration (IC₅₀) values after 24 h or 48 h of exposure. All experiments were performed in triplicate and repeated at least three times independently.

### Conditioned medium collection

Cells at 90% confluence in 10 cm dishes were washed twice with PBS, then the medium was replaced with 3 mL of serum-free medium and incubated for 24 h at 37 °C. The filtered medium was then concentrated ~10-fold using Amicon® Ultra-15 Centrifugal Filter Units (3 kDa molecular weight cutoff, MWCO; Cat. No. UFC900324, Merck Millipore, Burlington, MA, USA) via centrifugation at 8000 × *g* for 15 min at 4 °C. The retentate was collected as the protein-rich conditioned medium, and the filtrate was collected as the protein-depleted and metabolite-rich conditioned medium.

### Migration and invasion assays

A total of 5 × 10⁴ cells in serum-free medium were seeded into the upper chamber of a 24-well Transwell insert (Cat. No. 353097, Corning Inc., Corning, NY, USA). The lower chamber was filled with 800 μL of complete medium containing 10% FBS as a chemoattractant. After incubation for 15–24 h at 37 °C, the non-migrated cells on the upper surface were removed with a cotton swab. The migrated cells on the lower membrane were fixed with methanol, stained with 0.1% crystal violet, and imaged. The number of migrated cells was counted in at least nine random fields per well under a light microscope. For the chemotaxis assay evaluating the effect of BCAA, the medium in the lower chamber was replaced with serum-free medium supplemented with 400 μM total BCAA (L-leucine, Cat. No. ST1461; L-isoleucine, Cat. No. ST1486; L-valine, Cat. No. ST1502; all from Beyotime Biotechnology Co., Ltd., Shanghai, China). After incubation for 18 h at 37 °C, the medium in the upper chamber was aspirated, and non-migrated cells on the upper membrane surface were removed with a sterile cotton swab. The lower chamber was then supplemented with 10% serum, and cells remaining on the upper membrane were incubated for an additional 6 h to facilitate adhesion. For BMSCs, RAW264.7 and MC3T3 cells recruitment assays, the medium in the lower chamber was replaced with the corresponding conditioned medium. For the invasion assay, the procedure was similar to the migration assay, except that the Transwell membranes were pre-coated with Matrigel (Cat. No. 354480, Corning Inc). A total of 1 × 10⁵ cells were seeded into the Matrigel-coated upper chamber and incubated for 36 h at 37 °C. Subsequent fixation, staining, and counting steps were performed as described for the migration assay.

### Wound healing assay

Cells were seeded into six-well plates and cultured until they reached 90–100% confluence. A sterile 200 μL pipette tip was used to create a straight scratch wound across the cell monolayer. The detached cells were removed by washing twice with PBS. The cells were then incubated in low-serum (1% FBS) medium to minimize the impact of cell proliferation. Images of the same wound locations were captured at 0 h and 24 h. The relative wound width or the percentage of wound closure was quantified using ImageJ. Each experiment was performed in triplicate and repeated at least three times independently.

### RNA s**equencing**

#### RNA isolation and library preparation

Total RNA was extracted using the TRIzol reagent (Invitrogen, CA, USA) according to the manufacturer’s protocol. RNA purity and quantification were evaluated using the NanoDrop 2000 spectrophotometer (Thermo Scientific, USA). RNA integrity was assessed using the Agilent 2100 Bioanalyzer (Agilent Technologies, Santa Clara, CA, USA). Then the libraries were constructed using VAHTS Universal V6 RNA-seq Library Prep Kit according to the manufacturer’s instructions. The transcriptome sequencing and analysis were conducted by OE Biotech Co., Ltd. (Shanghai, China).

#### mRNA sequencing analysis process

Libraries were sequenced on an Illumina NovaSeq 6000 Platform, and 150 bp paired-end reads were generated. Approximately 7.2 Gb of raw reads were obtained per sample. Raw reads of fastq format were first processed using fastp, and the low-quality reads were removed to obtain the clean reads. Then, ~7.0 Gb clean reads for each sample were retained for subsequent analyses. The clean reads were mapped to the reference genome using HISAT2 (RRID:SCR_015530). FPKM of each gene was calculated, and the read counts of each gene were obtained by HTSeq-count (RRID:SCR_011867). Principal component analysis (PCA) was performed using R (v 3.2.0) to assess the reproducibility of biological replicates and the overall transcriptomic variation between groups. Differential gene expression analysis was performed using DESeq2 (RRID:SCR_000154). Genes with log₂ fold change (FC) ≥ 2 and adjusted *P* value (*Q* value) < 0.05 were defined as significantly differentially expressed genes (DEGs). Hierarchical clustering analysis of DEGs was performed using R (version 3.2.0) to visualize the expression patterns of DEGs across different groups and samples. A radar plot of the top 30 most significantly dysregulated DEGs was generated using the R package ggradar. Gene Ontology (GO), Kyoto Encyclopedia of Genes and Genomes (KEGG; http://www.genome.jp/kegg/; RRID:SCR_012773), Reactome (RRID:SCR_003487), and WikiPathways (RRID:SCR_002134) functional enrichment analyses of DEGs were performed based on the hypergeometric test using R (version 3.2.0). Terms with adjusted P-value < 0.05 were defined as significantly enriched. Bar plots, chord diagrams, and bubble plots of significantly enriched terms were visualized using R (version 3.2.0). Gene set enrichment analysis (GSEA) was performed using GSEA software with predefined gene sets from the Molecular Signatures Database (MSigDB). Genes were ranked by the signal-to-noise ratio based on their expression levels between the two groups, and permutation testing (*n* = 1000 permutations) was performed to determine whether the predefined gene set was significantly enriched at the top or bottom of the ranked gene list. Gene sets with normalized enrichment score (NES) ≥ 1.5, and adjusted *P* value < 0.05 were defined as significantly enriched.

### **Metabolomic profiling**

#### Sample preparation

An aliquot of each sample was treated with 1 mL of pre-chilled methanol-water (4:1, v/v) containing a mixture of internal standards (4 μg/mL). The solution was transferred in two steps to a 1.5 mL EP tube. Then, 200 μL of chloroform was added, and the mixture was homogenized by repeated pipetting. Samples were subjected to ice-bath sonication using a high-power ultrasonic disruptor (1620 W, 10 min; pulse cycle: 6 s on, 4 s off), followed by a second round of sonication in an ice-water bath for 20 min for complete metabolite extraction. The mixture was then incubated at −40 °C for 2 h to facilitate protein precipitation. After centrifugation at 13,000 rpm for 10 min at 4 °C, 800 μL of the supernatant was transferred to an LC-MS vial and evaporated to dryness under a gentle stream of nitrogen. The dried residue was reconstituted in 300 μL of methanol:water (1:4, v/v), vortexed for 30 s, and sonicated in an ice-water bath for 3 min. The solution was incubated at −40 °C for 2 h, then subjected to a second centrifugation step at 13,000 rpm for 10 min at 4 °C. A total of 150 μL of the supernatant was transferred to a fresh LC-MS vial and stored at −80 °C until LC-tandem mass spectrometry (LC-MS/MS) analysis. Quality control (QC) samples were prepared by pooling equal aliquots of all experimental samples.

#### LC-MS/MS analysis

Untargeted metabolomic data acquisition and bioinformatic analysis were performed by Shanghai Luming Biotechnology Co., Ltd. (Shanghai, China). Chromatographic separation was performed on an ACQUITY UPLC I-Class Plus System (Waters Corporation, Milford, MA, USA). Mass spectrometric detection was performed on a Q Exactive Hybrid Quadrupole-Orbitrap Mass Spectrometer equipped with a heated electrospray ionization (HESI) source (Thermo Fisher Scientific, Waltham, MA, USA). Metabolic profiling was acquired in both positive and negative HESI ion modes. An ACQUITY UPLC HSS T3 column (1.8 μm particle size, 2.1 mm × 100 mm; Waters Corporation) was used for chromatographic separation in both ion modes. The binary gradient elution system consisted of mobile phase A (water containing 0.1% (v/v) formic acid) and mobile phase B (acetonitrile containing 0.1% (v/v) formic acid). The gradient elution program was set as follows: 0.01 min, 5% B; 2 min, 5% B; 4 min, 30% B; 8 min, 50% B; 10 min, 80% B; 14 min, 100% B; 15 min, 100% B; 15.1 min, 5% B; 16 min, 5% B. The flow rate was set at 0.35 mL/min, the column temperature was maintained at 45 °C, and the sample manager temperature was set at 10 °C. The injection volume was 2 μL per sample.

The full MS scan range was set at m/z 100–1000. The resolution was set at 70,000 for full MS scans and 17,500 for higher-energy collisional dissociation (HCD) MS/MS scans. The collision energy was set at 10, 20, and 40 eV. The mass spectrometer parameters were set as follows: spray voltage, 3800 V (positive mode) and 3200 V (negative mode); sheath gas flow rate, 35 arbitrary units (a.u.); auxiliary gas flow rate, 8 a.u.; capillary temperature, 320 °C; auxiliary gas heater temperature, 350 °C; S-lens RF level, 50.

#### Data preprocessing and statistical analysis

Raw LC-MS/MS data were processed using Progenesis QI software (version 2.3; Nonlinear Dynamics, Newcastle, UK; RRID:SCR_014978) for baseline filtering, peak picking, integration, retention time correction, peak alignment, and normalization. The core parameters were set as follows: 5 ppm precursor tolerance, 10 ppm product tolerance, and 5% product-ion threshold. Metabolite identification was performed based on the accurate mass-to-charge ratio (m/z), MS/MS fragmentation patterns, and isotopic distribution, using the Human Metabolome Database (HMDB), LIPID MAPS (version 2.3), METLIN, and an in-house standard database. The preprocessed data were further filtered by: (1) removing peaks with missing values (ion intensity = 0) in more than 50% of samples per group; (2) replacing zero values with 1/2 of the minimum positive value in the dataset; (3) removing metabolites with identification scores <36 (out of 60) to ensure identification accuracy. A final data matrix was generated by combining data from positive and negative ion modes. The normalized data matrix was imported into R for PCA to visualize the overall metabolic variation between samples and the stability of the analytical process. Orthogonal partial least squares discriminant analysis (OPLS-DA) and partial least squares discriminant analysis (PLS-DA) were performed to identify differential metabolites between groups. To prevent model overfitting, sevenfold cross-validation and 200 permutation tests were performed to evaluate the goodness of fit and predictive ability of the model. Variable Importance in Projection (VIP) values from the OPLS-DA model were used to rank the contribution of each metabolite to group discrimination. A two-tailed Student’s *t* test was performed to assess the statistical significance of metabolite differences between groups. Metabolites with VIP > 1.0 and *P* value < 0.05 were defined as significantly differential metabolites. KEGG pathway enrichment analysis of differential metabolites was performed using the hypergeometric test, based on the official KEGG database (http://www.genome.jp/kegg/).

#### Mice and housing conditions

Female BALB/c nude mice (RRID: MGI:2683685; 7 weeks of age) were purchased from GemPharmatech Co., Ltd. (Nanjing, Jiangsu, China). All mice were housed under specific pathogen-free (SPF) conditions in individually ventilated cages (IVCs), with a 12 h/12 h light/dark cycle, controlled ambient temperature (22 ± 2 °C), and relative humidity (50% ± 10%). Sterile food and water were provided ad libitum throughout the study. The sample size was determined based on previously published literature and the animal welfare 3 R principles (Reduction, Replacement, Refinement), with no formal statistical power analysis performed. Pre-specified inclusion and exclusion criteria were defined before study initiation, and no mice were excluded from the final data analysis. Blinding was not performed during treatment administration, whereas outcome assessment via bioluminescence imaging was conducted in a fully blinded manner. Prior to model establishment and treatment allocation, mice were randomized into experimental groups using a computer-generated random number table. To establish the lung cancer bone metastasis model, mice were inoculated with 1 × 10⁶ BM5-Luc tumor cells suspended in 100 μL sterile PBS via left ventricular intracardiac injection. Following inoculation, mice were treated with the PARP10 inhibitor OUL232 (200 μL per mouse) via intraperitoneal injection every 2 days. Metastatic progression was monitored via in vivo bioluminescence imaging (BLI) at the 4-week time point post-inoculation. All animal experimental procedures were conducted in strict accordance with the ARRIVE 2.0 guidelines and approved by the Institutional Animal Care and Use Committee (IACUC) of Fudan University Shanghai Cancer Center (approval No. FUSCC-IACUC-2025087).

#### In vivo bioluminescence imaging

BLI was performed at the 4-week time point post-inoculation to monitor bone metastatic progression. Prior to imaging, mice were anesthetized and maintained with 3% isoflurane during image acquisition. Each mouse received an intraperitoneal injection of 200 μL D-Luciferin potassium salt (Cat. No. 40902ES08, Yeasen Biotechnology Co., Ltd., Shanghai, China) at a dose of 3 mg per mouse. Imaging was initiated 5 min after substrate injection using an IVIS SpectrumCT In Vivo Imaging System (PerkinElmer). Image acquisition parameters were set as follows: exposure time 1–60 s (automatically adjusted based on signal intensity), f/stop 1, and medium binning. Luminescent signals were quantified as radiance (photons/s/cm²/sr) by drawing uniform circular regions of interest (ROIs) around each metastatic lesion using Living Image software (version 4.7.4; PerkinElmer Inc.; RRID: SCR_014247).

#### Micro-computed tomography scans and analyses

Bilateral tibiofibular complexes were carefully dissected from euthanized mice, and soft tissue was completely removed. Bone specimens were fixed in Formalin for 24-48 hours and then stored at 4 °C until scanning. Fixed bone specimens were scanned using a SkyScan 1172 high-resolution micro-CT system (Bruker microCT, Kontich, Belgium; RRID: SCR_014316). Three-dimensional images were reconstructed from the 2D X-ray projections by implementing the Feldkamp algorithm using the commercial software package NRecon software (2.0.4.0 SkyScan). CTVox and CTAn software (1.13 SkyScan) were used for the 3D morphometric analyses of images. The grayscale threshold for bone segmentation was determined visually and kept identical for all samples within the study. The following core bone microarchitectural parameters were quantified: bone volume/total volume ratio (BV/TV, %), trabecular number (Tb.N, mm⁻¹), cross-sectional thickness (Cs.Th, μm), and mean polar moment of inertia (MMI, mm⁴).

### Hematoxylin and eosin staining

Bilateral tibiofibular complexes were dissected, fixed in 10% NBF, decalcified in 10% EDTA decalcification solution, and embedded in paraffin. H&E staining was performed on 4 μm-thick paraffin sections by Wuhan Servicebio Technology Co., Ltd. (Wuhan, Hubei, China) following a standard automated histology protocol. Stained slides were digitally scanned at ×40 magnification using an Aperio AT2 Digital Whole Slide Scanner (Leica Biosystems, Wetzlar, Germany; RRID: SCR_020031), and the resulting whole-slide images were used for histological evaluation of bone metastatic lesions.

### Comet assay

Cellular DNA damage was assessed using an Alkaline Comet Assay Kit (Cat. No. C2041M, Beyotime Institute of Biotechnology, Haimen, Jiangsu, China) following the manufacturer’s instructions. Briefly, treated cells were embedded in low-melting-point agarose on frosted microscope slides. After cell lysis at 4 °C for 2 h, DNA was denatured in pre-chilled alkaline electrophoresis buffer (300 mM NaOH, 1 mM EDTA, pH > 13) for 30 min at 4 °C in the dark, then electrophoresed at 25 V (300 mA) for 25 min at 4 °C in the dark. Slides were neutralized with Tris-HCl buffer (pH 7.4), stained with propidium iodide (PI; 10 μg/mL), and imaged using an inverted fluorescence microscope (Olympus Corporation, Tokyo, Japan). The percentage of tail DNA was quantified for at least 50 randomly selected cells per group using CASP software.

### ROS measurements

Intracellular ROS production was detected using the Reactive Oxygen Species Assay Kit (Cat. No. S0034S, Beyotime Biotechnology Co., Ltd., Shanghai, China) following the manufacturer’s instructions. Briefly, after the designated treatments, cells were incubated with 10 μM 2’,7’-dichlorodihydrofluorescein diacetate (DCFH-DA) in serum-free medium at 37 °C for 20 min in the dark, then rinsed three times with sterile PBS to completely remove the extracellular probe. Where indicated, cells were treated with Rosup (50 μg/mL, provided with the kit) for 30 min as a positive control. Fluorescence images were captured using an inverted fluorescence microscope with a FITC filter set (Olympus Corporation). The mean fluorescence intensity was quantified from at least five randomly selected high-power fields per group using ImageJ software.

### Mitochondrial membrane potential assay

Mitochondrial membrane potential (MMP) was assessed using the JC-1 Mitochondrial Membrane Potential Assay Kit (Cat. No. C2006, Beyotime Biotechnology) following the manufacturer’s instructions. Briefly, cells were seeded into 6-well plates and received the designated treatments. After treatment, cells were washed twice with pre-warmed PBS, then incubated with JC-1 working solution at 37 °C for 20 min in the dark. After staining, cells were washed twice with pre-chilled JC-1 staining buffer. Fluorescence images were immediately captured using an inverted fluorescence microscope (Olympus Corporation). The ratio of red fluorescence (excitation/emission: 525 nm/590 nm) to green fluorescence (excitation/emission: 490 nm/530 nm) was calculated to quantify MMP. A decrease in the red/green fluorescence ratio indicates mitochondrial depolarization.

### GSH and NADH measurements

Intracellular levels of reduced glutathione (GSH) and oxidized glutathione (GSSG) were measured using a GSH and GSSG Assay Kit (Cat. No. S0053, Beyotime Biotechnology Co., Ltd., Shanghai, China) following the manufacturer’s instructions. Briefly, 1 × 10⁶ treated cells were washed once with pre-chilled PBS, collected by centrifugation, and the supernatant was completely removed. Three volumes of protein precipitation reagent relative to the cell pellet volume were added, followed by thorough vortexing. Samples were subjected to two rapid freeze-thaw cycles using liquid nitrogen and a 37 °C water bath, then incubated on ice for 5 min. After centrifugation at 10,000 × *g* for 10 min at 4 °C, the supernatant was collected for total glutathione quantification.

The intracellular NADH/NAD⁺ ratio was determined using an NADH/NAD⁺ Assay Kit (Cat. No. S0175, Beyotime Biotechnology) following the manufacturer’s instructions. Briefly, 2 × 10⁶ treated cells were extracted with the specific extraction buffer provided with the kit. Half of the extract was heated at 60 °C for 30 min to decompose NAD⁺, then cooled on ice and centrifuged to obtain the supernatant for NADH measurement. The other half of the extract was kept on ice without heating for total NAD⁺/NADH measurement. The enzymatic cycling reaction was initiated by adding the kit working solution to the samples. After incubation at 37 °C for 30 min, the absorbance at 450 nm was measured using a microplate reader (BioTek Instruments Inc.). The concentrations of NADH and total NAD⁺/NADH were calculated from a standard curve generated in parallel. The NAD⁺ concentration was determined by subtracting the NADH concentration from the total NAD⁺/NADH concentration.

### BCAA assay

The concentrations of BCAA in cell culture supernatants were determined using a BCAA Assay Kit with WST-8 (Cat. No. S0535S, Beyotime Biotechnology Co., Ltd., Shanghai, China) following the manufacturer’s instructions. Equal volumes of supernatant and kit working solution were mixed and incubated at 37 °C for 30 min. The absorbance at 450 nm was measured using a Microplate Reader, consistent with previous assays. A standard curve was generated using the L-valine standard provided with the kit (0–1000 μM) to calculate the absolute BCAA concentration in each sample. Data from at least three independent experiments are presented as mean ± standard deviation (SD).

### Differentiation of osteoclasts and osteoblasts

Primary OCPs and BMSCs were isolated under sterile conditions from the femurs and tibiae of 7–9-week-old male C57BL/6 mice (RRID: IMSR_JAX:000664). Bone marrow was flushed out from the bone cavity into a 1.5 mL sterile microcentrifuge tube, then centrifuged at 10,000 × *g* for 5 s at 4 °C to collect bone marrow cells. Red blood cells were lysed and removed using Red Blood Cell Lysis Buffer (Cat. No. C3702, Beyotime Biotechnology) following the manufacturer’s instructions. The cell pellet was resuspended in complete α-MEM medium and incubated for 24 h. Adherent cells were primarily BMSCs, while the non-adherent cell suspension was collected for OCP isolation and differentiation. The suspension was collected and centrifuged at 850 rpm for 3 min at 4 °C, and the cell pellet was resuspended in complete α-MEM supplemented with 50 ng/mL recombinant mouse M-CSF (Cat. No. 416-ML-010, R&D Systems, Bio-Techne, Minneapolis, MN, USA) for 6 days of expansion culture. After 6 days of expansion, monocytes were washed, trypsinized, resuspended, counted, and seeded into 96-well plates at a density of 1 × 10⁴ cells per well in 200 μL of complete α-MEM. Osteoclast differentiation was induced by treatment with 25 ng/mL mouse M-CSF and 25 ng/mL recombinant mouse RANKL (Cat. No. 462-TEC-010, R&D Systems, Bio-Techne) for 6 days. The medium was replaced with fresh differentiation medium every 3 days. Cells were imaged under bright-field illumination using an inverted light microscope at ×40 and ×100 magnification for subsequent quantitative analysis. Only TRAP-positive (TRAP⁺, purple-red), multinucleated (≥3 nuclei), vacuolated cells with a scalloped edge and diameter ≥100 μm were defined as mature osteoclasts and quantified.

BMSCs and MC3T3-E1 cells were seeded into 6-well plates. Once cells reached 70–80% confluence, the medium was replaced with osteogenic induction DMEM supplemented with 10 mM β-glycerophosphate disodium salt (Cat. No. 50020, Sigma-Aldrich, St. Louis, MO, USA), 50 ng/mL L-ascorbic acid ( > 99%, Cat. No. ST1434, Beyotime Biotechnology), and 10 nM dexamethasone (Cat. No. D4902, Sigma-Aldrich). The osteogenic induction medium was changed every 3 days throughout the induction period. Alkaline phosphatase (ALP) staining was performed on day 7 of induction, and ALP activity was assessed using the BCIP/NBT Alkaline Phosphatase Color Development Kit (Cat. No. C3206, Beyotime Biotechnology) following the manufacturer’s instructions. Alizarin Red S staining for mineralized nodule formation was performed on day 21 of induction using the Alizarin Red S Staining Kit for Osteogenesis (Cat. No. C0148S, Beyotime Biotechnology) following the manufacturer’s instructions.

### TRAP staining

TRAP staining of cultured osteoclasts was performed using a TRAP/ALP Stain Kit (Cat. No. 294-67001, Fujifilm Wako Pure Chemical Industries, Ltd., Osaka, Japan) following the manufacturer’s instructions with minor modifications. Cells were fixed with 4% paraformaldehyde in PBS for 10 min at room temperature. After rinsing with PBS, samples were incubated in freshly prepared TRAP staining solution at 37 °C for 30–60 min in the dark until mature osteoclasts were stained purple-red. Samples were then counterstained with methyl green for 15 min at room temperature to visualize nuclei.

TRAP staining of osteoclasts in decalcified bone sections was performed by Wuhan Servicebio Technology Co., Ltd. (Wuhan, Hubei, China) following a standard automated histology protocol. Briefly, fixed tibiofibular complexes were decalcified, embedded in paraffin, and sectioned at 4 μm thickness for staining. Stained slides were digitally scanned at ×40 magnification using an Aperio AT2 Digital Whole Slide Scanner, and the resulting whole-slide images were used for histological evaluation.

### Cell mitochondrial function and glycolysis assay

Real-time measurement of the oxygen consumption rate (OCR) and extracellular acidification rate (ECAR) was performed using a Seahorse XFe96 Extracellular Flux Analyzer (Agilent Technologies) with the Seahorse XF Mito Stress Test Kit (Cat. No. 103015-100, Agilent) and Glycolysis Stress Test Kit (Cat. No. 103020-100, Agilent), following the manufacturer’s instructions. Tumor cells with corresponding gene interventions were seeded in XF96 microplates. Assay medium was prepared with indicated substrates (pH 7.4, pre-warmed to 37 °C), and the sensor cartridge was hydrated and calibrated prior to detection. Cells were washed and equilibrated in assay medium at 37 °C in a non-CO₂ incubator for 60 min before the assay. Compounds were injected sequentially at optimized final concentrations: 1.5 μM oligomycin, 1.5 μM FCCP, and 0.5 μM rotenone + antimycin A for the Mito Stress Test; 10 mM glucose, 2.0 μM oligomycin, and 50 mM 2-deoxy-D-glucose (2-DG) for the Glycolysis Stress Test. OCR and ECAR were recorded continuously throughout the assay. Key parameters of mitochondrial respiration and glycolysis were analyzed using Seahorse Wave Desktop Software. All experiments were repeated independently three times.

### Dual-luciferase reporter assay

Endo-free recombinant reporter plasmids were extracted using the Endo-Free Plasmid Mini/Midi Kit (Cat. No. DP118, Tiangen Biotech Co., Ltd., Beijing, China) following the manufacturer’s instructions. Tumor cells were seeded into 24-well plates, and transfection was performed when cells reached ~80% confluence using ExFect 2000 Transfection Reagent (Cat. No. T202-01, Vazyme Biotech) following the manufacturer’s instructions. At 24 h post-transfection, cells were harvested and lysed using the Dual Luciferase Reporter Assay Kit (Cat. No. DL101-01, Vazyme Biotech). Firefly and Renilla luciferase activities were measured sequentially using a GloMax 20/20 Luminometer (Promega). Relative firefly luciferase activity was normalized to the corresponding Renilla luciferase activity for each well. All assays were repeated in three independent biological replicates.

### Chromatin immunoprecipitation coupled with quantitative PCR (ChIP-qPCR) assay

Cells were cultured in DMEM (Gibco, Thermo Fisher Scientific) supplemented with 10% FBS (Cytiva, Hyclone) at 37 °C with 5% (v/v) CO₂, and passaged at ~90% confluence. Cells were fixed with 1% formaldehyde for 10 min at room temperature to crosslink protein-DNA complexes, and crosslinking was terminated with glycine. After washing twice with pre-chilled PBS, cells were scraped and collected by centrifugation. Cell pellets were resuspended in Buffer A (supplemented with DTT, protease inhibitor cocktail, and PMSF), incubated on ice for 10 min, and centrifuged to collect nuclear pellets. Nuclei were resuspended in Buffer B, treated with micrococcal nuclease at 37 °C for 20 min, and the reaction was terminated with EDTA. After centrifugation, pellets were resuspended in ChIP buffer, and chromatin was further sheared using a Covaris S220 Focused-ultrasonicator (2 s on, 2 s off, 2 cycles, 7% power). The supernatant containing sheared chromatin was collected after centrifugation at 12,000 × *g* for 10 min at 4 °C. The sheared chromatin was diluted with ChIP buffer, with 1% of each sample reserved as the input control. For each immunoprecipitation (IP) reaction, diluted chromatin was incubated with anti-MYC antibody or species-matched normal rabbit IgG control at 4 °C overnight with constant end-over-end rotation, followed by incubation with 25 μL of pre-blocked Protein G Magnetic Beads for an additional 2 h at 4 °C. The beads were washed three times with low-salt wash buffer, and once with high-salt wash buffer, then the bound chromatin was eluted using ChIP elution buffer. Reverse crosslinking was performed at 65 °C for 30 min, followed by proteinase K treatment at 65 °C for 2 h to degrade residual proteins. Immunoprecipitated DNA was purified using a Universal DNA Purification Kit. Quantitative PCR was performed using specific primers targeting the human BCAT2 promoter, and the relative enrichment of target DNA fragments was calculated using the % input method. All assays were repeated in three independent biological replicates.

### Statistical analysis

All statistical analyses were performed using GraphPad Prism 9.0 software (RRID: SCR_002798). A two-tailed *P* value < 0.05 was considered statistically significant. The normality of data distribution was assessed using the Shapiro–Wilk test, and homogeneity of variances was assessed using Bartlett’s test. For normally distributed data with equal variances, comparisons between two groups were performed using an unpaired two-tailed Student’s *t* test, and comparisons among multiple groups were performed using one-way analysis of variance (ANOVA) with Tukey’s post-hoc test for multiple comparisons. For non-normally distributed data, comparisons between two groups were performed using the Mann–Whitney *U* test. Data are presented as mean ± SD, with the number of biological replicates indicated in each figure legend.

## Results

### High expression of PARP10 in lung cancer tissues and the bone microenvironment and its negative correlation with prognosis

**Fig. 1:**
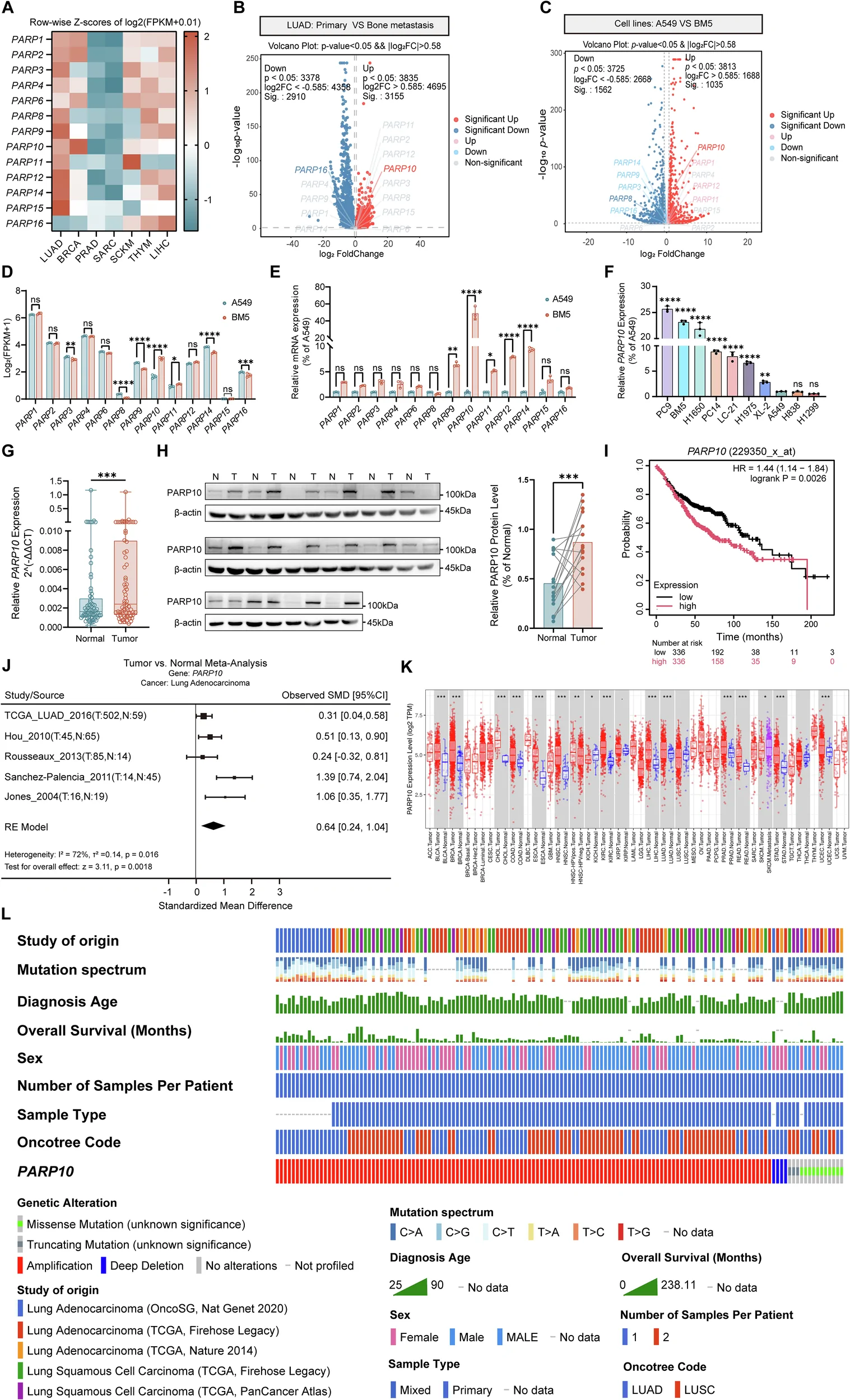
PARP10 is upregulated in LUAD bone metastases and correlates with poor patient prognosis. **A** Heatmap showing the expression profile of PARP family genes in bulk RNA-seq data from bone metastatic lesions of different solid tumor types. Expression values are presented as row-wise Z-scores of log₂(FPKM + 0.01). Tumor types: LUAD (*n* = 1), BRCA (*n* = 1), PRAD (*n* = 1), SKCM (*n* = 1), SARC (*n* = 1), THYM (*n* = 1), LIHC (*n* = 1). Data were obtained from GEO datasets GSE273023 and GSE274442. Color scale: red, high expression; green, low expression. **B** Expression of PARP family genes in bulk RNA sequencing data from primary lung cancer tissues (*n* = 19) and bone metastasis tissues (*n* = 8) (fold change >1.5, *P* < 0.05). GEO dataset: GSE225212. **C**, **D** Comparative transcriptomic analysis of PARP family genes between parental A549 cells and highly bone-metastatic BM5 cells (*n* = 3 biological replicates per group). **C** Volcano plot of differentially expressed PARP genes (fold change >1.5, *P* < 0.05). **D** log₂(FPKM + 1) values of PARP family genes. **E** RT-qPCR analysis of PARP family genes mRNA expression in A549 and BM5 cells. *n* = 3 independent experiments. **F** RT-qPCR analysis of *PARP10* mRNA expression in multiple LUAD cell lines, normalized to A549 cells. *n* = 3 independent experiments. **G** RT-qPCR analysis of *PARP10* mRNA expression in 72 paired LUAD tumor tissues and matched adjacent non-tumor lung tissues. Each dot represents an individual patient sample. **H** Western blotting analysis of PARP10 protein expression in 12 paired LUAD tumor tissues and matched adjacent non-tumor lung tissues. **I** Kaplan–Meier overall survival (OS) analysis of LUAD patients stratified by *PARP10* expression (*n* = 672). Data were obtained from the Kaplan–Meier Plotter database. **J** Meta-analysis of *PARP10* expression in normal lung tissues (*n* = 202) versus LUAD tissues (*n* = 662). Data were obtained from the Lung Cancer Explorer database, including five independent datasets. **K** Pan-cancer analysis of *PARP10* expression in tumor tissues (red) and matched normal tissues (blue) from TCGA. Expression values are presented as log₂(TPM + 1). Each dot represents an individual sample. Analysis was performed using the TIMER2.0 web server. **L** Waterfall plot showing the *PARP10* genomic alteration profile across five TCGA NSCLC cohorts (*n* = 142 tumor samples). Each column represents an individual sample; each row represents a genetic feature. Mutation types are color-coded as indicated in the legend. Data are presented as mean ± SD. Statistical significance was determined by unpaired two-tailed Student’s *t* test (**D**, **E**), one-way ANOVA with Dunnett’s post-hoc test (**F**), or paired two-tailed Student’s *t* test (**G**, **H**). *P < 0.05, **P < 0.01, ***P < 0.001, ****P < 0.0001.

To explore the potential role of PARP family genes in tumor-bone metastasis, we first analyzed their expression across various types of bone metastatic lesions. The results showed a global upregulation trend of PARP family genes in LUAD, breast invasive carcinoma (BRCA), skin cutaneous melanoma (SKCM), thymoma (THYM), and liver hepatocellular carcinoma (LIHC), whereas a downregulation trend was observed in prostate adenocarcinoma (PRAD) and sarcoma (SARC) (Fig. 1A). Given the limited sample size of the pan-cancer dataset, we utilized bulk RNA-seq data from 27 primary lung cancer samples and bone metastasis samples for further analysis. The results showed that *PARP10* was significantly upregulated PARP family member in bone metastatic lesions, while *PARP16* was significantly downregulated (Fig. 1B). Based on a highly bone-metastatic LUAD mouse model established via five rounds of in vivo selection in our previous study, we derived a highly bone-metastatic LUAD cell line (BM5) from the low-metastatic parental A549 cell line. Transcriptome sequencing of A549 and BM5 cells was subsequently performed with three biological replicates per group [32]. Transcriptome analysis revealed that *PARP10* was the most significantly upregulated PARP family member in BM5 cells (Fig. 1C, D). RT-qPCR validation of PARP family gene expression in A549 and BM5 cells further confirmed the significant upregulation of PARP10 in BM5 cells (Fig. 1E). Consistent with sequencing data, PARP10 expression was significantly higher in BM5 cells than in A549 cells (Fig. 1F). Together, these data suggest a positive correlation between elevated *PARP10* expression and bone metastatic potential in LUAD. To assess the clinical relevance of *PARP10*, we collected 72 pairs of lung cancer and adjacent normal lung tissues to detect *PARP10* expression levels. The results showed significantly elevated *PARP10* mRNA levels in tumor tissues (Fig. 1G). Similarly, PARP10 protein expression was markedly higher in cancerous tissues compared to normal tissues (Fig. 1H). Furthermore, Kaplan–Meier survival analysis revealed that high *PARP10* expression was associated with shorter overall survival and poorer prognosis in patients (Fig. 1I). A meta-analysis of a total of 864 samples from different datasets indicated that *PARP10* expression is upregulated in LUAD compared with normal tissue (Fig. 1J). Analysis of the Cancer Genome Atlas (TCGA) database indicated that PARP10 expression is significantly increased not only in LUAD tissues but also in various other cancer types, including bladder cancer, breast cancer, colon cancer, and liver cancer, suggesting a potential broad oncogenic role for PARP10 (Fig. 1K). Further analysis of TCGA NSCLC RNA-seq data revealed that ~87% of *PARP10* genomic alterations were gene amplification events, with similar amplification frequencies observed in LUAD and lung squamous cell carcinoma (LUSC) (Fig. 1L). This provides supporting evidence for the oncogenic function of PARP10 in tumor development. In summary, these findings indicate that elevated *PARP10* expression is a feature of LUAD, correlates with poor patient prognosis, and suggests its involvement in LUAD bone metastasis.

### PARP10 promotes LUAD tumorigenesis and bone metastasis in vivo

**Fig. 2:**
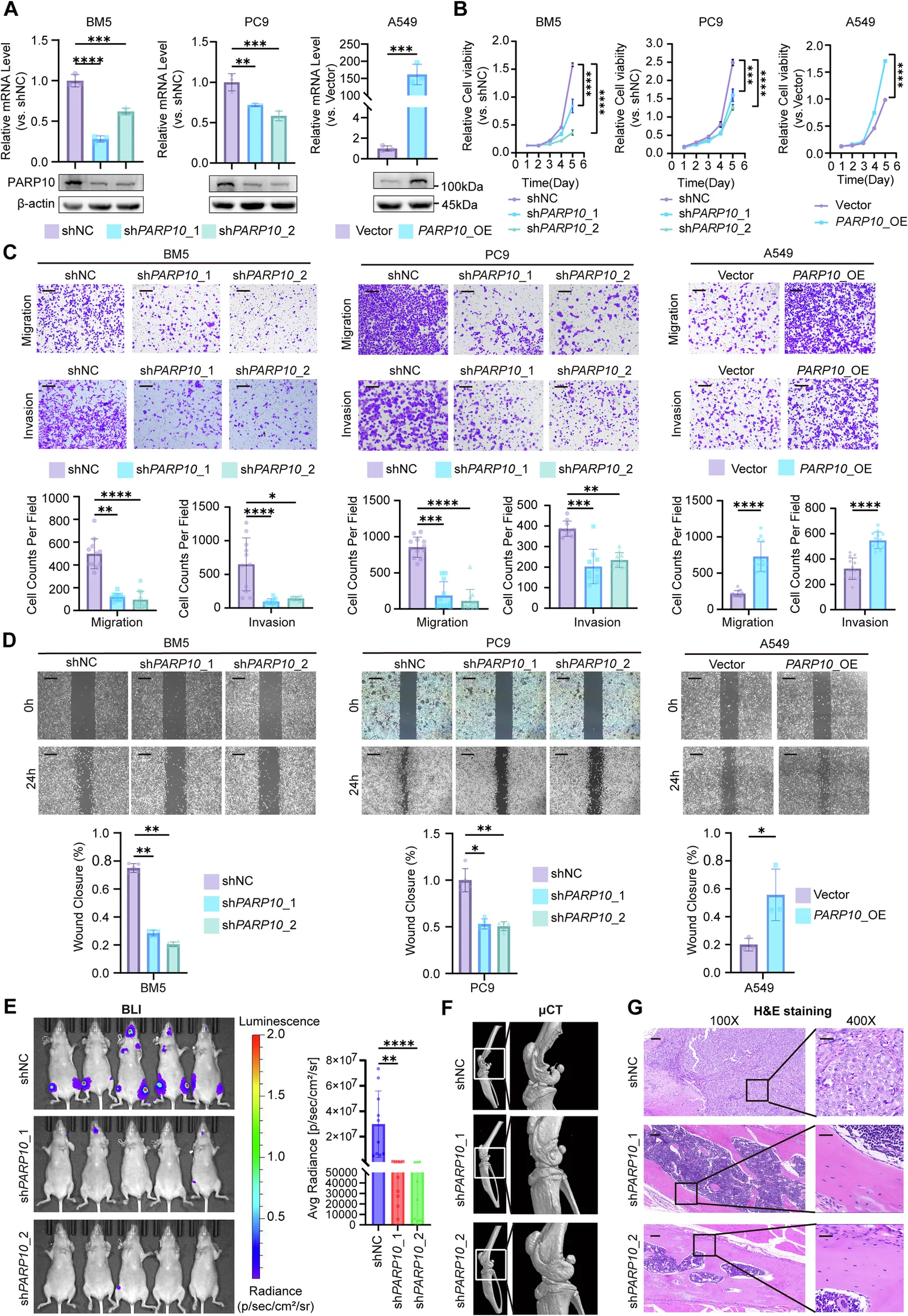
*PARP10* promotes LUAD tumorigenesis and bone metastasis in vivo. **A** Validation of *PARP10* knockdown (BM5, PC9) and overexpression (A549) by RT-qPCR and Western blotting. *n* = 3 independent experiments. **B** CCK-8 assay showing cell proliferation over 5 days in *PARP10*-knockdown (BM5, PC9) and *PARP10*-overexpressing (A549) cells. *n* = 3 independent experiments. **C** Transwell migration and Matrigel invasion assays in *PARP10*-knockdown (PC9, BM5) and *PARP10*-overexpressing (A549) cells. *n* = 9–13 random fields per group. Scale bar, 200 μm. **D** Wound healing assays in *PARP10*-knockdown (PC9, BM5) and *PARP10*-overexpressing (A549) cells at 0 and 24 h post-wounding. Wound closure was quantified using ImageJ software and expressed as a percentage of wound closure. *n* = 5 independent experiments. Scale bar, 500 μm. **E**–**G** In vivo functional validation of PARP10 in bone metastasis. Bone metastasis models were established by left ventricular intracardiac injection of BM5 cells transduced with PARP10 shRNA (sh*PARP10*) or control shRNA (shNC). Mice were sacrificed 4 weeks post inoculation. **E** Representative bioluminescence imaging (BLI) showing metastatic lesions. Signal intensity is expressed as average radiance (p/sec/cm²/sr). *n* = 10 mice per group. **F** Representative 3D reconstructed micro-CT images of femurs and tibiae. **G** Representative H&E staining showing bone structure and intraosseous tumor lesions. Scale bars: 100 μm (×100 magnification), 40 μm (×400 magnification). Data are presented as mean ± SD. Statistical significance was determined by one-way ANOVA with Dunnett’s post-hoc test for multiple comparisons to the control group, or unpaired two-tailed Student’s *t* test for comparisons between two groups. *P < 0.05, **P < 0.01, ***P < 0.001, ****P < 0.0001.

To investigate the functional role of PARP10 in LUAD, we established stable *PARP10*-knockdown cell lines using PC9 and BM5 cells, and generated a stable *PARP10*-overexpressing cell line using A549 cells (Fig. 2A). *PARP10* knockdown suppressed cell proliferation (Fig. 2B), as well as migratory and invasive capacities, whereas *PARP10* overexpression enhanced these malignant phenotypes (Fig. 2C, D). These results indicate that PARP10 promotes the proliferation and metastatic potential of LUAD cells. We next evaluated the effect of PARP10 on bone metastasis using the left ventricular intracardiac injection bone metastasis model. In vivo bioluminescence imaging (BLI) at 4 weeks post-inoculation revealed that both the incidence of bone metastasis and the luminescent signal intensity of metastatic lesions were significantly reduced in the *PARP10*-knockdown group compared with the control group (Fig. 2E). Subsequent micro-CT and H&E staining of dissected femora and tibiae revealed that *PARP10* knockdown markedly reduced osteolytic bone destruction and tumor burden (Fig. 2F, G). Collectively, these findings suggest that *PARP10* knockdown impairs LUAD bone metastasis and mitigates osteolytic bone destruction in vivo.

### PARP10 deficiency inhibits osteoclast differentiation via enhancing BCAA catabolism in LUAD cells

**Fig. 3:**
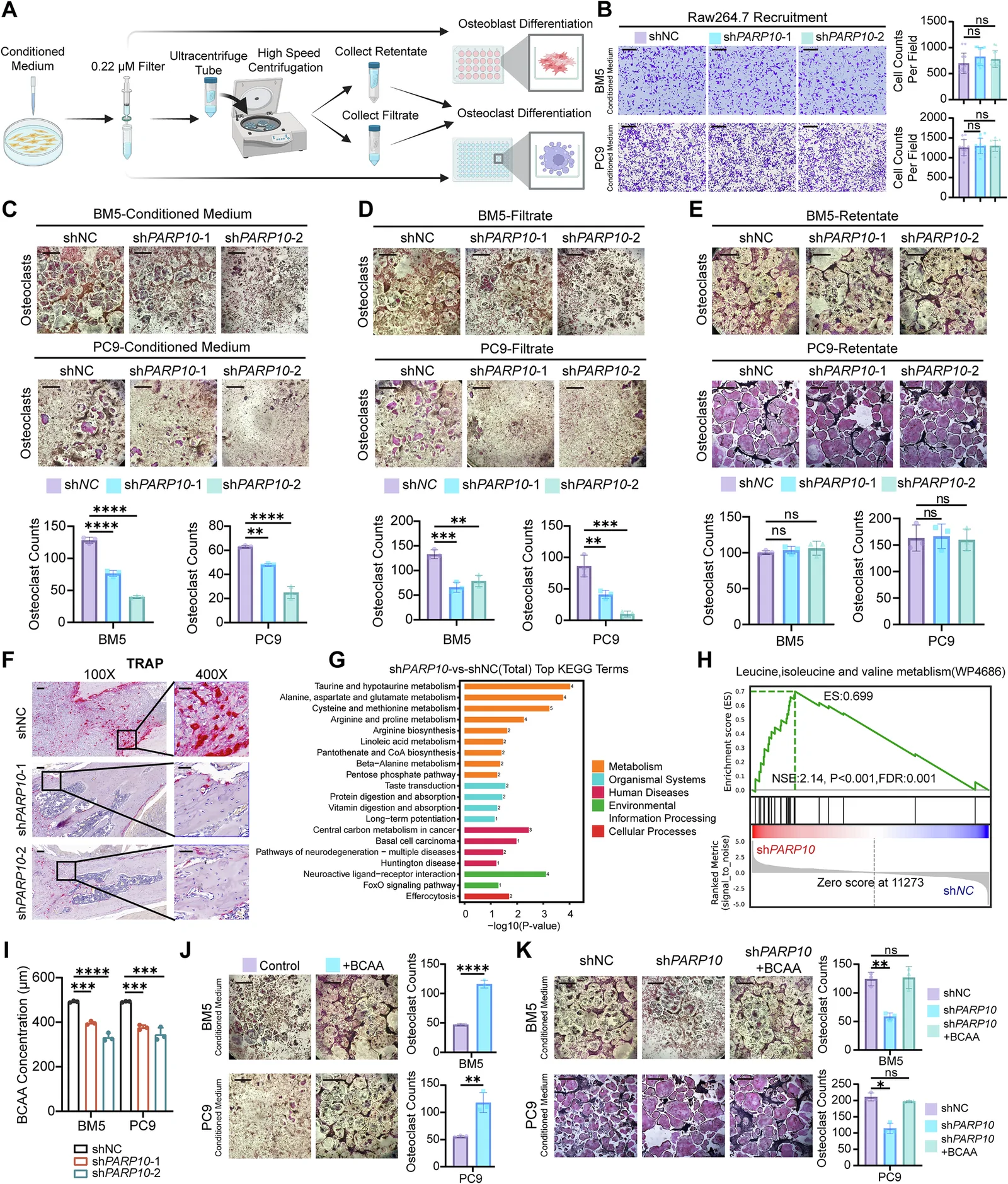
PARP10 deficiency inhibits osteoclast differentiation via enhancing BCAA catabolism in LUAD cells. **A** Schematic showing the collection of serum-free conditioned medium (CM) from control and *PARP10*-knockdown LUAD cells and the subsequent experimental design. **B**–**E** Osteoclast recruitment and differentiation assays using CM from control and *PARP10*-knockdown BM5 and PC9 cells. **B** RAW264.7 cell recruitment assay. *n* = 9–13 random fields per group. **C** Primary mouse OCP differentiation assay. *n* = 3 independent experiments. **D** OCP differentiation assay using protein-free metabolite-rich CM (ultrafiltrate fraction). *n* = 3 independent experiments. **E** OCP differentiation assay using protein-rich concentrated CM (retentate fraction). *n* = 3 independent experiments. Scale bar, 200 μm. **F** TRAP staining of hind limb tibiofibular complexes isolated from mice 4 weeks after intracardiac injection of control or PARP10-knockdown BM5 cells. Scale bars: 100 μm (×100 magnification), 40 μm (×400 magnification). **G** KEGG pathway enrichment analysis of differential metabolites in CM from control and *PARP10*-knockdown BM5 cells. **H** GSEA showing significant enrichment of the BCAA catabolic pathway in transcriptomic data from *PARP10*-knockdown BM5 cells (WikiPathways database). **I** BCAA concentration in CM from control and *PARP10*-knockdown BM5 and PC9 cells. *n* = 3 independent experiments. **J** OCP differentiation assay with supplementation of 400 μM BCAA mixture (leucine:isoleucine:valine = 1:1:1). *n* = 3 independent experiments. Scale bar, 200 μm. **K** Rescue assay showing that supplementation of 400 μM BCAA mixture completely reverses the impaired OCP differentiation induced by CM from PARP10-knockdown BM5 and PC9 cells. Scale bar, 200 μm. The schematic diagrams were created using BioRender. Data are presented as mean ± SD. Statistical significance was determined by one-way ANOVA with Tukey’s post-hoc test for multiple comparisons, or unpaired two-tailed Student’s *t* test for comparisons between two groups. *P < 0.05, **P < 0.01, ***P < 0.001, ****P < 0.0001.

To examine how *PARP10* knockdown in tumor cells influences the bone microenvironment, we collected conditioned medium (CM) from control and *PARP10*-knockdown BM5 and PC9 cells (Fig. 3A). Initial cell recruitment assays using MC3T3 pre-osteoblasts, bone marrow-derived mesenchymal stem cells (BMSCs), and RAW264.7 pre-osteoclasts showed no significant differences between the control and PARP10-knockdown groups (Figs. 3B and S1A). These results suggest that *PARP10* knockdown does not alter the recruitment capacity of osteoblast or osteoclast precursors (OCPs). We next performed indirect co-culture experiments, in which primary mouse OCPs and BMSCs were treated with CM from control or PARP10-knockdown LUAD cells. CM from *PARP10*-knockdown cells significantly suppressed osteoclast differentiation (Fig. 3C), but had no significant effect on osteoblast mineralization and maturation (Fig. S1B–E). To identify the key component in CM responsible for inhibiting osteoclastogenesis, we used protein-depleted CM and found that osteoclast differentiation remained suppressed (Fig. 3D), indicating the involvement of metabolites. By contrast, addition of the protein-rich retentate from CM to the culture medium had no significant effect on osteoclast differentiation (Fig. 3E), confirming that metabolites, rather than secreted proteins, might play a dominant role in mediating the inhibitory effect on osteoclastogenesis. Consistent with the in vitro findings, TRAP staining of hind limb tibiofibular complexes from mice inoculated with *PARP10*-knockdown BM5 cells showed fewer mature osteoclasts within metastatic lesions (Fig. 3F). These results suggest that *PARP10* knockdown triggers metabolic reprogramming in tumor cells, which in turn impairs osteoclast differentiation in the bone metastatic microenvironment.

To characterize the metabolic changes induced by *PARP10* knockdown, we performed transcriptomic and metabolomic analyses of LUAD cells with or without *PARP10* knockdown. Metabolomics revealed significant alterations in amino acid metabolism in CM (Fig. 3G), while transcriptomic analysis showed significant enrichment of genes involved in amino acid metabolic pathways, particularly BCAA catabolism (Figs. 3H and S3A, B). Since BCAA (leucine, isoleucine, valine) are known to promote osteoclast differentiation [33], we measured BCAA levels in CM. *PARP10* knockdown led to a reduction in extracellular BCAA levels (Fig. 3I). Although decreased proliferation might be expected to increase extracellular BCAA, the observed reduction suggests enhanced BCAA catabolism in *PARP10*-knockdown cells, implying that BCAA are diverted toward biological processes rather than being used for cell proliferation. We further supplemented osteoclast differentiation medium with a mixture of BCAA, and found that elevated BCAA concentrations significantly promoted osteoclast differentiation (Fig. 3J), consistent with previous published studies [33]. Furthermore, supplementation of BCAA to CM from *PARP10*-knockdown cells completely rescued the impaired osteoclast differentiation (Fig. 3K). Collectively, these data suggest that PARP10 deficiency enhances BCAA catabolism and consumption in LUAD cells, which depletes extracellular BCAA and thereby inhibits osteoclast differentiation.

### PARP10 knockdown enhances BCAA-directed metabolic chemotaxis in LUAD cells

**Fig. 4:**
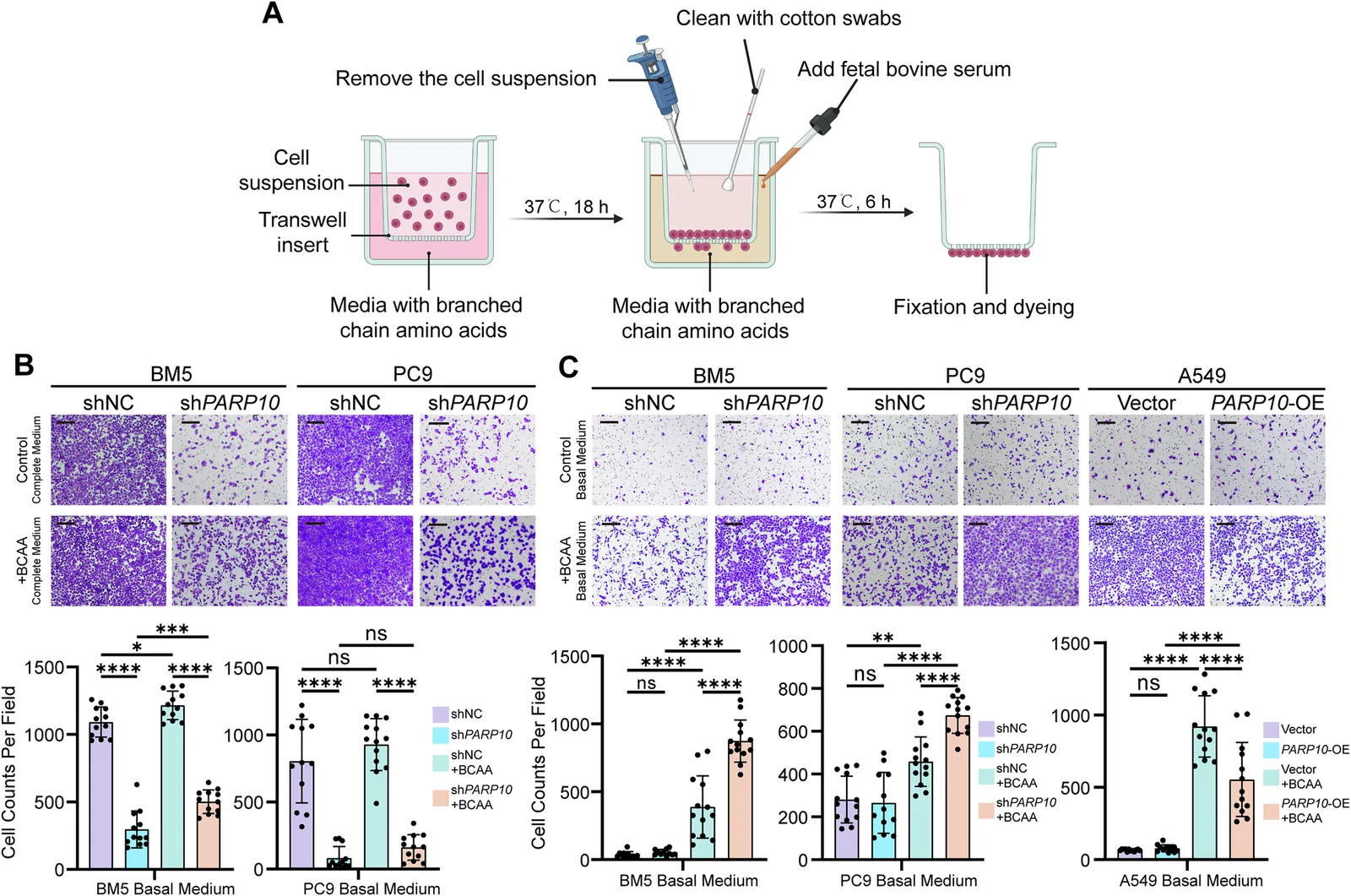
*PARP10* knockdown enhances BCAA-directed metabolic chemotaxis in LUAD cells. Schematic illustrating BCAA-directed metabolic chemotaxis in LUAD cells. **B**, **C** Transwell chemotaxis assays showing the migratory response of *PARP10*-modulated LUAD cells to BCAA. *PARP10*-knockdown (BM5, PC9) or *PARP10*-overexpressing (A549) cells were seeded in the upper chamber, with 400 μM BCAA mixture added exclusively to the lower chamber to establish a concentration gradient. **B** Assays performed with complete medium (10% FBS) in the lower chamber. **C** Assays performed with serum-free basal medium in the lower chamber. *n* = 9–13 random fields per group. Scale bar, 200 μm. The schematic diagrams were created using BioRender. Data are presented as mean ± SD. Statistical significance was determined by one-way ANOVA with Tukey’s post-hoc test, two-way ANOVA with Tukey’s post-hoc test for multi-factor grouped data, or unpaired two-tailed Student’s *t* test for comparisons between two groups. *P < 0.05, **P < 0.01, ***P < 0.001, ****P < 0.0001.

When tumor cells exhibit an increased demand for specific nutrients, they can sense extracellular concentration gradients of metabolites, including amino acids and glucose, and migrate directionally toward nutrient-rich regions, a process defined as metabolic chemotaxis. [34–39]. We therefore investigated whether *PARP10* knockdown enhances the chemotactic response of LUAD cells to BCAA (Fig. 4A). When serum was routinely added to the lower chamber, Transwell assays revealed that although 400 μM BCAAs in the lower chamber increased overall cell migration, *PARP10*-knockdown cells still migrated less than control cells (Fig. 4B). To minimize proliferation-related effects, serum-free basal medium was used in both chambers, with 400 μM BCAA added exclusively to the lower chamber to establish a nutrient concentration gradient. After 18 h of incubation, non-migrated cells on the upper membrane surface were removed, and 10% FBS was added to the lower chamber for 6 h to allow migrated cells on the lower membrane surface to fully adhere, before fixation and staining. In the absence of a BCAA gradient, there was no significant difference in migration between groups. However, when BCAA was added exclusively to the lower chamber, *PARP10*-knockdown cells exhibited significantly enhanced migration compared with control cells (Fig. 4C), consistent with an enhanced chemotactic response toward BCAA. This pro-chemotactic effect was observed for each individual BCAA (leucine, isoleucine, and valine) (Fig. S2A), implying that a shared downstream metabolite of BCAA catabolism may mediate the response. Furthermore, wound healing scratch assays performed in 1% FBS medium with equal, uniform BCAA supplementation showed no significant difference in cell migration between groups (Fig. S2B, C). This confirms that the enhanced migration observed under gradient conditions reflects specific BCAA-directed metabolic chemotaxis, rather than a general increase in cell motility. In summary, these findings establish that *PARP10* knockdown increases the intracellular demand for BCAA in LUAD cells, which drives both BCAA-directed metabolic chemotaxis and depletion of extracellular BCAA in the bone microenvironment, thereby inhibiting osteoclast differentiation. The biological implications of enhanced BCAA metabolism in PARP10-knockdown cells warrant further investigation.

### PARP10 deficiency-induced DNA damage and cycle arrest in cancer and compensatory protection by enhanced BCAA catabolism

**Fig. 5:**
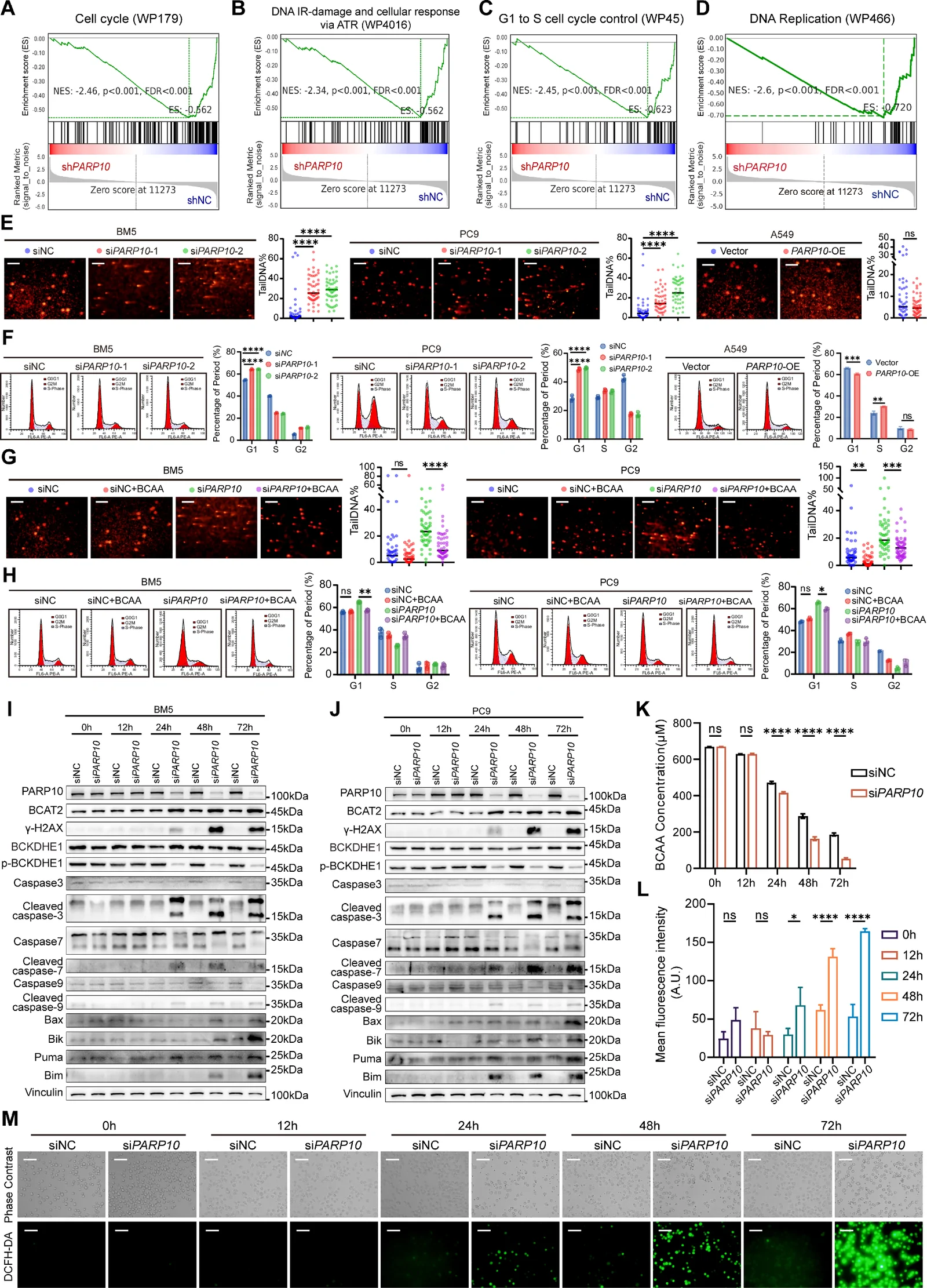
PARP10 deficiency induces DNA damage and cell cycle arrest and compensatory protection by enhanced BCAA catabolism. **A**–**D** GSEA of signaling pathways in transcriptomic data from *PARP10*-knockdown BM5 cells (WikiPathways database). **A** Cell cycle (WP179). **B** DNA IR-damage and cellular response via ATR (WP4016). **C** G1/S phase cell cycle control (WP45). **D** DNA replication (WP466). Normalized enrichment score (NES) and false discovery rate (FDR) are indicated for each plot. **E** Comet assay showing DNA damage (quantified by tail DNA percentage) in *PARP10*-knockdown (BM5, PC9) or *PARP10*-overexpressing (A549) cells. *n* = 50 comets per group. Scale bar, 100 μm. **F** Flow cytometry-based cell cycle analysis in *PARP10*-knockdown (BM5, PC9) or *PARP10*-overexpressing (A549) cells. *n* = 3 independent experiments. **G** Comet assay showing that BCAA supplementation attenuates DNA damage in *PARP10*-knockdown cells. Cells were treated with 400 μM BCAA mixture for 24 h. *n* = 50 comets per group. Scale bar, 100 μm. **H** Flow cytometric cell cycle analysis after *PARP10* modulation and 400 μM BCAA treatment for 24 h. *n* = 3 independent experiments. **I**–**M** Time-series analysis of cellular phenotypes following *PARP10* knockdown. PC9 and BM5 cells were transfected with *PARP10*-specific siRNA and analyzed at 0, 12, 24, 48, and 72 h post-transfection. **I**, **J** Western blotting analysis of γ-H2AX, BCAA catabolic enzymes, and apoptosis-related proteins in BM5 (**I**) and PC9 (**J**) cells. **K** BCAA concentration in cell culture medium. *n* = 3 independent experiments. **L**, **M** Intracellular reactive oxygen species (ROS) levels. **L** Quantification of ROS fluorescence intensity. **M** Representative fluorescence images. *n* = 3 independent experiments. Scale bar, 100 μm. Data are presented as mean ± SD. Statistical significance was determined by one-way ANOVA with Dunnett’s post-hoc test for comparisons to the control group, two-way ANOVA with Tukey’s post-hoc test for multi-factor grouped data, or unpaired two-tailed Student’s *t* test for comparisons between two groups. *P < 0.05, **P < 0.01, ***P < 0.001, ****P < 0.0001.

Previous studies indicate that PARP10 participates in single-strand break repair and homologous recombination [40]. Transcriptomic profiling after *PARP10* knockdown highlighted its association with cell proliferation and cell cycle regulation (Figs. 5A and S3C, E). *PARP10* knockdown downregulated DNA damage repair pathways (Figs. 5B and S3F), arrested cells at the G1-S transition (Figs. 5C and S3C, G), and impaired cell division (Figs. 5D and S3H). We therefore assessed DNA damage and cell cycle distribution following *PARP10* modulation. Comet assay revealed significantly longer tail moments in *PARP10*-knockdown cells, indicating accumulated DNA damage, whereas *PARP10* overexpression reduced tail moments, consistent with enhanced repair capacity (Fig. 5E). Cell cycle analysis showed an increased G1-phase population upon *PARP10* knockdown (Fig. 5F), suggesting G1 arrest due to unresolved DNA damage. Overexpression of *PARP10* decreased the G1 fraction, supporting unimpaired cell cycle progression. These data indicate that PARP10 loss induces DNA damage in tumor cells.

DNA repair is energy-intensive. BCAA catabolism can generate energy and substrates for DNA repair enzymes [41]. Therefore, we hypothesized that the enhanced BCAA catabolism associated with PARP10 deficiency may serve as a compensatory protective mechanism for tumor cells in response to DNA damage. To test this, we cultured *PARP10*-knockdown cells in BCAA-supplemented medium for 24 h. Comet assay showed shorter tails (Fig. 5G), and cell cycle analysis indicated a reduced G1 phase proportion (Fig. 5H). Collectively, these results demonstrate that exogenous BCAA supplementation markedly attenuates DNA damage and G1/S phase cell cycle arrest induced by PARP10 deficiency, suggesting that enhanced BCAA catabolism exerts a compensatory protective effect in PARP10-deficient tumor cells.

DNA damage commonly induces cellular oxidative stress and subsequent apoptosis [42]. To clarify the temporal relationship among DNA damage, BCAA catabolism, oxidative stress, and apoptosis in response to PARP10 deficiency, we transfected LUAD cells with *PARP10*-specific small interfering RNA (siRNA) and assessed dynamic phenotypic changes at sequential time points. Following *PARP10* knockdown, we observed synchronous upregulation of the DNA damage marker γ-H2AX (Fig. 5I, J), increased expression of the BCAA catabolic enzyme BCAT2, reduced levels of inactive phosphorylated BCKDH-E1 (p-BCKDH-E1), and time-dependent depletion of BCAA in the culture medium (Fig. 5K) within 24 h. These results collectively demonstrate that PARP10 deficiency concurrently induces DNA damage and enhances BCAA catabolism in LUAD cells. This was accompanied by a gradual increase in intracellular ROS levels after 24 h (Fig. 5L, M), in parallel with the upregulation of apoptosis-related proteins. At 48–72 h post-knockdown, we observed persistent accumulation of DNA damage, sustained high BCAT2 expression, further robust elevation of ROS and apoptotic proteins, and exacerbated apoptotic cell death. Collectively, these findings establish that PARP10 deficiency synchronously induces DNA damage accumulation and enhances BCAA catabolism in LUAD cells, and that upregulation of BCAA catabolism is not a secondary downstream consequence of DNA damage. The compensatory activation of BCAA metabolism cannot fully counteract persistent DNA damage over time, which ultimately leads to exacerbated oxidative stress and apoptotic cell death in PARP10-deficient tumor cells.

### PARP10 regulates BCAA catabolism via MYC-mediated BCAT2 transcriptional activation

**Fig. 6:**
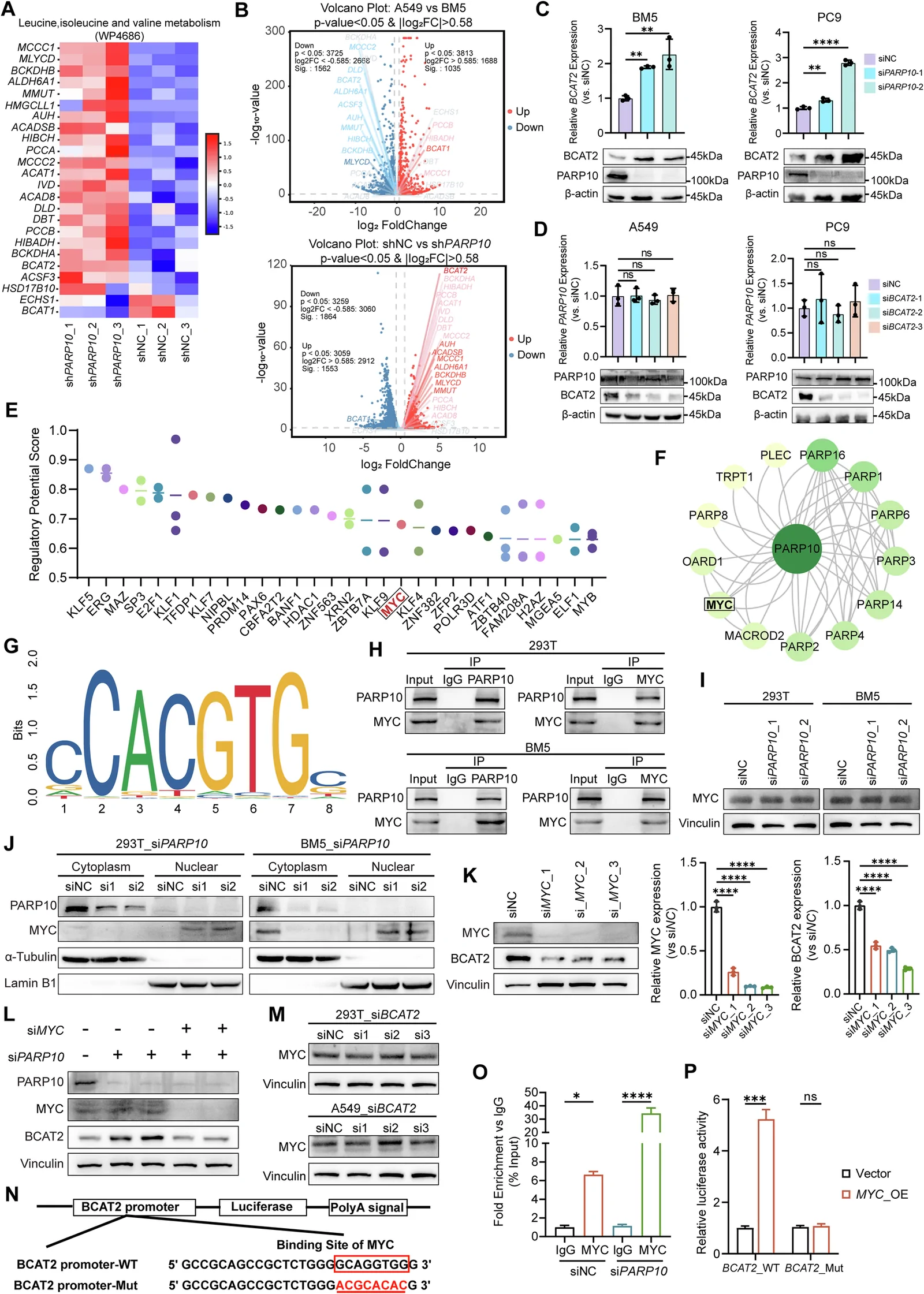
PARP10 regulates BCAA catabolism via MYC-mediated BCAT2 transcriptional activation. **A** Heatmap of gene expression in the Leucine, Isoleucine, and Valine Metabolism pathway (WP4686), showing enriched genes from WikiPathways in *PARP10*-knockdown tumor cells via RNA-seq. **B** Volcano plots showing expression changes of BCAA catabolic genes in RNA-seq data: BM5 vs. parental A549 cells (top), control vs. *PARP10*-knockdown BM5 cells (bottom). **C**. RT-qPCR and Western blotting analysis of BCAT2 expression in control and *PARP10*-knockdown BM5 and PC9 cells. *n* = 3 independent experiments. **D** RT-qPCR and Western blotting analysis of PARP10 expression in control and *BCAT2*-knockdown A549 and PC9 cells. *n* = 3 independent experiments. **E** Prediction of transcription factor binding sites in the human *BCAT2* promoter using the JASPAR database. **F** Prediction of PARP10-interacting proteins using the STRING database. **G** Prediction of MYC binding site in *BCAT2* promoter using the JASPAR database. **H** Co-IP assay validating the endogenous interaction between PARP10 and MYC in 293 T and BM5 cells. **I** Western blotting analysis of total MYC expression in control and *PARP10*-knockdown 293 T and BM5 cells. **J** Nucleocytoplasmic fractionation assay showing MYC subcellular localization in control and *PARP10*-knockdown cells. **K** RT-qPCR and Western blotting analysis of BCAT2 expression in control and *MYC*-knockdown BM5 cells. *n* = 3 independent experiments. **L** Western blotting analysis of BCAT2 expression in BM5 cells with sequential *PARP10* and *MYC* knockdown. **M** Western blot of MYC expression in *BCAT2*-knockdown 293 T and A549 cells. **N** Schematic showing the prediction of MYC binding sites in the *BCAT2* promoter and construction of wild-type and mutant luciferase reporter vectors. **O** ChIP-qPCR assay showing MYC enrichment at the *BCAT2* promoter in control and PARP10-knockdown cells. *n* = 3 independent experiments. **P** Dual-luciferase reporter assay showing MYC-mediated transcriptional activation of the *BCAT2* promoter. *n* = 3 independent experiments. Data are presented as mean ± SD. Statistical significance was determined by one-way ANOVA with Dunnett’s post-hoc test for comparisons to the control group, two-way ANOVA with Tukey’s post-hoc test for multi-factor grouped data, or unpaired two-tailed Student’s *t* test for comparisons between two groups. *P < 0.05, **P < 0.01, ***P < 0.001, ****P < 0.0001.

To explore how BCAA catabolism mitigates DNA damage, we analyzed genes identified by GSEA. The expression of most BCAA catabolic genes was upregulated upon *PARP10* knockdown (Fig. 6A), which was validated by mRNA quantification in vitro (Fig. S4A). Comparison of transcriptomic profiles between BM5 and A549 cells revealed that multiple genes in the BCAA catabolic pathway, including *BCAT2*, were upregulated after *PARP10* knockdown, whereas *BCAT1* and *ECHS1* were downregulated (Fig. 6B). BCAT1 and BCAT2 catalyze the initial steps of BCAA catabolism and regulate metabolic flux. BCAT1 is cytoplasmic, while BCAT2 is mitochondrial [43]. Since BCAA-derived metabolites likely mediate the functional effects, the observed downregulation of *BCAT1* seemed inconsistent with enhanced BCAA catabolism, whereas upregulated *BCAT2* expression aligned with increased catabolic activity. We therefore focused on *BCAT2* for subsequent studies. *PARP10* knockdown increased BCAT2 expression (Fig. 6C), but *BCAT2* knockdown did not affect PARP10 levels (Fig. 6D), indicating that PARP10 acts upstream of BCAT2.

To further elucidate the mechanism of PARP10-mediated transcriptional regulation of BCAT2, we first predicted transcription factor binding sites in the *BCAT2* promoter via the JASPAR database (Fig. 6E) and PARP10-interacting proteins via the STRING database (Fig. 6F), identifying a highly conserved MYC binding motif in the *BCAT2* promoter (Fig. 6G) and a predicted protein–protein interaction between MYC and PARP10. Co-IP assays demonstrated a direct endogenous protein-protein interaction between PARP10 and MYC (Fig. 6H). *PARP10* knockdown did not alter total MYC protein expression (Fig. 6I), while nucleocytoplasmic fractionation revealed markedly reduced cytoplasmic MYC and significantly elevated nuclear MYC upon *PARP10* silencing, indicating that PARP10 deficiency promotes MYC nuclear translocation (Fig. 6J). *MYC* knockdown significantly reduced BCAT2 expression at both the mRNA and protein levels (Fig. 6K). Concomitant *MYC* knockdown in *PARP10*-silenced cells completely abrogated the upregulation of BCAT2 induced by *PARP10* deficiency (Fig. 6L). Notably, *BCAT2* knockdown had no significant effect on MYC expression (Fig. 6M). Together, these findings indicate that MYC may mediate the transcriptional regulation of *BCAT2*. We next predicted and constructed luciferase reporter plasmids containing the wild-type human *BCAT2* promoter or a mutant promoter with disruption of the MYC-binding E-box motif (Fig. 6N). ChIP-qPCR assays verified the direct binding of MYC to the predicted motif in the *BCAT2* promoter, with significantly enhanced MYC enrichment at the *BCAT2* promoter observed upon *PARP10* knockdown (Fig. 6O). Dual-luciferase reporter assays showed that *MYC* overexpression significantly enhanced the transcriptional activity of the wild-type *BCAT2* promoter, which was completely abolished by mutation of the *MYC*-binding site (Fig. 6P). Taken together, our findings indicate that PARP10 restricts MYC nuclear translocation via direct protein–protein interaction. Upon PARP10 deficiency, unbound MYC accumulates and translocates into the nucleus, where it directly binds to the *BCAT2* promoter and transcriptionally activates its expression, thereby driving enhanced BCAA catabolism in LUAD cells.

### BCAT2 drives compensatory anti-apoptosis via mitochondrial OXPHOS enhancement

**Fig. 7:**
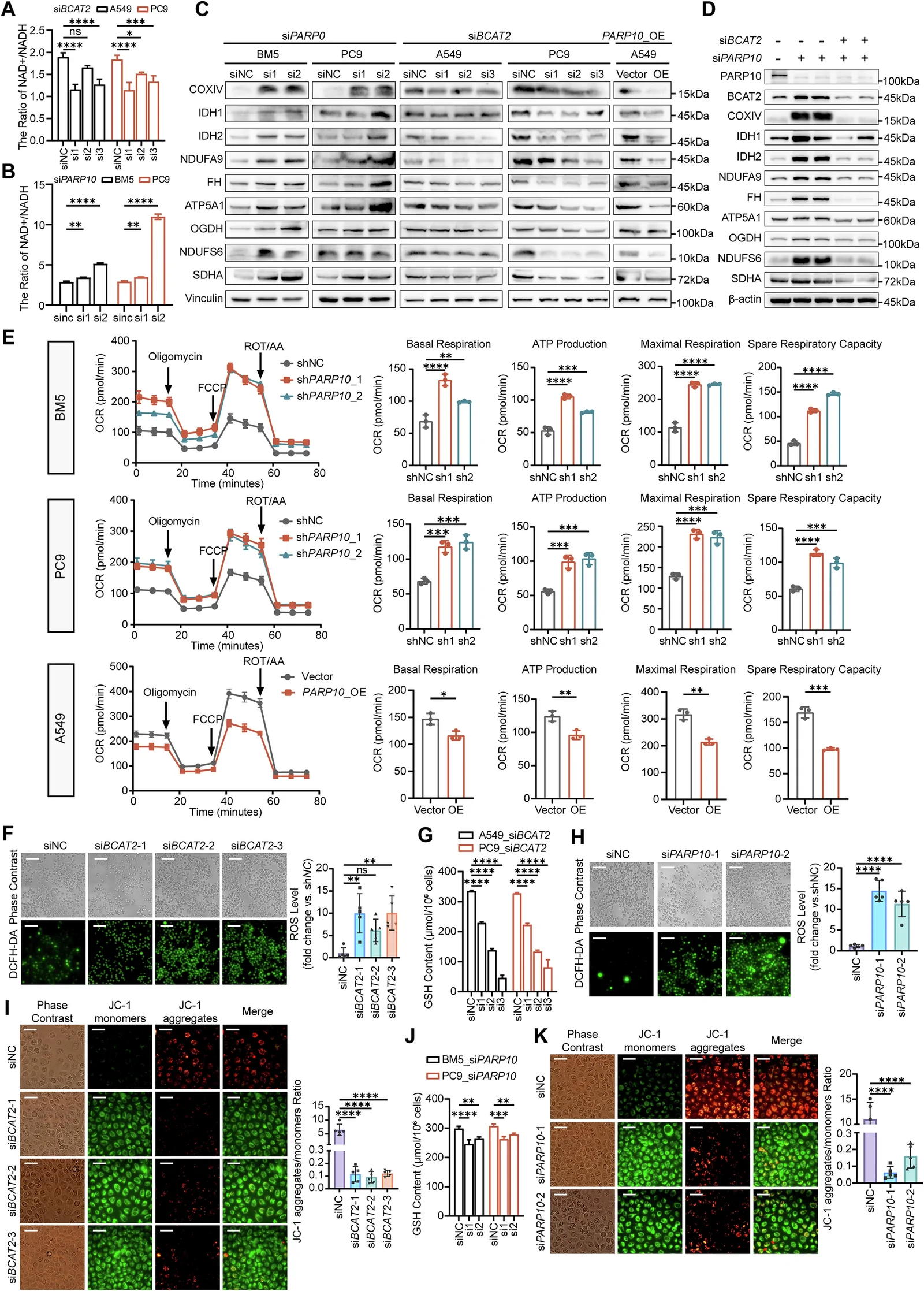
BCAT2 exerts a compensatory anti-apoptotic effect via enhancing OXPHOS. **A, B** Intracellular NAD⁺/NADH ratio analysis. **A** *BCAT2*-knockdown A549 and PC9 cells. **B** *PARP10*-knockdown BM5 and PC9 cells. *n* = 3 independent experiments. **C** Western blotting analysis of mitochondrial oxidative respiratory chain proteins in *BCAT2*-knockdown, *PARP10*-knockdown, or *PARP10*-overexpressing cells. **D** Western blotting analysis of mitochondrial oxidative respiratory chain proteins in cells with sequential *PARP10* and *BCAT2* knockdown. **E** Mitochondrial stress test. Real-time oxygen consumption rate (OCR) was measured in *PARP10*-knockdown or -overexpressing BM5, PC9, and A549 cells using a Seahorse XFe96 Extracellular Flux Analyzer. Oligomycin (1.5 μM), FCCP (1.5 μM), and rotenone/antimycin A (0.5 μM) were sequentially injected at the indicated time points. Basal respiration, ATP production, maximal respiration, and spare respiratory capacity were calculated. *n* = 3 independent experiments. **F** Intracellular reactive oxygen species (ROS) levels in *BCAT2*-knockdown PC9 cells, detected by DCFH-DA staining. *n* = 5 independent experiments. Scale bar, 100 μm. **G** Intracellular reduced glutathione (GSH) content in *BCAT2*-knockdown A549 and PC9 cells. *n* = 3 independent experiments. **H** Intracellular ROS levels in *PARP10*-knockdown PC9 cells, detected by DCFH-DA staining. *n* = 5 independent experiments. Scale bar, 100 μm. **I** Mitochondrial membrane potential (MMP, ΔΨm) measured by JC-1 staining (red: high ΔΨm, green: low ΔΨm) in *BCAT2*-knockdown PC9 cells. The JC-1 aggregate/monomer ratio was normalized to the control group. *n* = 5 independent experiments. Scale bar, 100 μm. **J** Intracellular reduced GSH content in *PARP10*-knockdown BM5 and PC9 cells. *n* = 3 independent experiments. **K** MMP (ΔΨm) measured by JC-1 staining in *PARP10*-knockdown PC9 cells. The JC-1 aggregate/monomer ratio was normalized to the control group. *n* = 5 independent experiments. Scale bar, 100 μm. Data are presented as mean ± SD. Statistical significance was determined by one-way ANOVA with Dunnett’s post-hoc test for comparisons to the control group, two-way ANOVA with Tukey’s post-hoc test for multi-factor grouped data, or unpaired two-tailed Student’s t test for comparisons between two groups.

BCAT2-mediated BCAA catabolism occurs primarily in the mitochondria. BCAA are first transaminated to branched-chain α-keto acids (BCKAs), then oxidized by the branched-chain α-keto acid dehydrogenase complex (BCKDC), and further metabolized to generate acetyl-CoA, ketone bodies, and reducing equivalents for energy production. During this process, NAD⁺ is reduced to NADH, a key electron donor for the mitochondrial electron transport chain (ETC) [43, 44]. We therefore investigated whether BCAT2-mediated mitochondrial BCAA catabolism affects ETC function. First, the regulatory effect of BCAT2 on cellular NAD⁺/NADH homeostasis was assessed. *BCAT2* knockdown reduced the intracellular NAD⁺/NADH ratio (Fig. 7A), indicating impaired ETC electron supply. Transcriptomic analysis revealed that *PARP10* knockdown upregulated the NAD⁺ biosynthesis pathway (Fig. S3D), consistent with concurrent BCAT2 upregulation. In addition, *PARP10* knockdown increased the intracellular NAD⁺/NADH ratio, a change that aligned with the upregulation of BCAT2 expression (Fig. 7B). Subsequently, analysis of mitochondrial oxidative respiratory chain proteins revealed that both *BCAT2* knockdown and *PARP10* overexpression significantly downregulated key NADH-driven respiratory chain components (Fig. 7C). Concurrent *BCAT2* knockdown in PARP10-silenced cells reversed the significant upregulation of key ETC proteins induced by PARP10 deficiency, confirming that BCAT2 is an essential mediator of PARP10-regulated mitochondrial oxidative phosphorylation (OXPHOS) in LUAD cells (Fig. 7D).

To further functionally validate the regulation of mitochondrial OXPHOS by the PARP10-BCAT2 axis, mitochondrial and glycolysis stress tests were performed in A549, BM5 and PC9 cells using a Seahorse XF Analyzer. OCR and extracellular acidification rate (ECAR) were measured to assess mitochondrial respiration and glycolysis, respectively. Mitochondrial stress tests showed that *PARP10*-knockdown cells exhibited significant increases in basal respiration, ATP production, maximal respiratory capacity, and spare respiratory capacity (Fig. 7E). In contrast, *PARP10* overexpression significantly reduced basal OCR and all related respiratory parameters. These results establish that PARP10 deficiency enhances mitochondrial OXPHOS in LUAD cells.

Glycolysis stress tests (Fig. S4B–D) showed that *PARP10* knockdown significantly decreased basal ECAR, glycolytic rate, and glycolytic capacity, while increasing glycolytic reserve. Conversely, *PARP10* overexpression exerted the opposite effects on glycolytic parameters. These findings are consistent with the regulatory effect of the PARP10-BCAT2 axis on BCAA catabolism, identifying BCAT2 as the core mediator of PARP10-modulated energy metabolism in LUAD cells. PARP10 functions as a key metabolic regulator in LUAD. High PARP10 expression drives a glycolysis-dominant Warburg phenotype, whereas PARP10 deficiency reverses the Warburg effect and promotes an OXPHOS-dominant metabolic program.

As the oxidative respiratory chain links energy metabolism to redox homeostasis, its dysfunction can induce oxidative stress. Mitochondria are both the primary source of intracellular reactive oxygen species (ROS) and the main target of oxidative damage. Our results showed that *BCAT2* knockdown increased intracellular ROS levels (Fig. 7F) and reduced the levels of reduced glutathione (GSH) (Fig. 7G), indicating that BCAT2 deficiency directly disrupts cellular redox homeostasis and induces oxidative stress injury in LUAD cells. Further JC-1 staining assay revealed that *BCAT2* knockdown induced loss of mitochondrial membrane potential and triggered early cellular apoptosis (Fig. 7I). Combined with the time-series experimental results (Fig. 5I–M), the burst of ROS accumulation and apoptosis was delayed relative to BCAT2 upregulation. These findings confirm that BCAT2 upregulation serves as an adaptive compensatory mechanism to maintain mitochondrial function and suppress apoptosis in PARP10-deficient LUAD cells.

To further assess the extent of this protective effect, mitochondrial membrane potential, ROS and GSH levels were measured in *PARP10*-knockdown tumor cells. Results showed that *PARP10* knockdown increased intracellular ROS levels (Fig. 7H) and reduced the levels of GSH (Fig. 7J), with a concomitant reduction in mitochondrial membrane potential (Fig. 7K), suggesting that cells activate antioxidant defense mechanisms in response to oxidative stress injury. Notably, despite BCAT2 upregulation following *PARP10* knockdown, the levels of cellular apoptosis markers remained elevated (Fig. S4E), indicating that mitochondrial apoptosis triggered by PARP10 deficiency could not be reversed by BCAT2. Taken together, although BCAT2 enhances mitochondrial OXPHOS to support tumor cell survival under stress, it is insufficient to completely counteract the pro-apoptotic effect induced by PARP10 deficiency. The dual role of PARP10 in regulating apoptosis and BCAA metabolism highlights its value as a potential therapeutic target for LUAD bone metastasis.

### Inhibition of PARP10 by OUL232 suppresses LUAD bone metastasis in vivo

**Fig. 8:**
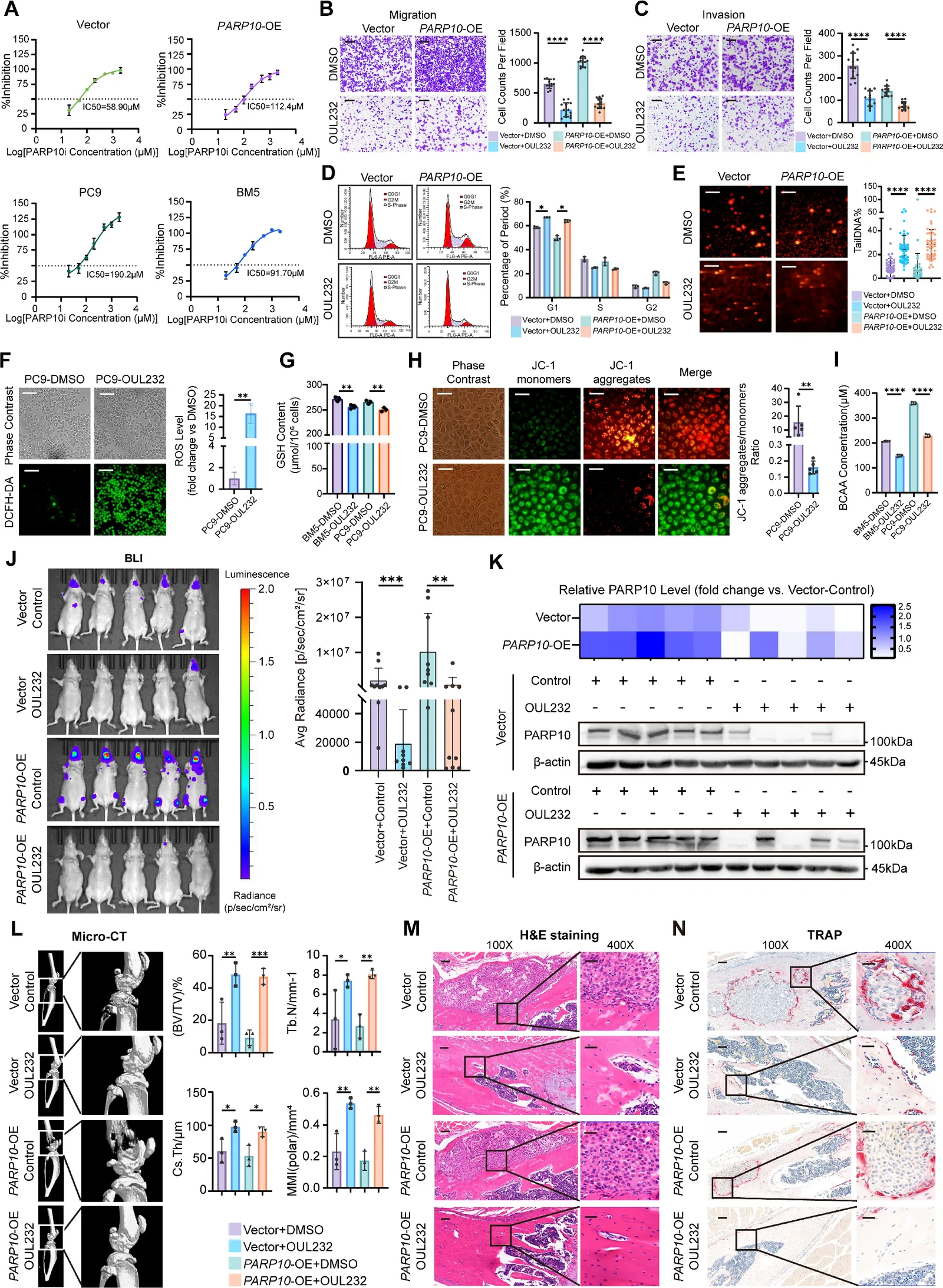
Inhibition of PARP10 by OUL232 suppresses LUAD bone metastasis in vivo. **A** Half-maximal inhibitory concentration (IC₅₀) of OUL232 in PC9, BM5, and *PARP10*-overexpressing A549 cells after 24 h treatment. *n* = 3 independent experiments. **B**, **C** Transwell migration (**B**) and Matrigel invasion (**C**) assays in vector control and *PARP10*-overexpressing A549 cells treated with 20 μM OUL232. *n* = 9–13 random fields per group. Scale bar, 200 μm. **D** Flow cytometry-based cell cycle analysis of A549 cells treated with 20 μM OUL232 or vehicle control (DMSO) for 24 h. *n* = 3 independent experiments. **E** Comet assay assessing DNA damage (quantified by tail DNA percentage) in A549 cells treated with 200 μM OUL232 or vehicle control for 1 h. *n* = 50 comets per group. Scale bar, 100 μm. **F** Intracellular ROS levels in PC9 cells treated with 20 μM OUL232 for 24 h, detected by DCFH-DA staining. *n* = 5 independent experiments. Scale bar, 100 μm. **G** Intracellular GSH content in A549 cells treated with 20 μM OUL232 for 24 h. *n* = 3 independent experiments. **H** MMP (ΔΨm) in PC9 cells treated with 20 μM OUL232 for 24 h, measured by JC-1 staining. The JC-1 aggregate/monomer ratio was normalized to the control group. *n* = 5 independent experiments. Scale bar, 100 μm. **I** BCAA concentrations in serum-free culture supernatant after 20 μM OUL232 treatment for 24 h. *n* = 3 independent experiments. **J**–**N** In vivo anti-metastatic efficacy of OUL232. Bone metastasis models were established by left ventricular intracardiac injection of A549 cells. OUL232 (50 mg/kg, 200 μL per mouse) or vehicle control was administered intraperitoneally every 2 days for 4 weeks, starting 1 day post-inoculation. Mice were sacrificed 4 weeks post-inoculation. **J** Representative bioluminescence imaging (BLI) of metastatic lesions. Signal intensity is expressed as average radiance (p/sec/cm²/sr). *n* = 9–10 mice per group. **K** Western blotting analysis of PARP10 expression in isolated bone metastatic lesions. *n* = 5 mice per group. **L** Micro-CT analysis of osteolytic bone destruction. Quantified parameters include bone volume/total volume (BV/TV), trabecular number (Tb.N), cross-sectional thickness (Cs.Th), and mean polar moment of inertia (MMI). *n* = 3 mice per group. **M** Representative H&E staining of bone structure and metastasis. **N** Representative TRAP staining of osteoclast infiltration. Scale bars: 100 μm (×100 magnification), 40 μm (×400 magnification). Data are presented as mean ± SD. Statistical significance was determined by unpaired two-tailed Student’s *t* test for comparisons between two groups. *P < 0.05, **P < 0.01, ***P < 0.001, ****P < 0.0001.

OUL232 is the most potent and selective PARP10-specific inhibitor reported to date [31]. We first determined the half-maximal inhibitory concentration (IC_50_) of OUL232 in LUAD cell lines (Fig. 8A). Corresponding DMSO concentrations showed no significant effect on cell viability (Fig. S5A). BM5 cells exhibited higher IC50 values than A549 cells, and higher PARP10 expression was positively correlated with increased OUL232 tolerance, whereas *PARP10* knockdown sensitized cells to the inhibitor (Fig. S5B). These results indicate that the inhibitory effect of OUL232 is PARP10-dependent. Treatment with a sublethal OUL232 concentration suppressed migration and invasion (Fig. 8B, C), induced G1/S phase cell cycle arrest (Fig. 8D), and increased DNA damage (Fig. 8E), demonstrating that OUL232 effectively inhibit PARP10 function in vitro. Consistent results were observed in A549, BM5, and PC9 cells (Fig. S5C–F). Short-term high-dose OUL232 treatment elevated ROS (Fig. 8F), decreased GSH (Fig. 8G), and reduced mitochondrial membrane potential (Figs. 8H and S5G), indicating strong oxidative stress and a pro-apoptotic state. After 24 h of low-dose OUL232 treatment, BCAA levels in serum-free conditioned medium (CM) were significantly reduced (Fig. 8I), suggesting that PARP10 inhibition enhances BCAA metabolism to support survival.

To investigate whether altered BCAA metabolism is specifically associated with PARP10 inhibition, or represents a general response to DNA damage, we treated LUAD cells with paclitaxel (PTX) or camptothecin (CPT) and assessed DNA damage and BCAA levels in the CM. PTX is a microtubule stabilizer that inhibits microtubule depolymerization, primarily disrupting mitosis without directly inducing DNA damage, whereas CPT is a topoisomerase I inhibitor that directly induces DNA single-strand breaks and cell cycle arrest. We first determined the IC_50_ values of PTX and CPT in PC9 and BM5 cells (Fig. S5H). We then treated cells with high concentrations of the drugs for 1 h to assess DNA damage (Fig. S5I). PTX induced no significant DNA damage, whereas CPT resulted in significantly elongated comet tails, confirming the distinct mechanisms of action of the two drugs. Next, cells were treated with sublethal doses of the drugs (1 nM PTX or 0.5 nM CPT) for 24 h. BCAA levels in the CM were significantly increased in both treatment groups compared with the vehicle control group (Fig. S5J), indicating that under non-lethal genotoxic stress, tumor cells reduce BCAA consumption as cell proliferation is inhibited. These results establish that altered BCAA catabolism is not a general DNA damage response, but is specifically linked to PARP10 function in LUAD cells.

In summary, PARP10 inhibition disrupts DNA single-strand break repair, leading to replication fork collapse and double-strand break accumulation in LUAD cells. Lethal DNA damage can directly induce tumor cell death, whereas sublethal damage triggers an adaptive stress response that enhances BCAA catabolism to mitigate DNA damage. Although this metabolic adaptation supports tumor cell survival under stress, it traps cells in a chronic stress state characterized by persistent cell cycle arrest and oxidative damage, at the cost of reduced invasive and metastatic capacity. Concurrently, PARP10-inhibited tumor cells consume local BCAA in the bone metastatic niche, resulting in metabolic competition with osteoclast precursors, thereby inhibiting osteoclast differentiation and osteolytic bone destruction. Thus, the therapeutic efficacy of PARP10 inhibition stems from both direct tumor cytotoxicity and microenvironmental metabolic remodeling via BCAA competition.

**Fig. 9:**
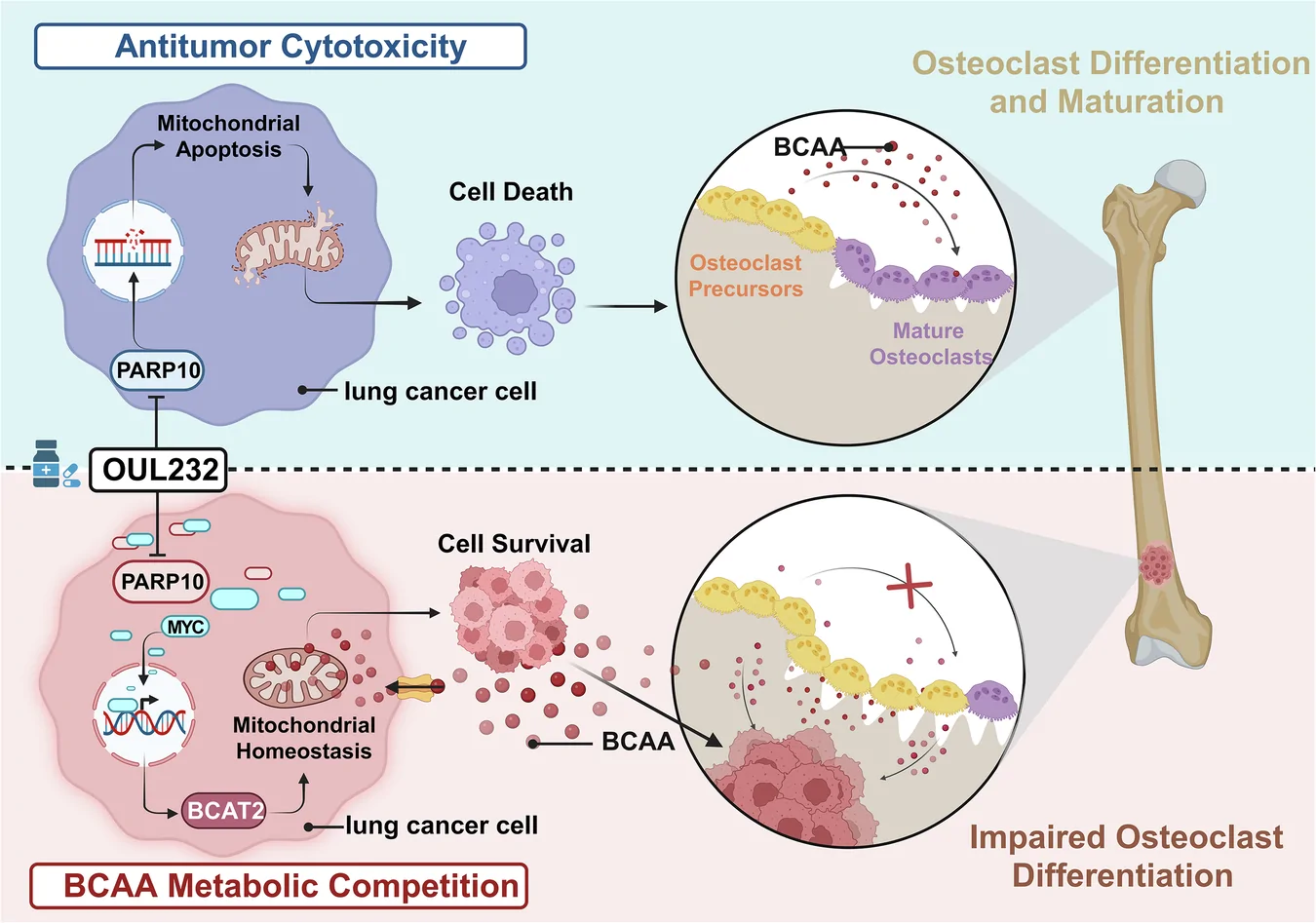
Graphical abstract: dual-mechanism anti-metastatic therapy via PARP10 inhibition by OUL232. Pharmacological inhibition of PARP10 by OUL232 exerts dual anti-metastatic effects in LUAD. On one hand, OUL232 induces DNA damage that triggers mitochondrial apoptosis, leading to direct tumor cell death. On the other hand, it provokes a BCAT2-mediated adaptive survival response via enhanced BCAA catabolism in surviving tumor cells. This increased BCAA consumption creates a metabolic competition in the bone metastatic niche that deprives osteoclast precursors of essential BCAA, thereby suppressing osteoclast differentiation and breaking the vicious cycle of osteolytic bone destruction. The schematic diagram was created using BioRender.

We next evaluated the therapeutic efficacy of the PARP10 inhibitor OUL232 in a mouse model of bone metastasis. Mice received intraperitoneal injections every other day for four weeks before assessment of metastasis (Fig. S6A). OUL232 treatment significantly reduced the incidence of bone metastasis and the bioluminescence intensity of metastatic foci compared with the control group (Fig. 8J). The inhibitor also reversed the pro-metastatic phenotype induced by *PARP10* overexpression, indicating strong therapeutic benefit. Higher PARP10 expression was associated with reduced sensitivity to OUL232, which could inform future dose optimization. PARP10 expression in isolated bone metastatic lesions was lower after OUL232 treatment (Fig. 8K), consistent with on-target activity of the inhibitor. Micro-CT (Fig. 8L), H&E (Fig. 8M), and TRAP staining (Fig. 8N) revealed that OUL232 attenuated osteolytic destruction and decreased osteoclast numbers, supporting the conclusion that OUL232 remodels the bone microenvironment by limiting BCAA availability and impairing osteoclast differentiation. To assess the potential systemic toxicity of OUL232, we performed H&E staining of the major organs (heart, liver, spleen, lungs, kidneys, and intestines) from mice after 4 weeks of treatment (Fig. S6B). The histological architecture of all major organs remained intact, with no obvious inflammatory infiltration or tissue damage, indicating that OUL232 has no significant off-target systemic toxicity while exerting its anti-tumor and anti-metastatic effects. As a PARP10 inhibitor, OUL232 can effectively suppress the progression of lung cancer bone metastasis both in vitro and in vivo, highlighting its potential as a therapeutic candidate for LUAD bone metastasis and warranting further clinical investigation. (Fig. 9).

## Discussion

Bone metastasis from LUAD is a highly destructive and therapy-resistant clinical condition, driven by reciprocal crosstalk between tumor cells and the bone metastatic microenvironment. Targeting this tumor–bone interaction represents a promising therapeutic strategy for metastatic LUAD. This study investigated the anti-metastatic potential of PARP10 and its inhibitor OUL232, and uncovered a previously unrecognized dual mechanism of action. We find that PARP10 inhibition induces DNA damage and oxidative stress in LUAD cells, triggering an adaptive upregulation of BCAA catabolism to support cell survival. This metabolic shift, in turn, creates a nutrient-competitive microenvironment that deprives osteoclast precursors of essential BCAA, thereby suppressing osteoclast differentiation and breaking the vicious cycle of bone metastasis. Collectively, our work defines a novel PARP10-MYC-BCAT2-BCAA metabolic competition axis between LUAD cells and osteoclasts, and proposes PARP10 inhibition as a promising therapeutic strategy for LUAD bone metastasis.

PARP10 has been implicated in the development and progression of multiple human malignancies. Our study provides the first comprehensive mechanistic insight into the oncogenic role of PARP10 in LUAD. We find that PARP10 promotes LUAD cell proliferation, migration, invasion, G1/S phase cell cycle transition, and DNA single-strand break repair in vitro, and enhances bone metastatic colonization in vivo. These pro-tumorigenic roles are consistent with its functions in acute myeloid leukemia [21], oral squamous cell carcinoma [24], colorectal cancer [25], and pancreatic cancer [26]. Conversely, PARP10 can act as a tumor suppressor in other contexts, inhibiting the formation and metastasis of liver cancer [45, 46] and cervical cancer progression [47]. This functional duality, known as context-dependent oncogenicity, is shared by other well-characterized molecules such as TGF-β [48], MYC [49], DMD [50], FAT1 [51], and LMO2 [52]. The role of PARP10 is likely influenced by factors including cellular origin, differentiation status, tumor stage, epigenetic landscape, and the tumor microenvironment. This complexity highlights a key challenge in precision oncology. Therefore, the clinical application of PARP10 inhibitors will require a detailed understanding of their context-dependent functions, with therapeutic strategies tailored to specific patient populations.

This study finds that the metabolic chemotaxis of tumor cells towards BCAAs is significantly enhanced following PARP10 inhibition. Distinct from chemokine-receptor signaling, metabolic chemotaxis relies on chemical gradients of metabolites, such as lactate [34], lipoproteins [35], amino acids [36–38], and lipids [39], to direct cell migration or functional polarization, thereby influencing immune evasion, invasion, colonization, and therapy resistance. Using a modified Transwell system with a precisely defined BCAA concentration gradient, we observed that *PARP10*-knockdown LUAD cells exhibit enhanced directional migration towards BCAA. The role of BCAA and their downstream metabolites in guiding cell migration is increasingly appreciated. For example, gain-of-function mutations in *BCAT1* promote tumor proliferation and migration [45, 53], and leucine, the most abundant BCAA, activates mTORC1 signaling to modulate cytoskeletal dynamics and cell motility [54].

The role of BCAA metabolism in tumor progression has attracted growing interest. While current research has predominantly highlighted the tumor-promoting functions of BCAT1 in various cancers [53, 55–58], the role of BCAT2 remains less clearly defined. Recent evidence indicates that BCAT2 is upregulated in several malignancies-including prostate cancer [59], pancreatic ductal adenocarcinoma [44, 60], and melanoma [61], where it supports tumor growth and progression, and can confer resistance to anti-PD-1/PD-L1 immunotherapy in bladder cancer [62]. This study defines a critical, previously unrecognized role for BCAT2 in LUAD, and elucidates a novel molecular mechanism whereby PARP10 regulates *BCAT2* transcription via MYC nuclear translocation. It shows that BCAT2 enhances mitochondrial OXPHOS in LUAD cells, thereby improving cellular resistance to oxidative stress and suppressing intrinsic apoptosis. Importantly, this adaptive metabolic response is specifically triggered by PARP10 inhibition and is not observed following treatment with other DNA-damaging or cytotoxic agents such as PTX or CPT [63, 64]. Enhanced BCAA catabolism, therefore, represents a novel adaptive cell survival pathway in response to PARP10 inhibition. Although this pathway transiently delays apoptosis, it cannot counteract the lethal DNA damage induced by sustained PARP10 inhibition, ultimately leading to tumor cell death.

Furthermore, this study systematically investigated the role of BCAA metabolism in regulating the bone metastatic microenvironment of LUAD. A metabolic competition for BCAA was observed between tumor cells and osteoclasts. Osteoclast differentiation and maturation rely on BCCAs [33]. Under pathological conditions, mature osteoclasts interact with tumor cells to drive the vicious cycle of bone metastasis, which accelerates osteolytic bone destruction and tumor growth [65, 66]. This study finds that PARP10 inhibition reshapes tumor-osteoclast metabolic crosstalk by enhancing BCAA consumption in tumor cells, leading to BCAA deprivation in the bone microenvironment. This nutrient deprivation impairs osteoclast precursor differentiation and maturation, ultimately suppressing osteoclast-mediated bone destruction and LUAD bone metastasis. This dual anti-tumor and anti-osteoclast effect highlights PARP10 as a unique therapeutic target with a two-pronged mechanism of action for bone metastasis. To evaluate the therapeutic potential of targeting PARP10 in LUAD bone metastasis, the in vivo efficacy of the PARP10 inhibitor OUL232 was assessed in a mouse bone metastasis model. Results confirmed that OUL232 significantly suppresses bone metastasis progression in vivo.

Several limitations of this study should be acknowledged. First, the therapeutic efficacy of PARP10 inhibition has not yet been validated in clinical samples. Although no significant systemic toxicity of OUL232 was observed in the major organs of mice, this inhibitor was only recently developed [31]. Therefore, further comprehensive pharmacokinetic and pharmacodynamic studies are required before clinical translation can be considered. Second, the precise mechanism by which BCAT2 enhances mitochondrial OXPHOS remains to be fully elucidated. Specifically, it is unclear whether enhanced mitochondrial metabolism is driven directly by BCAT2 enzymatic activity, or by downstream metabolites of BCAA catabolism, such as acetyl-CoA or succinyl-CoA. In addition, the metabolic chemotactic effect of BCAA on PARP10-deficient tumor cells requires further validation using more sophisticated experimental approaches. Current Transwell-based assays cannot fully exclude the confounding effect of cell proliferation on migration results. More precise quantitative methods, such as stable isotope labeling combined with live-cell imaging or mass spectrometry [55, 67, 68], will help provide definitive molecular validation of this chemotactic effect. Finally, predictive biomarkers for PARP10-targeted therapy have not yet been established. Clinical use of conventional PARP1/2 inhibitors relies heavily on biomarkers of homologous recombination deficiency (HRD), such as *BRCA1/2* and *PALB2* mutations in breast cancer, and *ATM* or *BRCA2* mutations in prostate cancer [15–18]. In LUAD, *PARP10* expression shows no or only weak correlation with these traditional HRD-related biomarkers, indicating that they are not suitable for predicting response to PARP10 inhibition. Therefore, future large-scale clinical studies are needed to collect samples from LUAD patients with bone metastasis, to identify novel predictive molecular biomarkers that can accurately stratify patients most likely to benefit from PARP10-targeted therapy.

## Supplementary information


Supplementary Information
Original Western Blots


## Data Availability

The original transcriptomic datasets generated in this study have been deposited in the GEO repository under accession numbers GSE273023, GSE274442 and GSE225212. Kaplan–Meier overall survival analysis was performed using the Kaplan–Meier Plotter database (https://kmplot.com/analysis/). Pan-cancer *PARP10* expression analysis in tumor versus adjacent normal tissues was performed using the TIMER2.0 web server (http://timer.cistrome.org/). Meta-analysis of *PARP10* expression in normal lung versus LUAD tissues was performed using the Lung Cancer Explorer database (https://lce.biohpc.swmed.edu/lungcancer/). *PARP10* genomic alteration profiles were obtained from the cBioPortal for Cancer Genomics (https://www.cbioportal.org/). All other data generated or analyzed during this study are available from the corresponding authors upon reasonable request.
