# Supplementary material for: Targeting the PARP10-BCAT2 axis disrupts branched-chain amino-acid metabolism to suppress bone metastasis in lung cancer
Source: Cell Death Dis. 2026 Jun 19;17(1):763. doi: 10.1038/s41419-026-08989-3 (PMC13526790; doi:10.1038/s41419-026-08989-3)

Figure	Merged	Stiched full image

Fig.1H

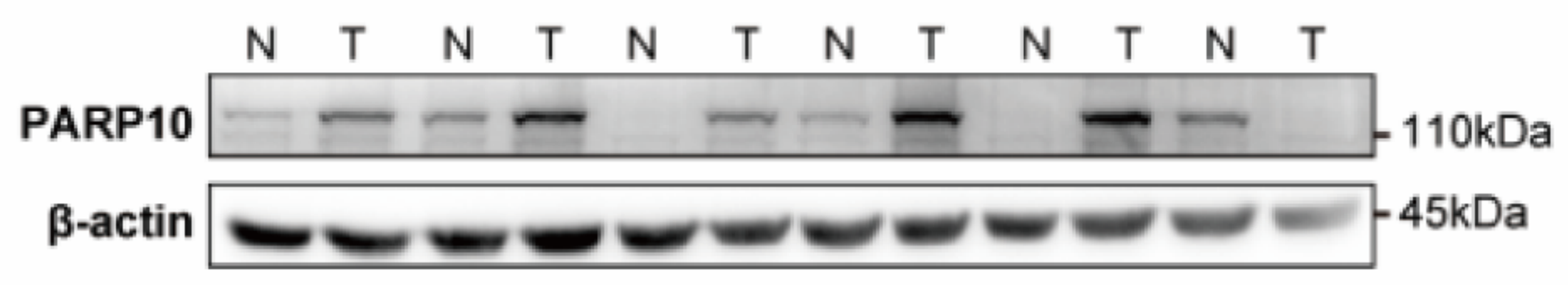

PARP10

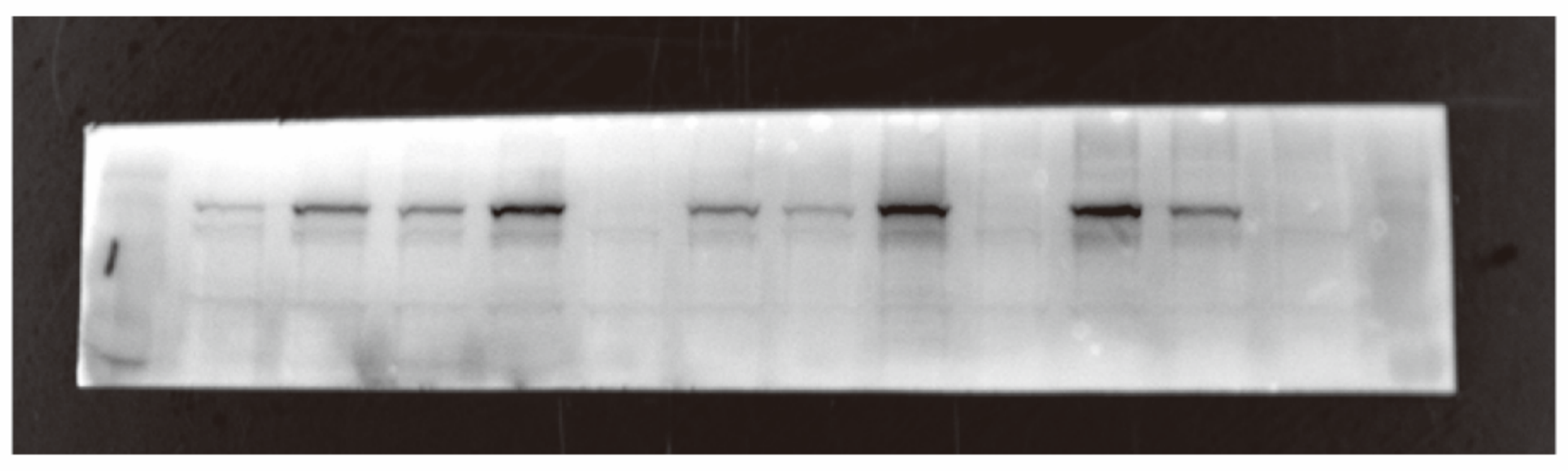

β-actin

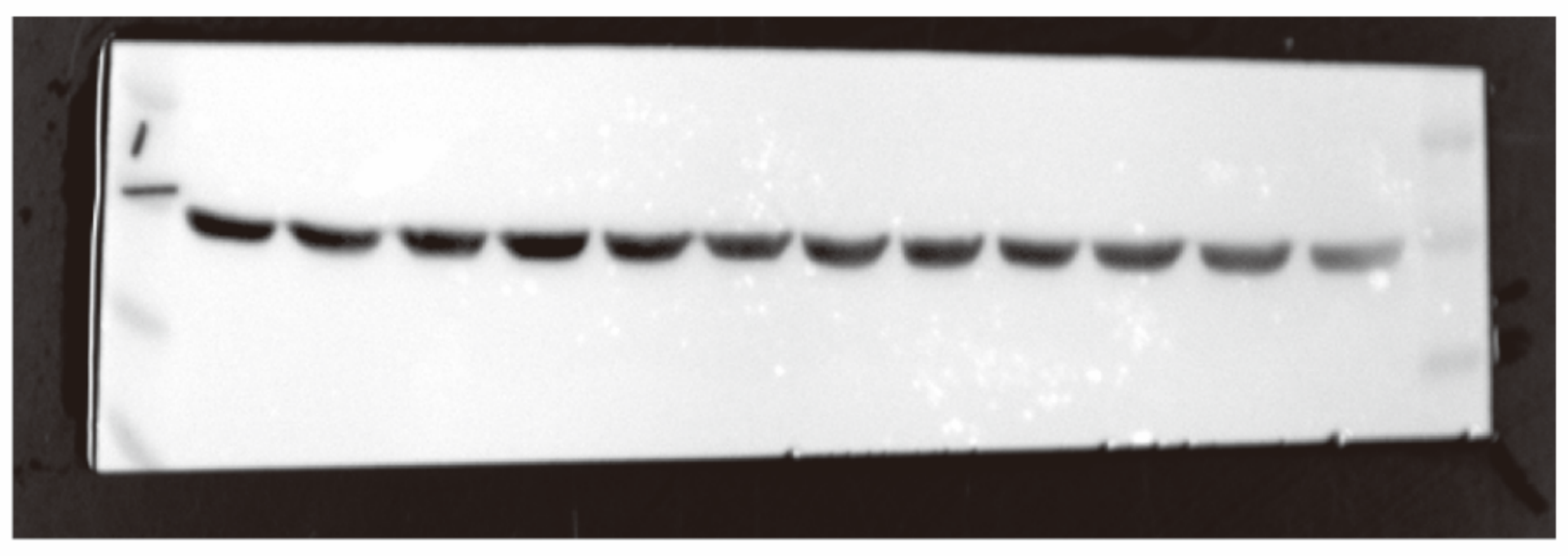

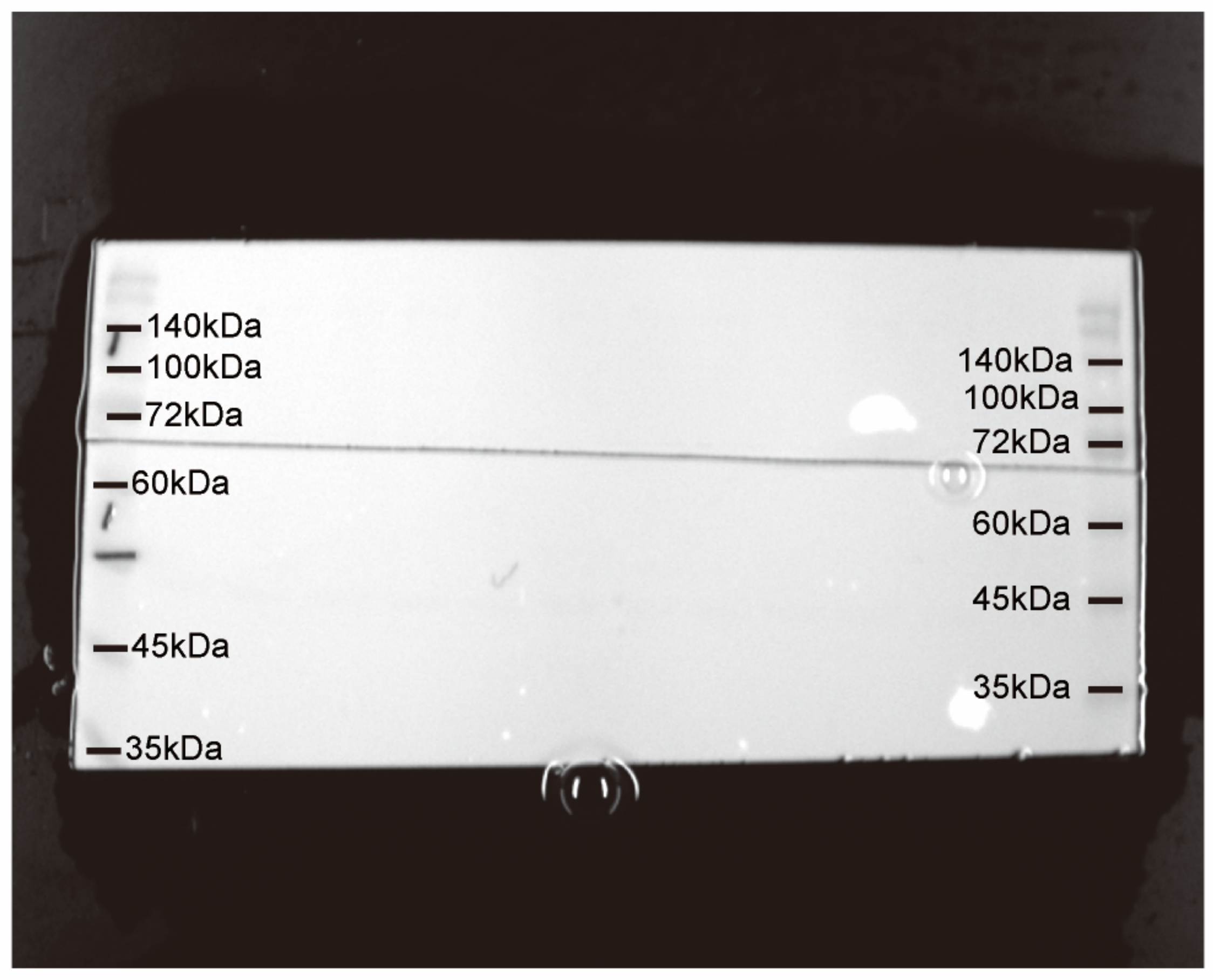

Fig.1H

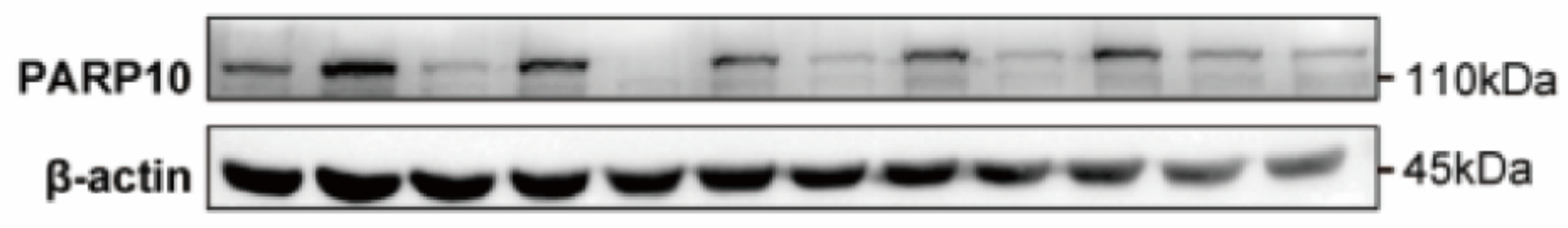

PARP10

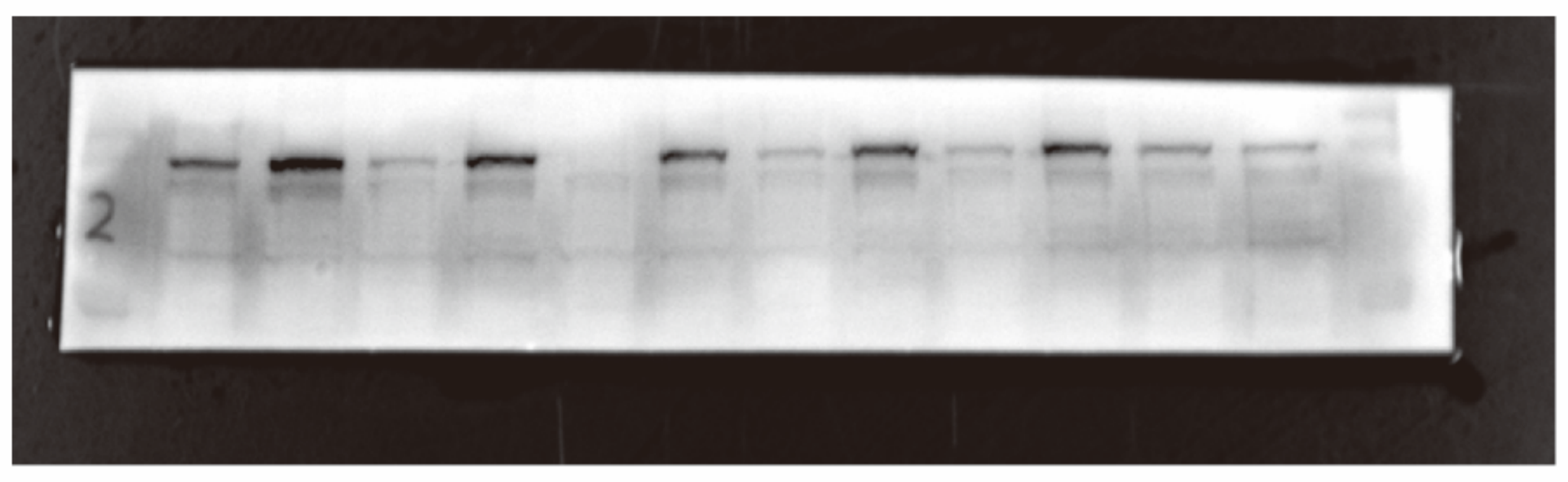

β-actin

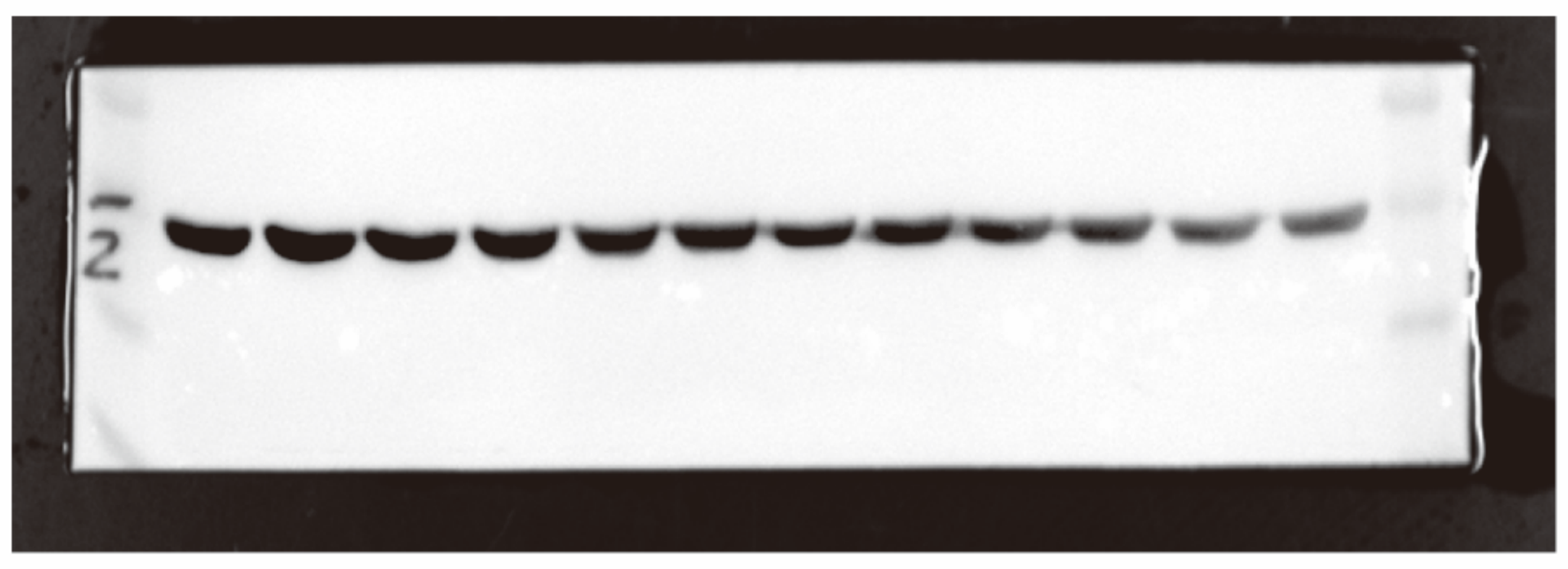

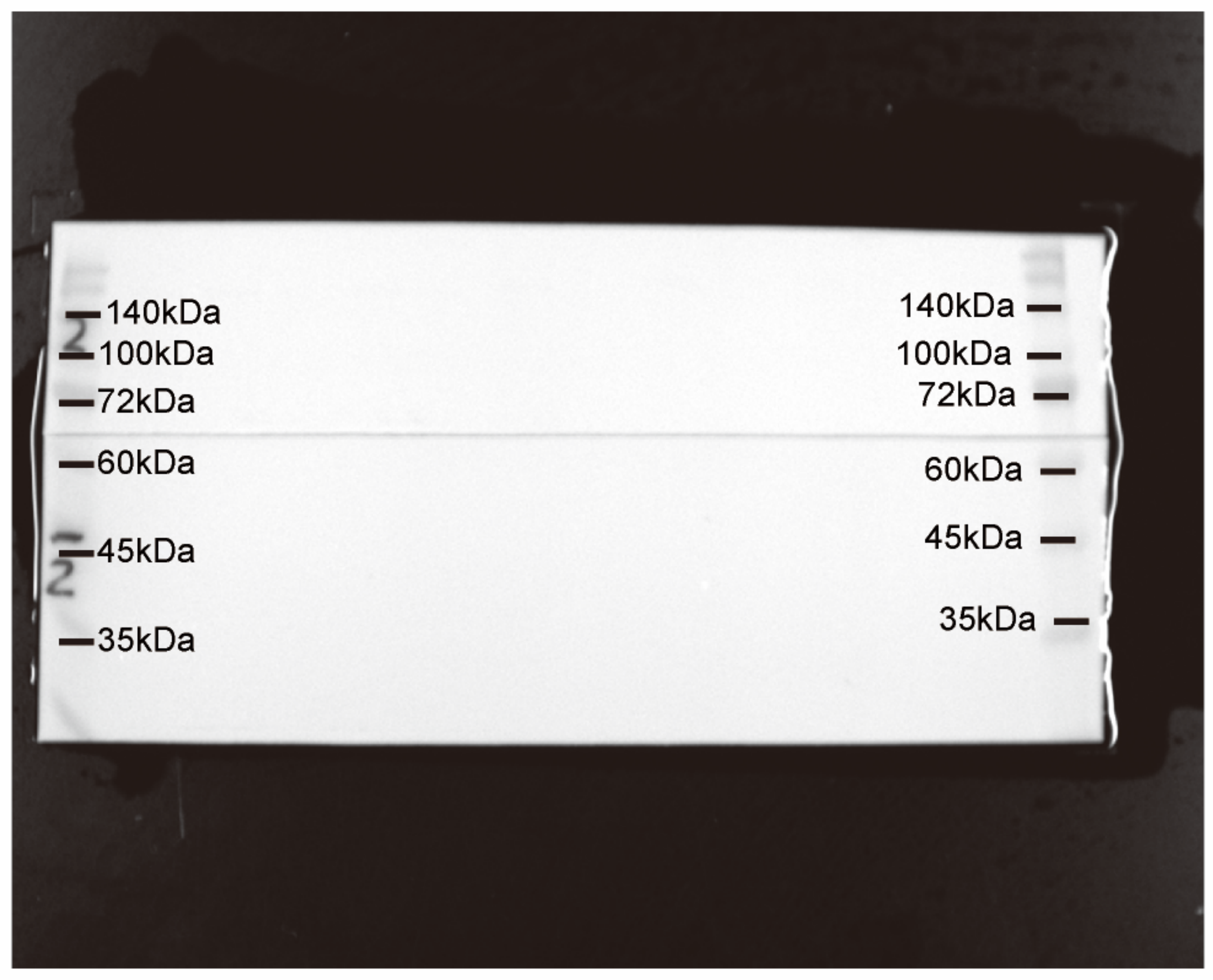

Fig.1H

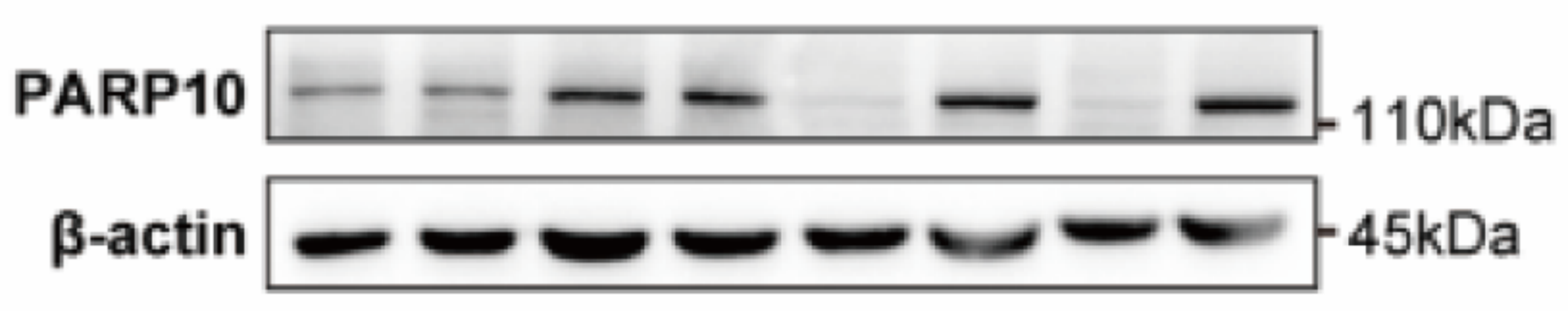

PARP10

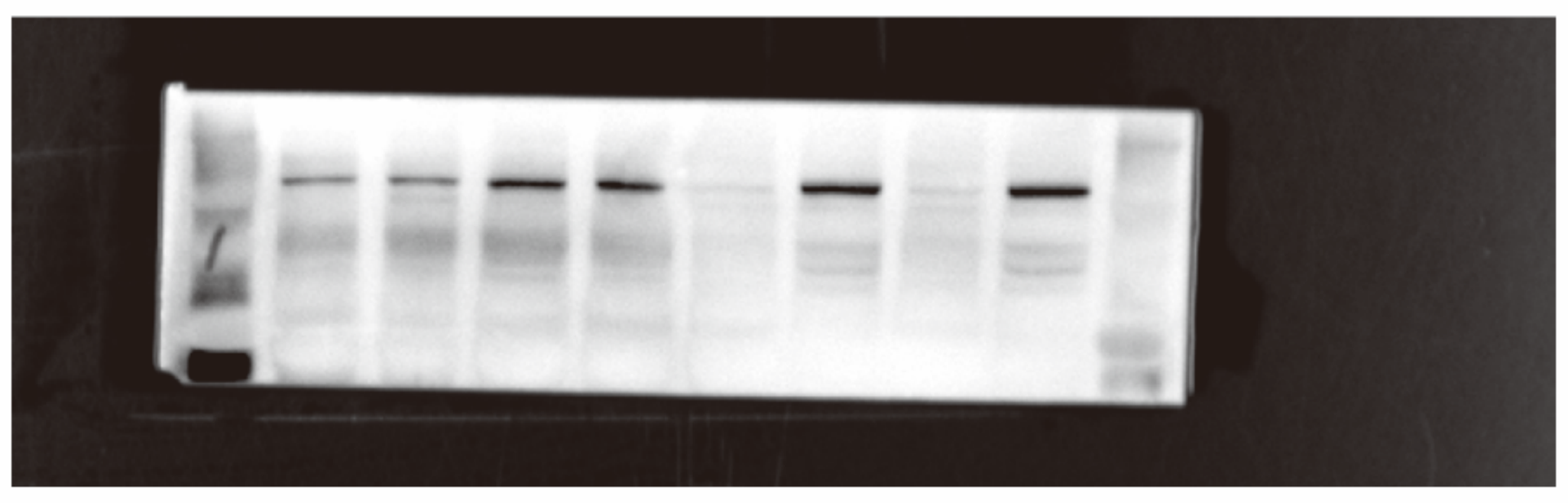

β-actin

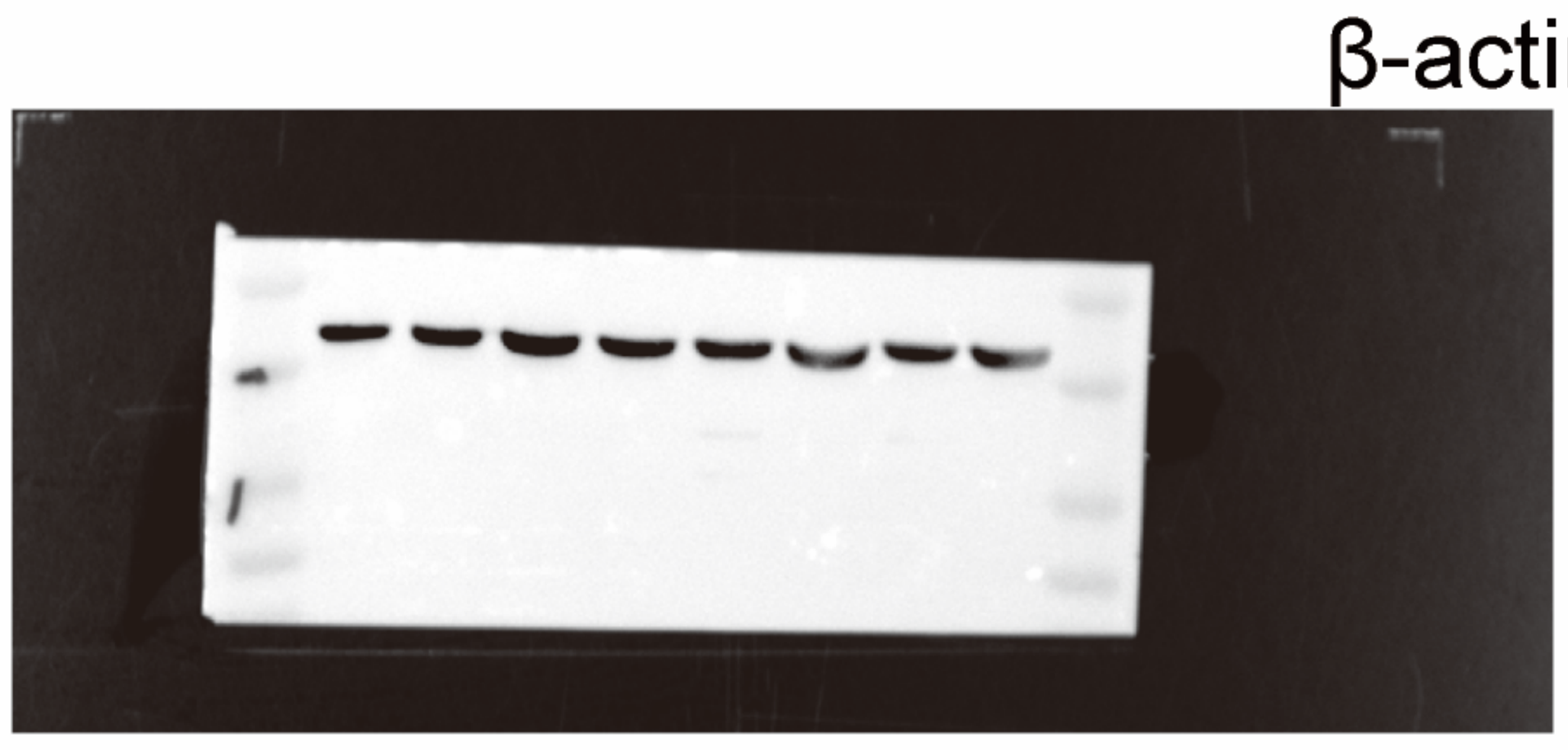

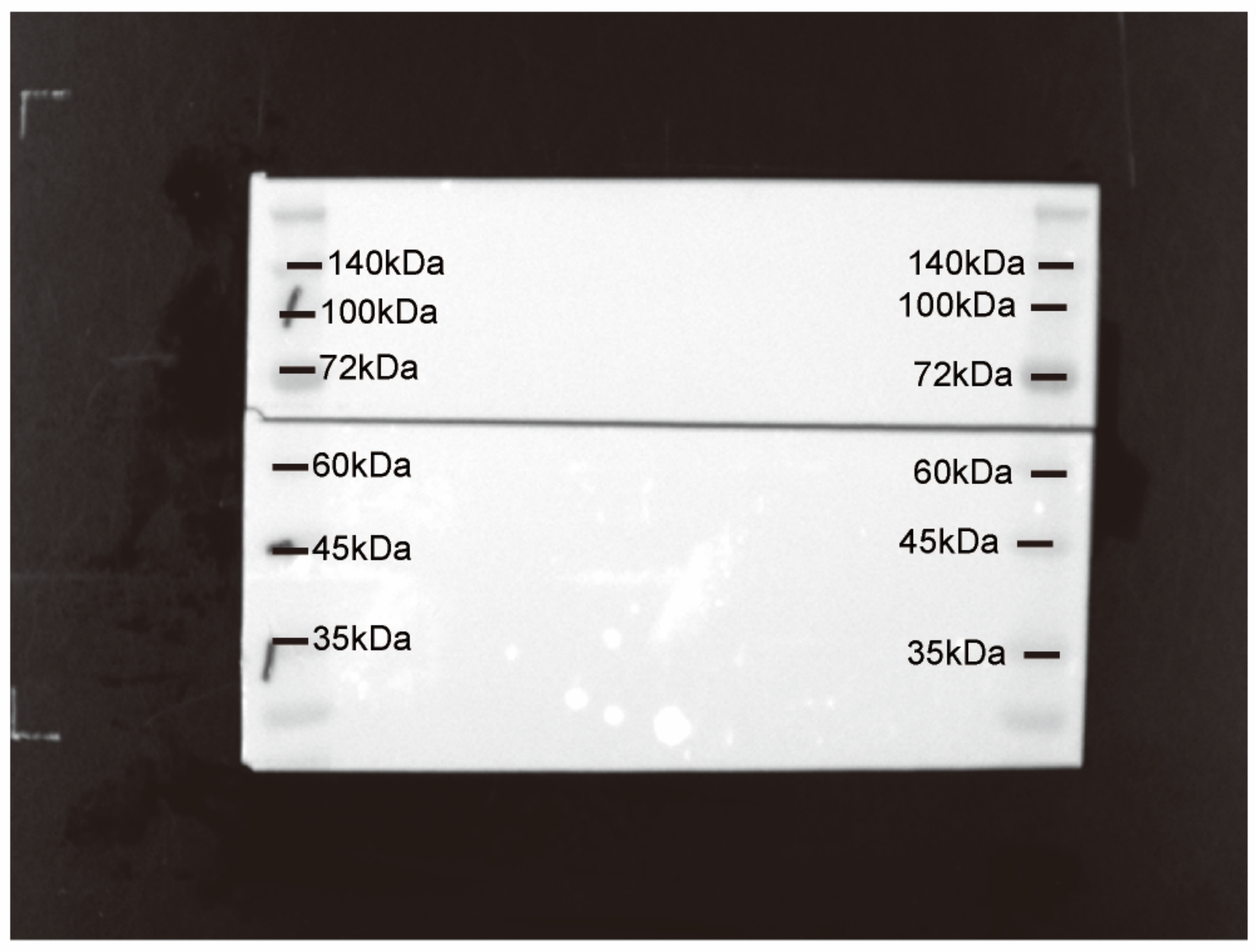

Fig.2A

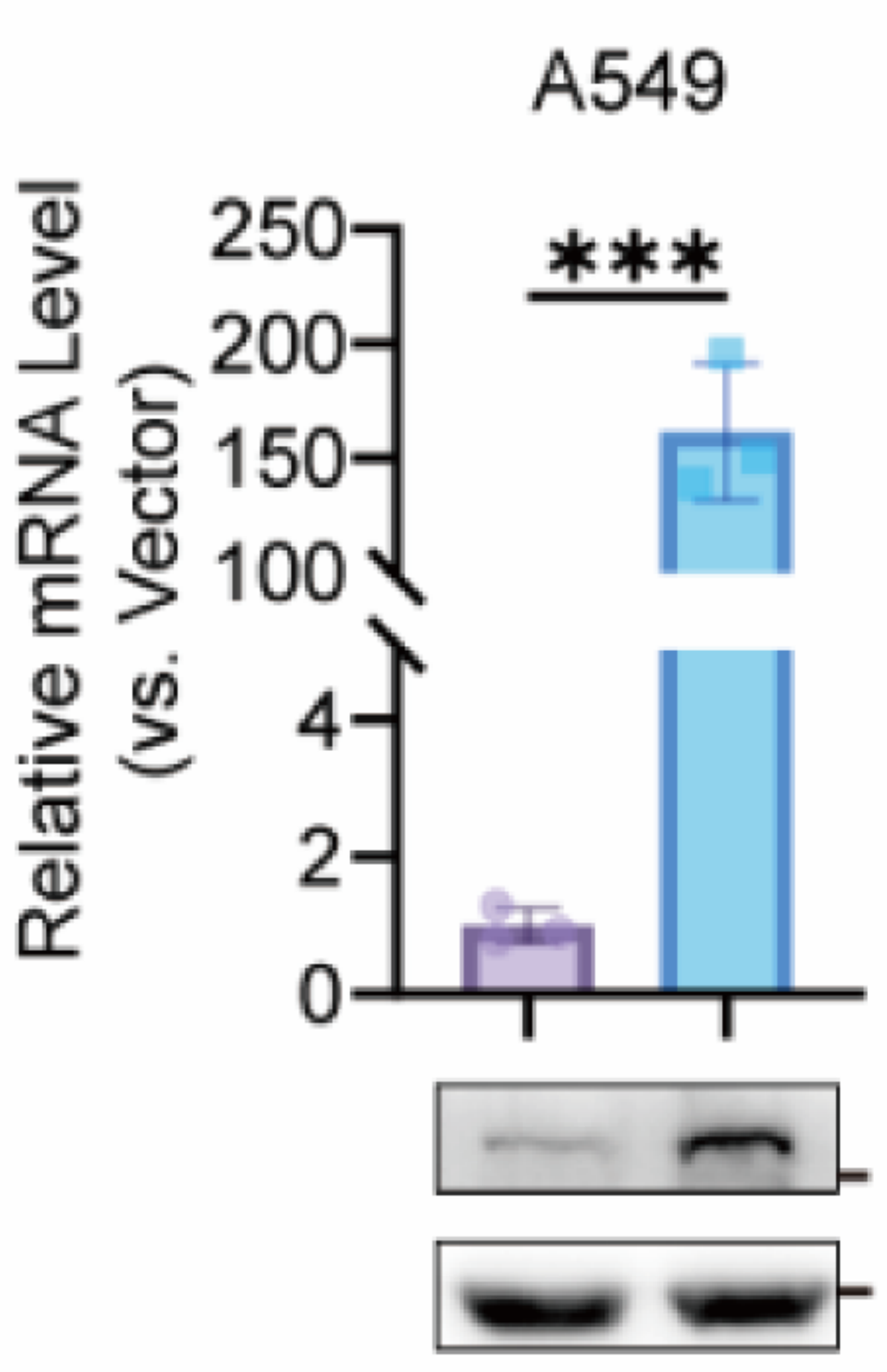

PARP10

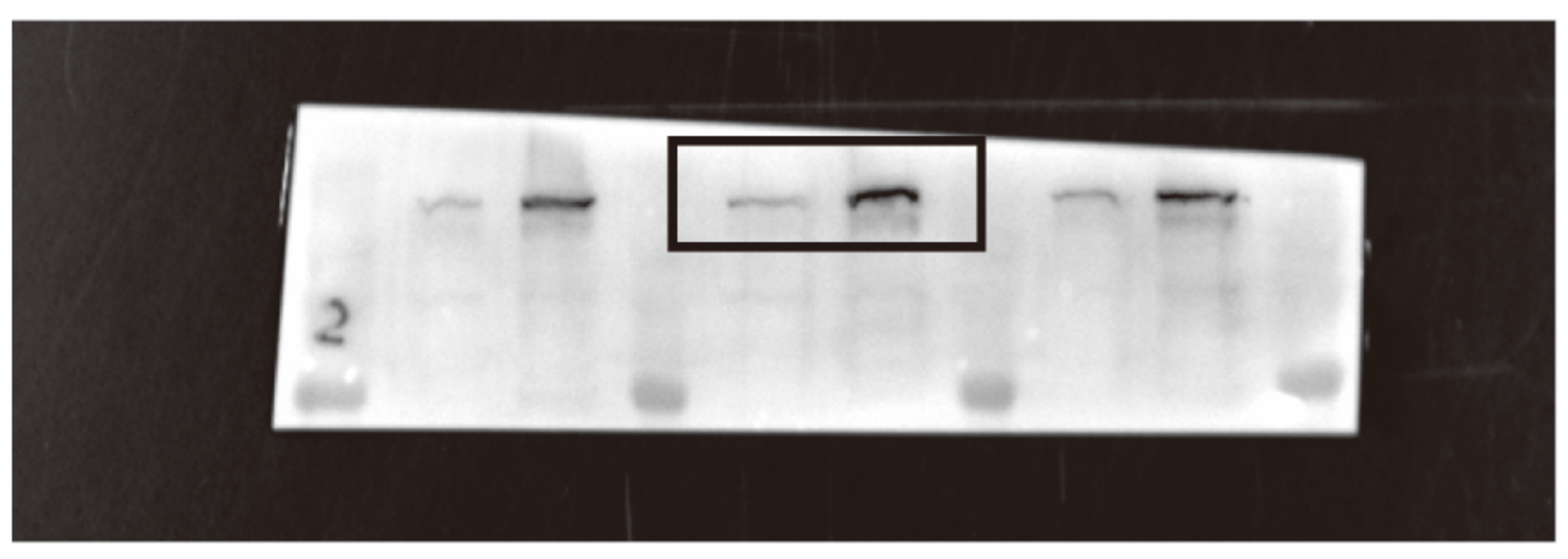

β-actin

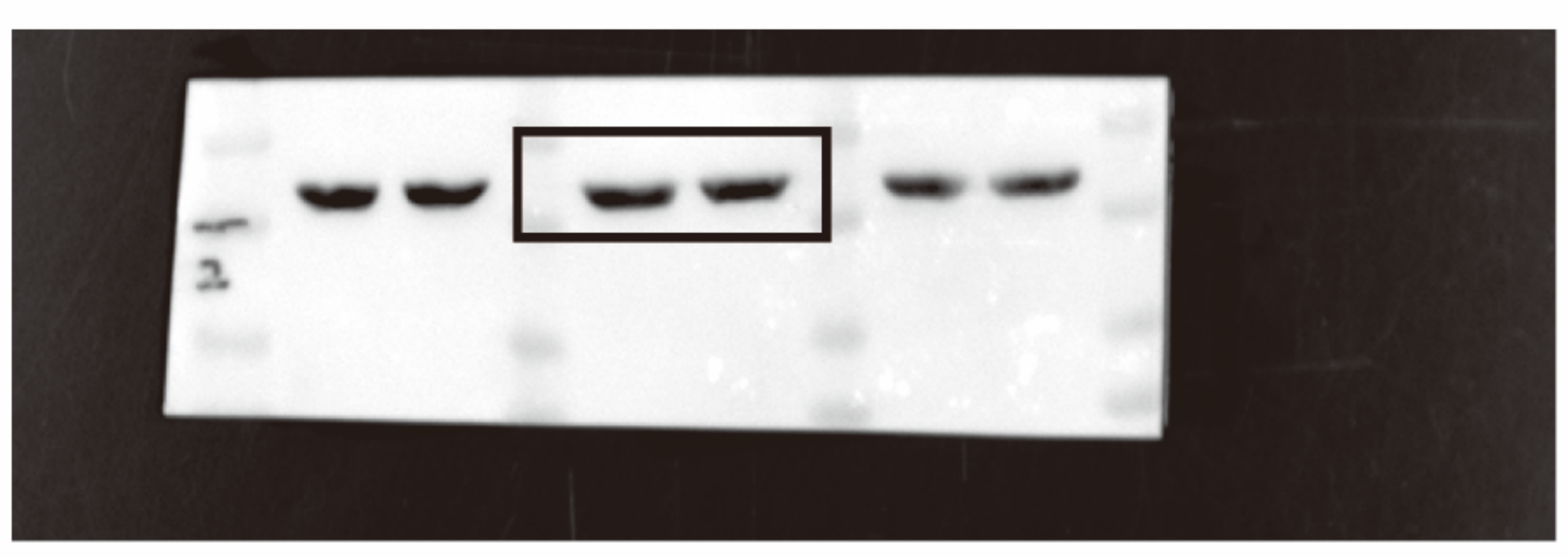

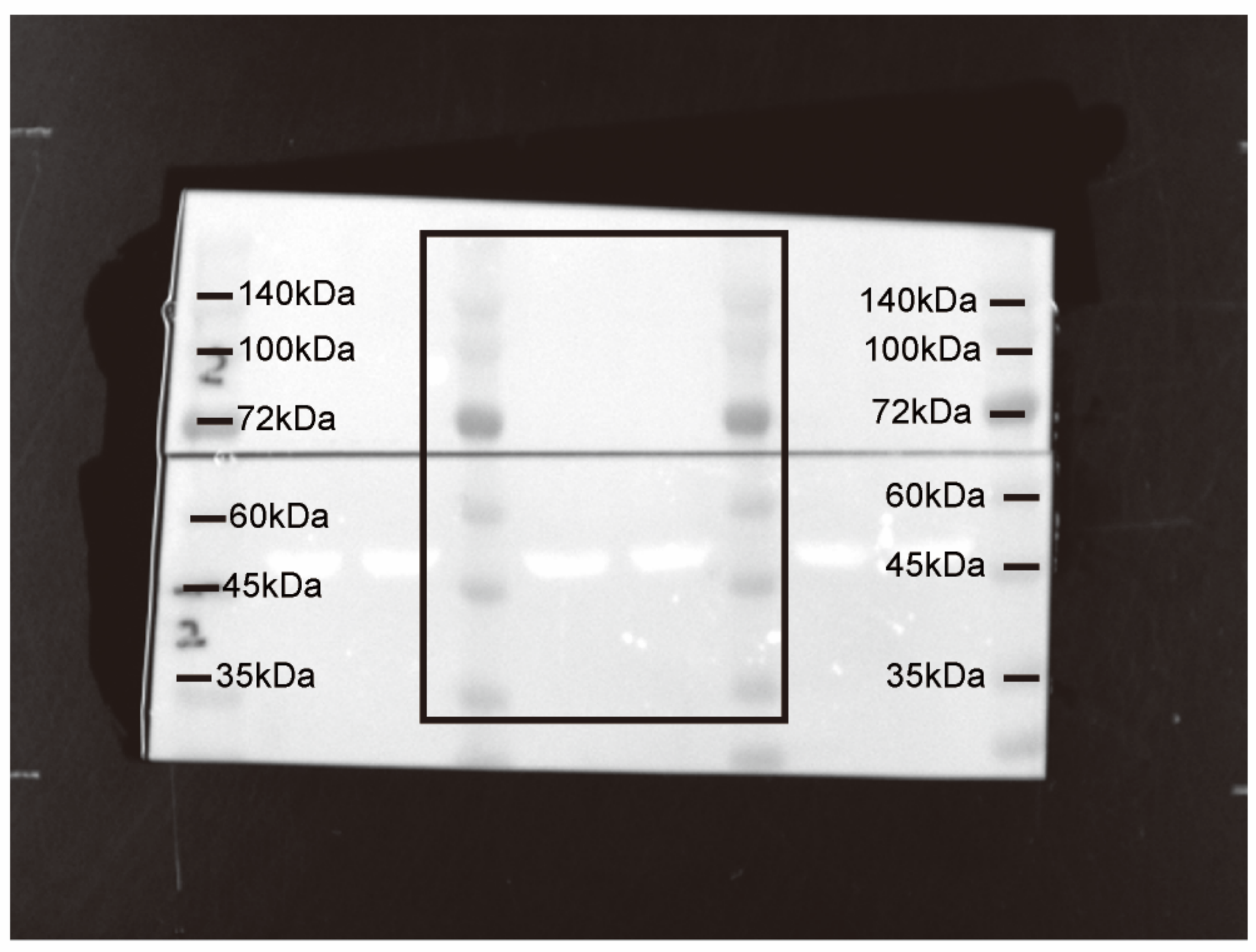

Figure	Merged	Stiched full image

Fig.2A

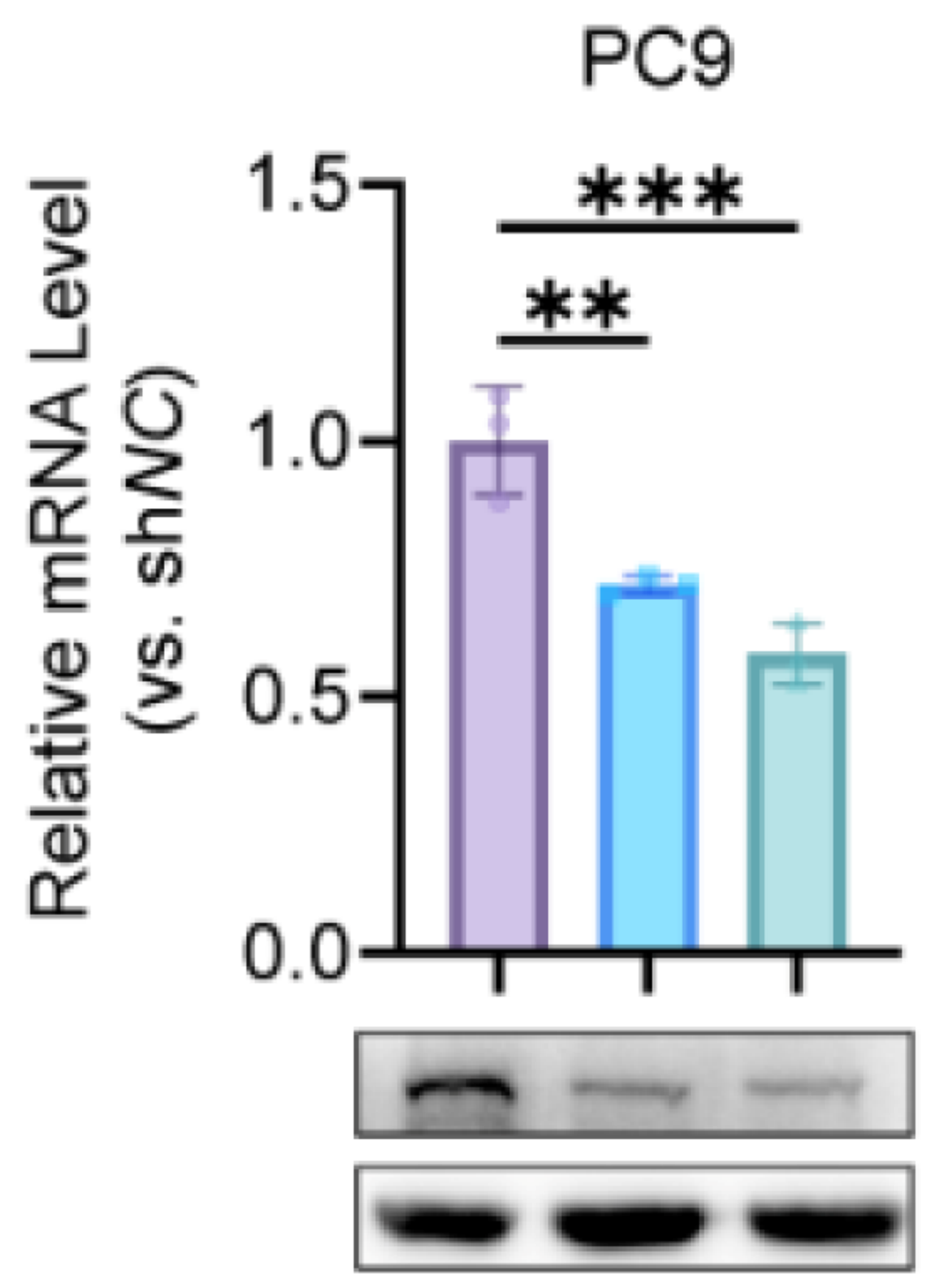

PARP10

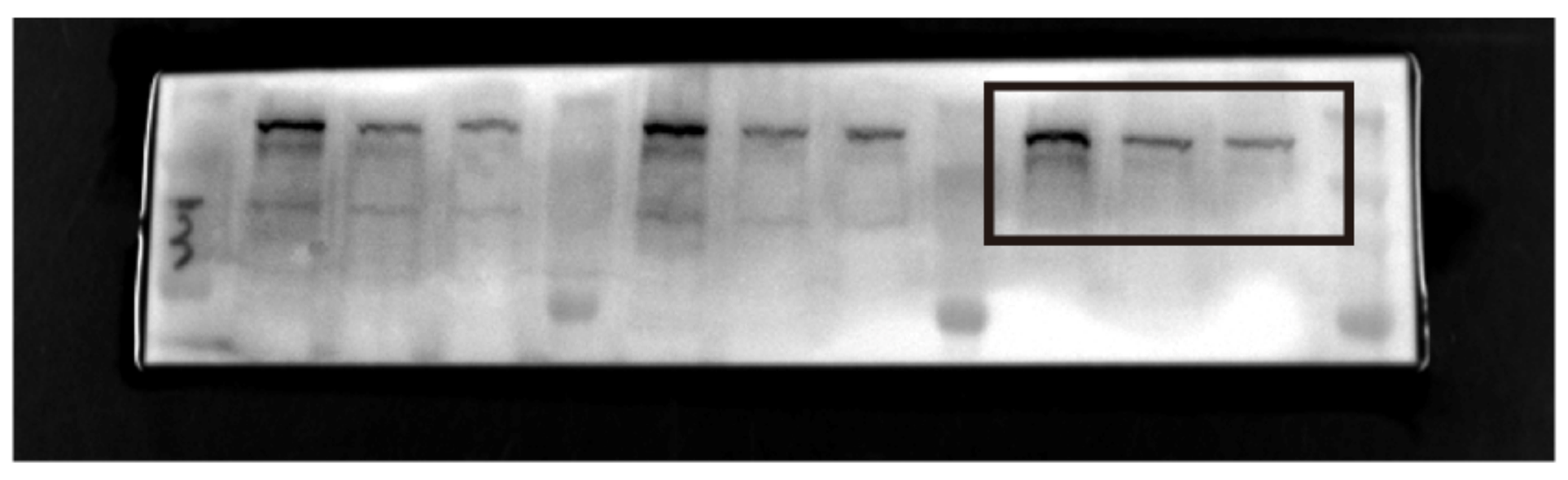

β-actin

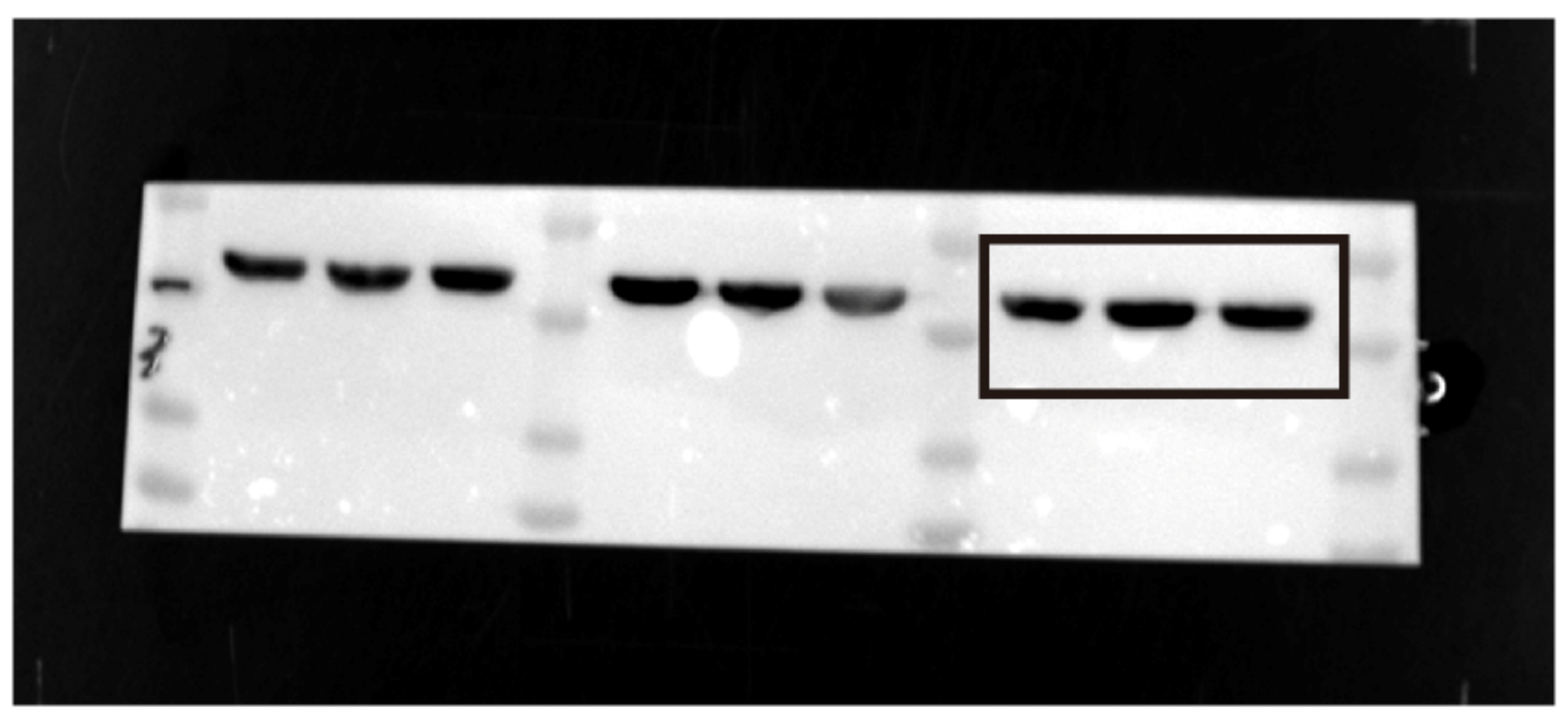

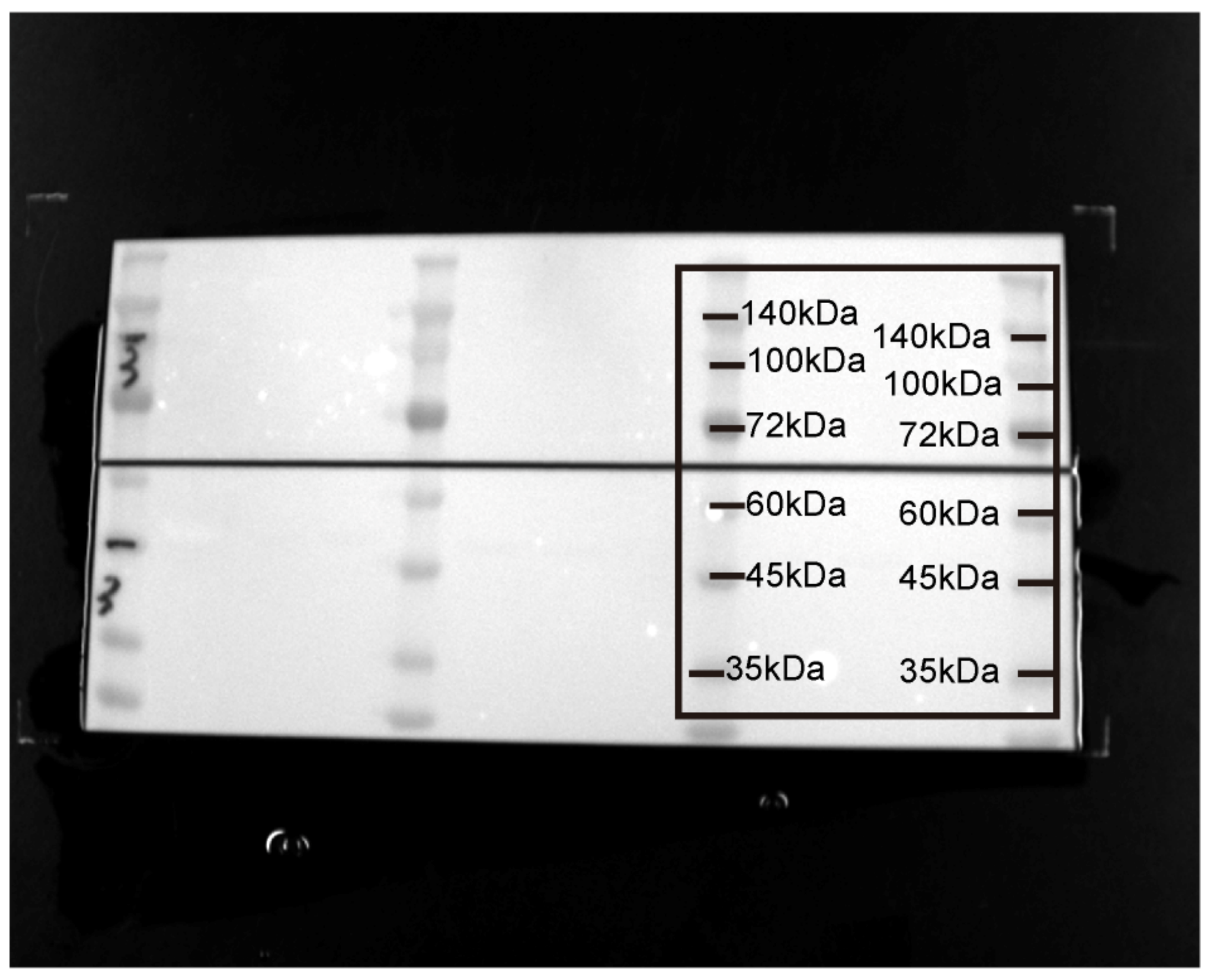

Fig.2A

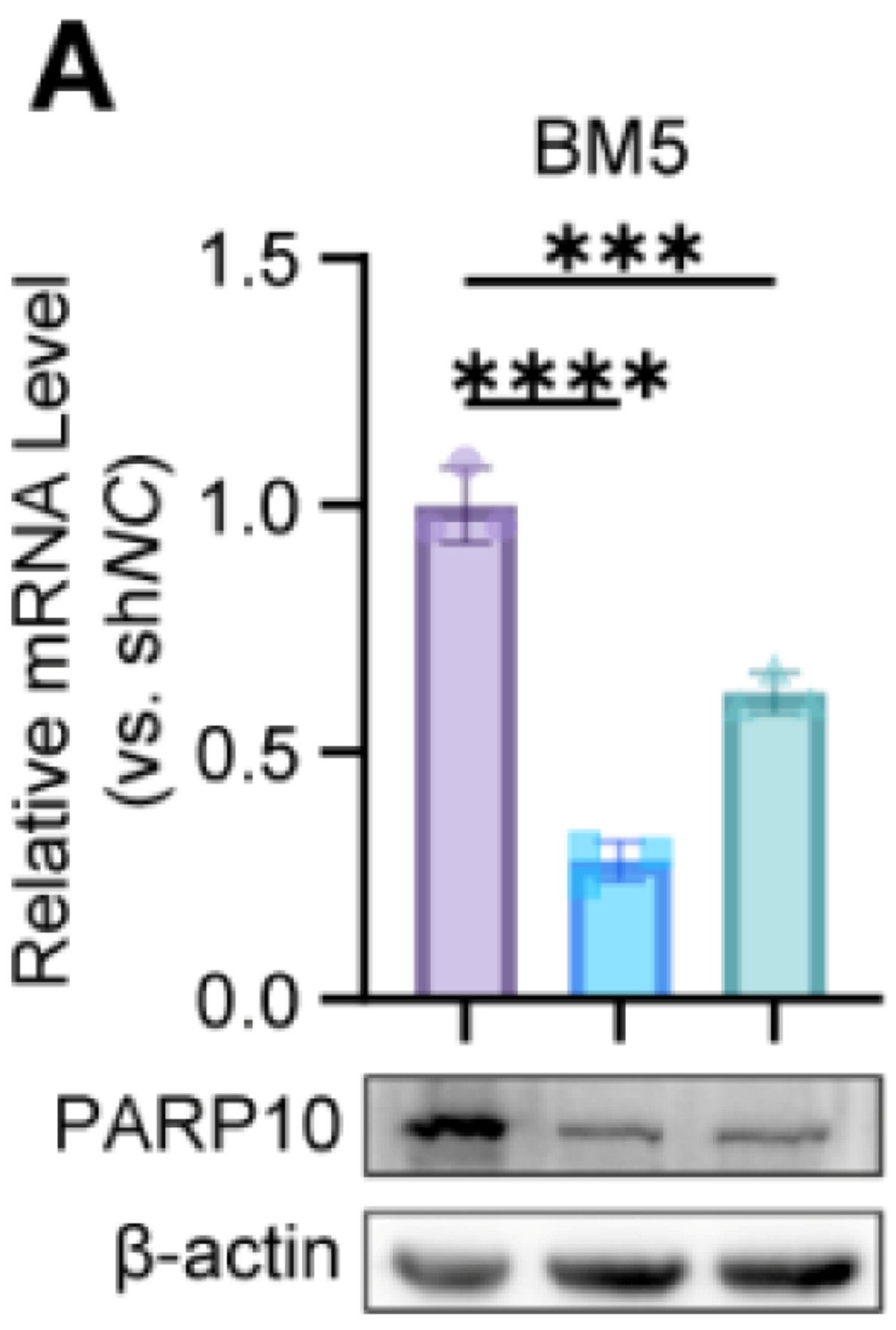

PARP10

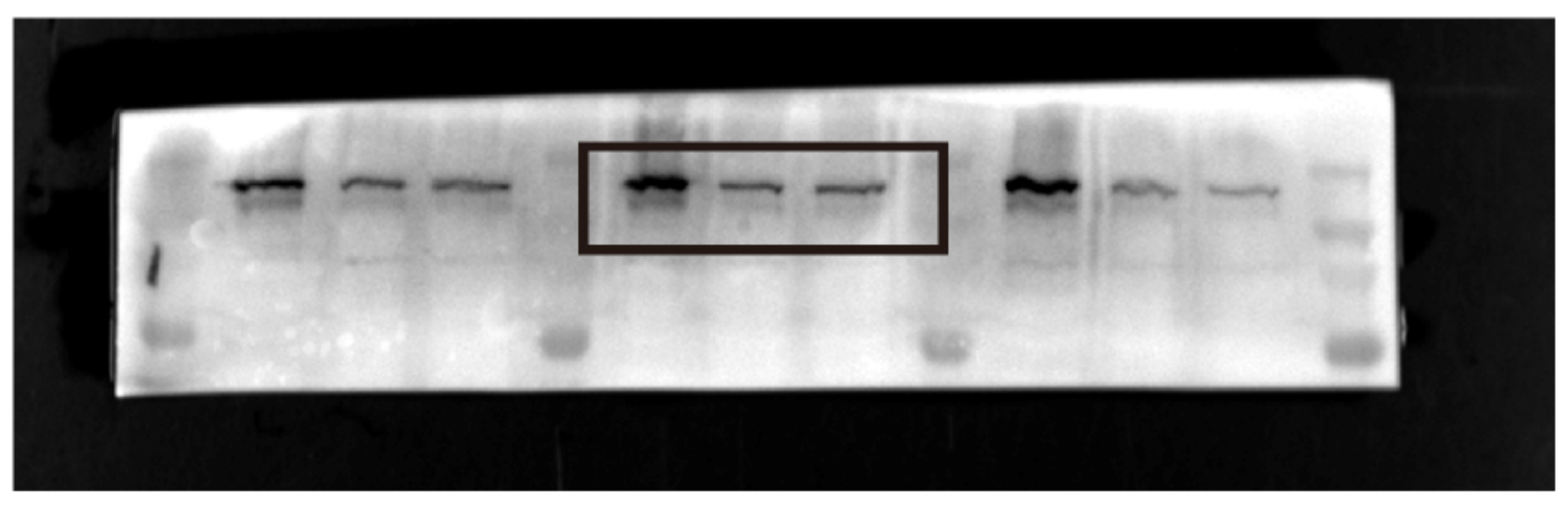

β-actin

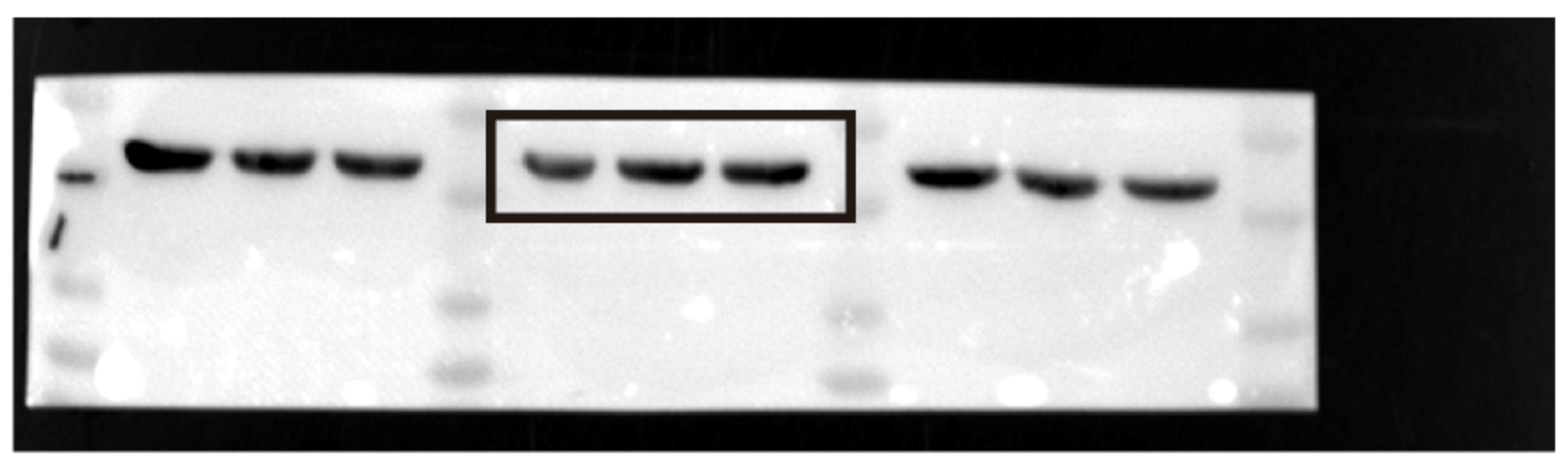

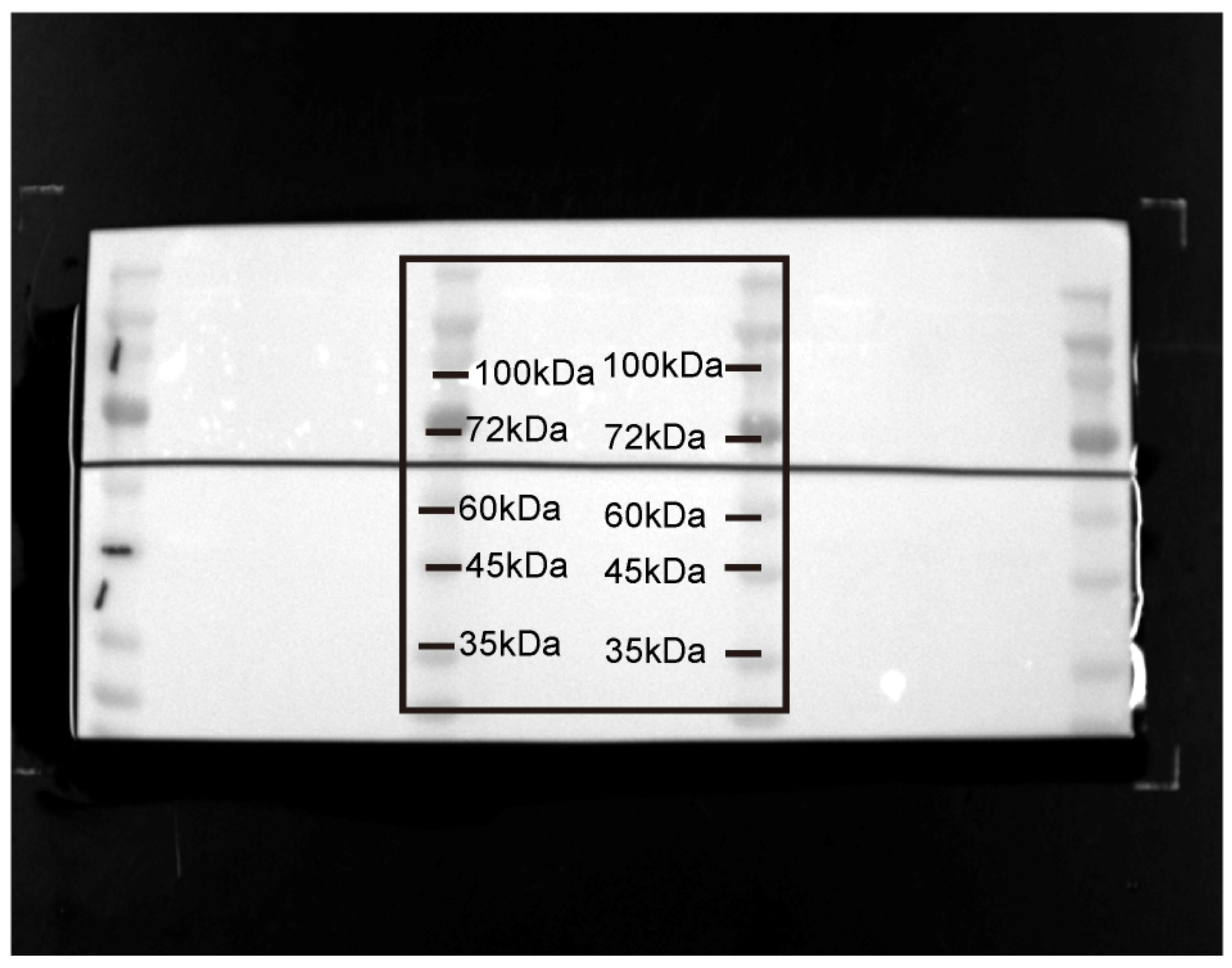

Fig.5I

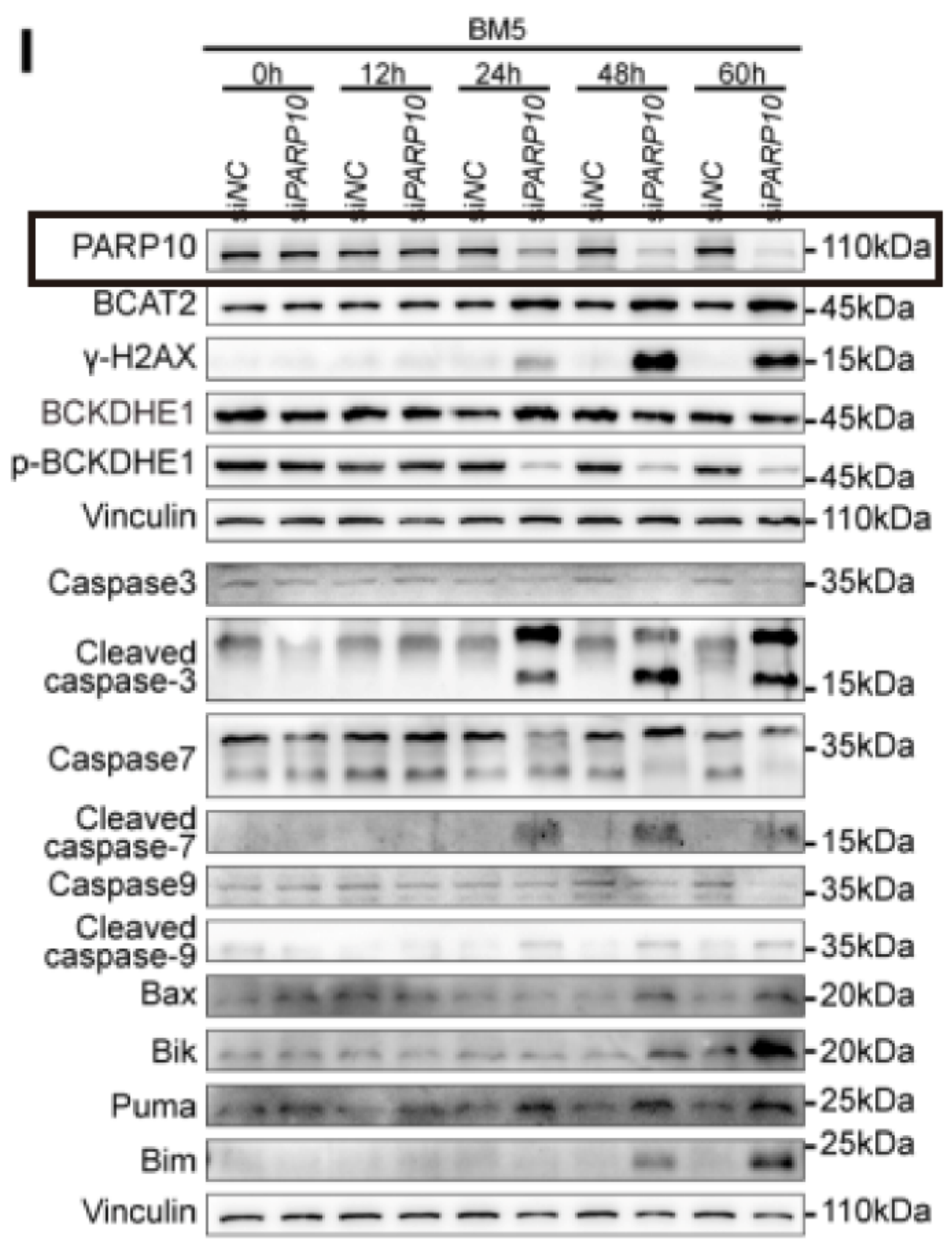

PARP10

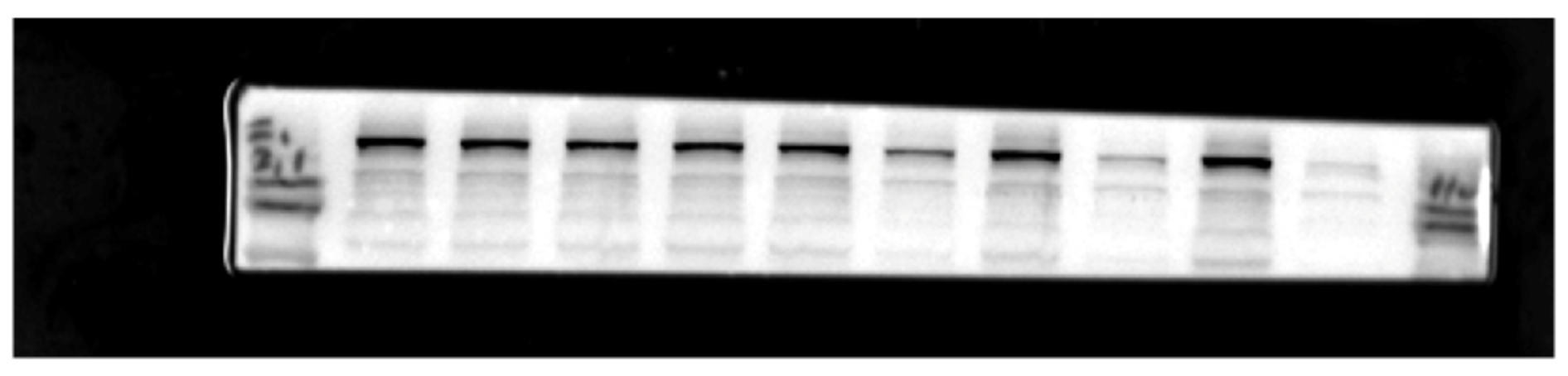

GAPDH

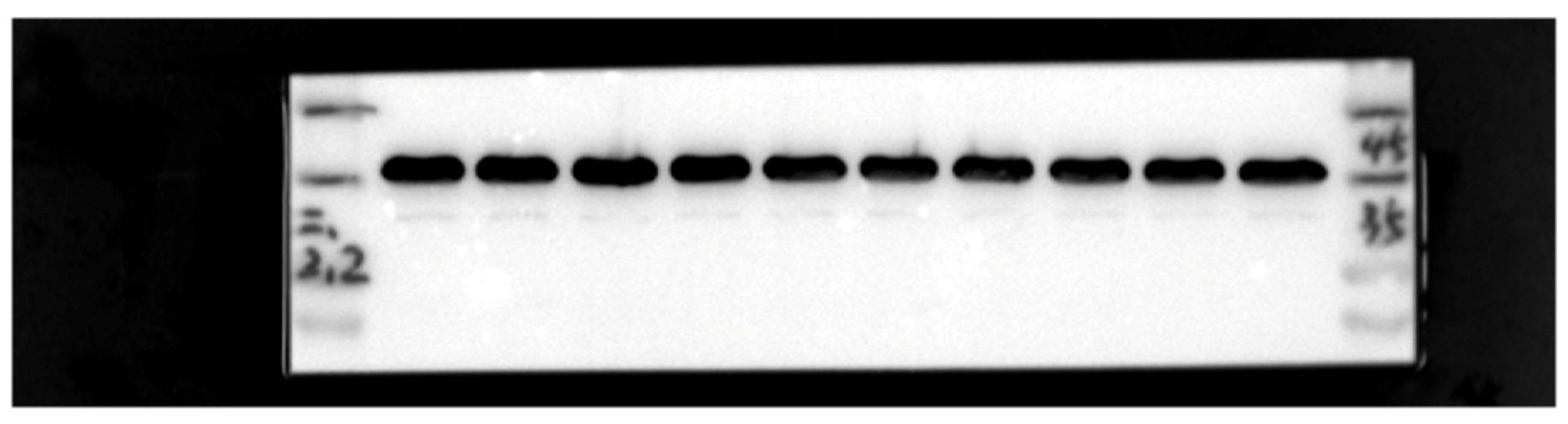

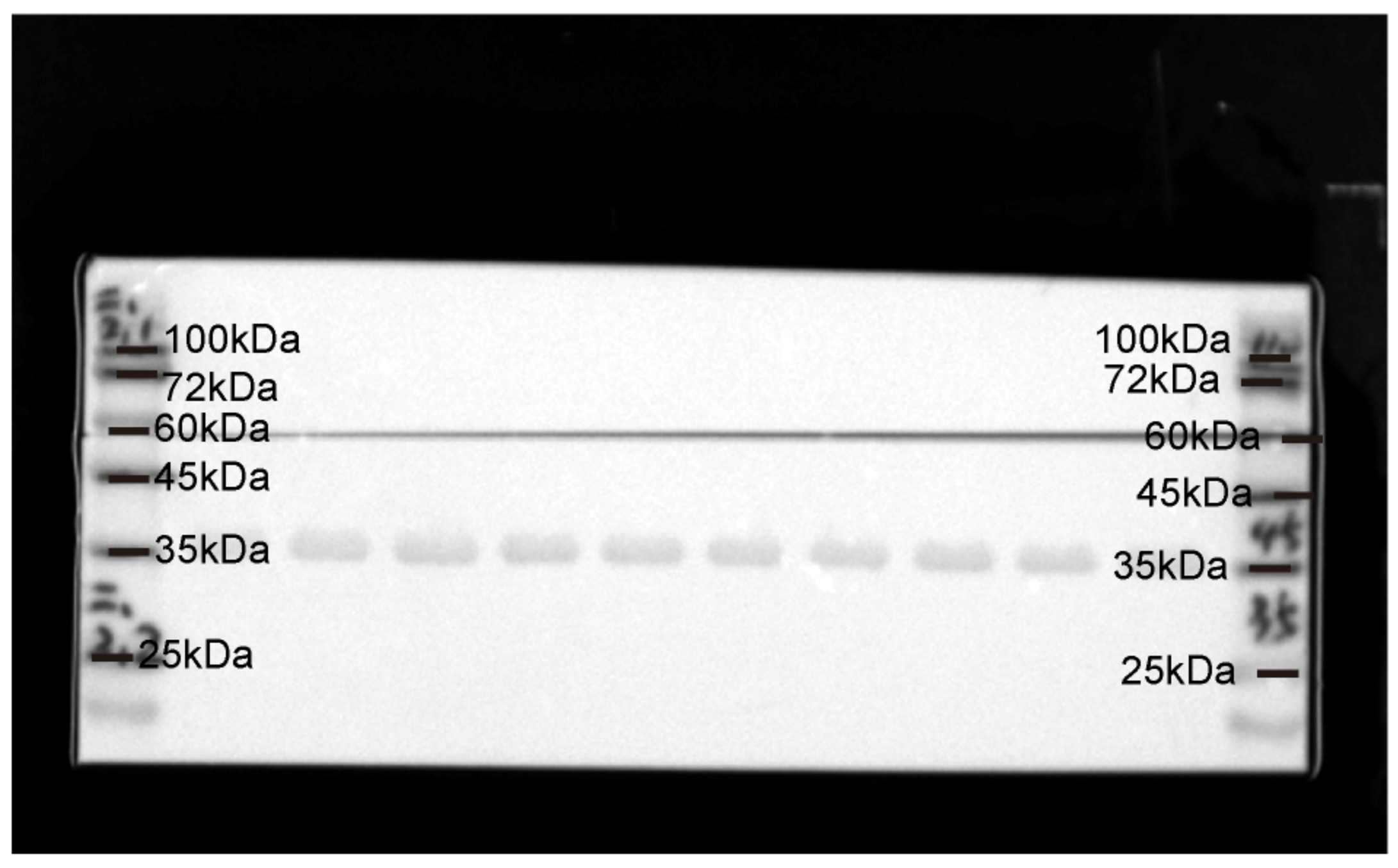

Fig.5I

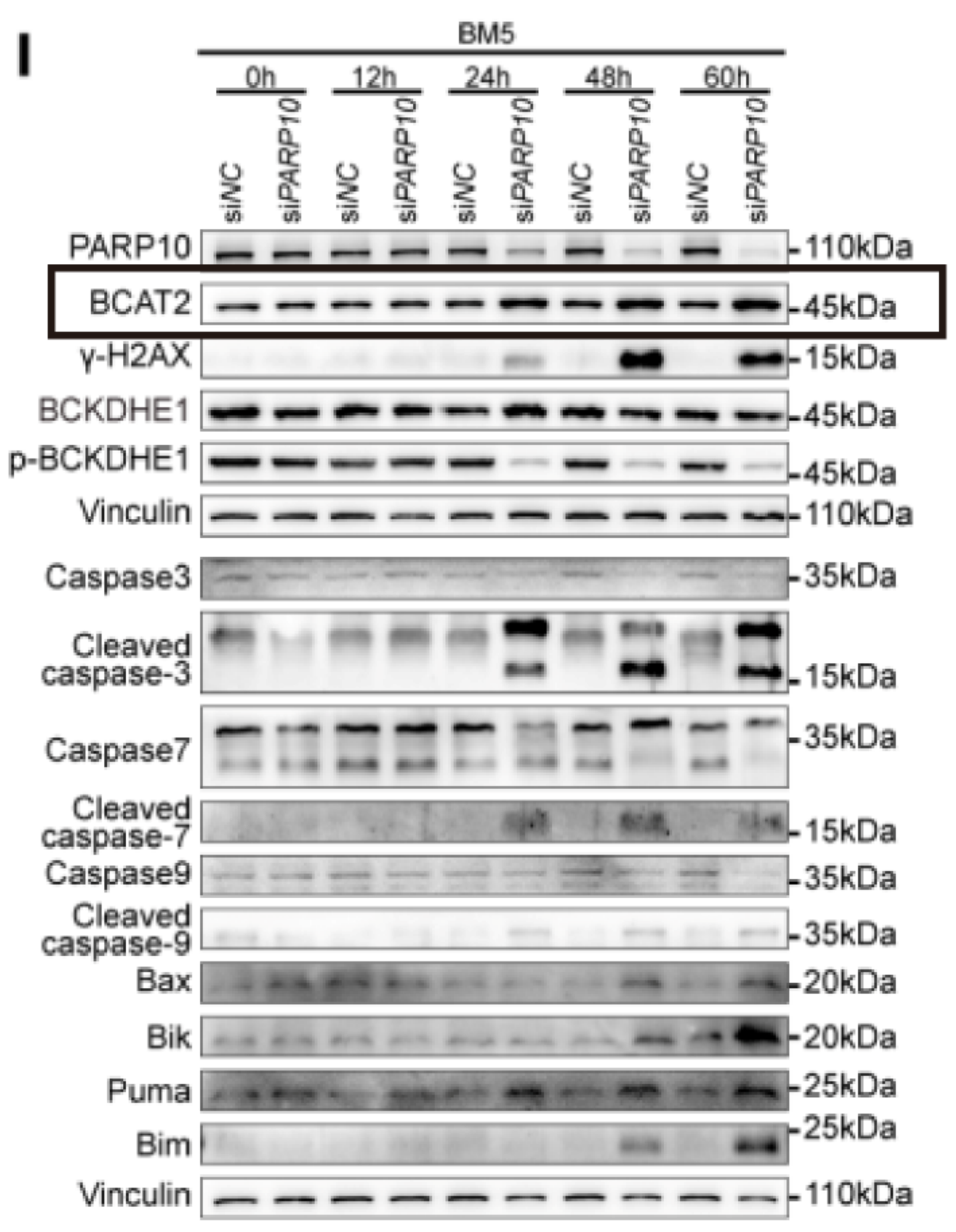

BCAT2

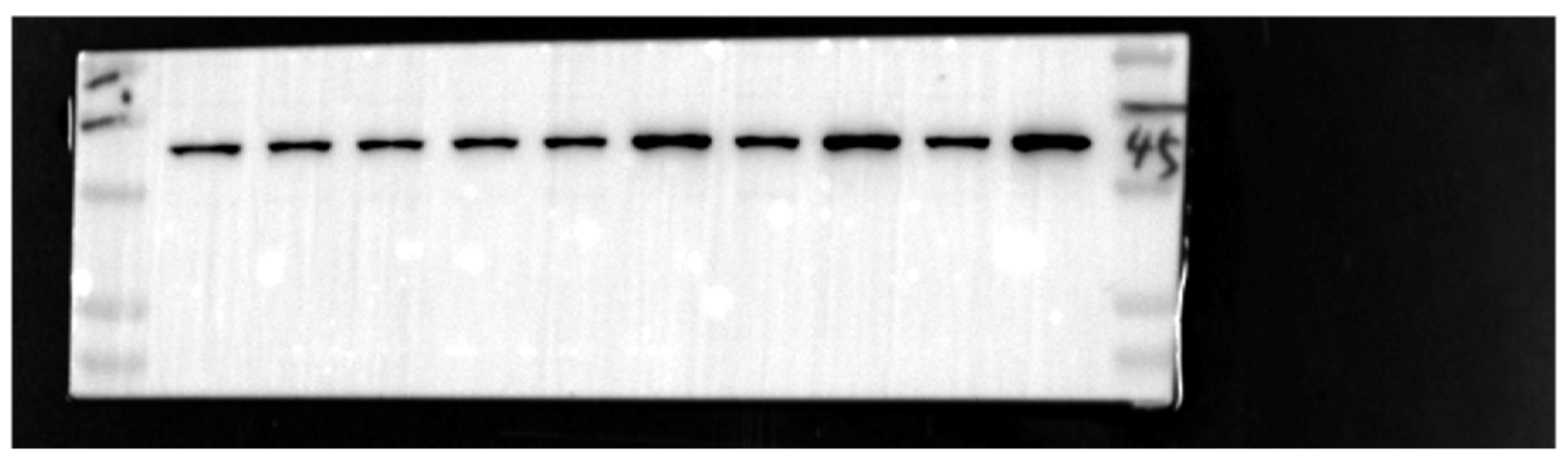

Vinculin

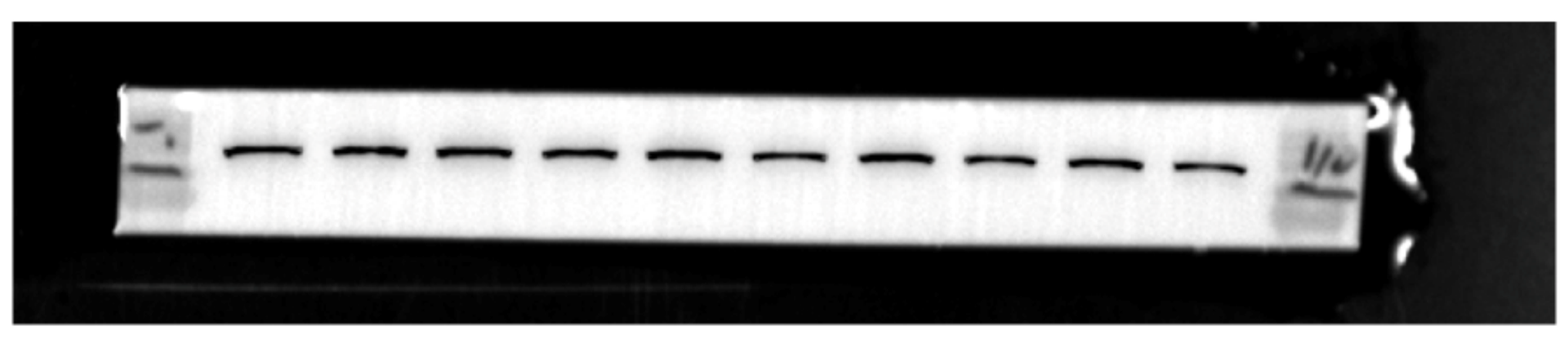

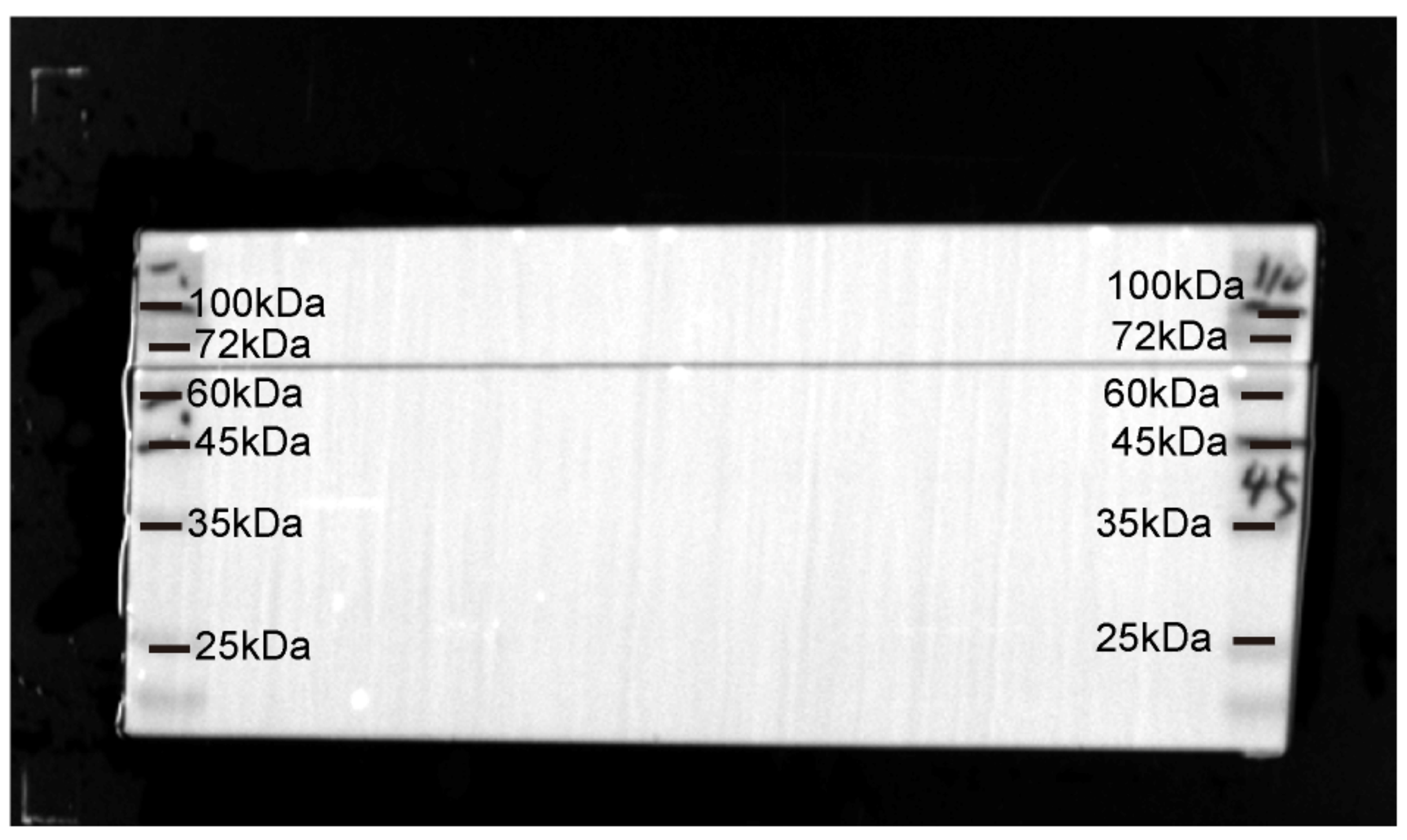

Figure	Merged	Stiched full image

Fig.5I

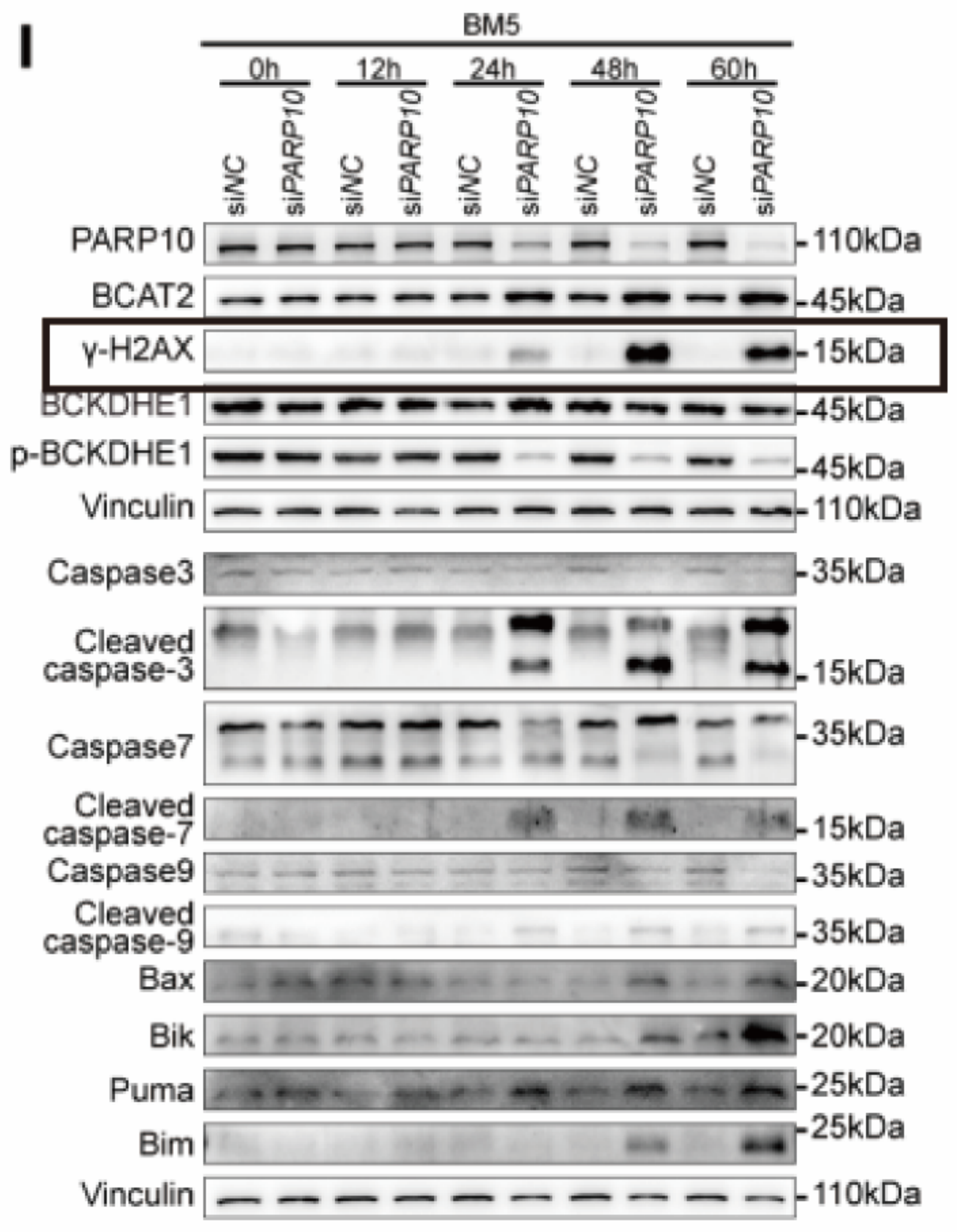

γ-H2AX

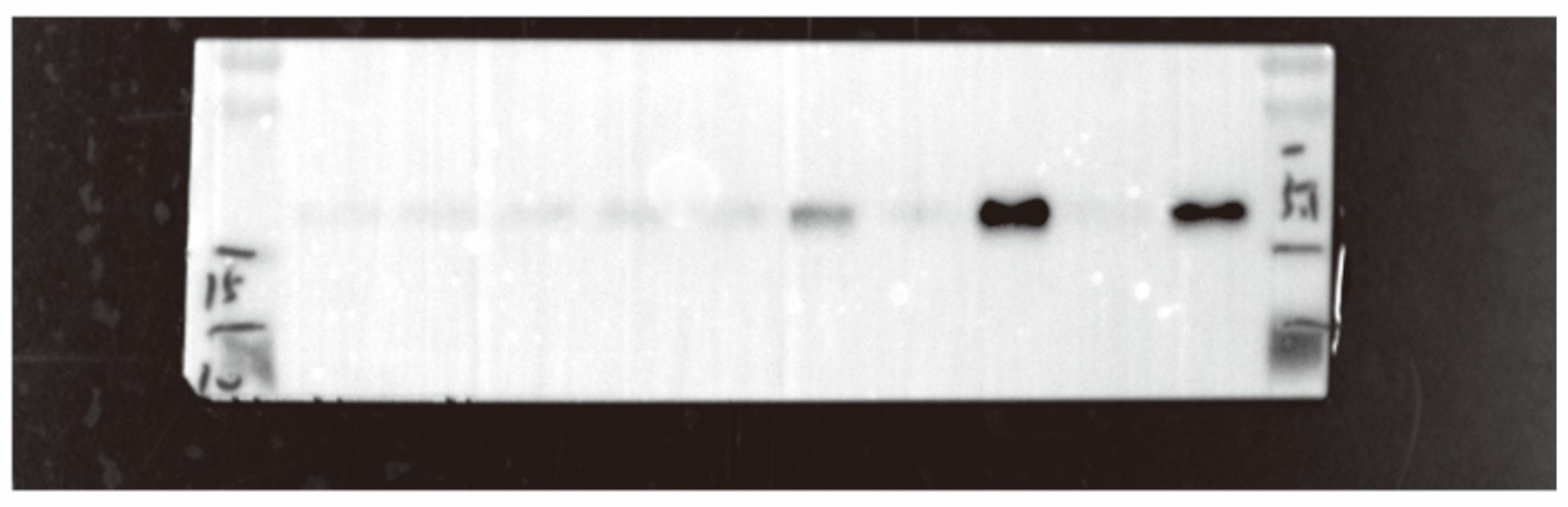

β-actin

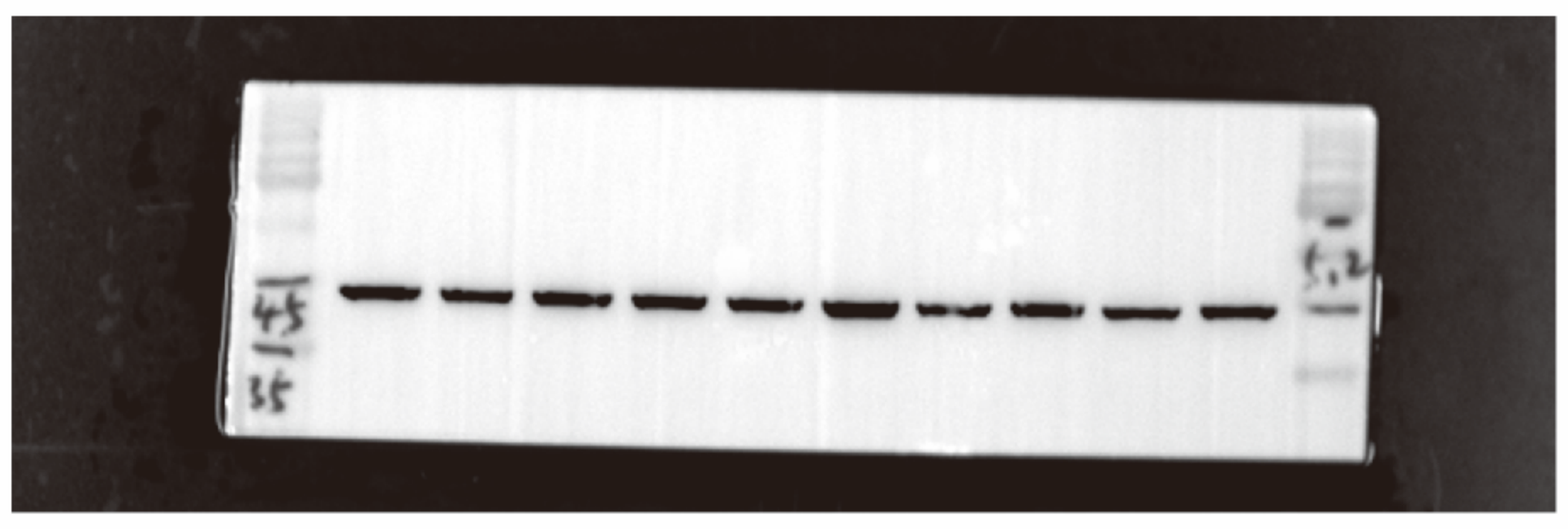

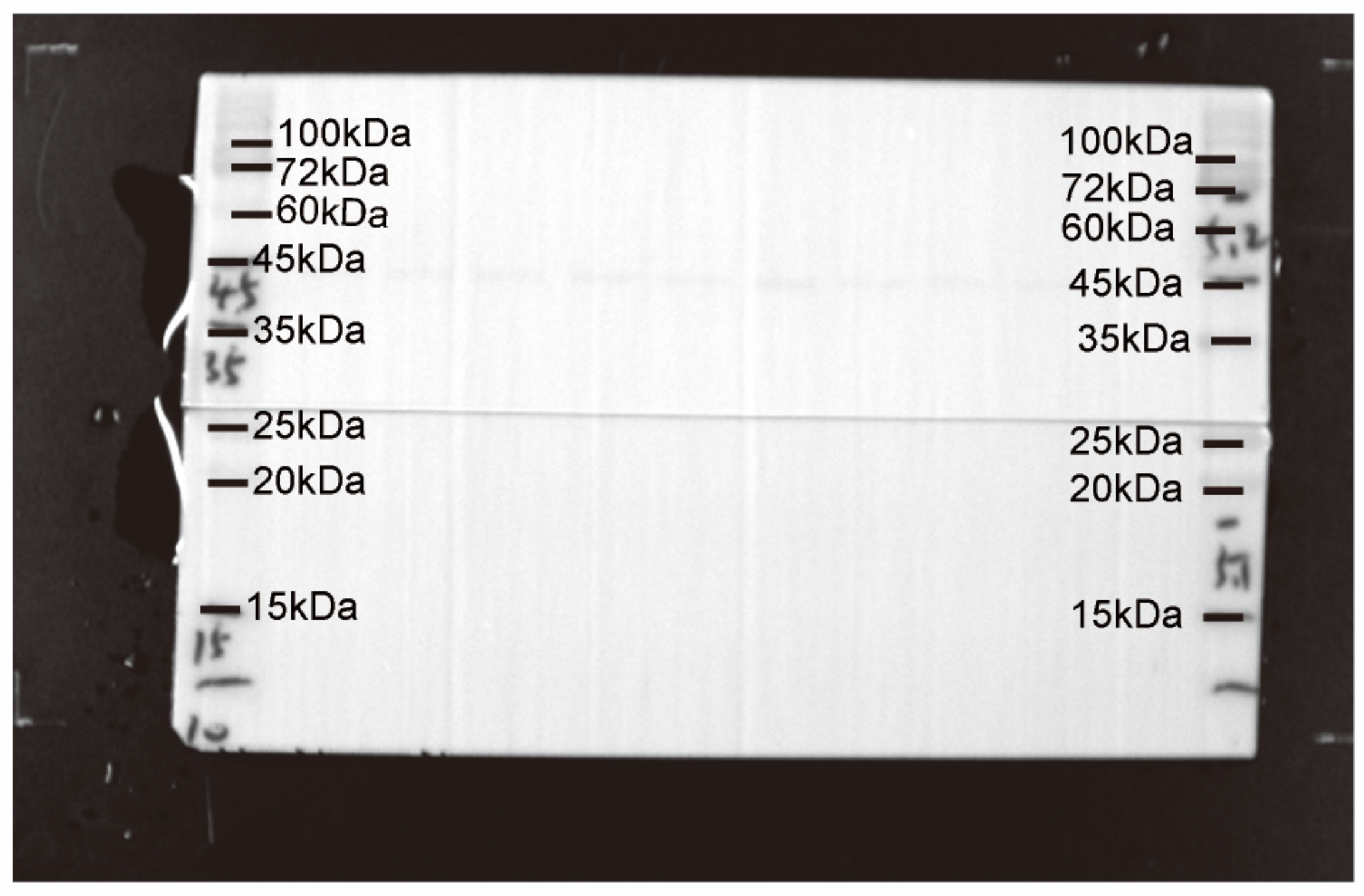

Fig.5I

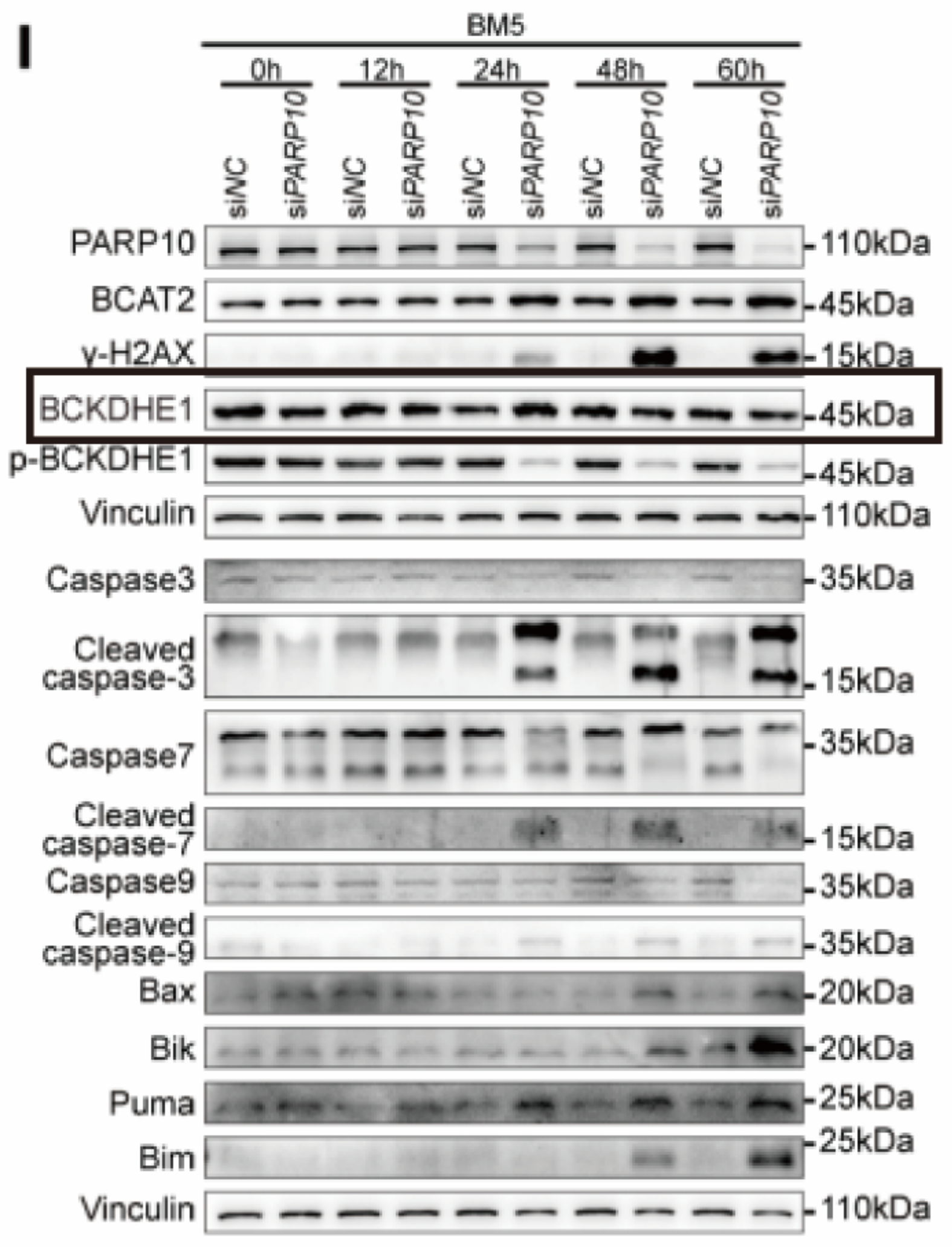

BCKDHE1

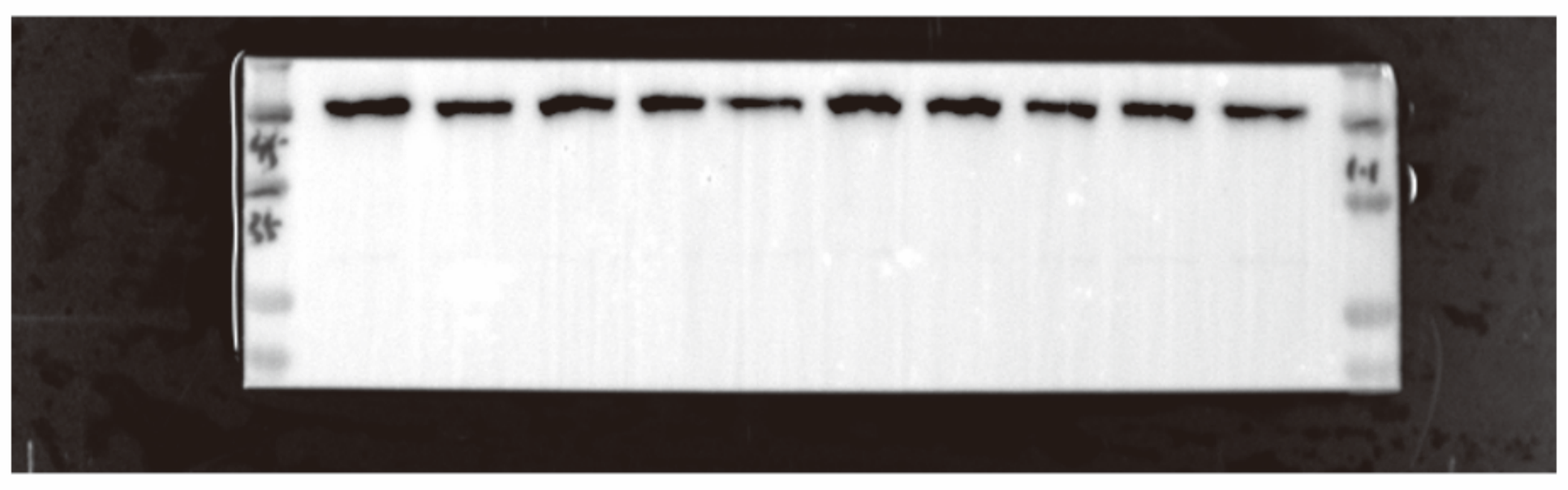

Vinculin

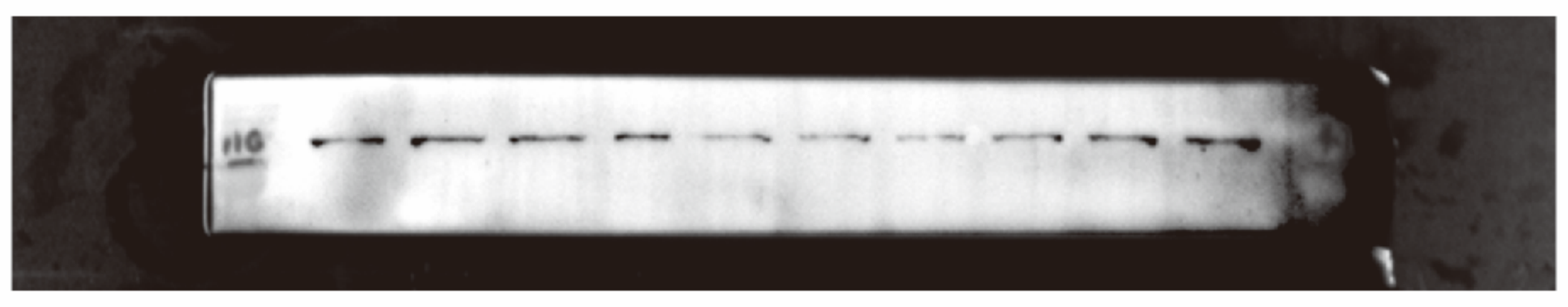

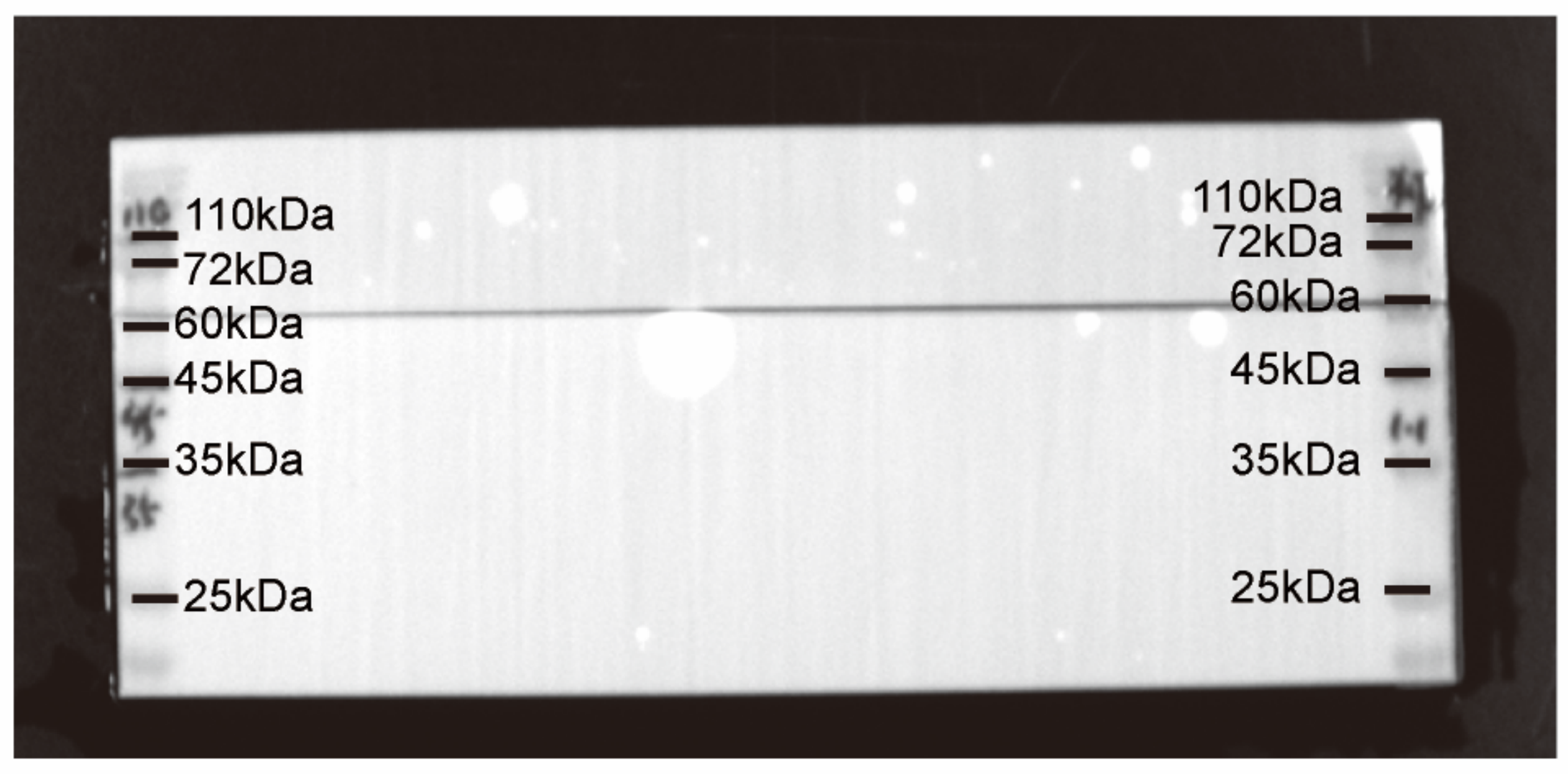

Fig.5I

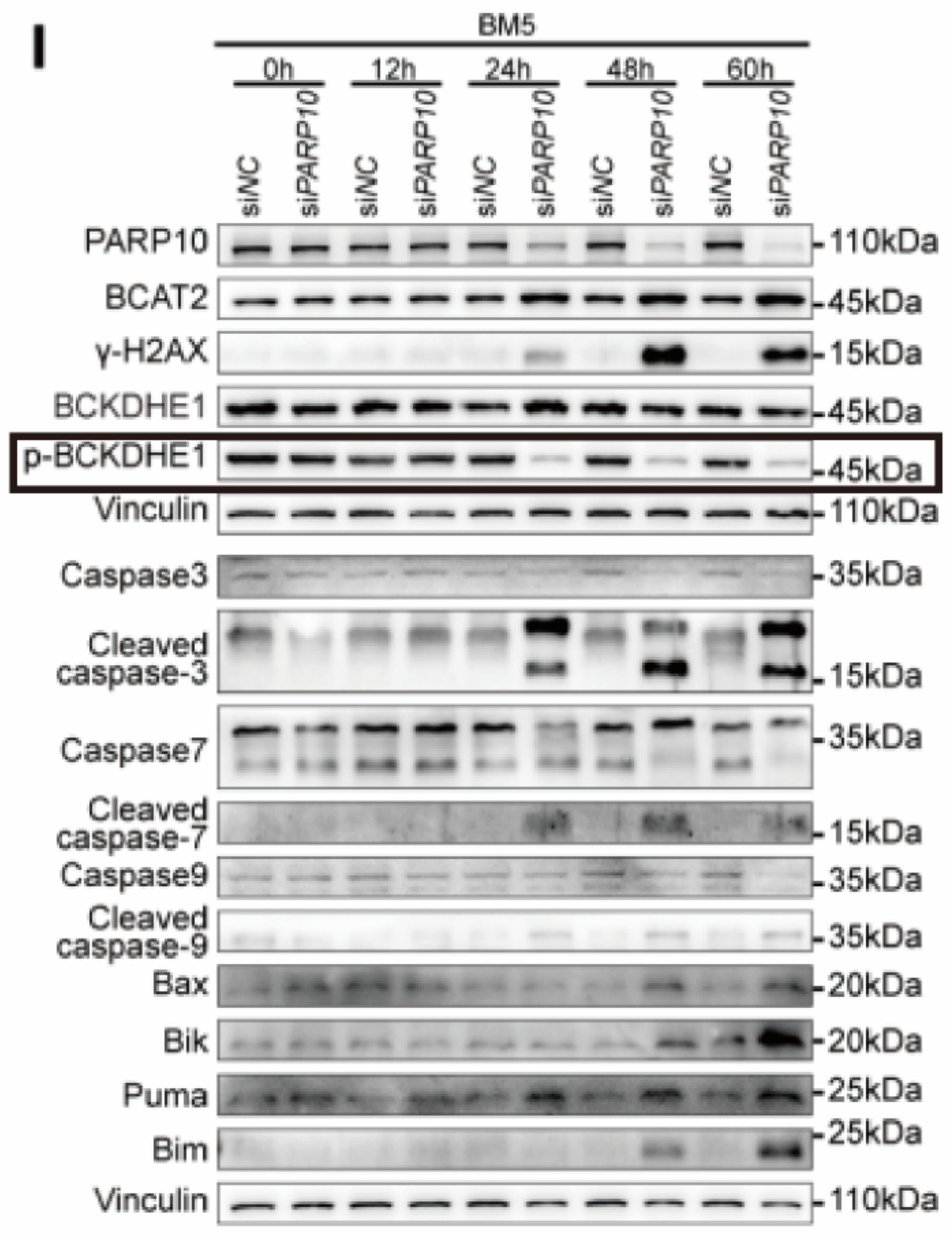

p-BCKDHE1

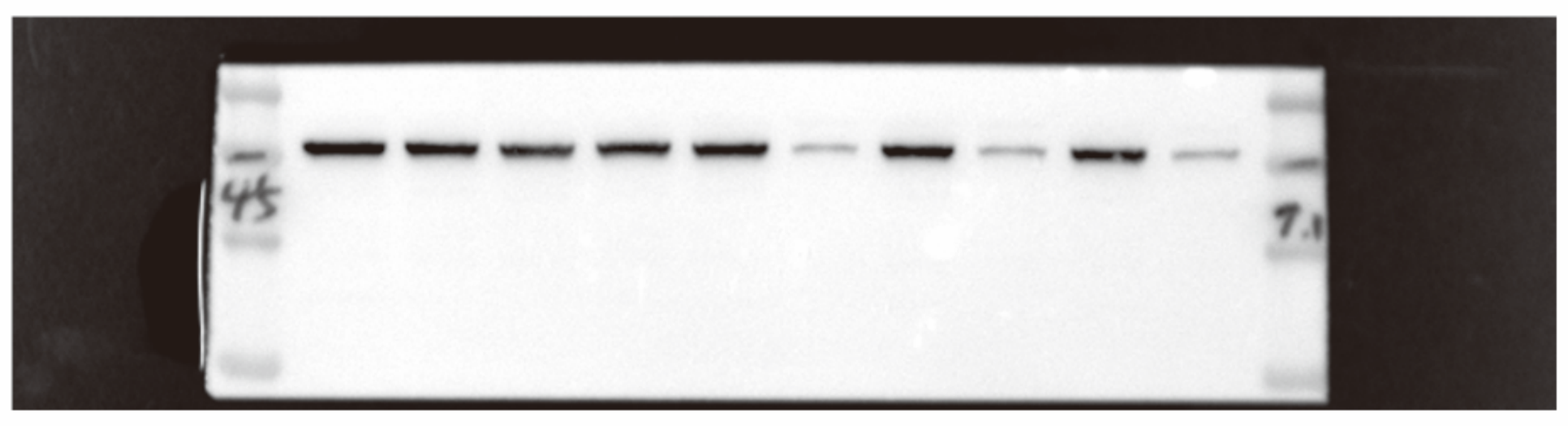

Vinculin

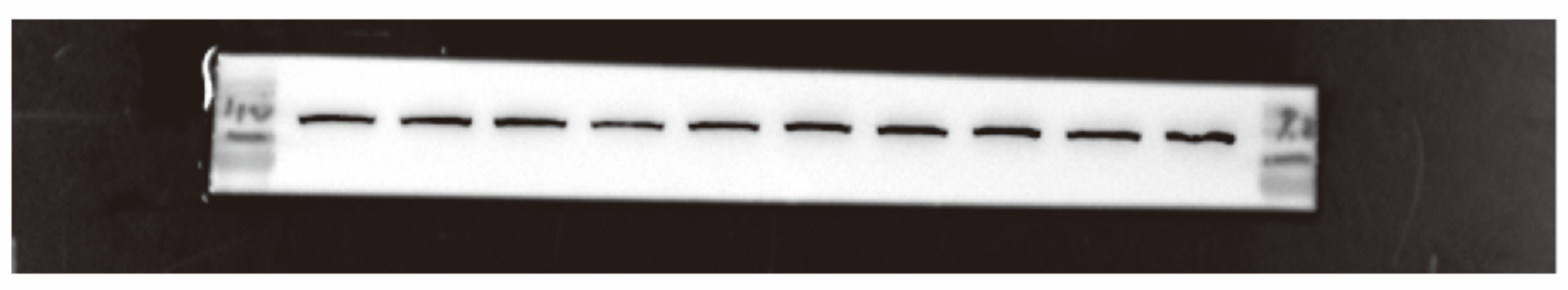

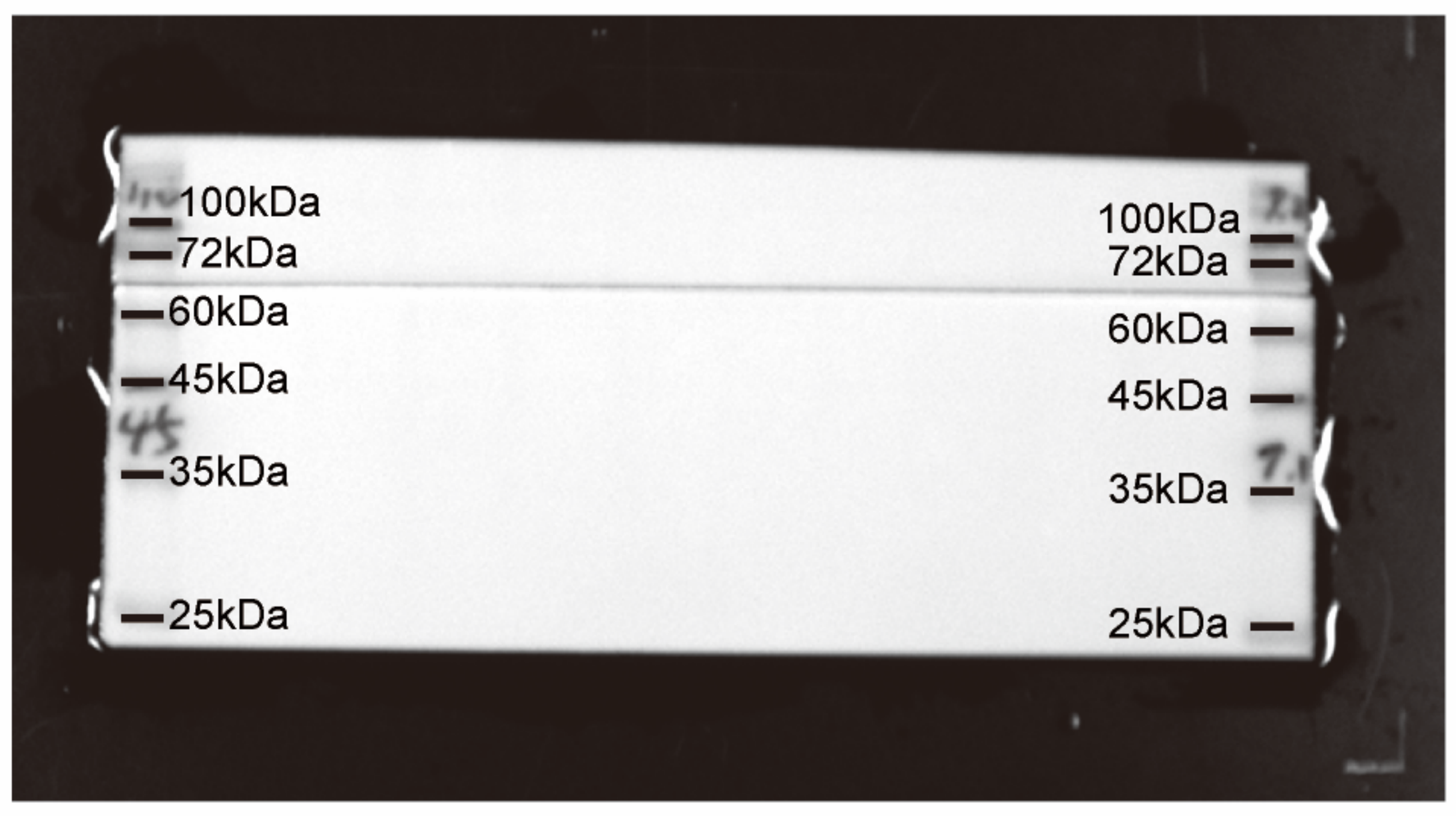

Fig.5I

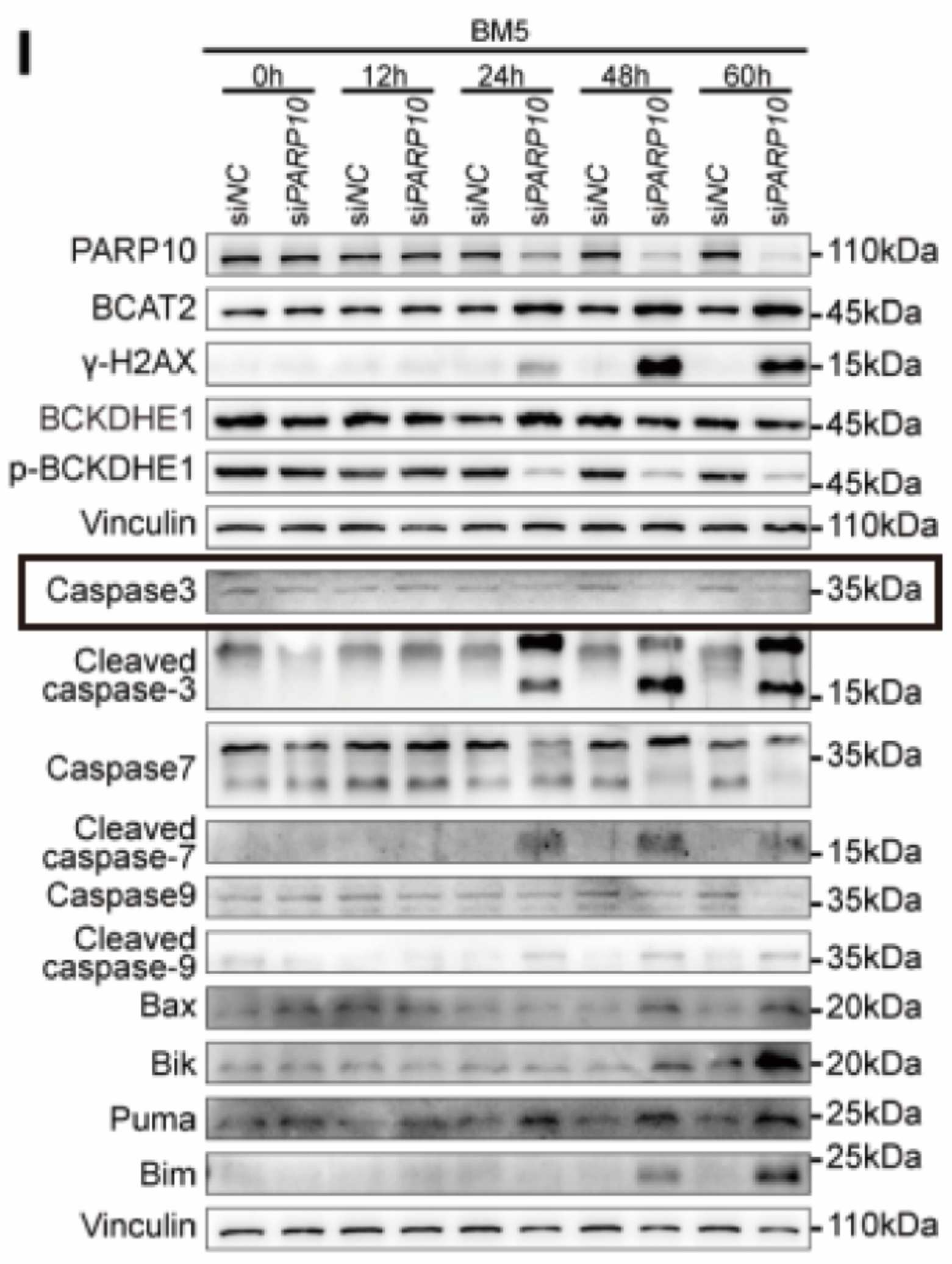

Caspase3

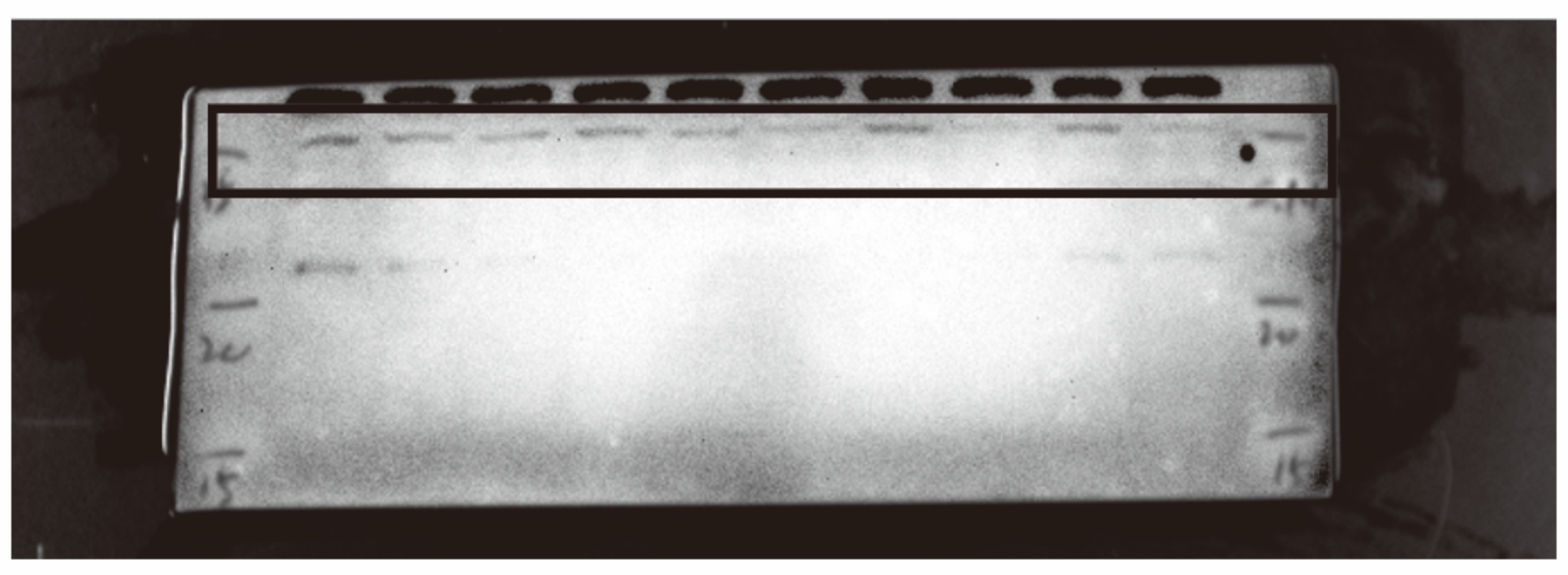

Vinculin

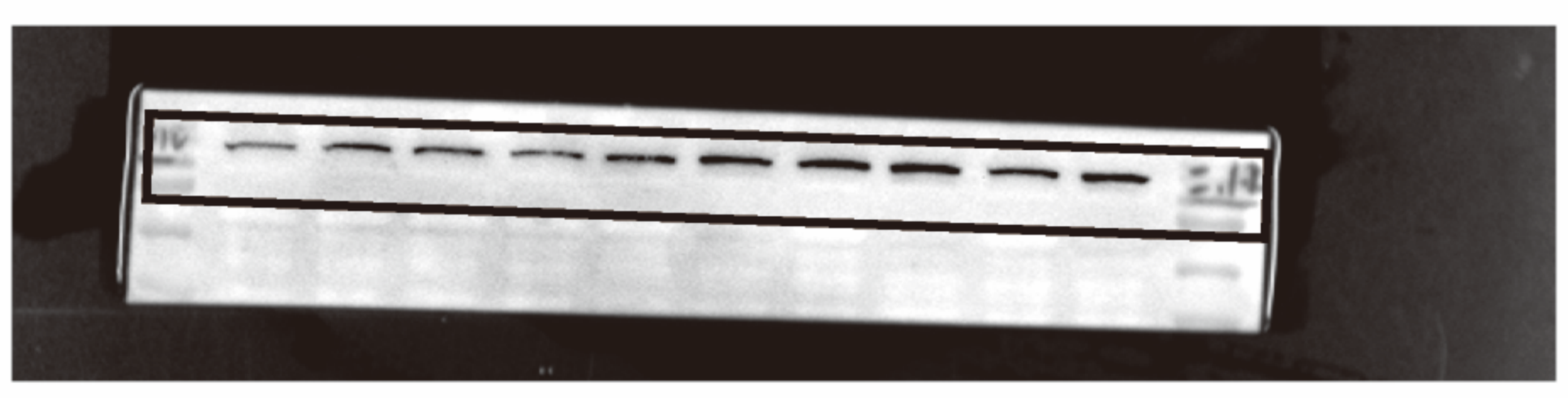

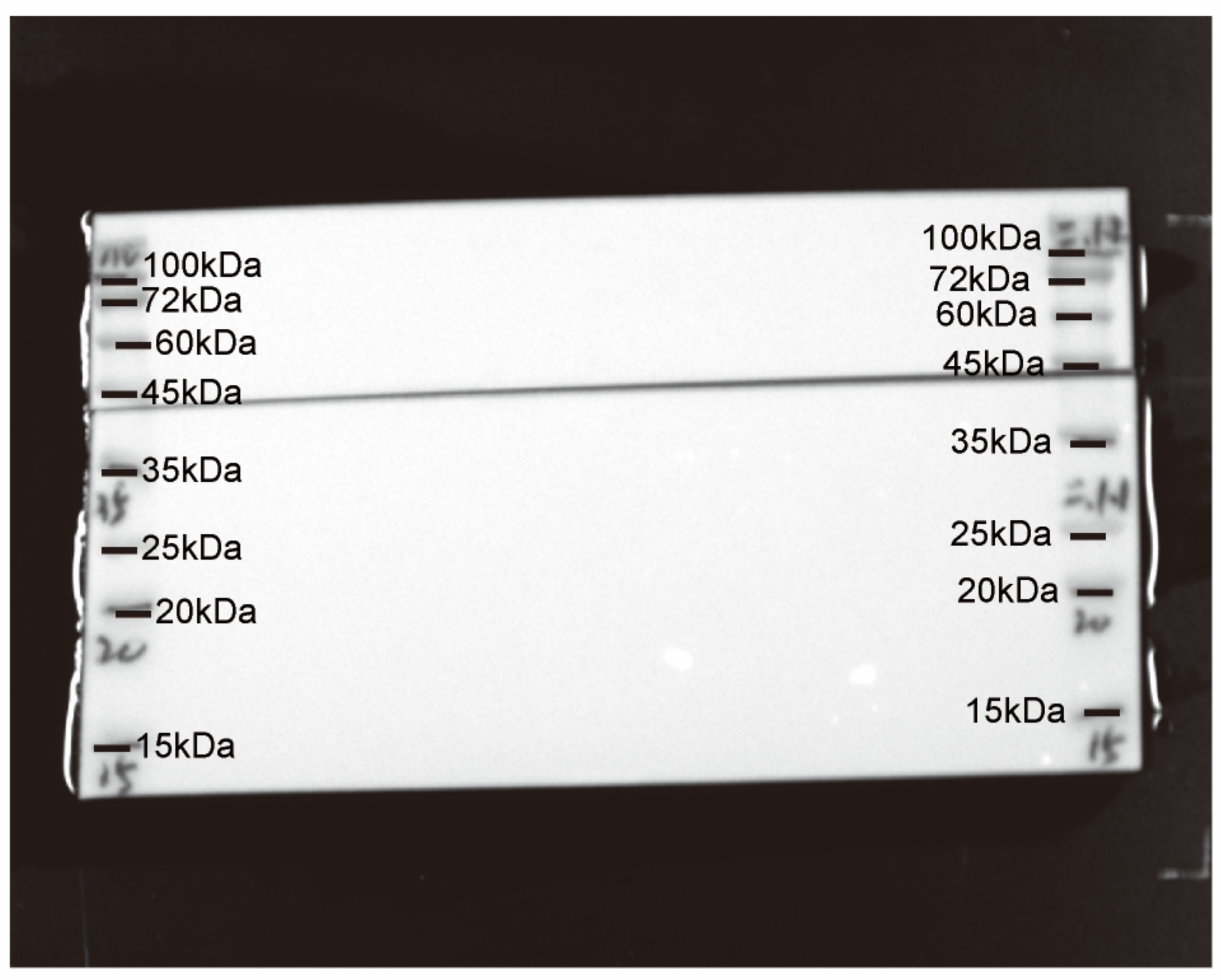

Figure	Merged	Stiched full image

Fig.5l

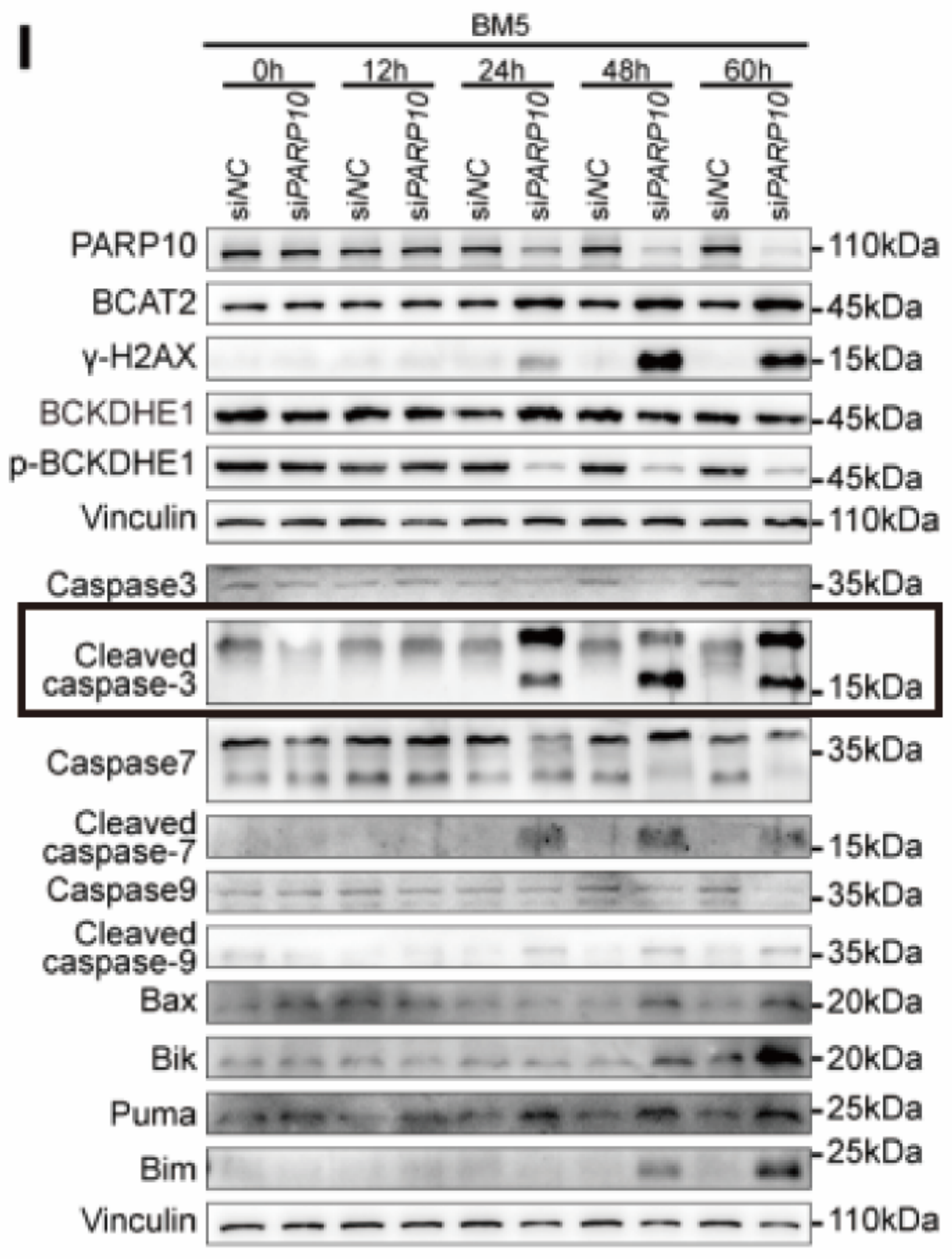

Cleaved caspase3

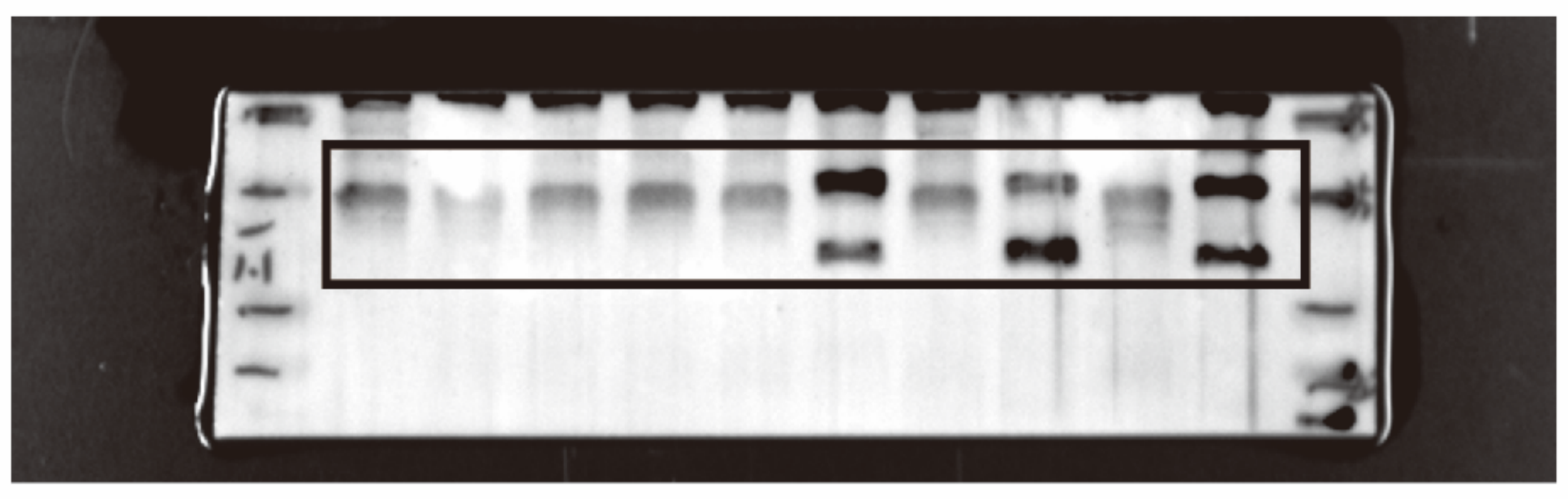

Vinculin

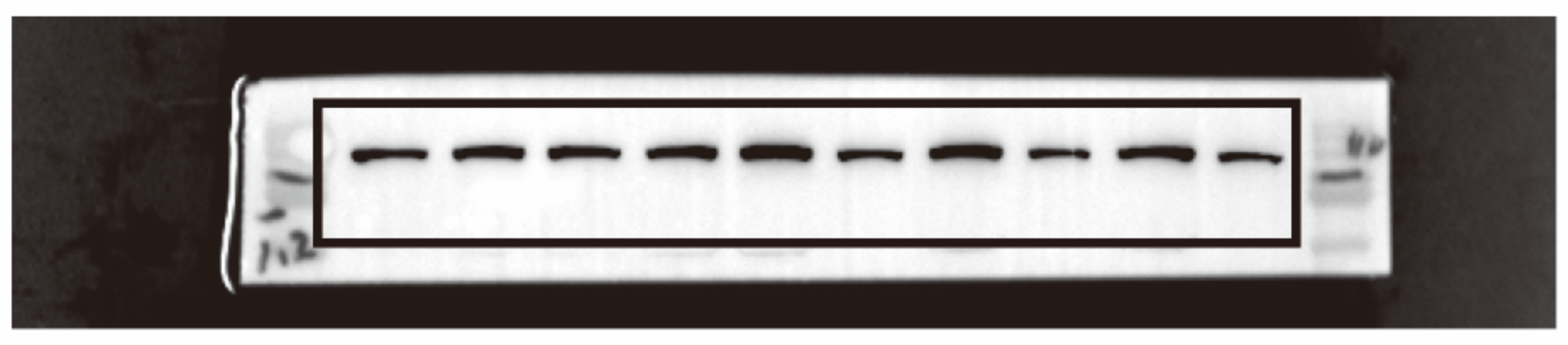

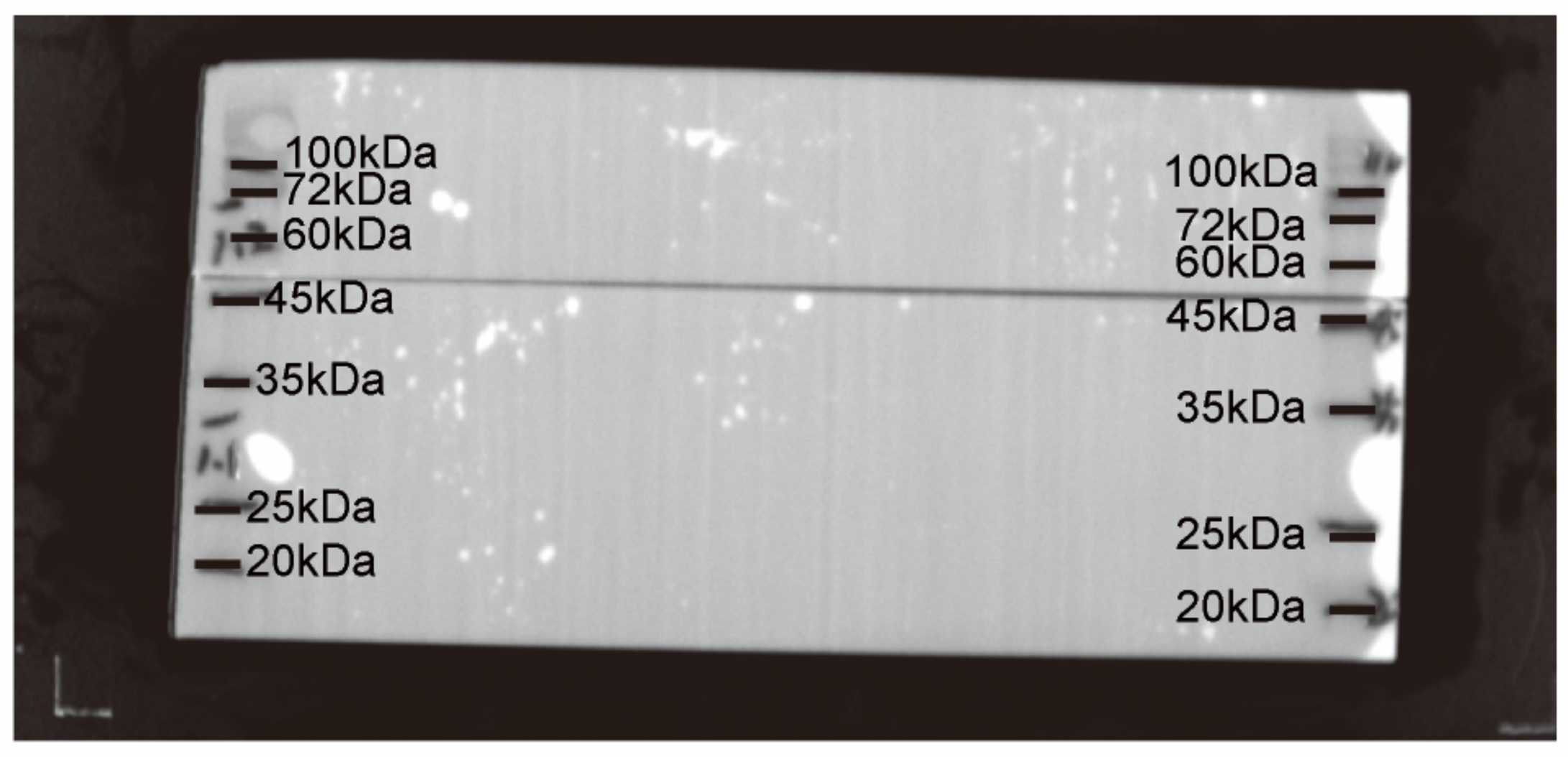

Fig.5l

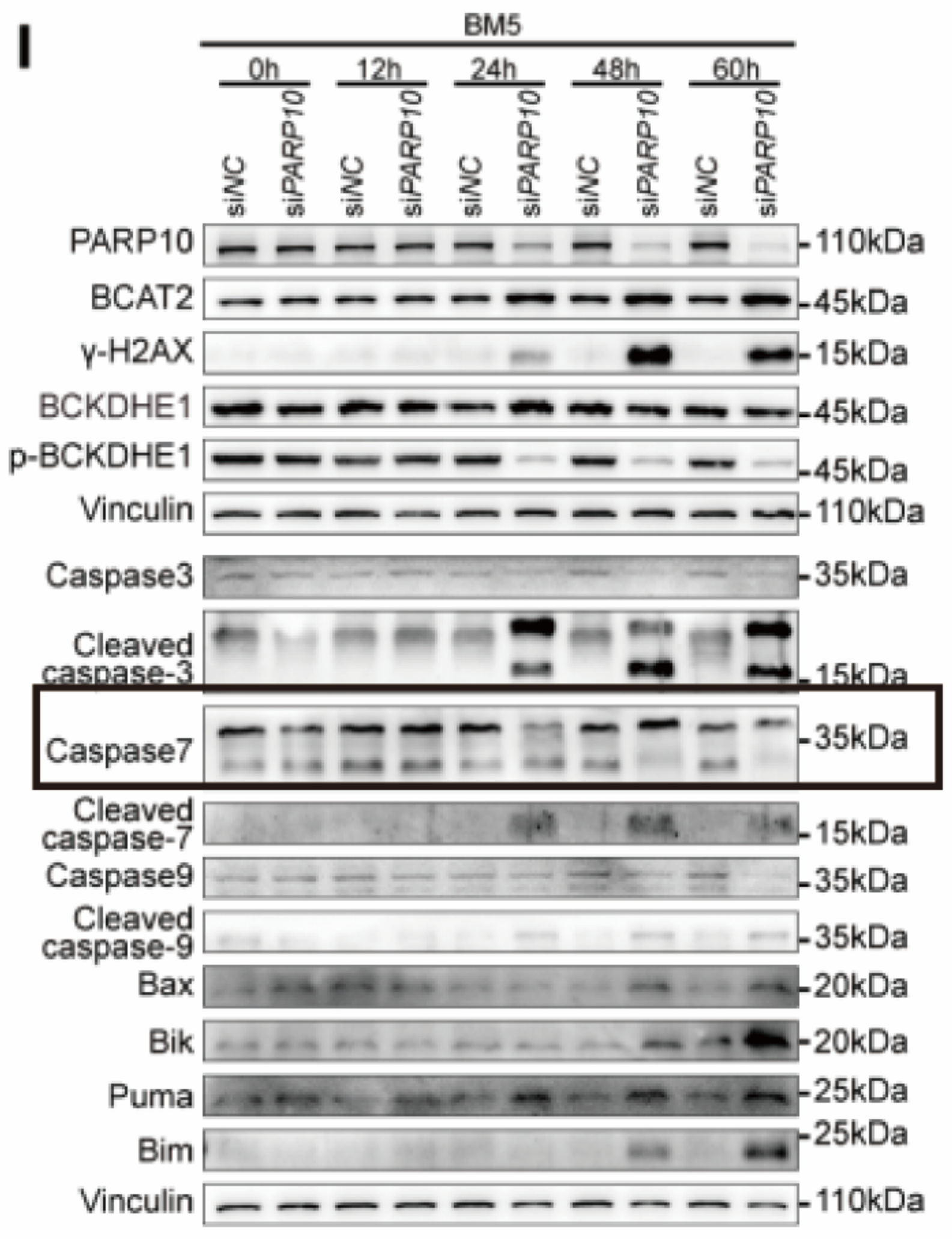

Caspase7

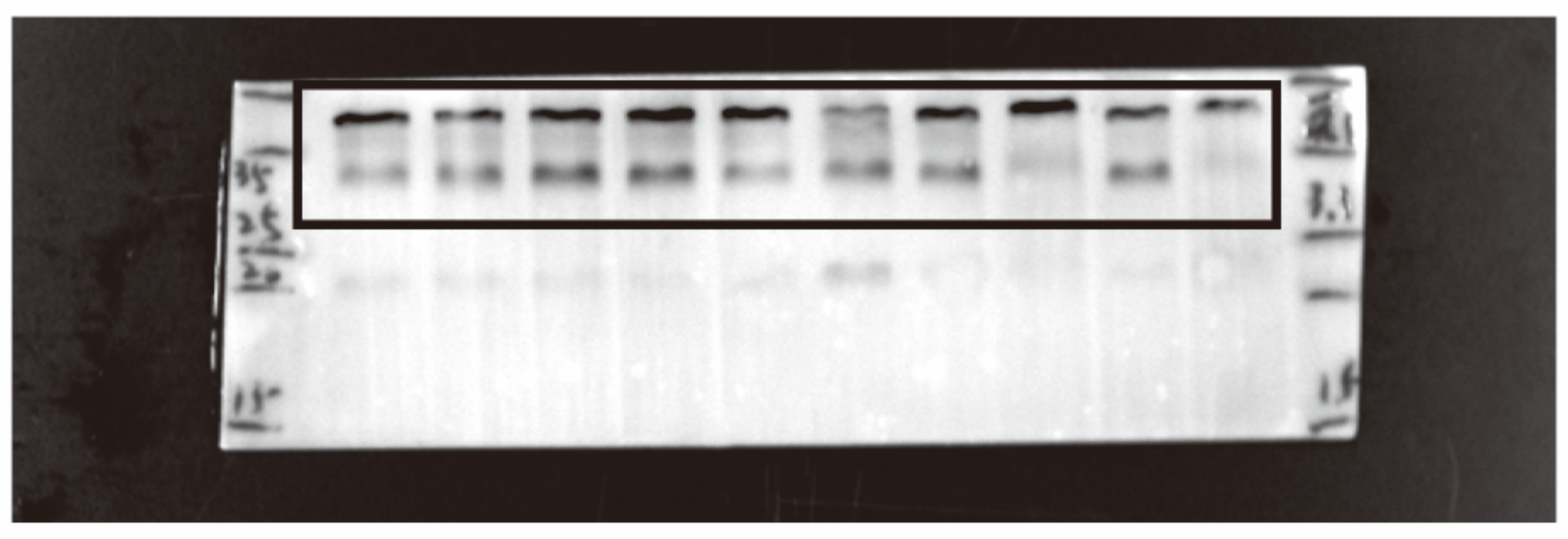

Vinculin

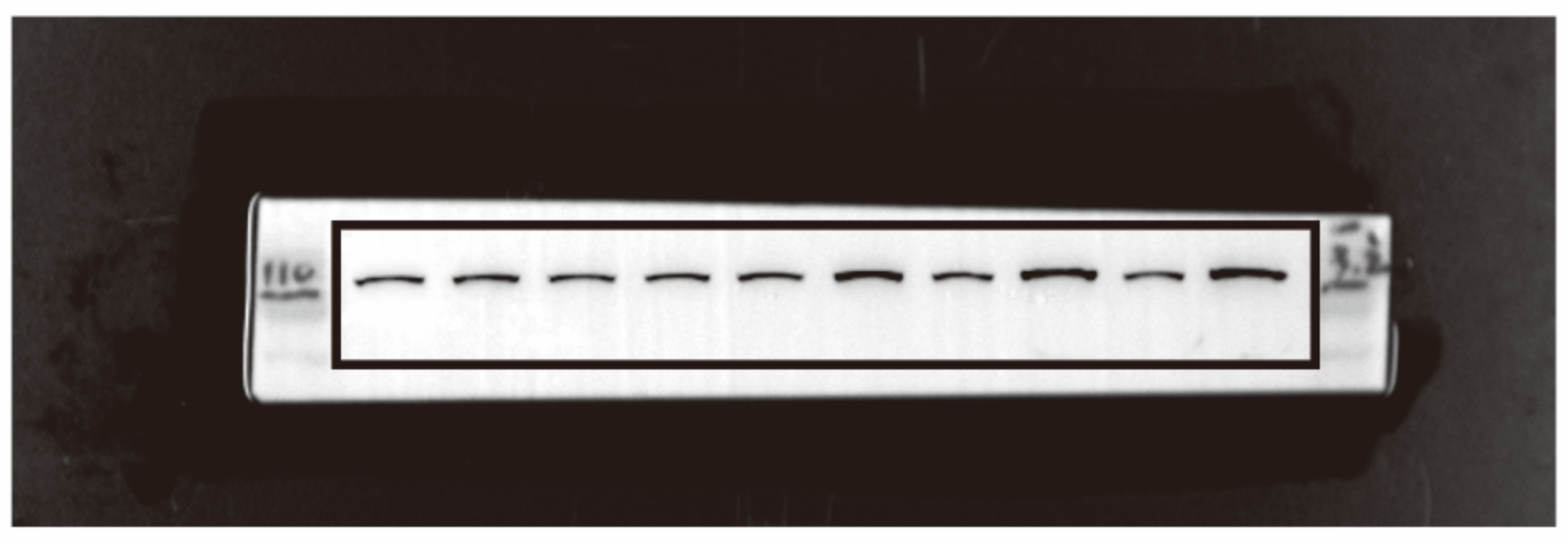

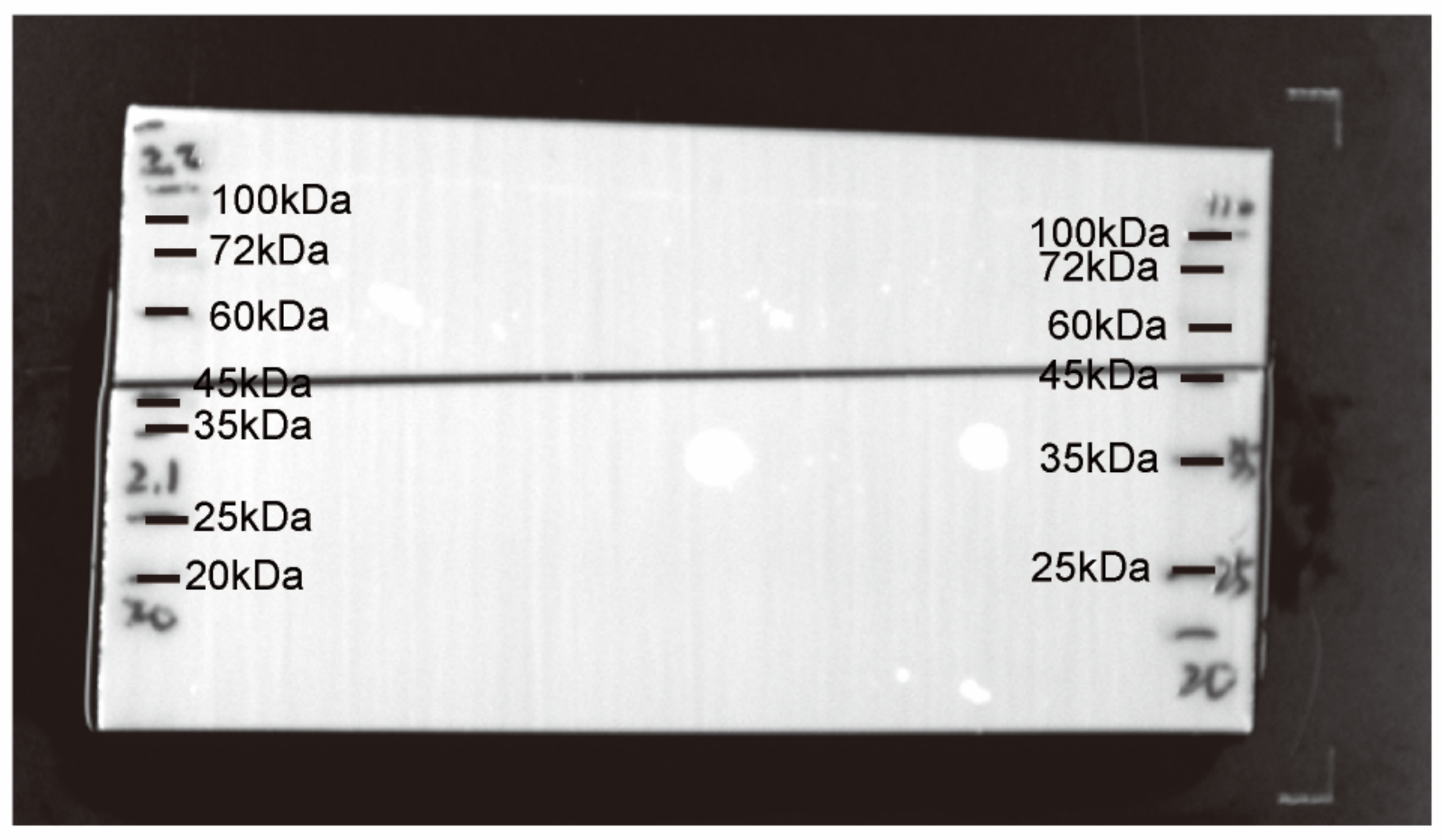

Fig.5l

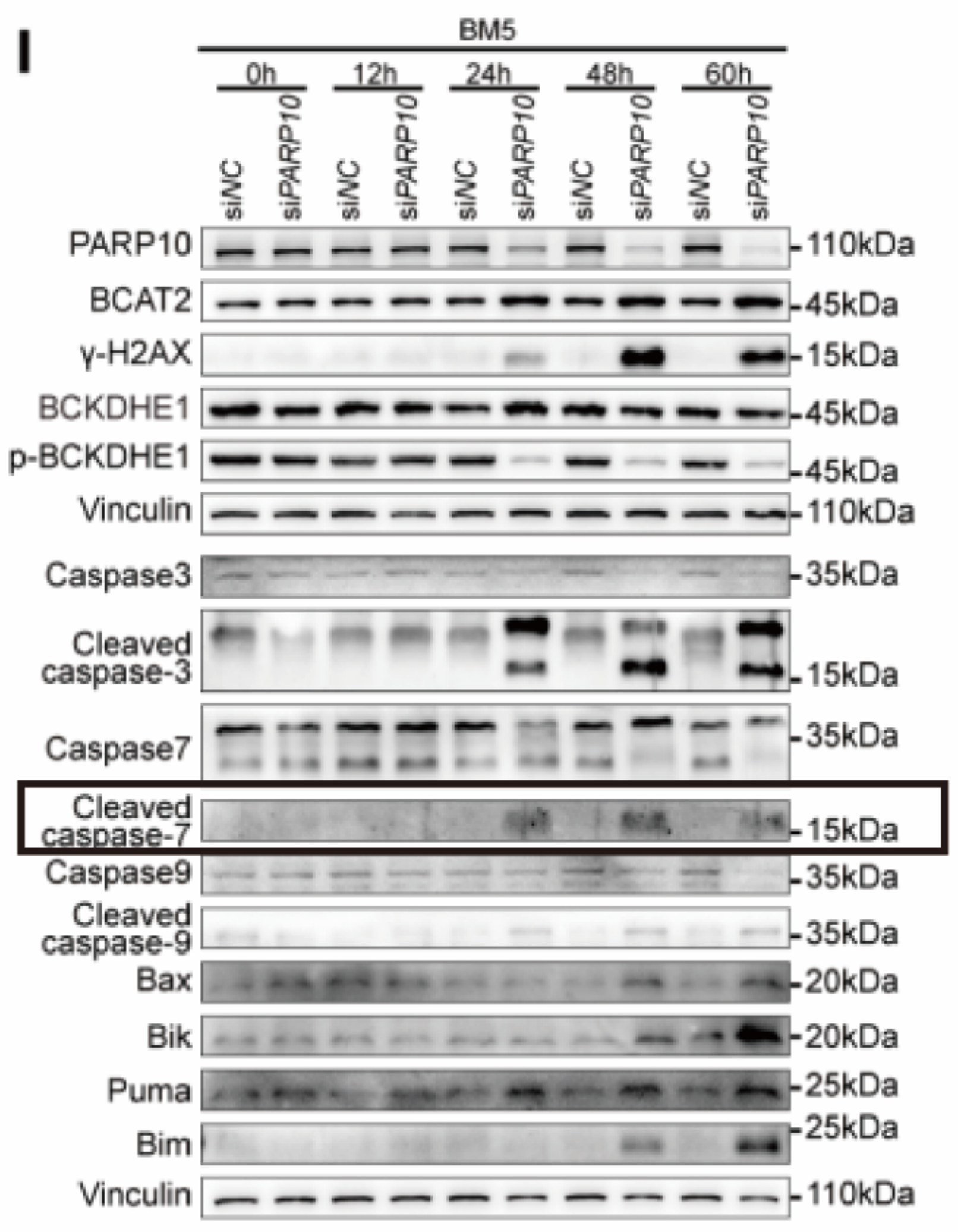

Cleaved caspase7

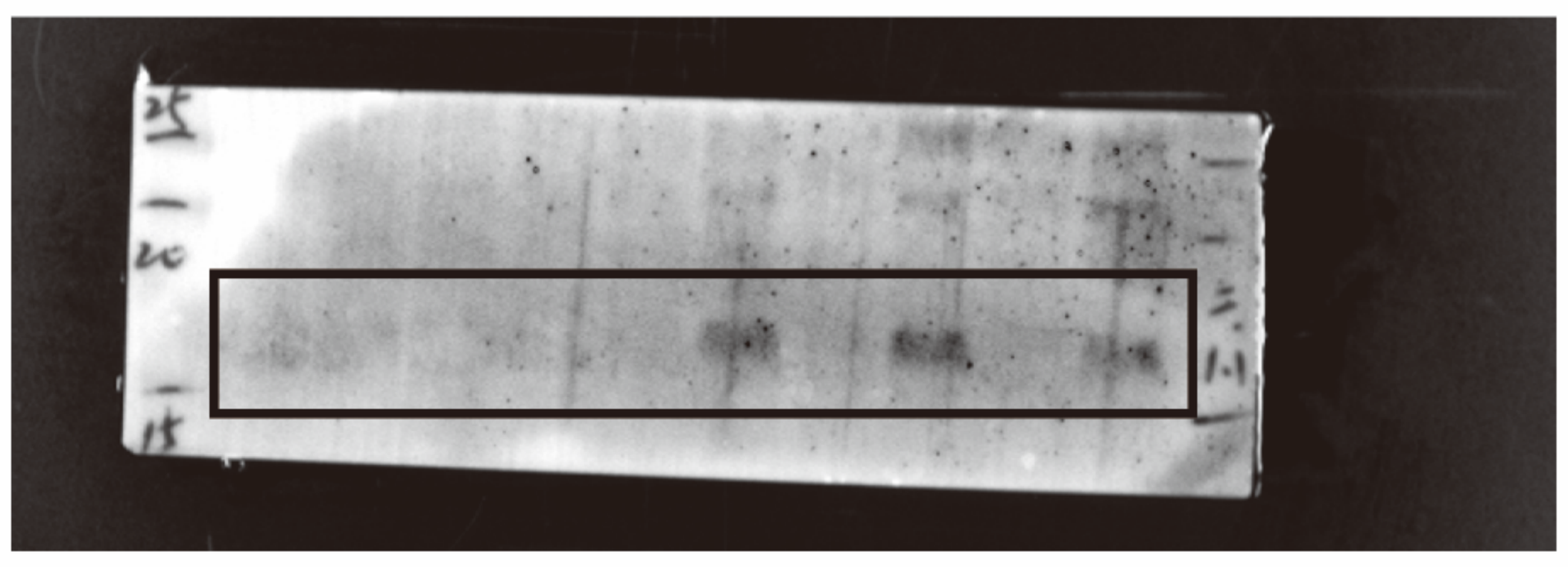

β-actin

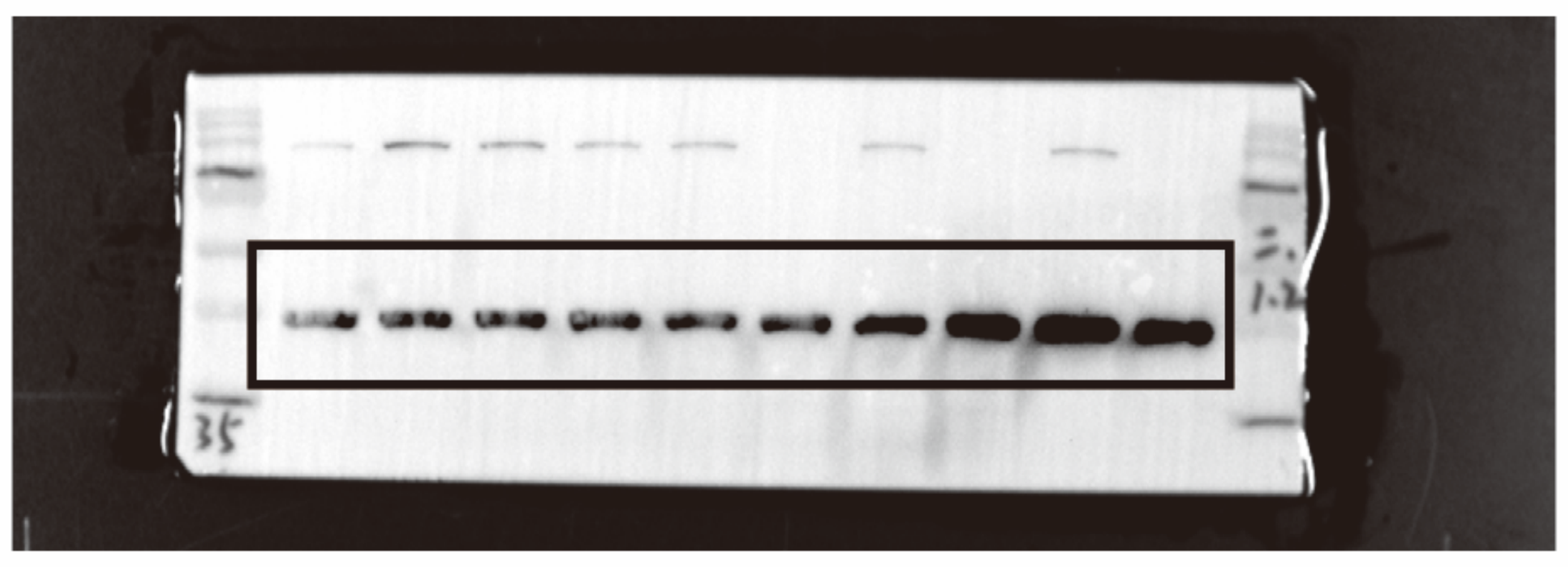

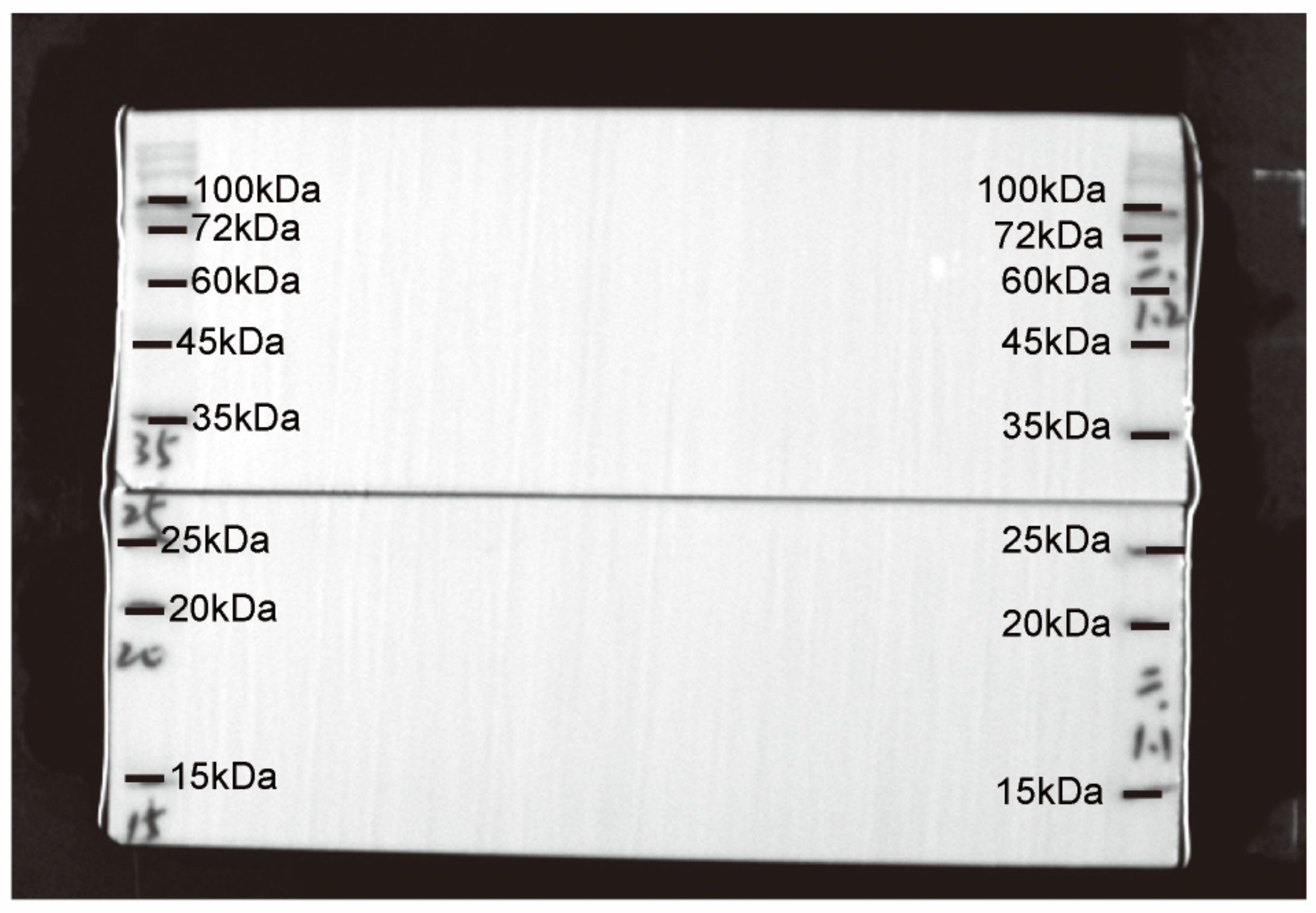

Fig.5l

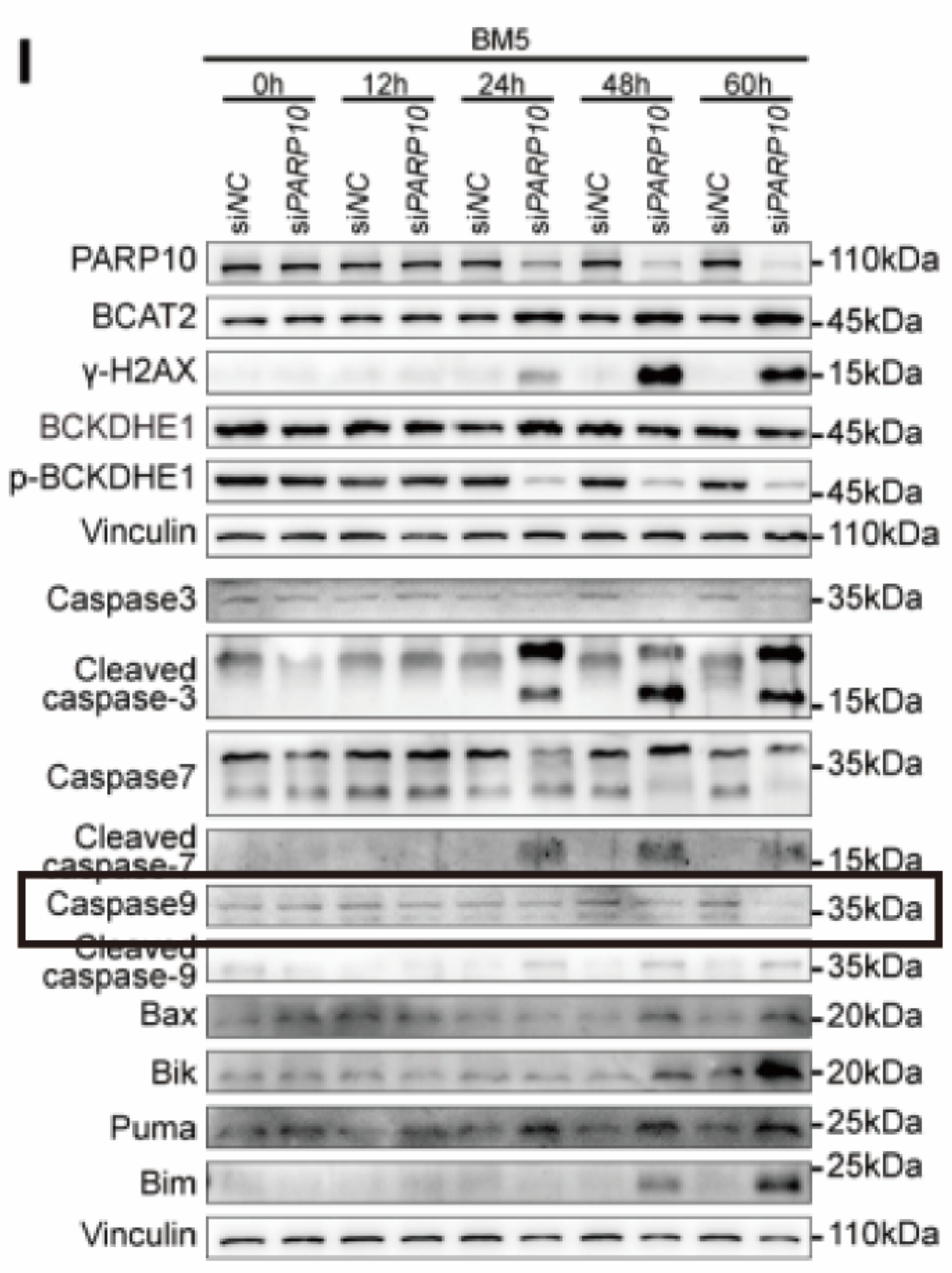

Caspase9

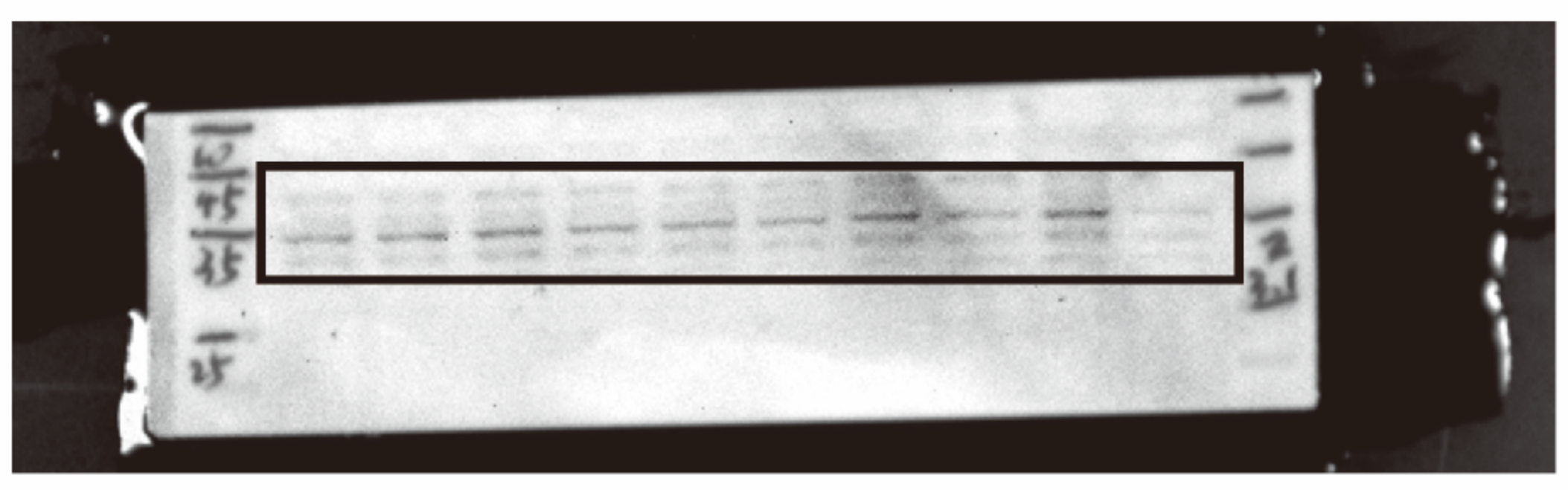

Vinculin

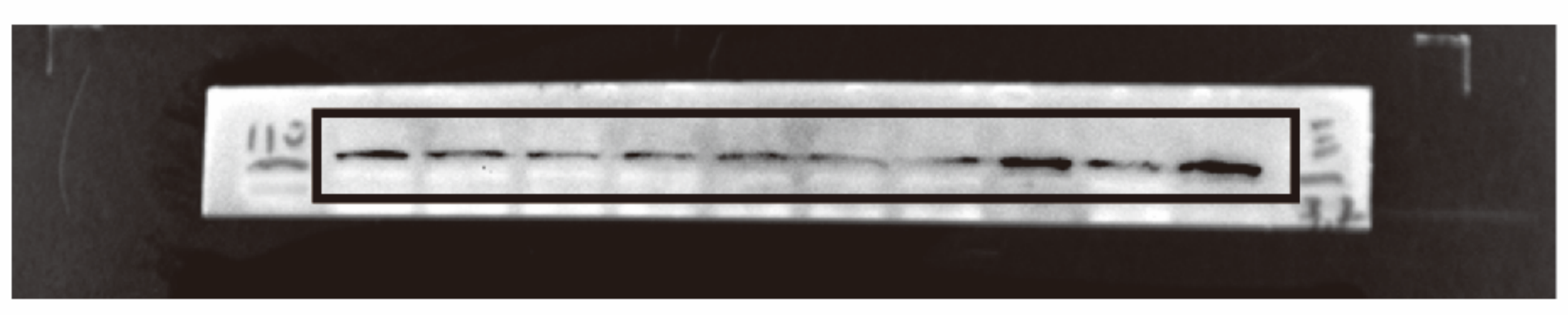

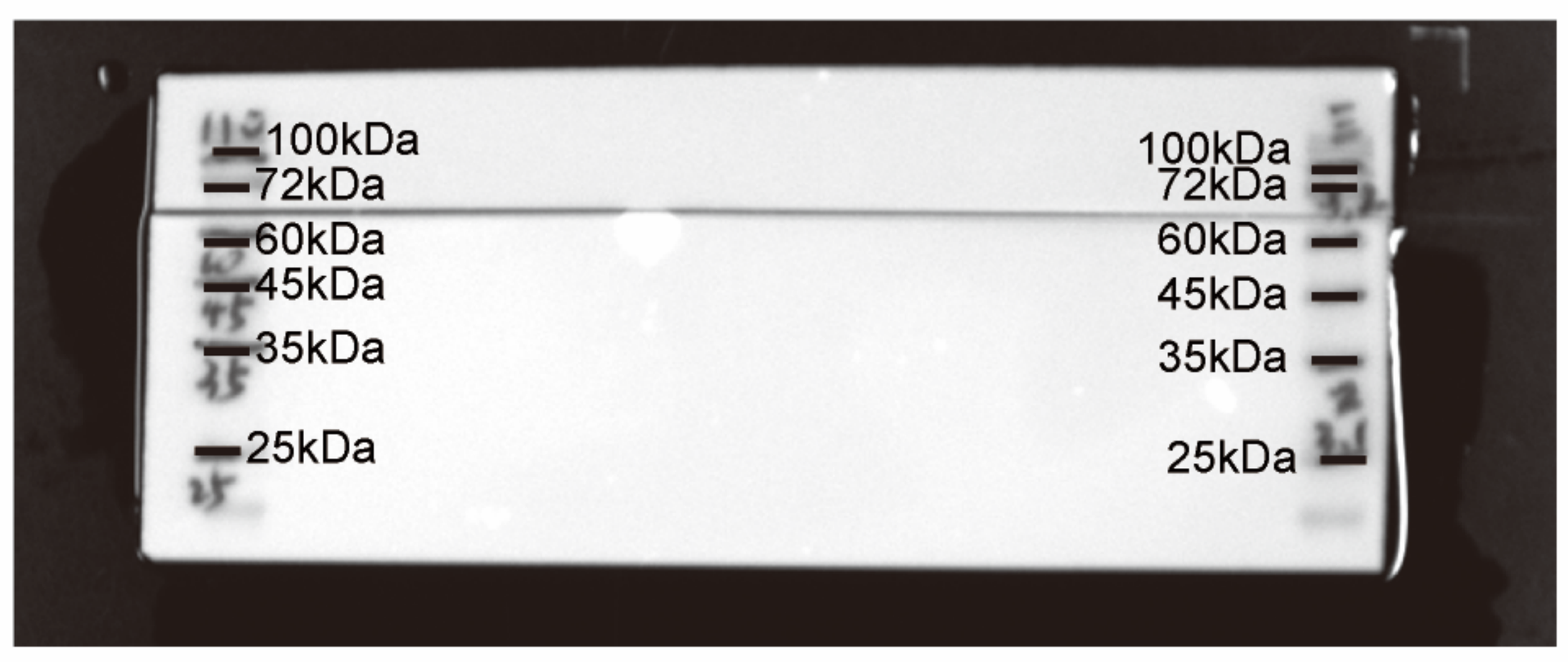

Figure	Merged	Stiched full image

Fig.5I

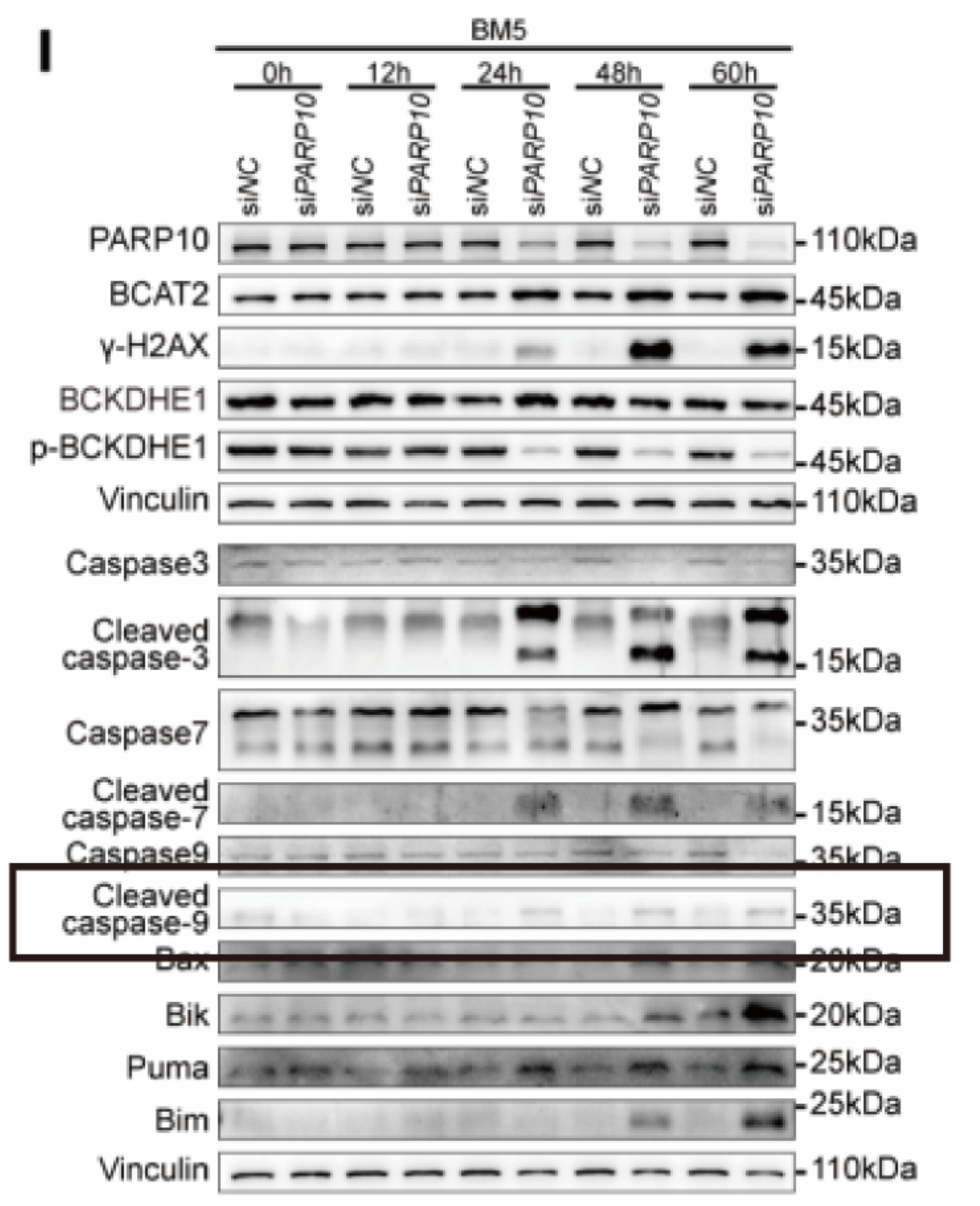

Cleaved caspase9

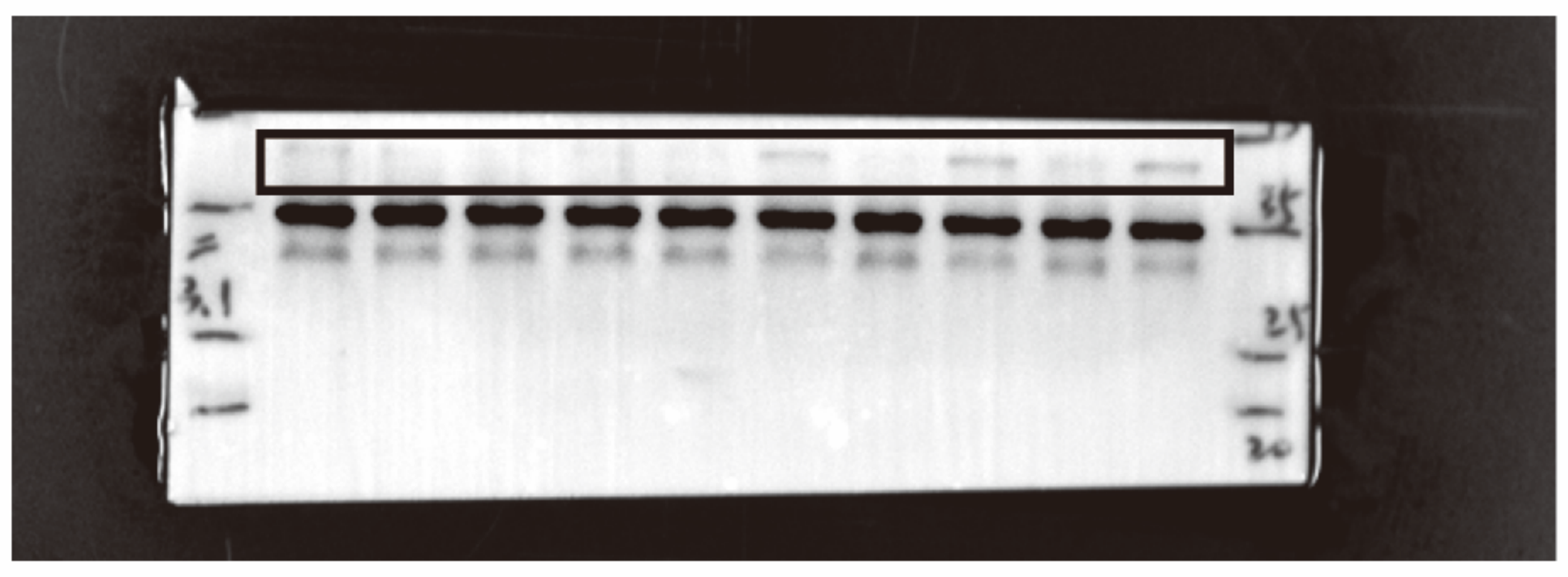

Vinculin

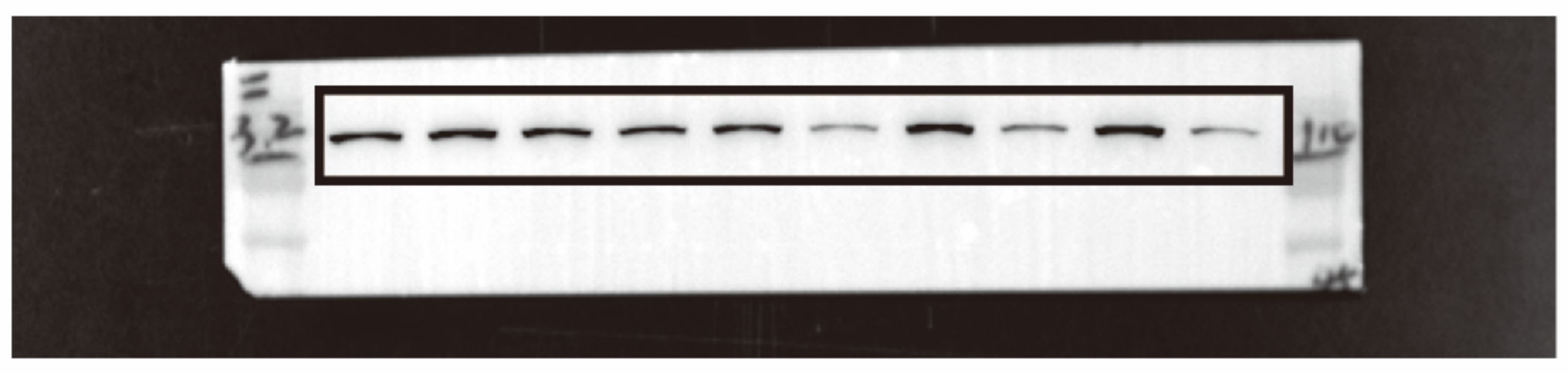

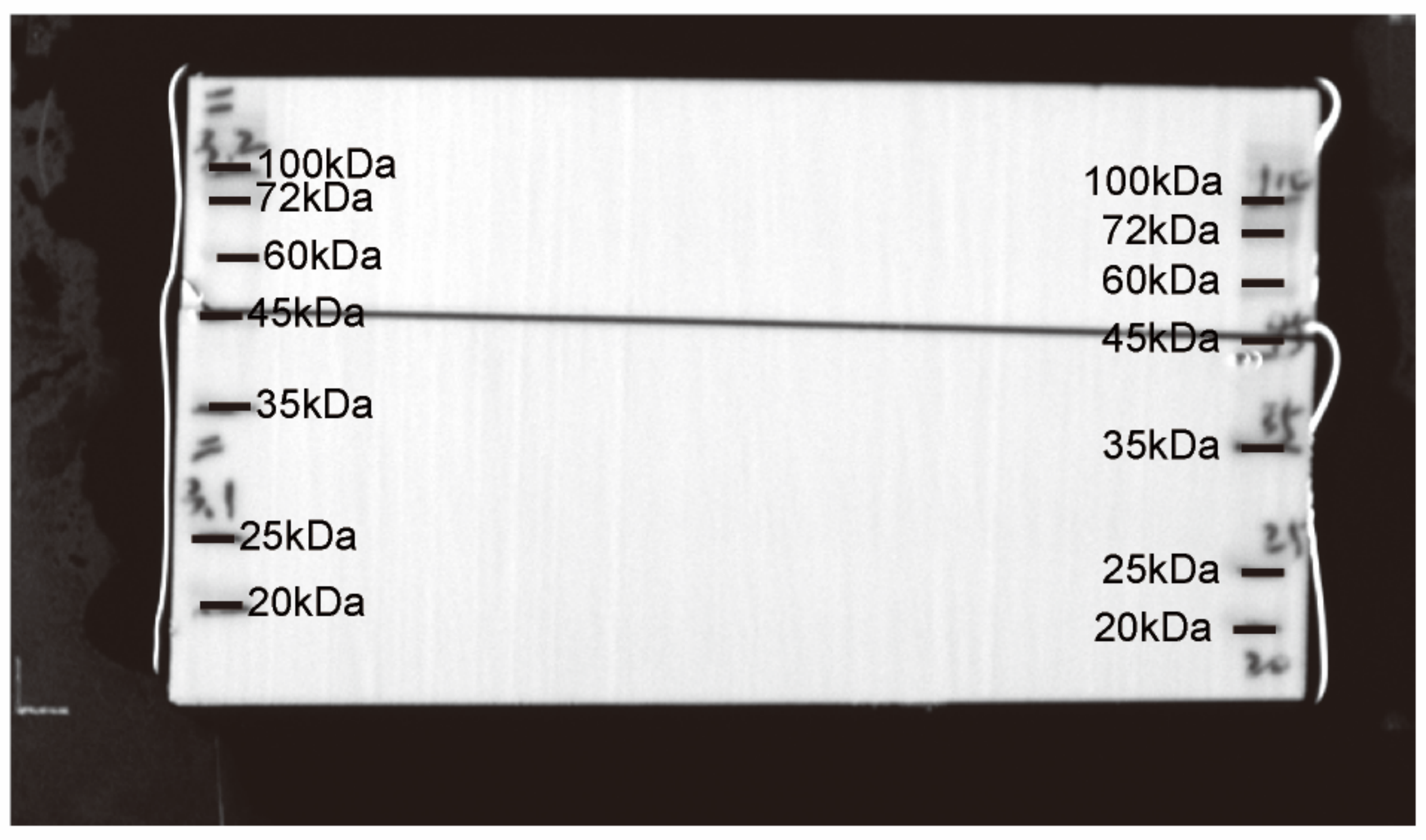

Fig.5I

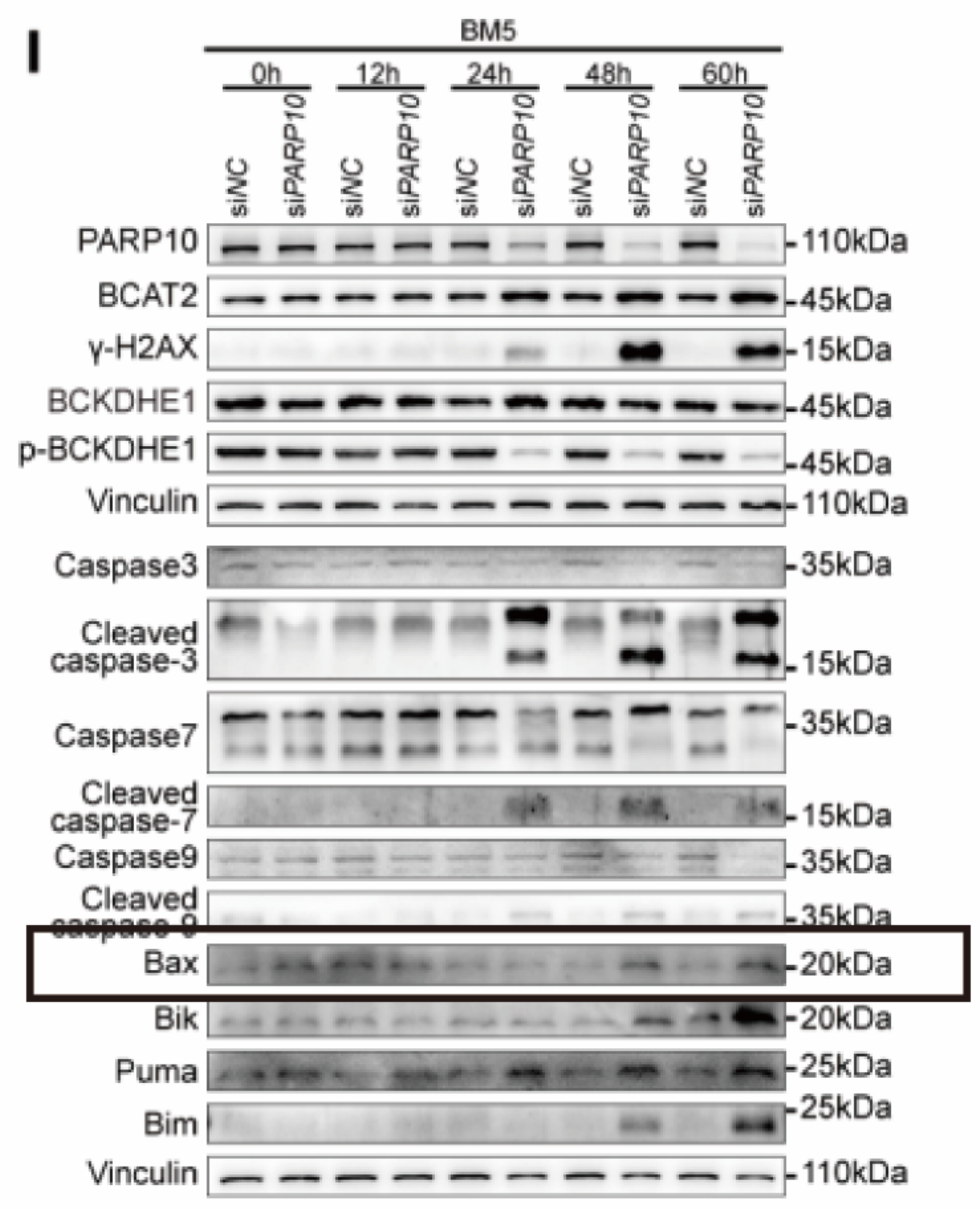

Bax

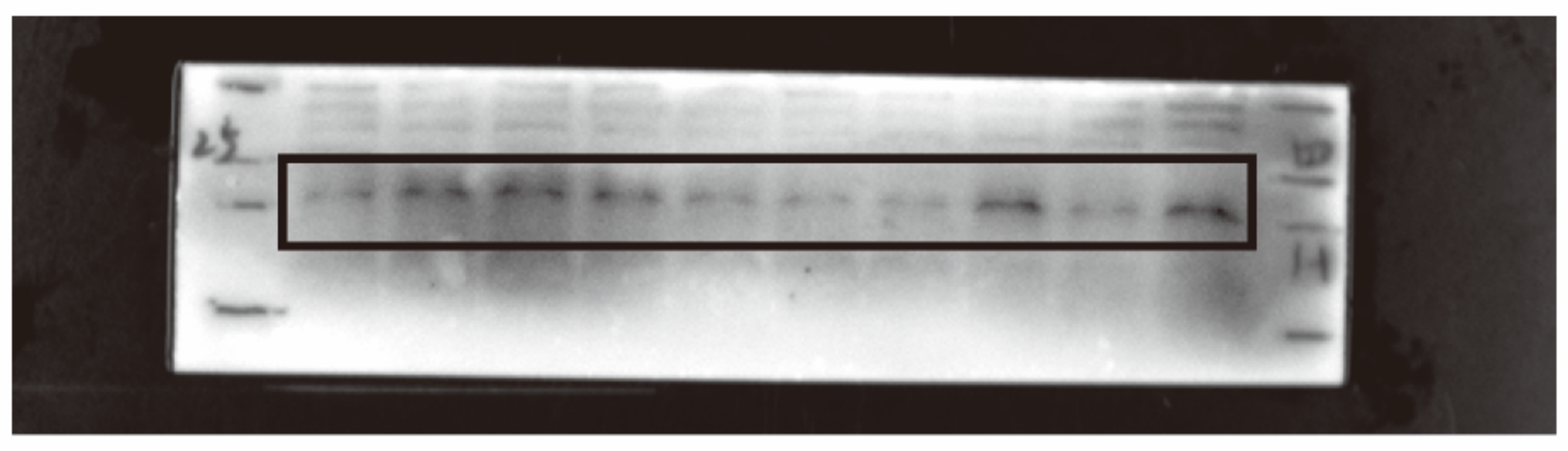

Vinculin

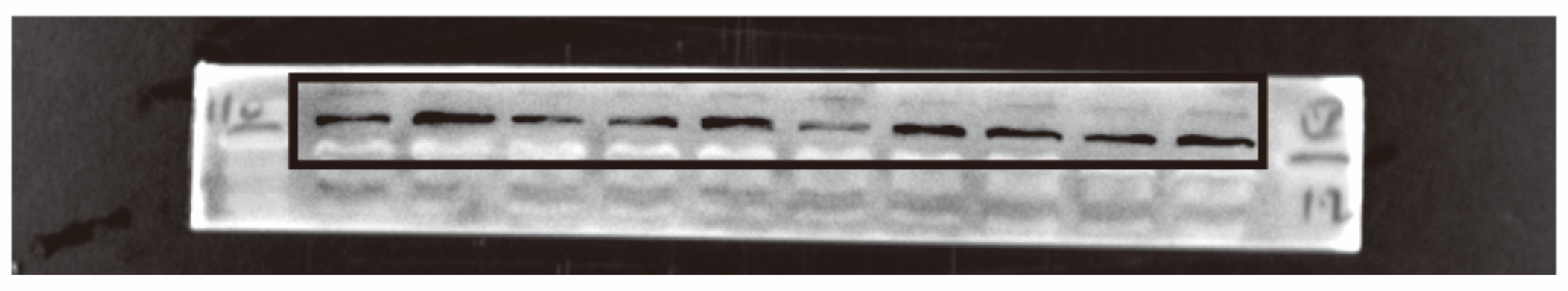

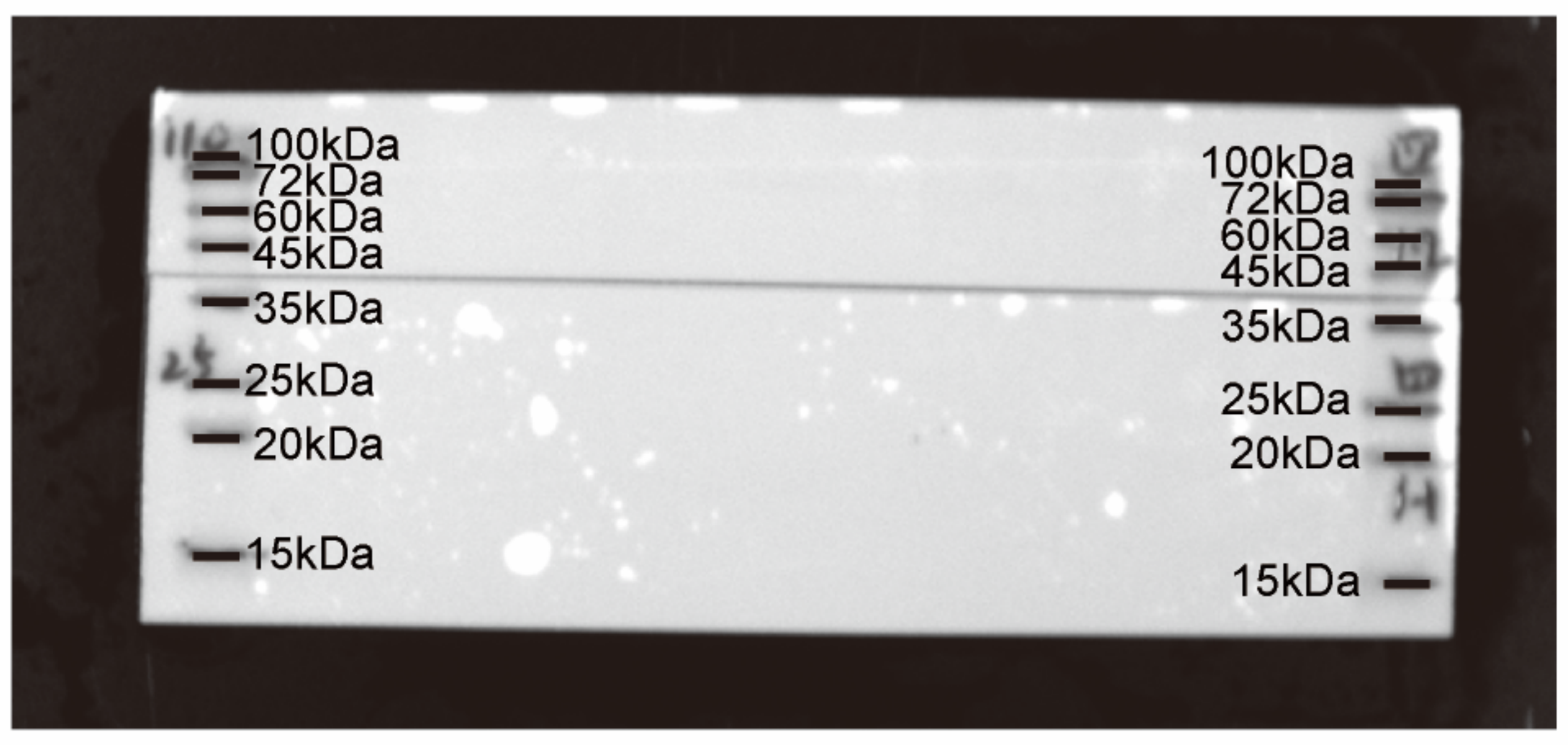

Fig.5I

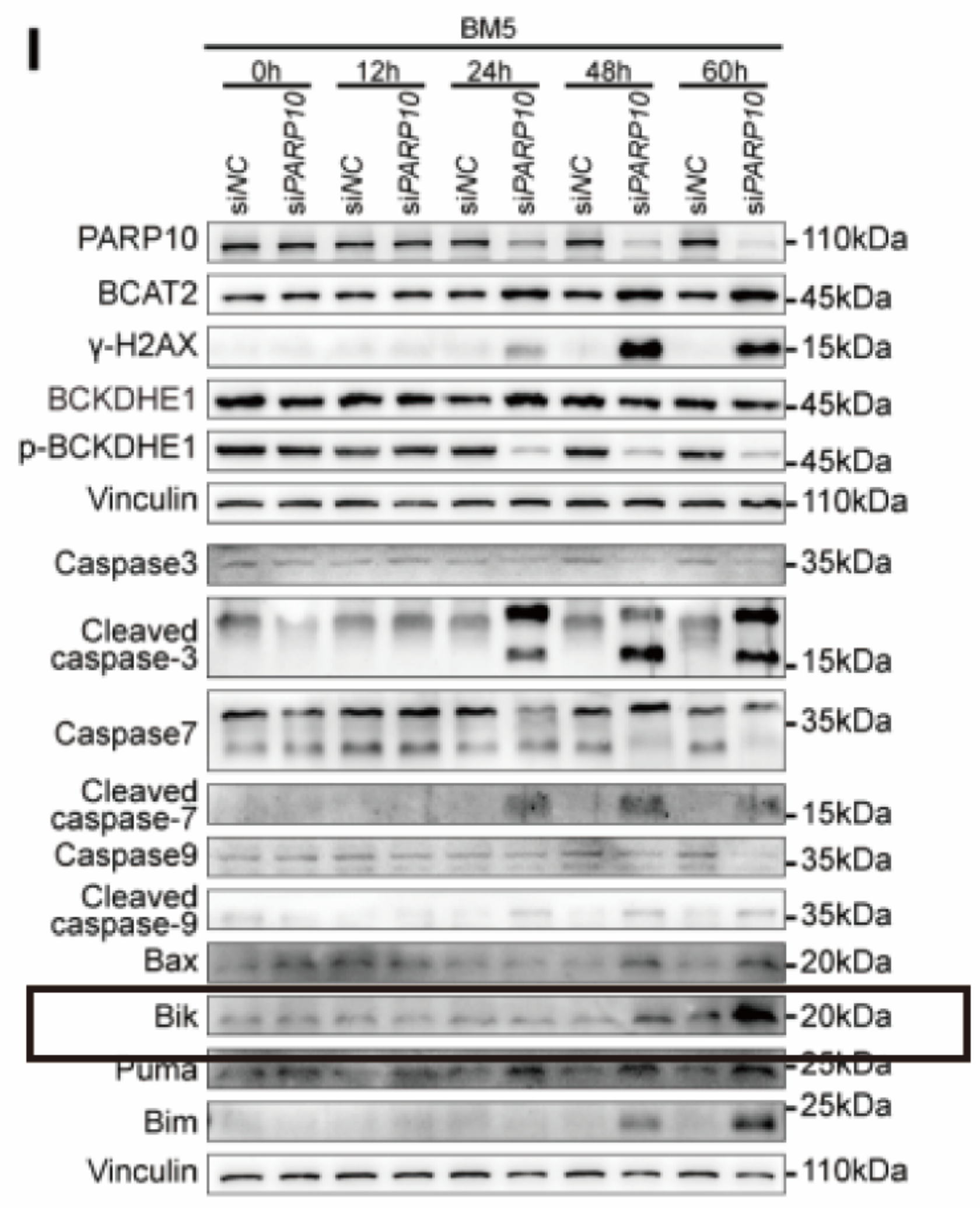

Bik

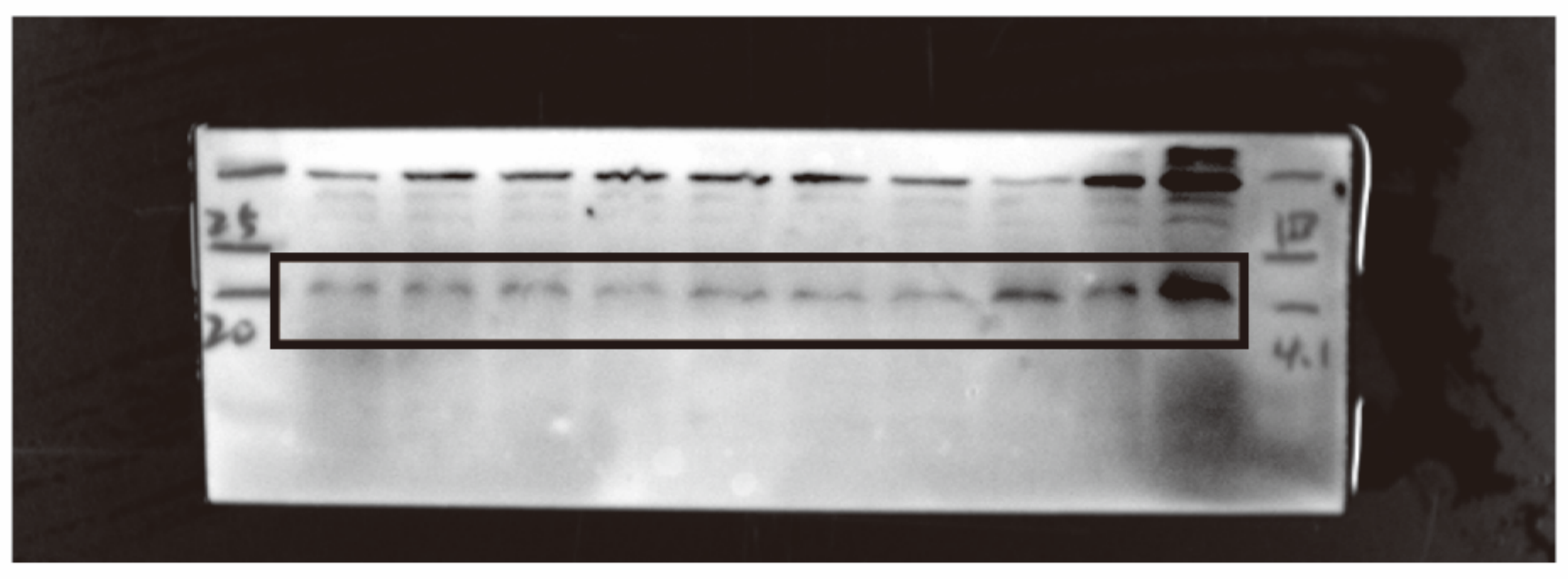

Vinculin

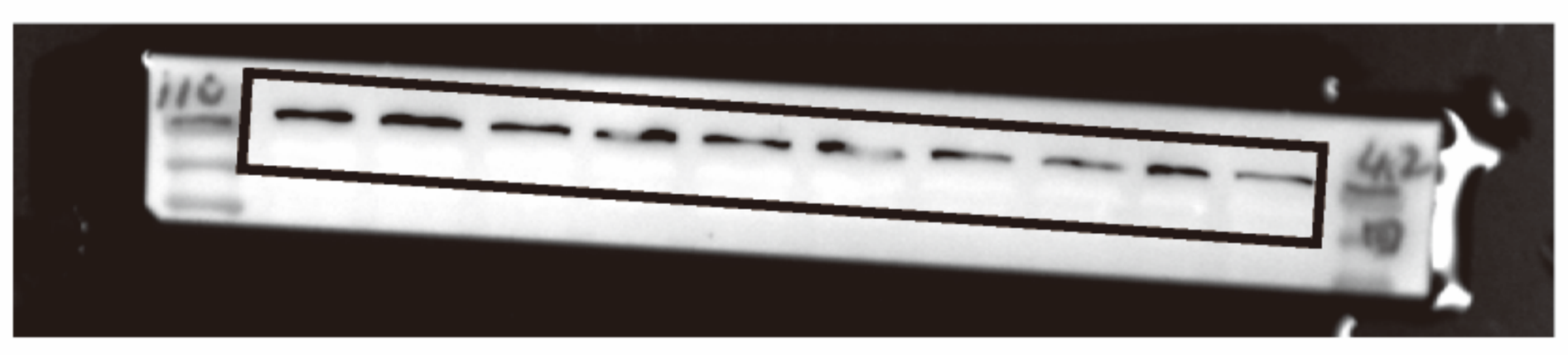

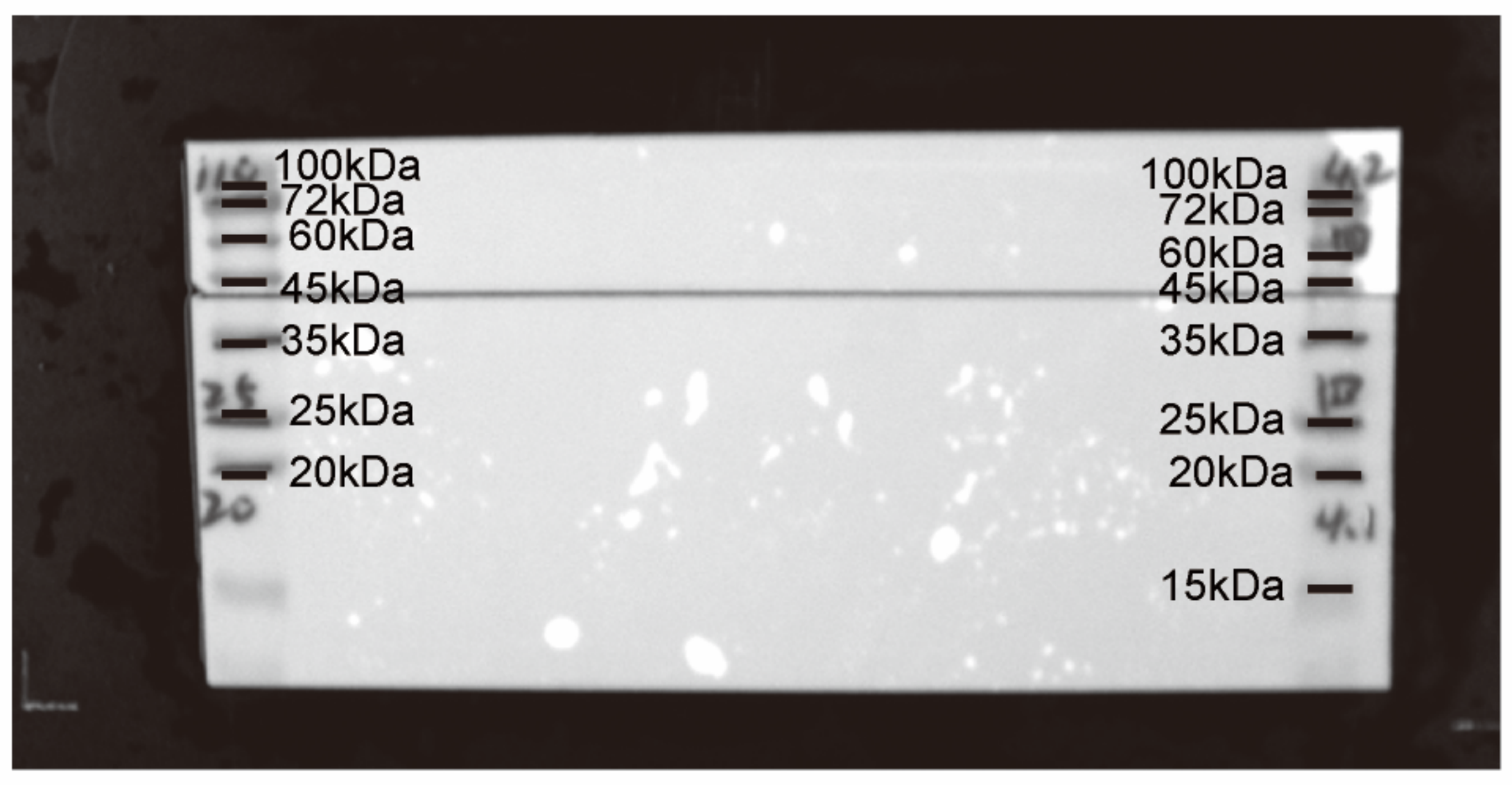

Fig.5I

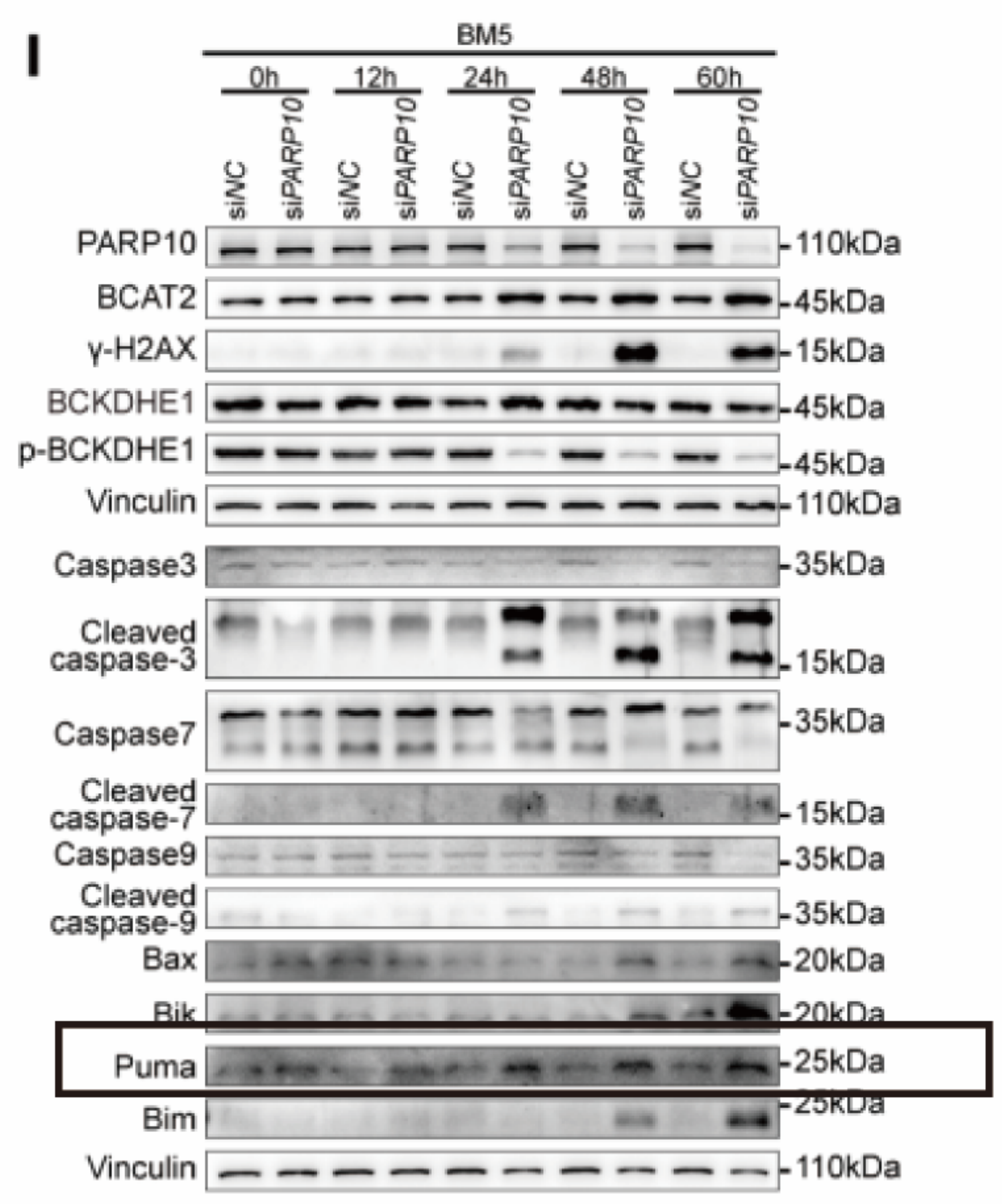

Puma

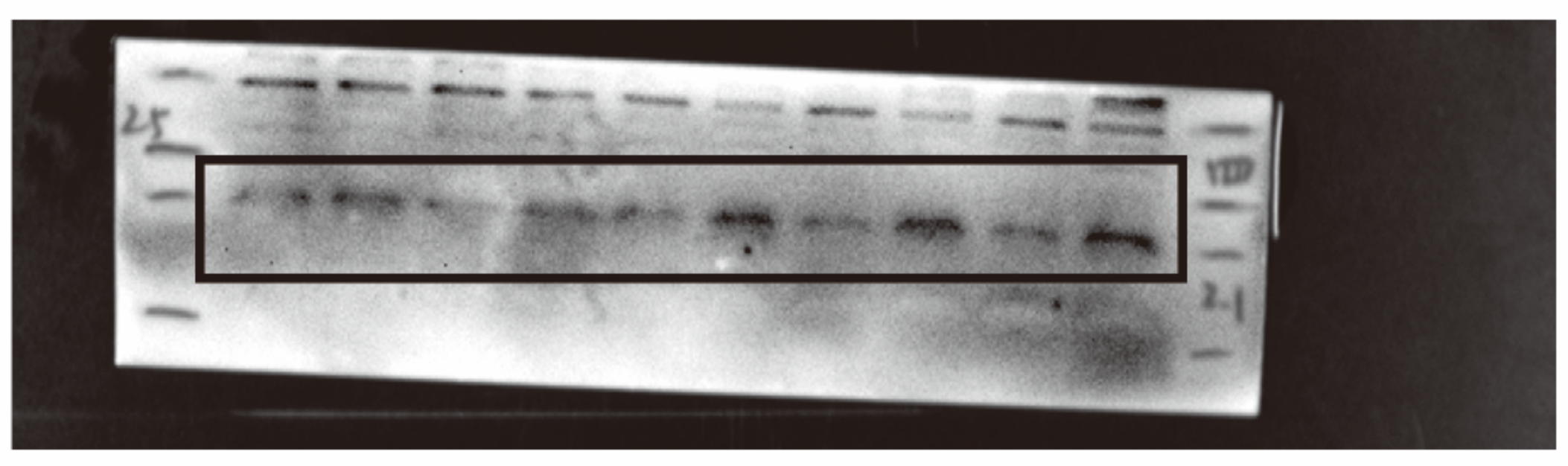

Vinculin

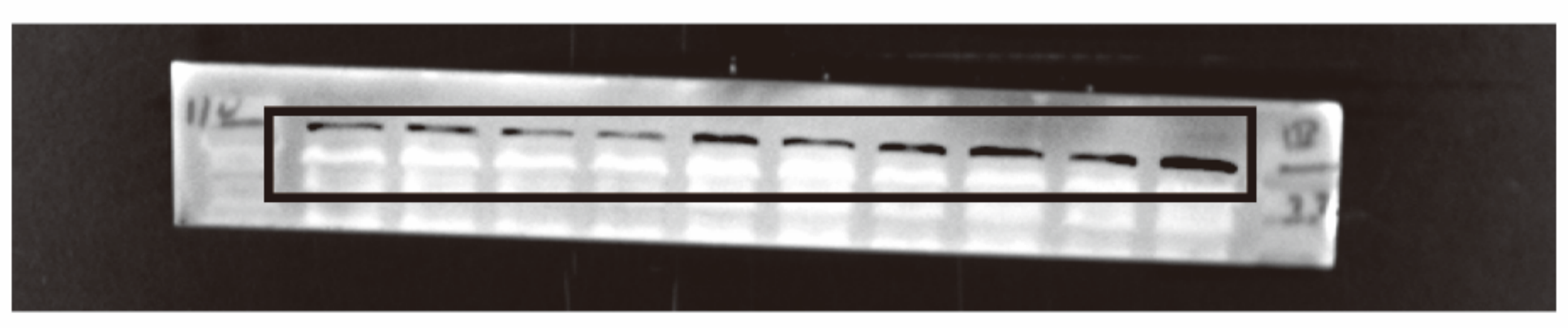

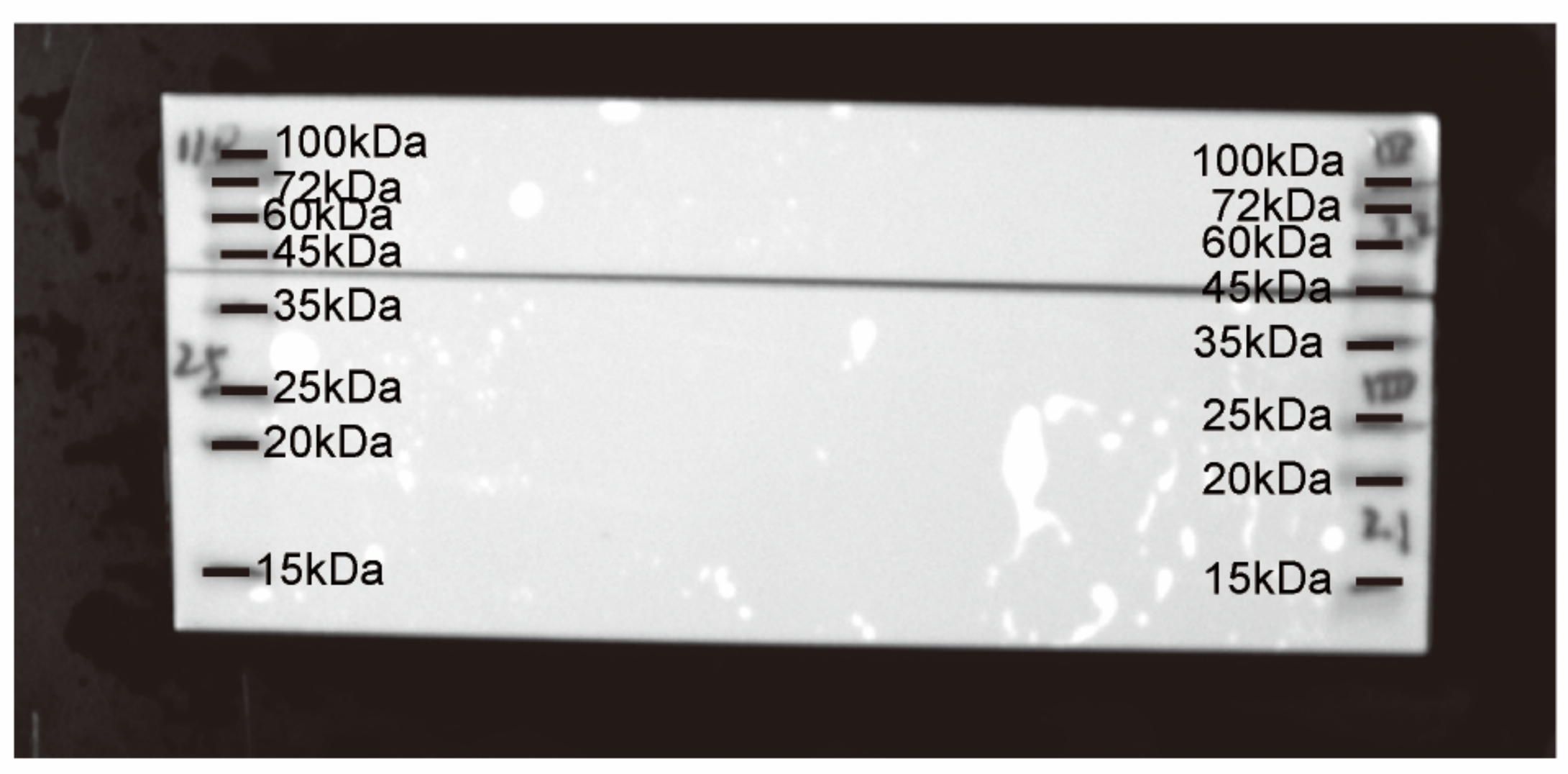

Figure	Merged	Stiched full image

Fig.5I

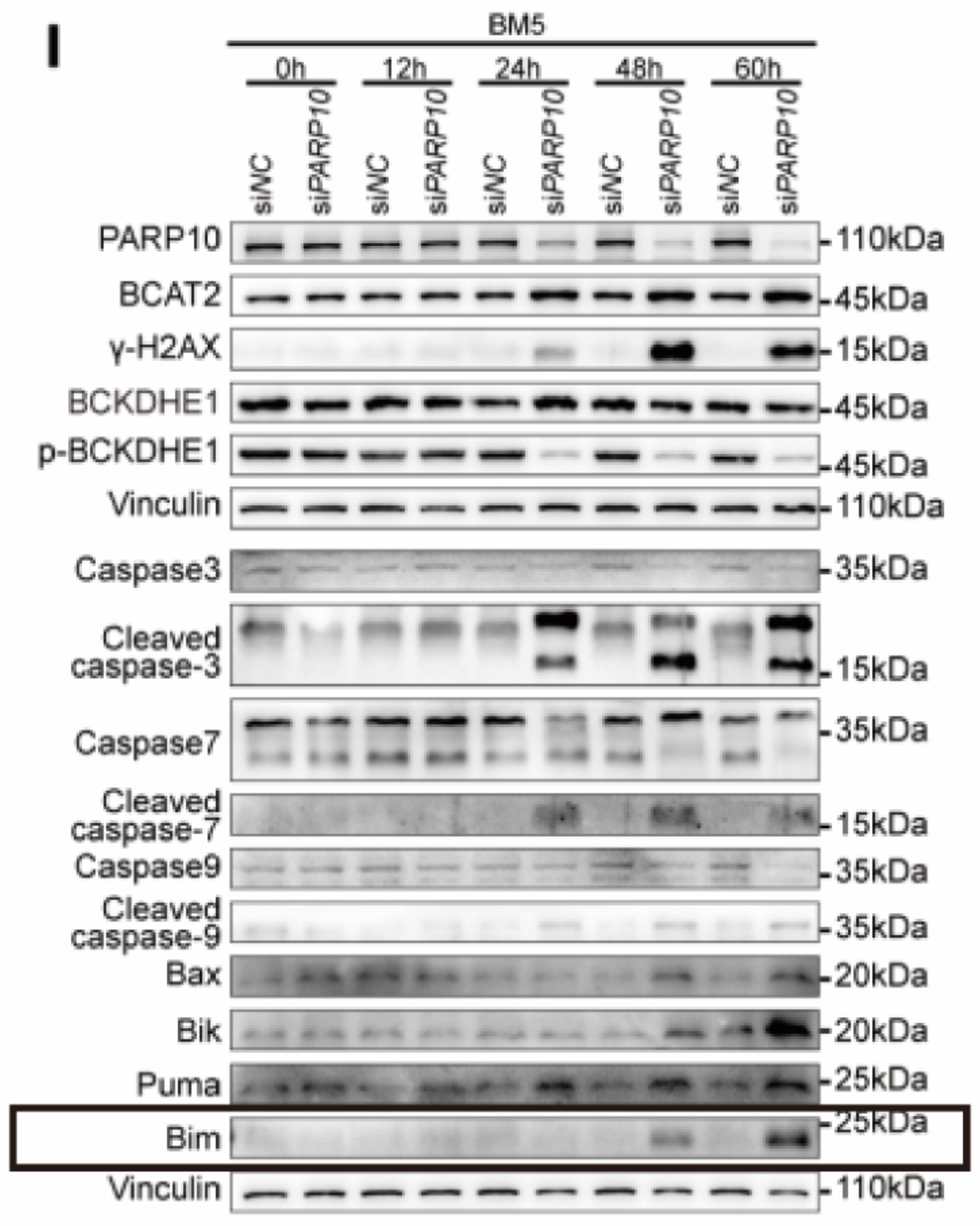

Bim

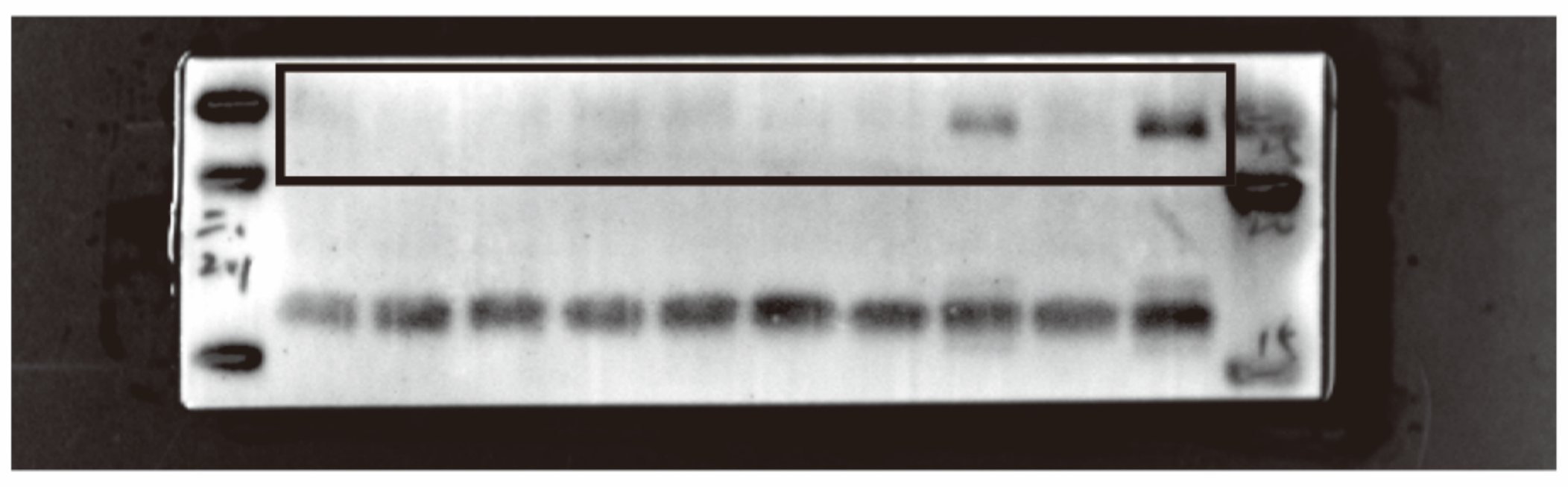

β-actin

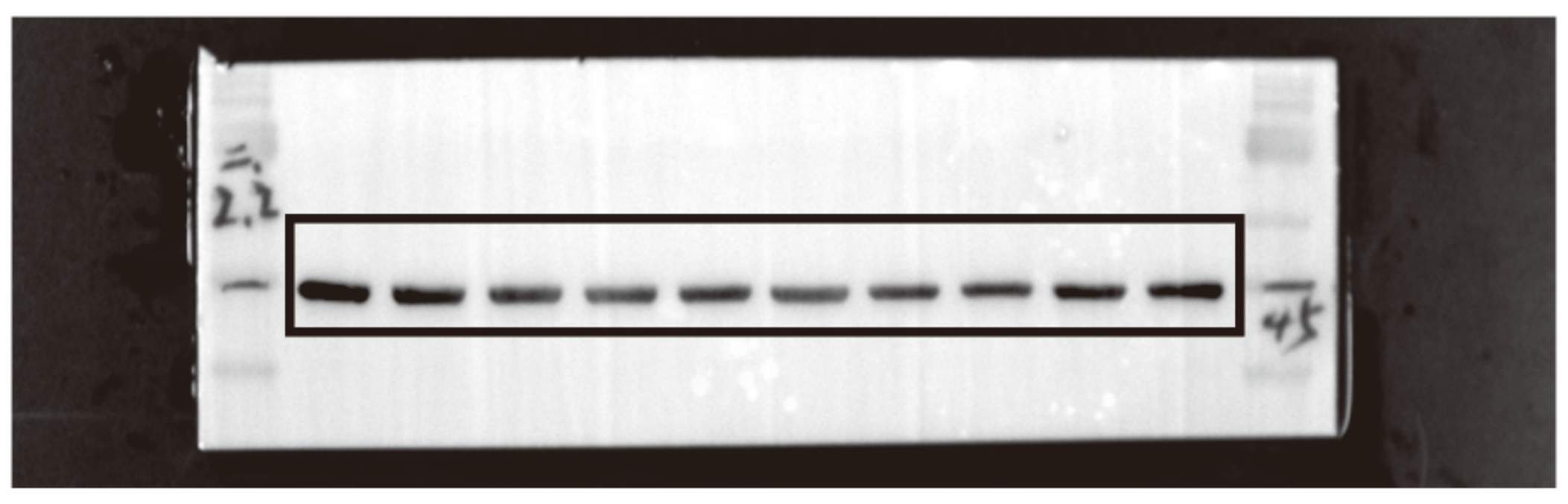

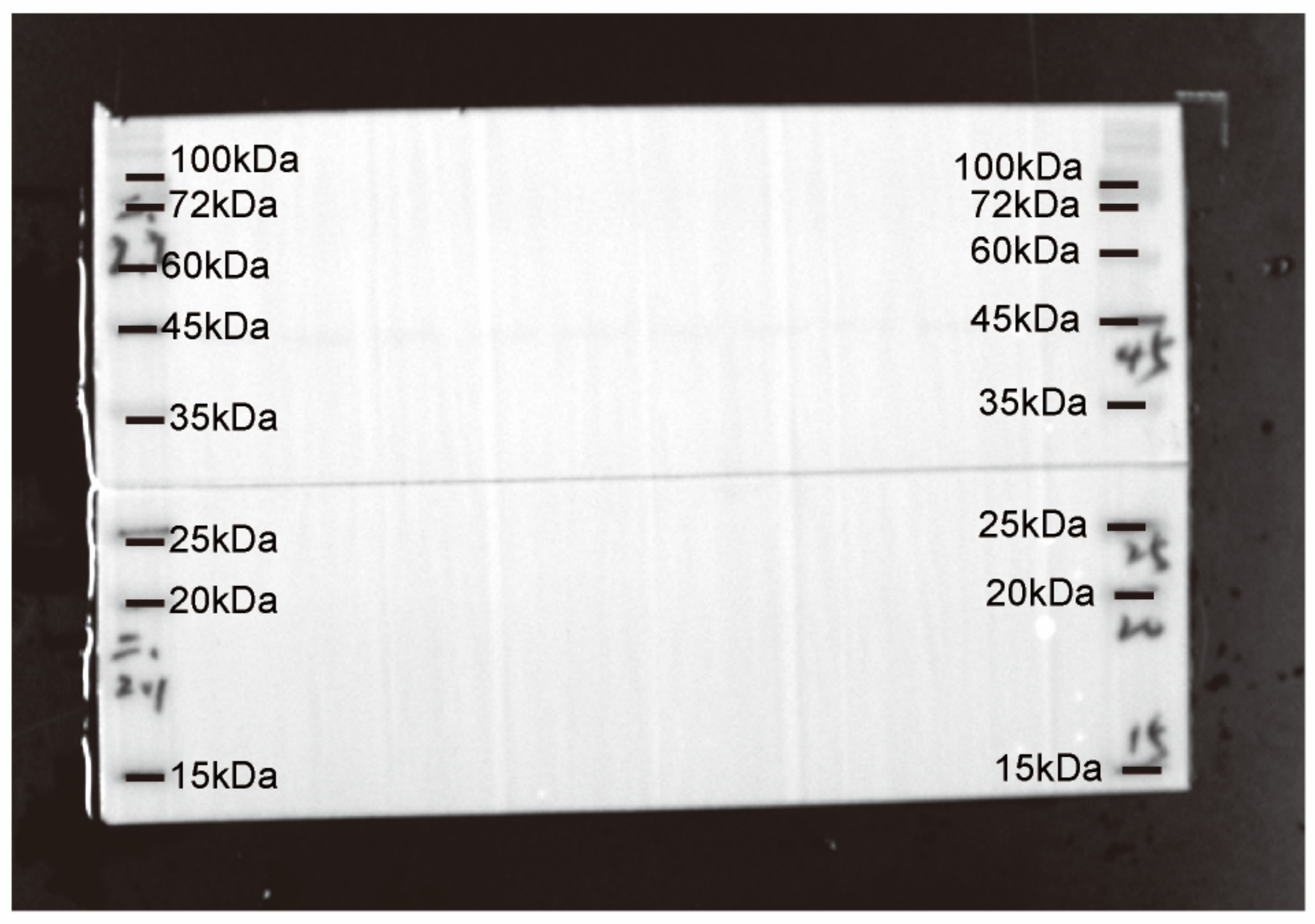

Fig.5J

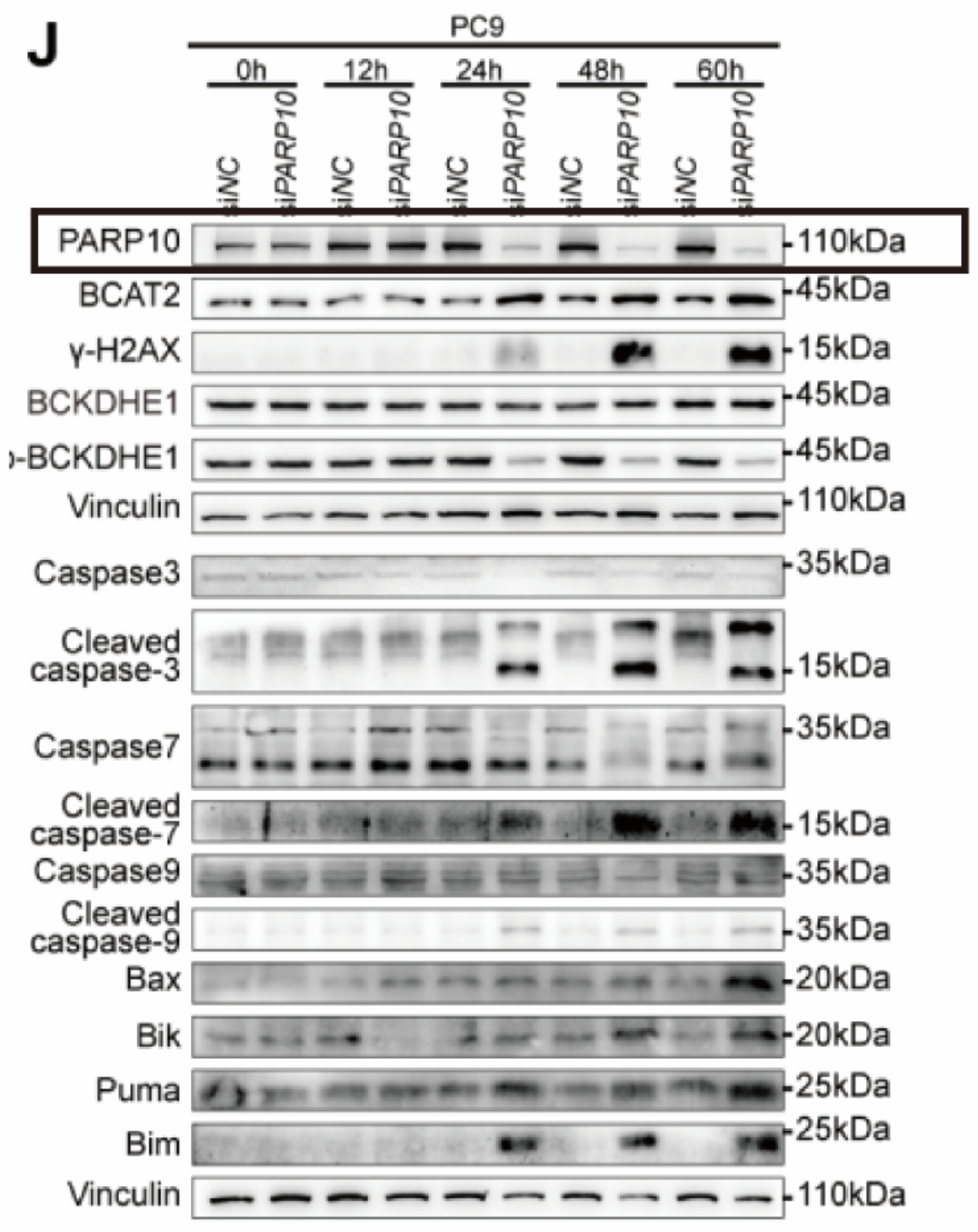

PARP10

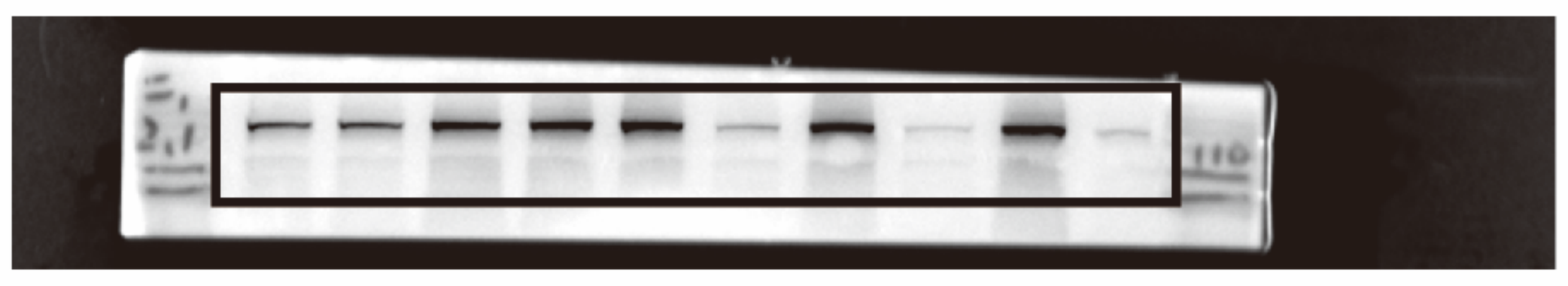

GAPDH

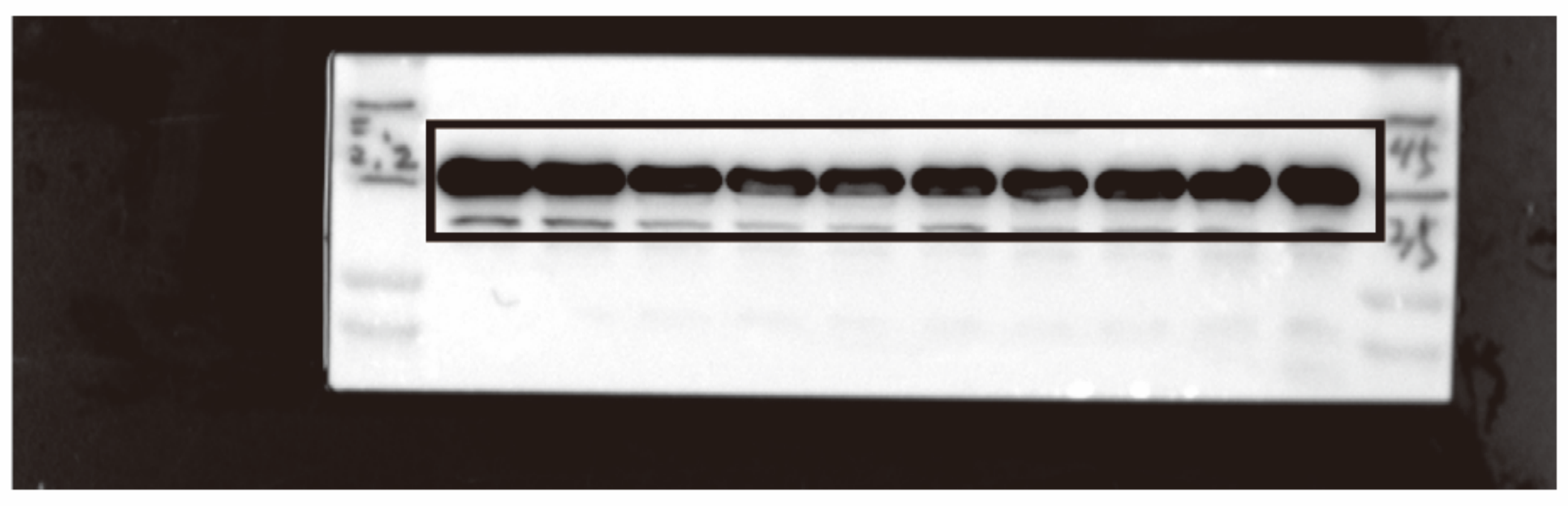

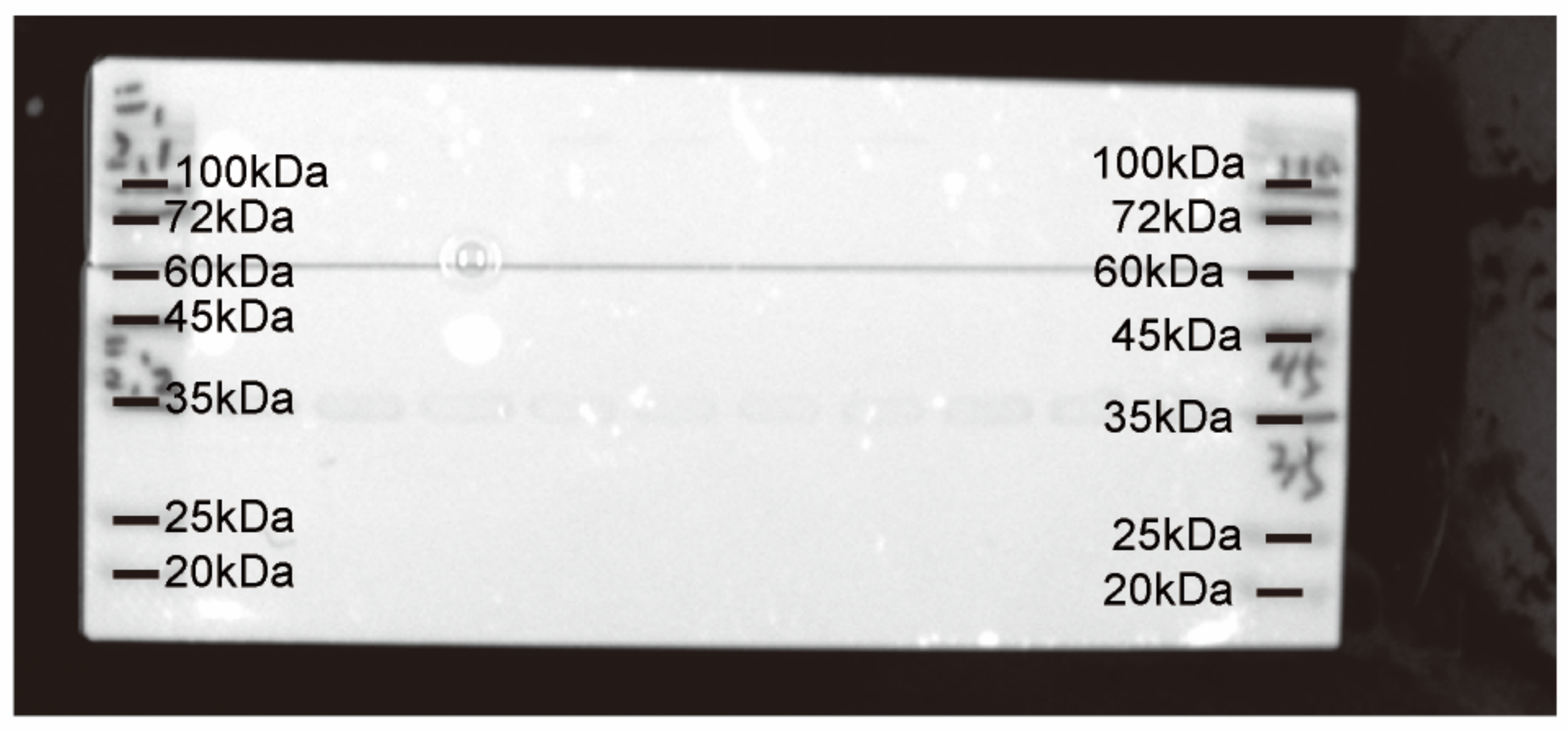

Fig.5J

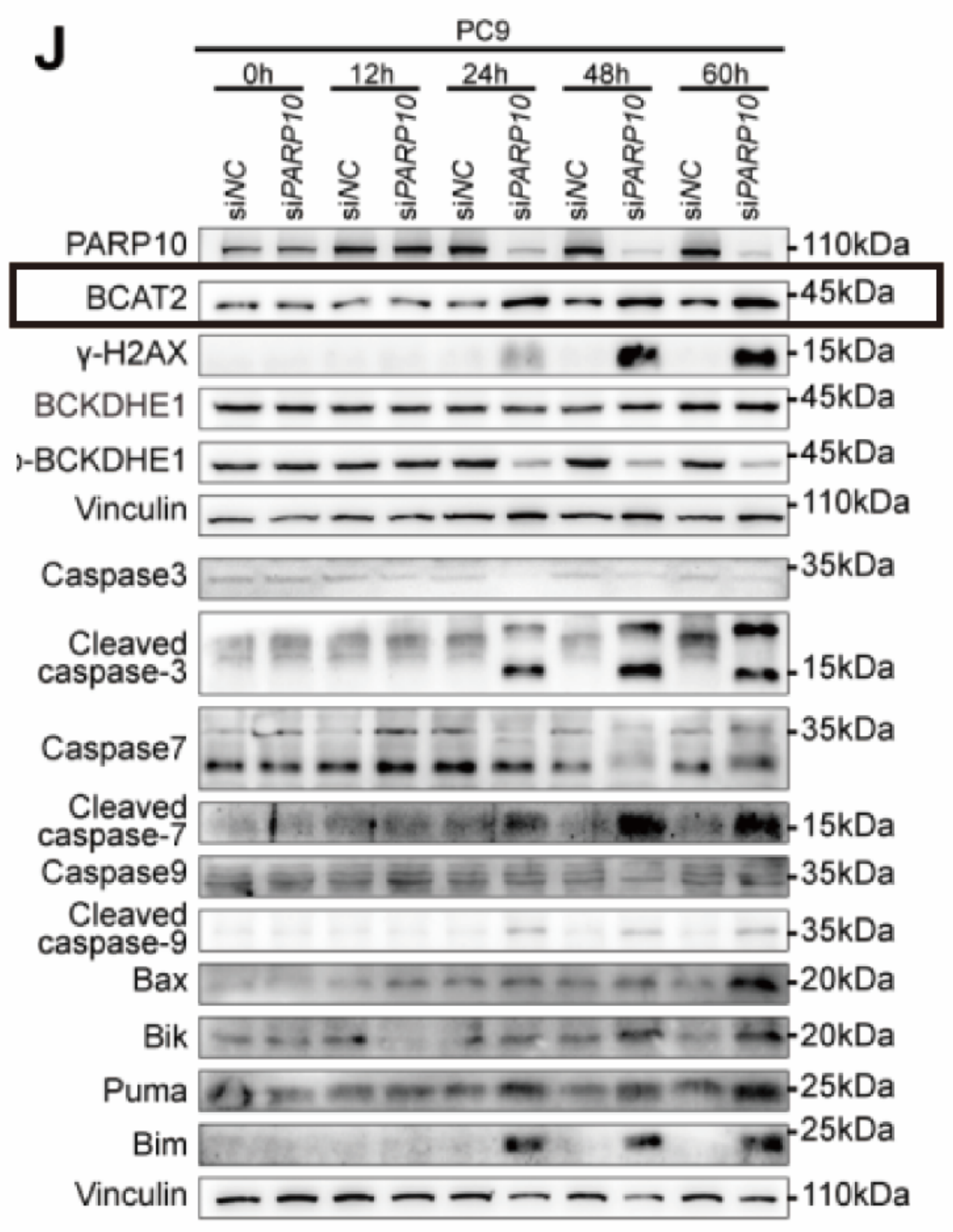

BCAT2

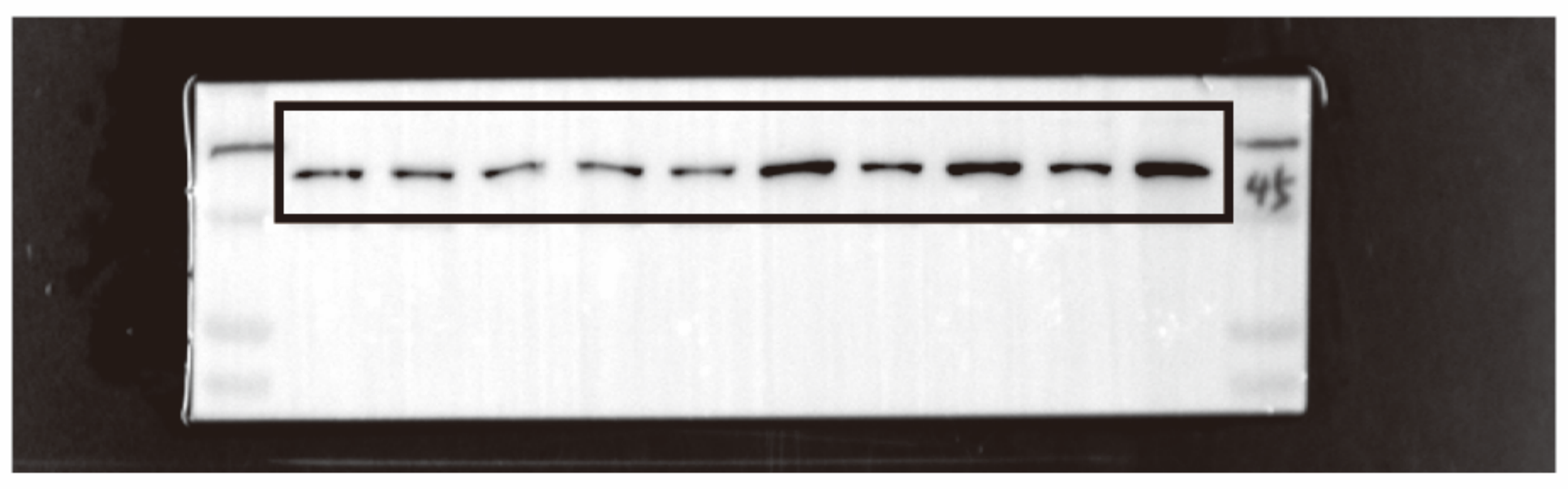

Vinculin

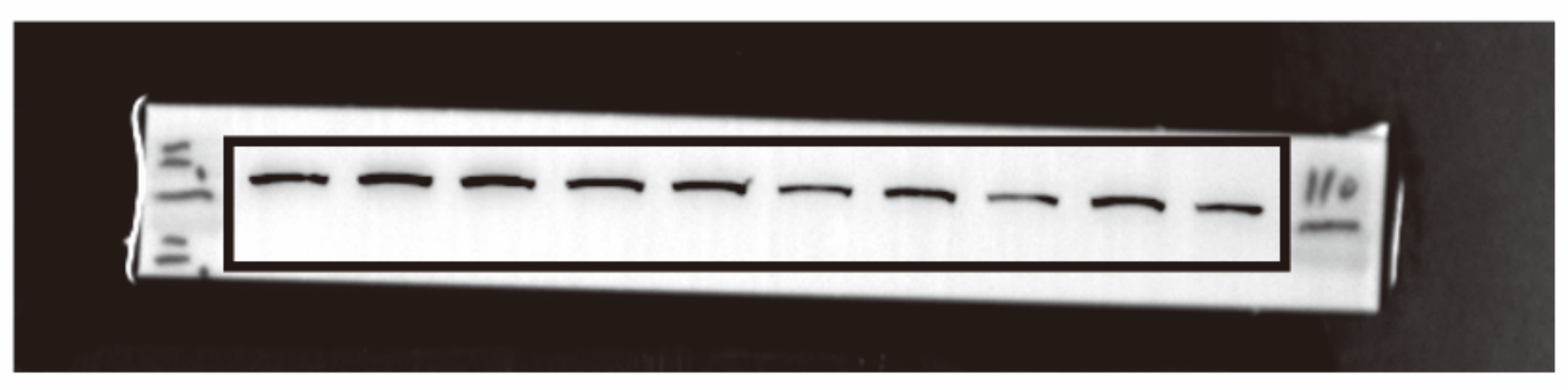

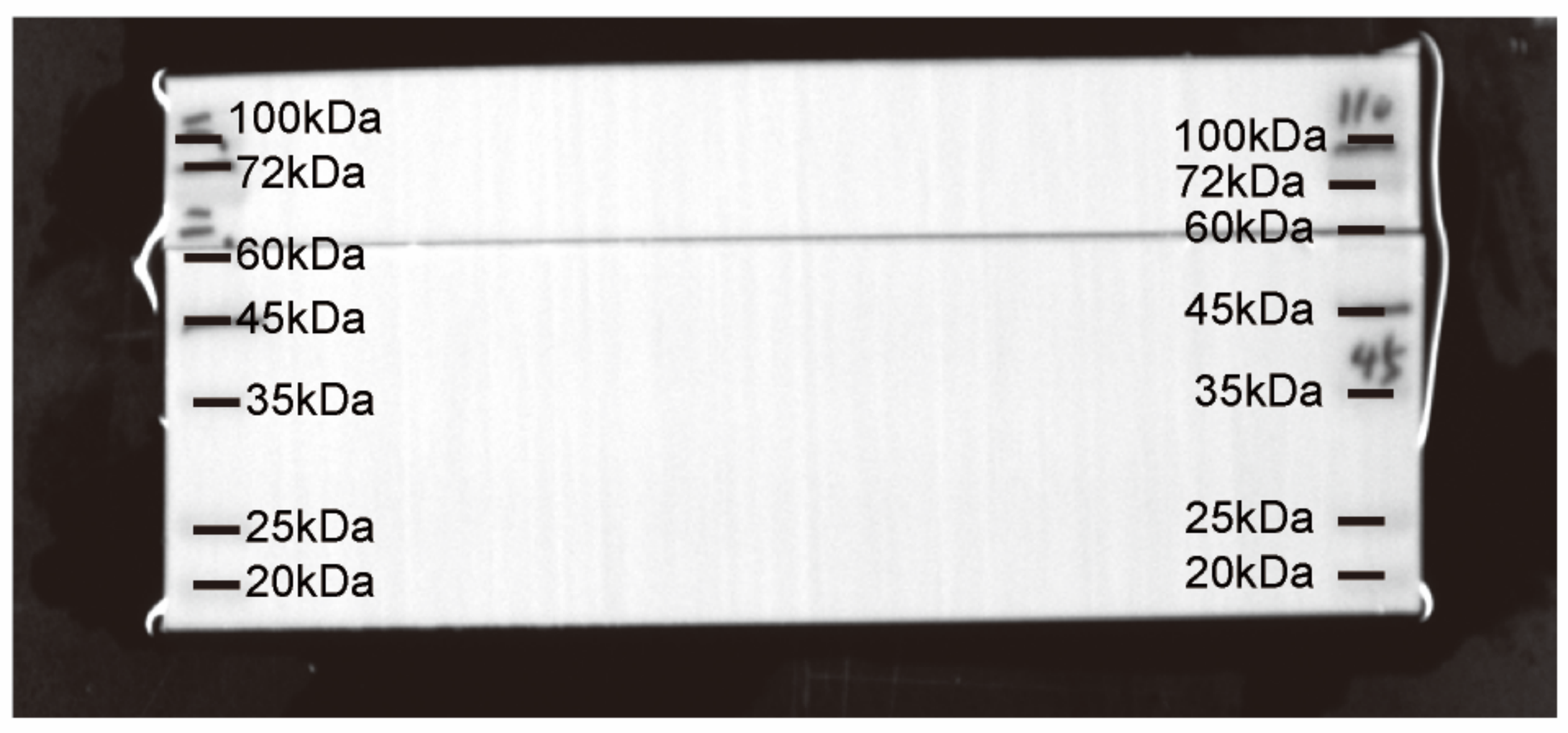

Fig.5J

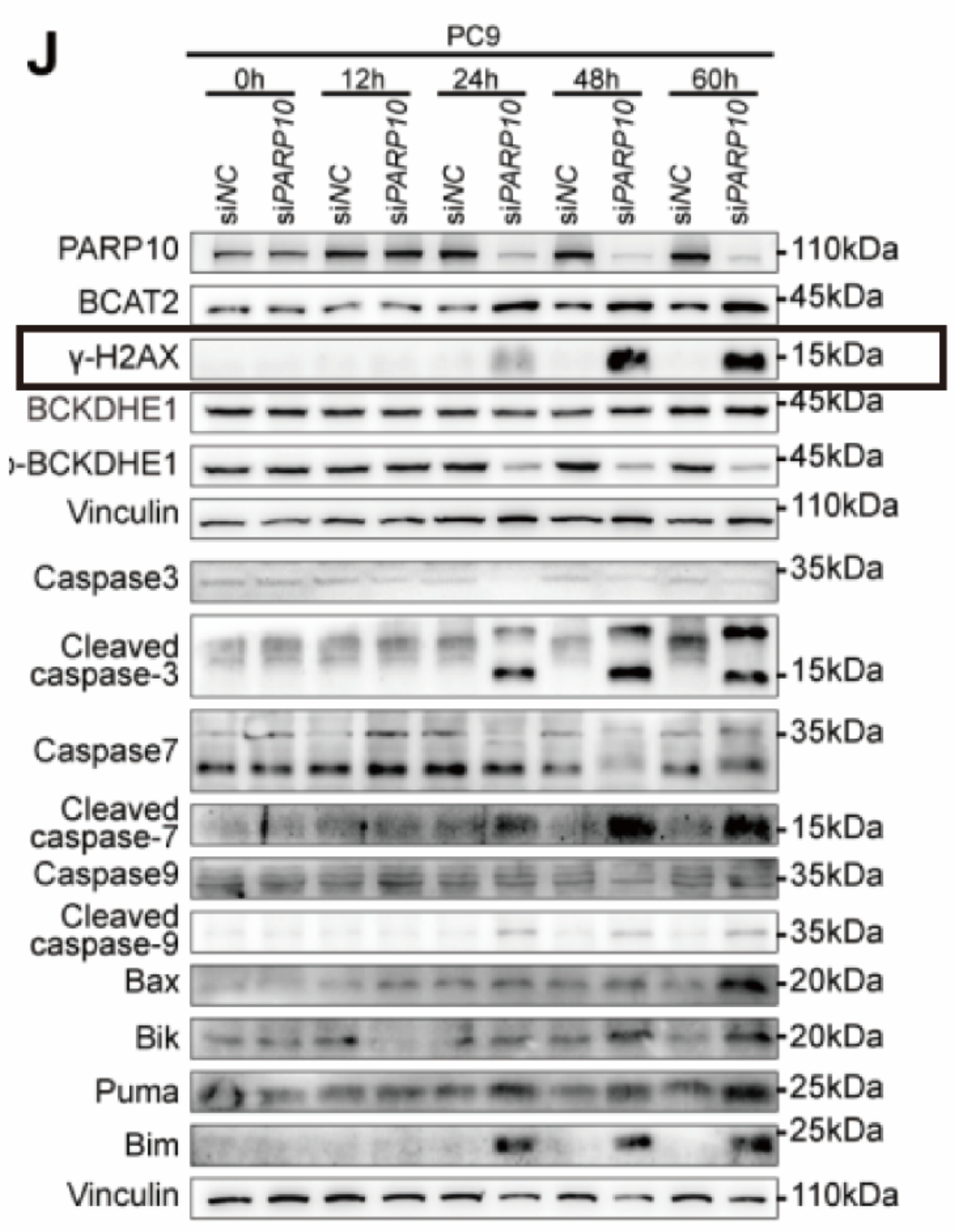

γ-H2AX

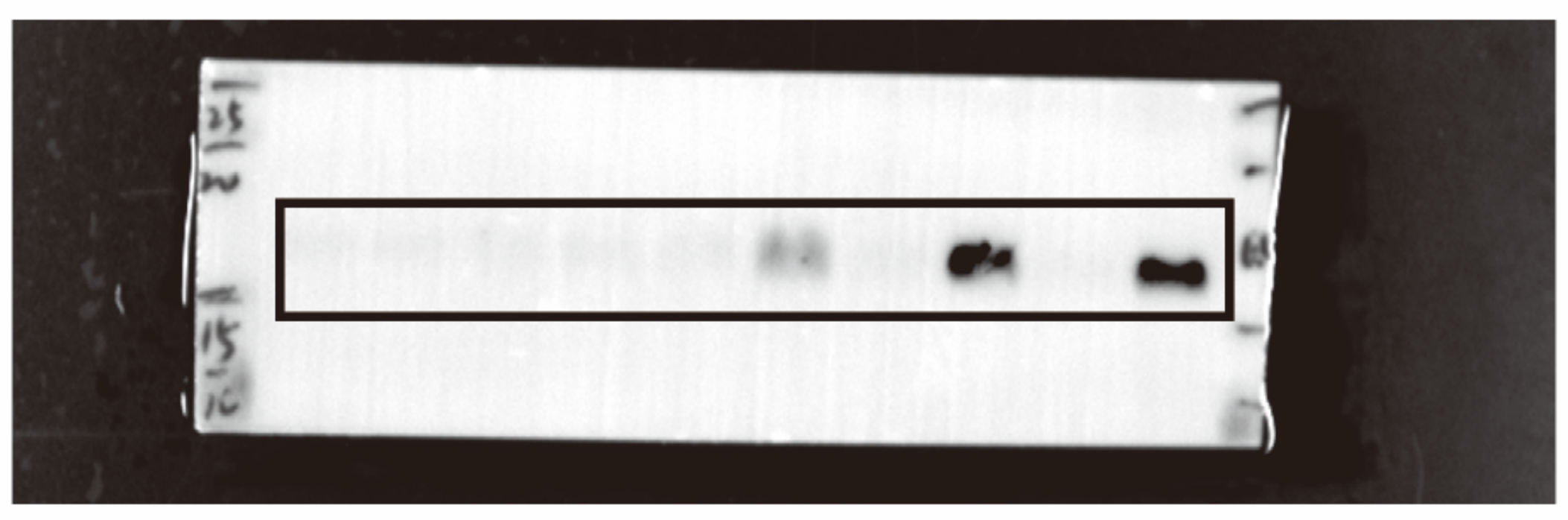

β-actin

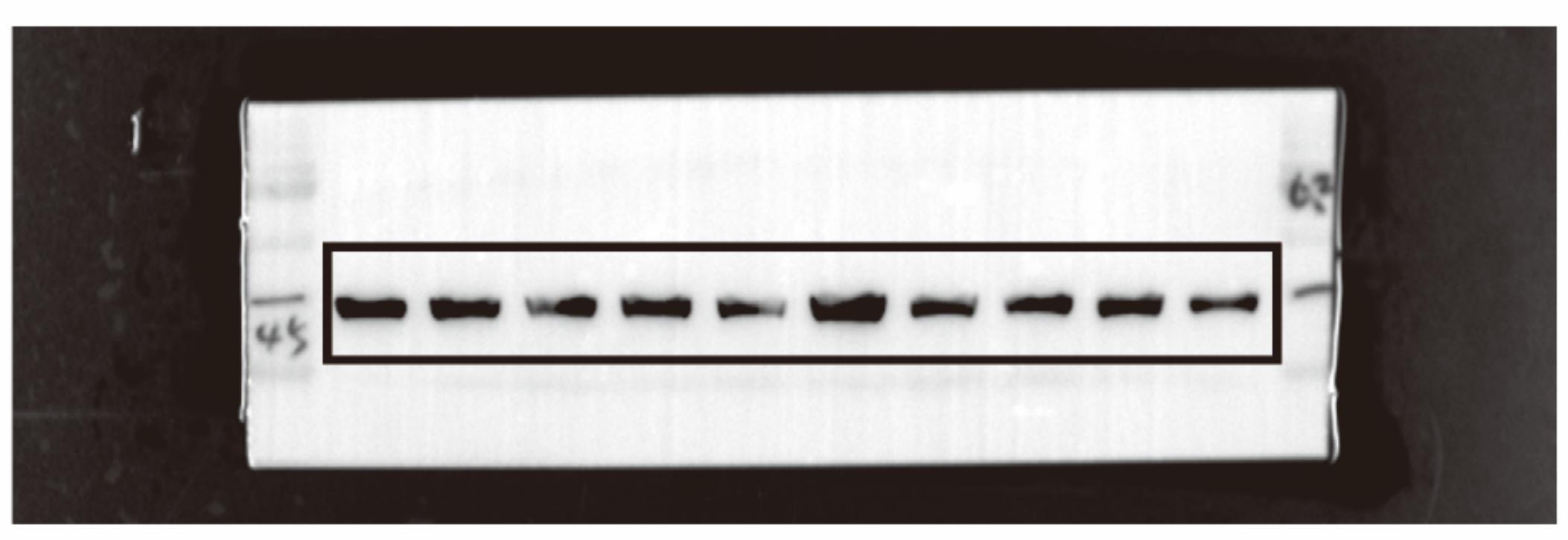

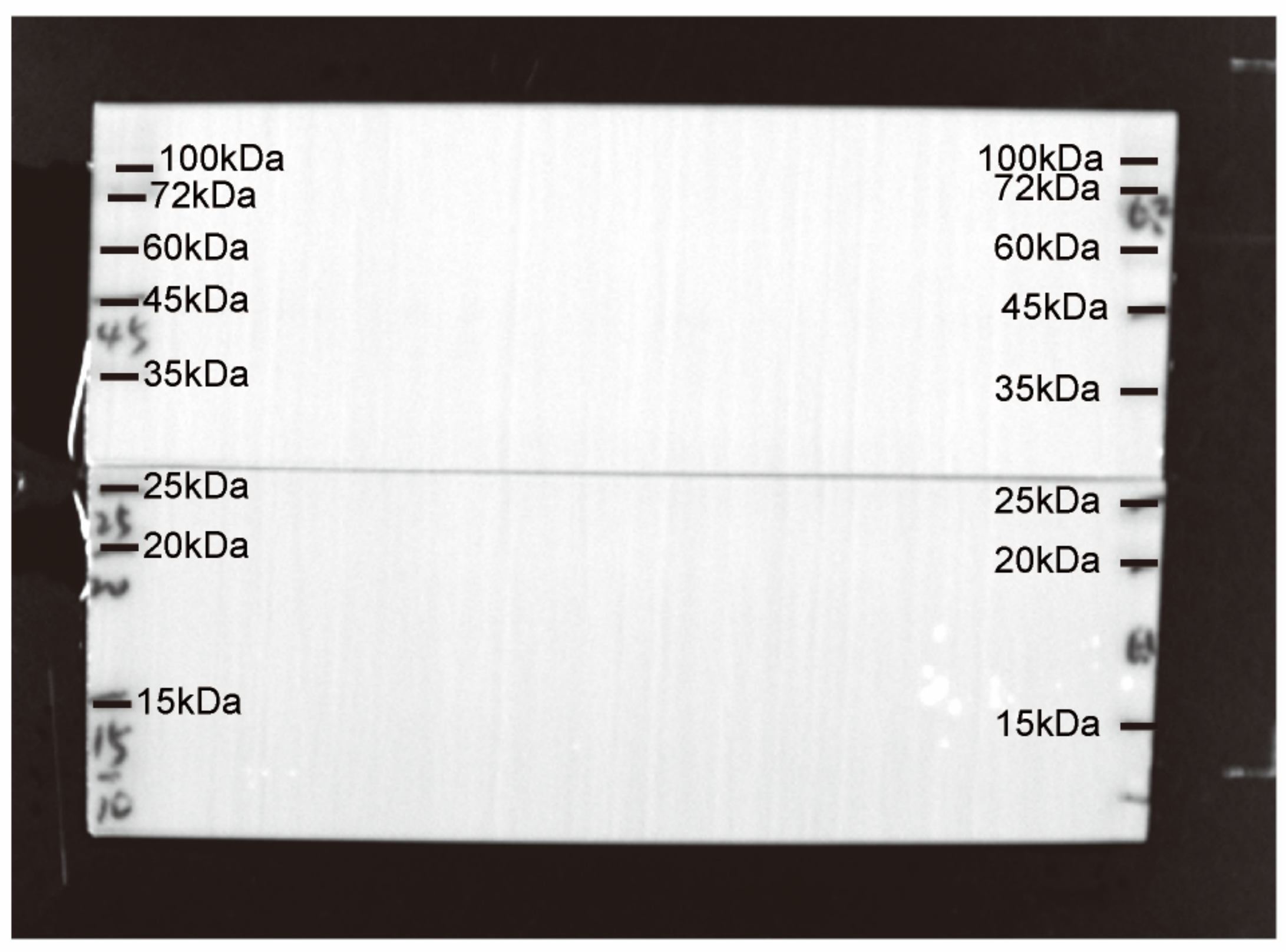

Figure	Merged	Stiched full image

Fig.5J

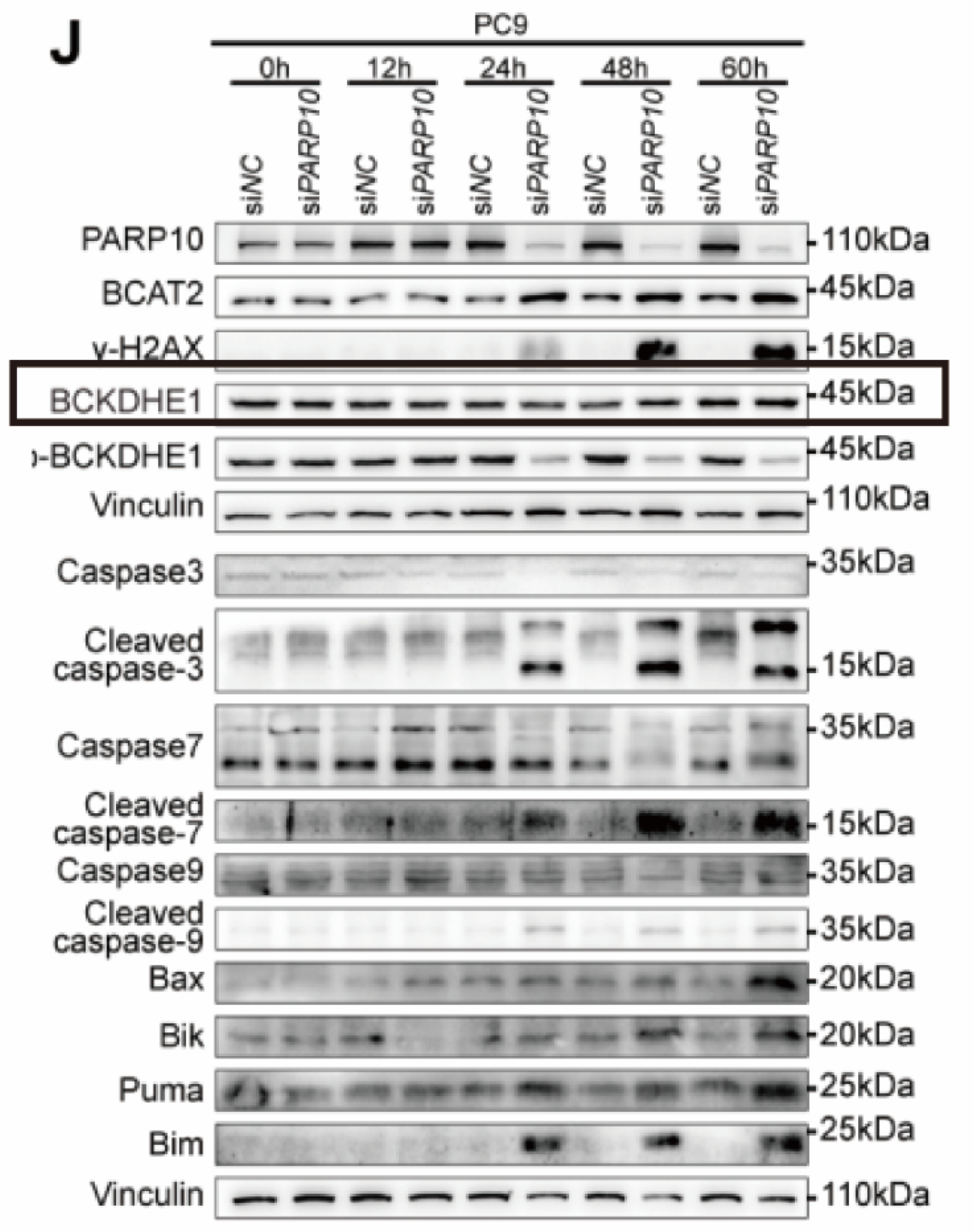

BCKDHE1

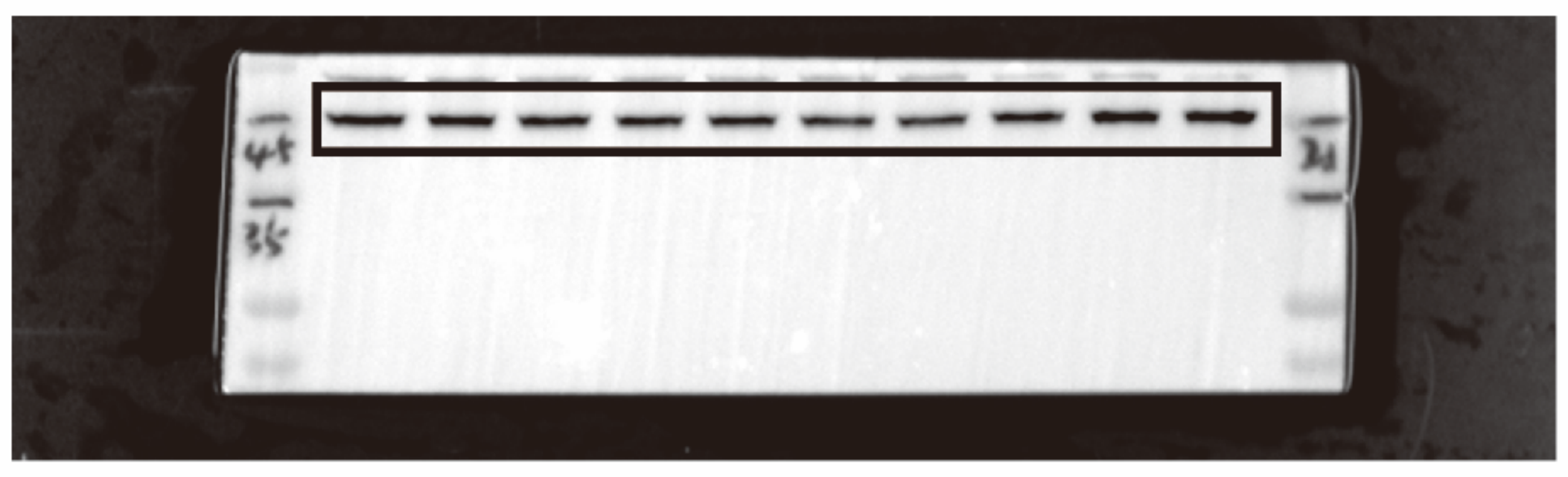

Vinculin

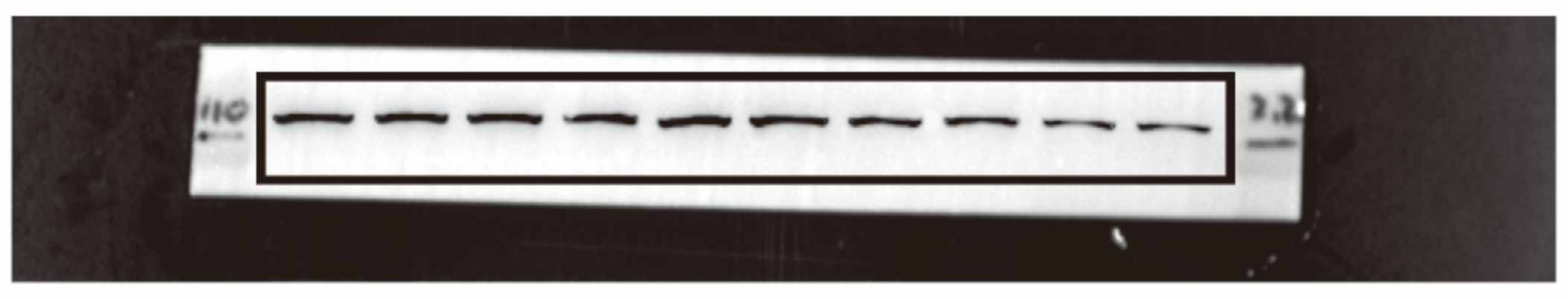

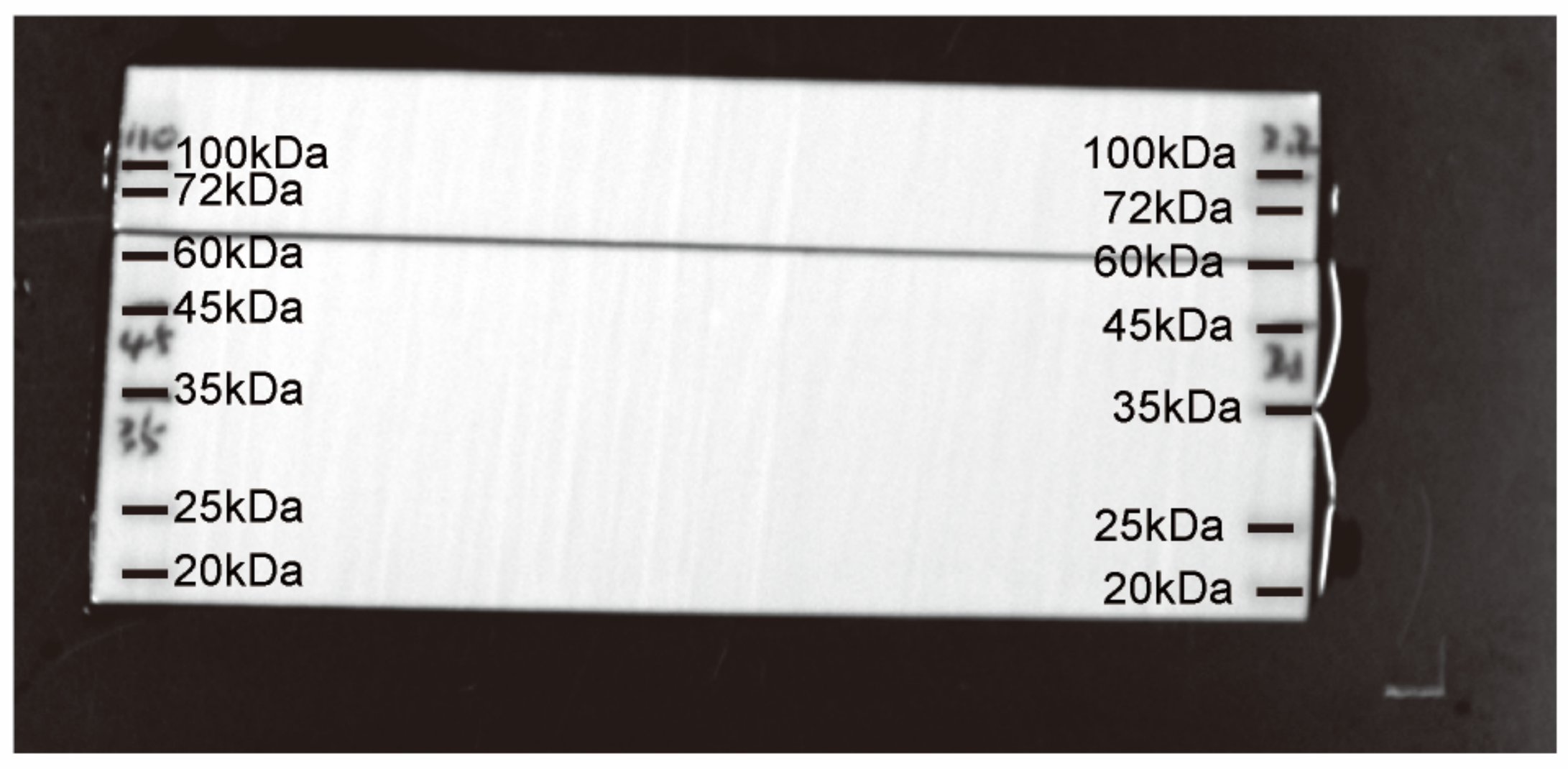

Fig.5J

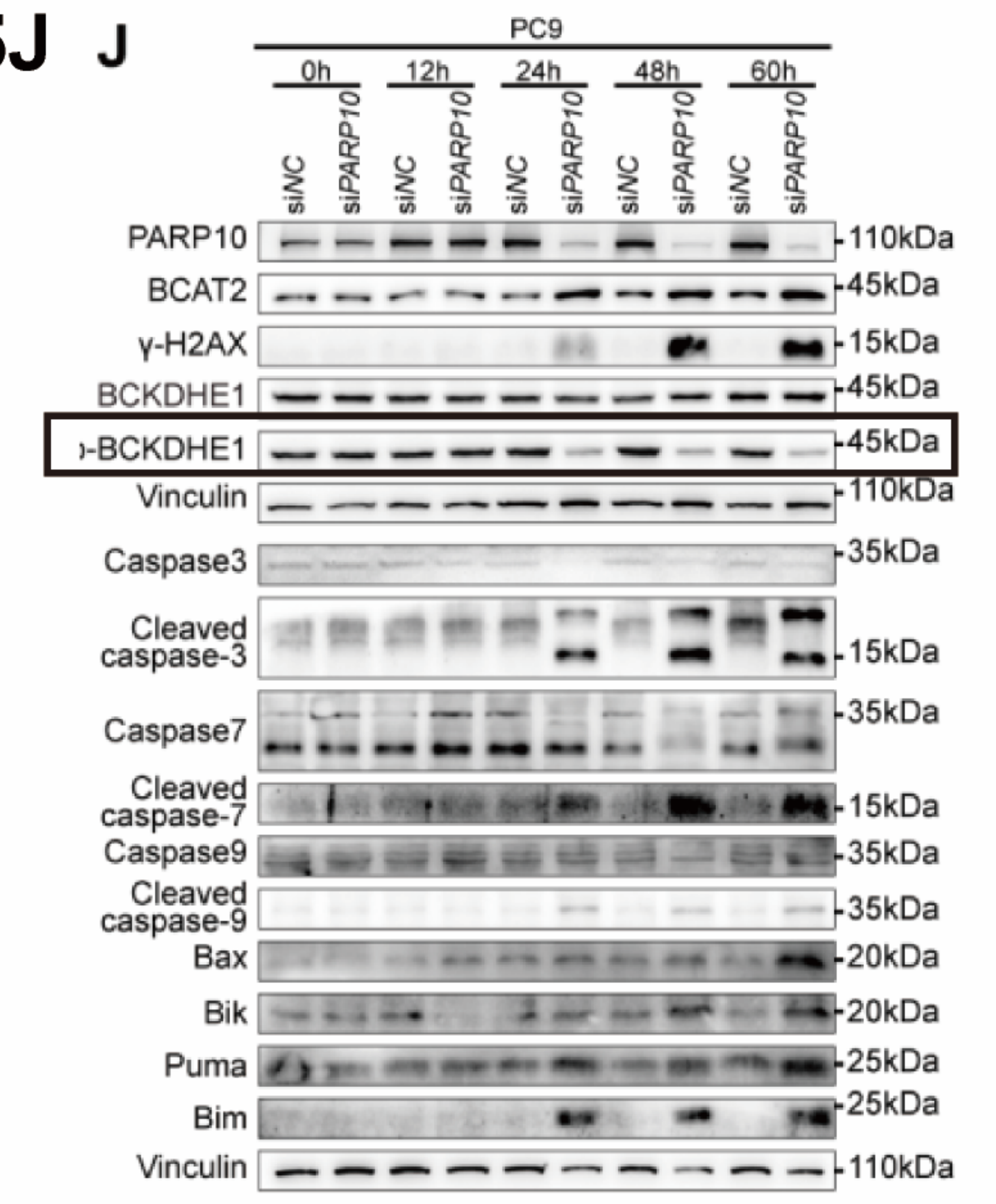

p-BCKDHE1

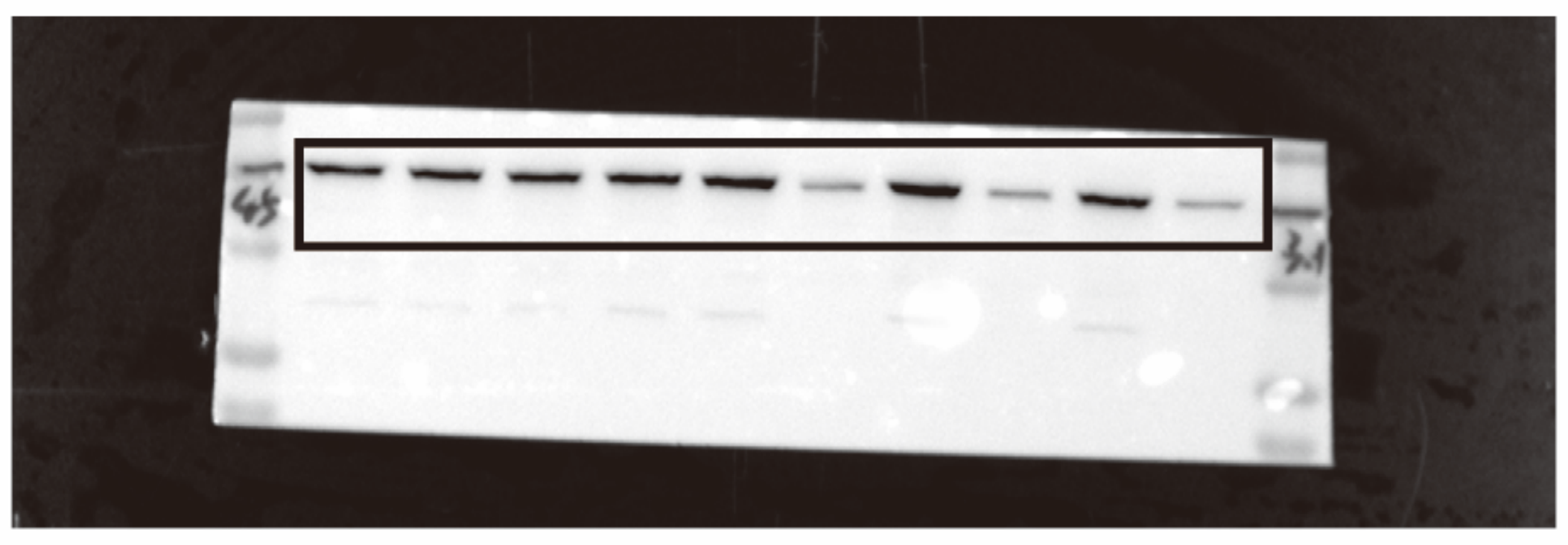

Vinculin

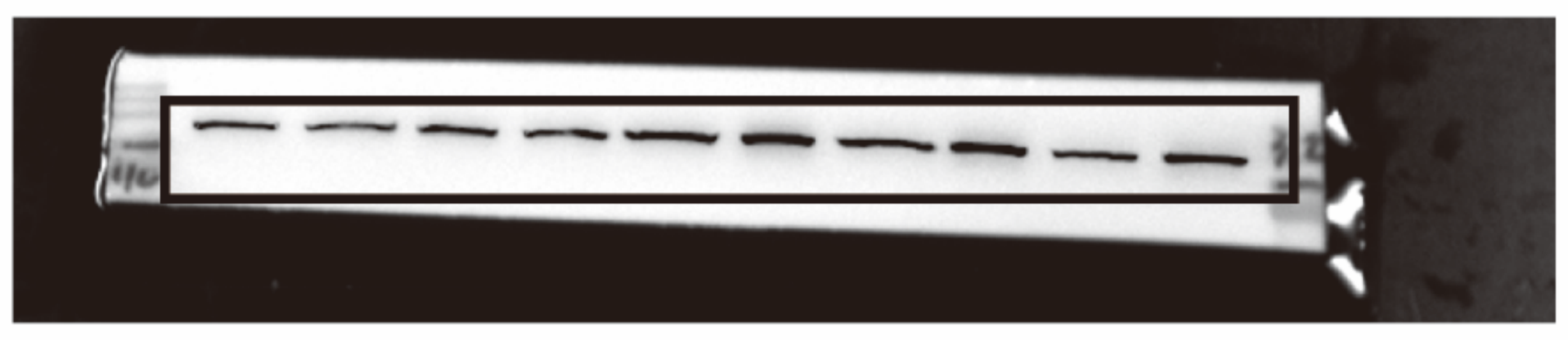

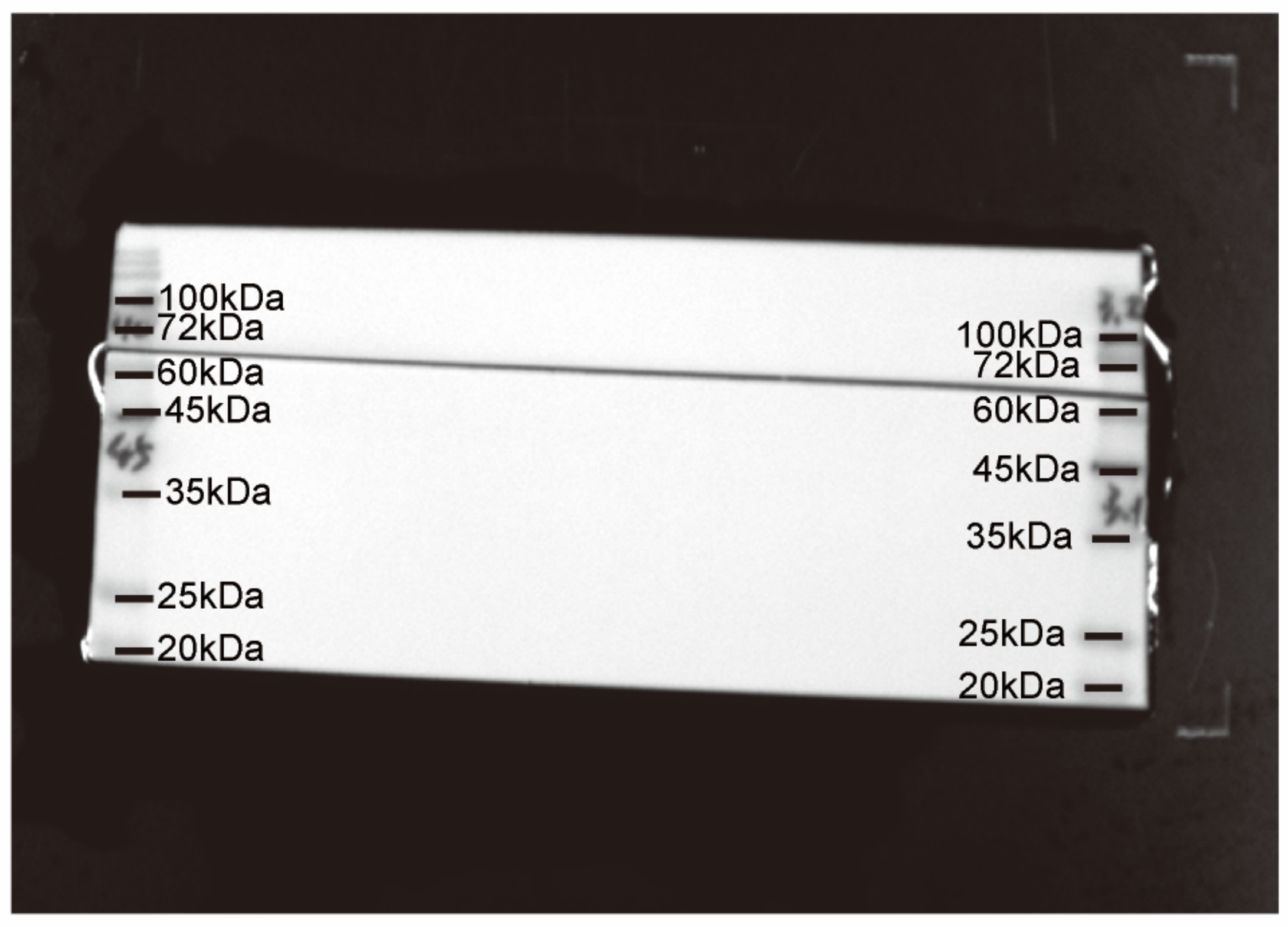

Fig.5J

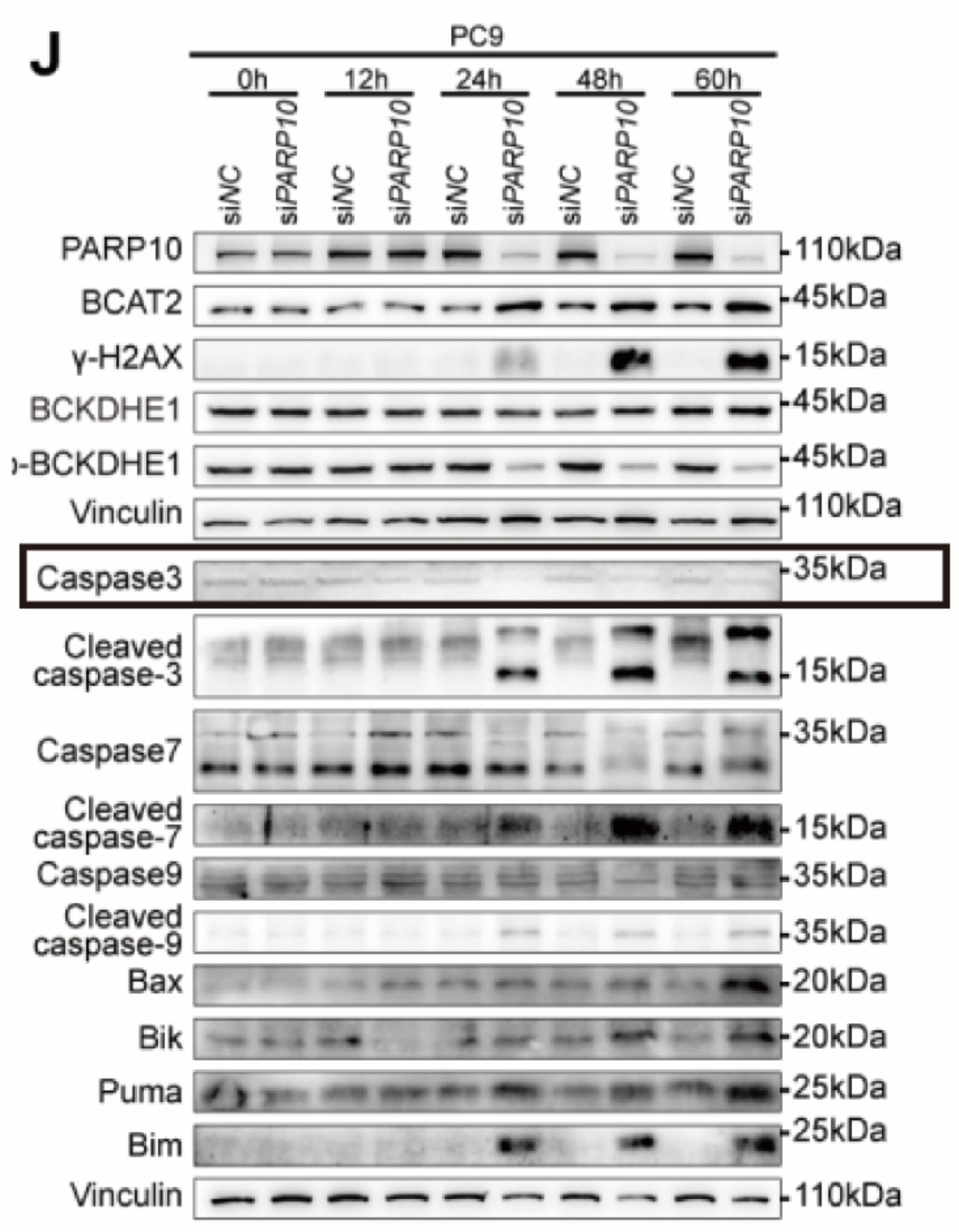

Caspase3

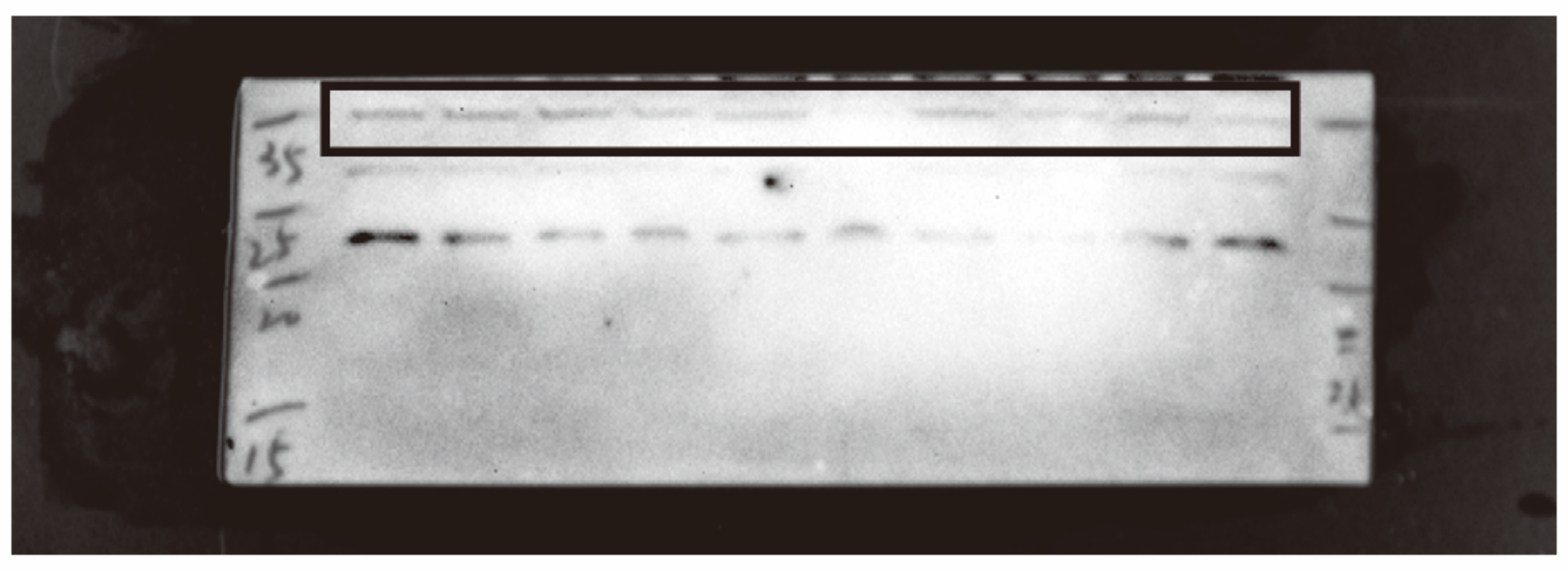

Vinculin

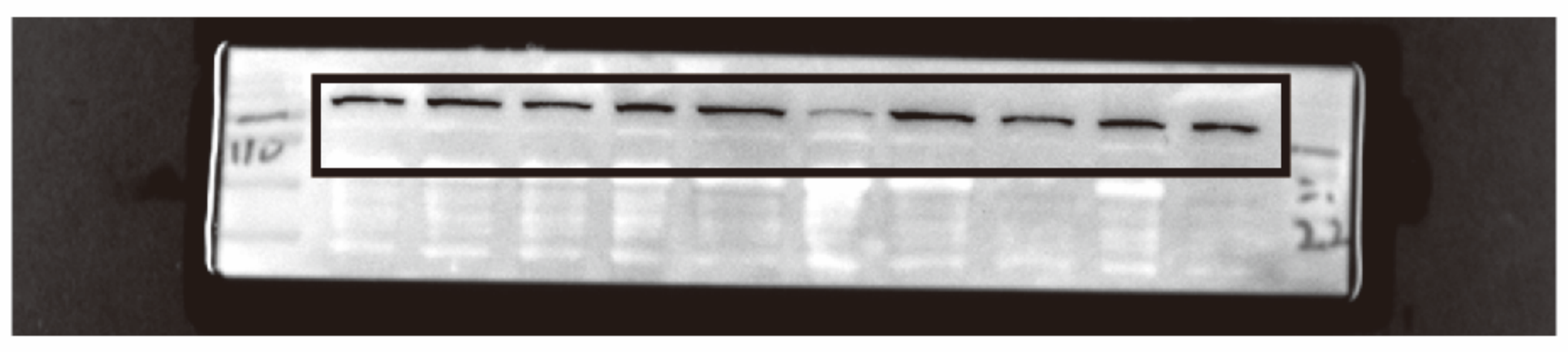

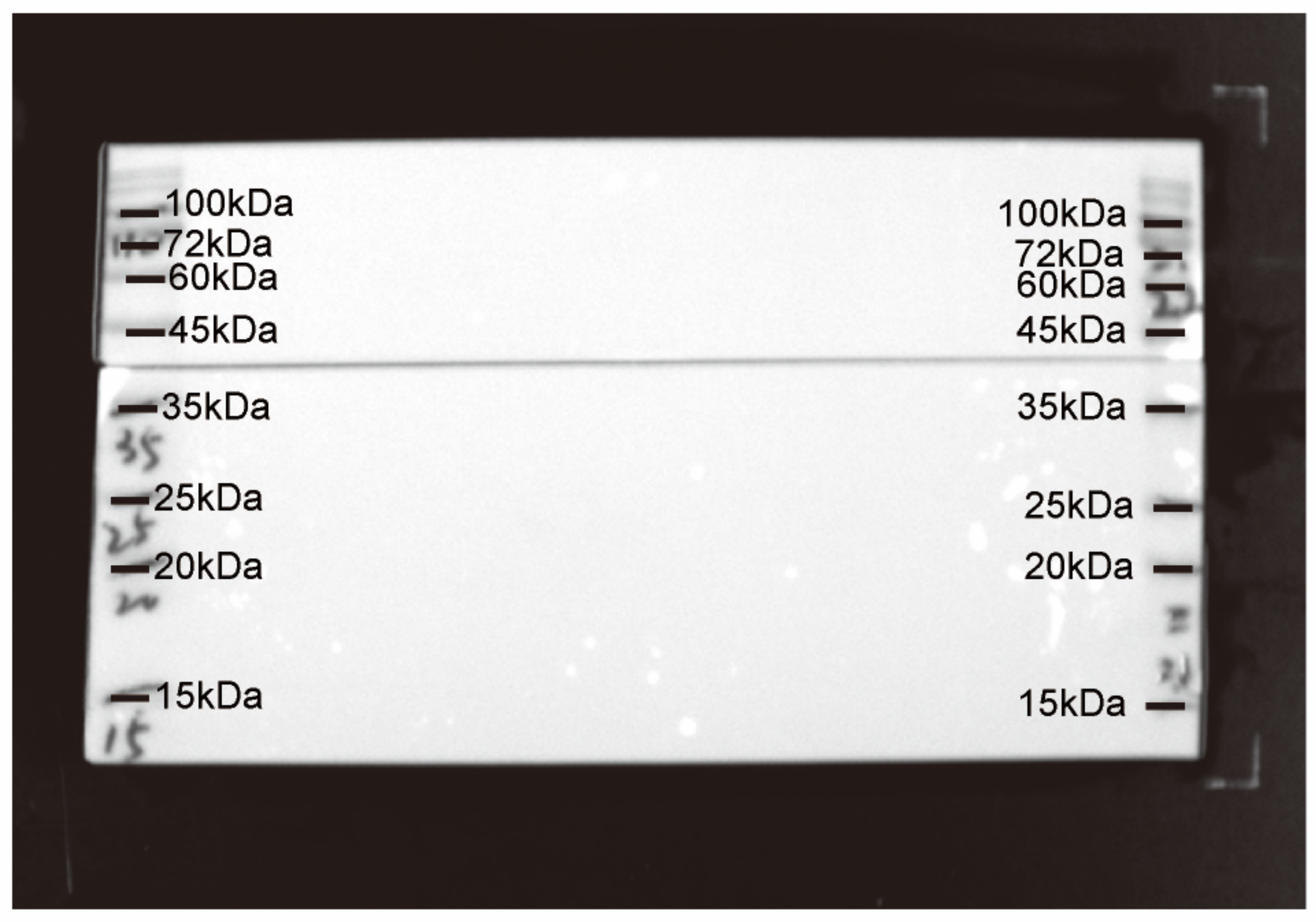

Fig.5J

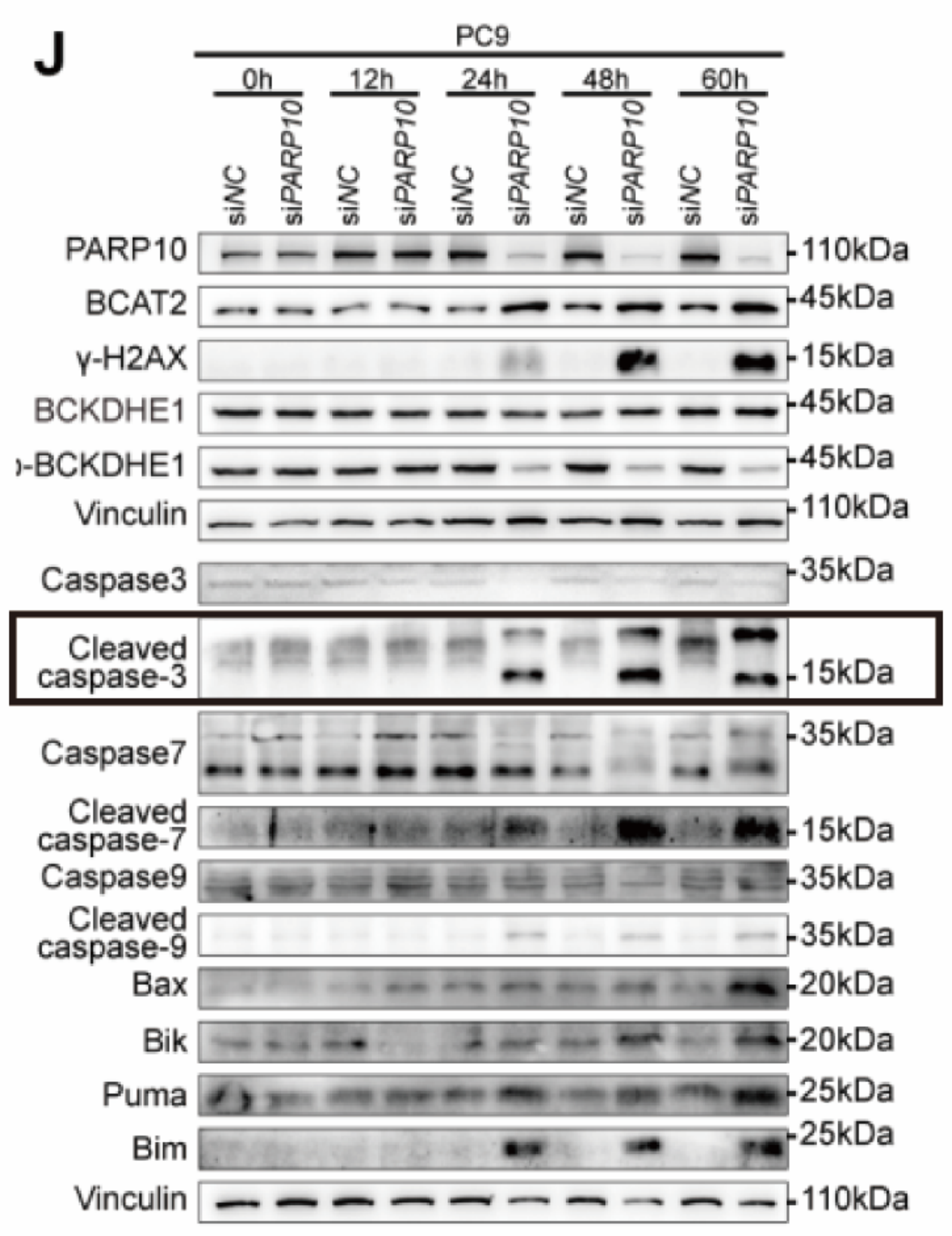

Cleaved caspase3

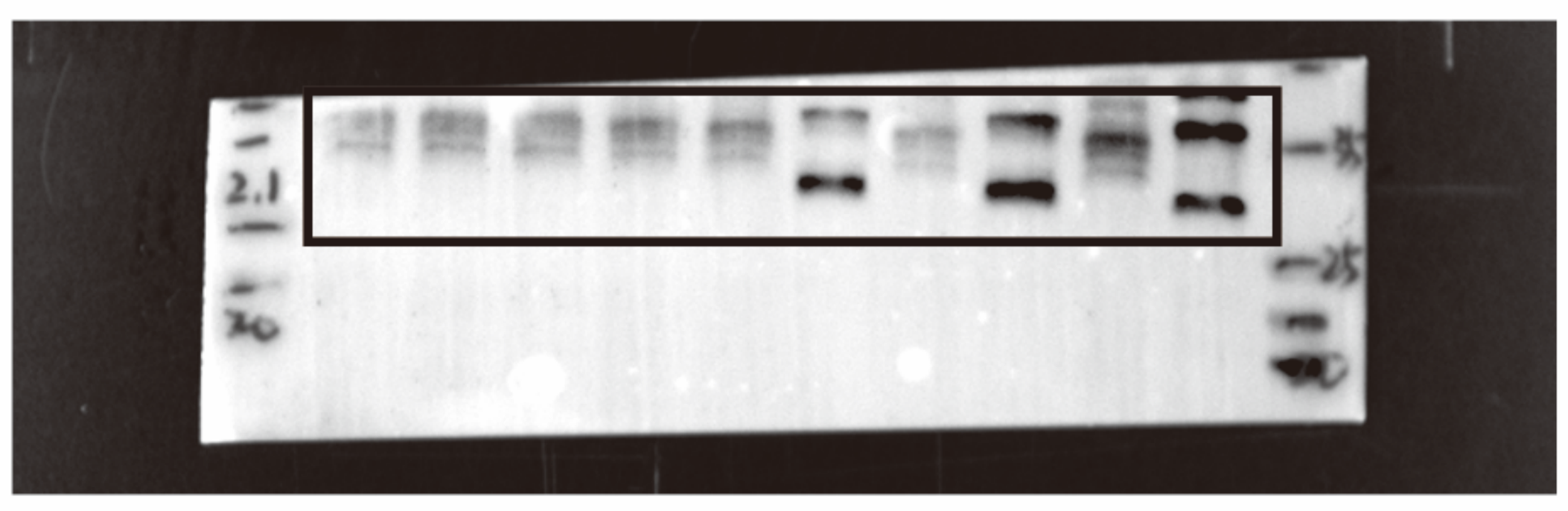

Vinculin

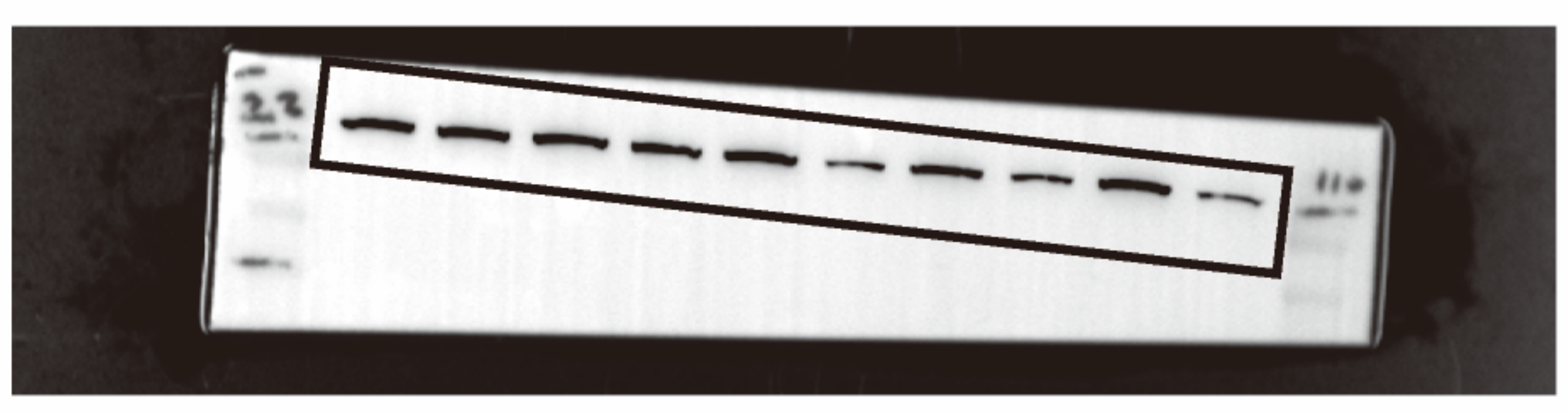

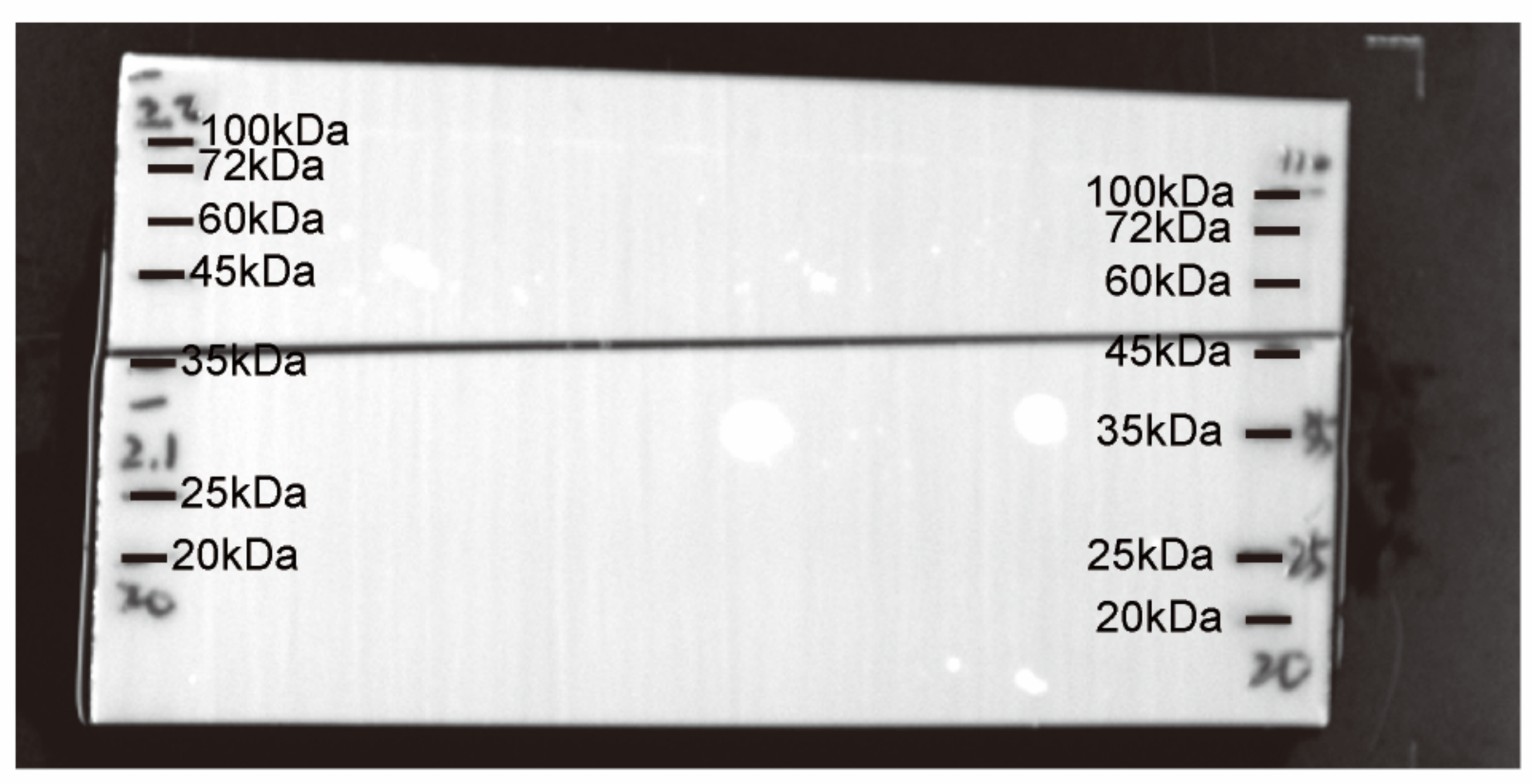

Figure	Merged	Stiched full image

Fig.5J

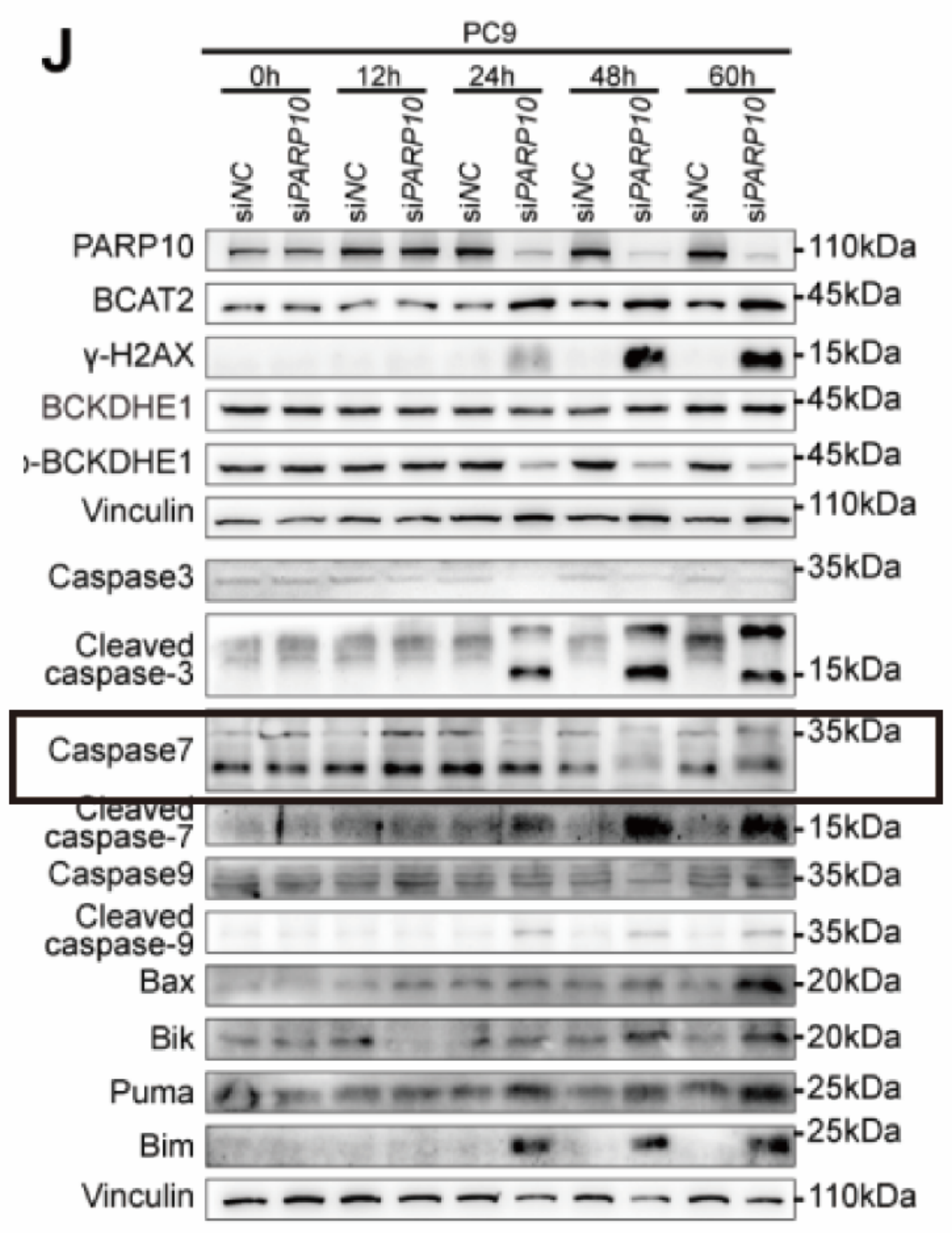

Caspase7

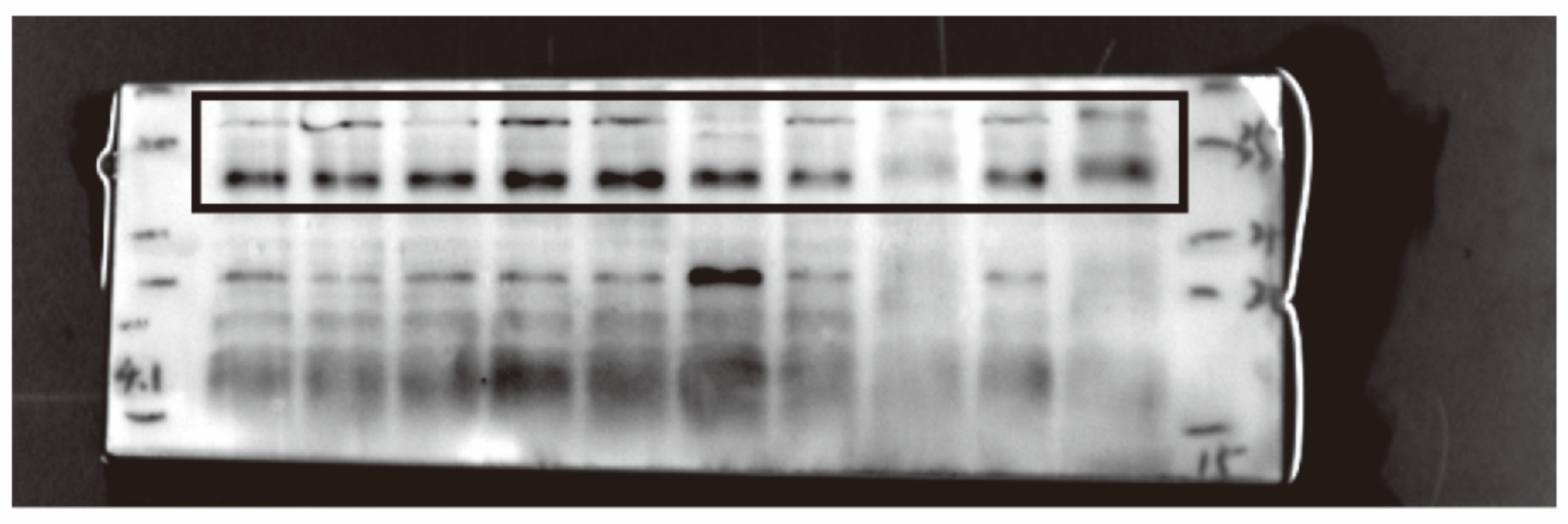

Vinculin

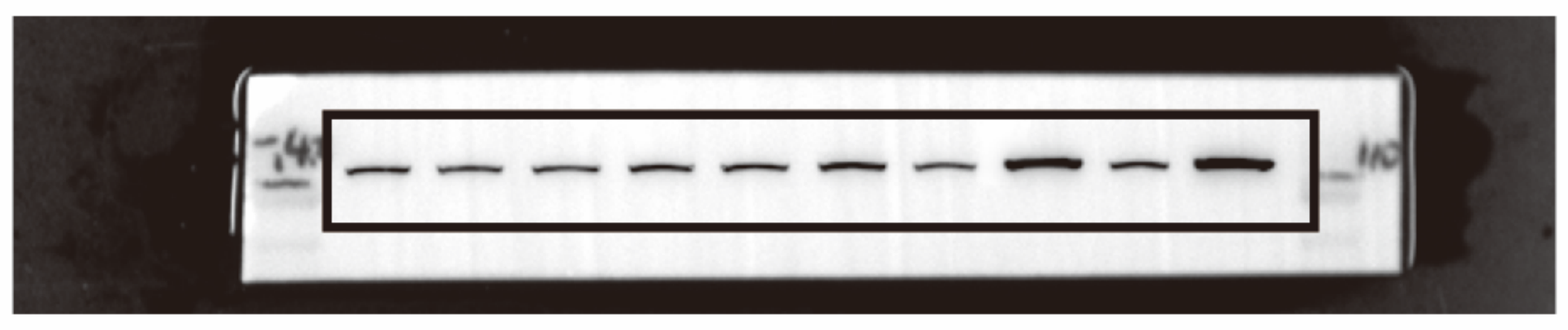

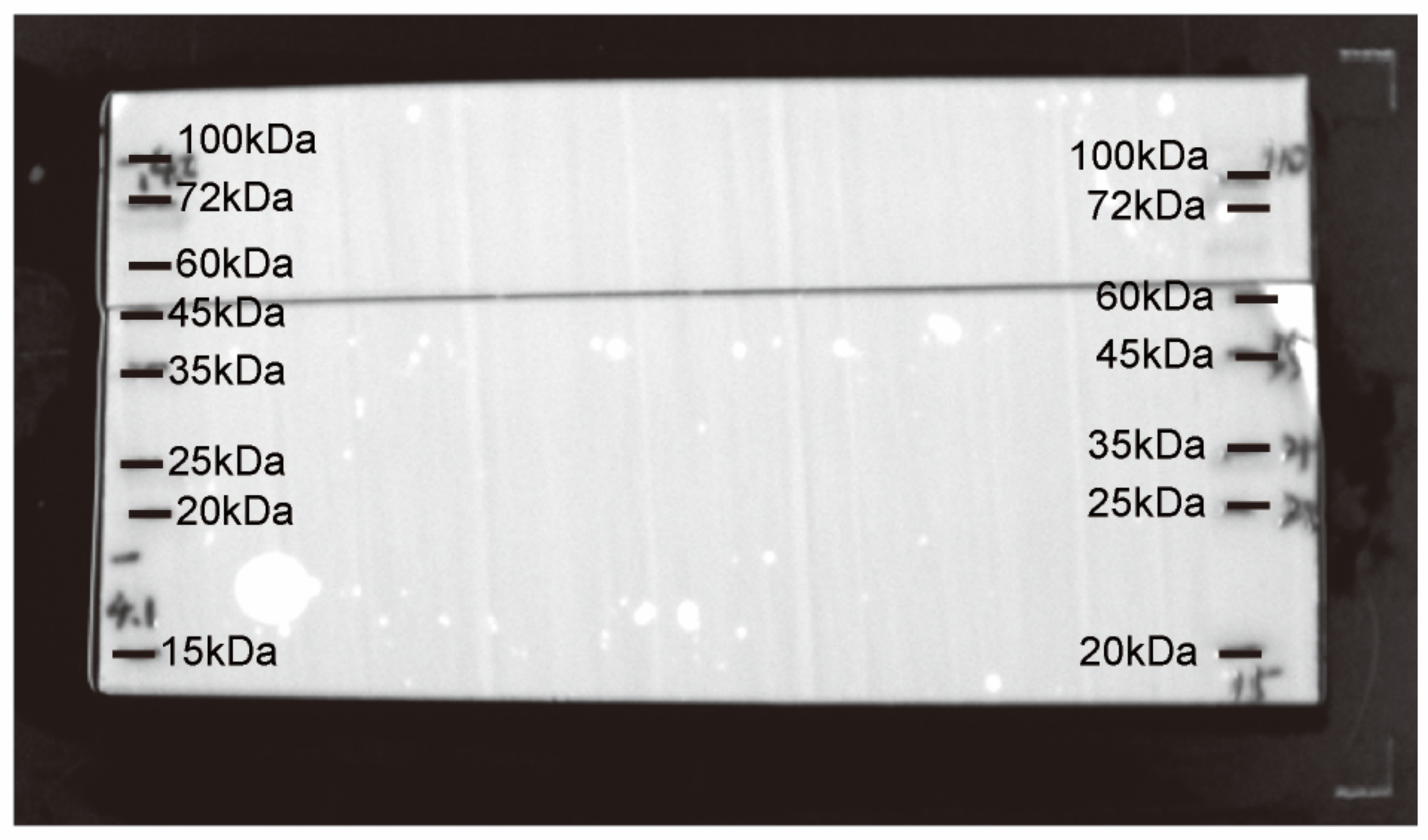

Fig.5J

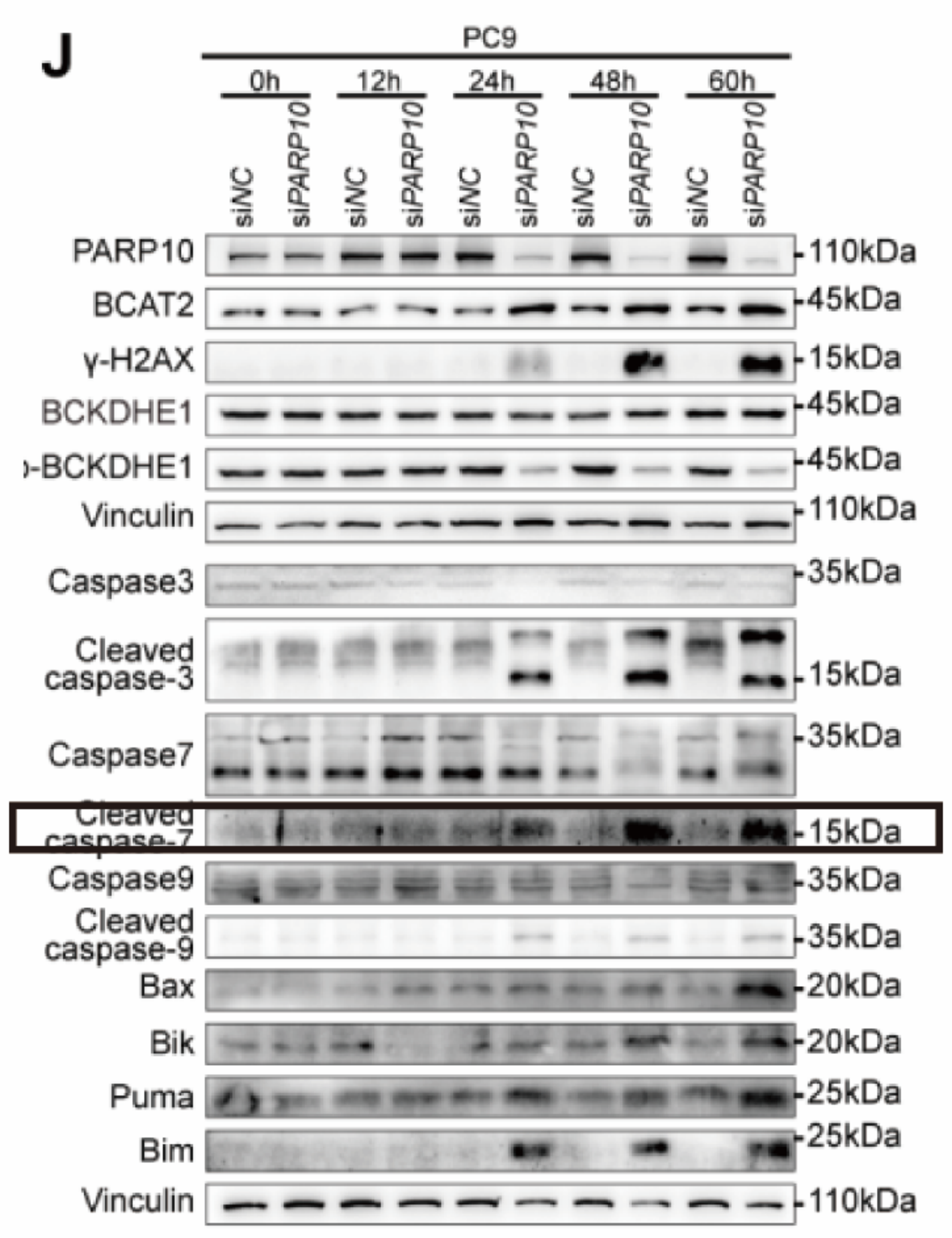

Cleaved caspase7

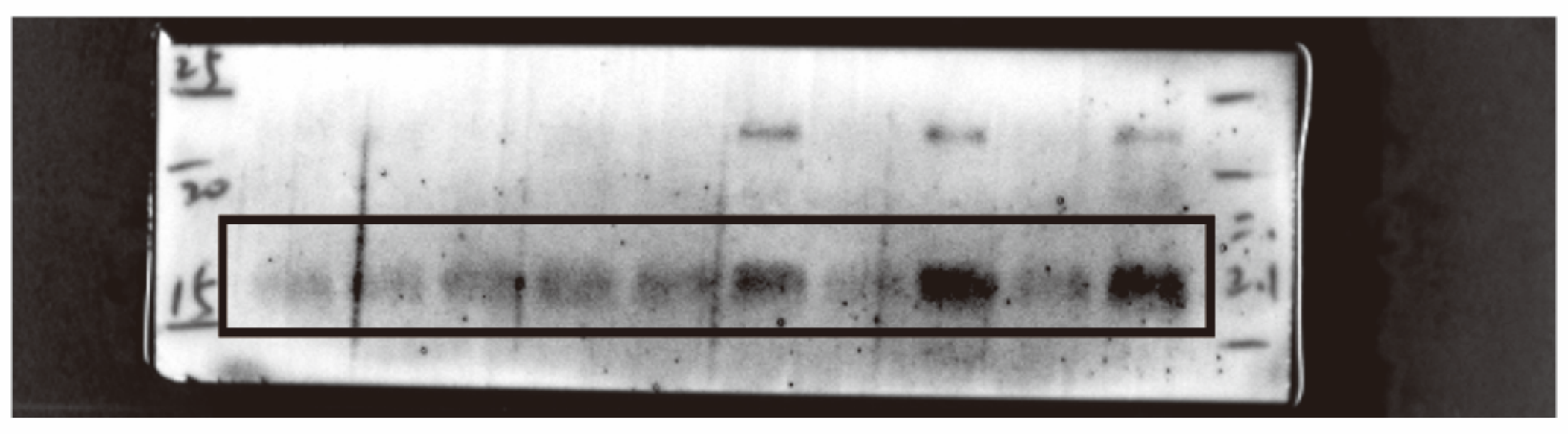

β-actin

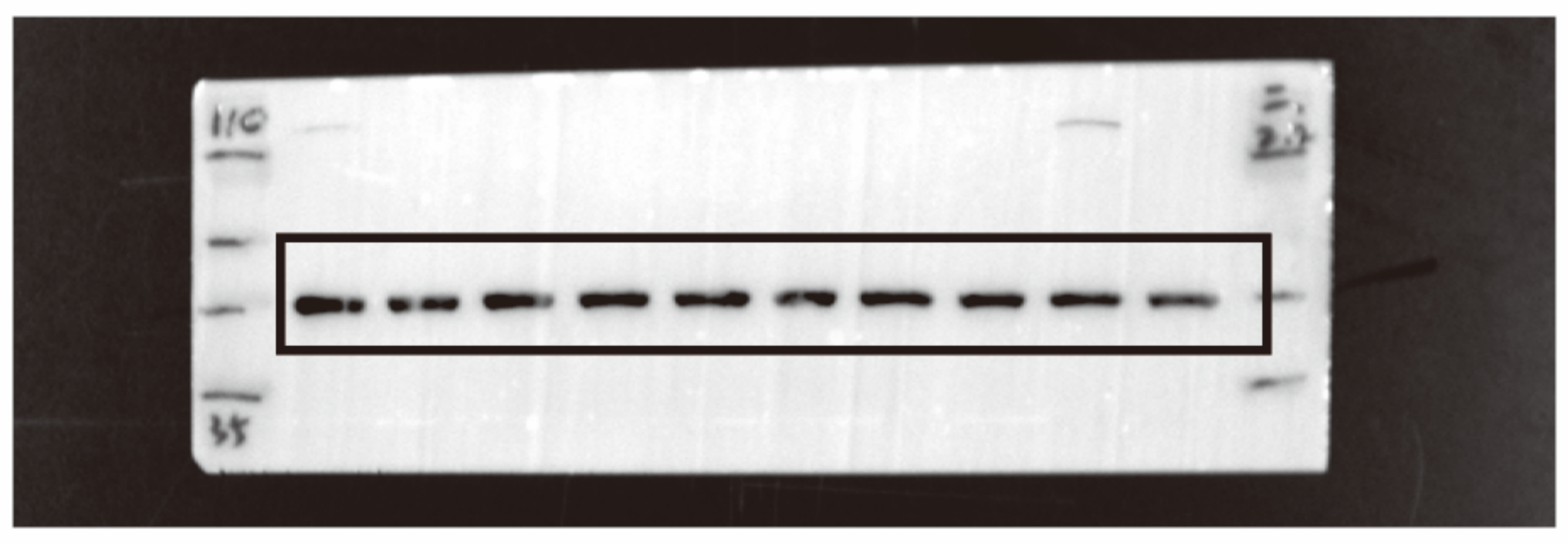

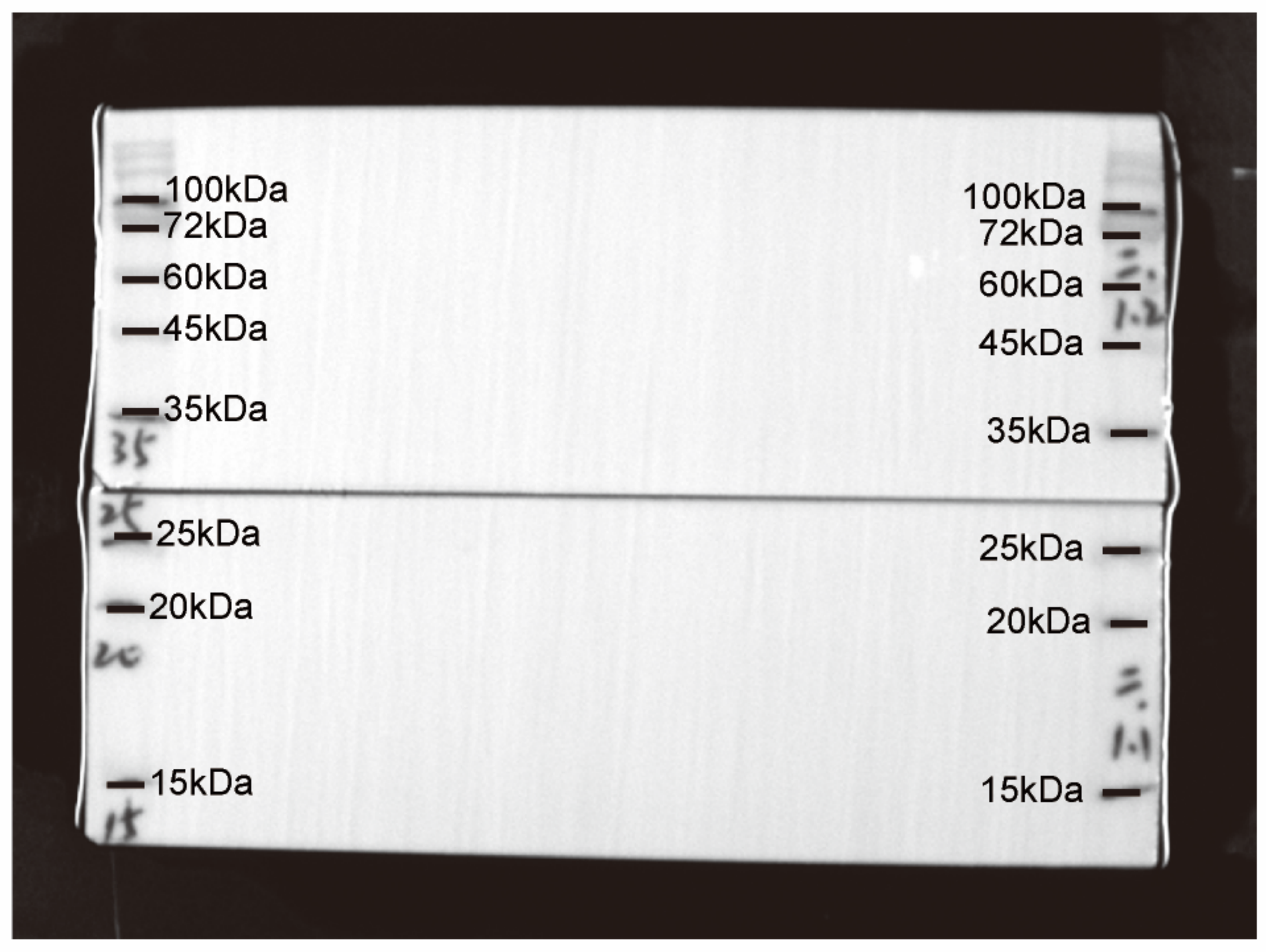

Fig.5J

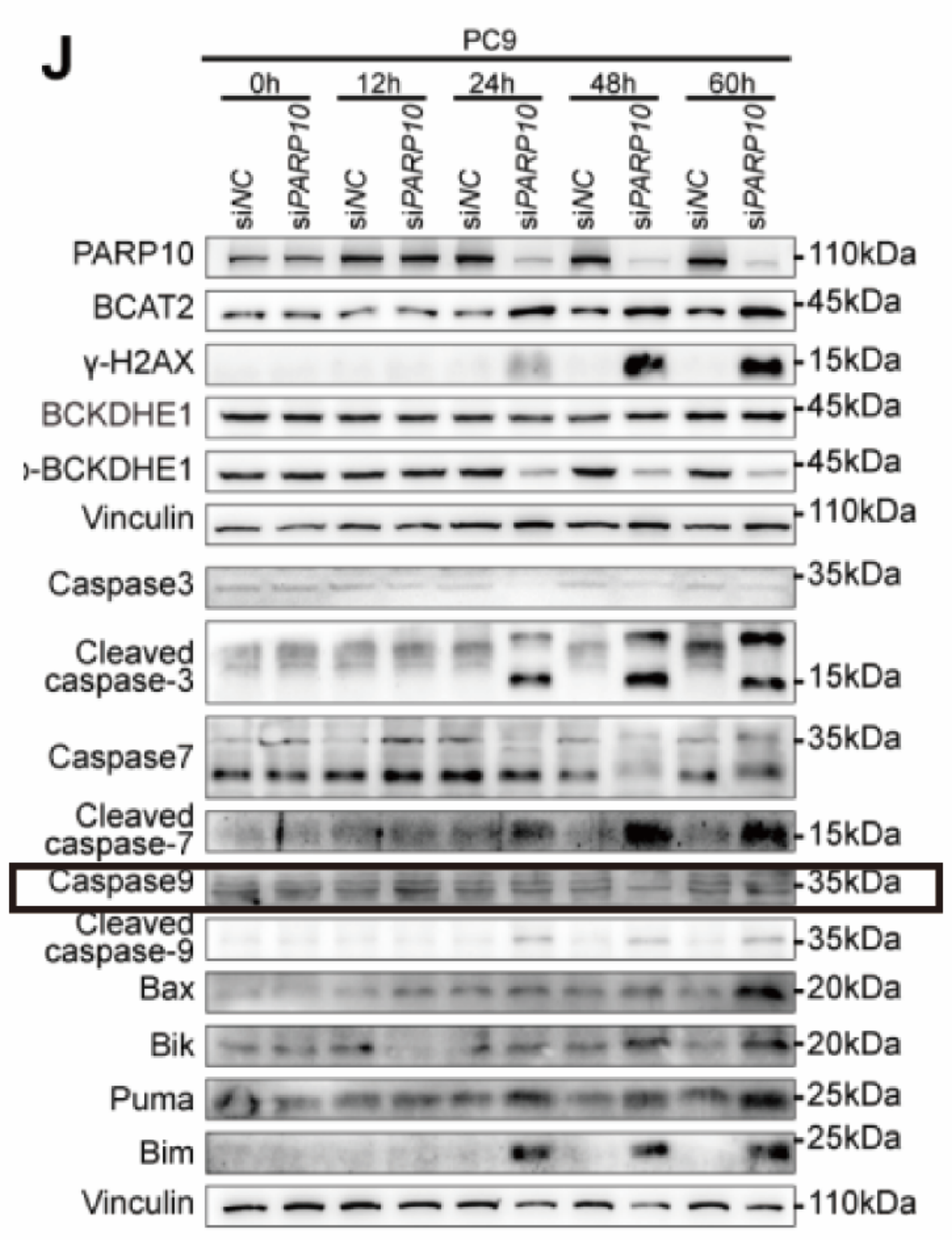

Caspase9

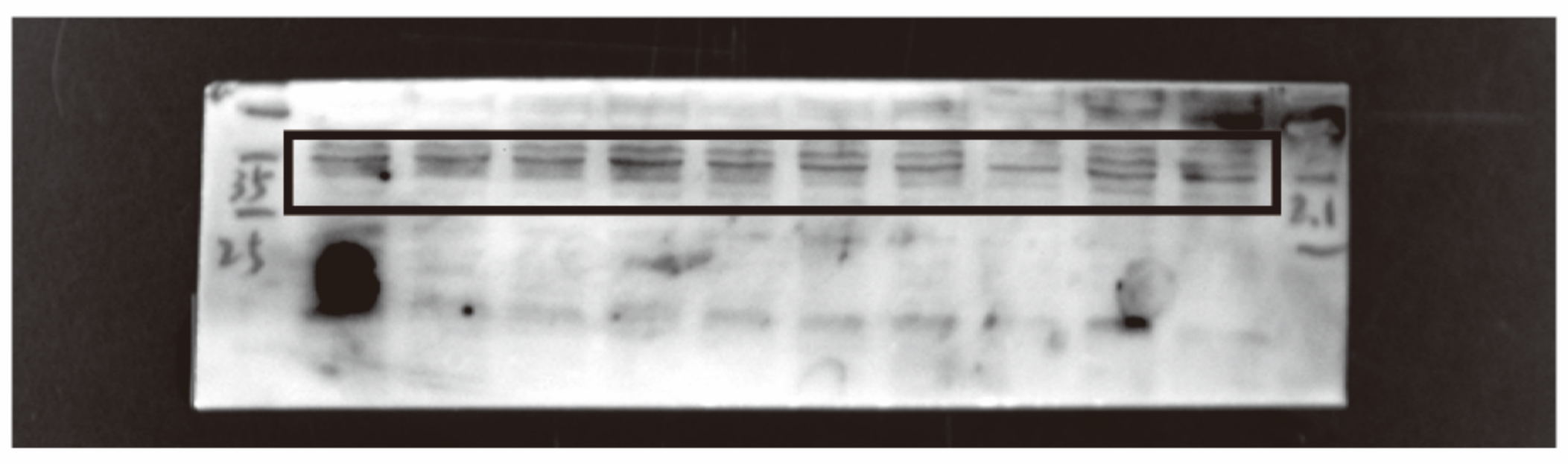

Vinculin

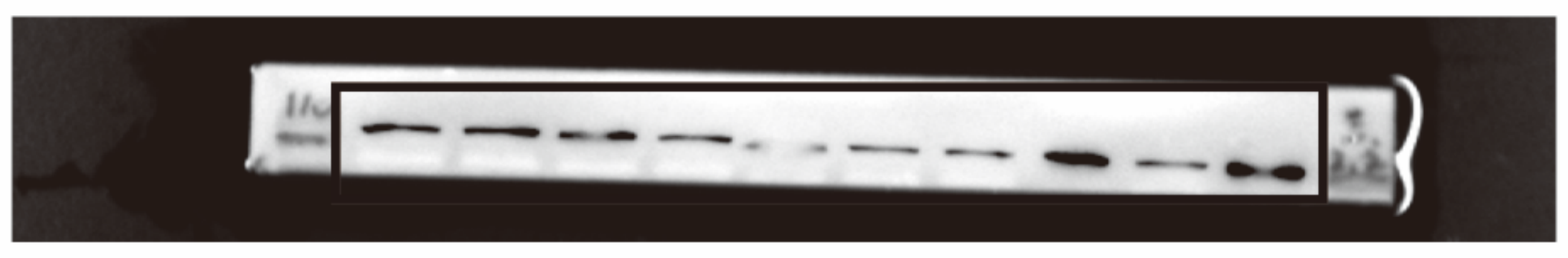

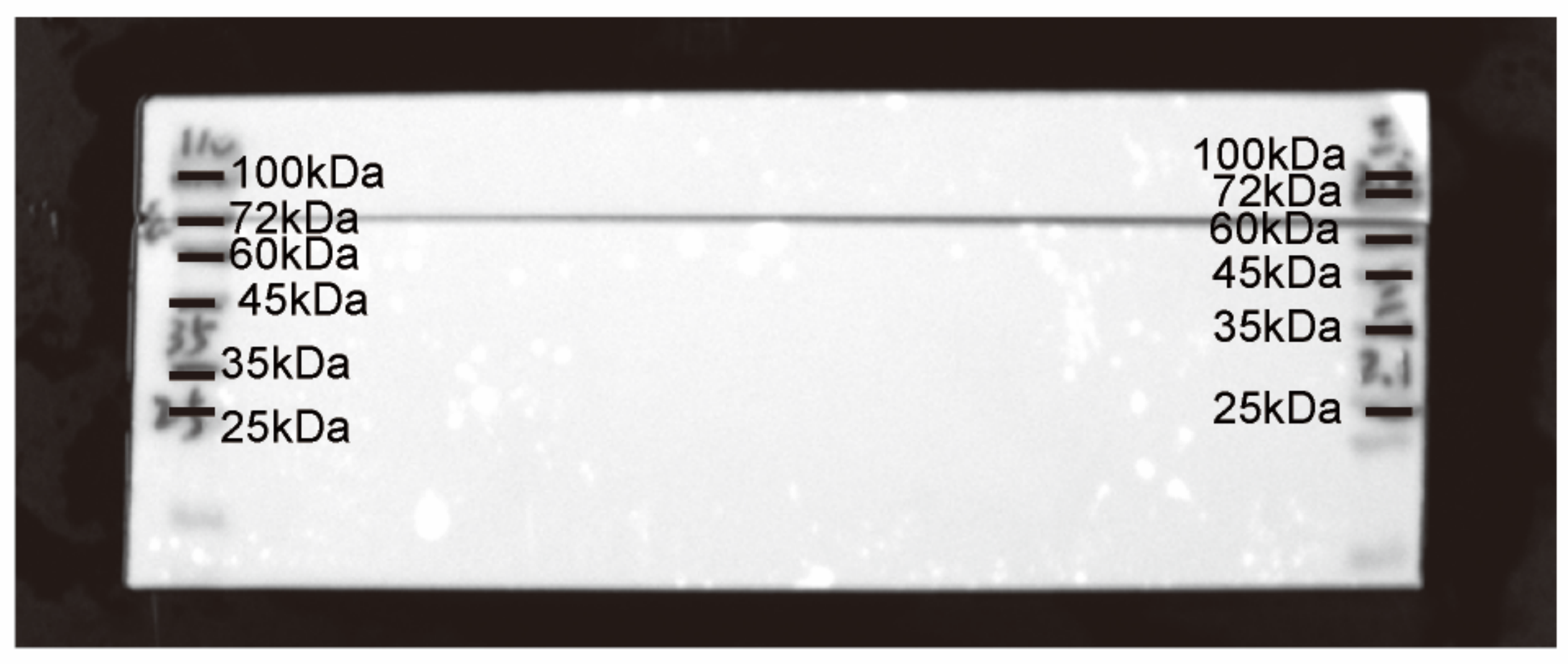

Fig.5J

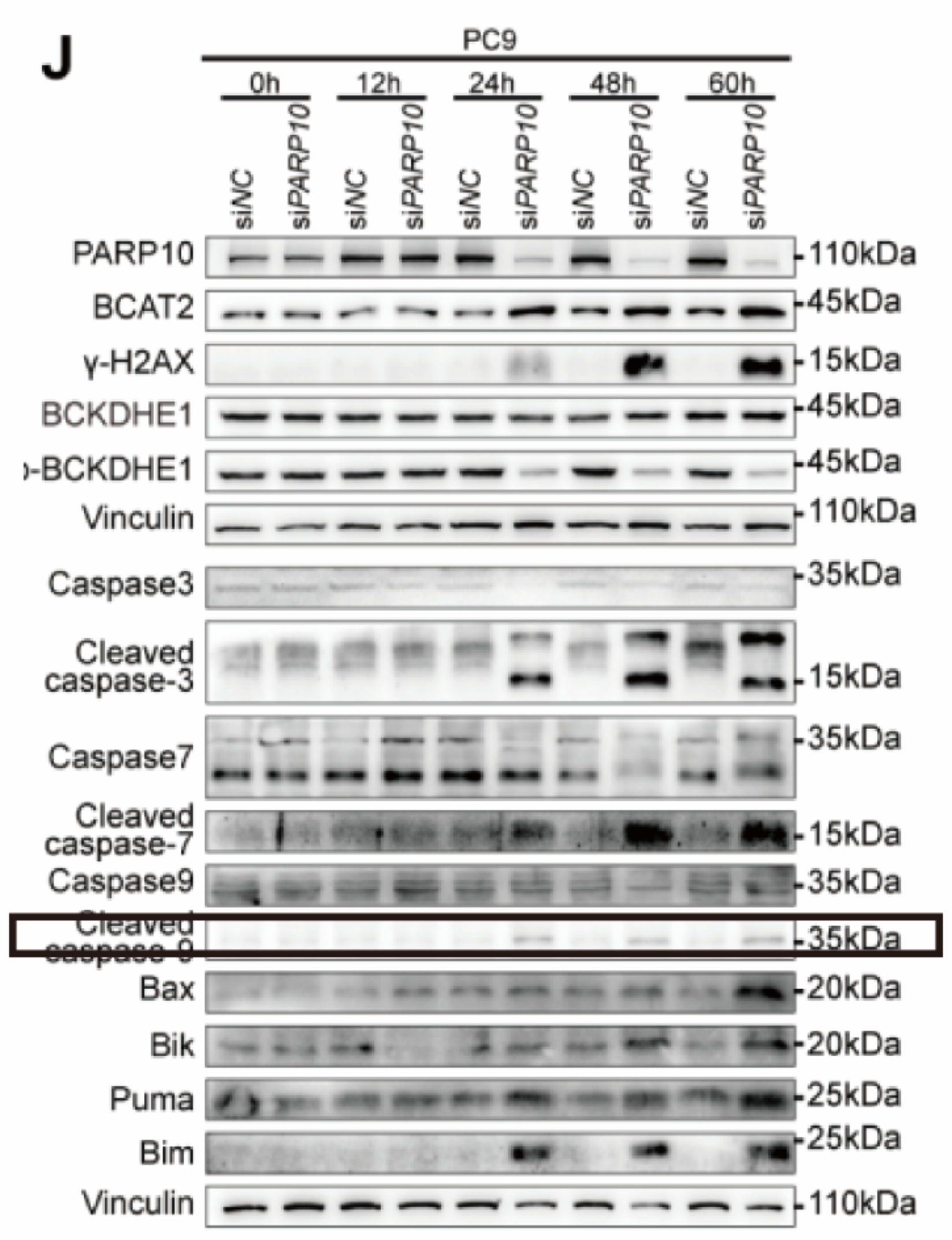

Cleaved caspase9

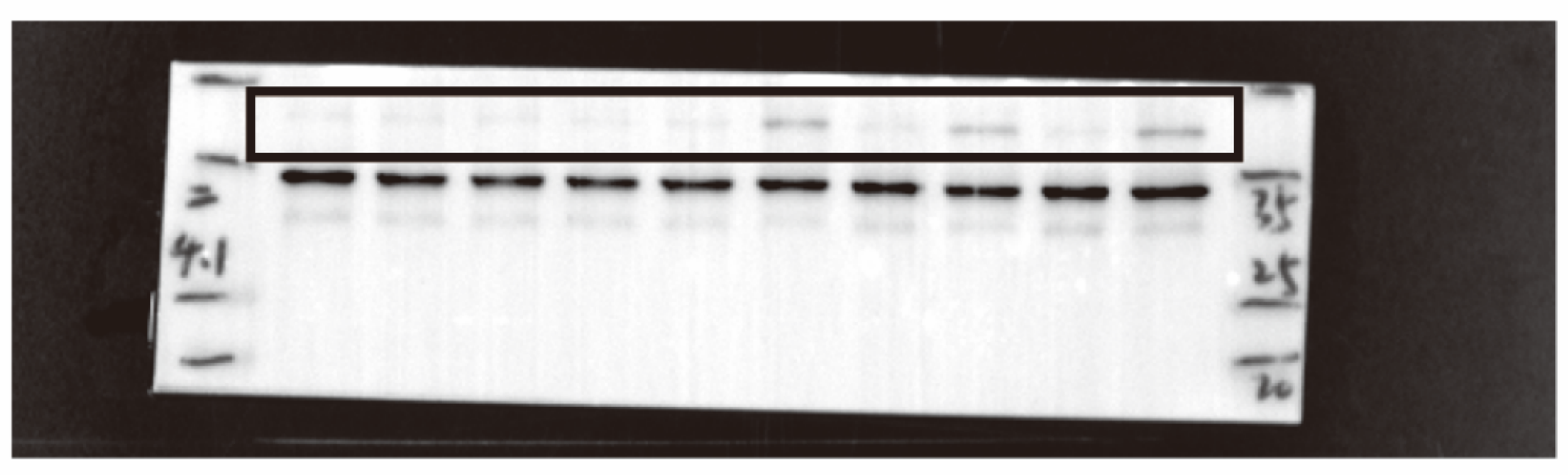

Vinculin

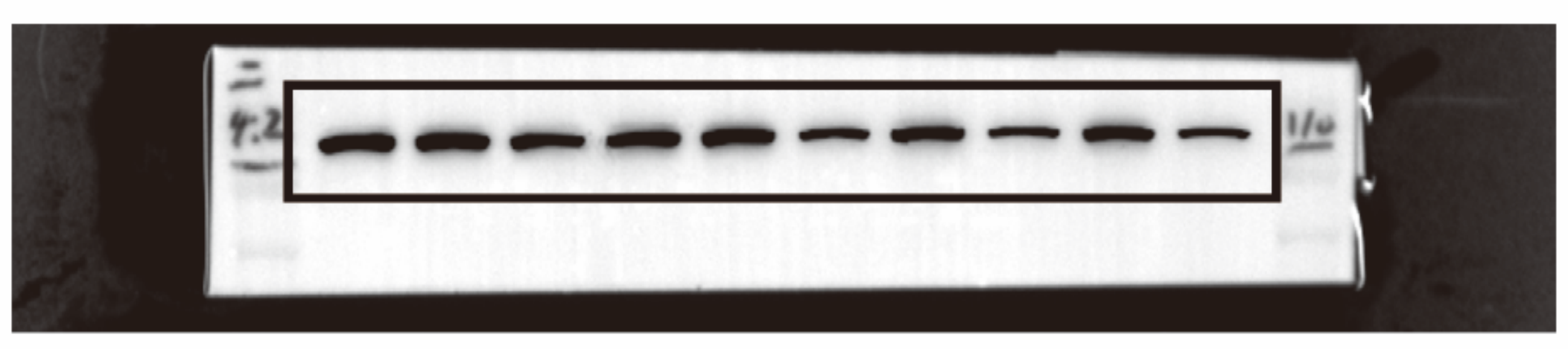

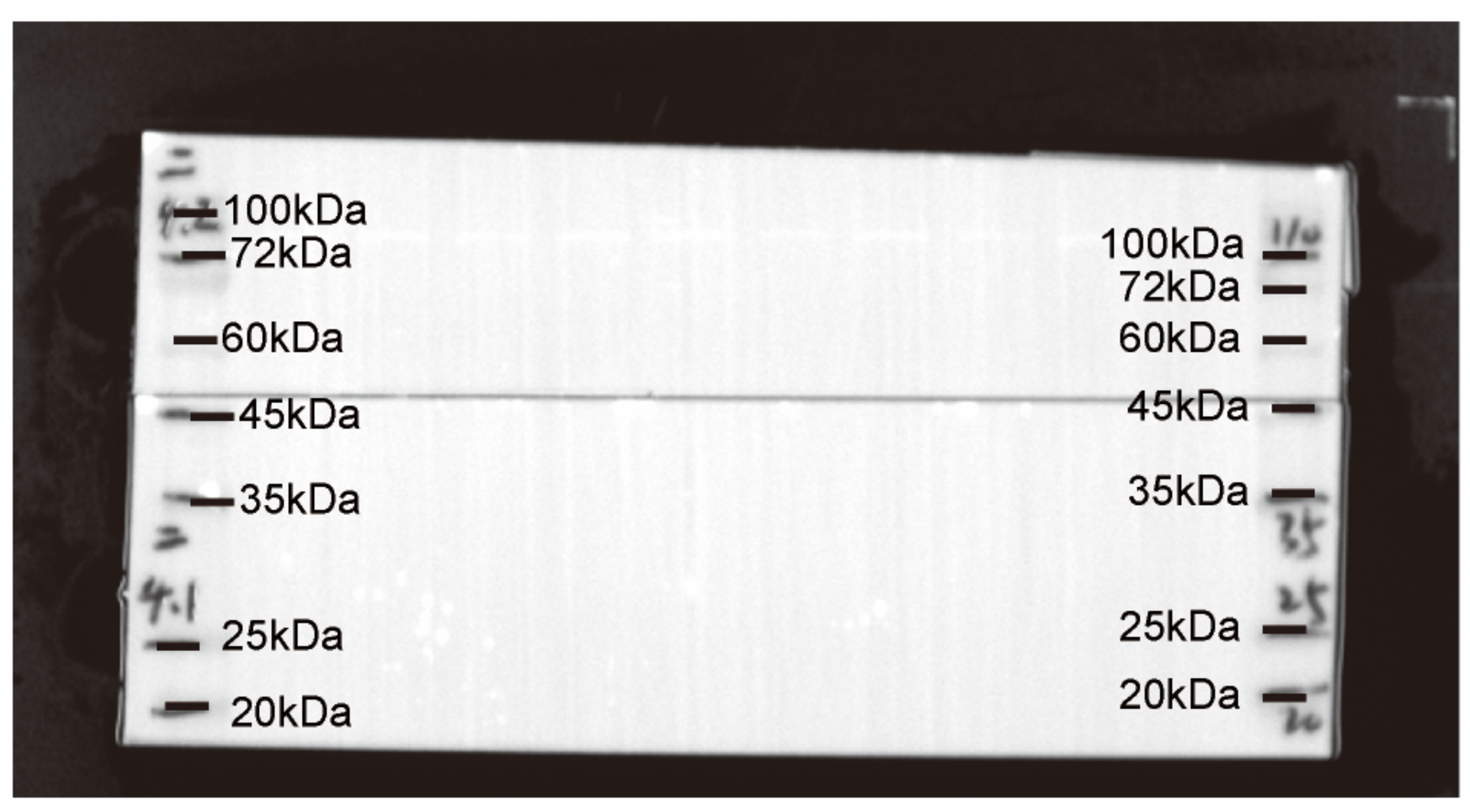

Figure	Merged	Stiched full image

Fig.5J

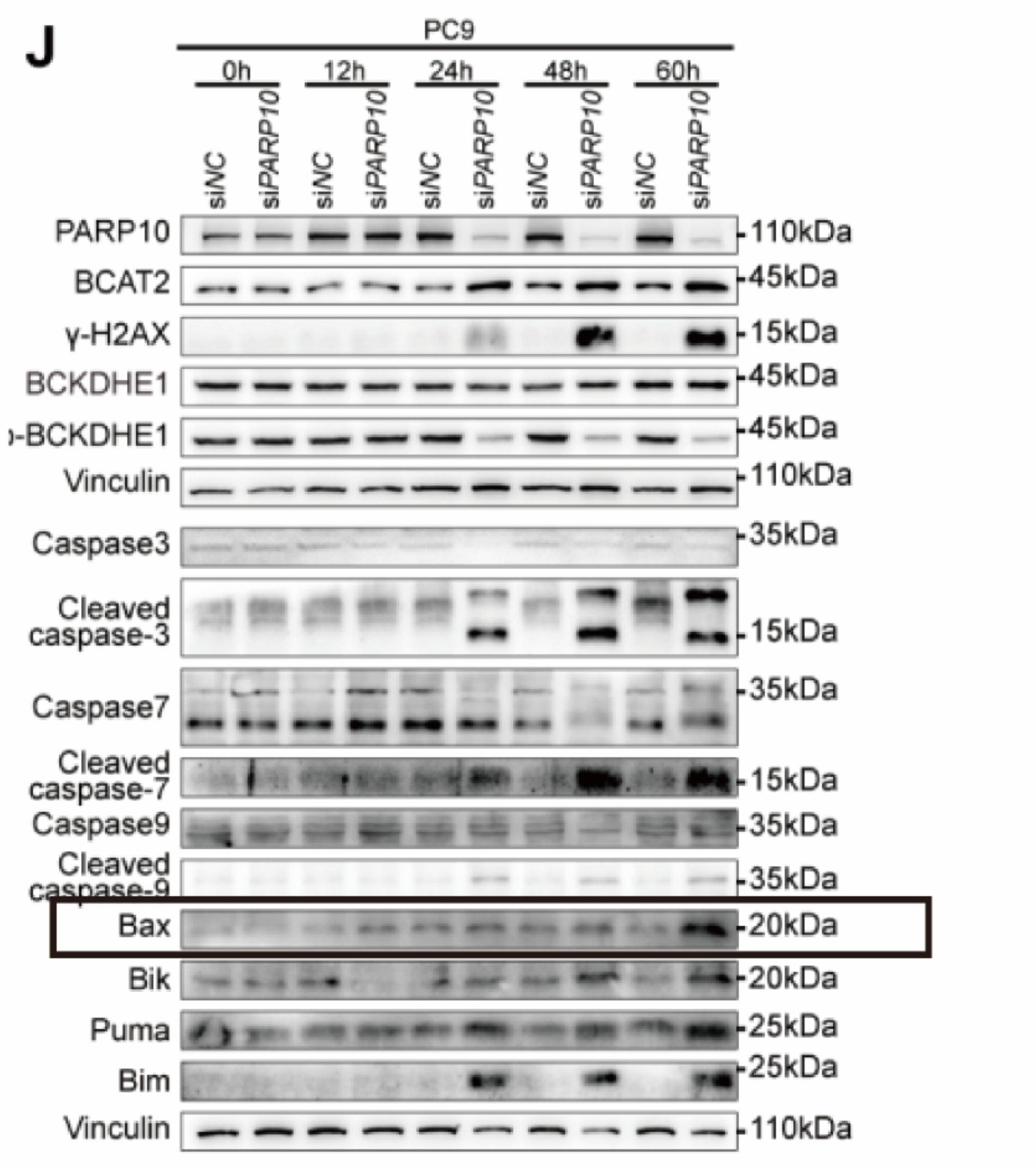

Bax

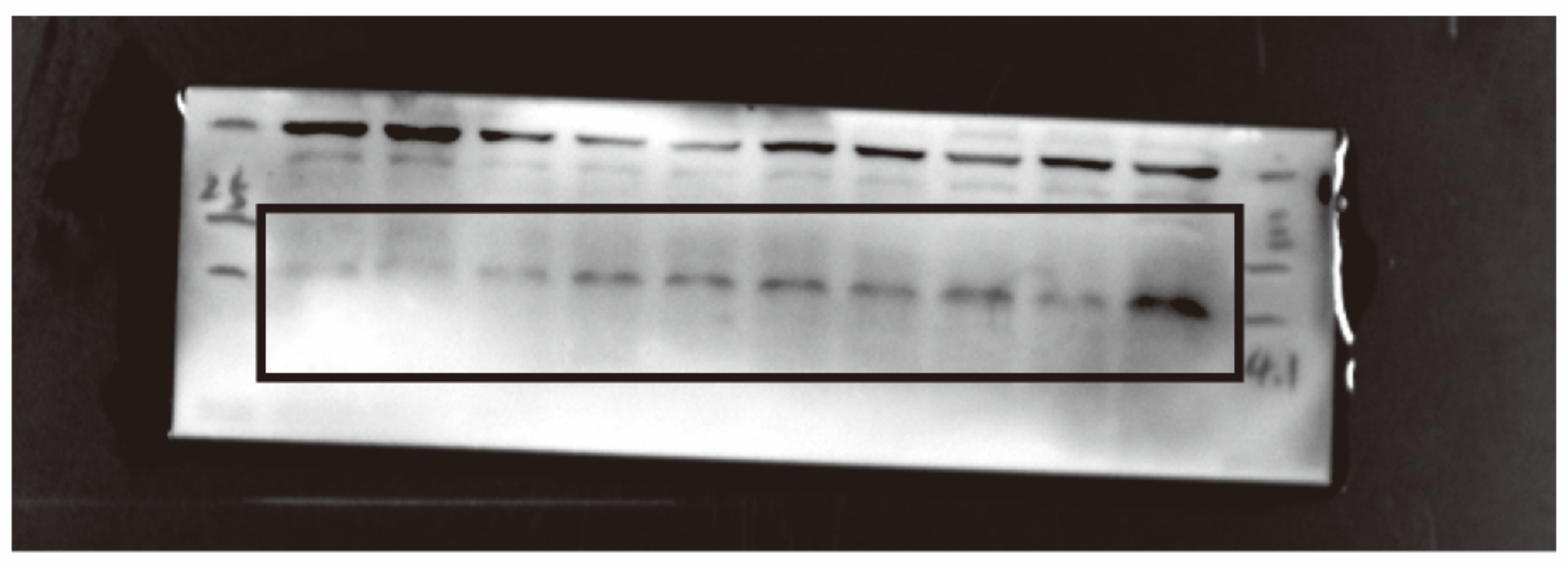

Vinculin

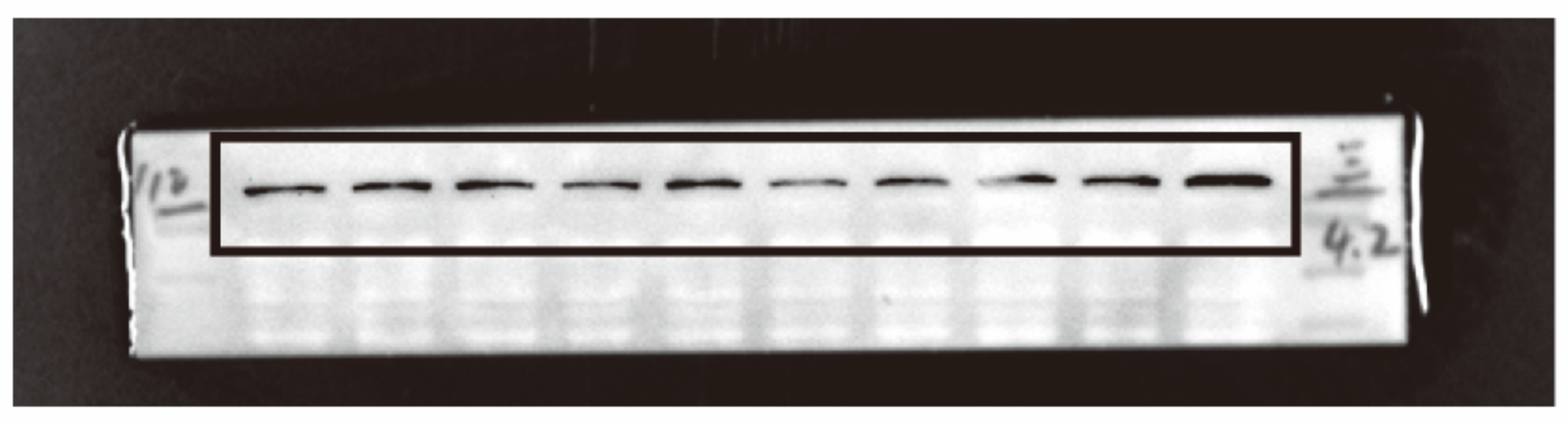

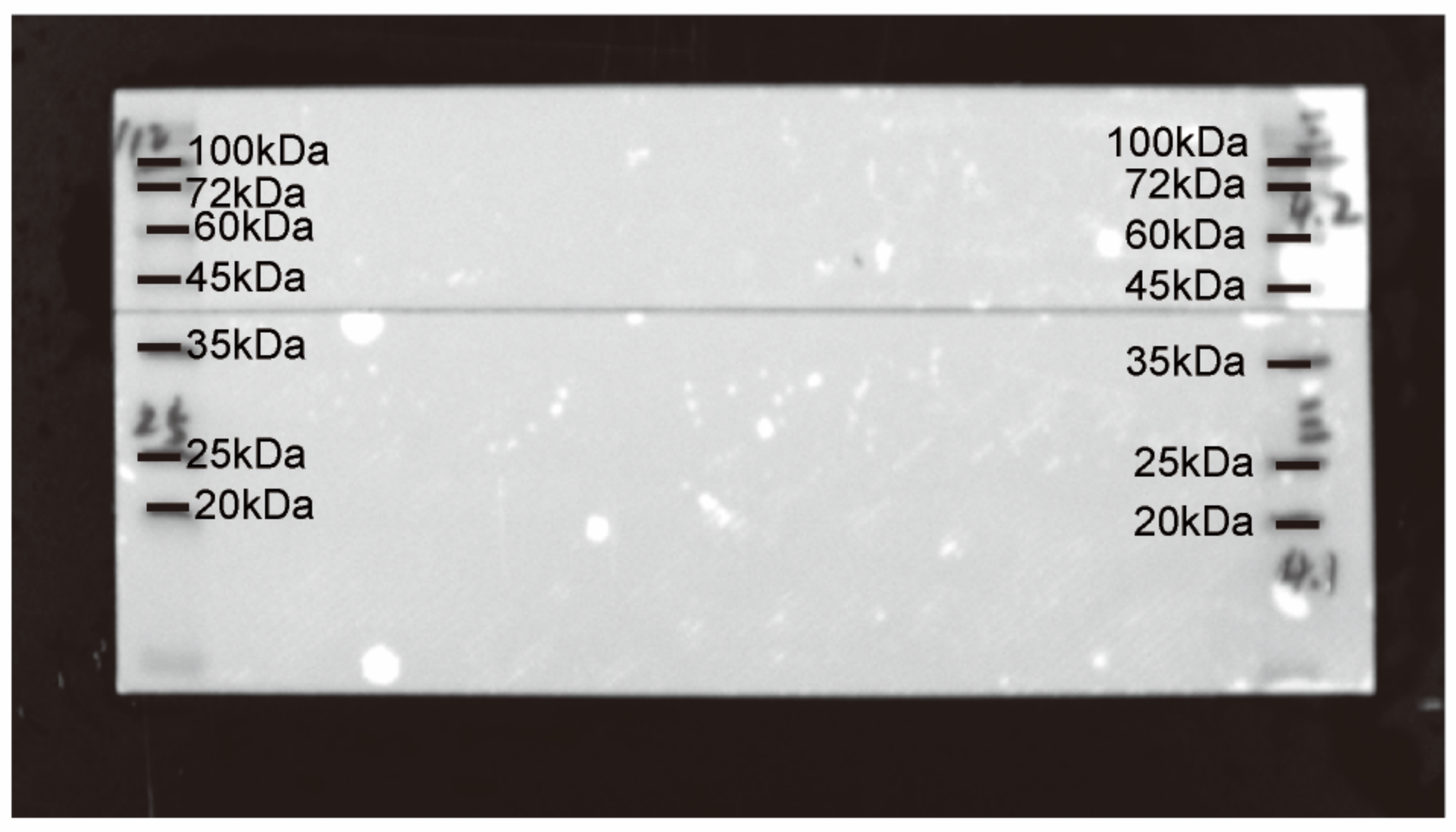

Fig.5J

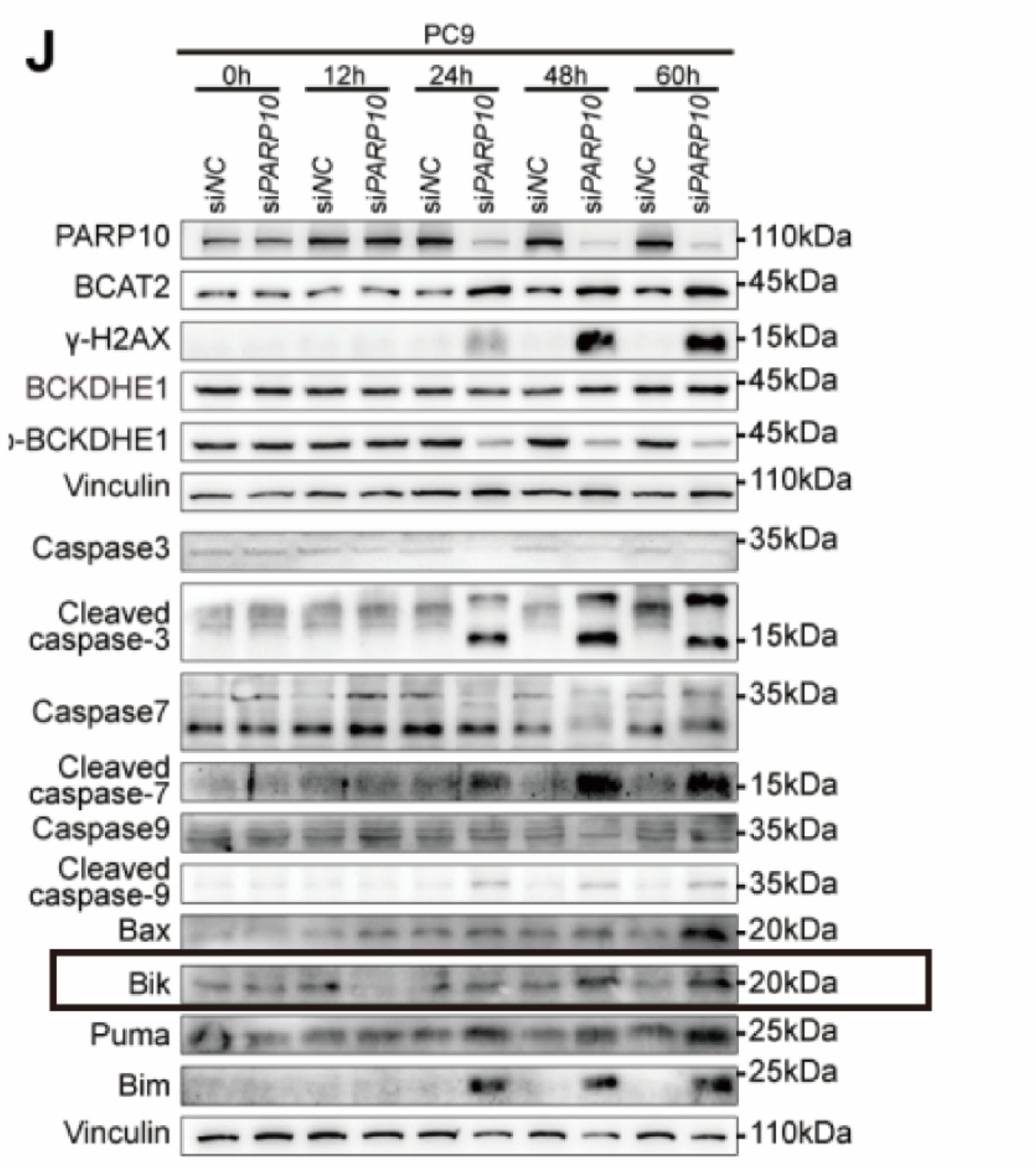

Bik

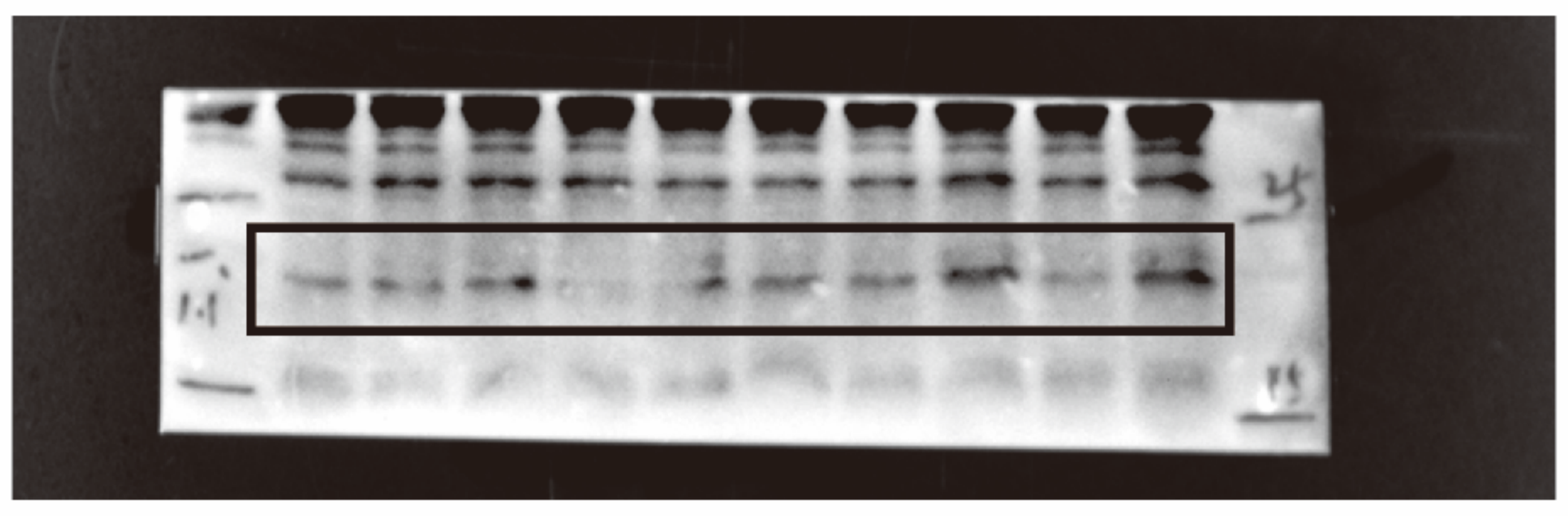

Vinculin

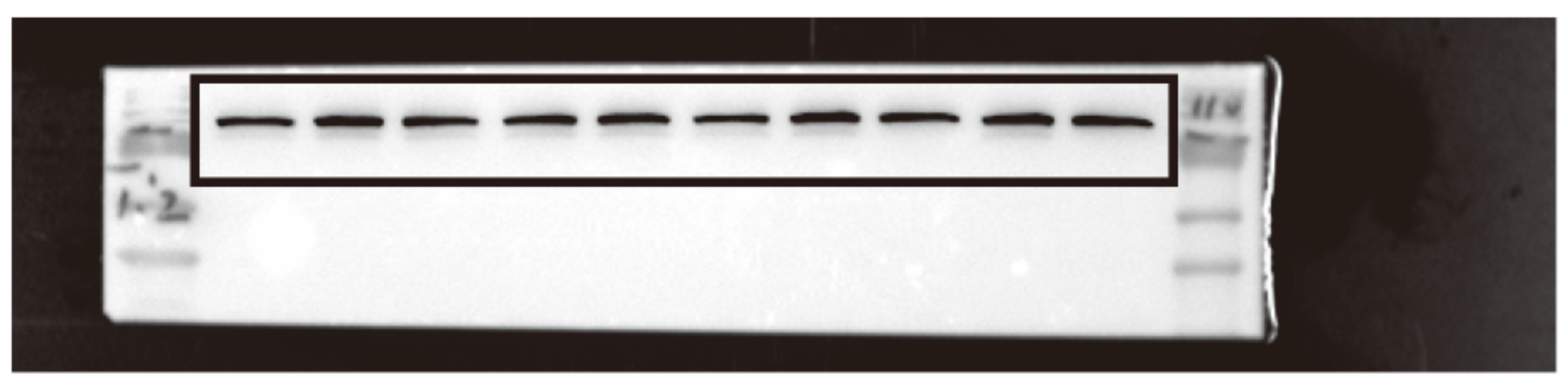

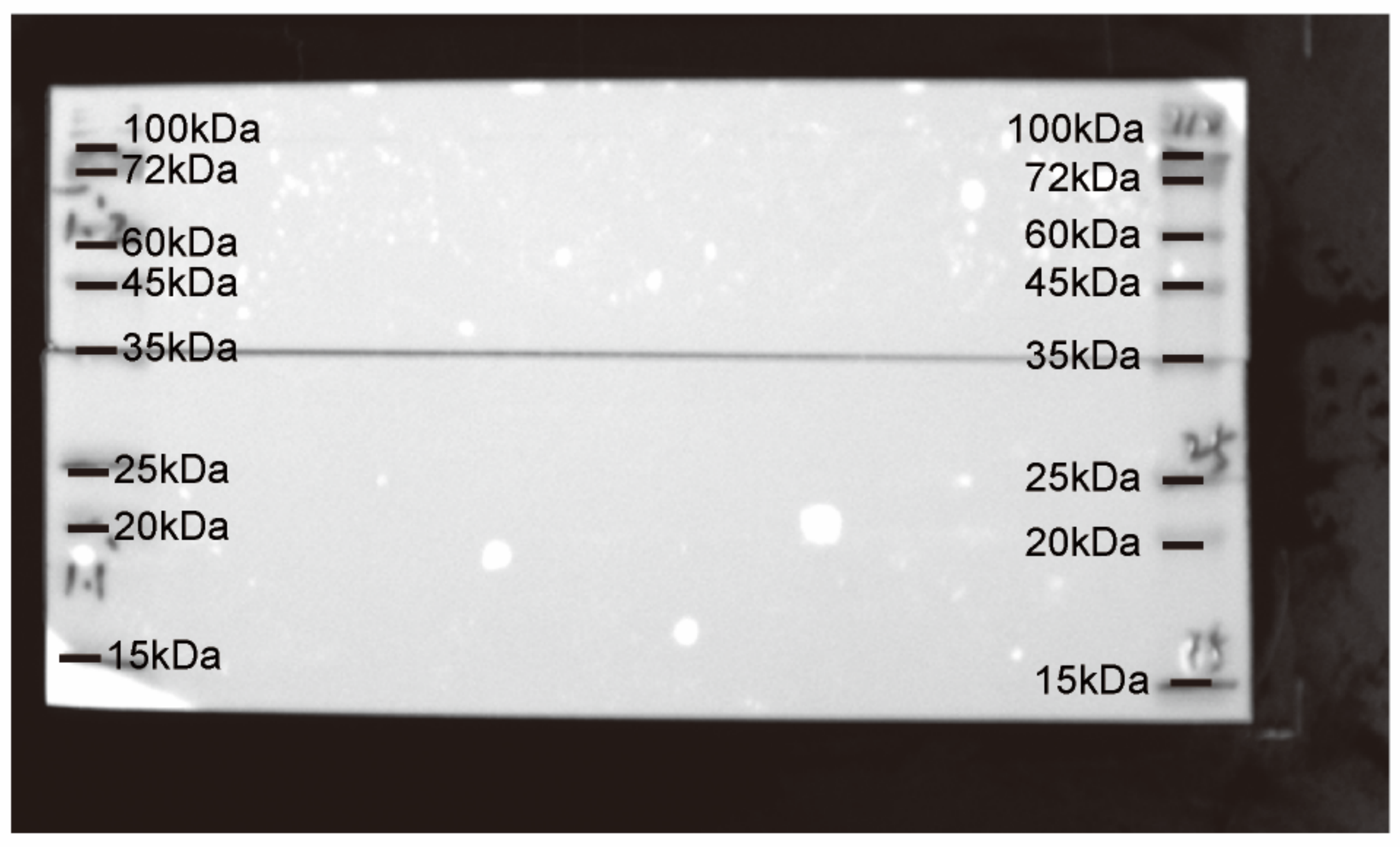

Fig.5J

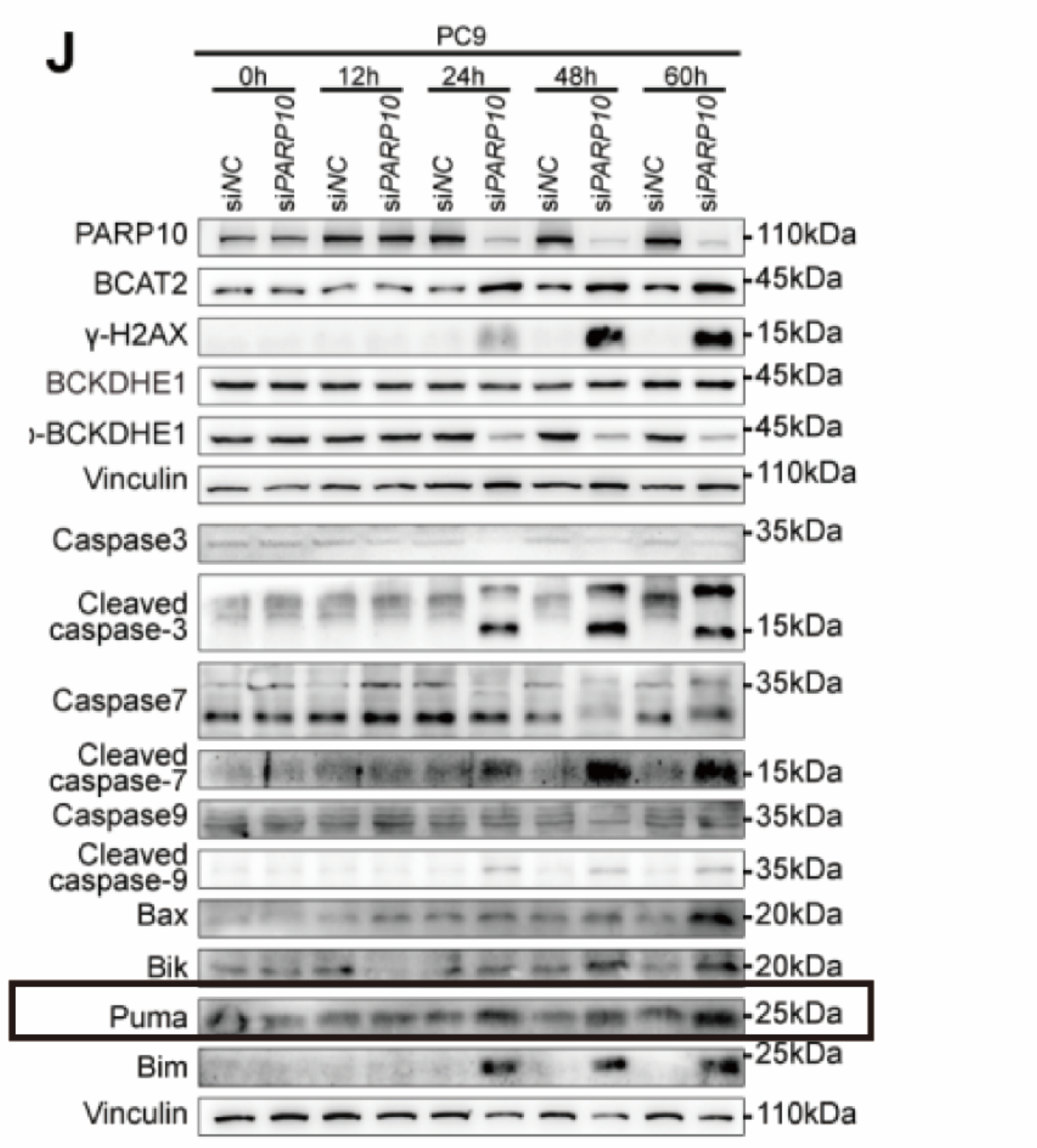

Puma

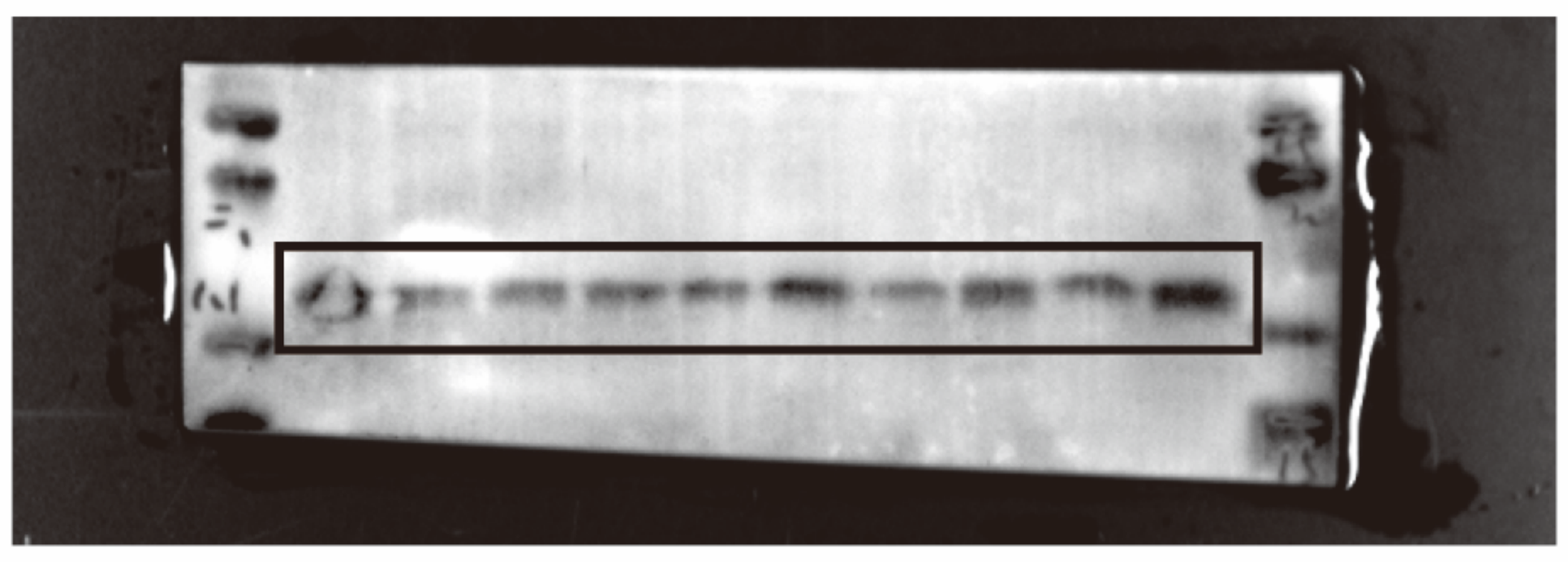

β-actin

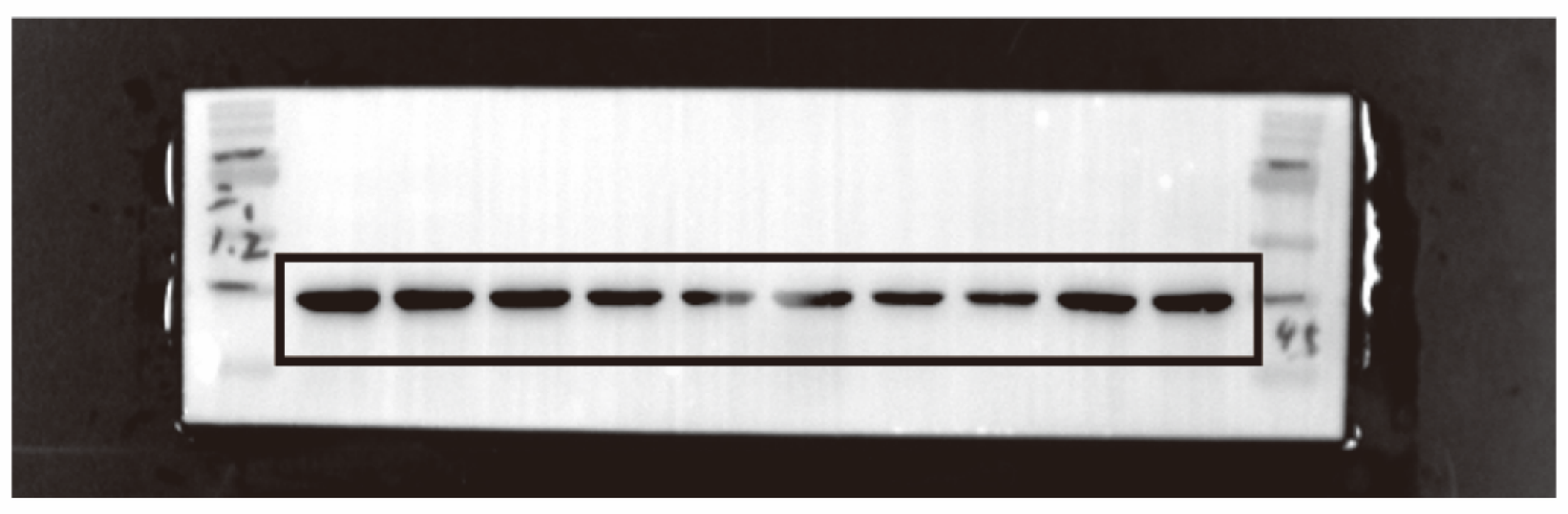

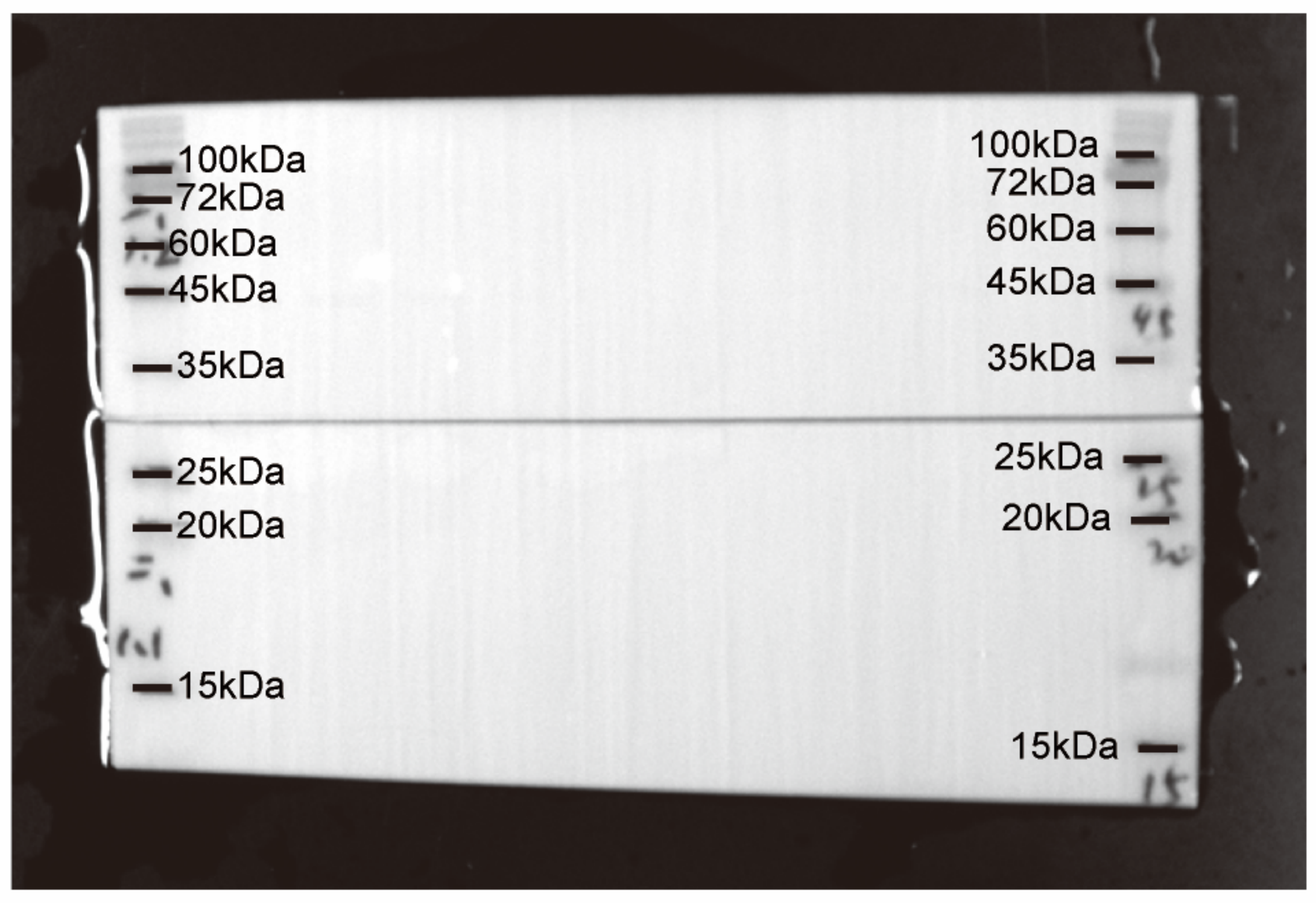

Fig.5J

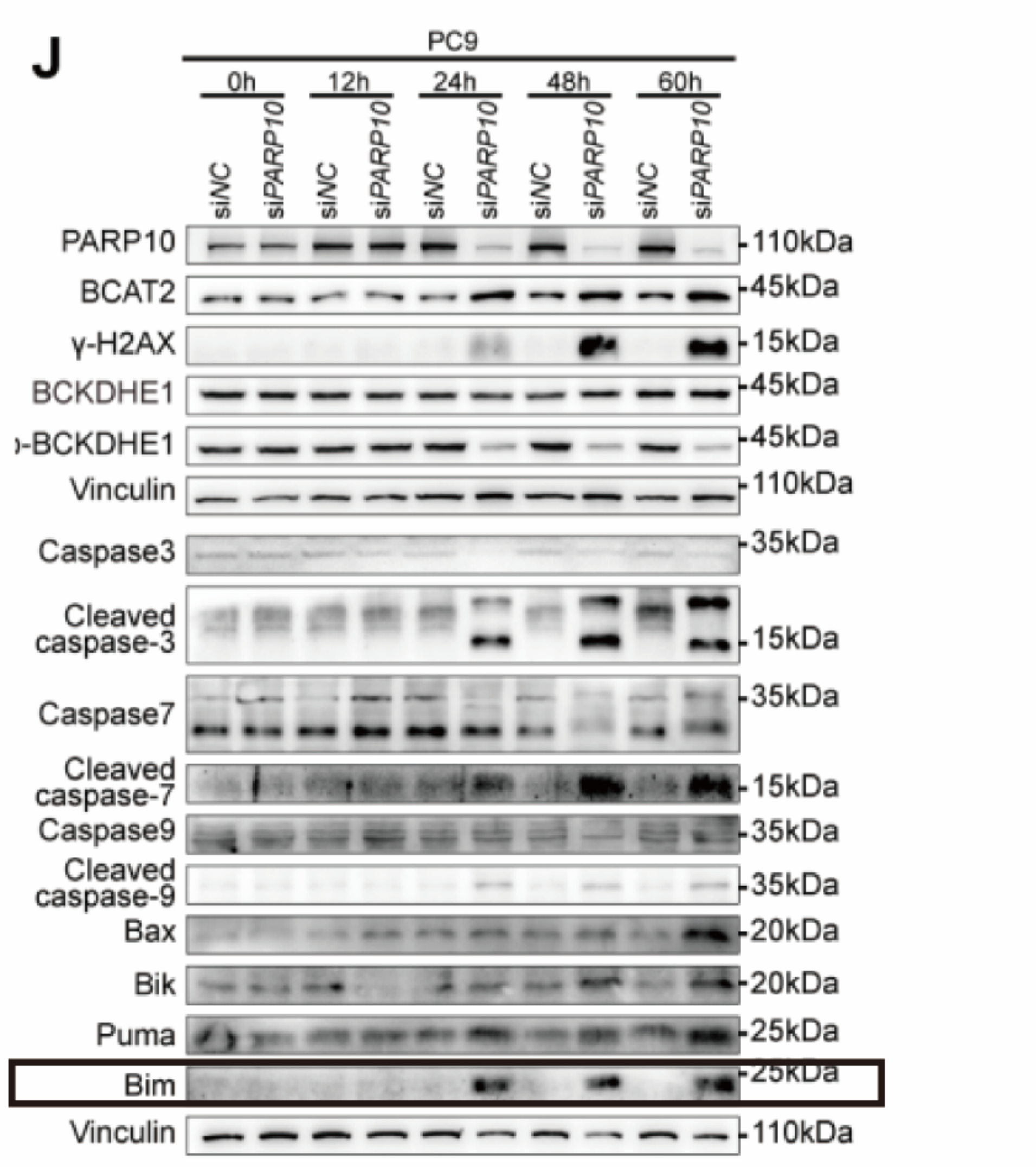

Bim

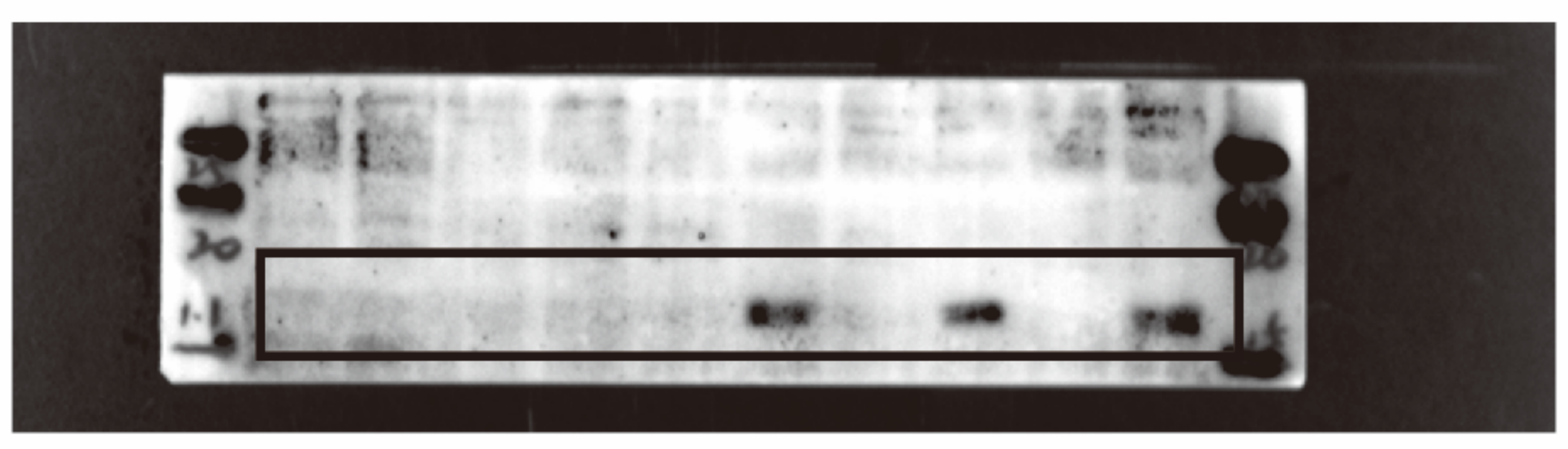

β-actin

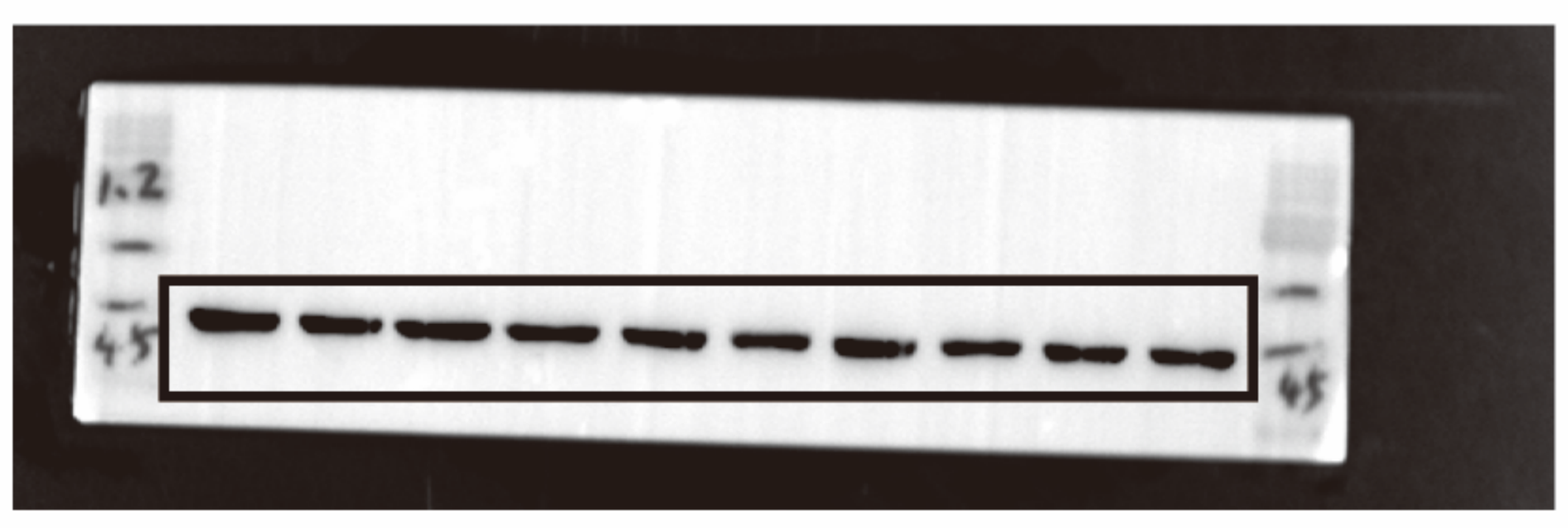

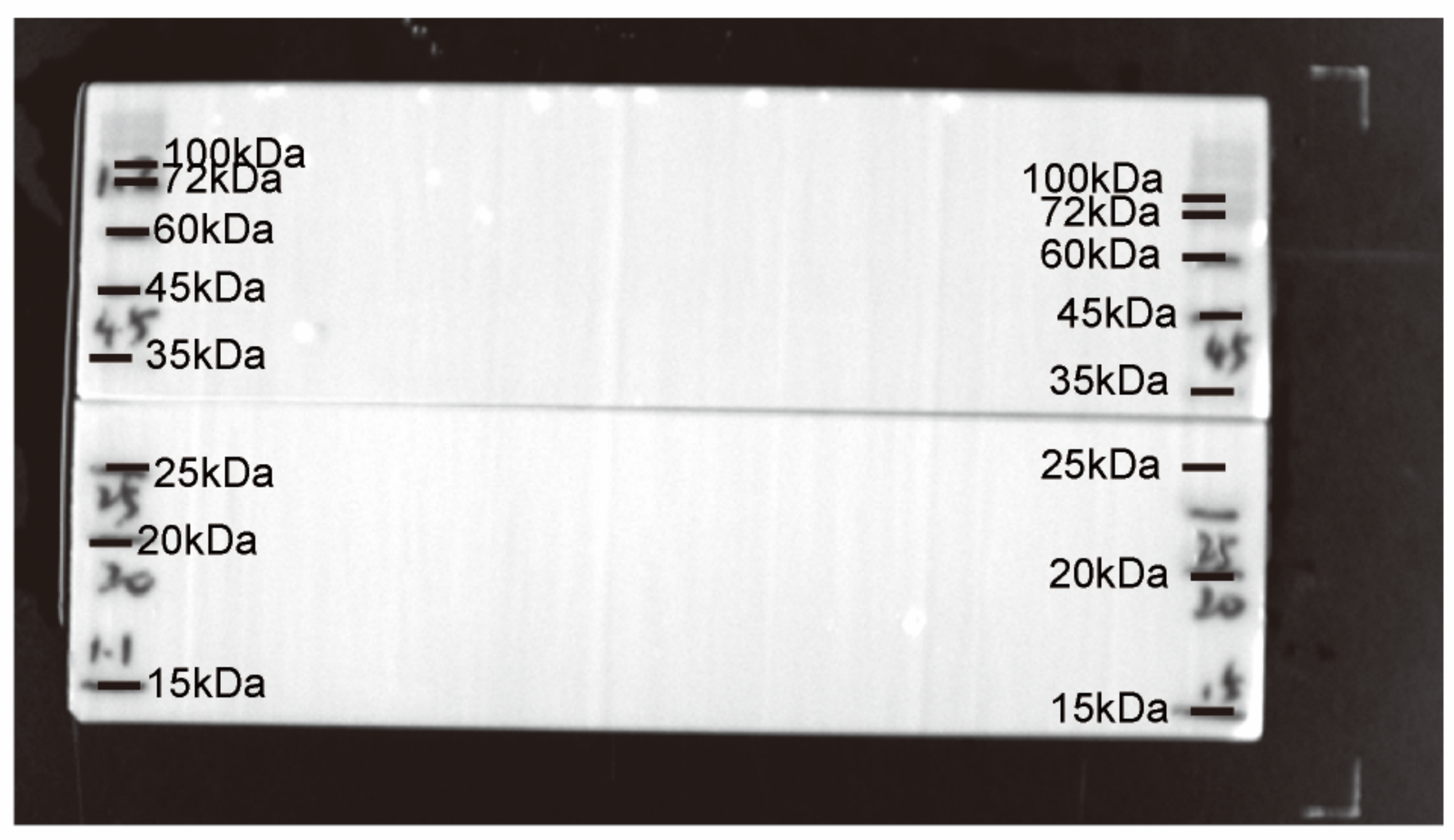

Figure	Merged	Stiched full image

Fig.6H

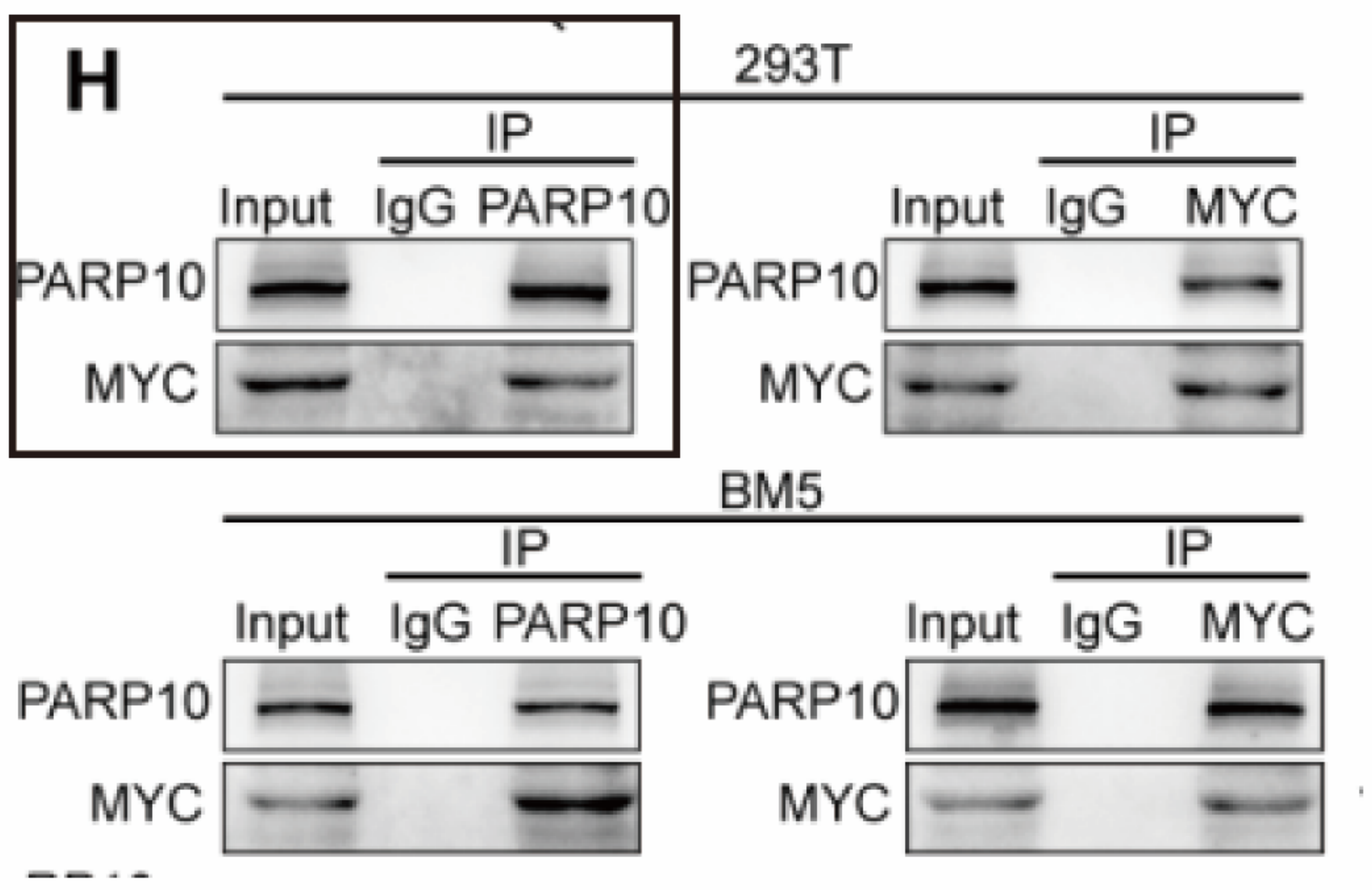

PARP10

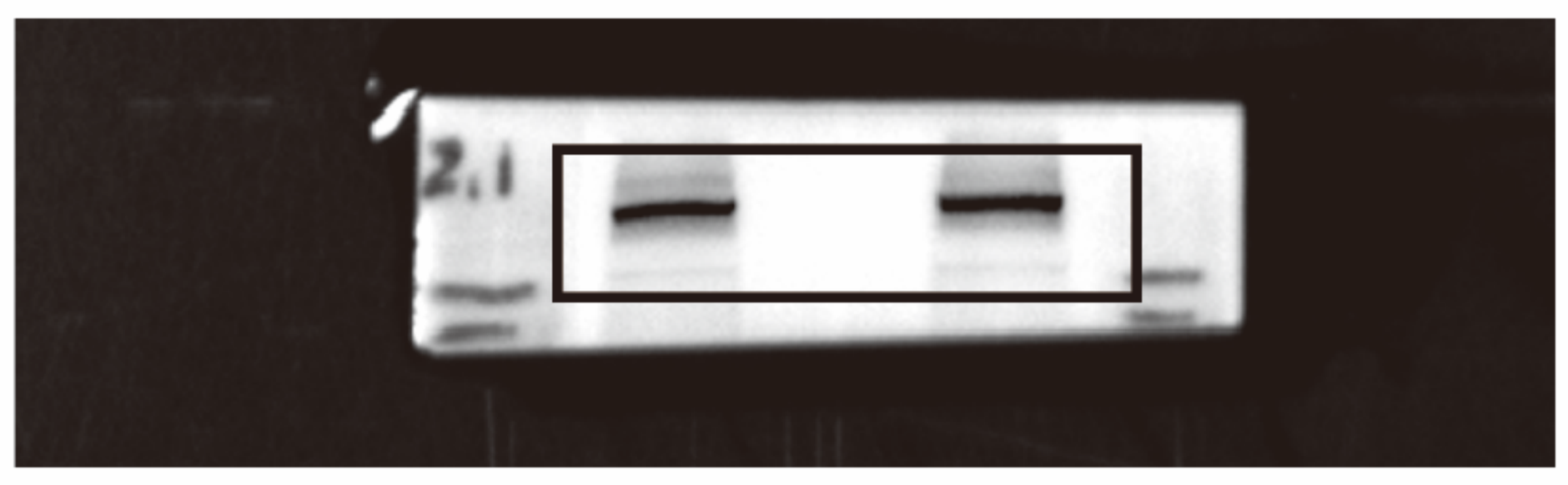

MYC

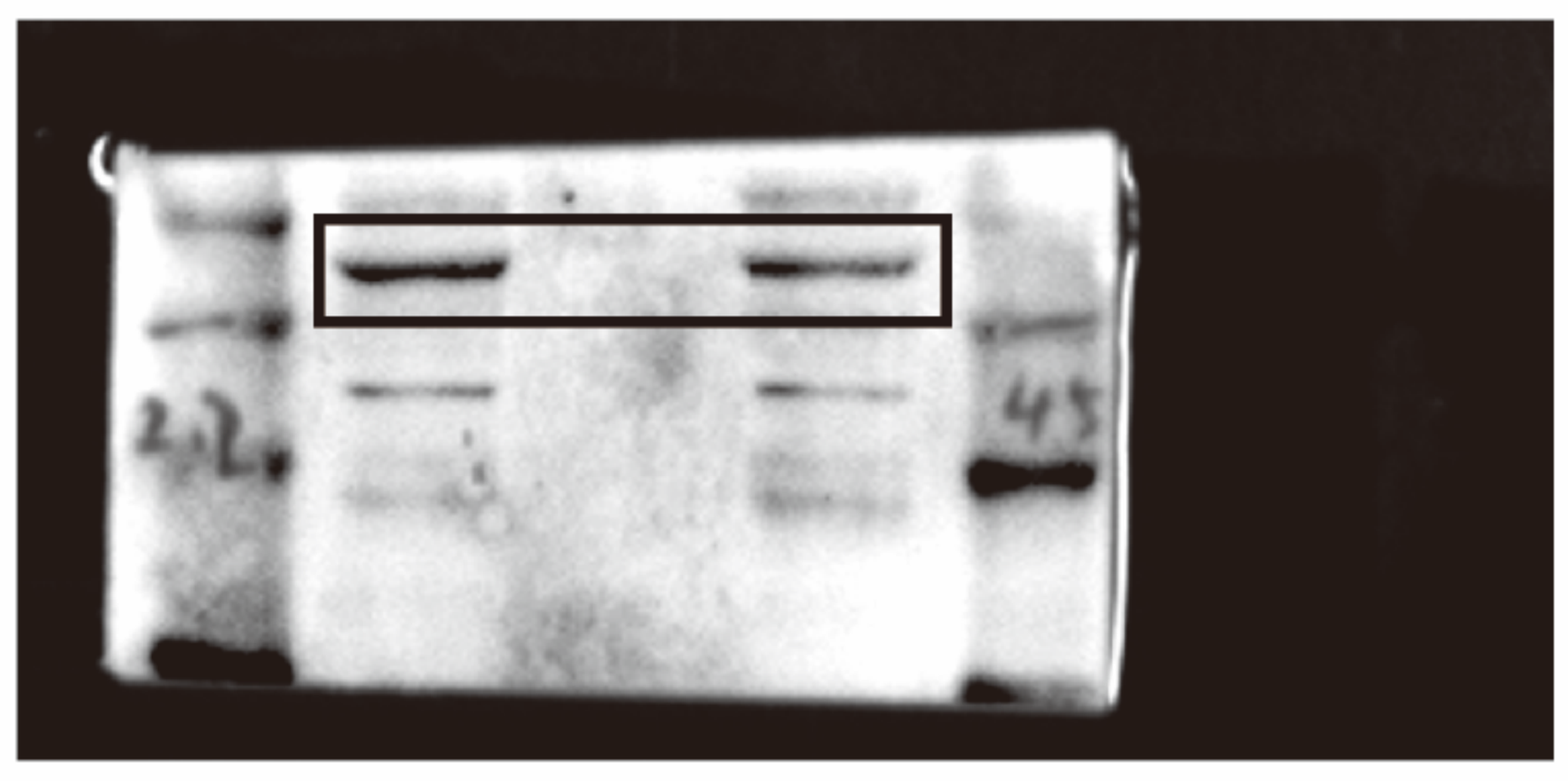

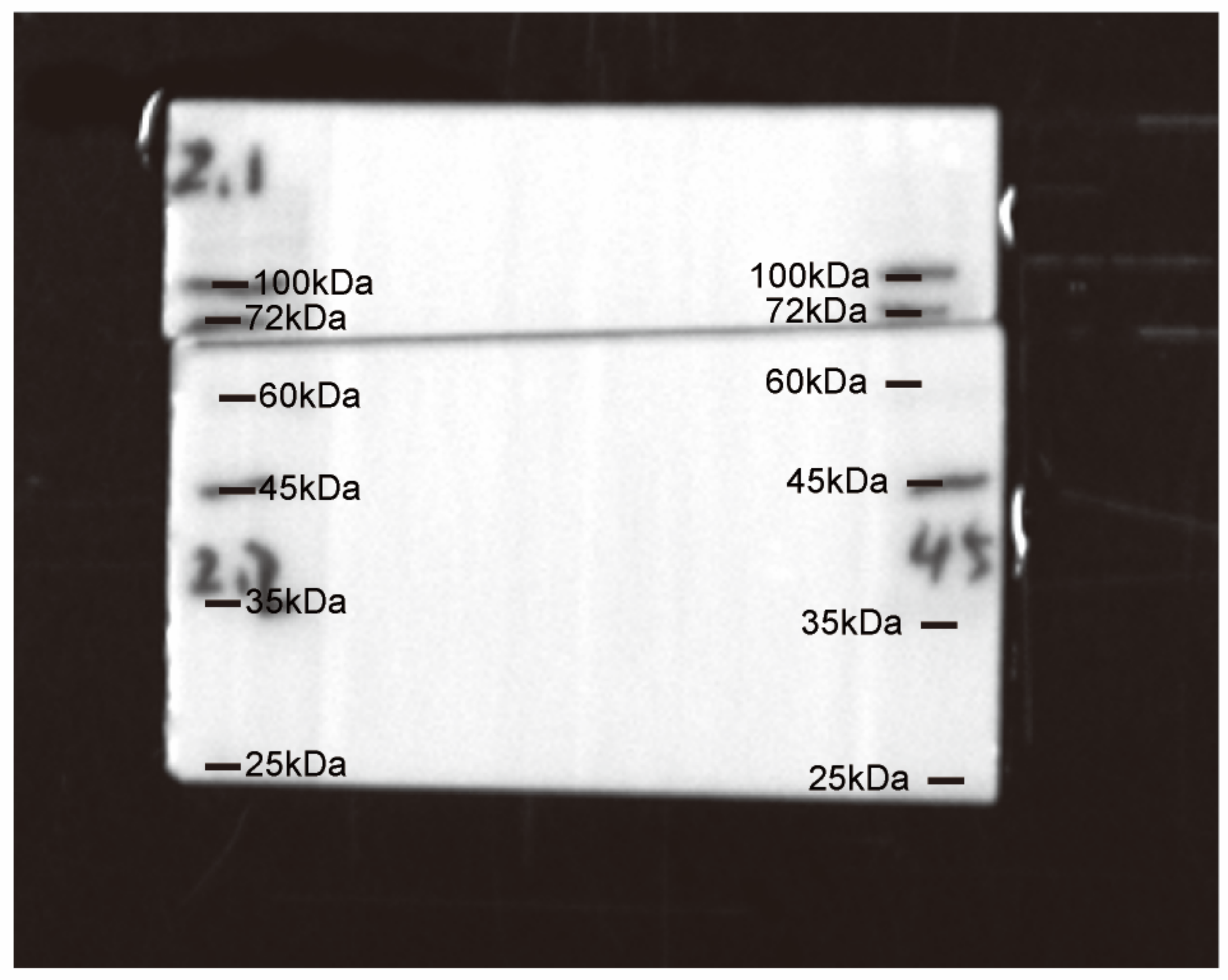

Fig.6H

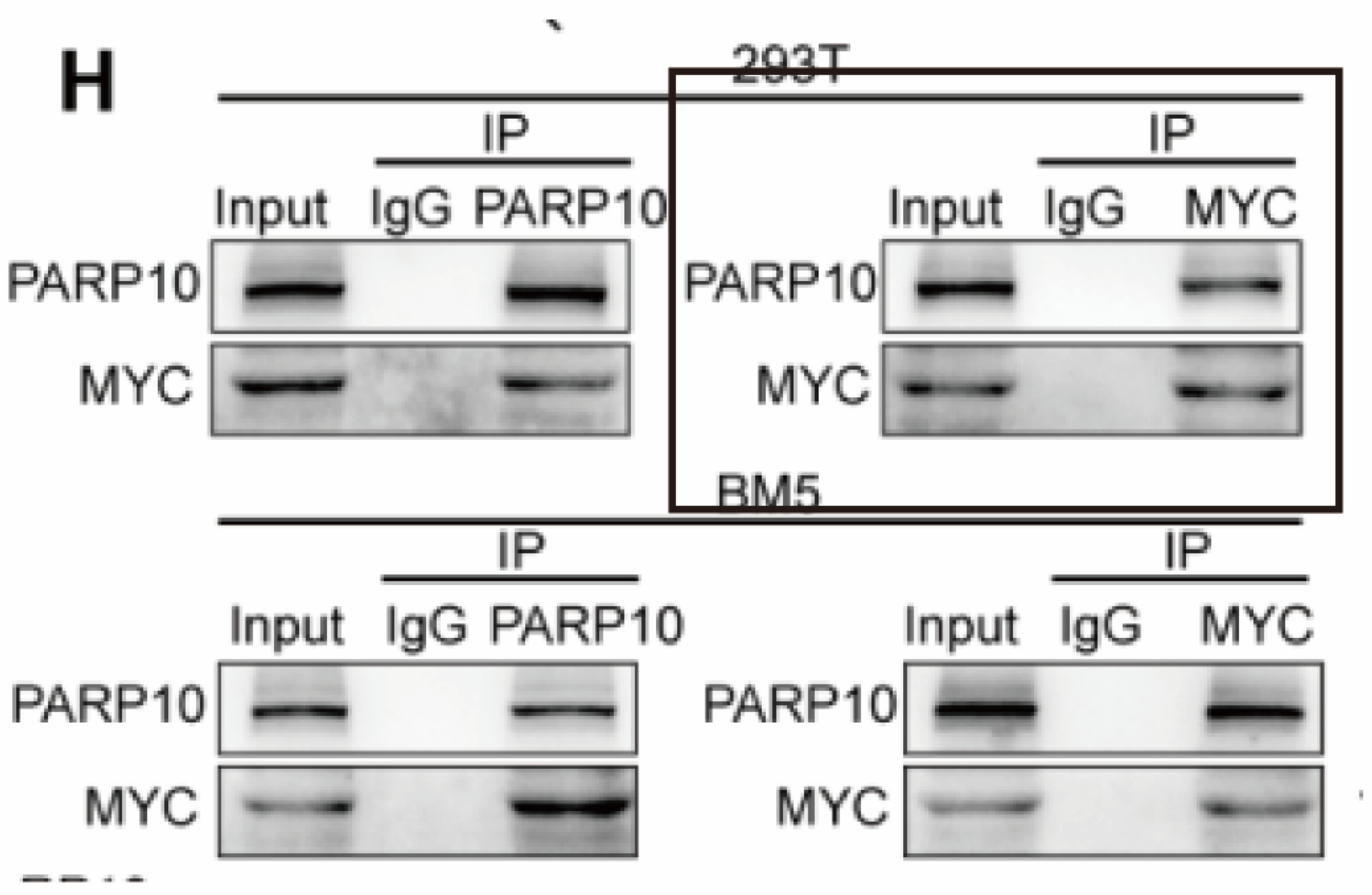

PARP10

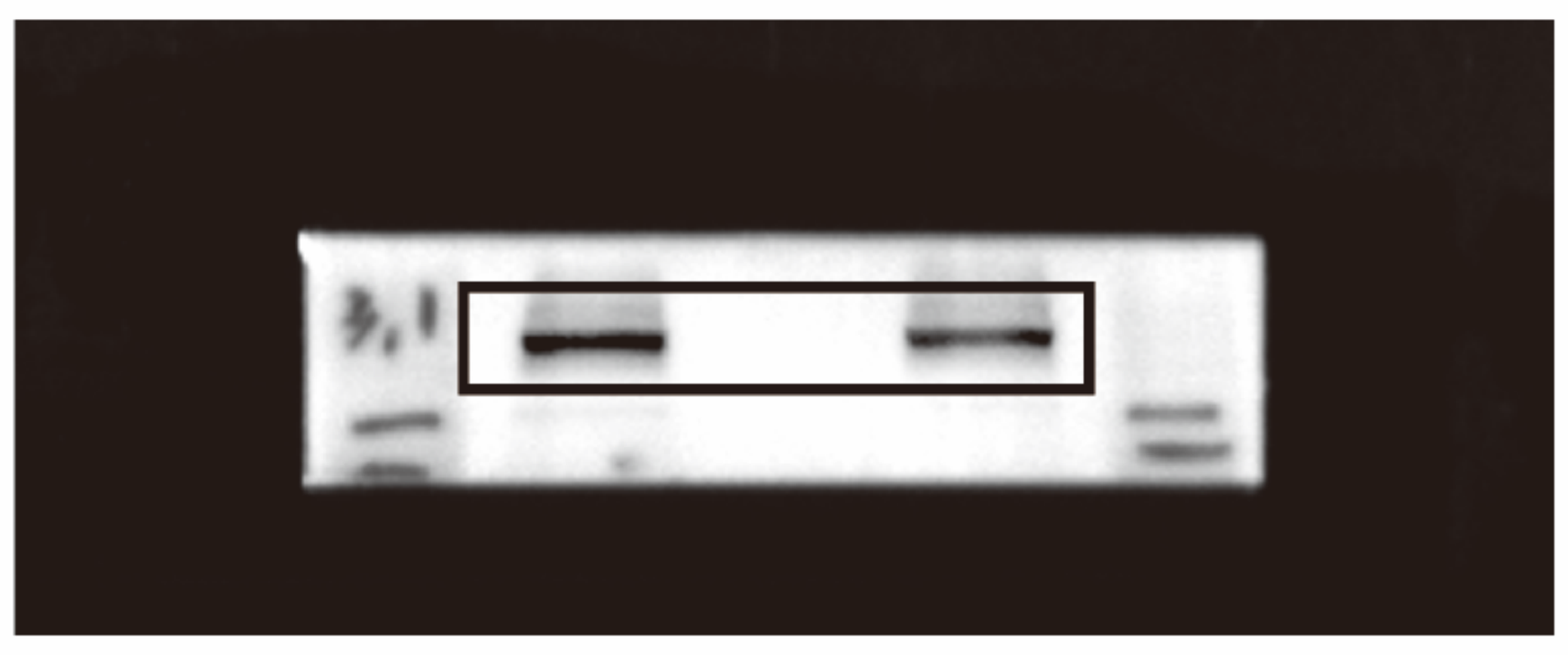

MYC

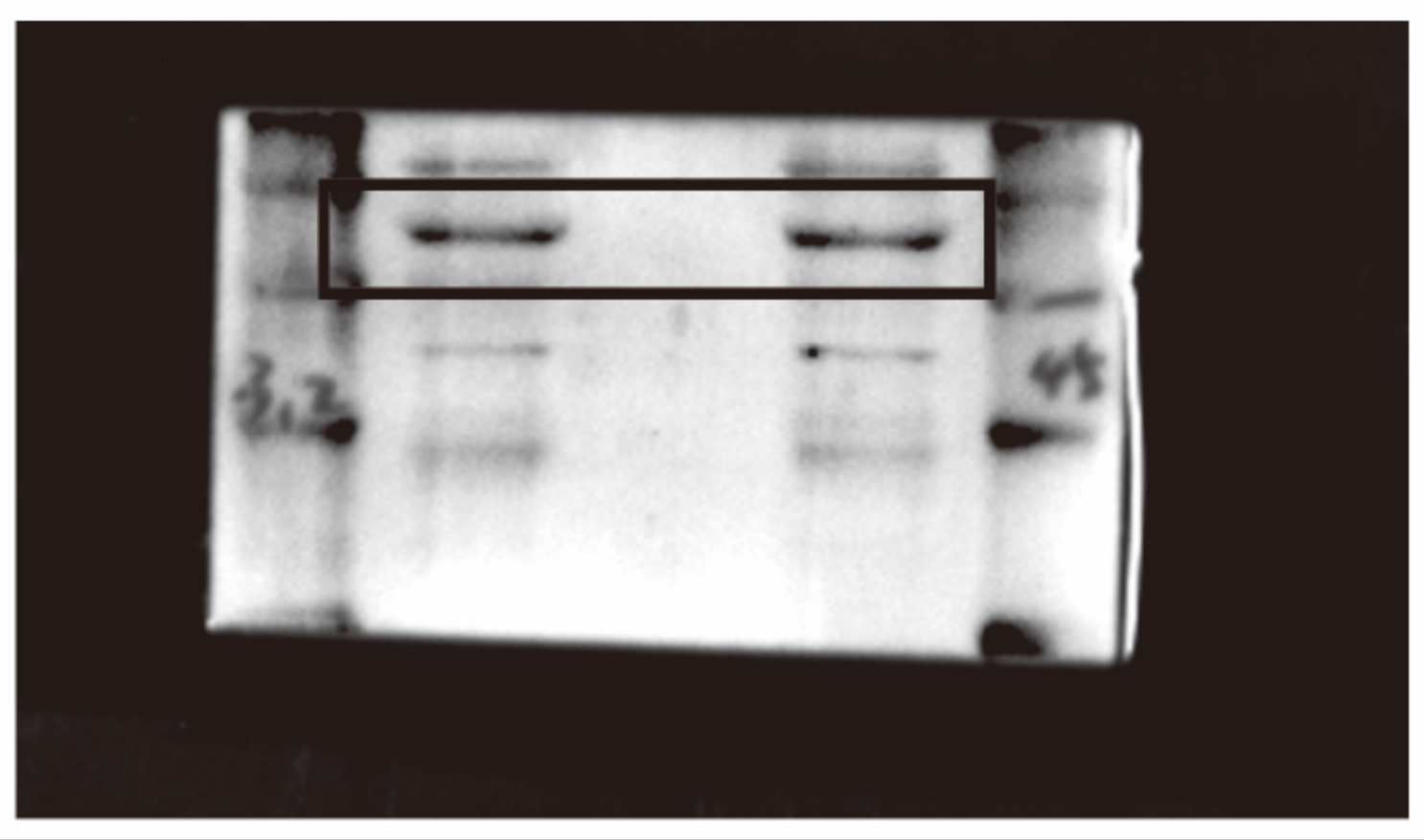

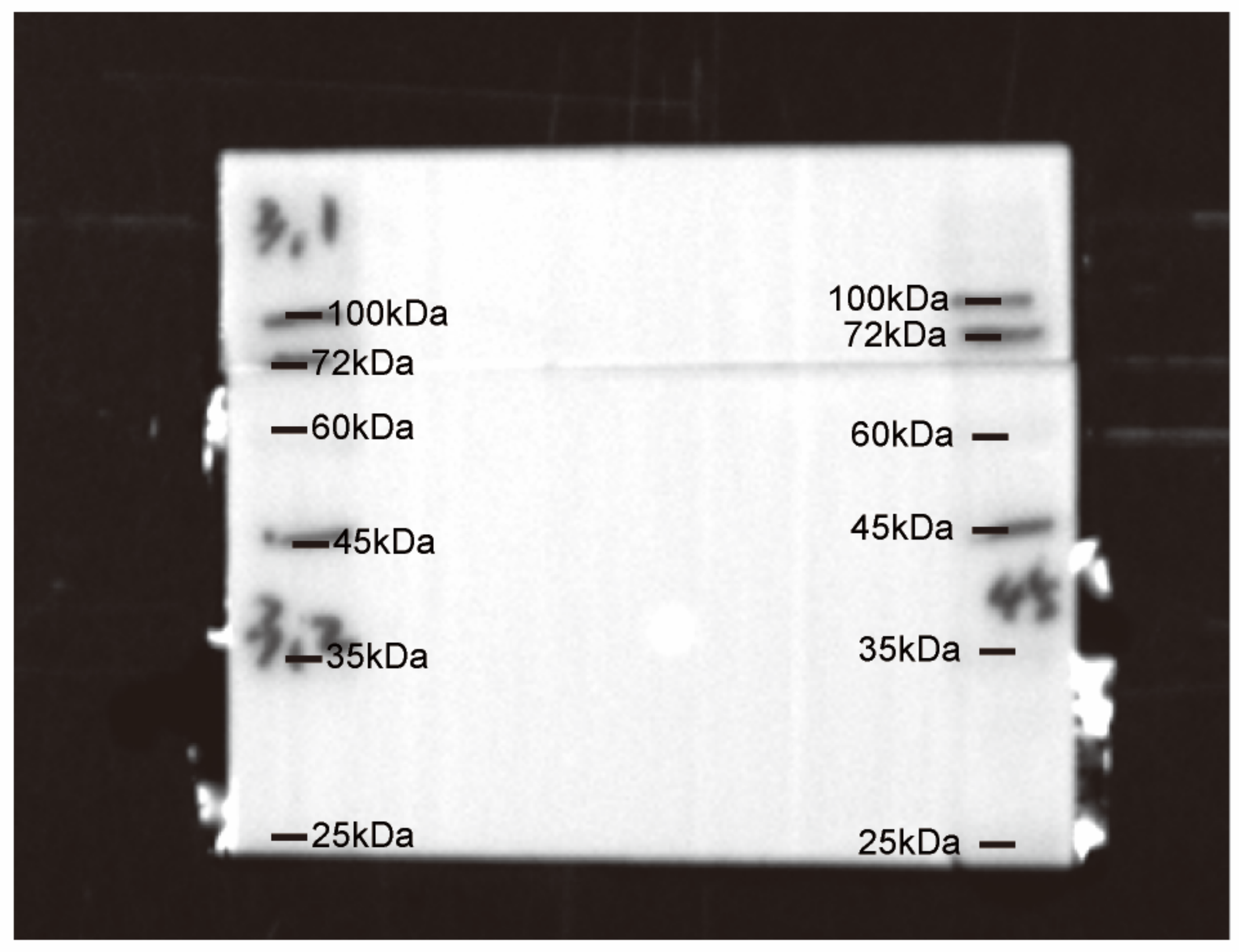

Fig.6I

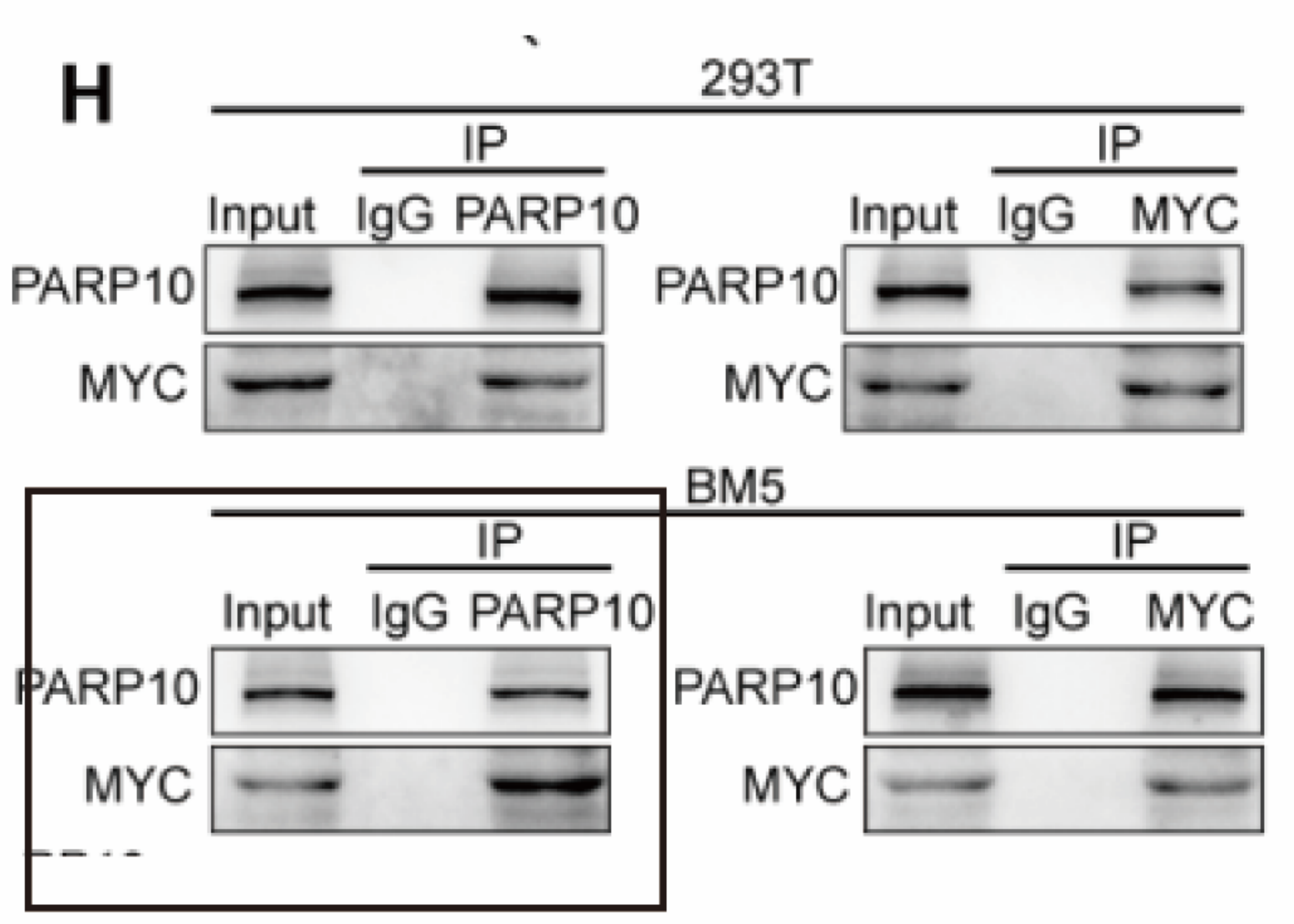

PARP10

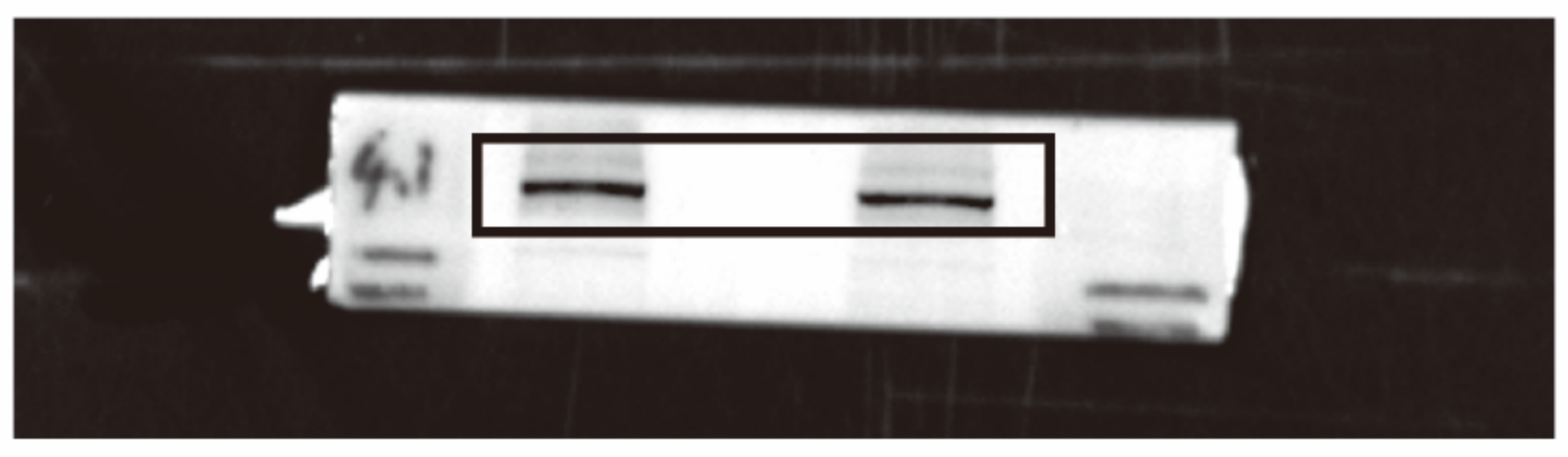

MYC

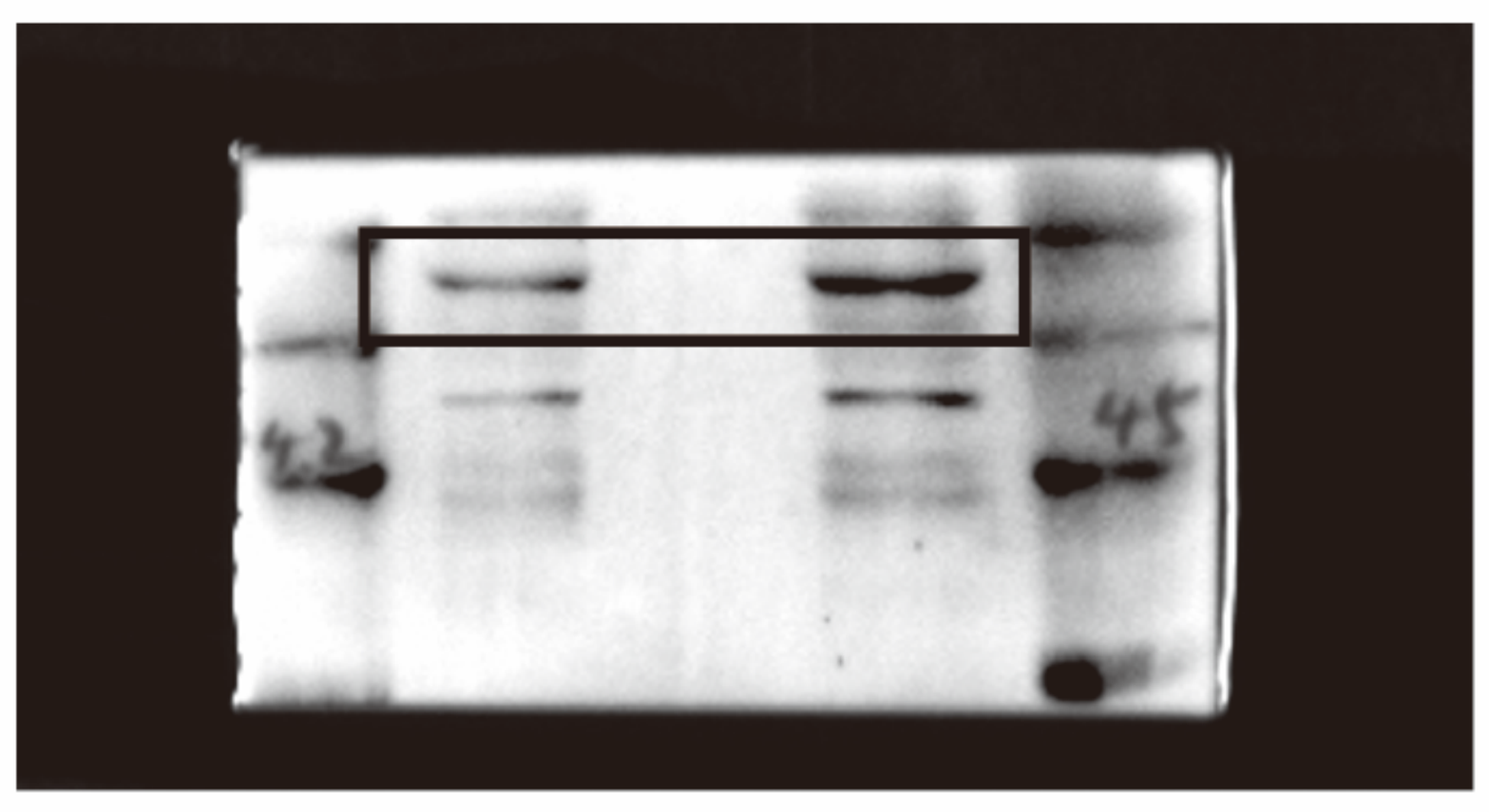

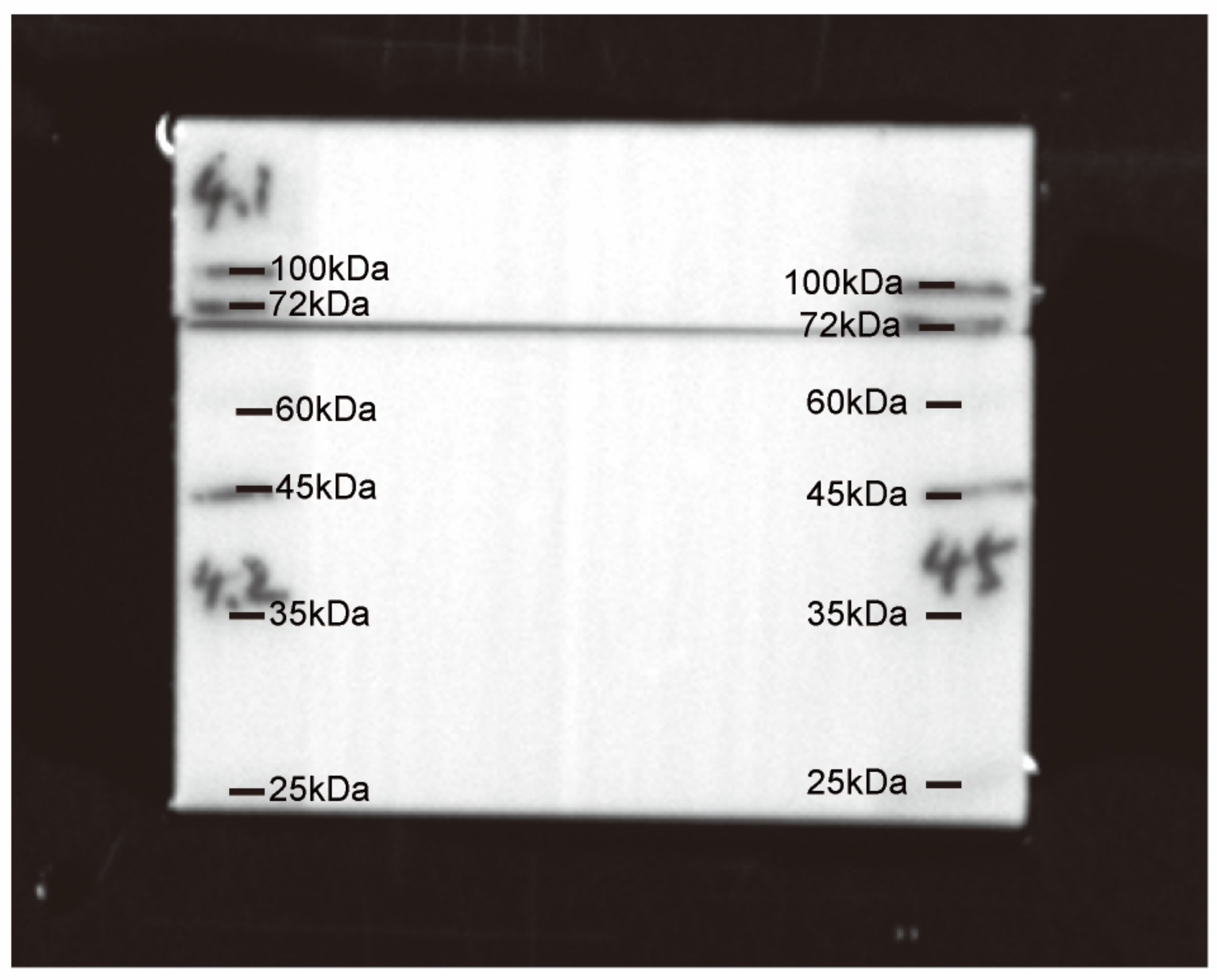

Fig.6I

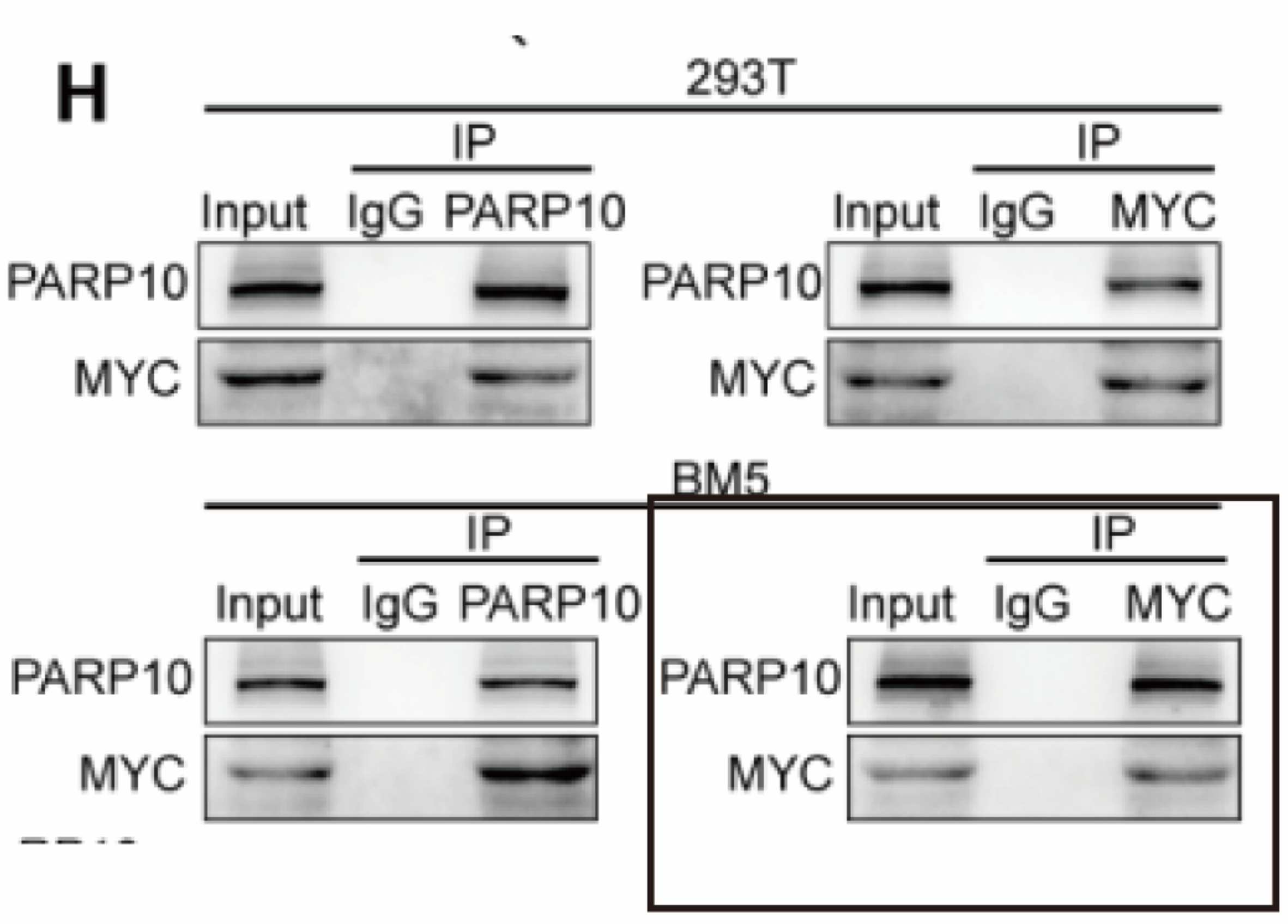

PARP10

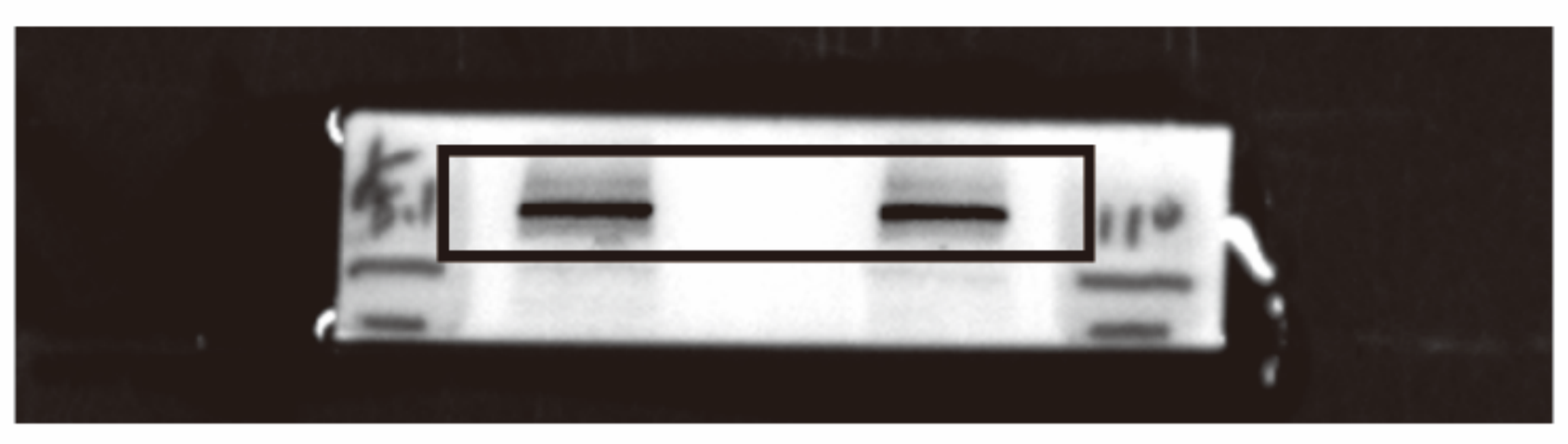

MYC

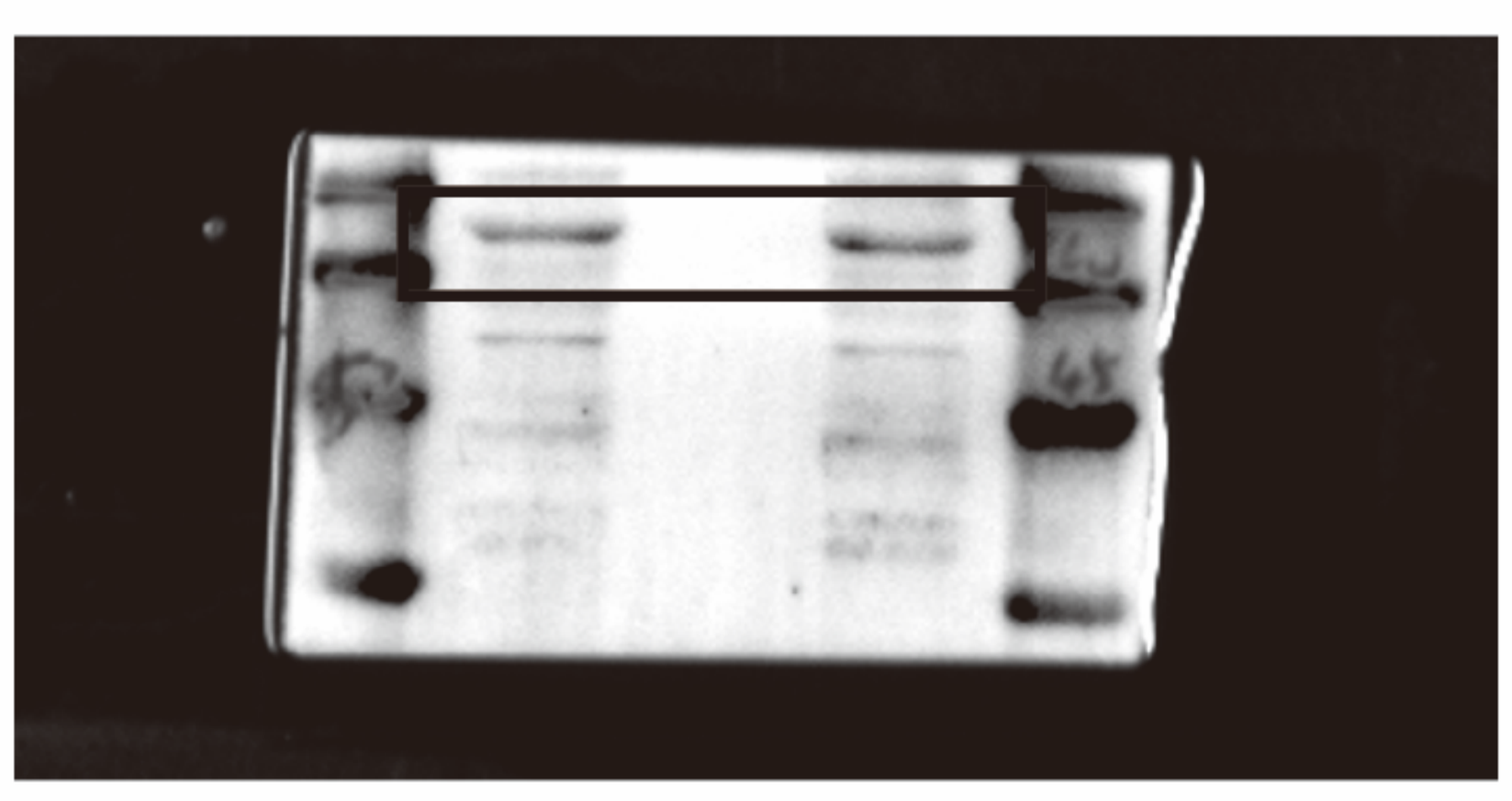

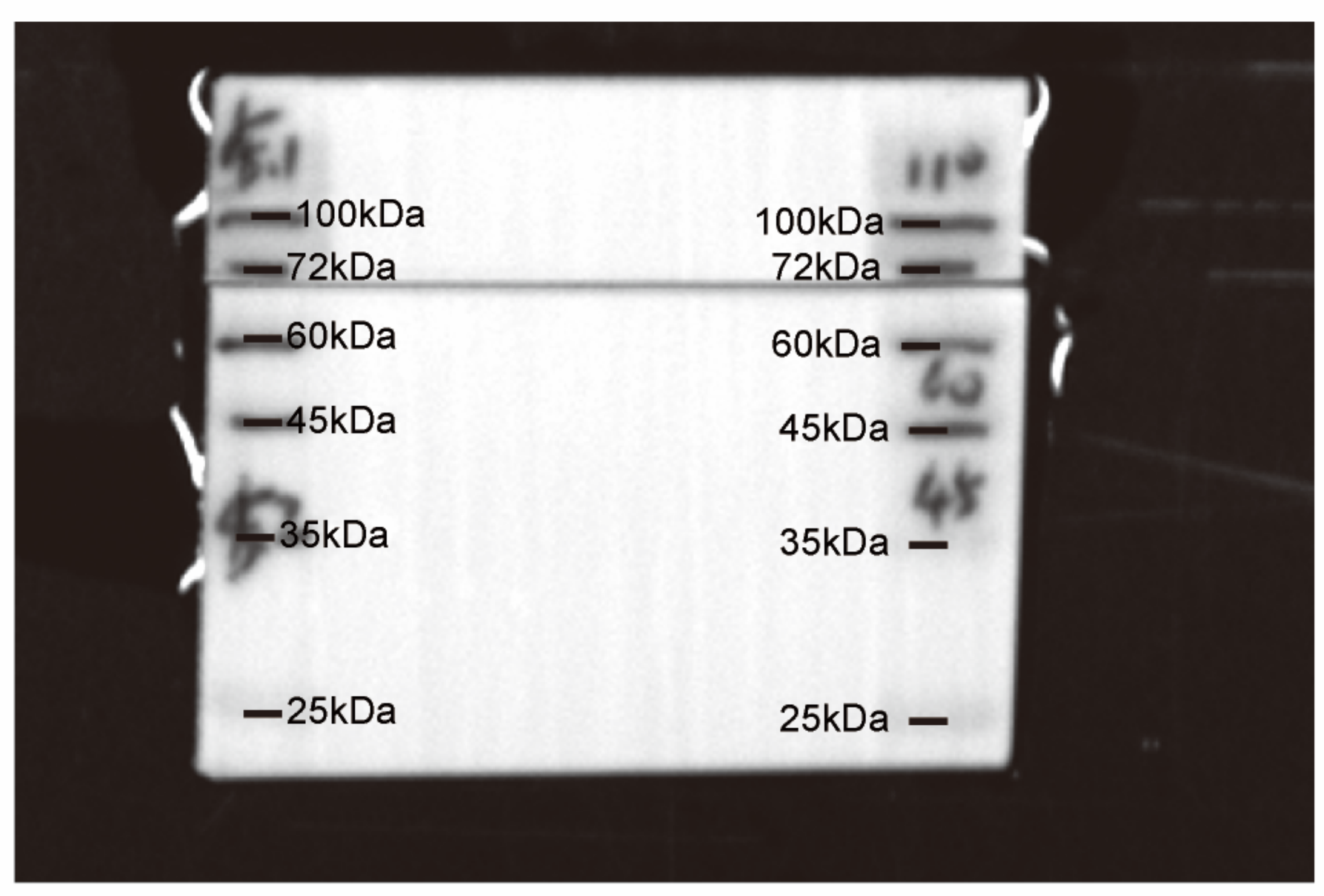

Figure	Merged	Stiched full image

Fig.6I

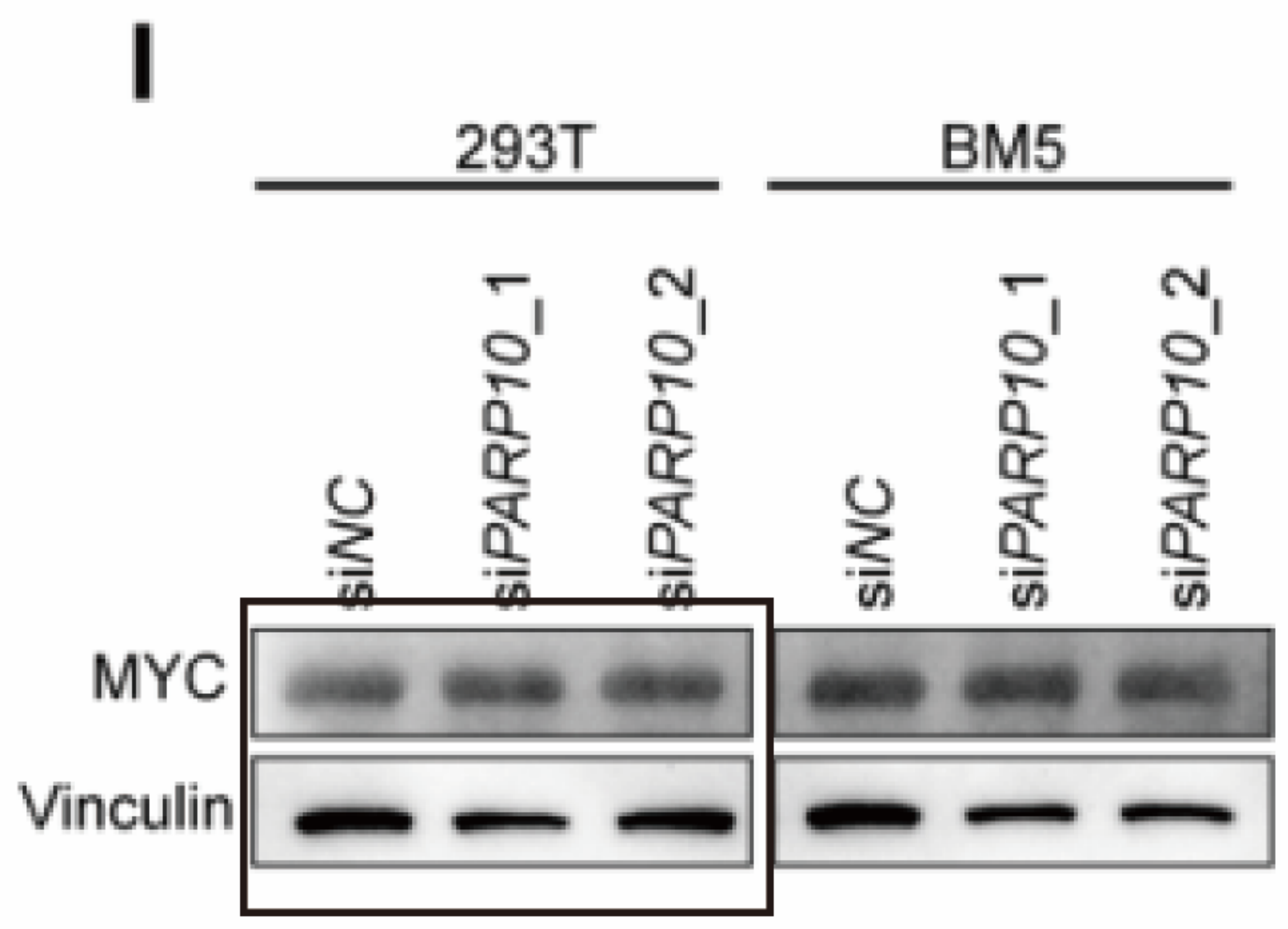

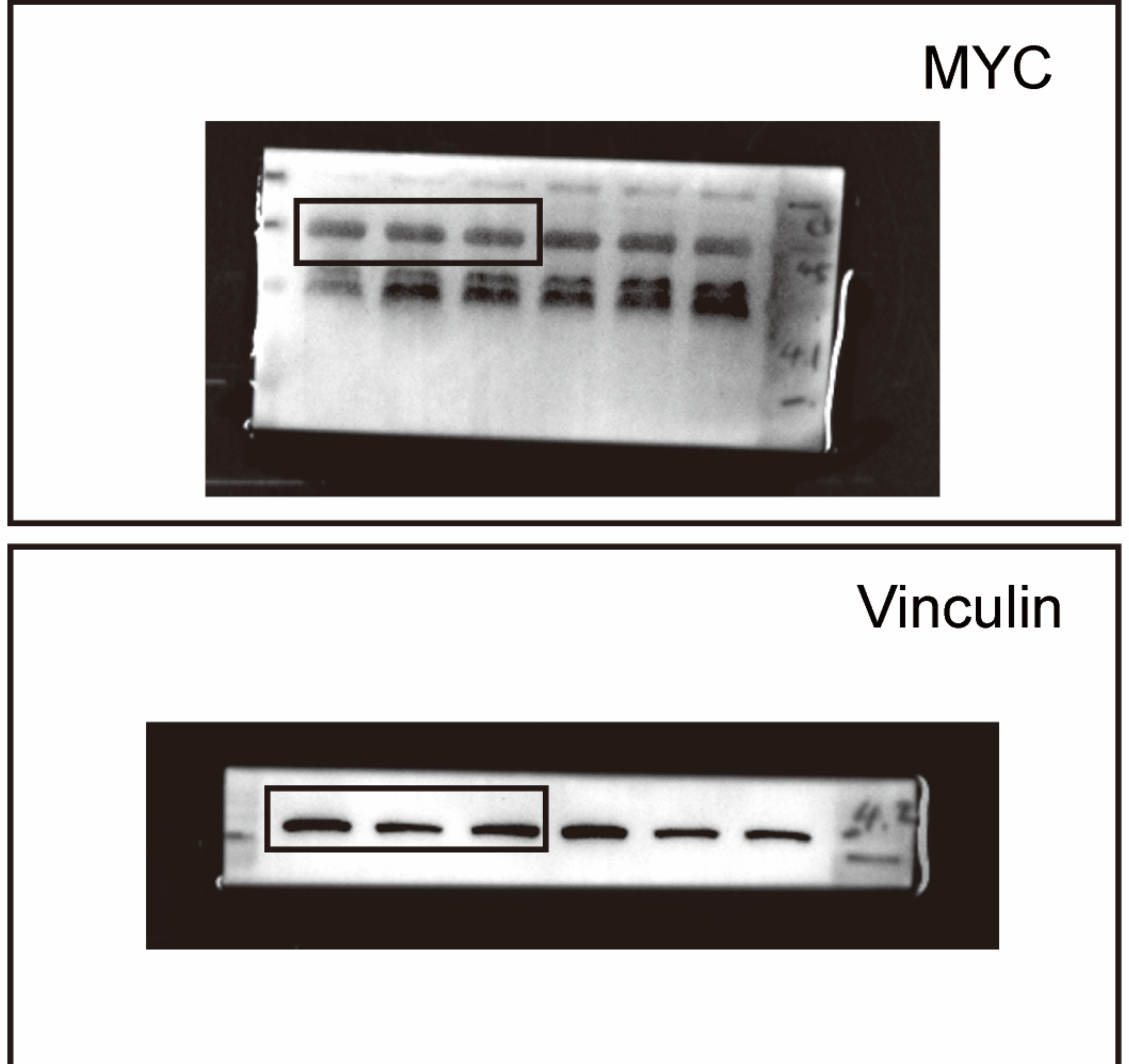

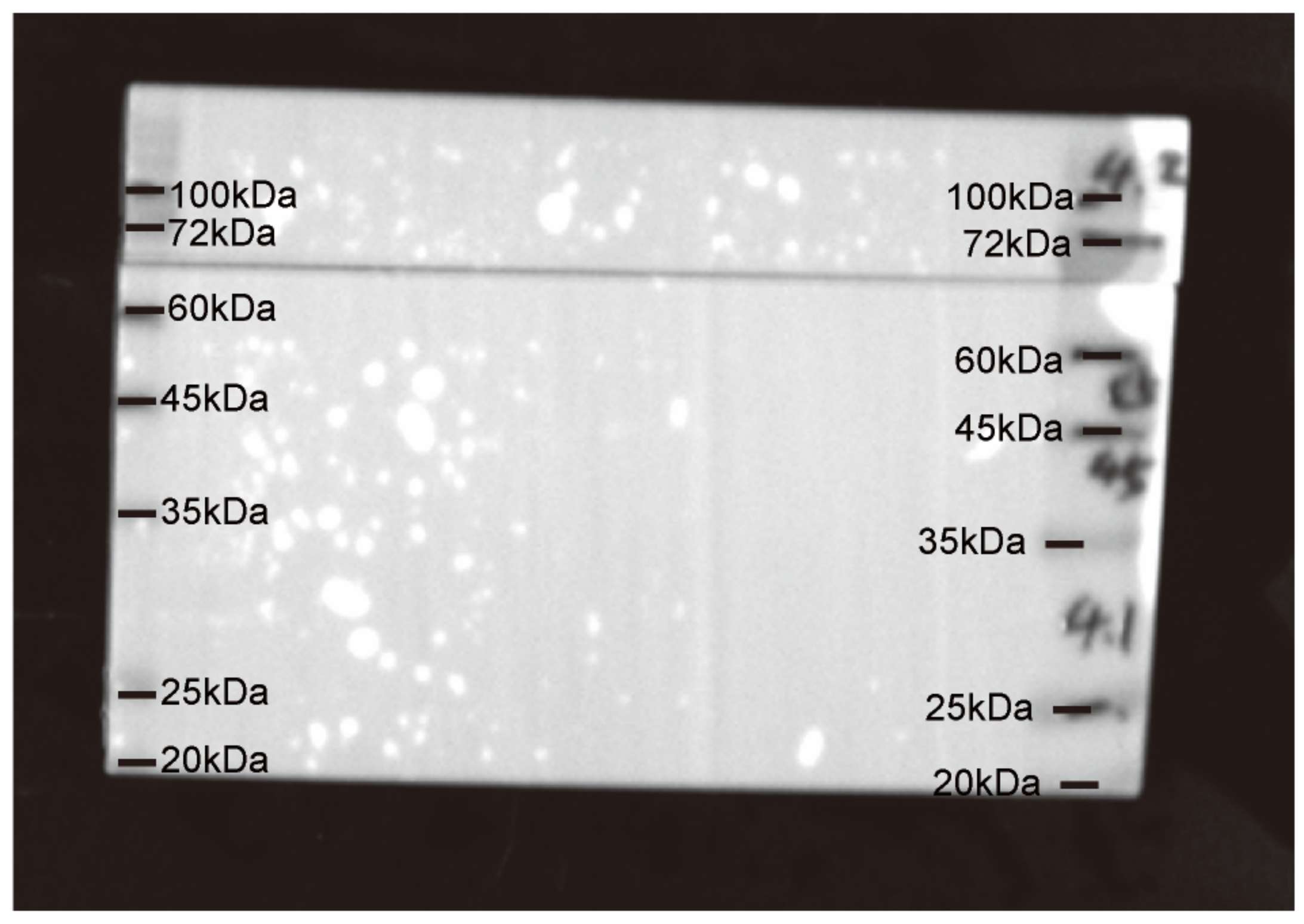

Fig.6I

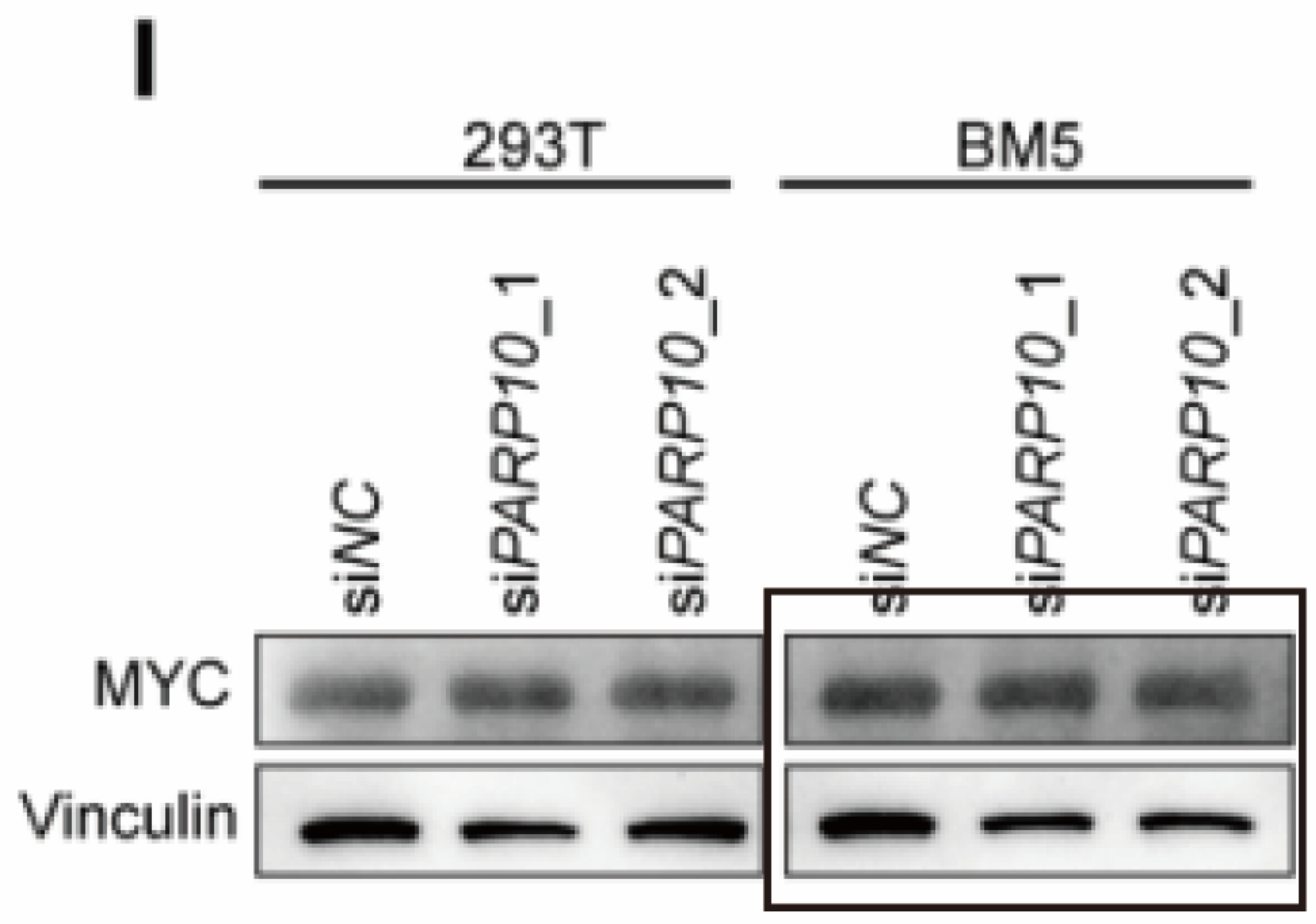

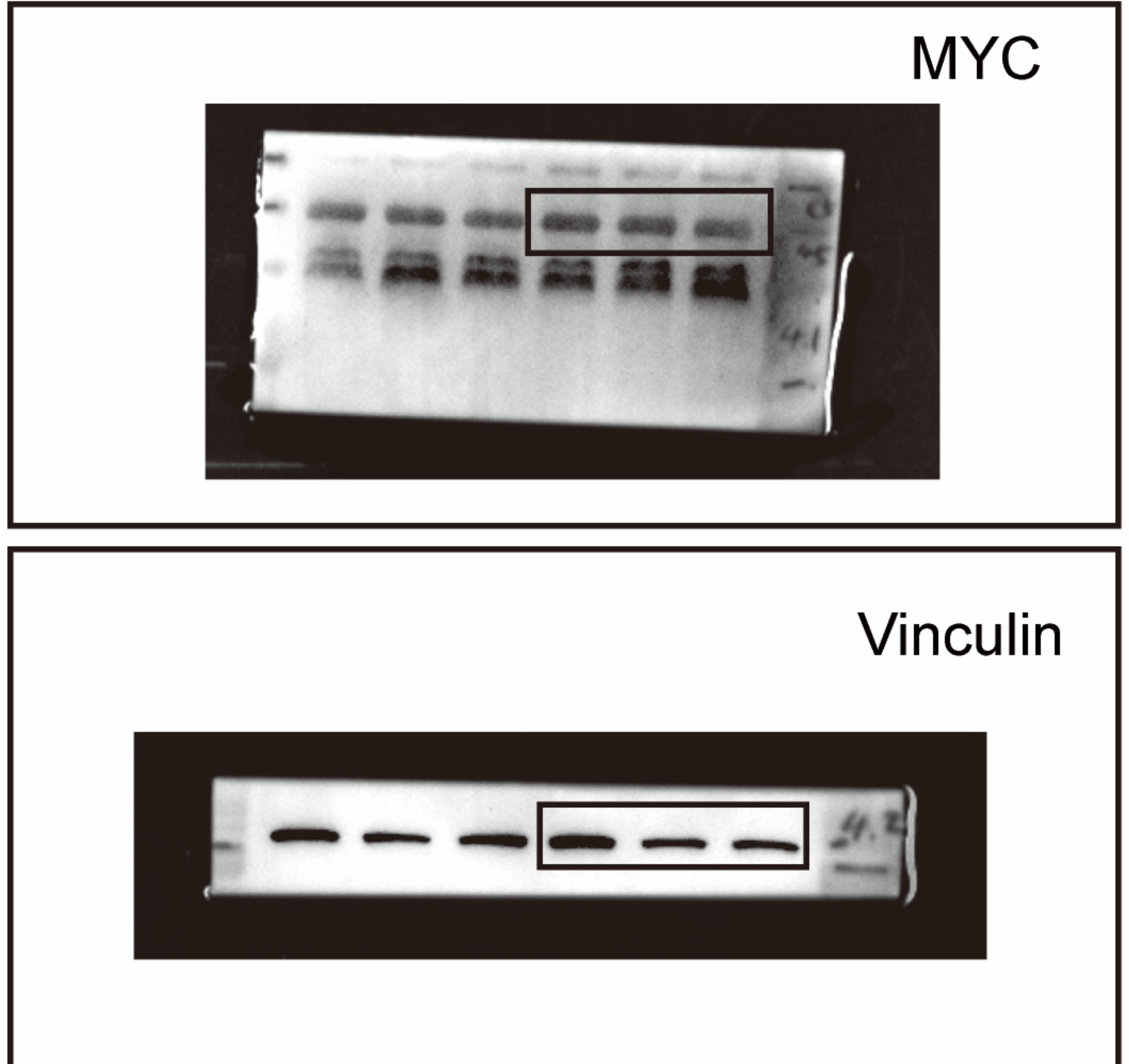

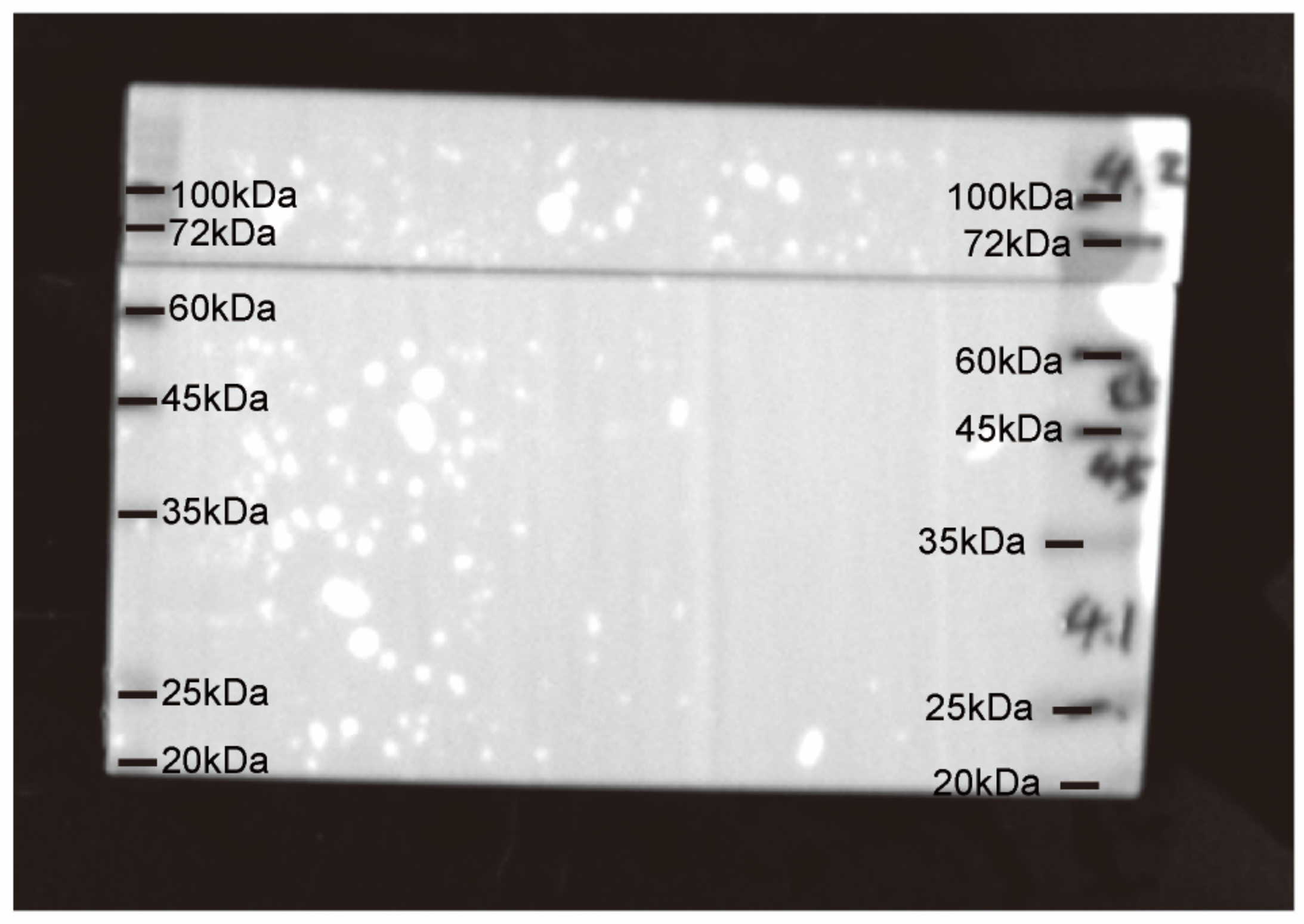

Fig.6I

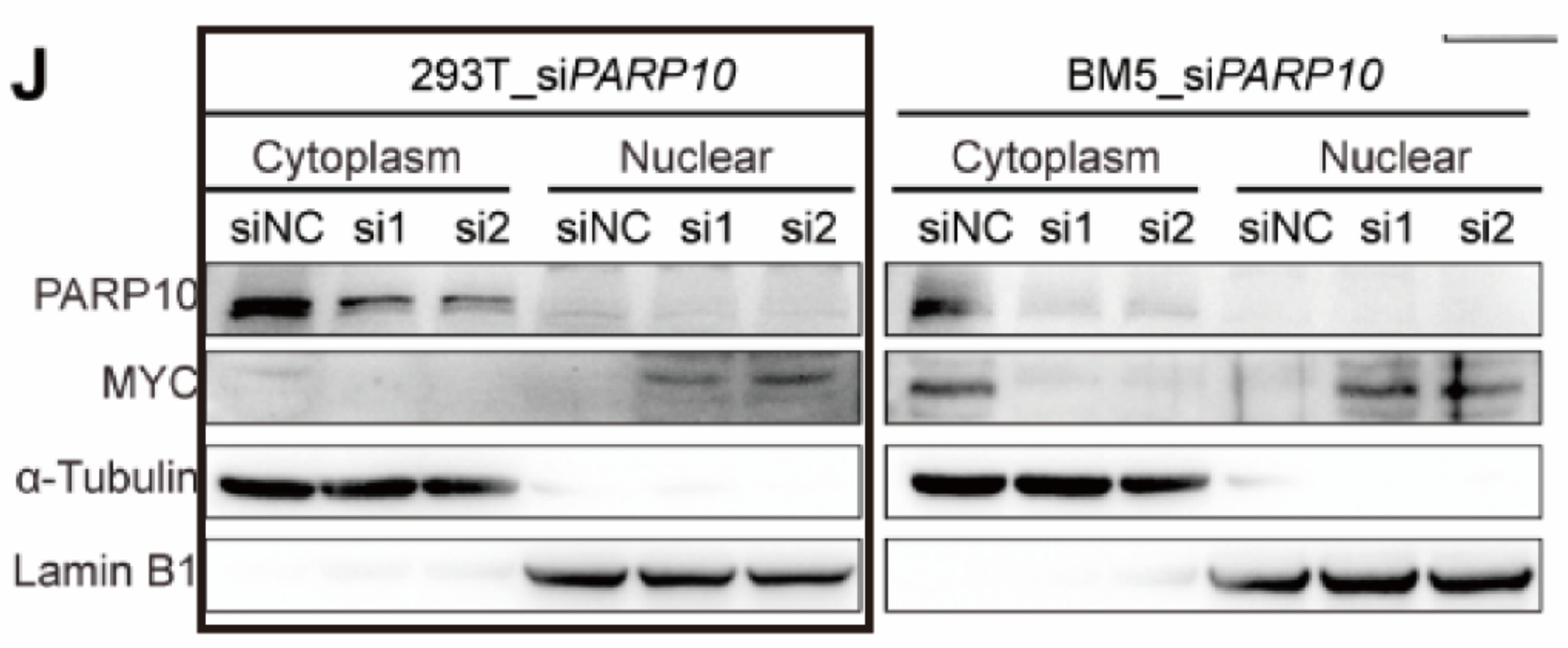

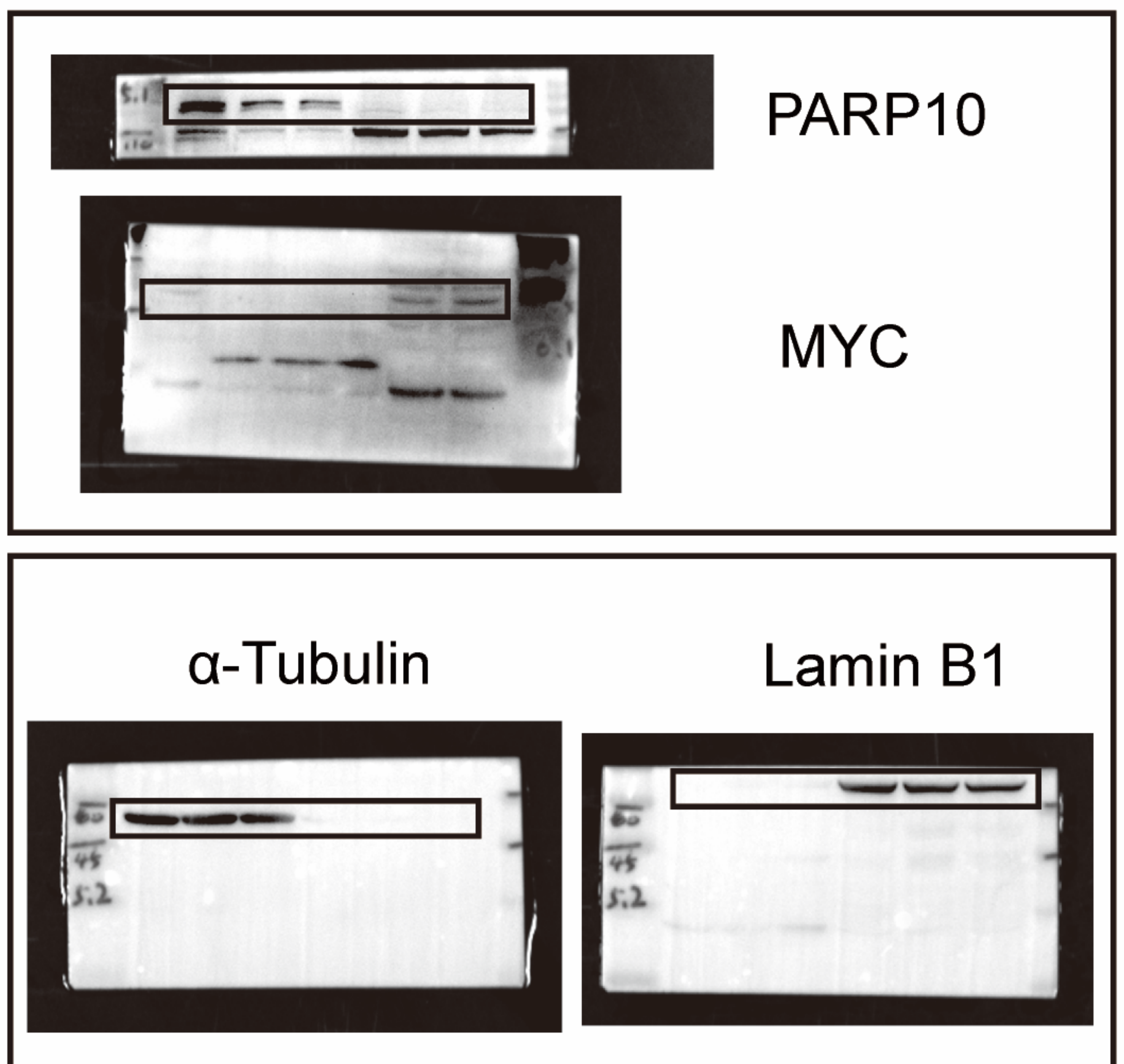

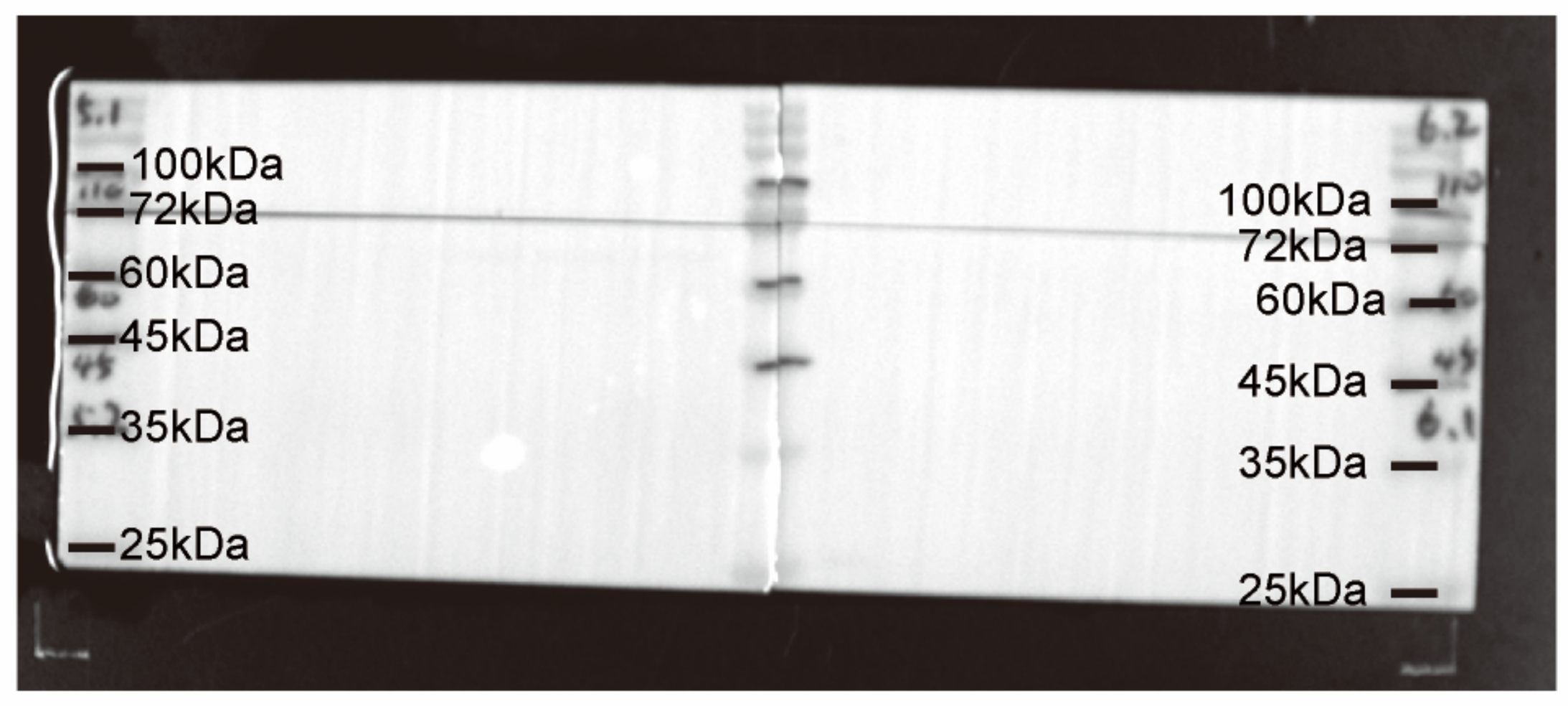

Fig.6I

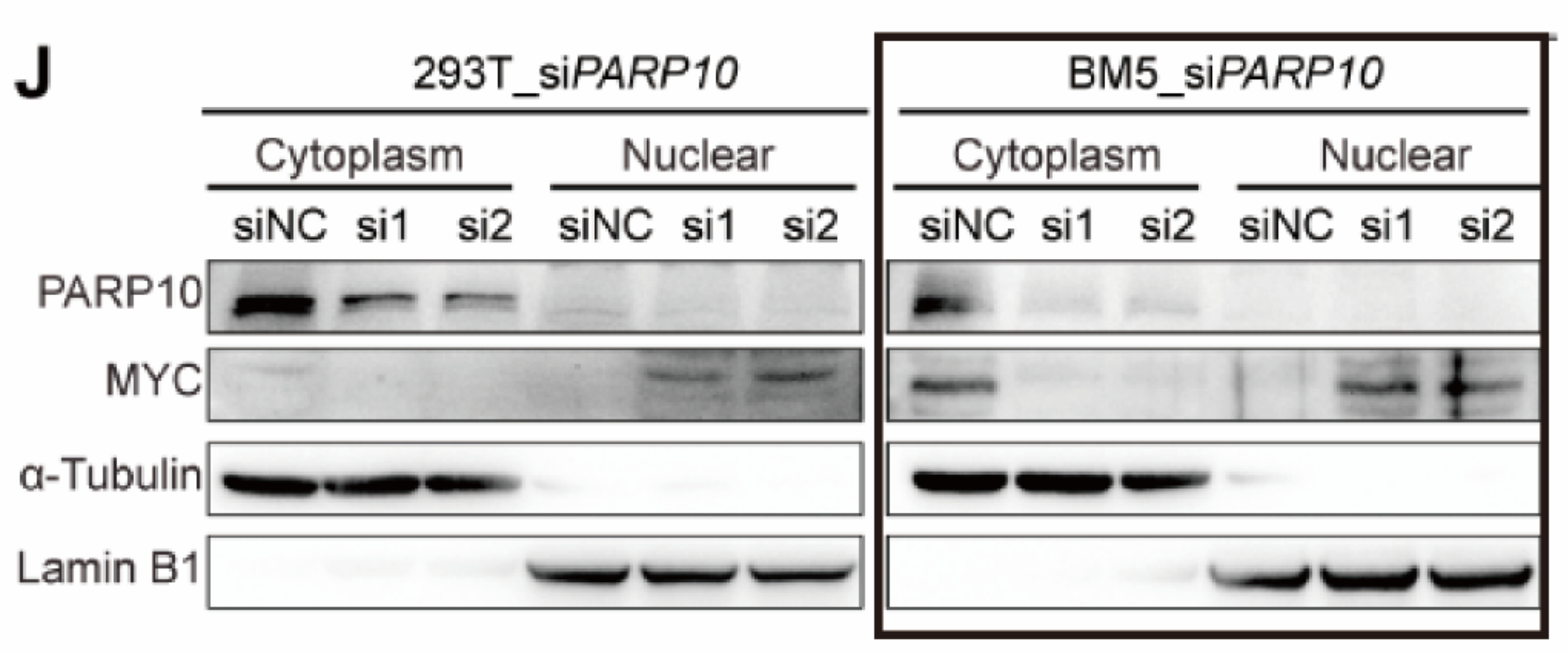

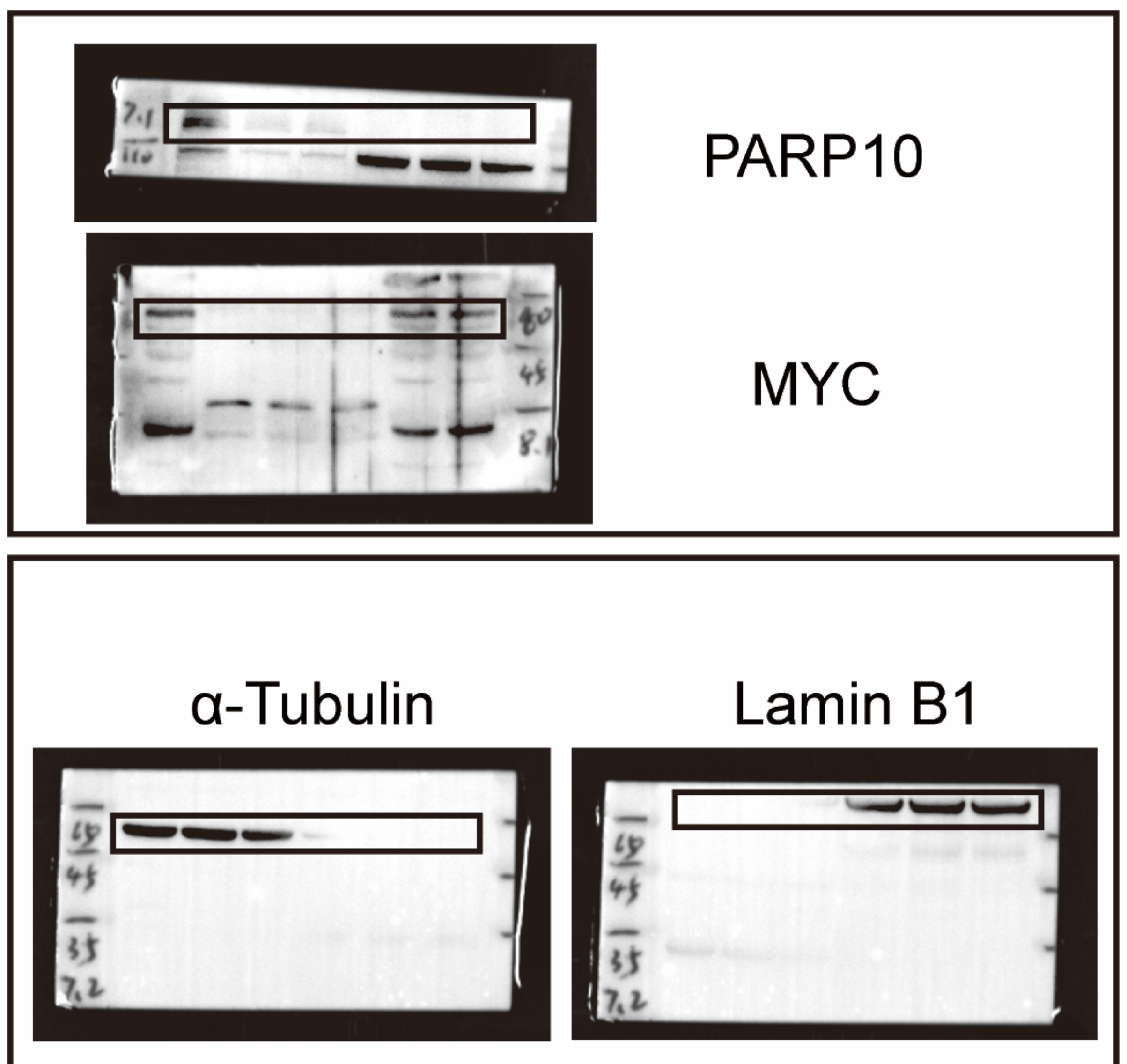

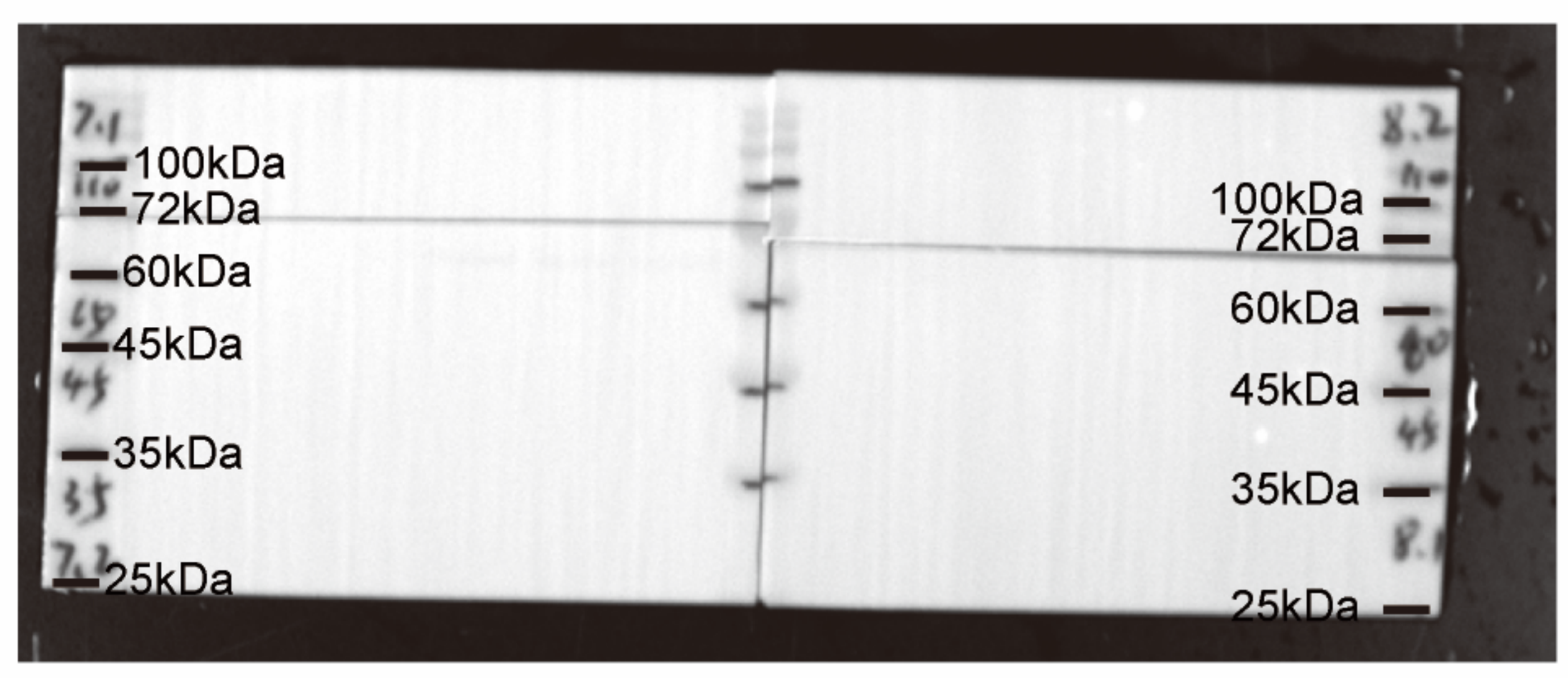

Figure	Merged	Stiched full image

Fig.6K

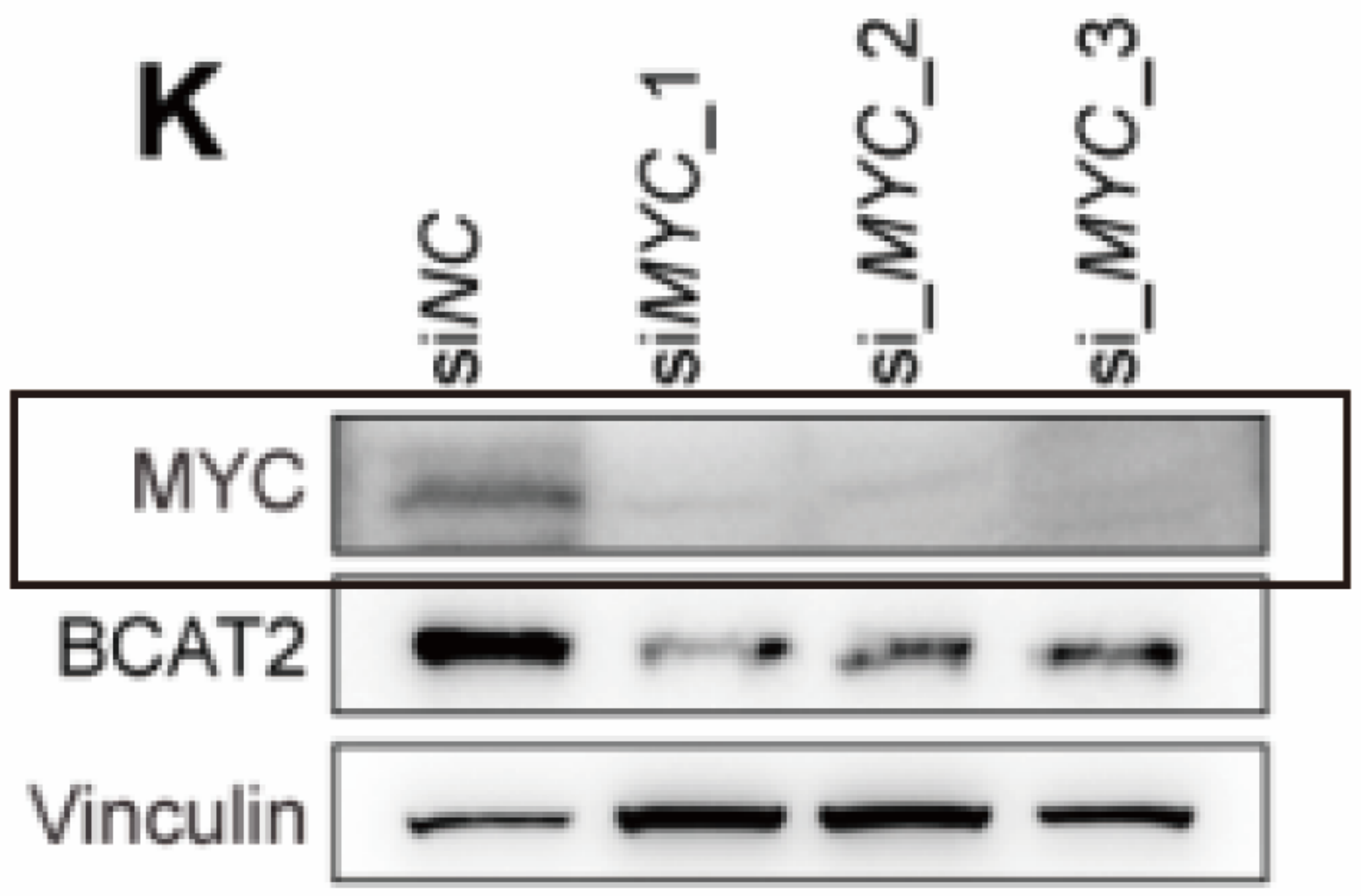

MYC

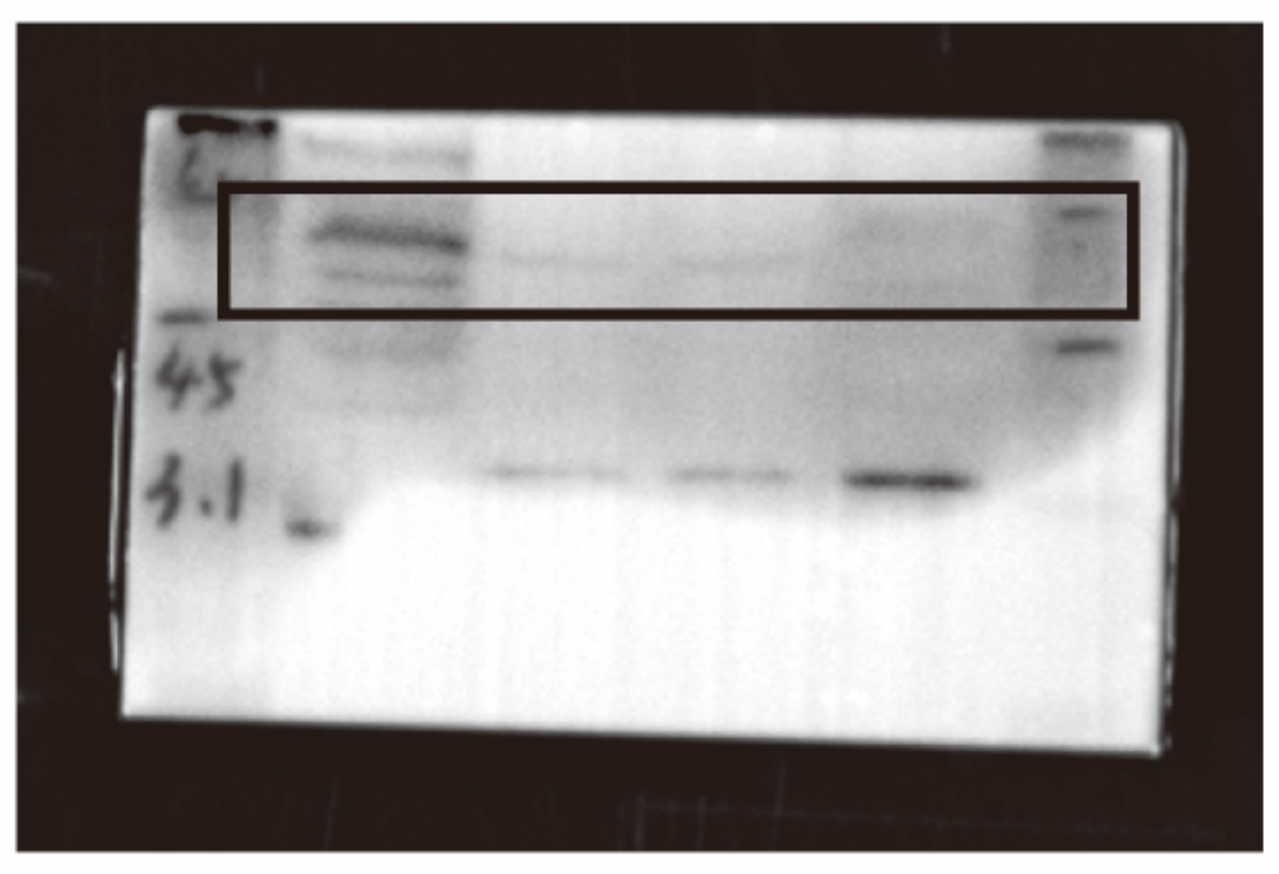

Vinculin

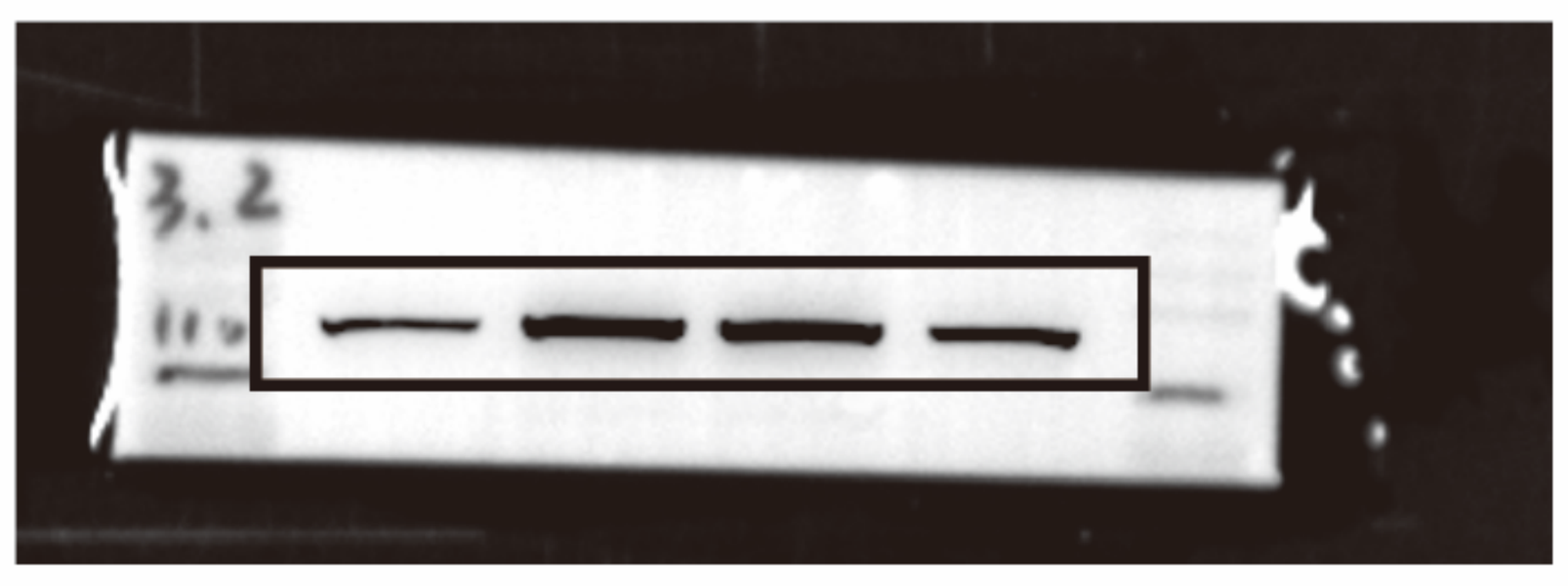

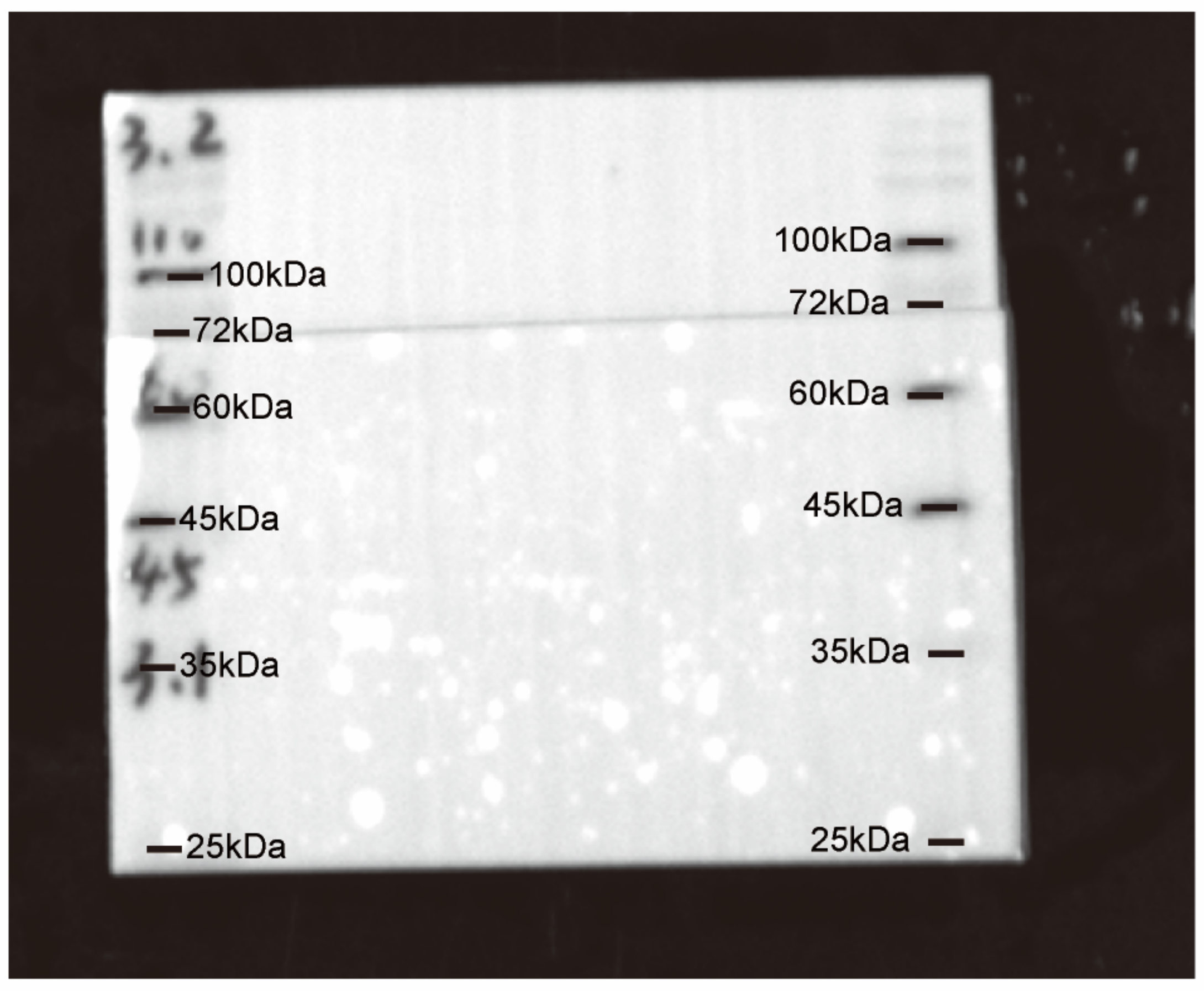

Fig.6K

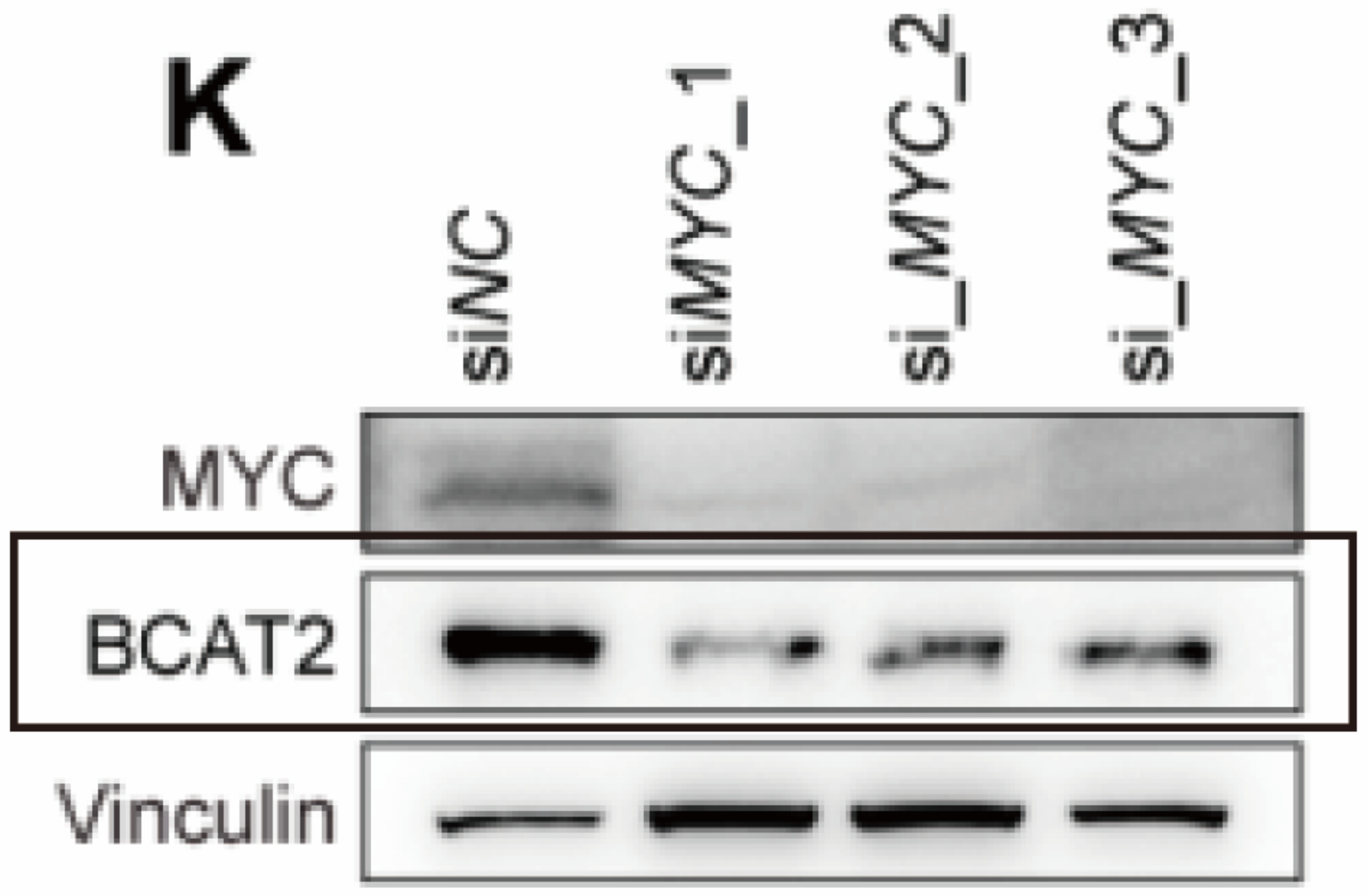

BCAT2

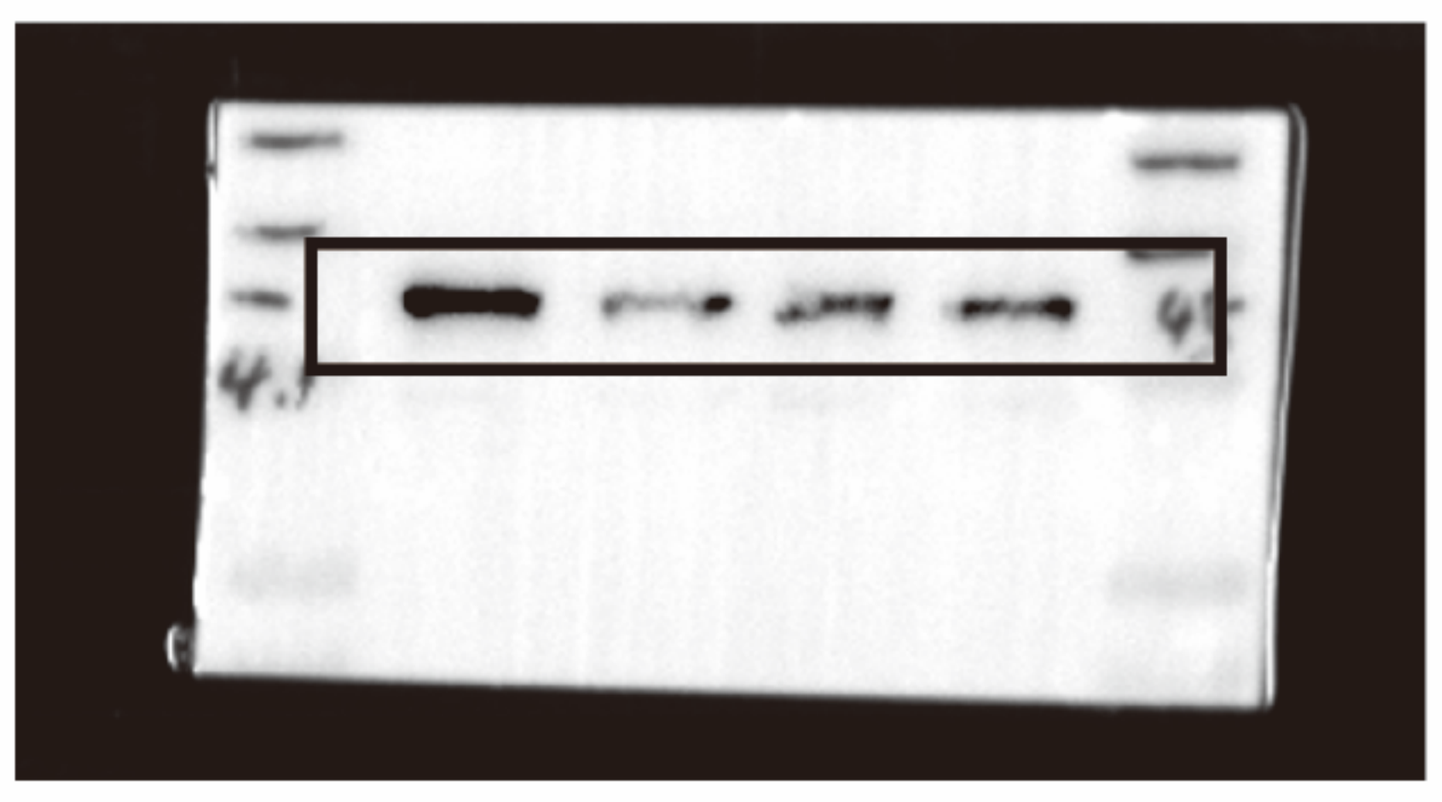

Vinculin

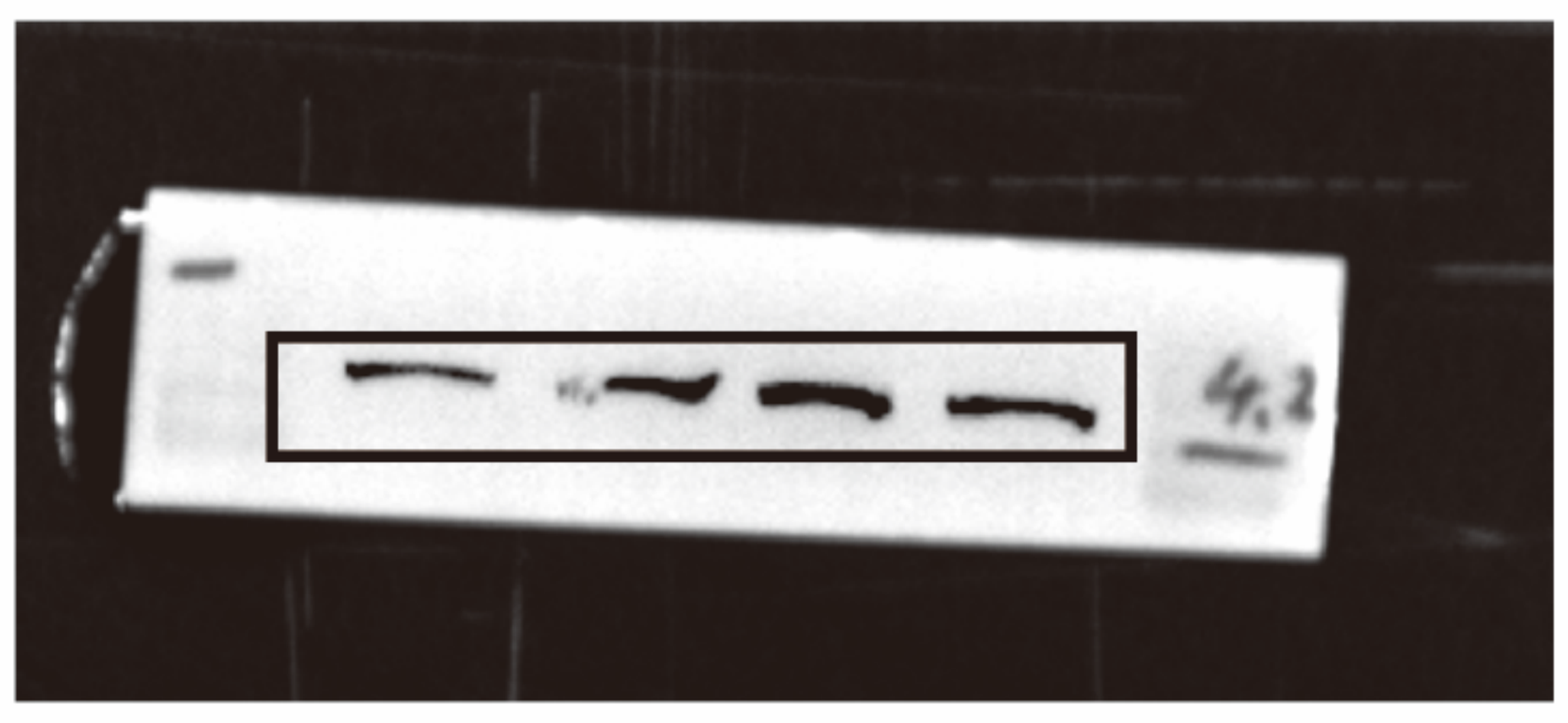

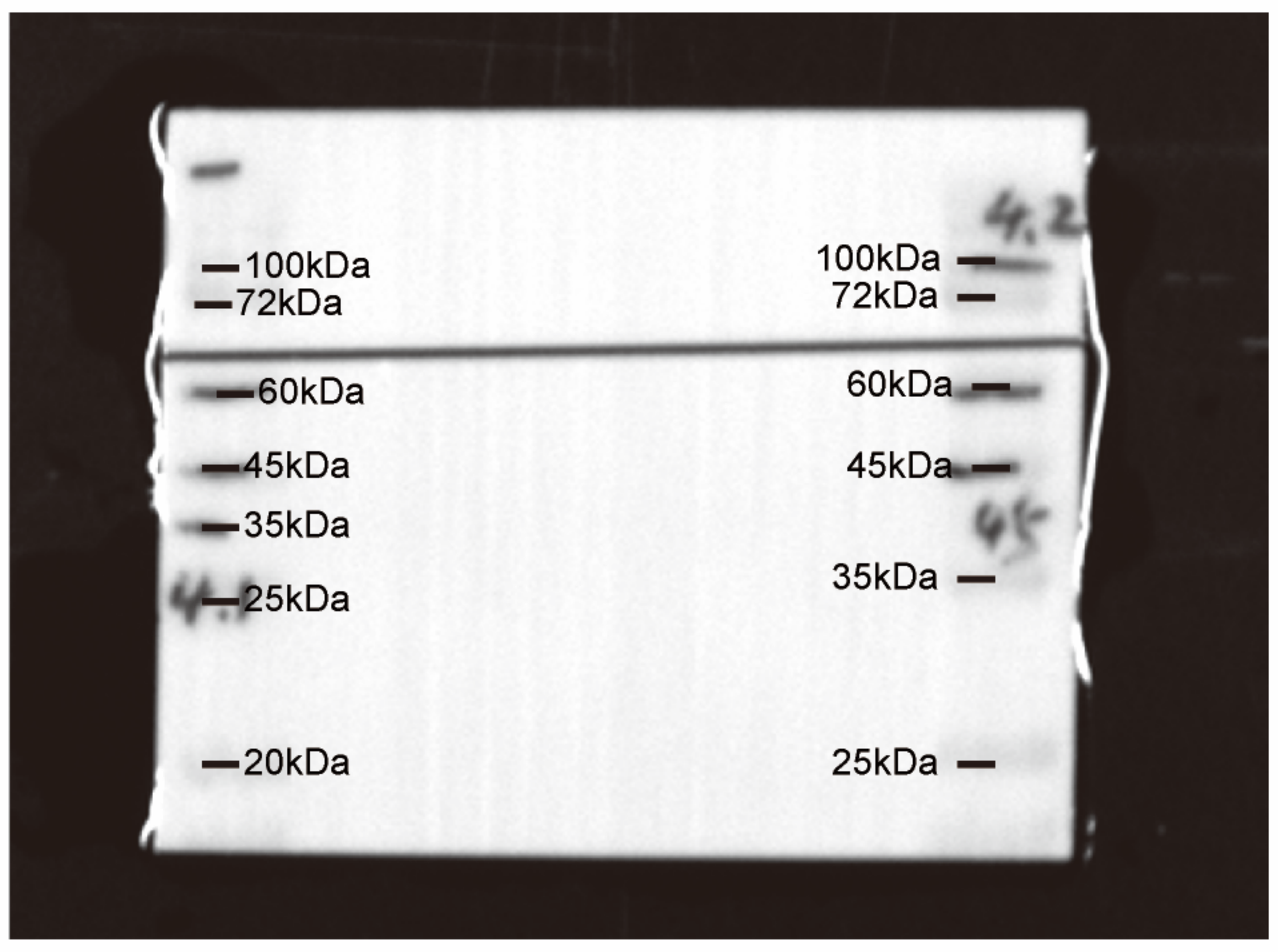

Fig.6L

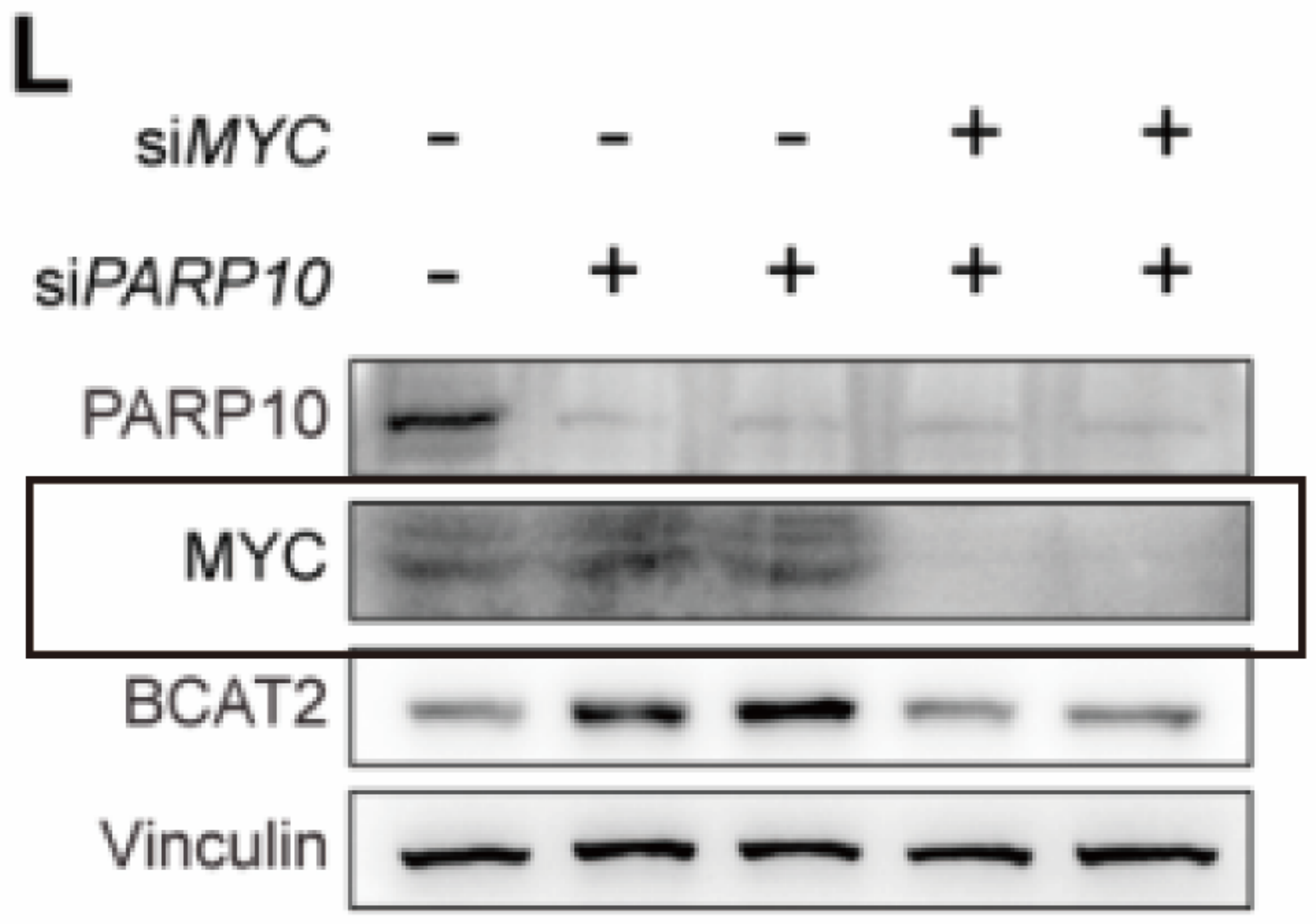

MYC

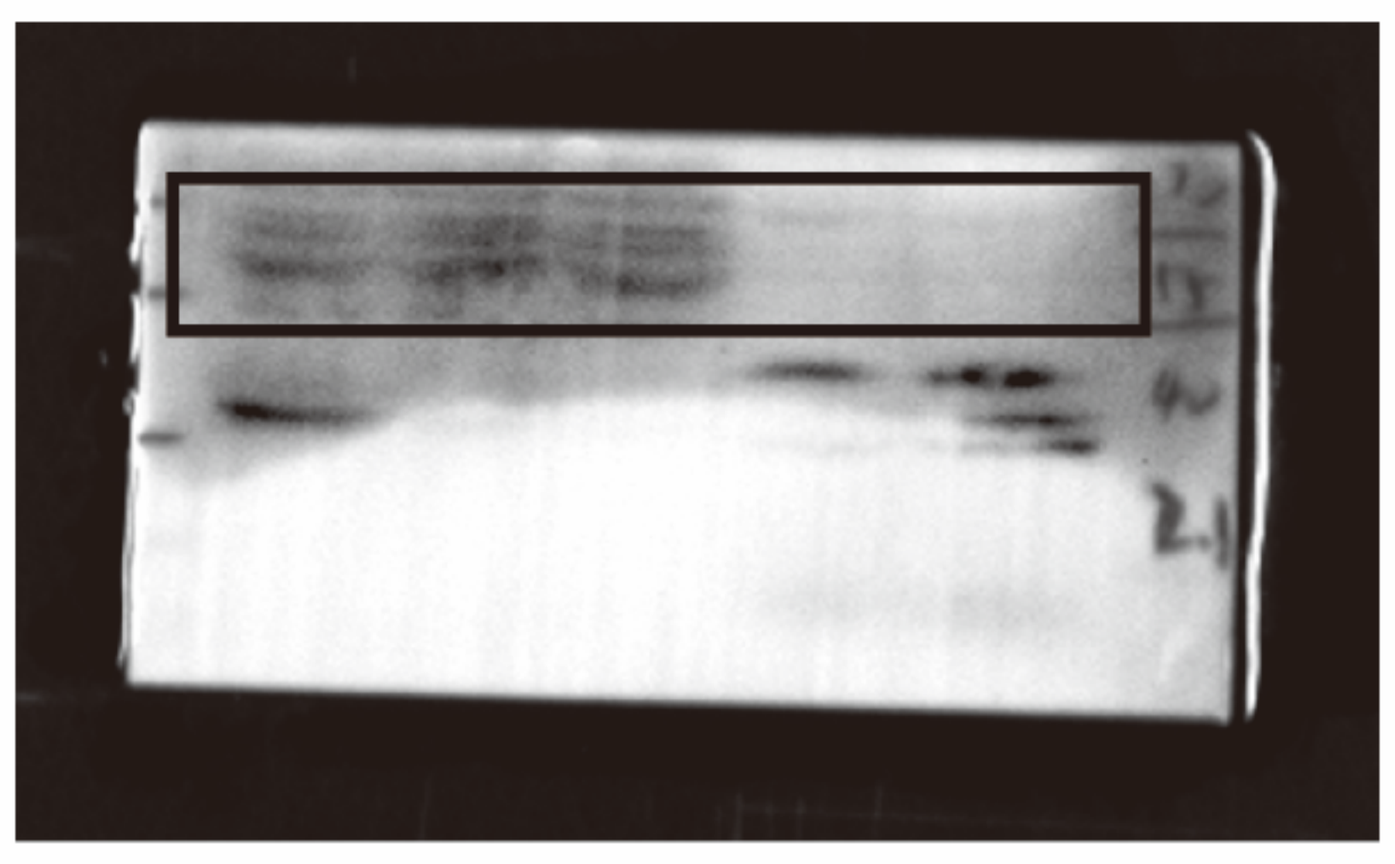

Vinculin

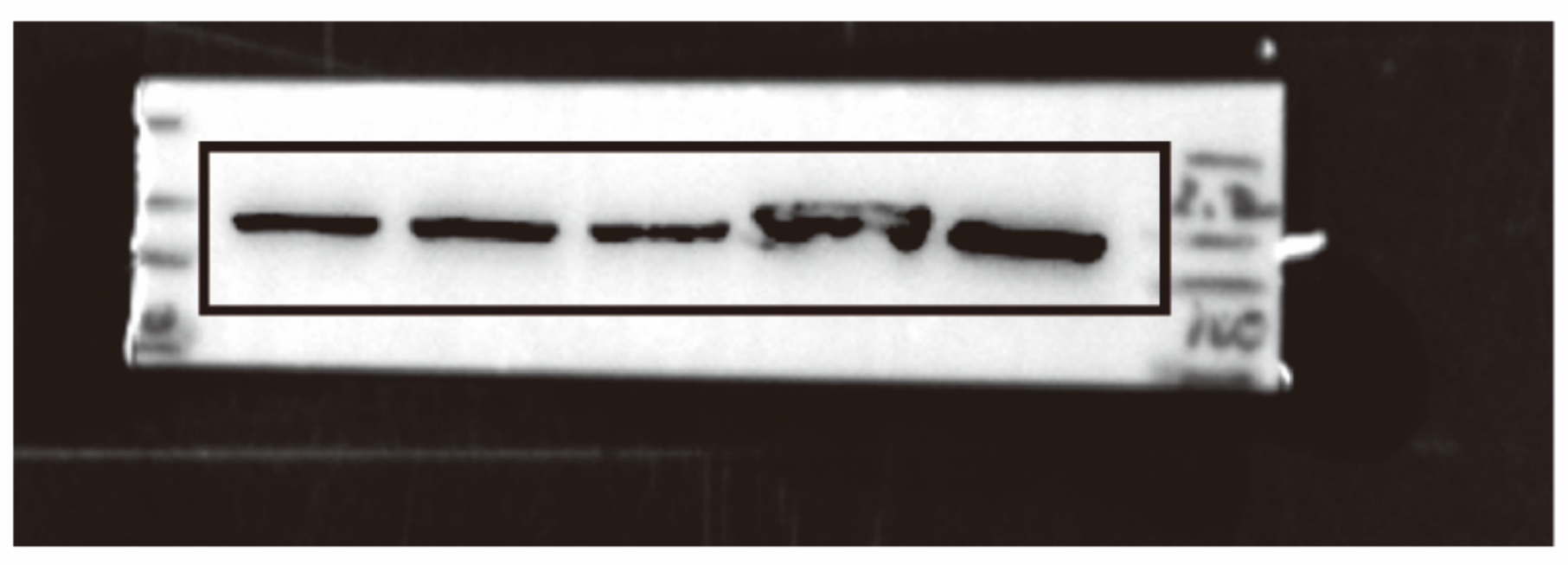

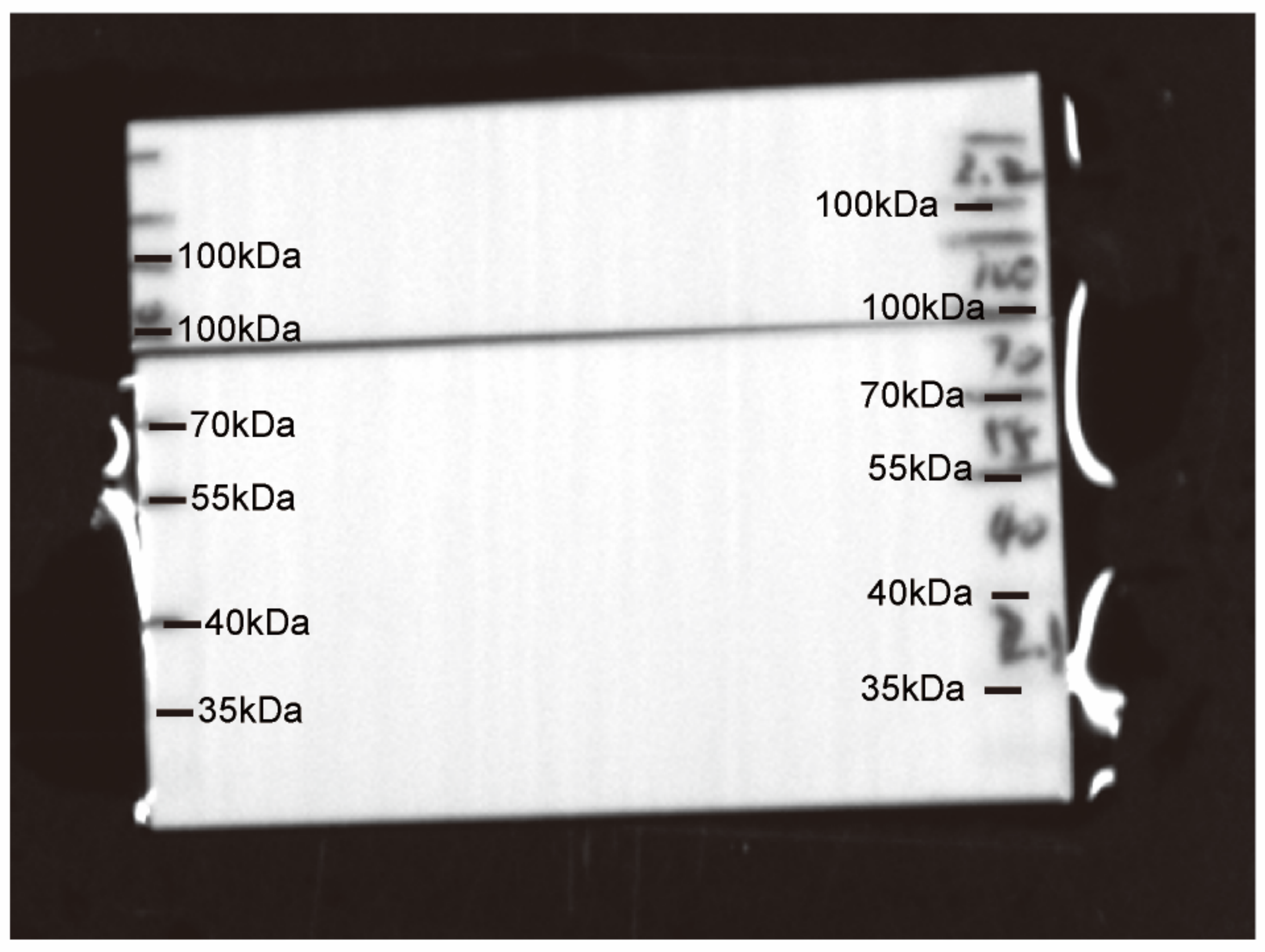

Fig.6L

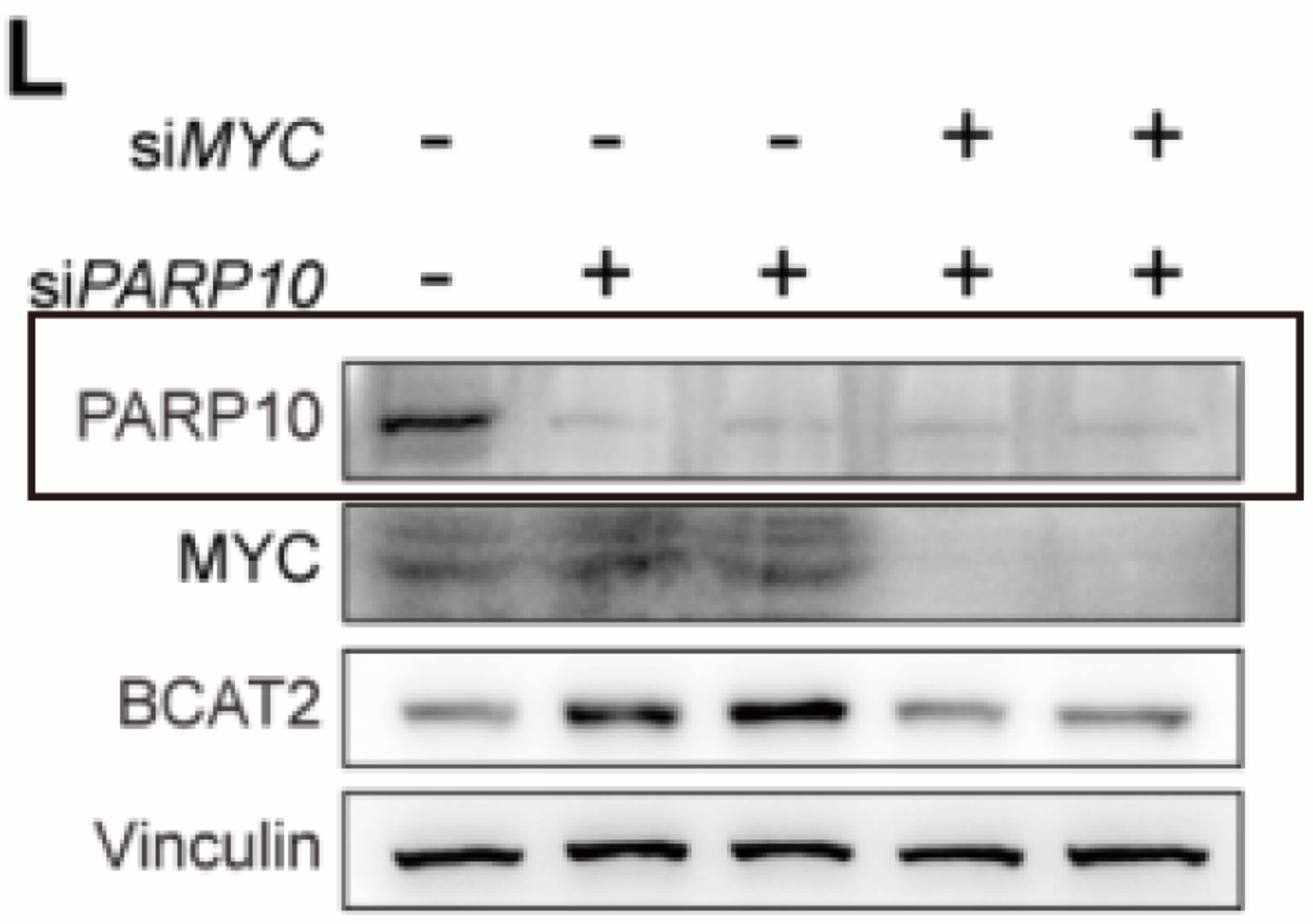

PARP10

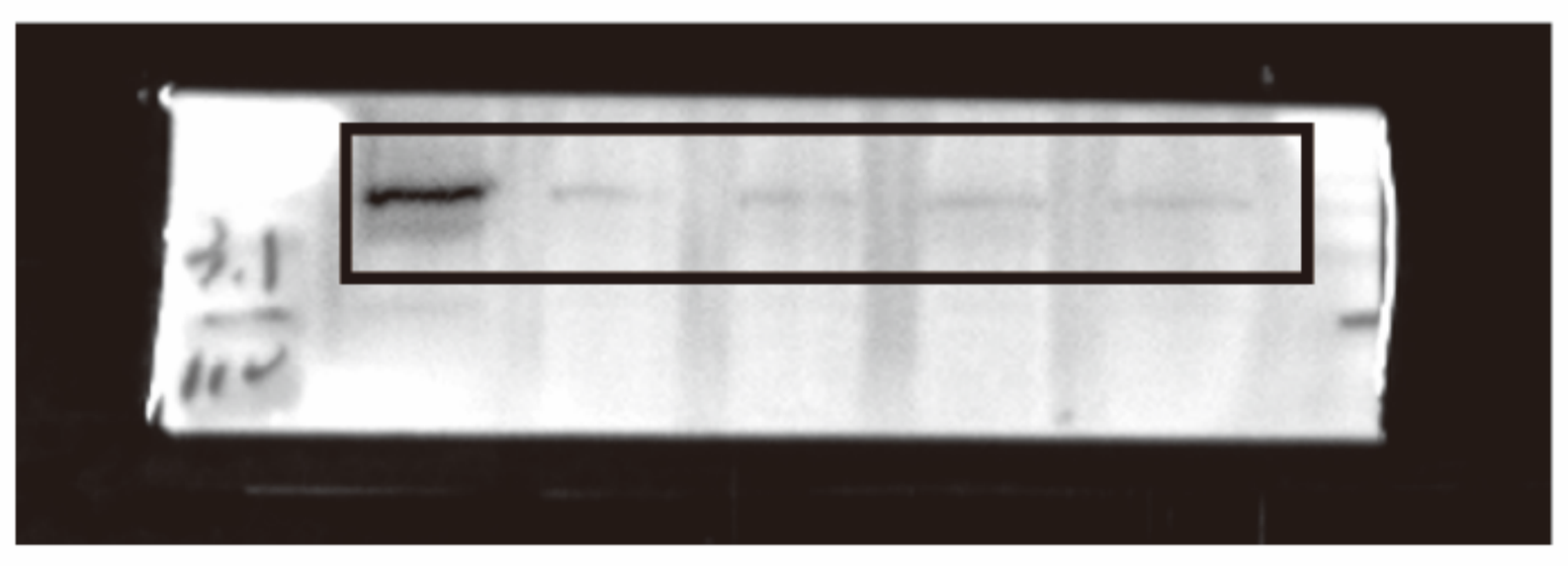

β-actin

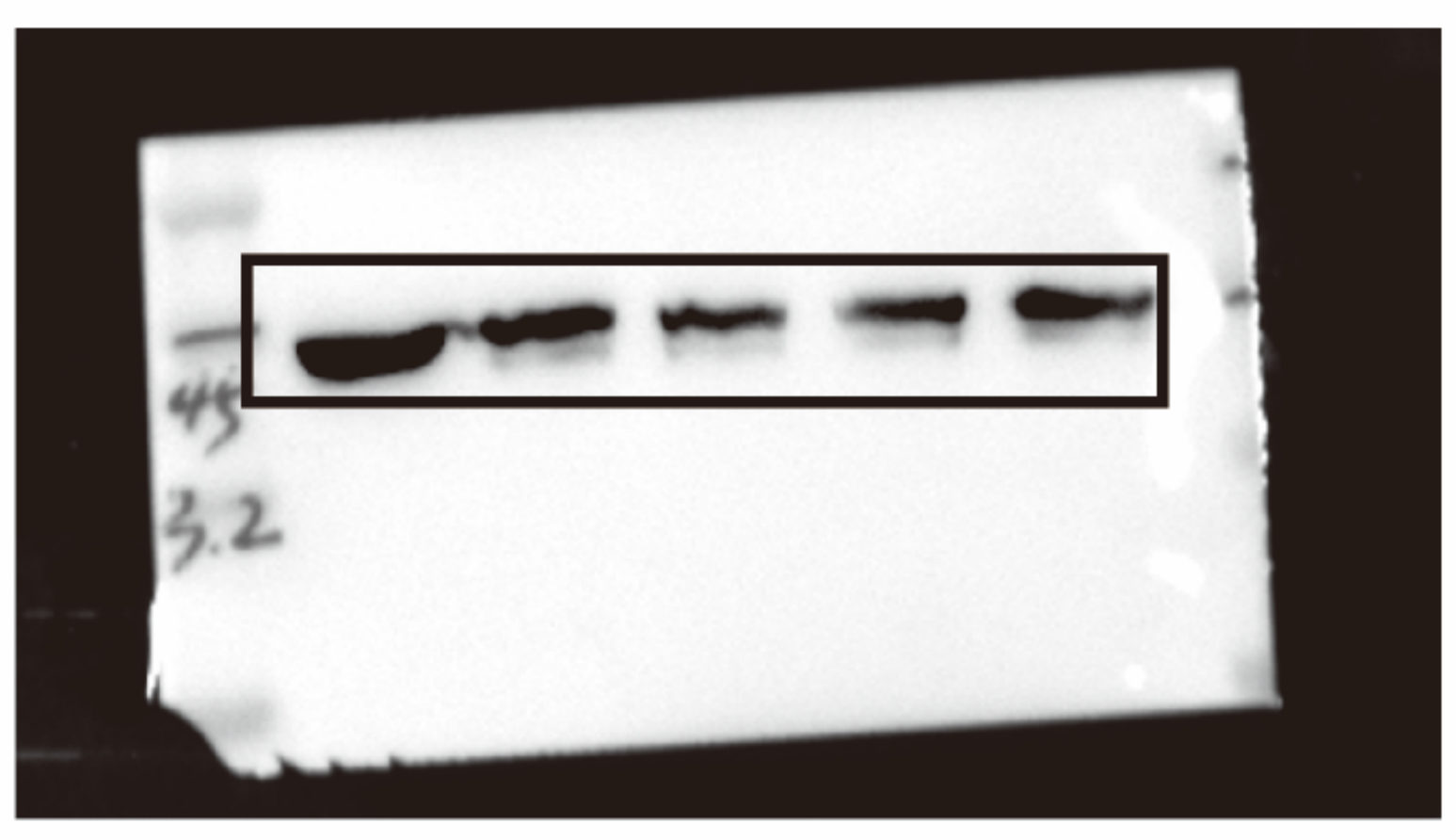

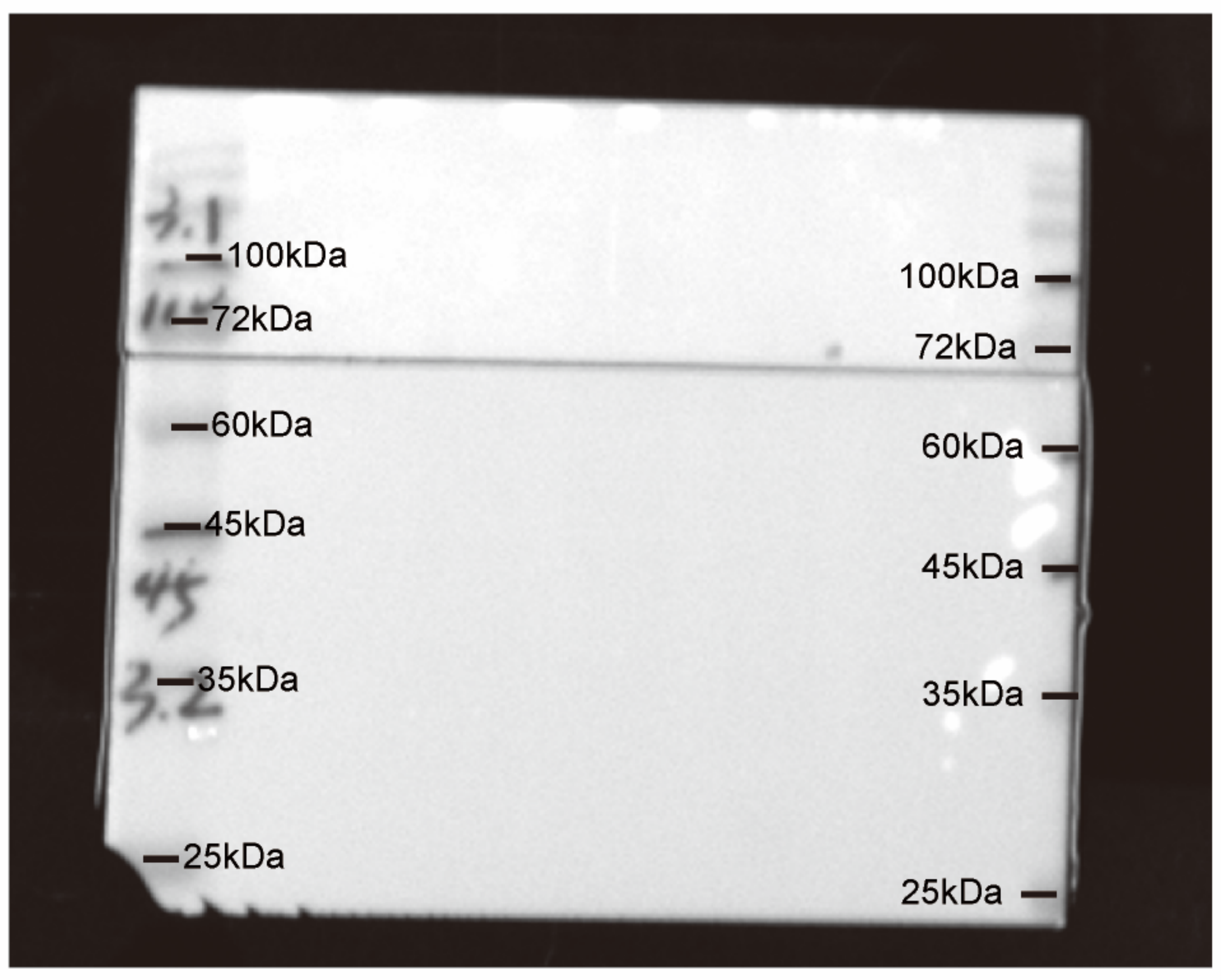

Figure	Merged	Stiched full image

Fig.6L

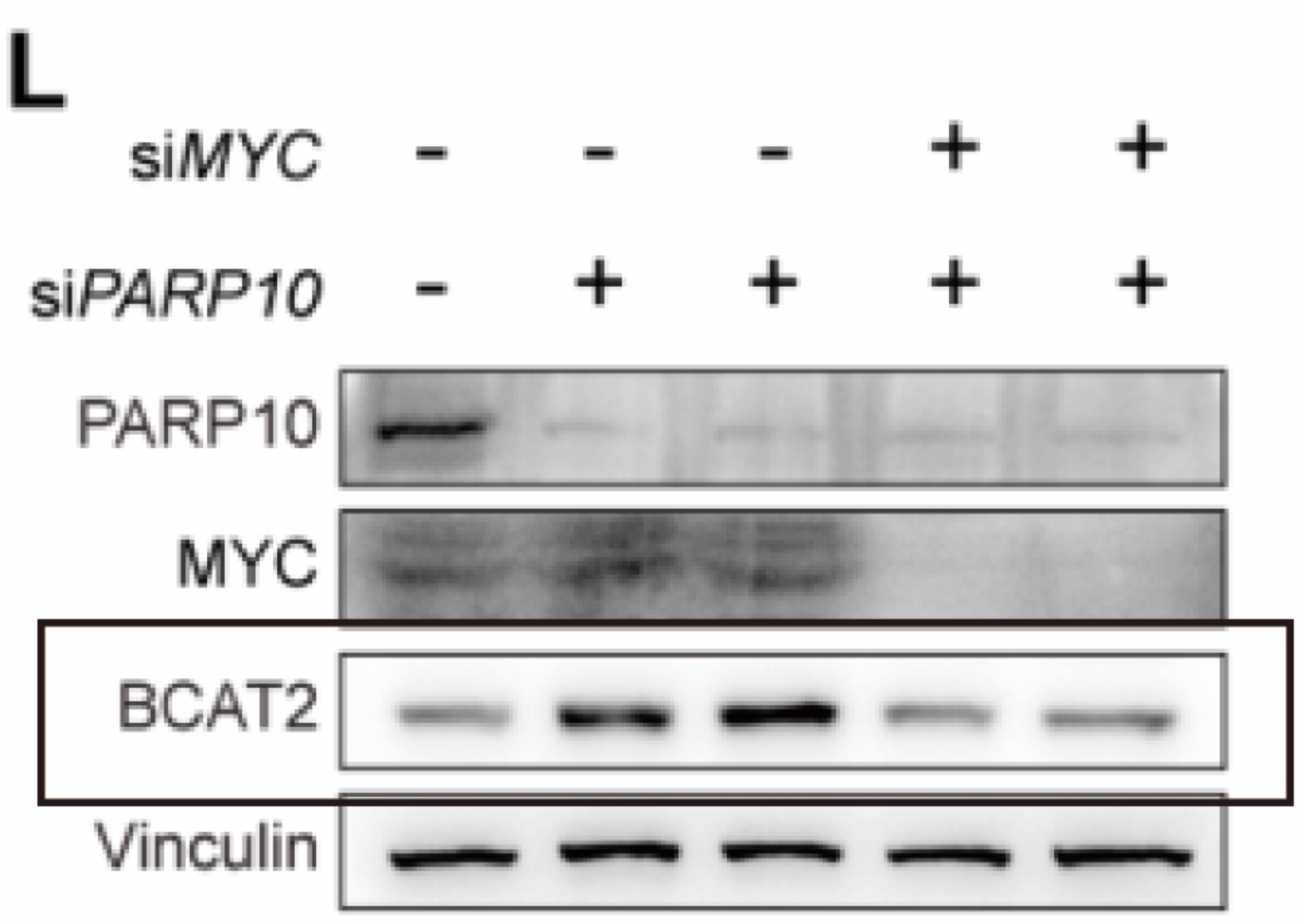

BCAT2

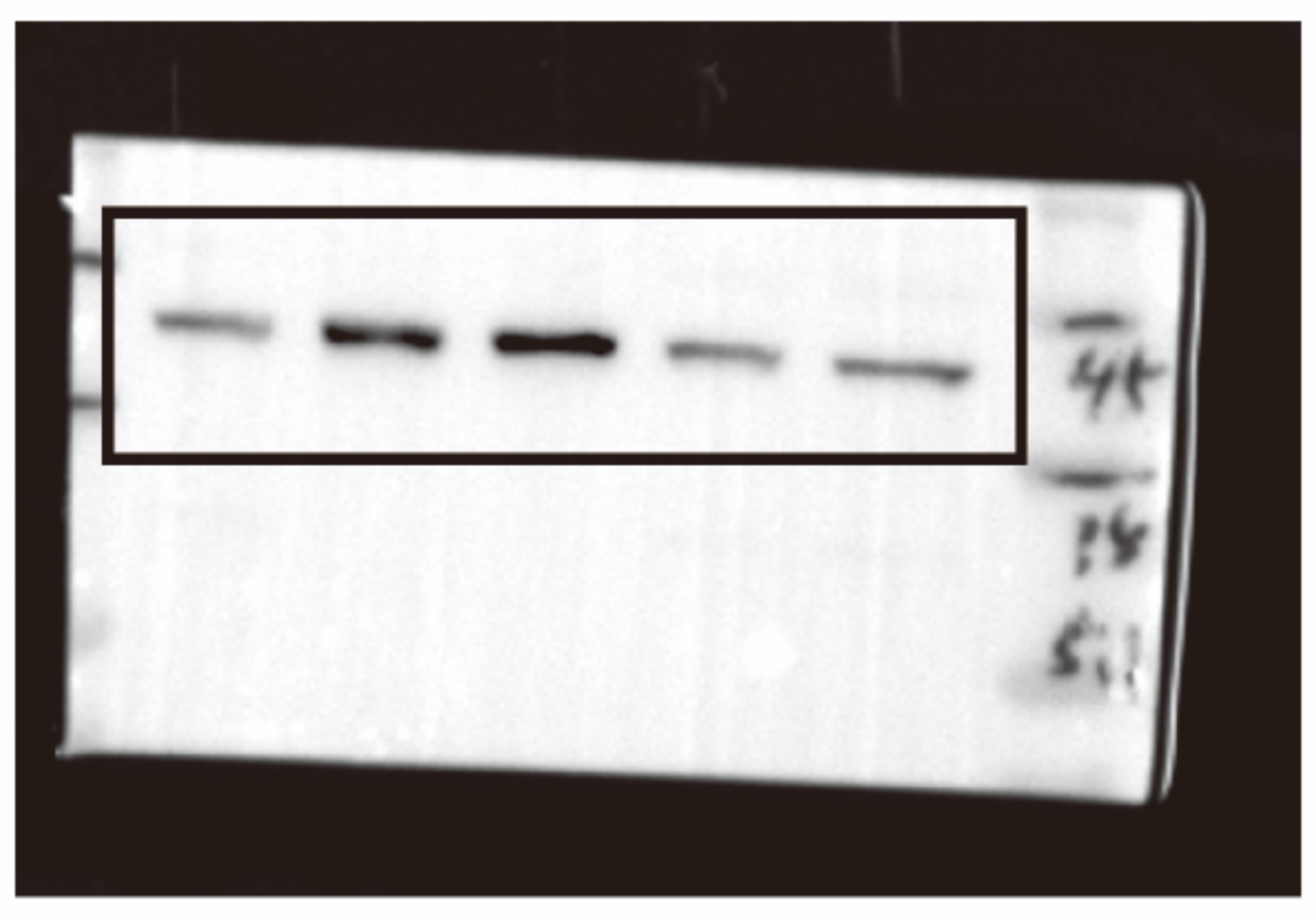

Vinculin

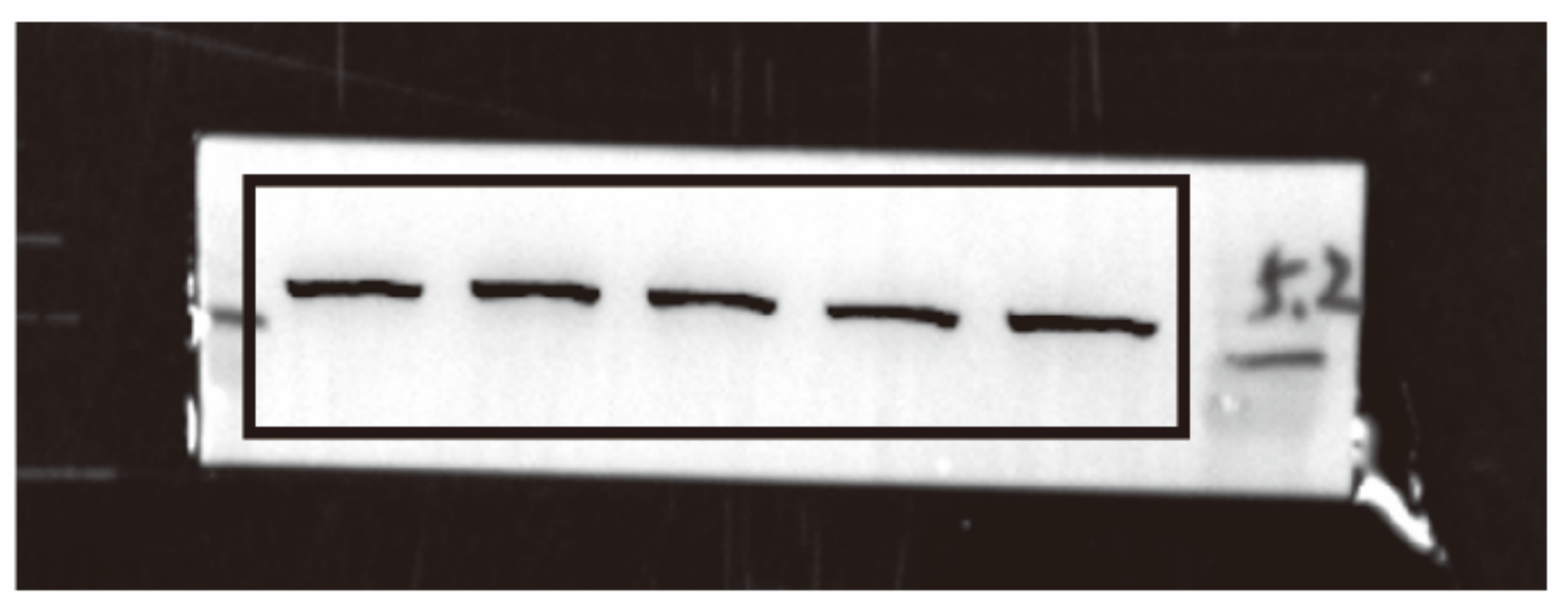

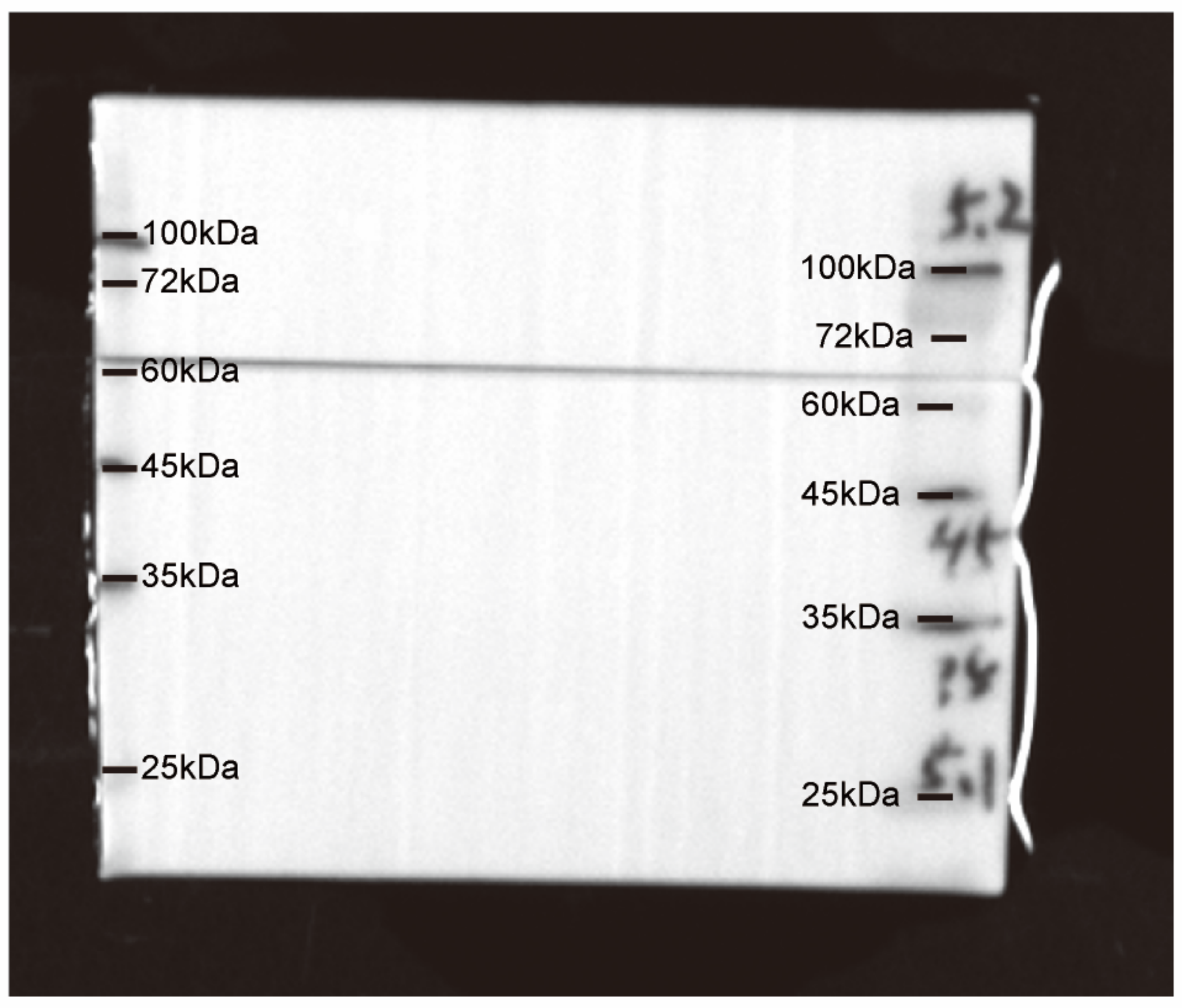

Fig.6M

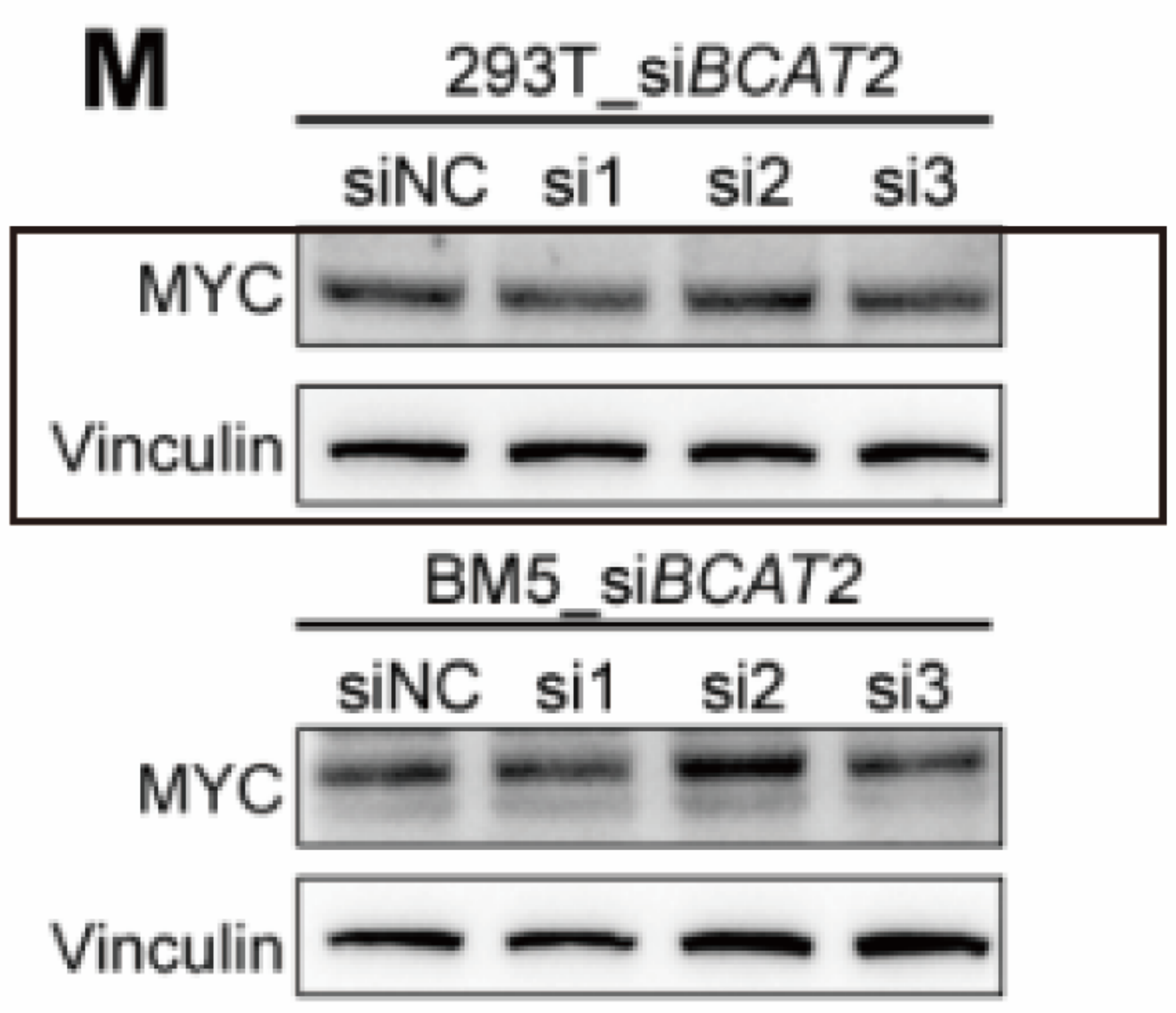

MYC

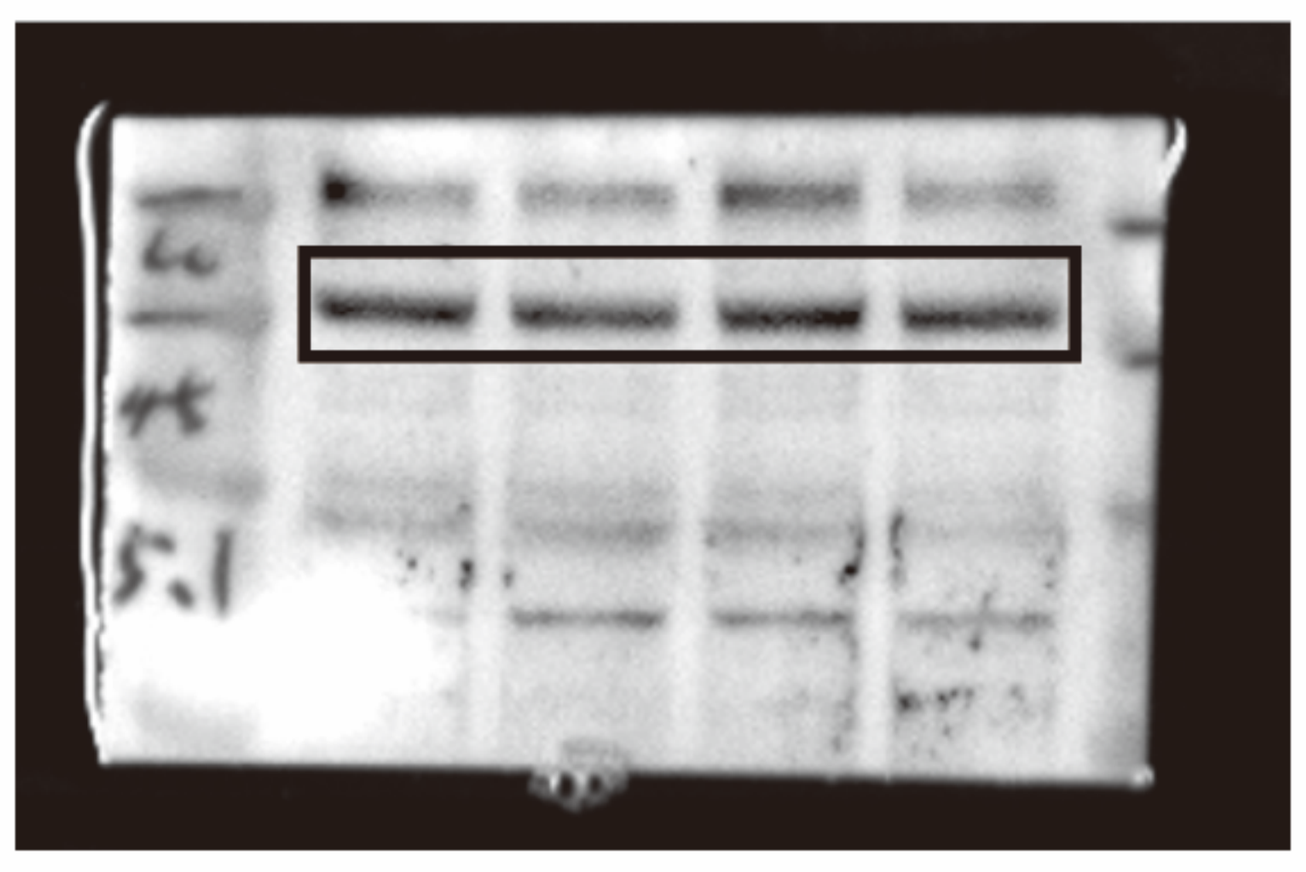

Vinculin

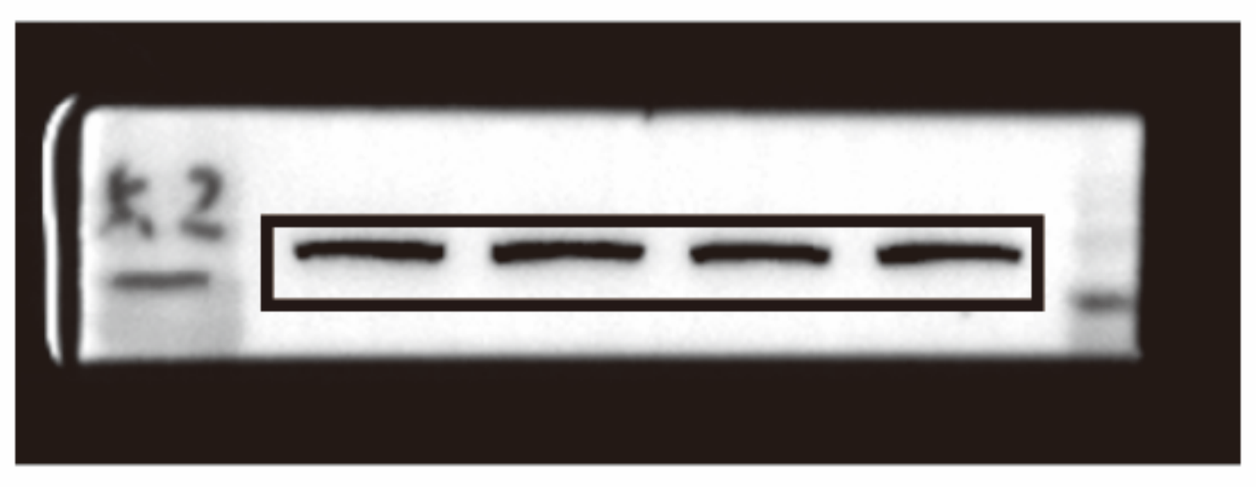

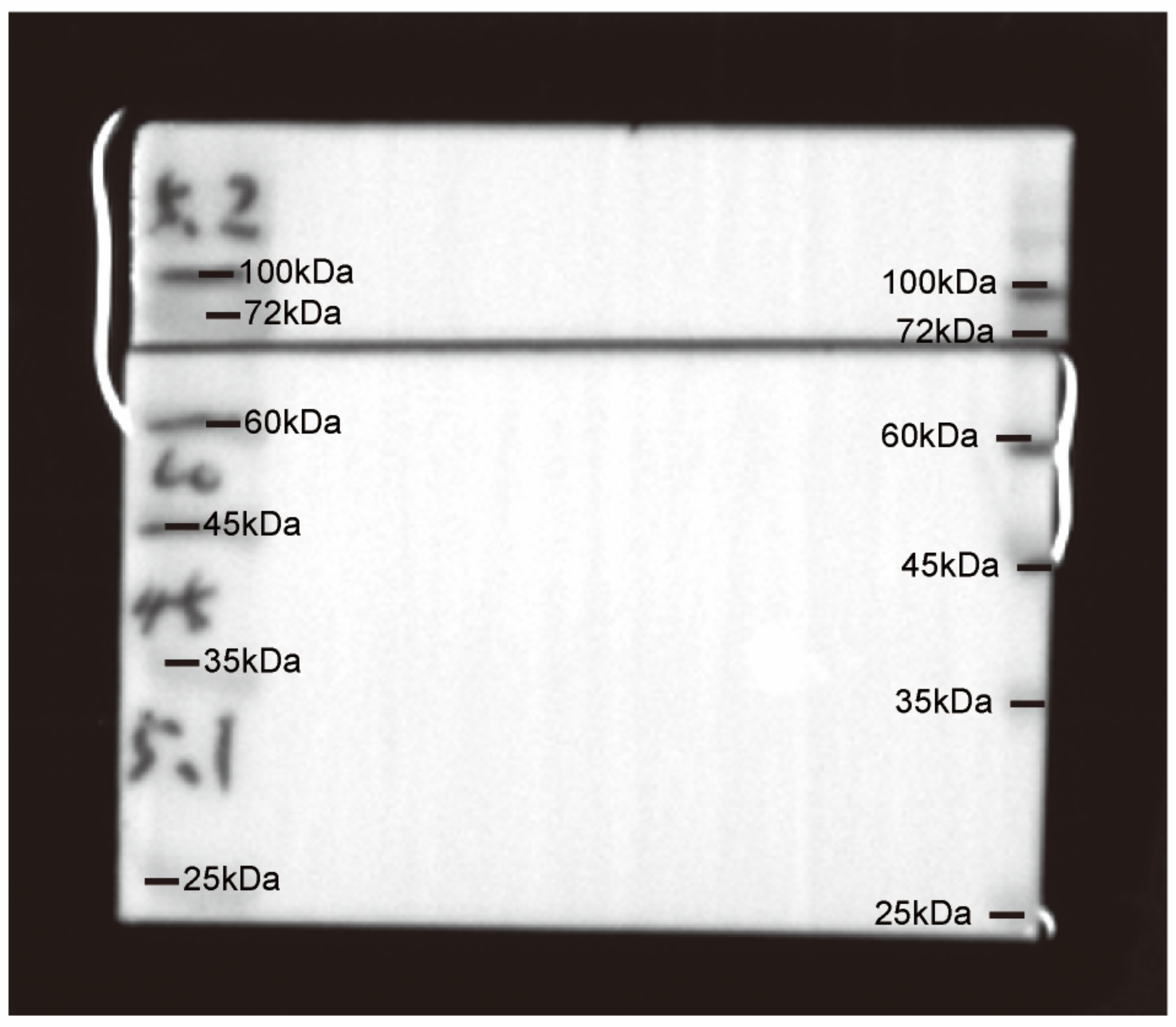

Fig.6M

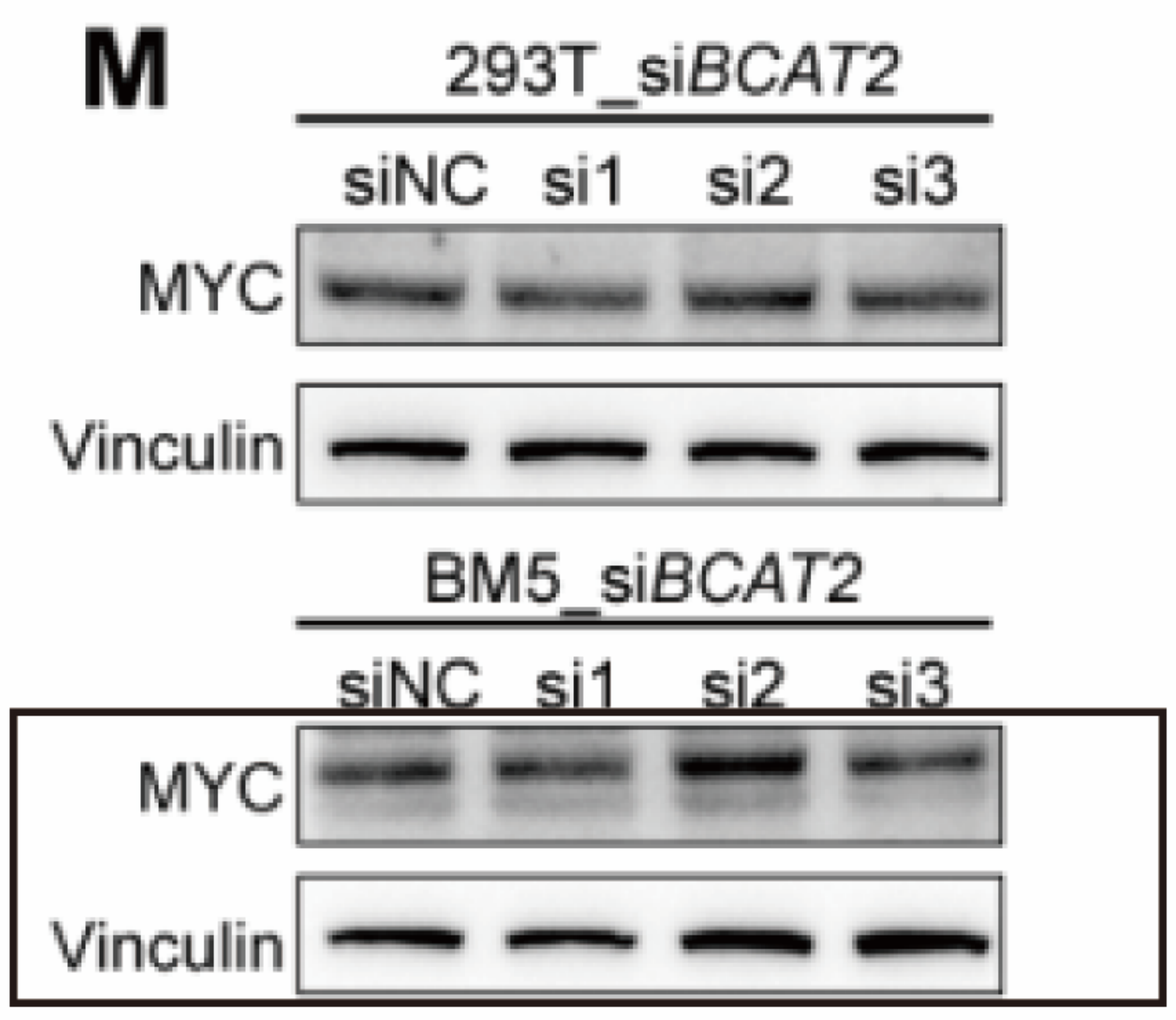

MYC

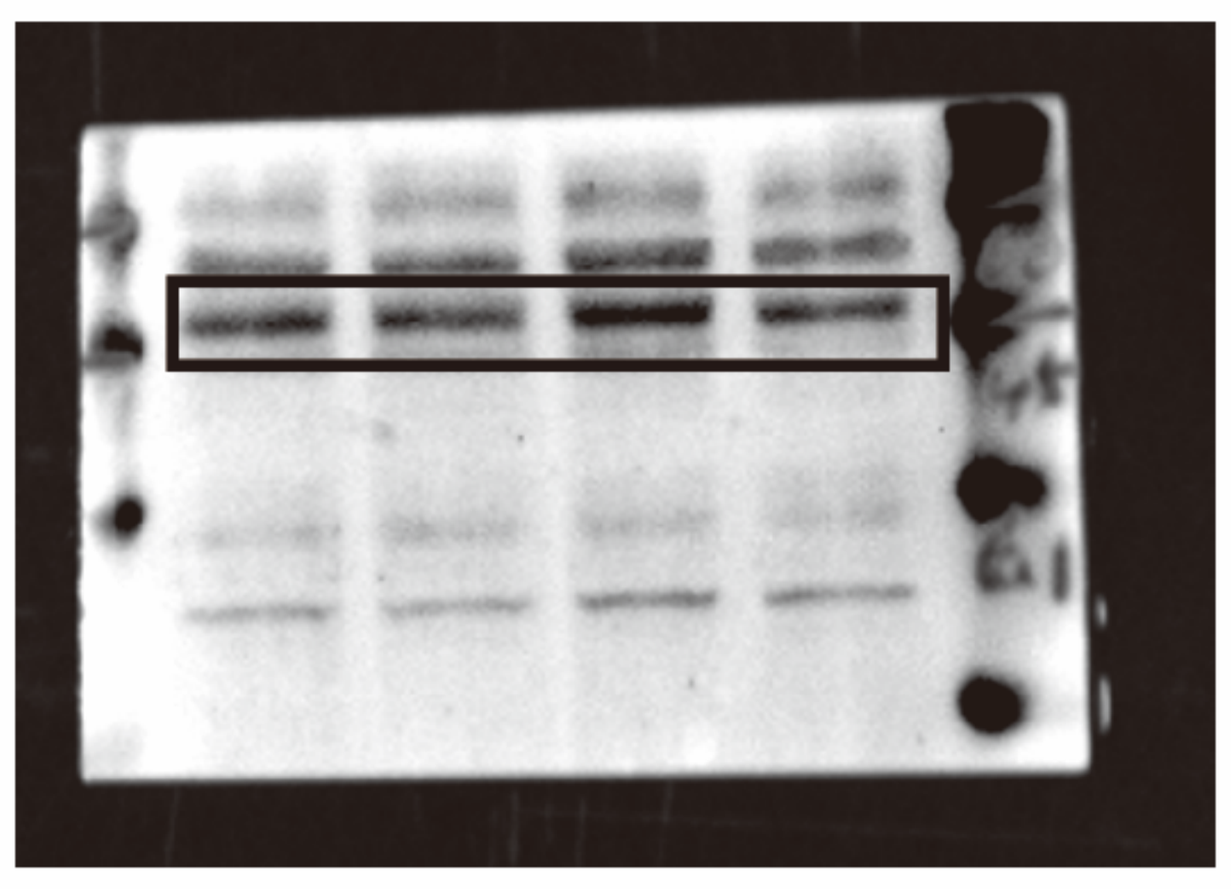

Vinculin

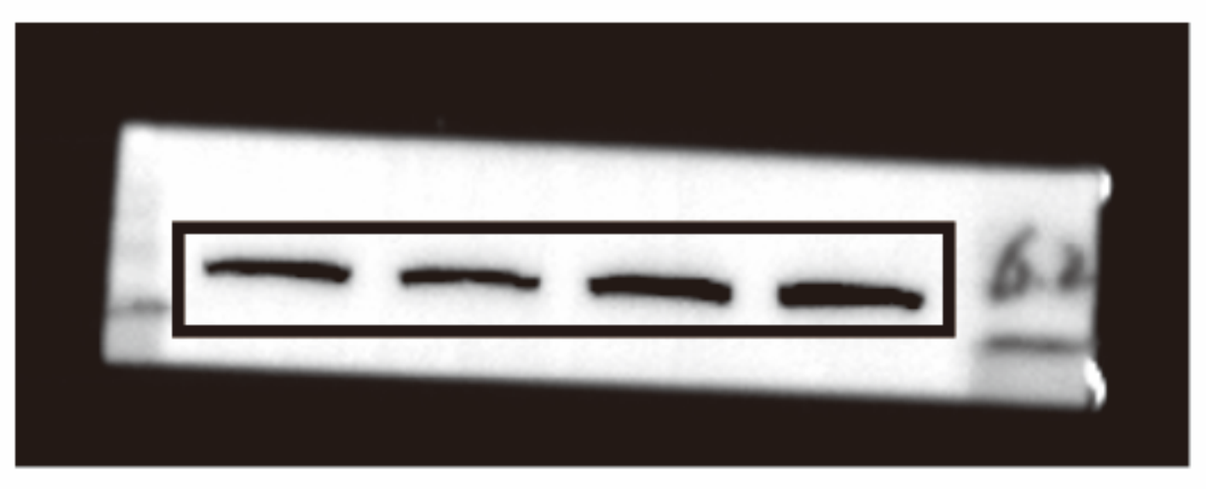

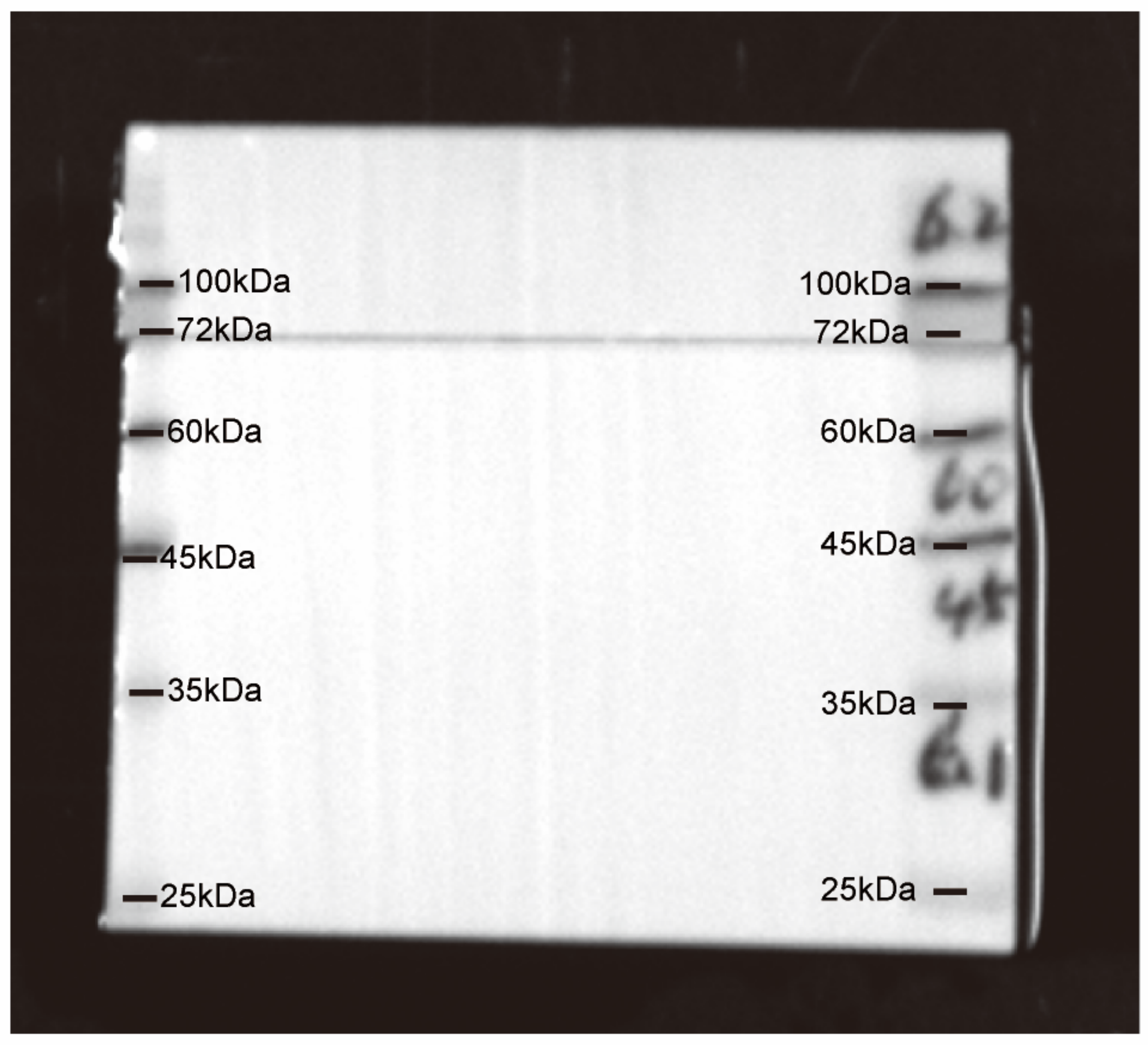

Fig.7D

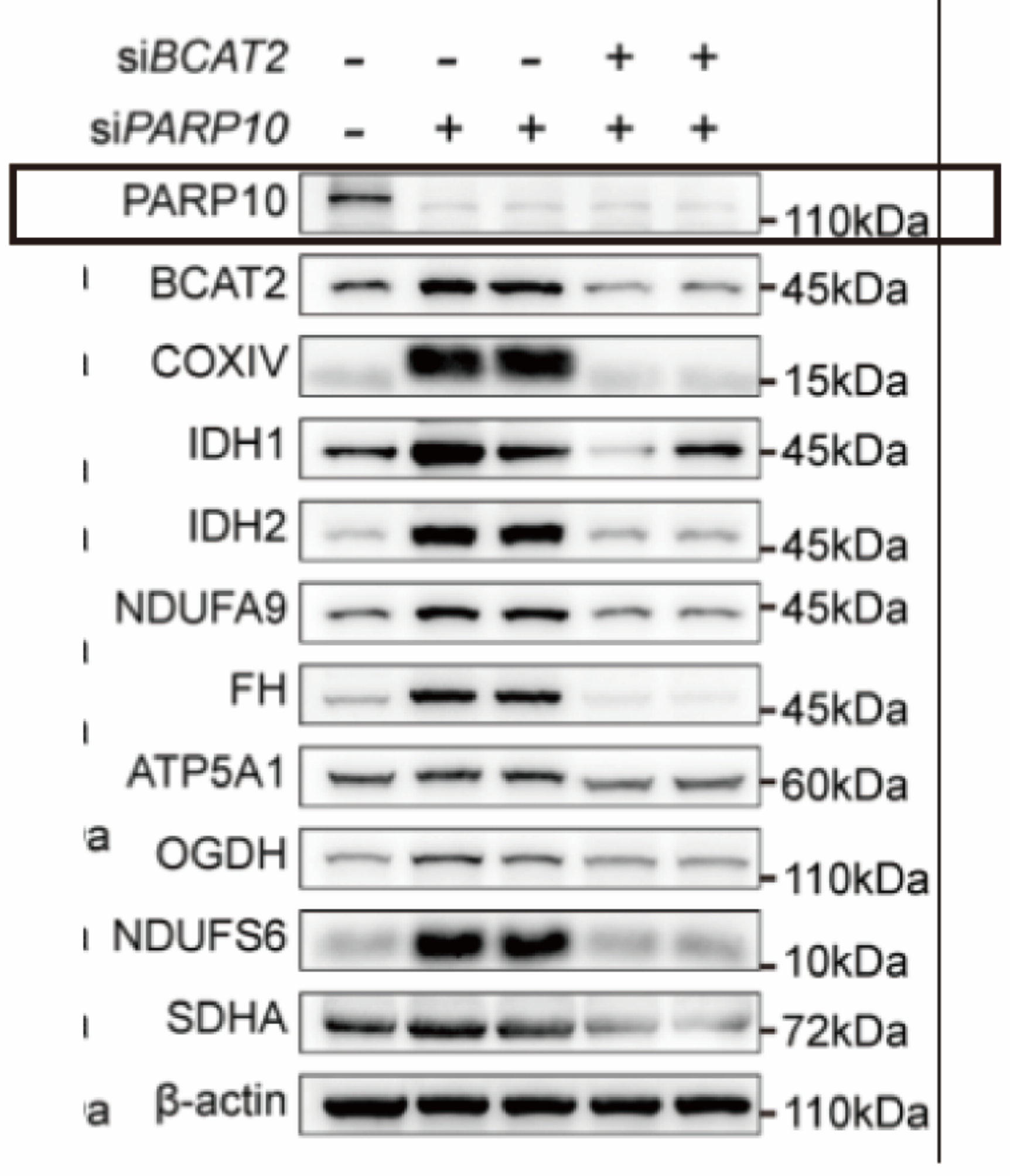

PARP10

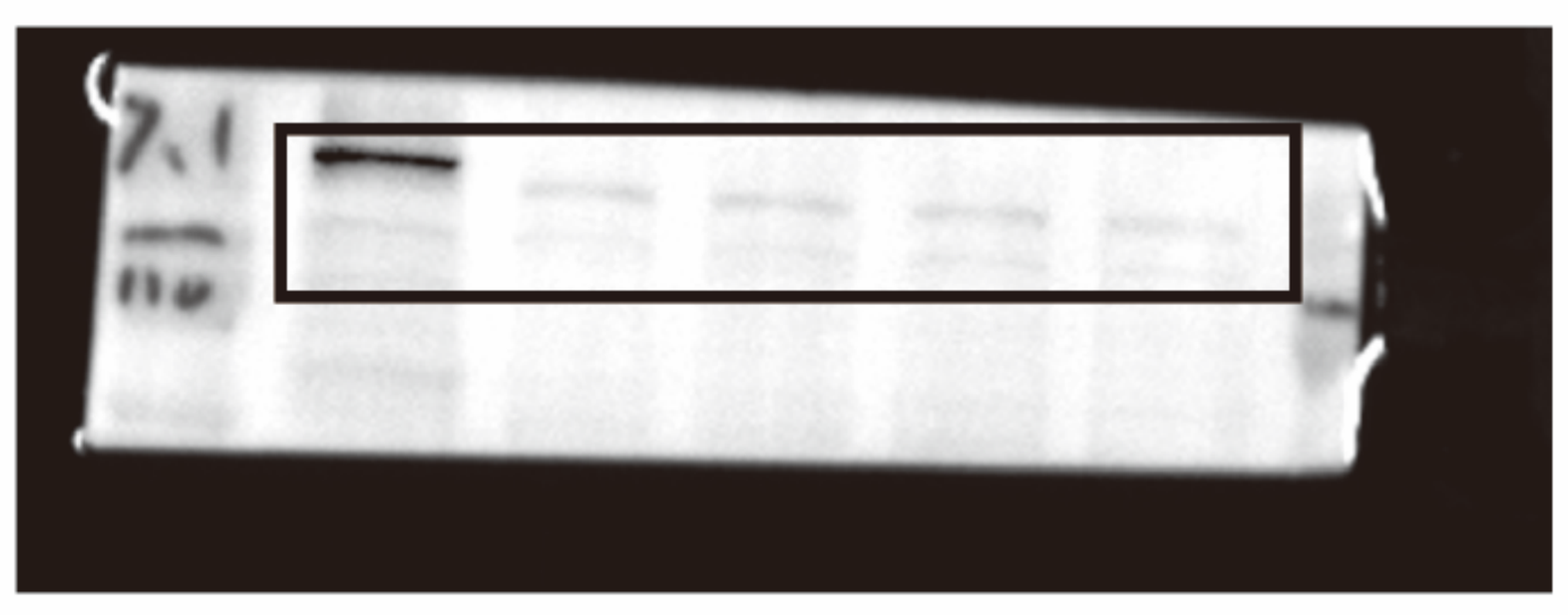

β-actin

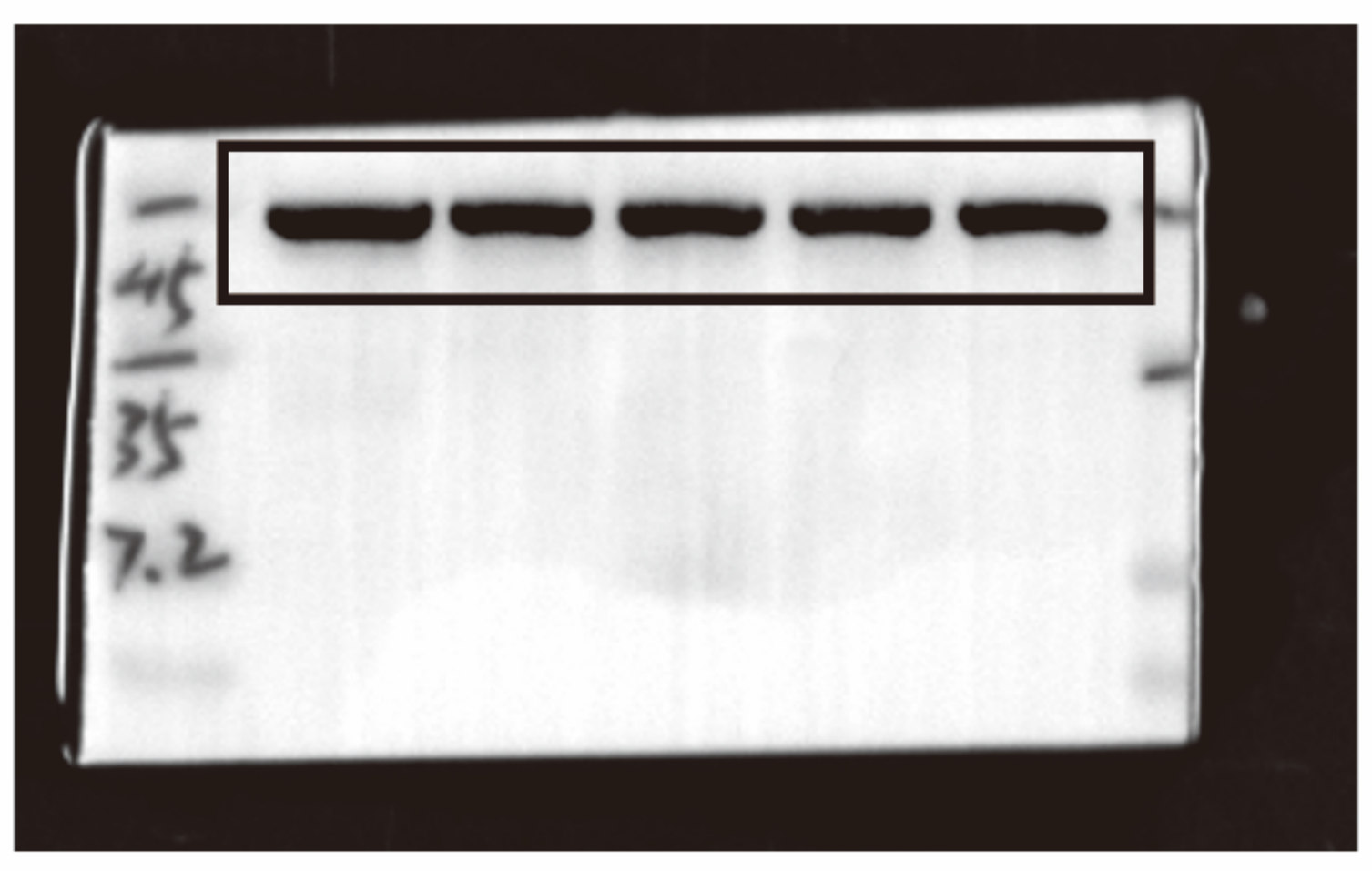

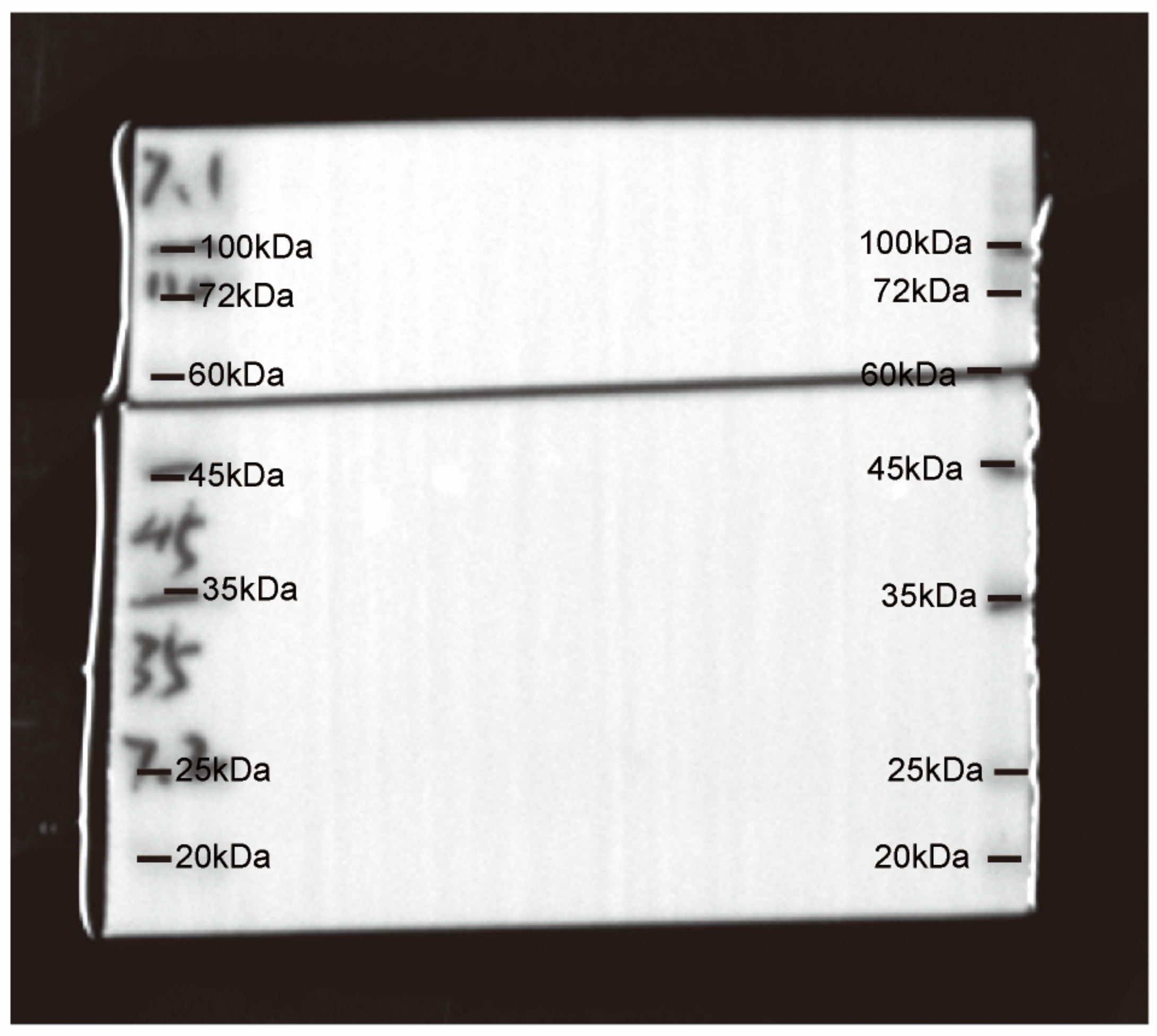

Figure	Merged	Stiched full image

Fig.7D ’

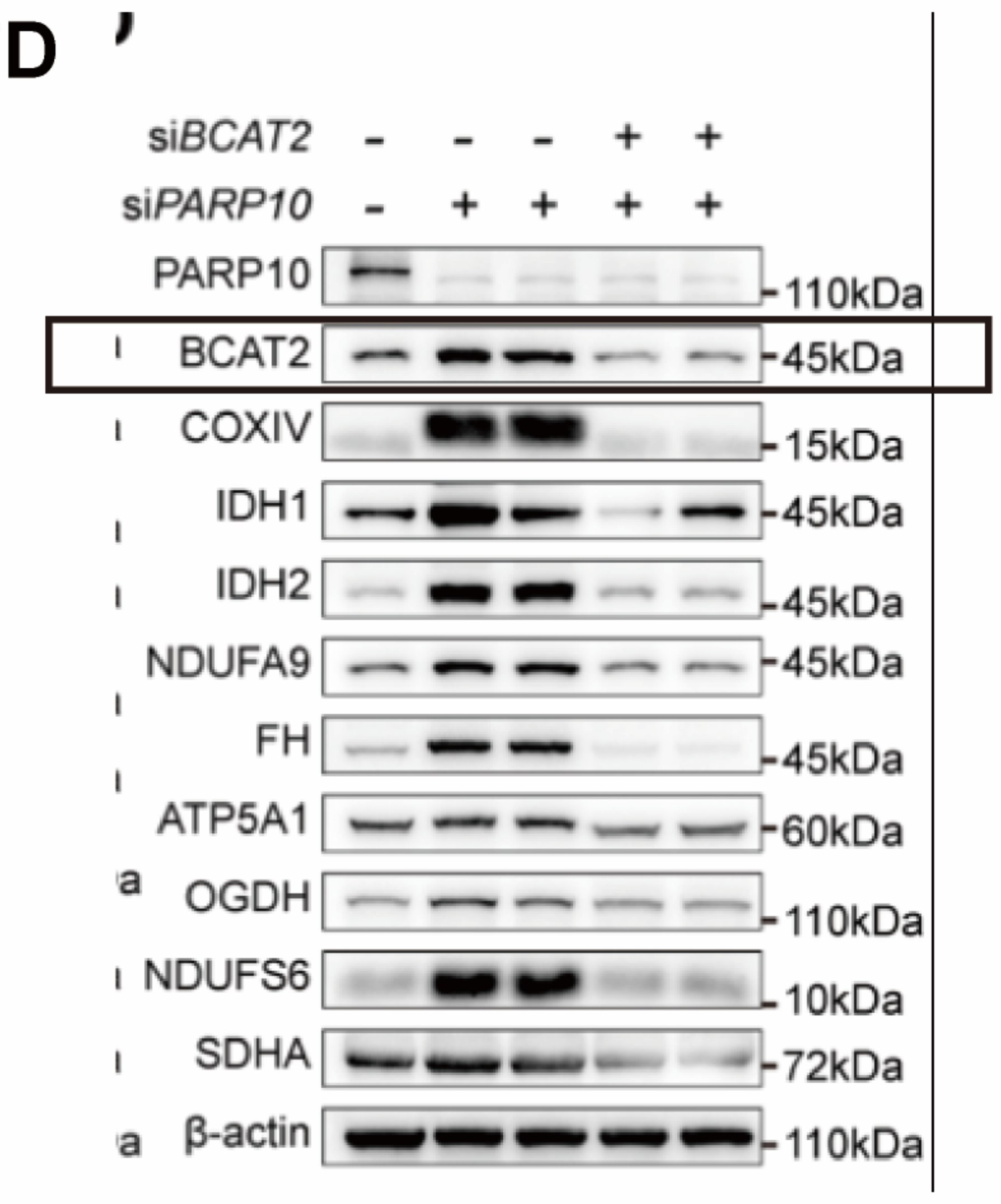

BCAT2

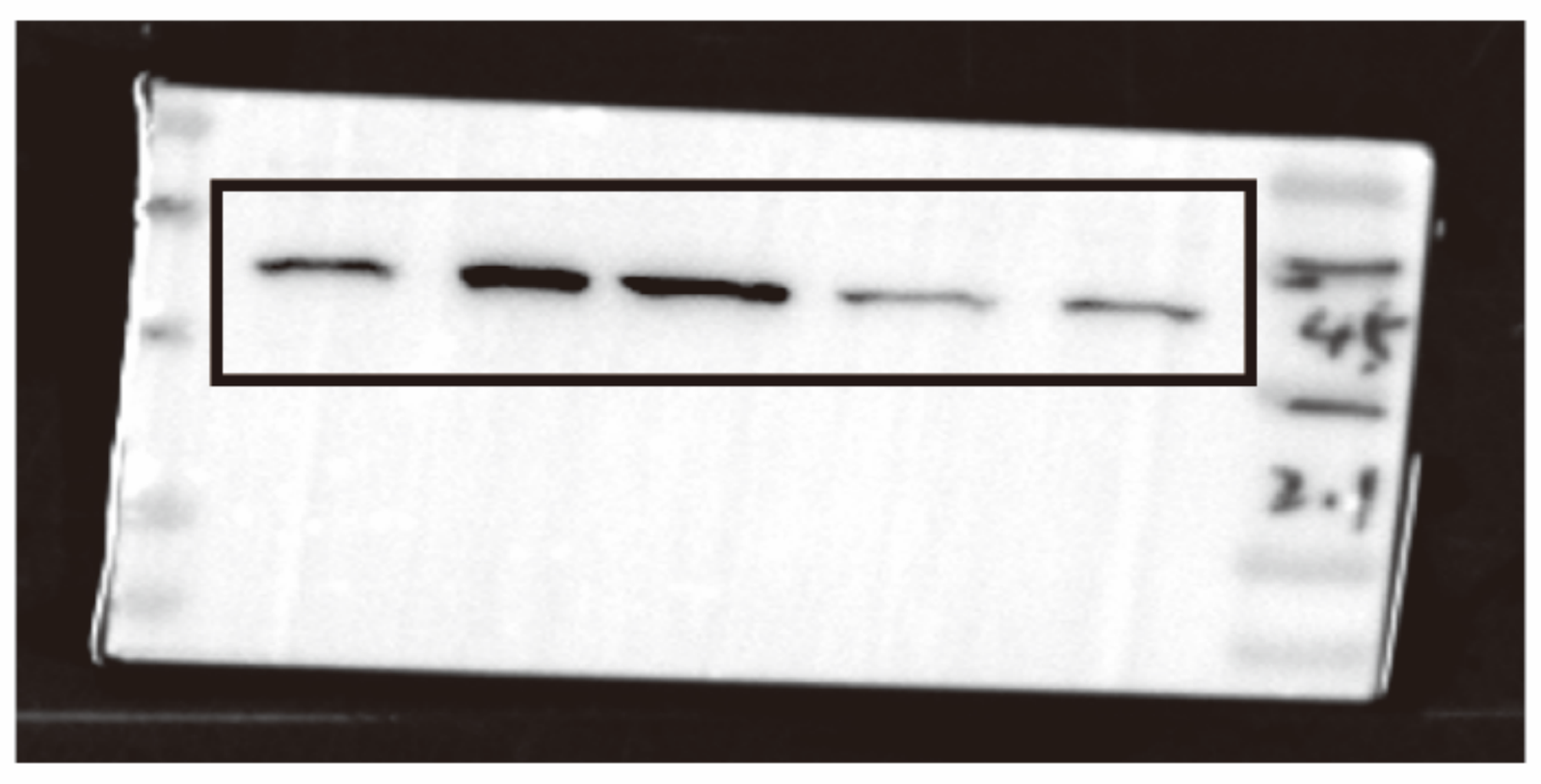

β-actin

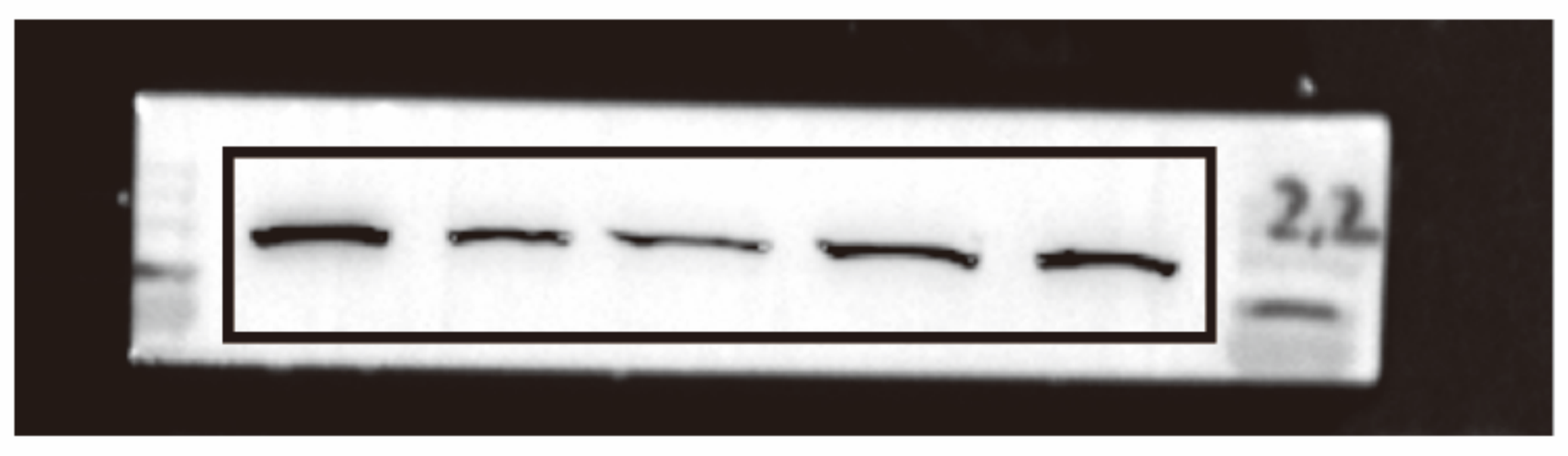

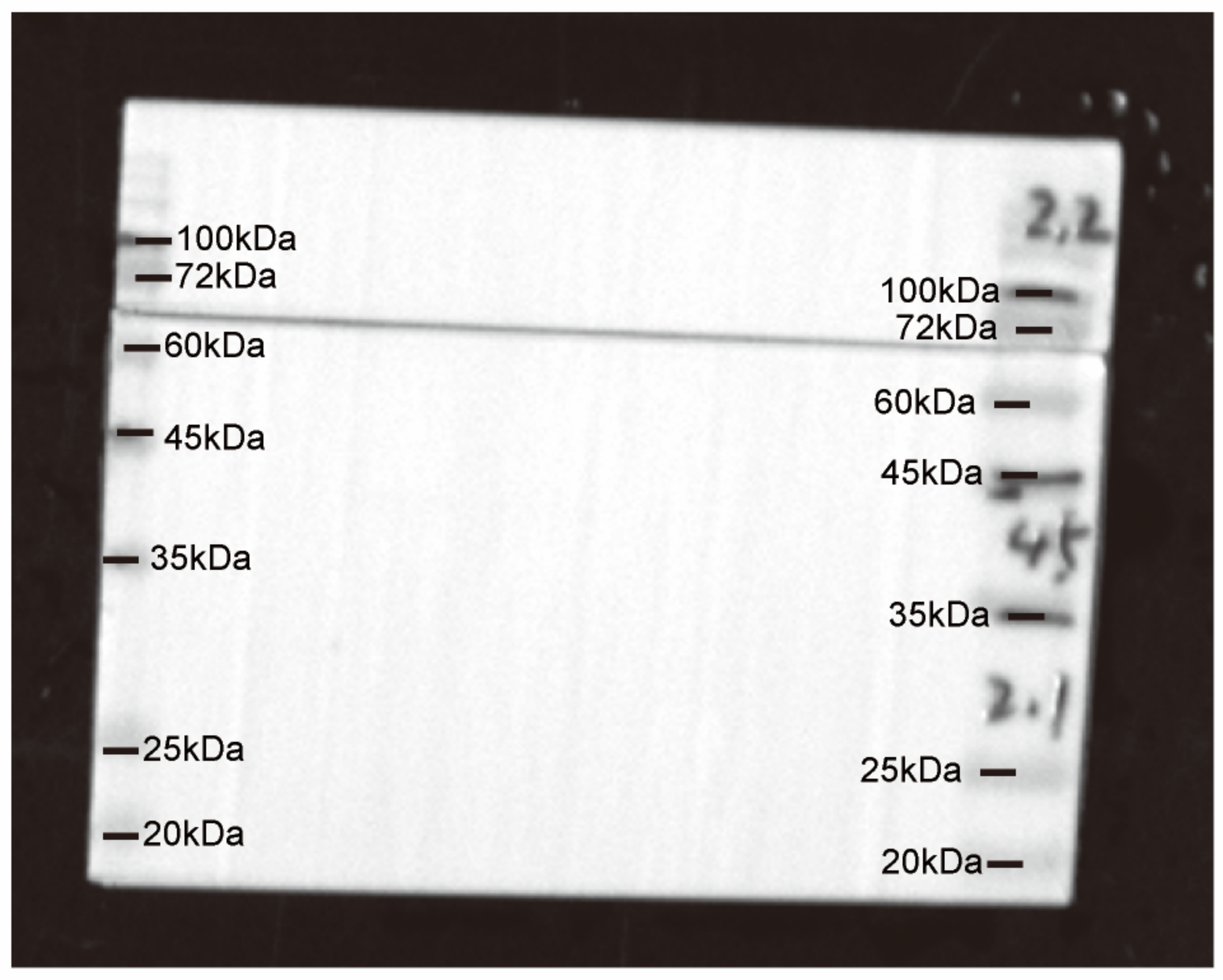

Fig.7D ’

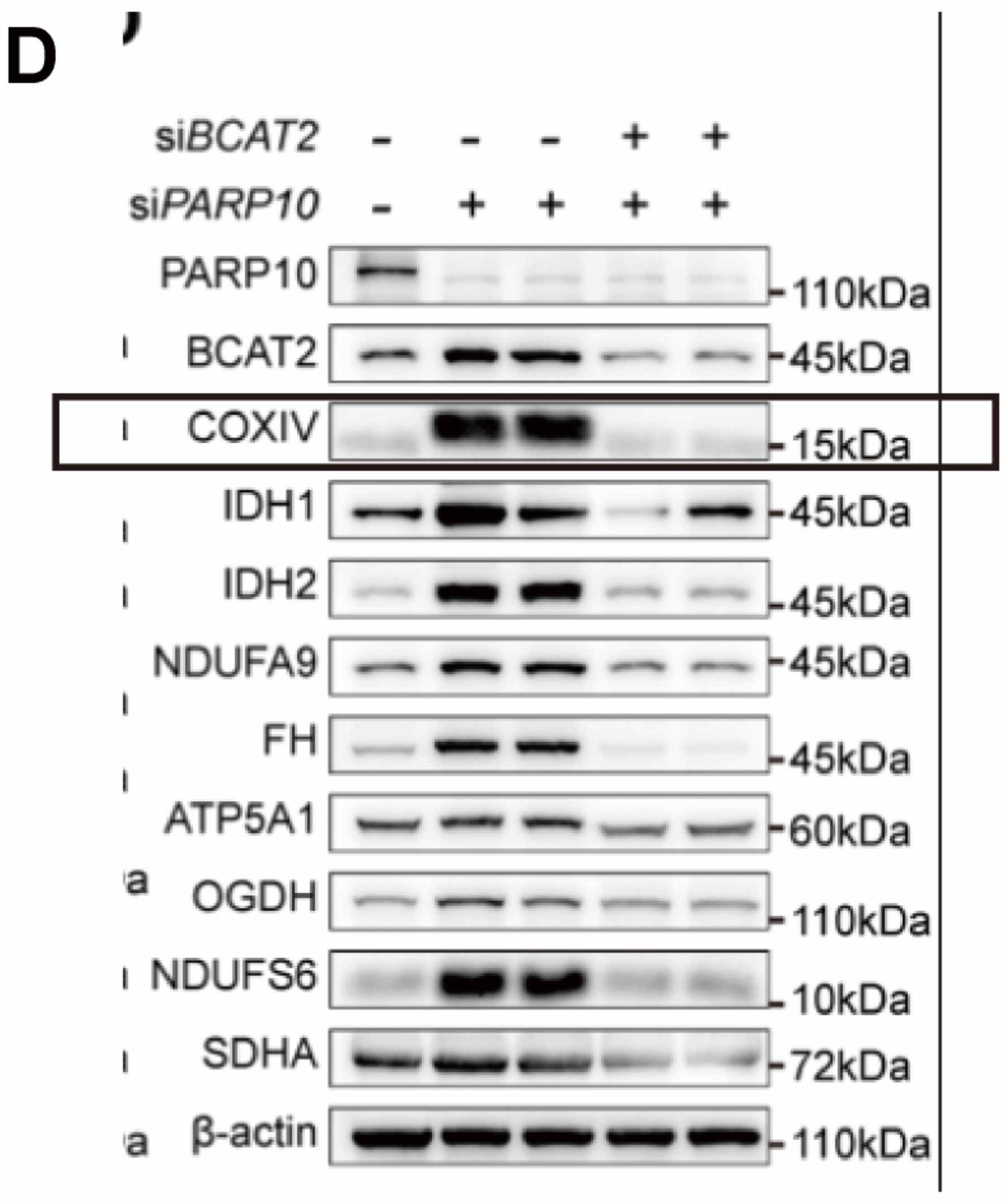

COXIV

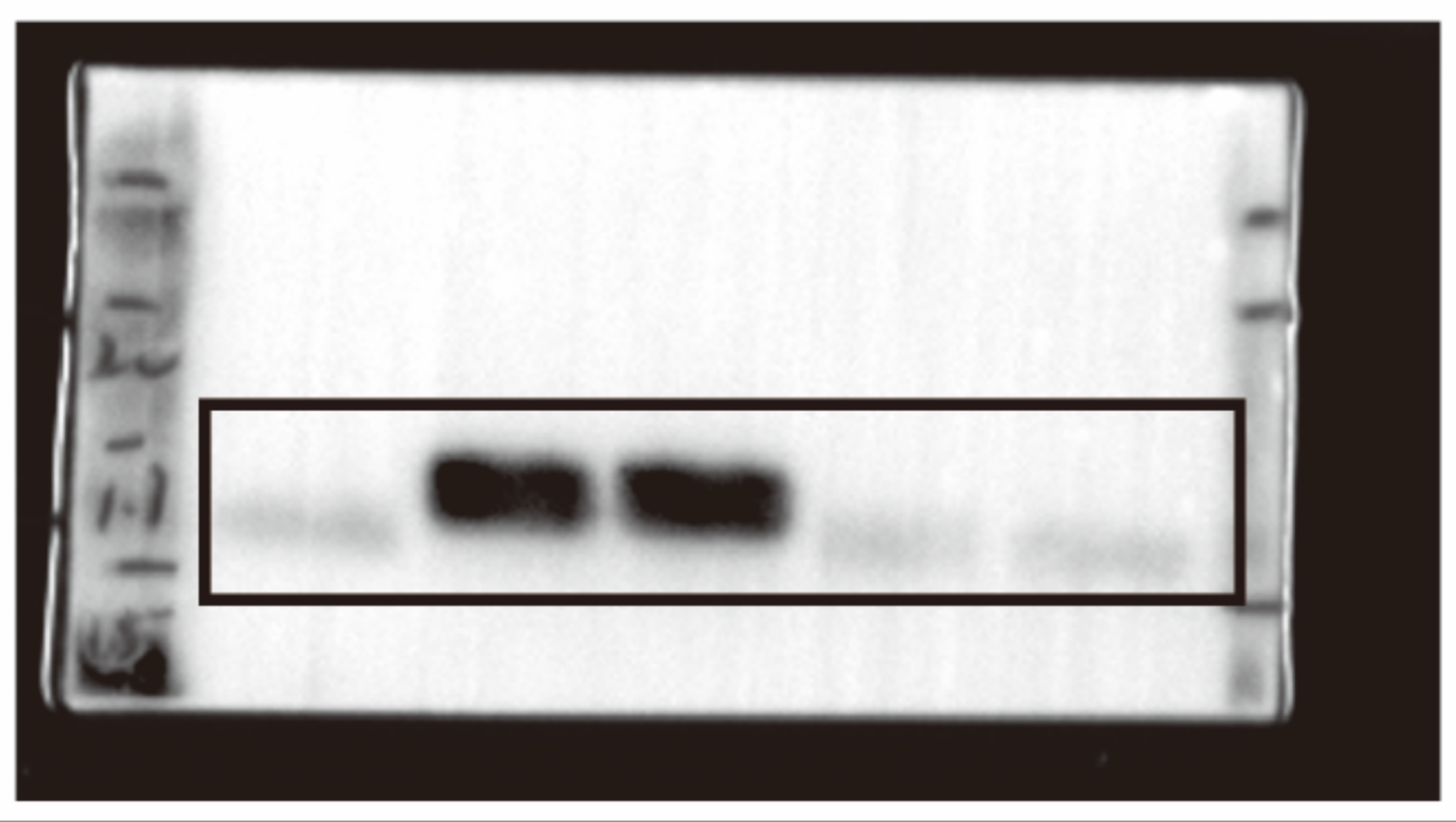

Vinculin

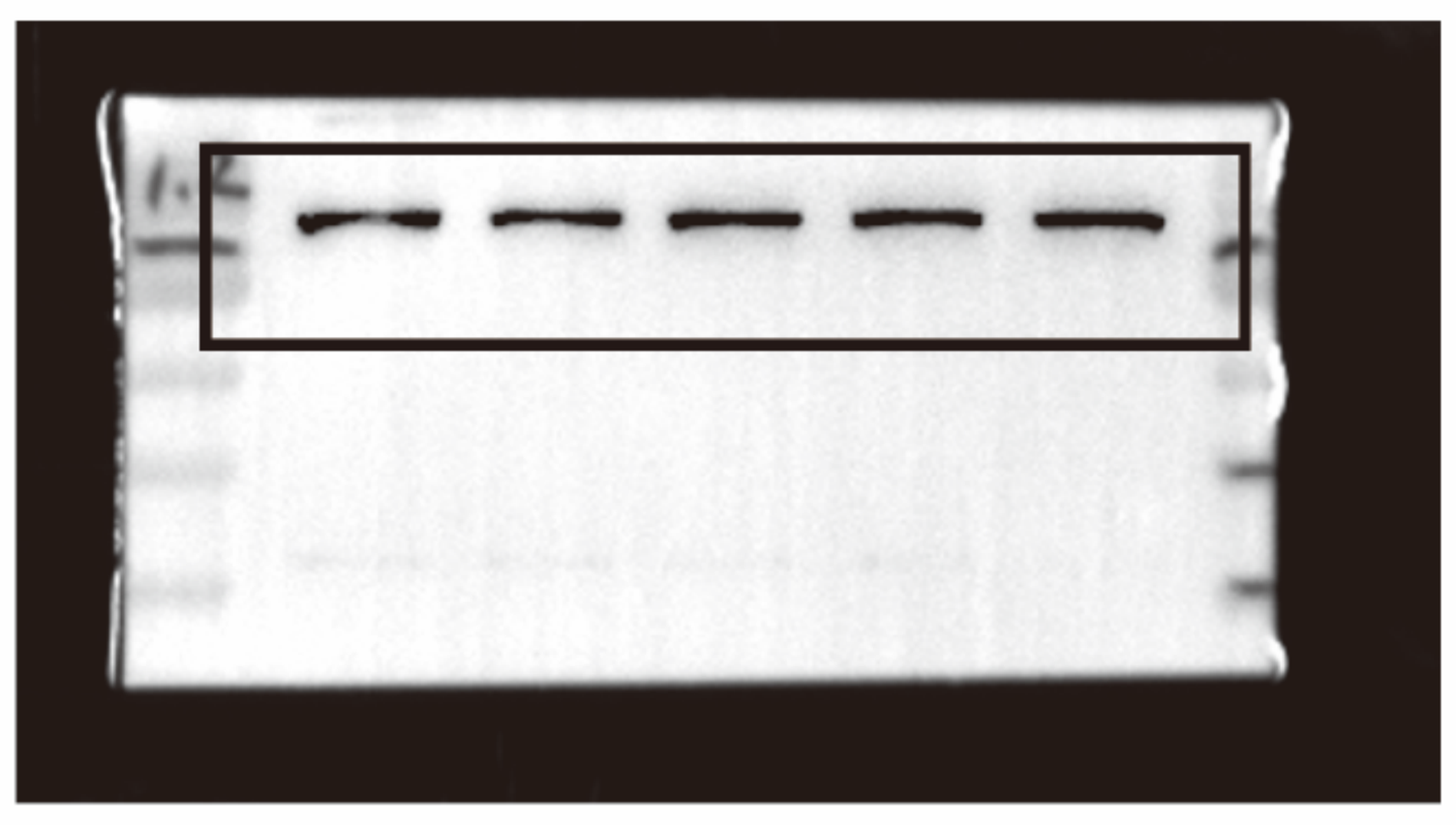

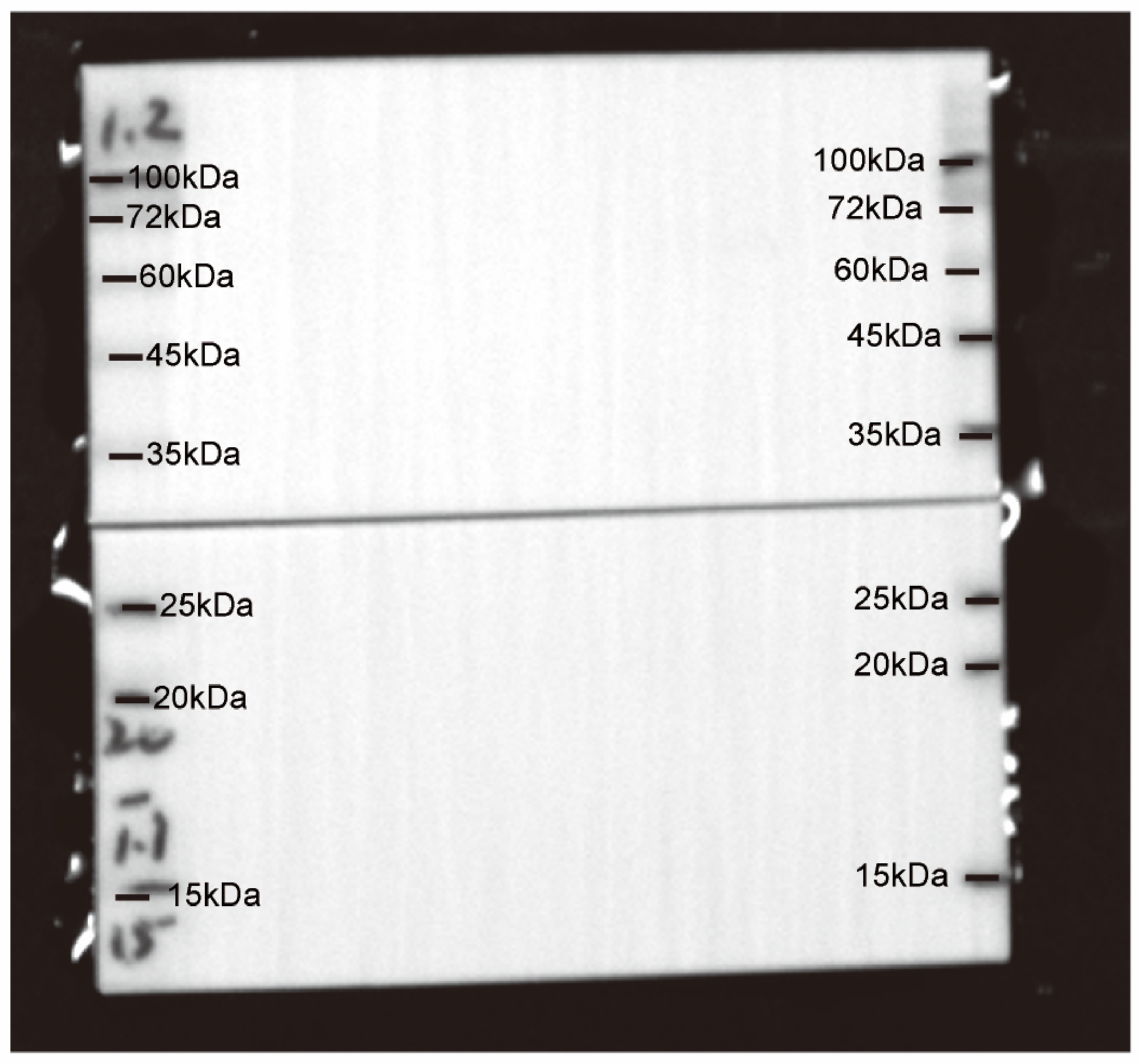

Fig.7D ’

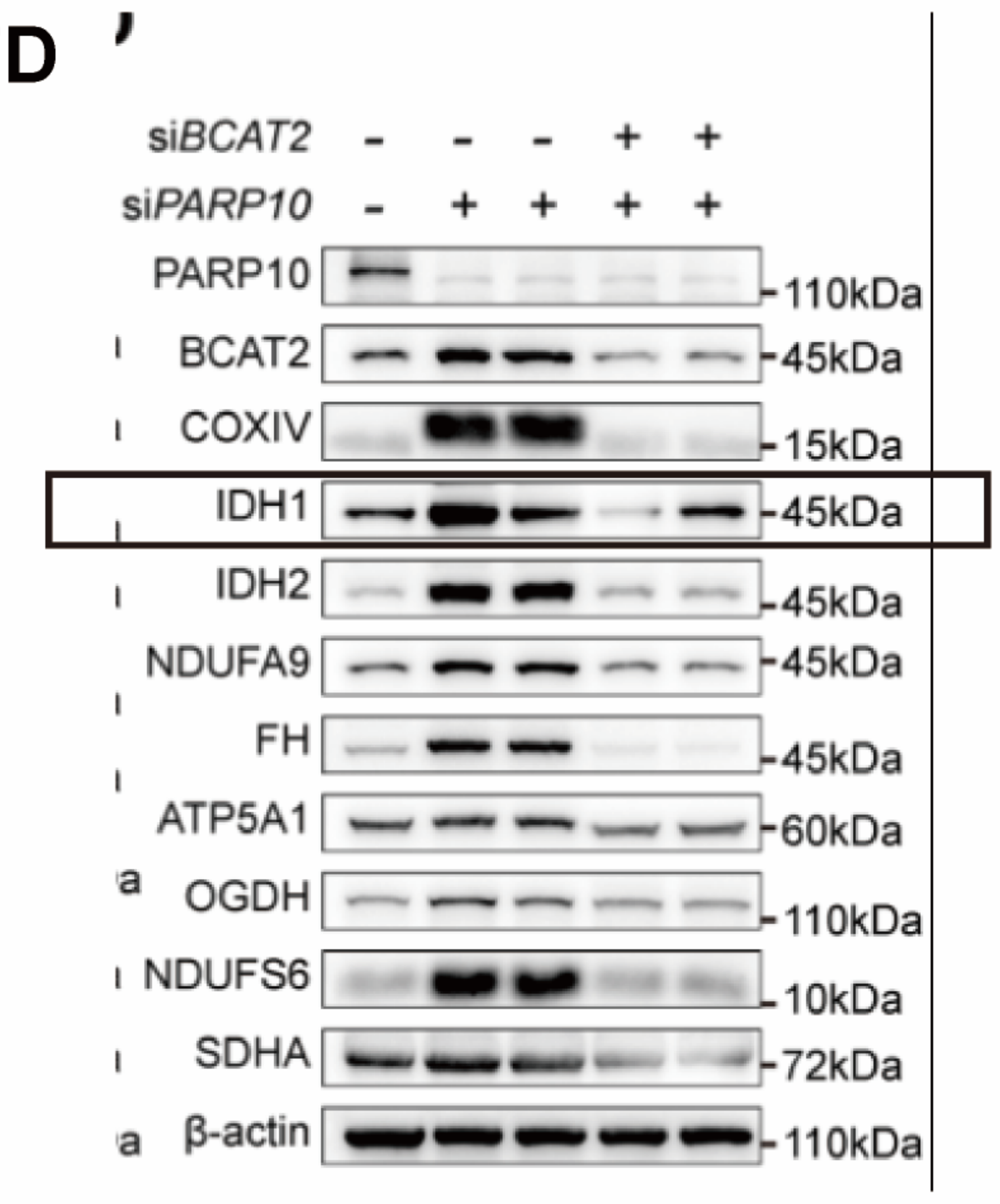

IDH1

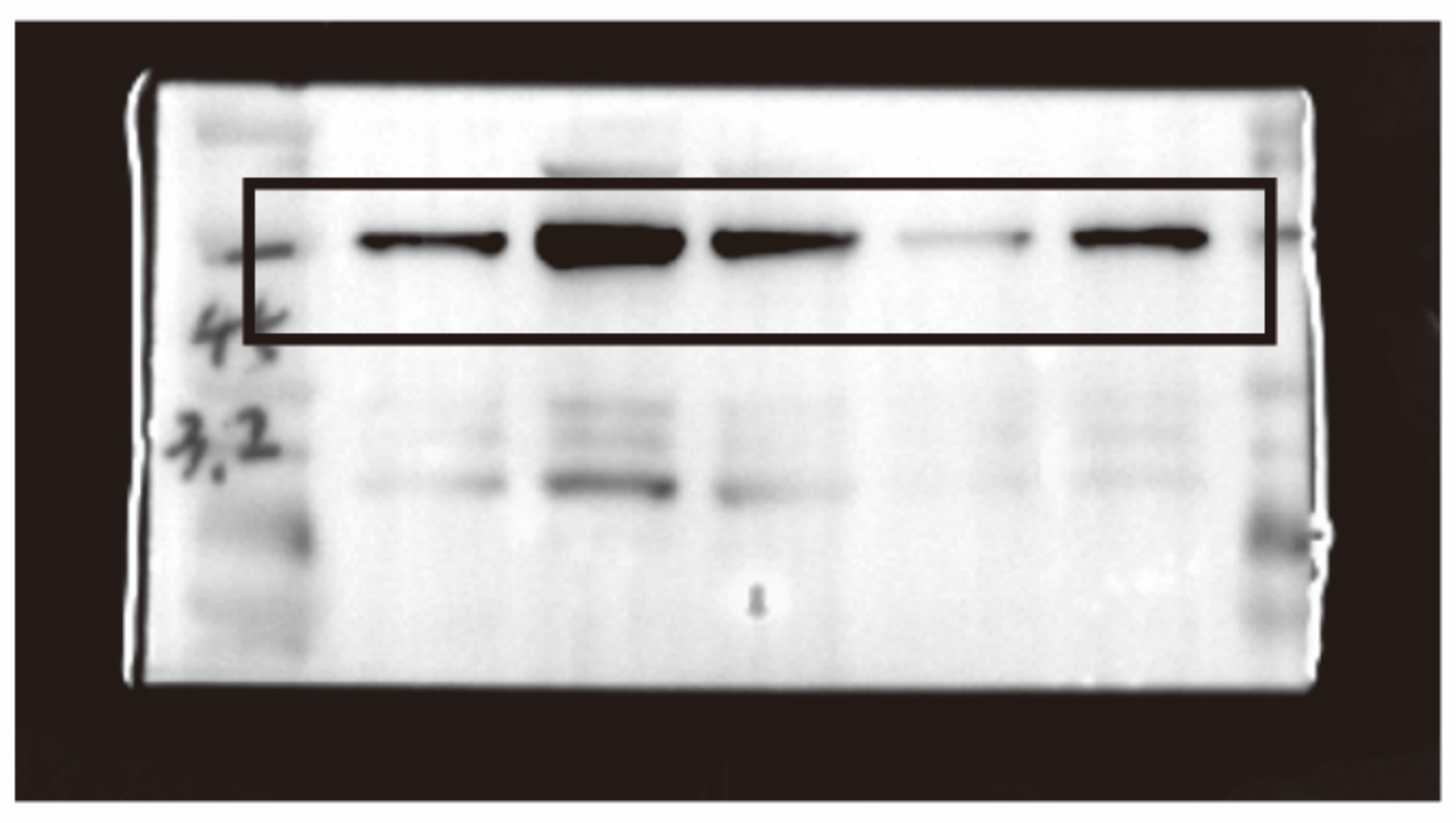

Vinculin

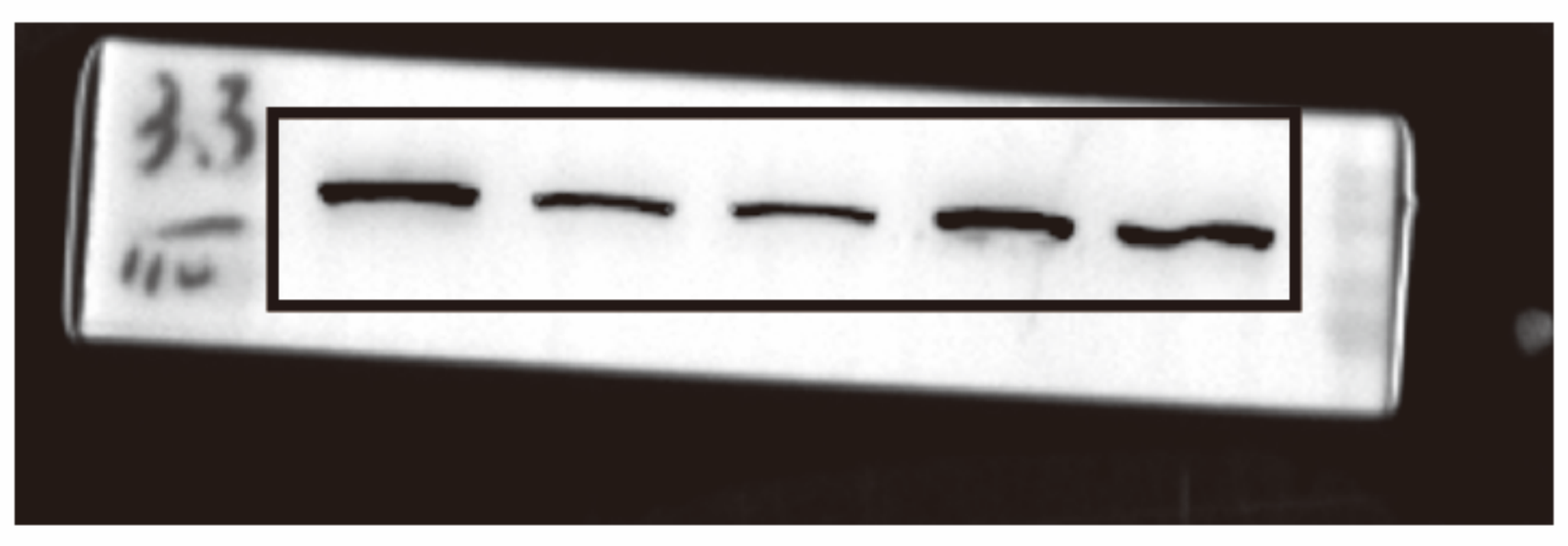

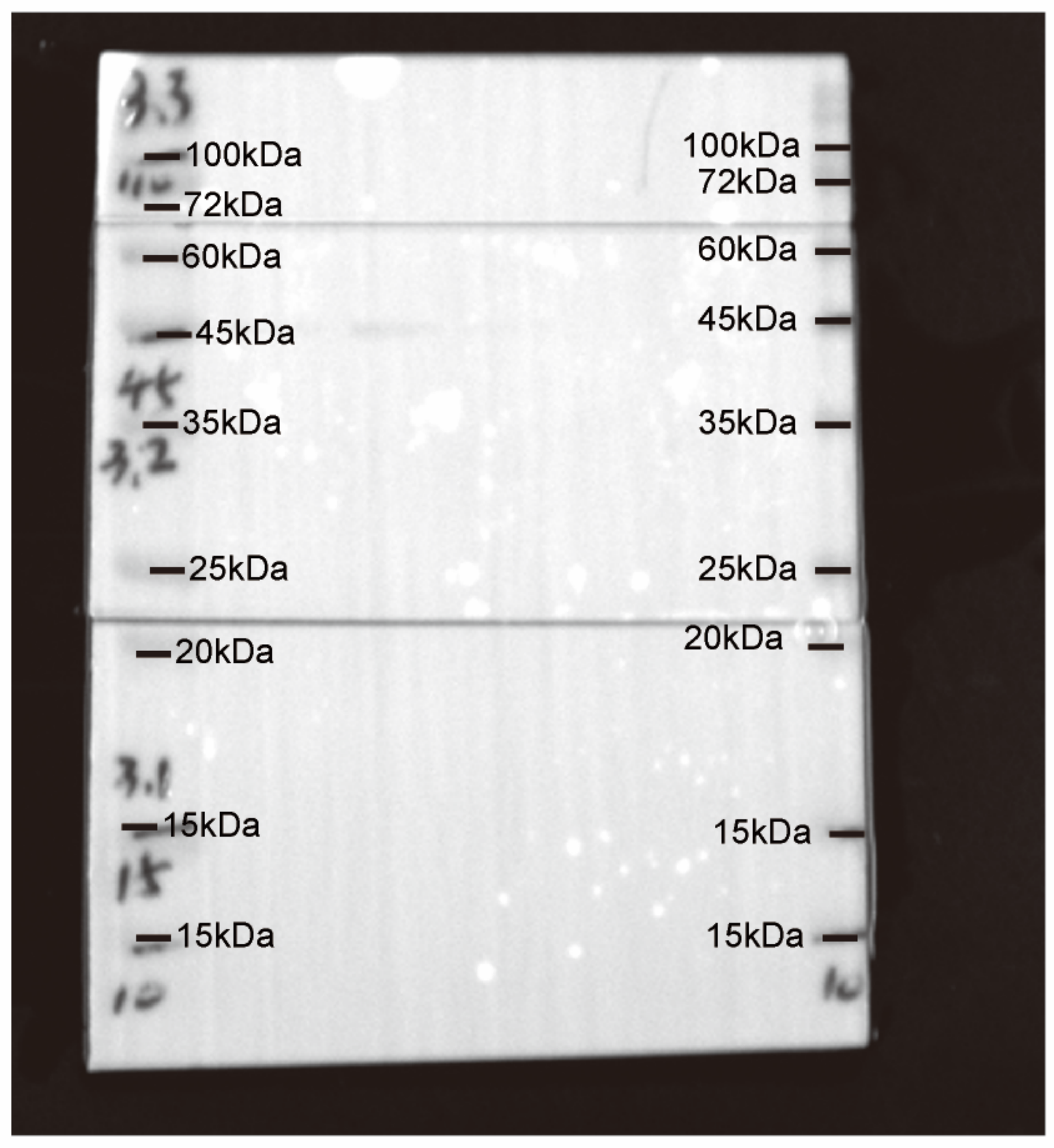

Fig.7D ’

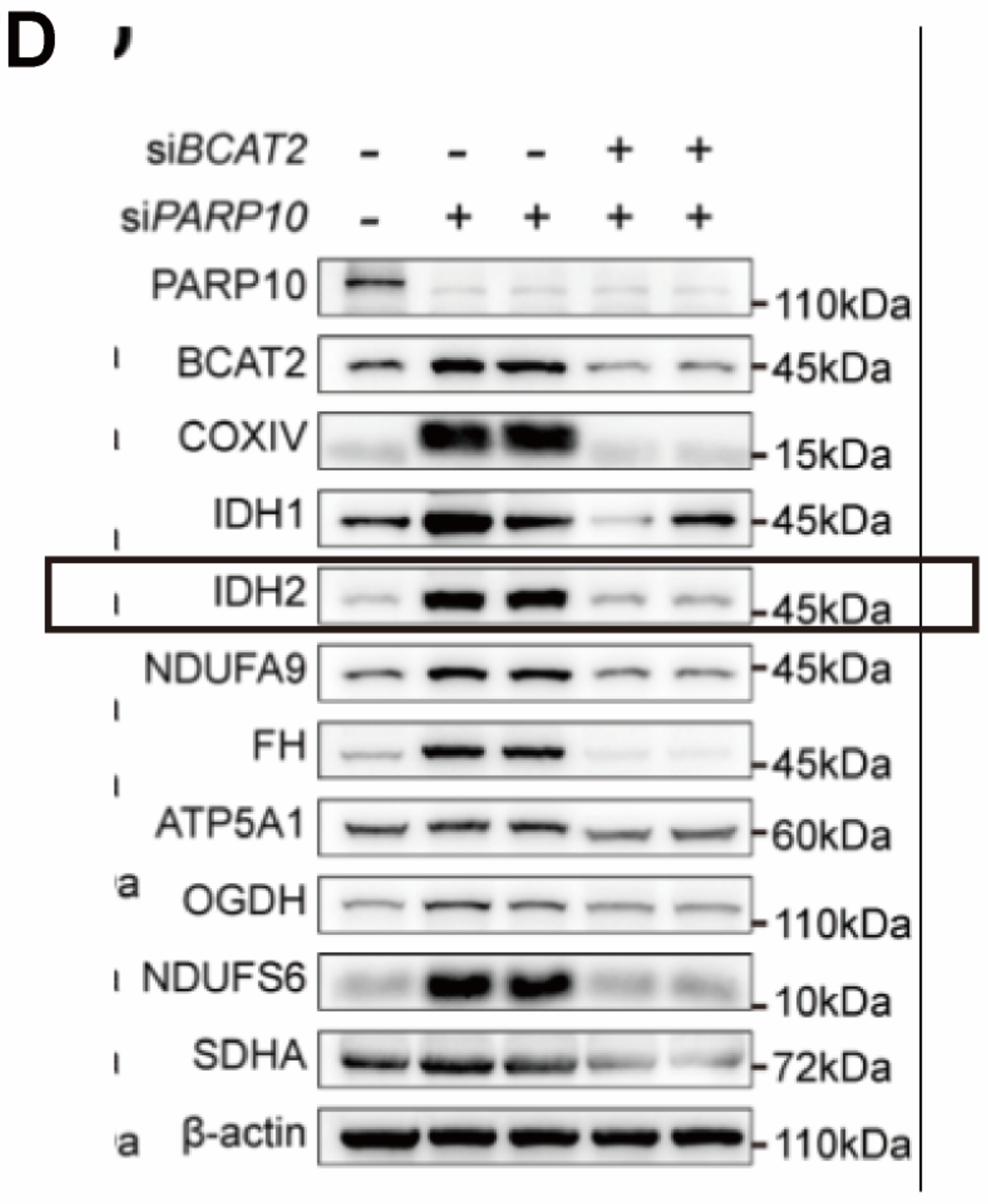

IDH2

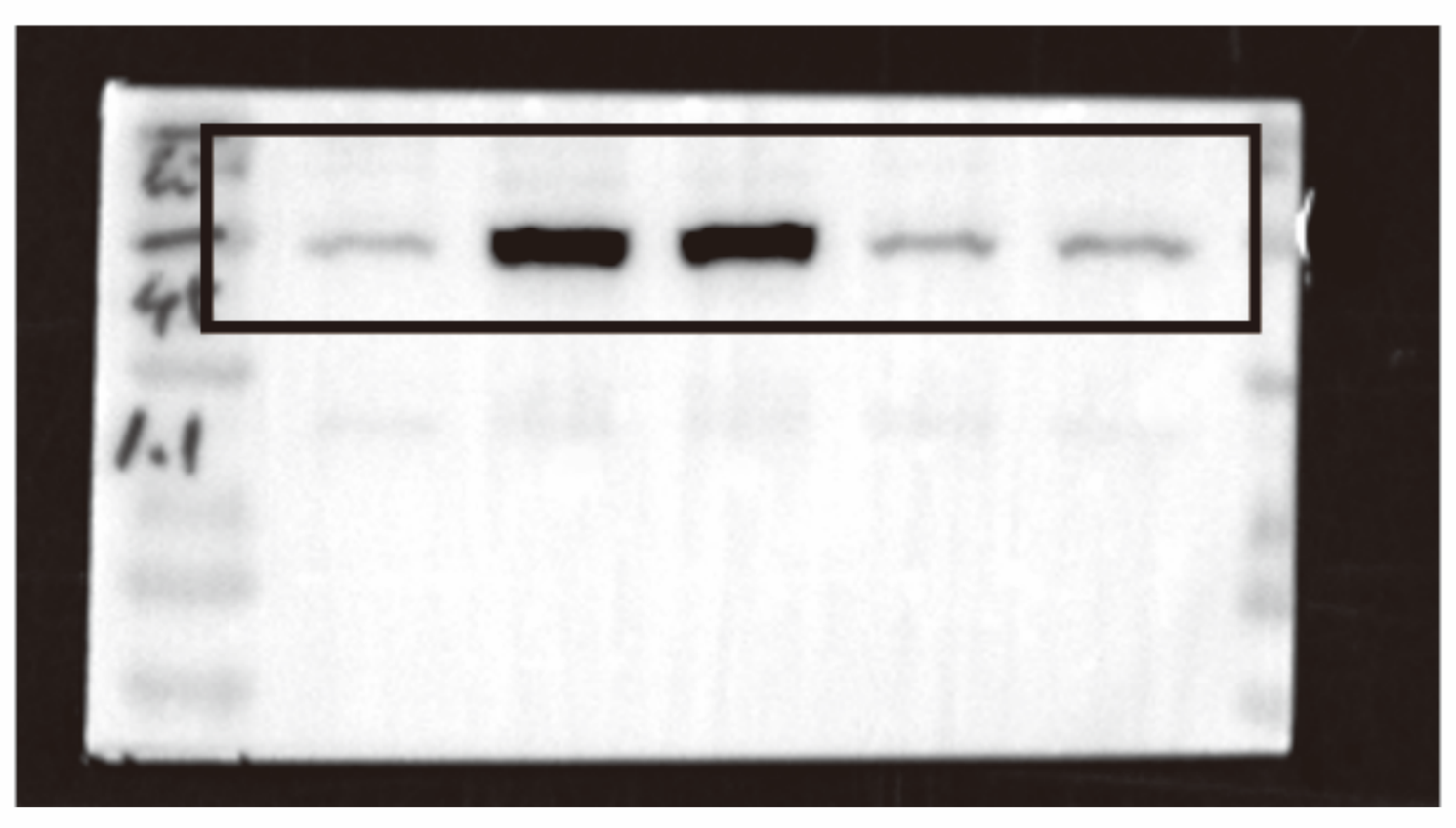

Vinculin

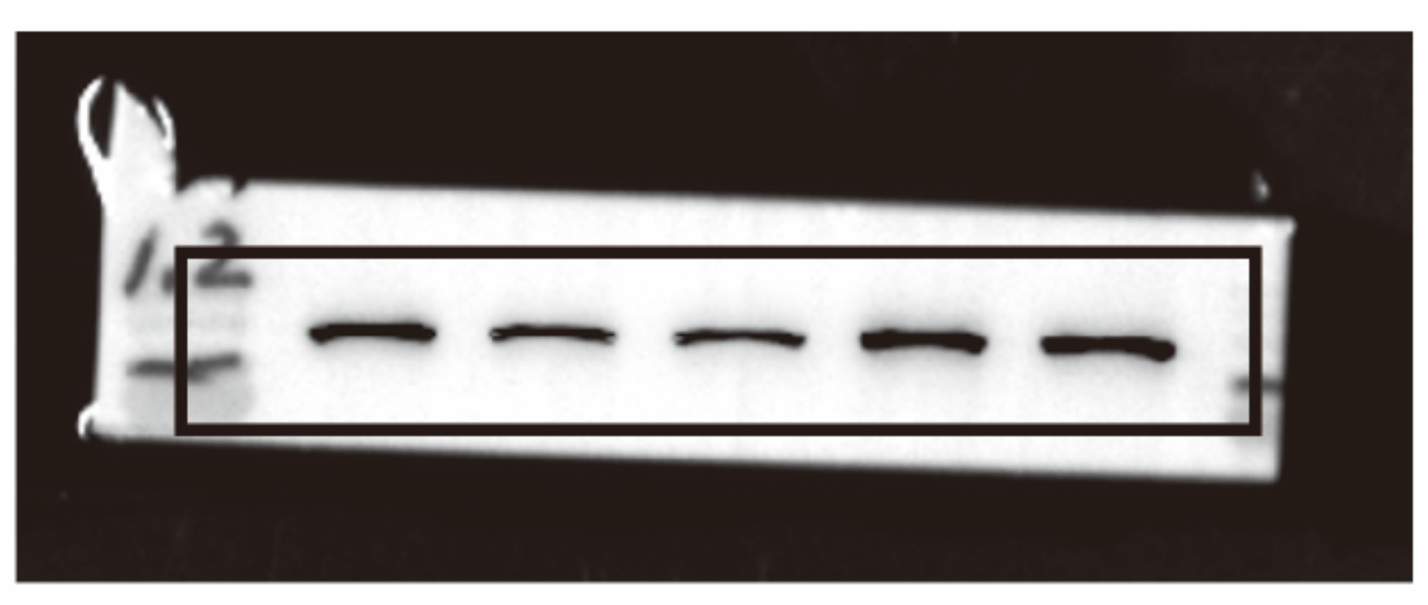

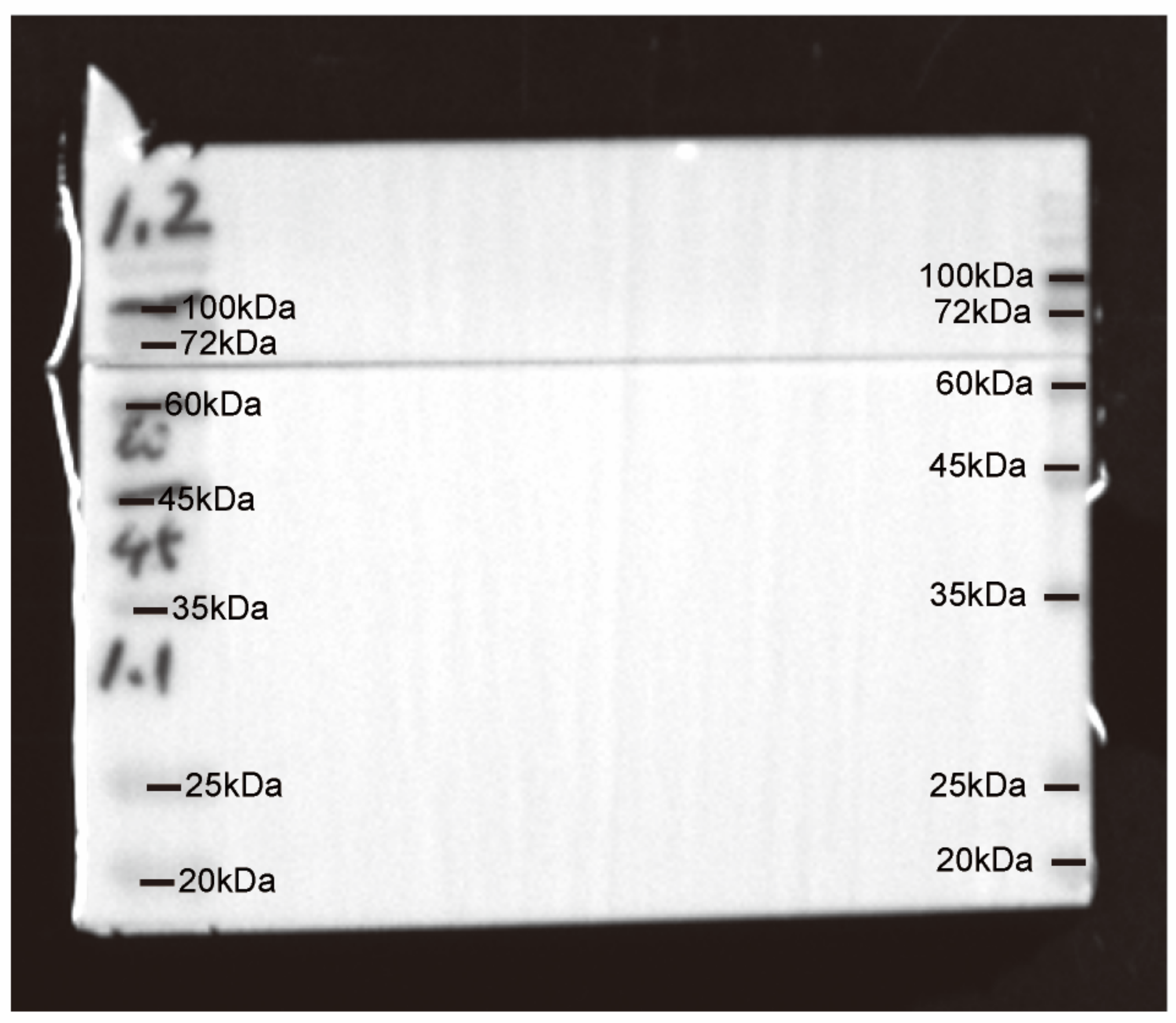

Figure	Merged	Stiched full image

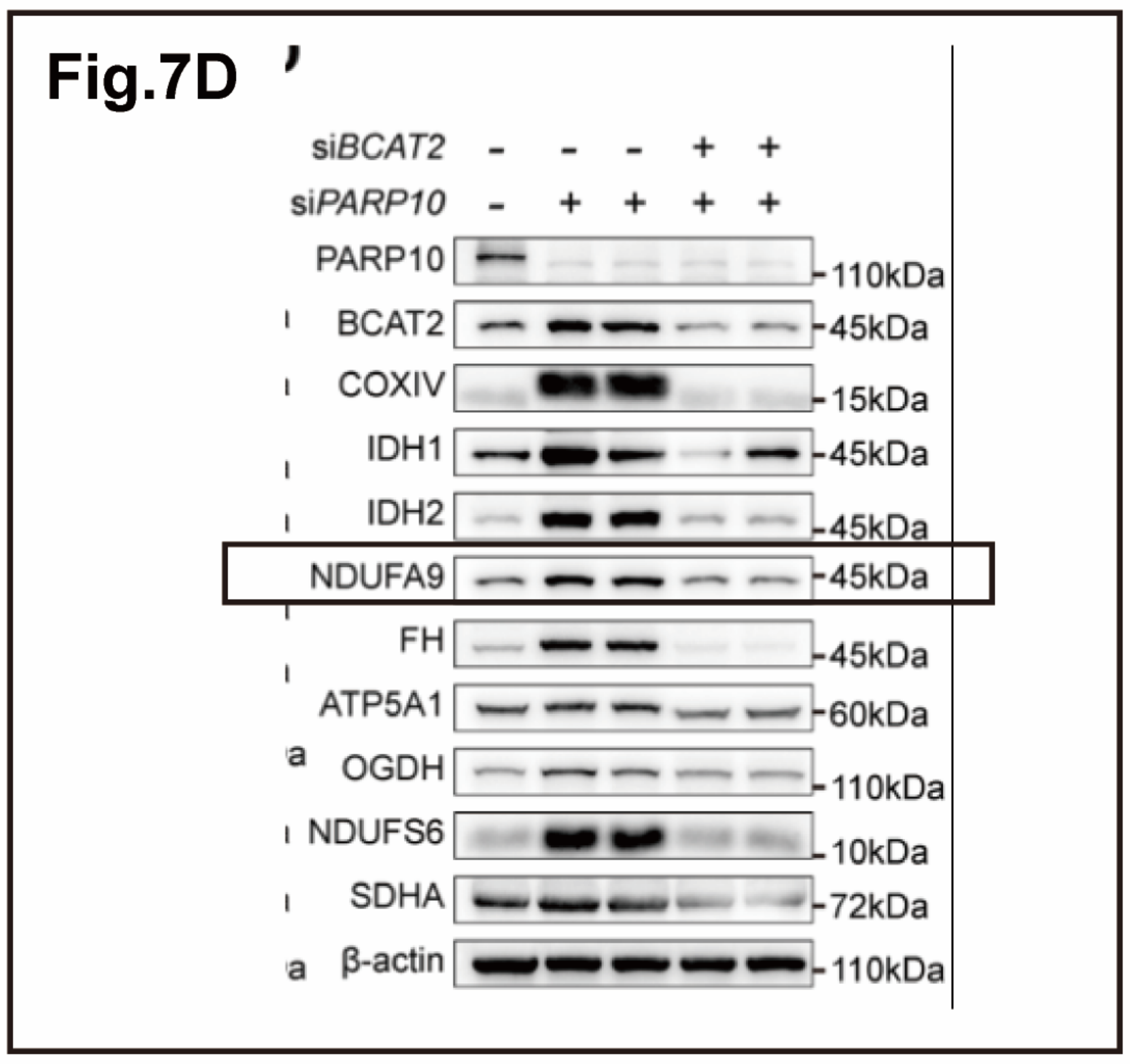

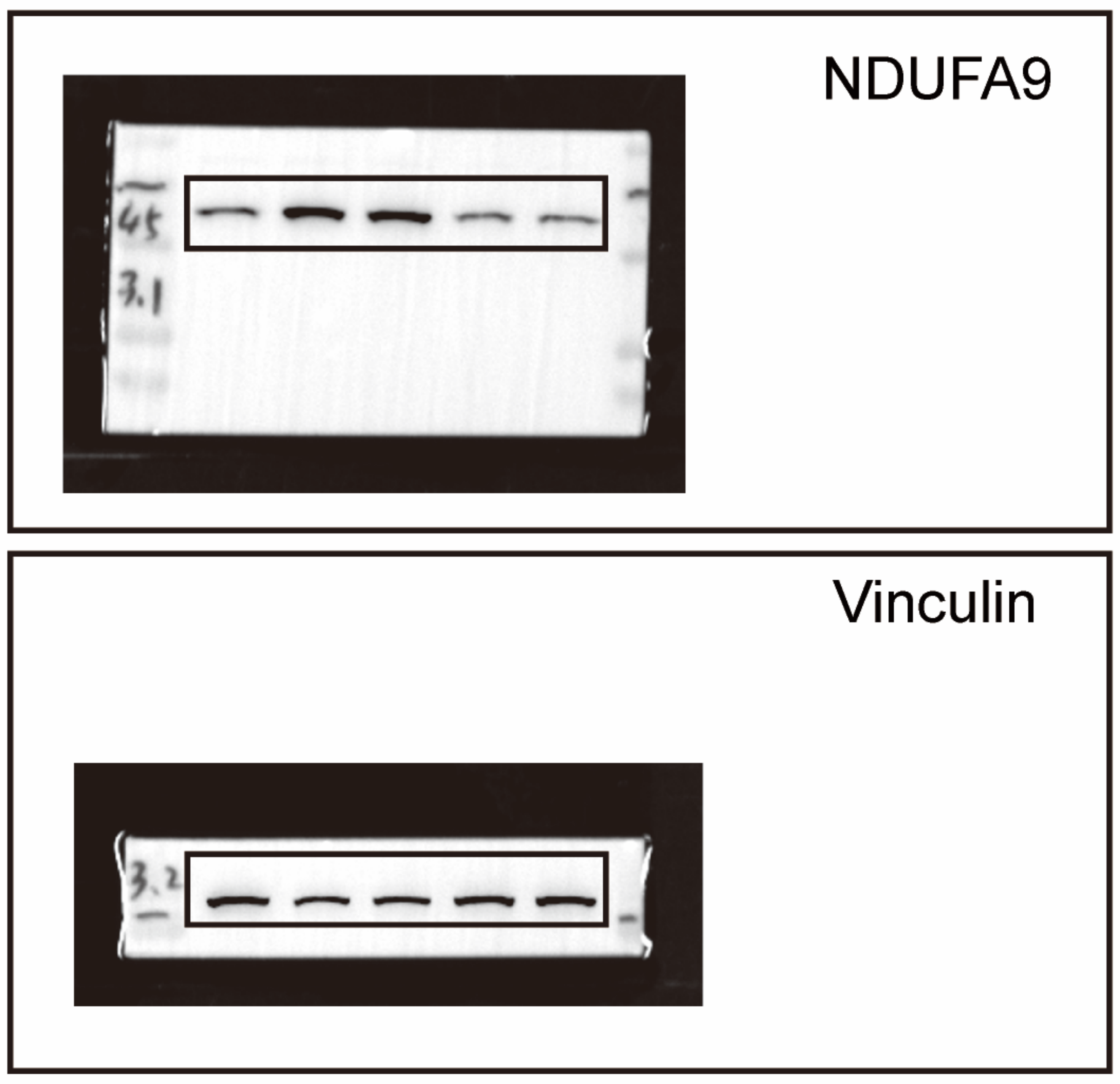

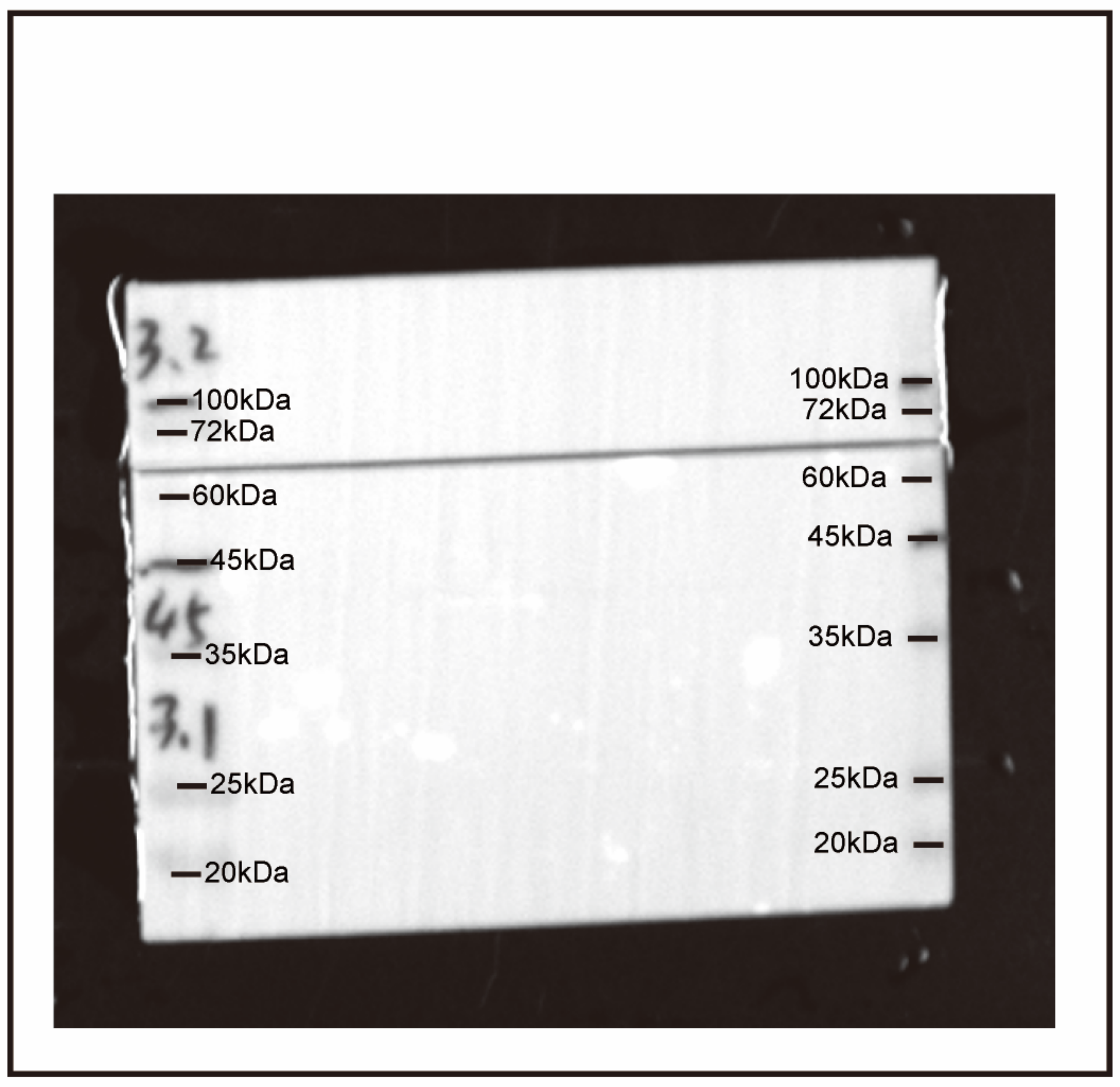

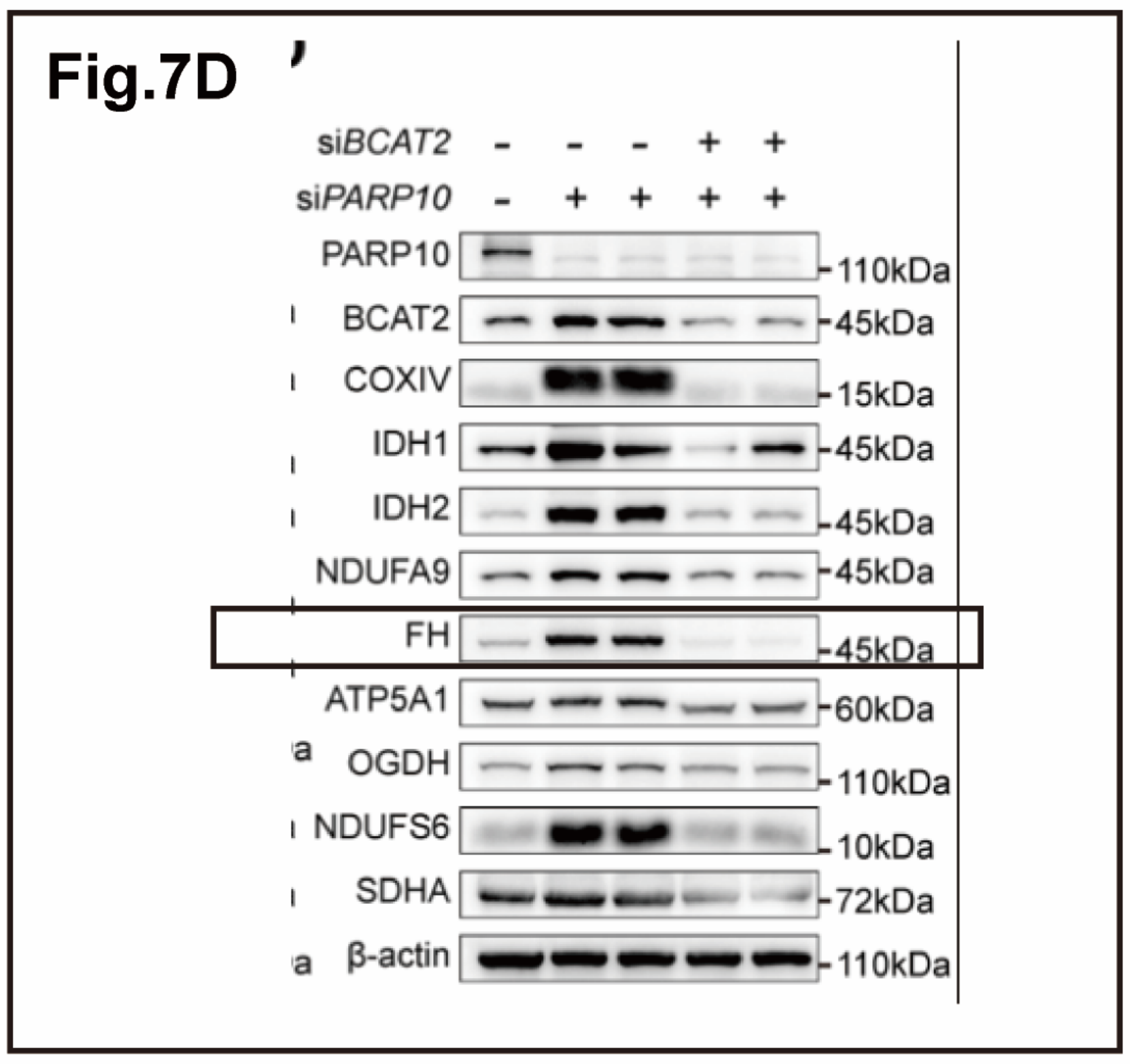

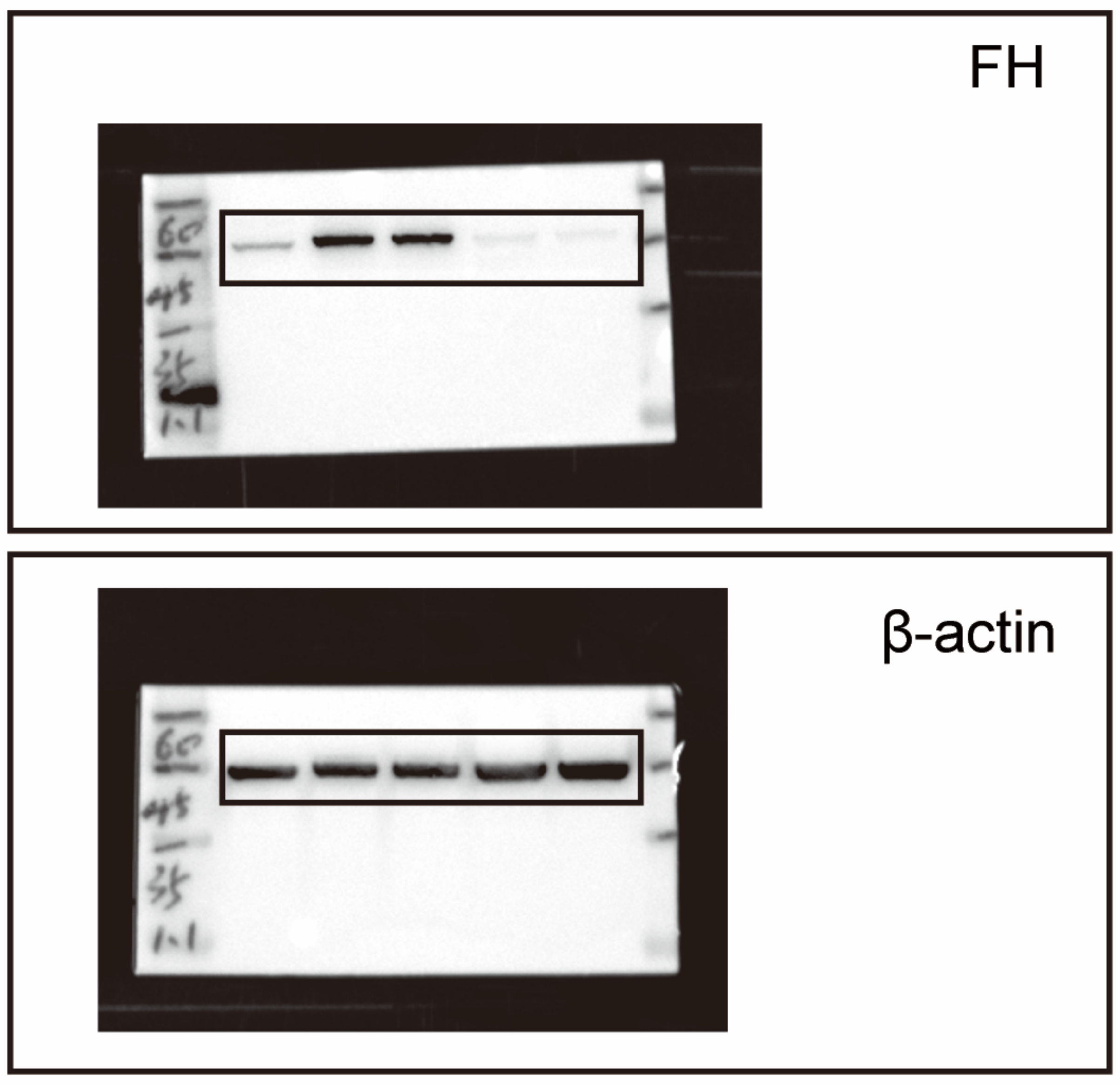

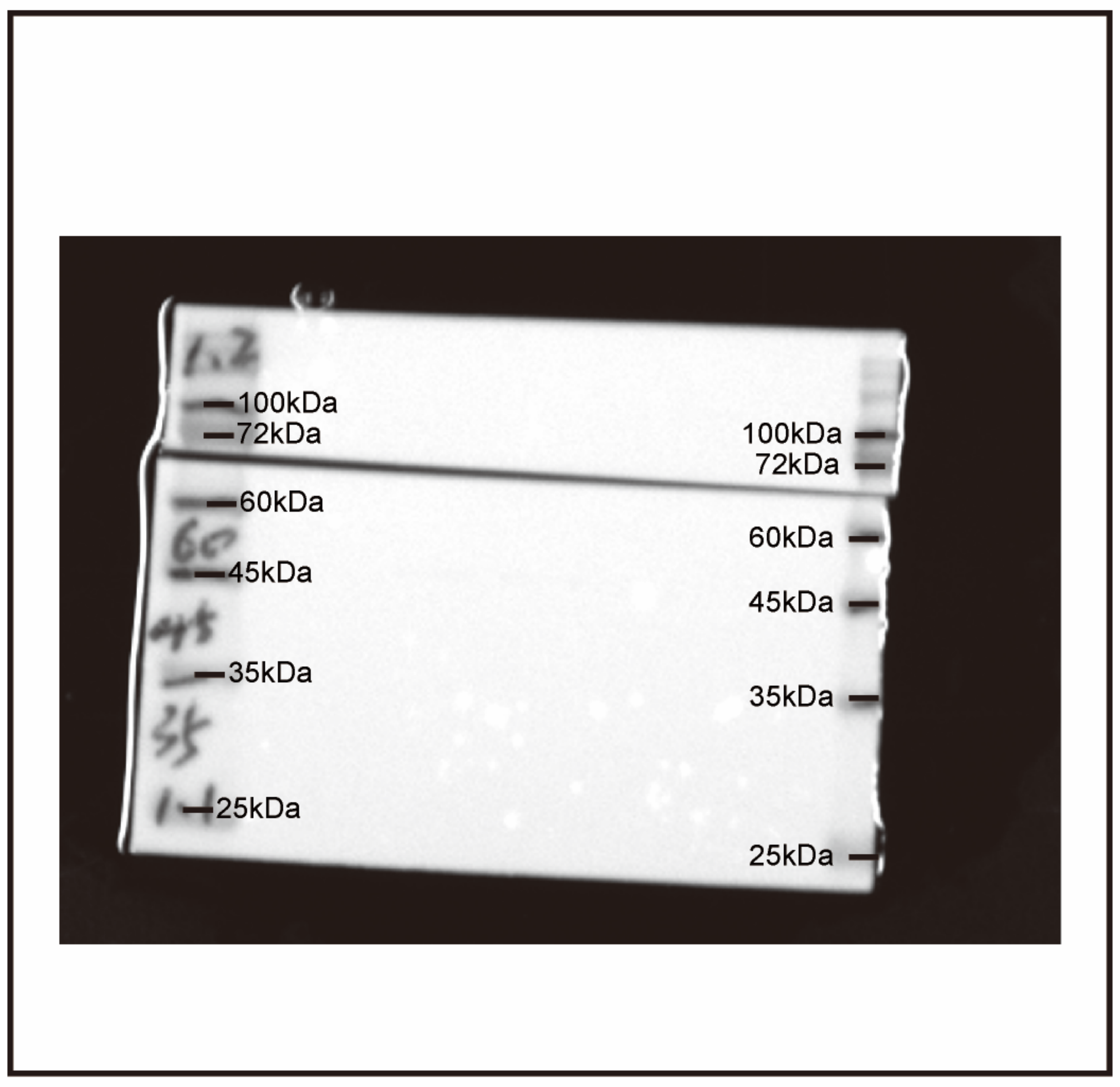

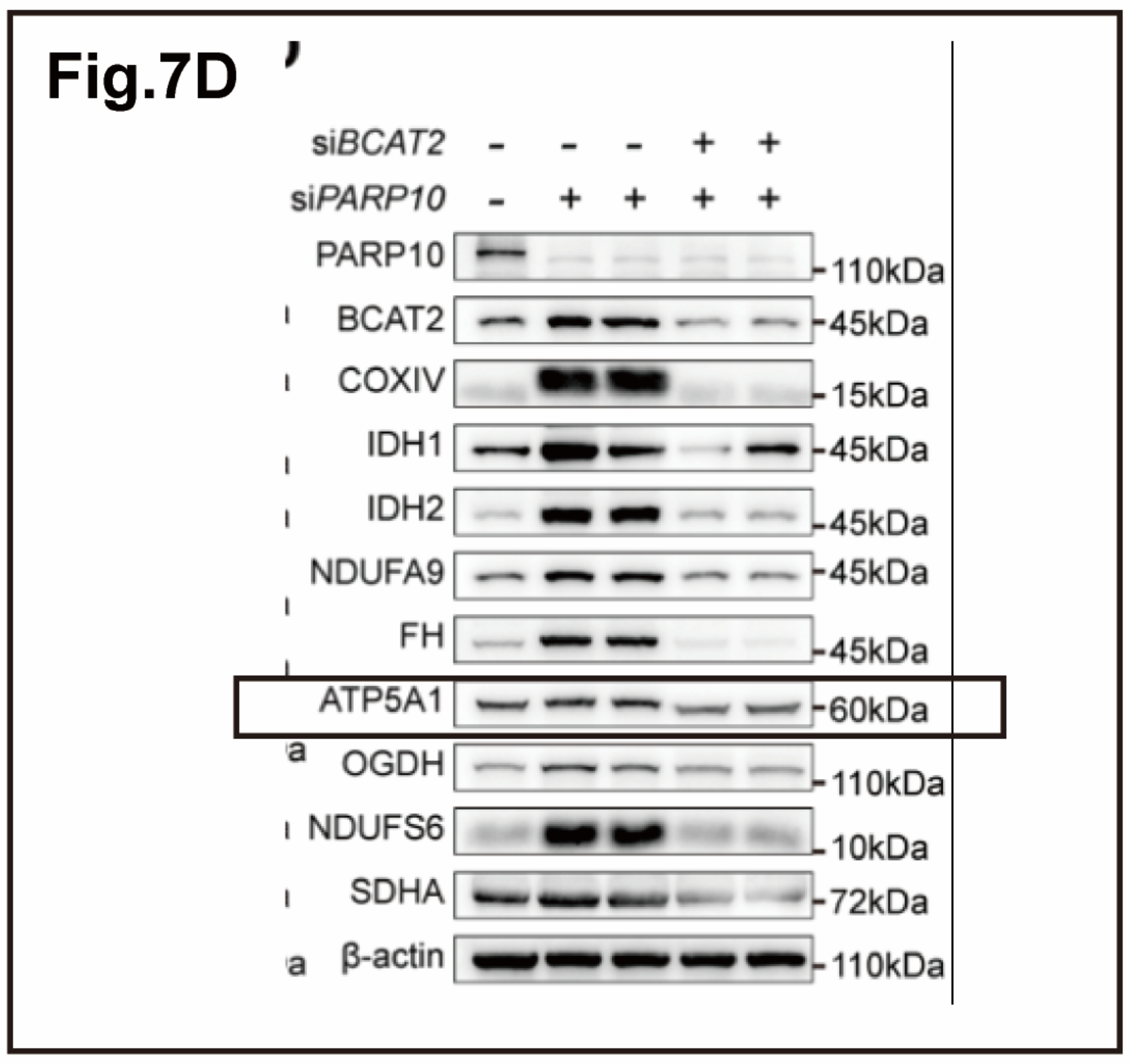

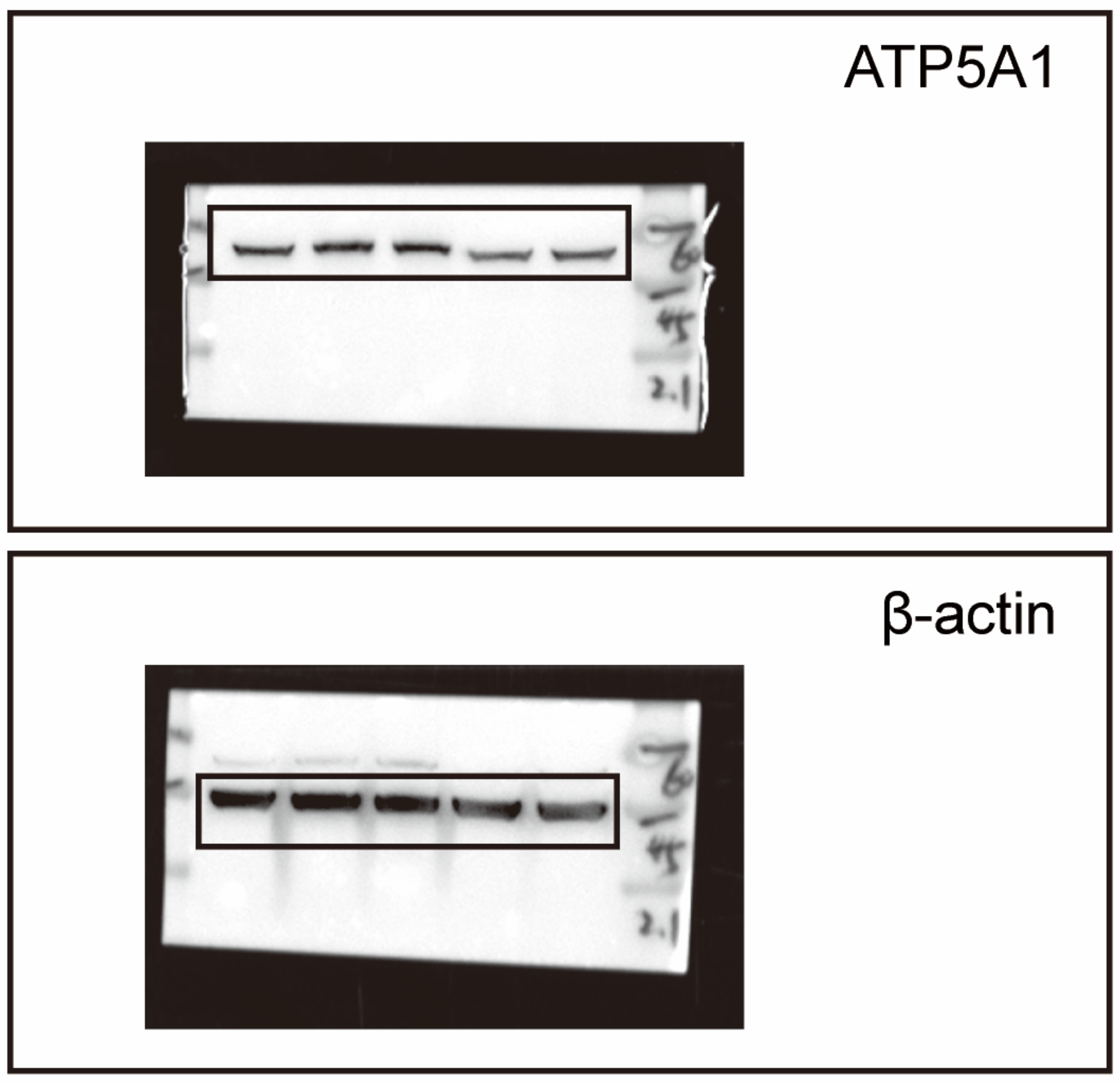

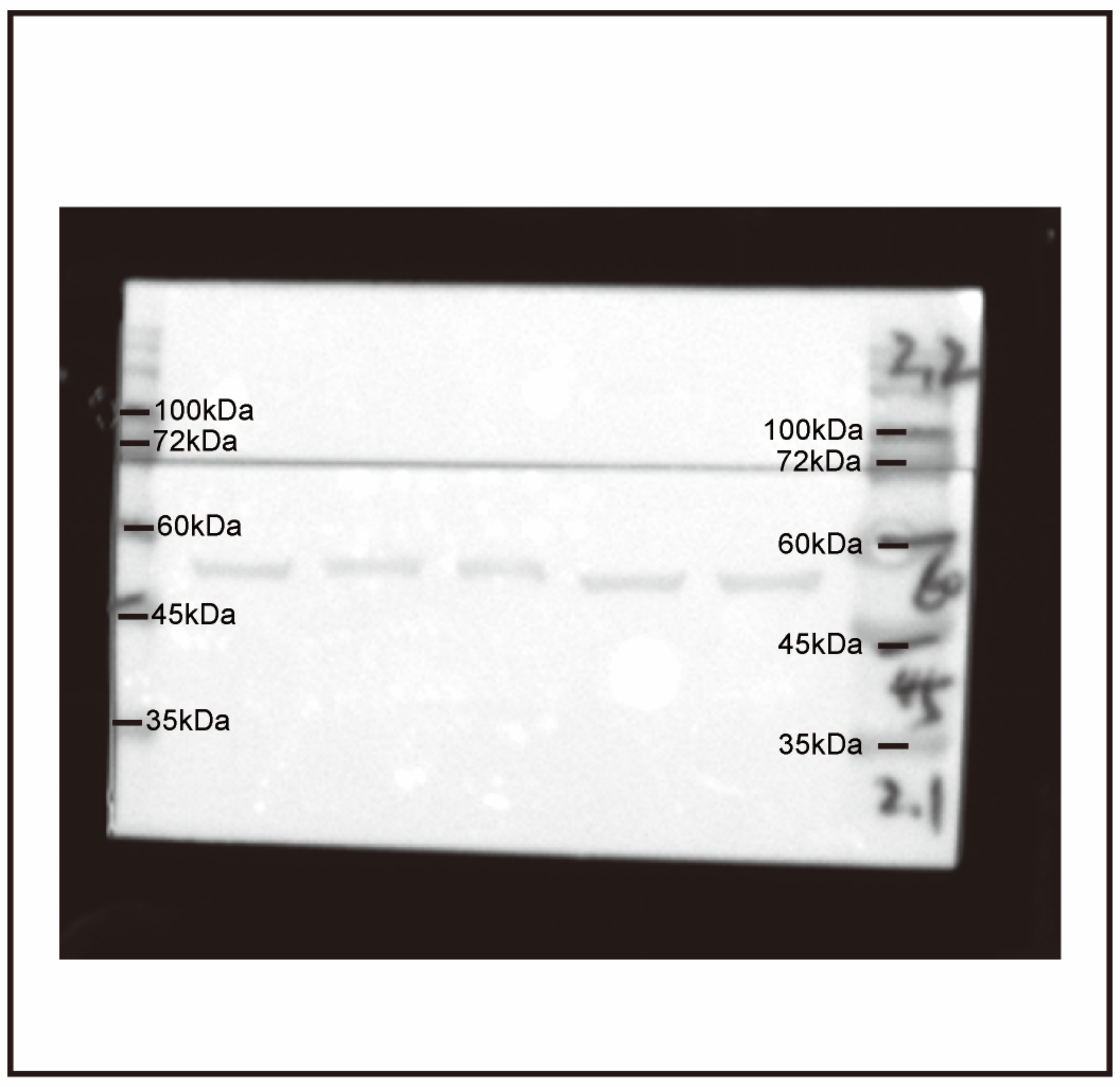

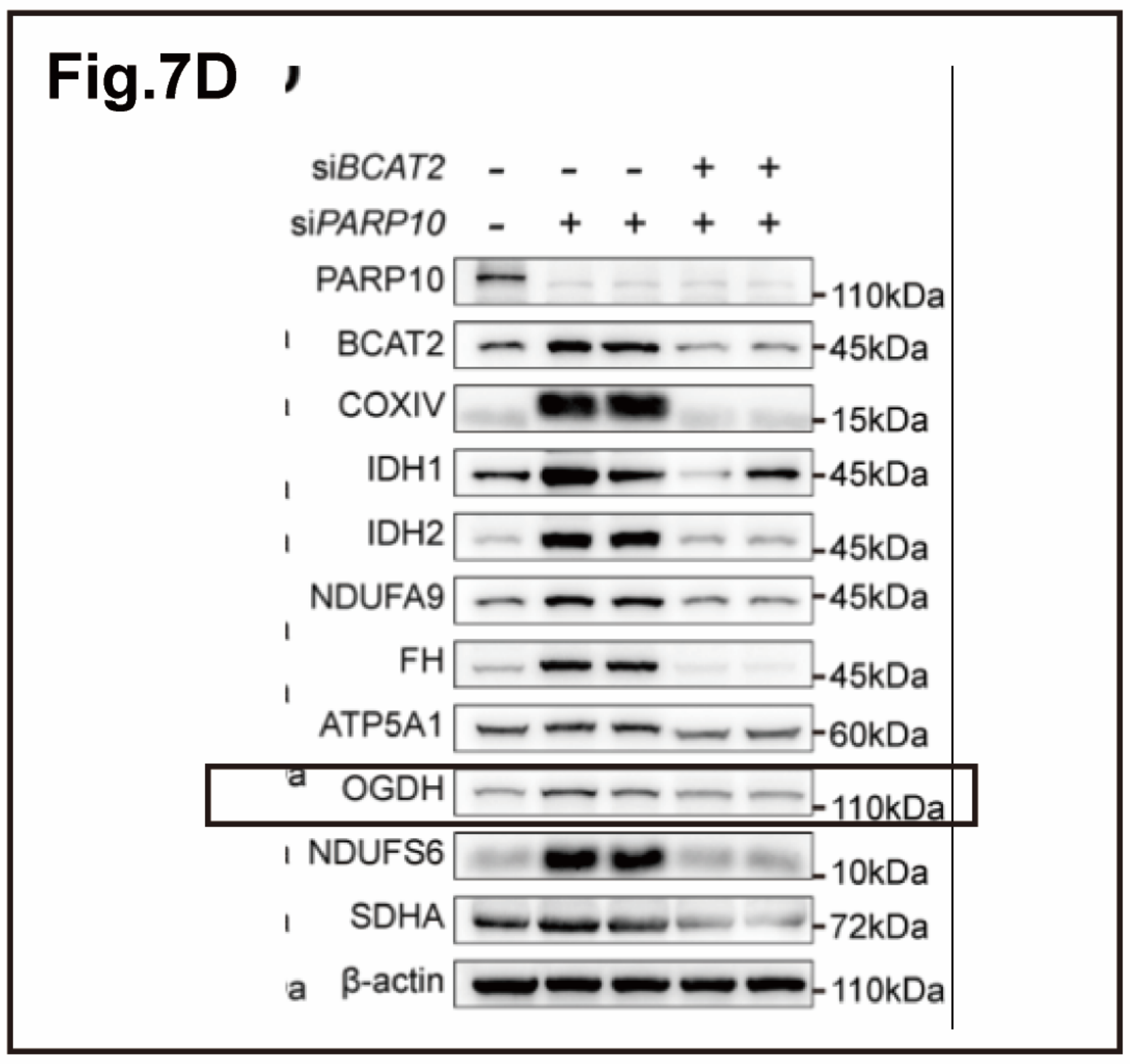

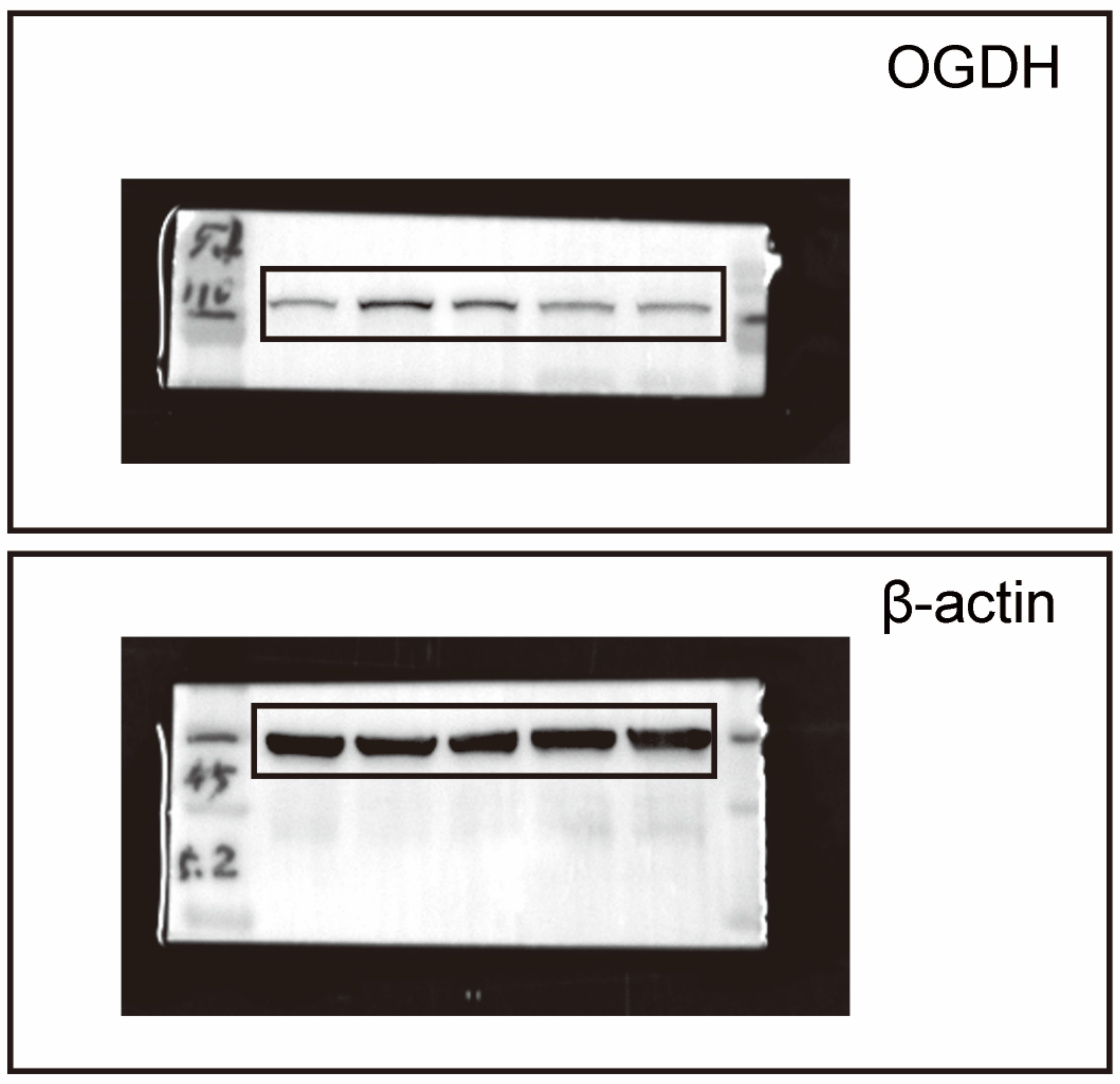

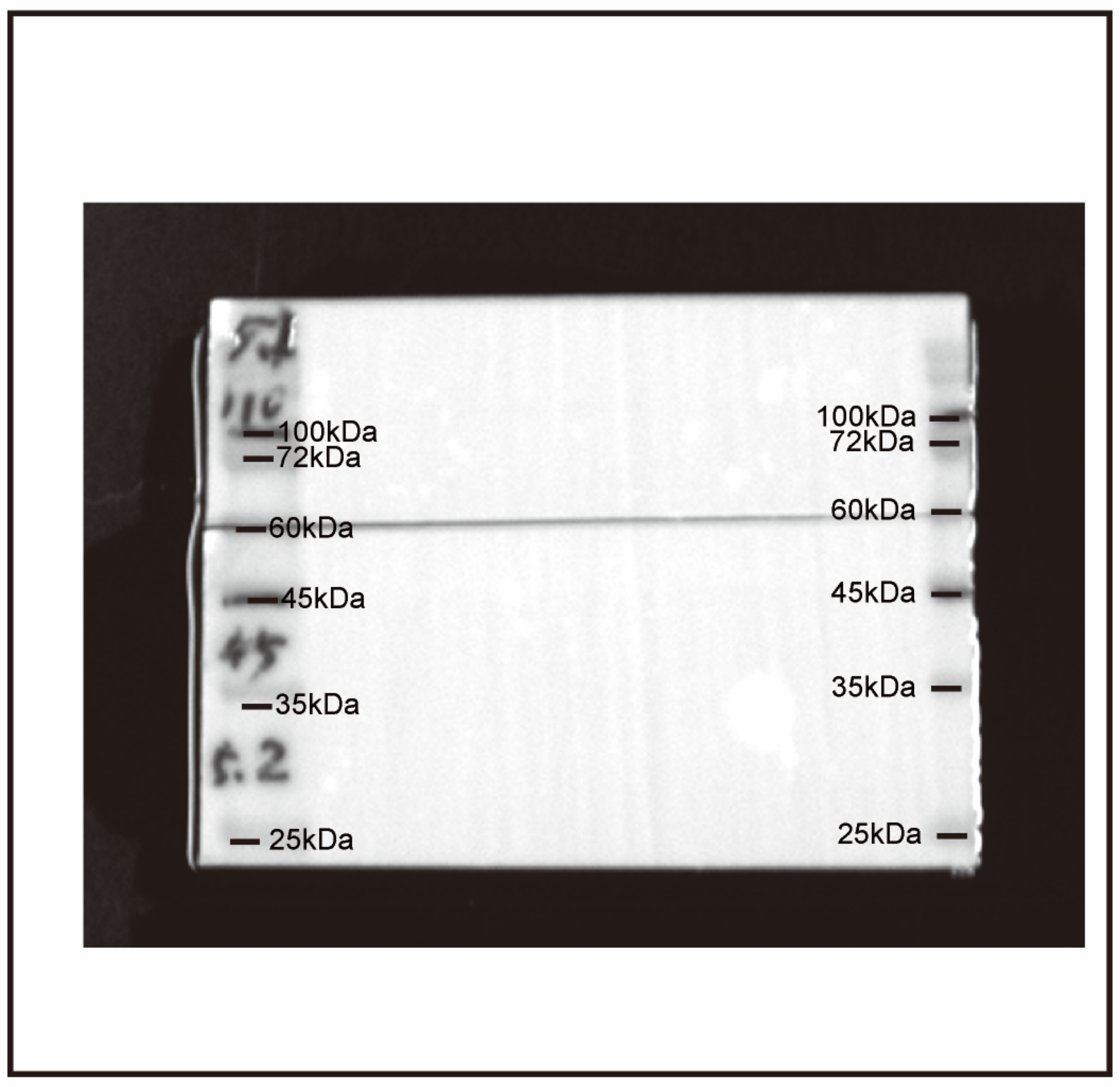

Figure	Merged	Stiched full image

Fig.7D ’

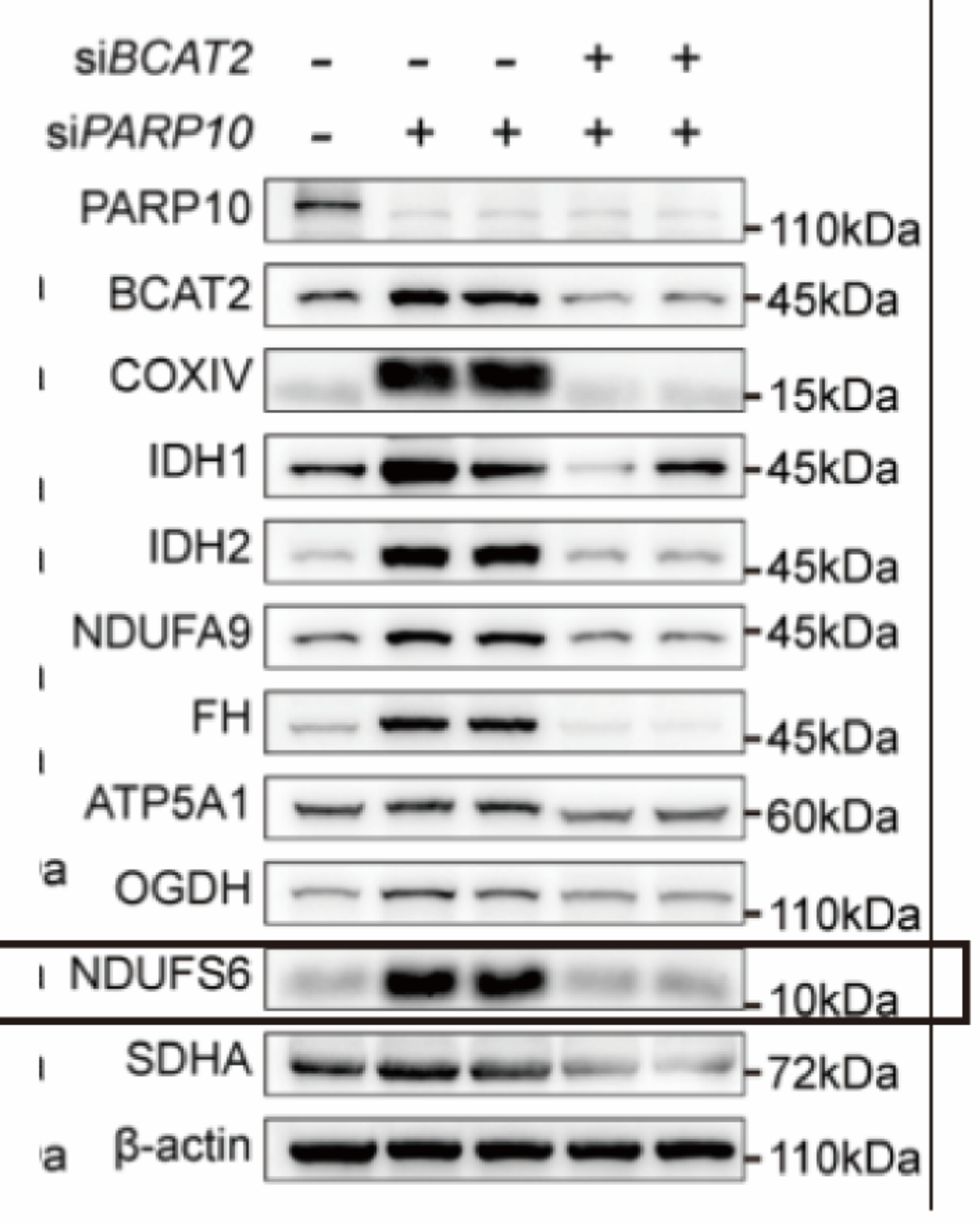

NDUFS6

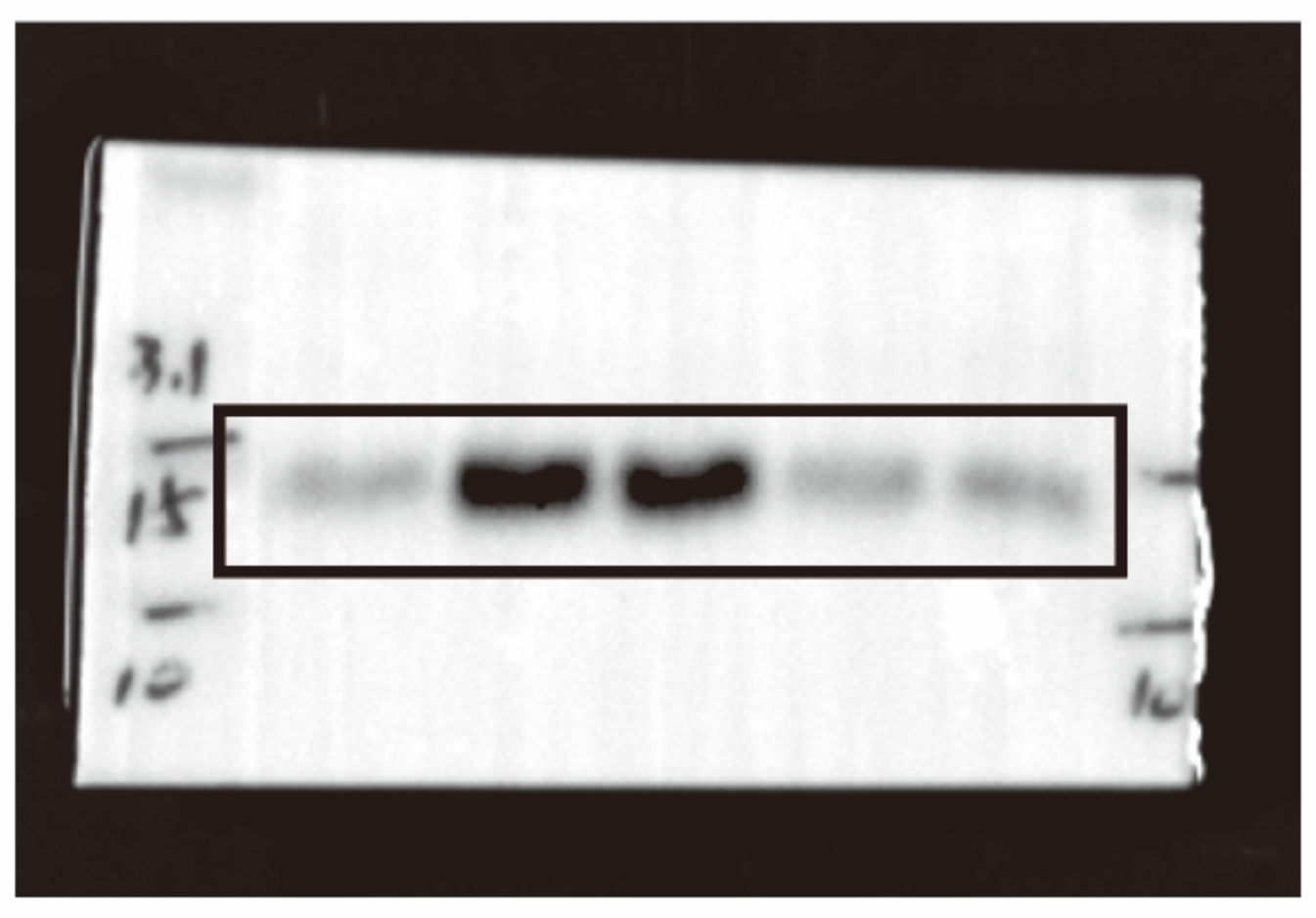

Vinculin

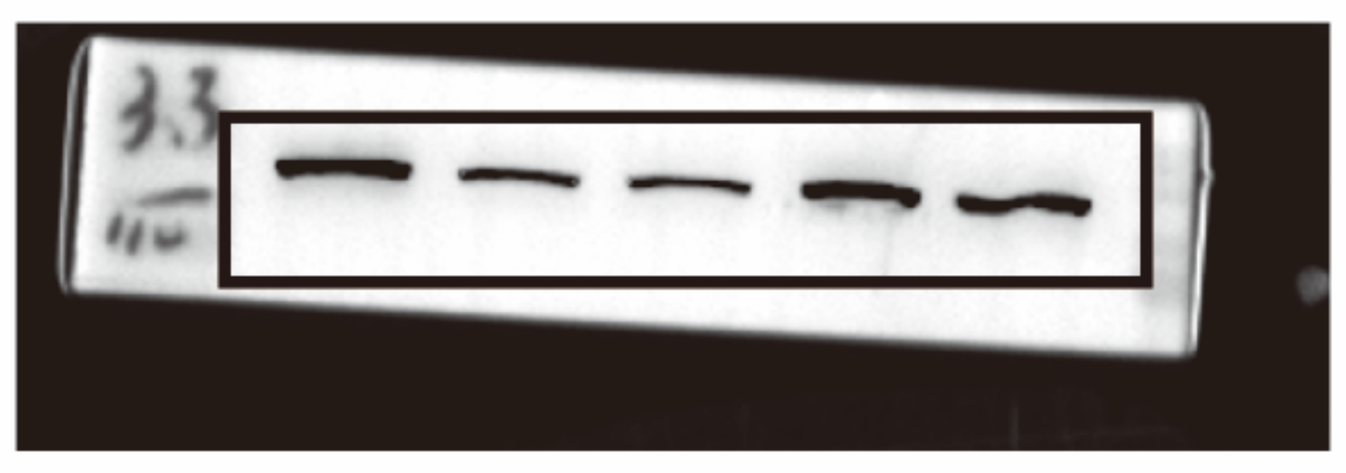

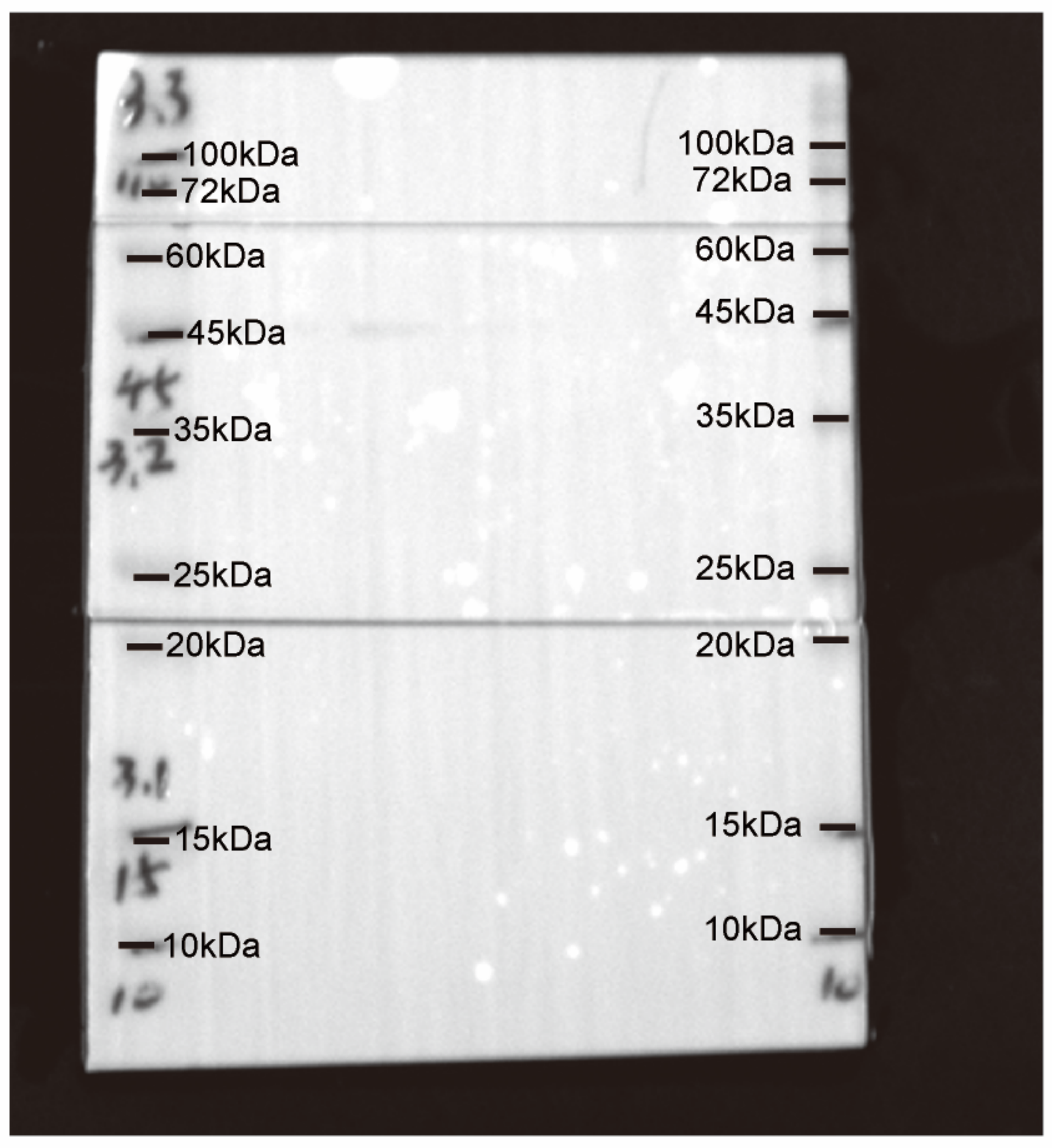

Fig.7D ’

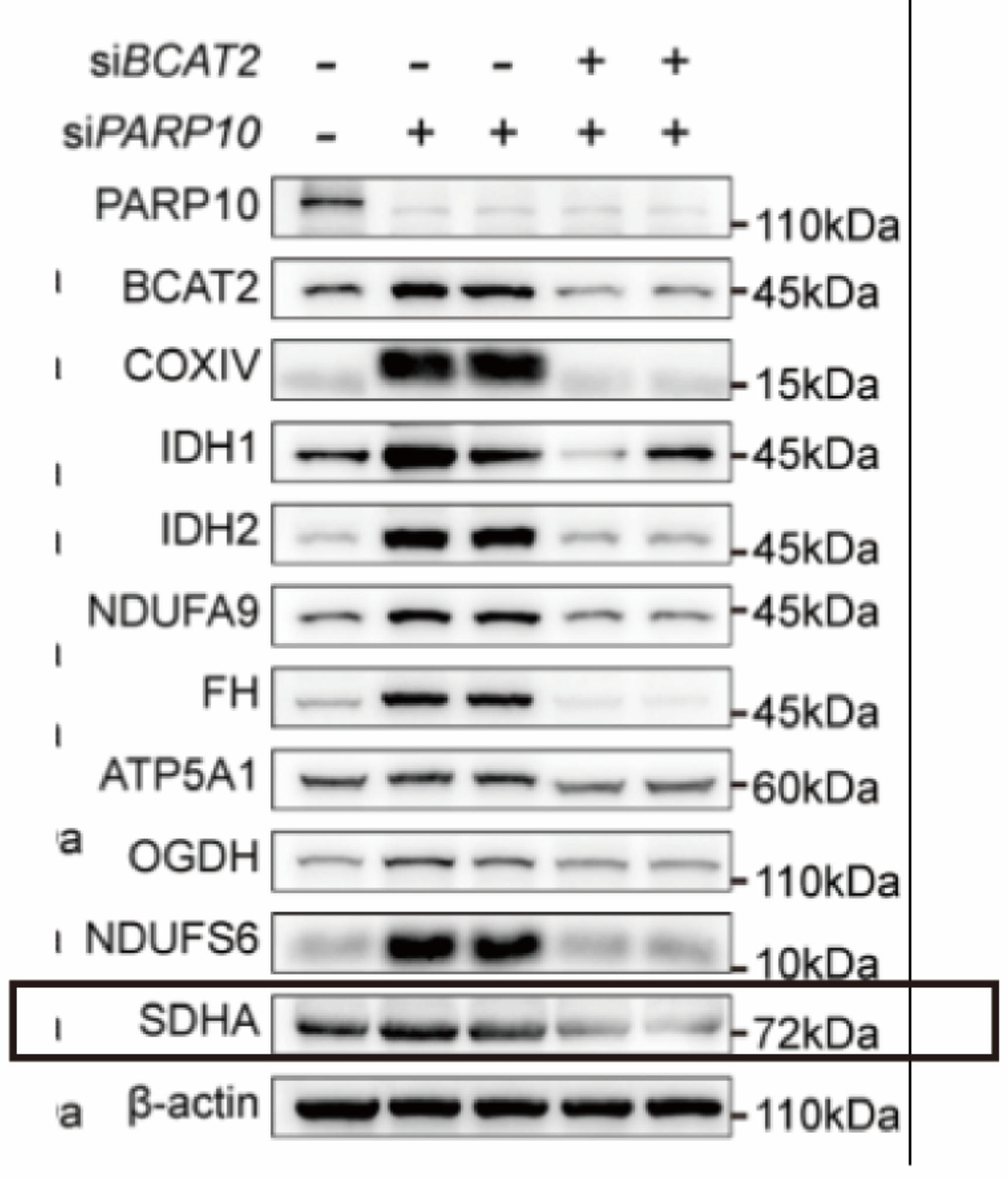

SDHA

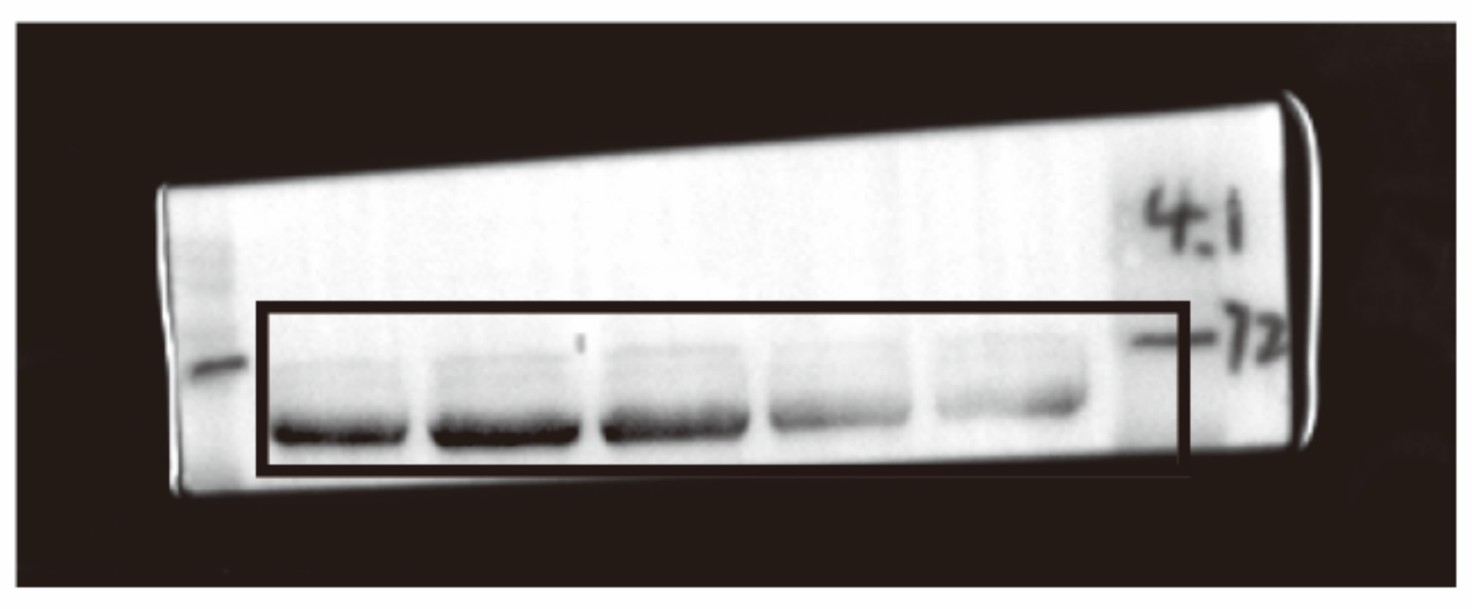

β-actin

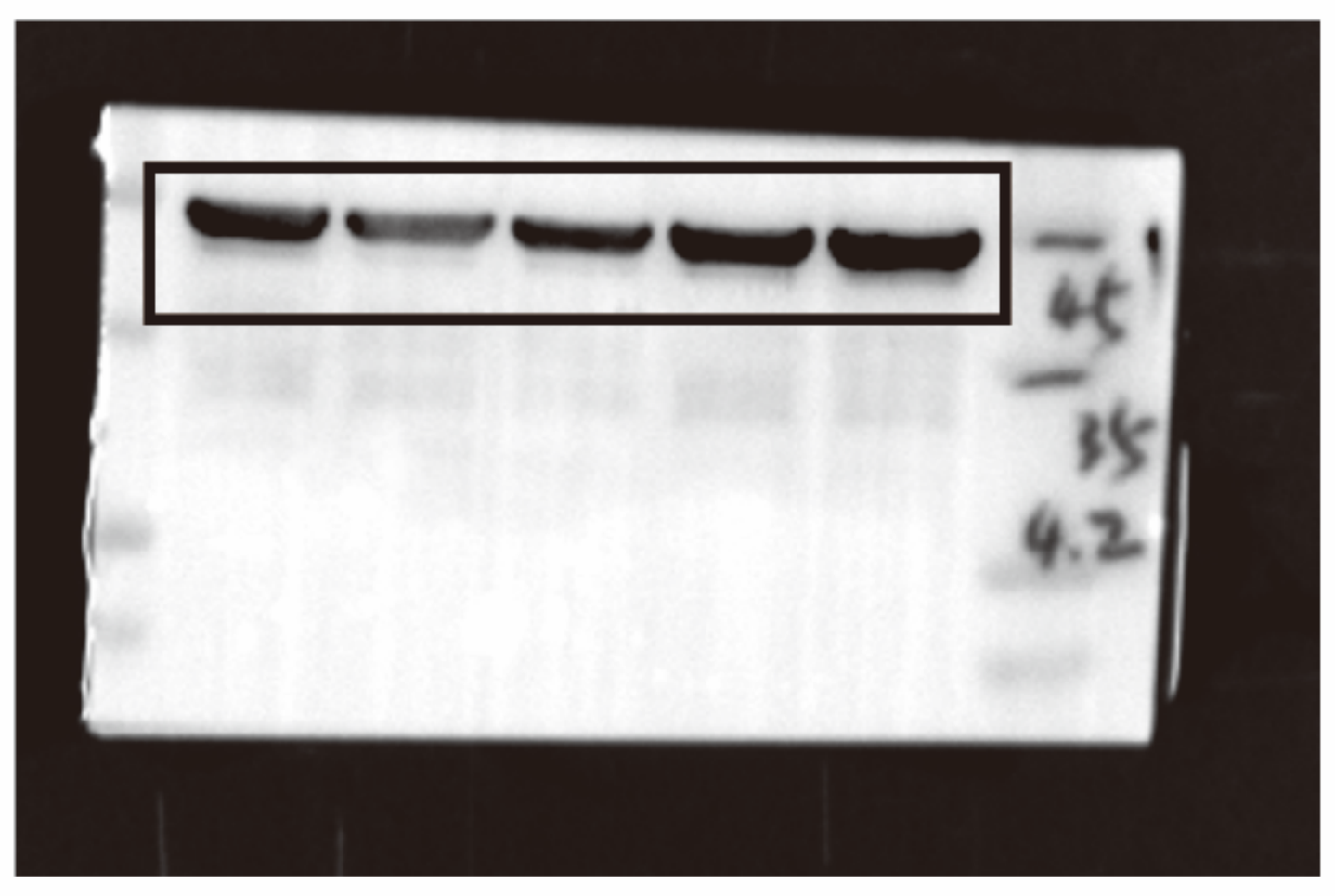

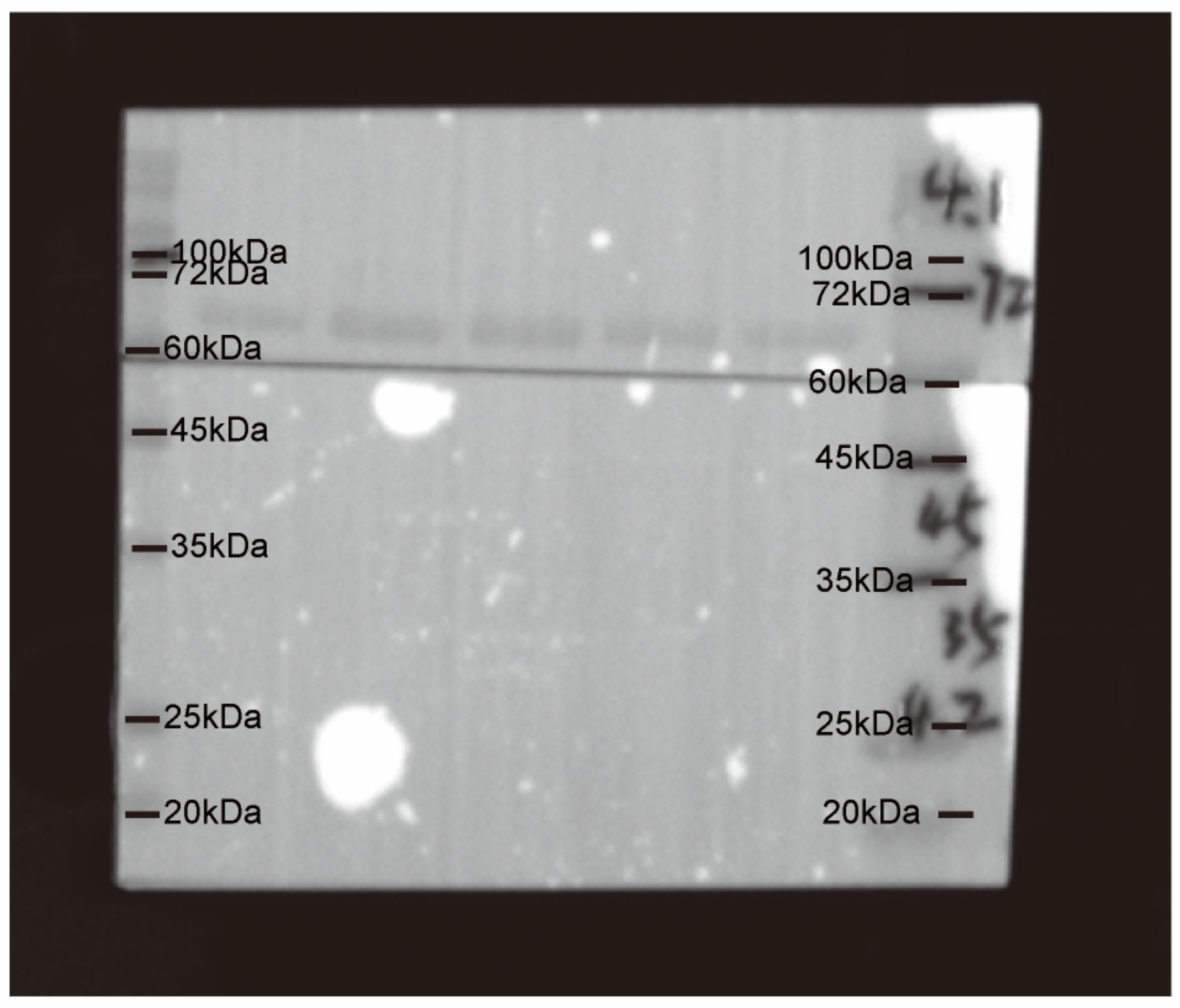

Fig.S1E

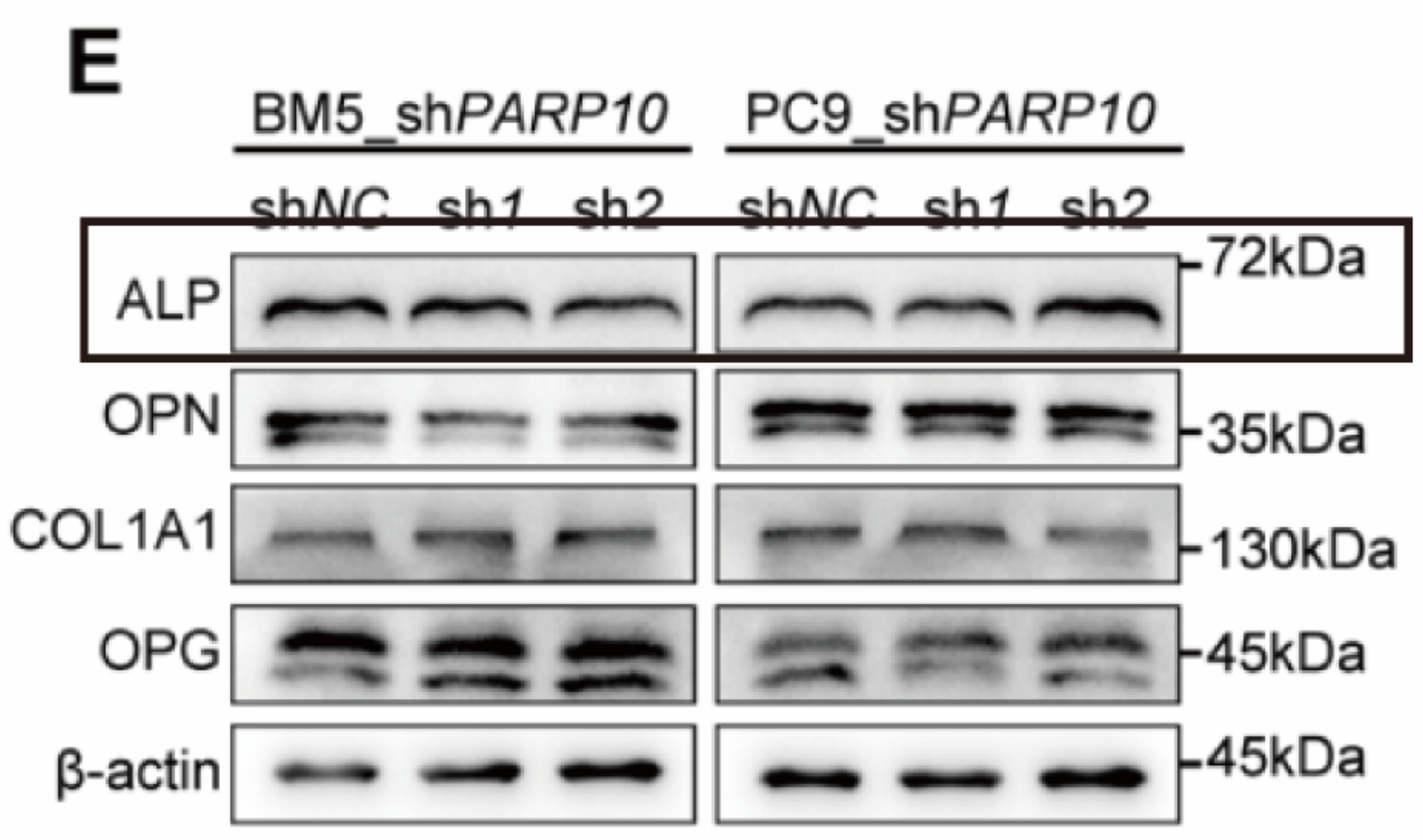

ALP

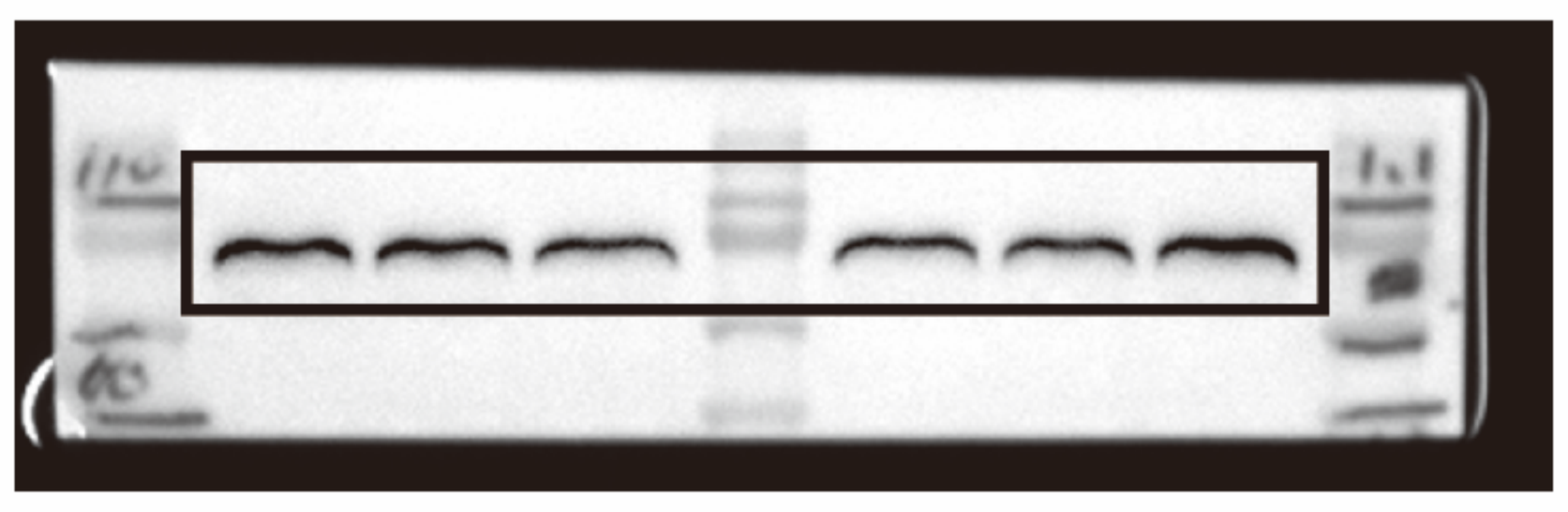

GAPDH

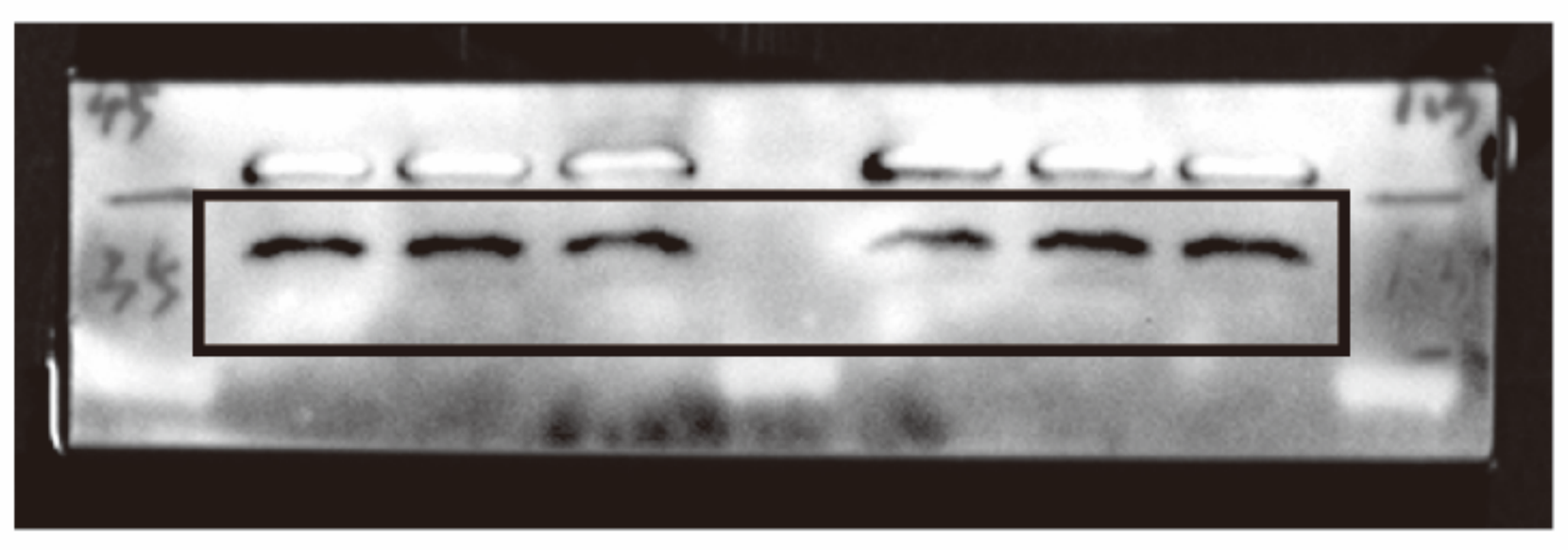

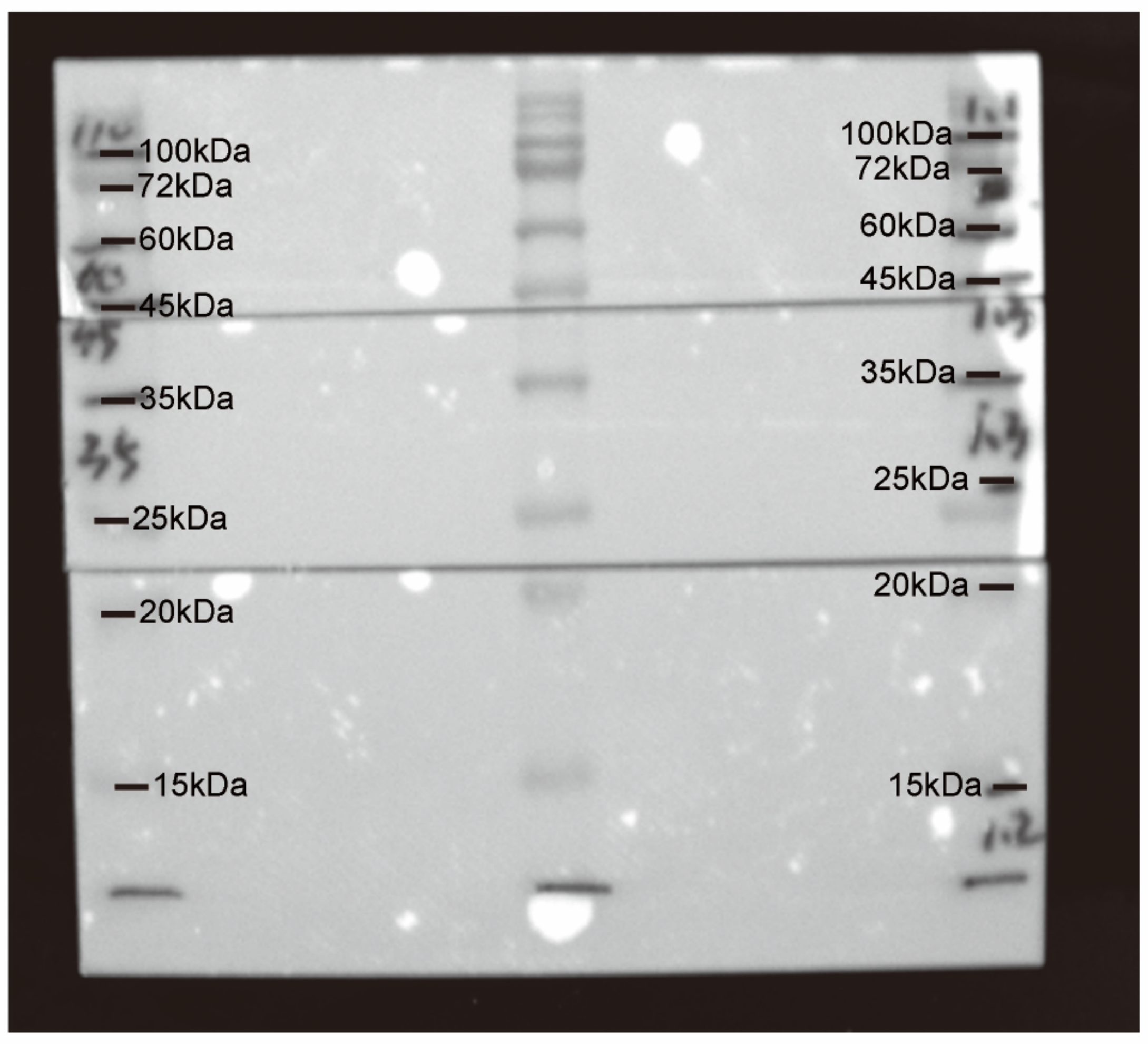

Fig.S1E

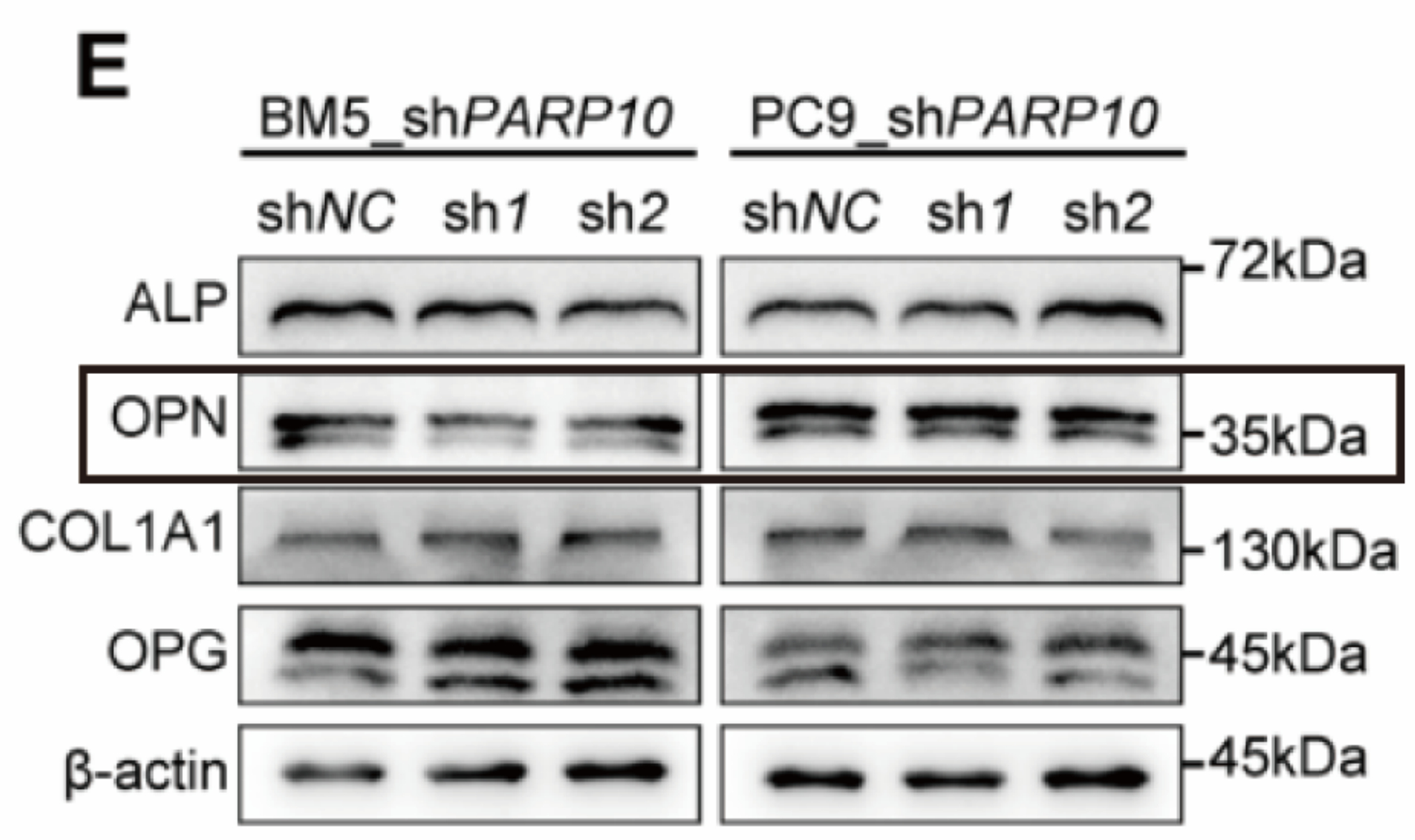

OPN

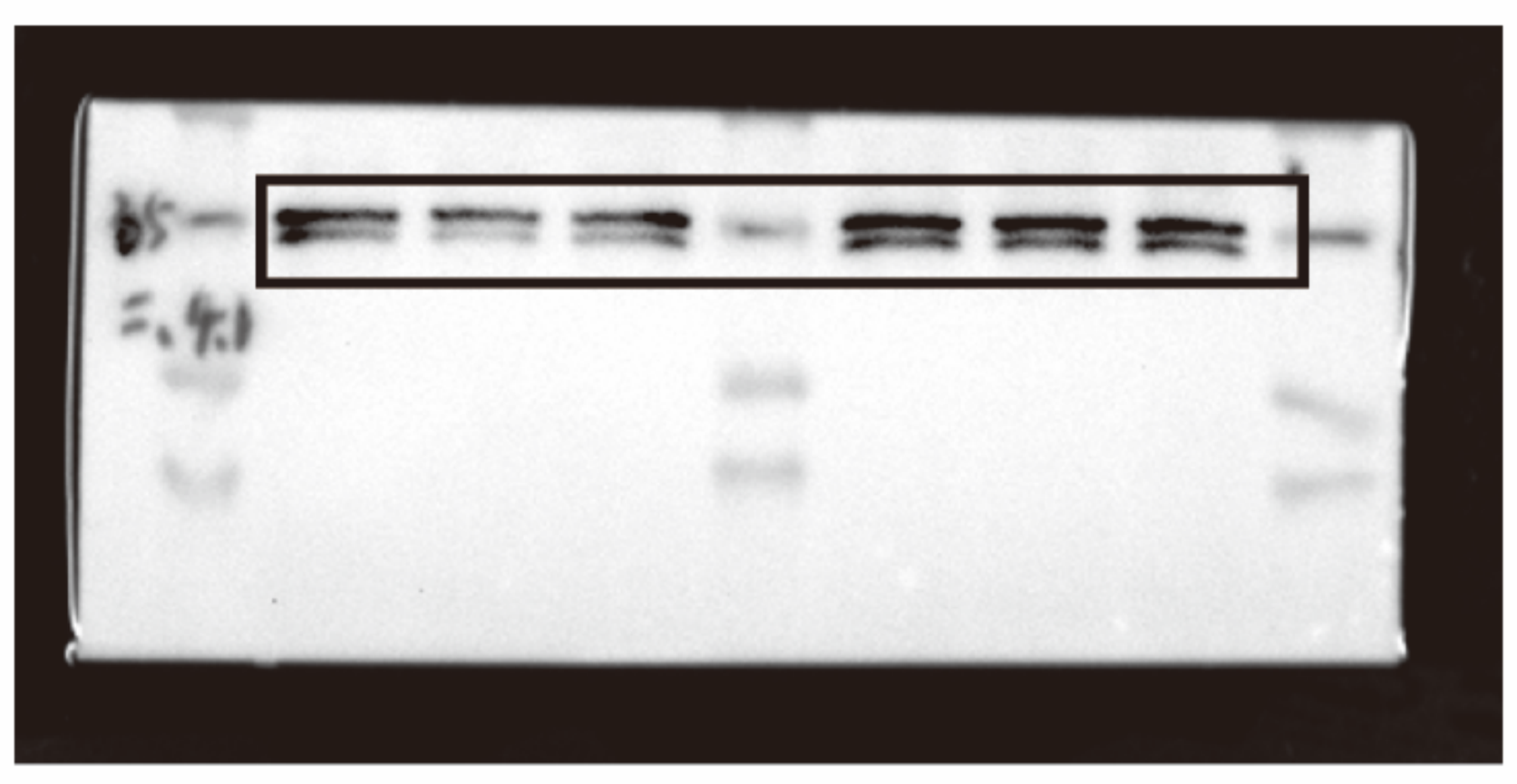

Vinculin

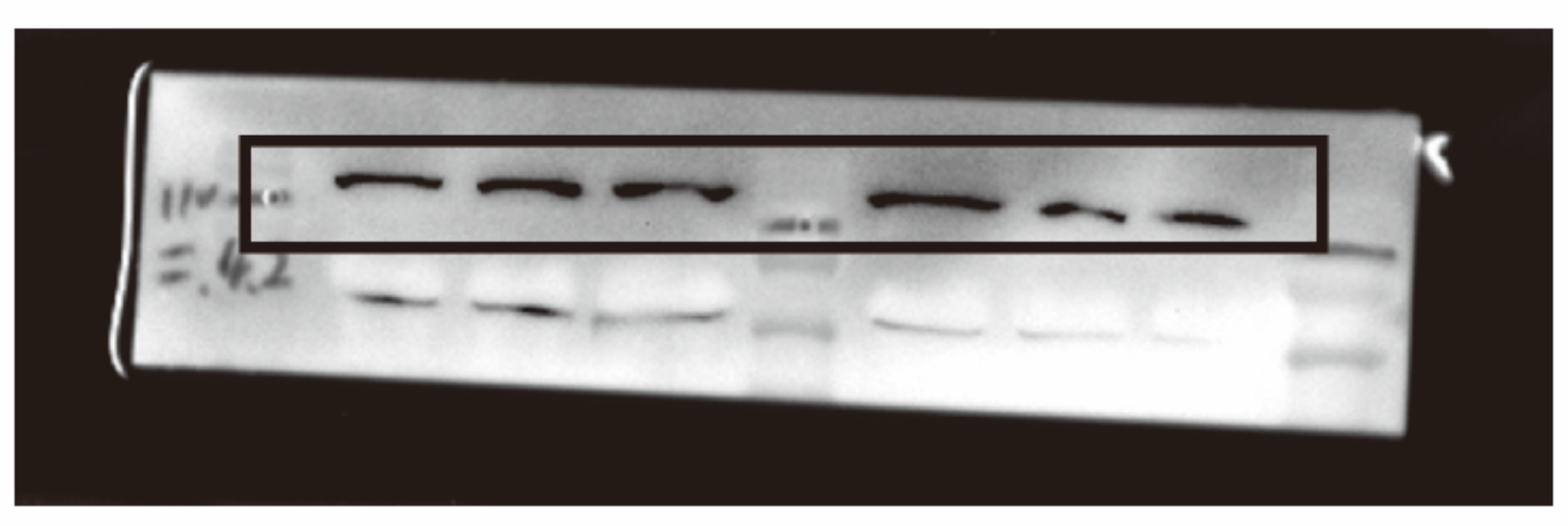

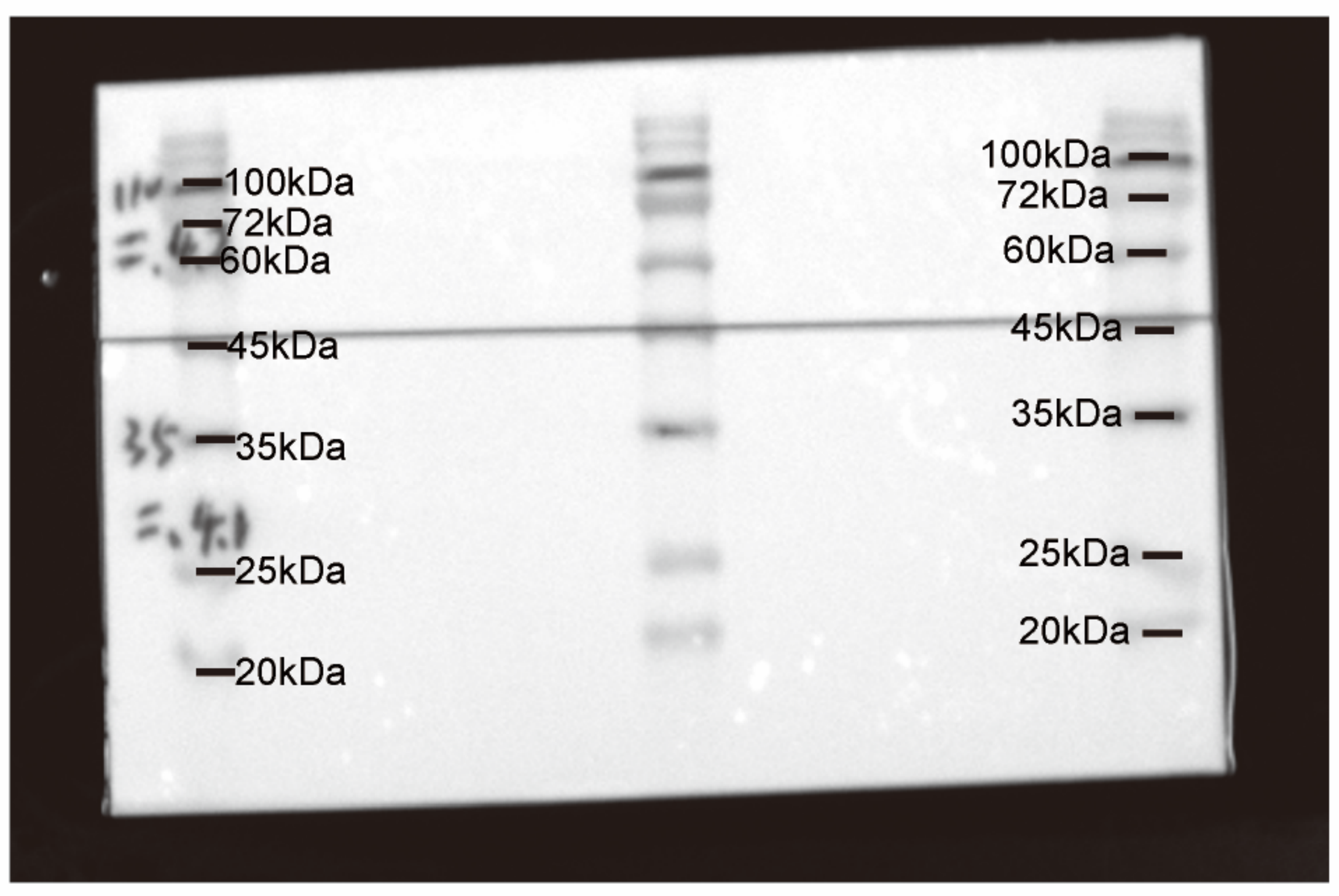

Figure	Merged	Stiched full image

Fig.S1E

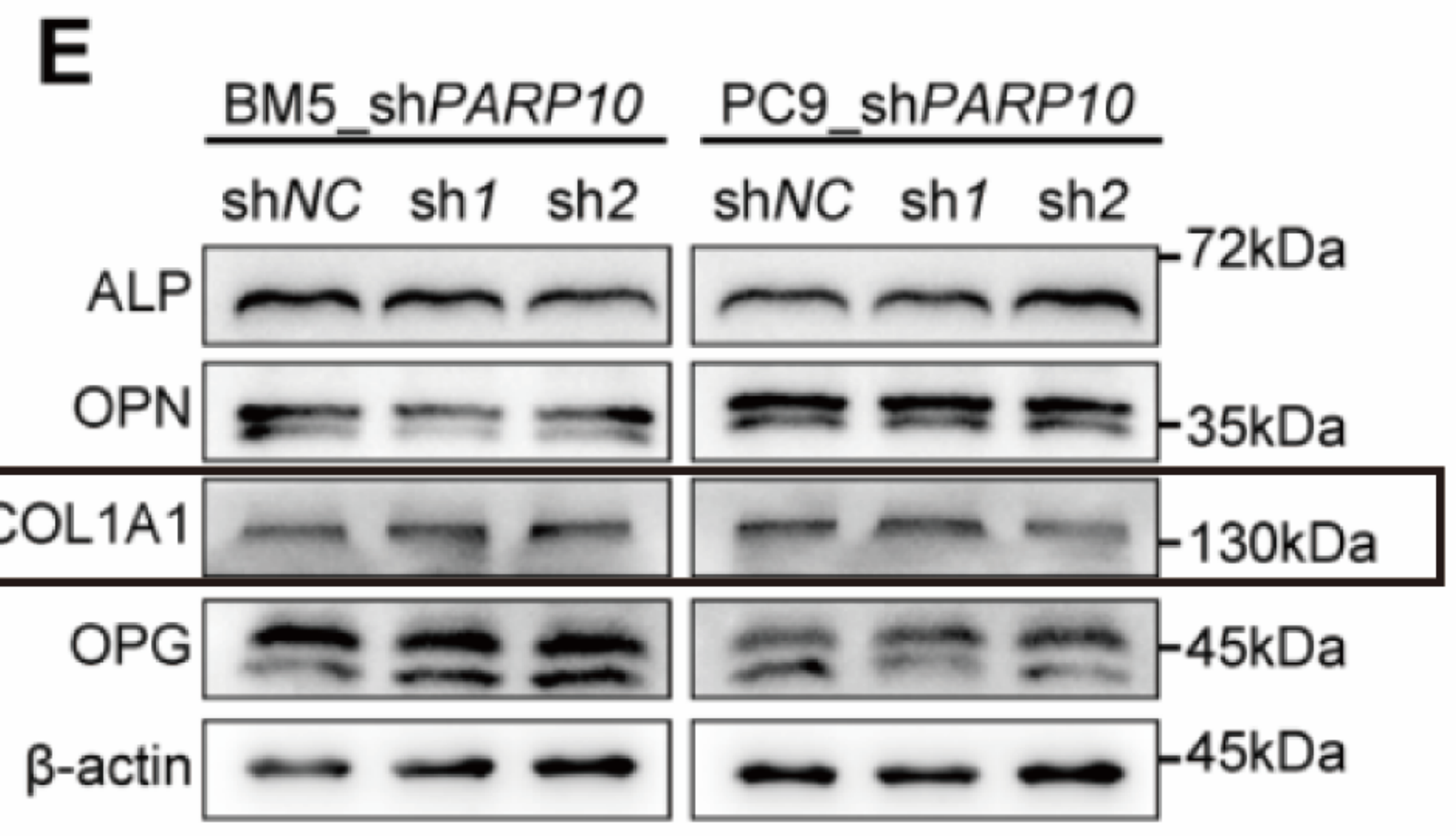

COL1A1

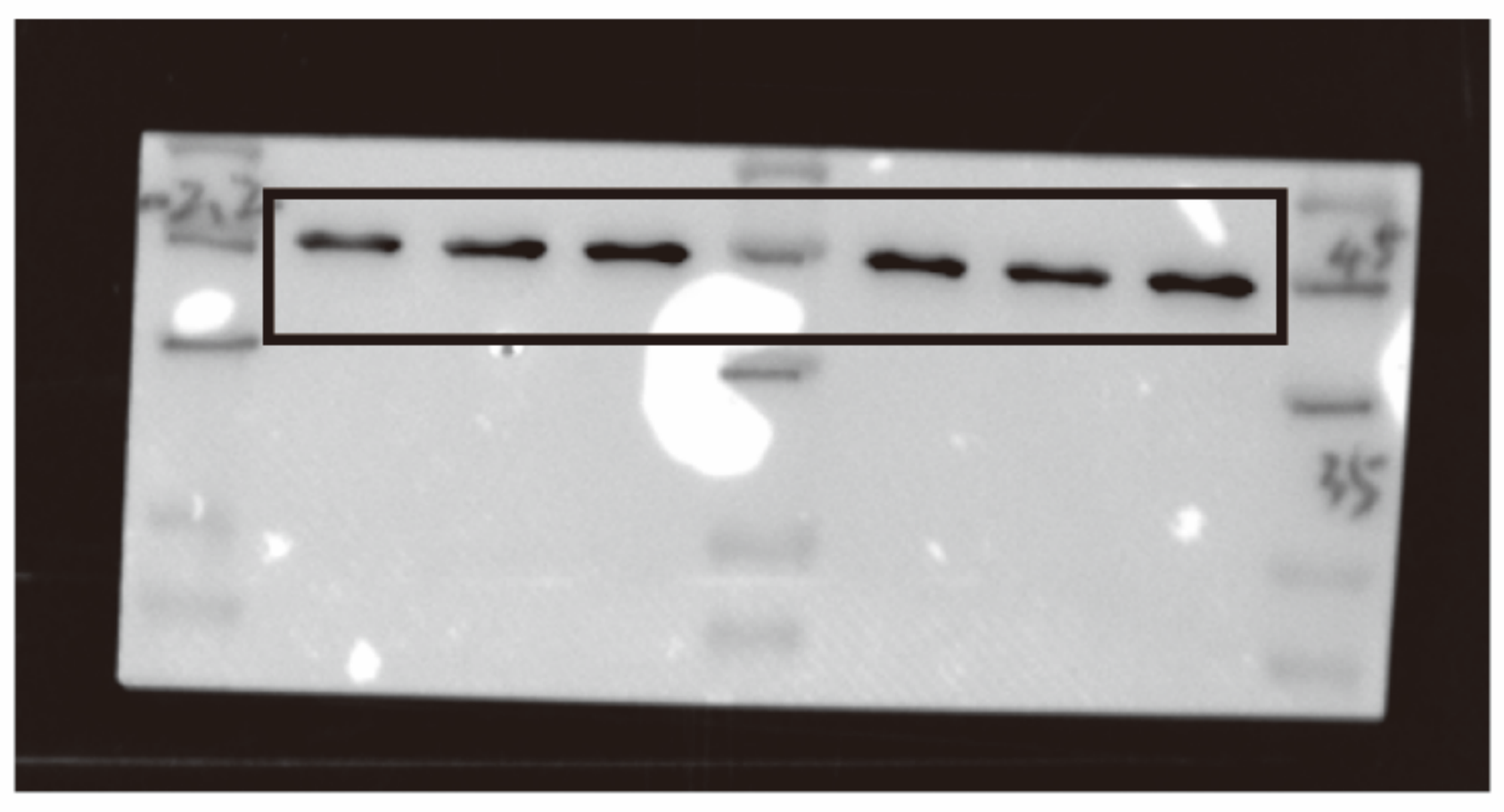

Vinculin

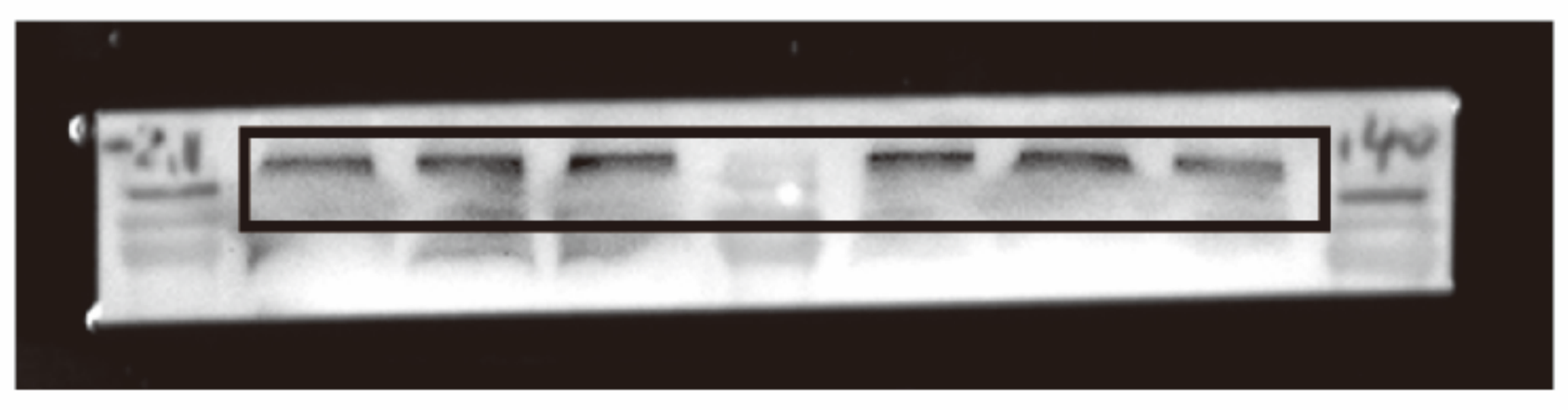

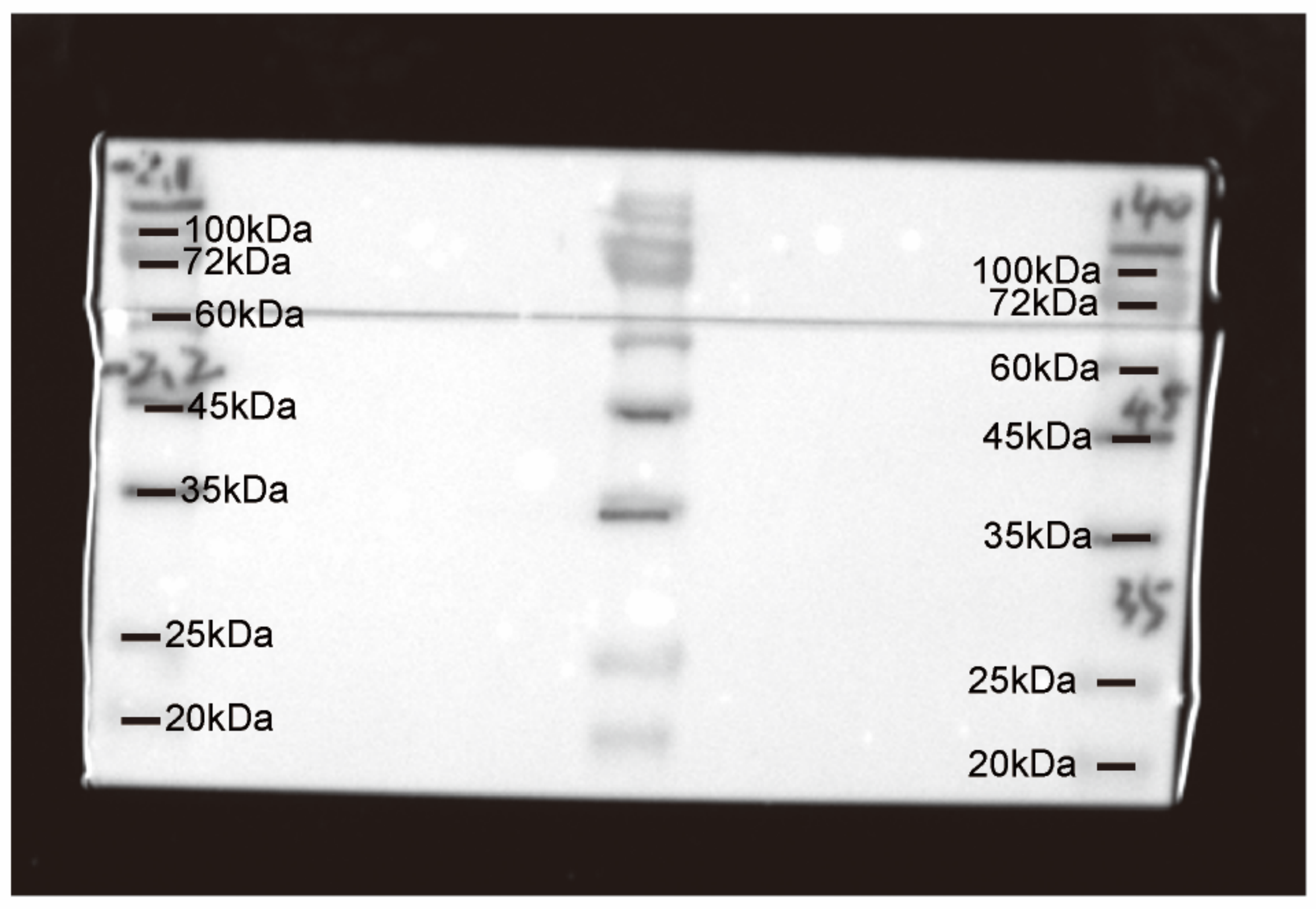

Fig.S1E

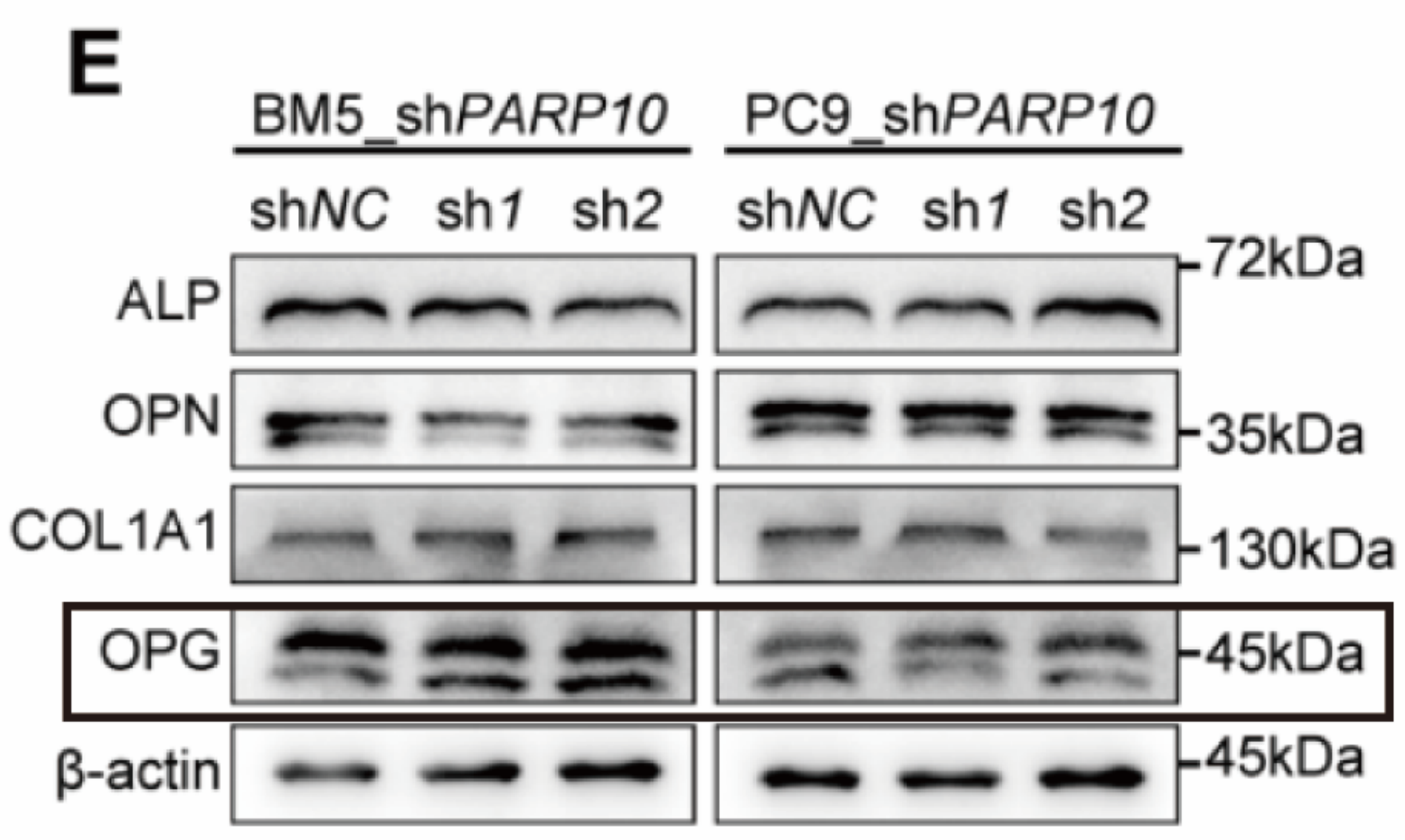

OPG

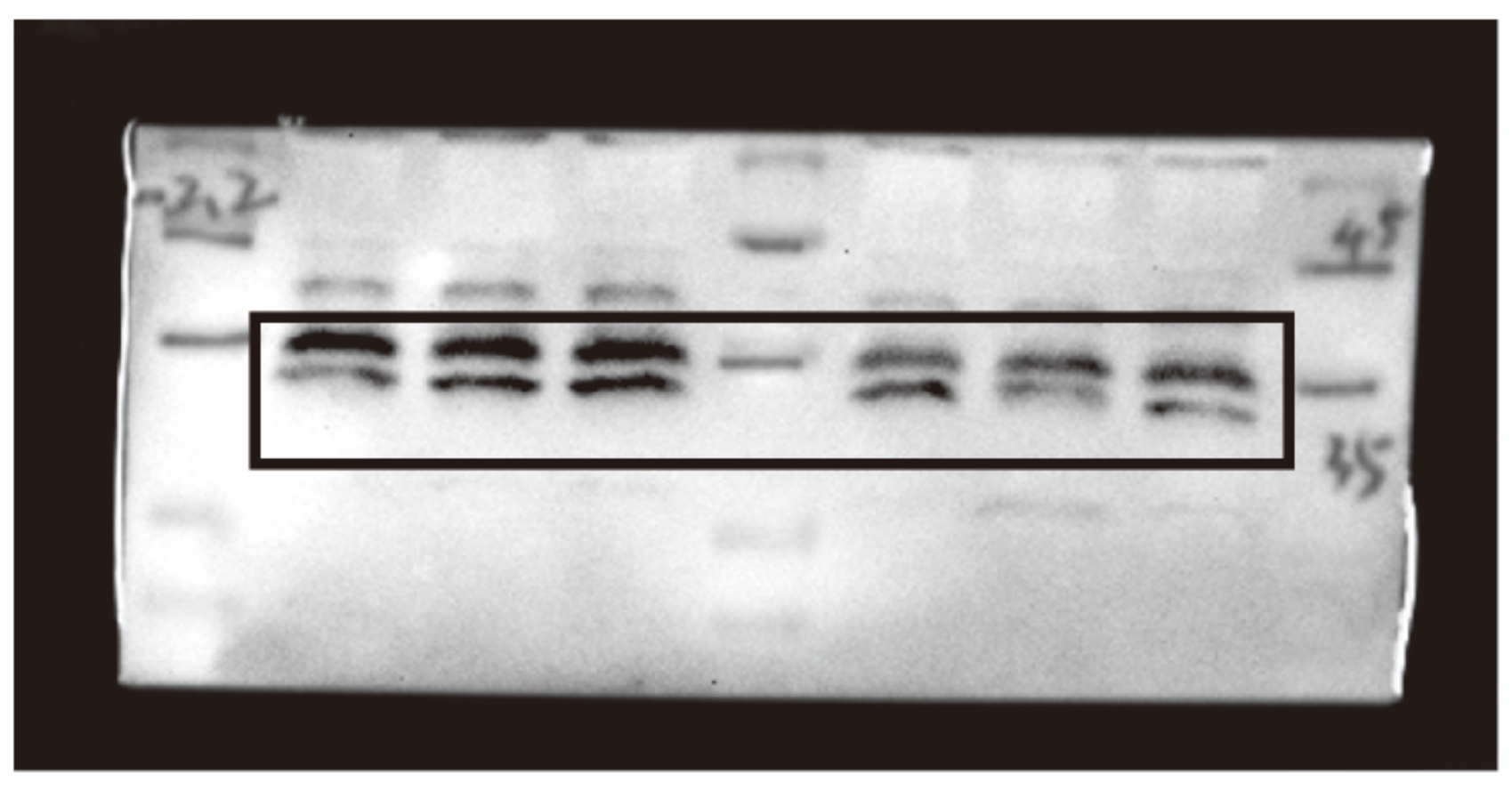

Vinculin

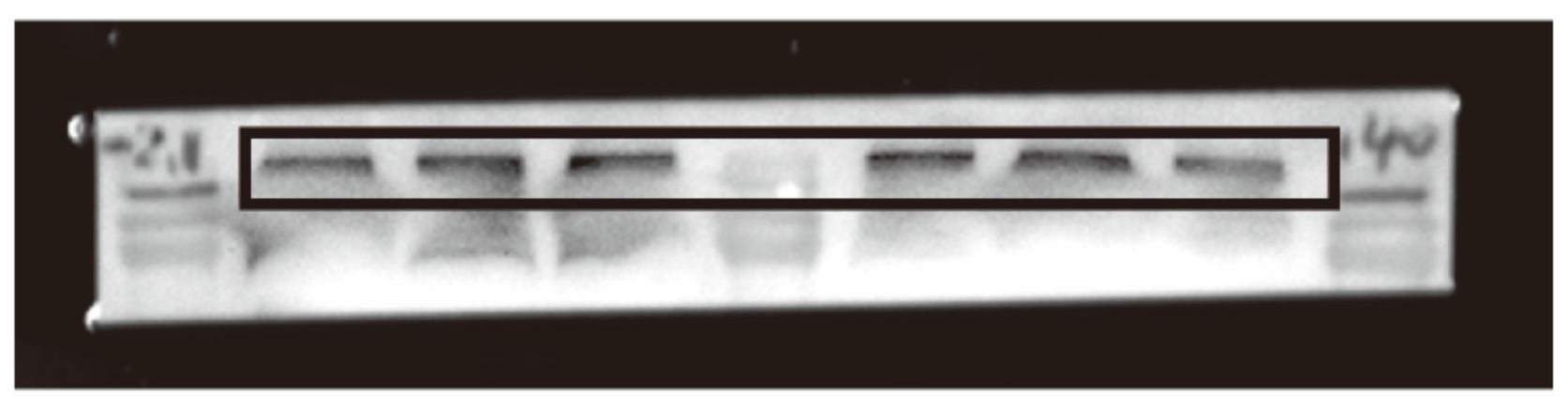

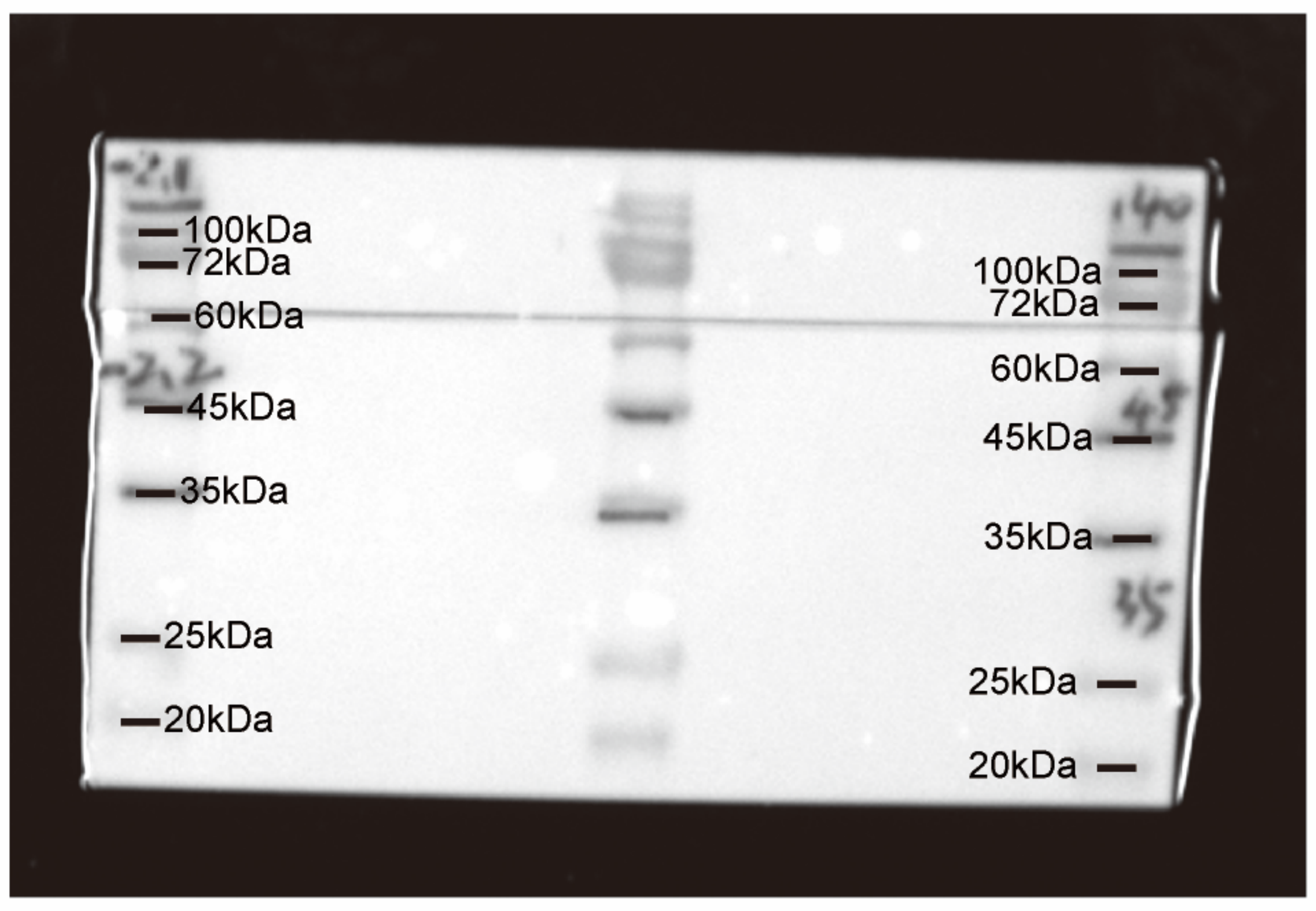

Fig.6C

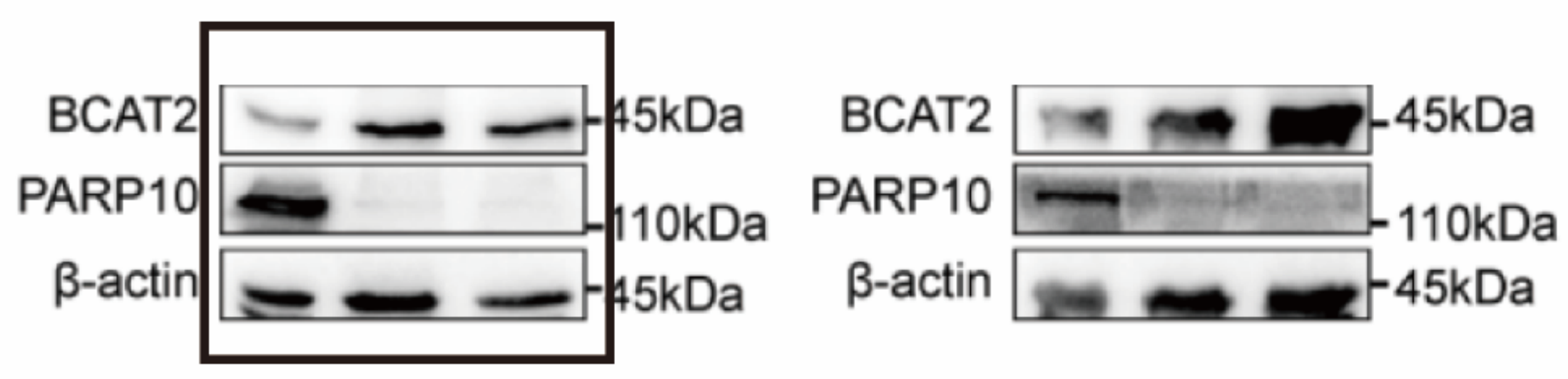

BCAT2

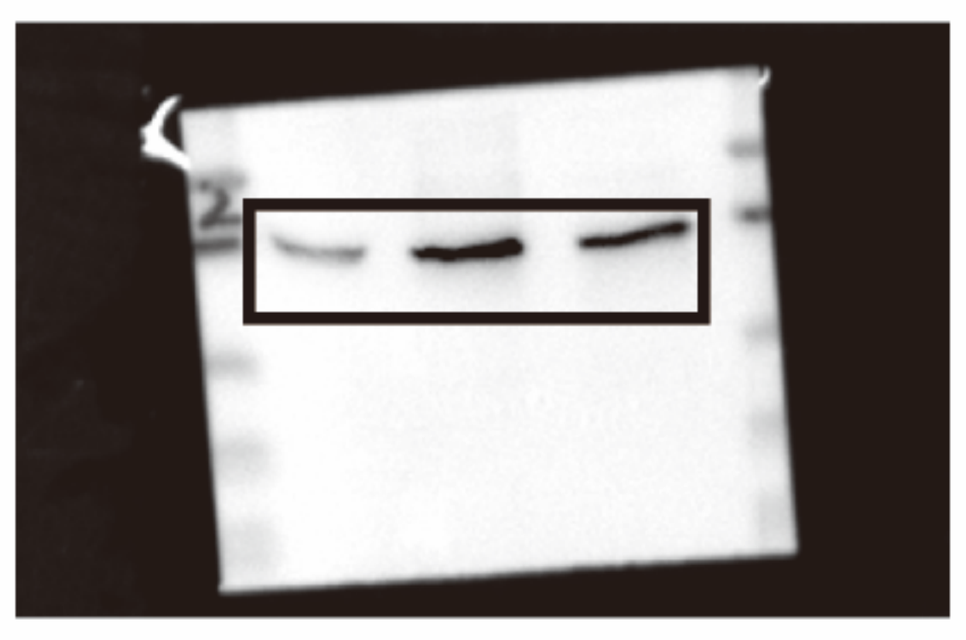

PARP10

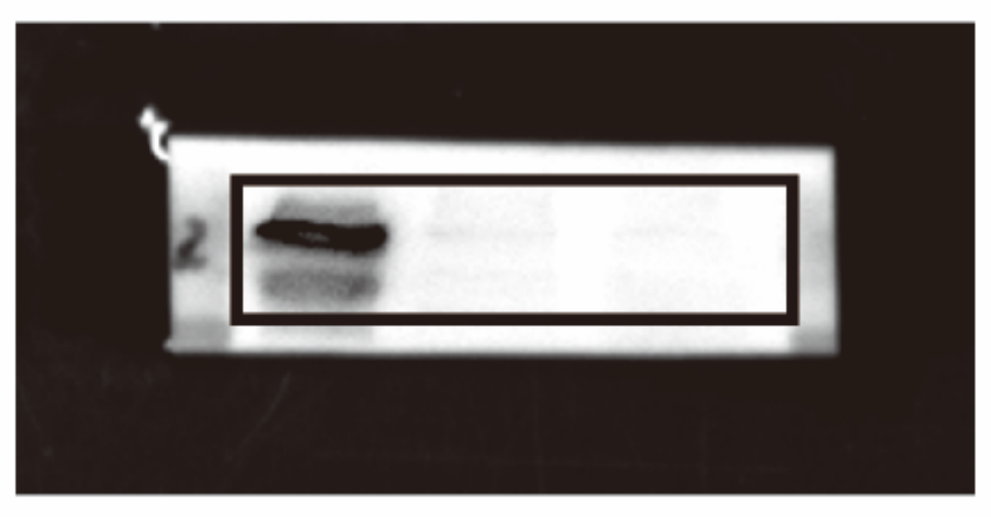

β-actin

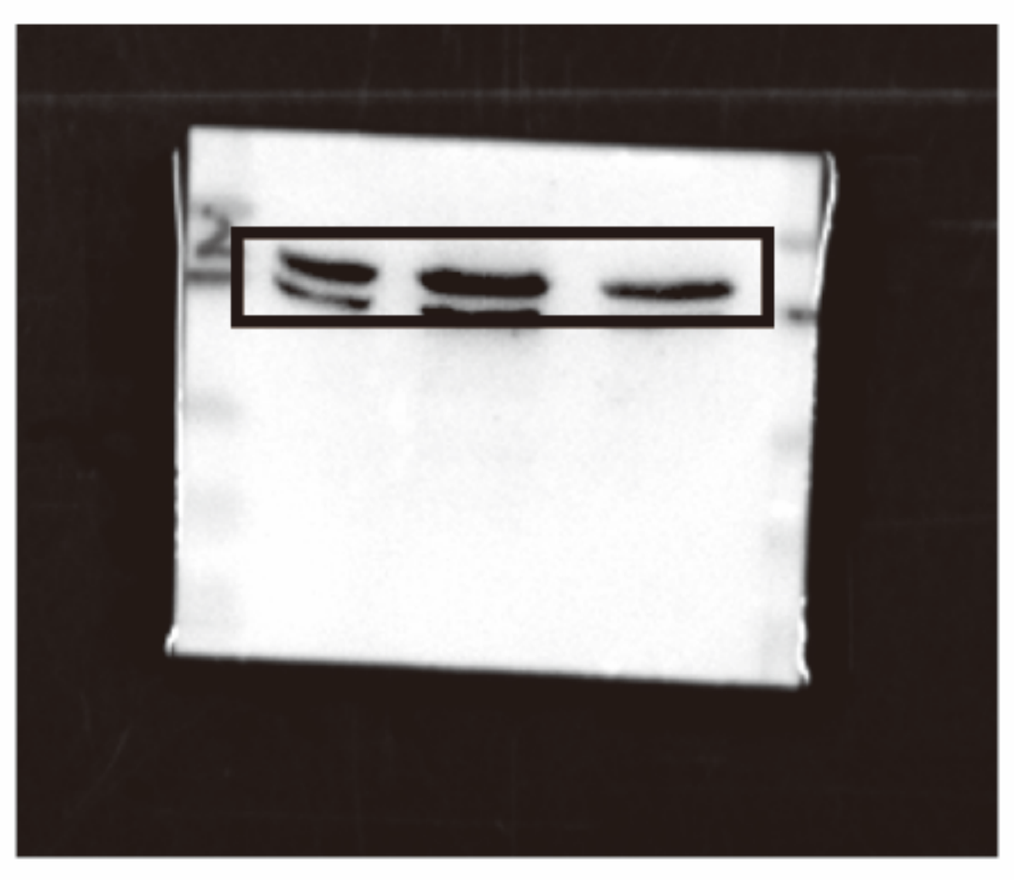

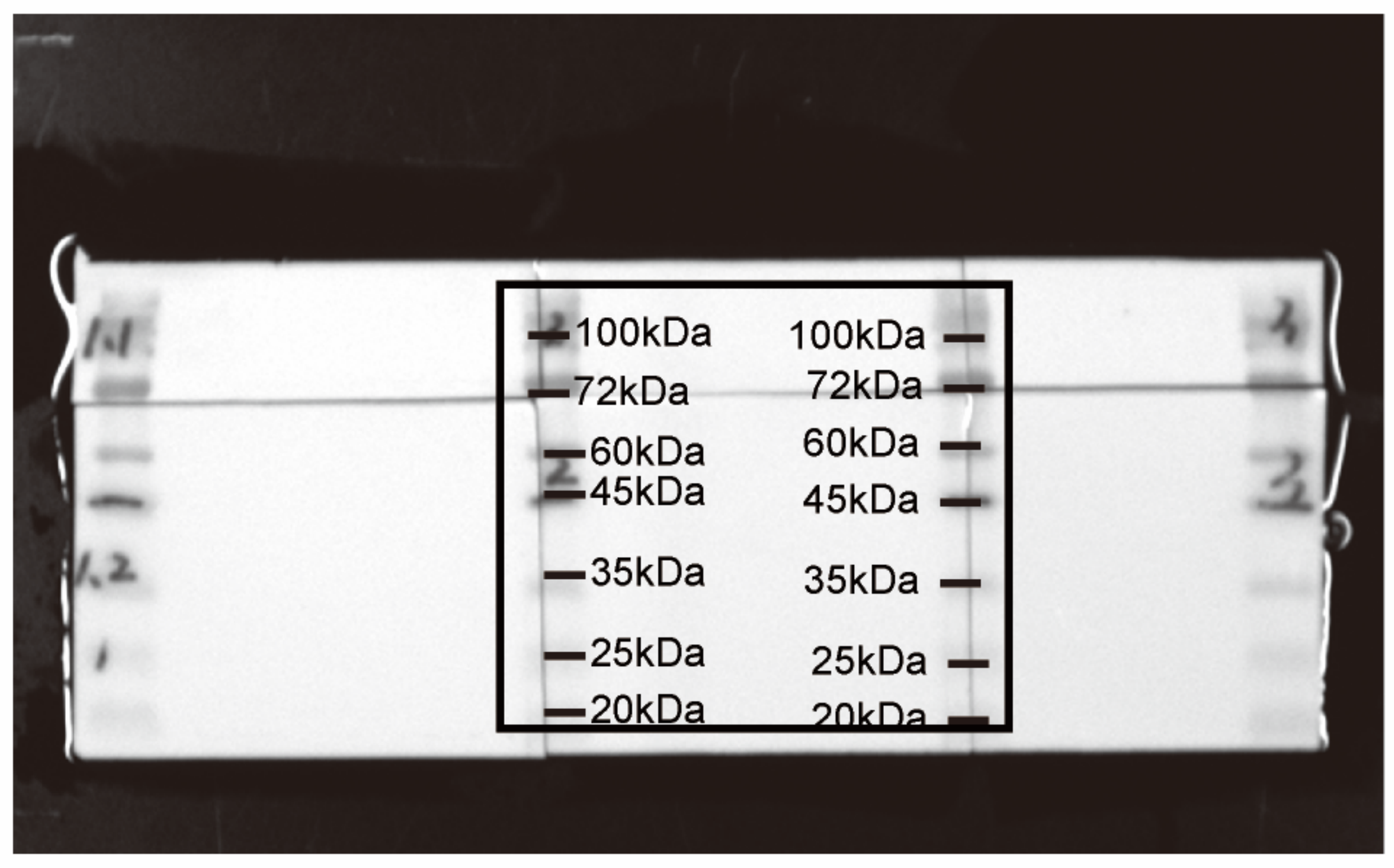

Fig.6C

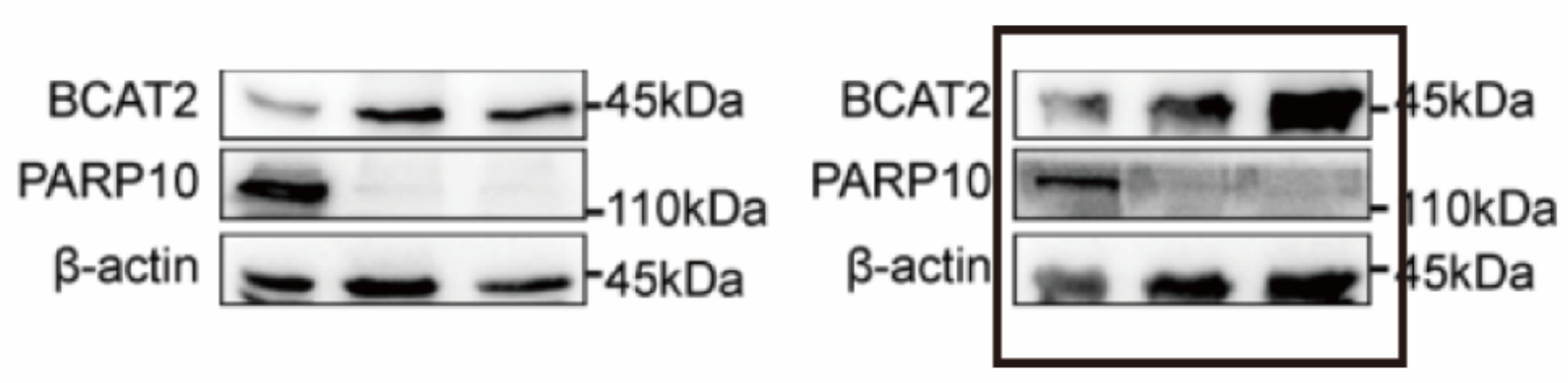

BCAT2

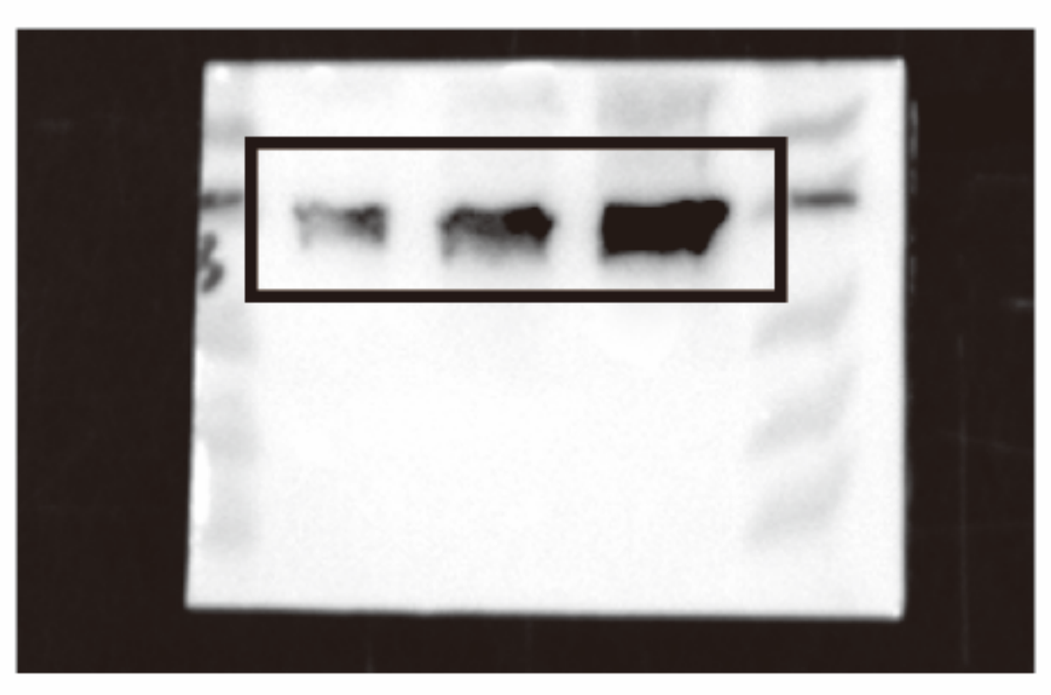

PARP10

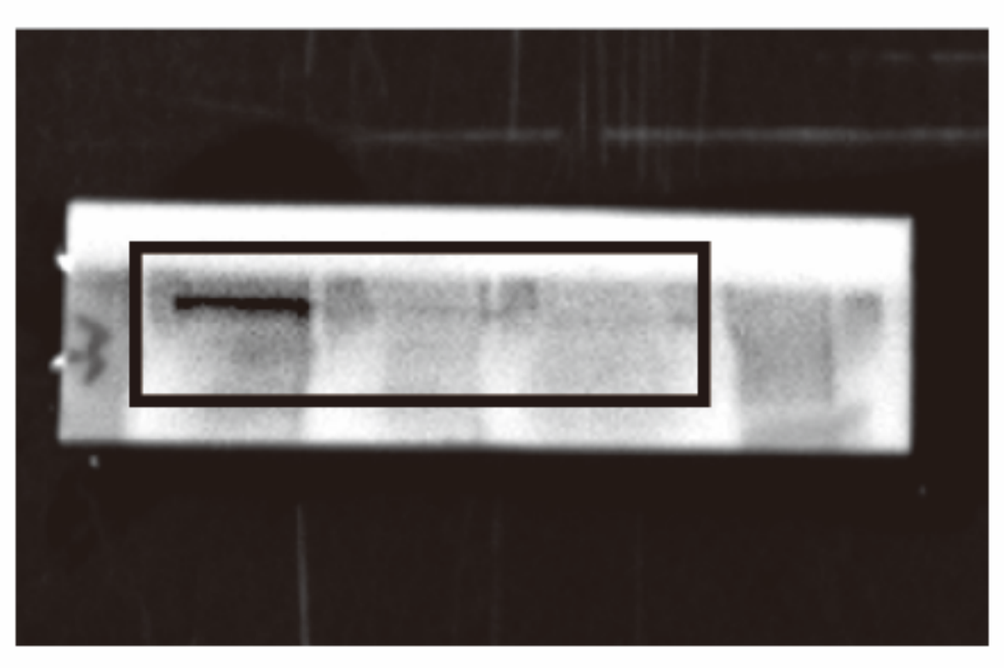

β-actin

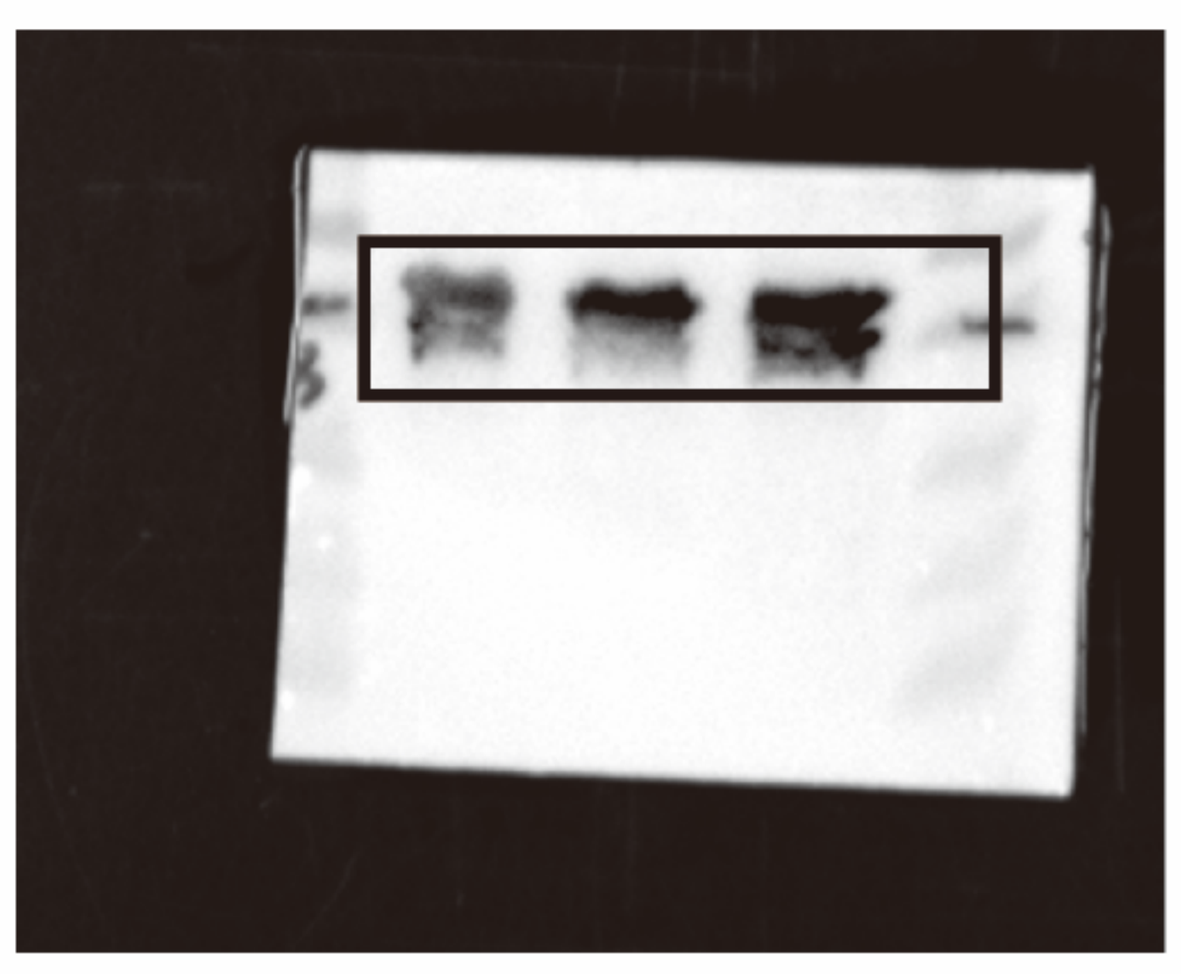

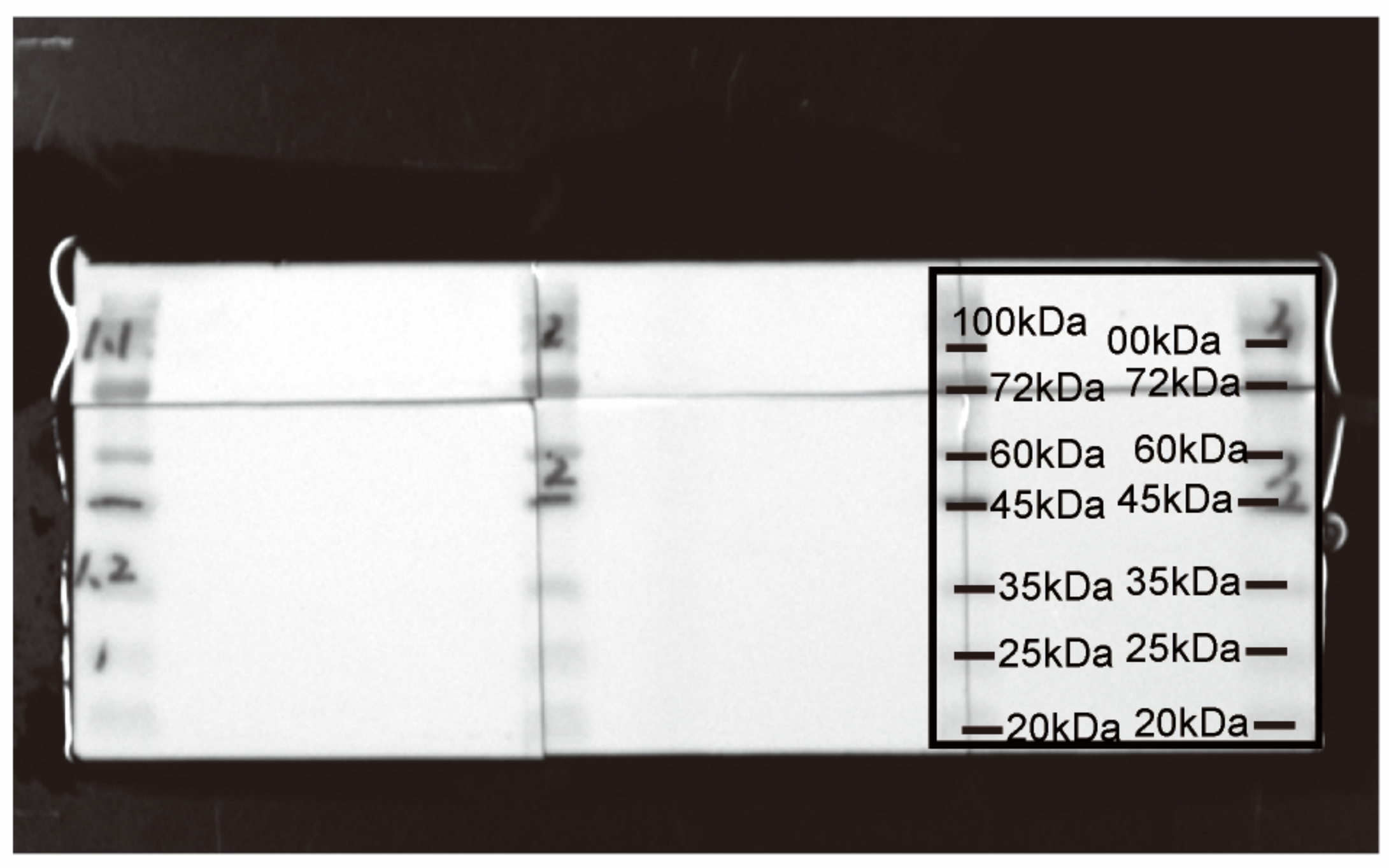

Figure	Merged	Stiched full image

Fig.6D

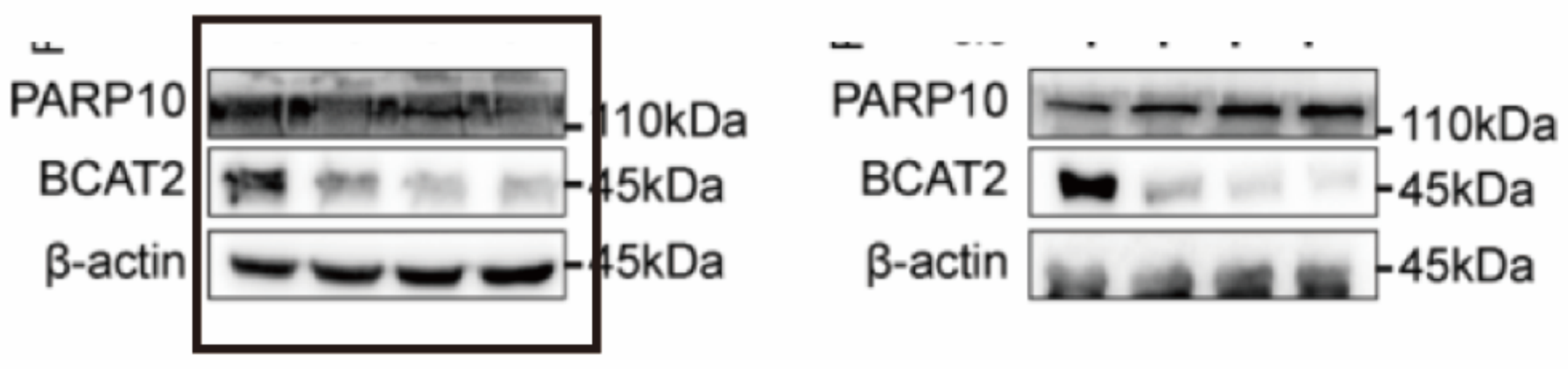

PARP10 BCAT2

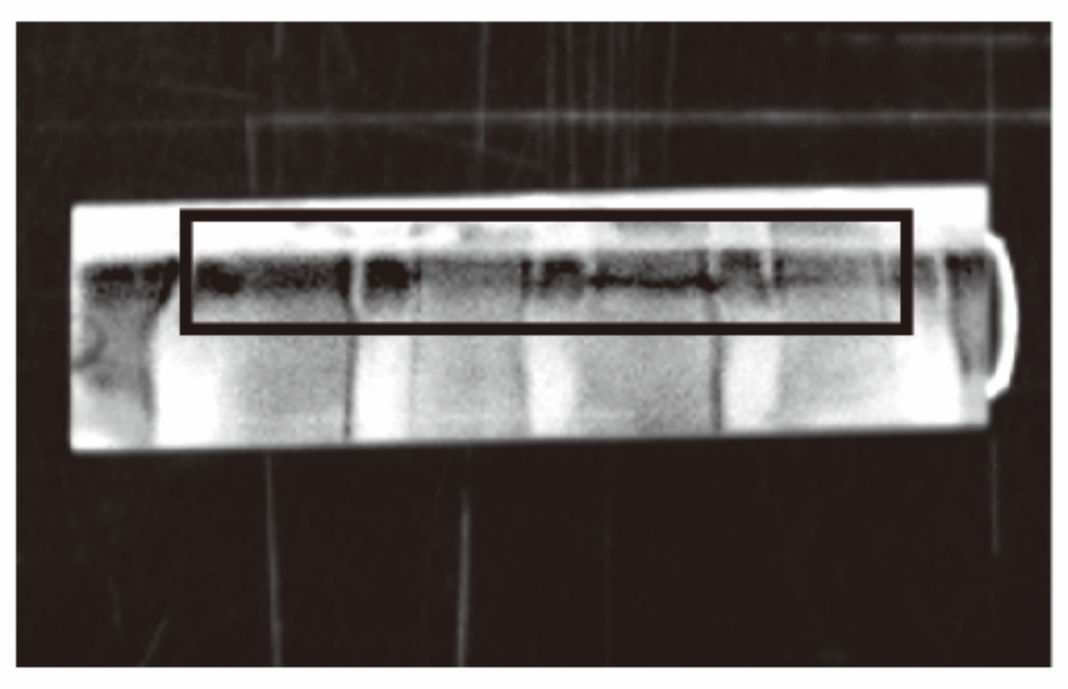

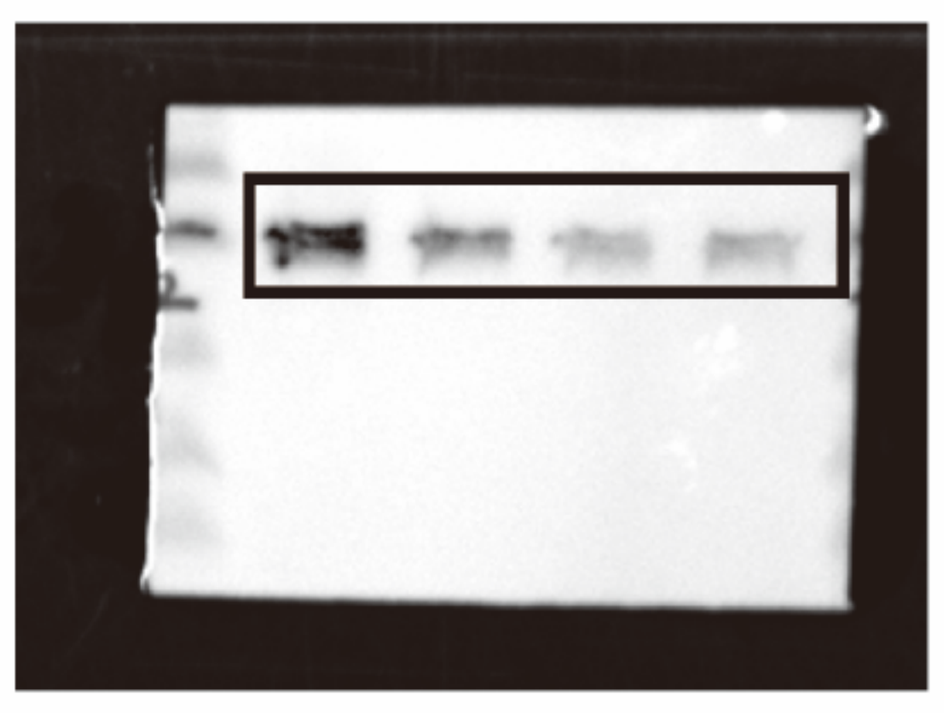

β-actin

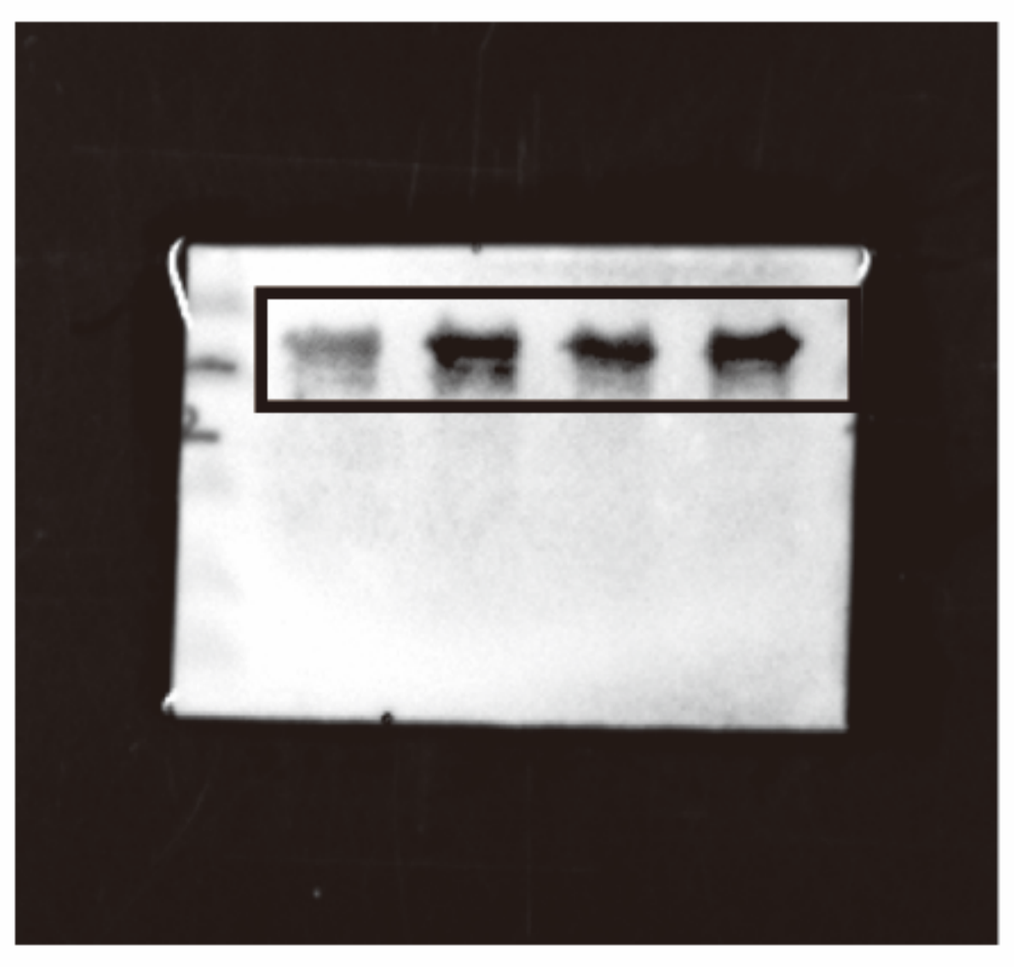

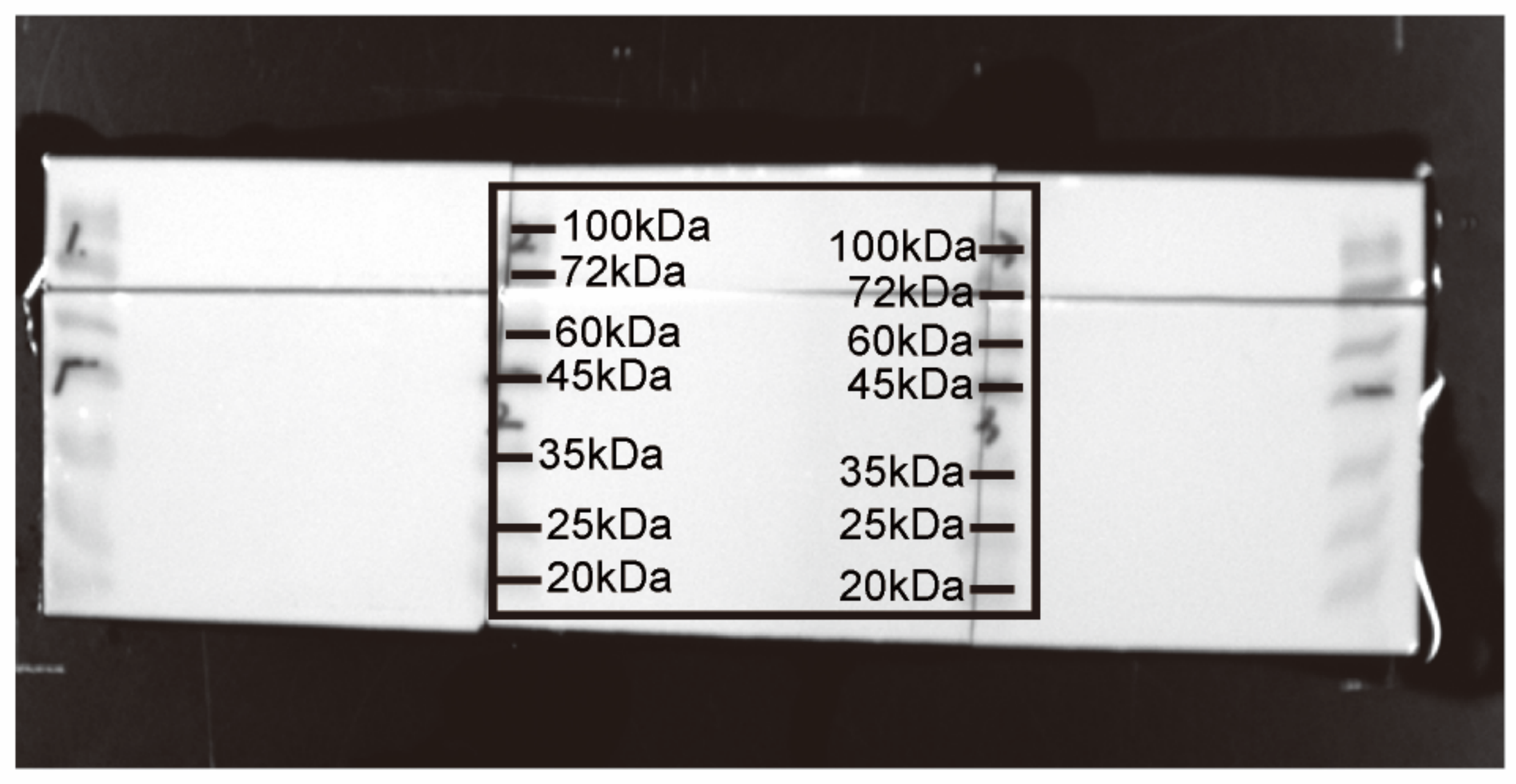

Fig.6D

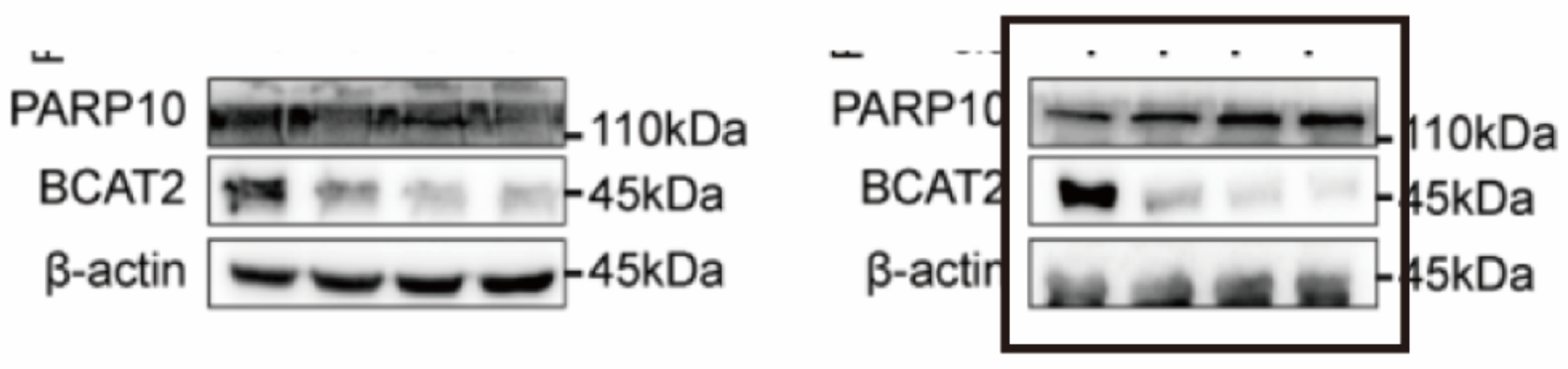

PARP10 BCAT2

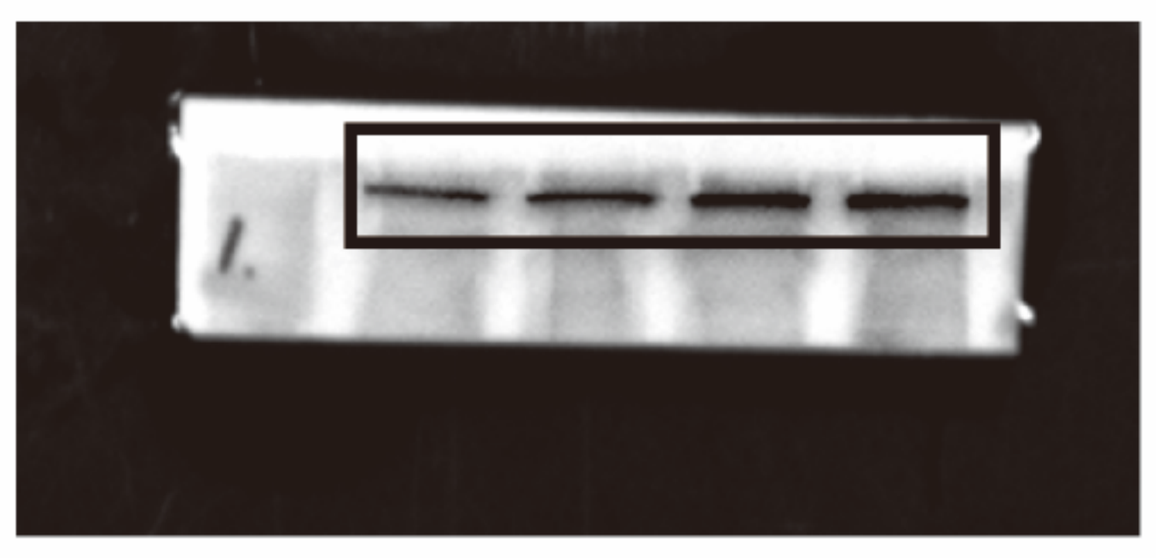

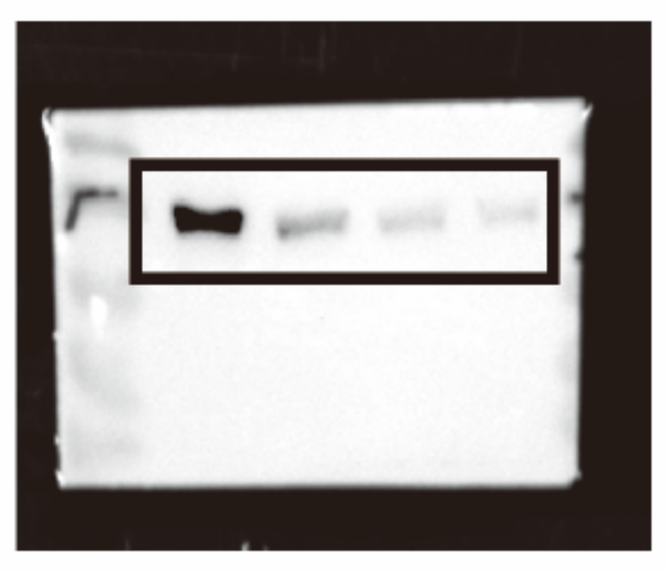

β-actin

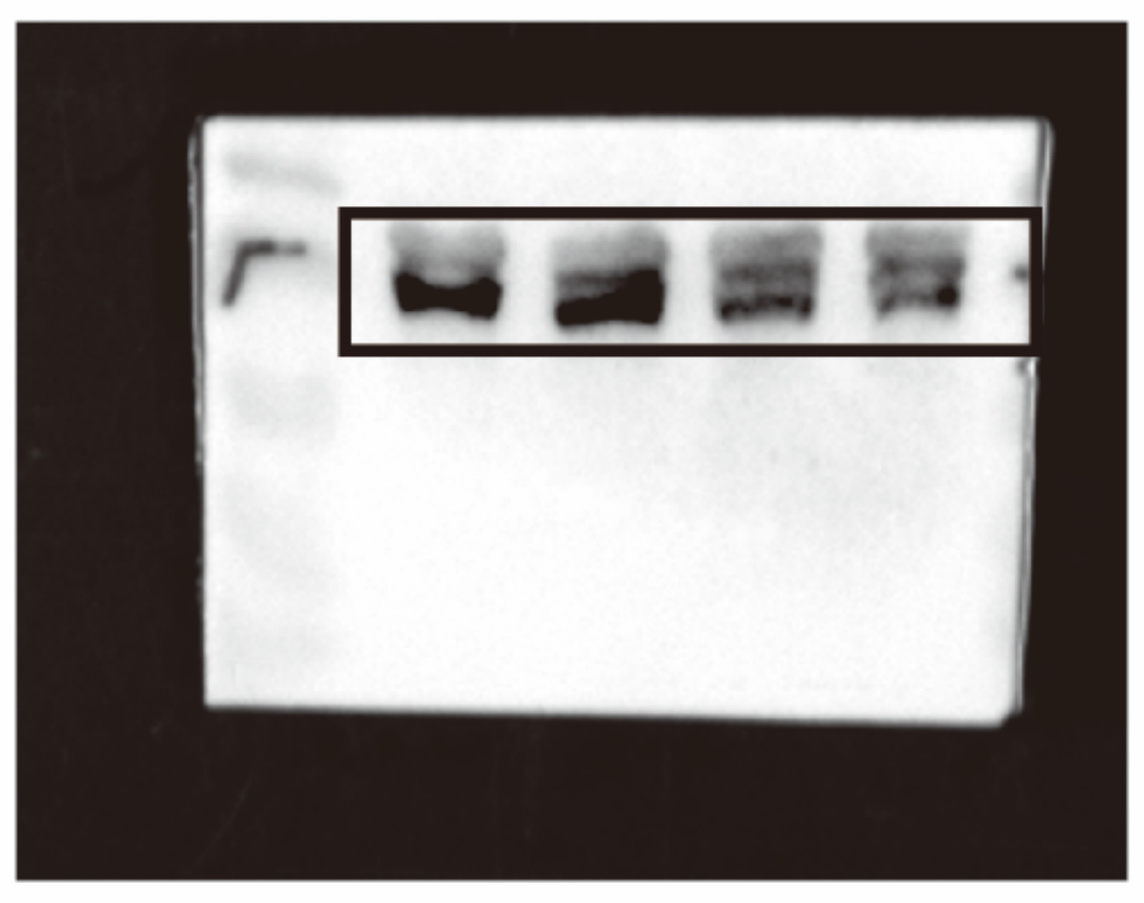

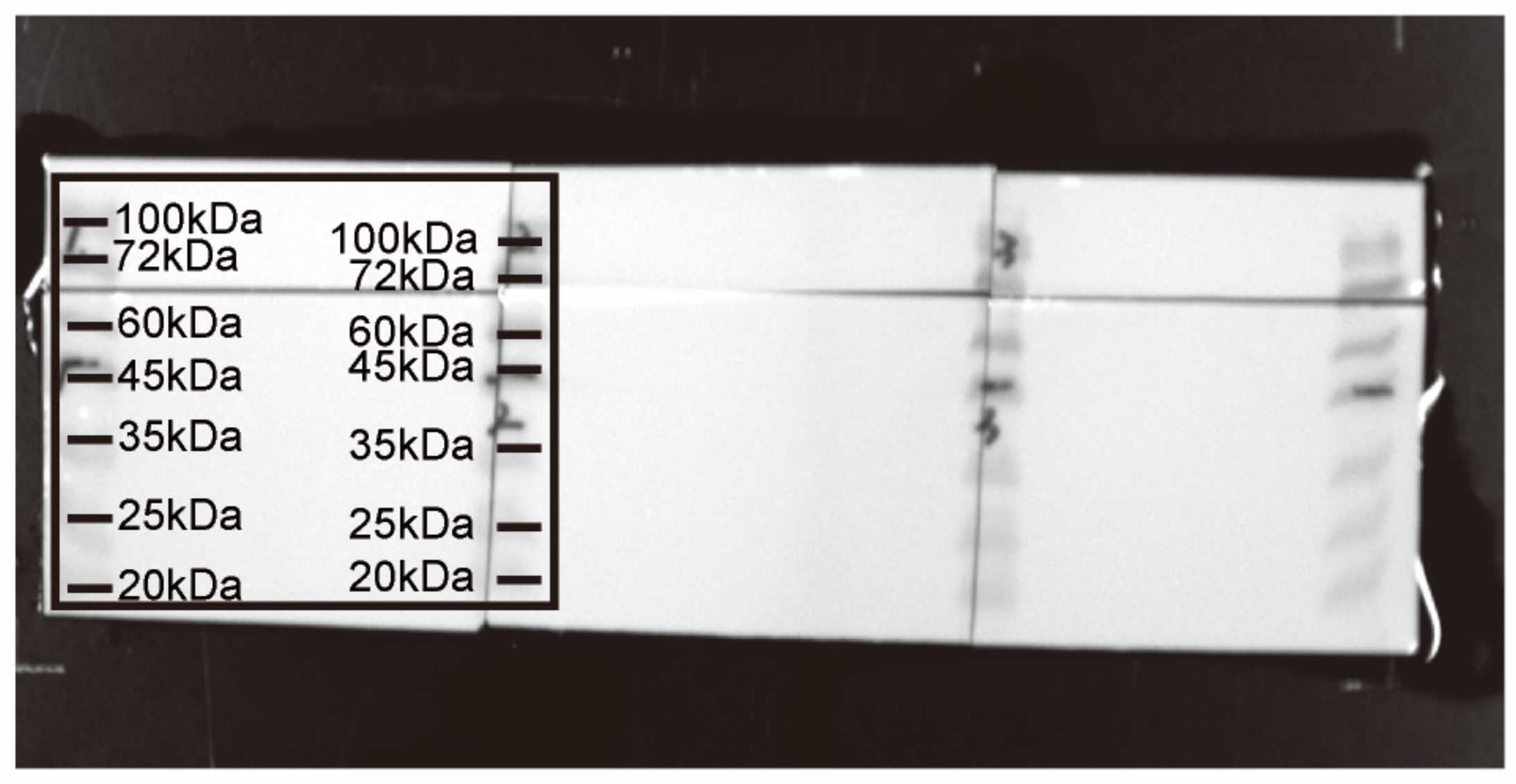

Fig.7C

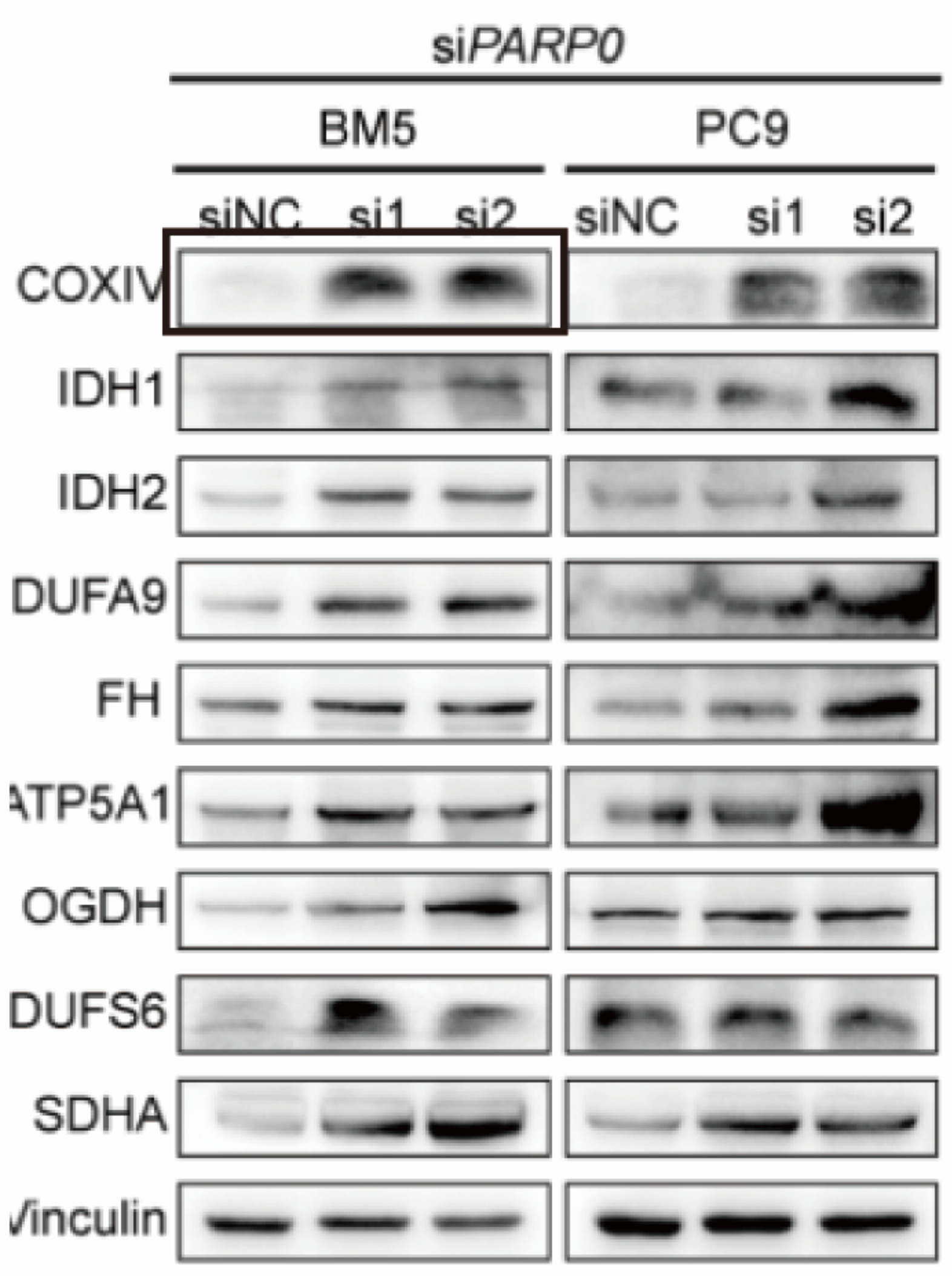

COXIV

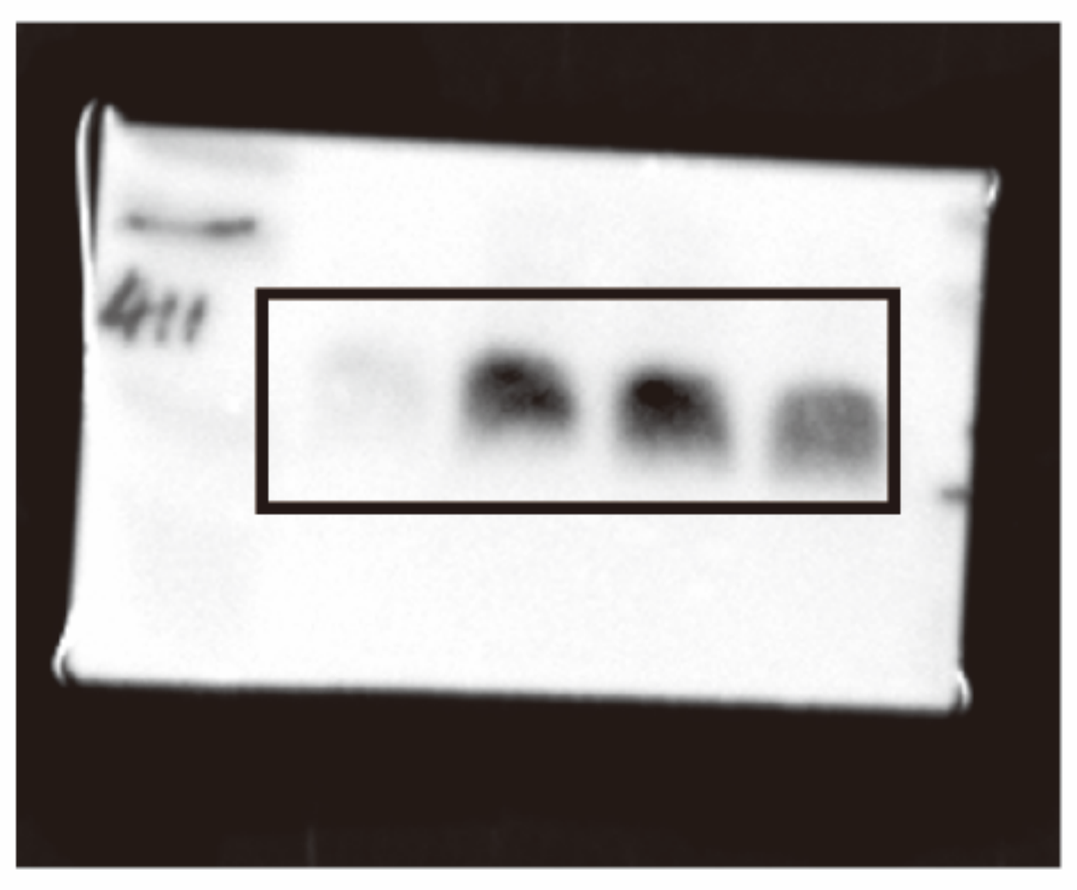

Vinculin

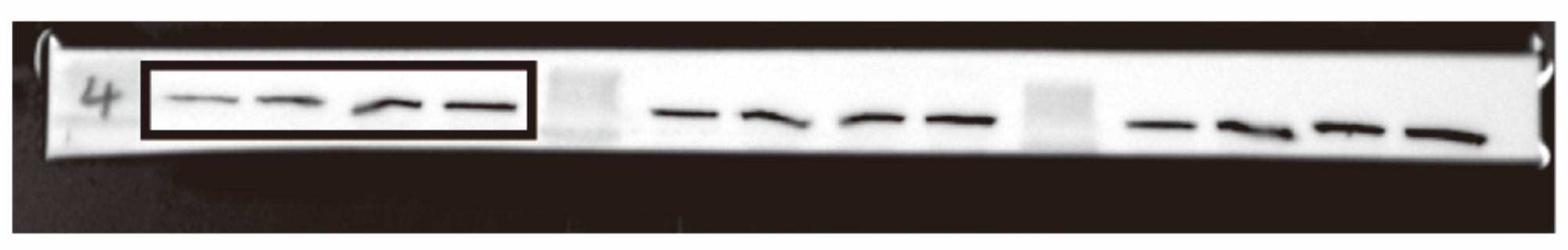

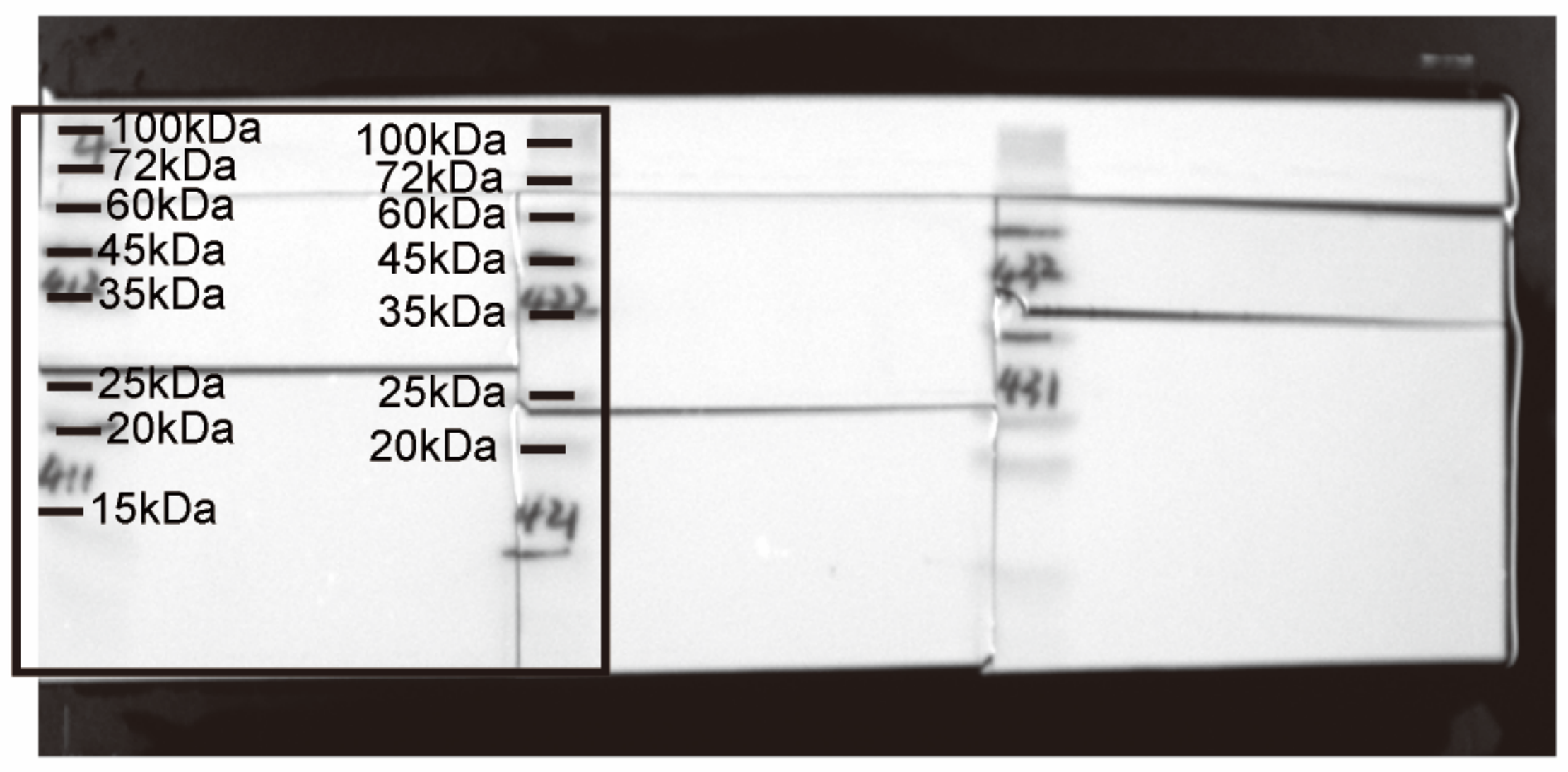

Fig.7C

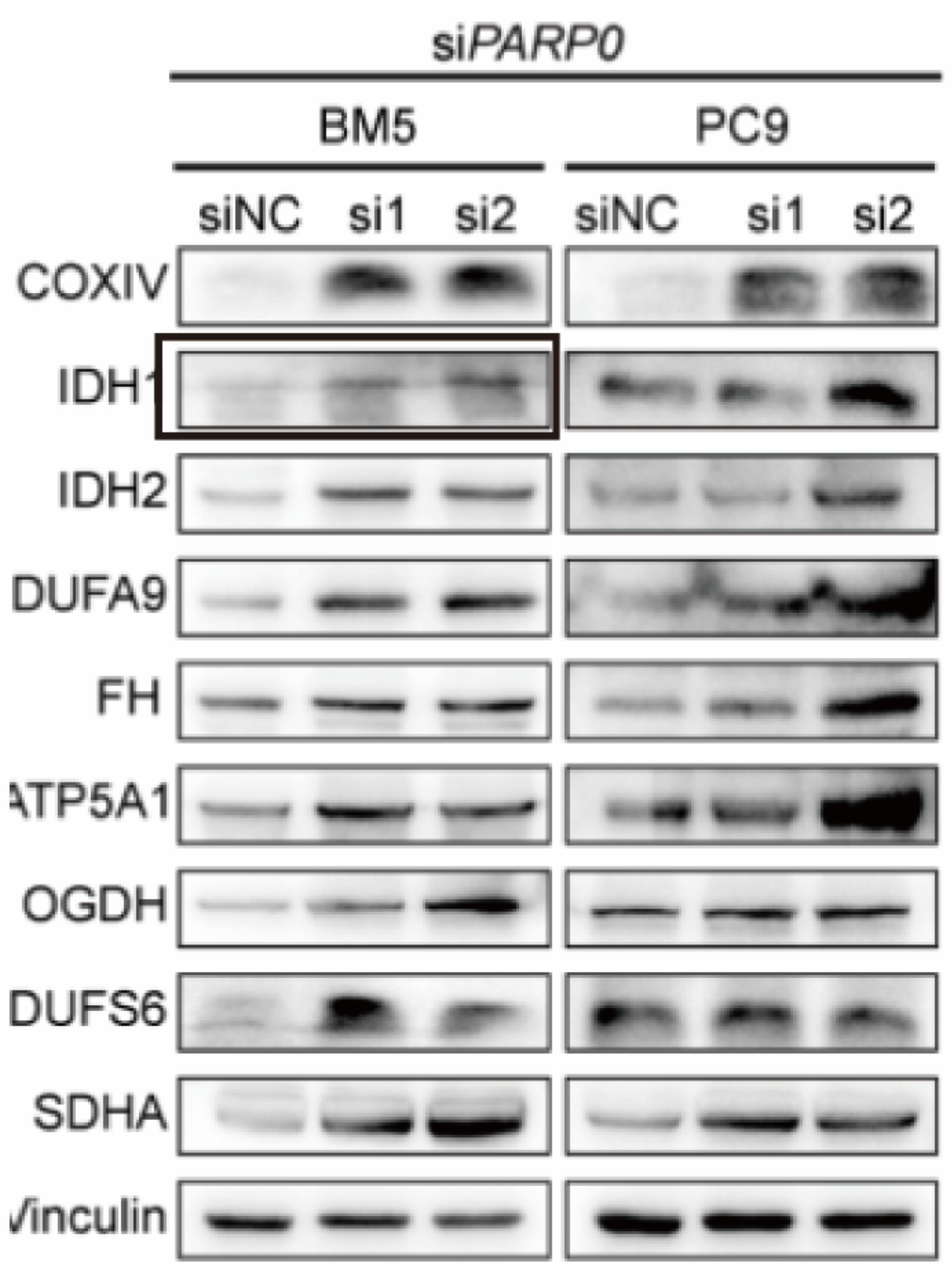

Vinculin

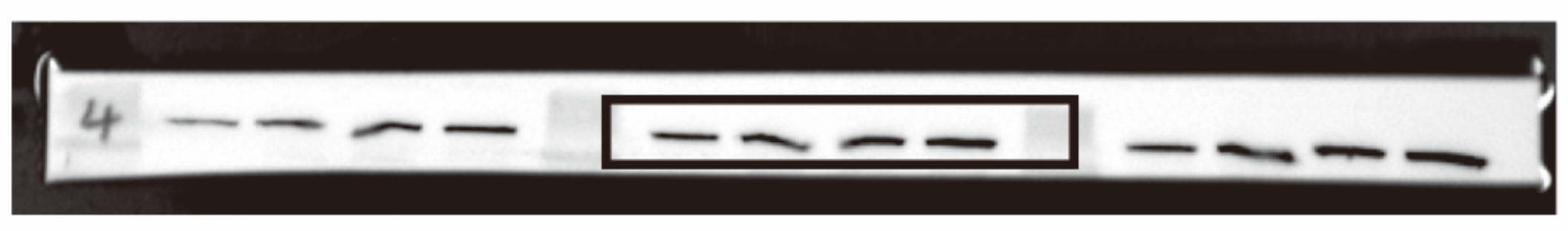

IDH1

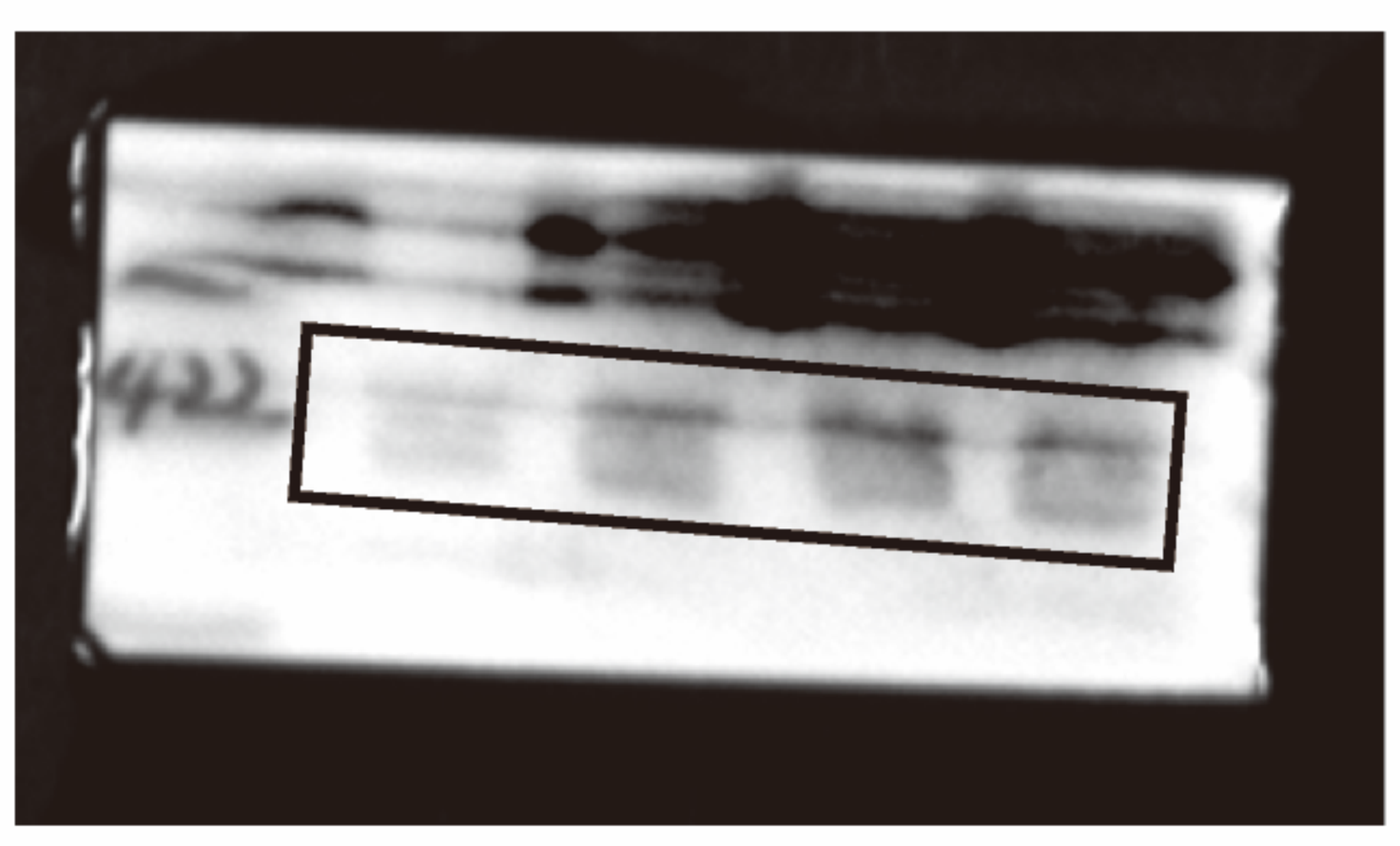

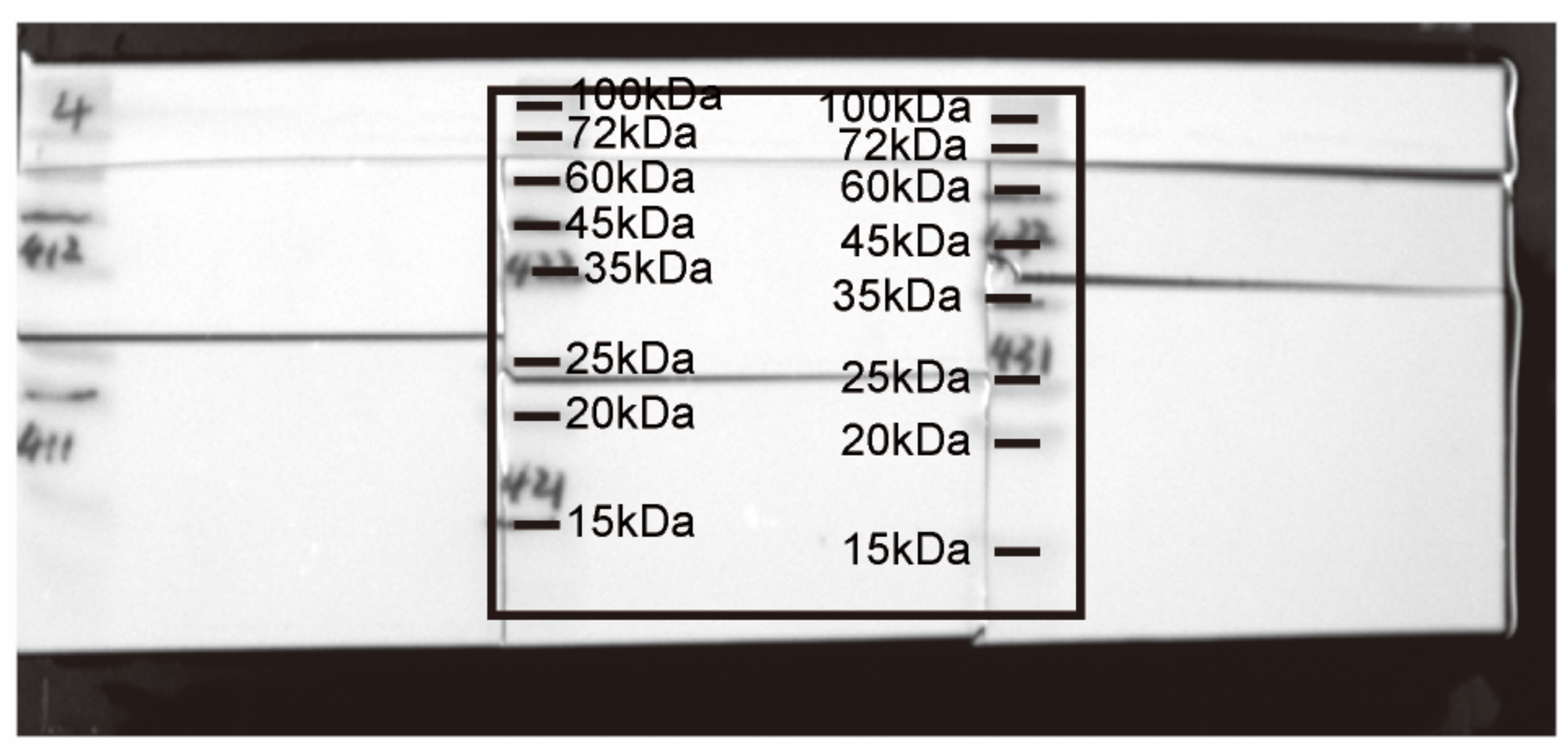

Figure	Merged	Stiched full image

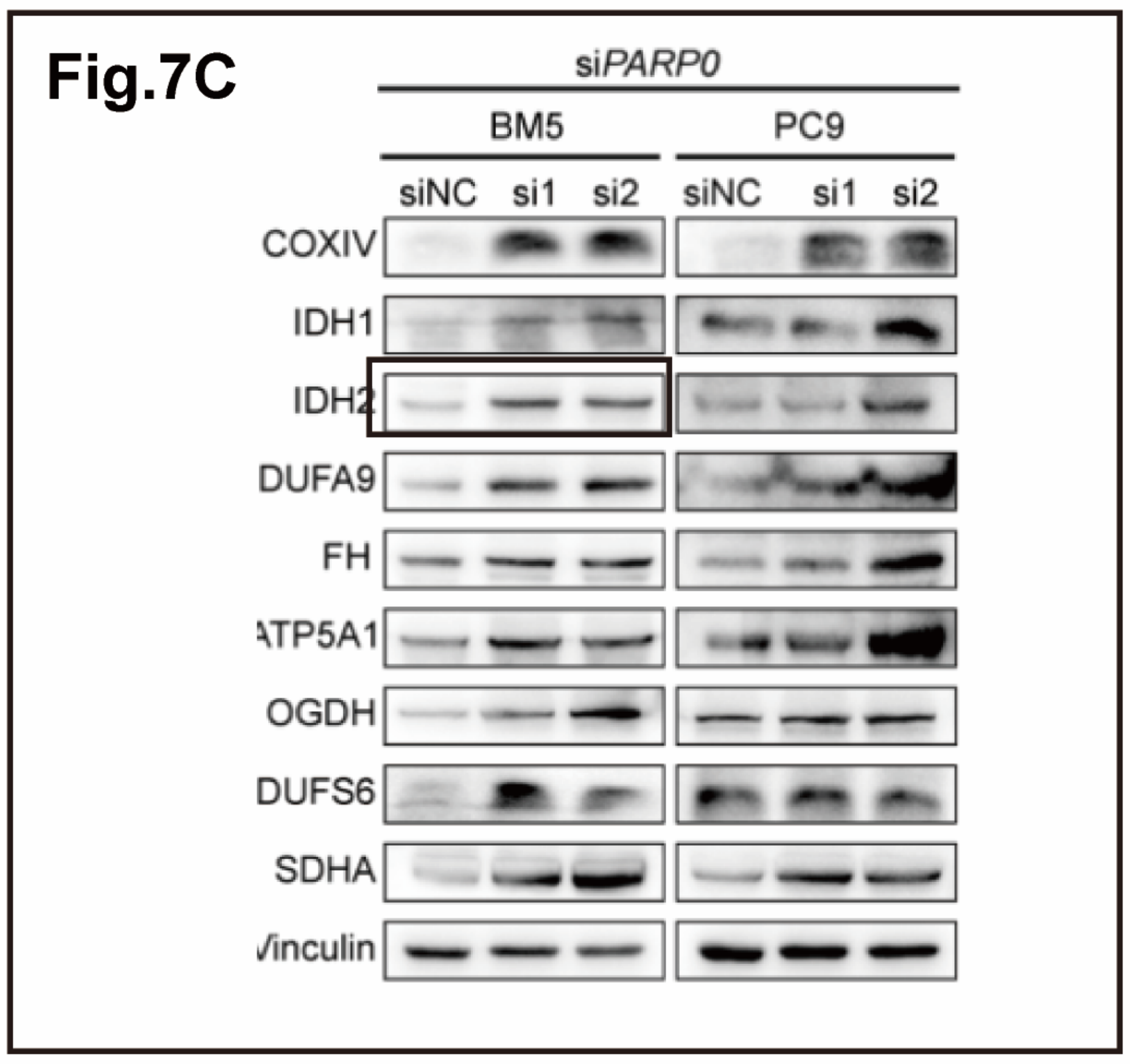

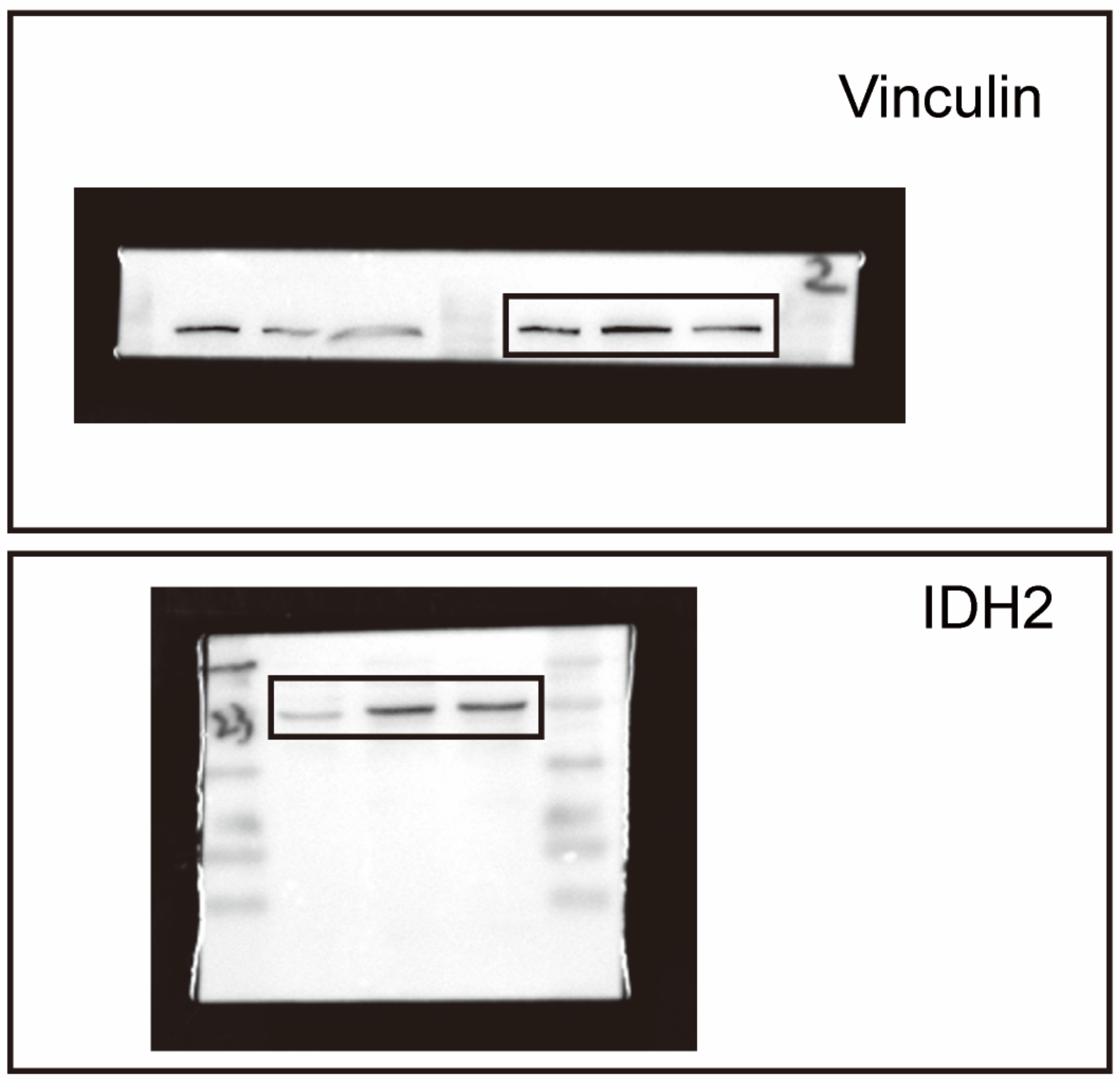

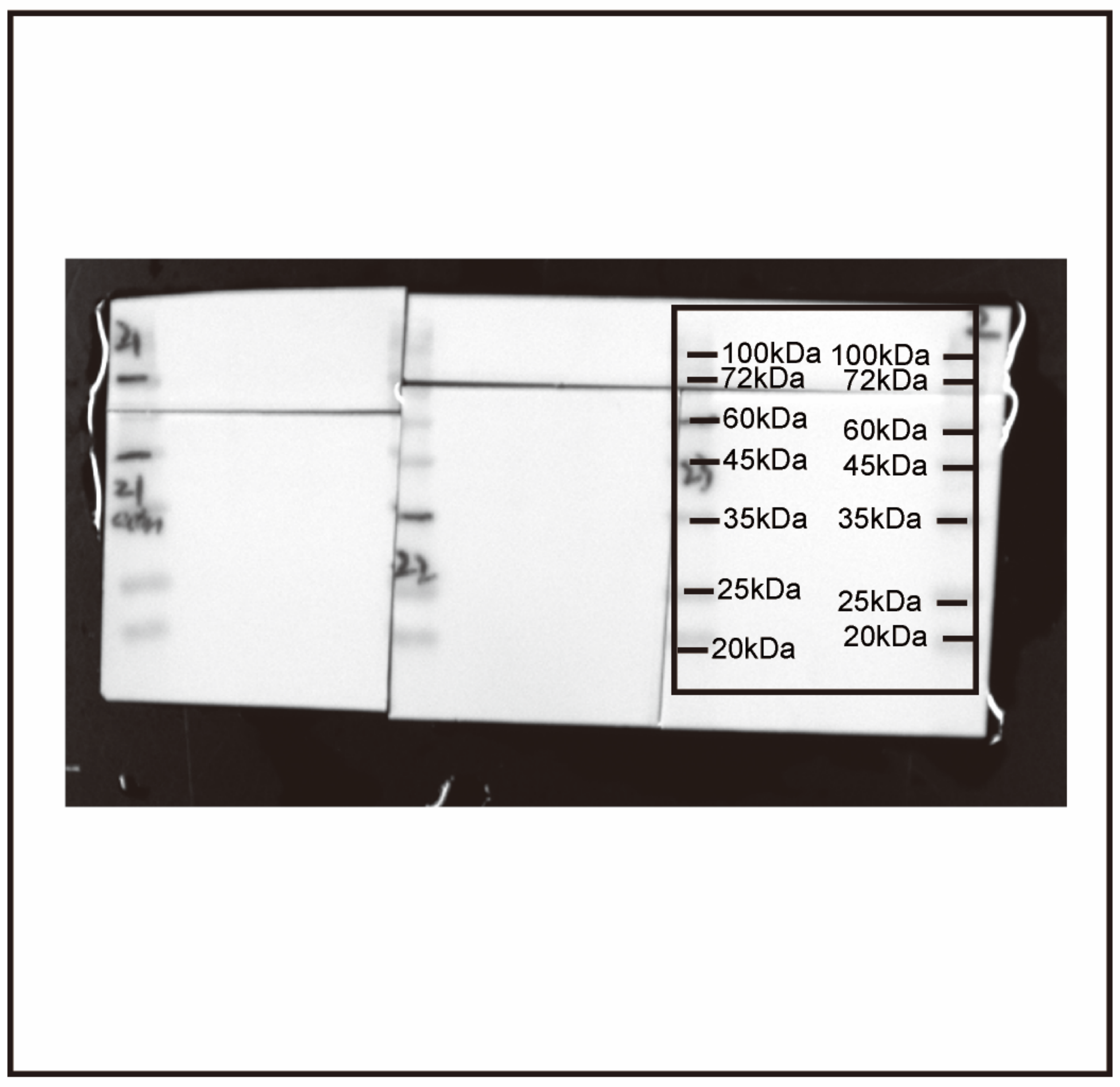

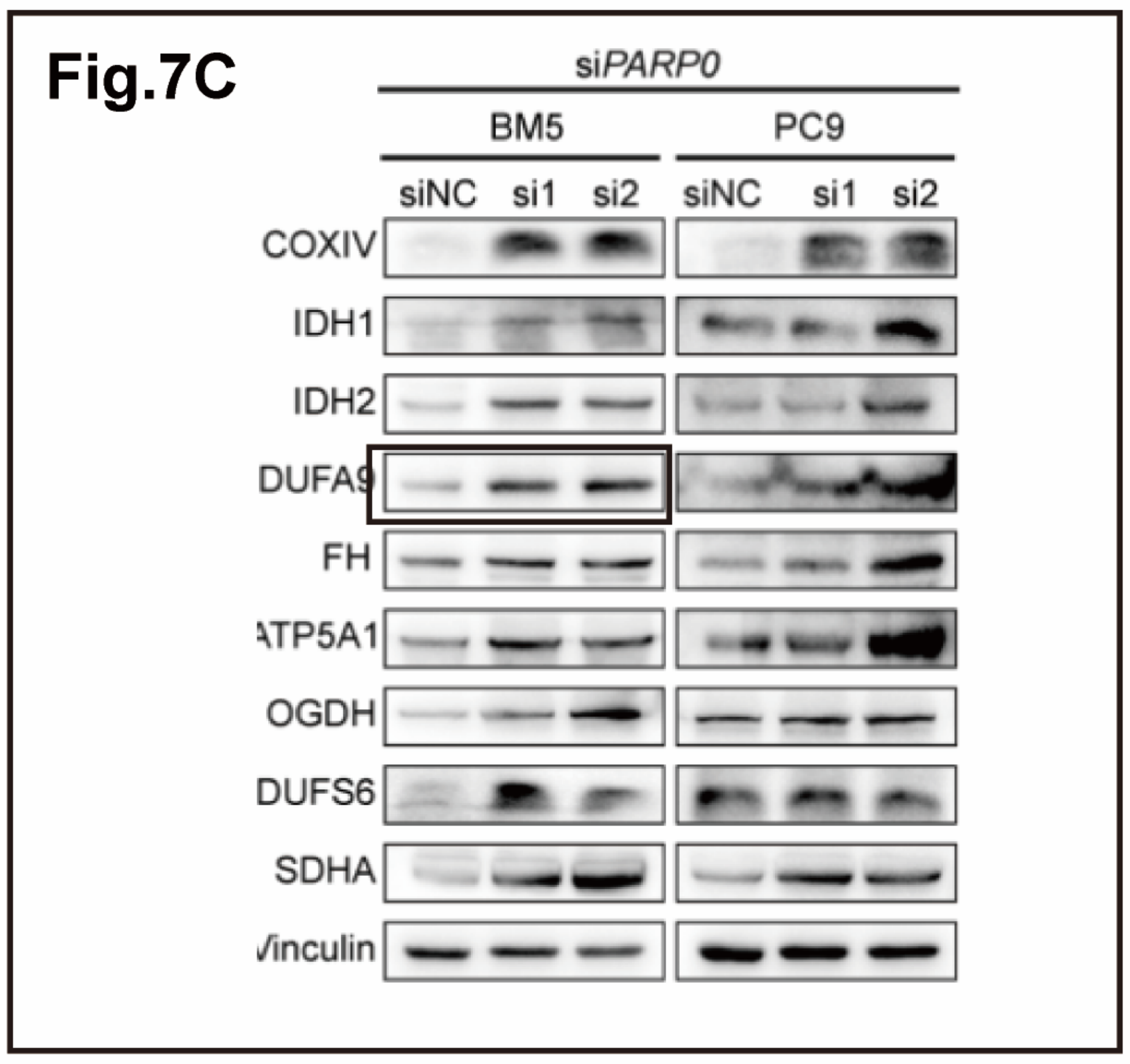

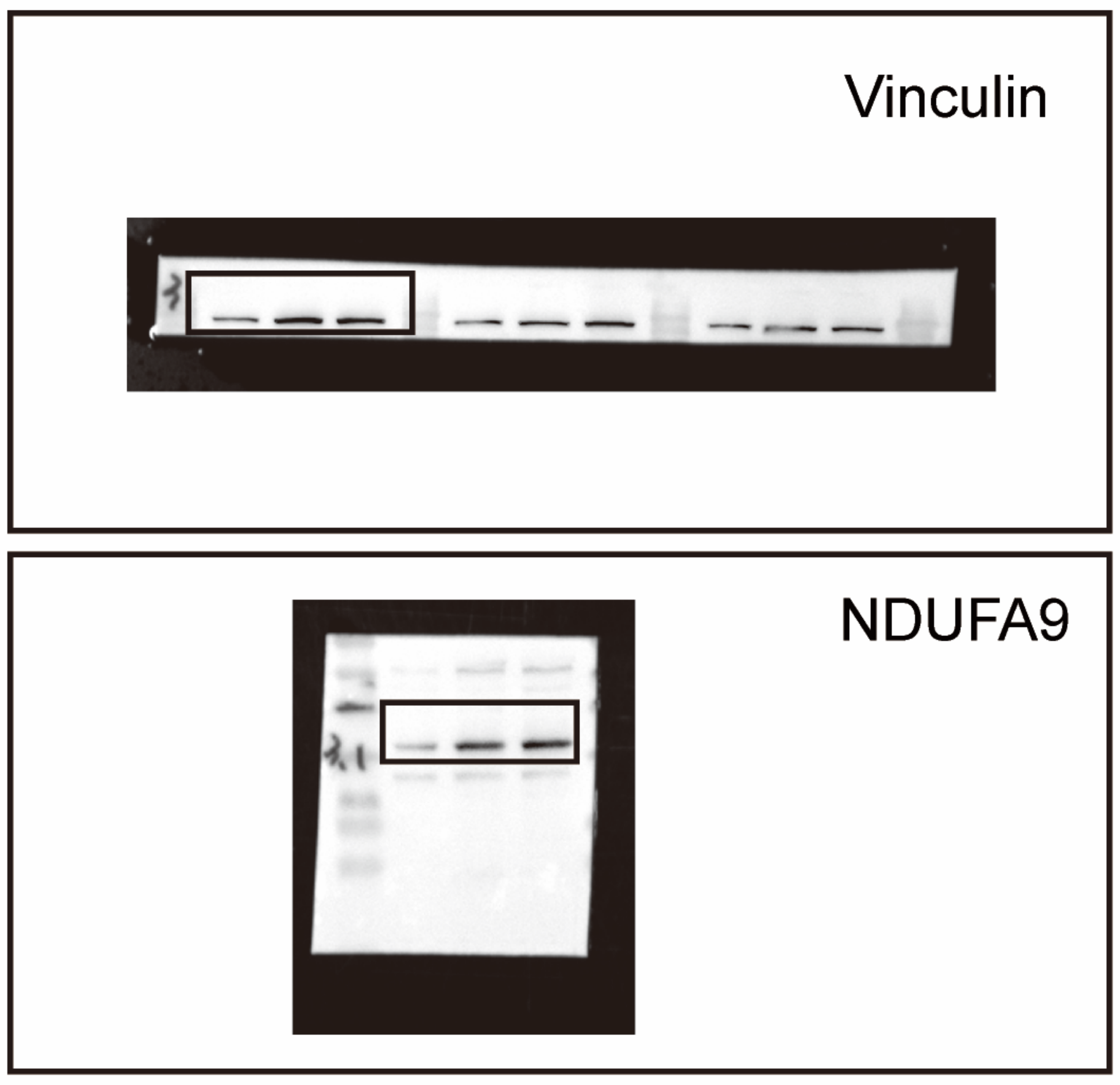

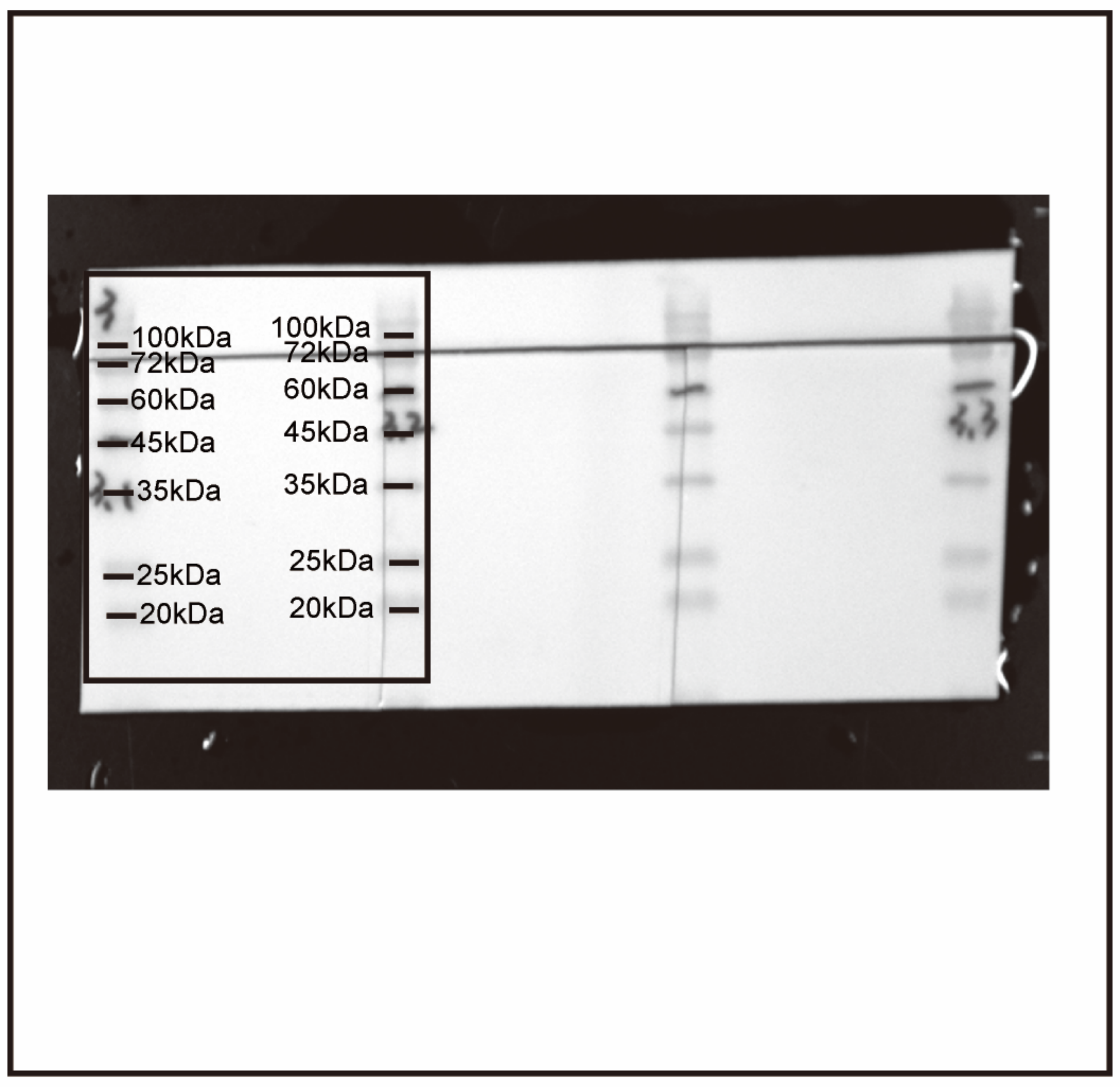

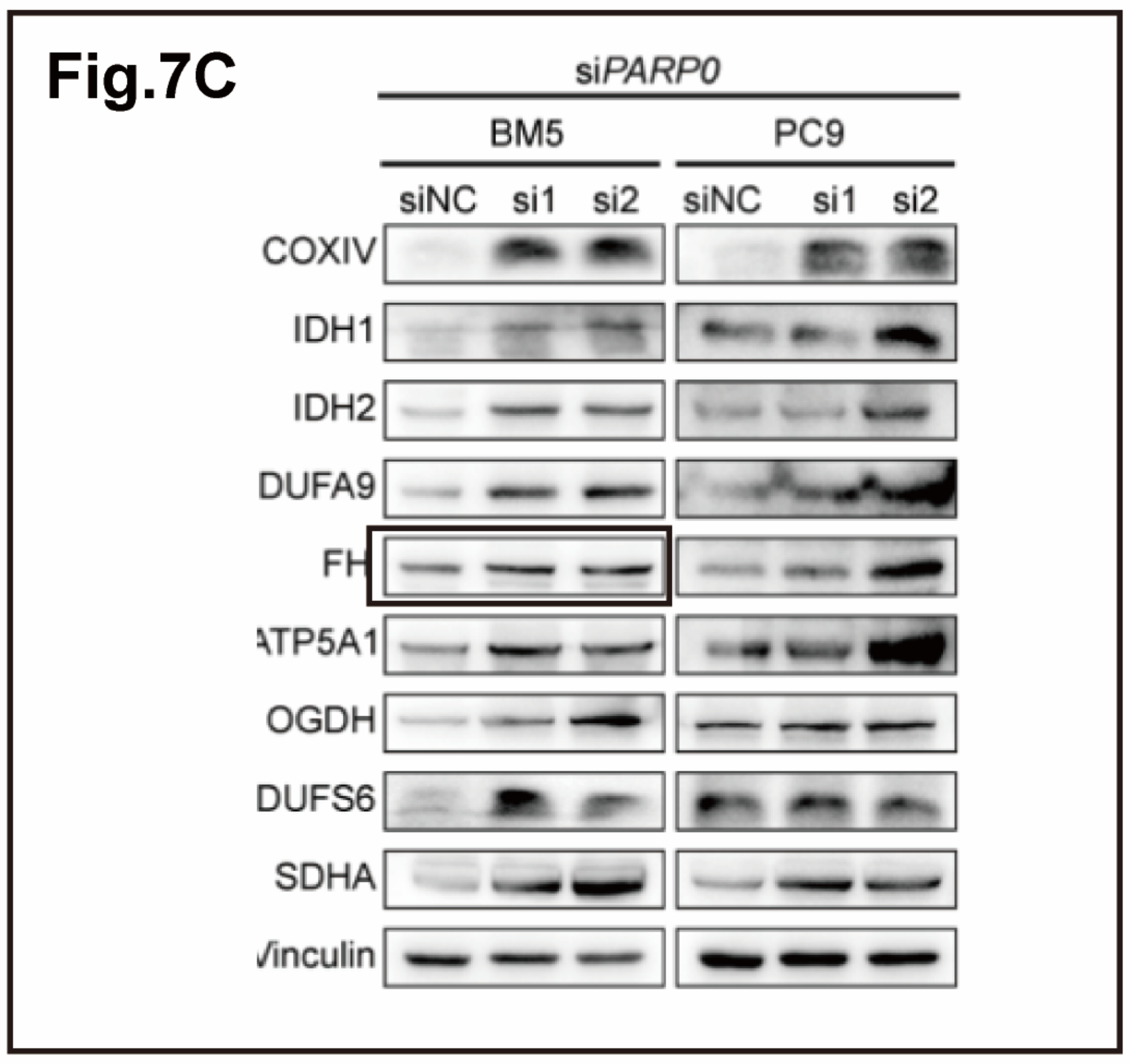

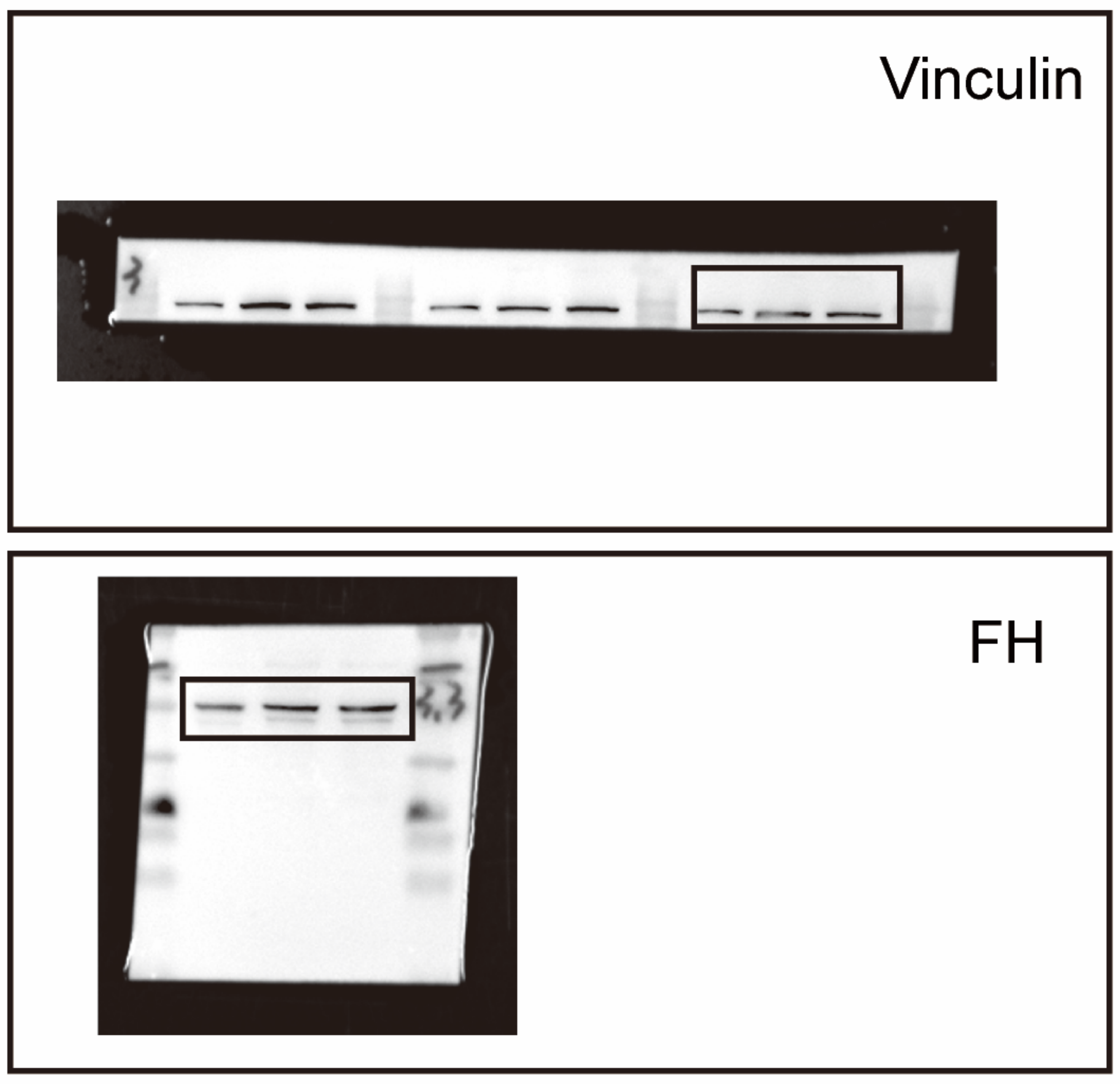

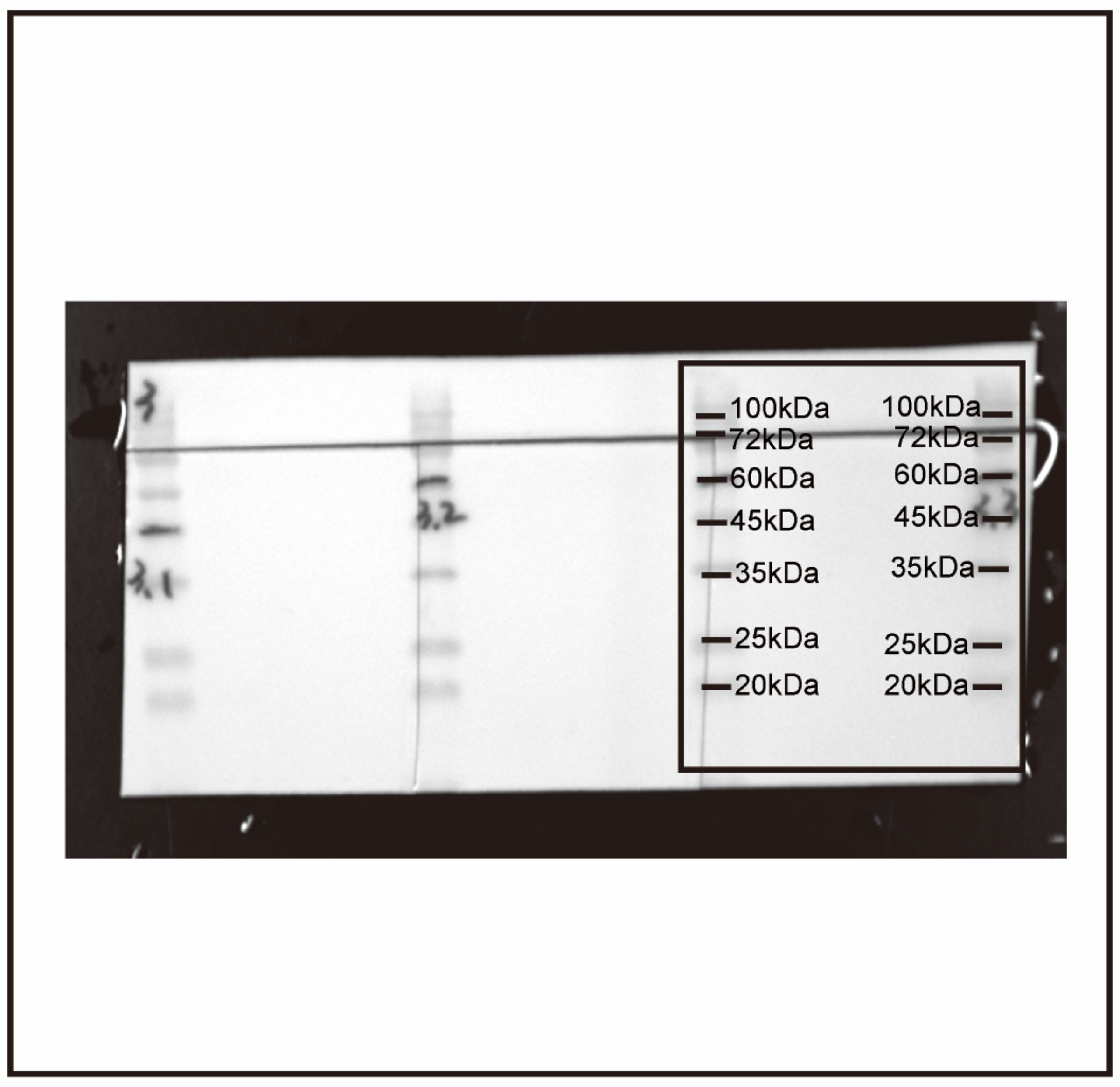

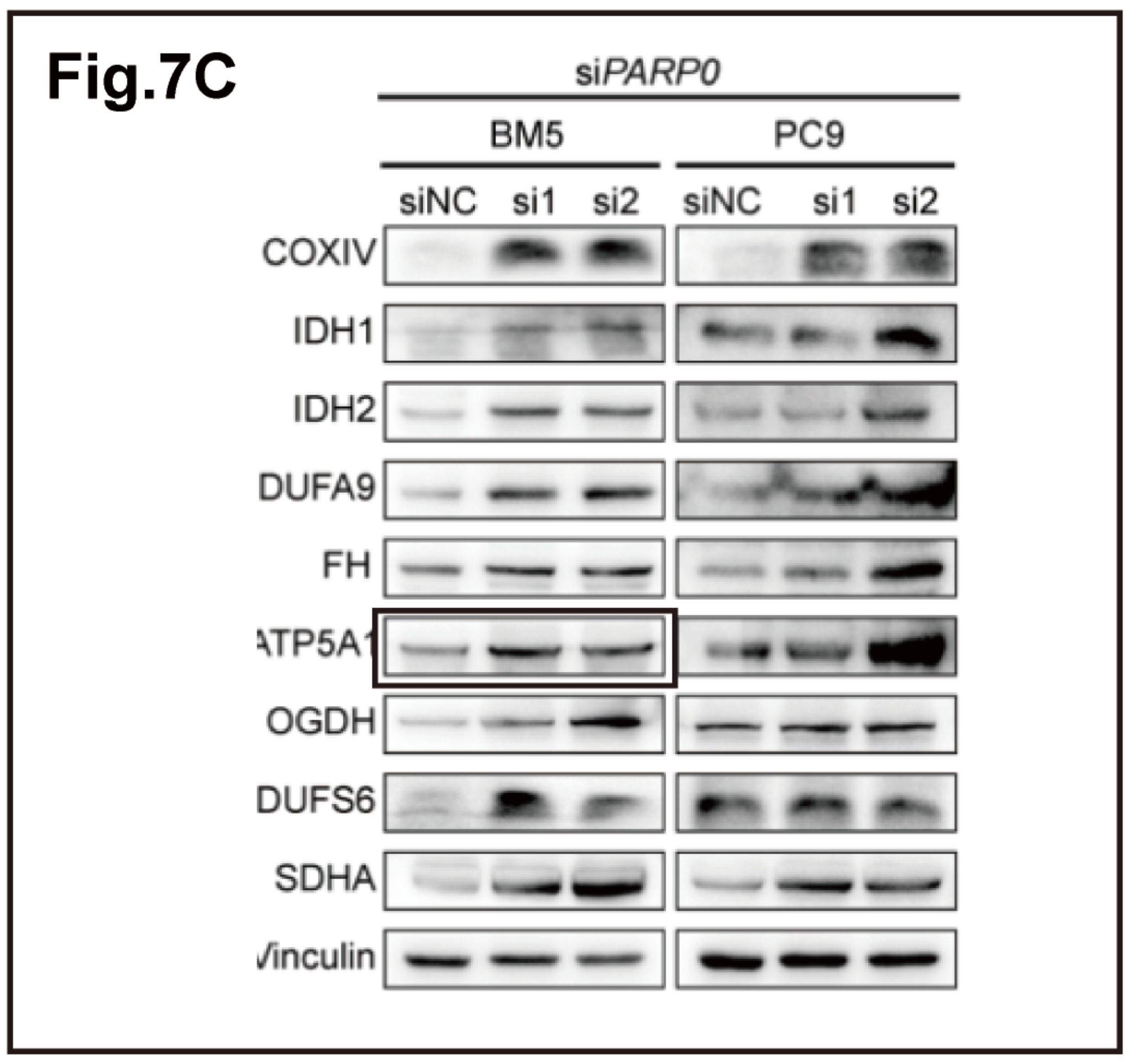

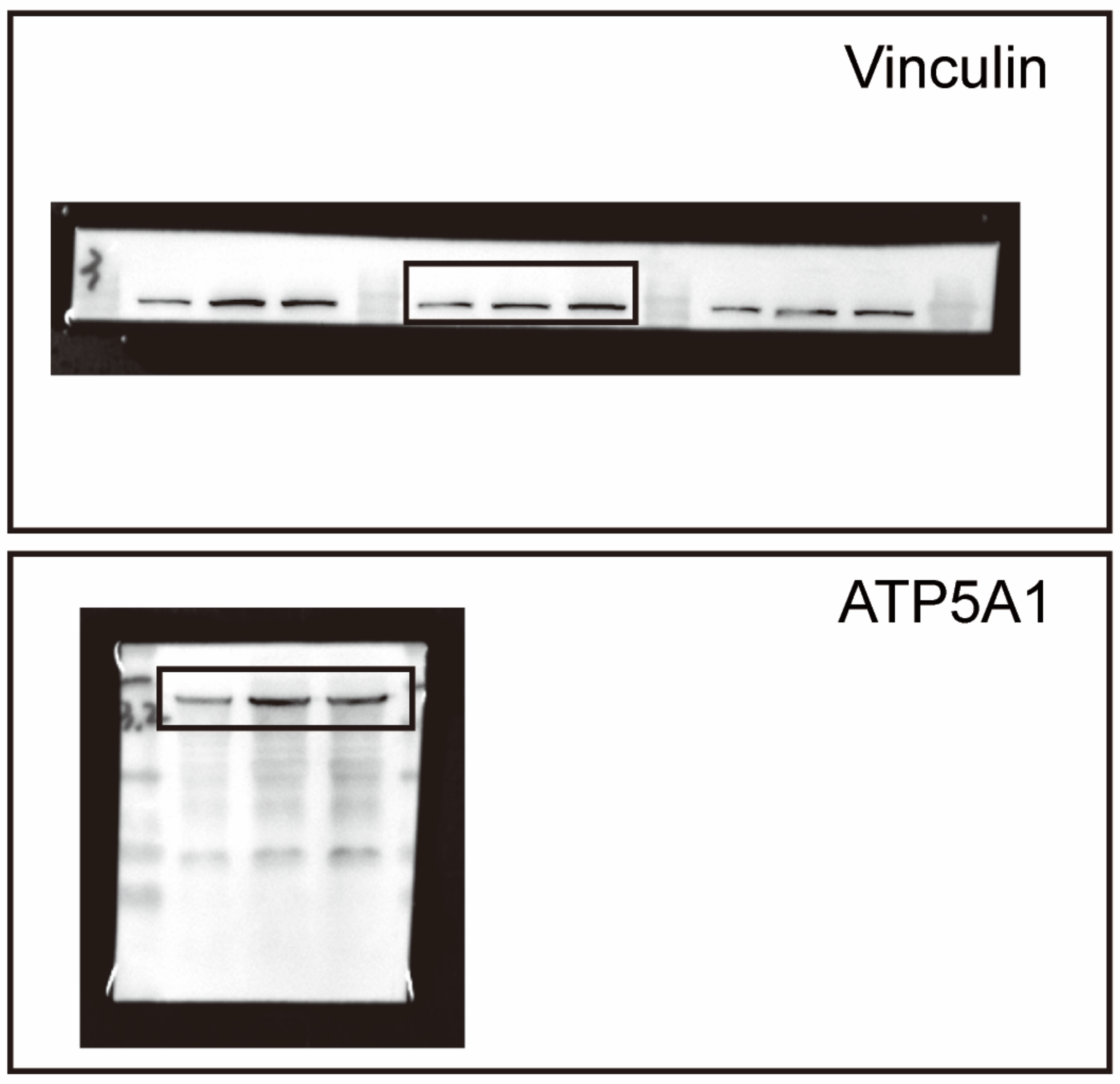

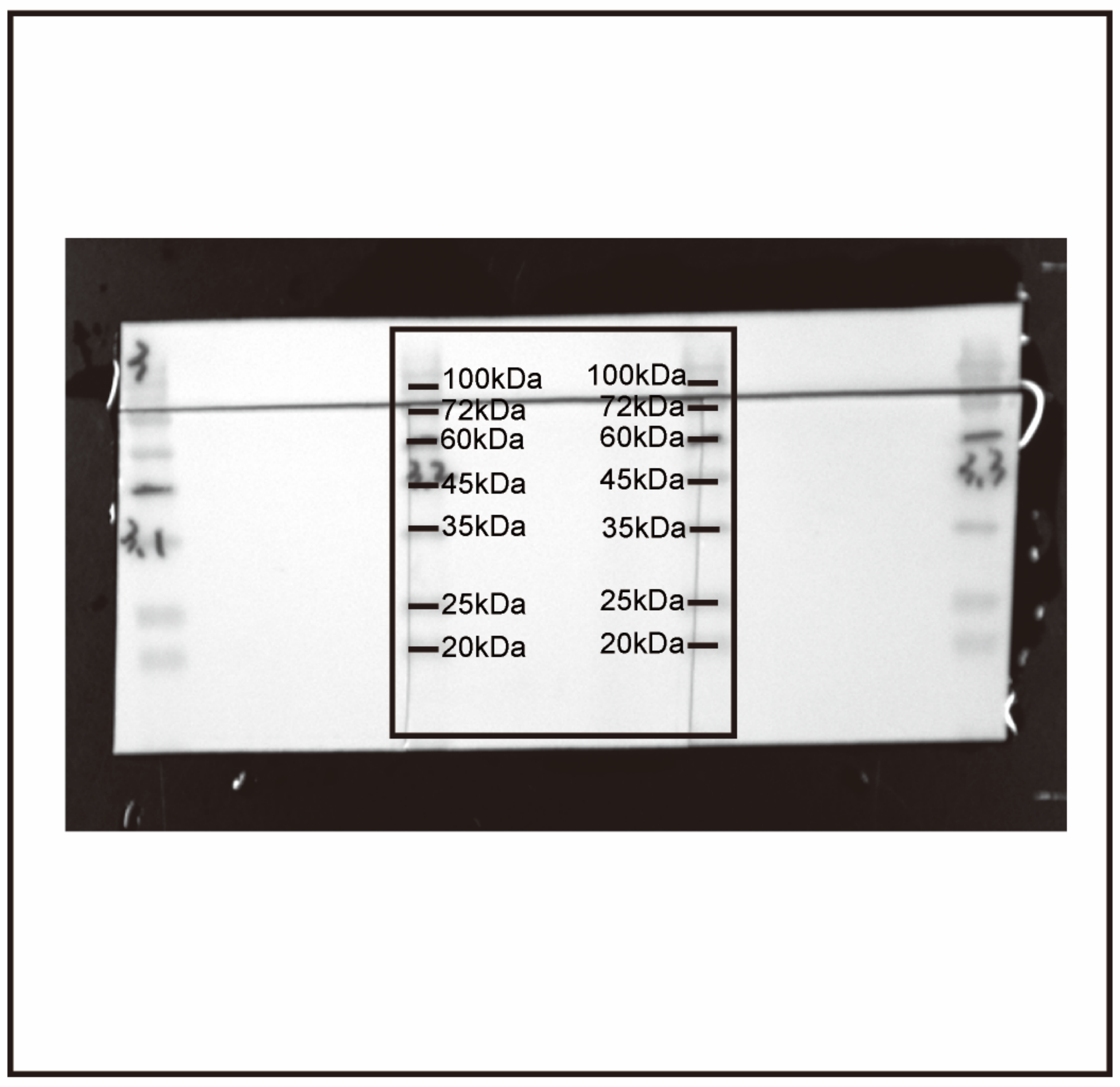

Figure	Merged	Stiched full image

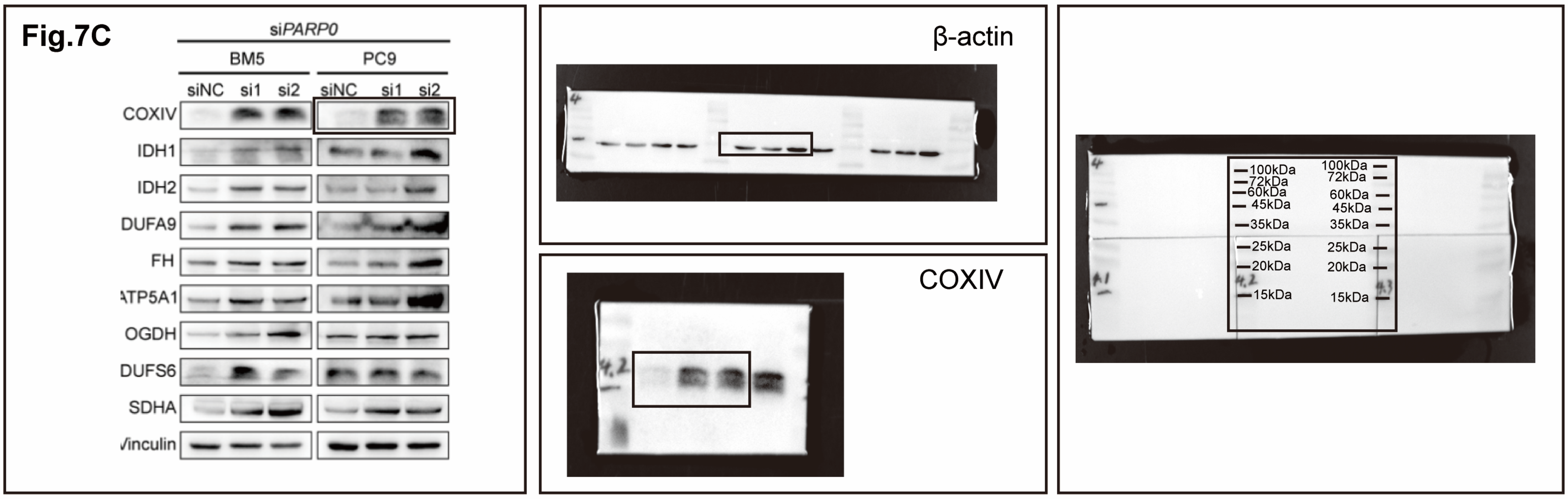

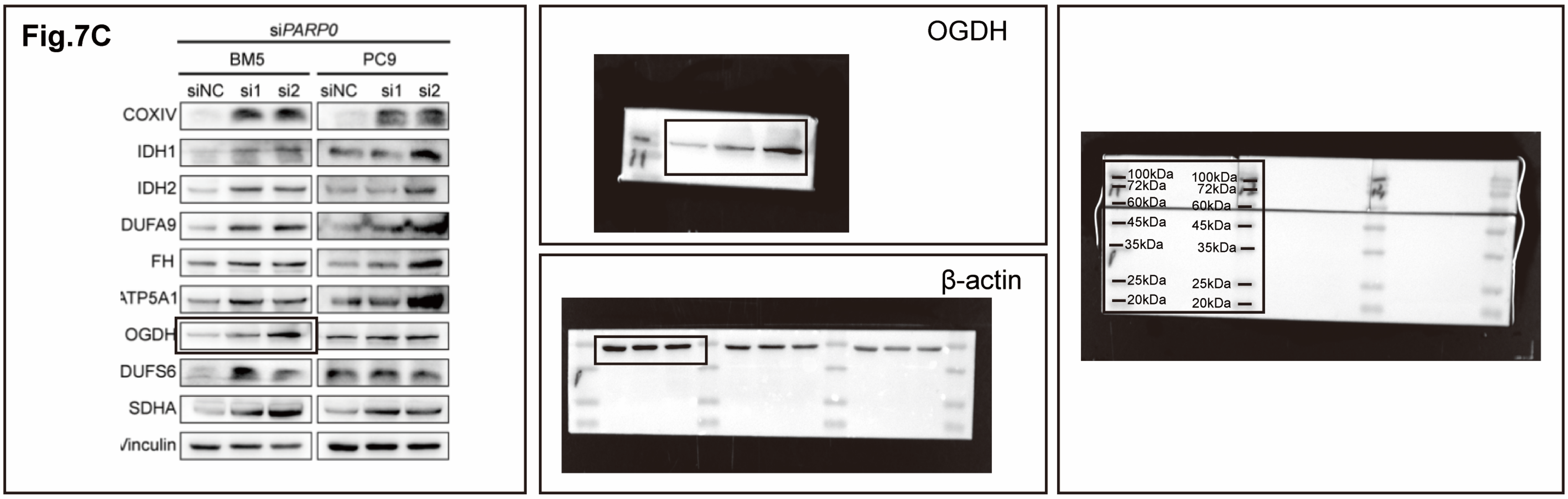

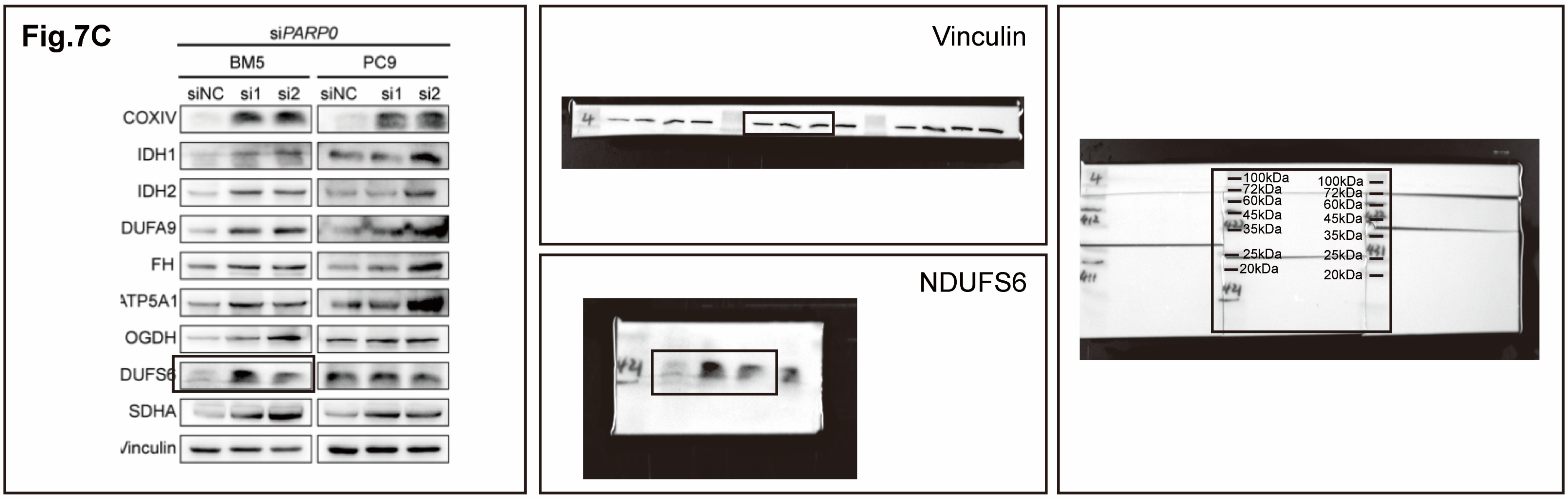

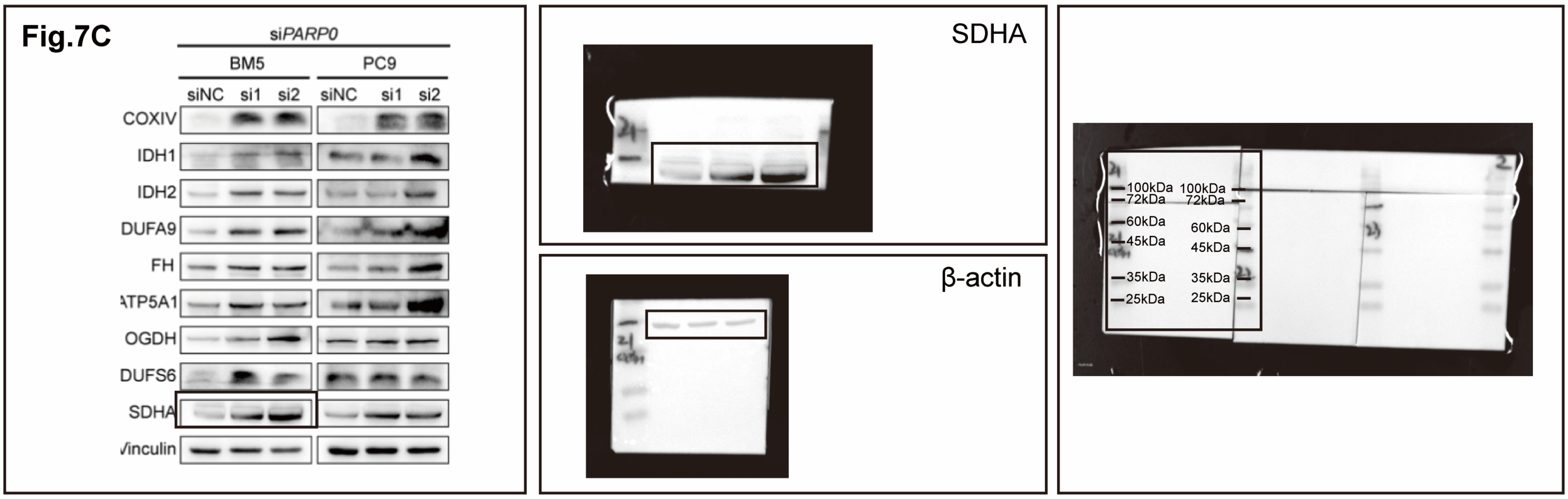

Figure	Merged	Stiched full image

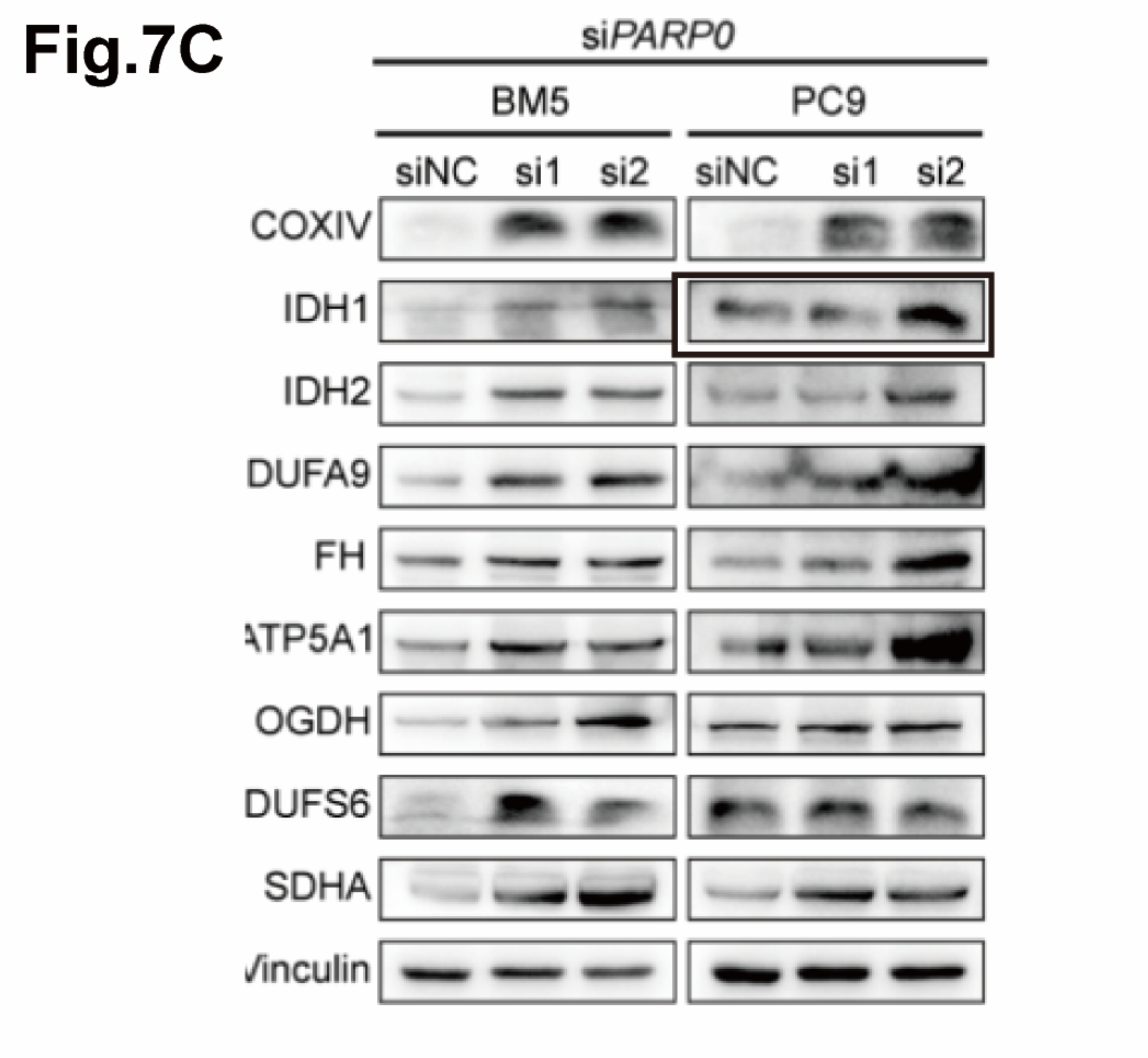

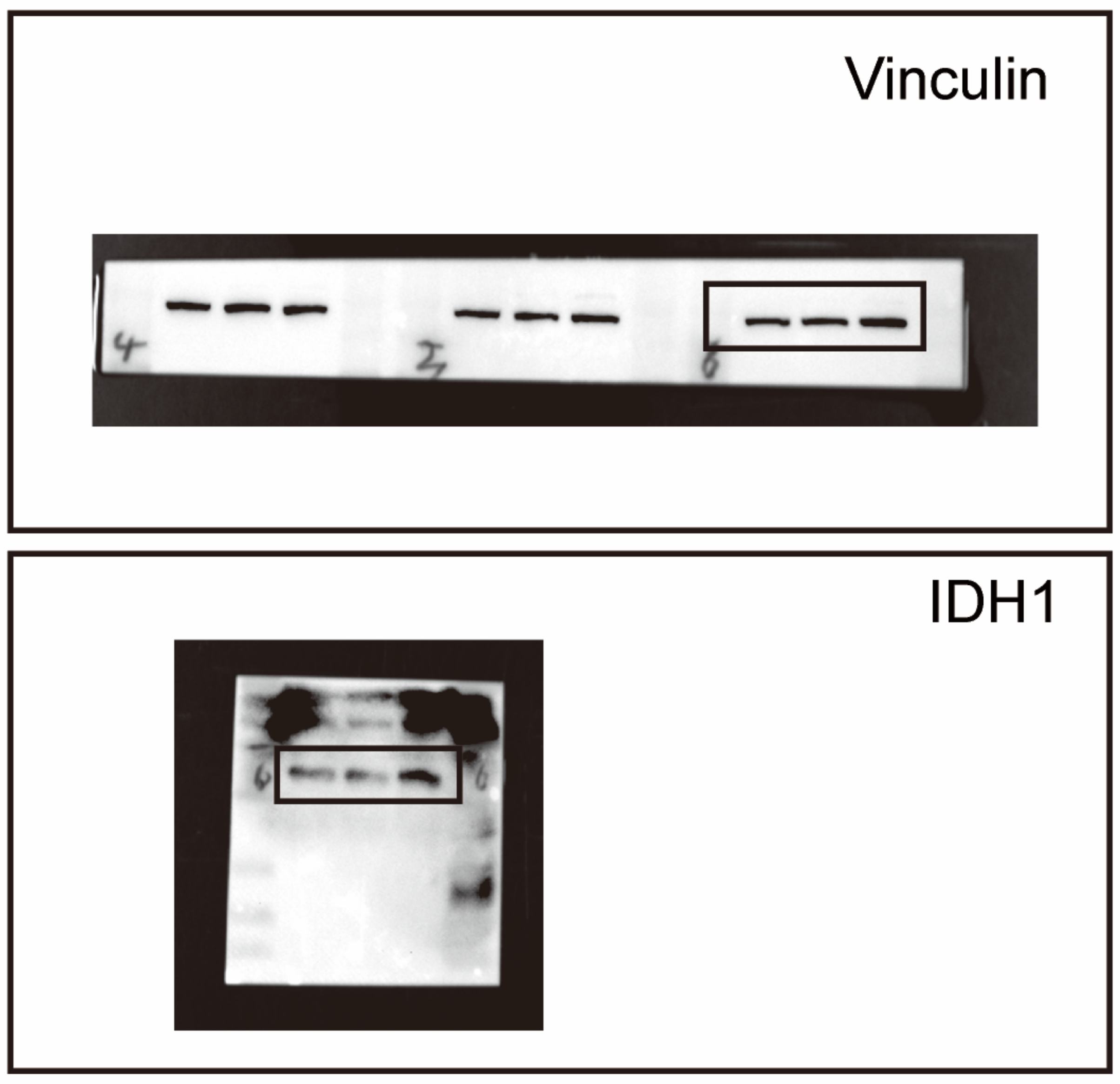

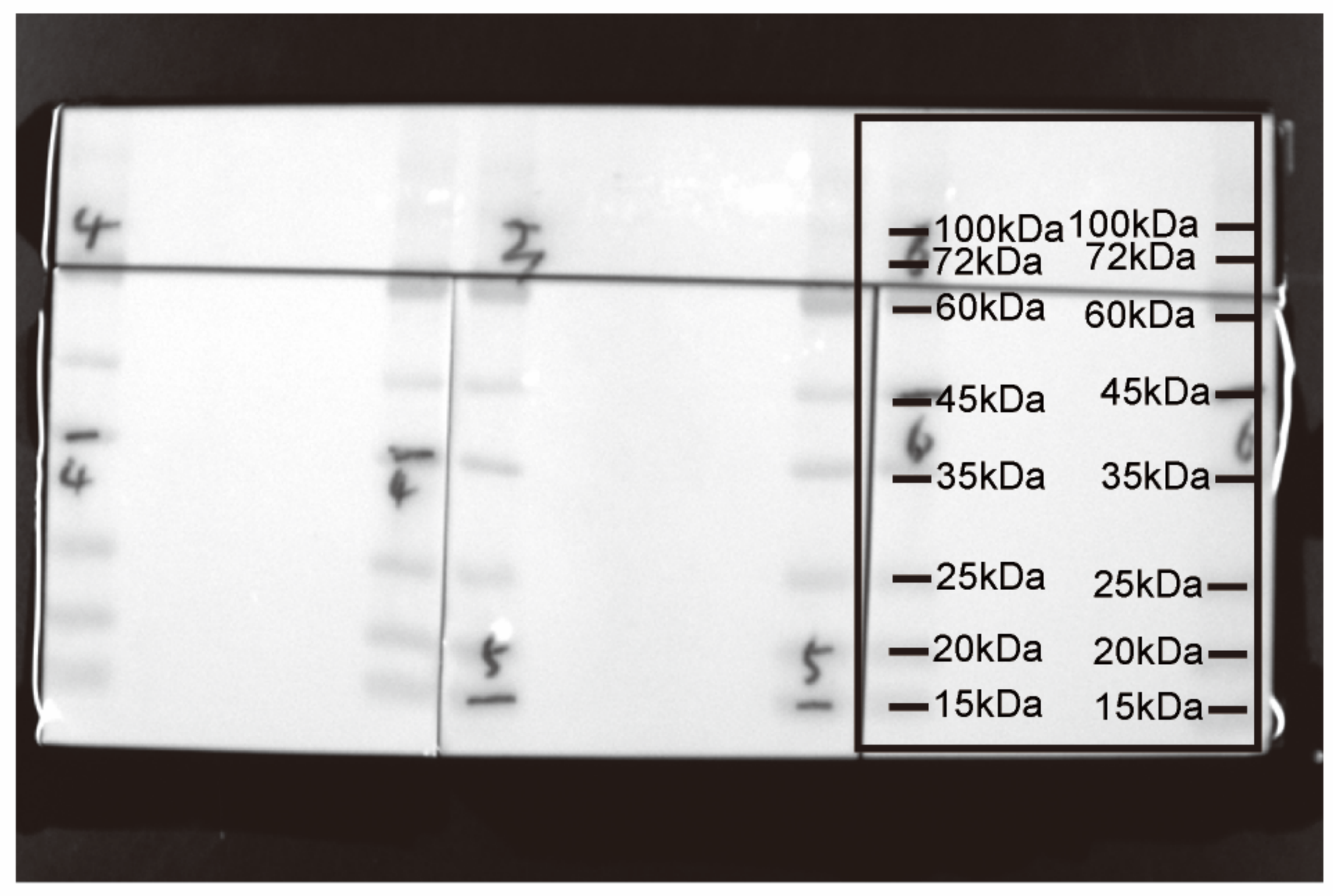

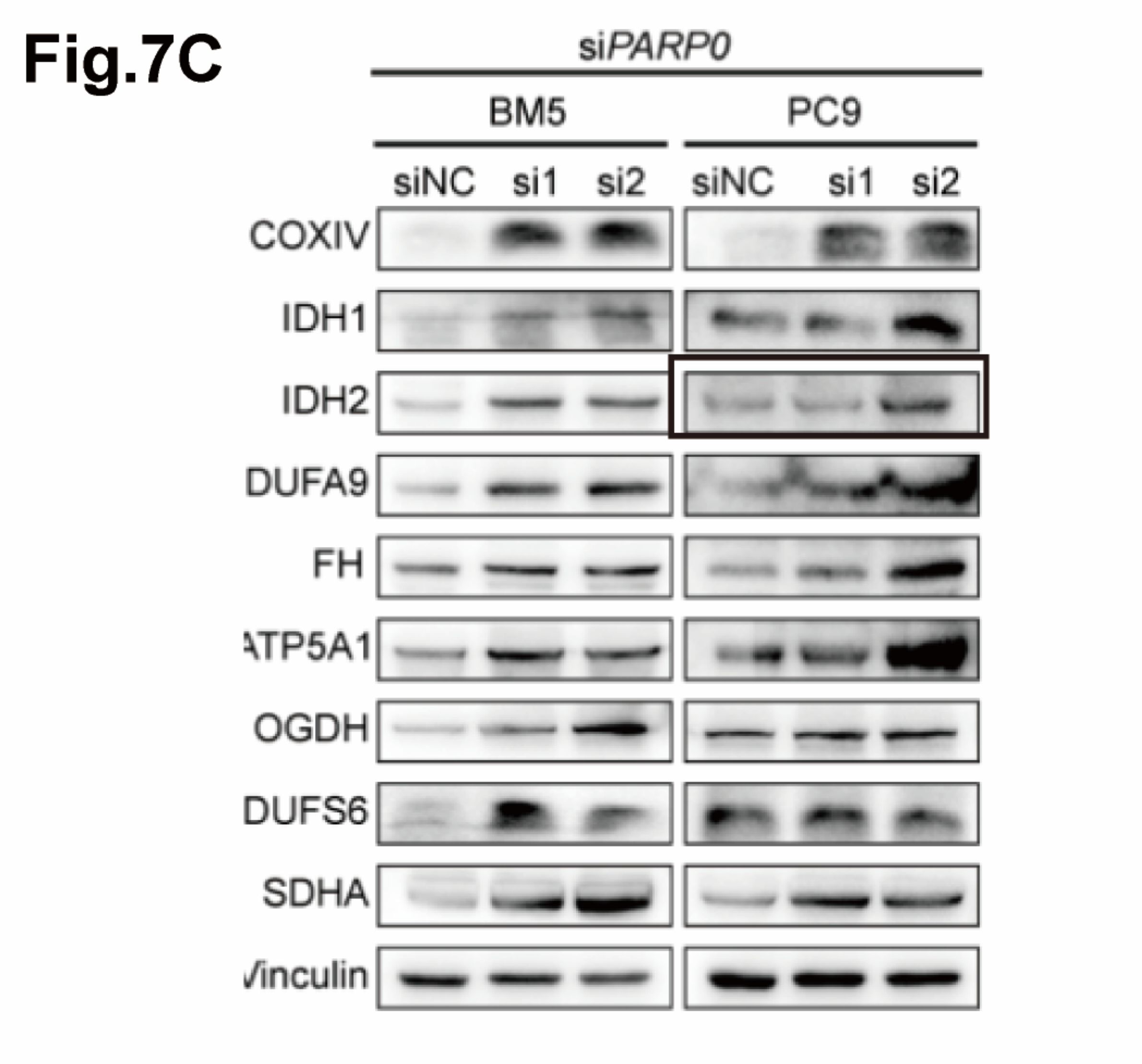

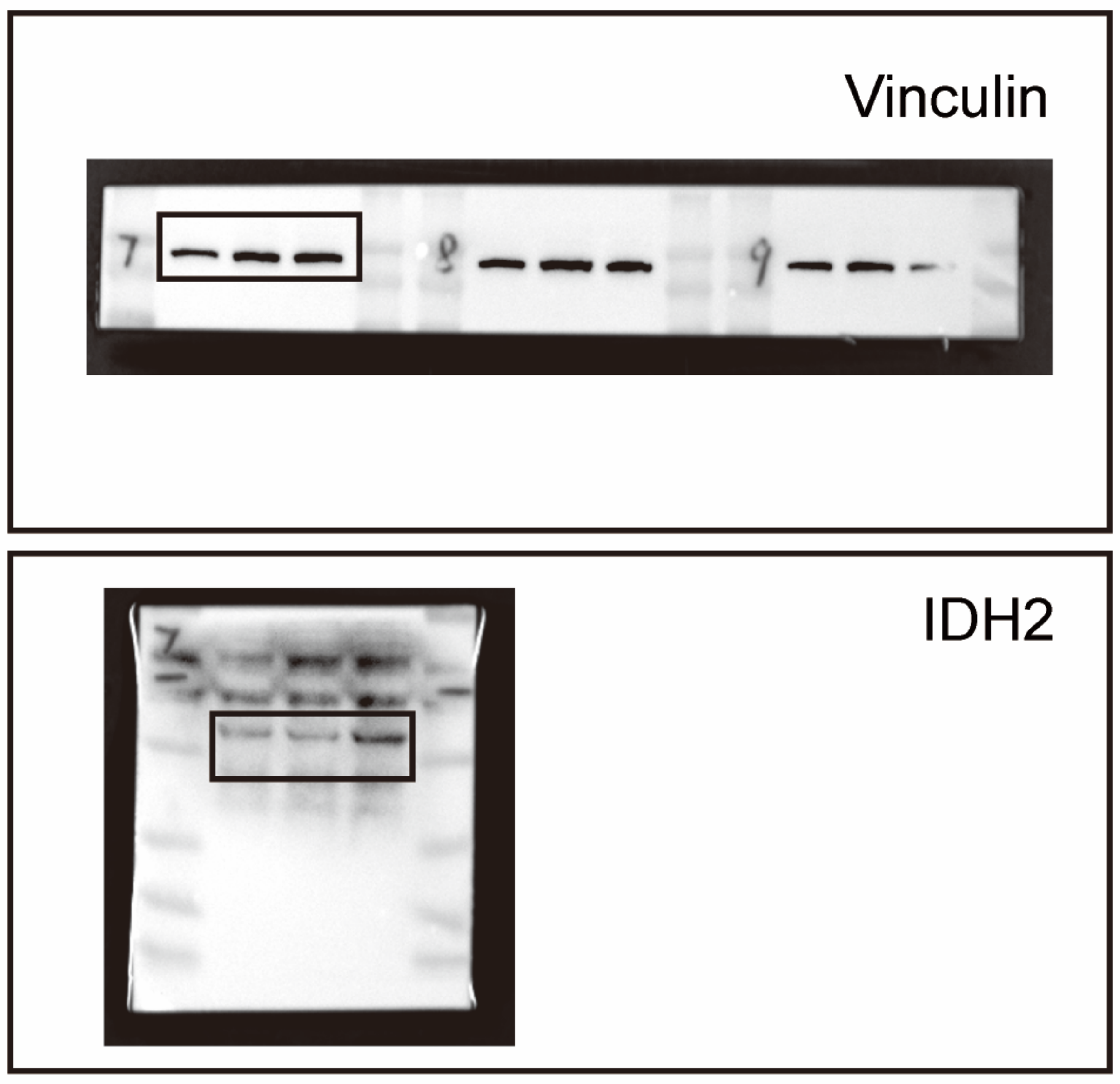

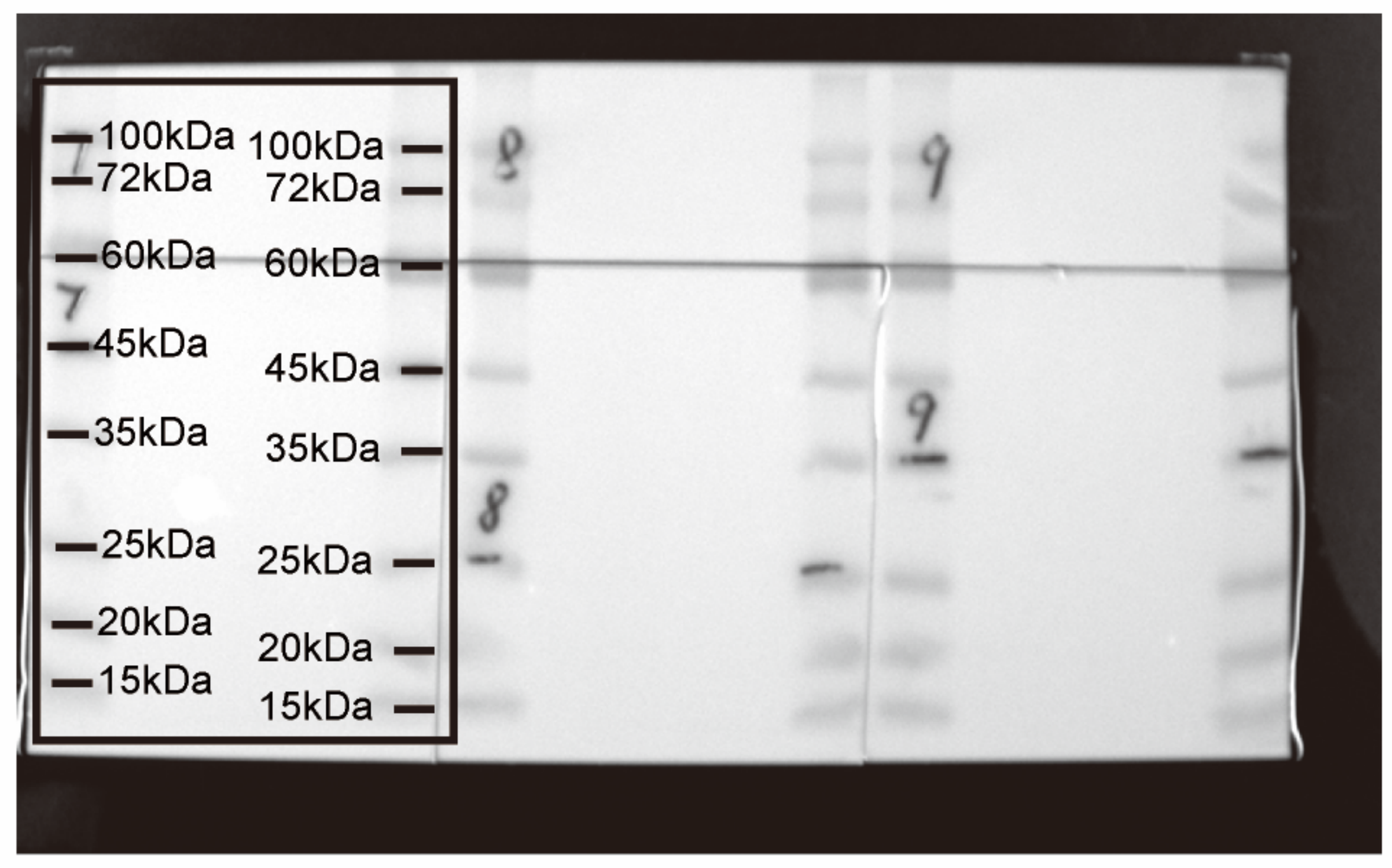

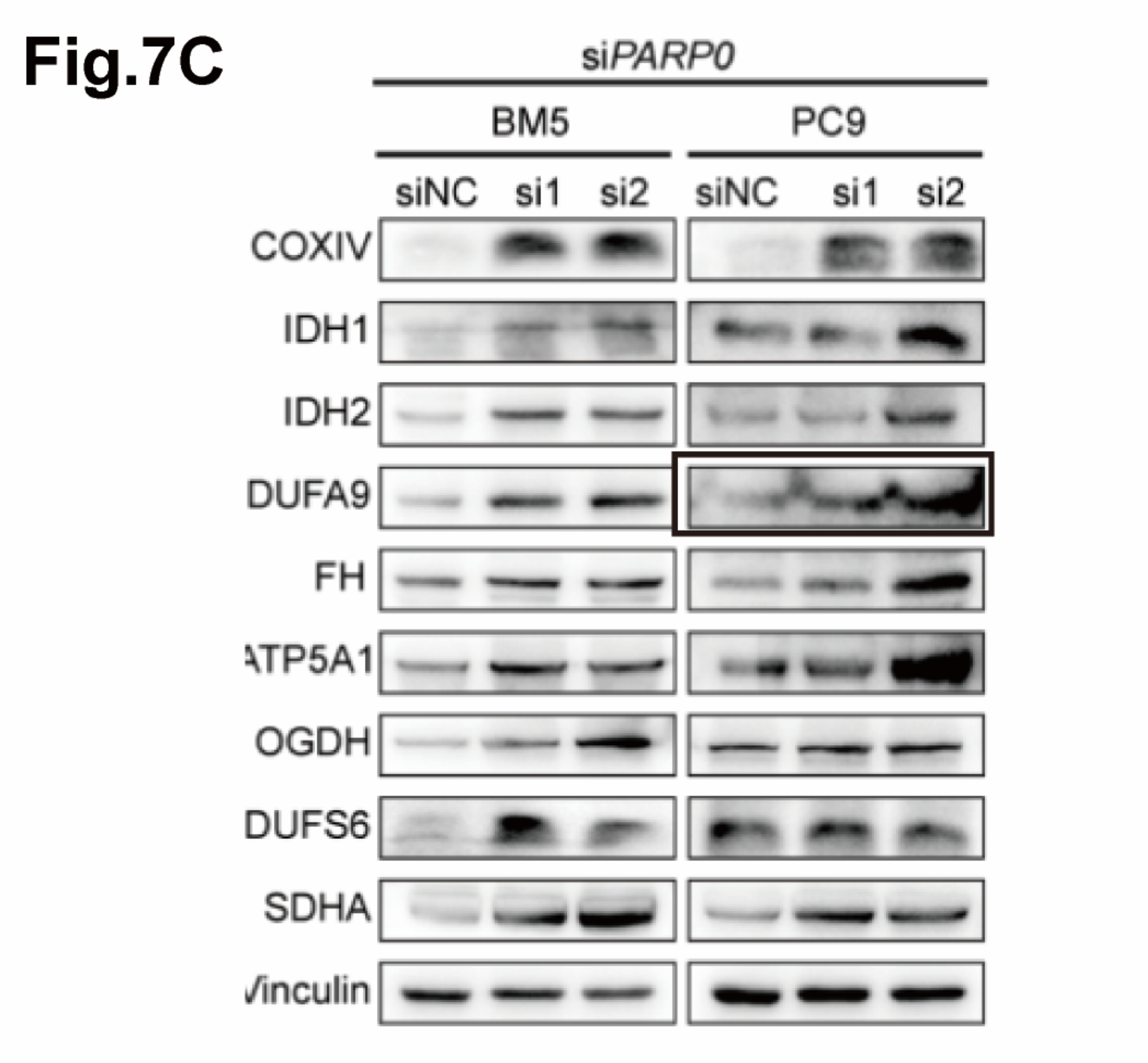

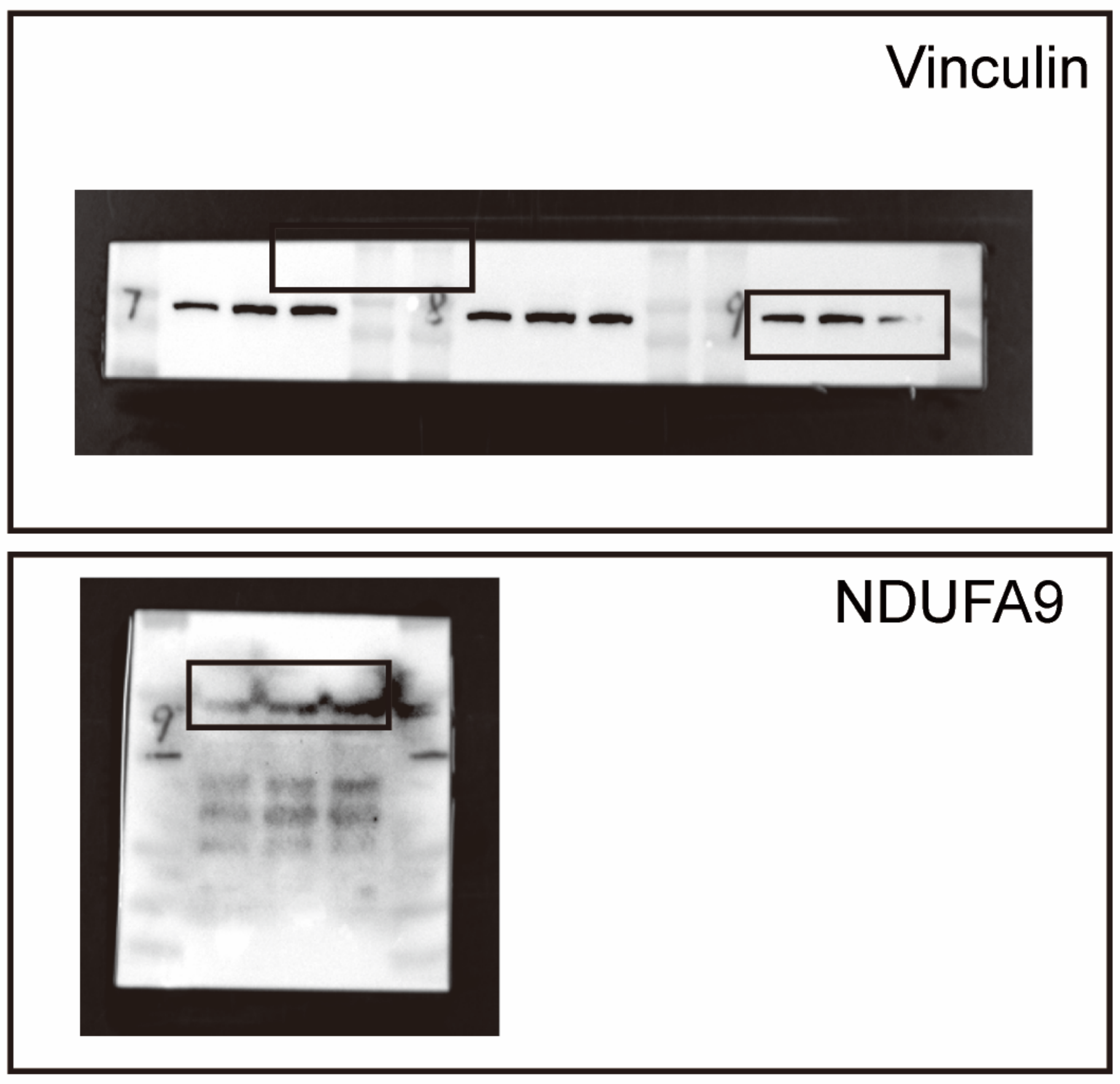

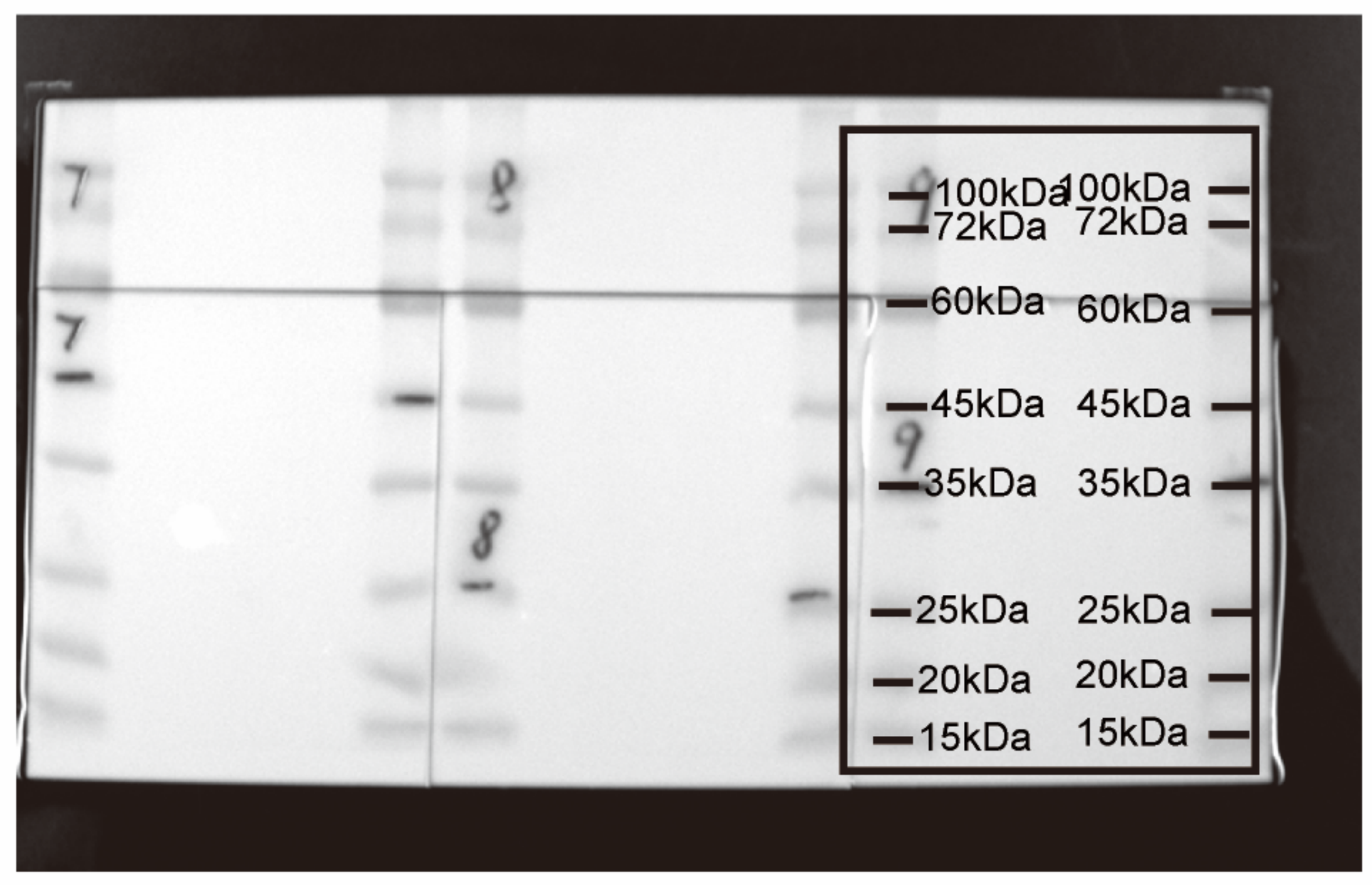

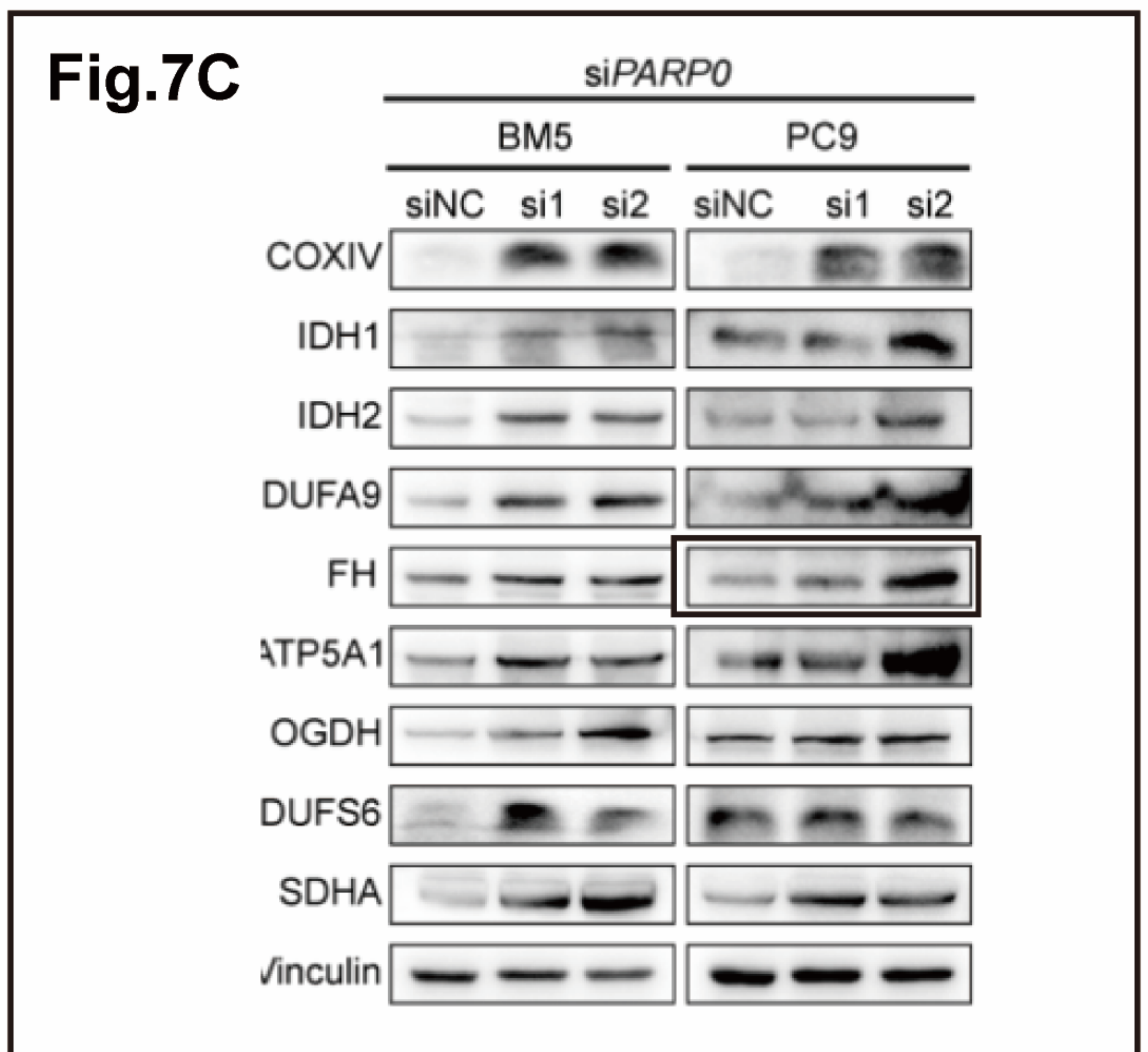

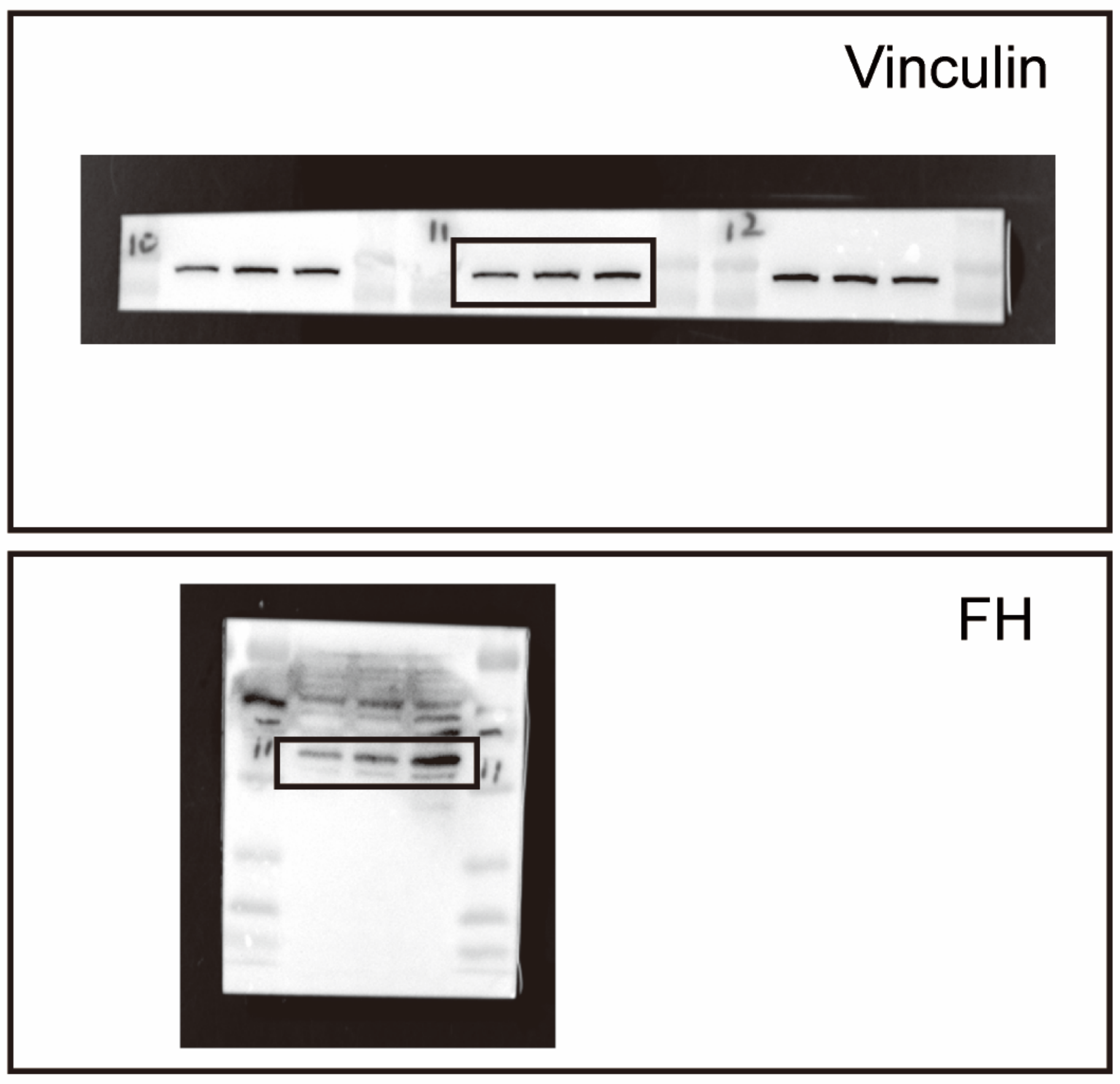

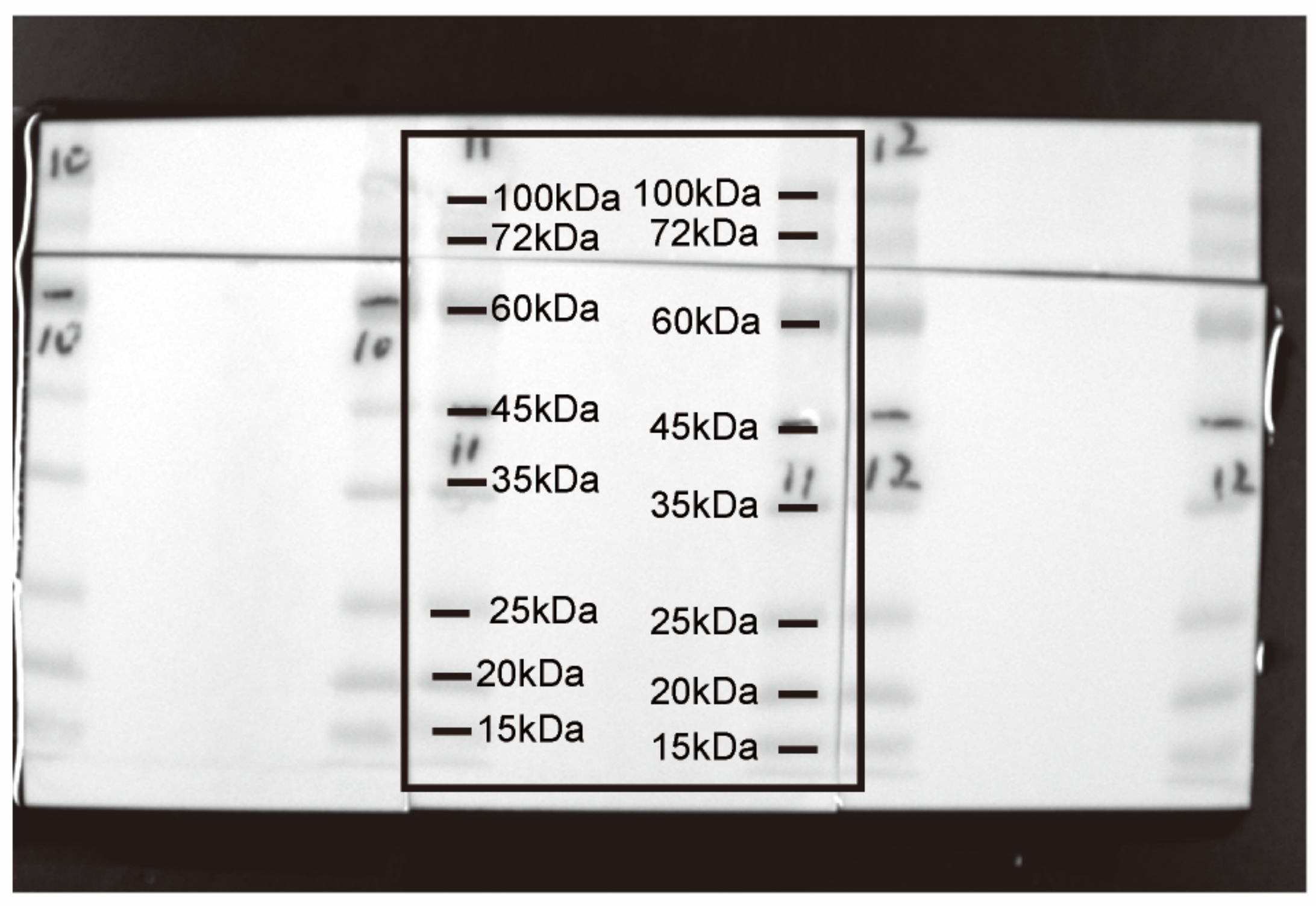

Figure	Merged	Stiched full image

Fig.7C

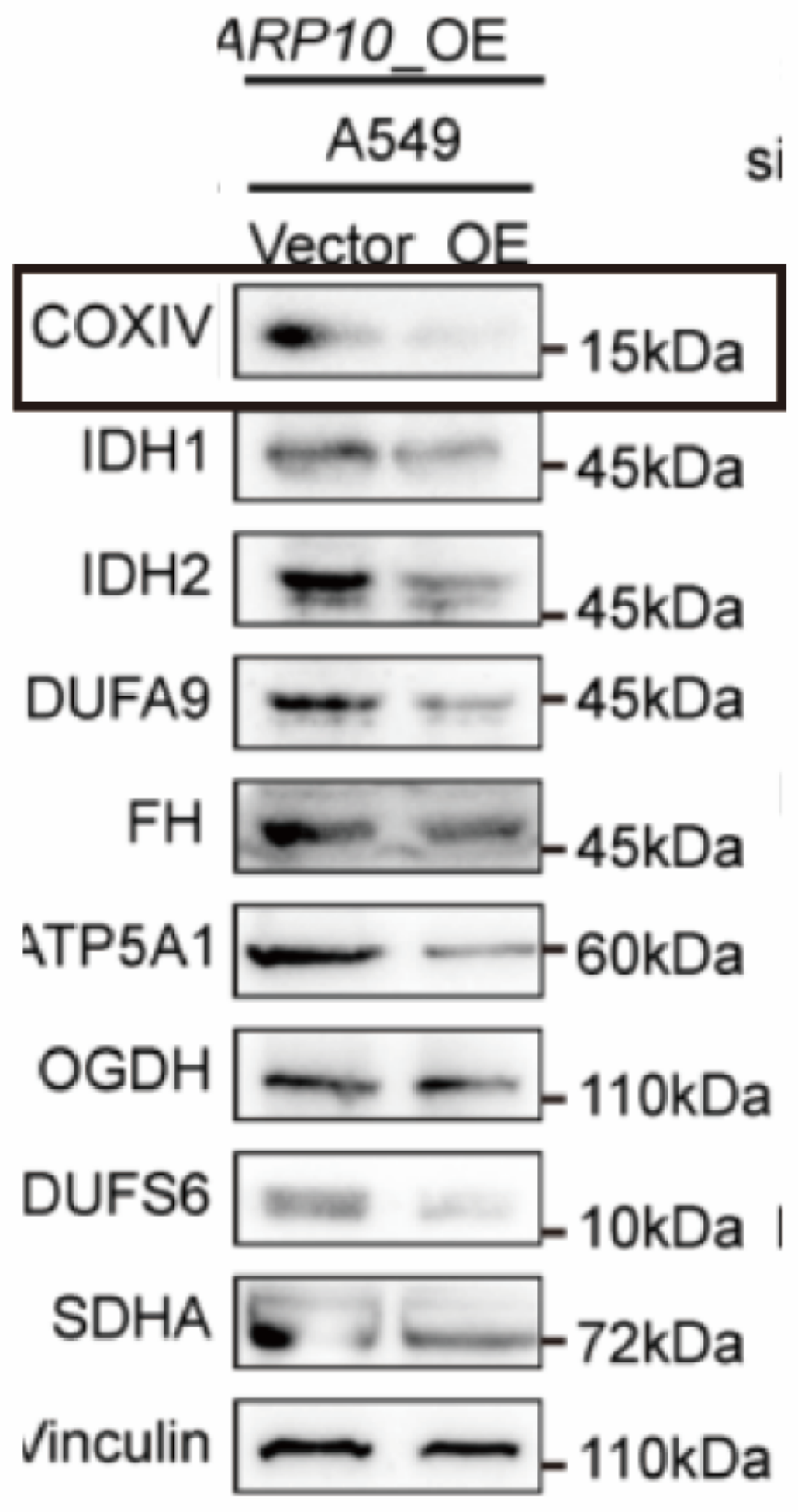

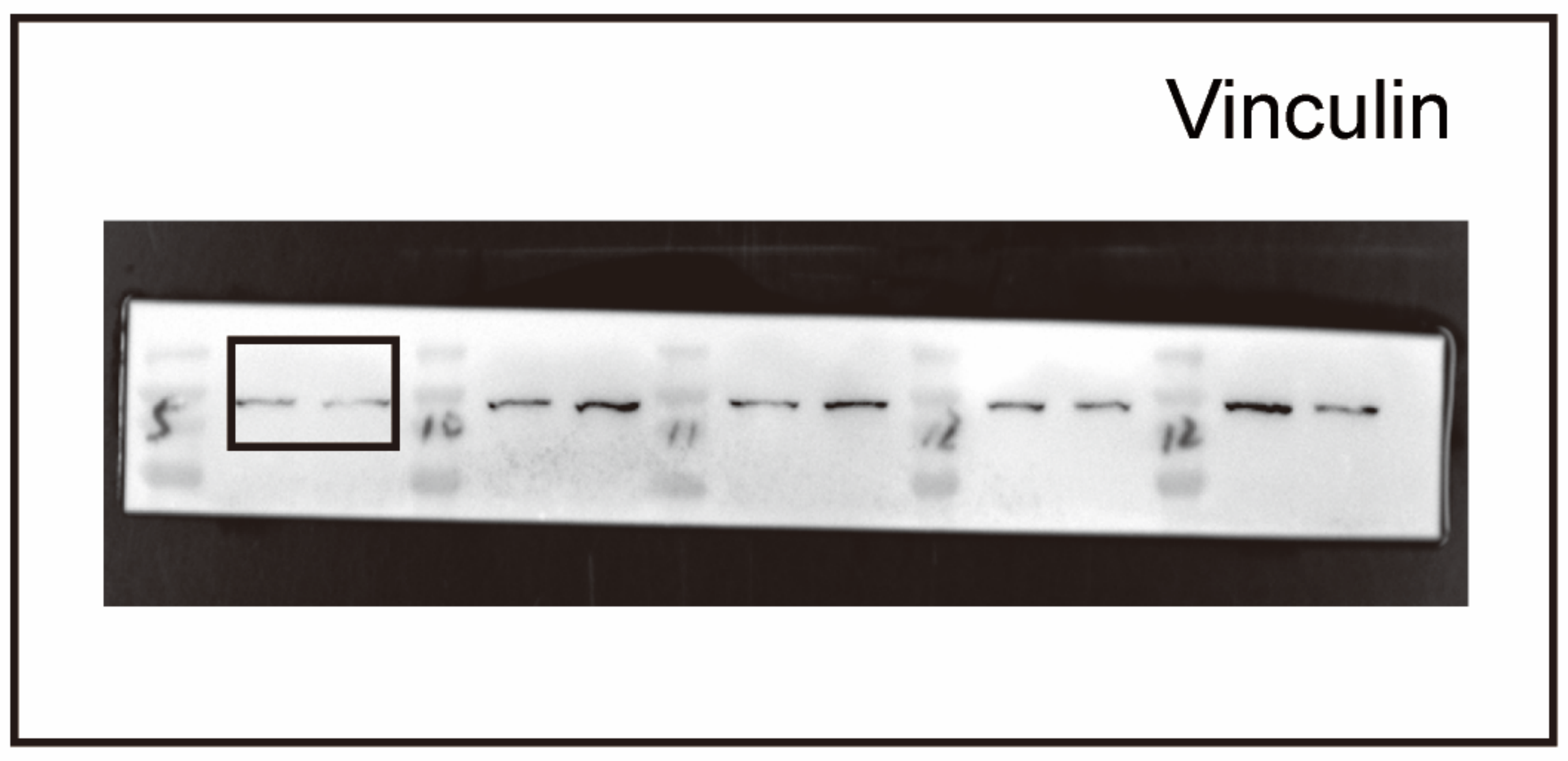

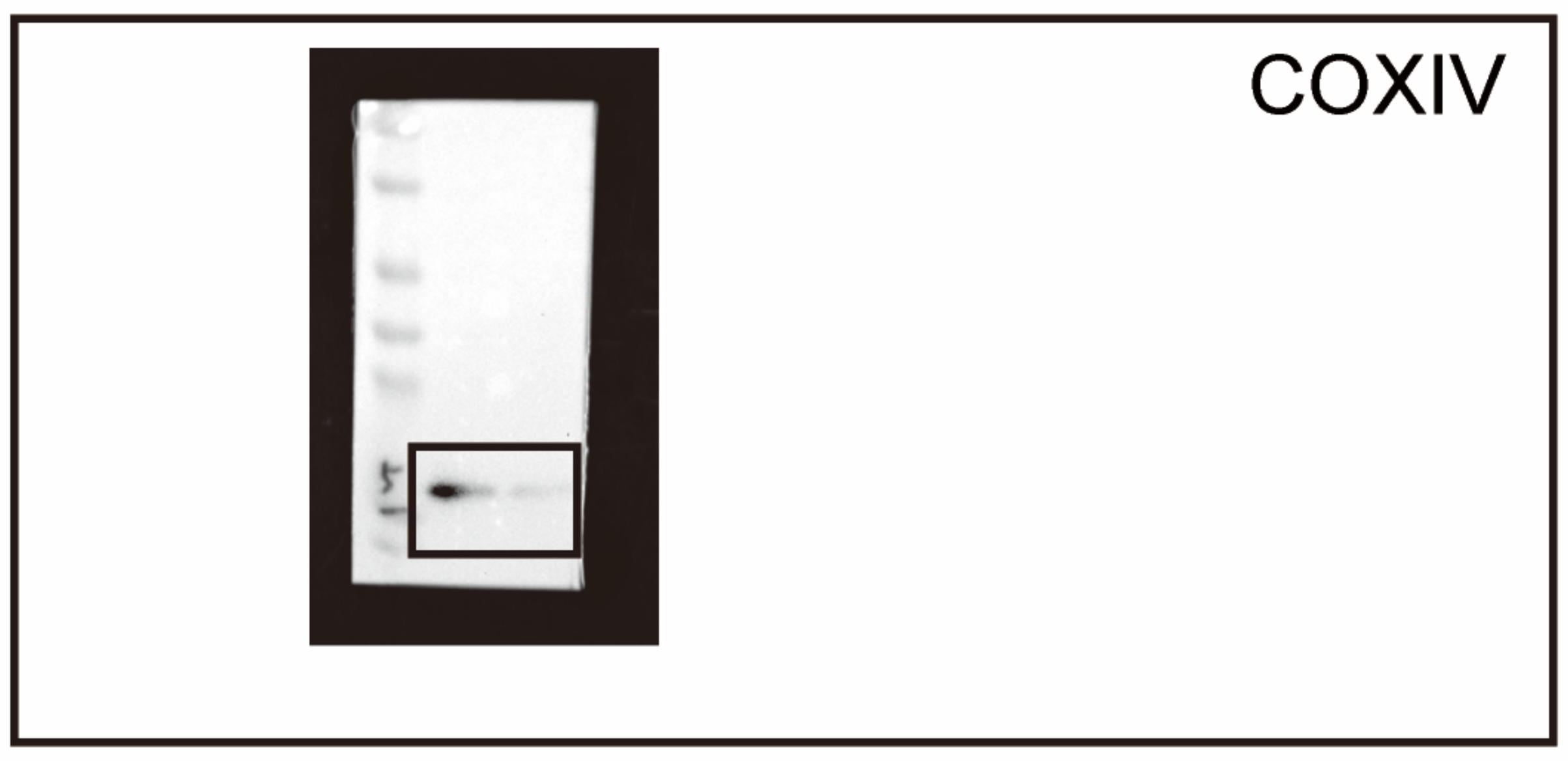

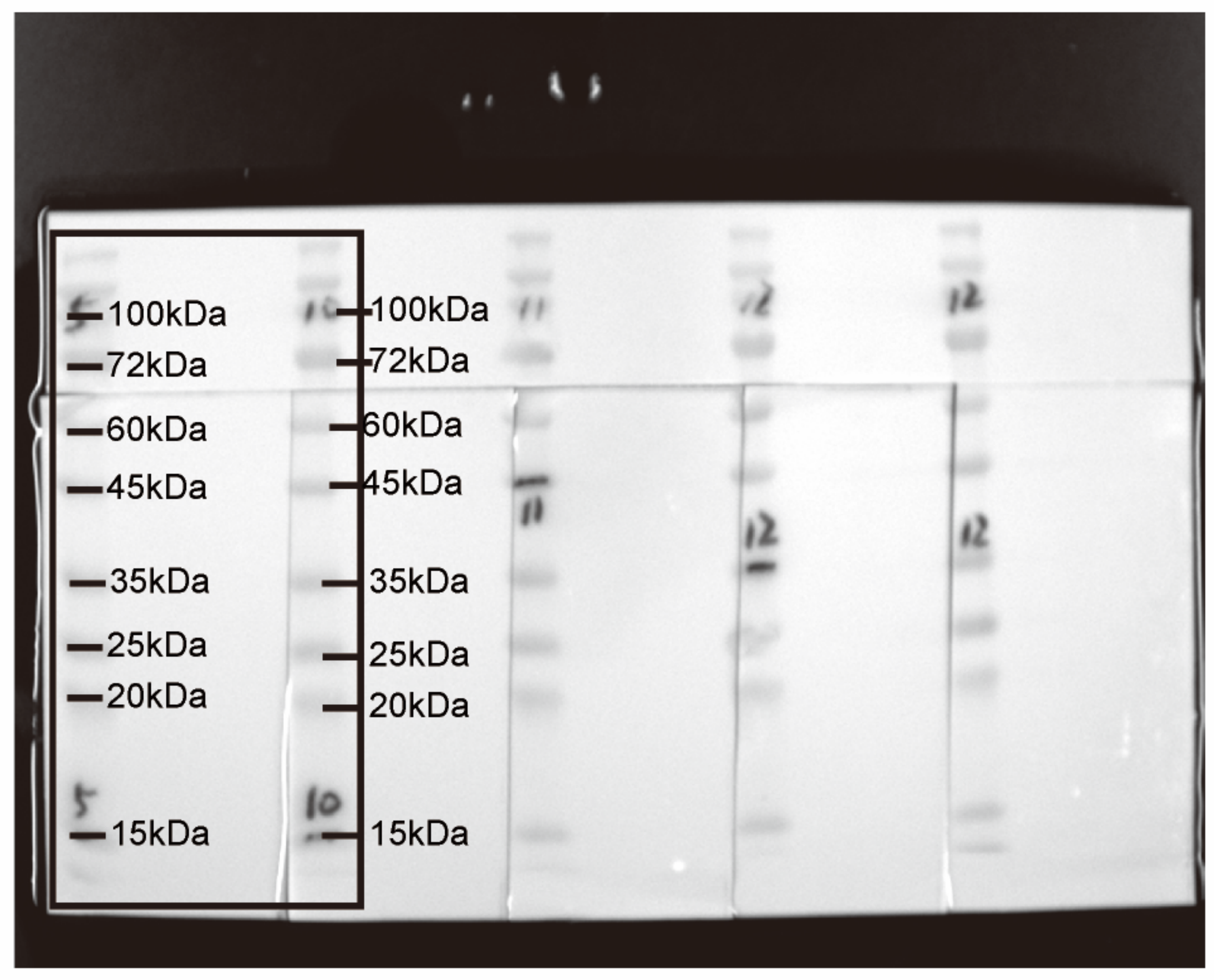

Fig.7C

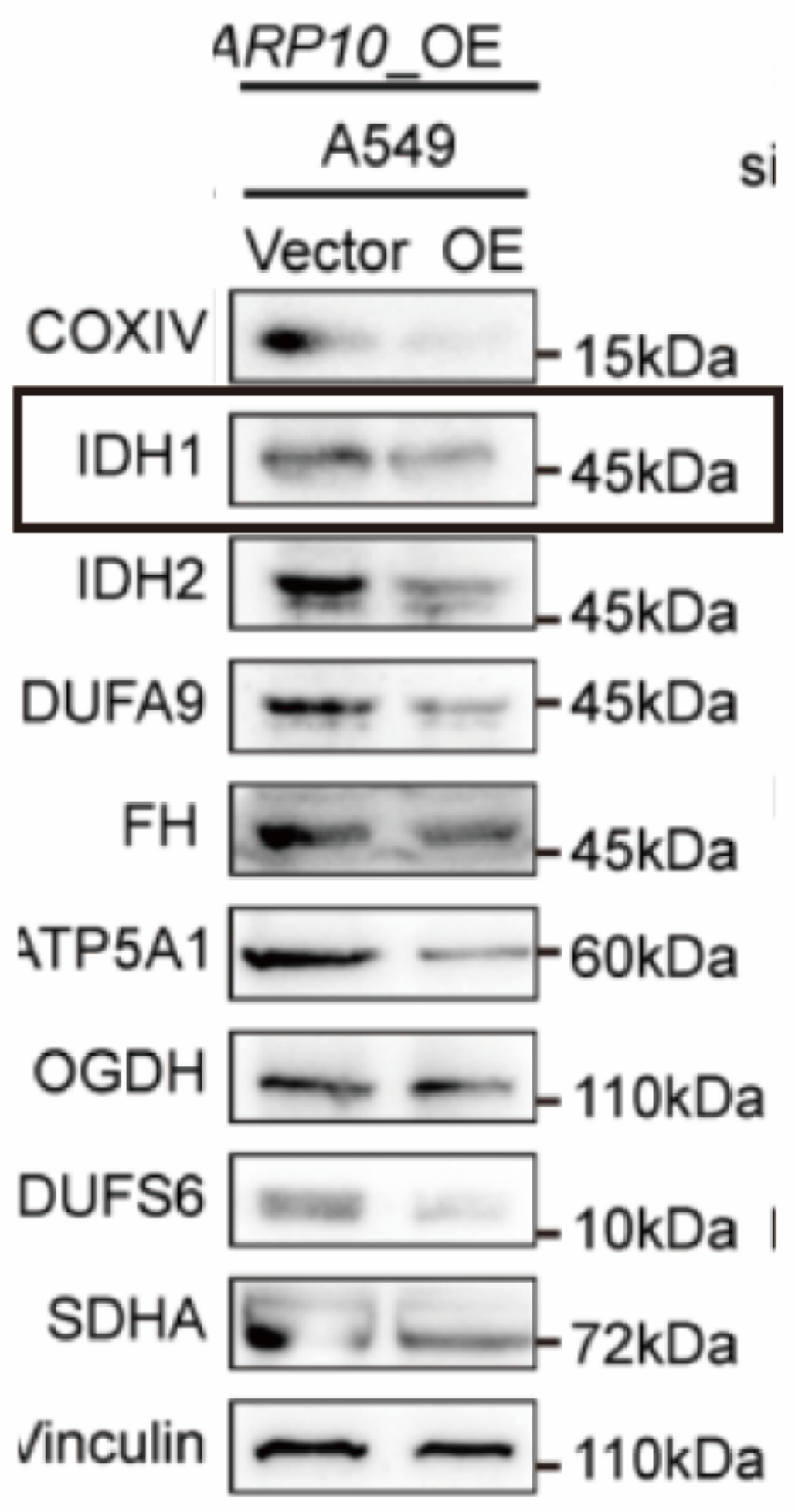

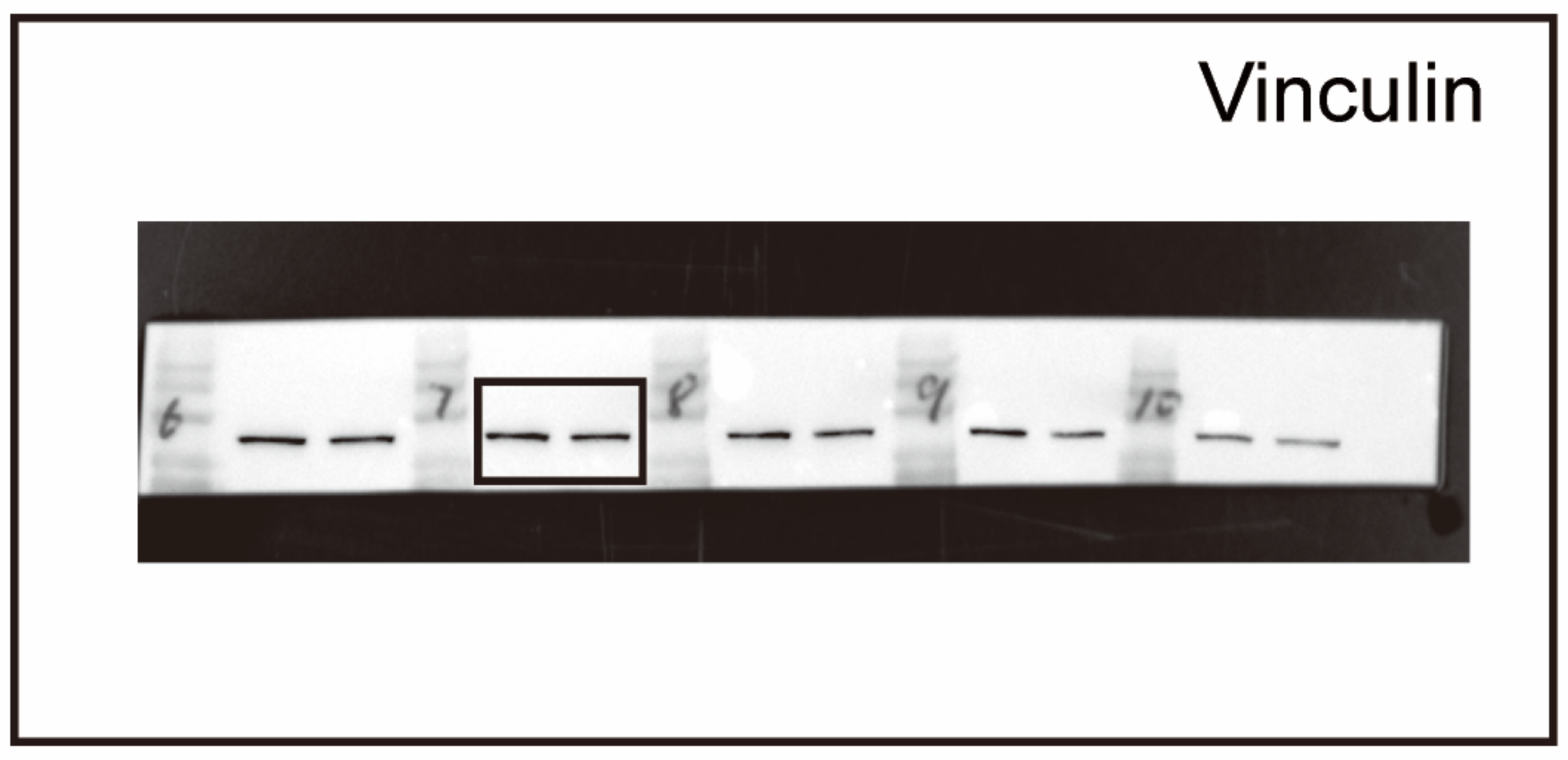

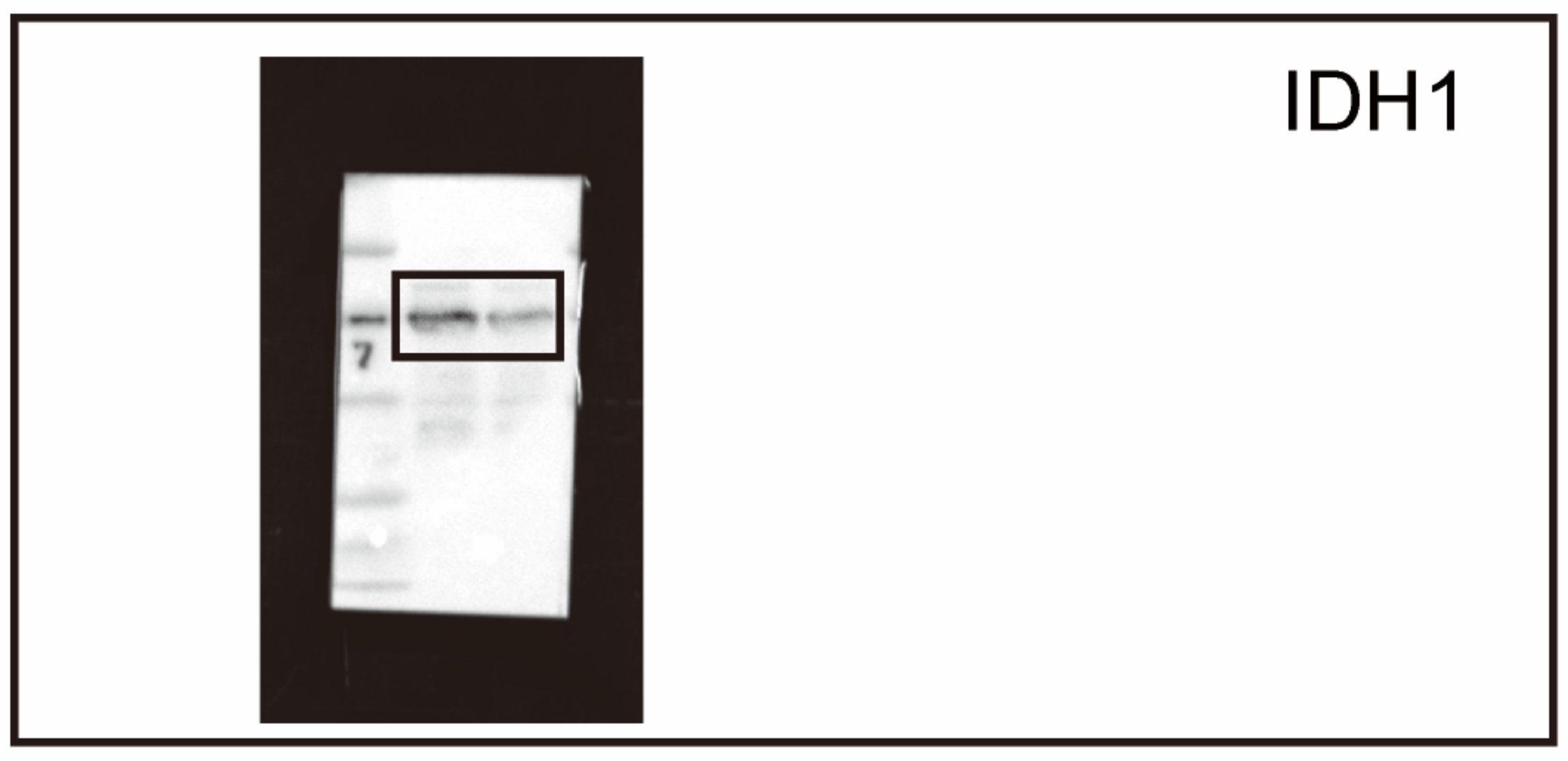

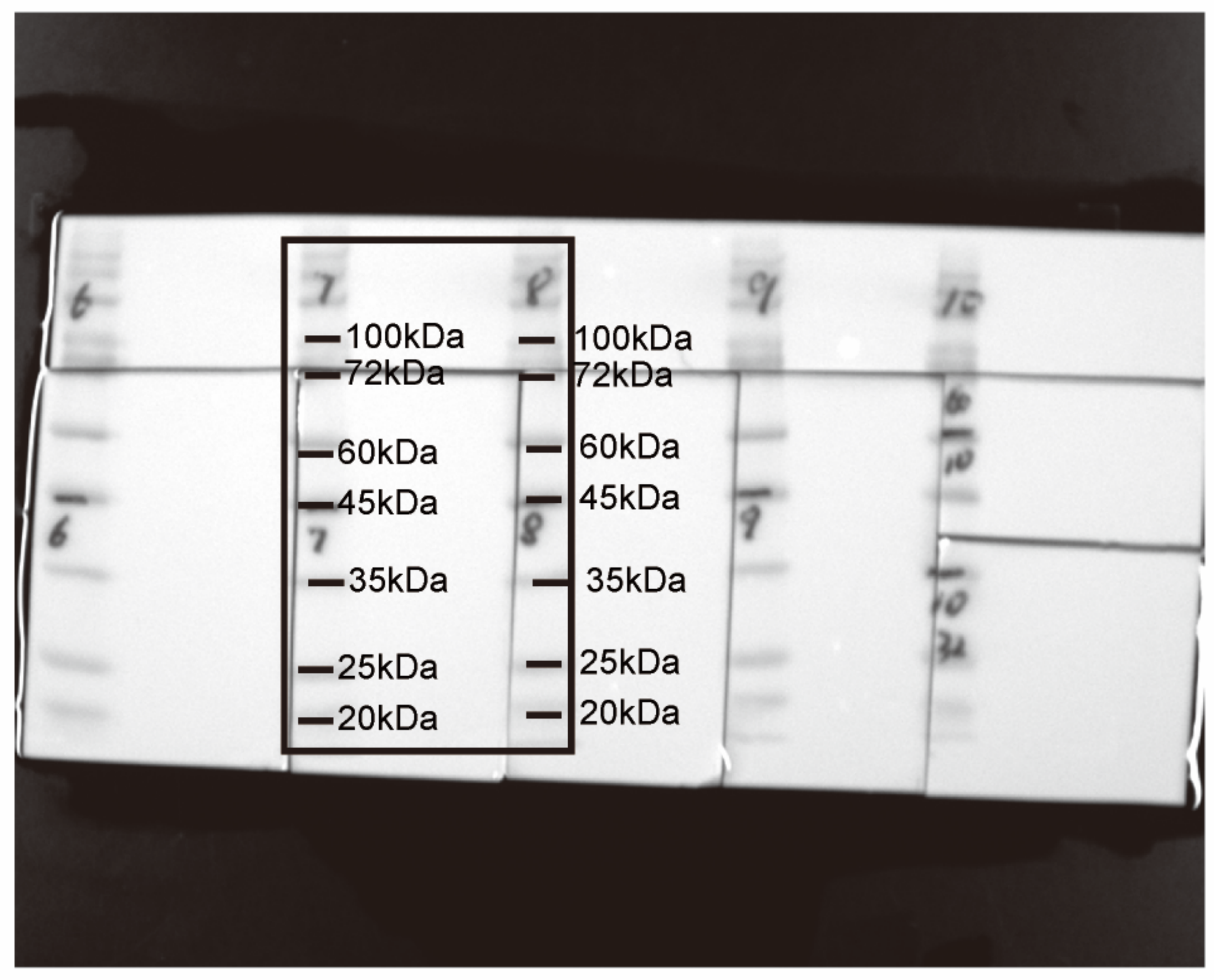

Fig.7C

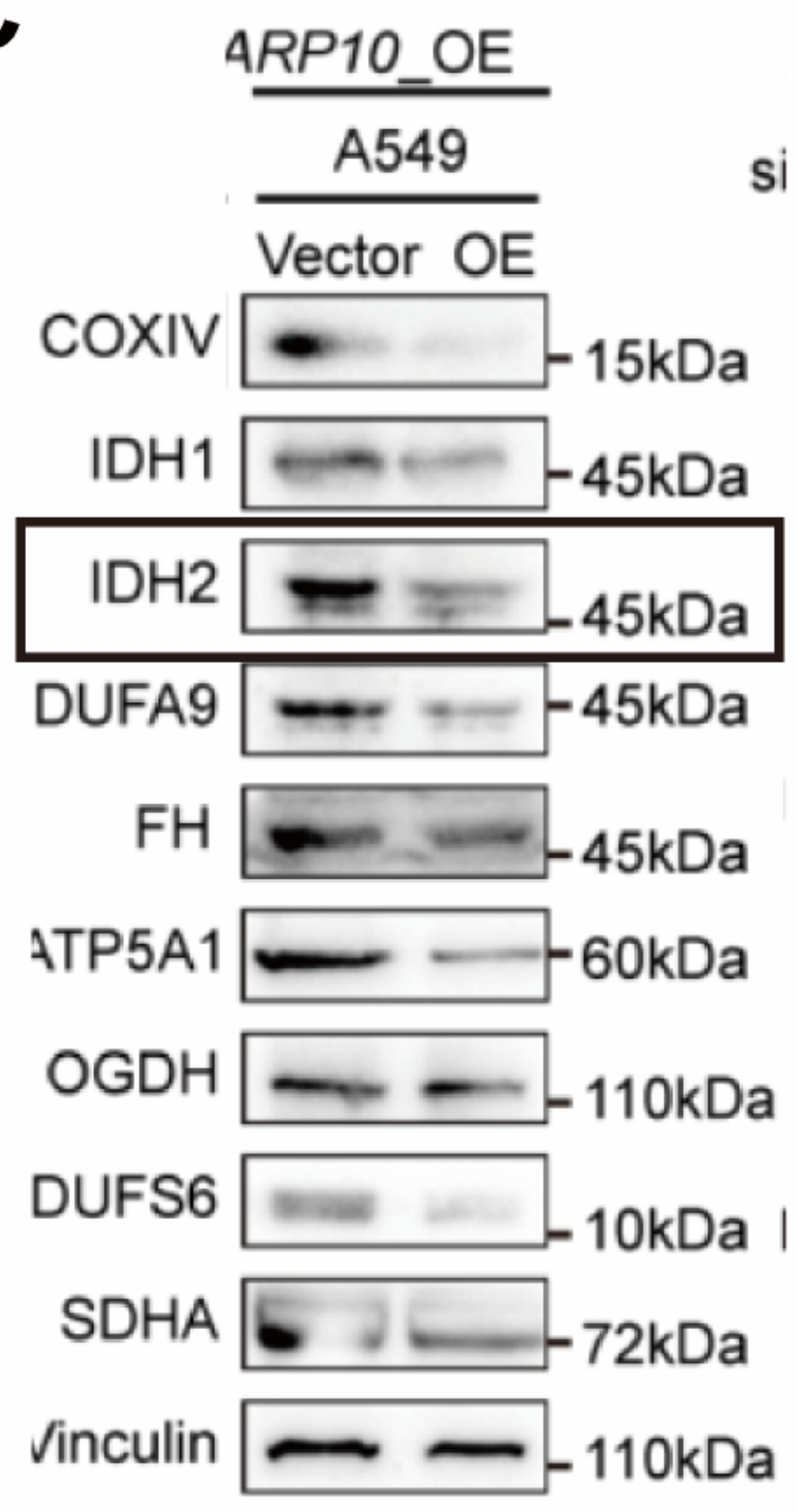

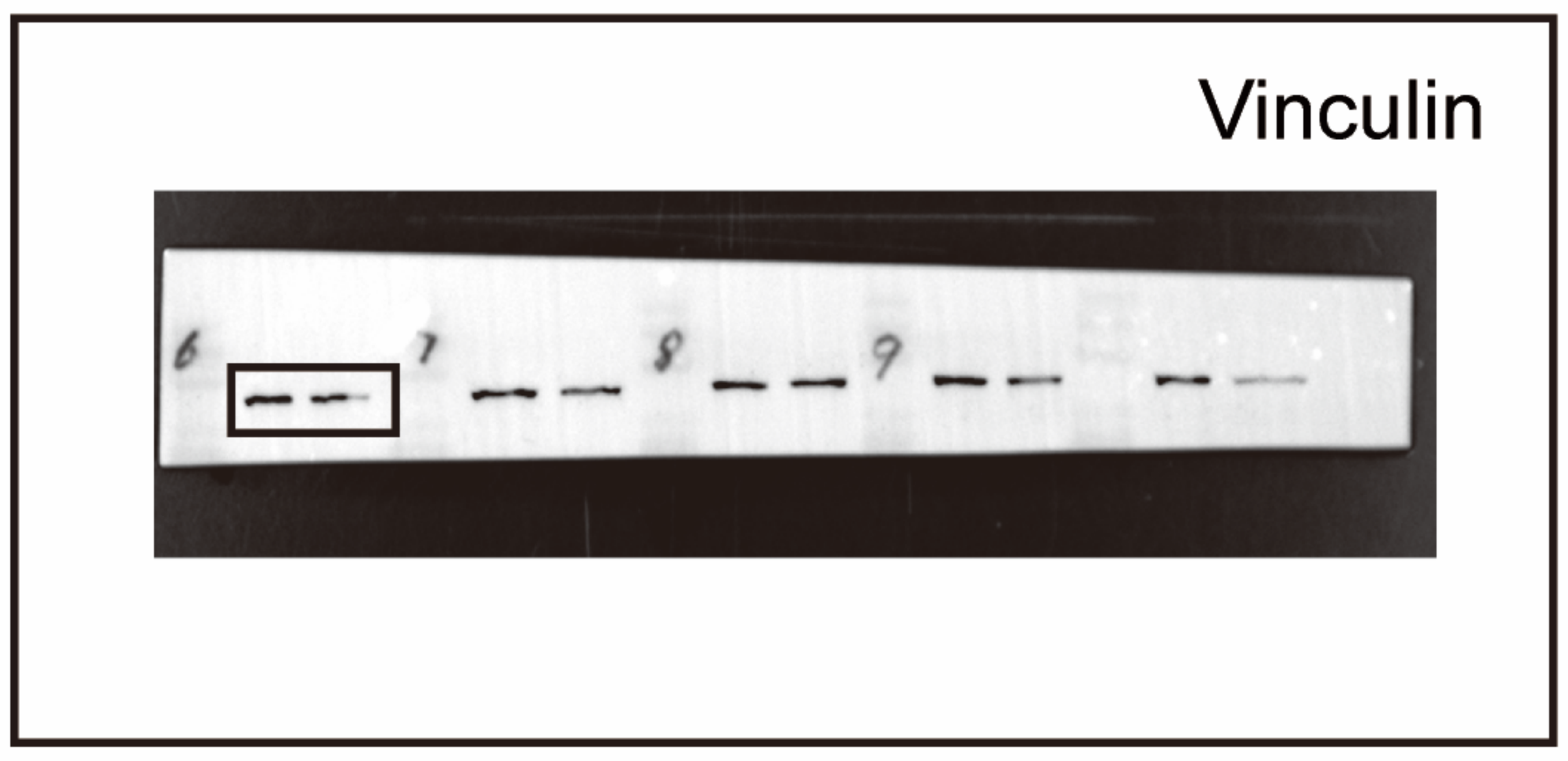

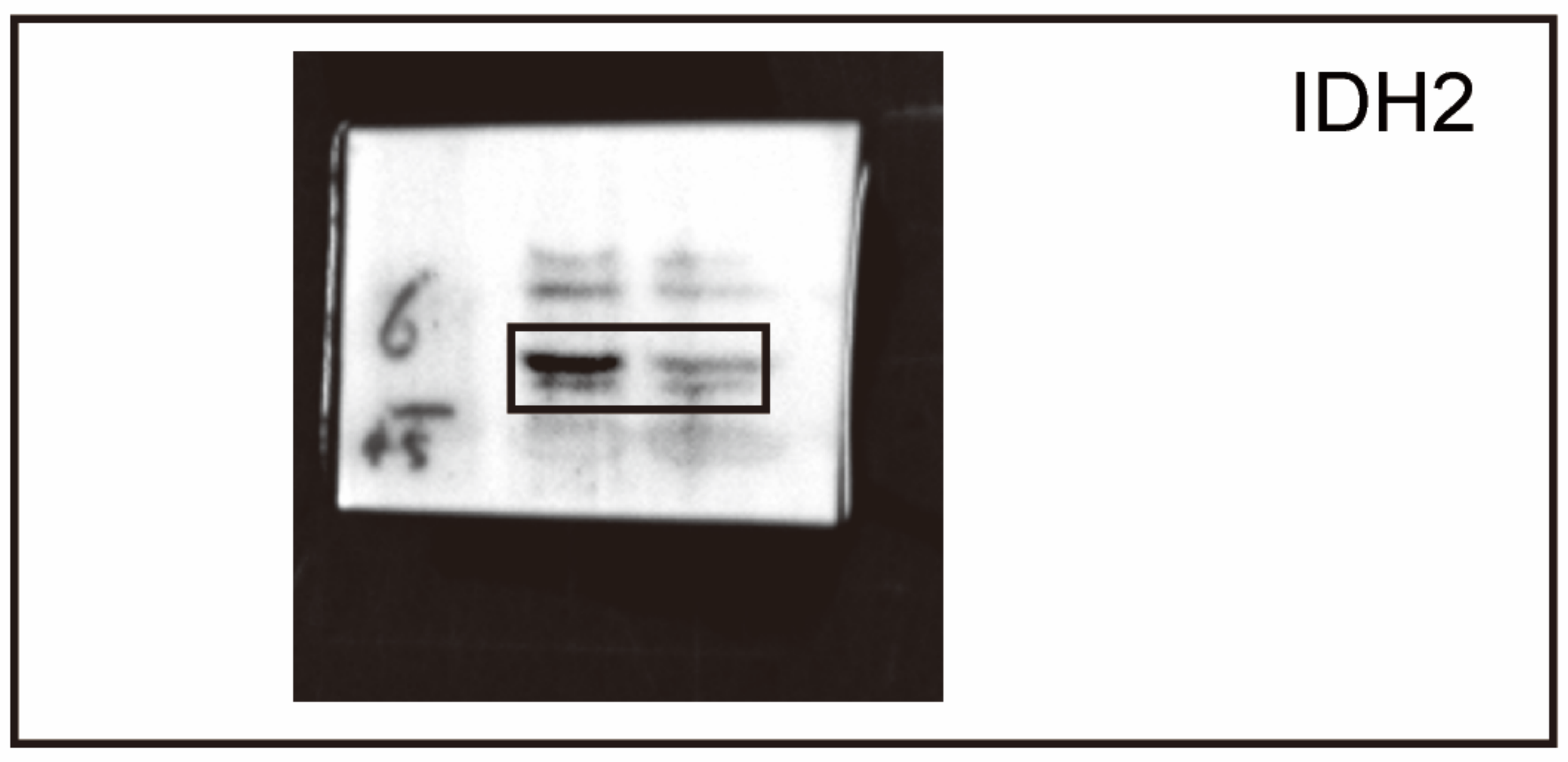

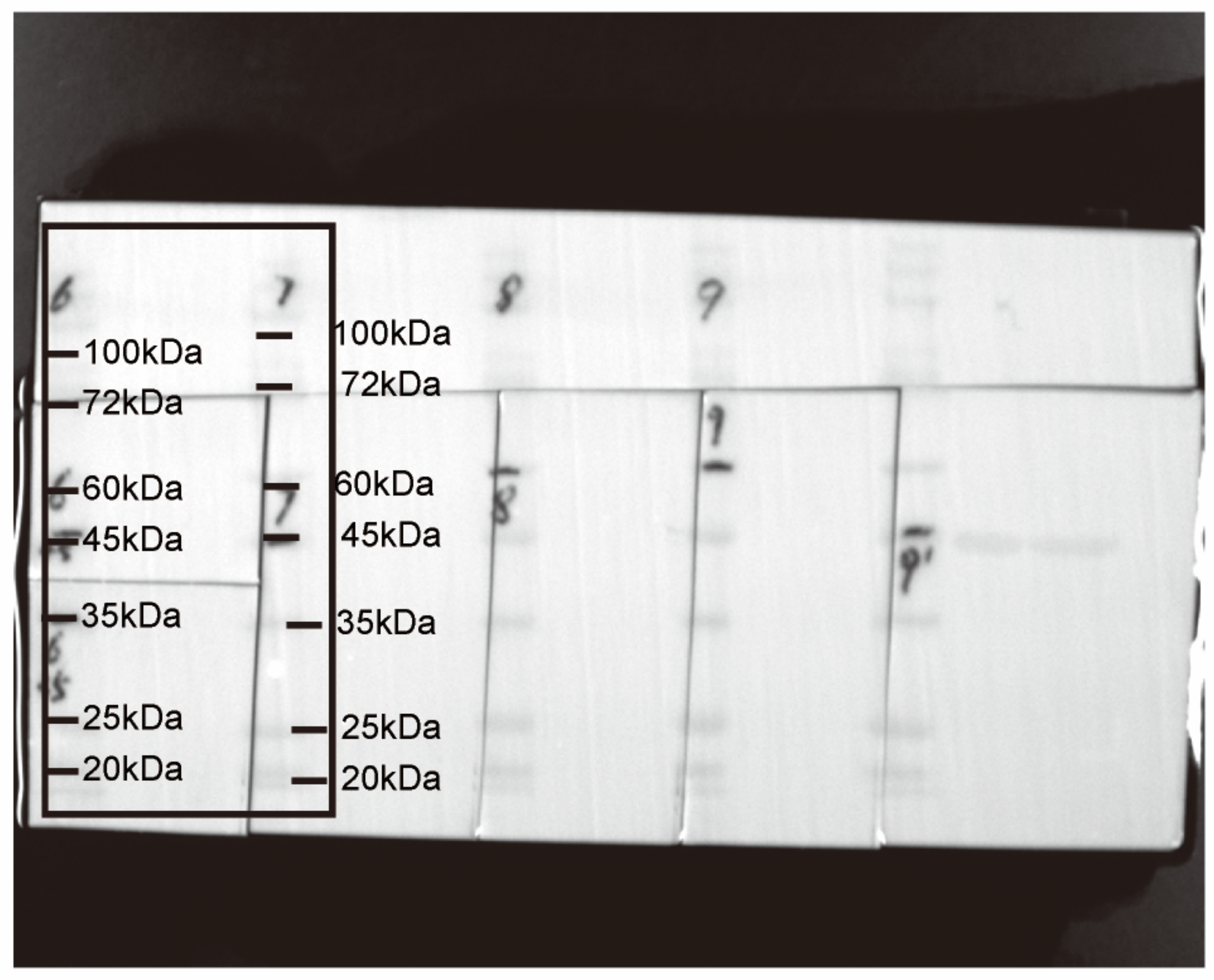

Fig.7C

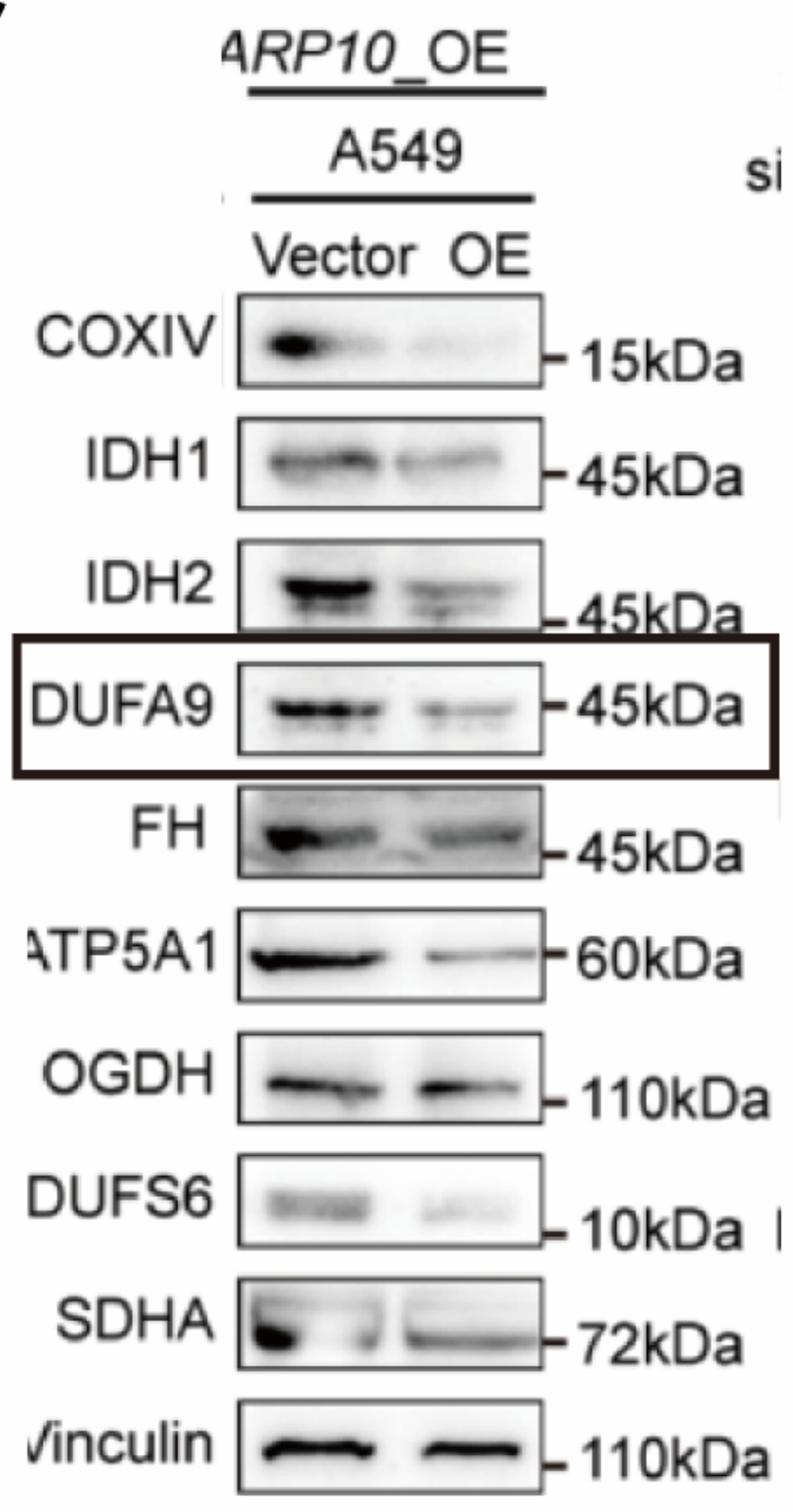

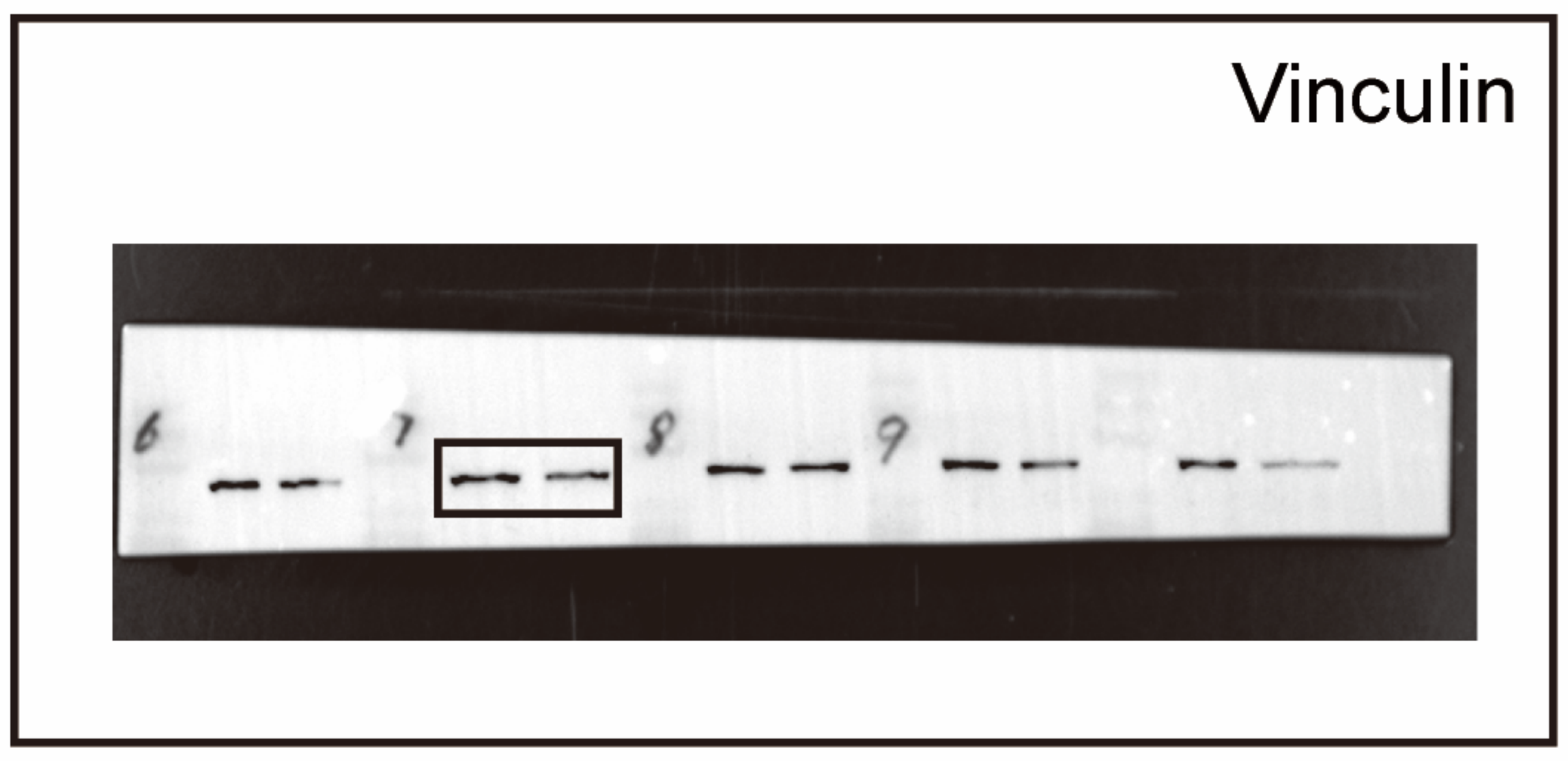

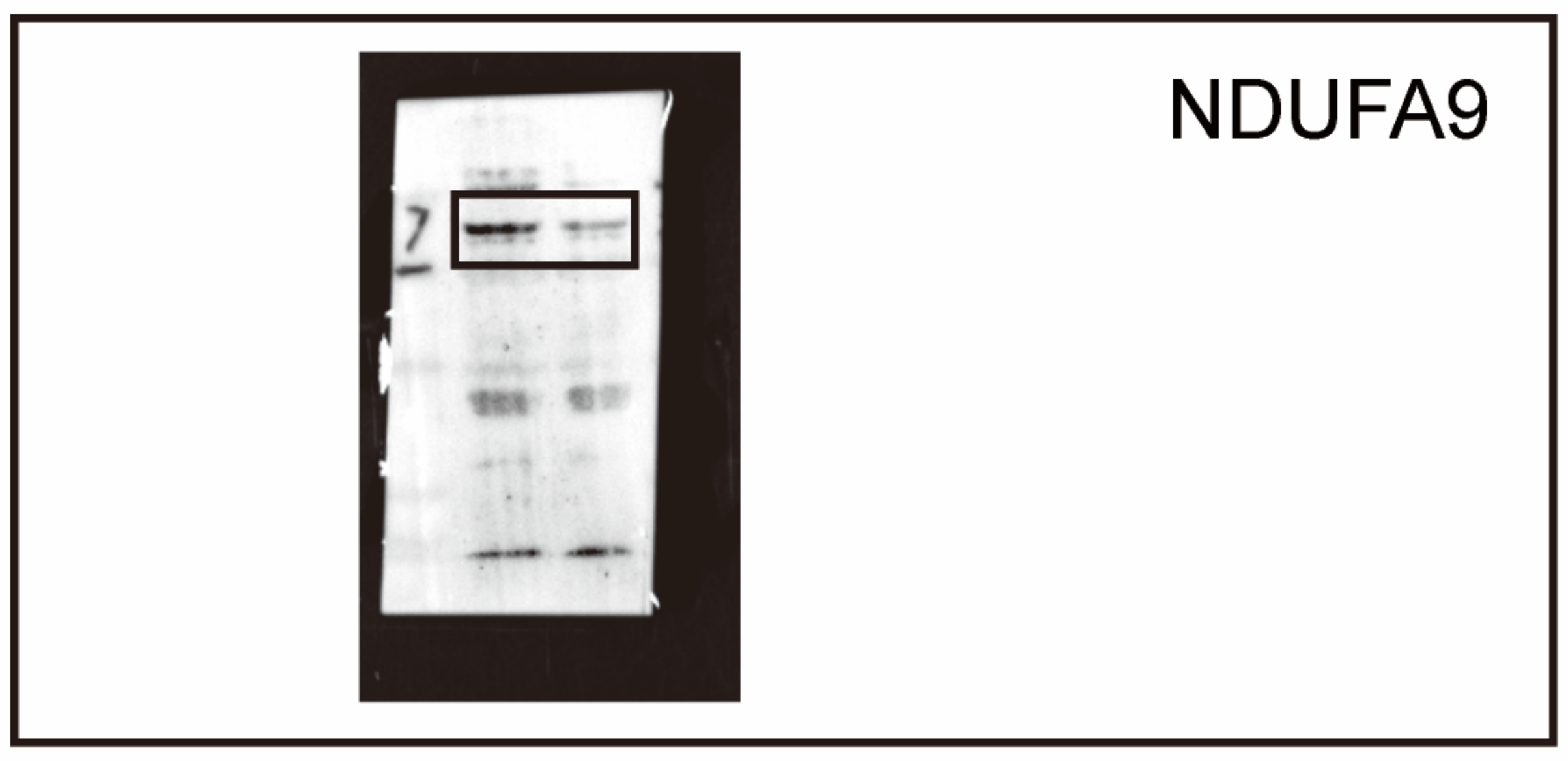

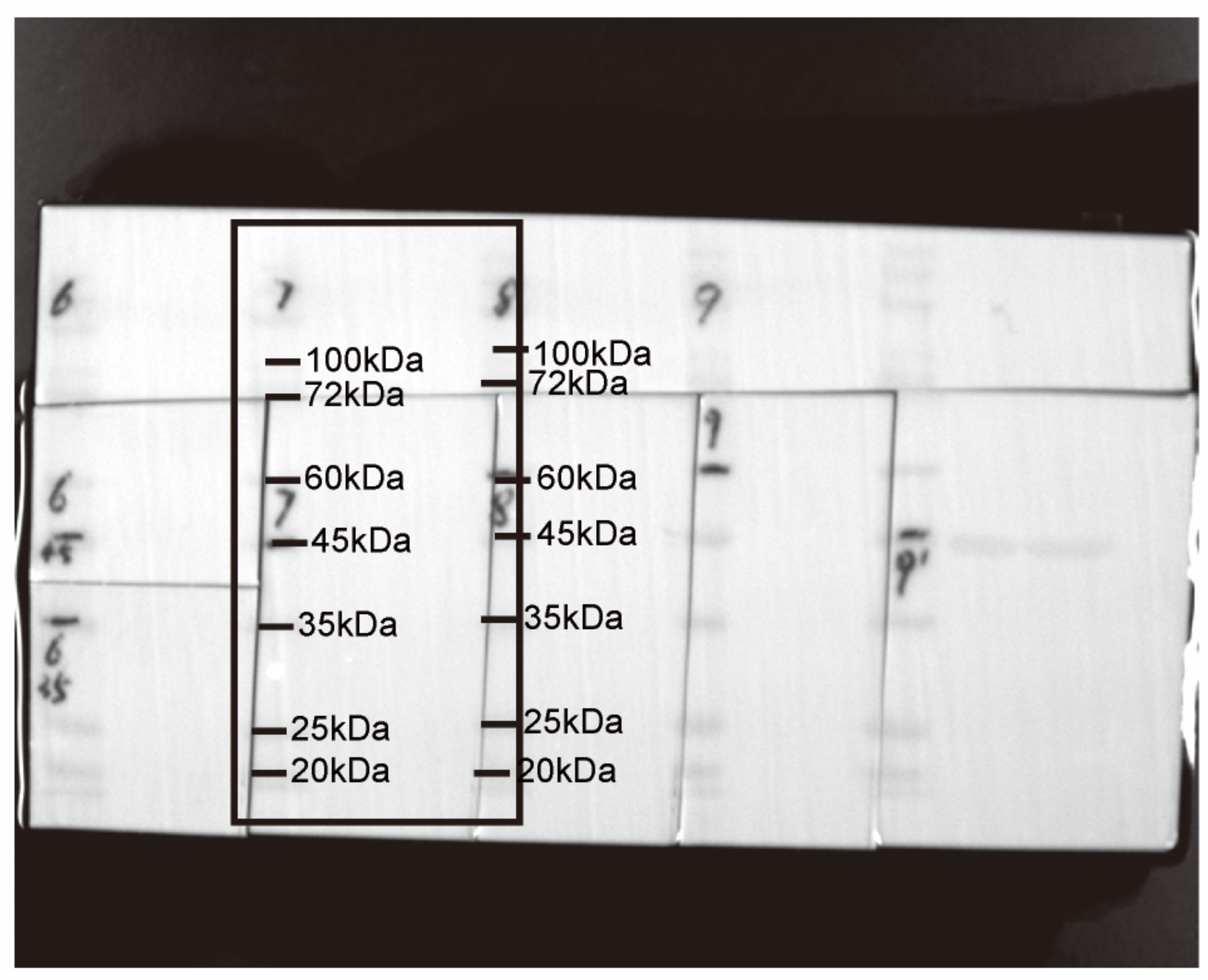

Figure	Merged	Stiched full image

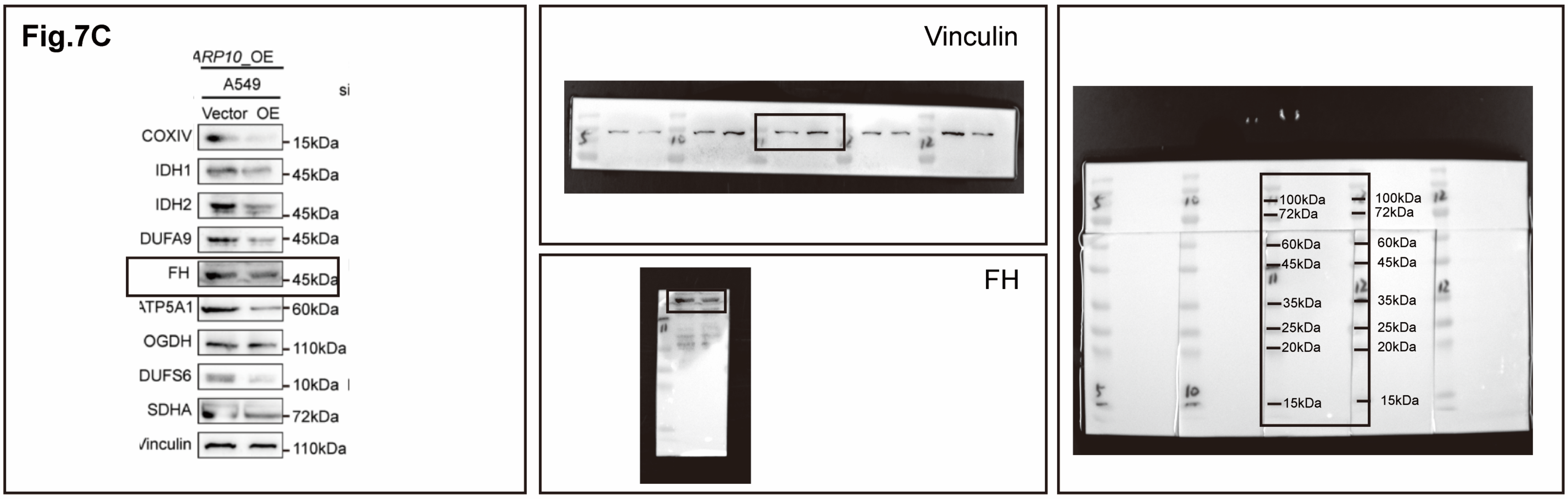

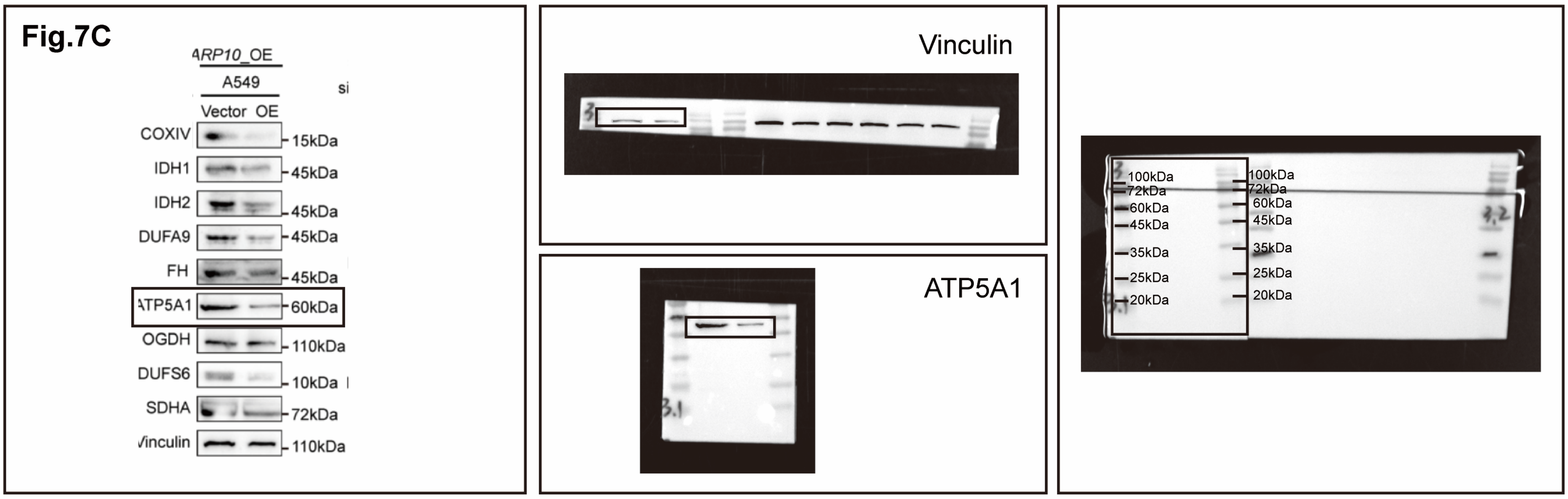

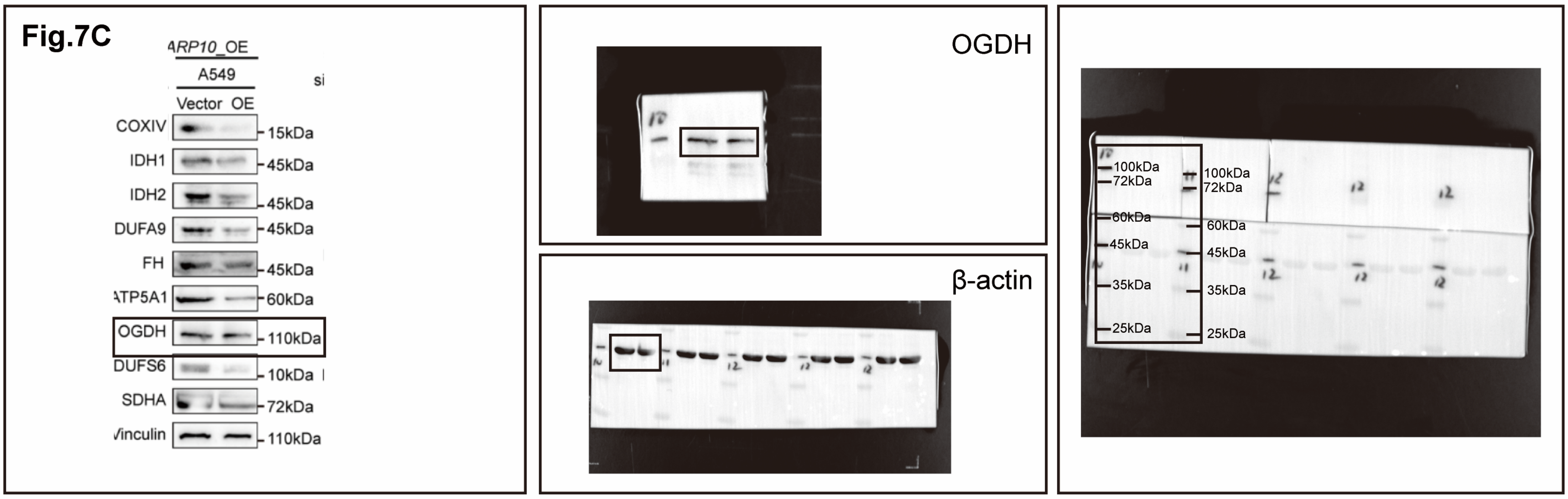

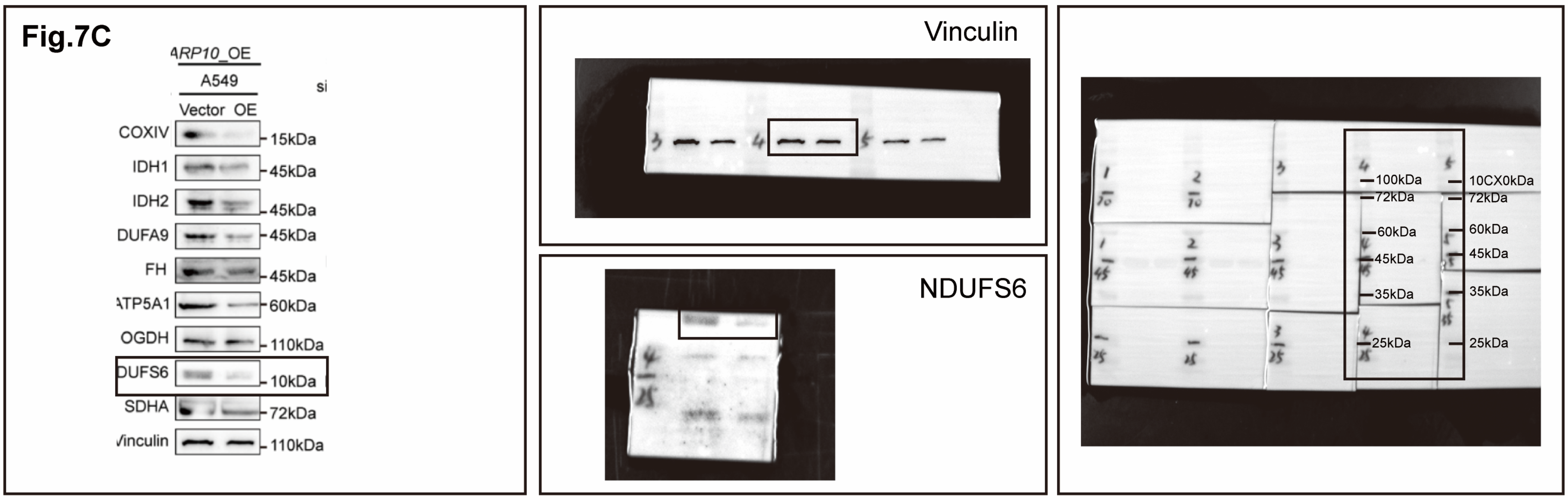

Figure	Merged	Stiched full image

Fig.7C

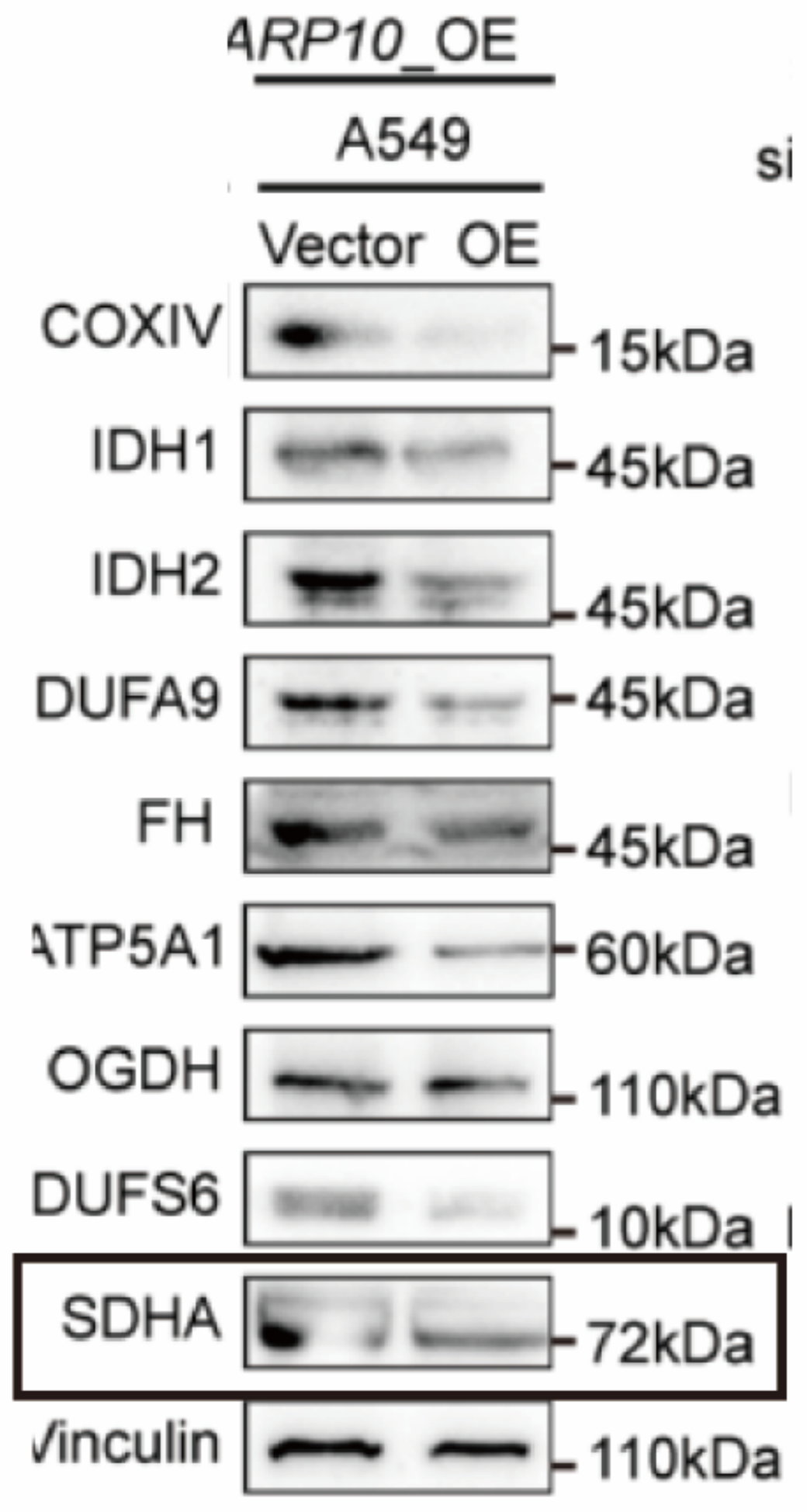

SDHA

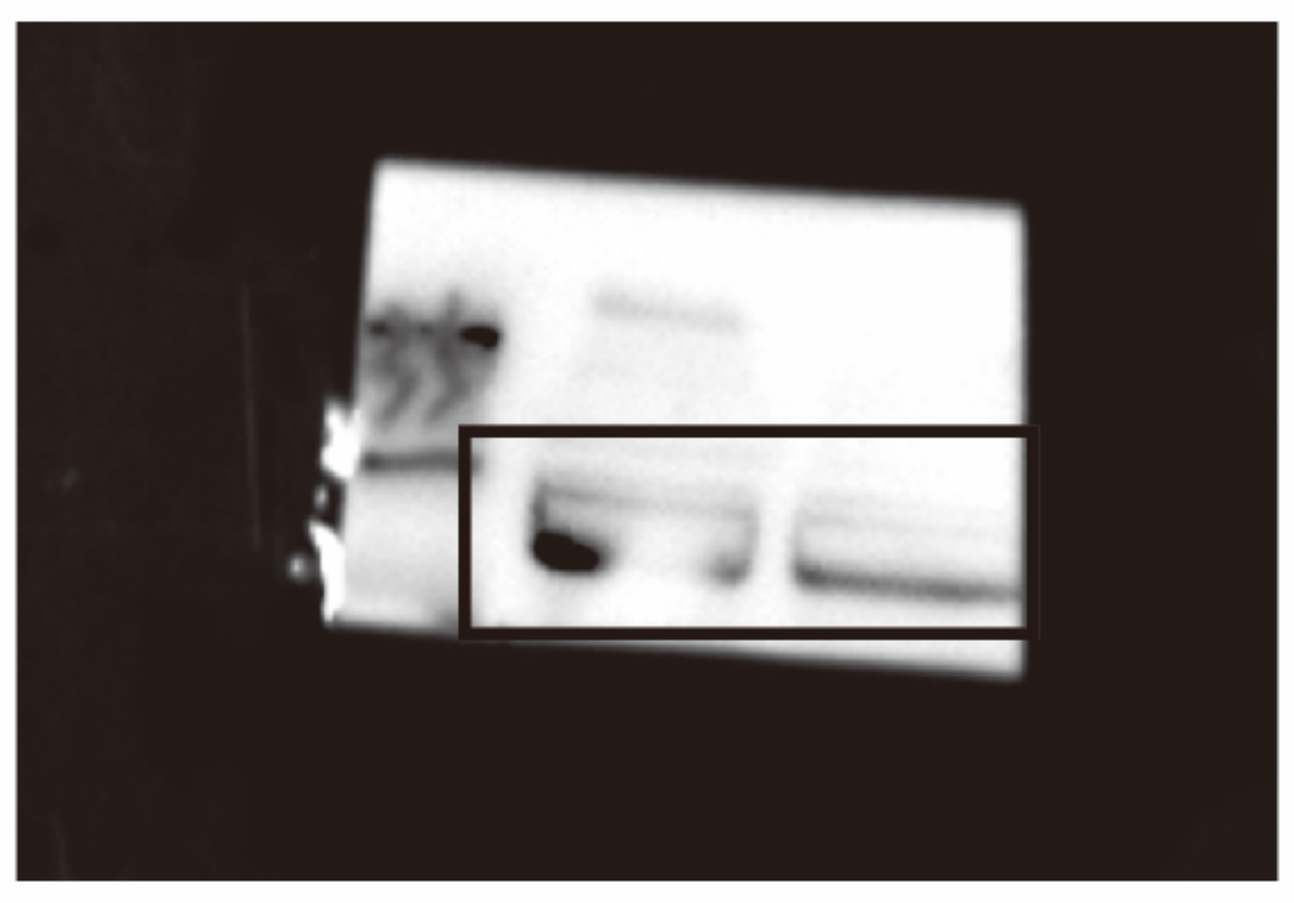

β-actin

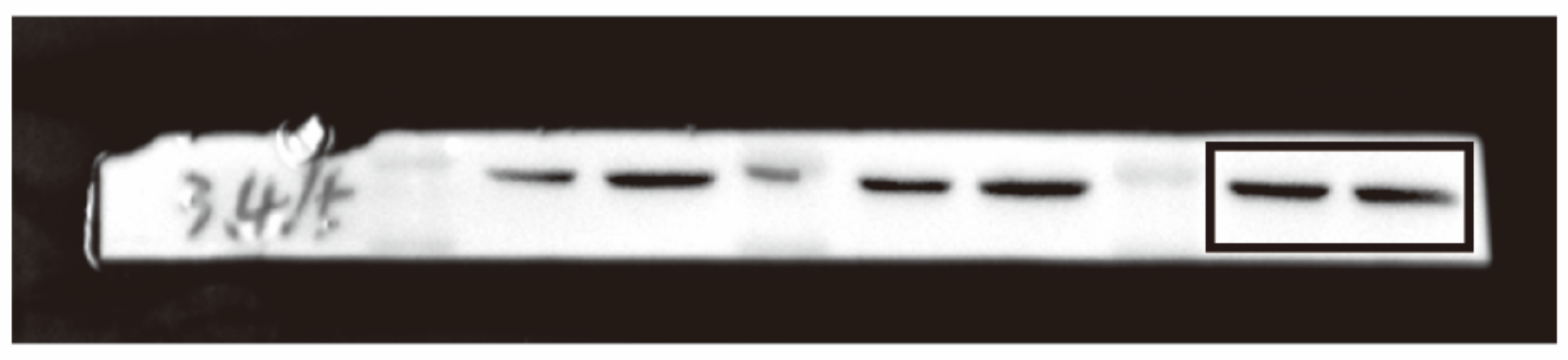

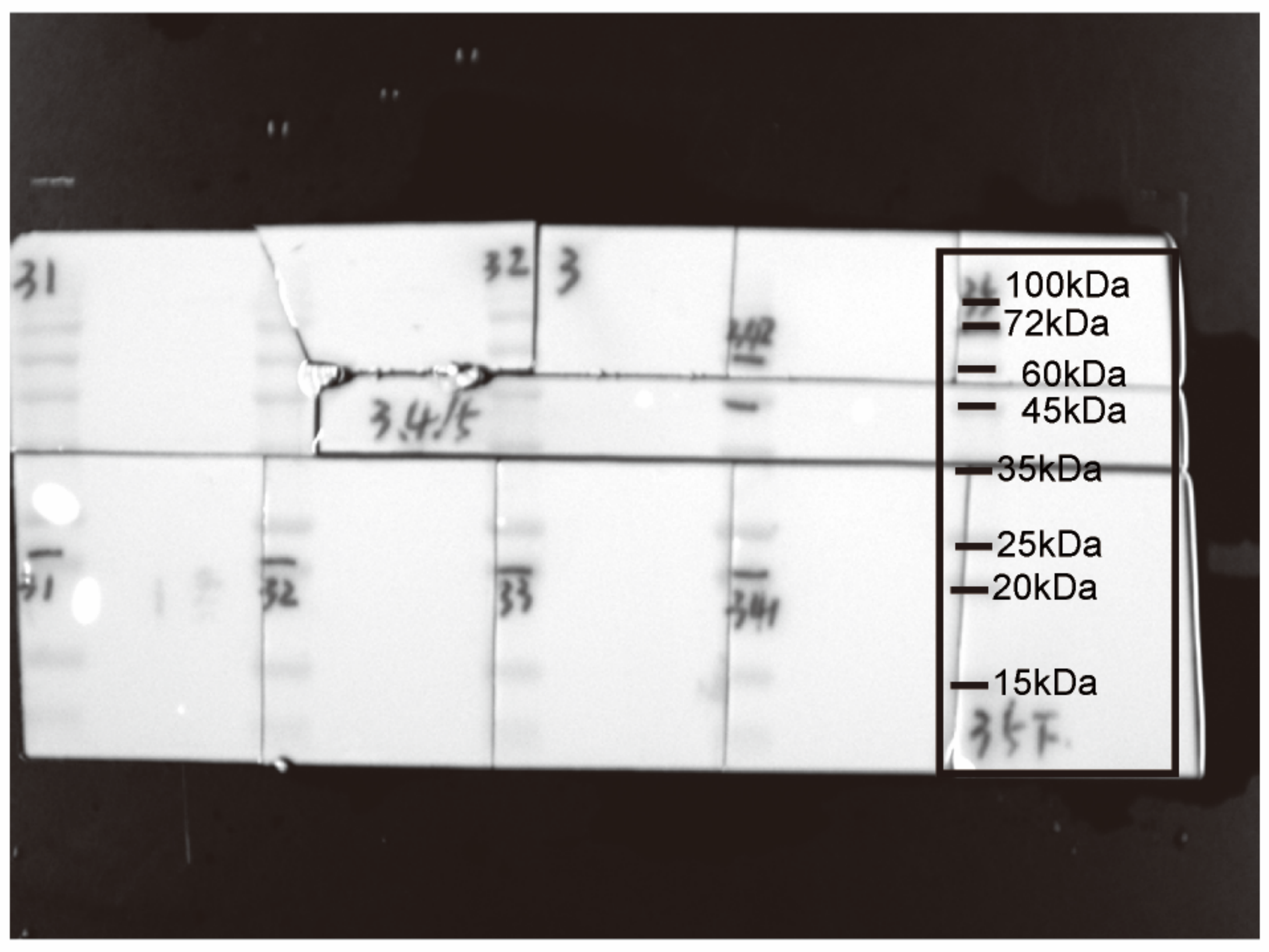

Fig.7C

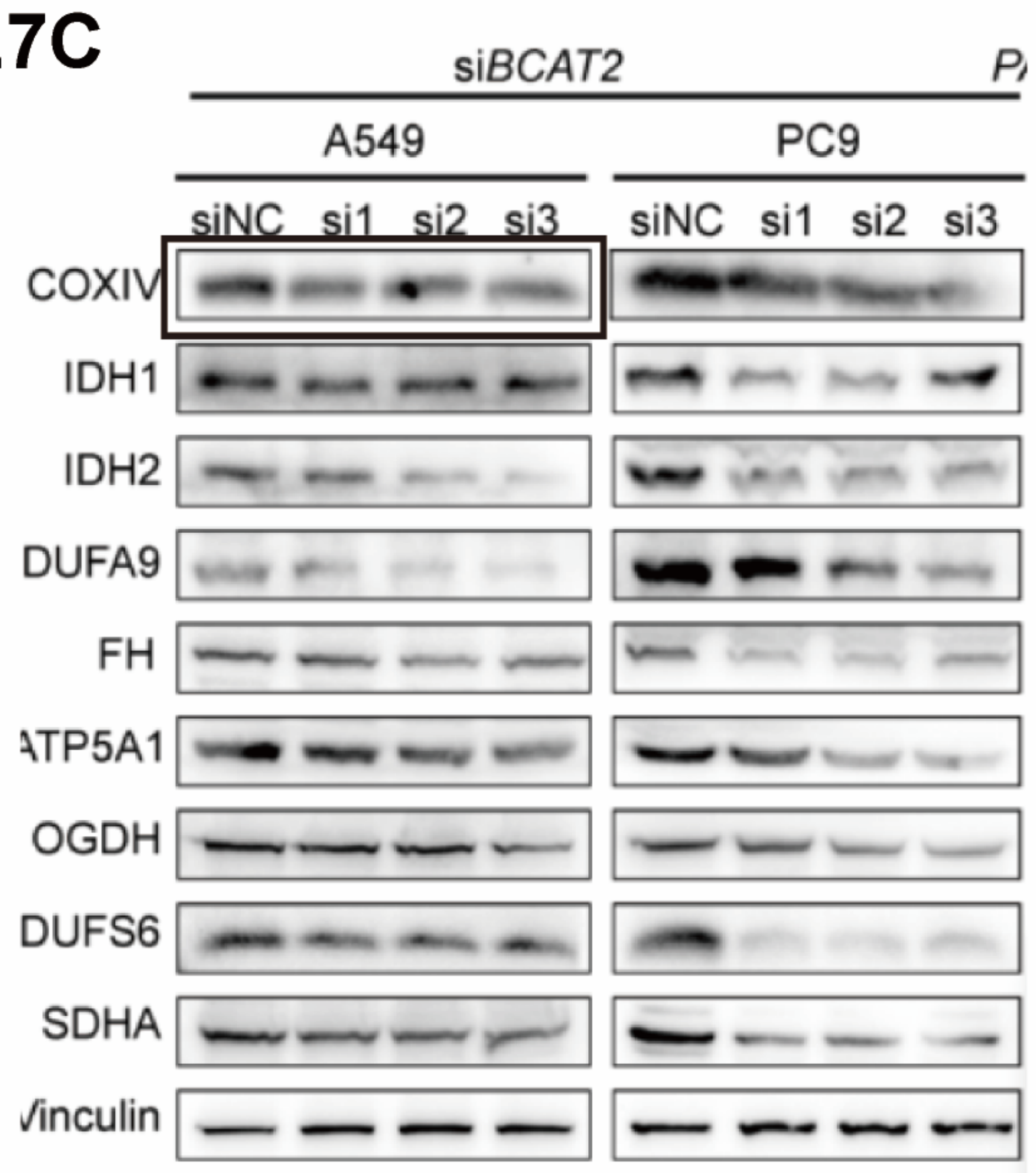

Vinculin

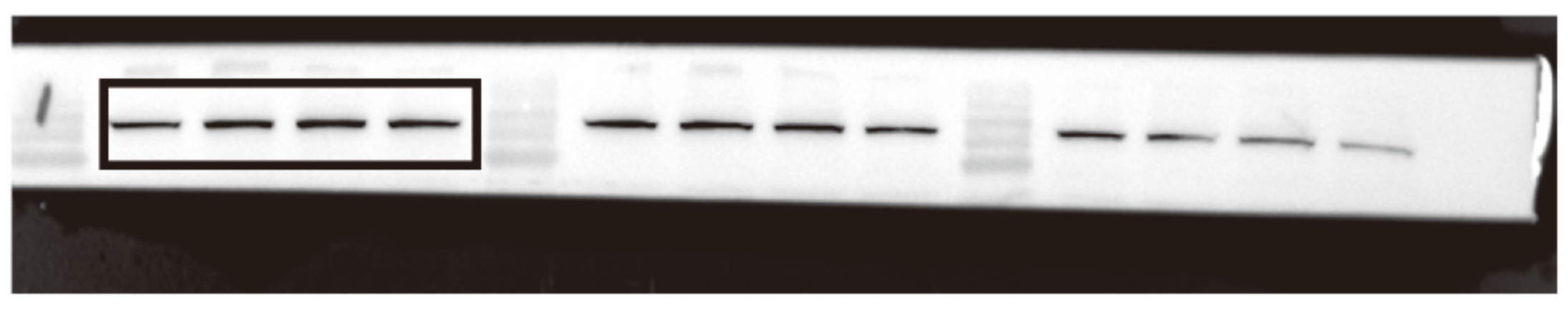

COXIV

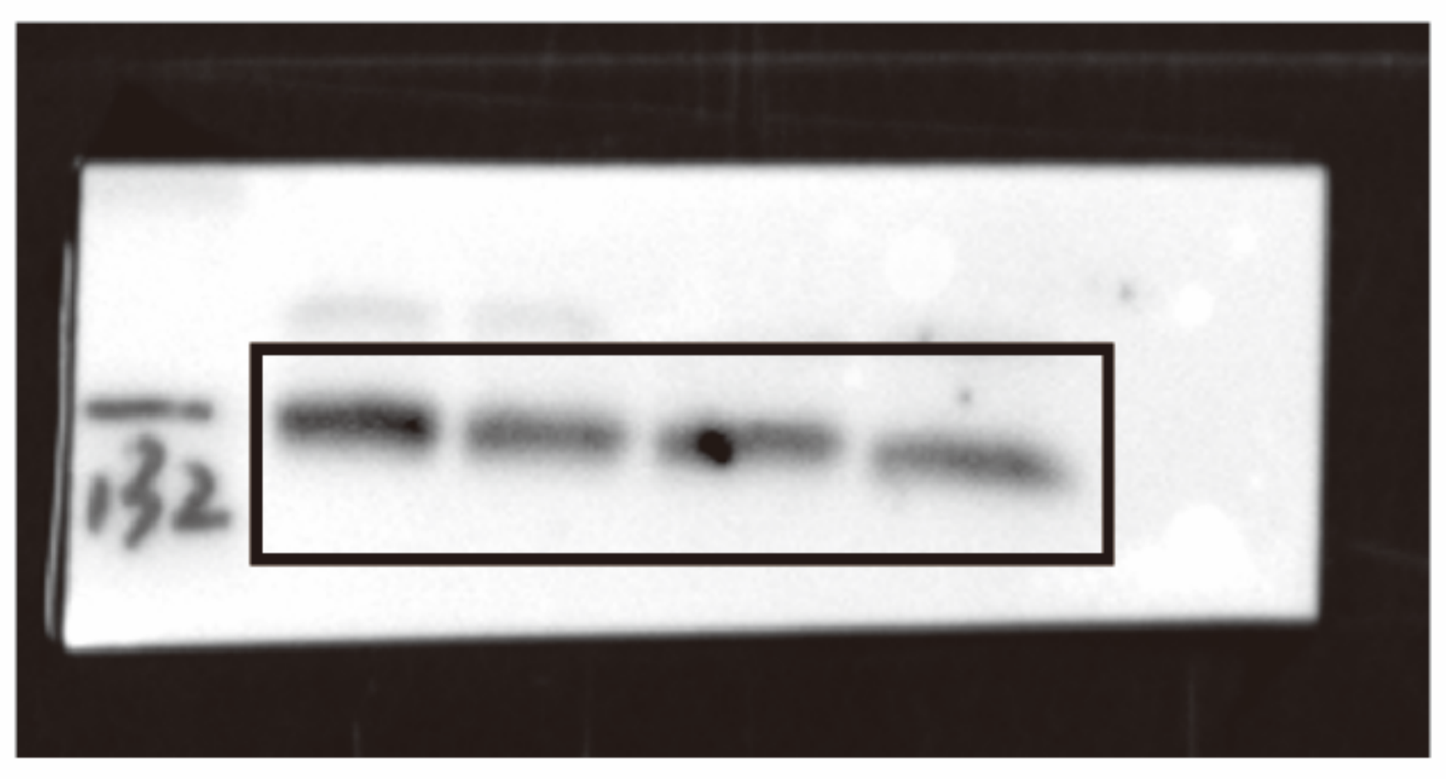

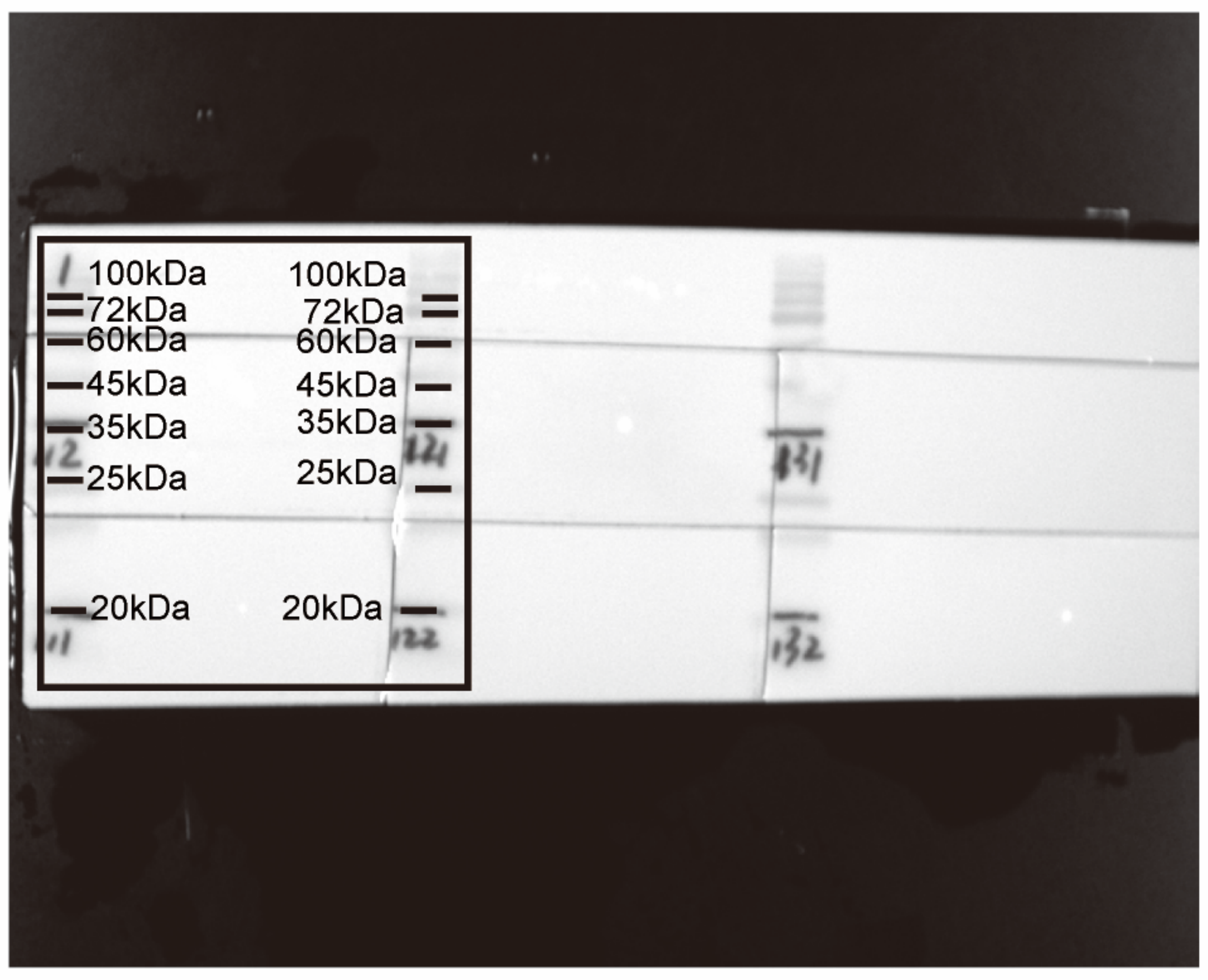

Fig.7C

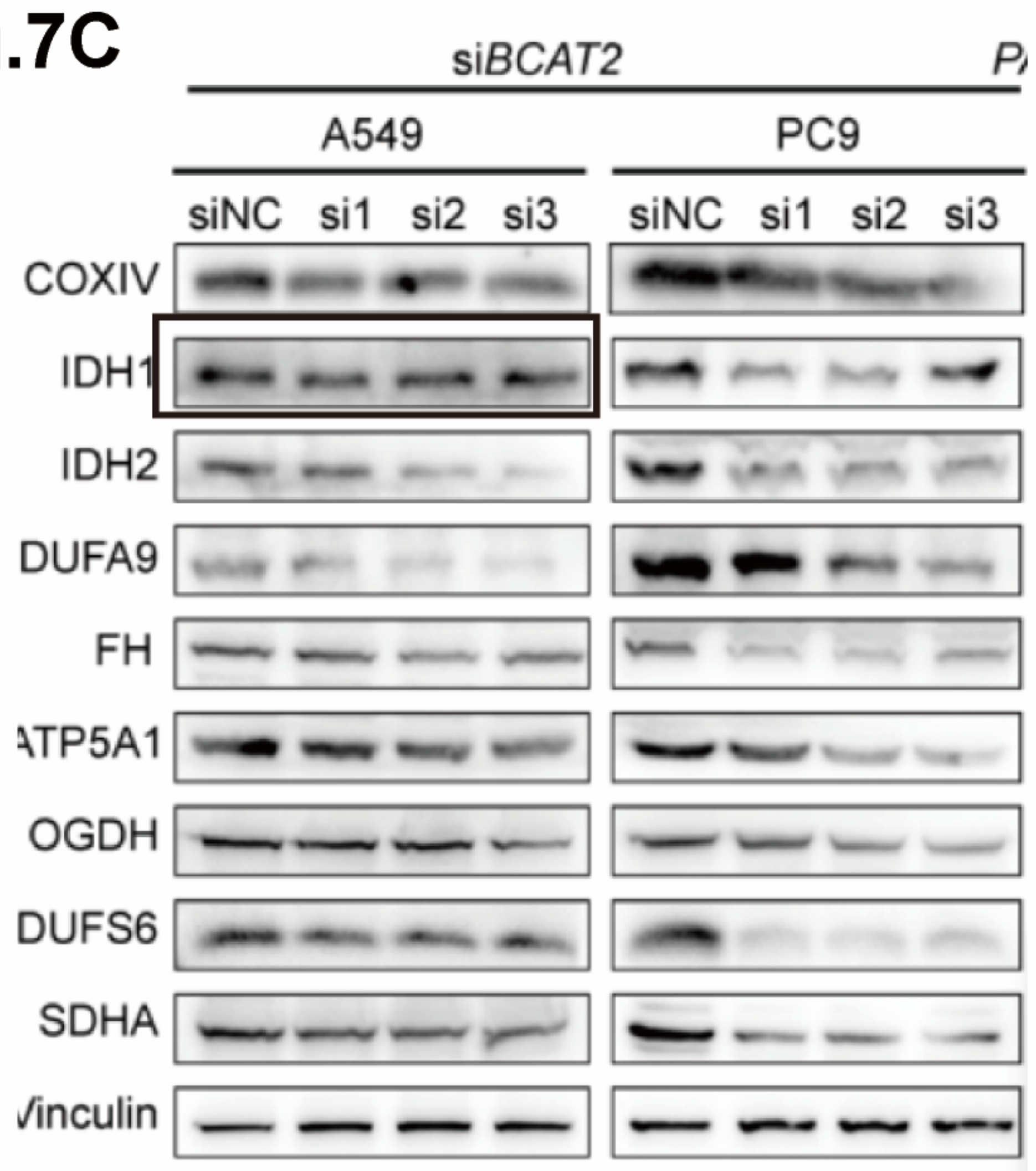

Vinculin

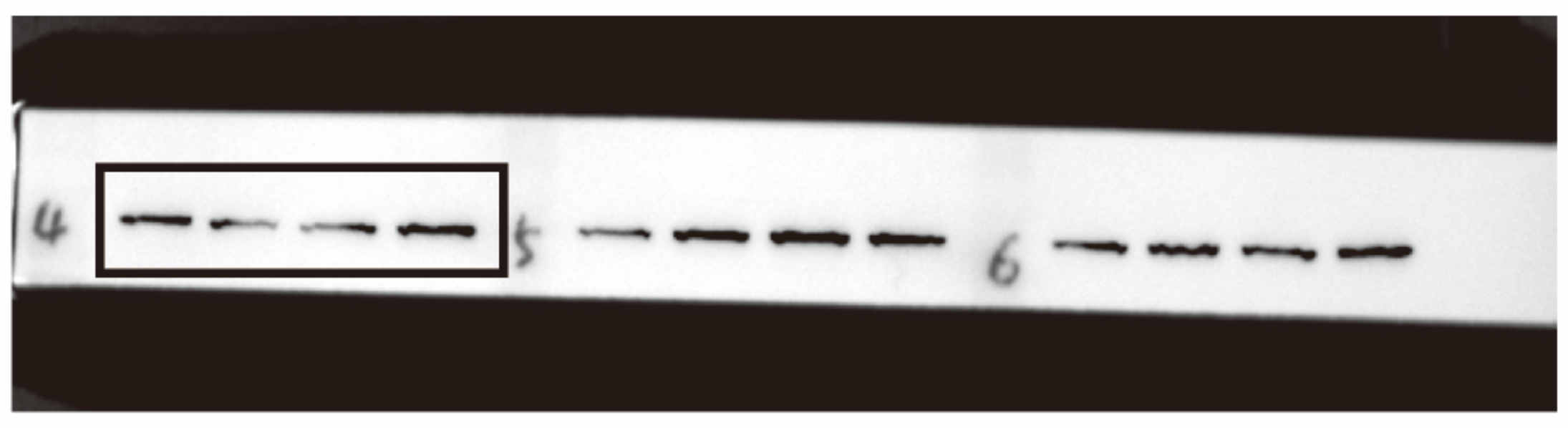

IDH1

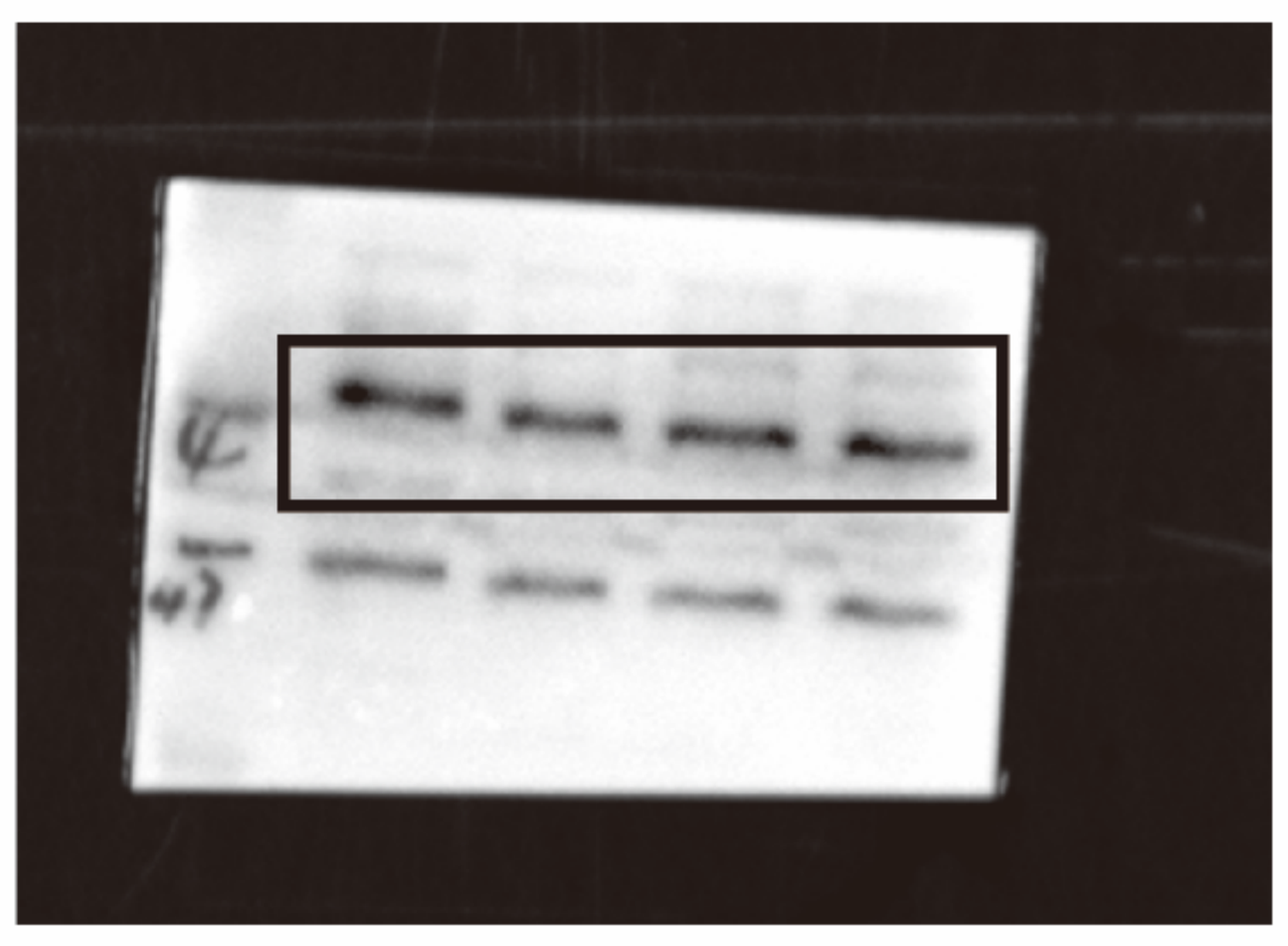

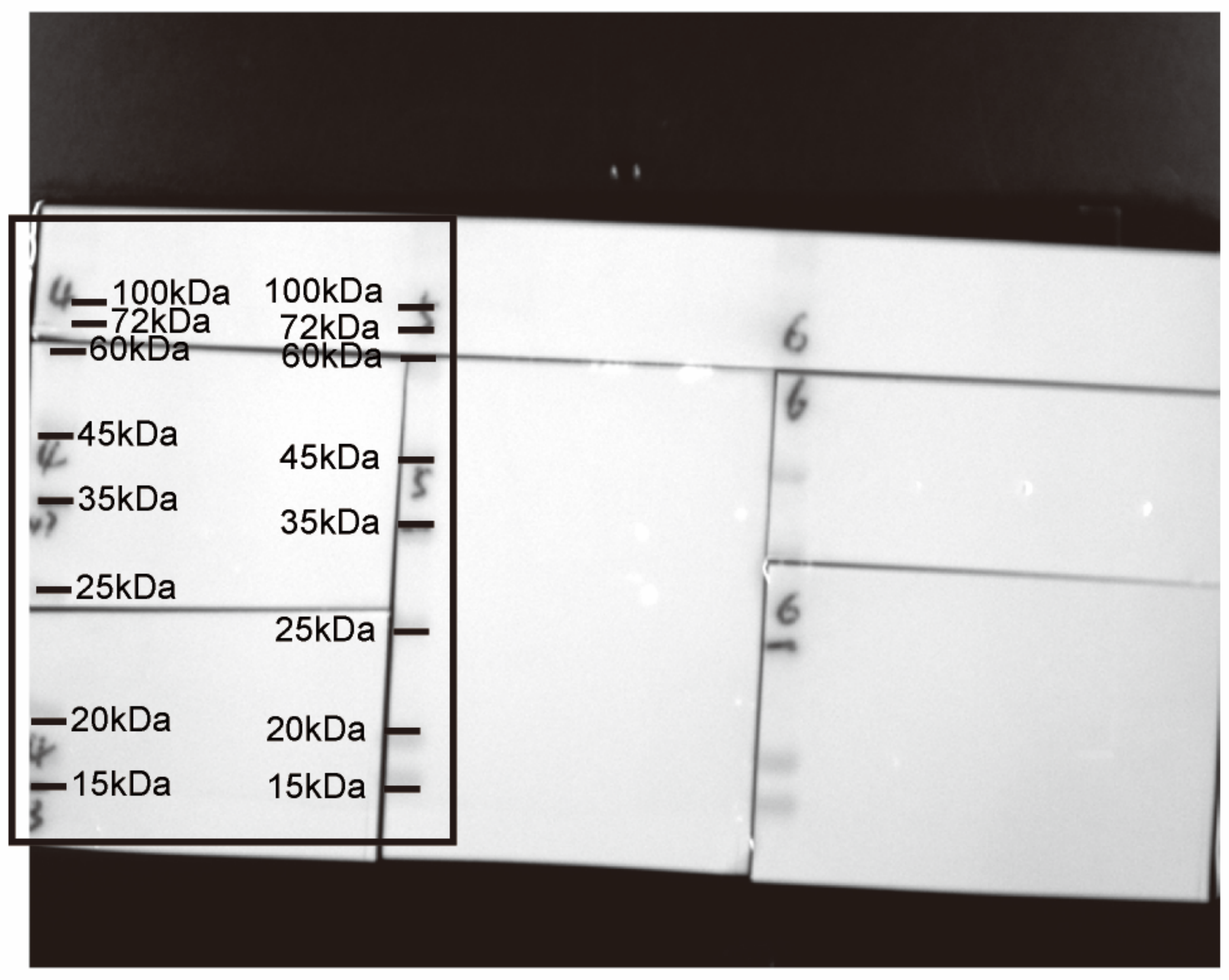

Fig.7C

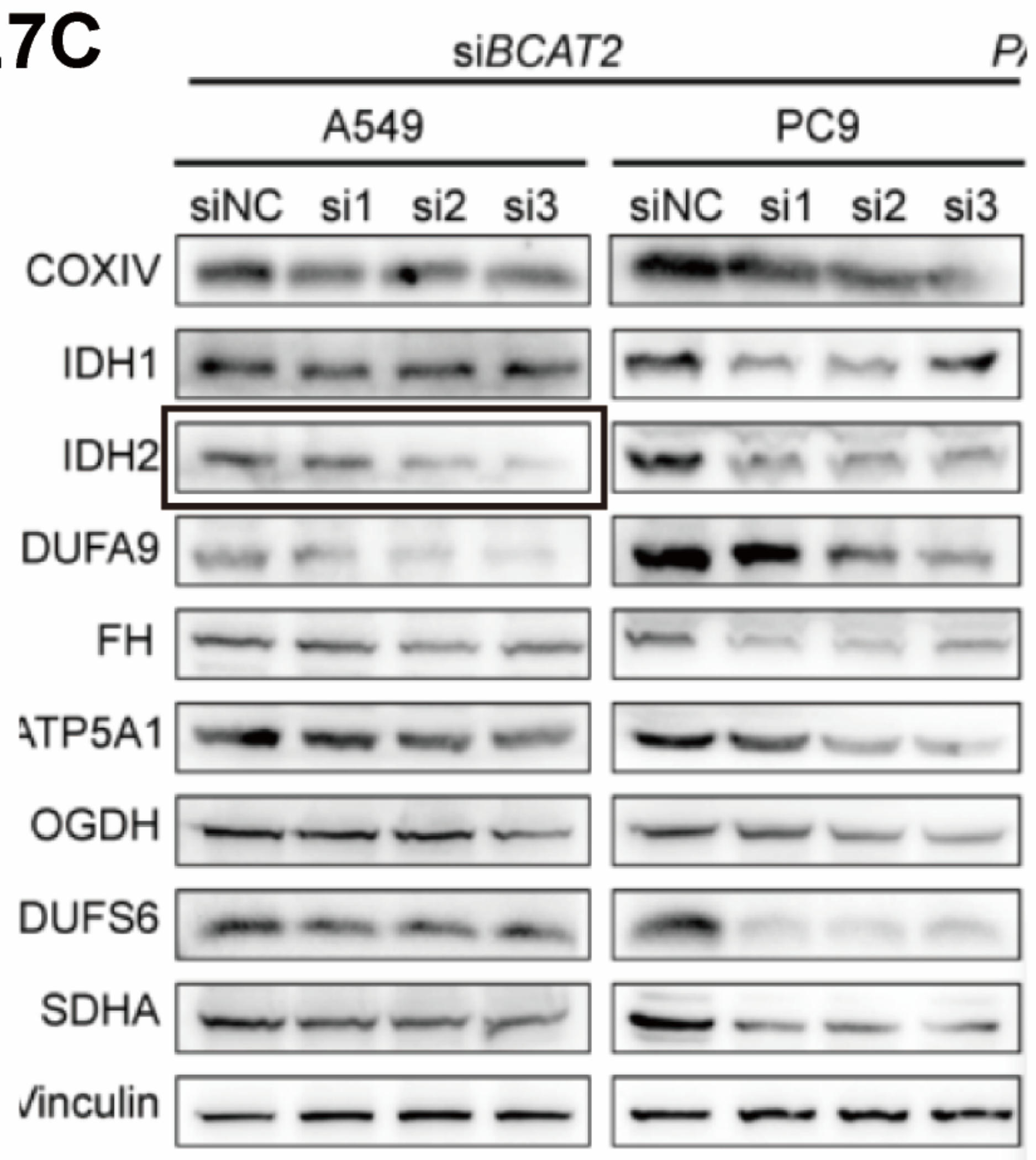

Vinculin

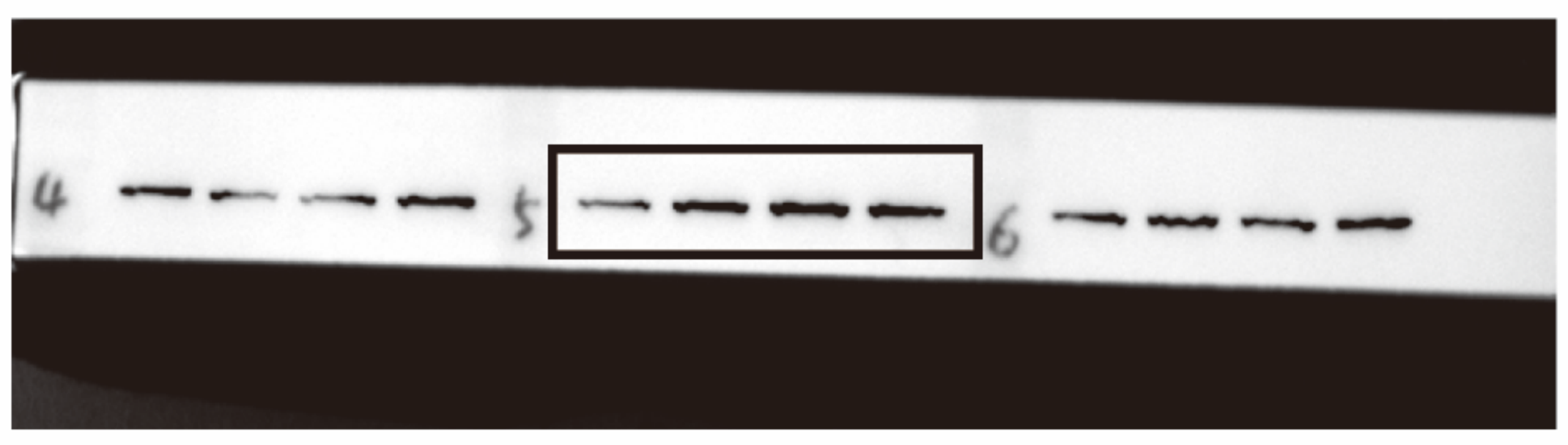

IDH2

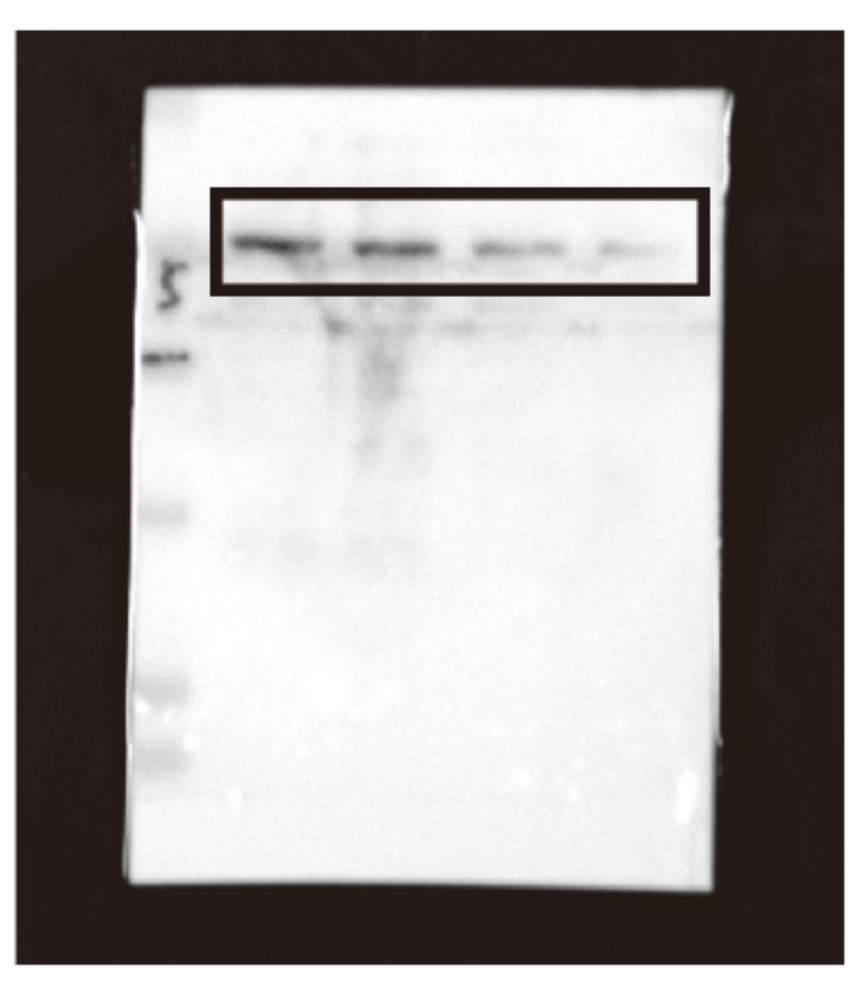

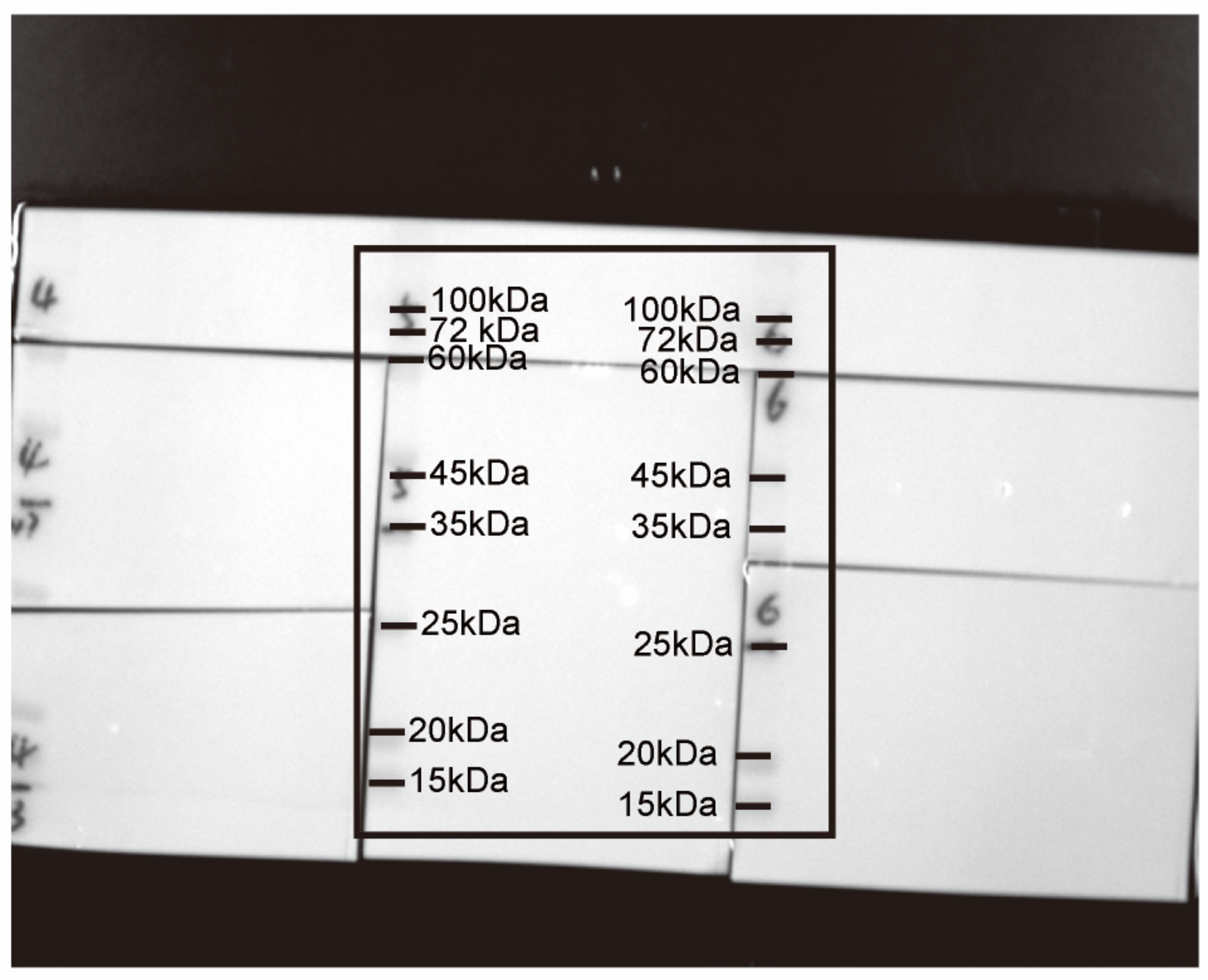

Figure	Merged	Stiched full image

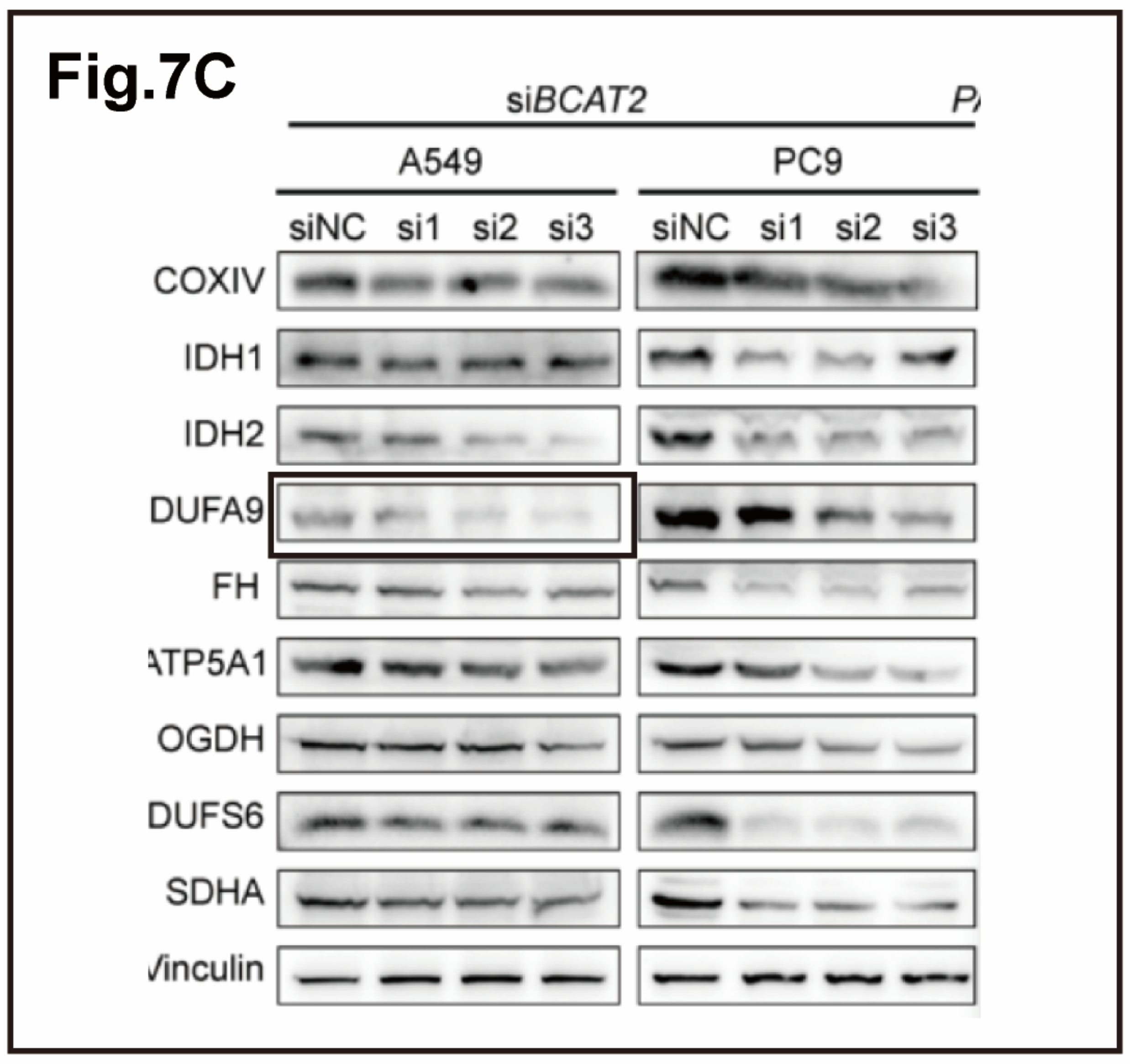

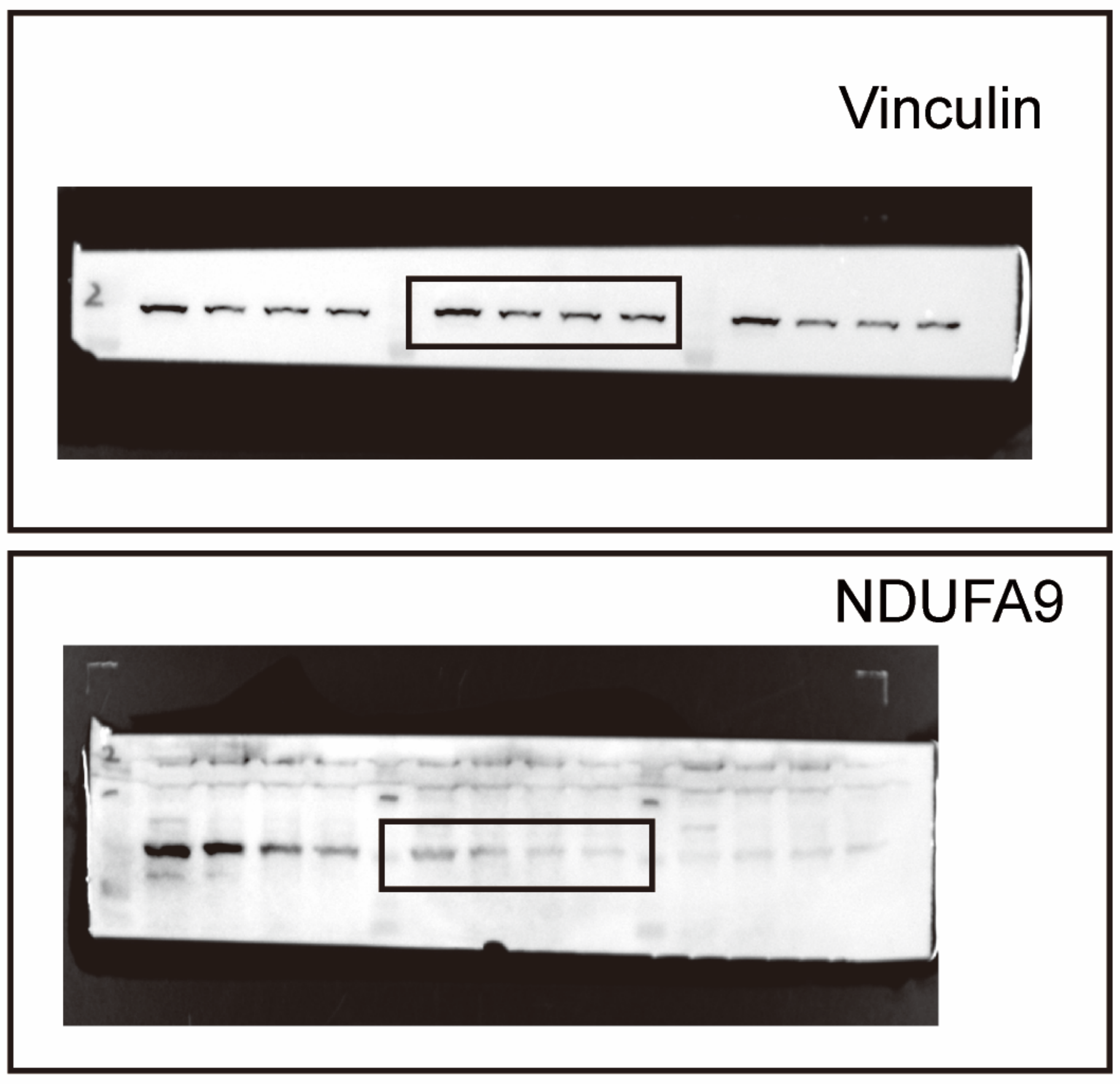

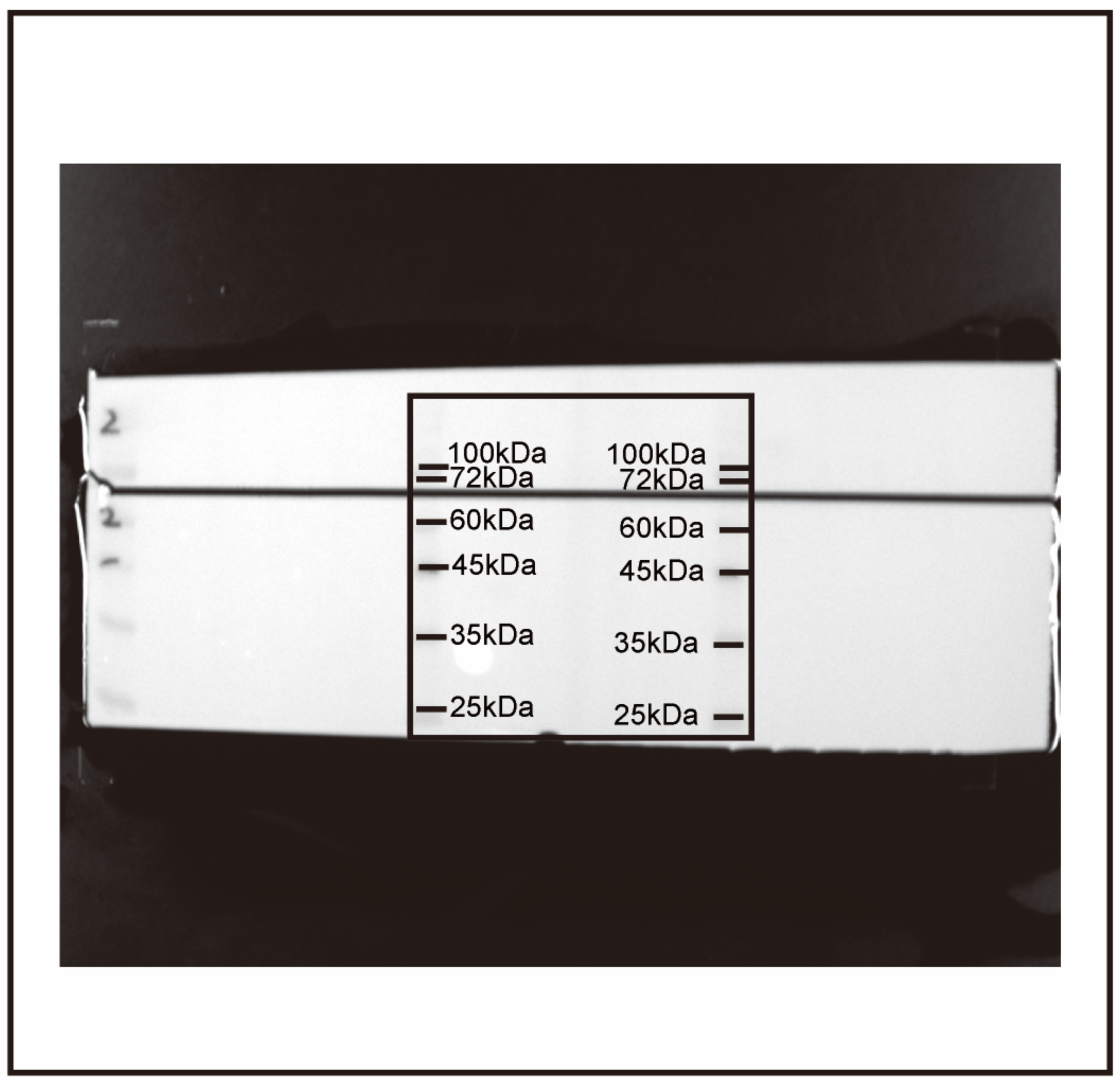

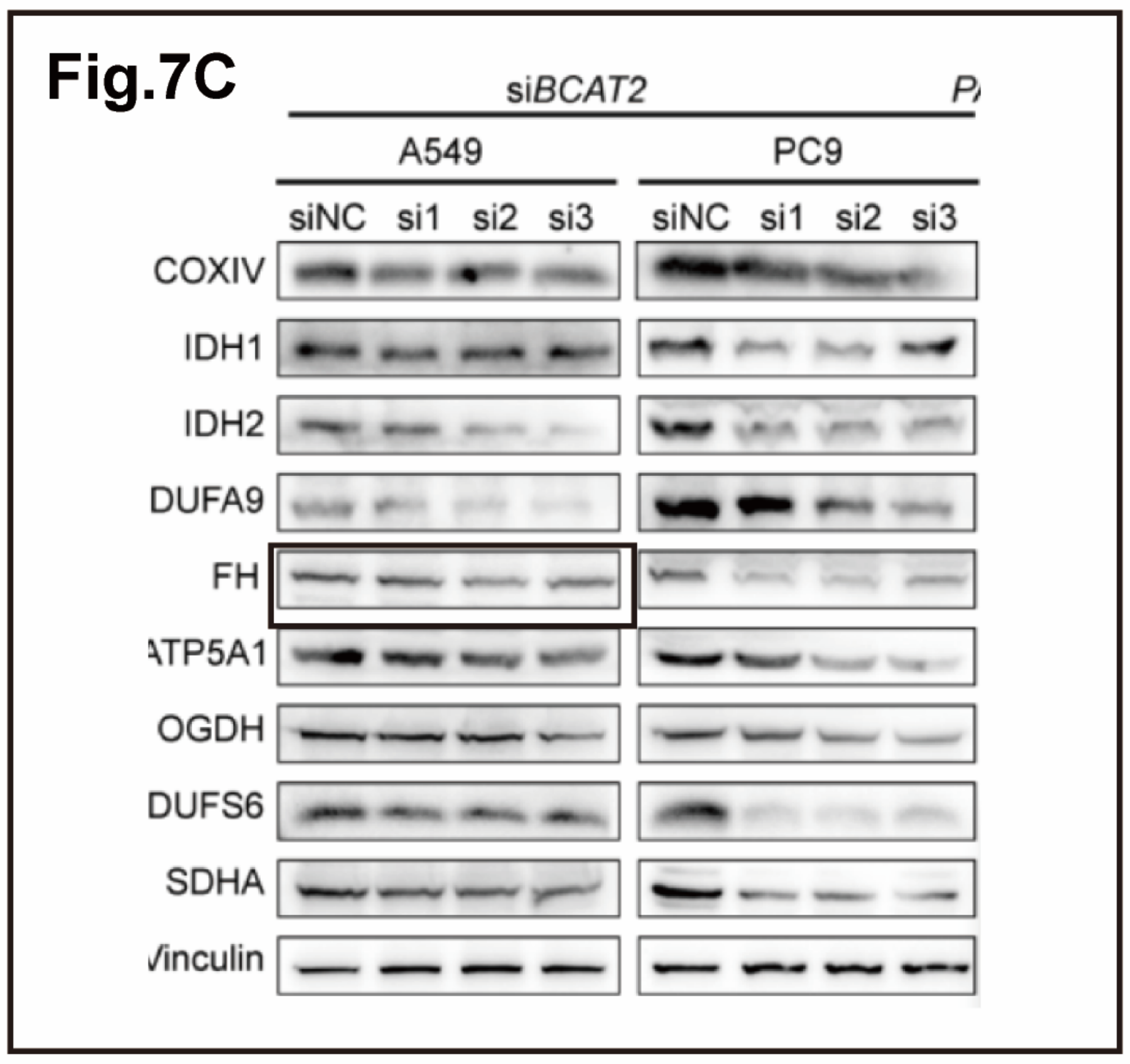

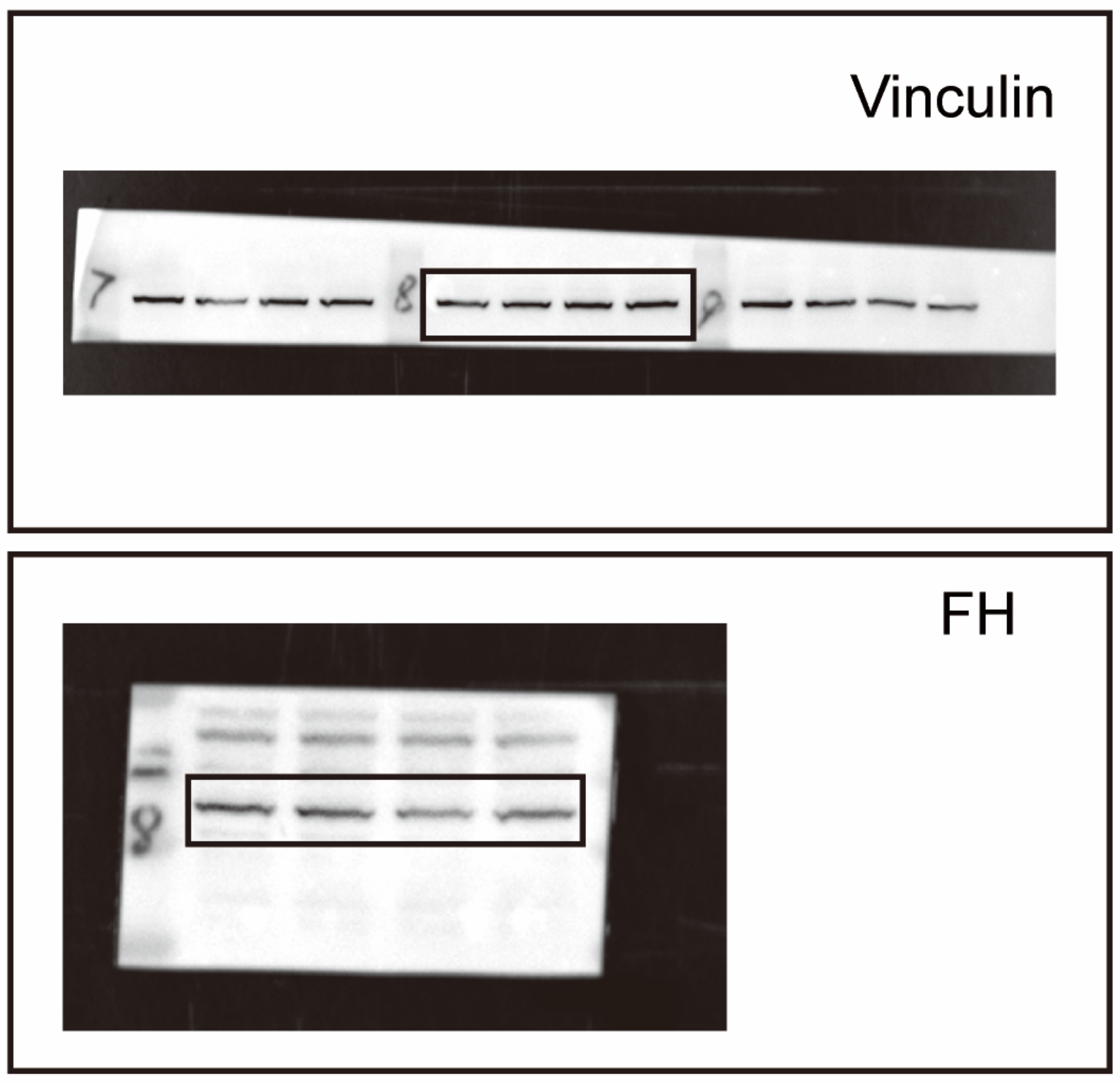

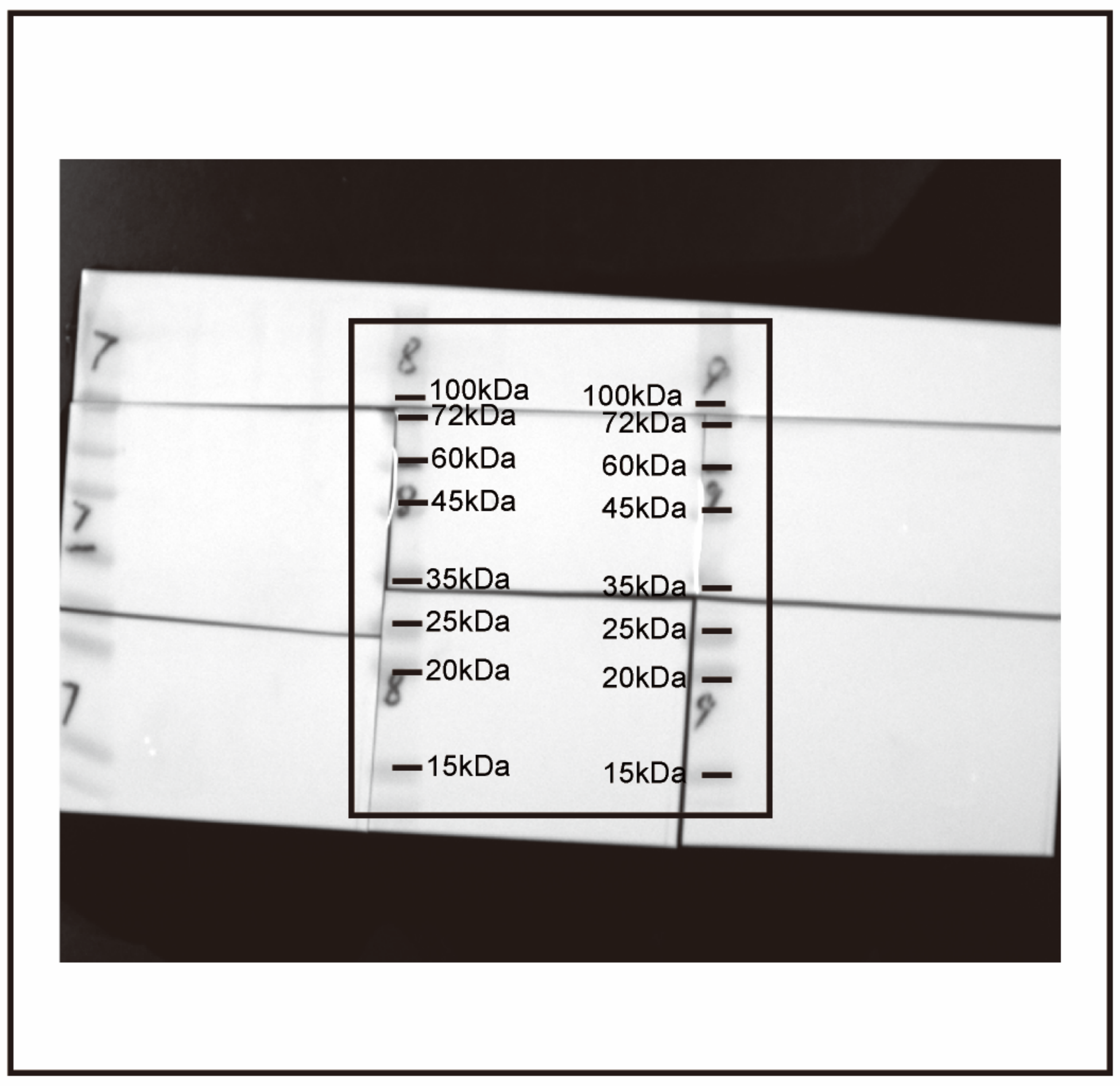

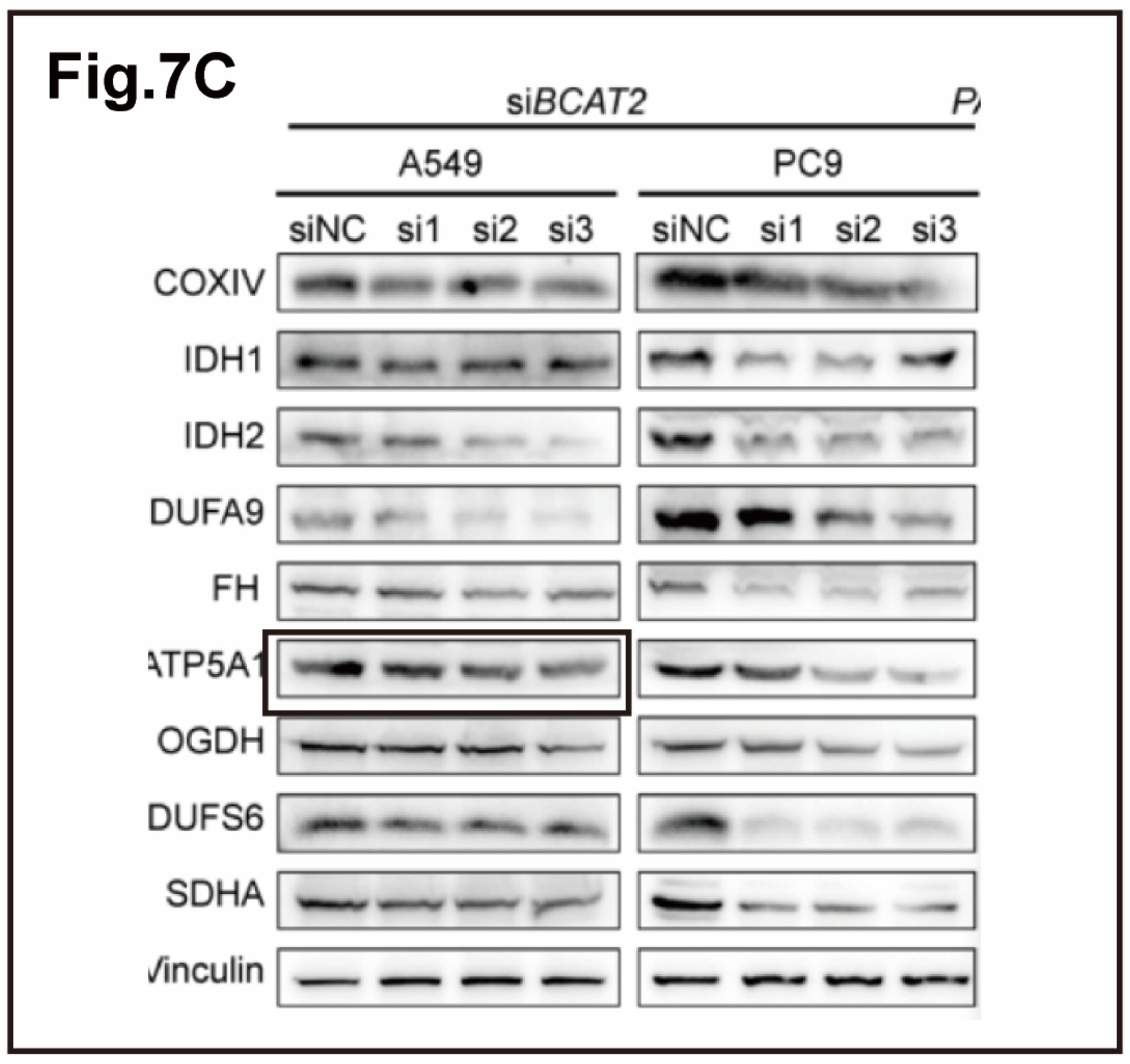

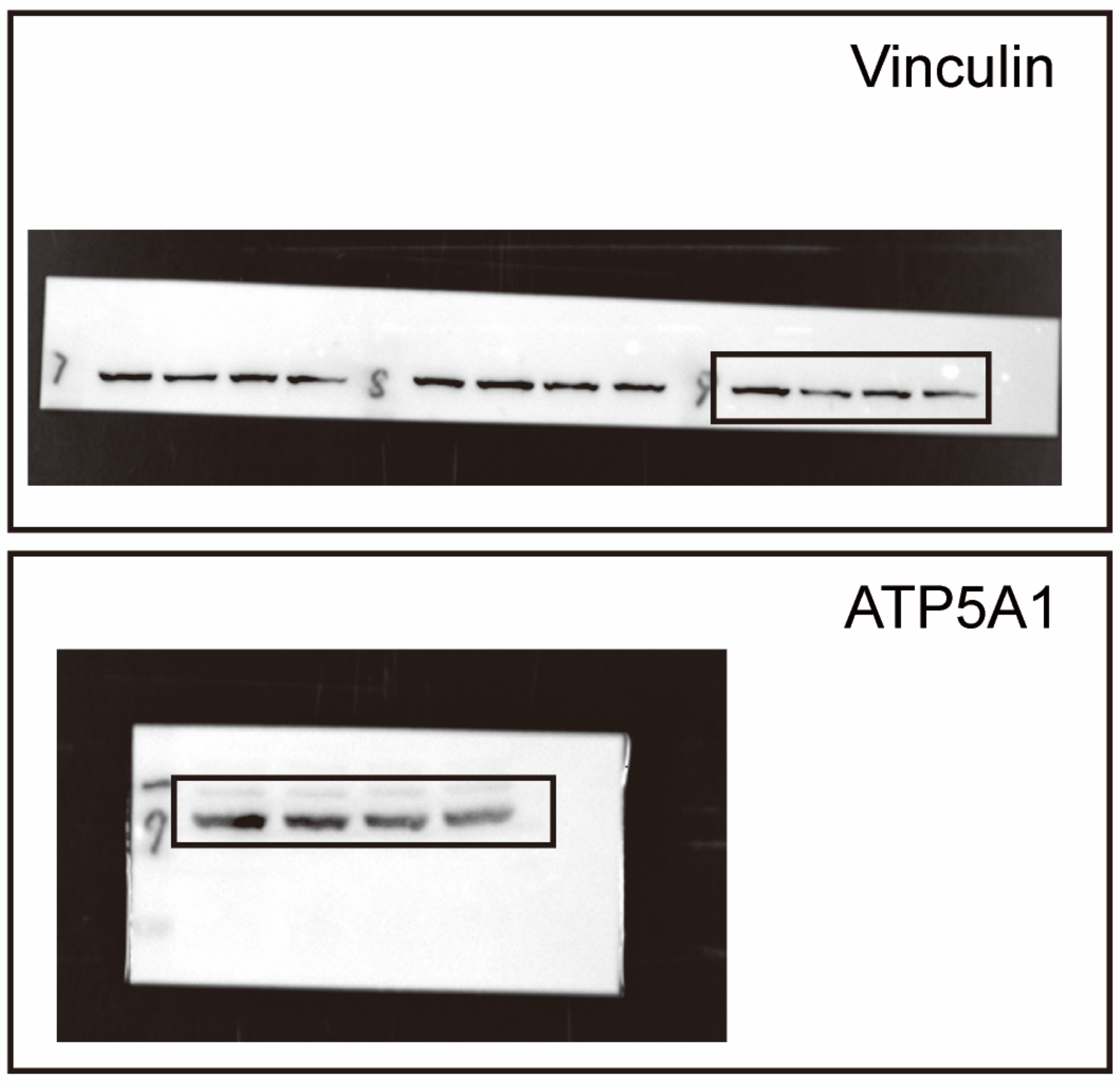

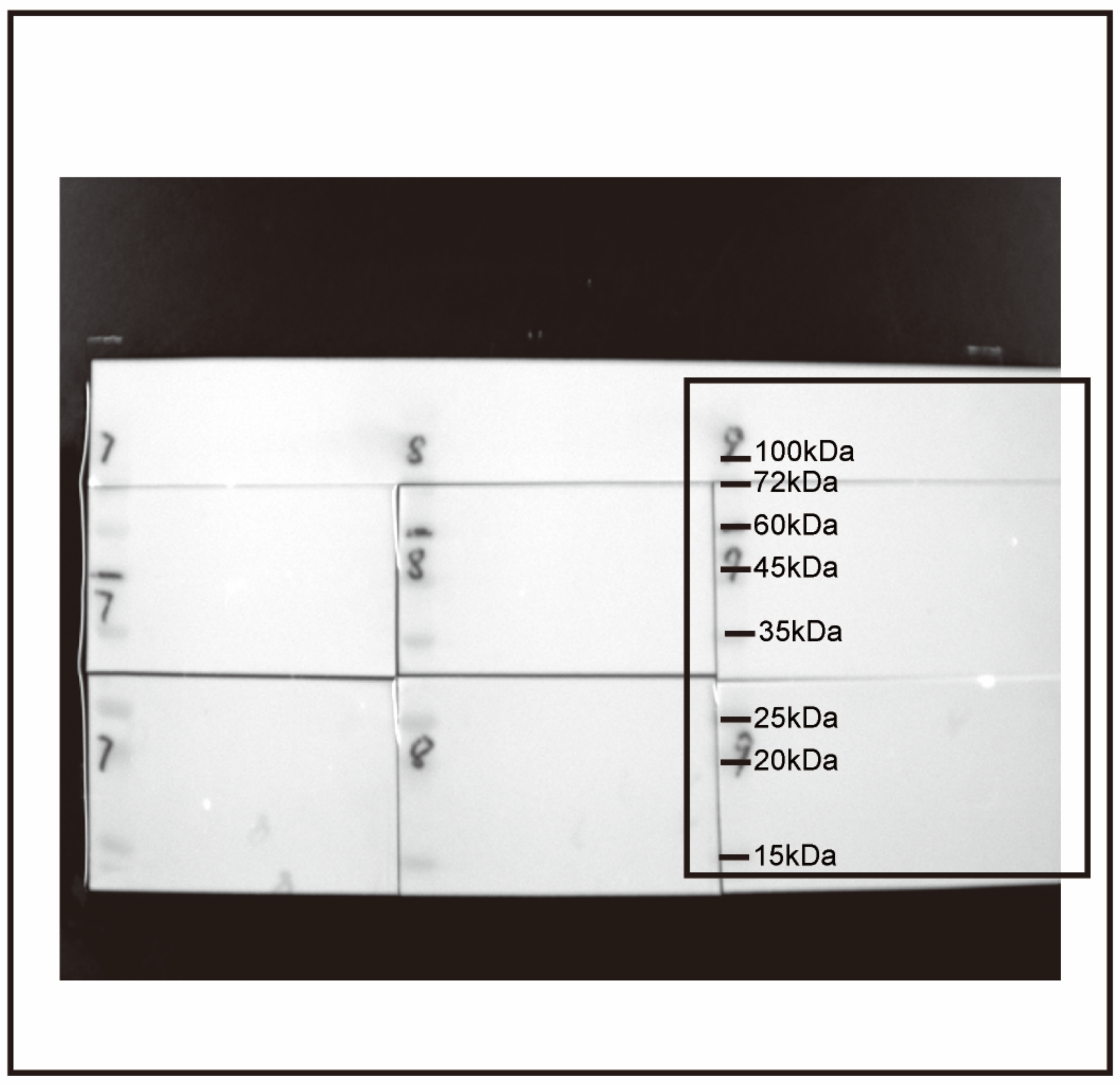

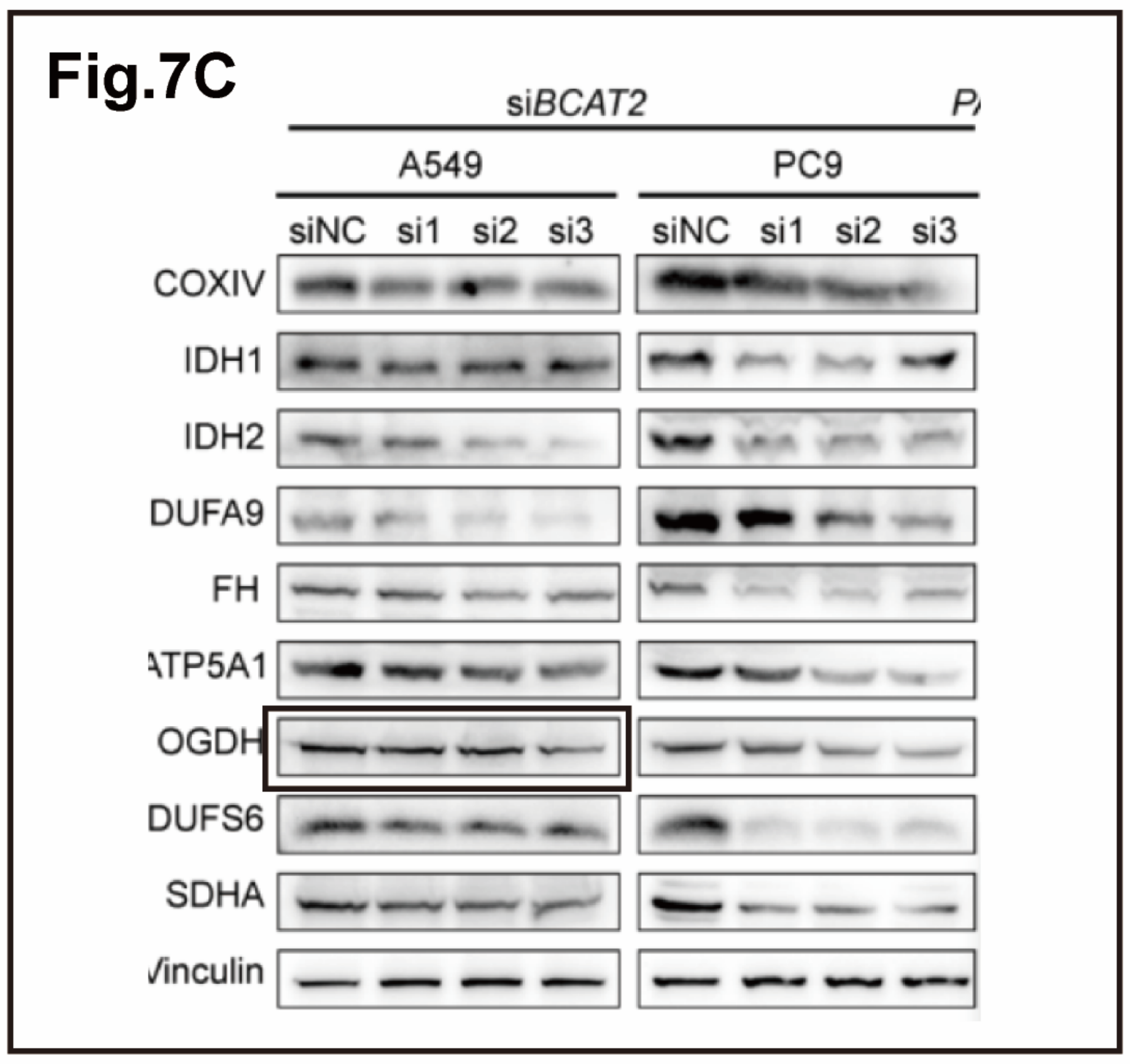

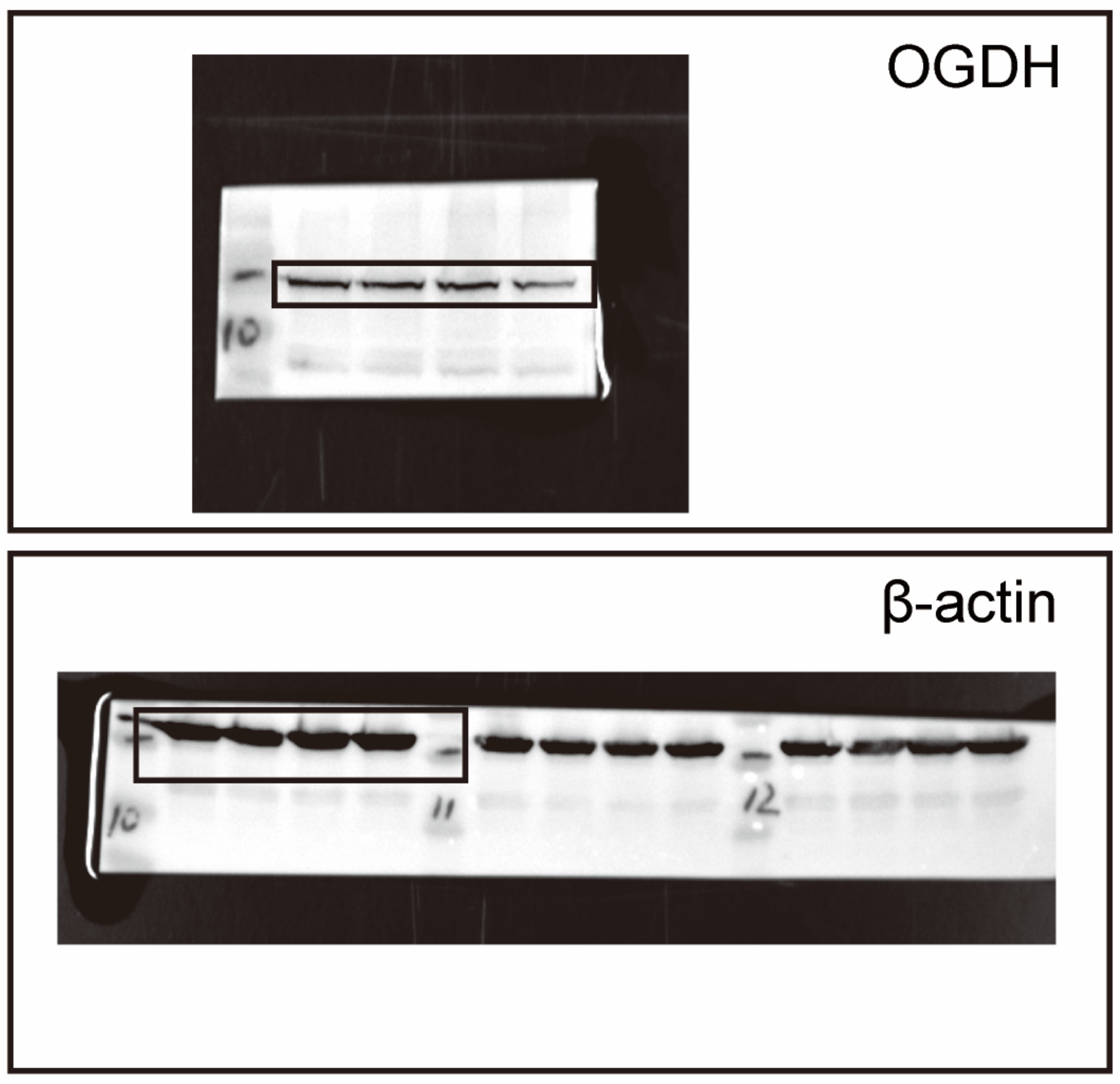

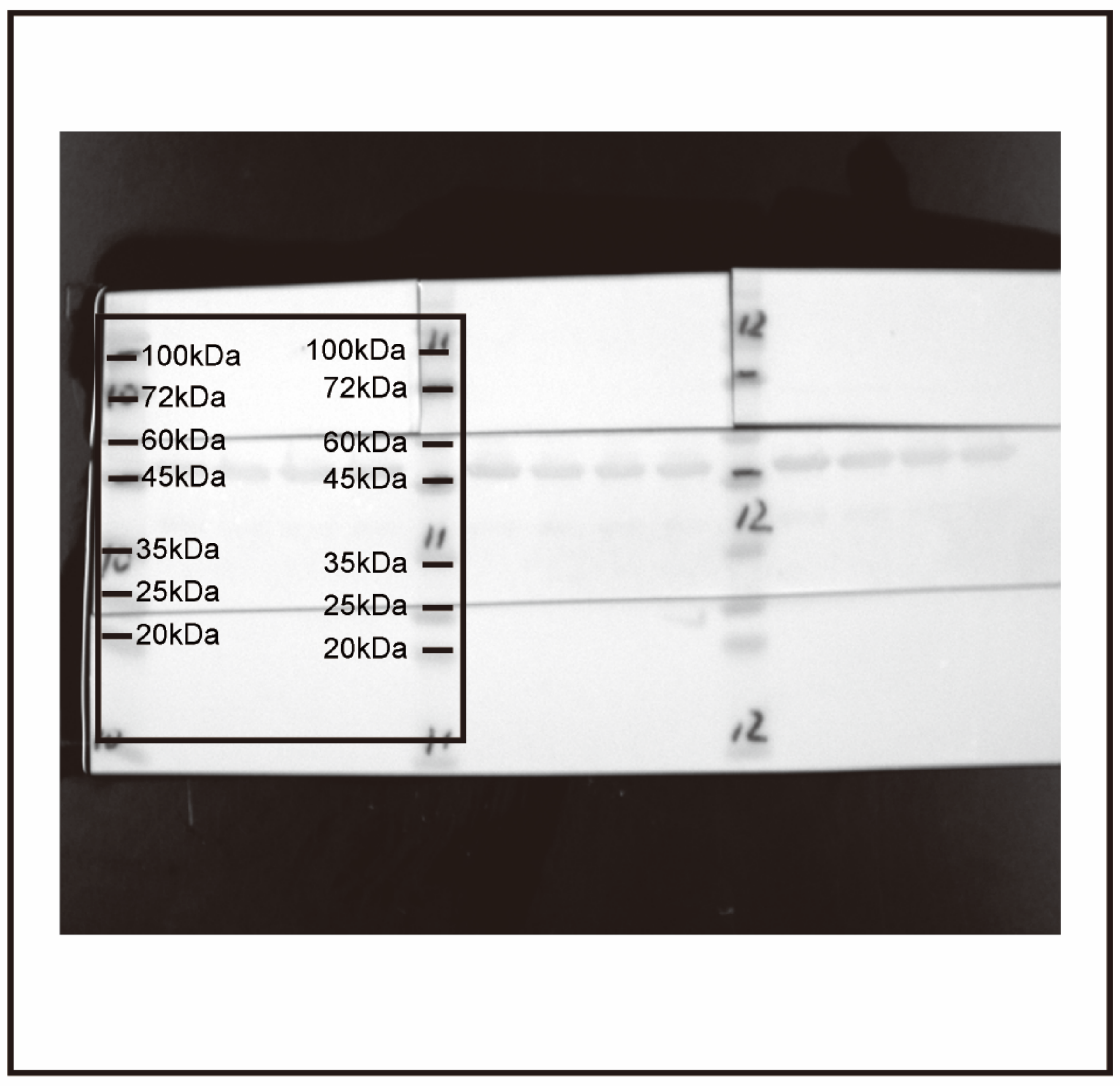

Figure	Merged	Stiched full image

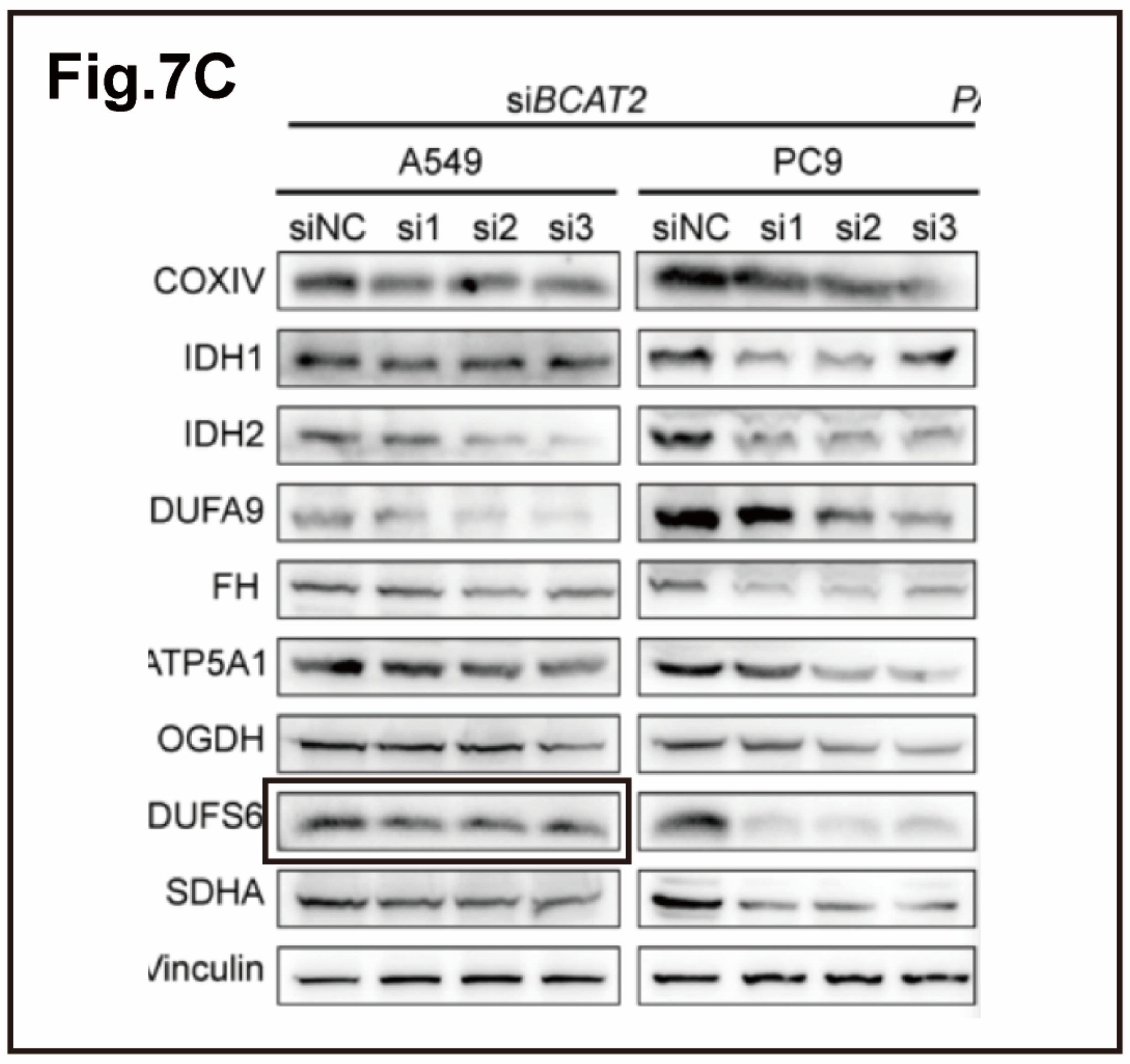

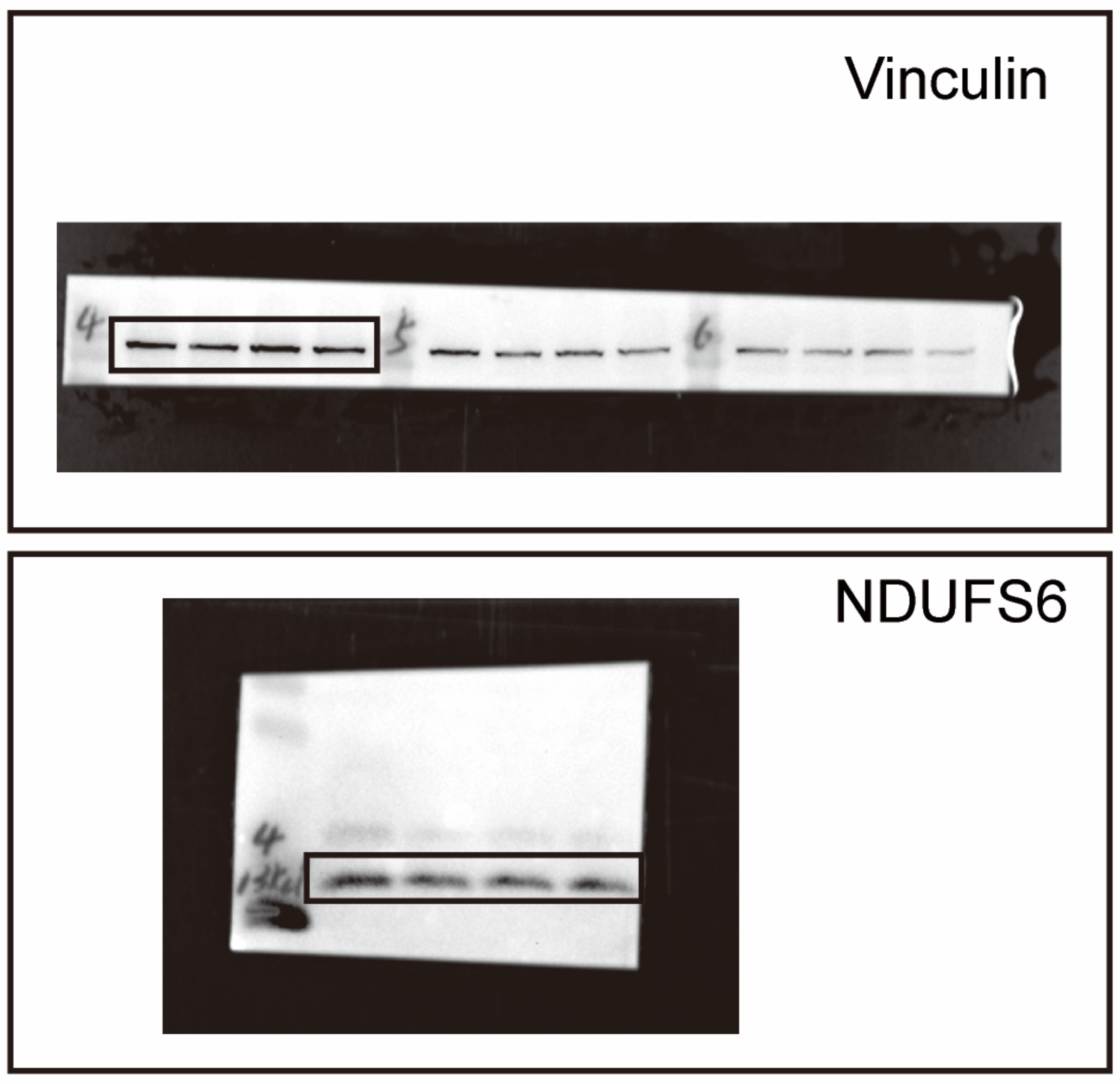

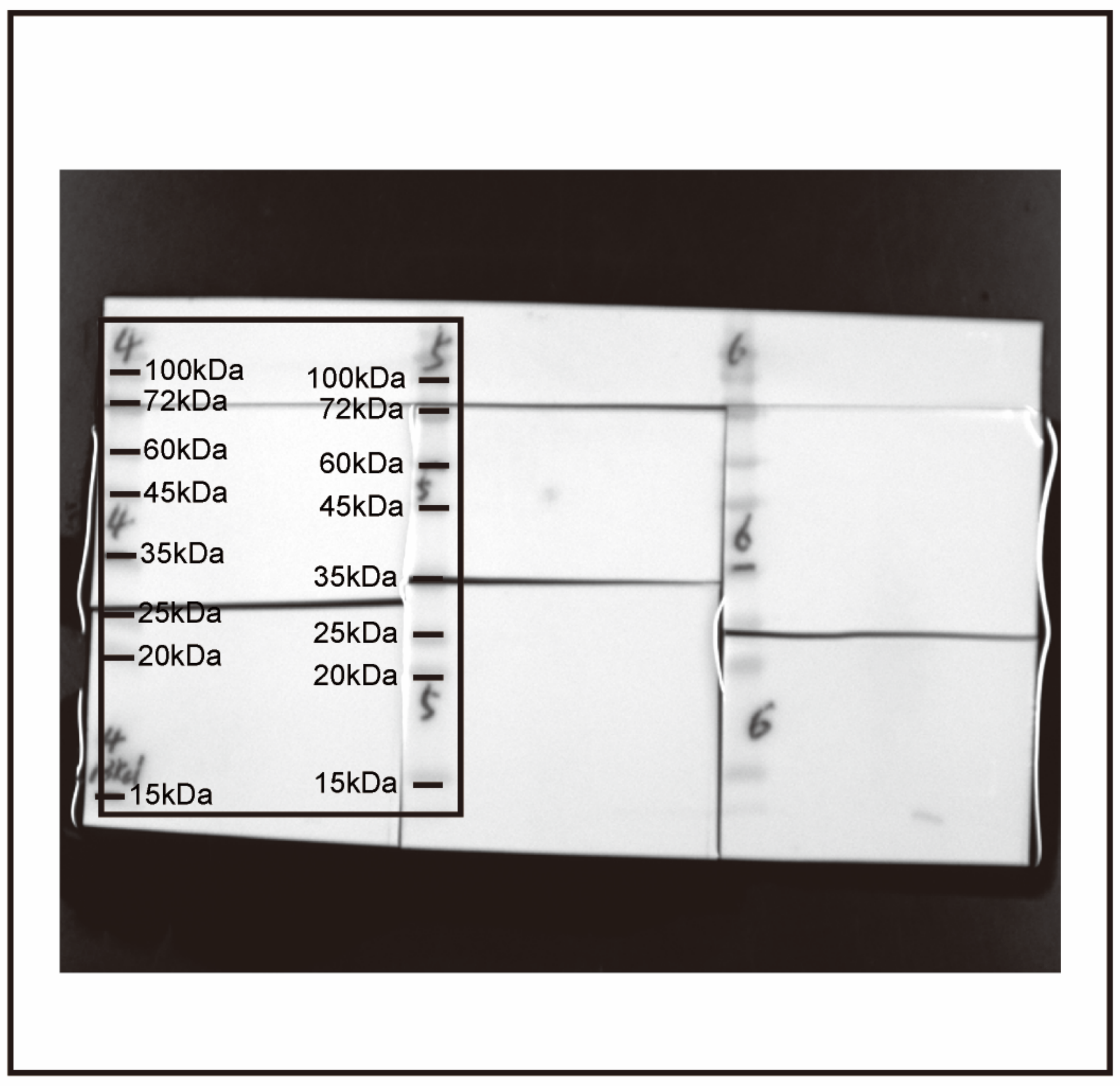

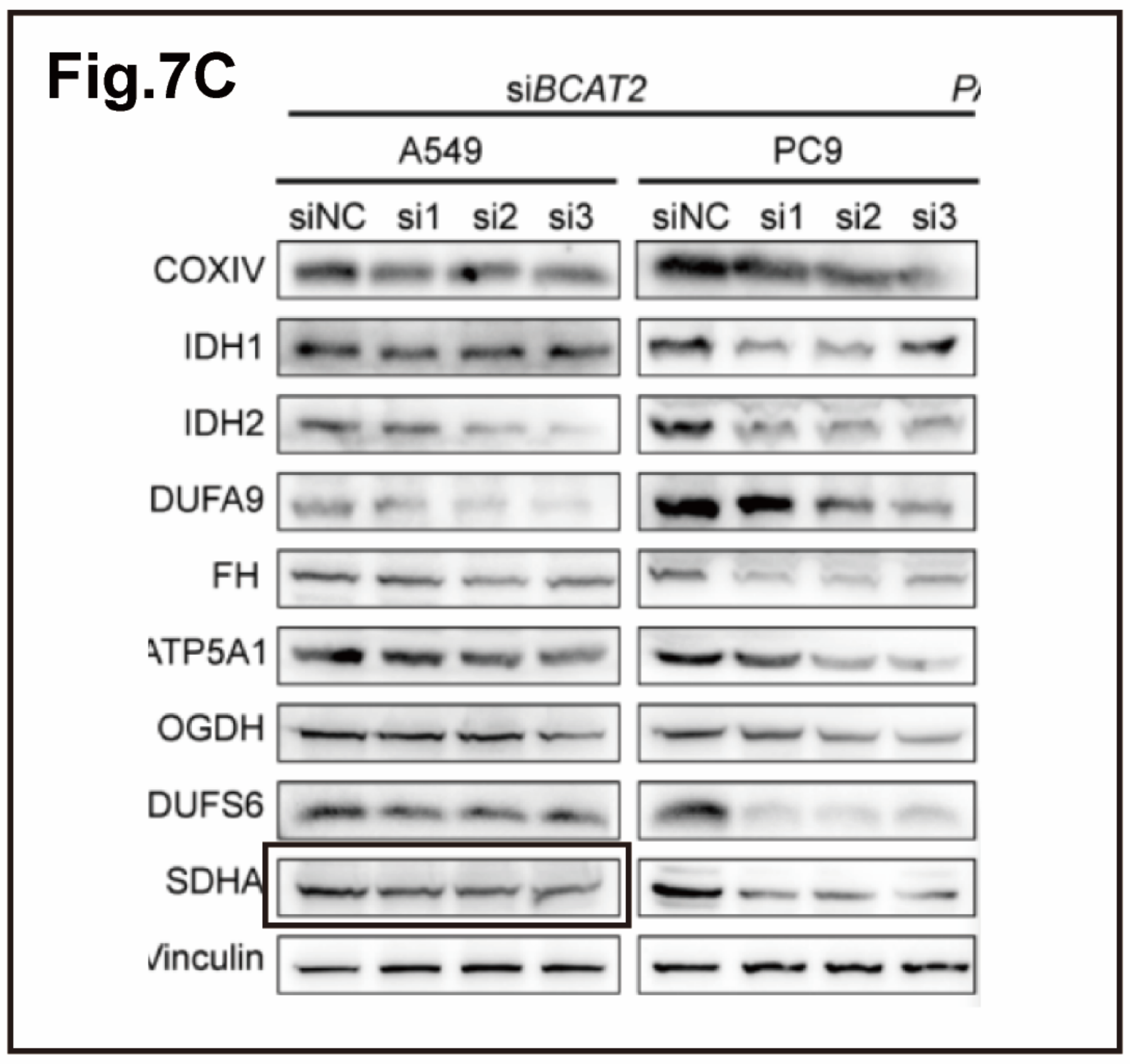

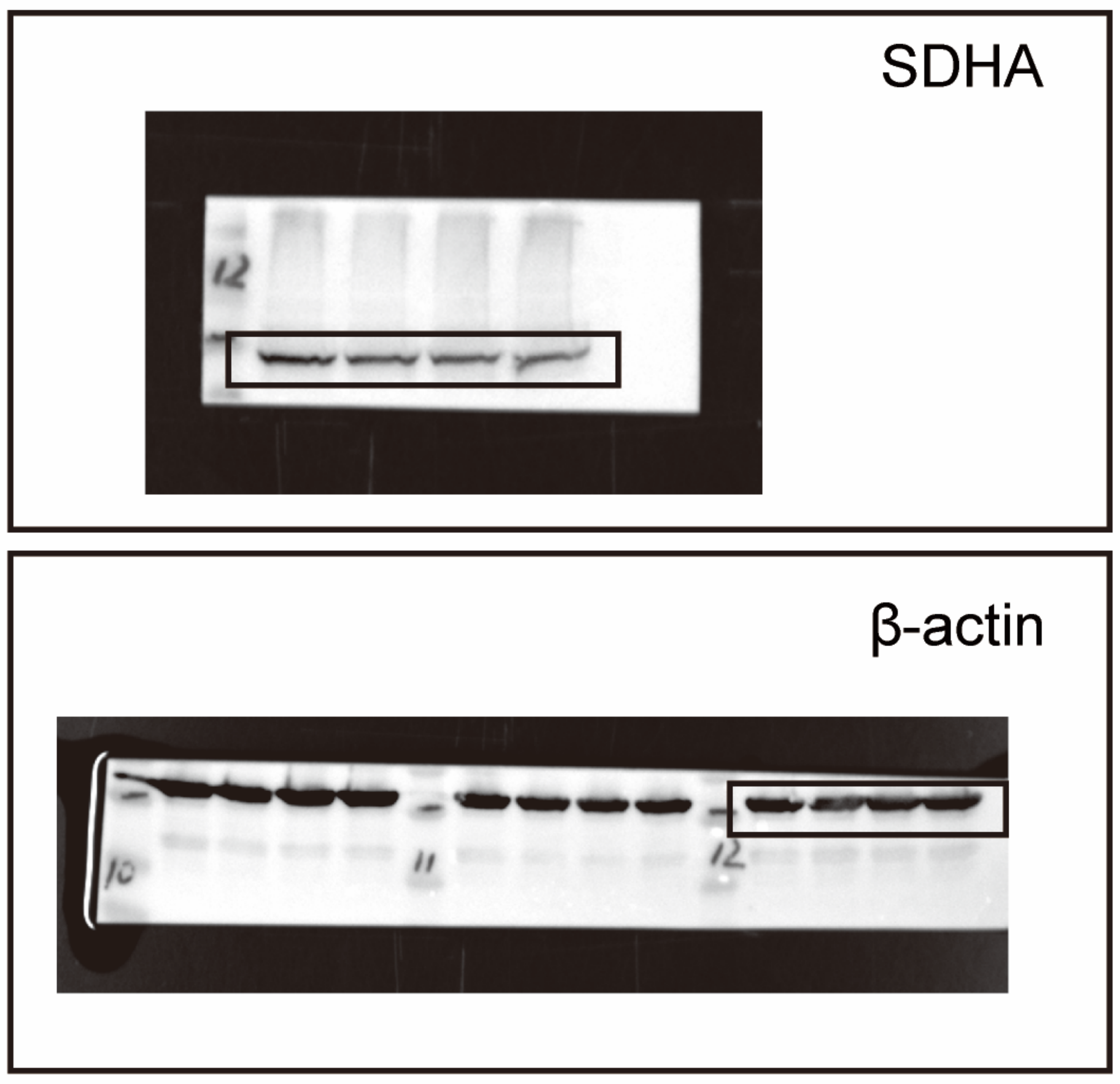

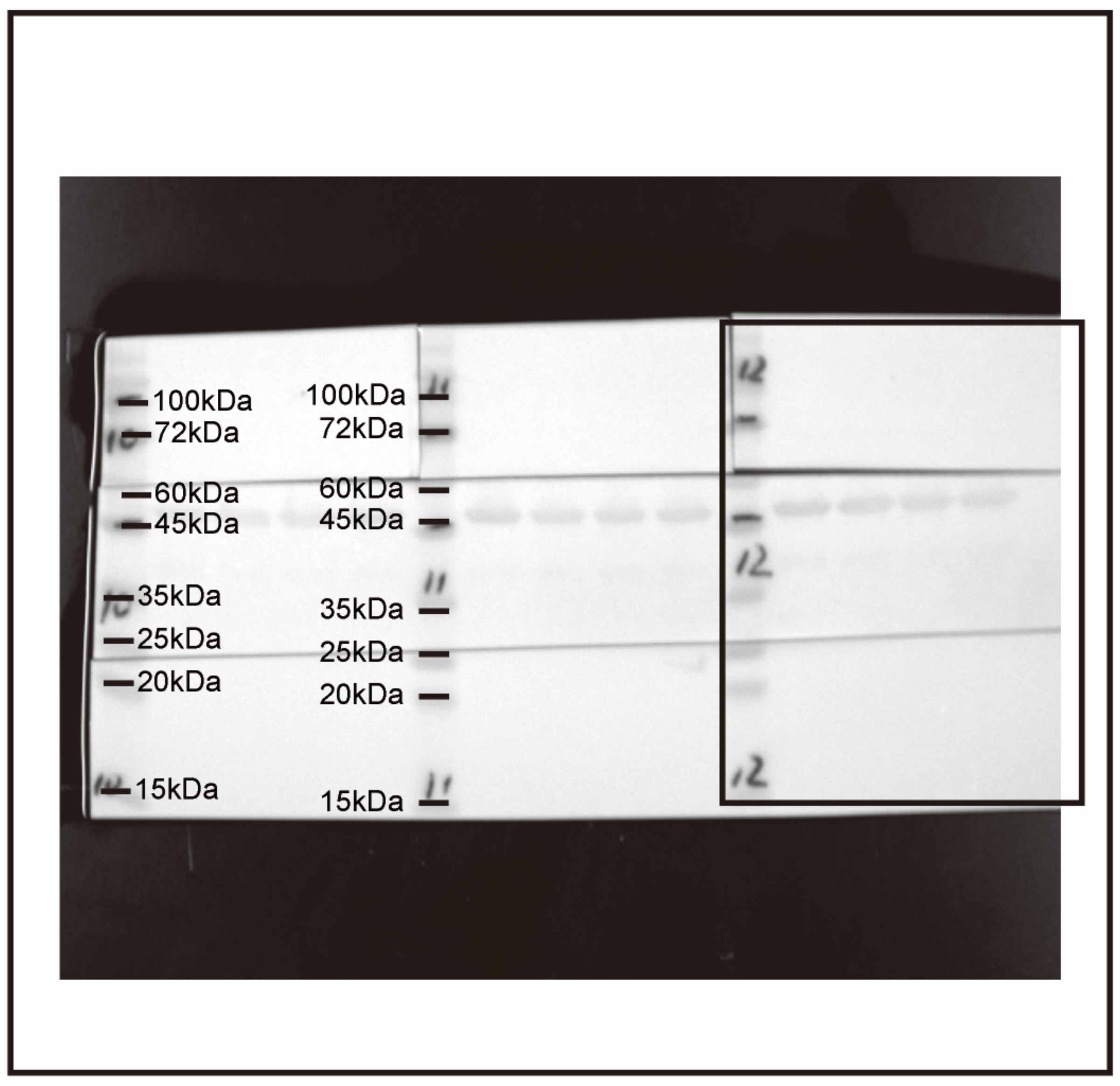

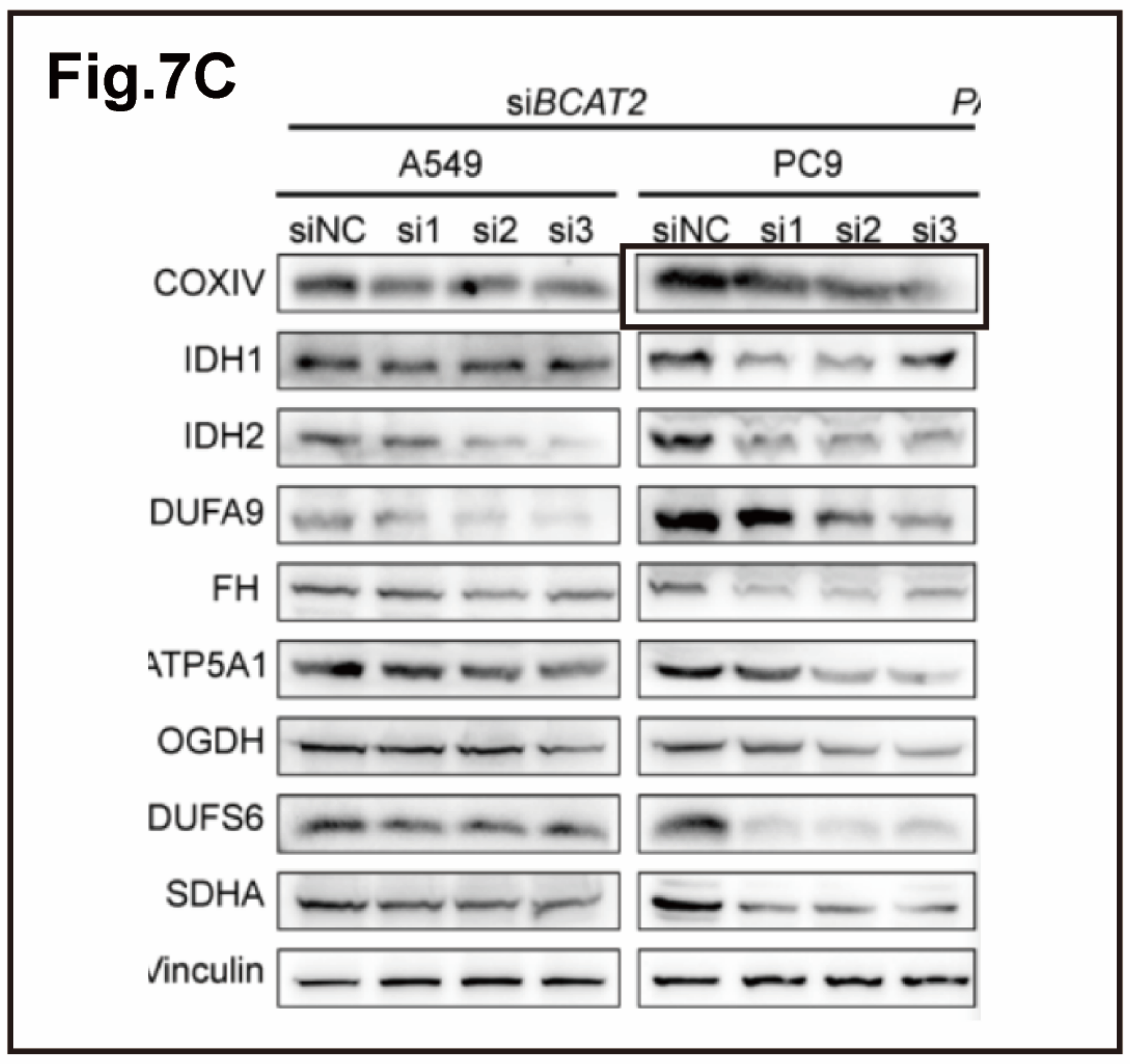

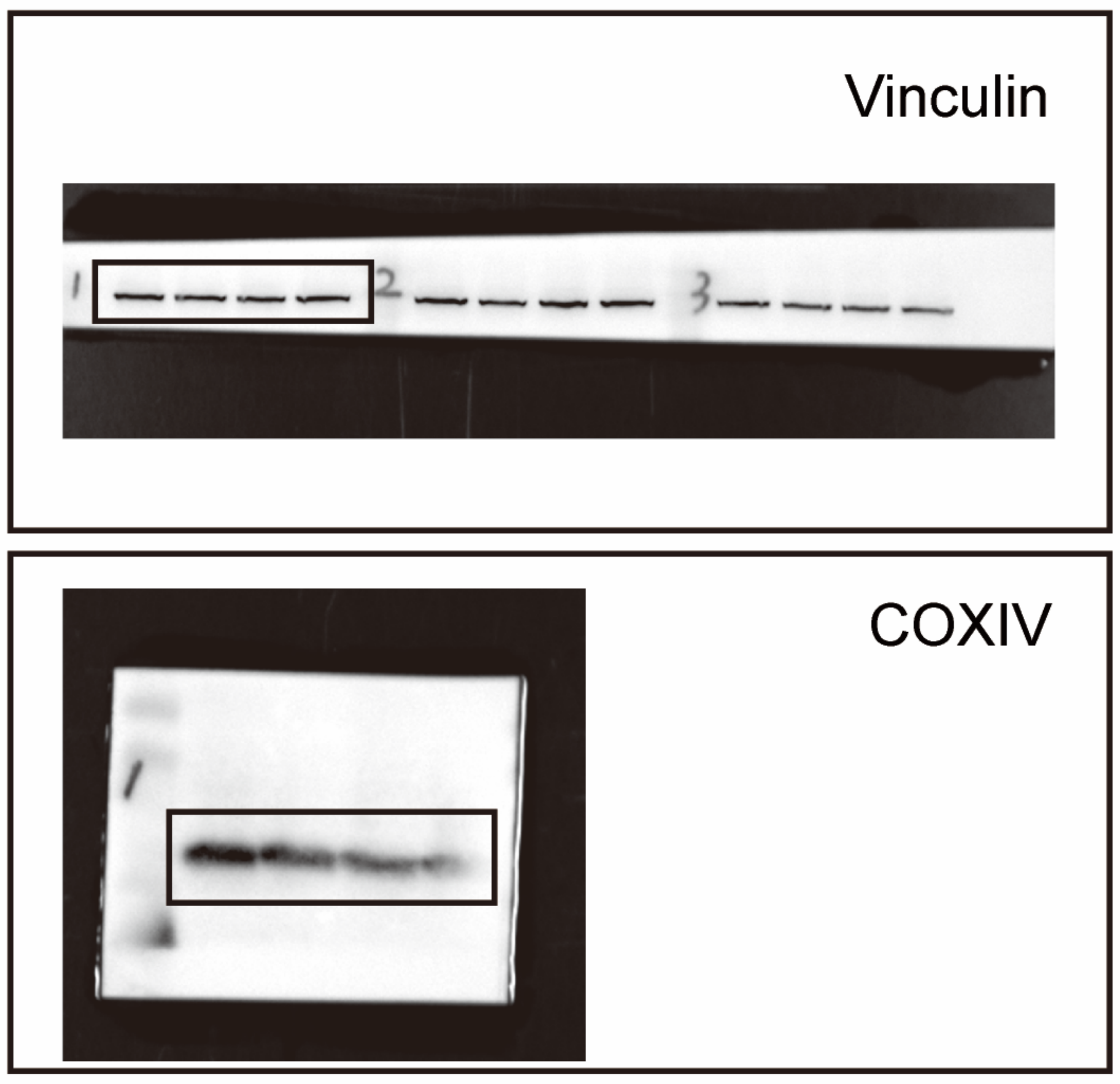

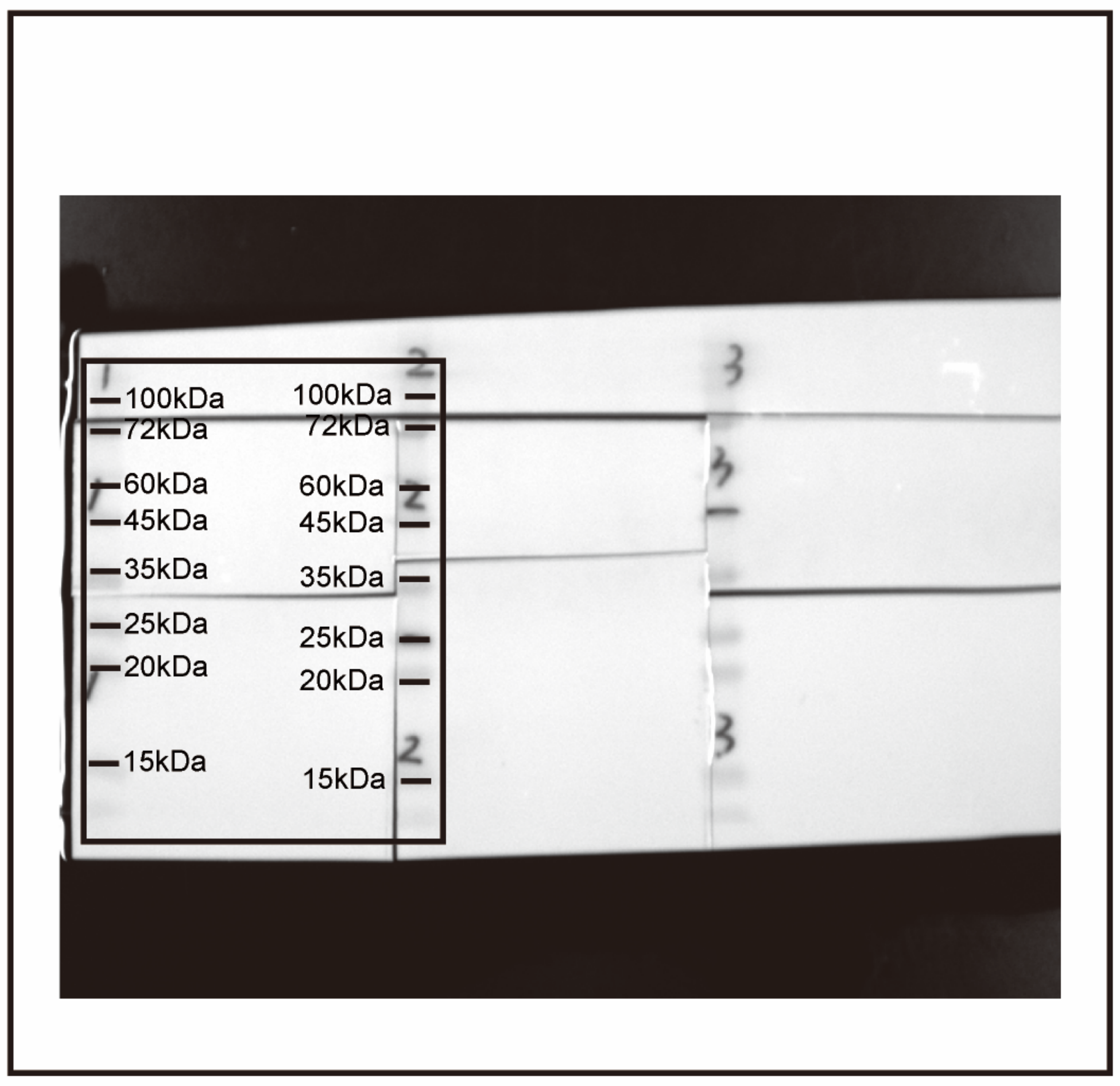

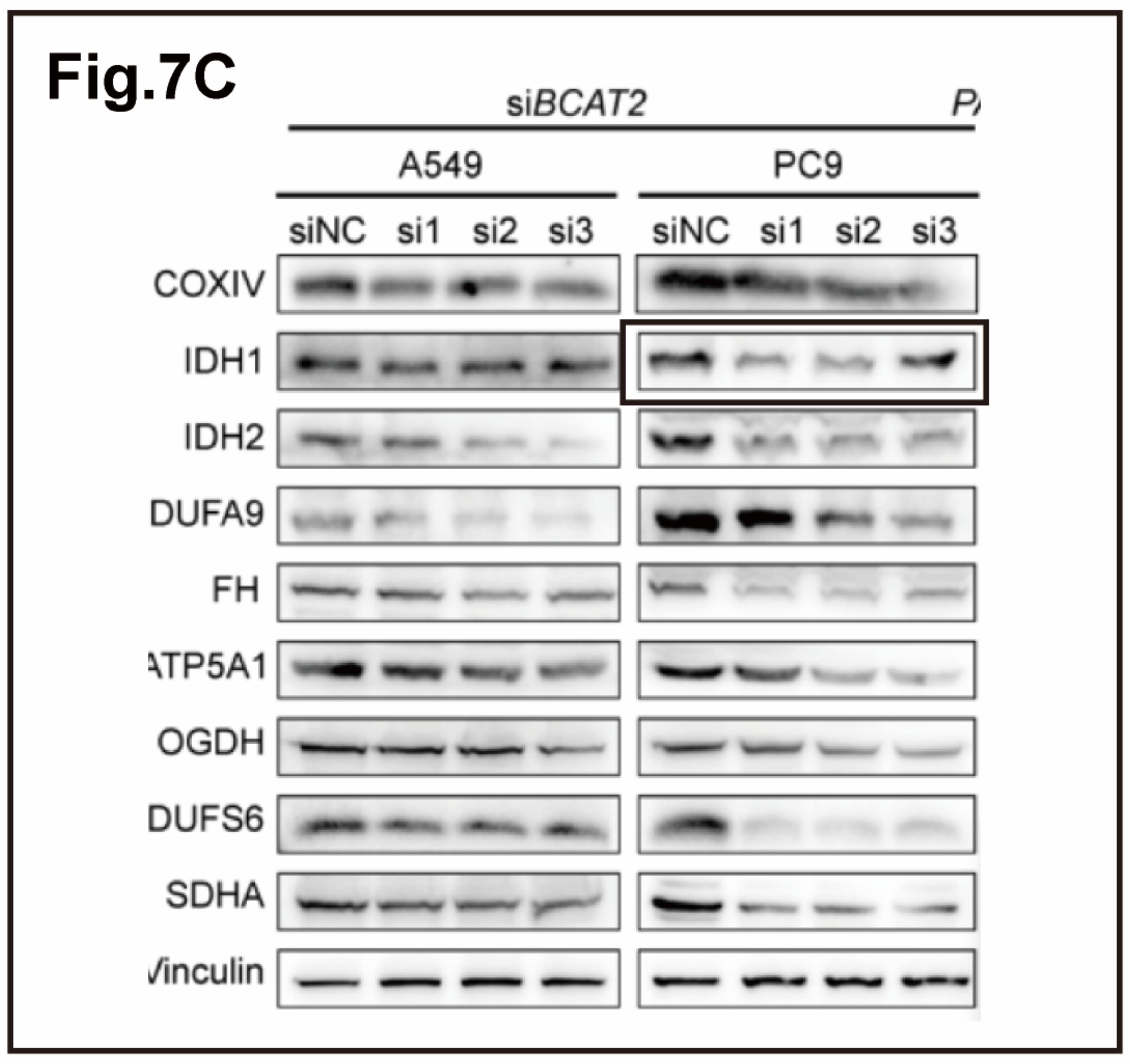

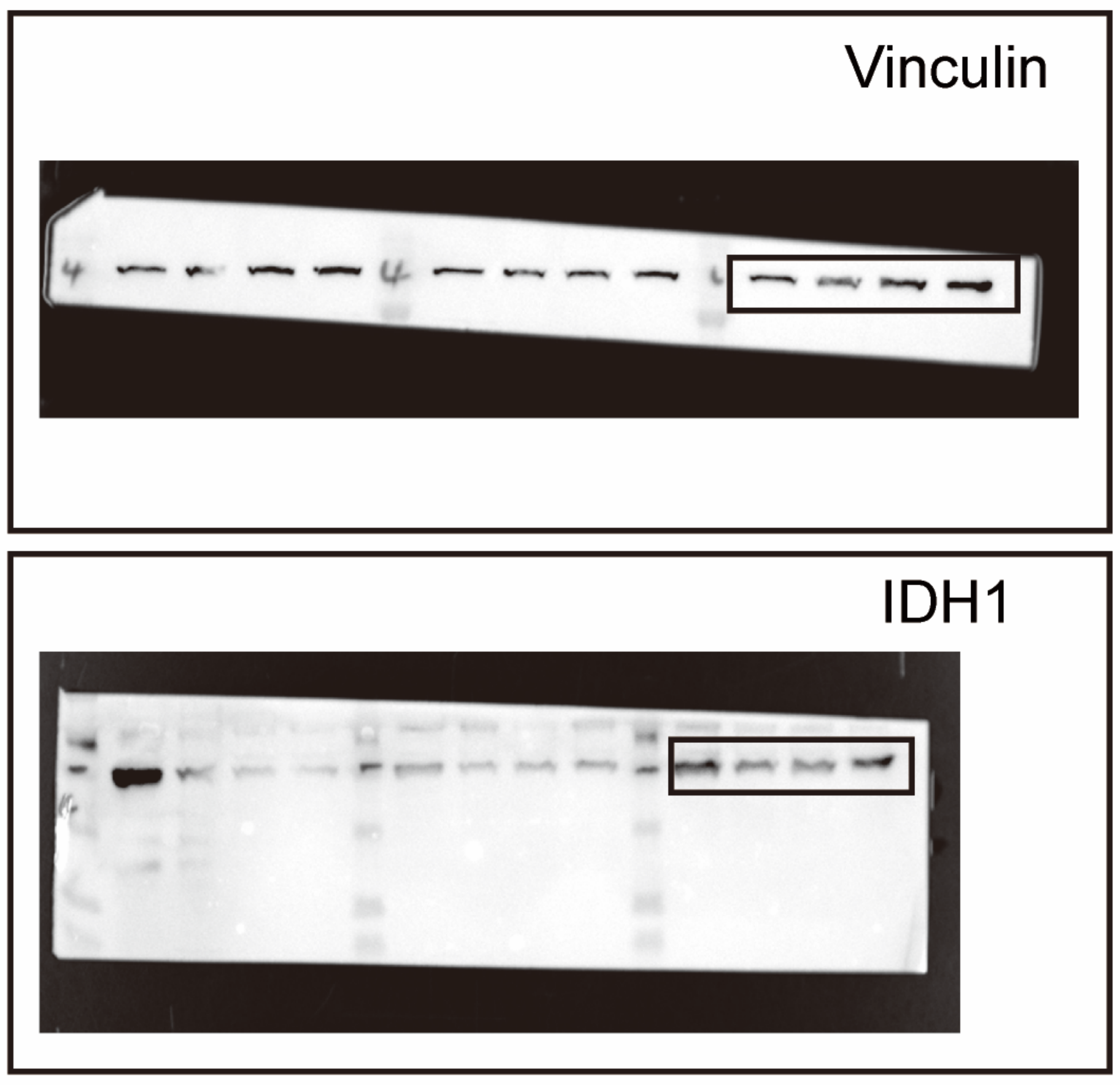

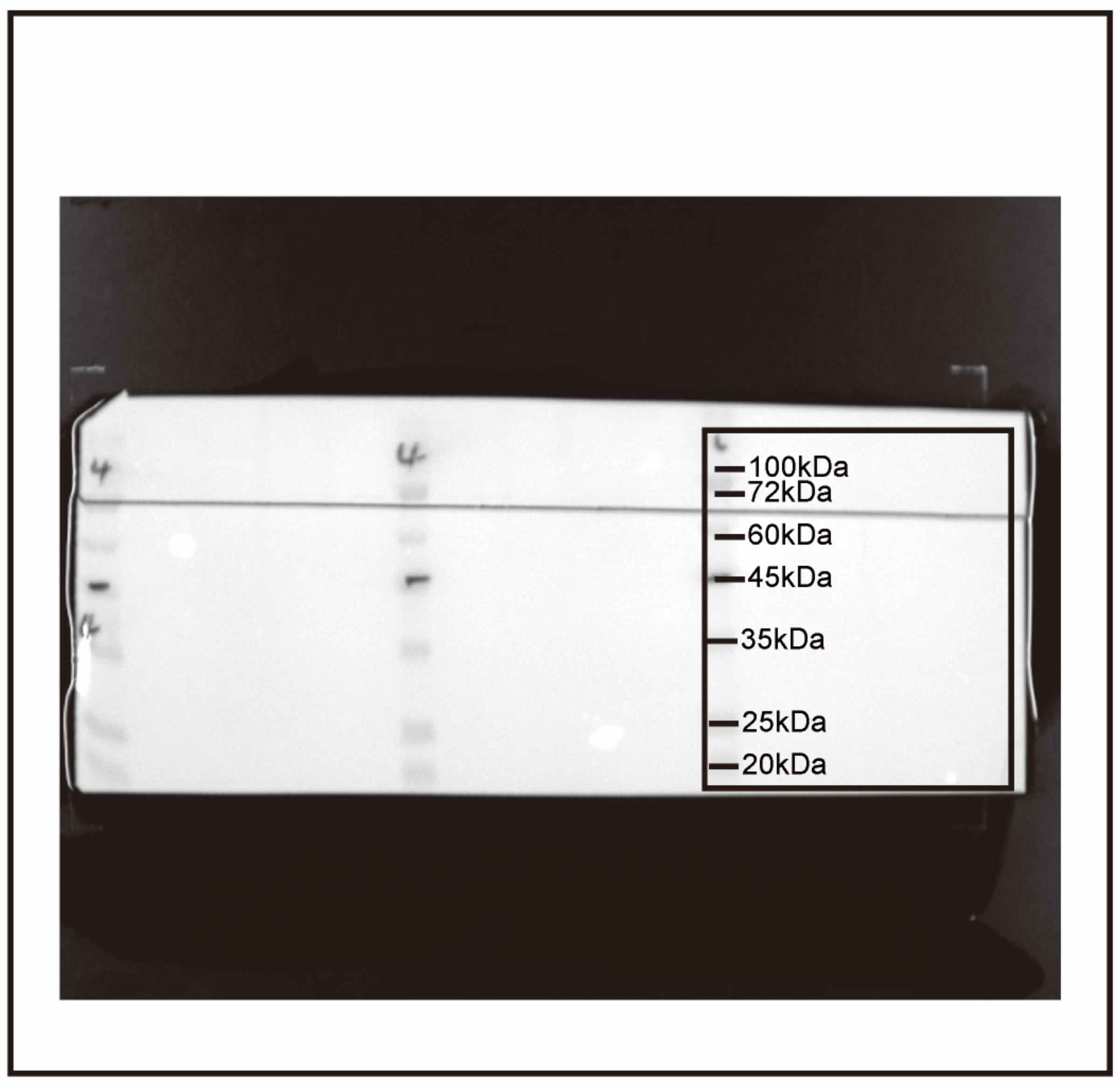

Figure	Merged	Stiched full image

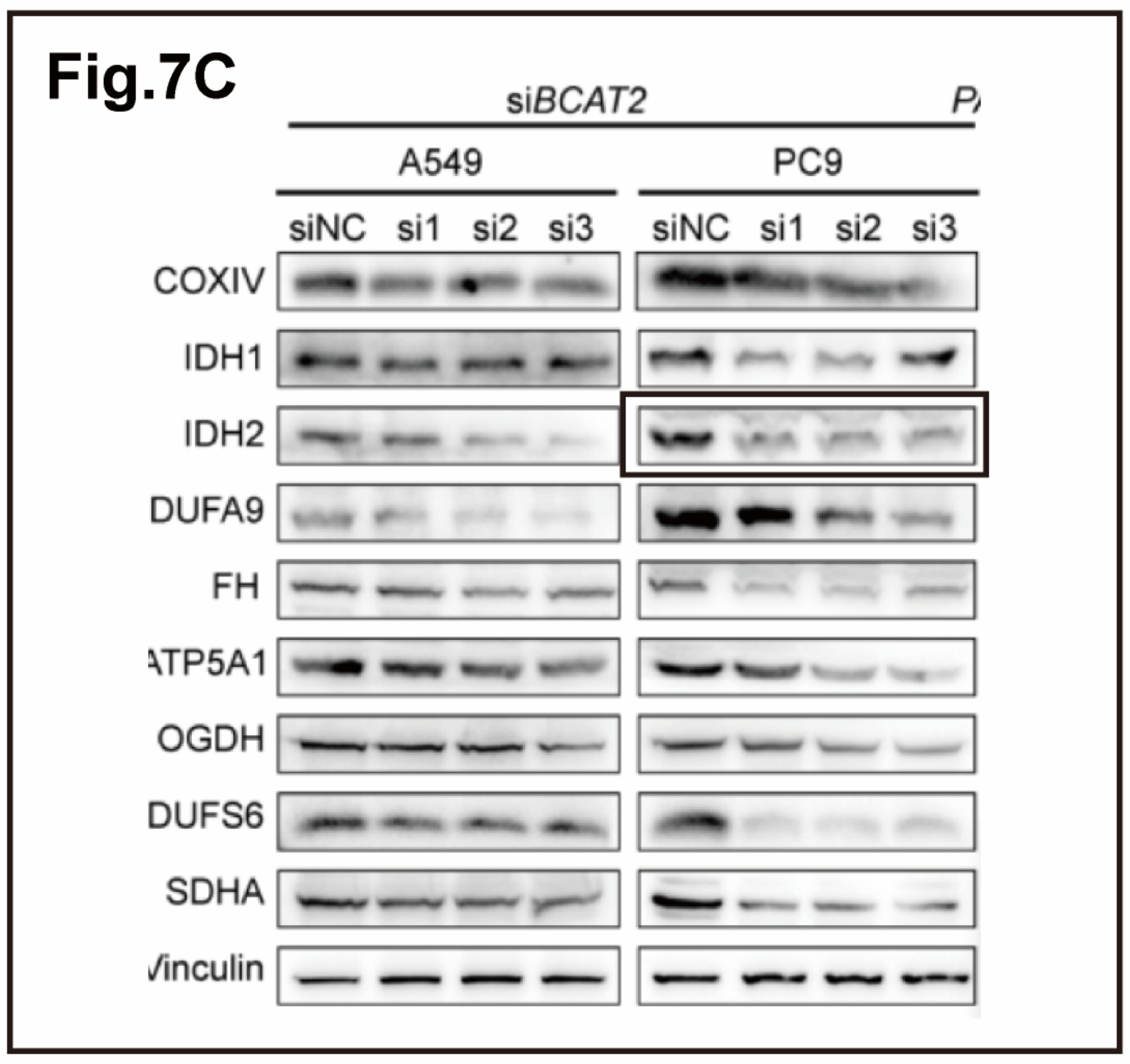

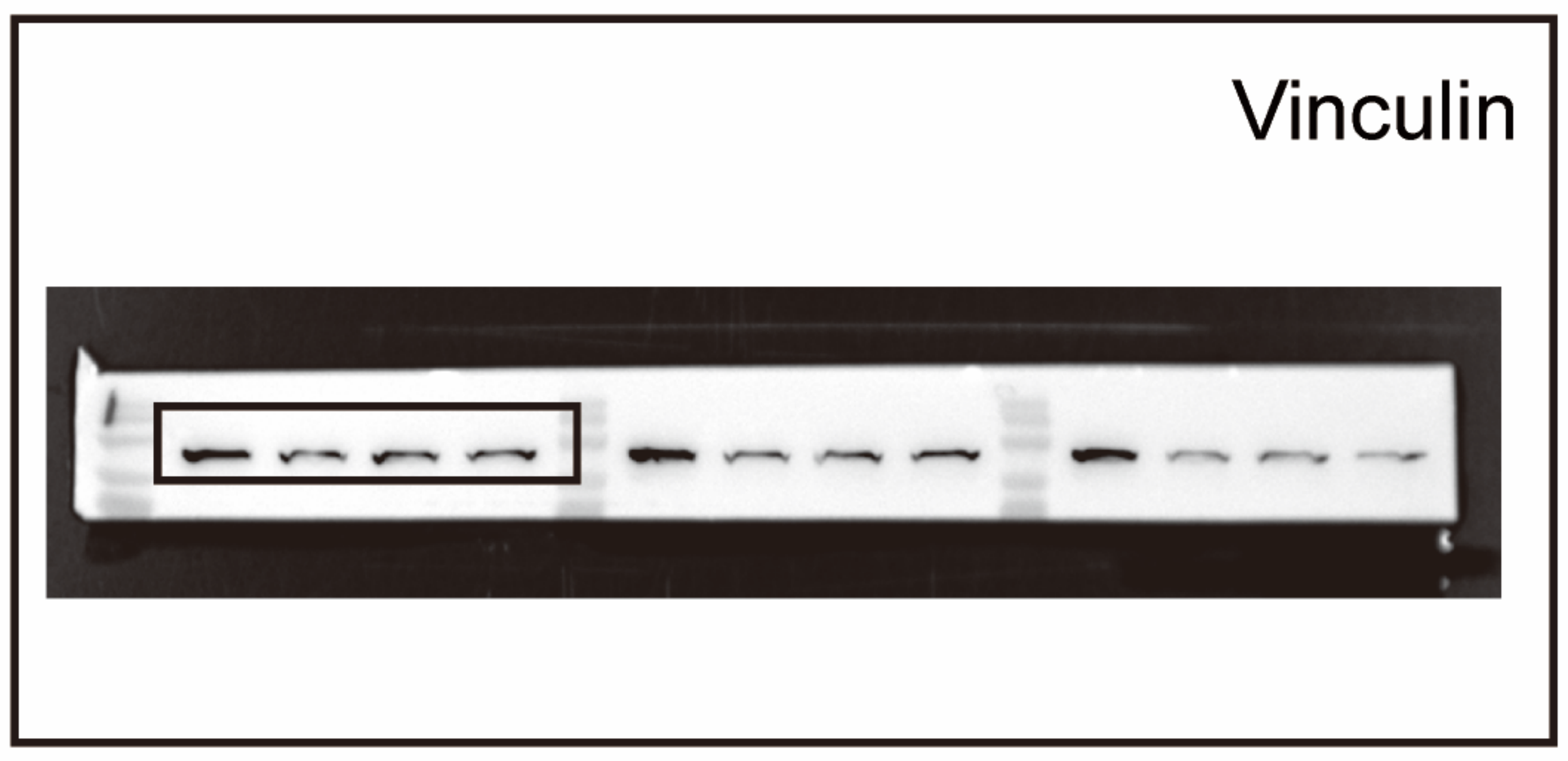

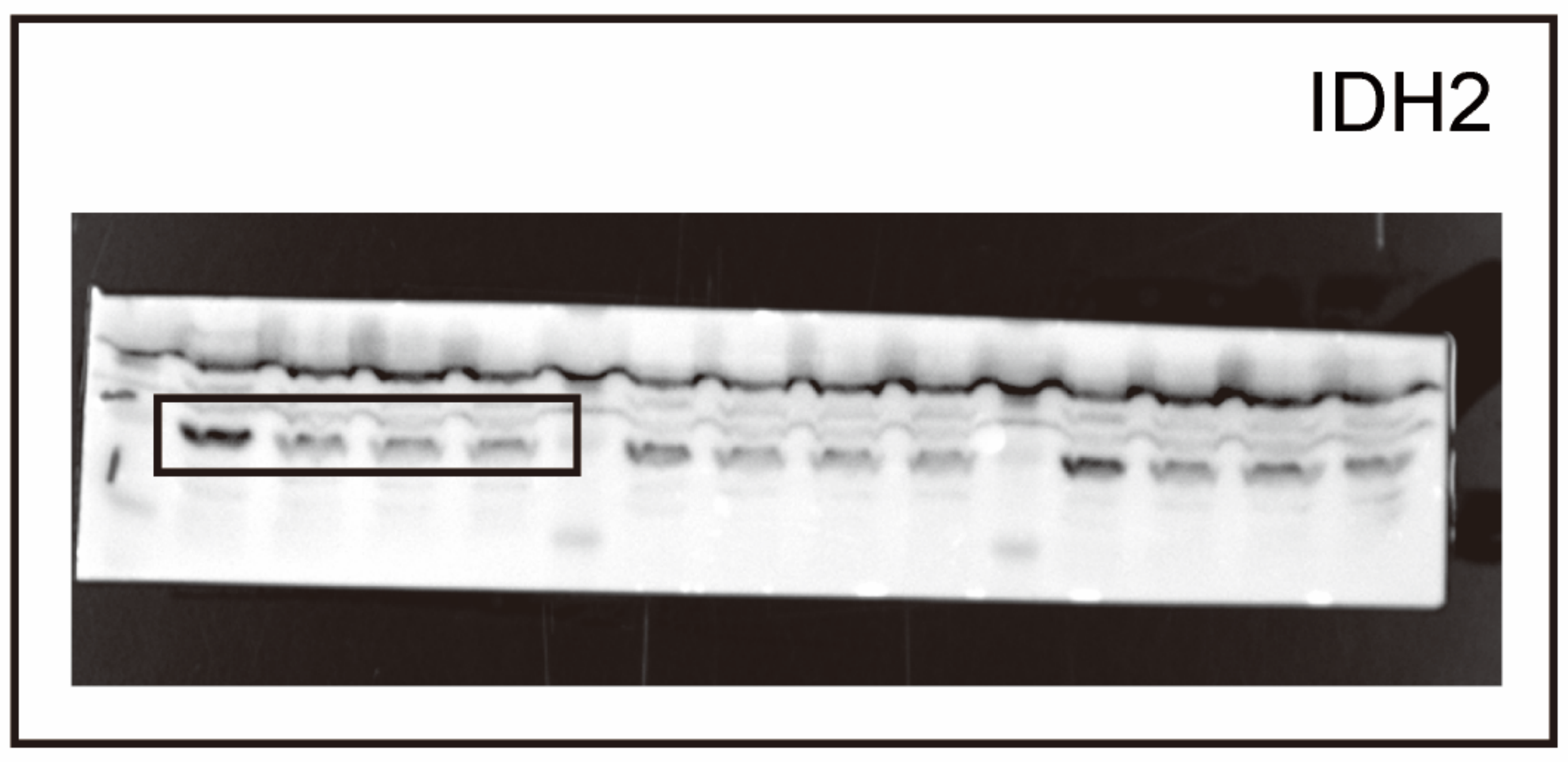

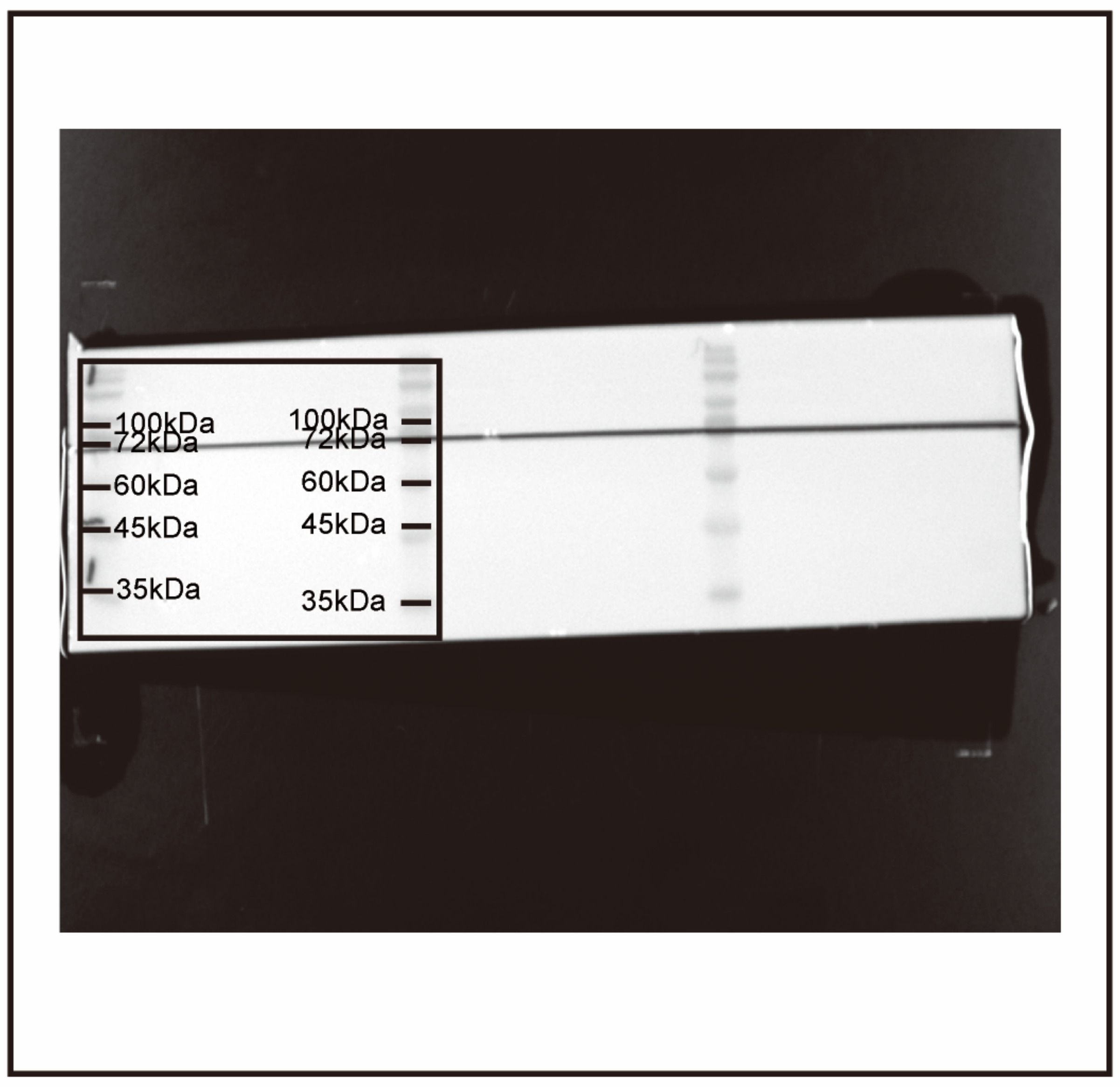

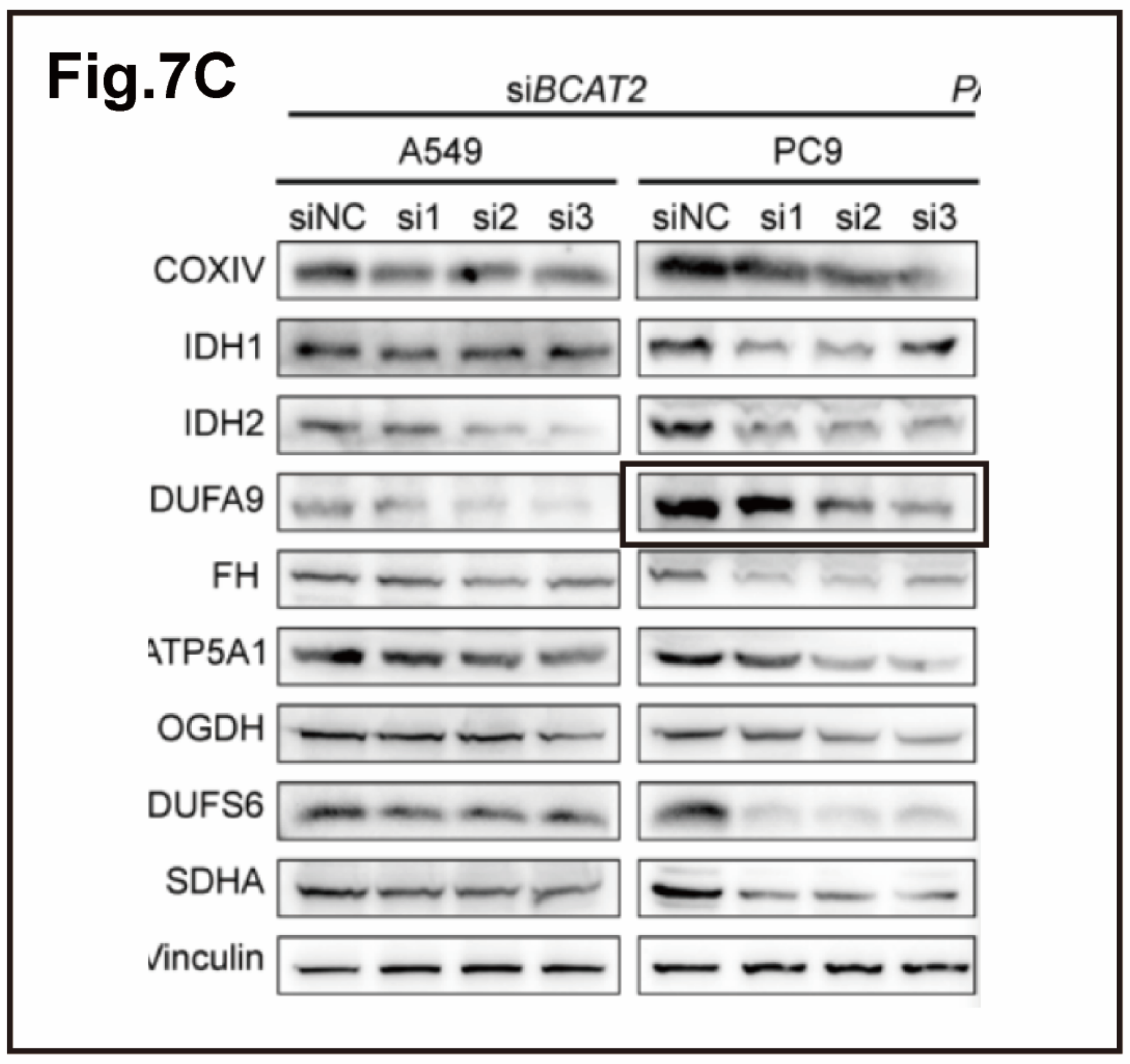

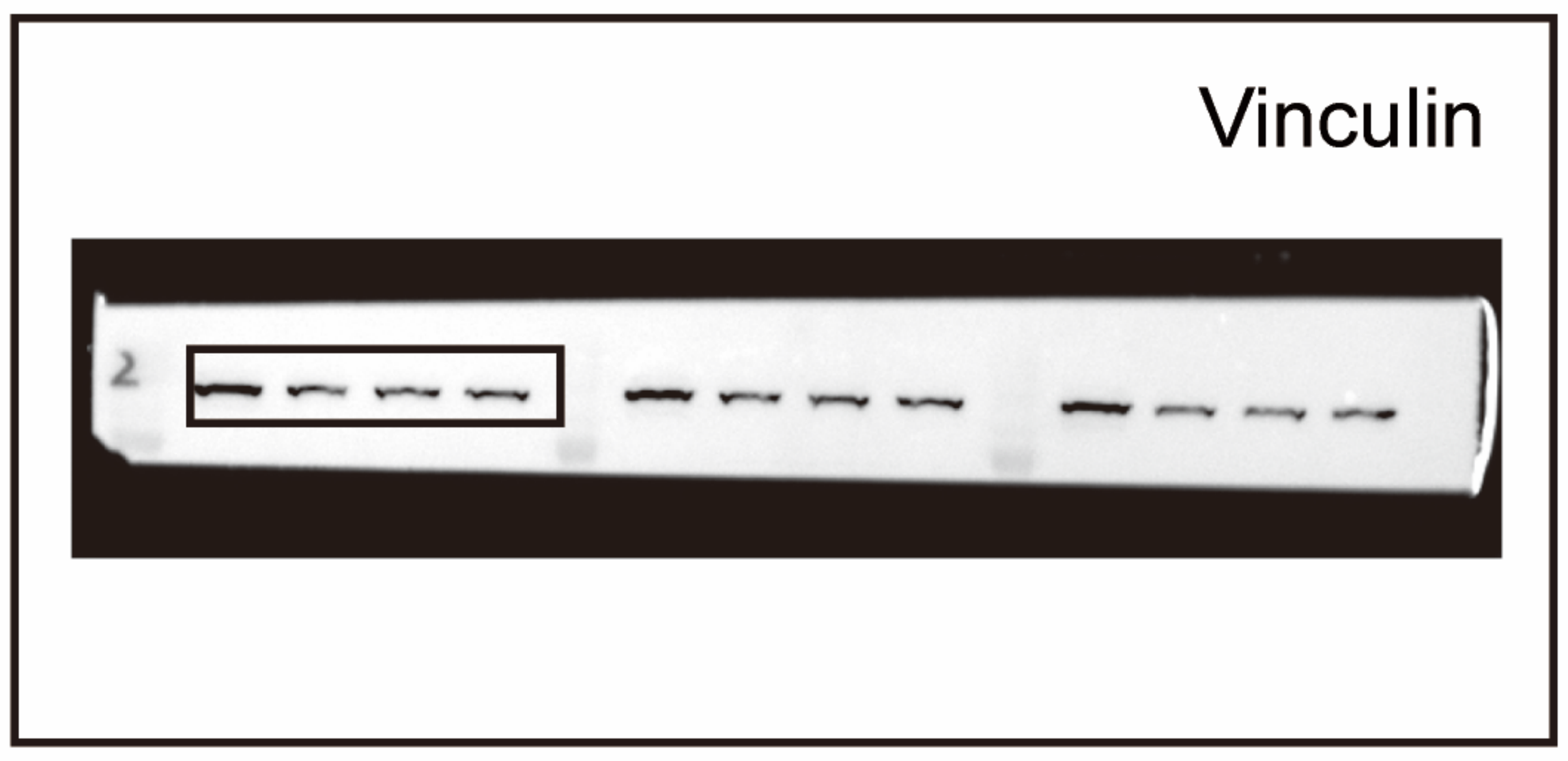

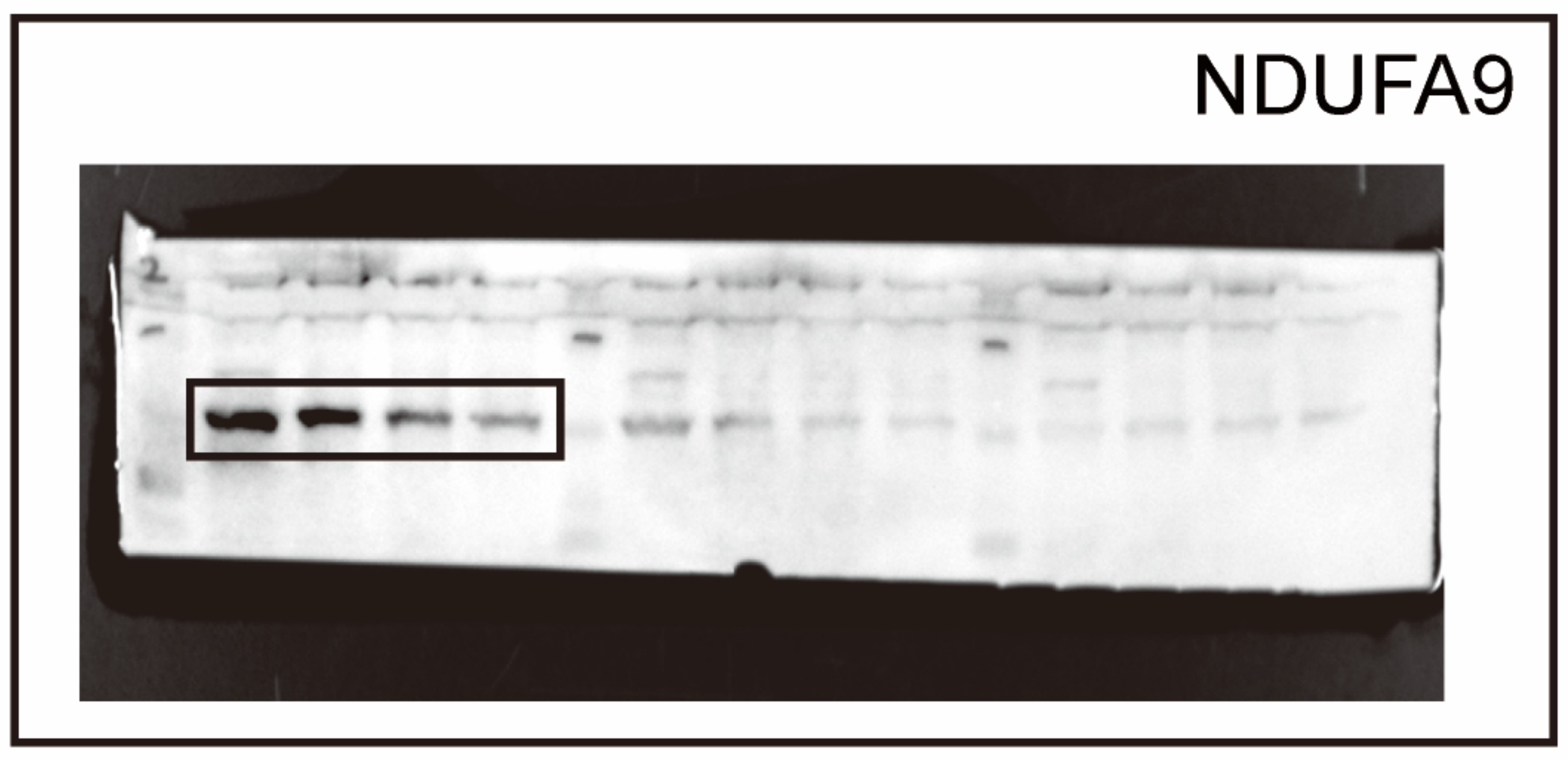

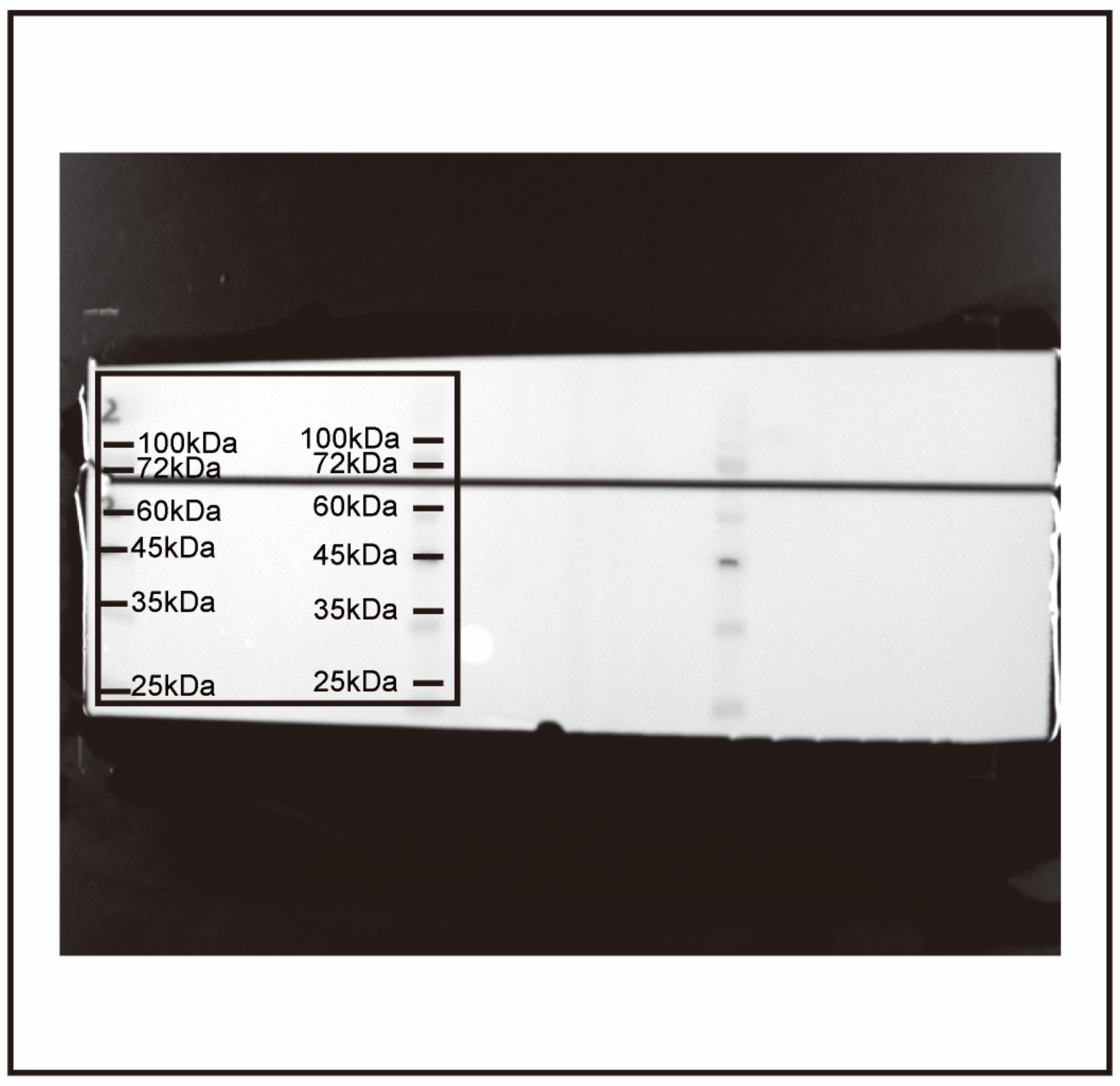

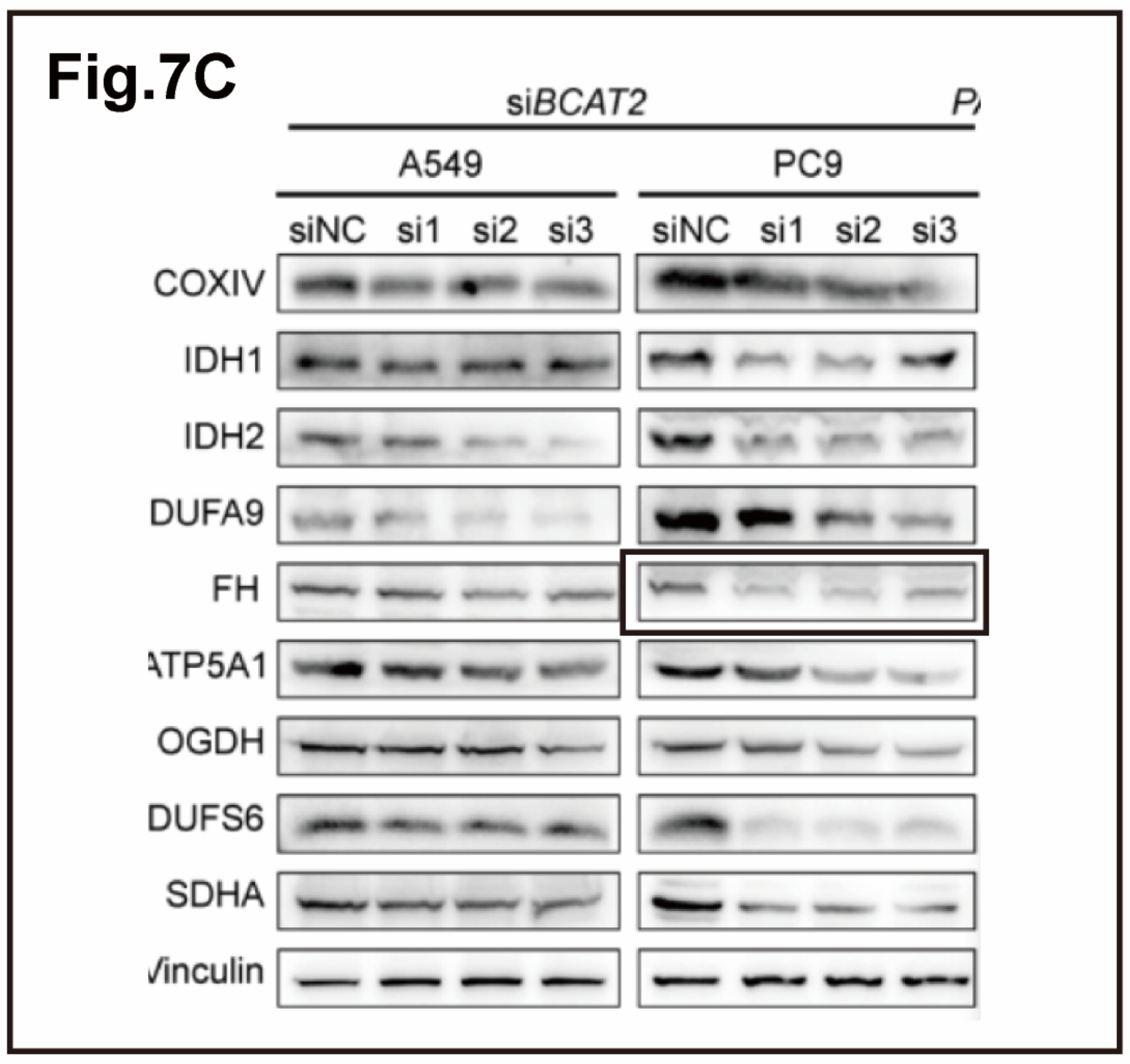

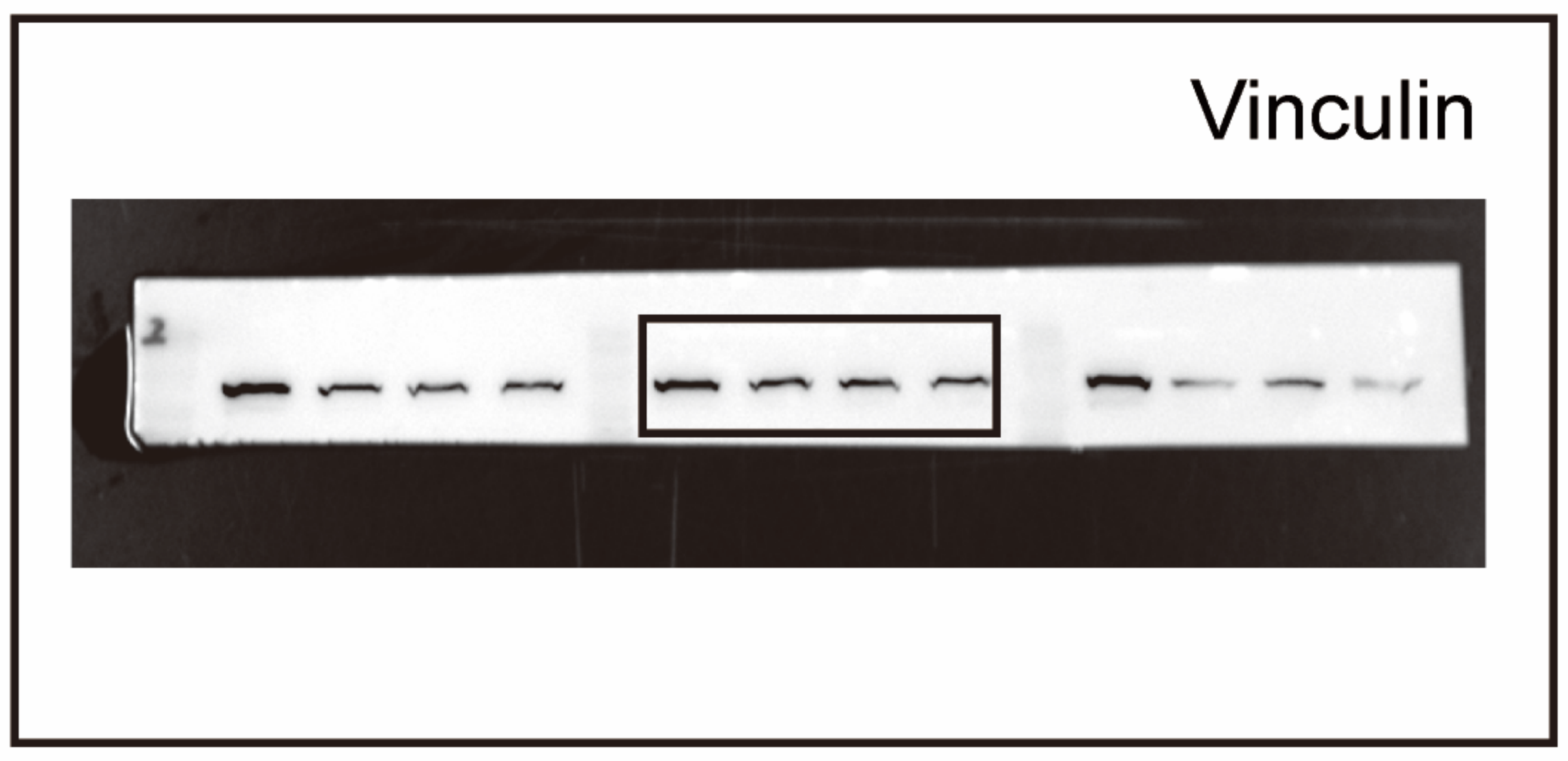

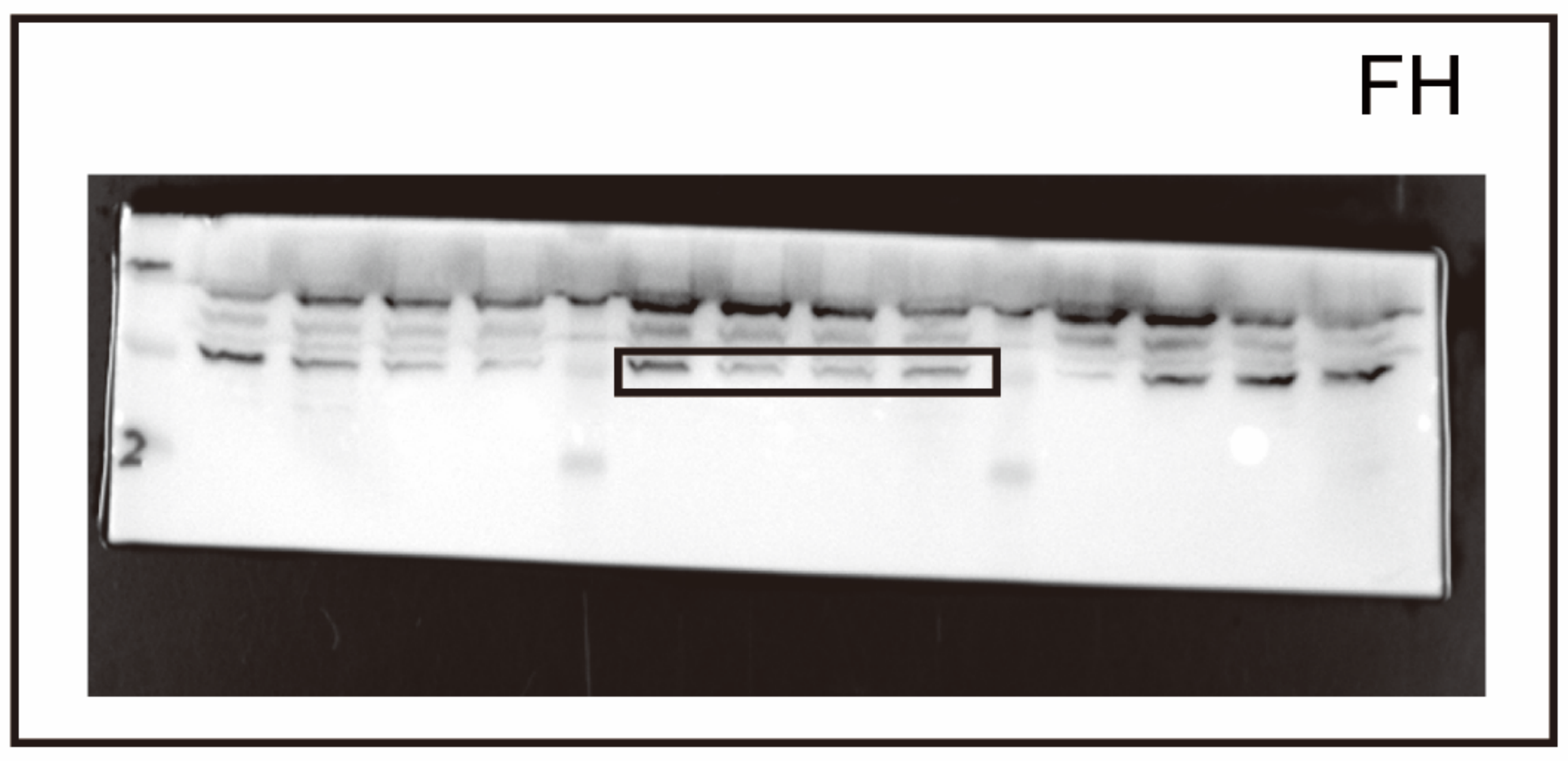

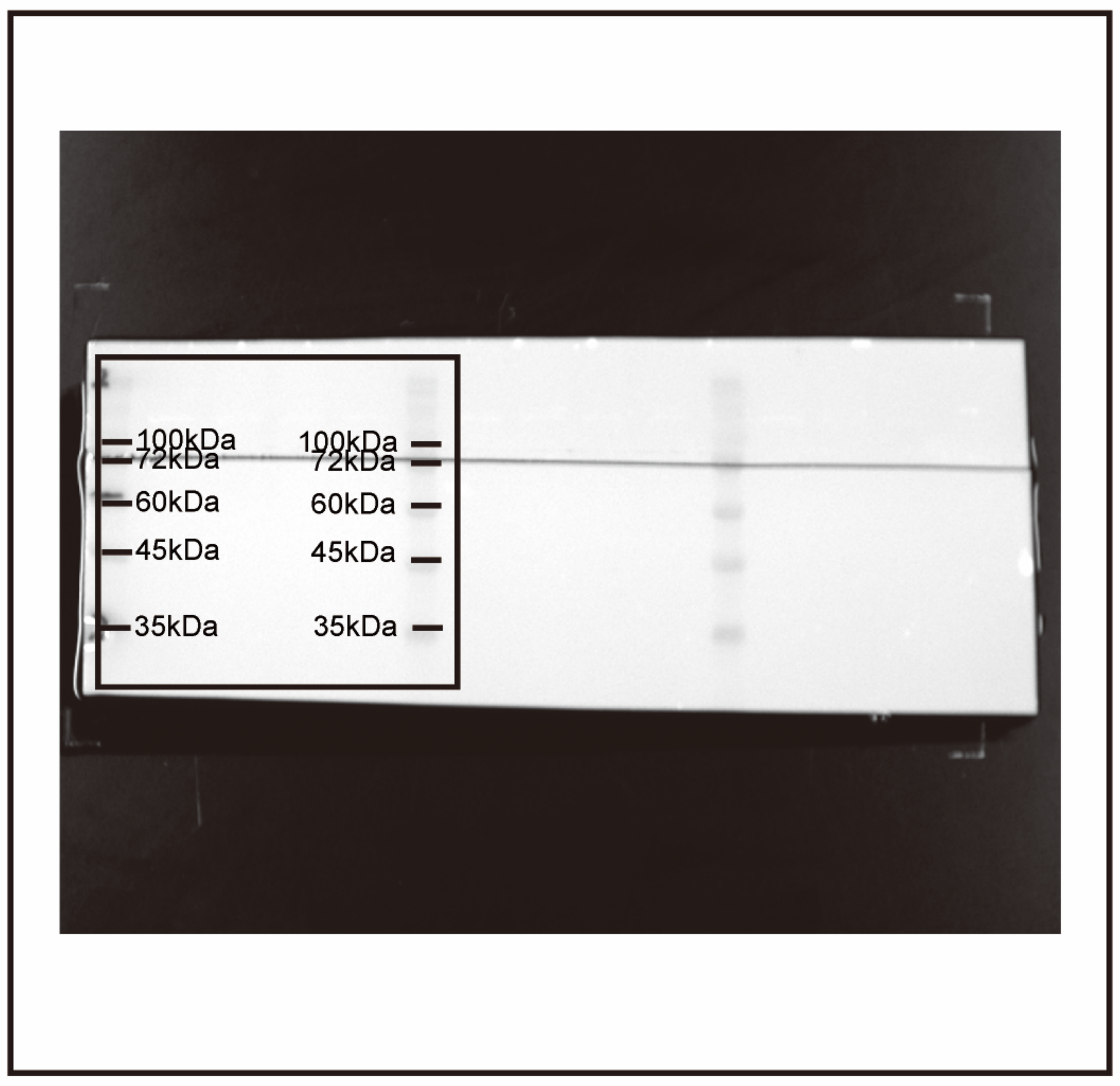

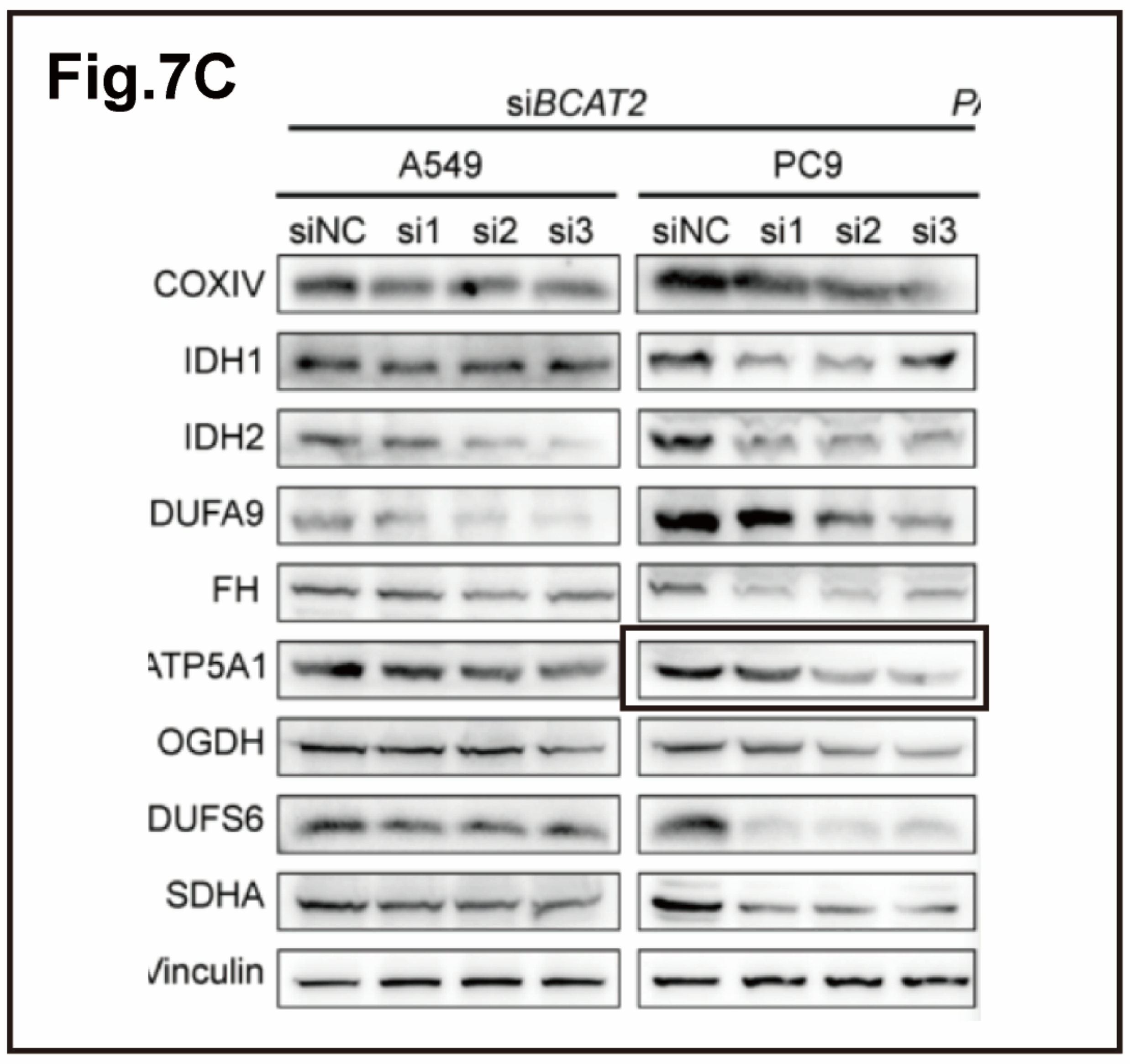

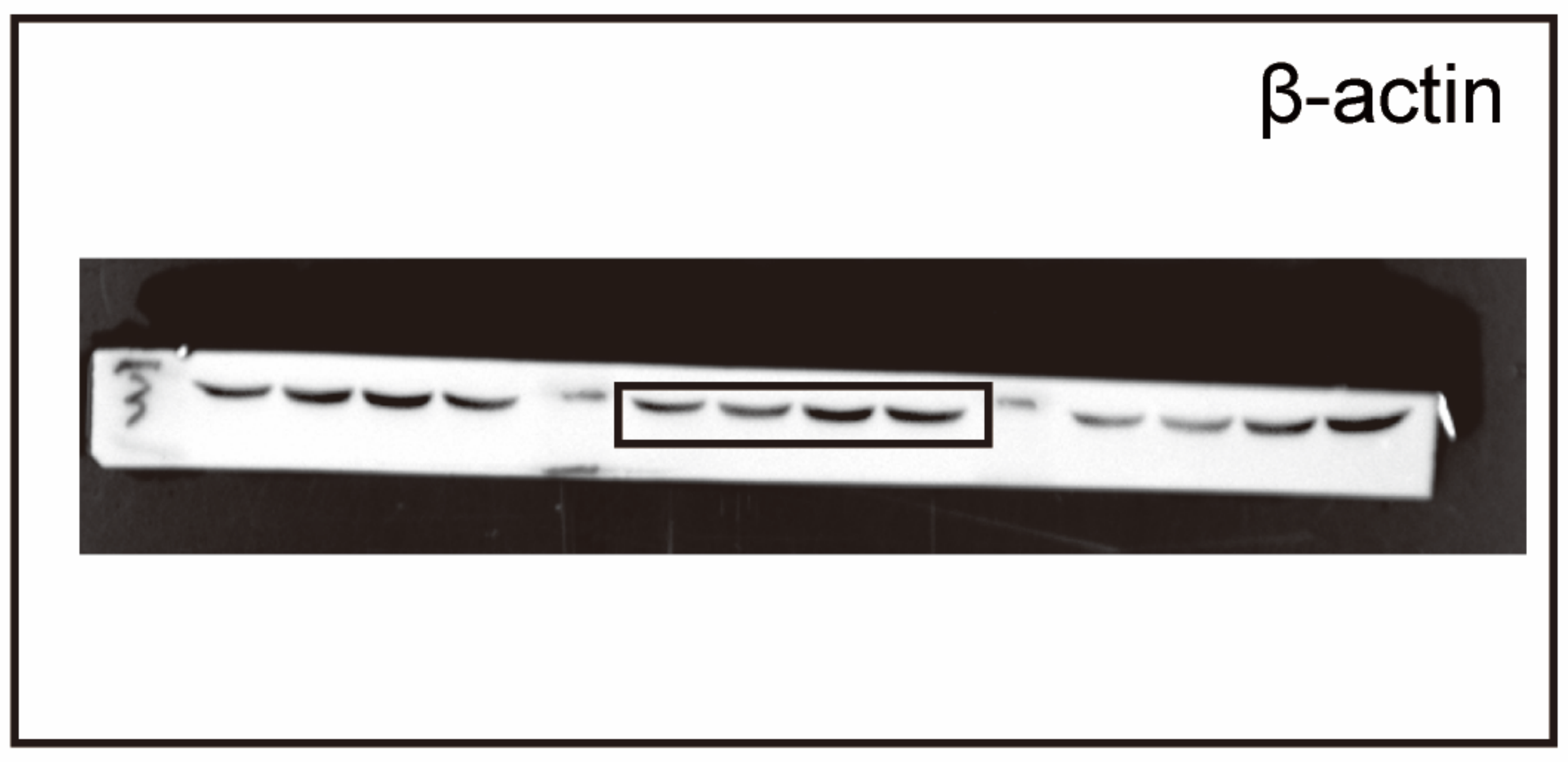

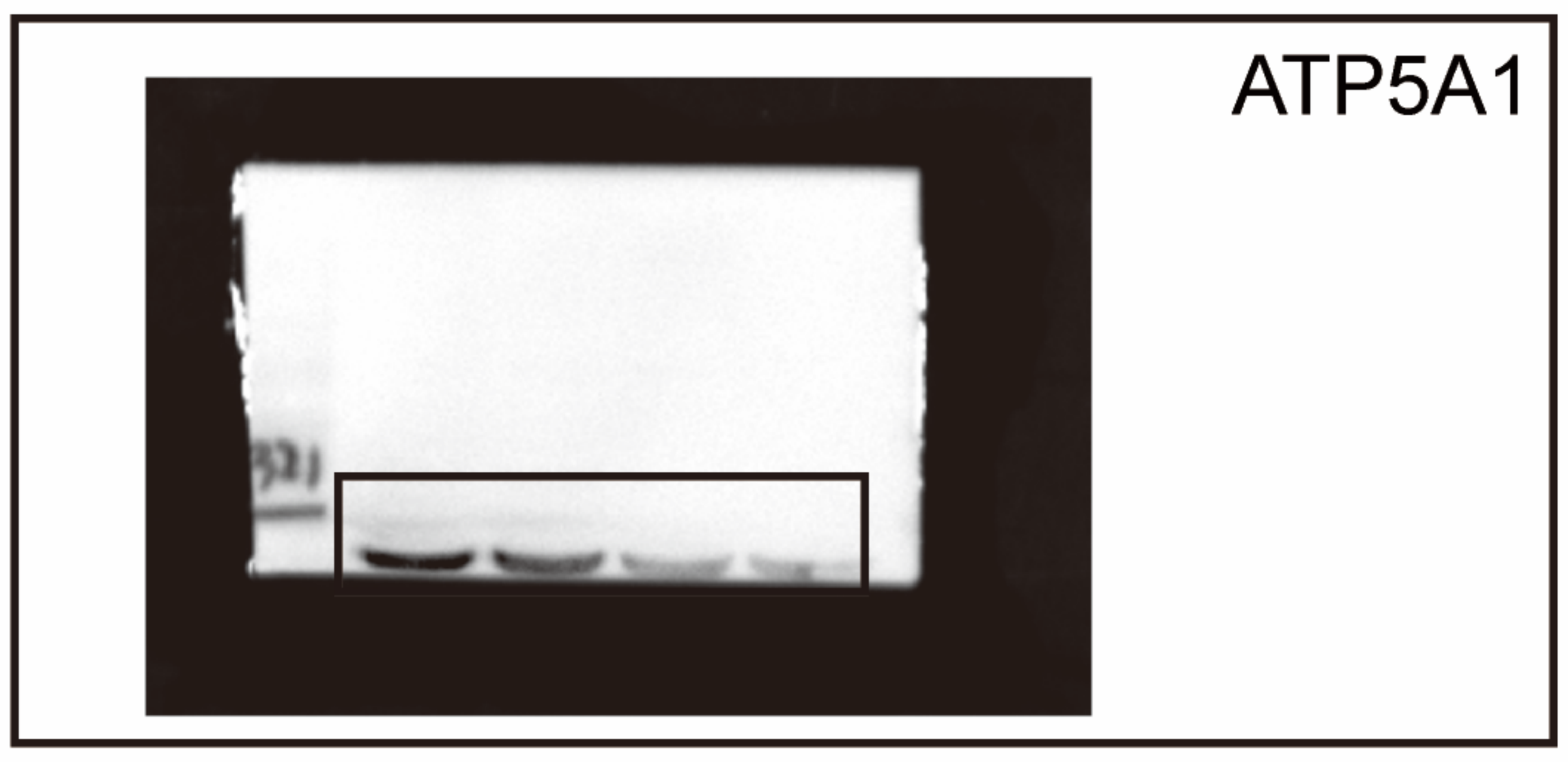

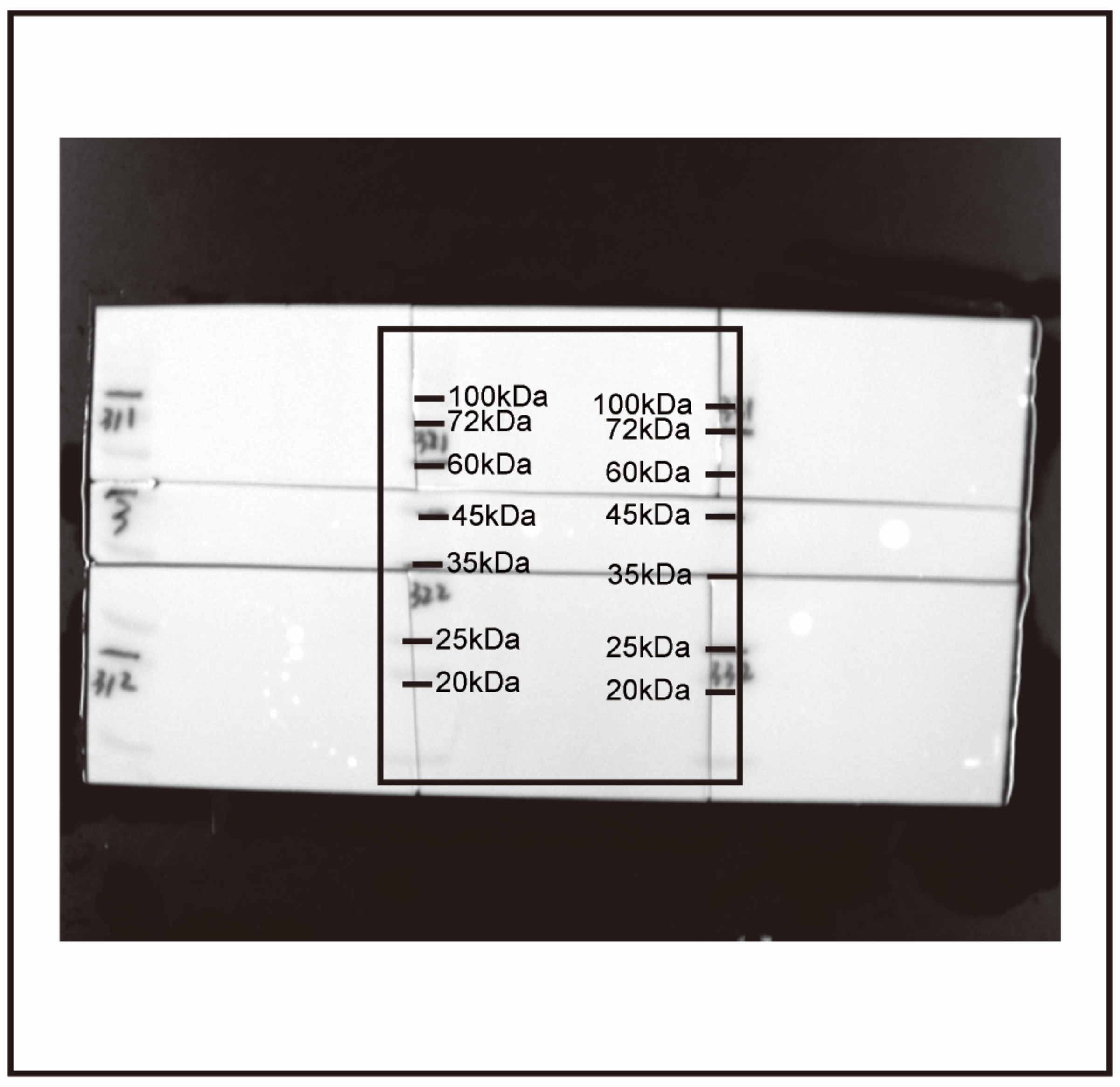

Figure	Merged	Stiched full image

Fig.7C

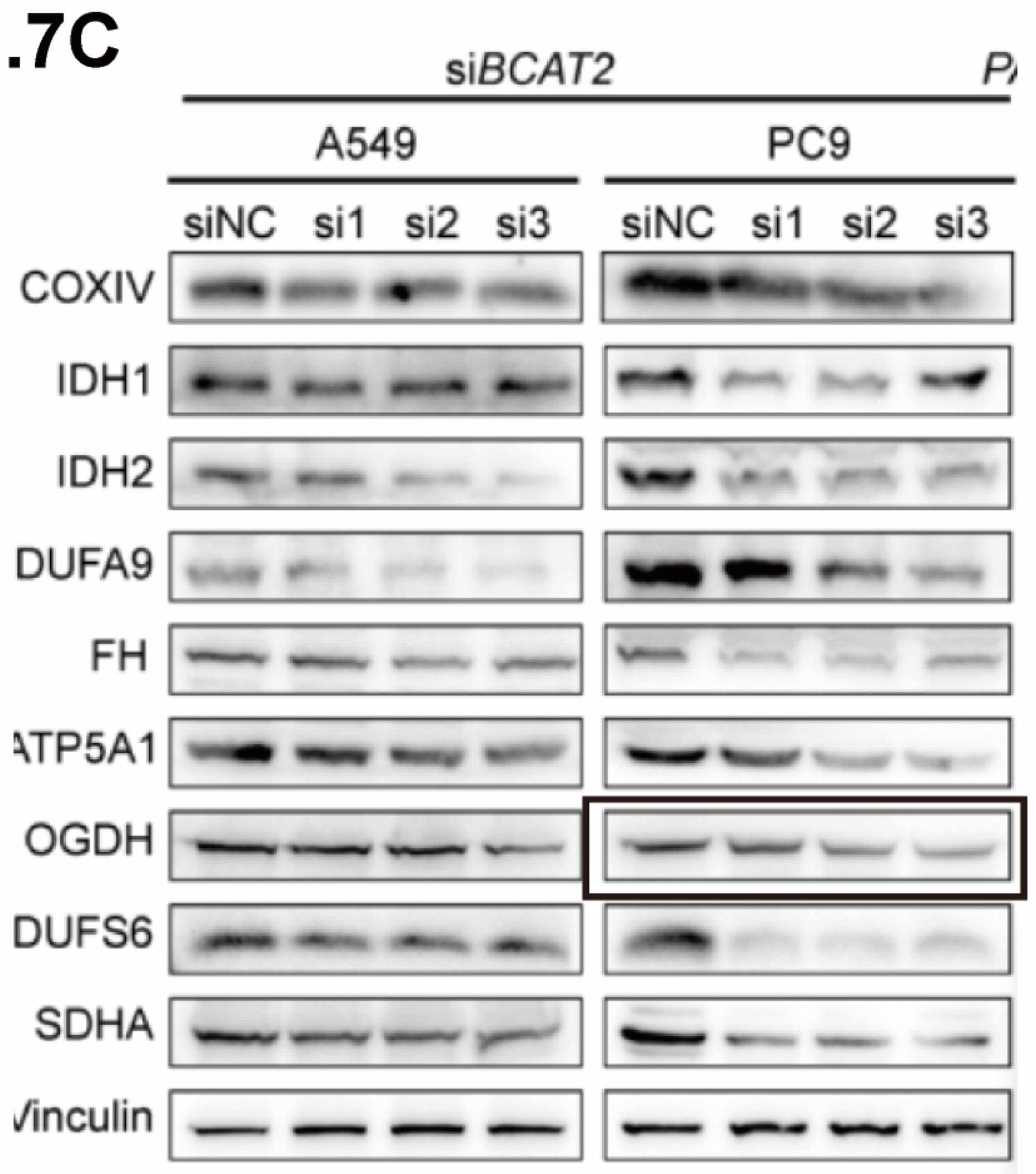

OGDH

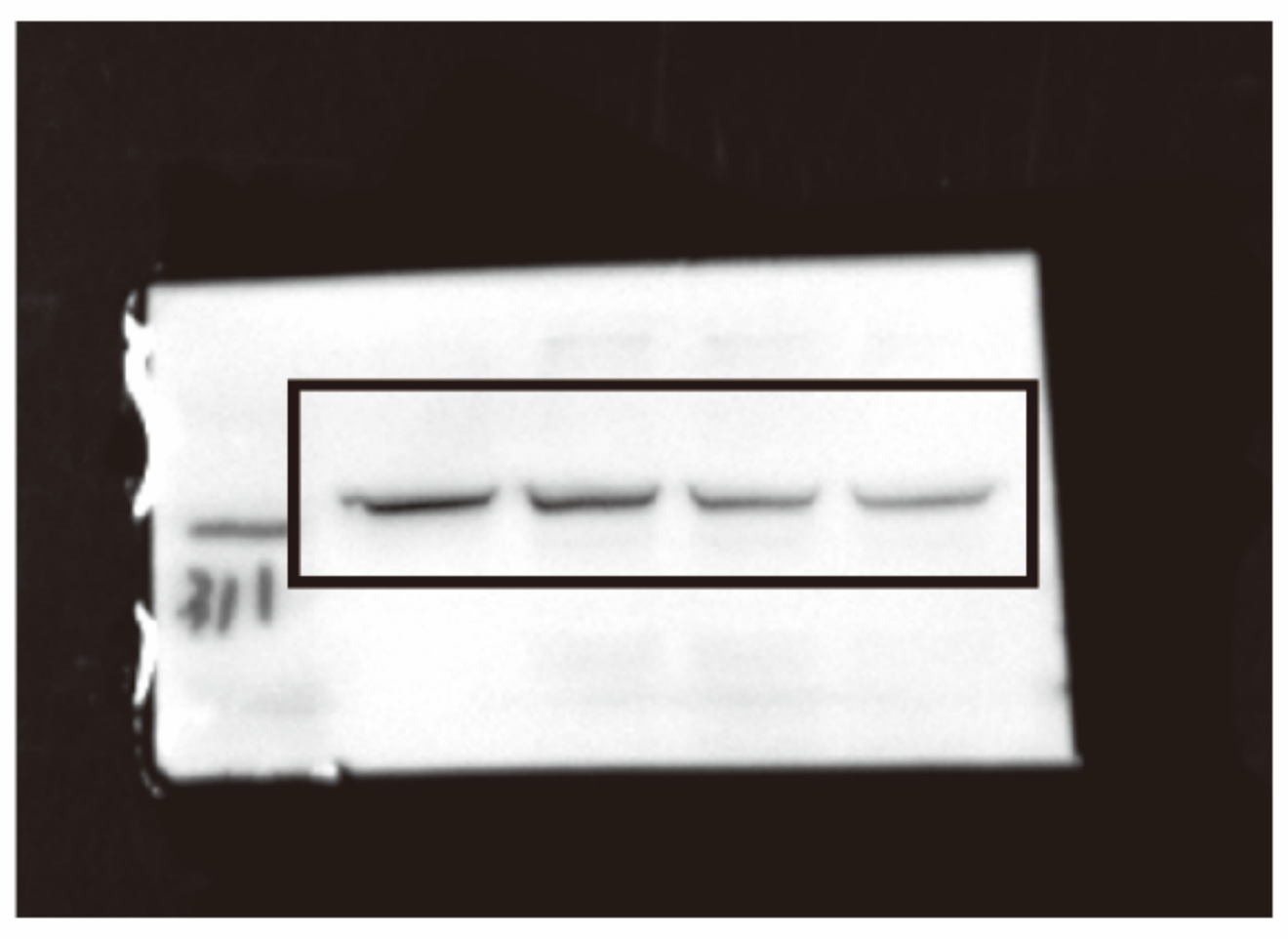

β-actin

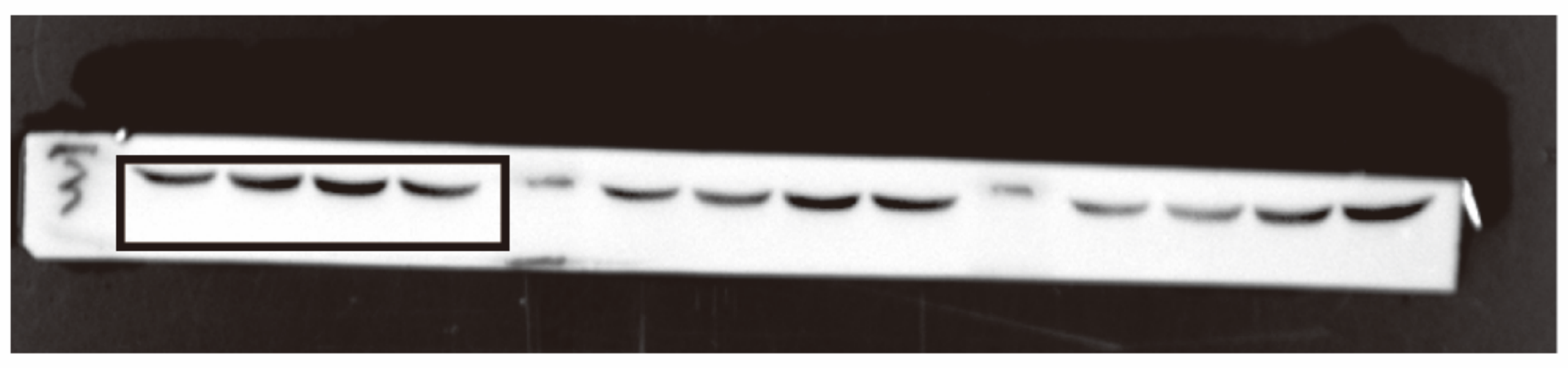

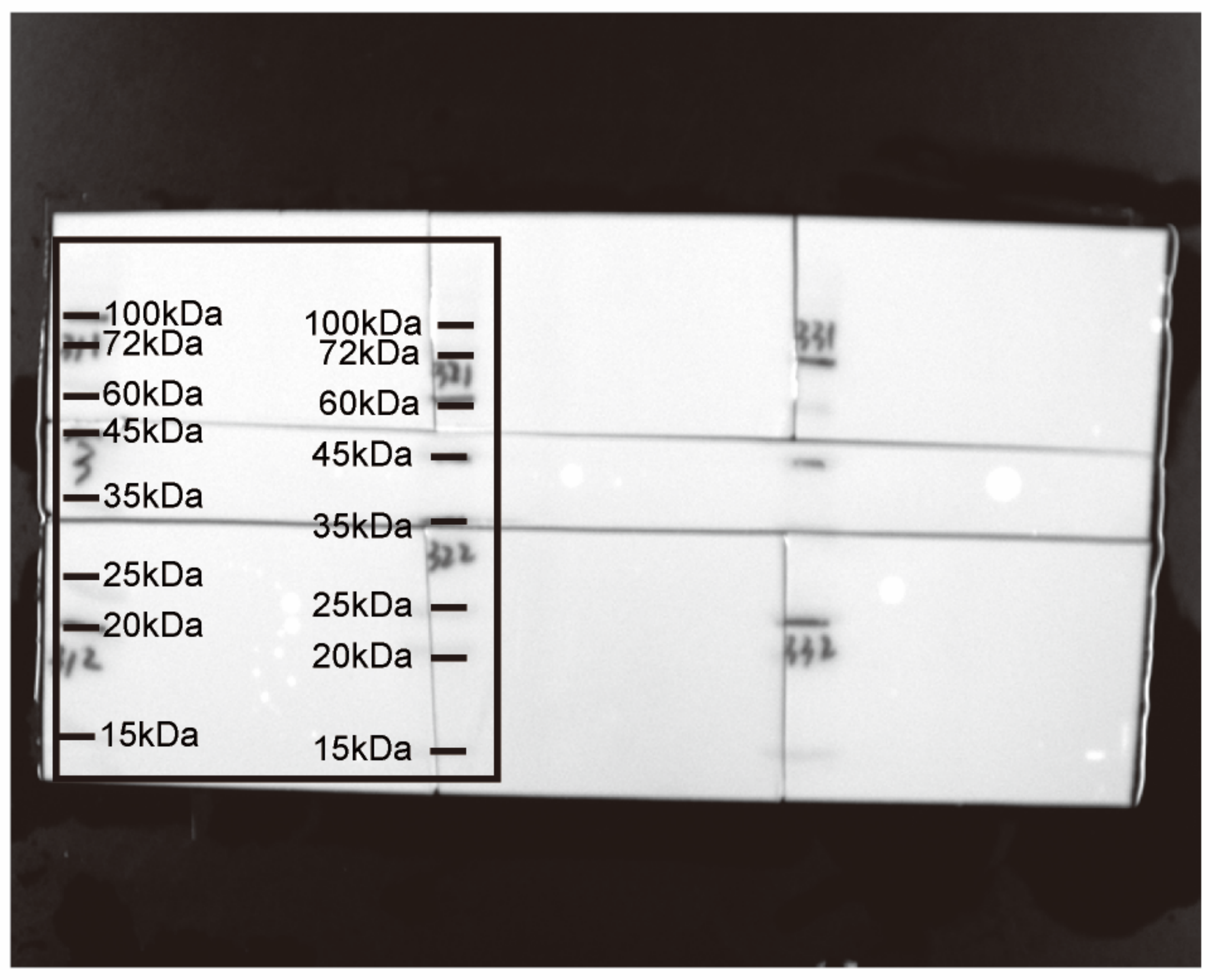

Fig.7C

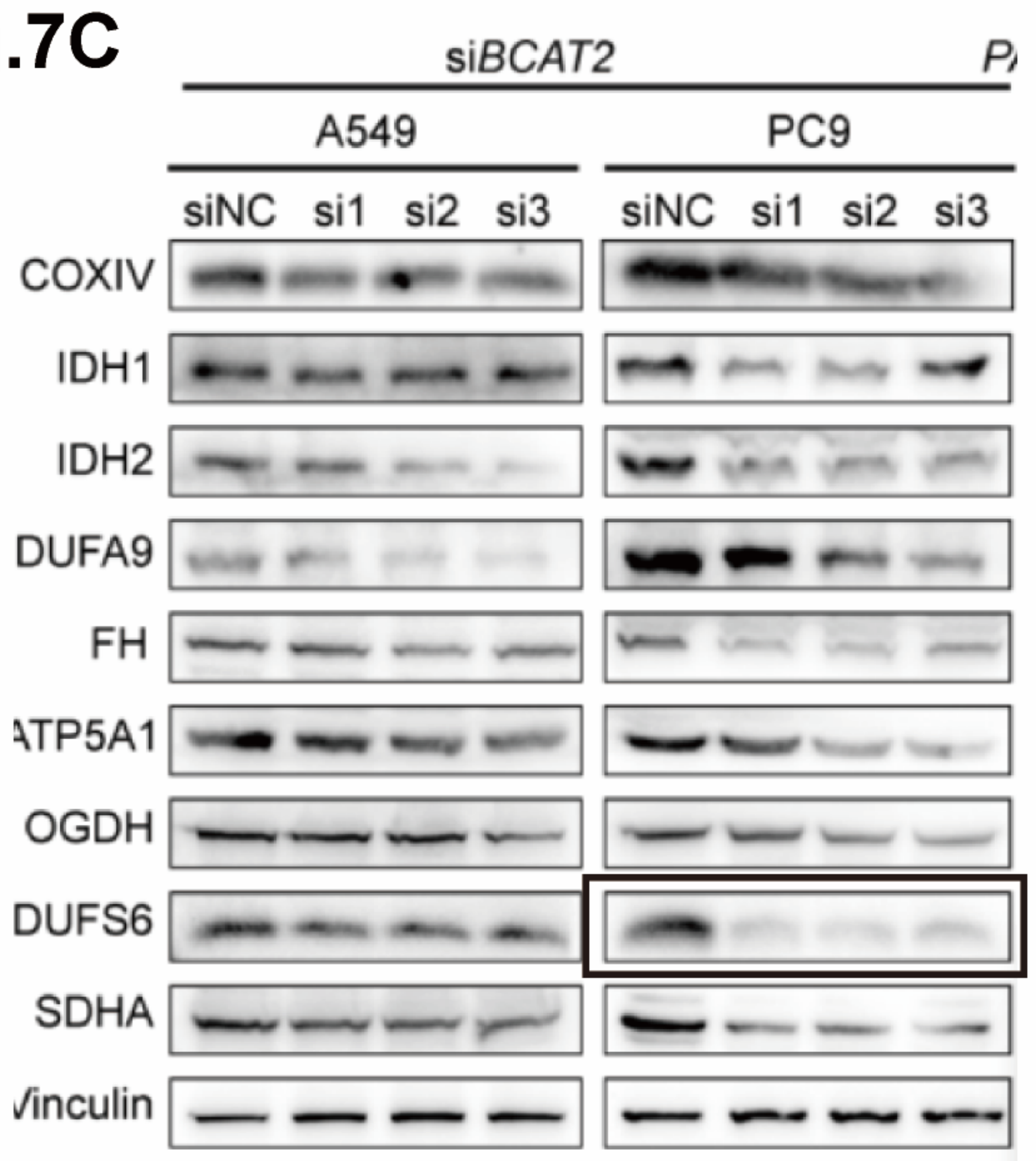

Vinculin

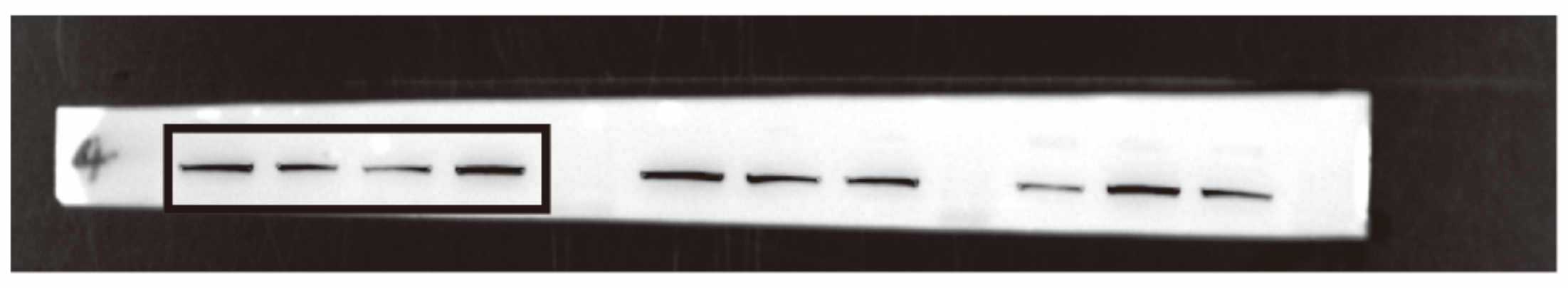

NDUFS6

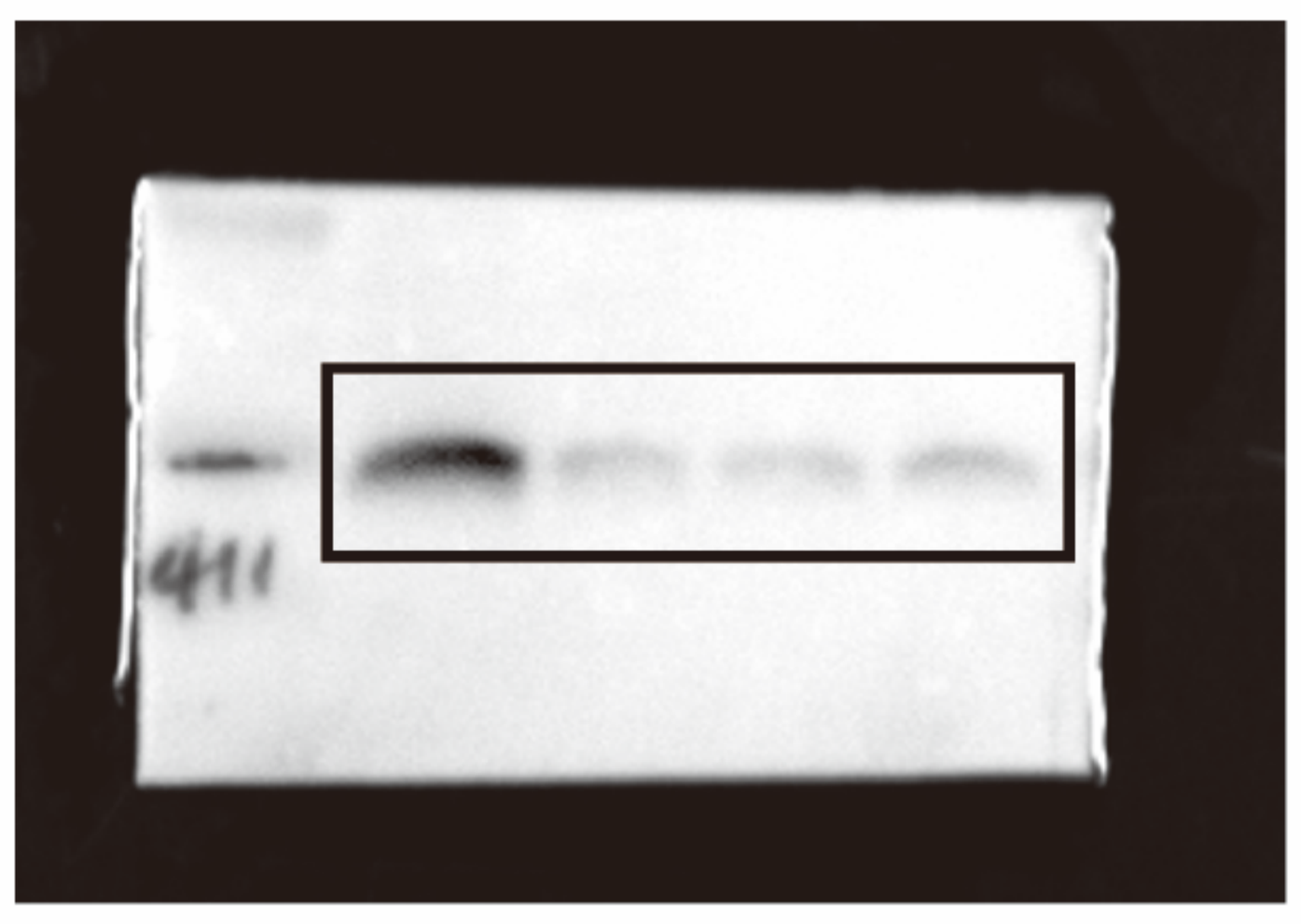

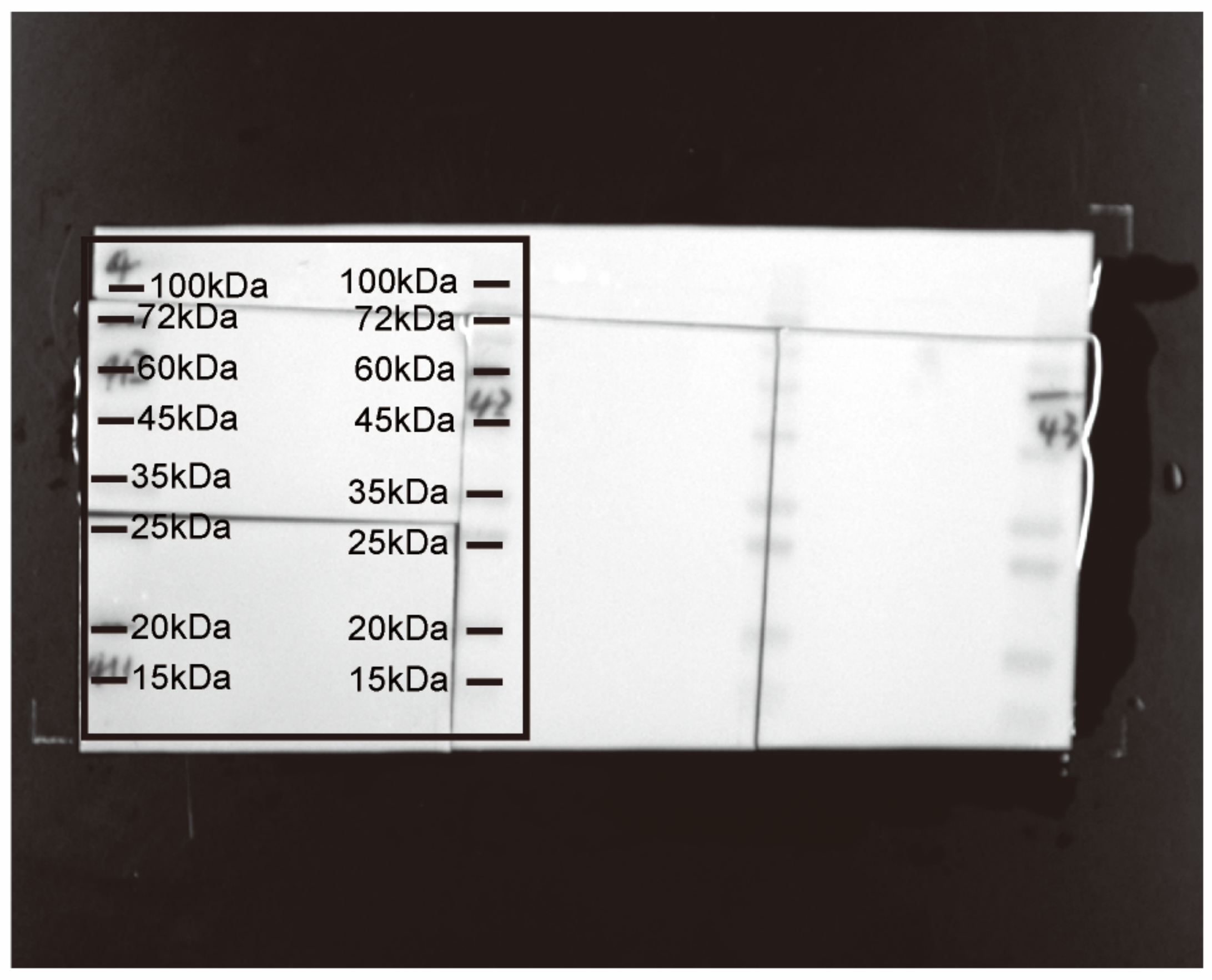

Fig.7C

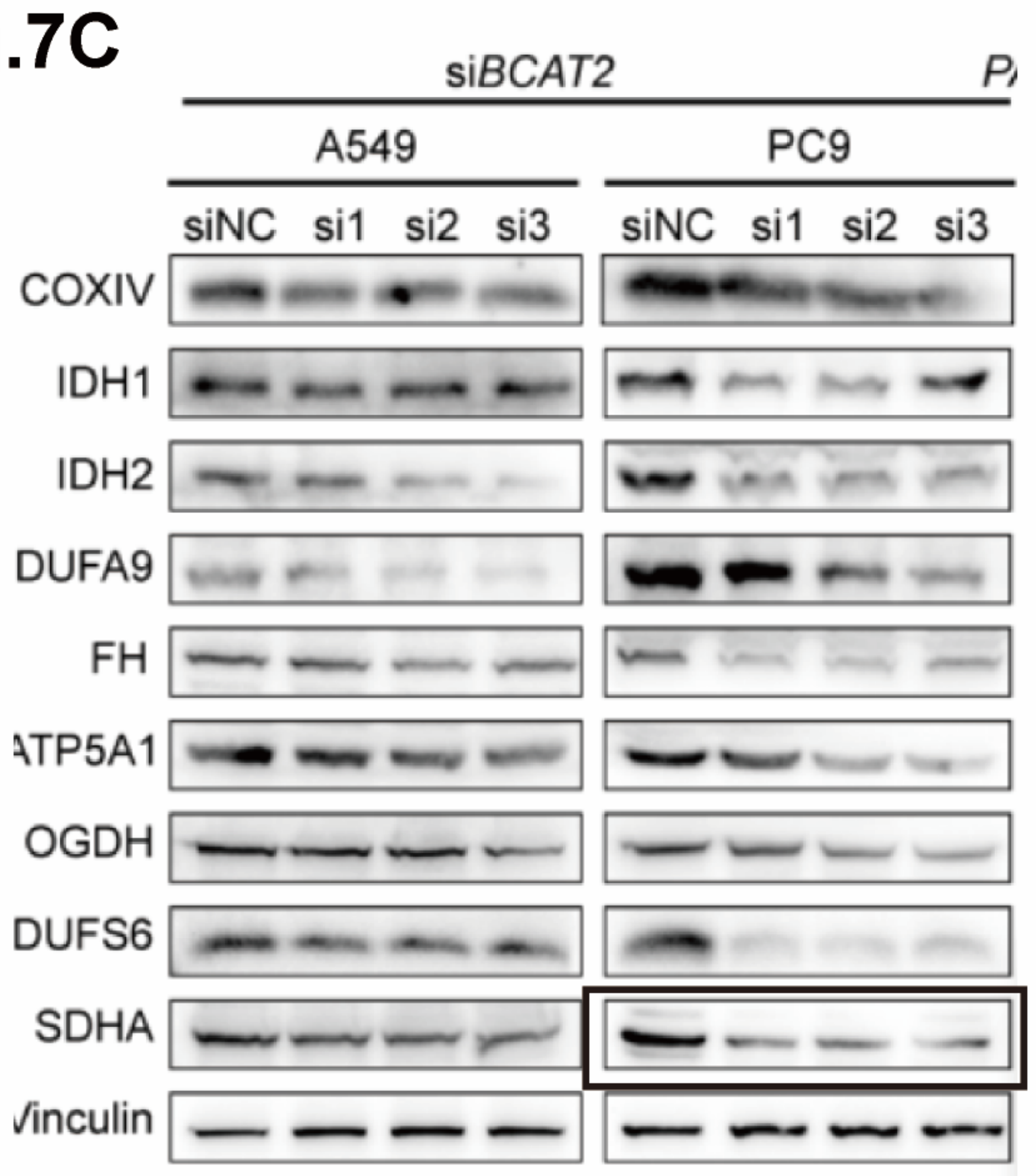

Vinculin

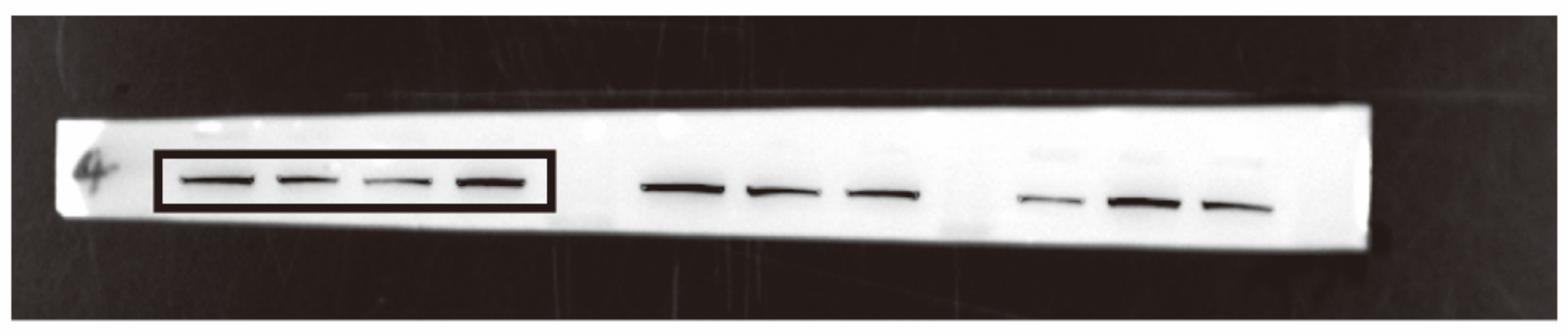

SDHA

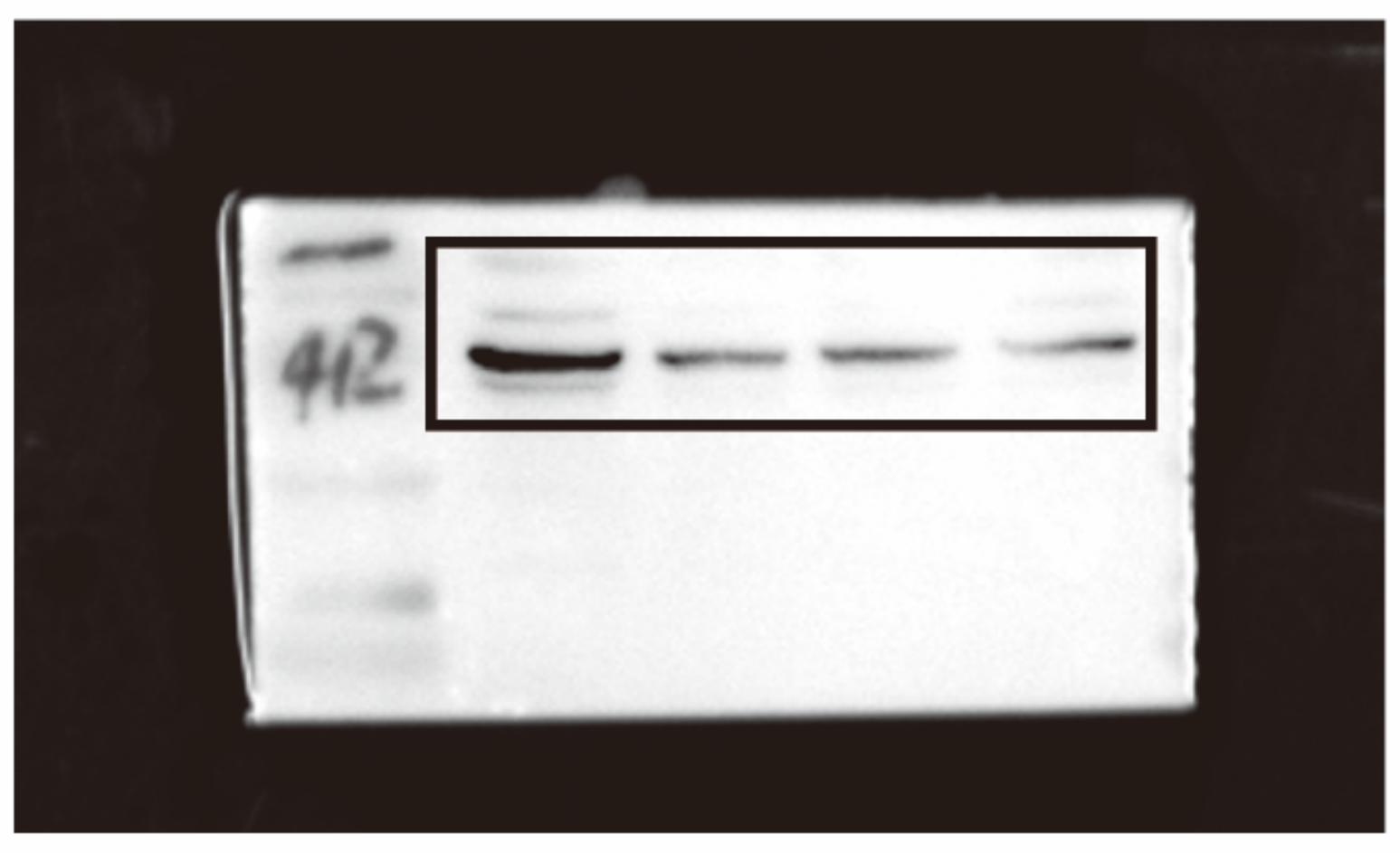

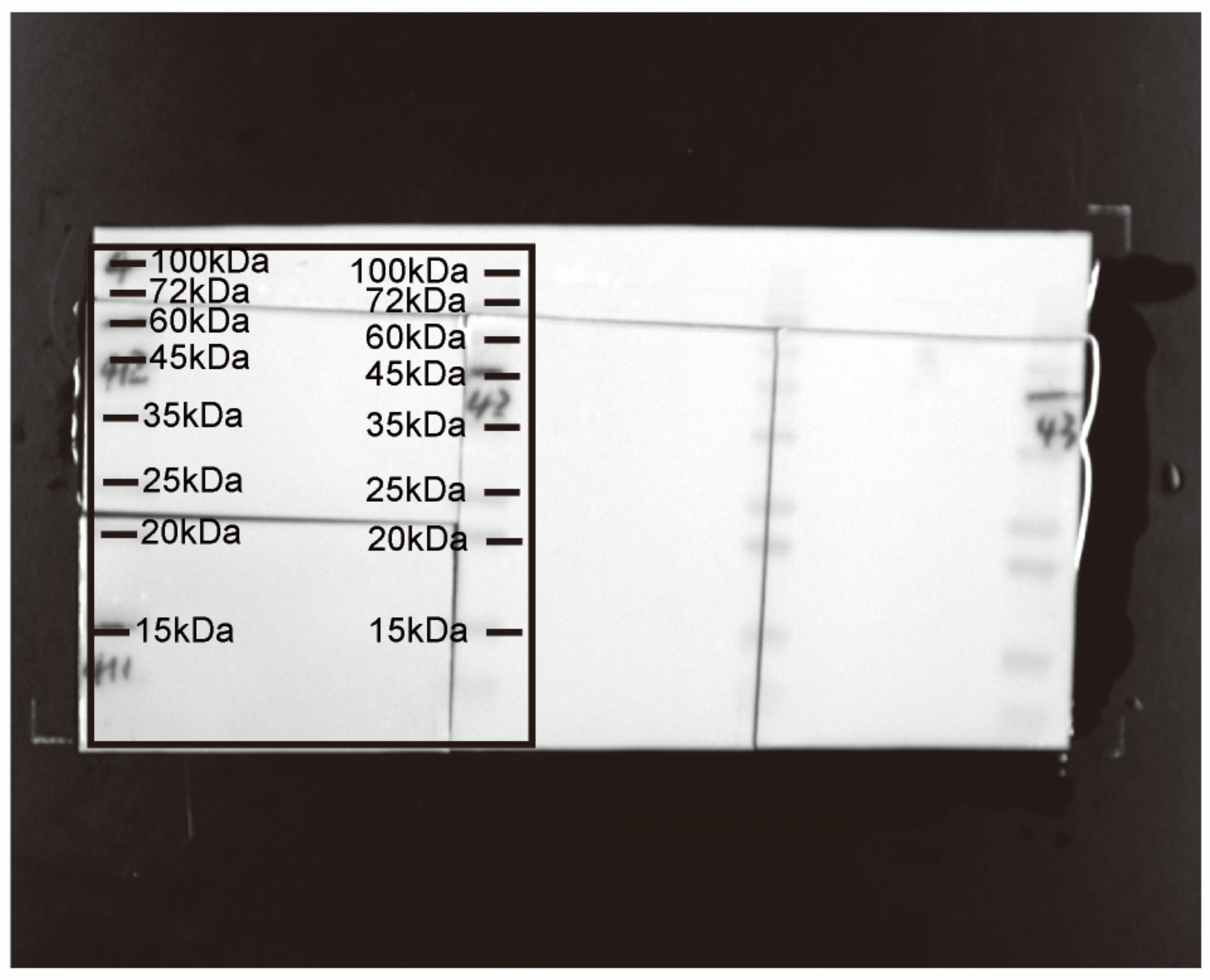

Fig.S4E

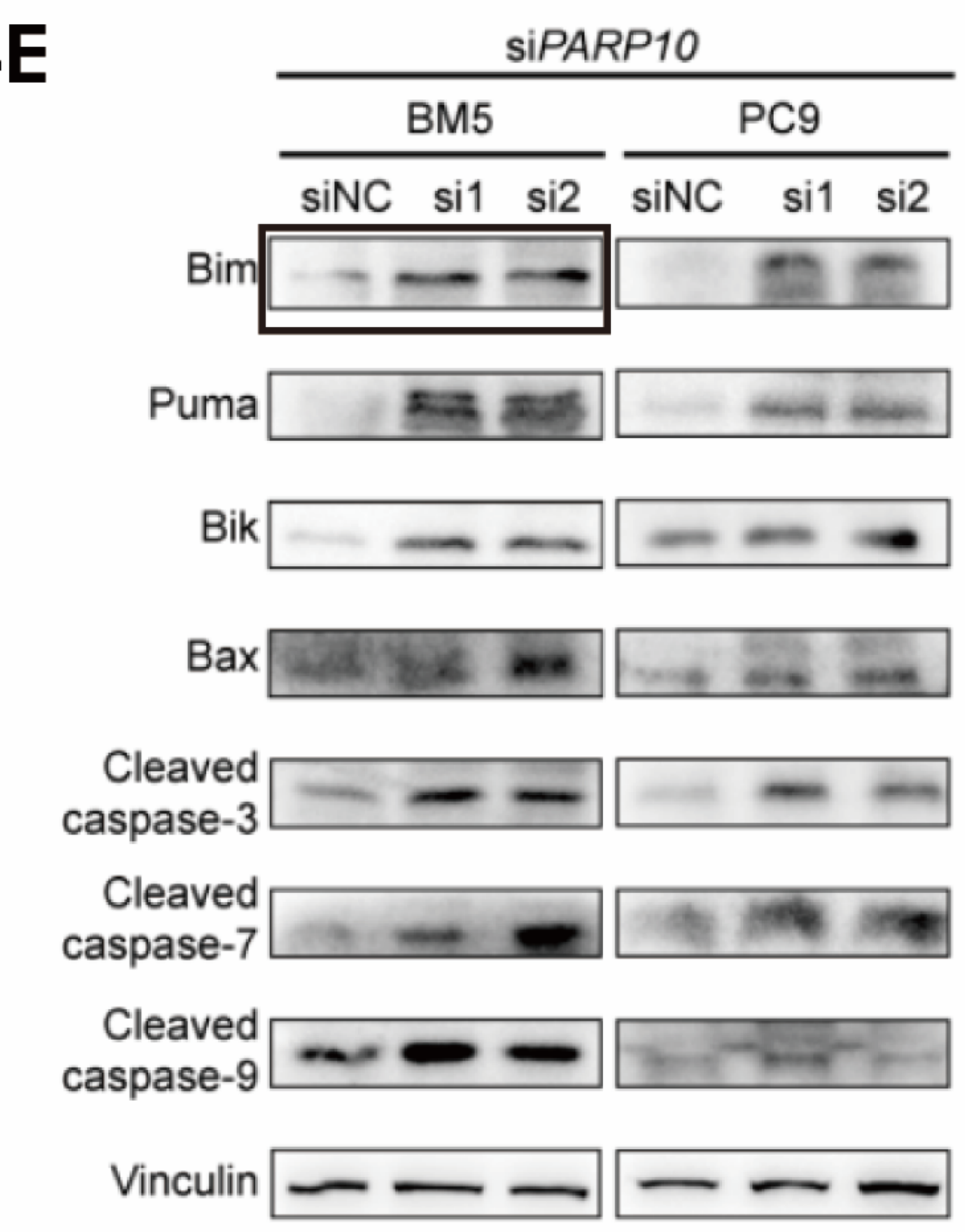

Vinculin

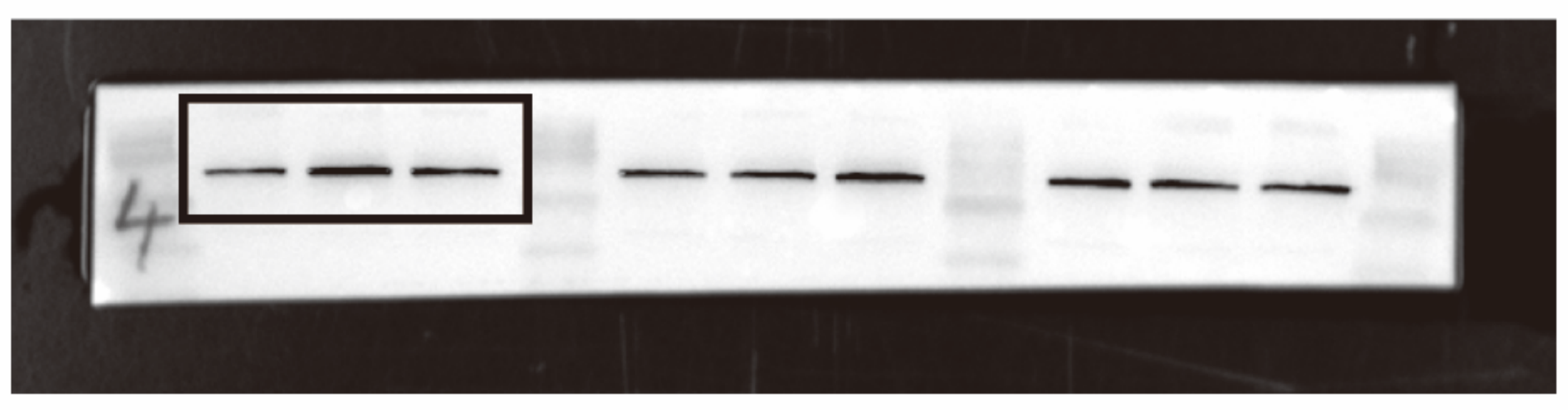

Bim

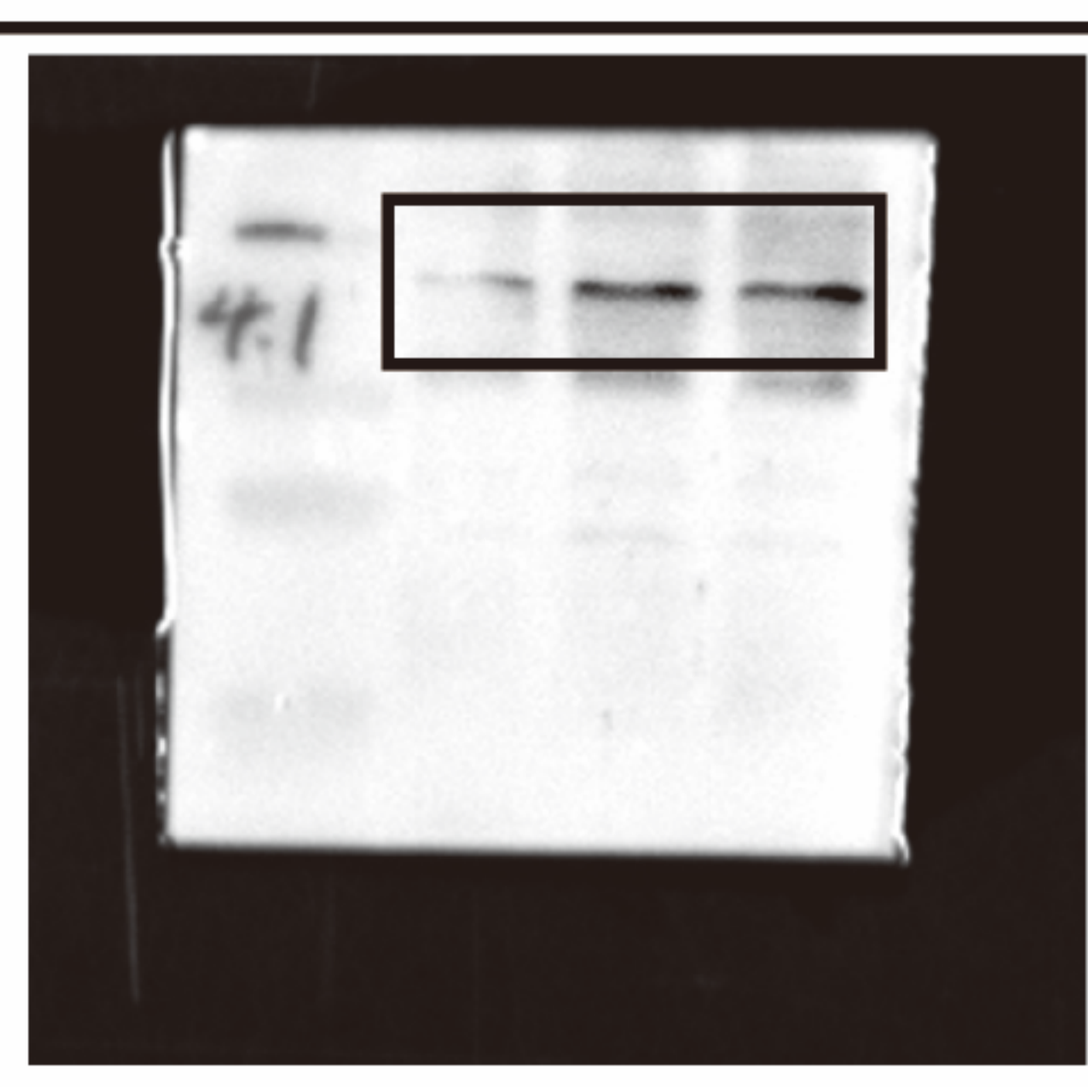

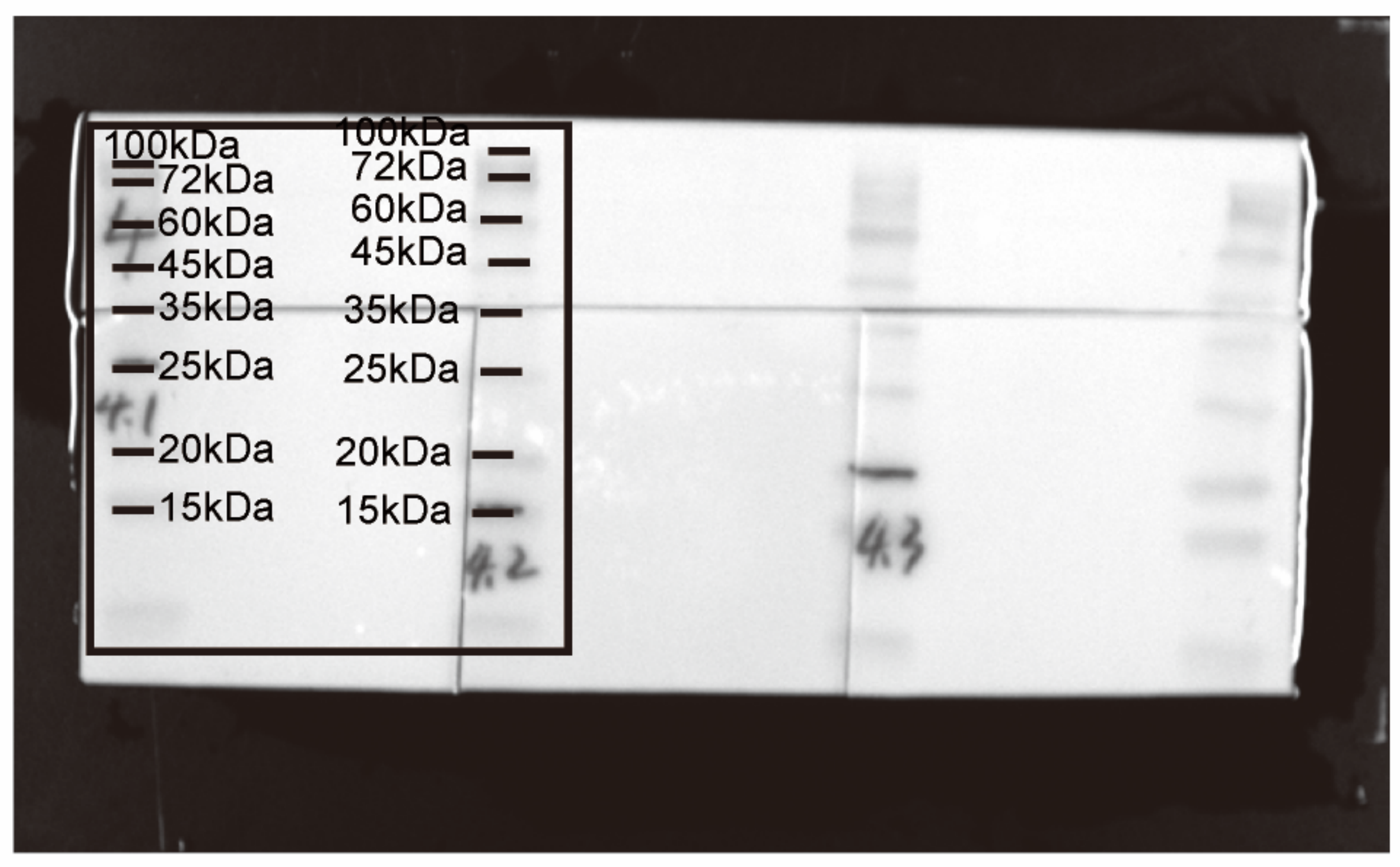

Figure	Merged	Stiched full image

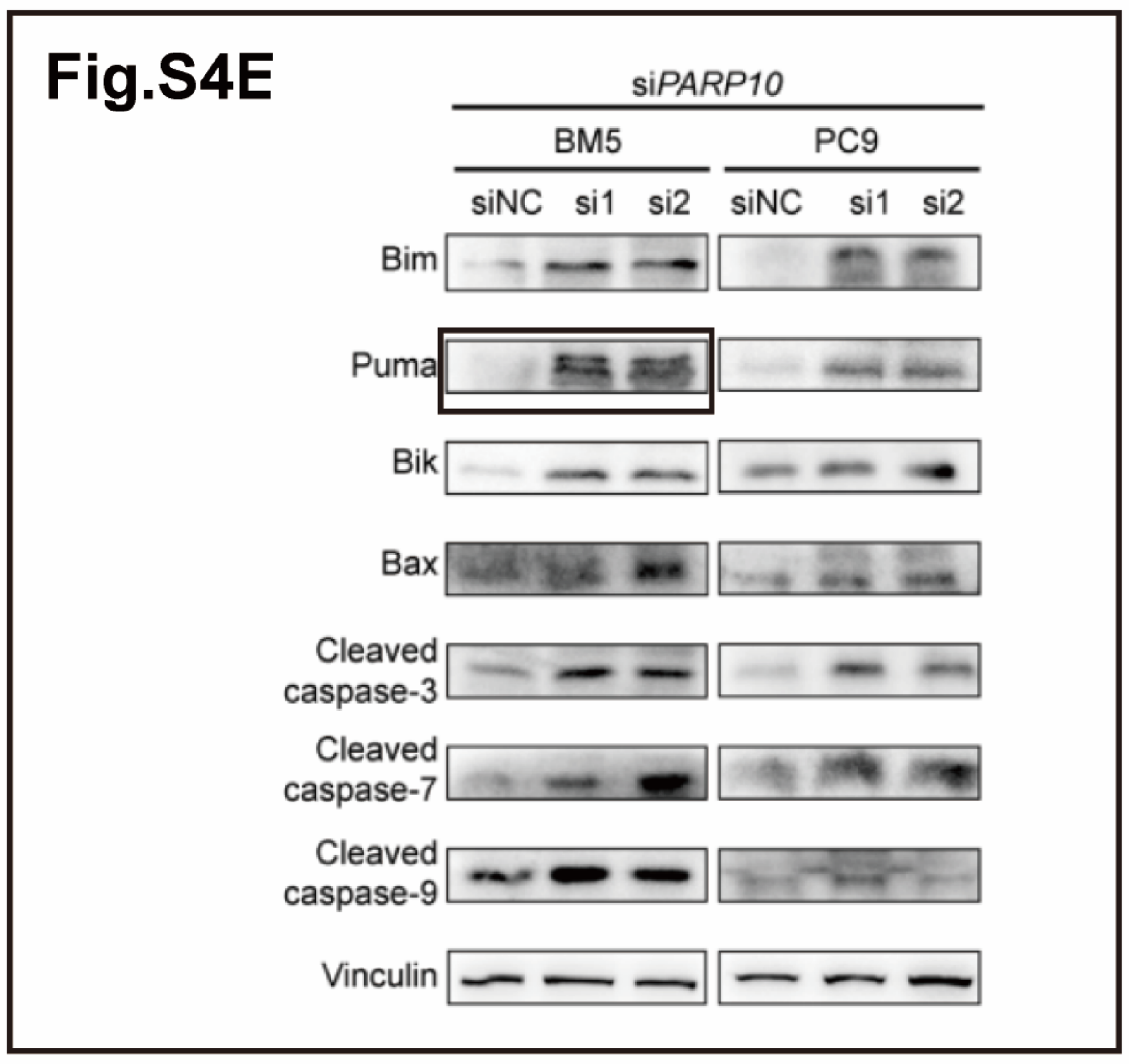

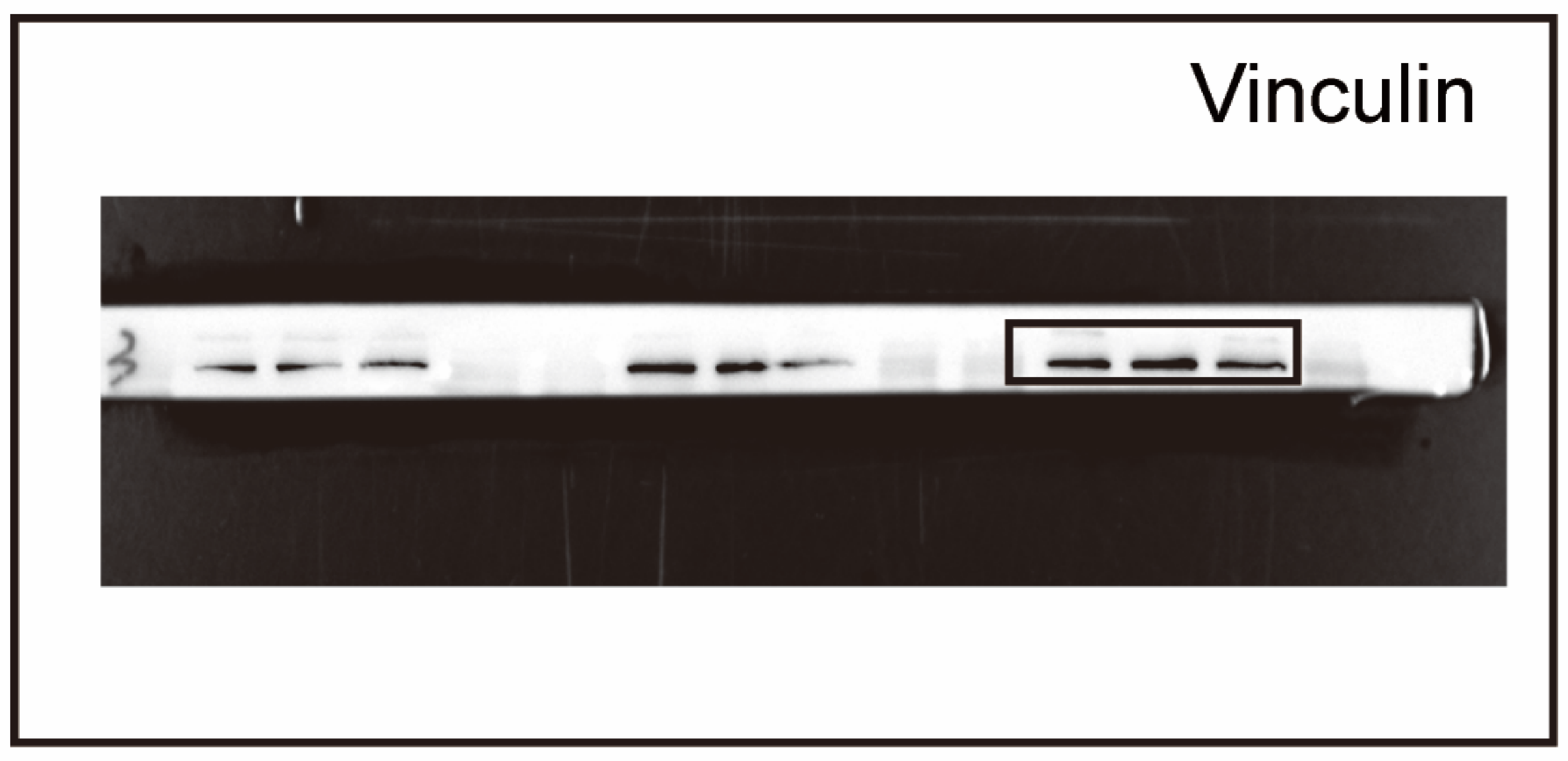

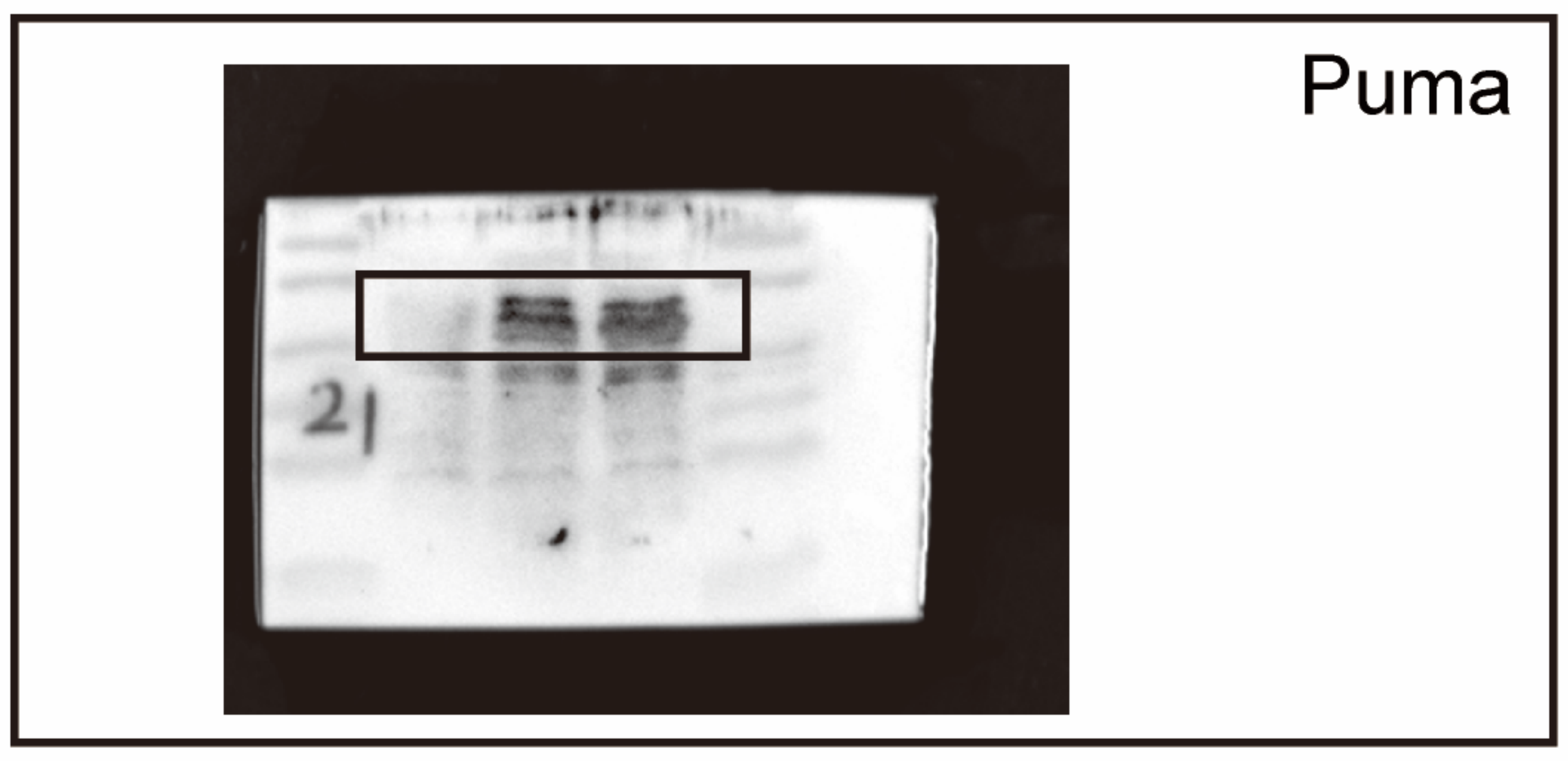

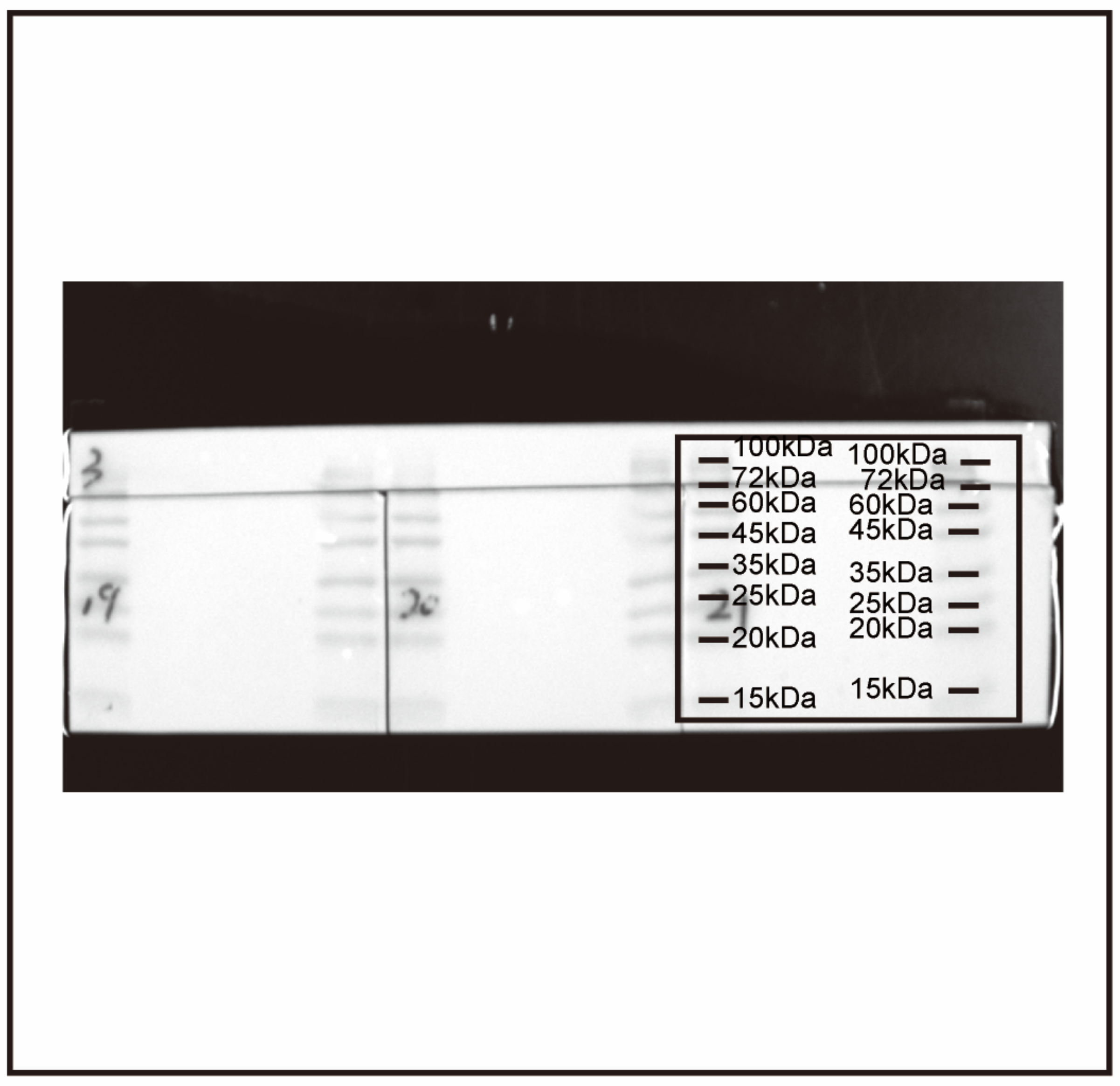

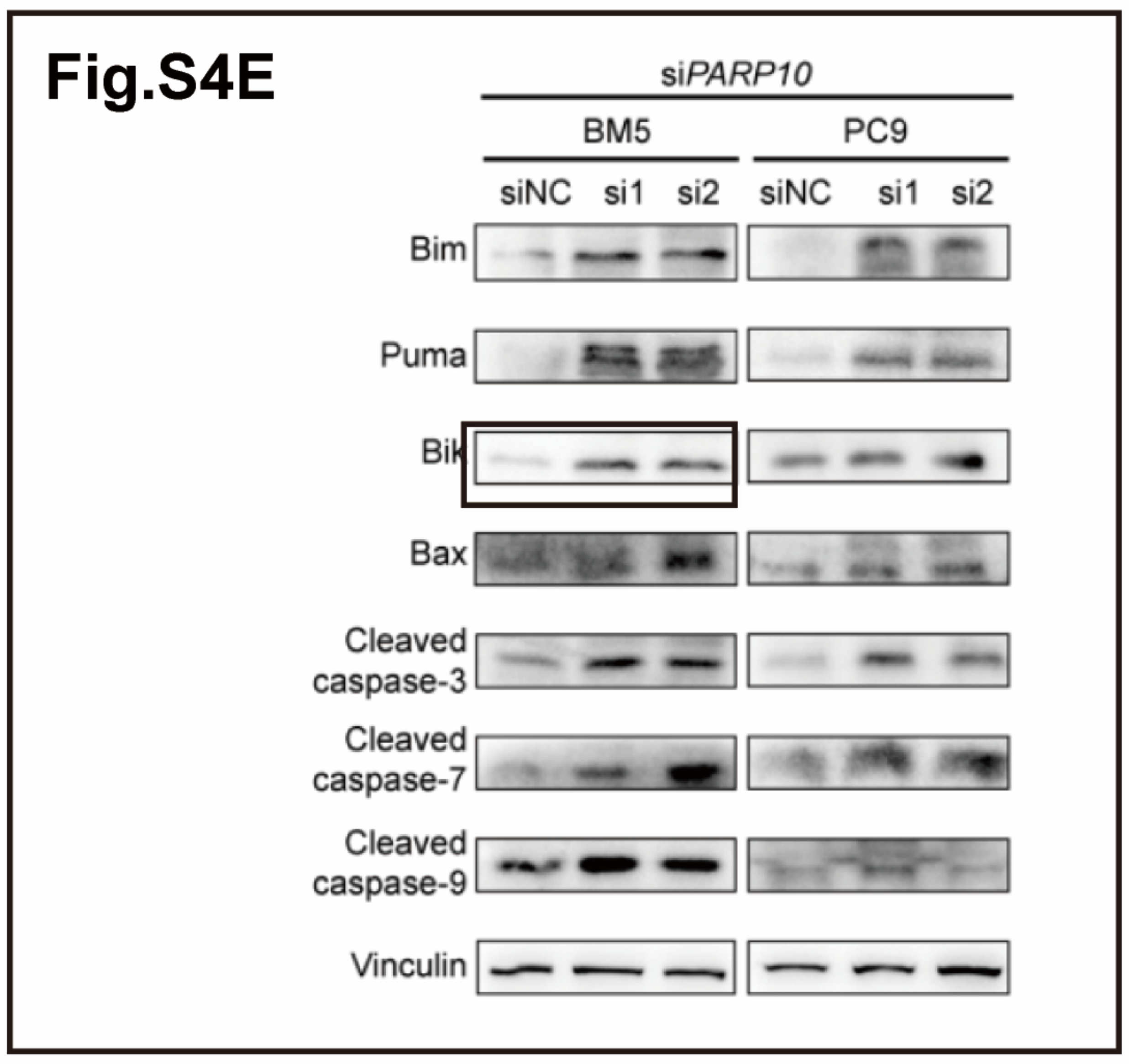

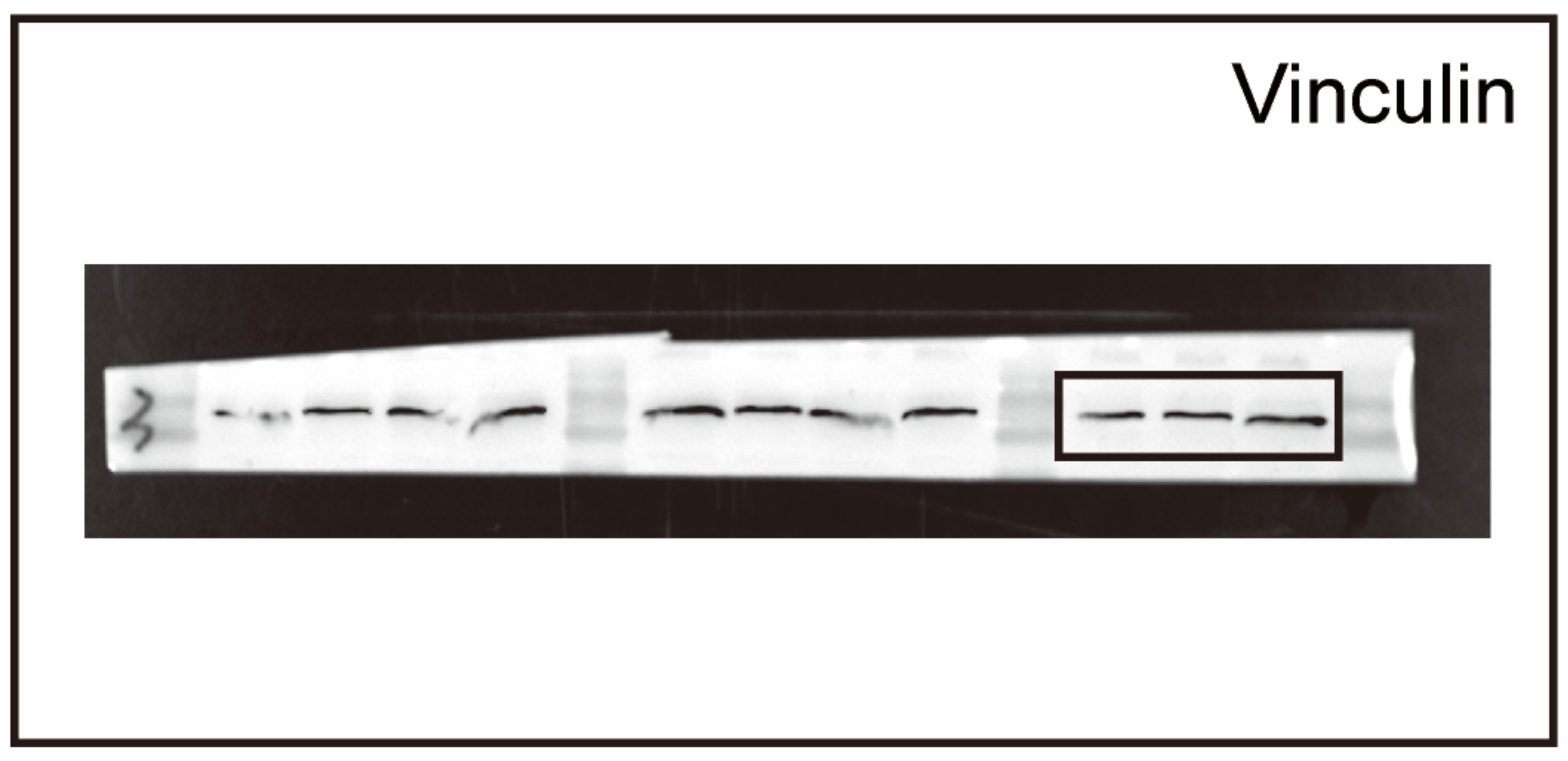

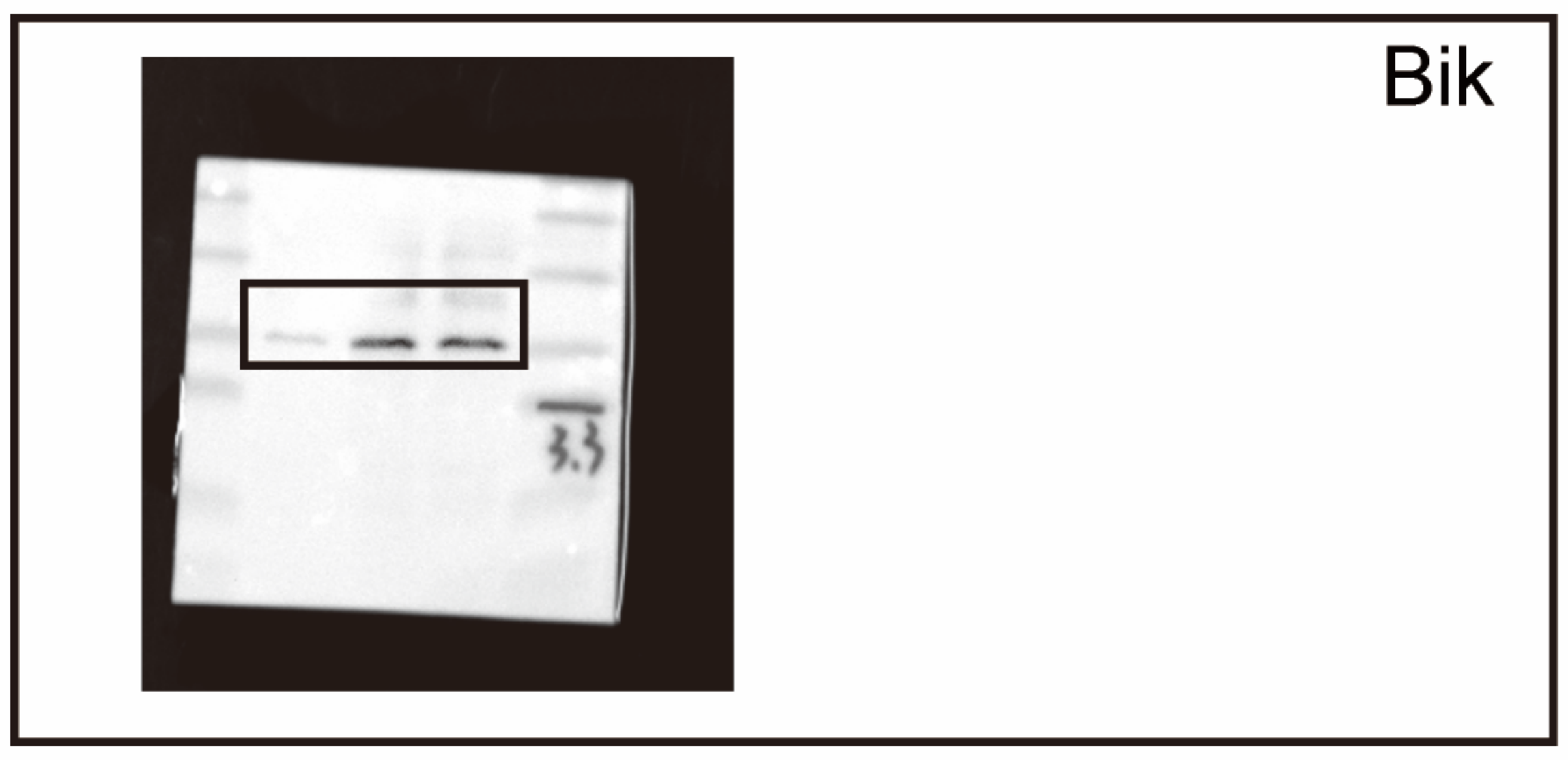

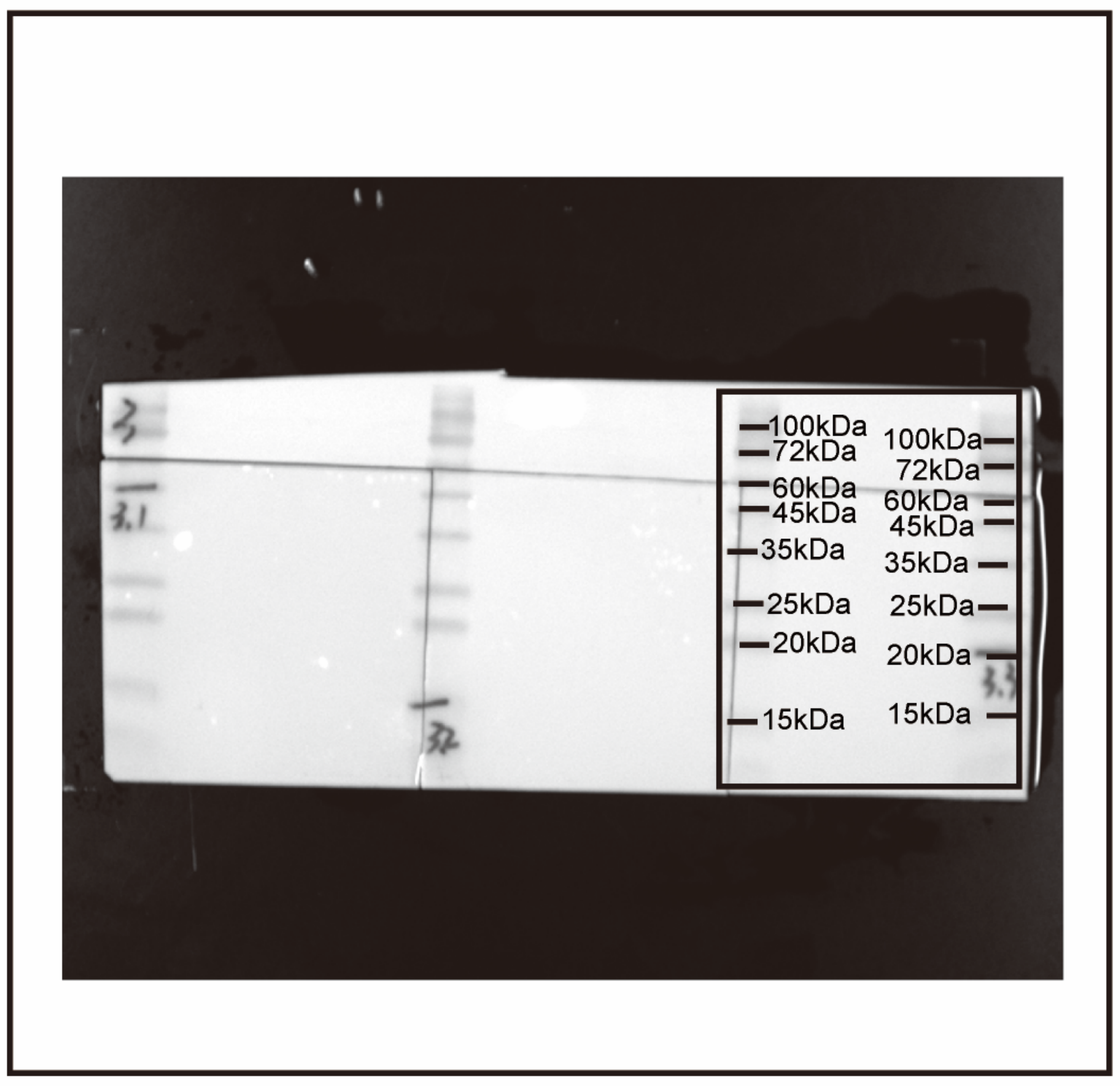

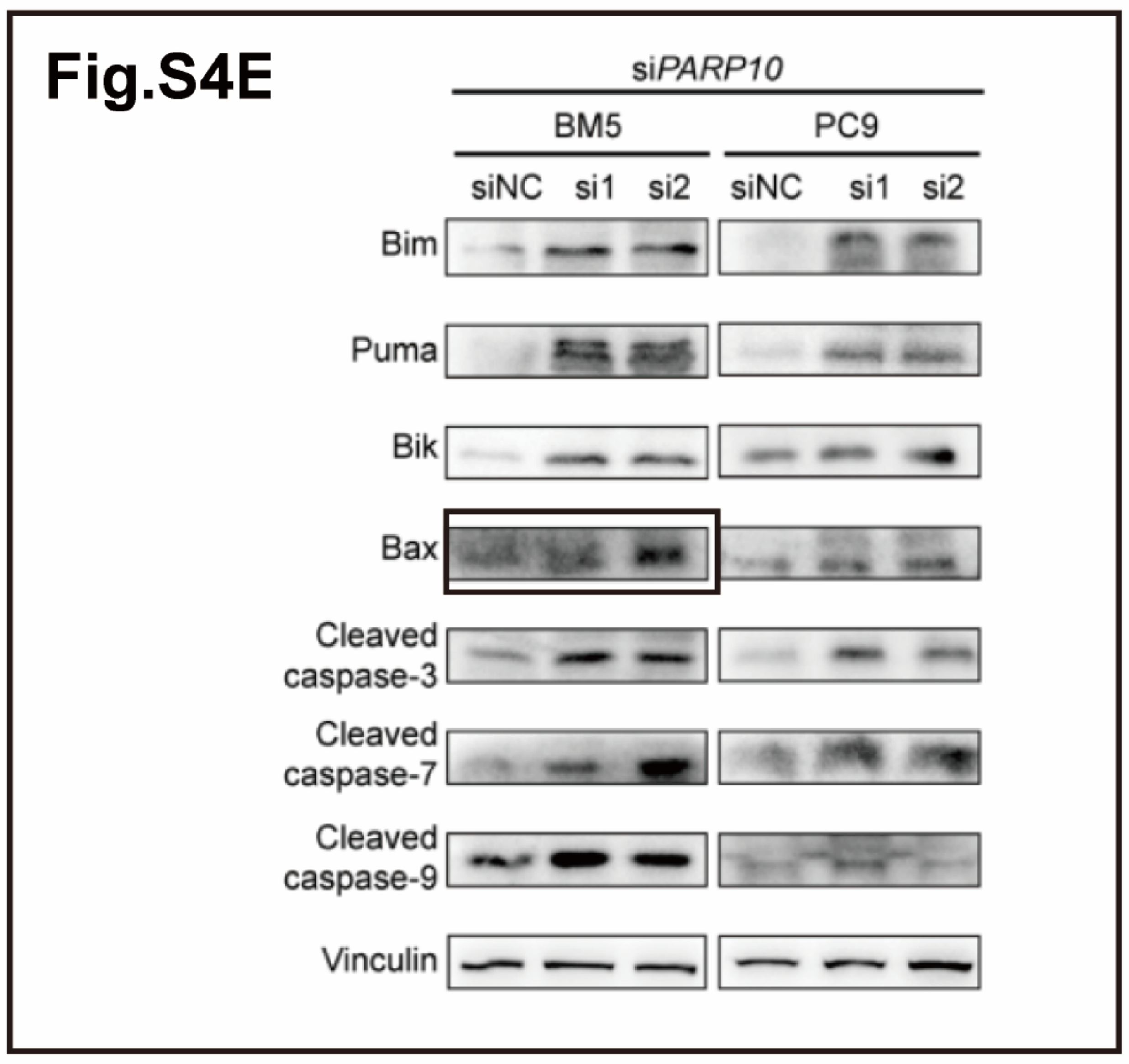

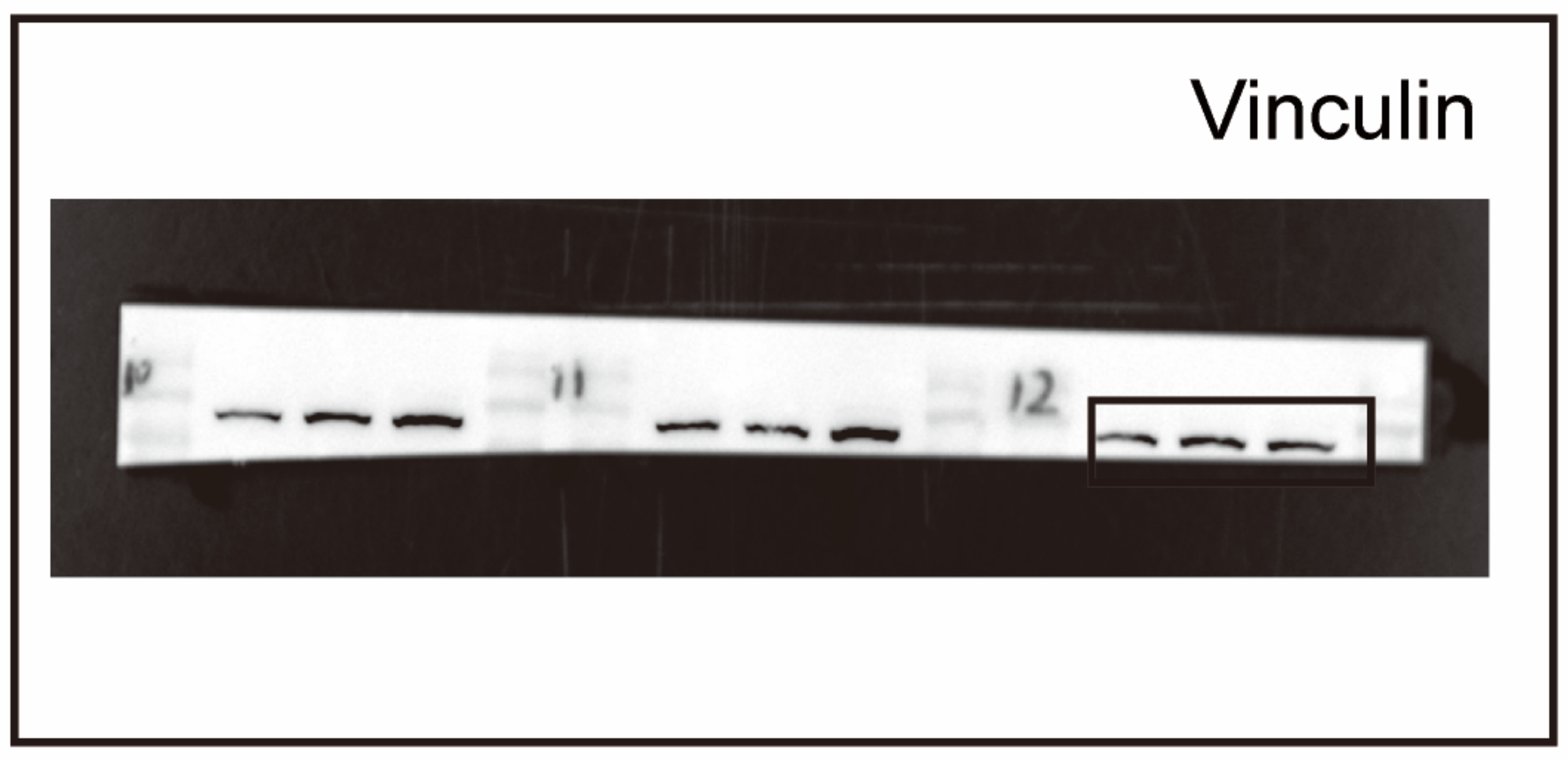

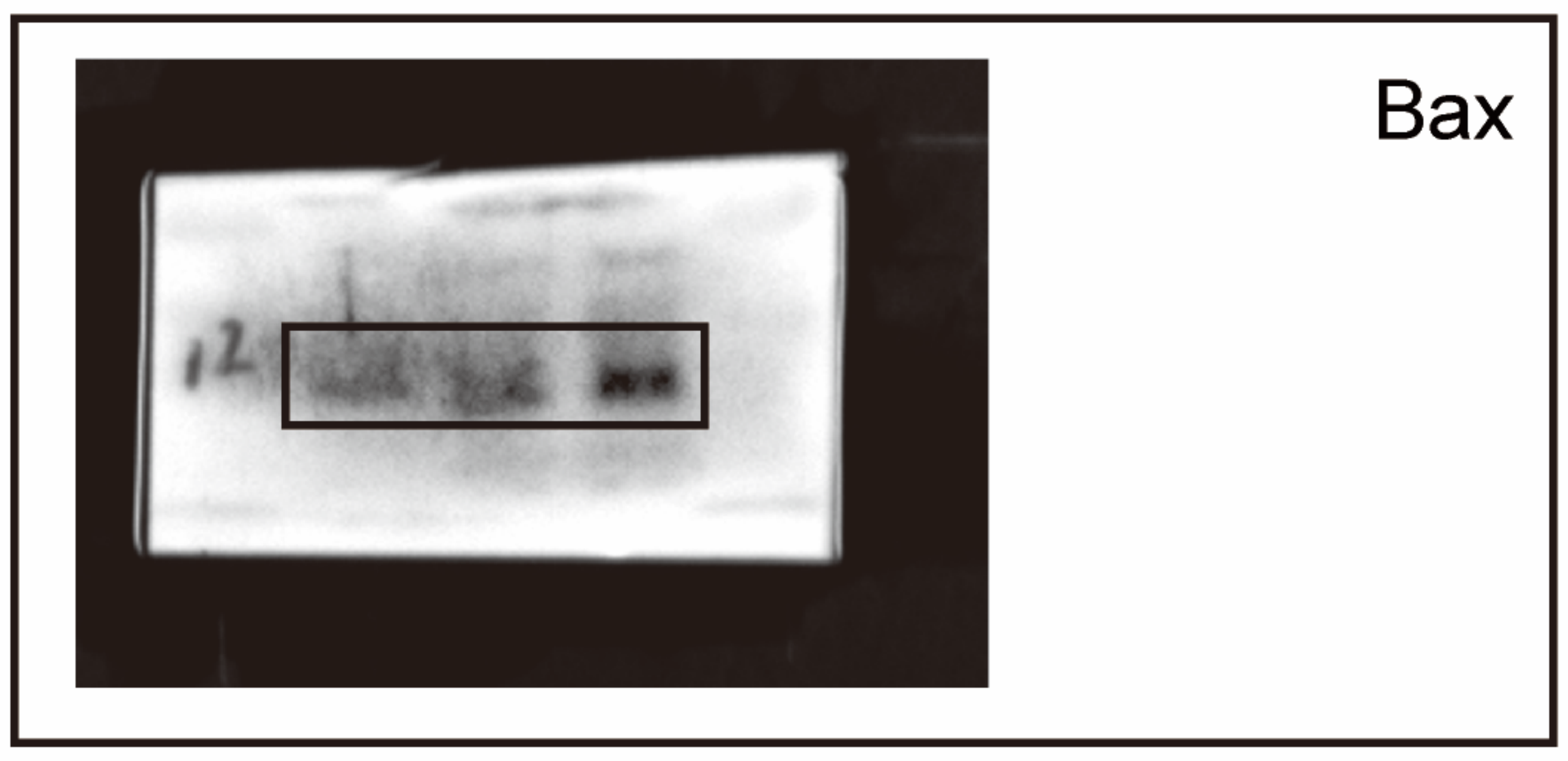

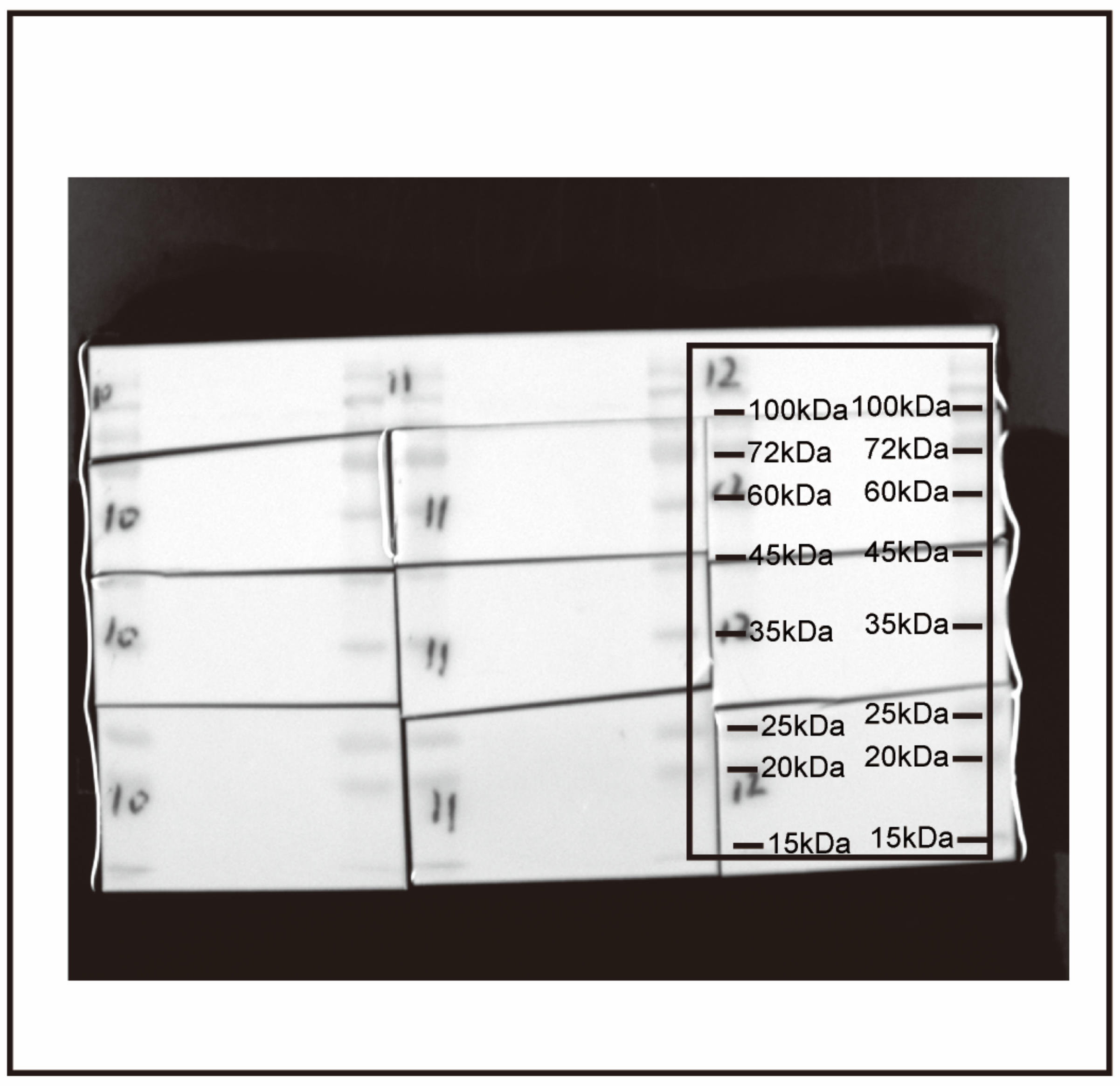

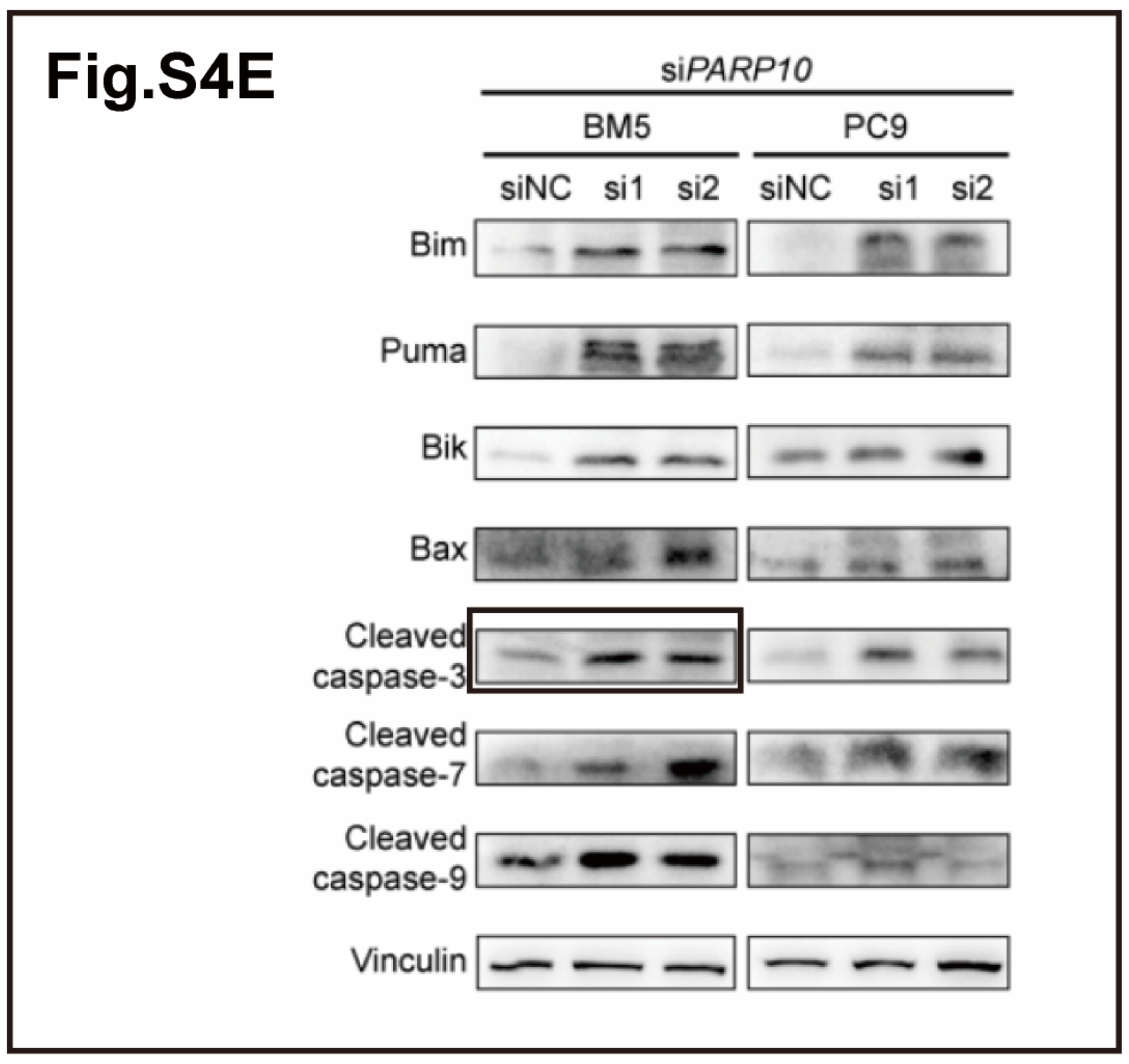

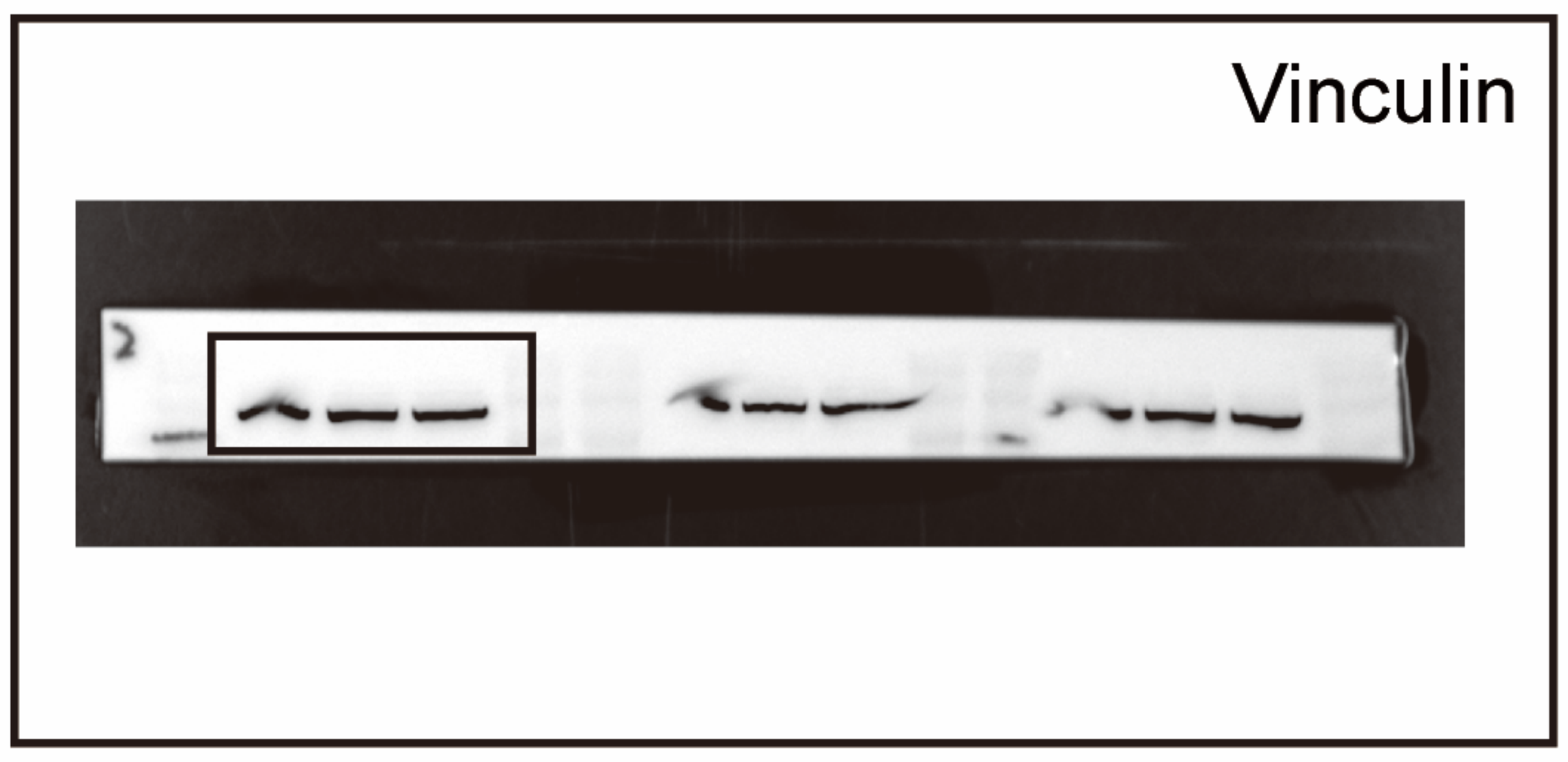

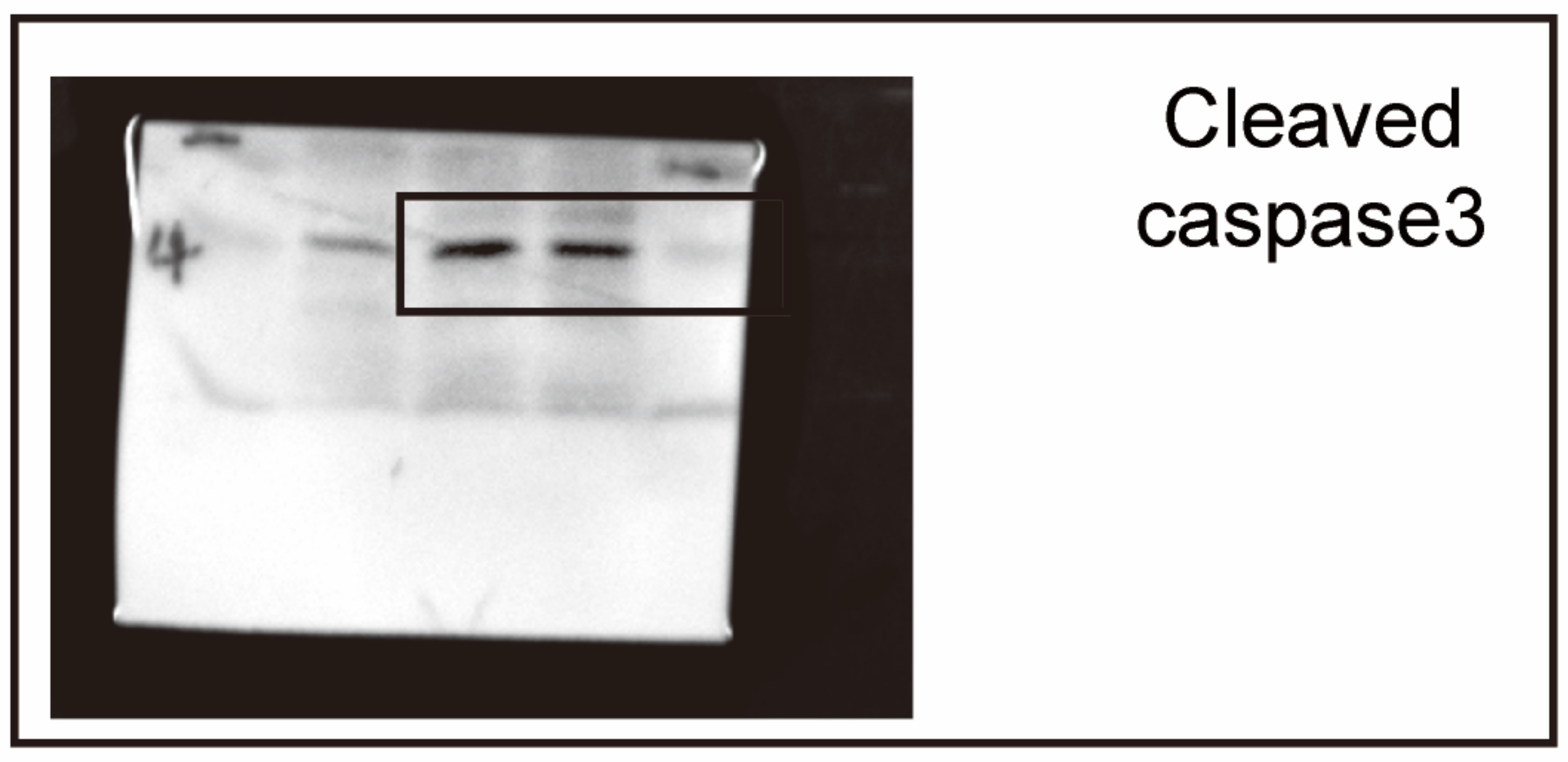

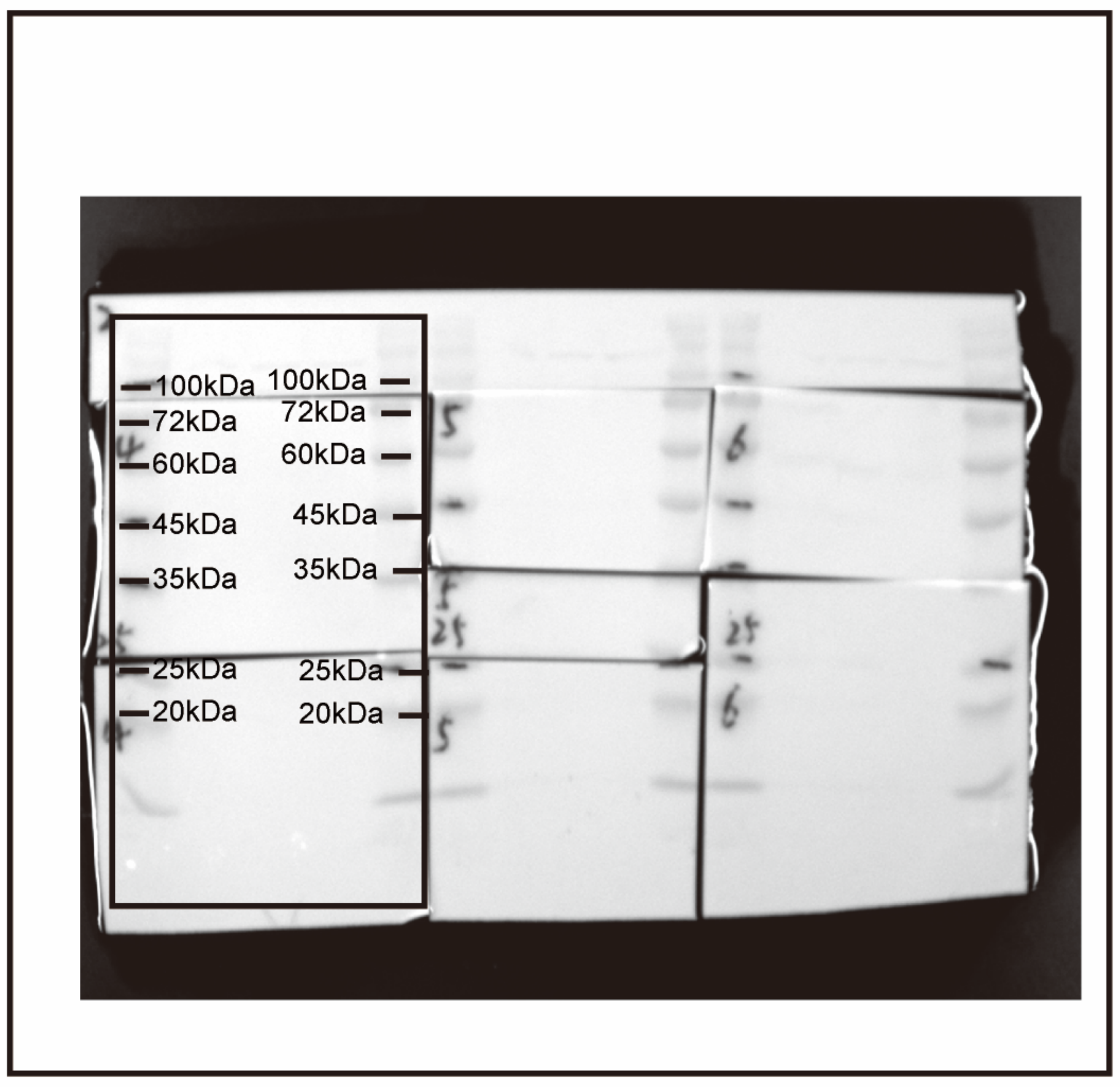

Figure	Merged	Stiched full image

Fig.S4E

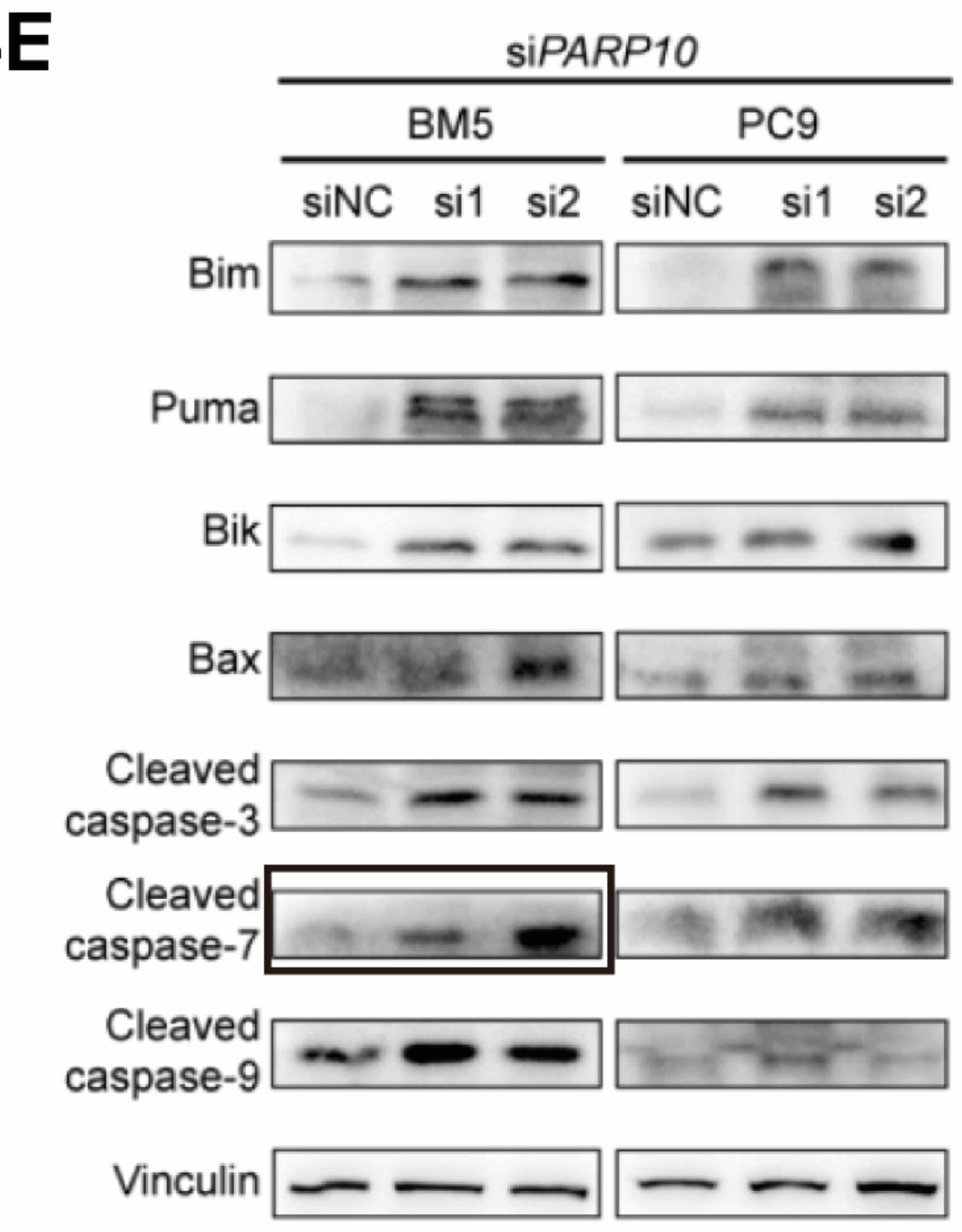

Vinculin

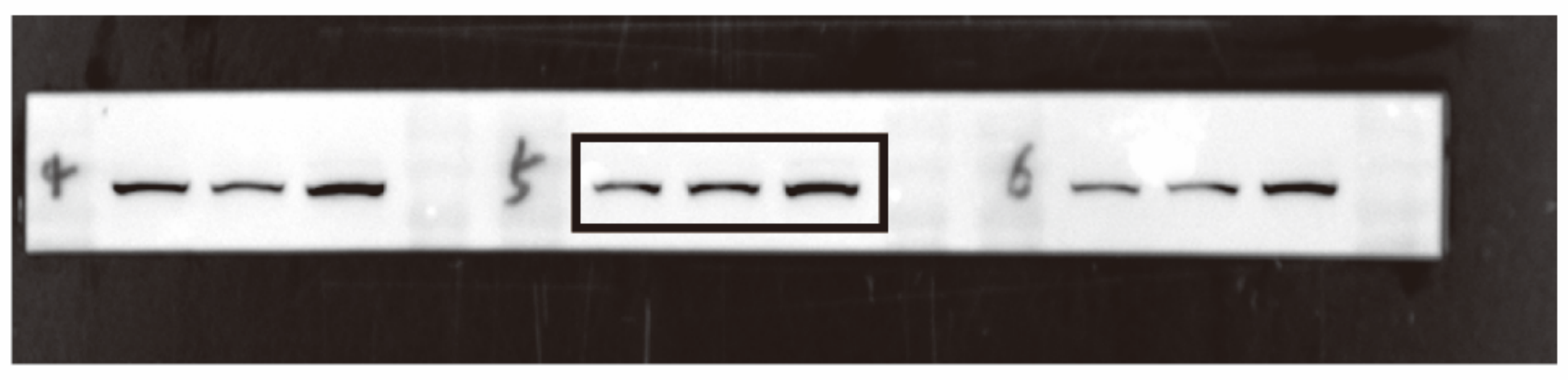

Cleaved caspase7

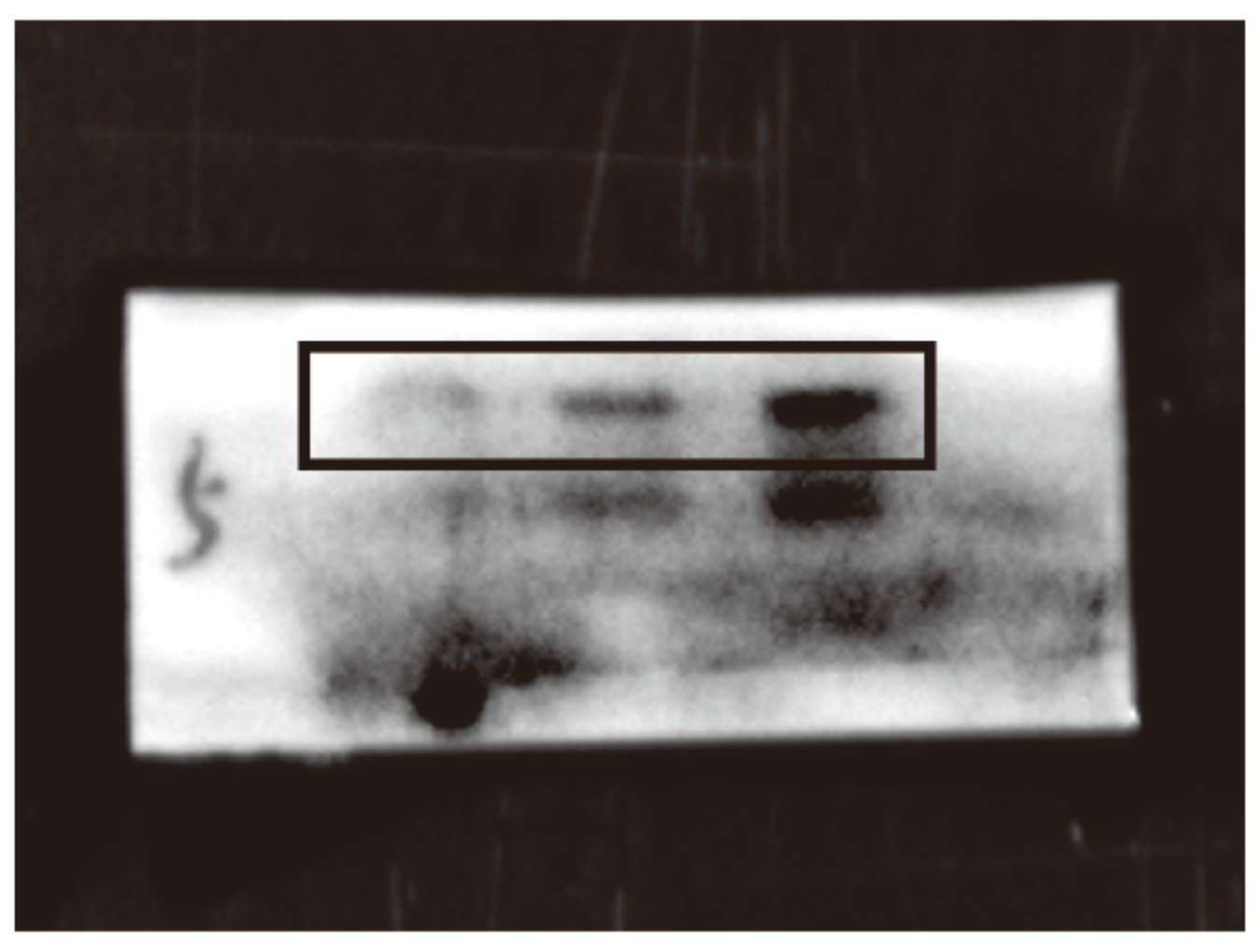

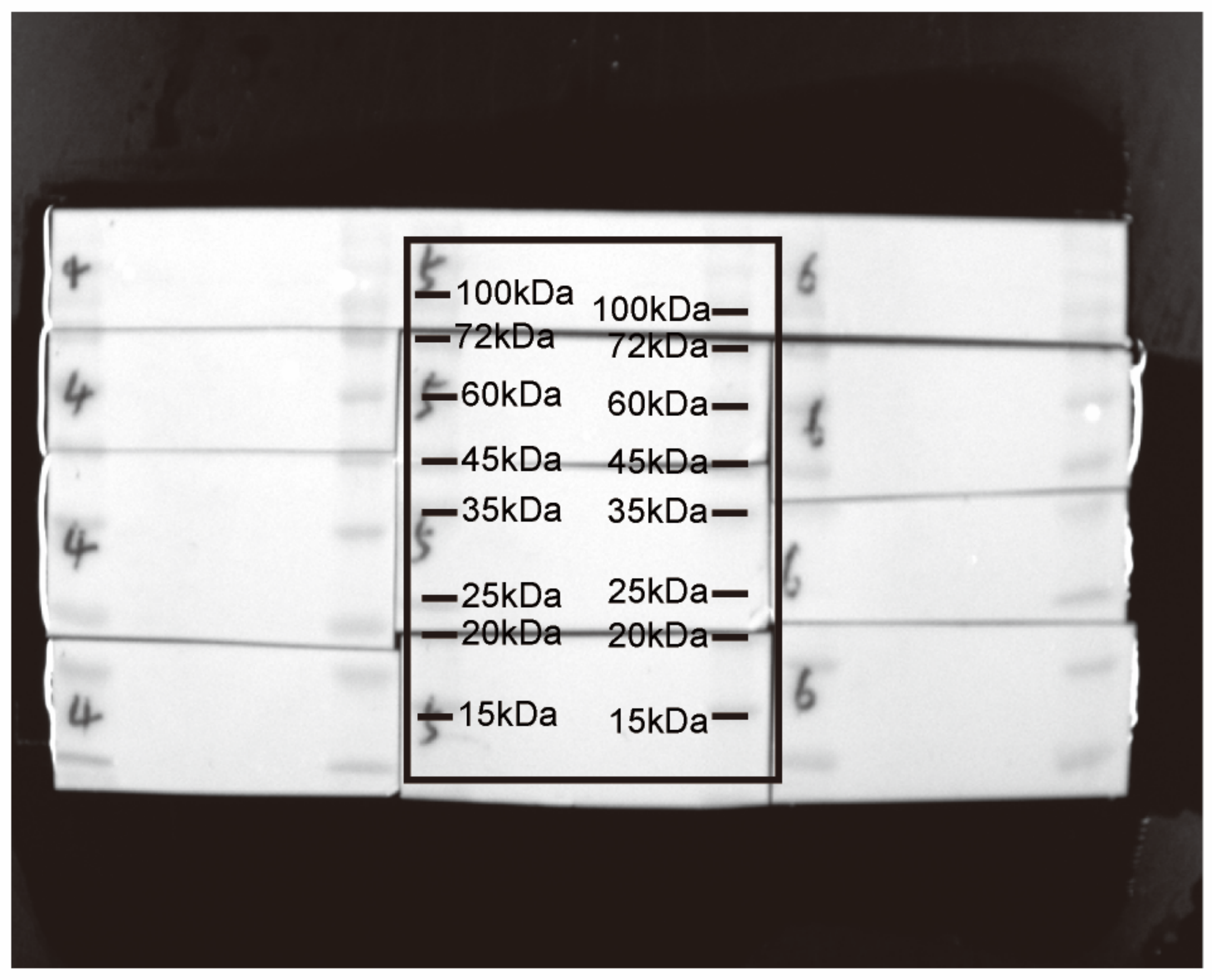

Fig.S4E

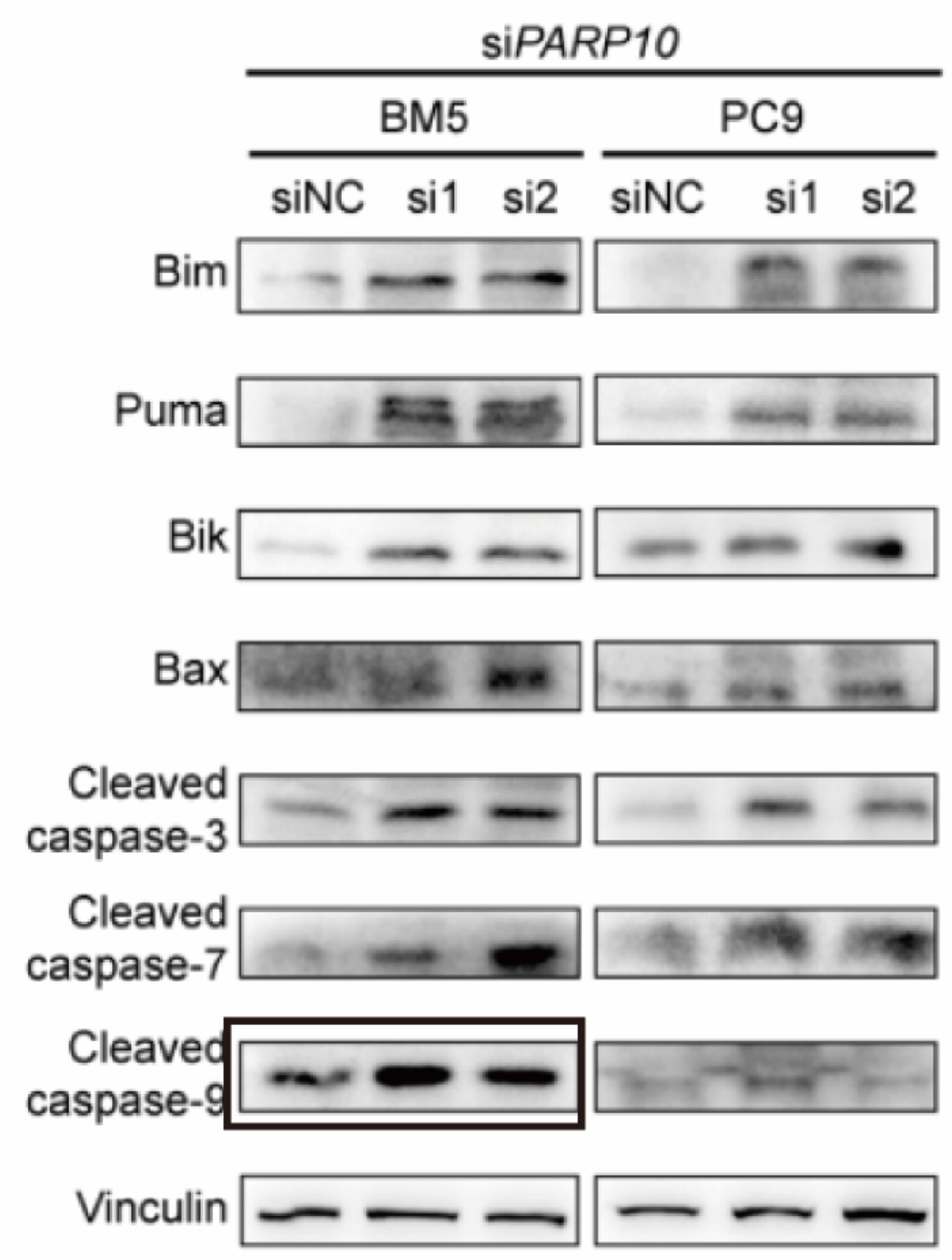

Vinculin

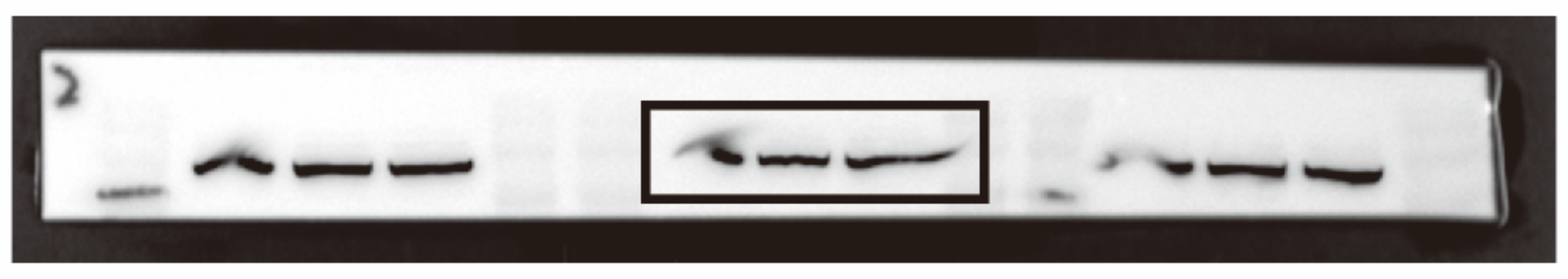

Cleaved caspase9

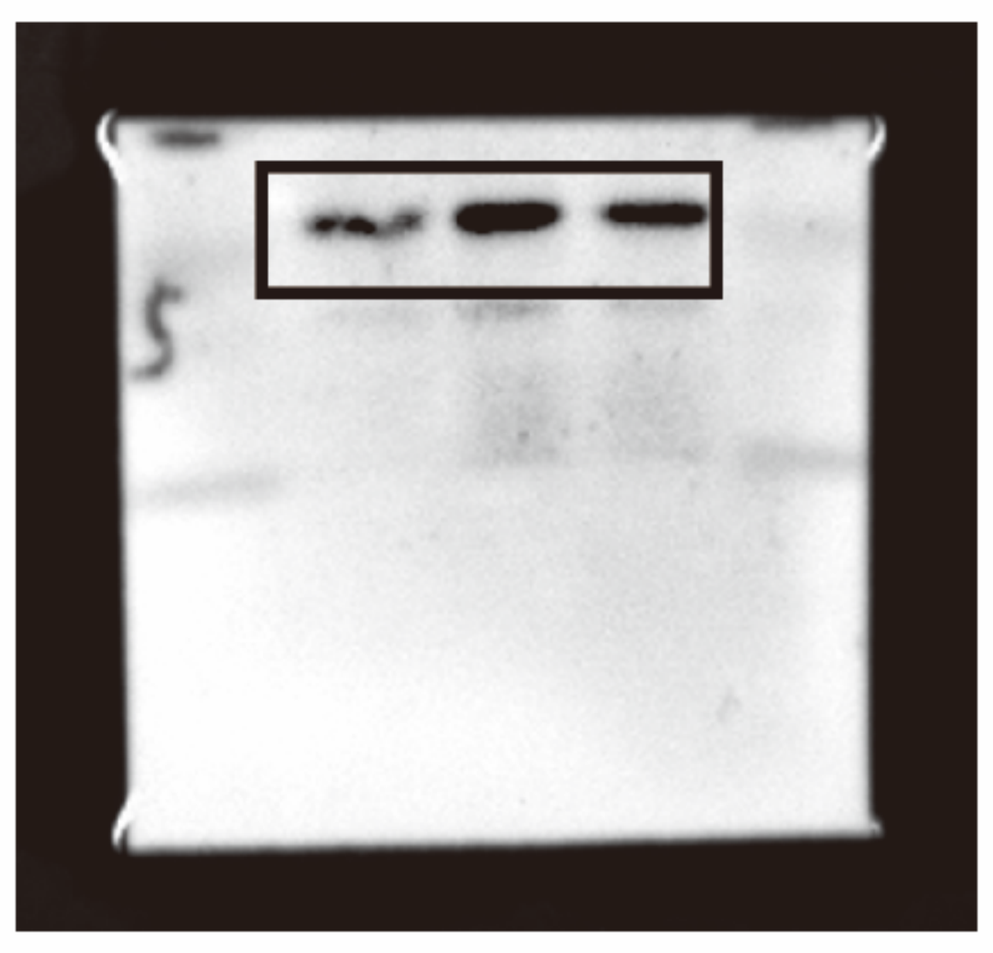

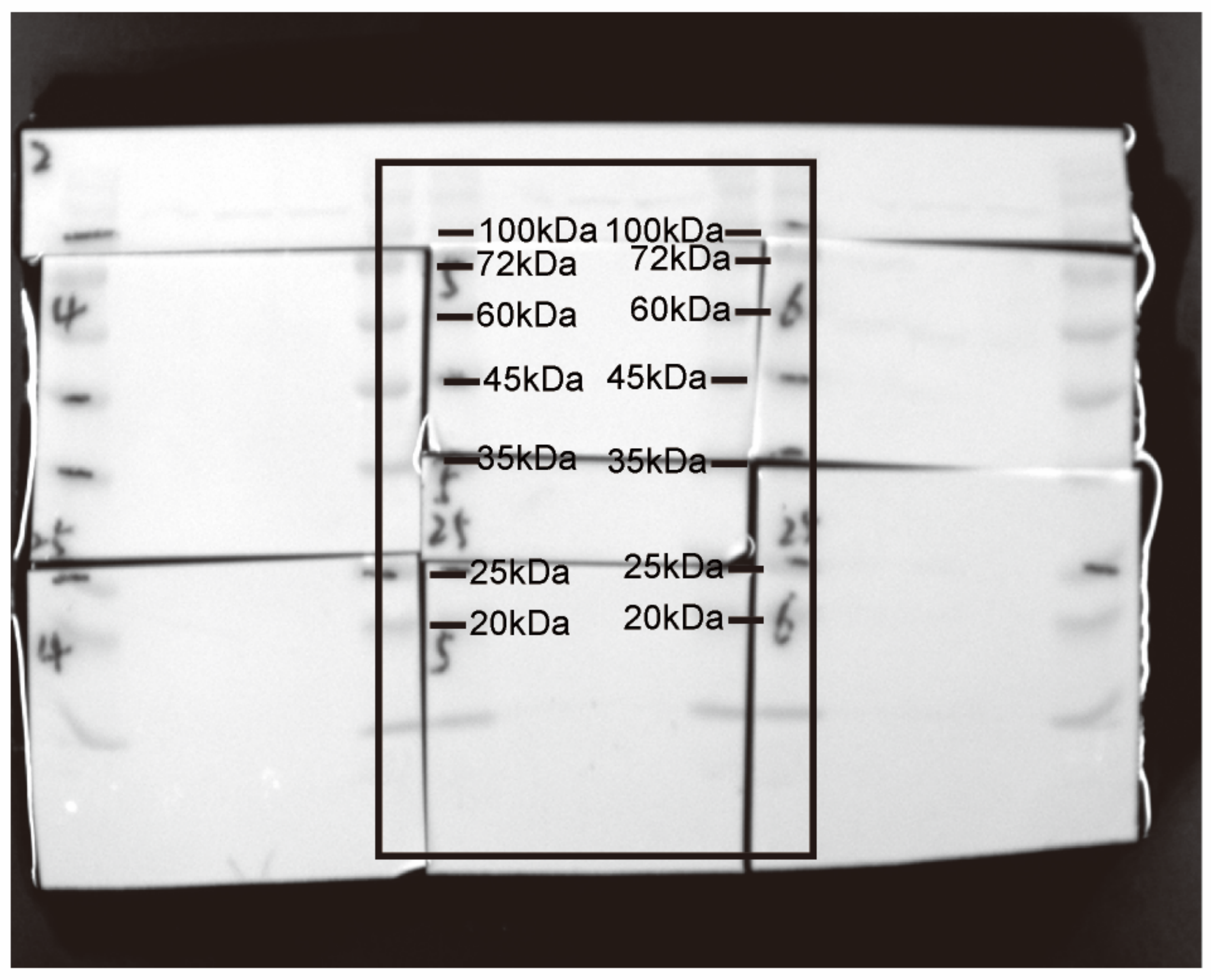

Fig.S4E

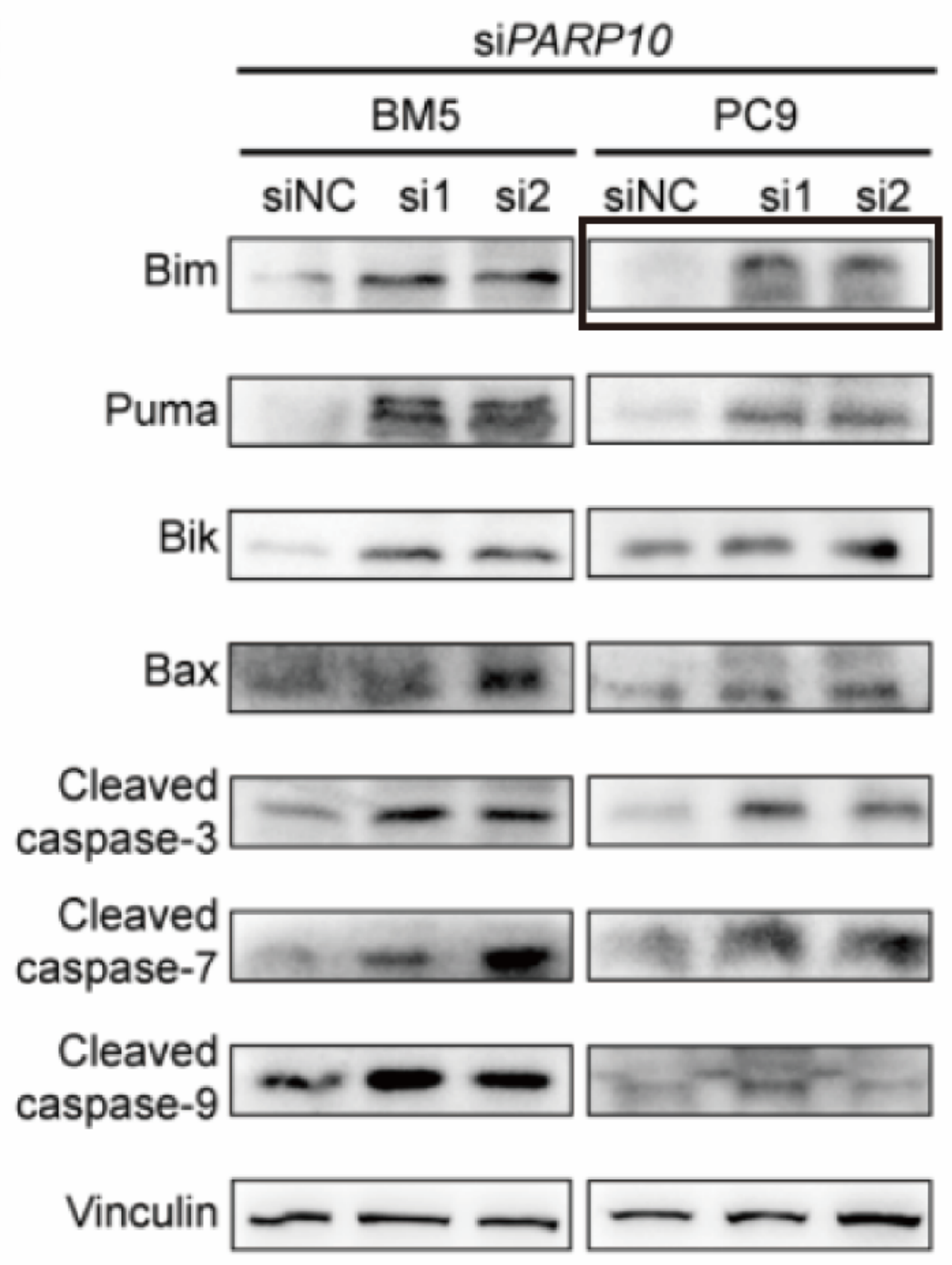

Vinculin

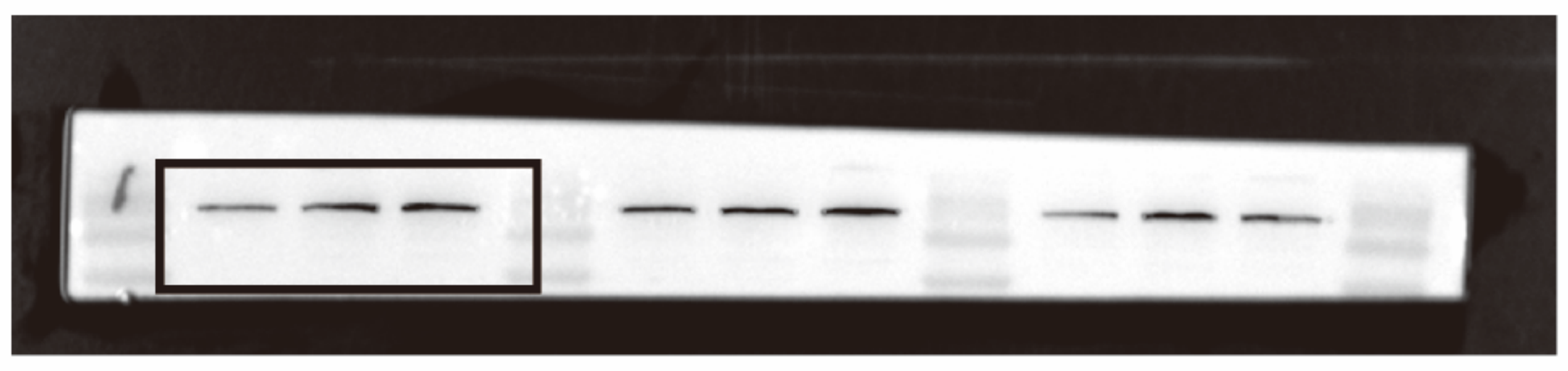

Bim

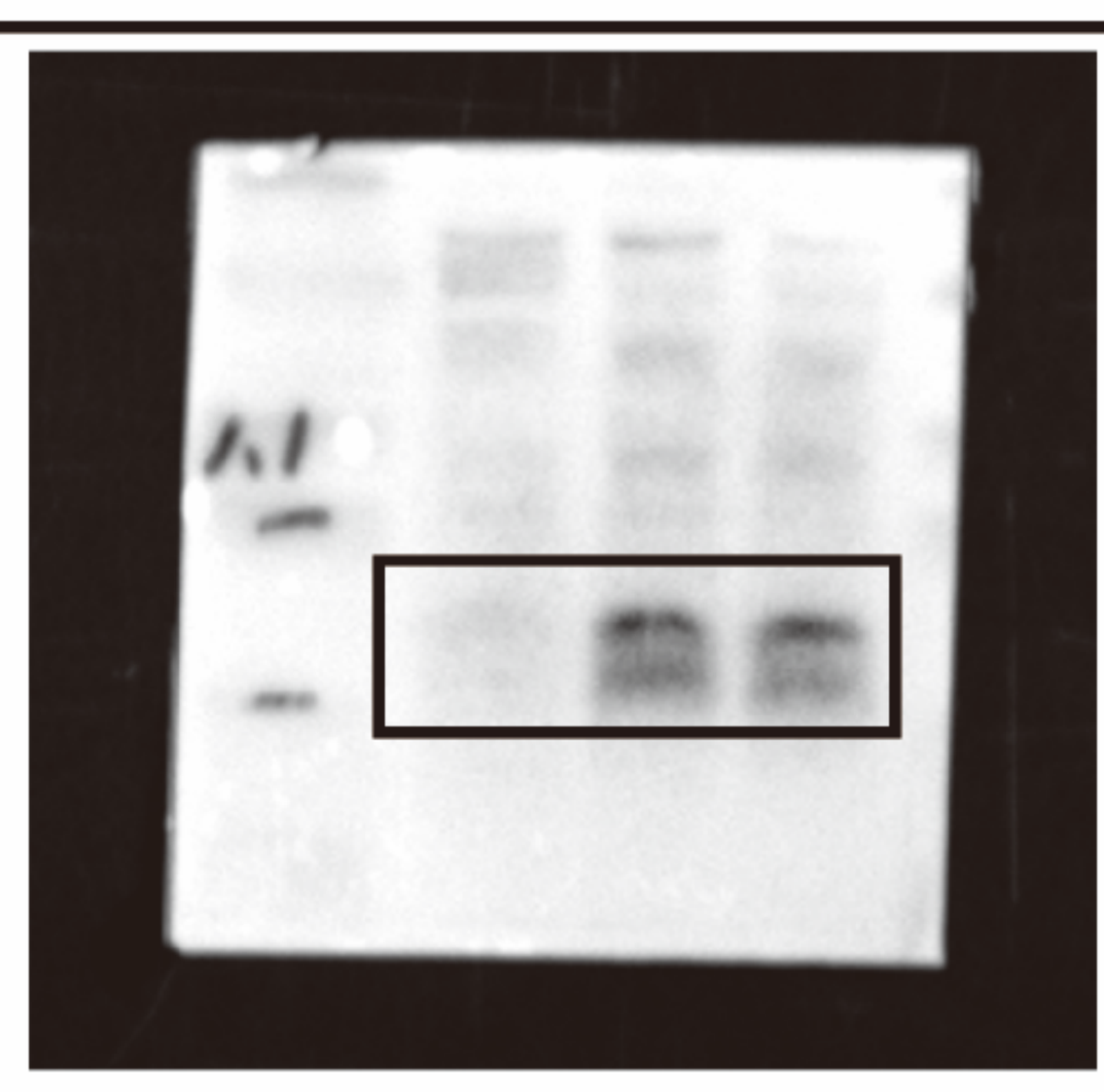

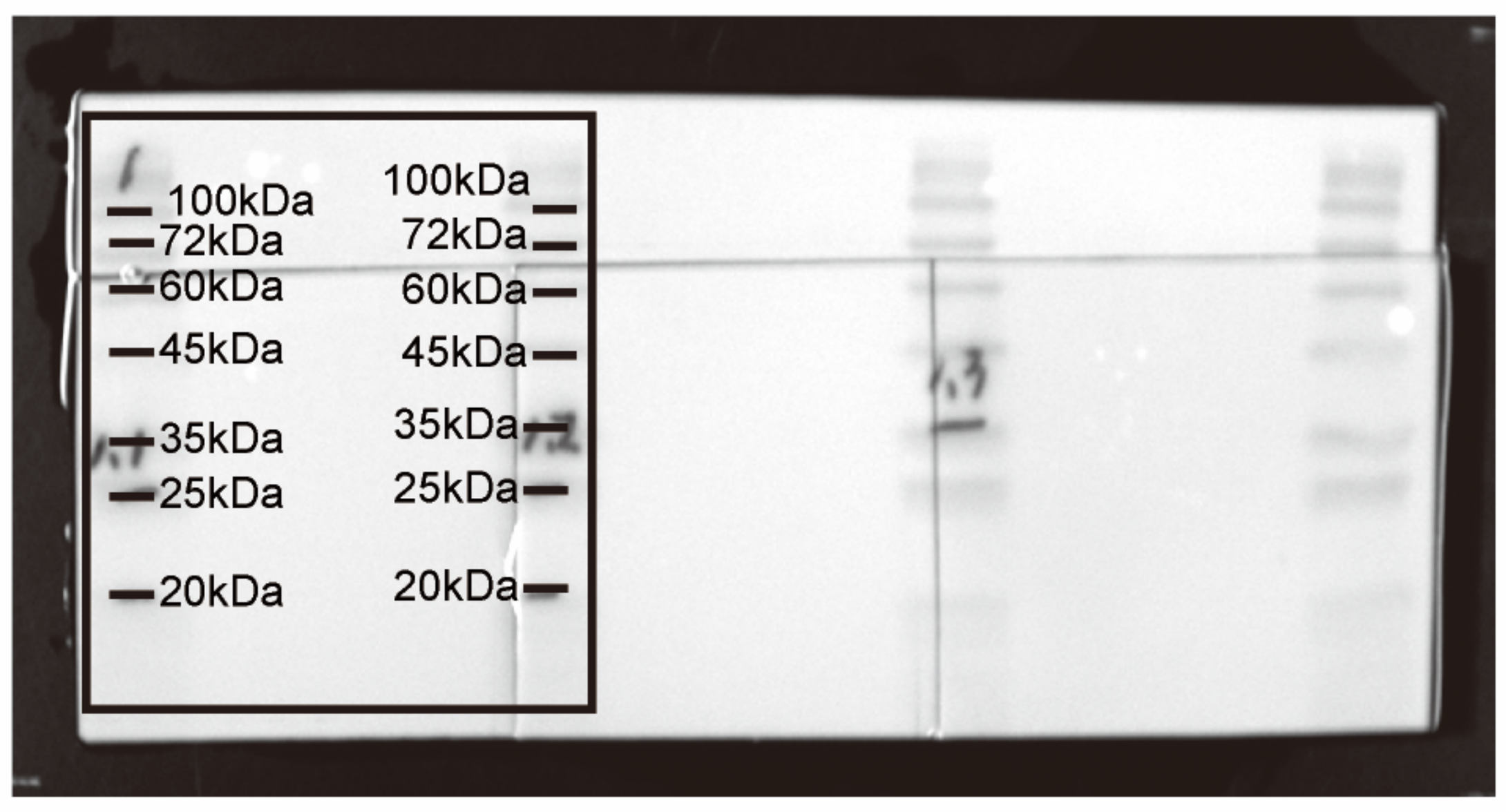

Fig.S4E

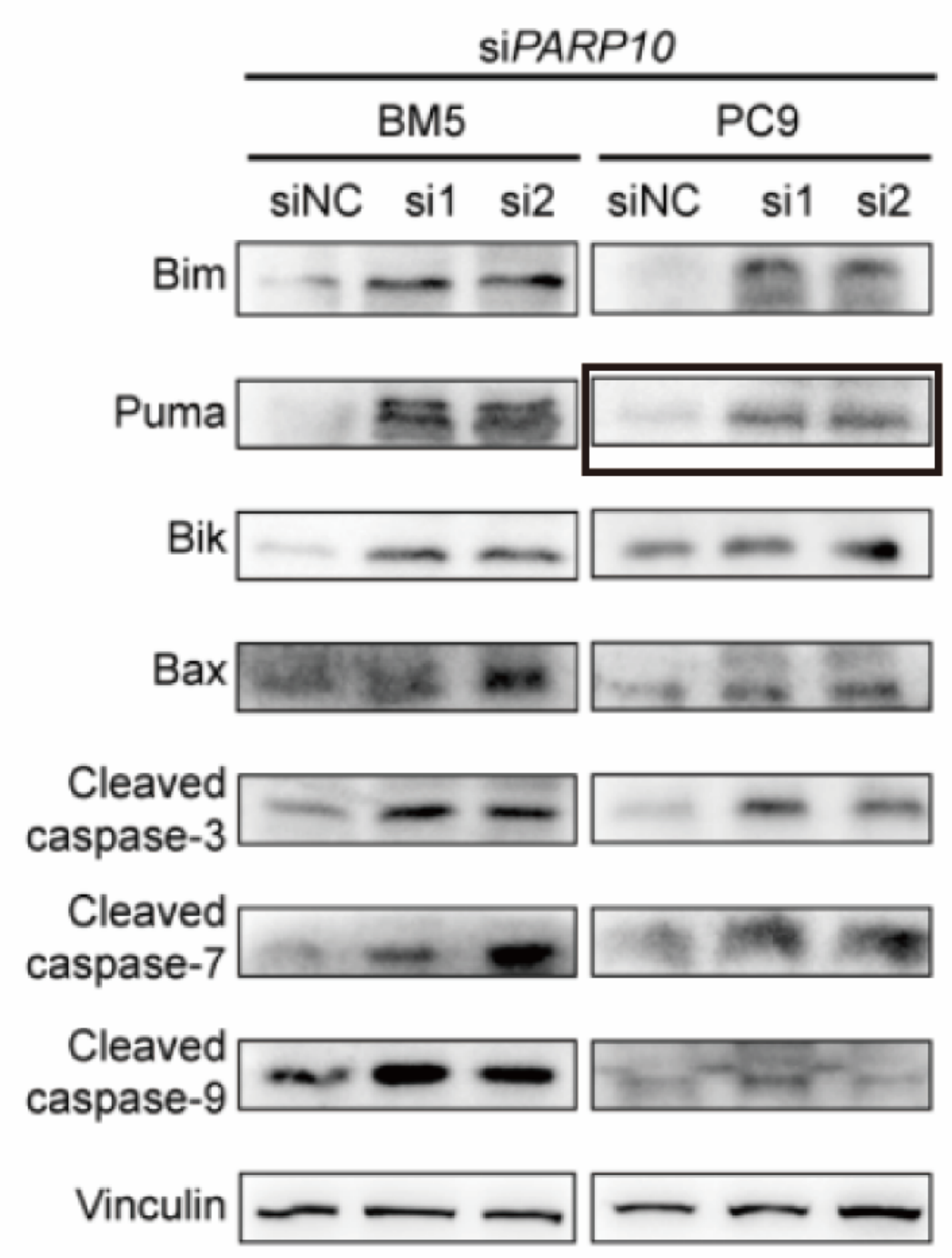

Vinculin

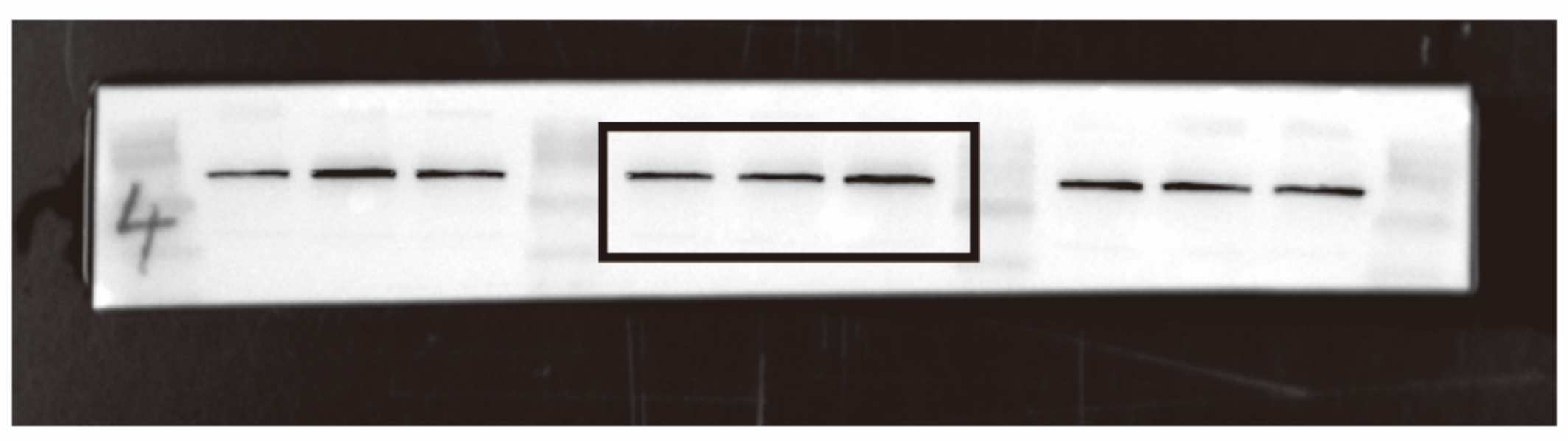

Puma

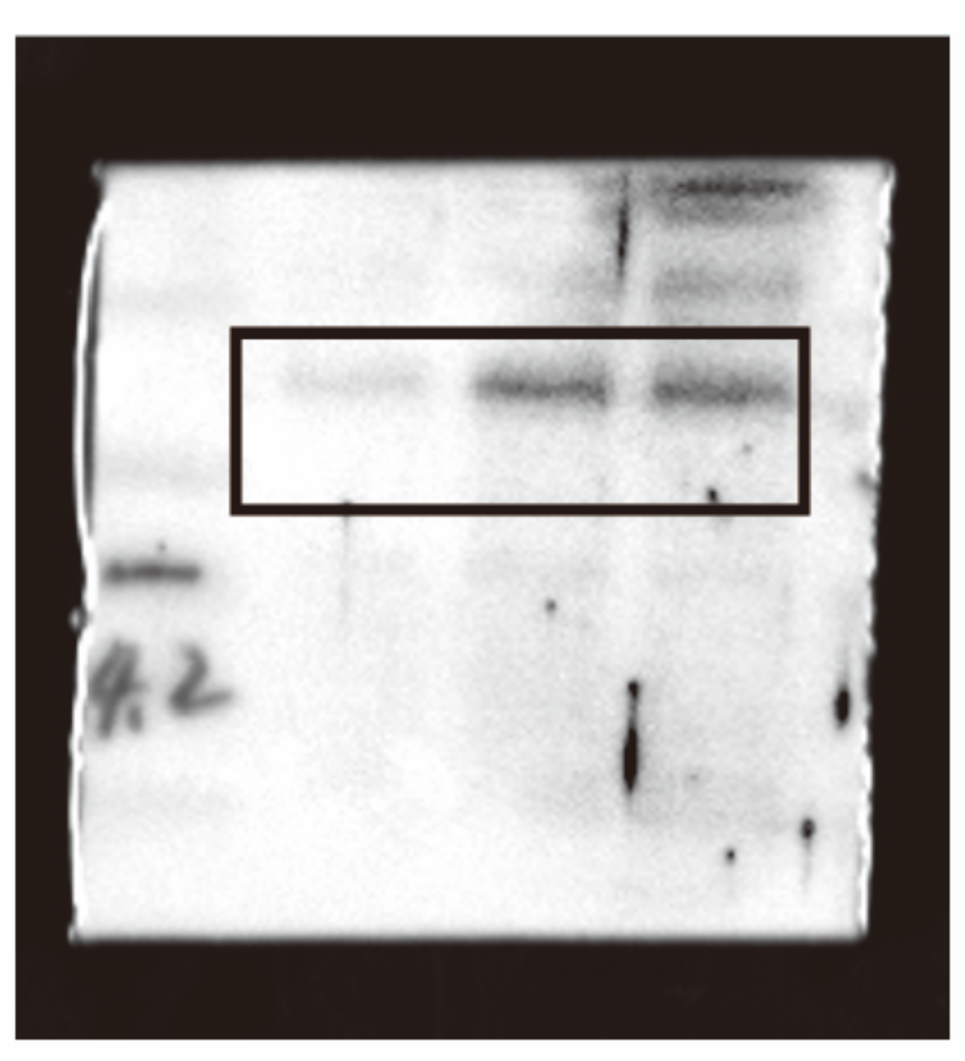

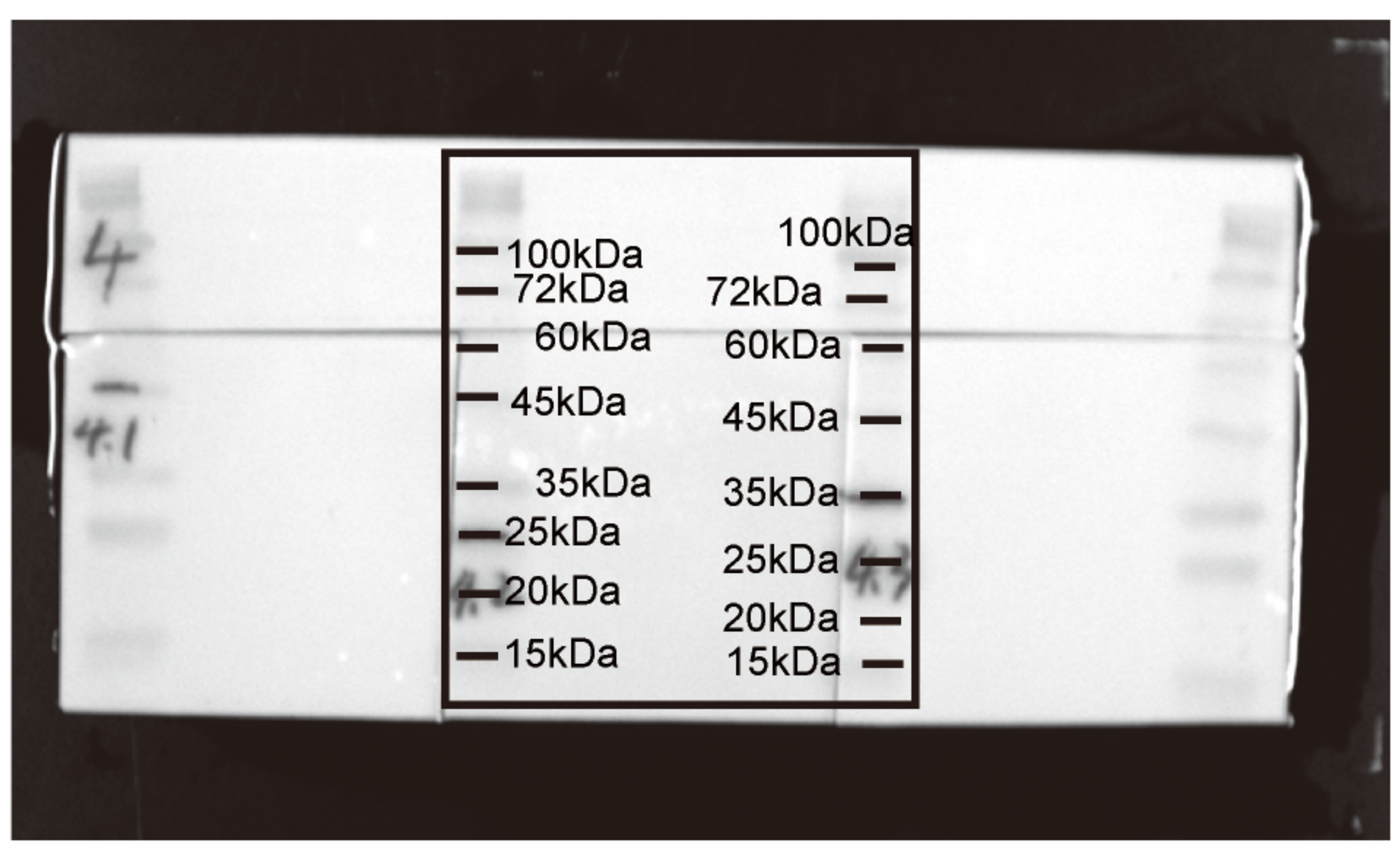

Figure	Merged	Stiched full image

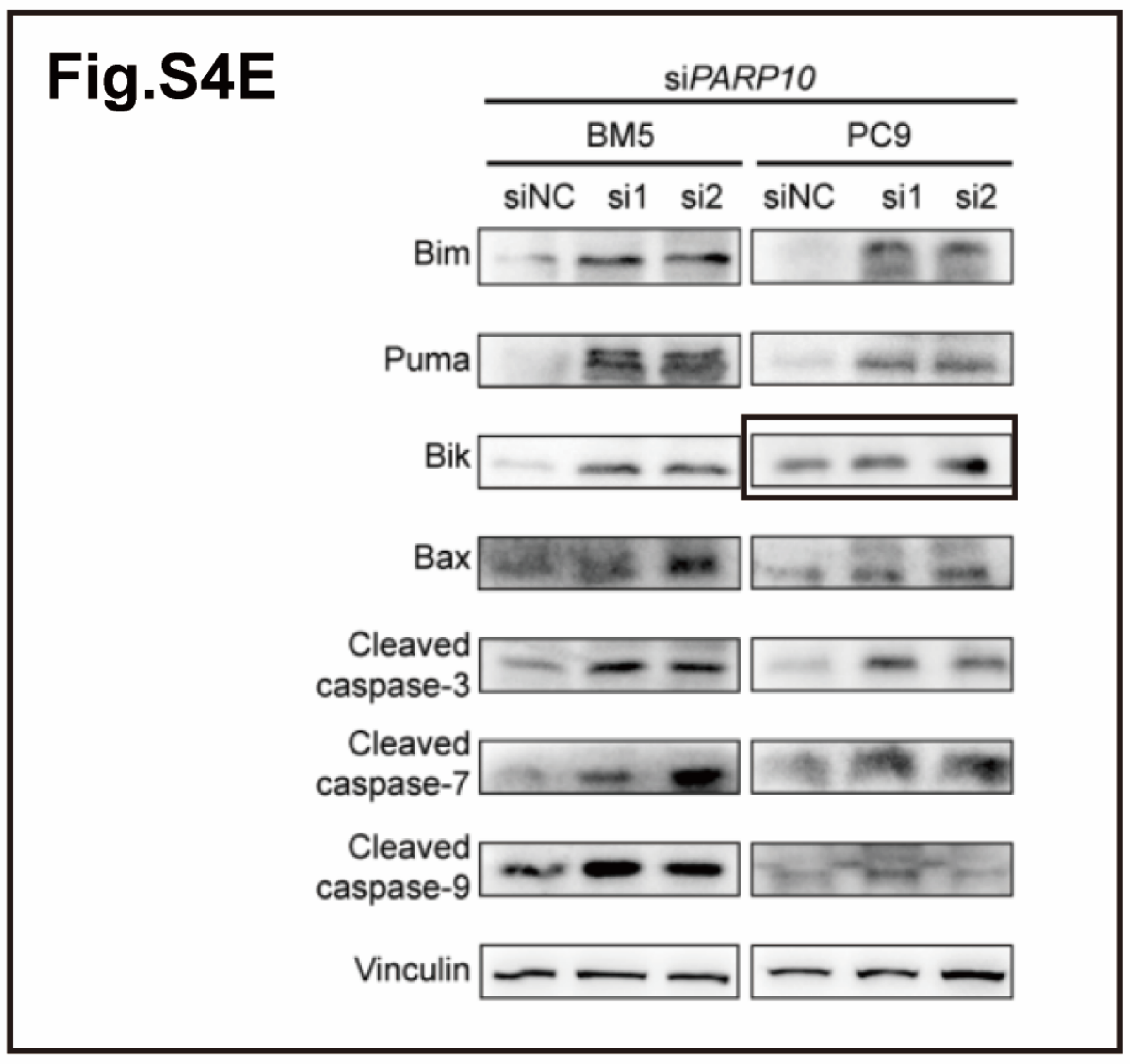

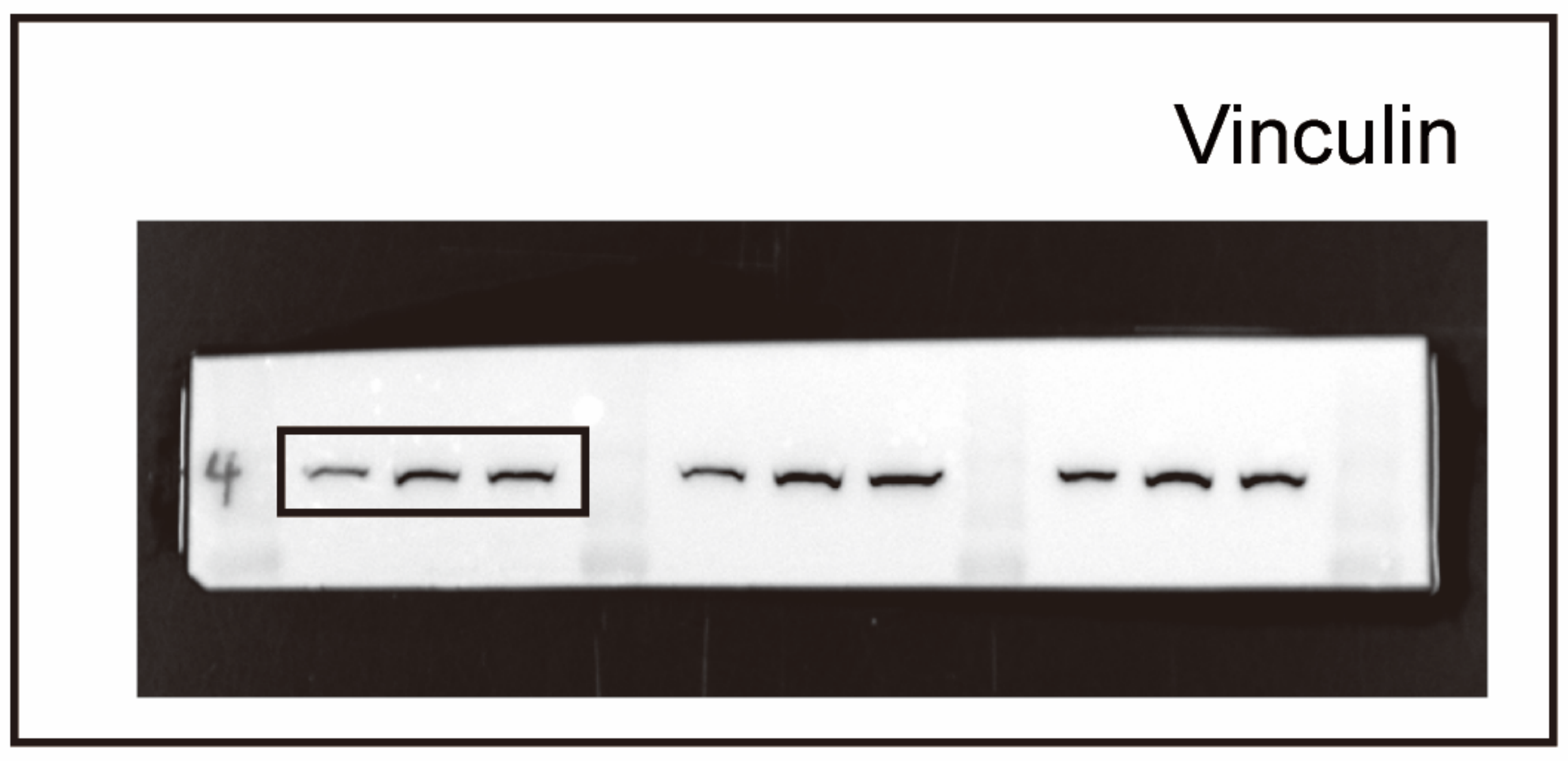

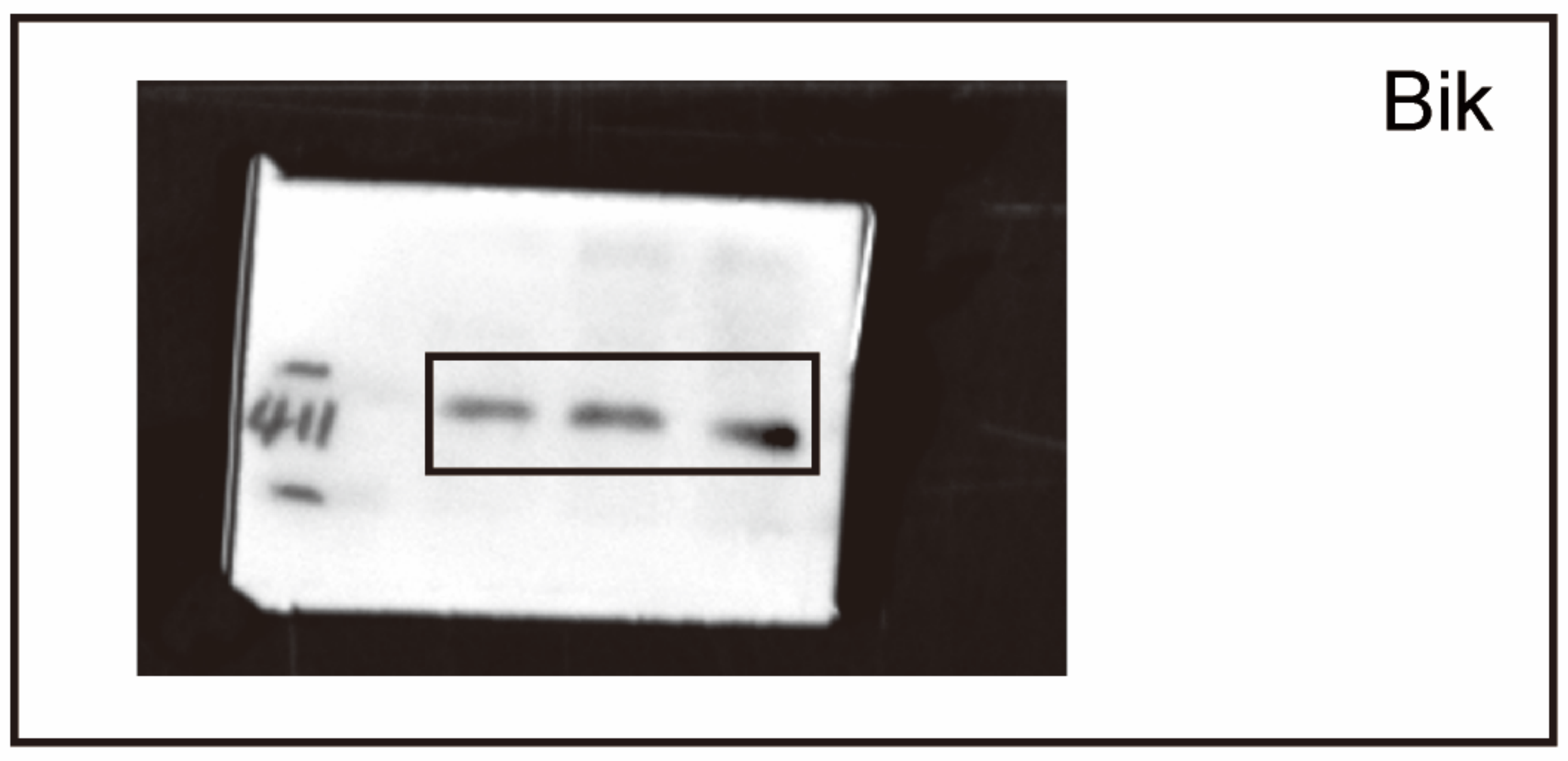

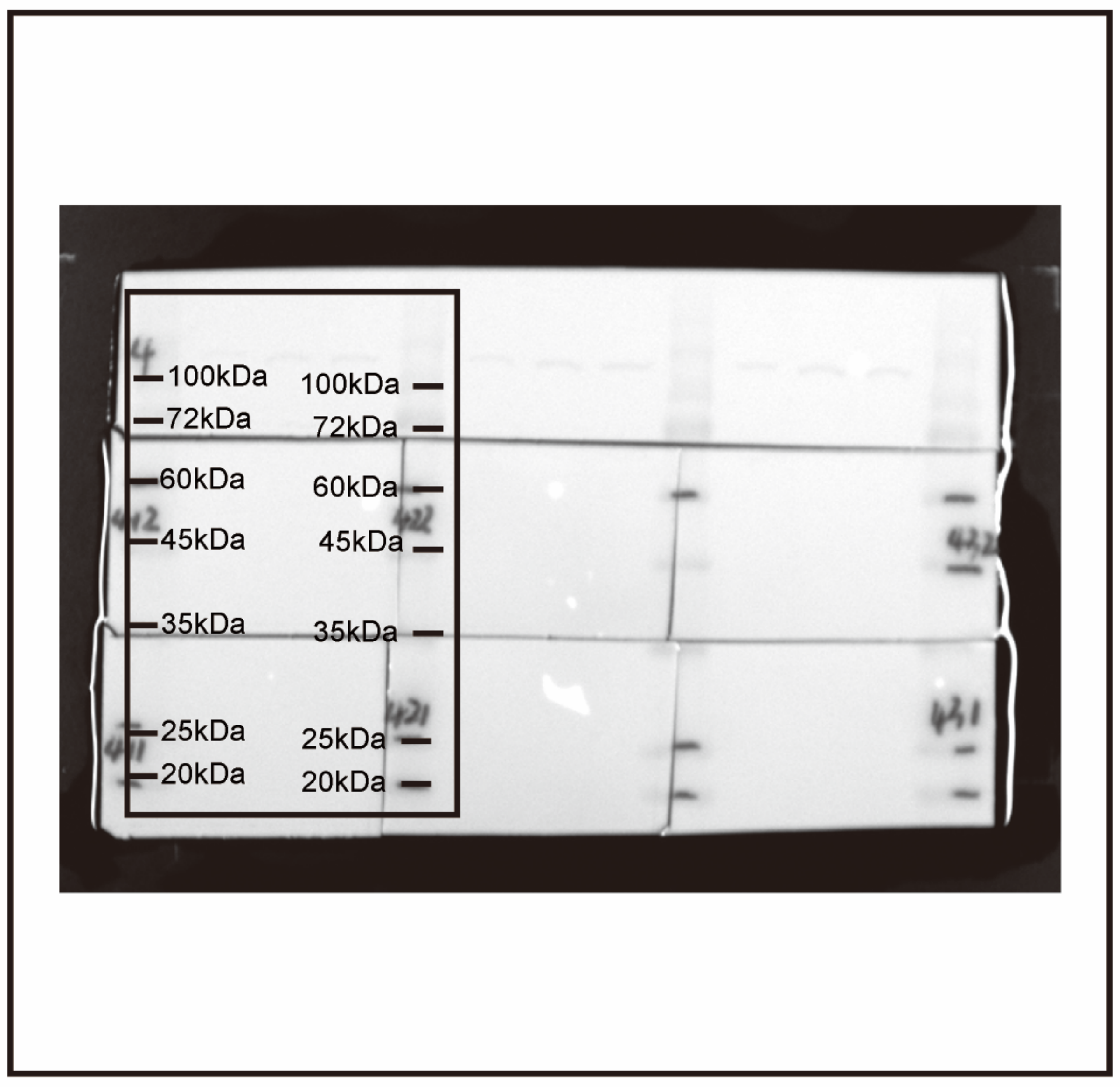

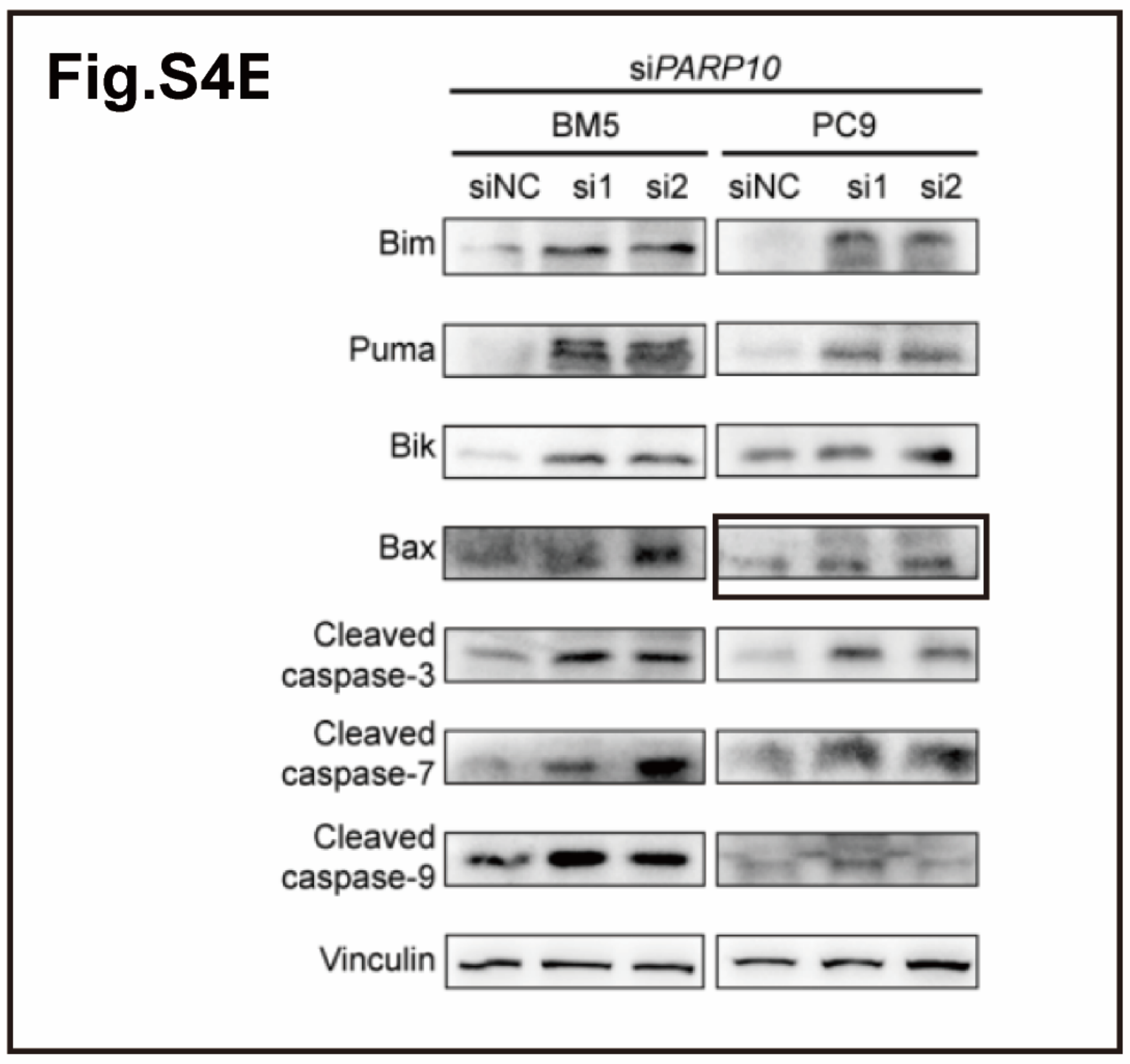

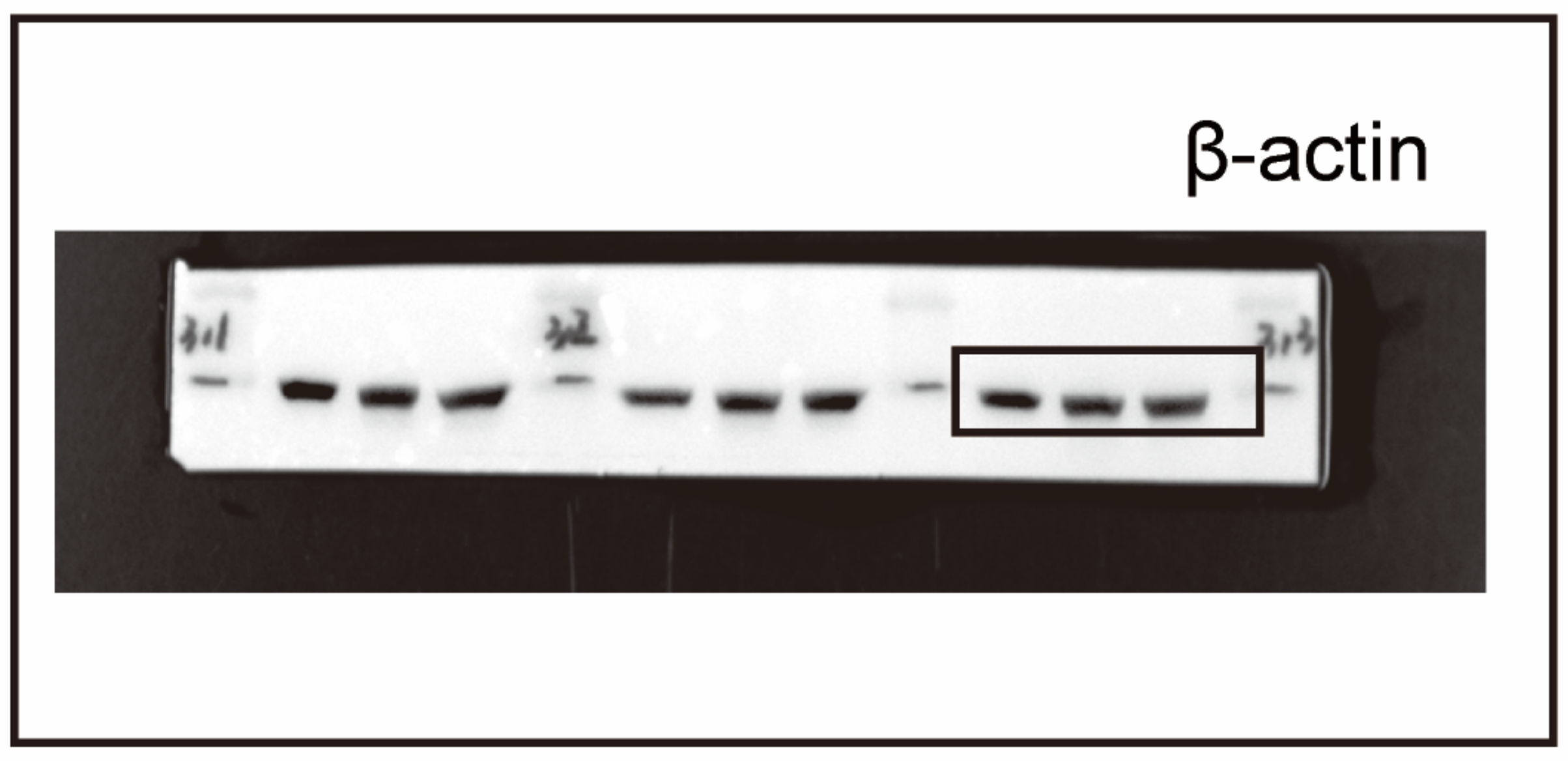

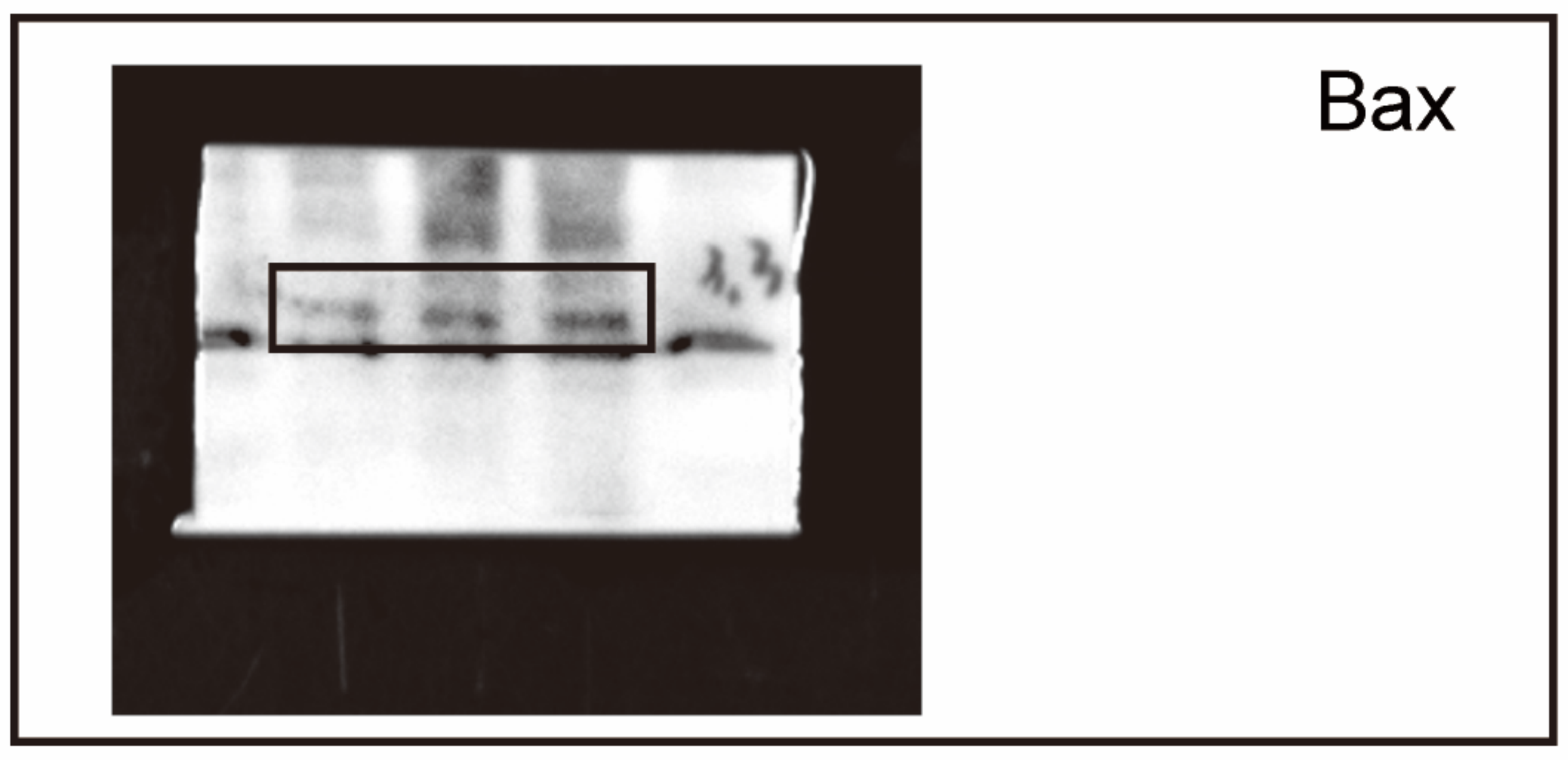

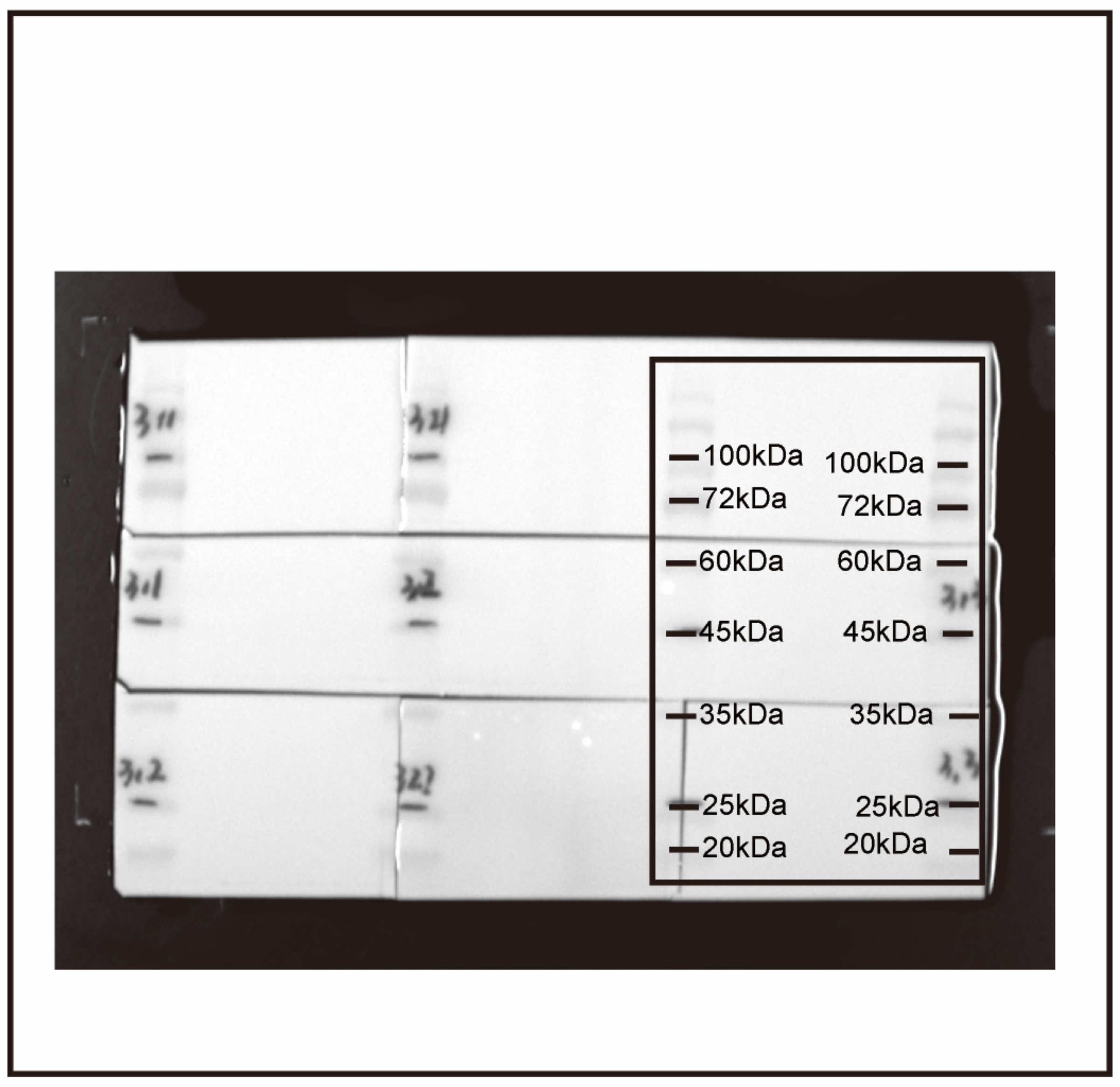

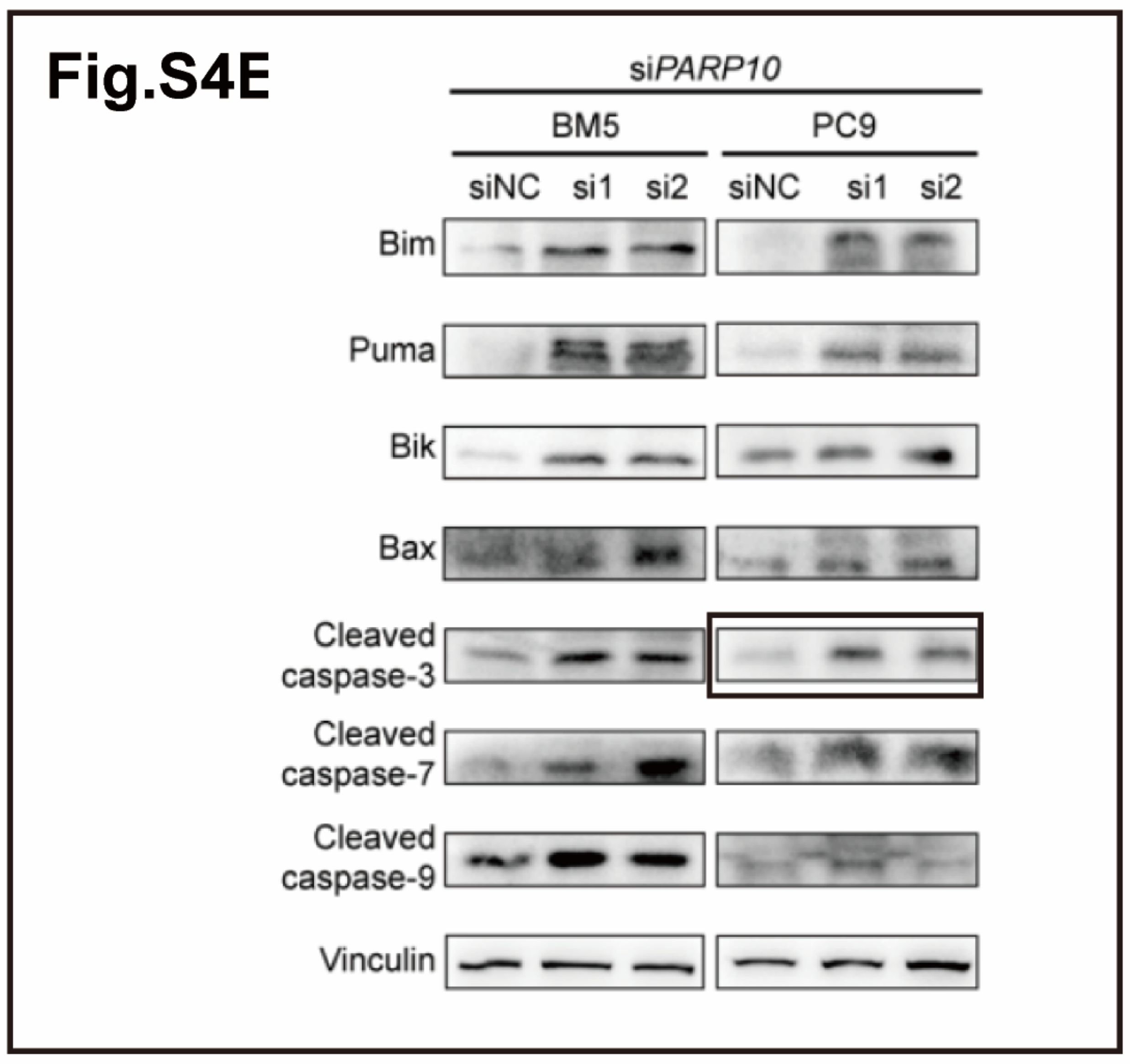

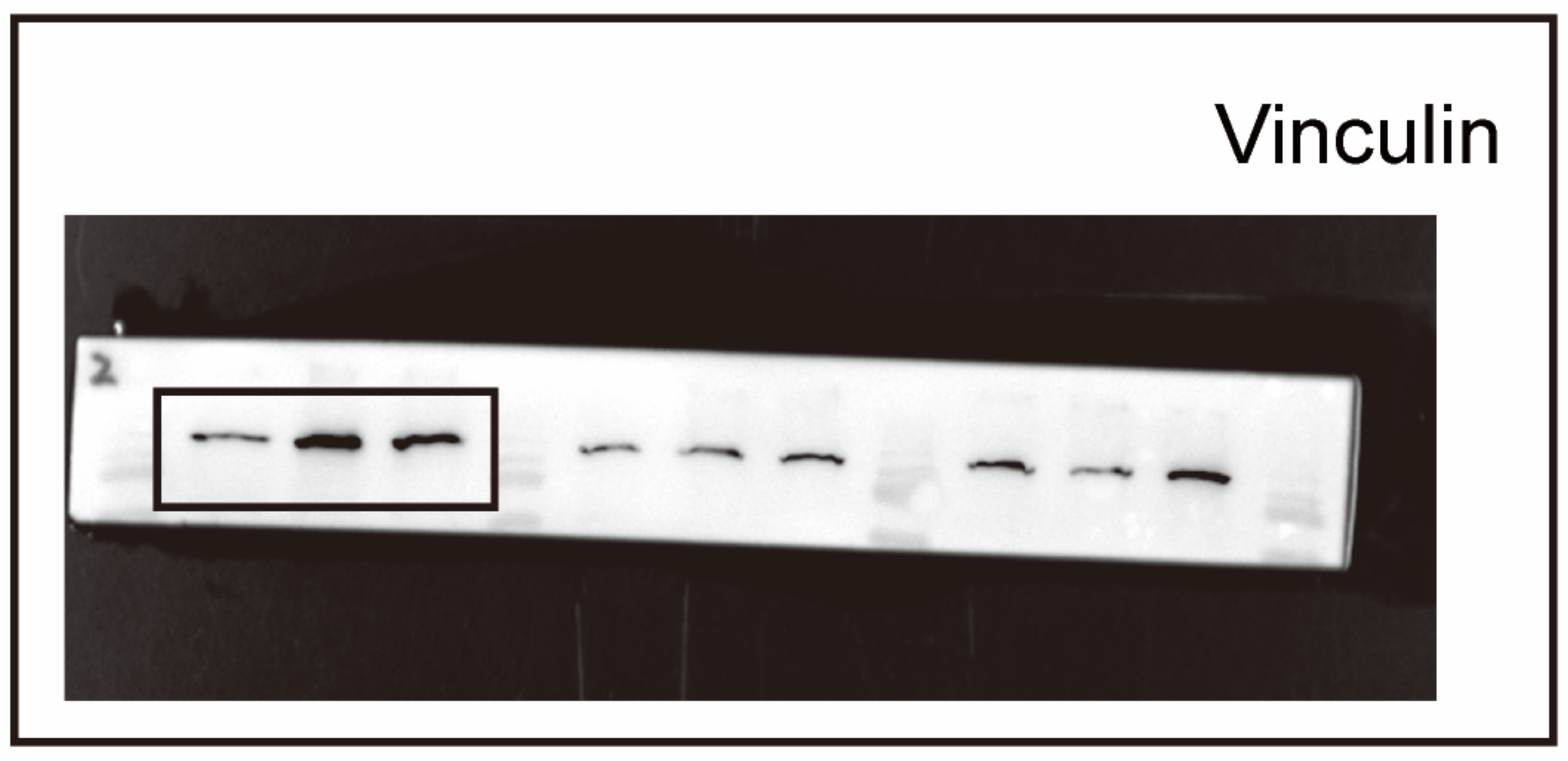

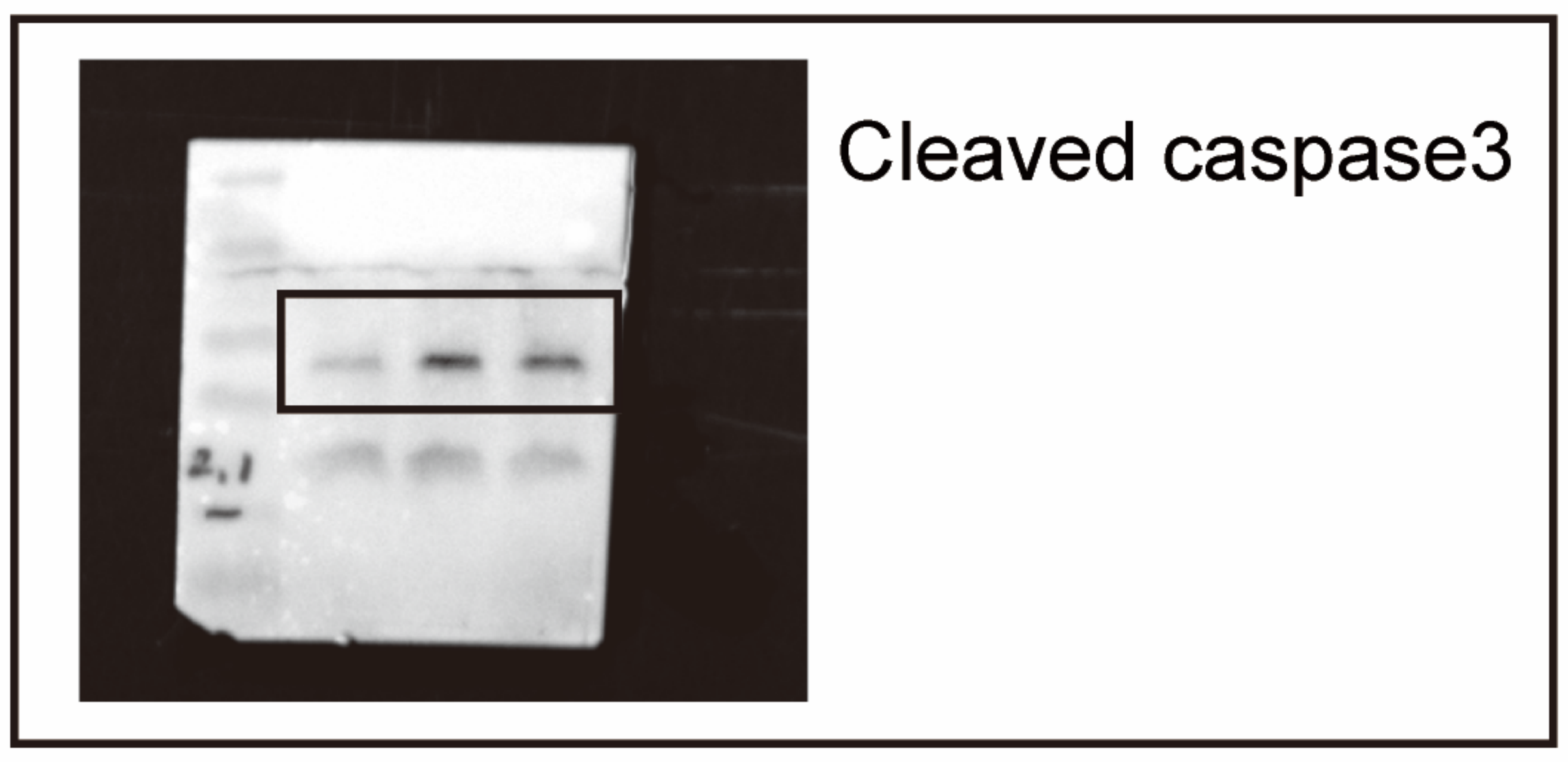

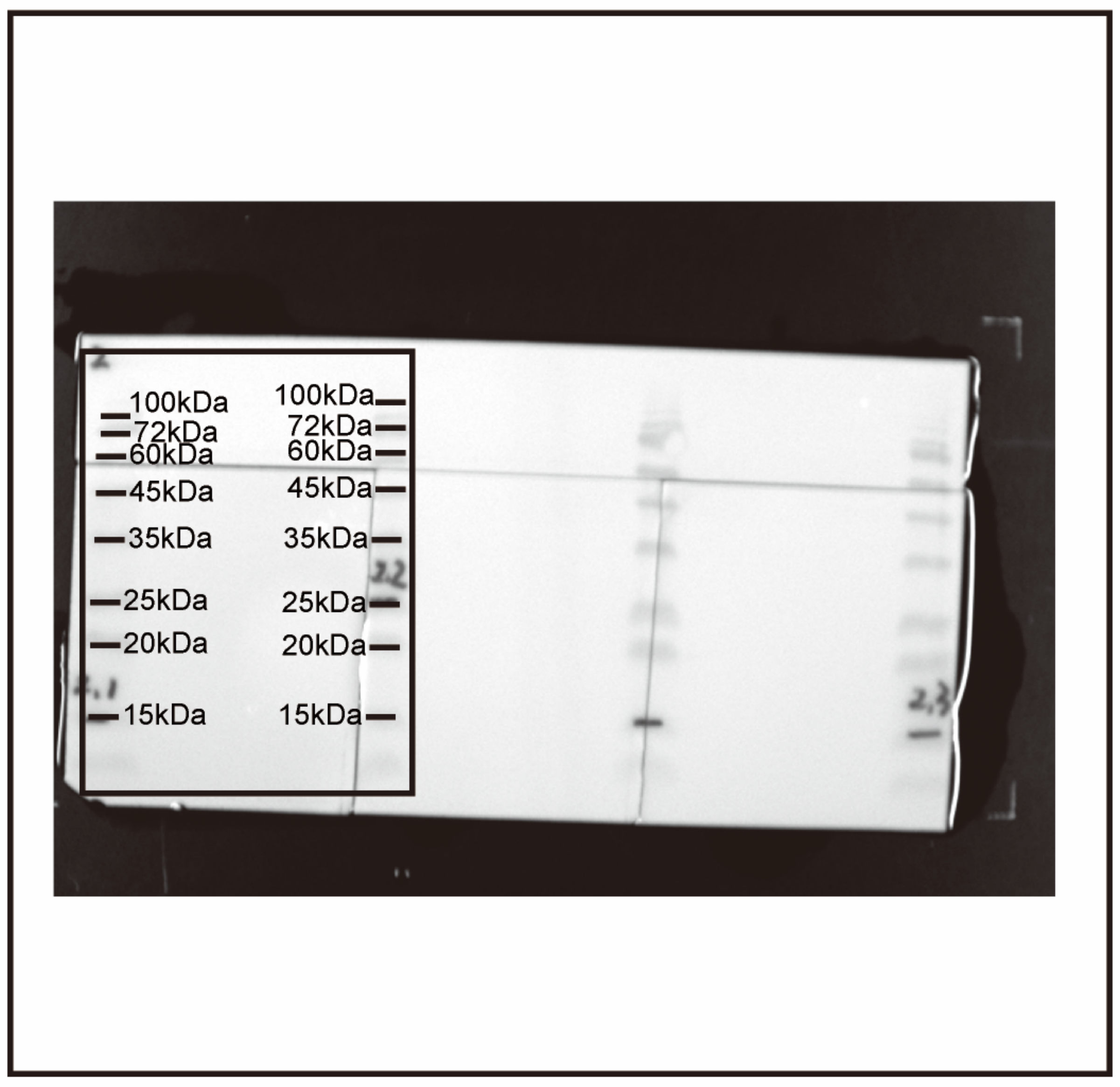

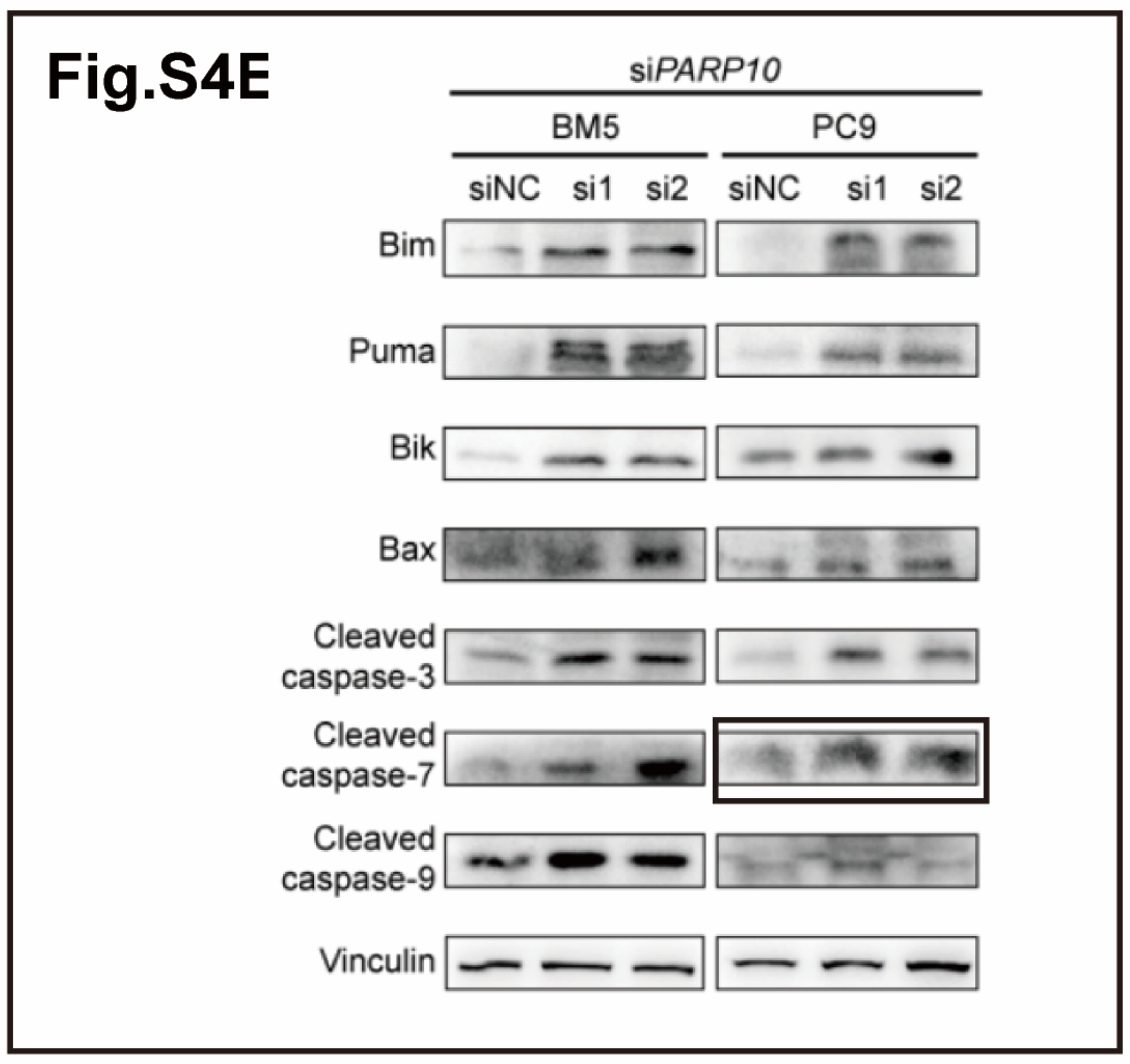

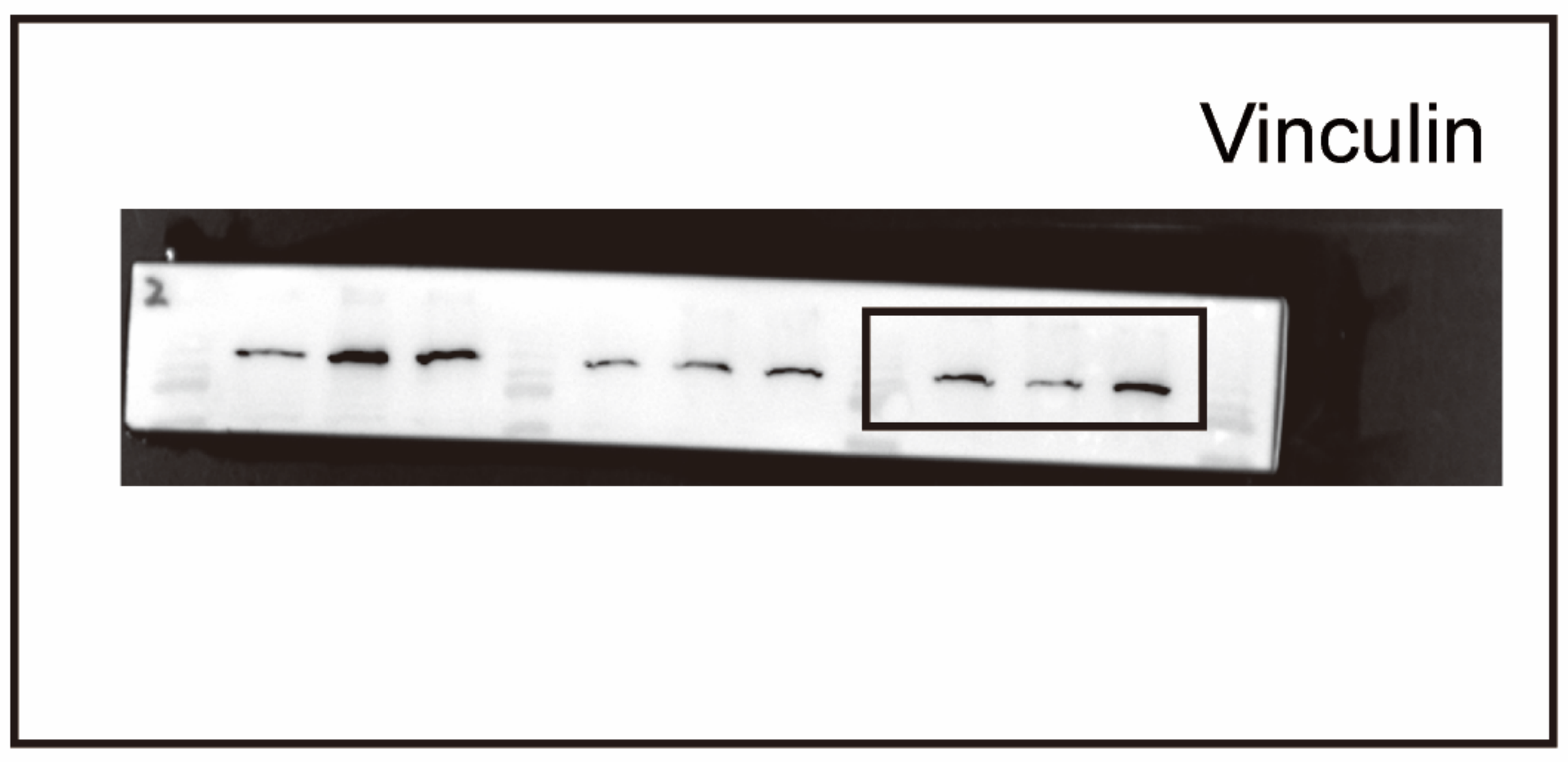

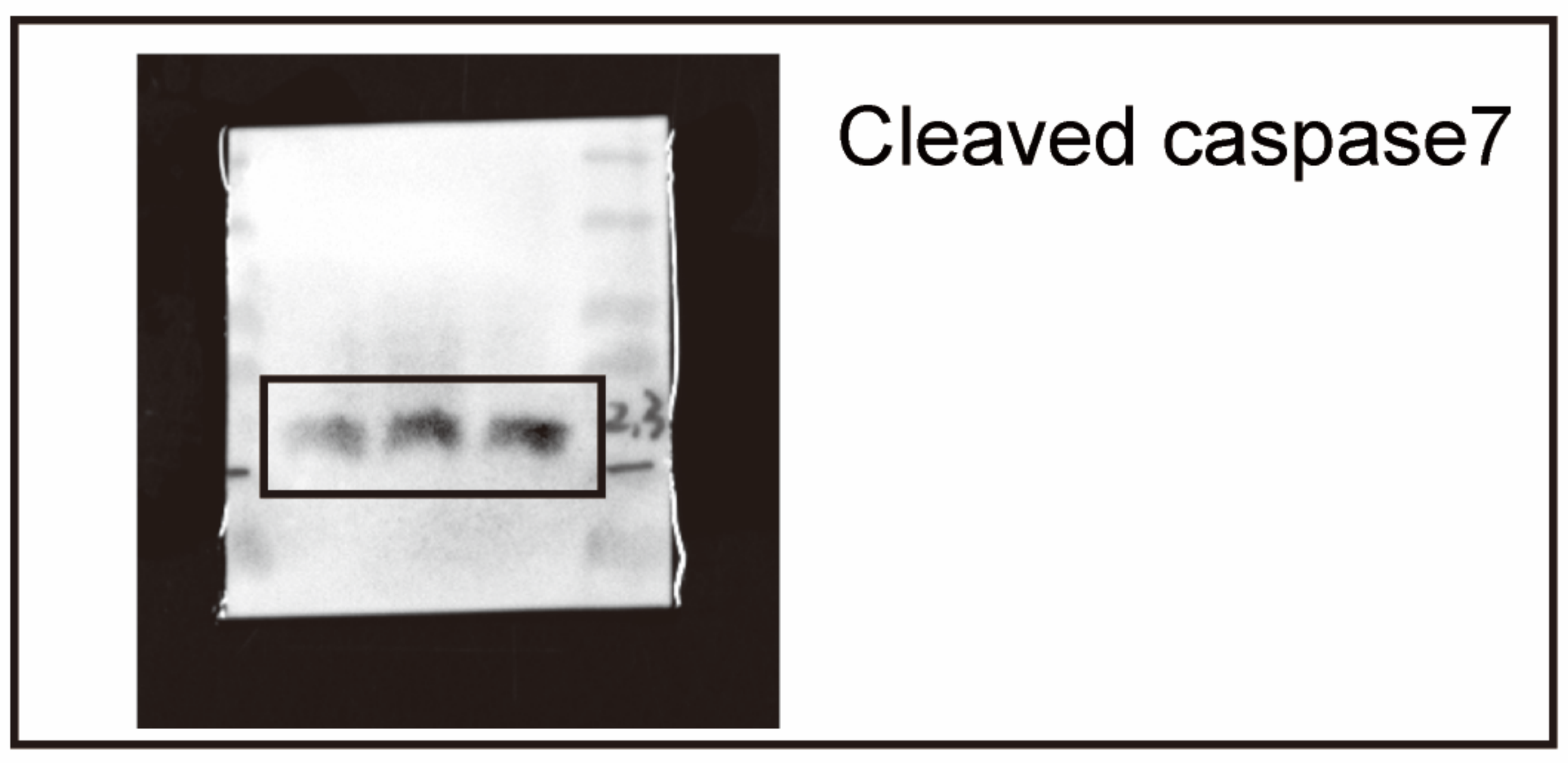

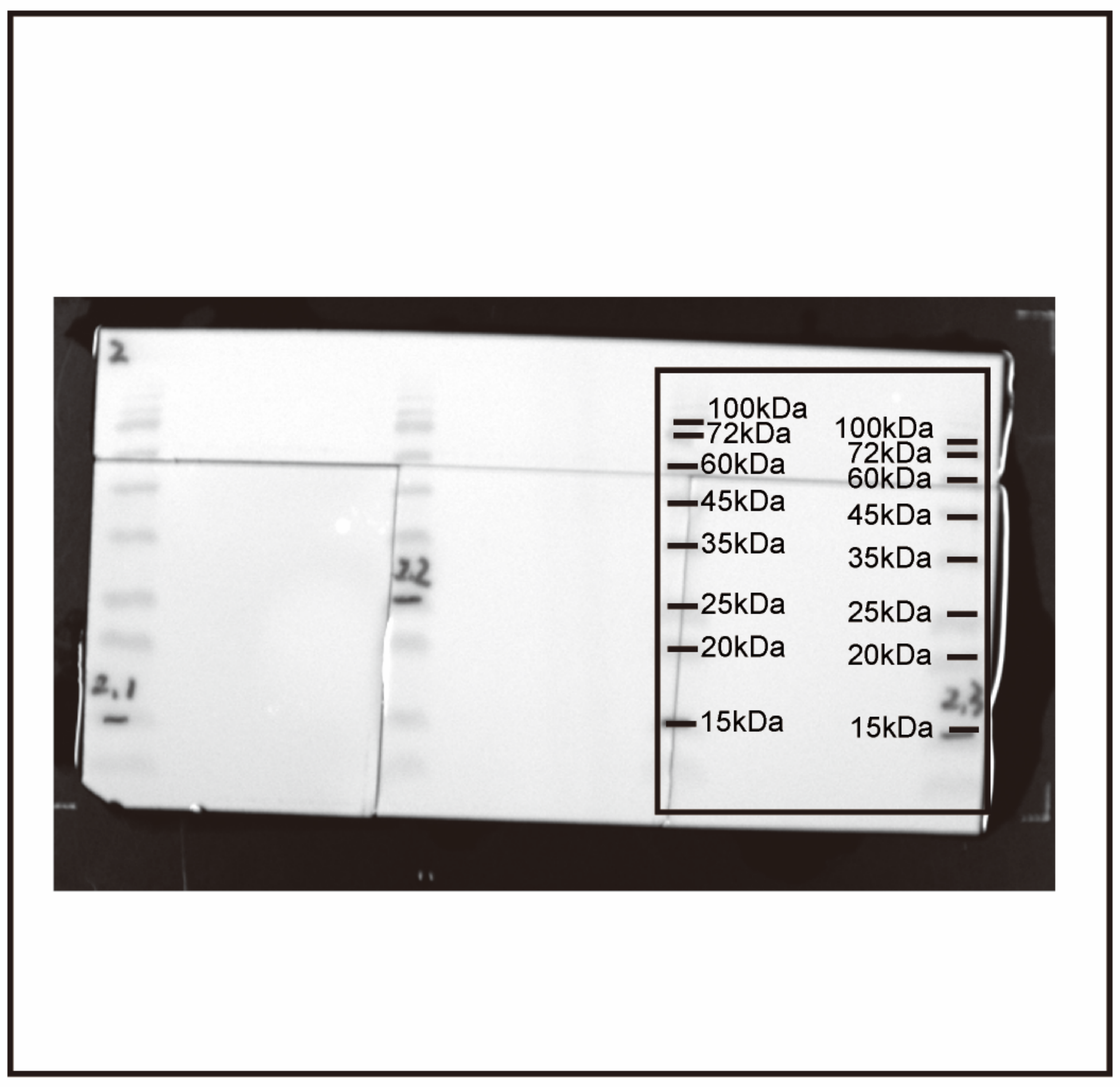

Figure	Merged	Stiched full image

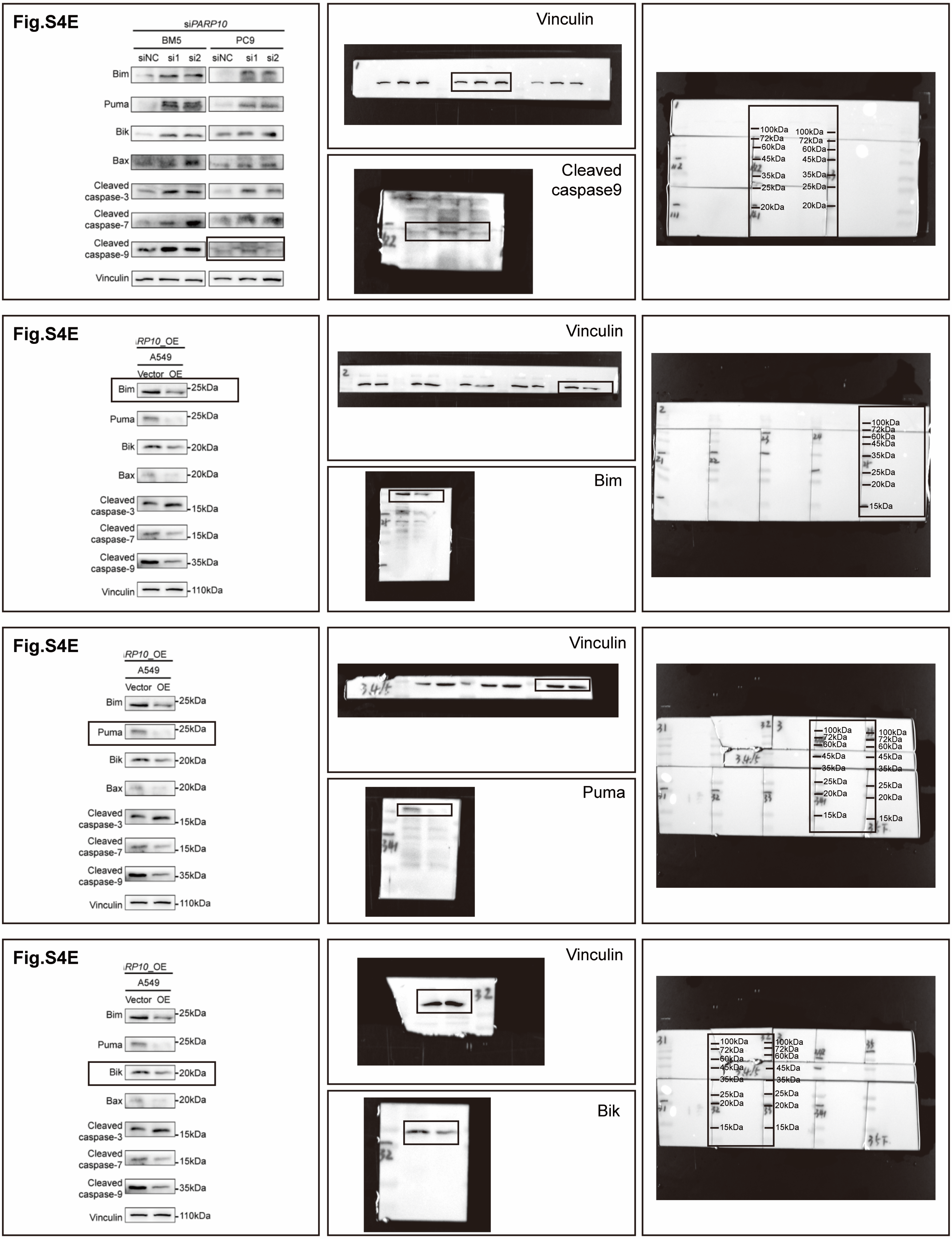

Figure	Merged	Stiched full image

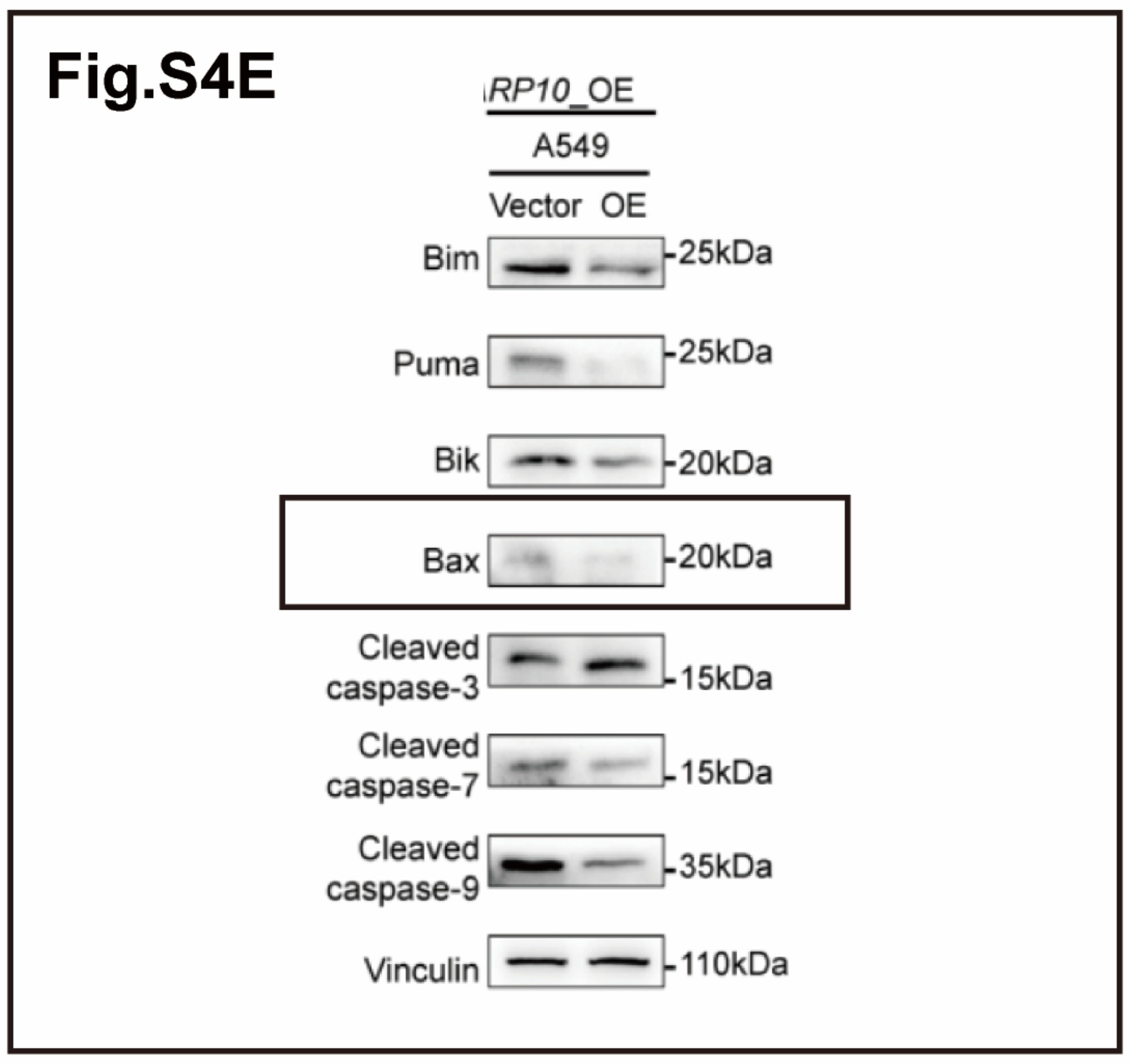

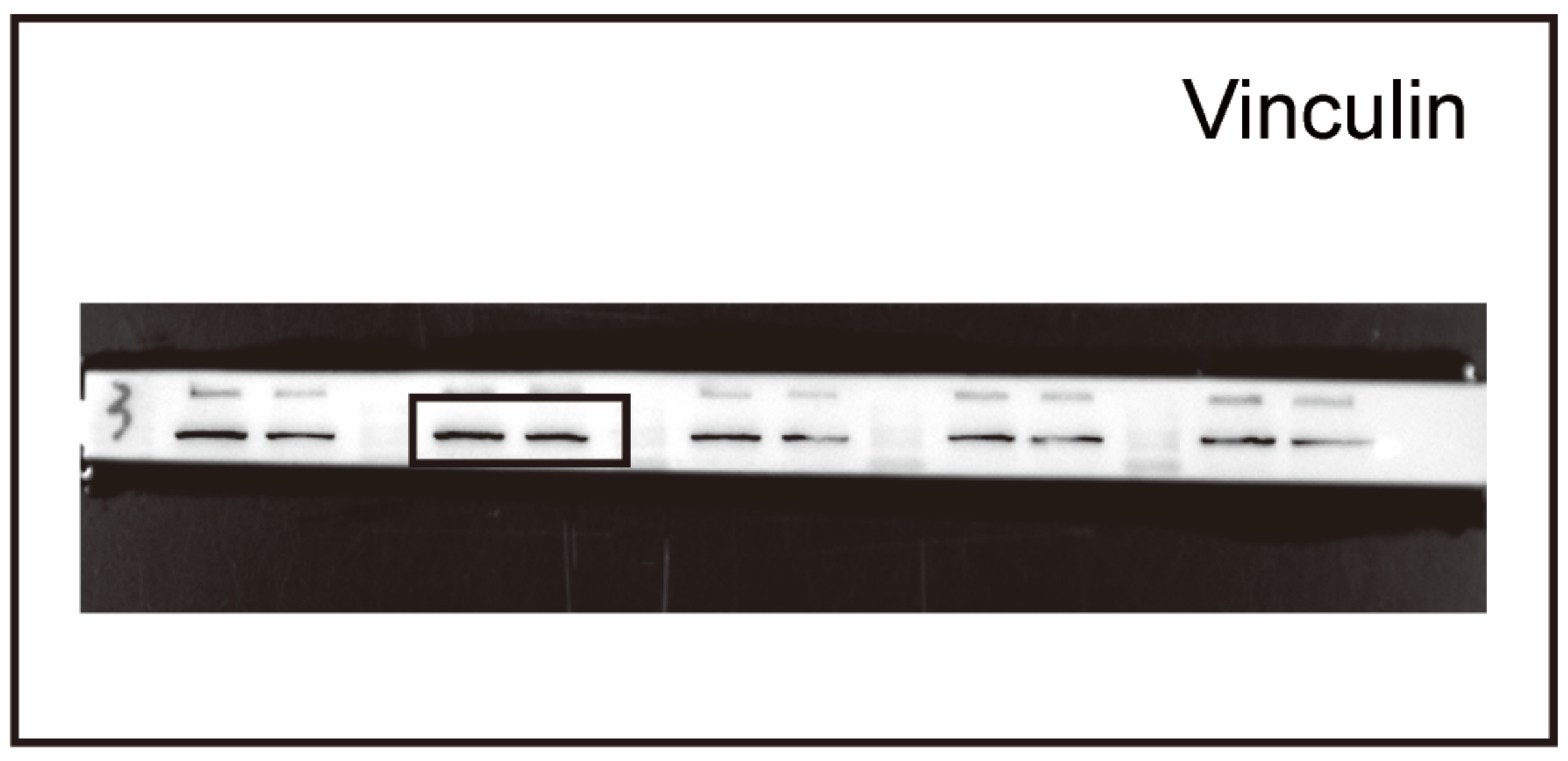

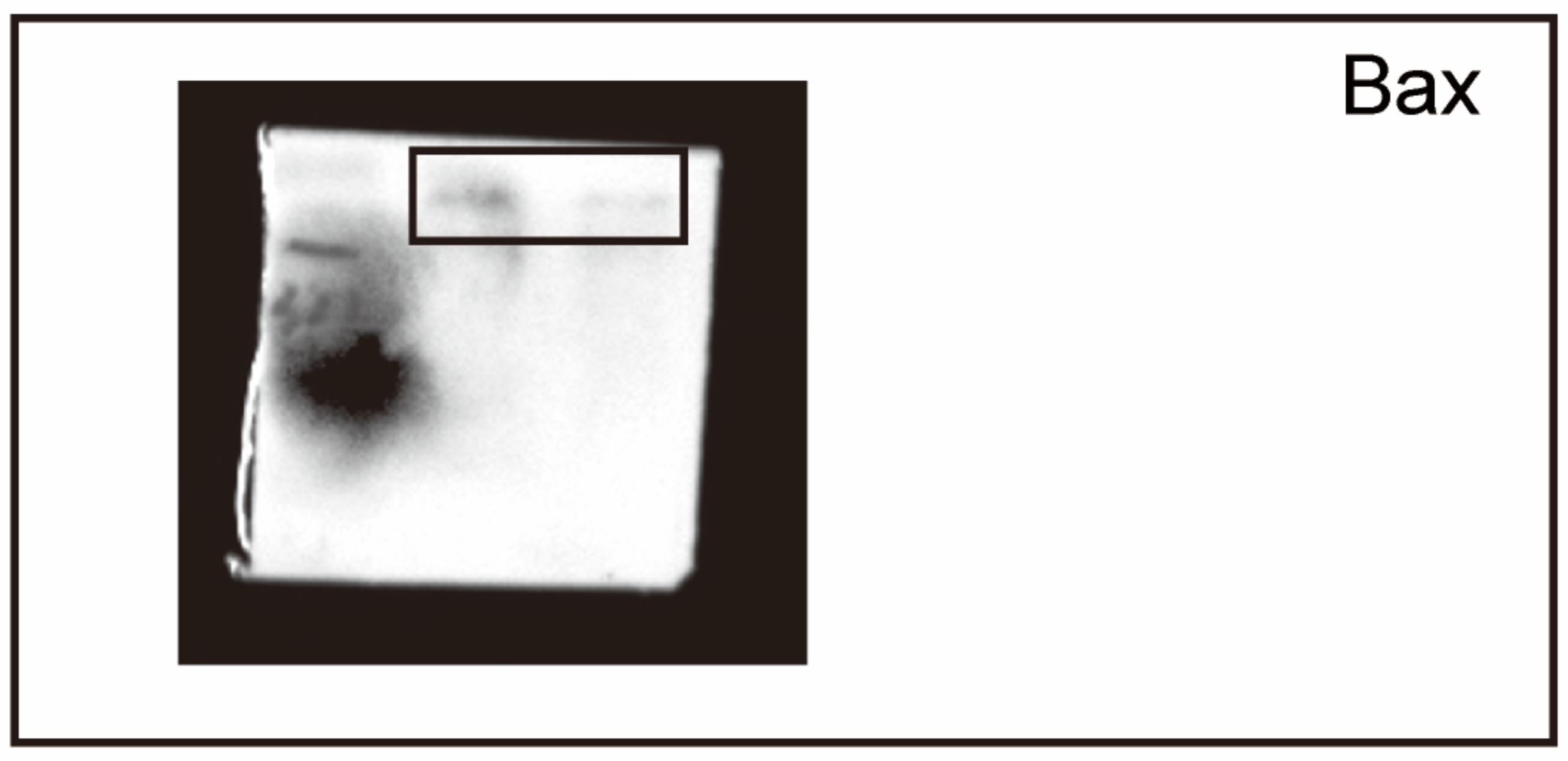

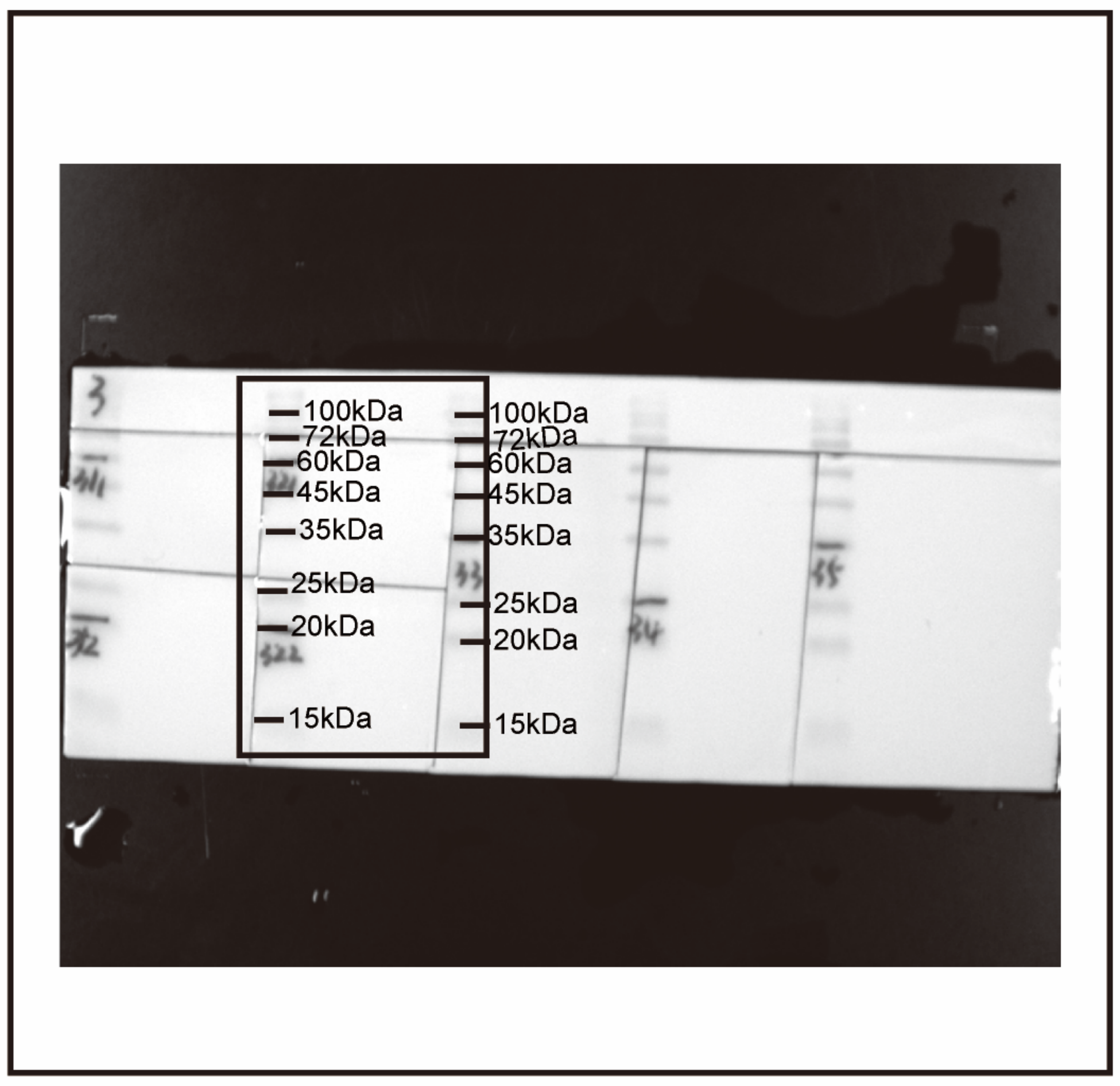

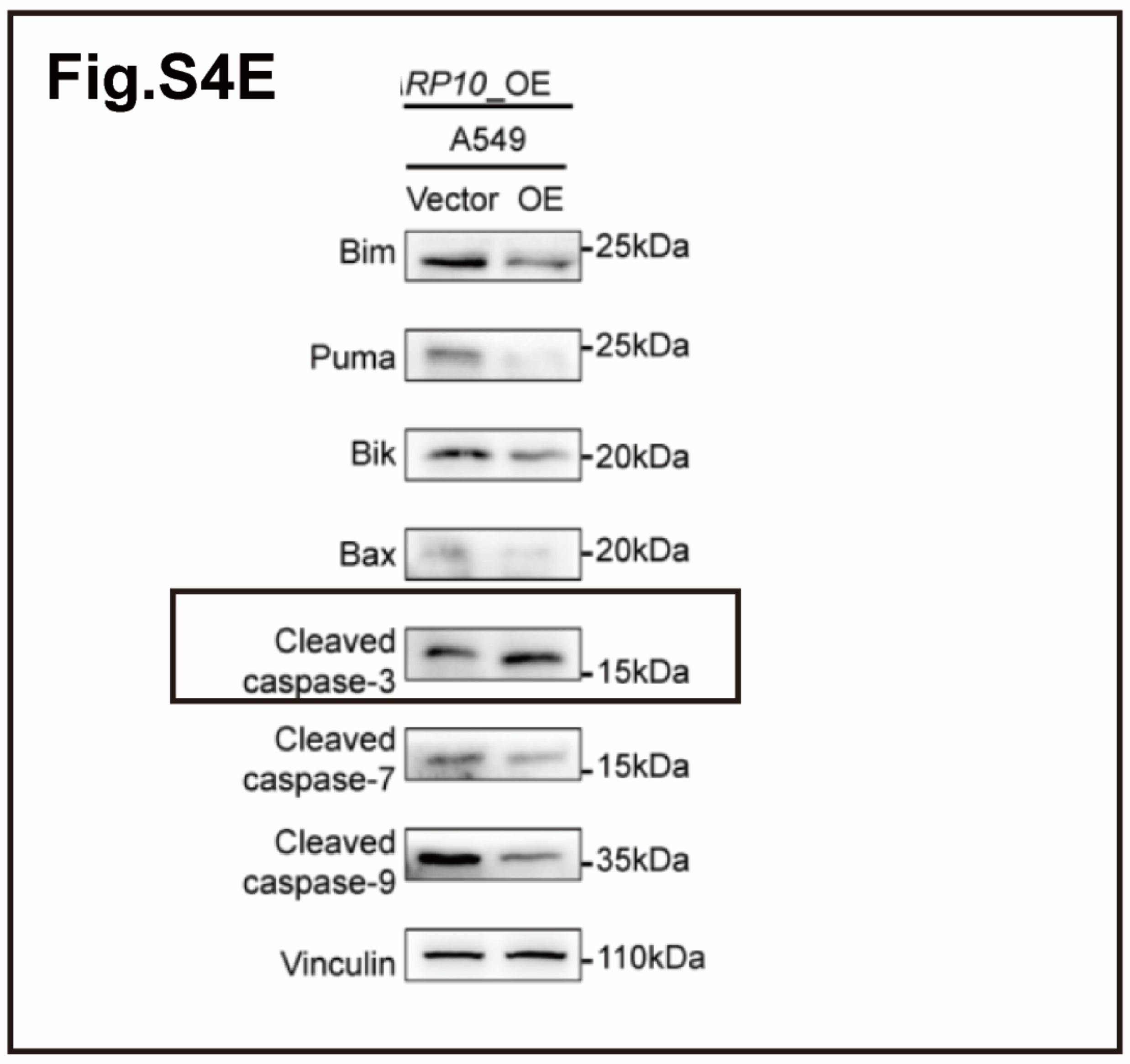

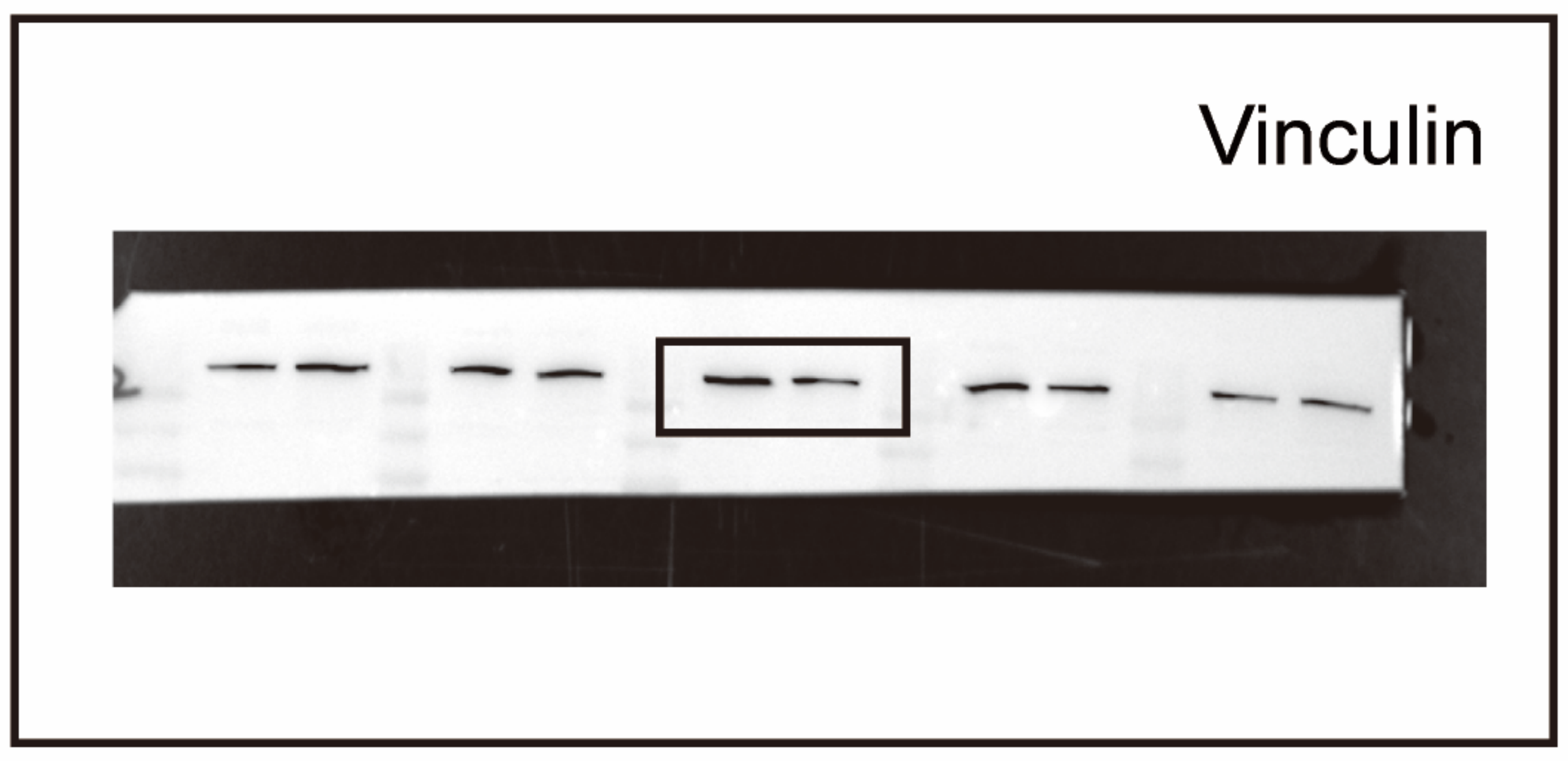

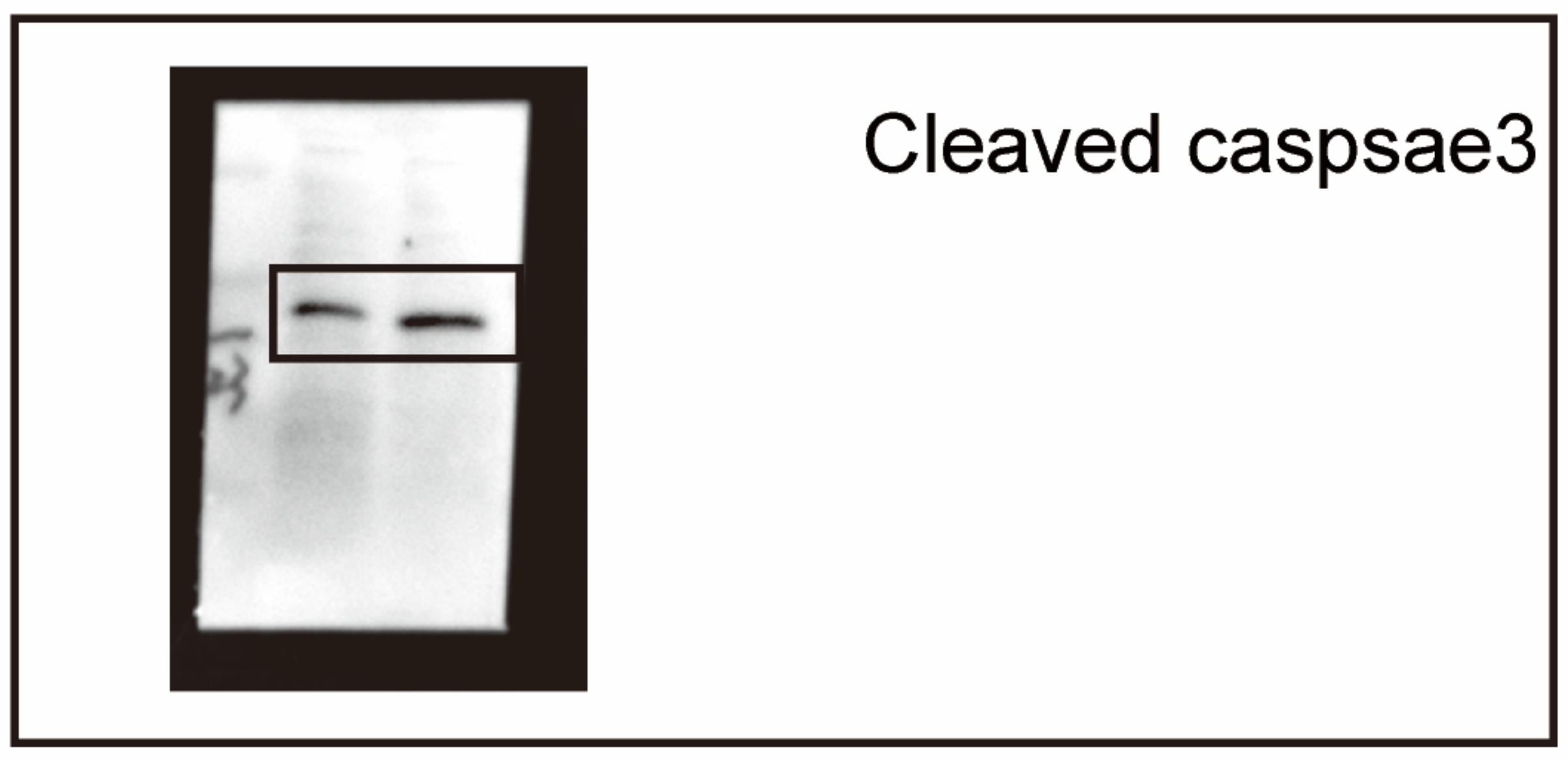

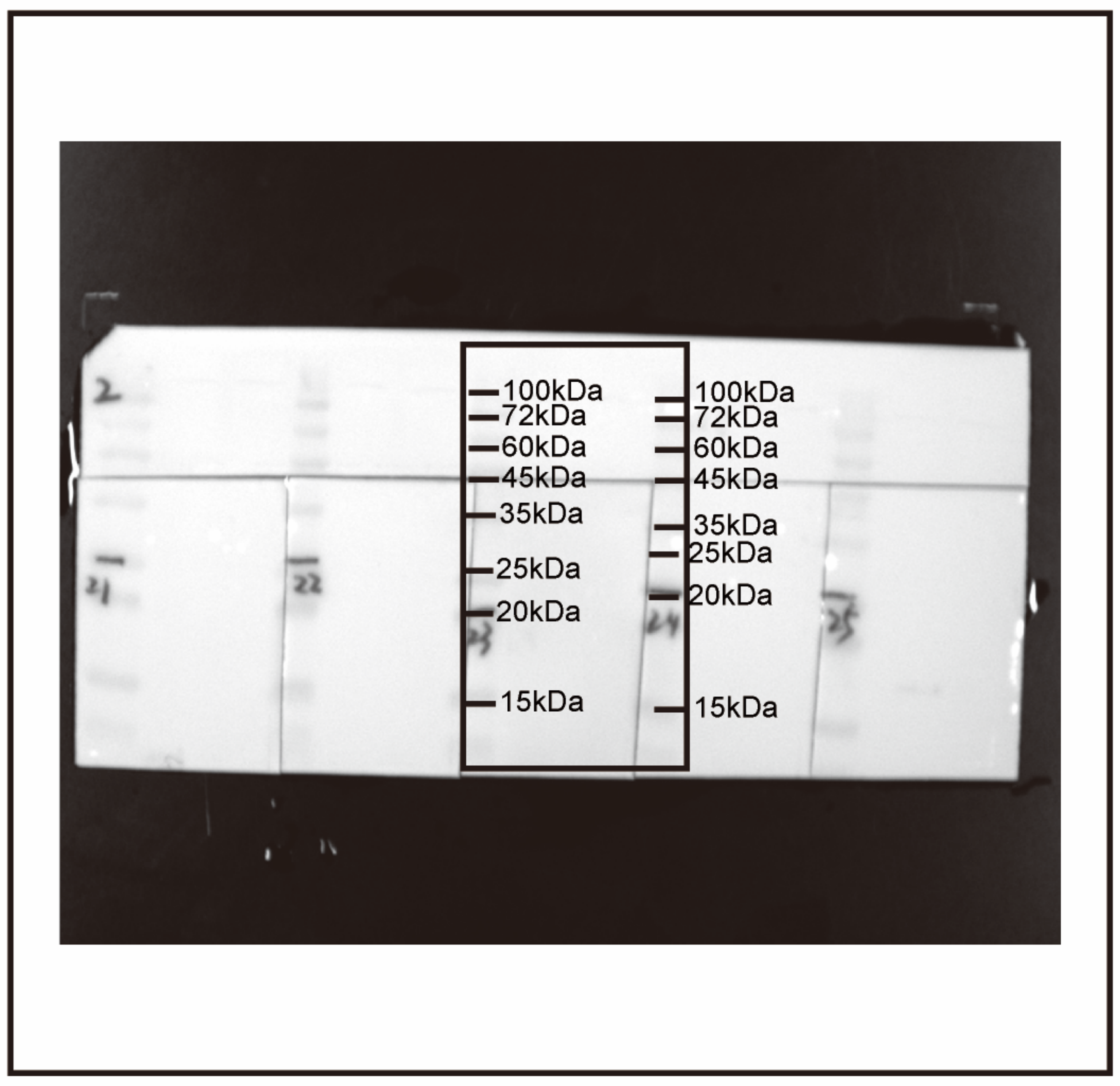

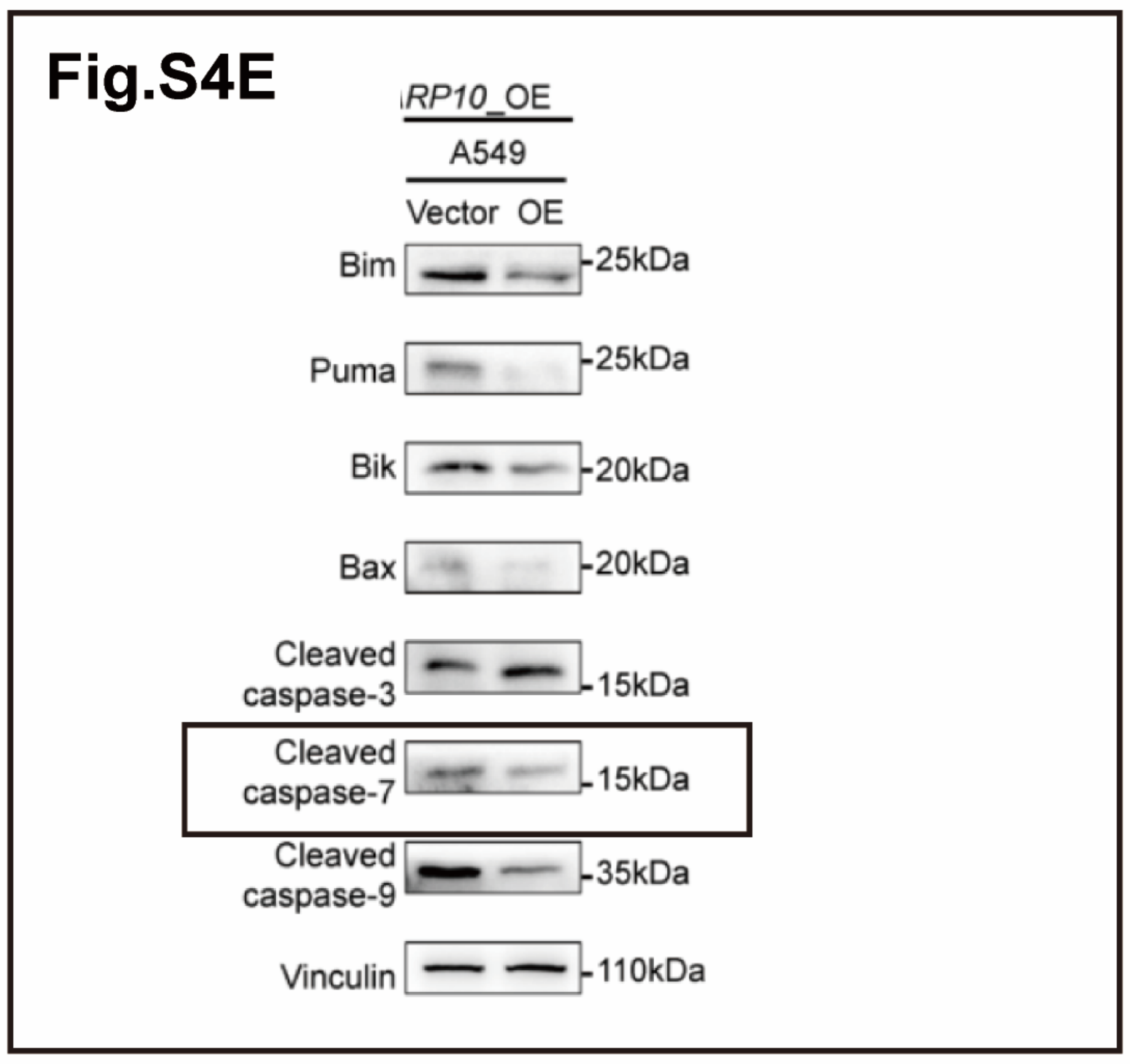

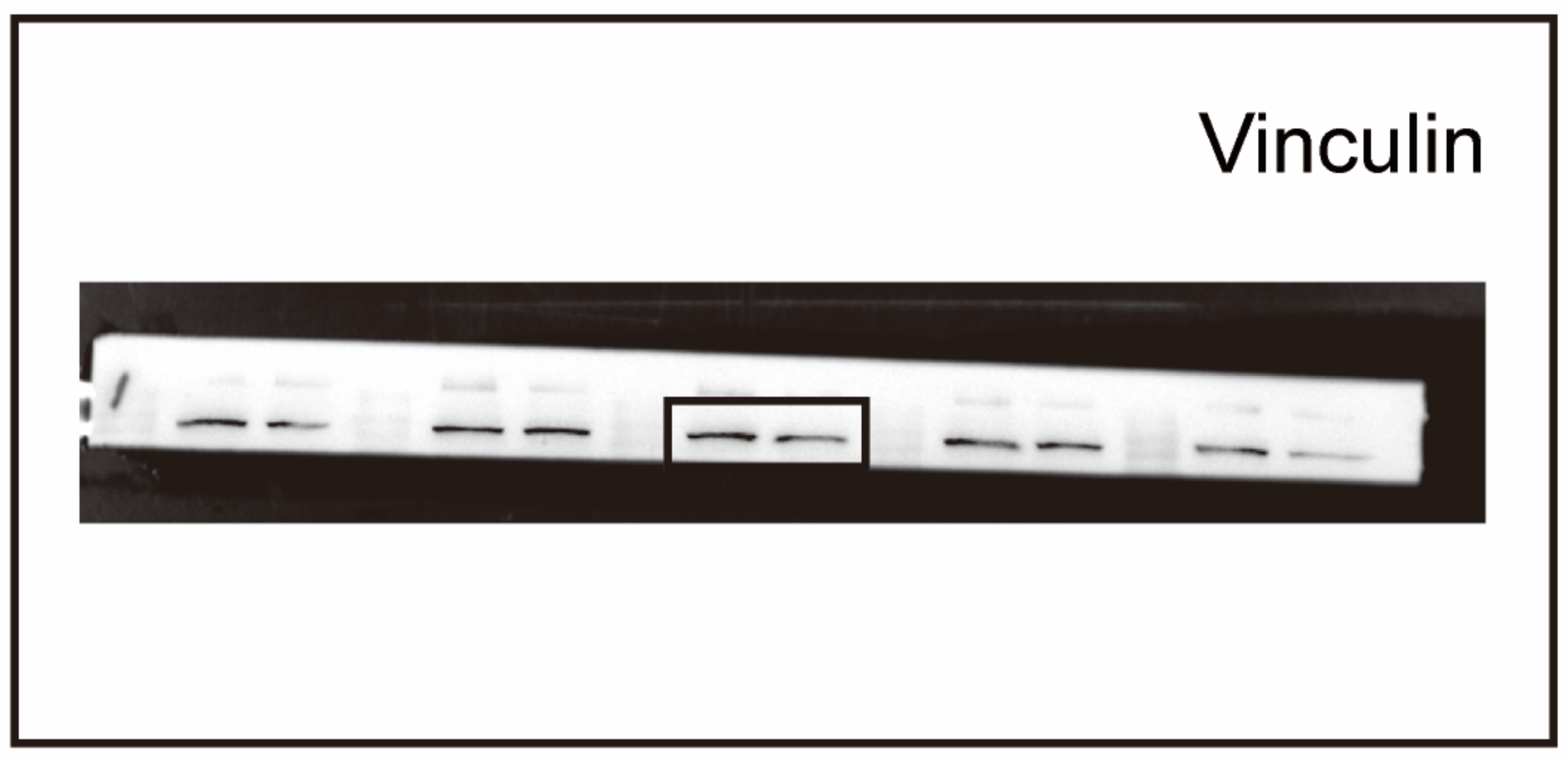

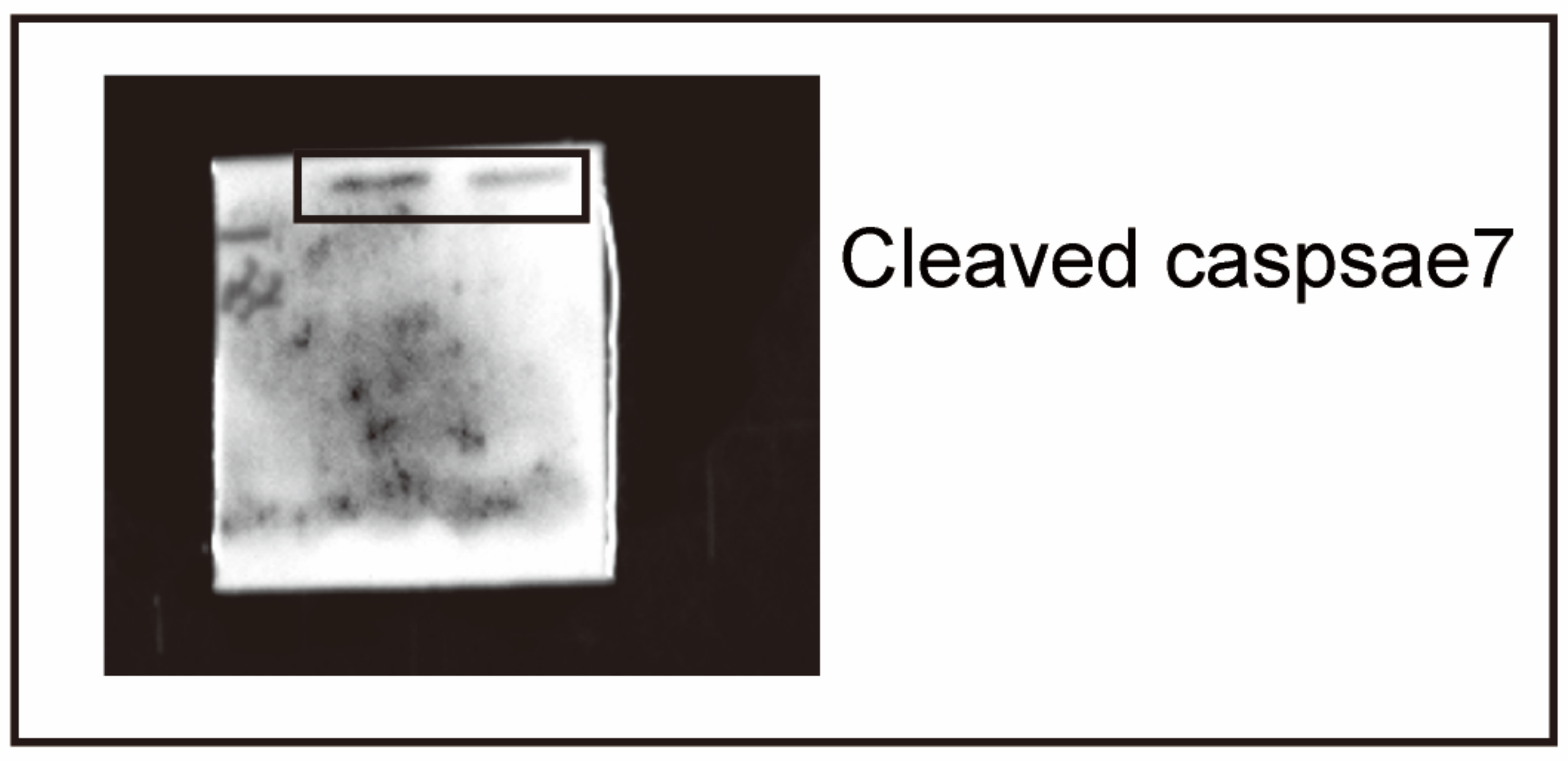

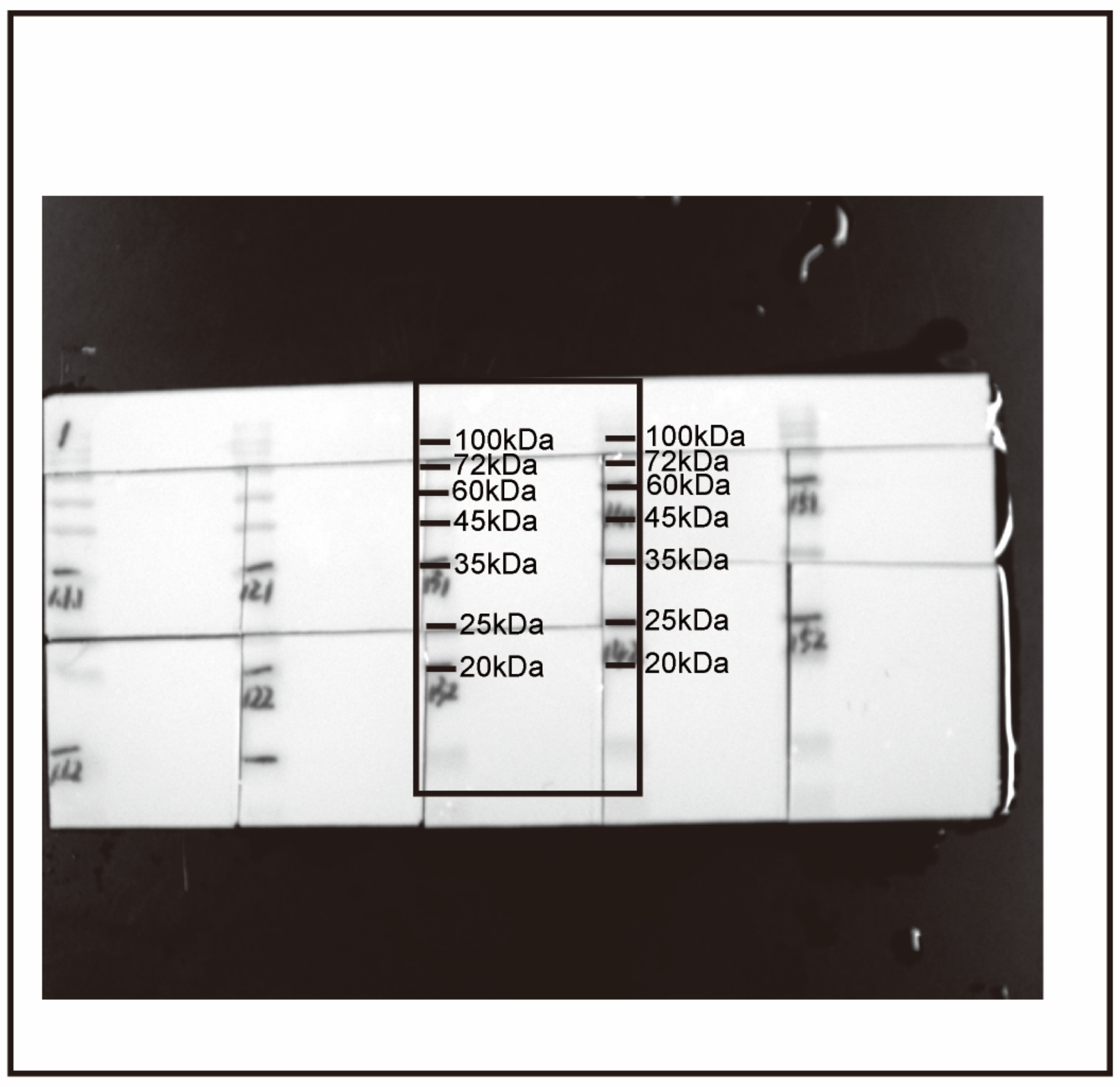

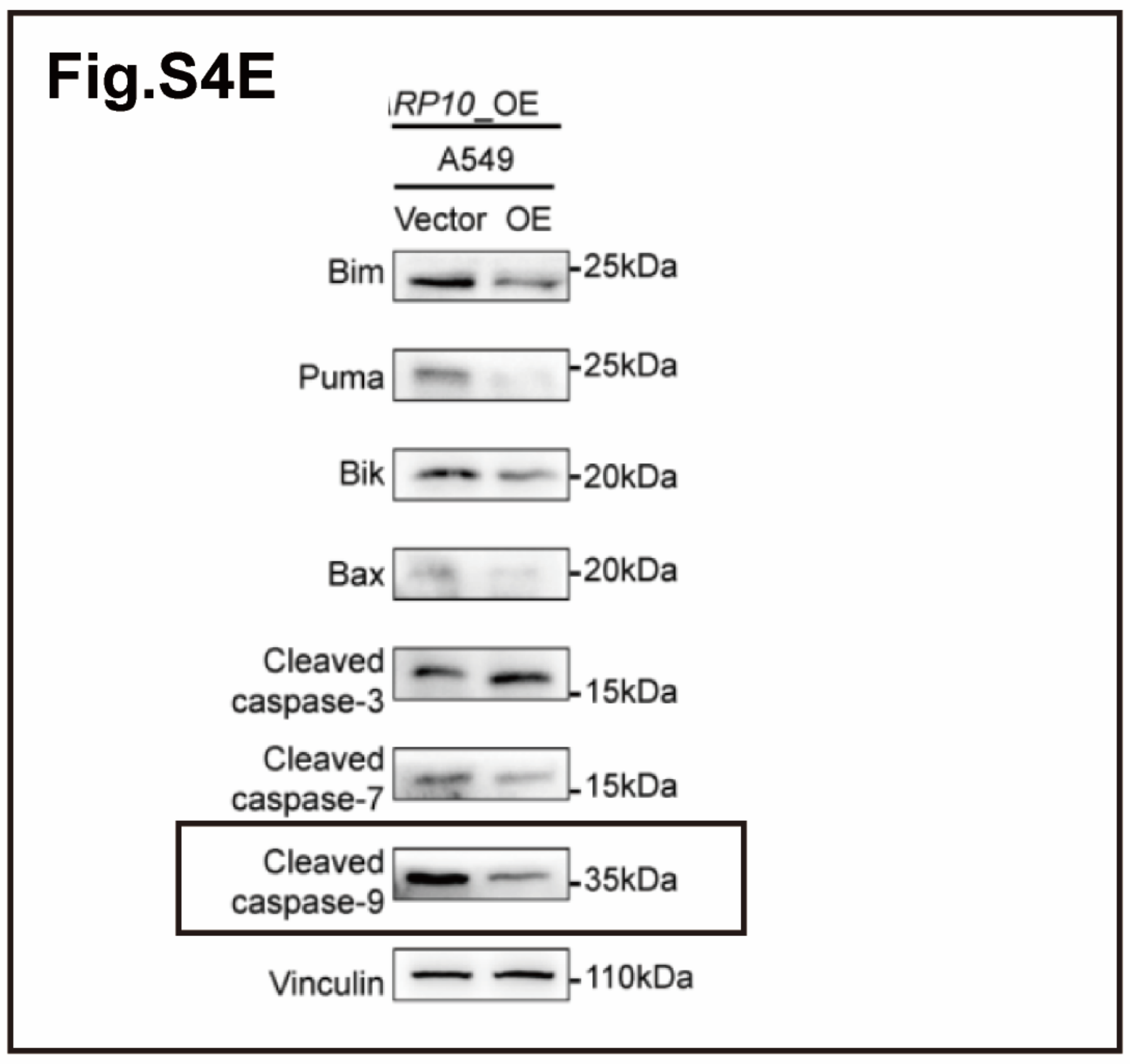

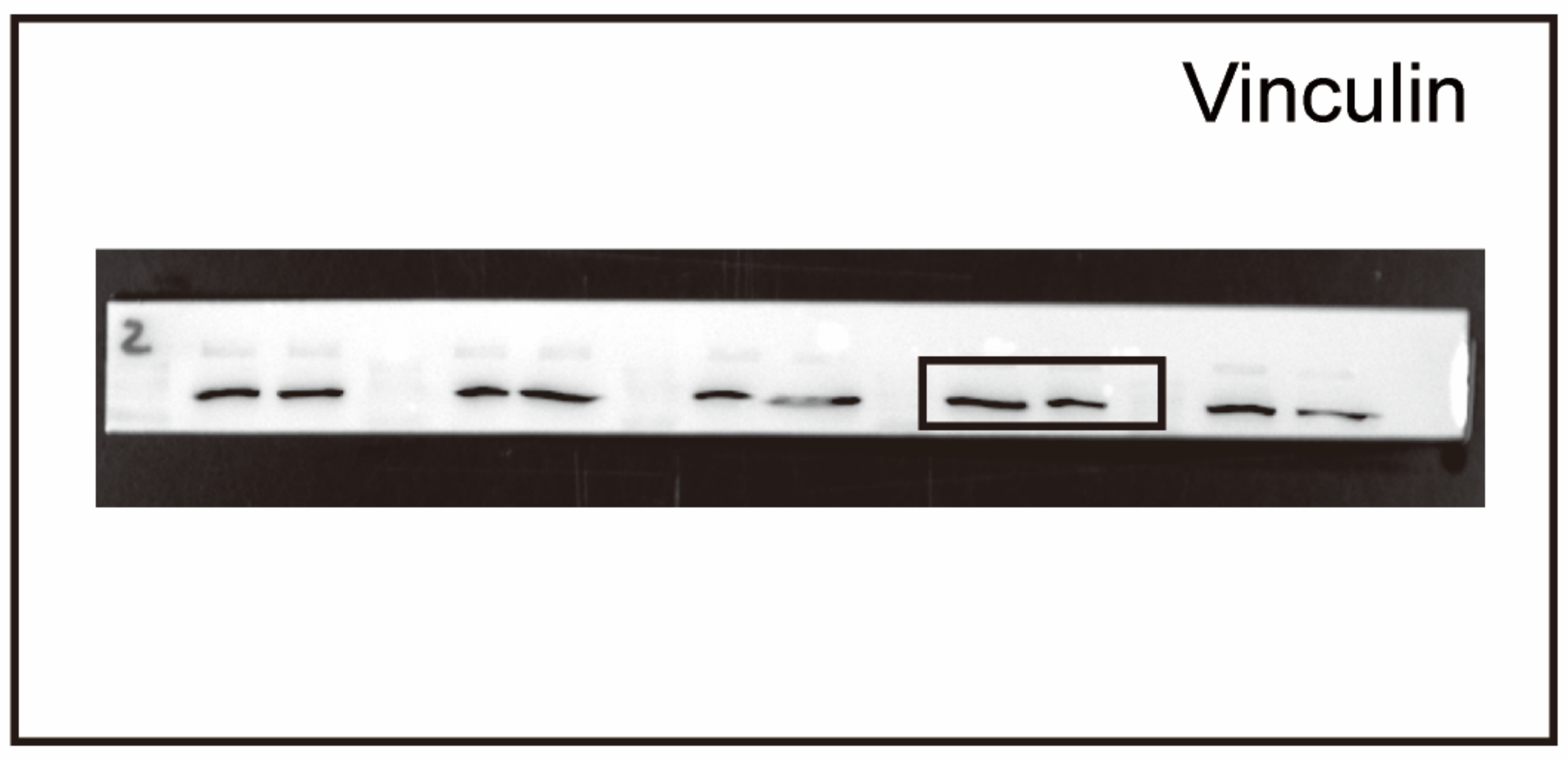

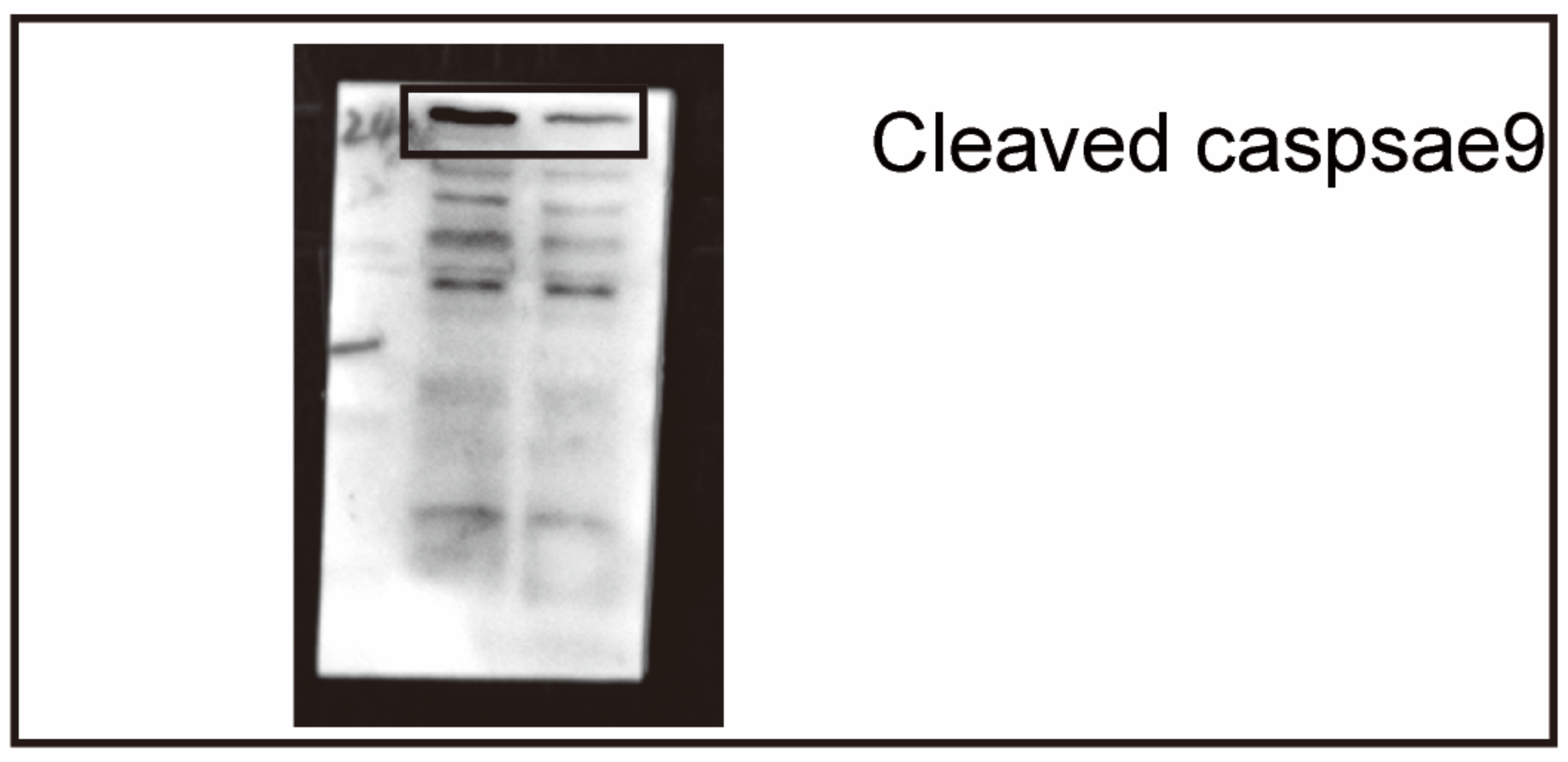

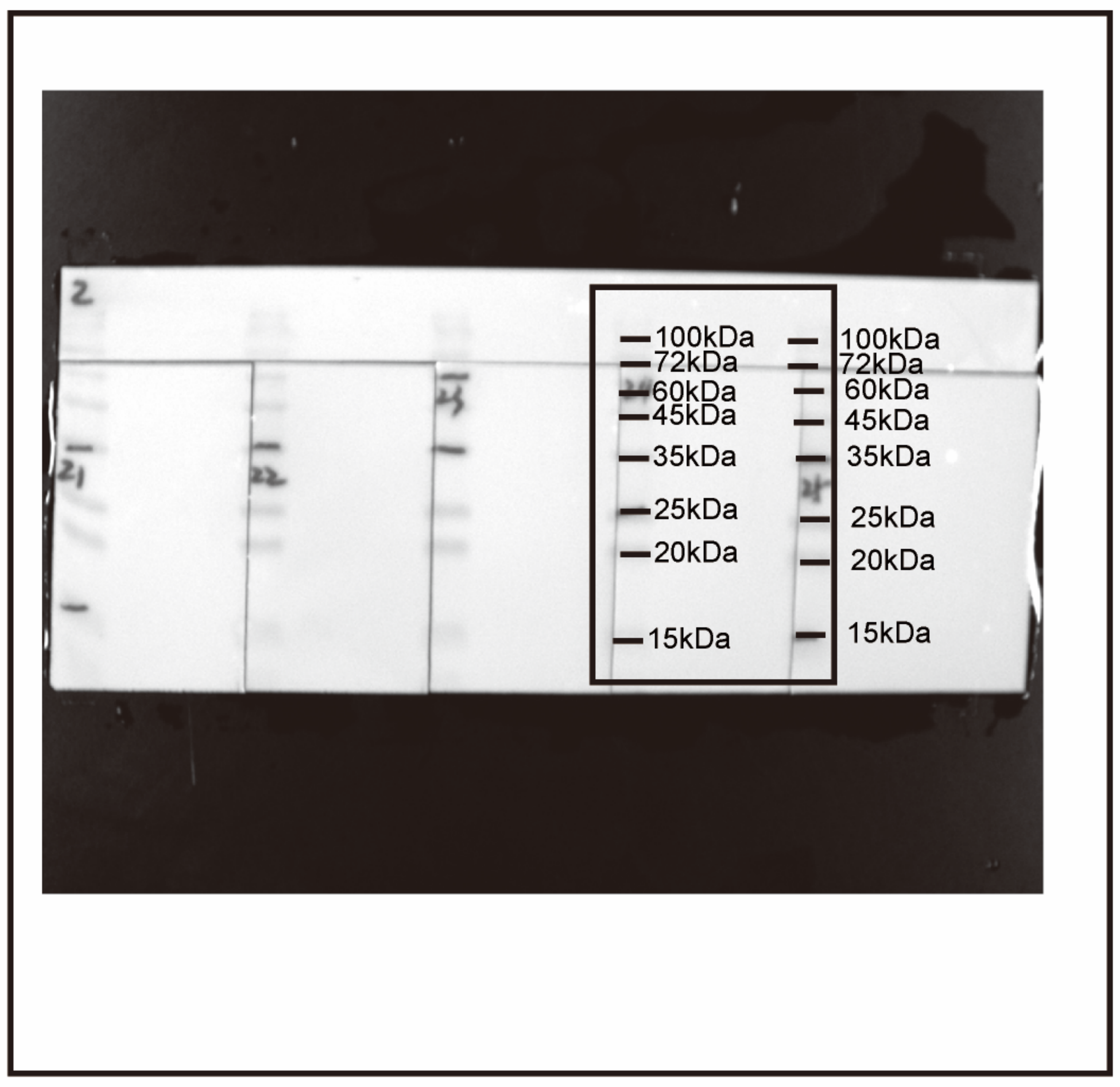

Figure	Merged	Stiched full image

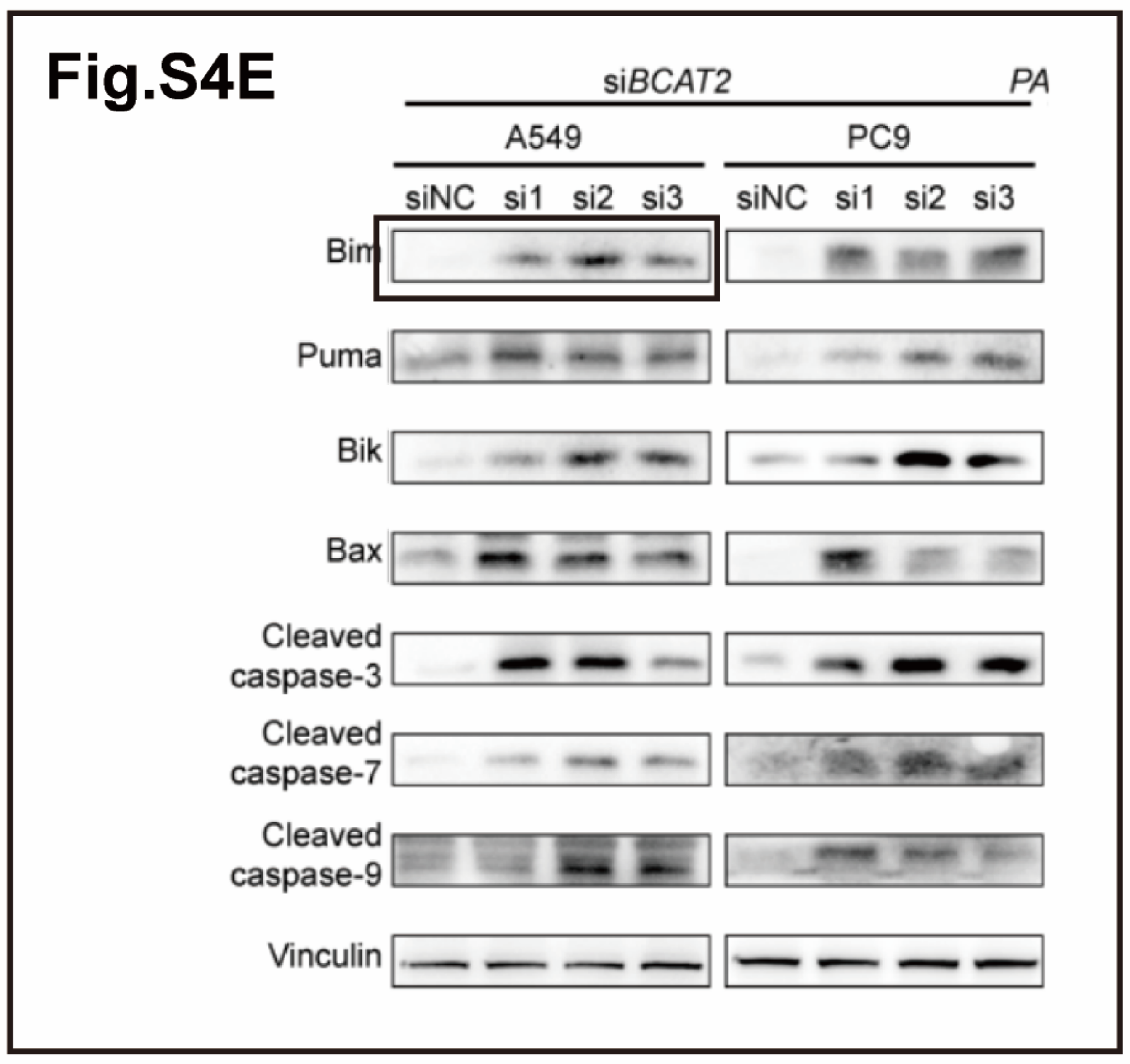

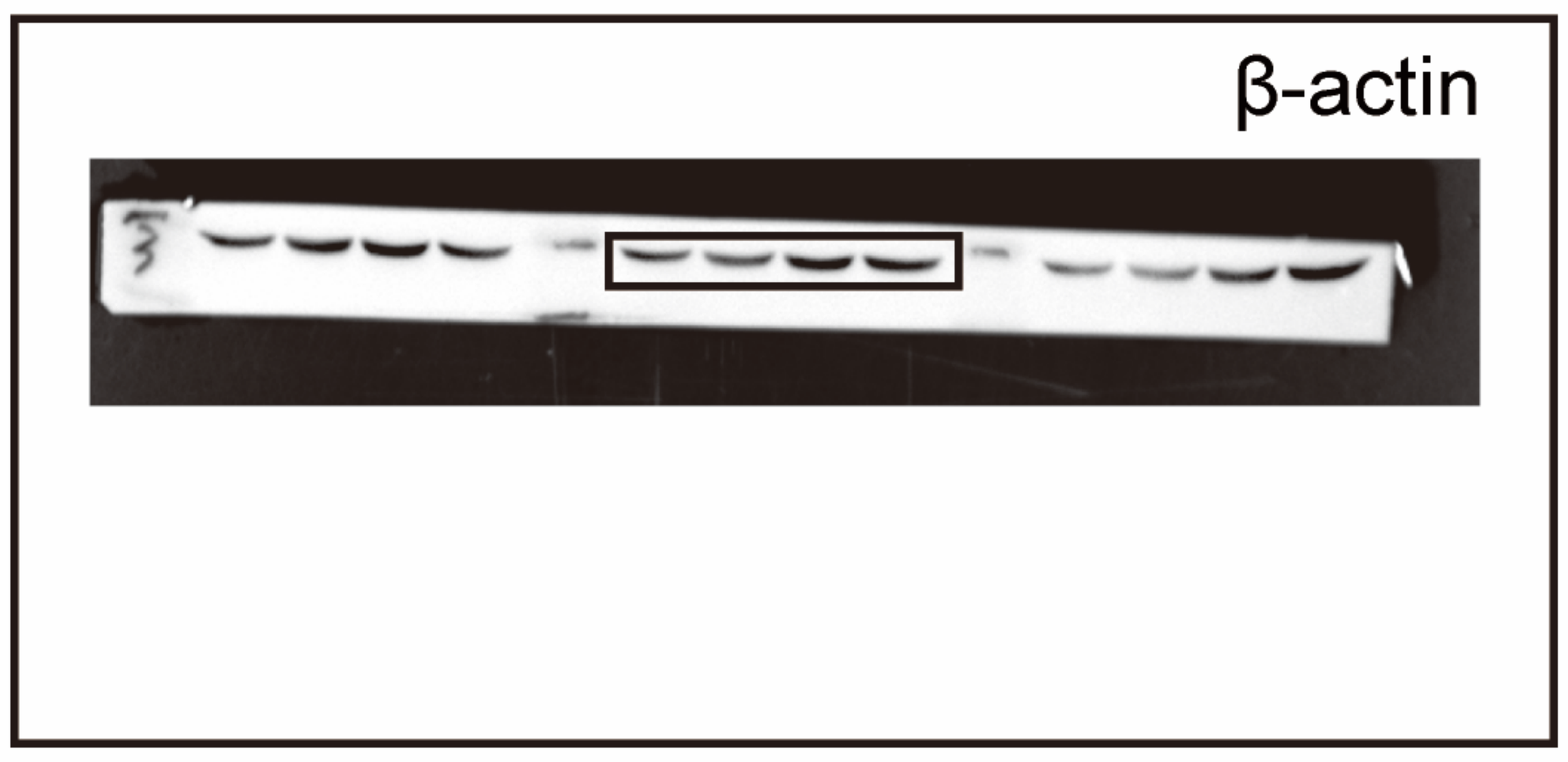

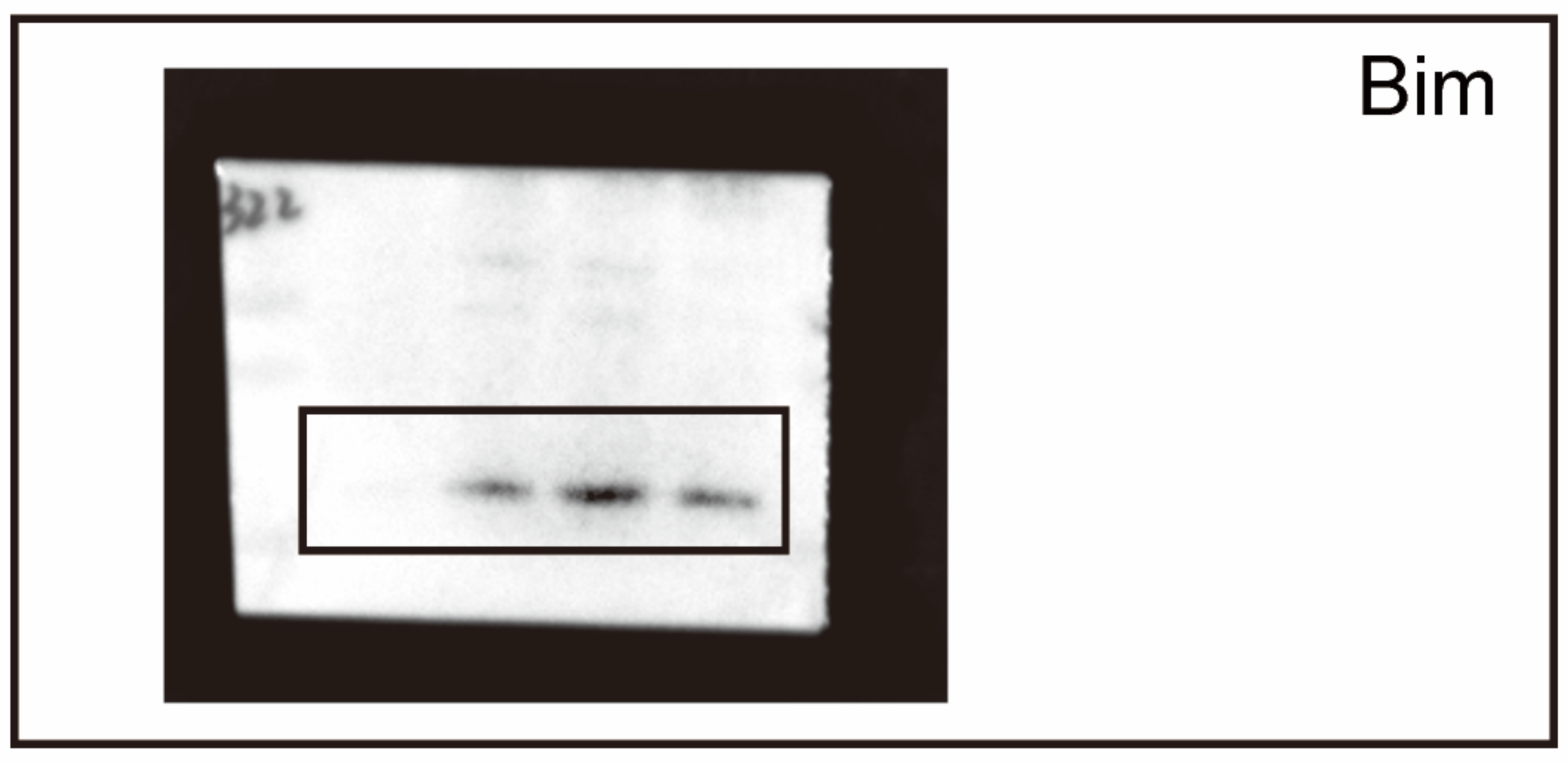

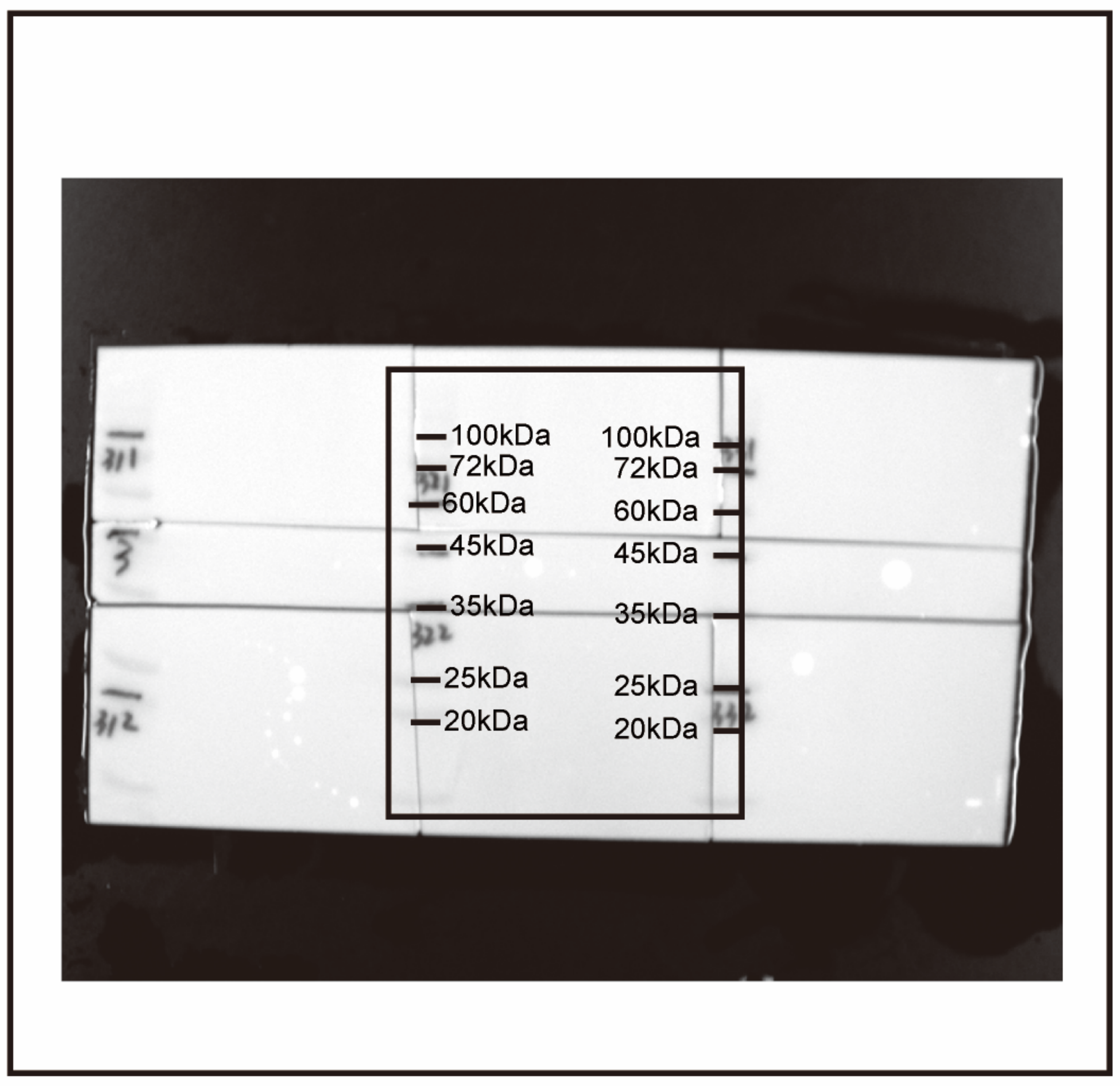

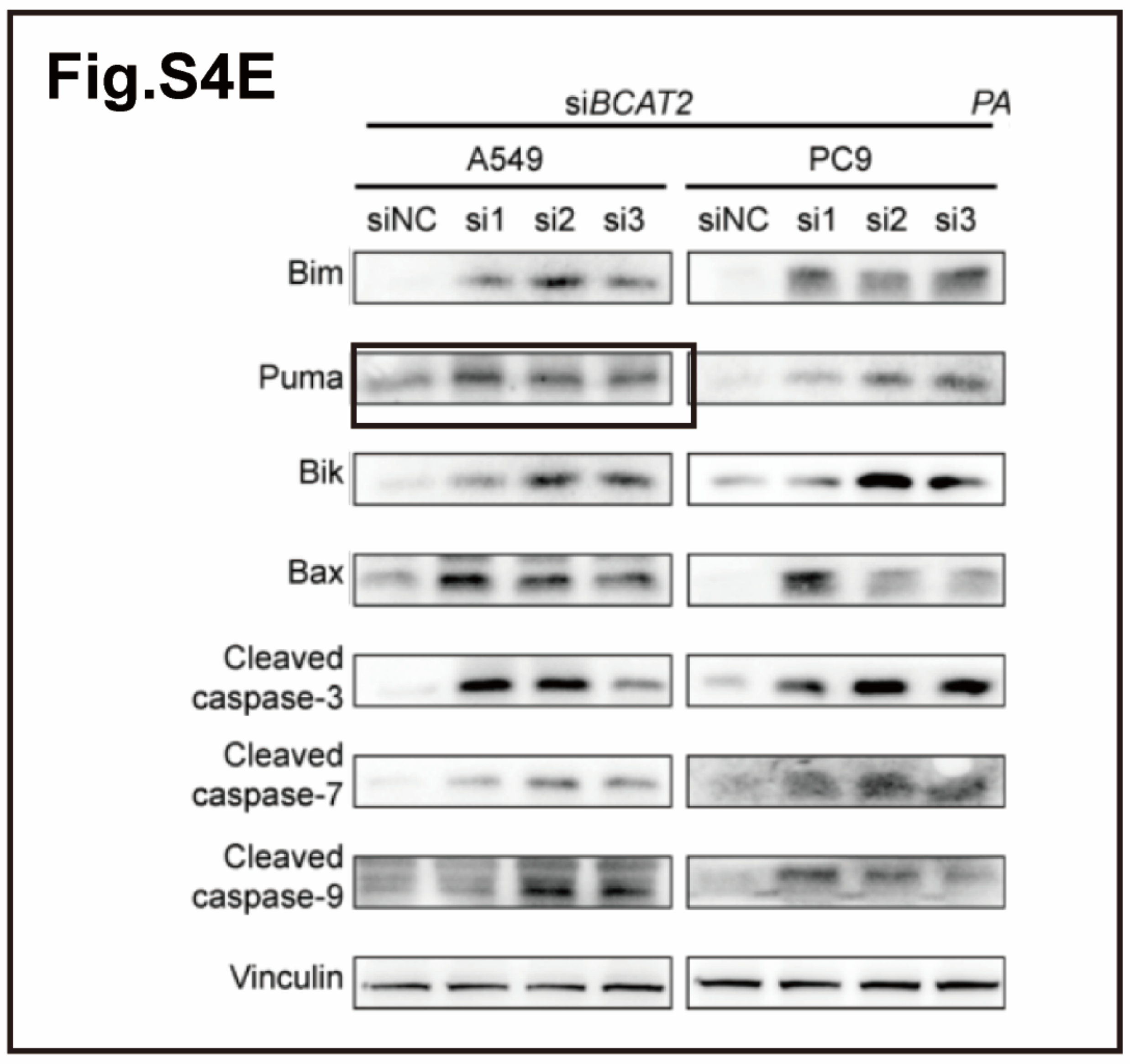

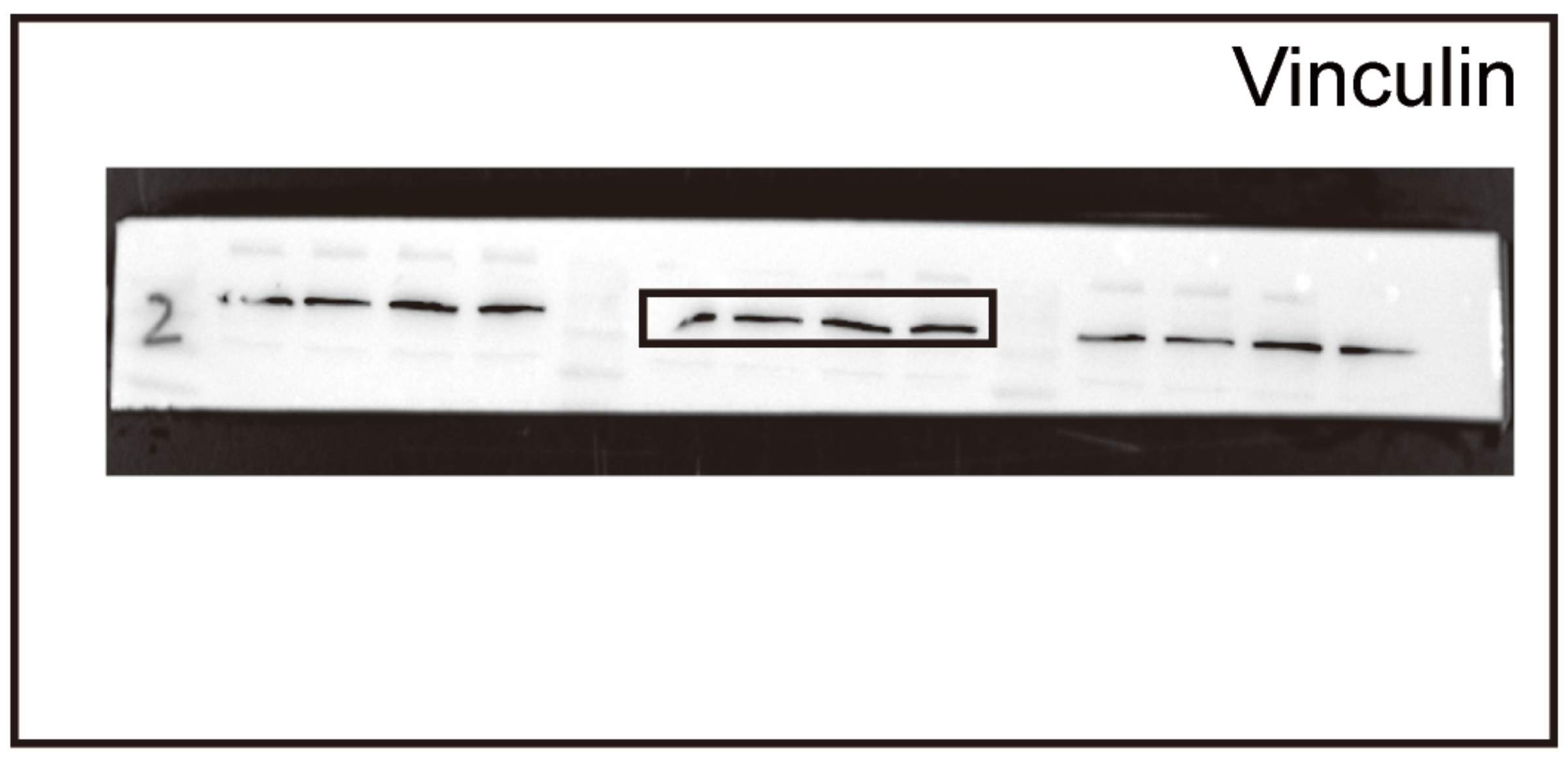

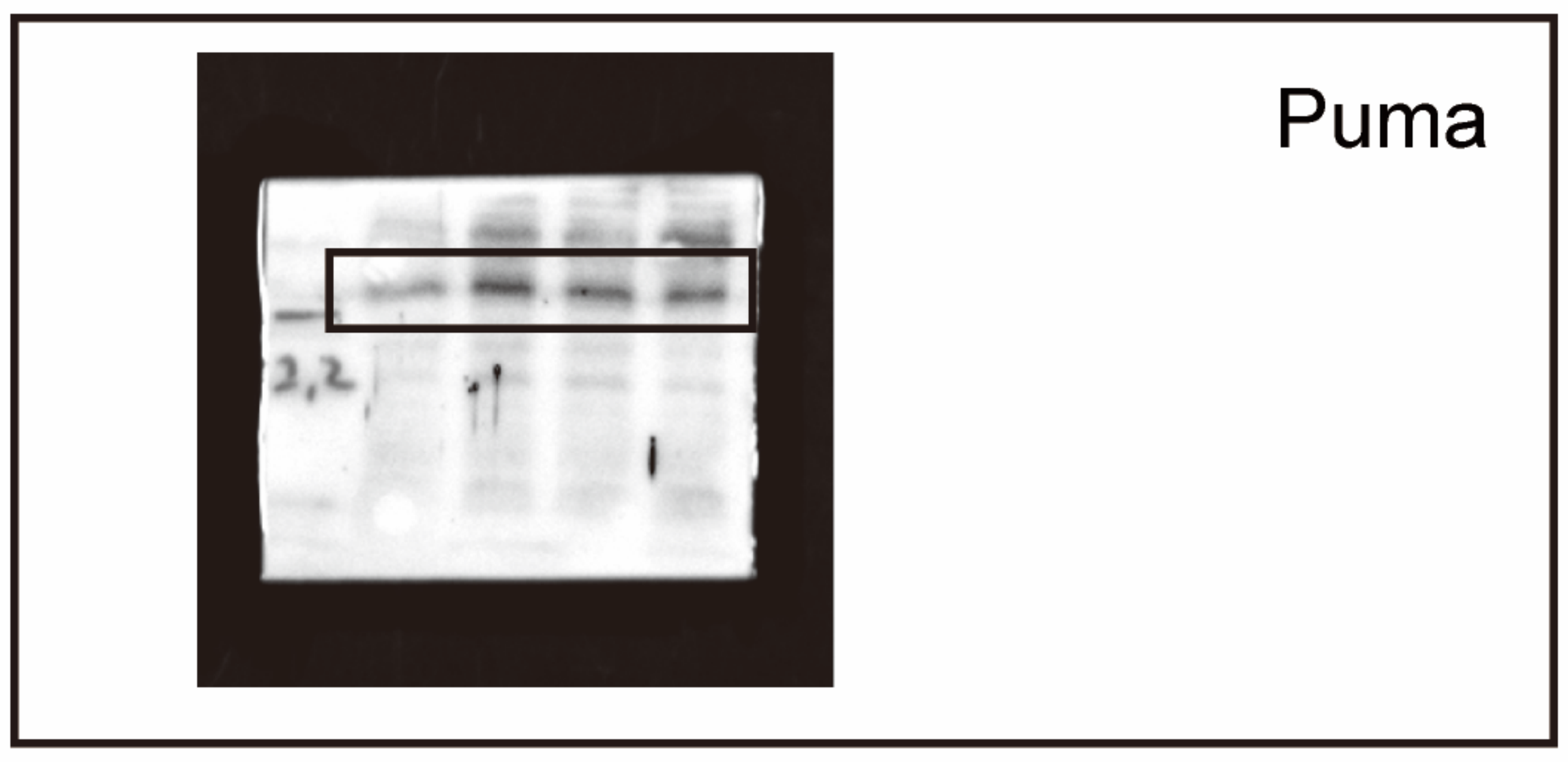

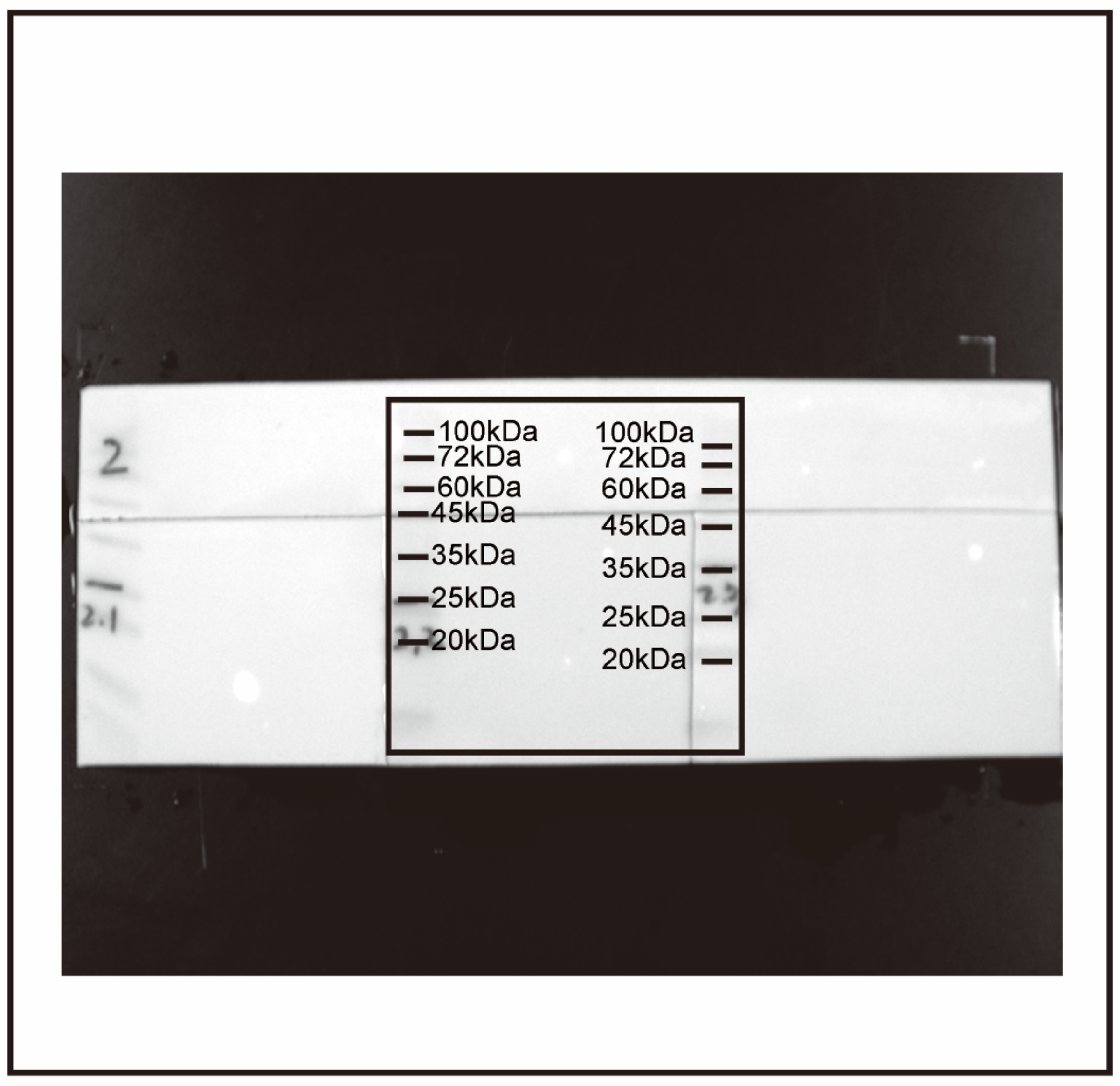

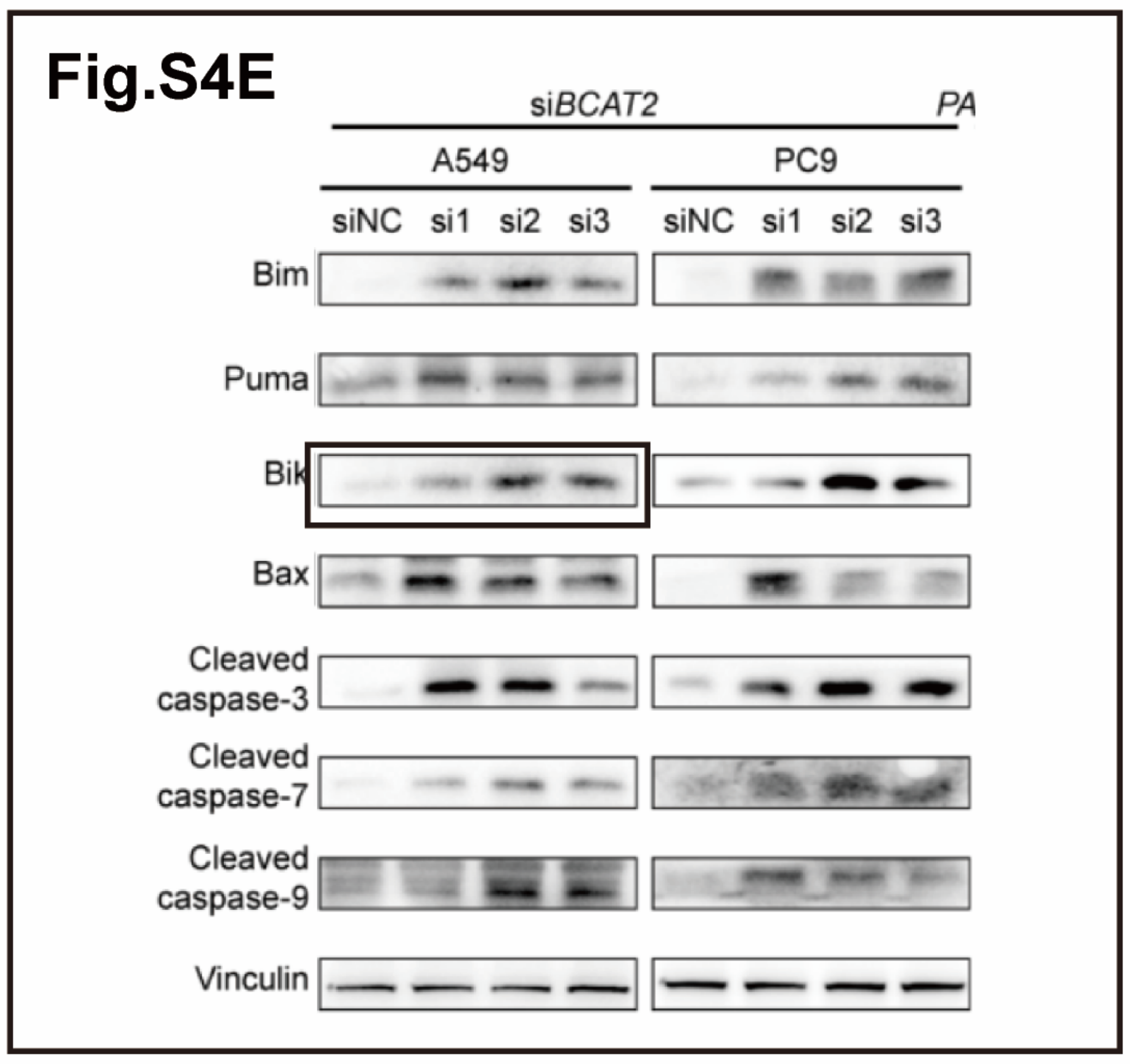

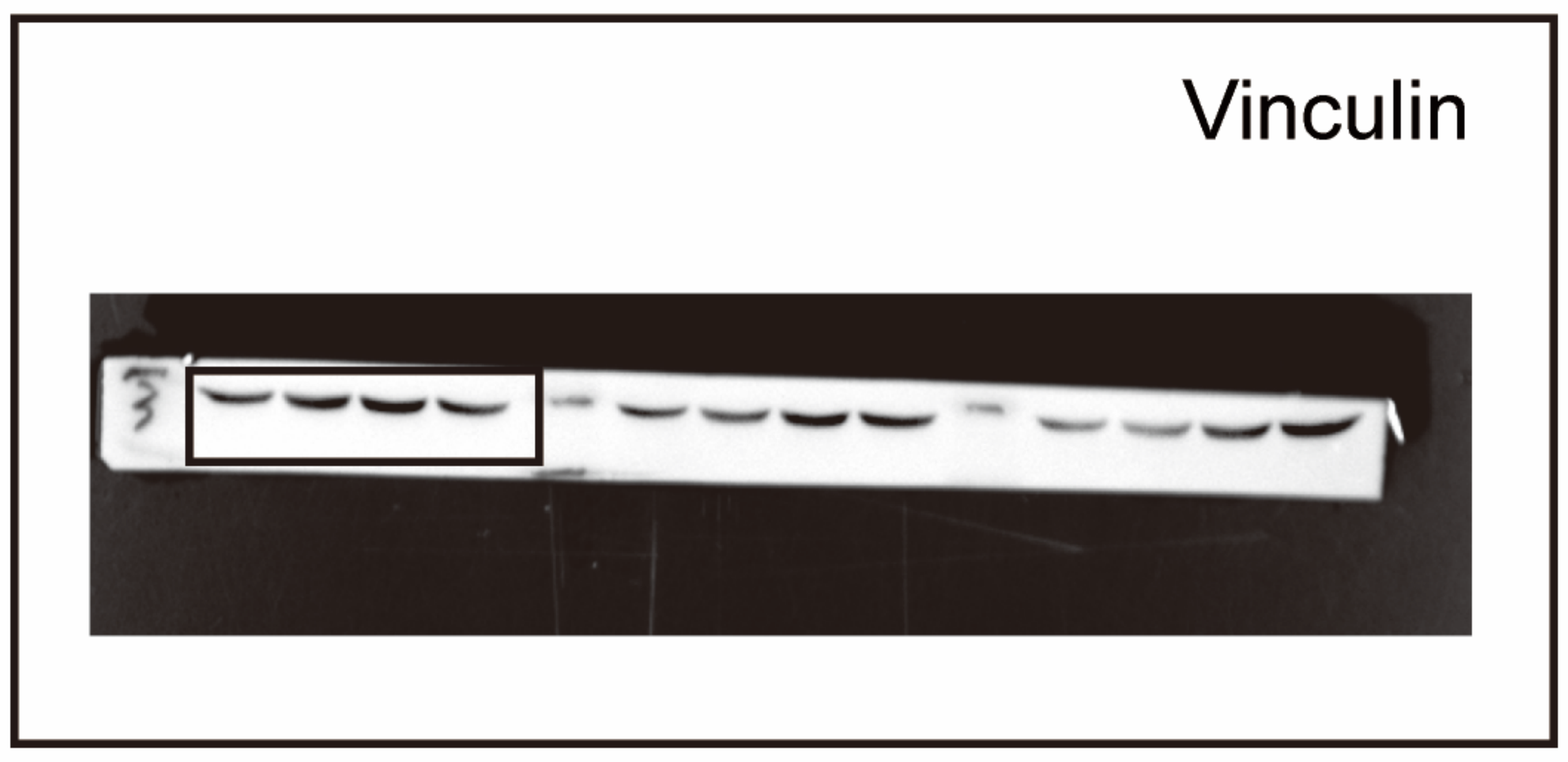

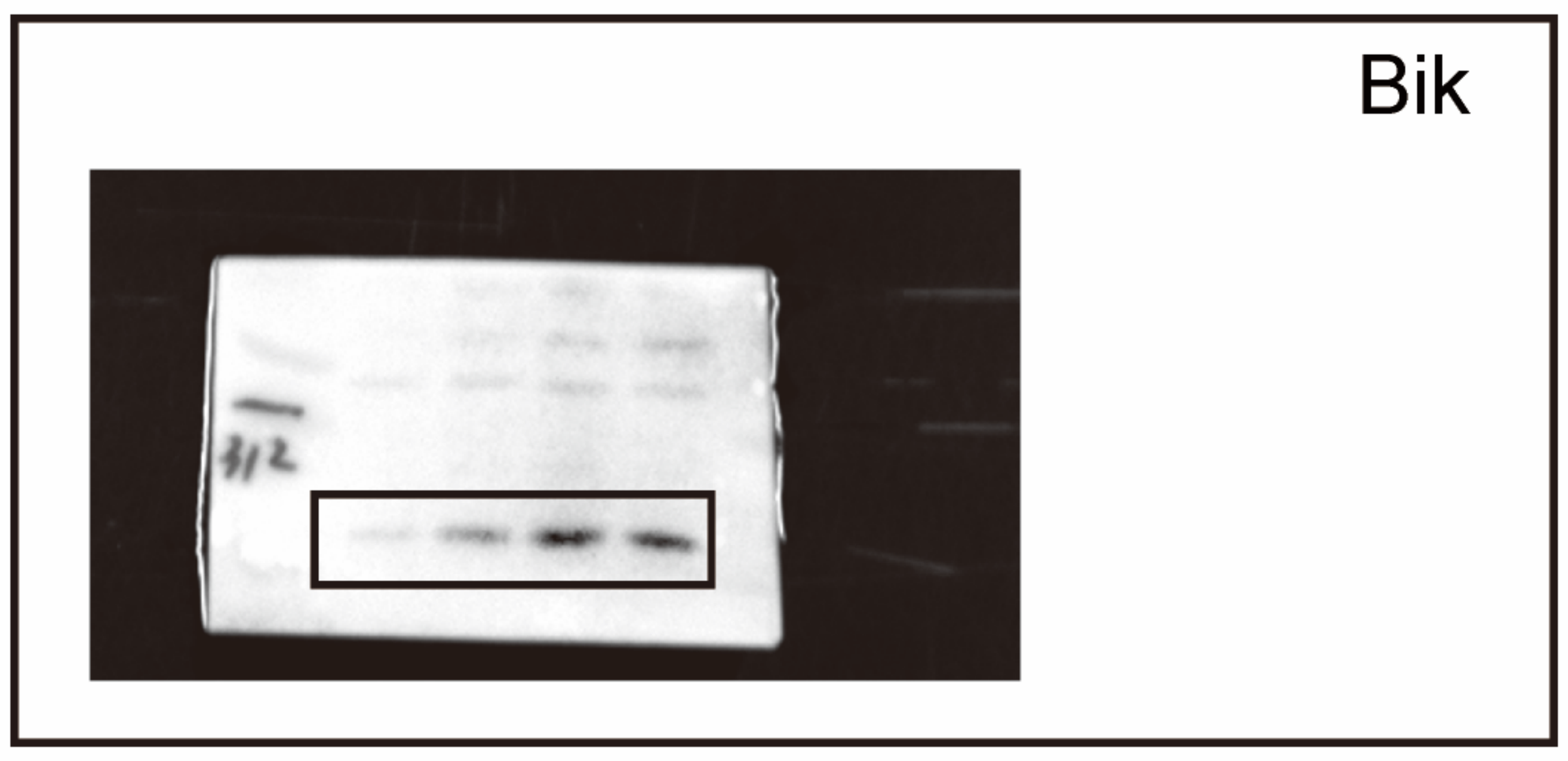

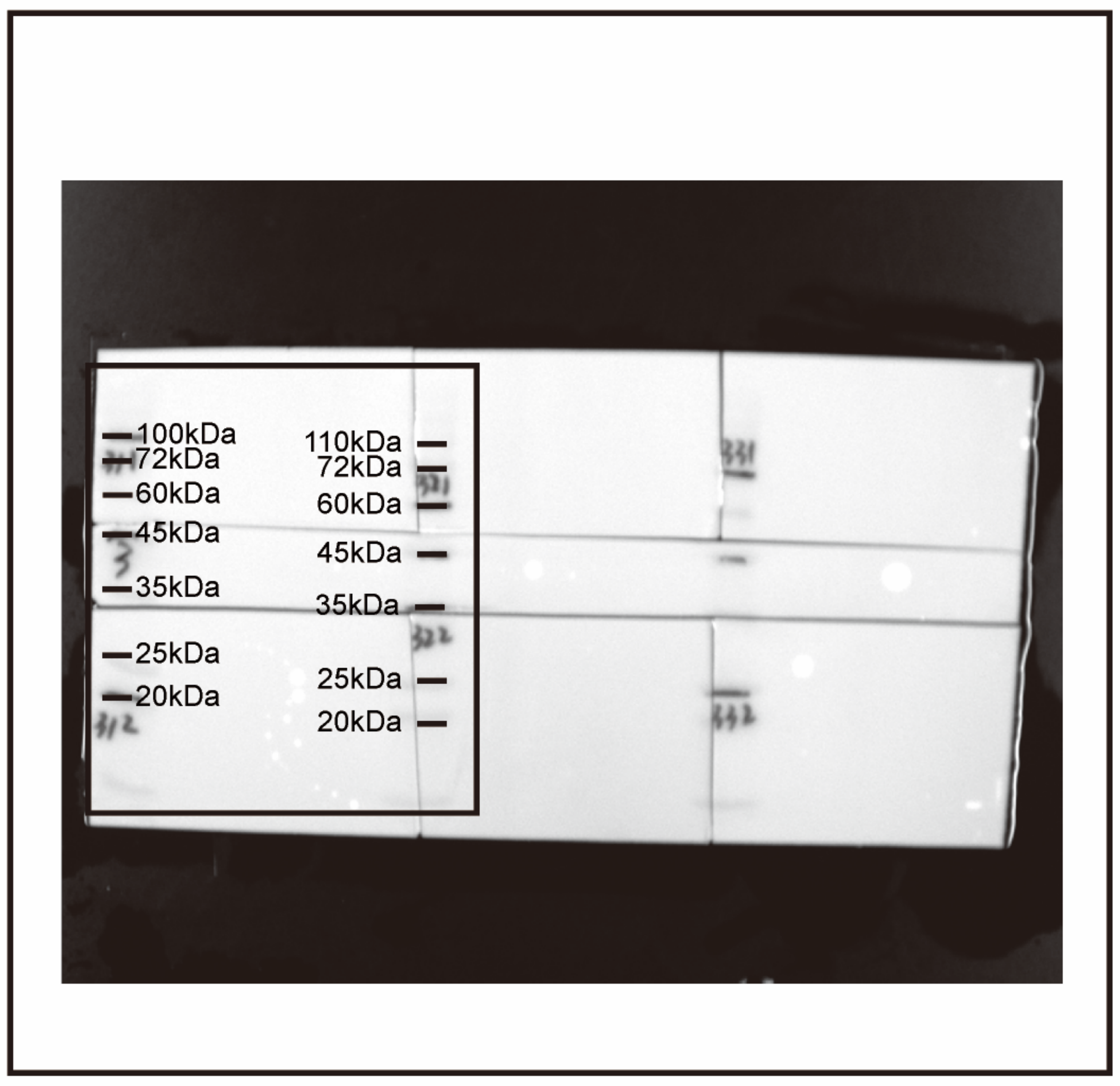

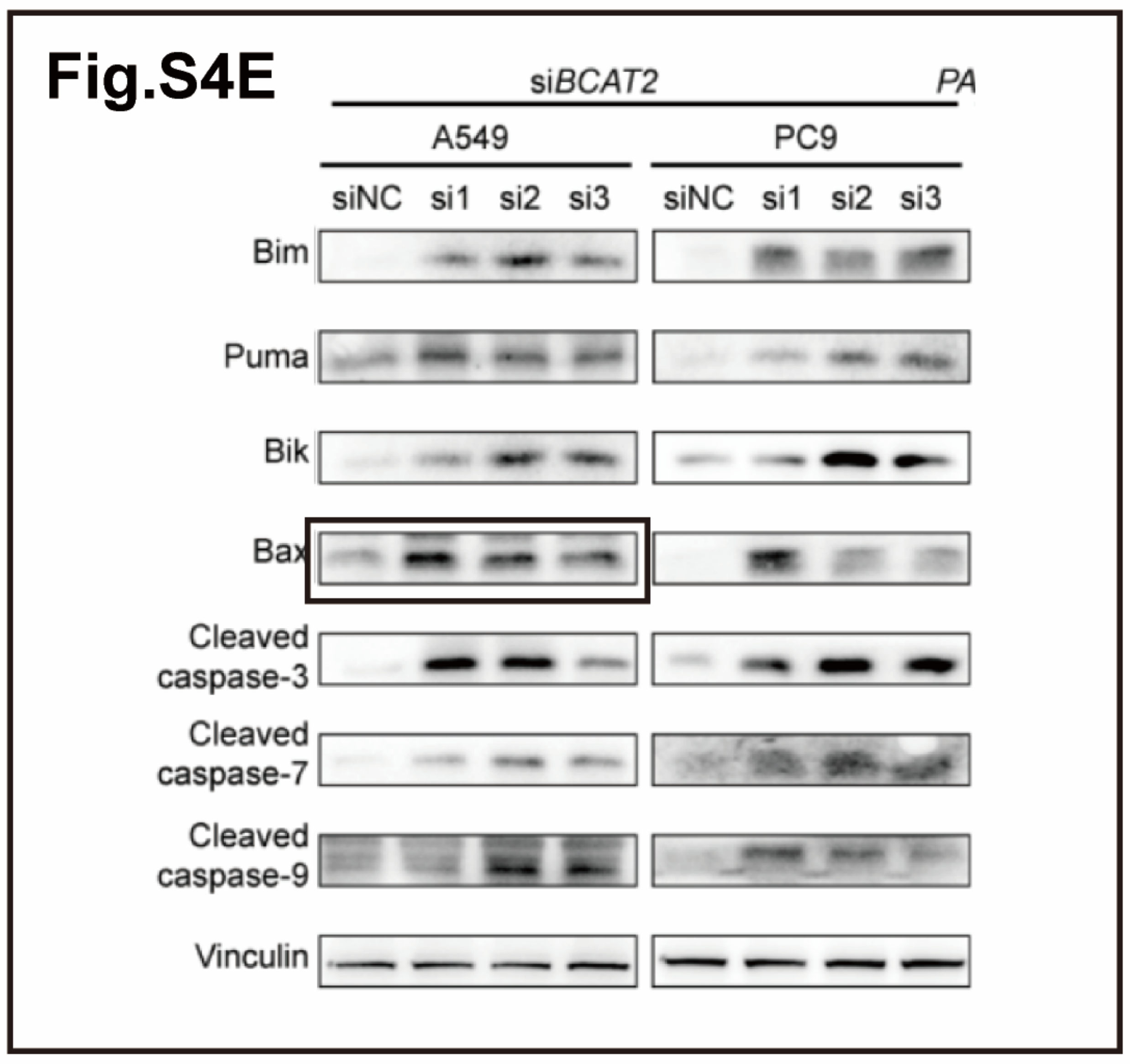

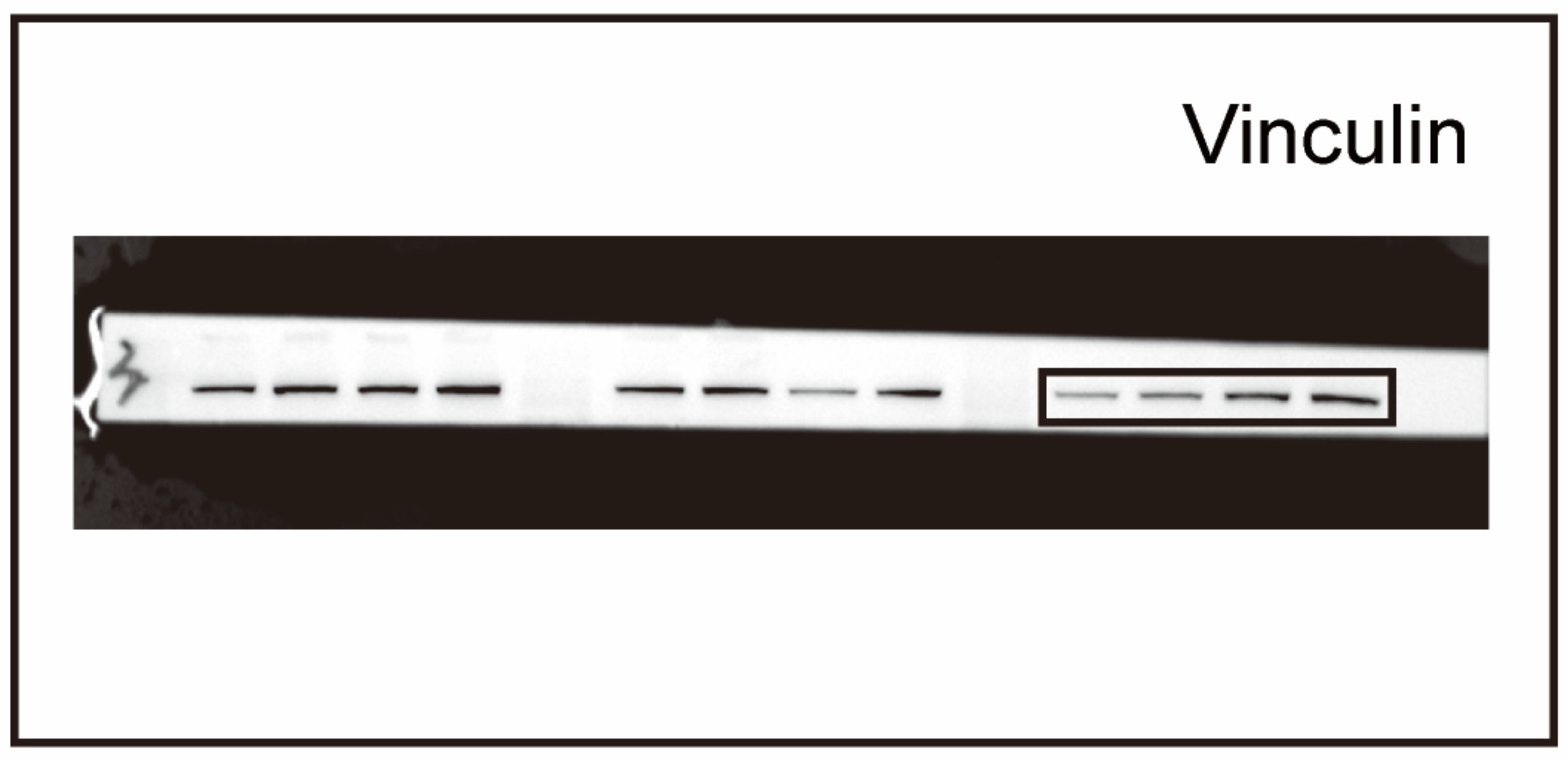

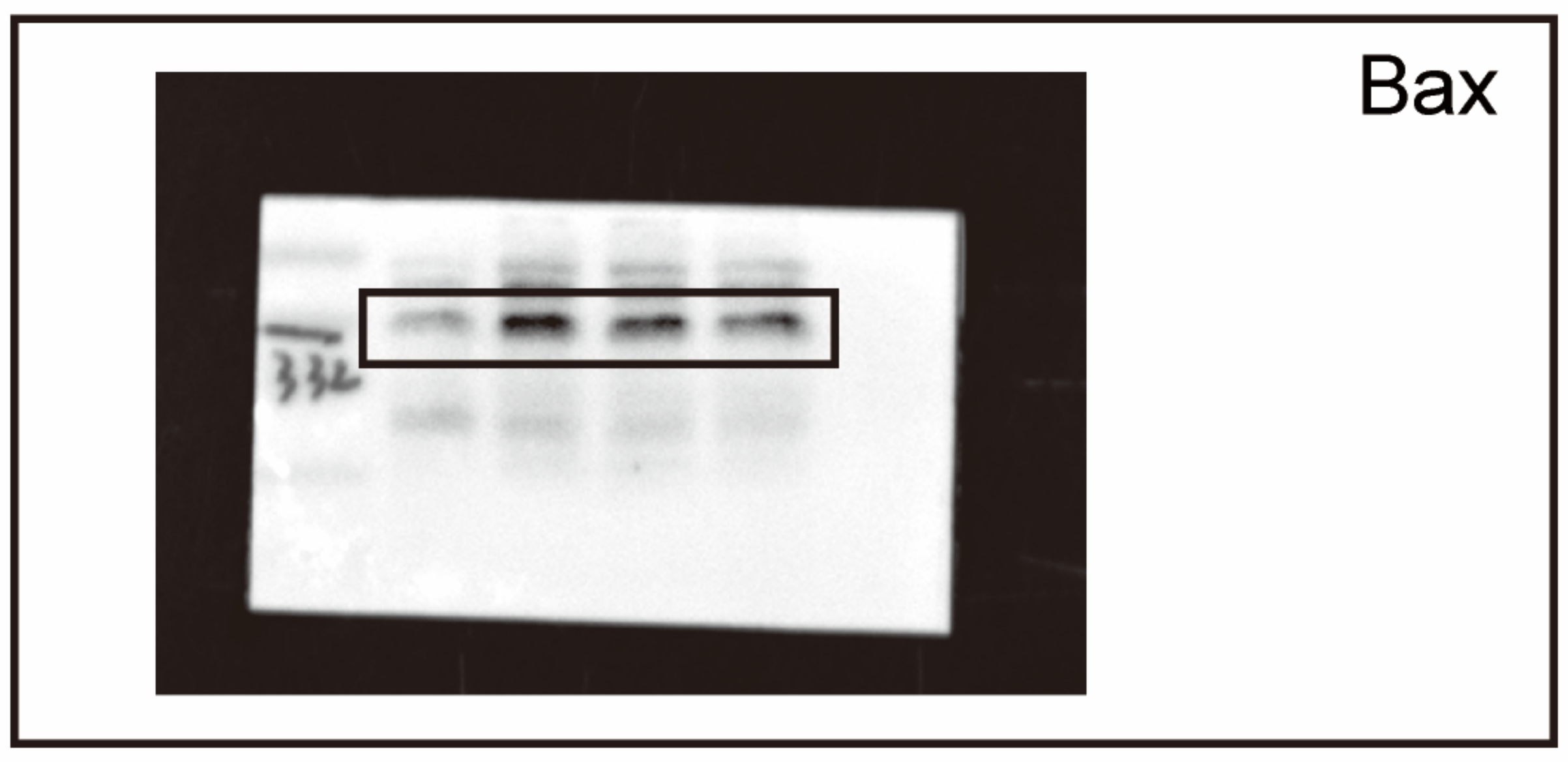

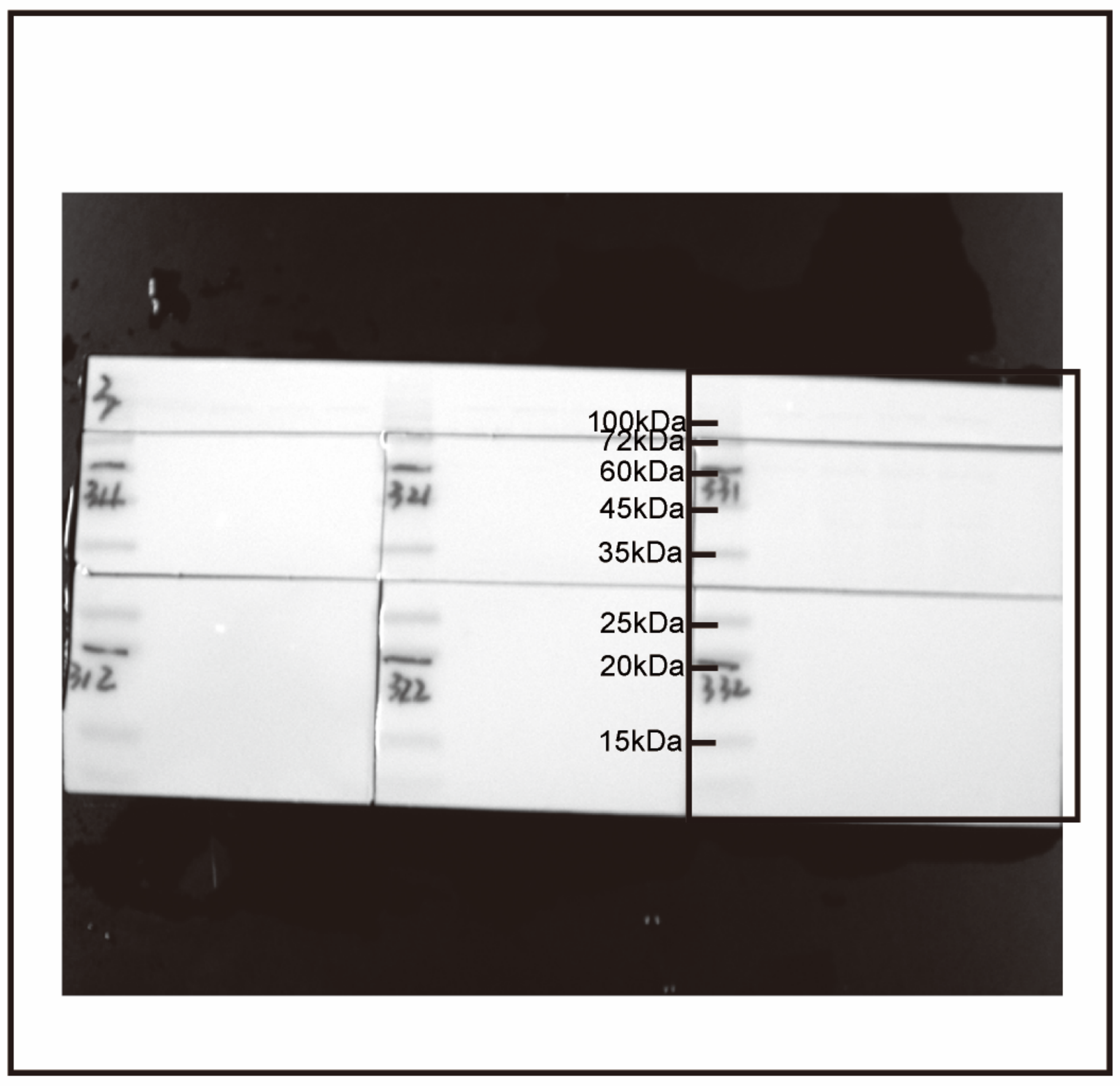

Figure	Merged	Stiched full image

Fig.S4E

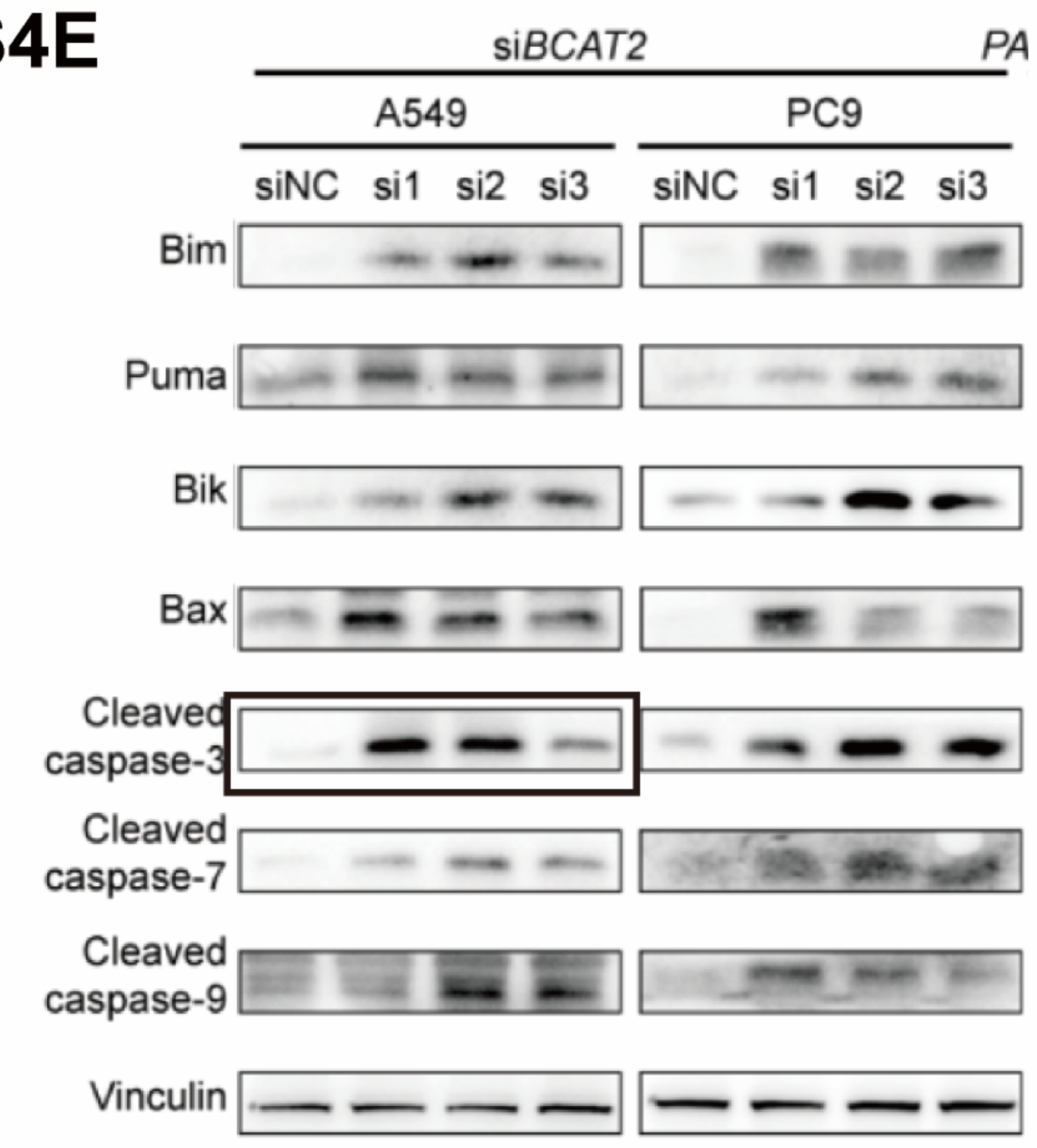

Vinculin

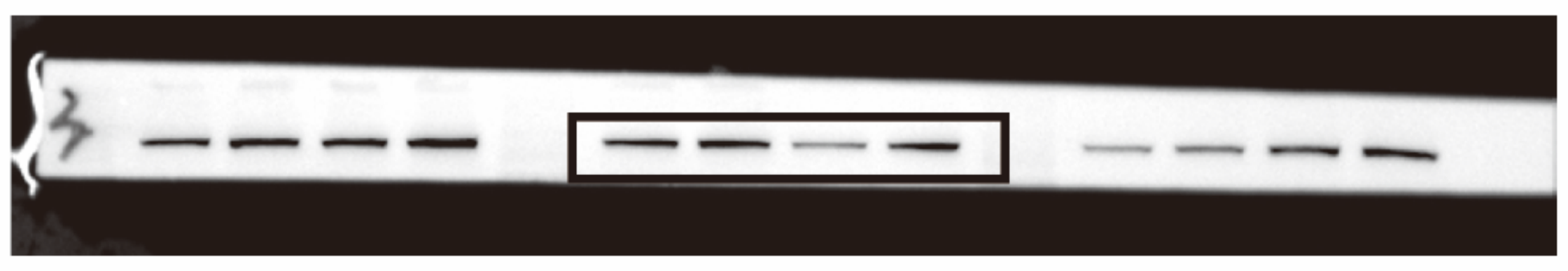

Cleaved caspase3

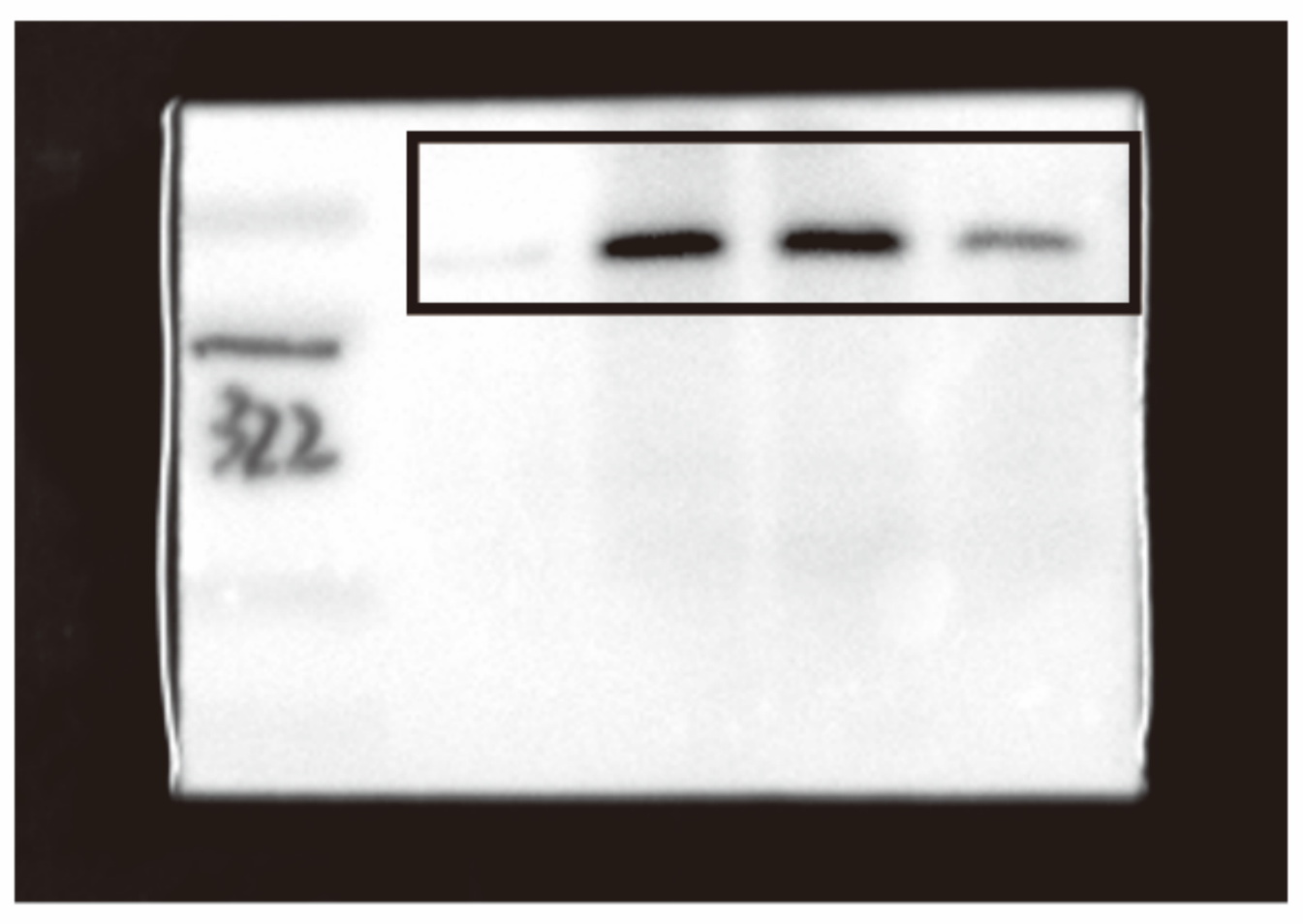

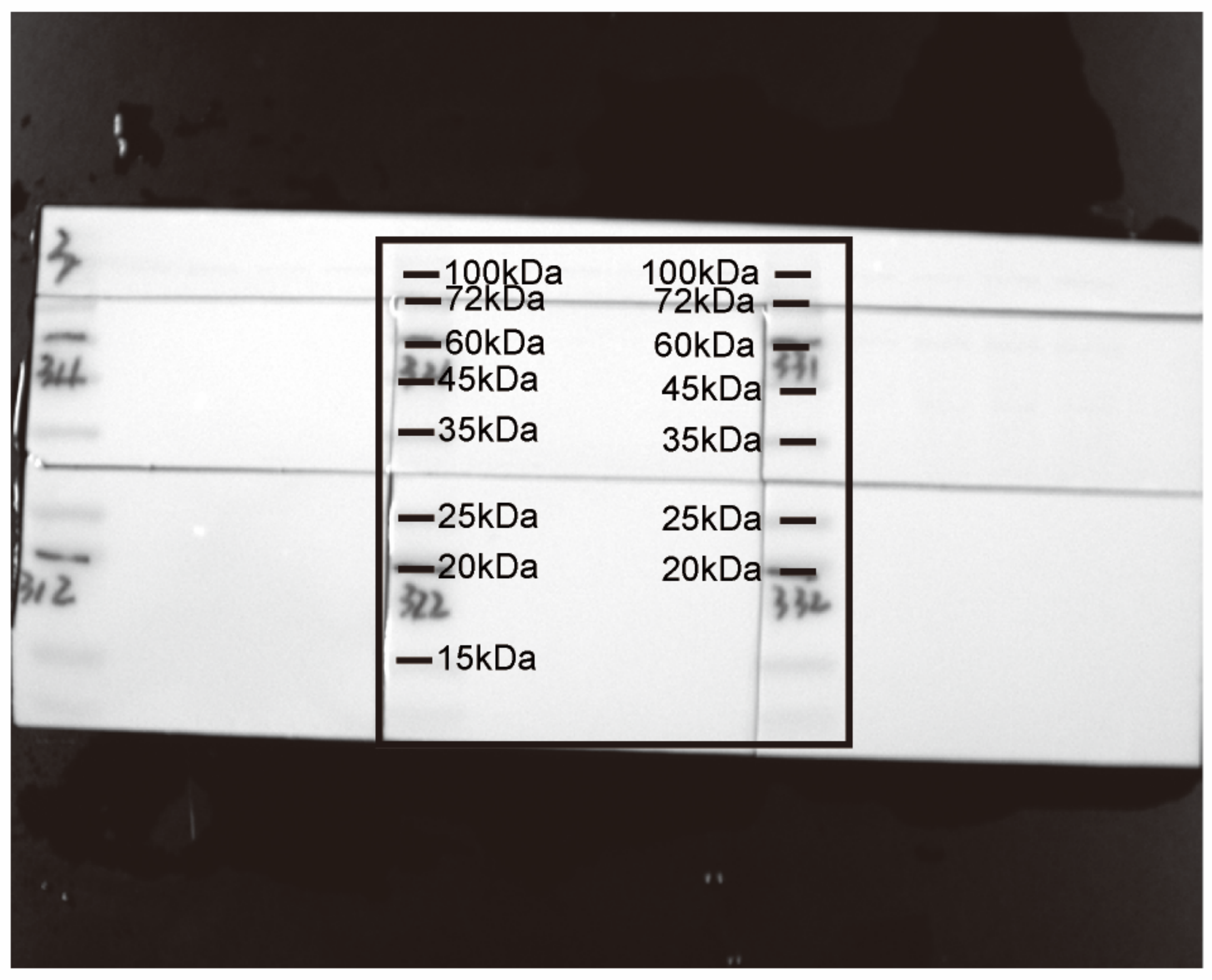

Fig.S4E

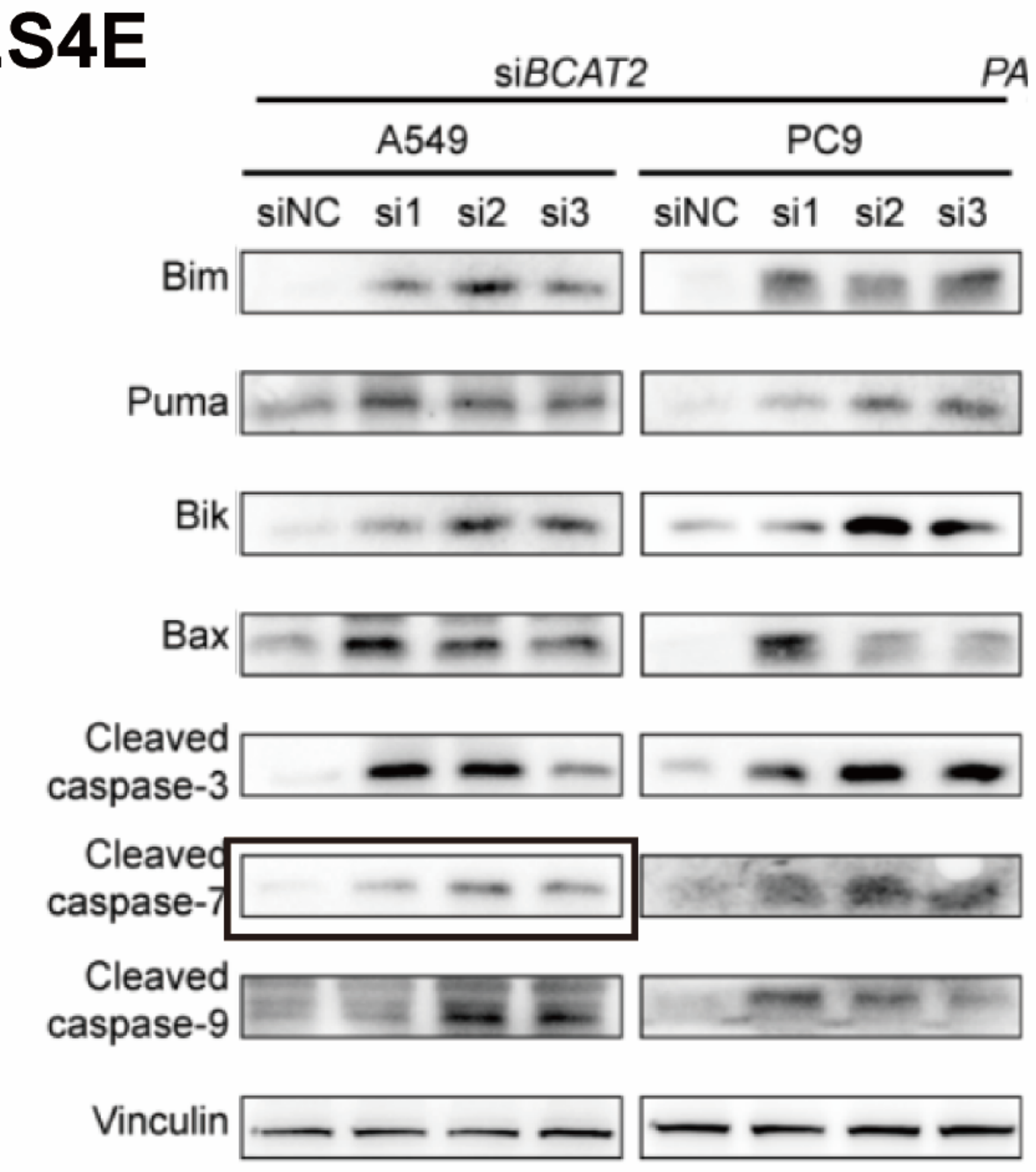

Vinculin

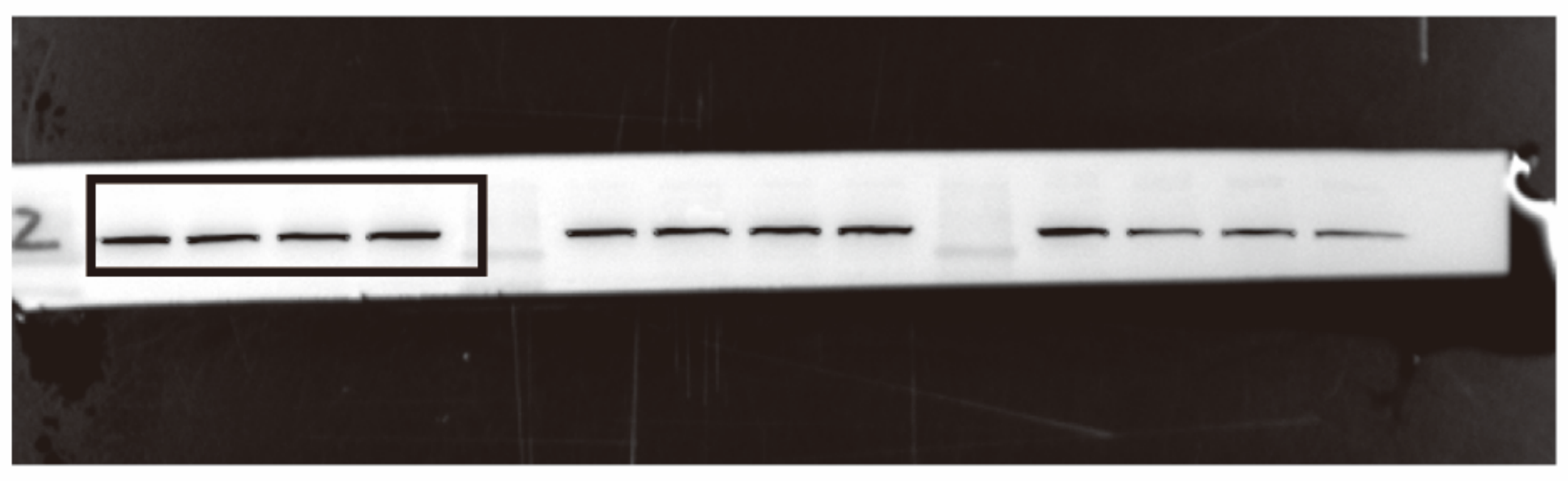

Cleaved caspase7

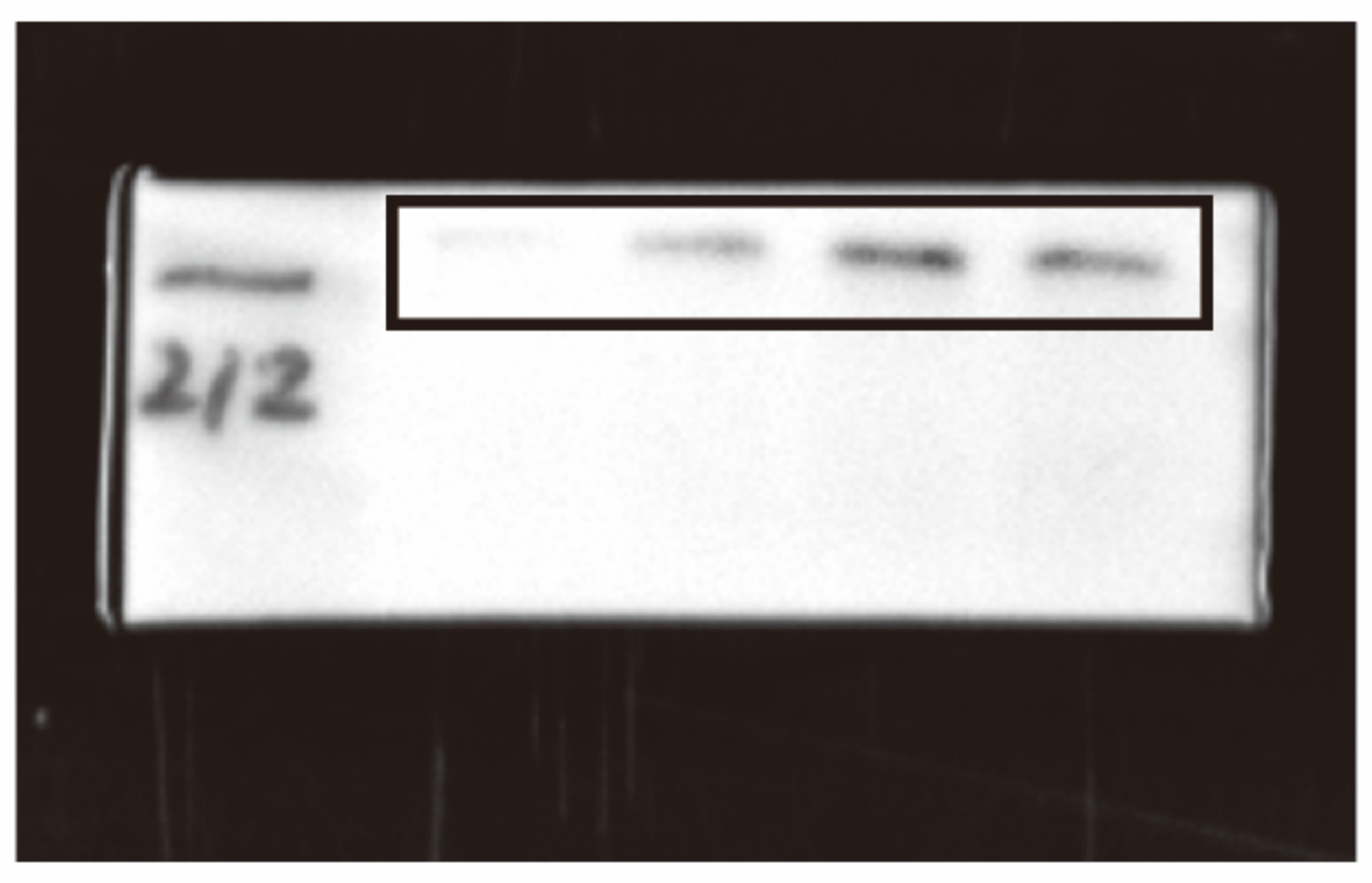

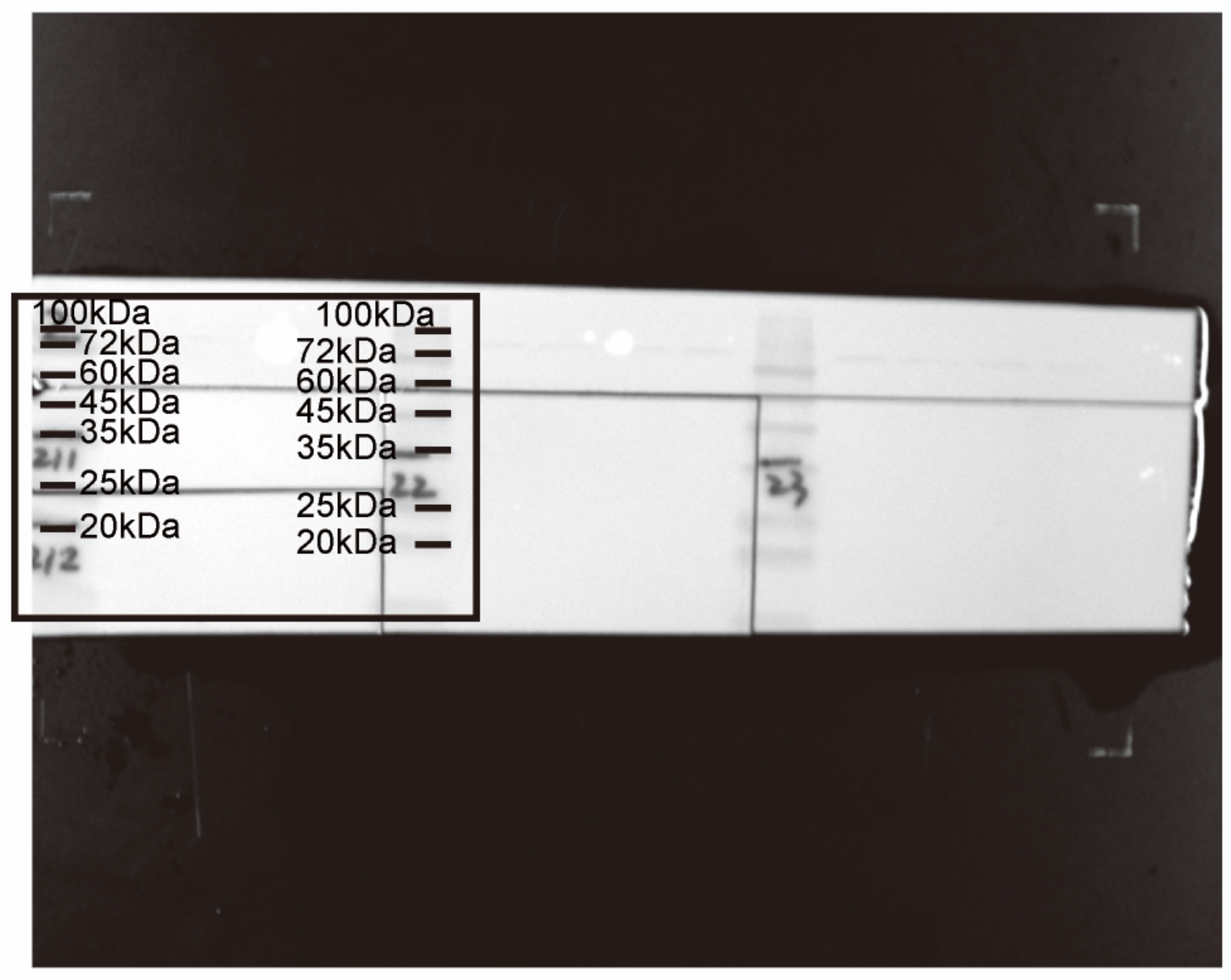

Fig.S4E

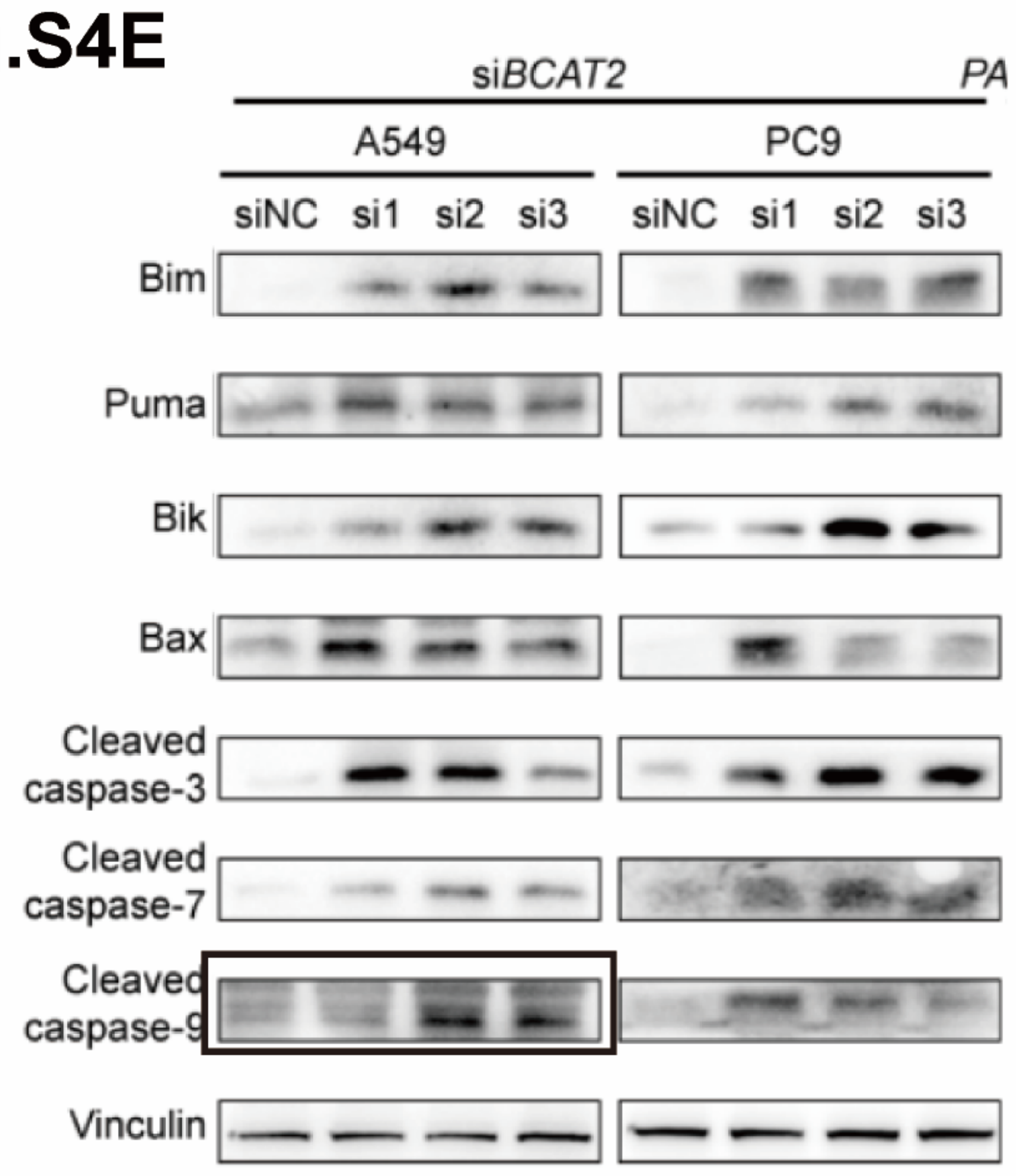

Vinculin

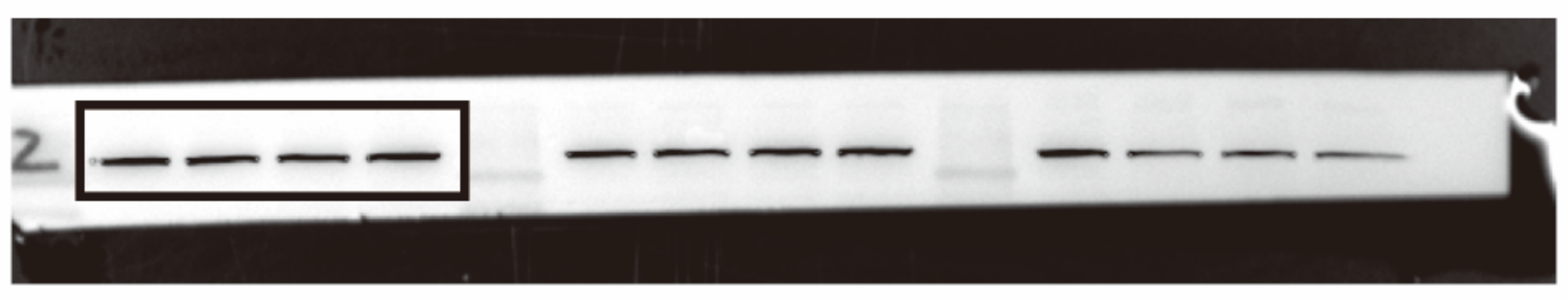

Cleaved caspase9

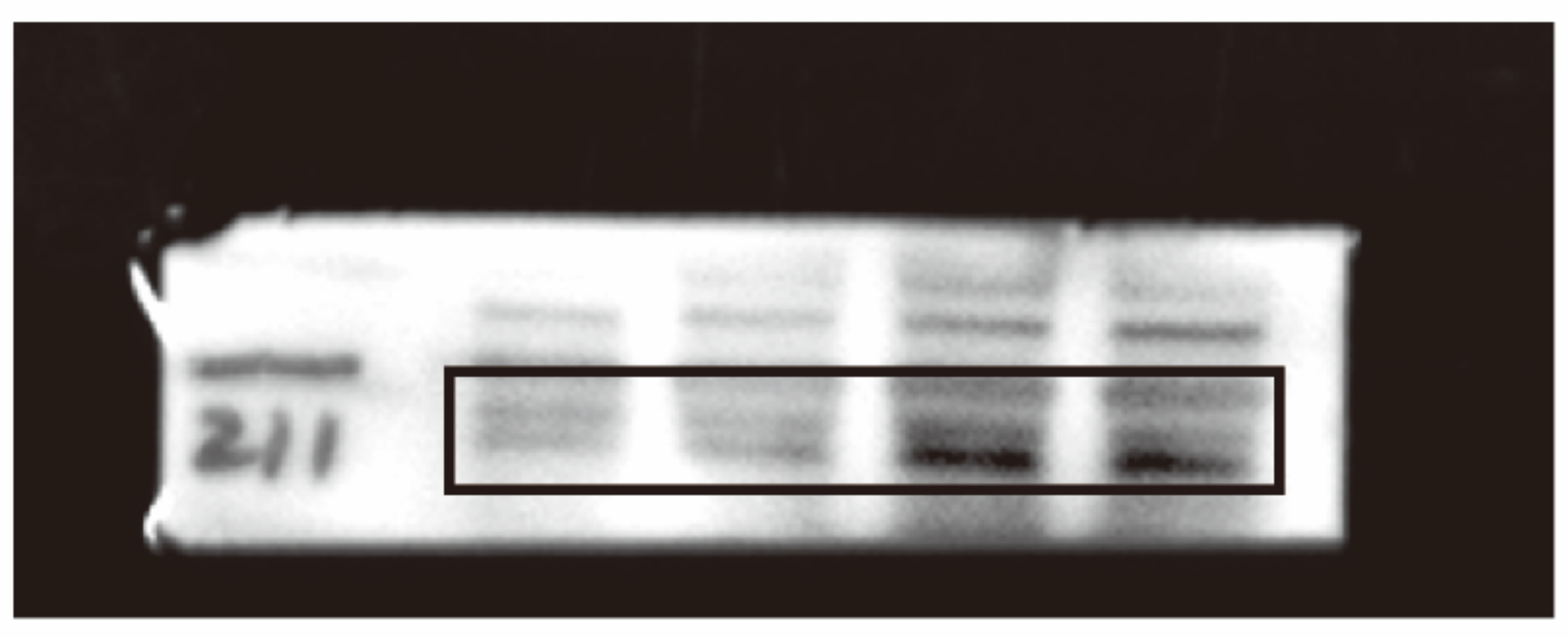

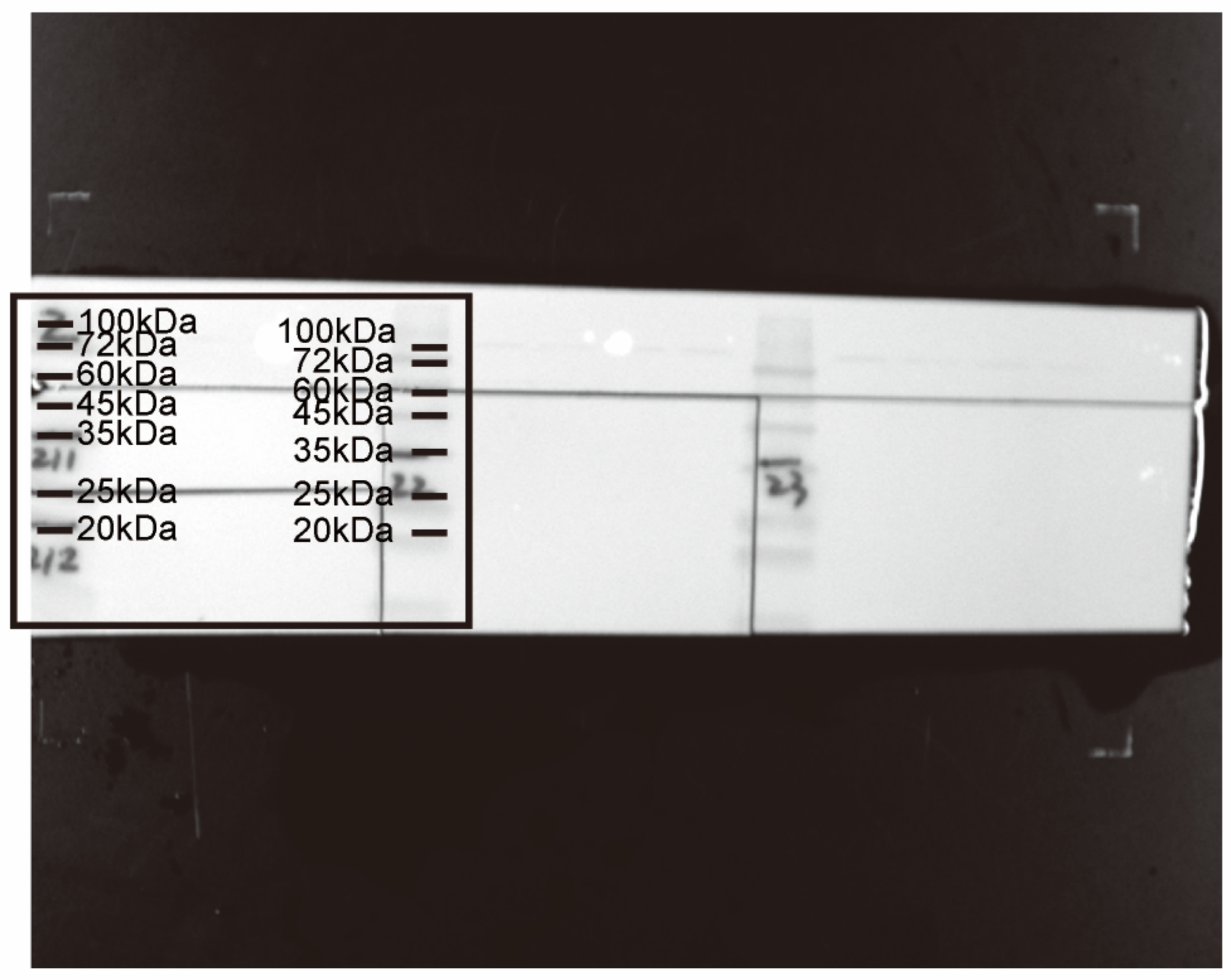

Fig.S4E

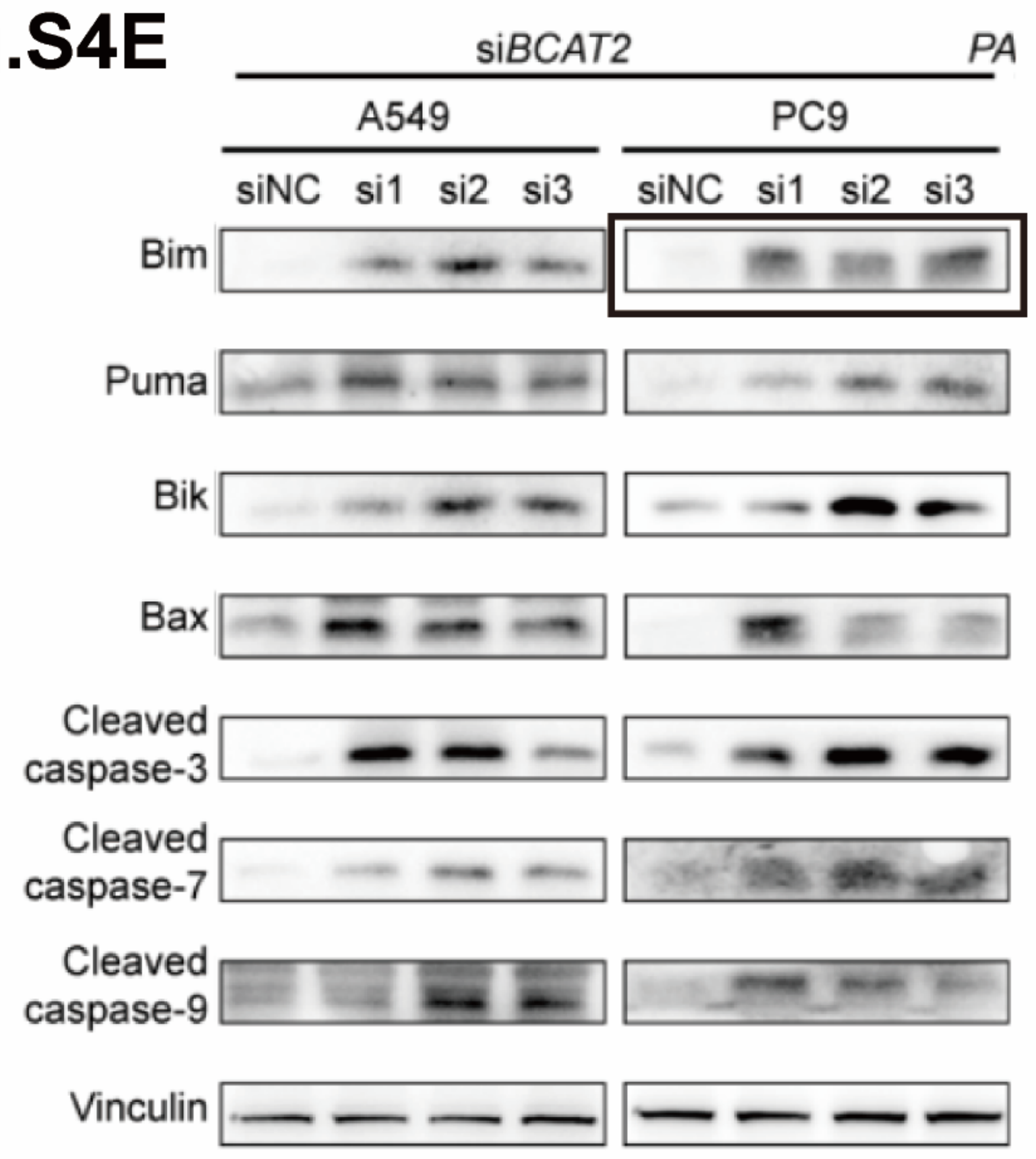

Vinculin

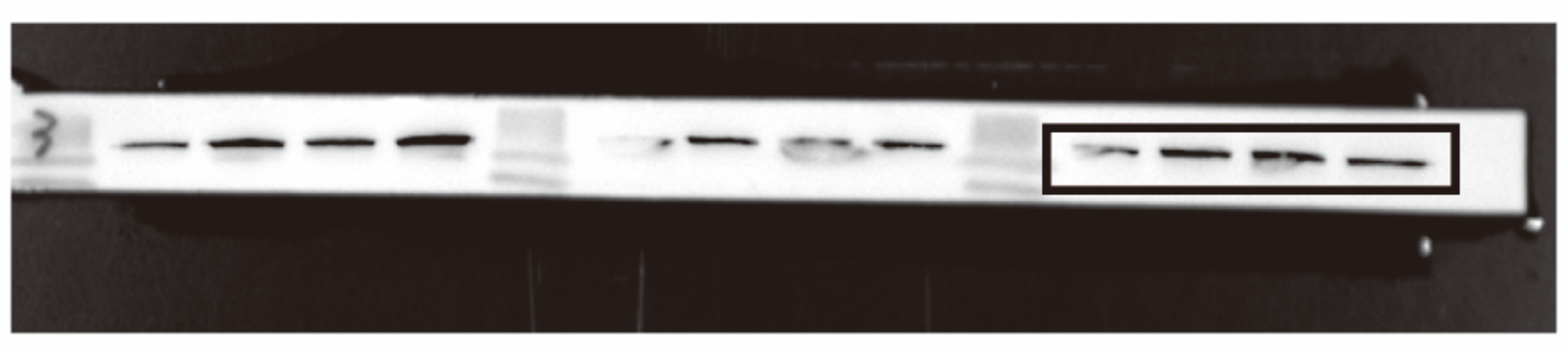

Bim

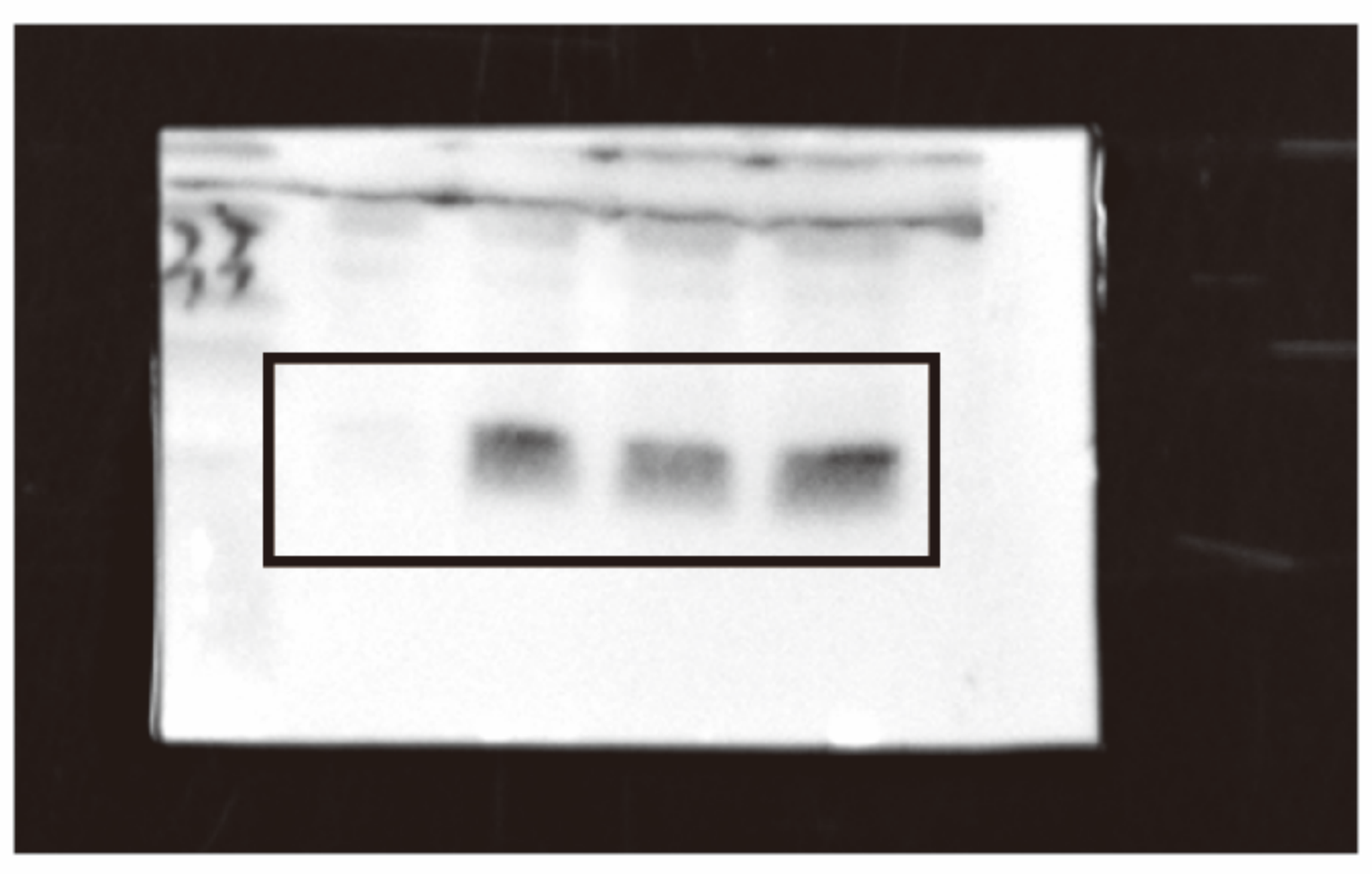

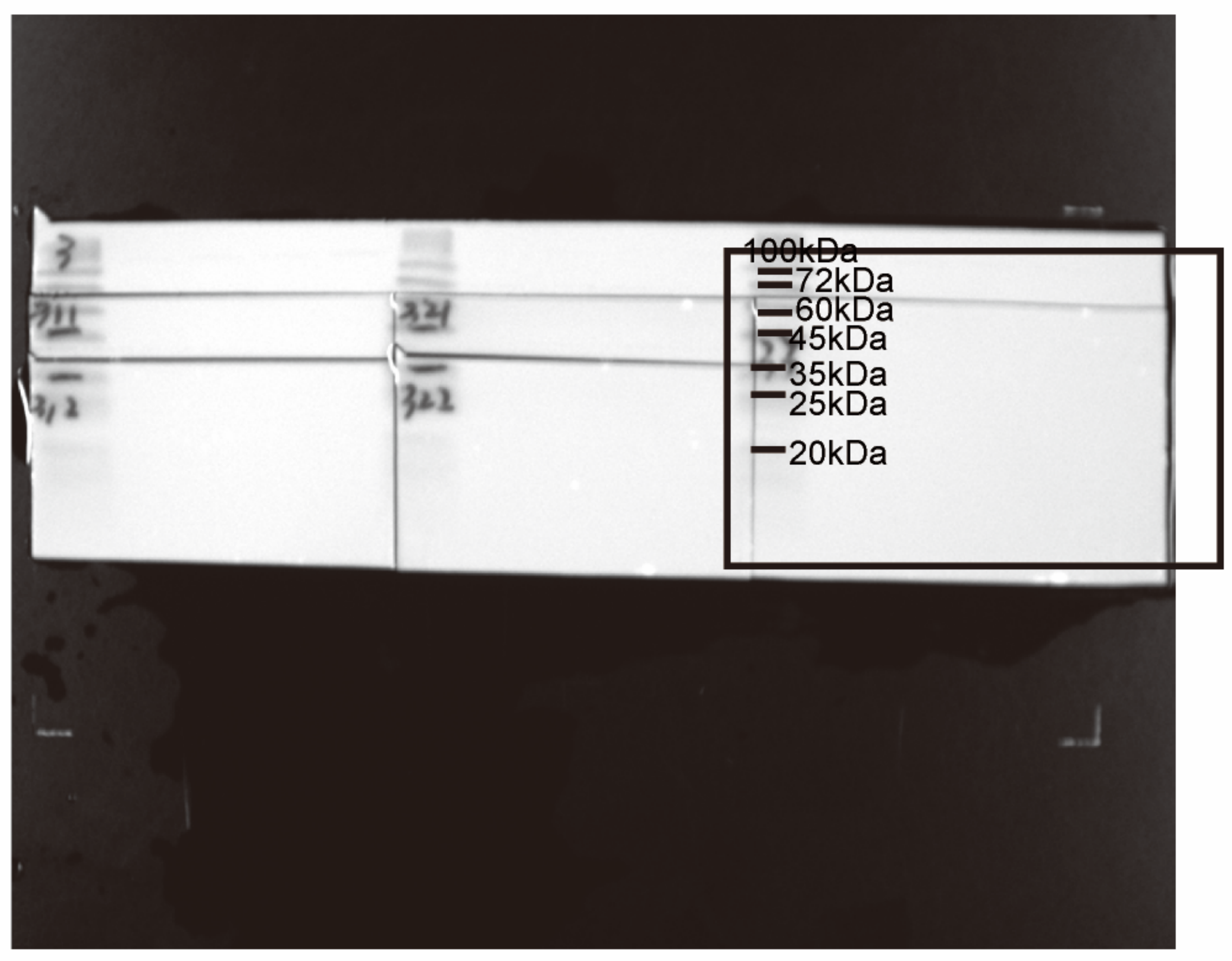

Figure	Merged	Stiched full image

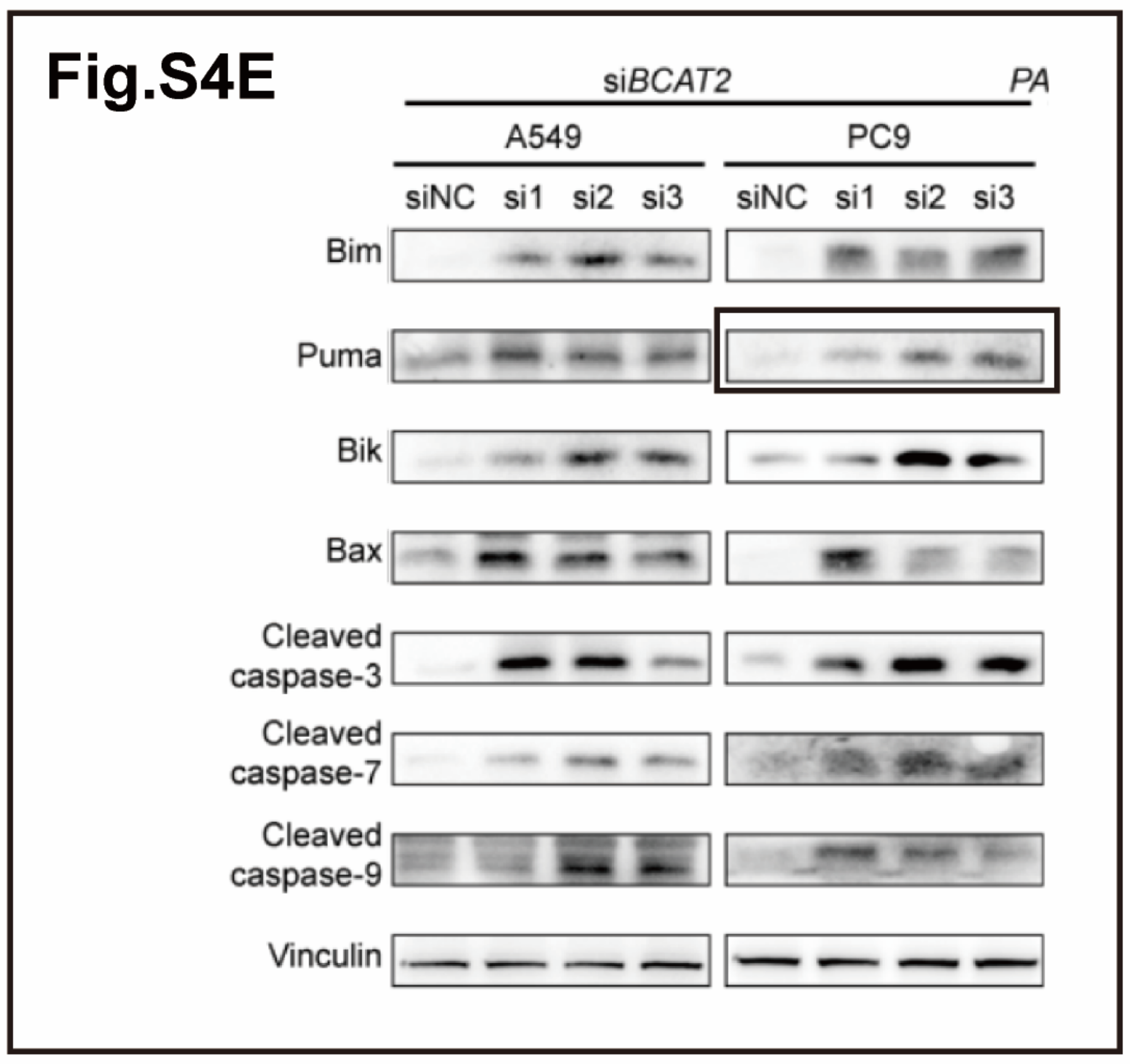

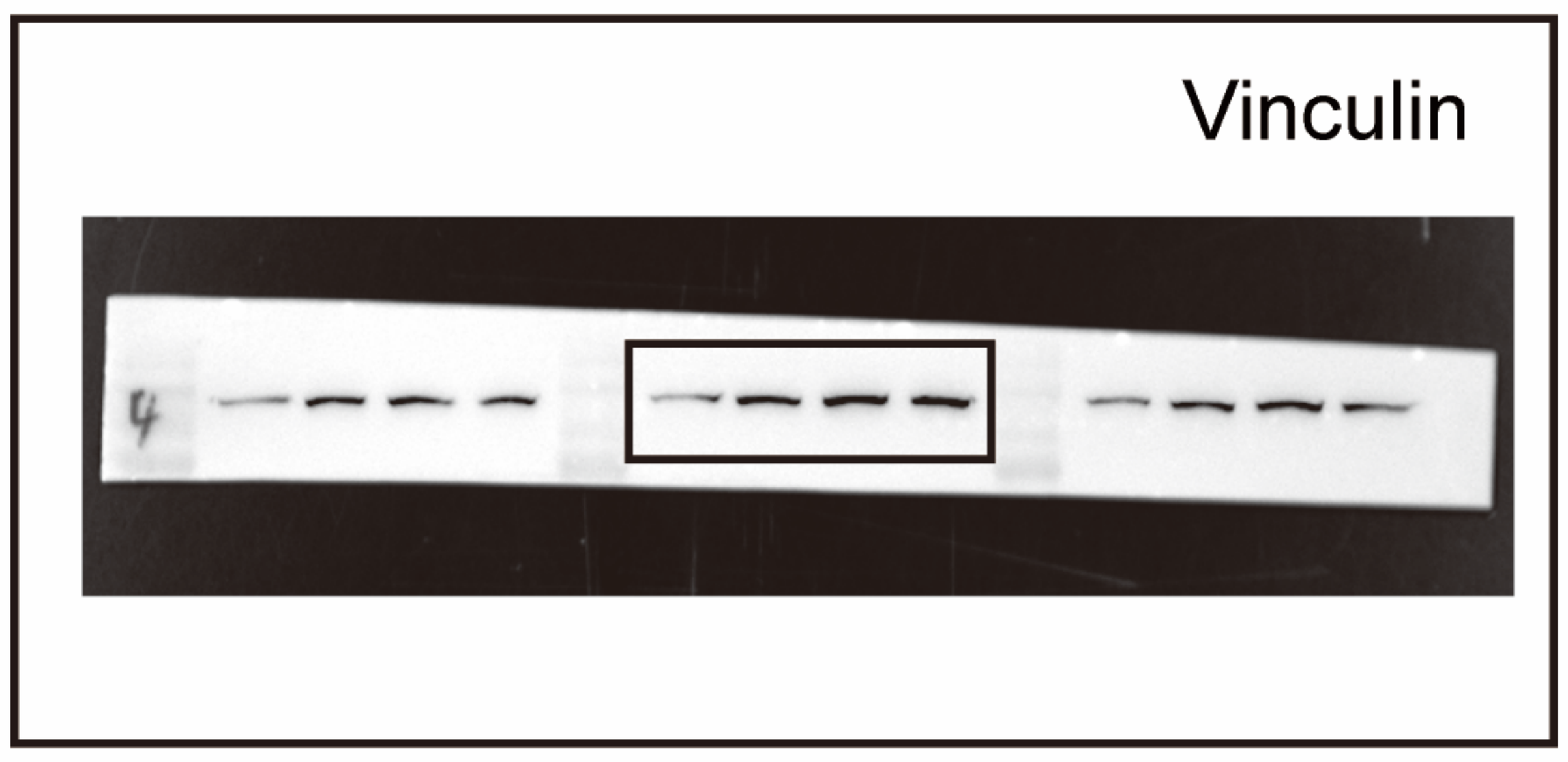

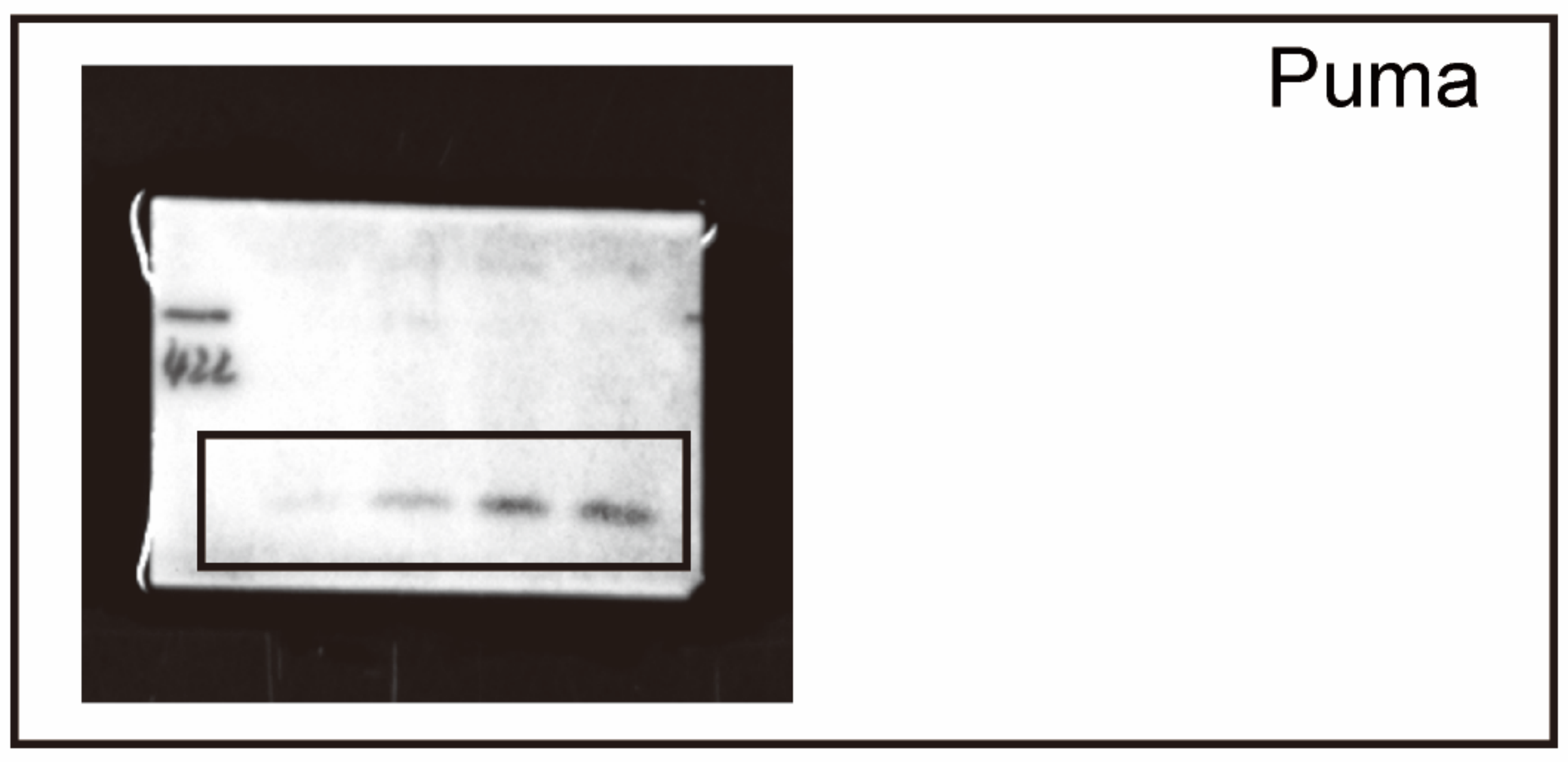

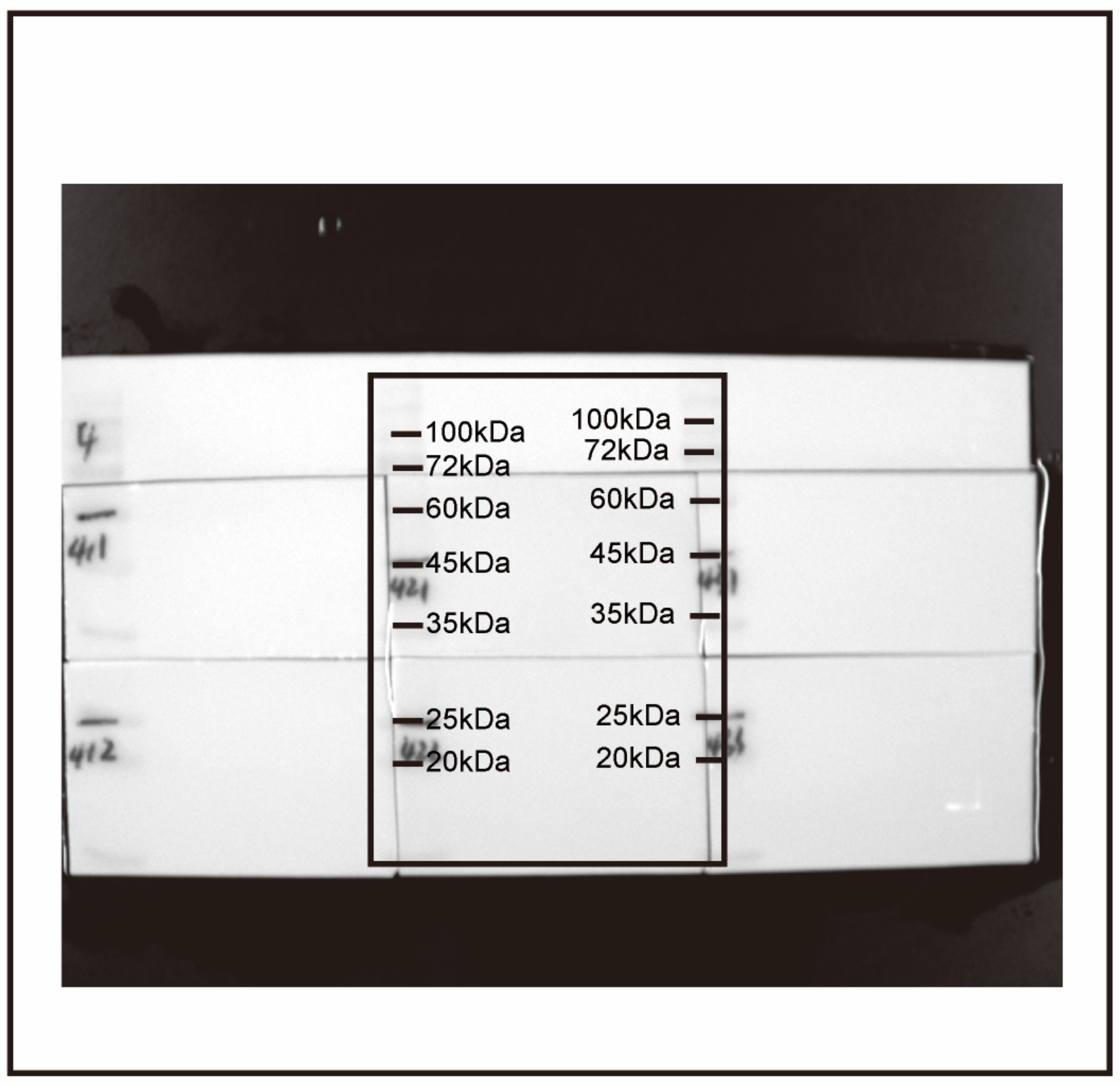

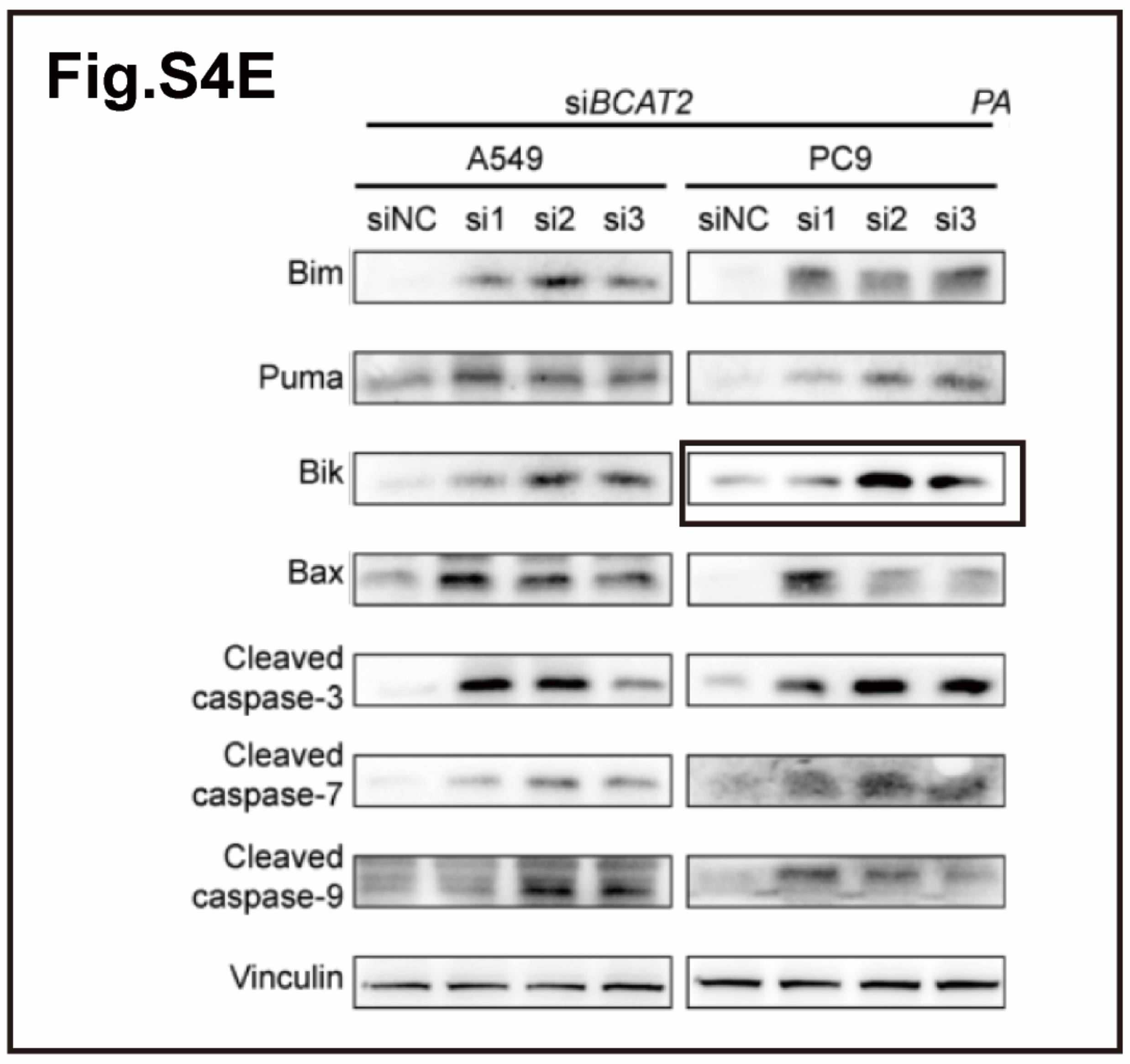

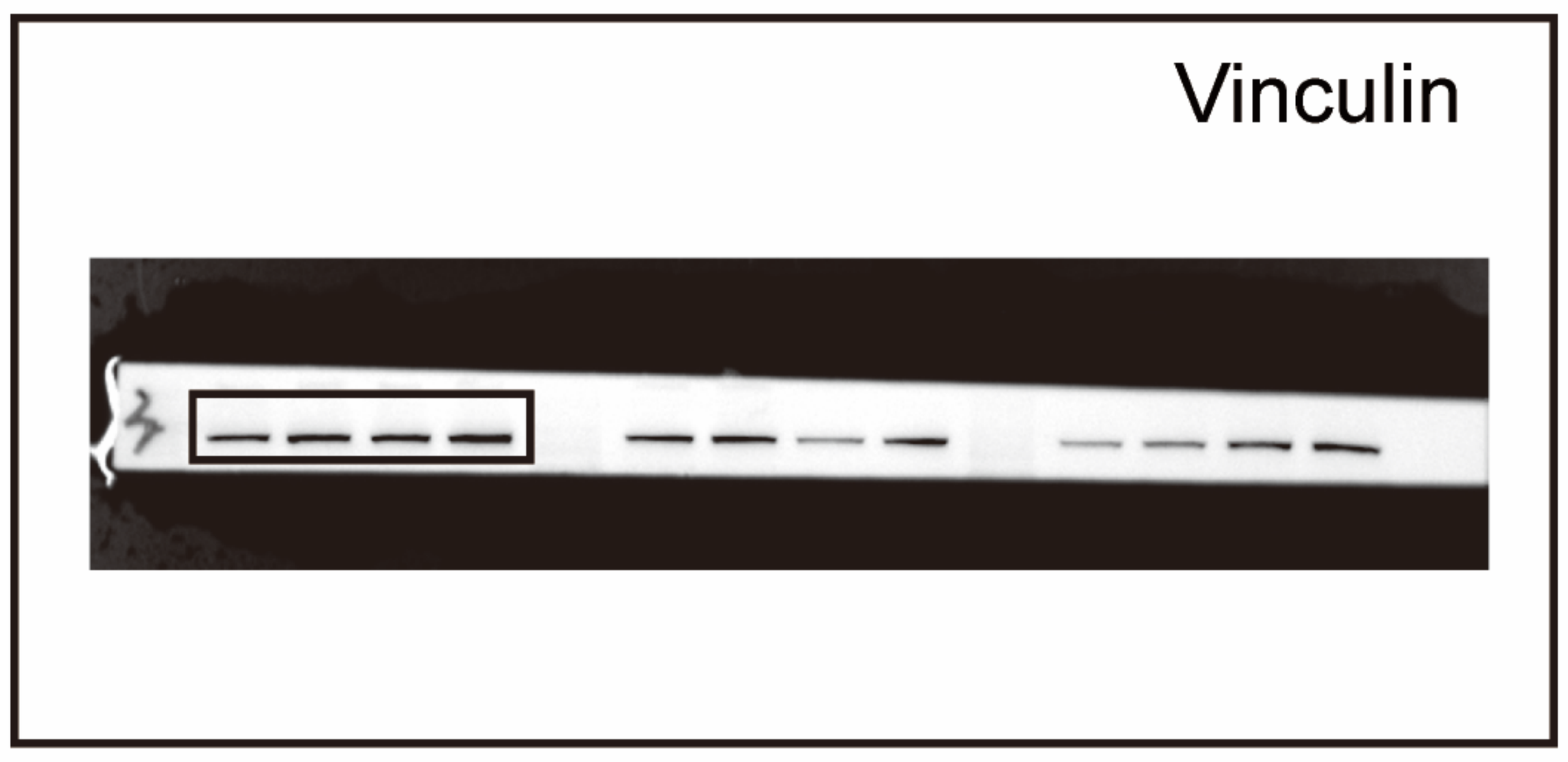

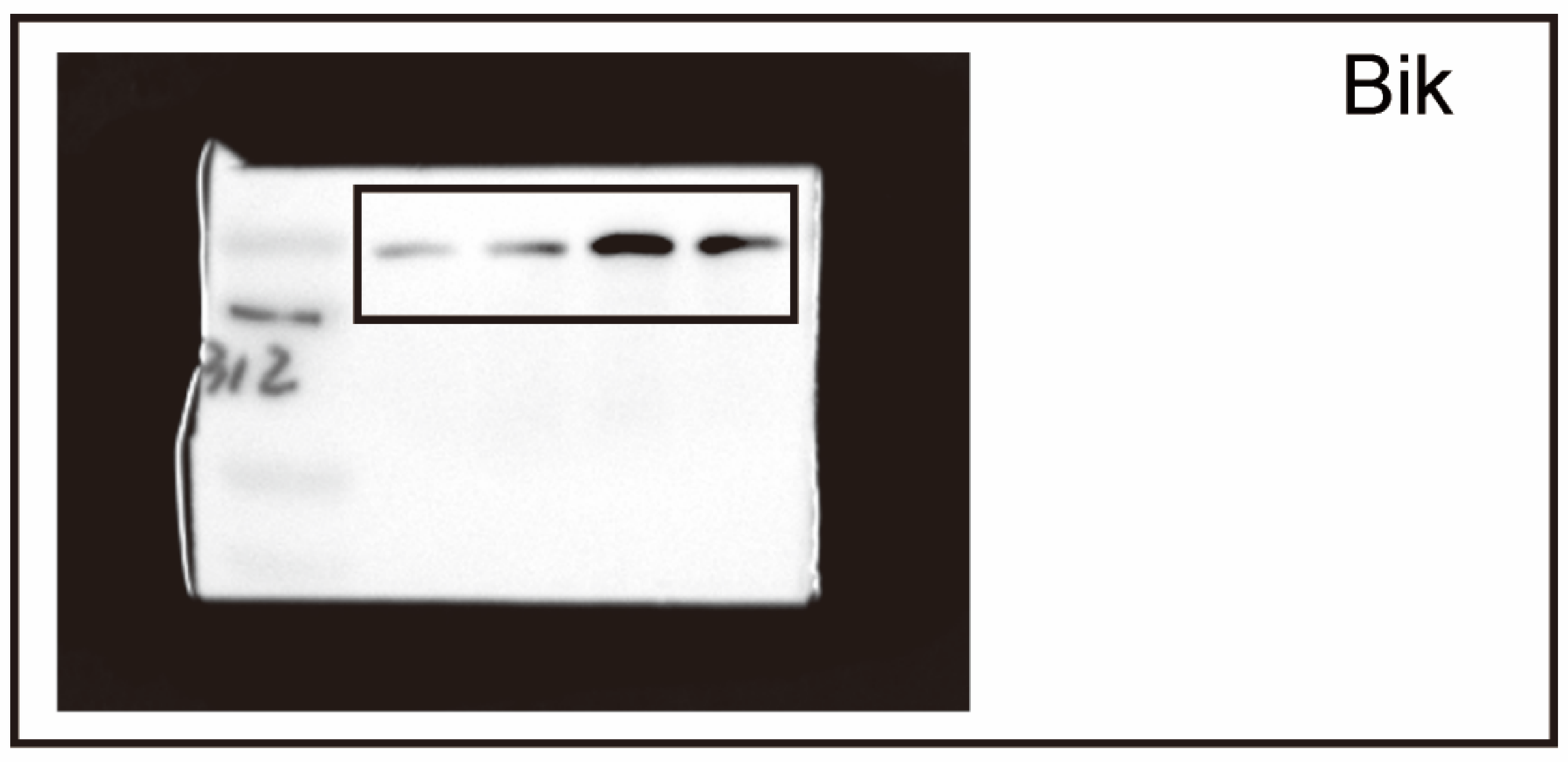

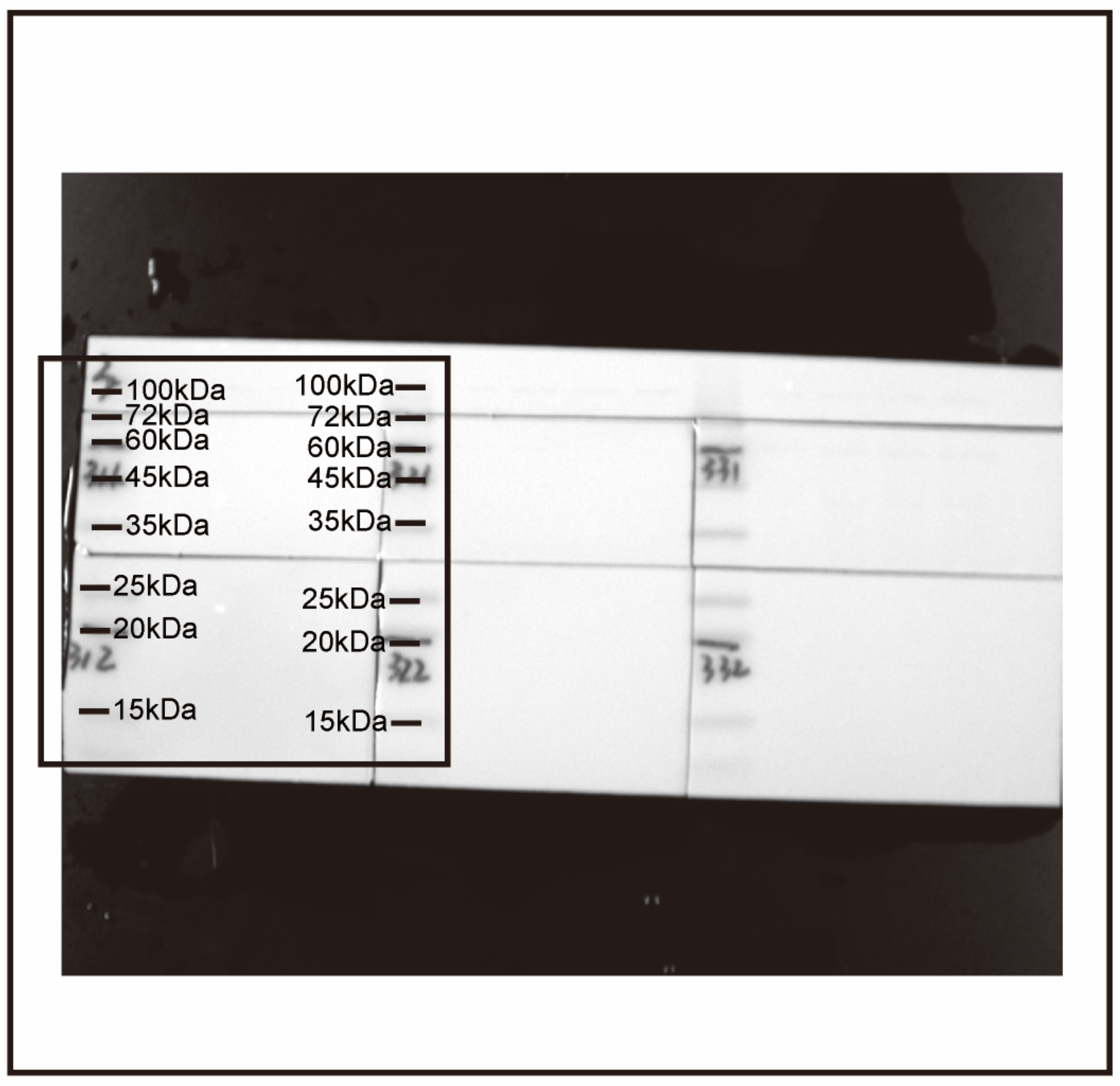

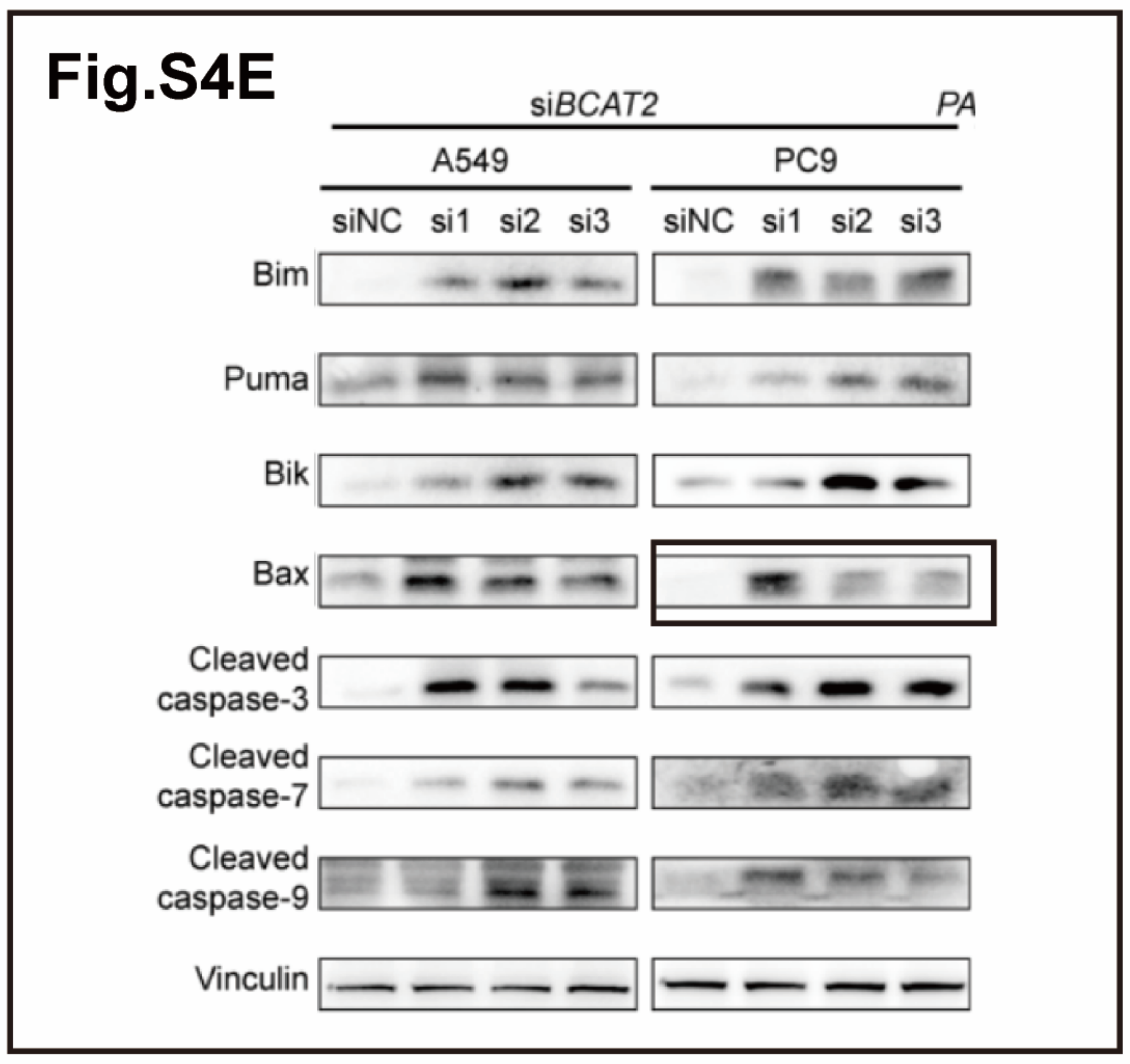

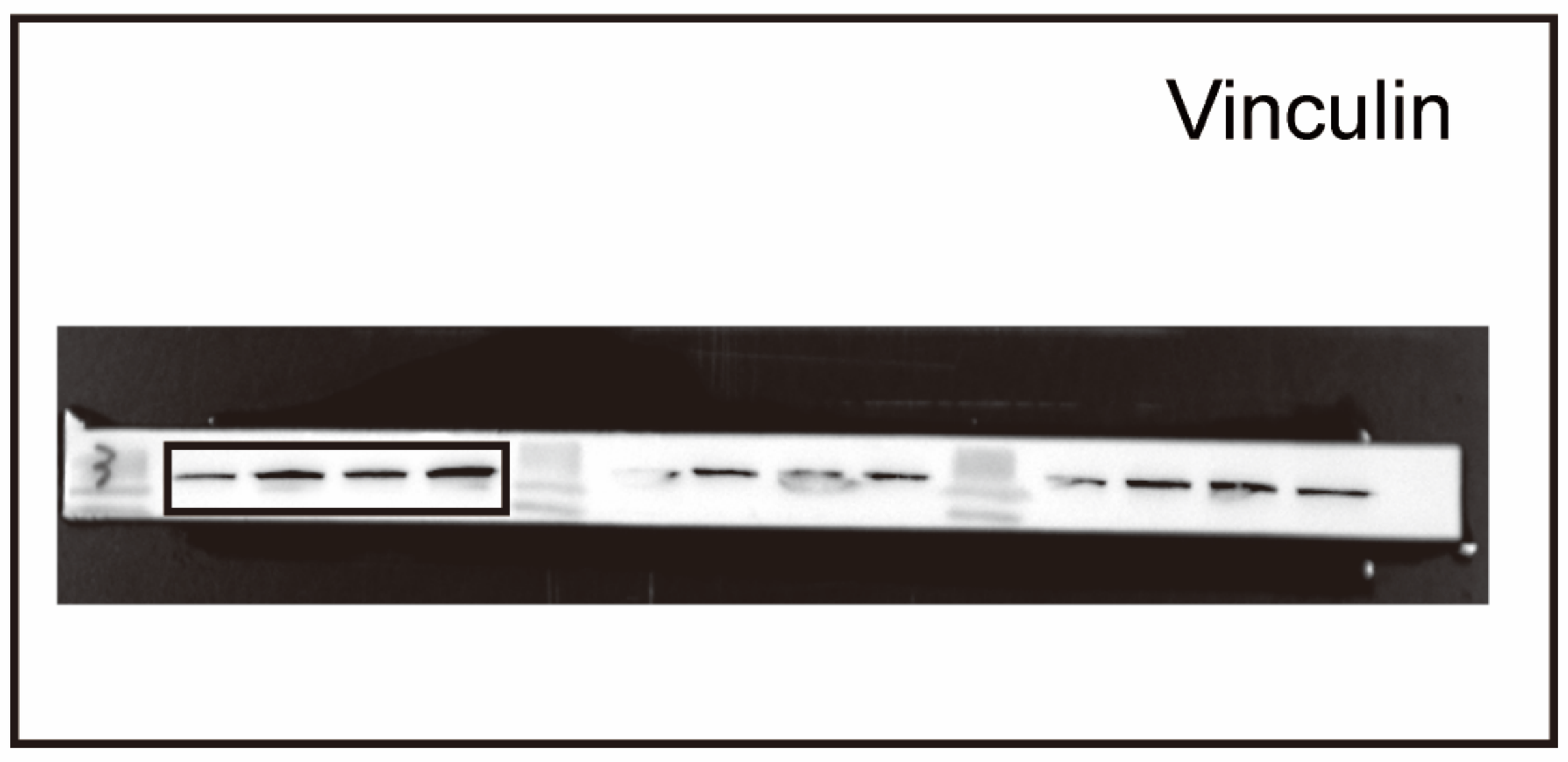

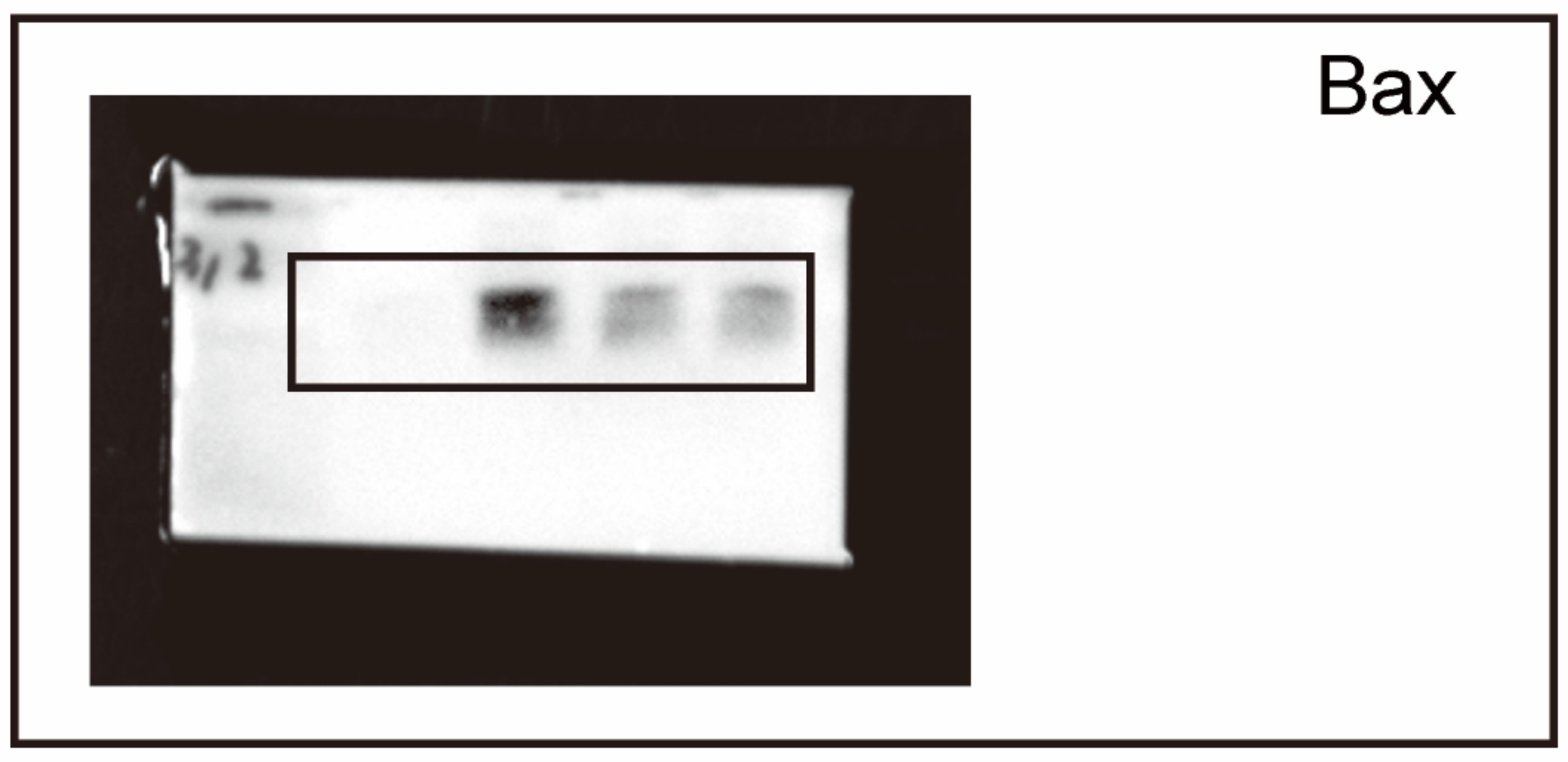

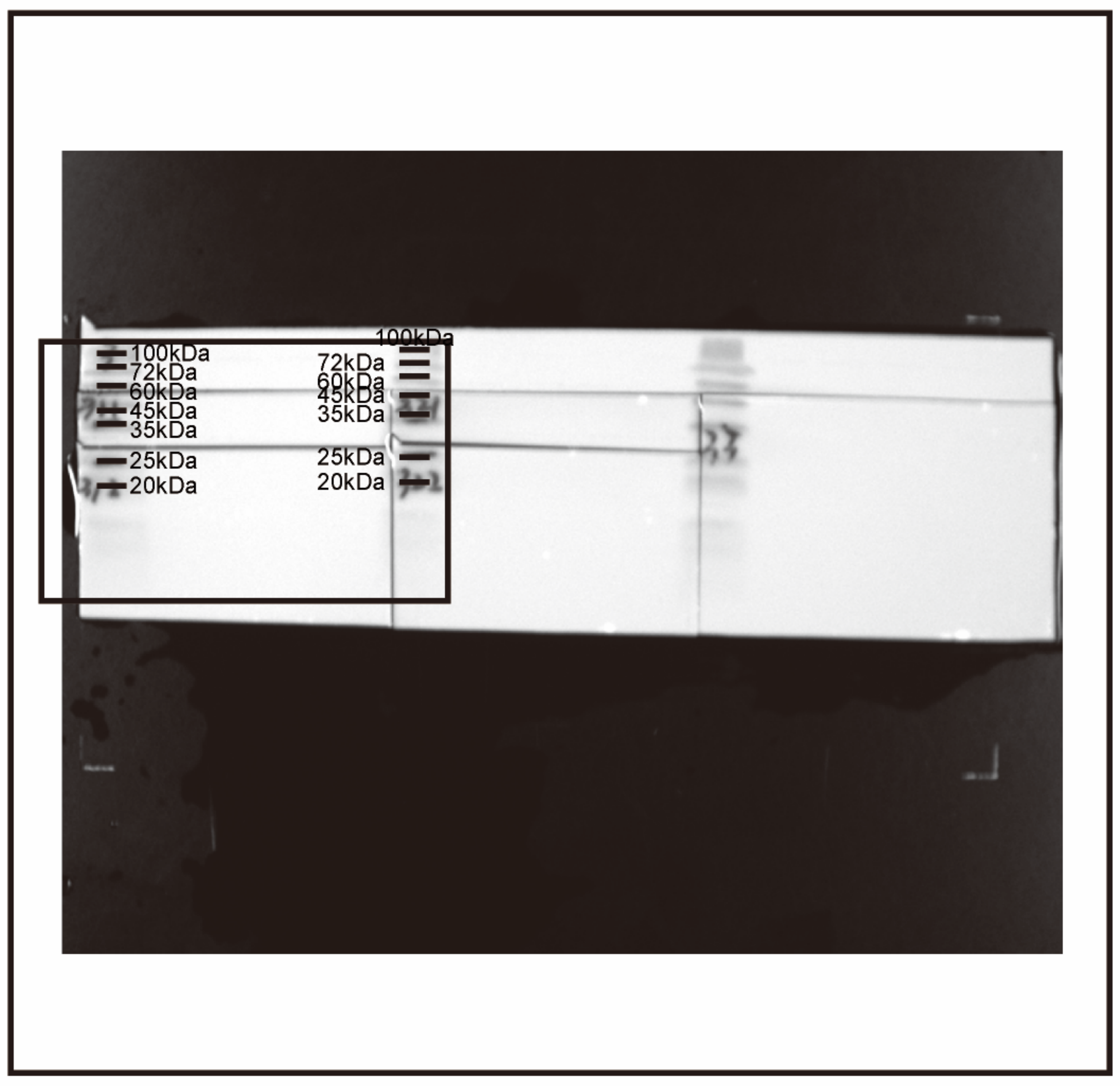

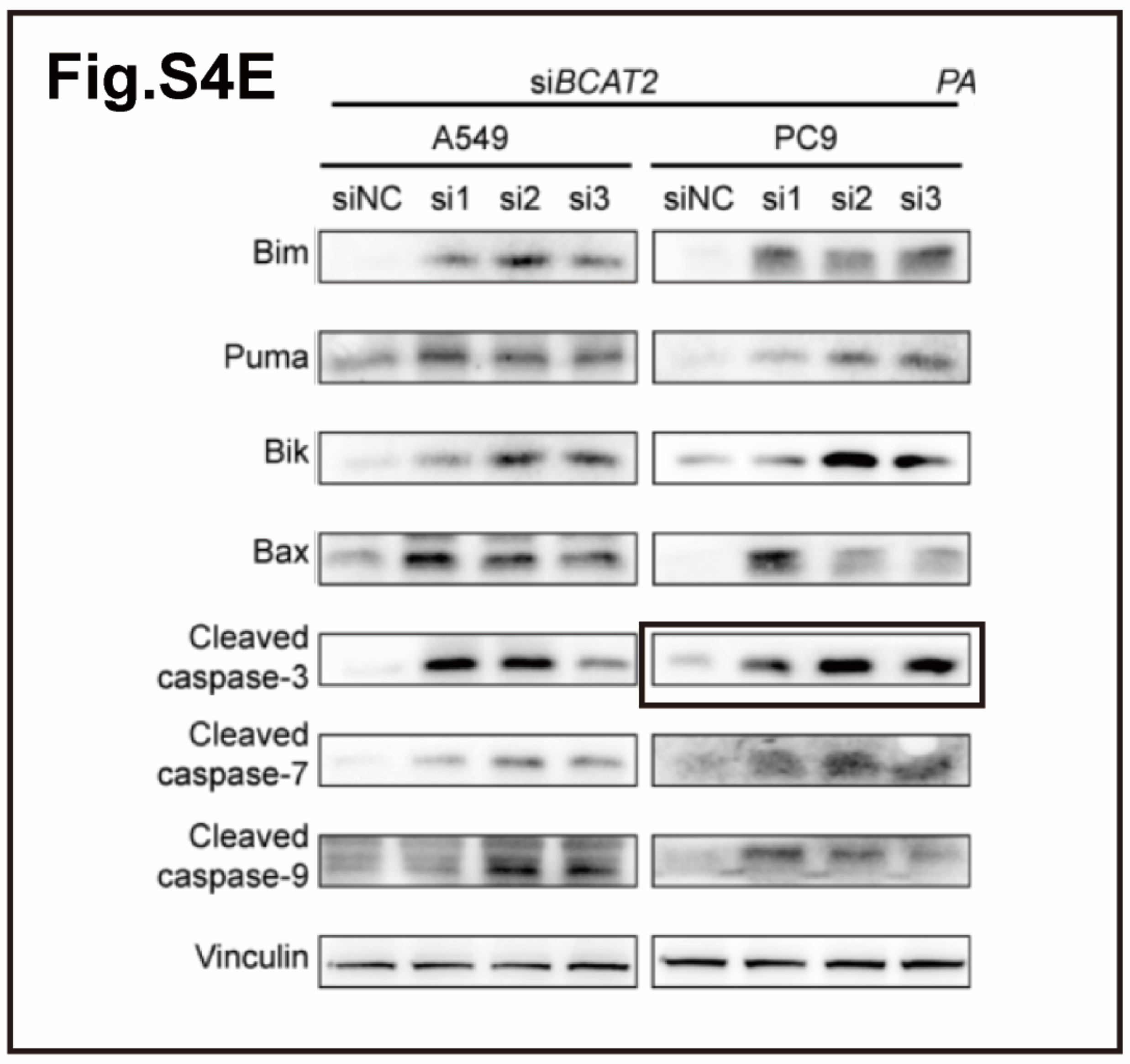

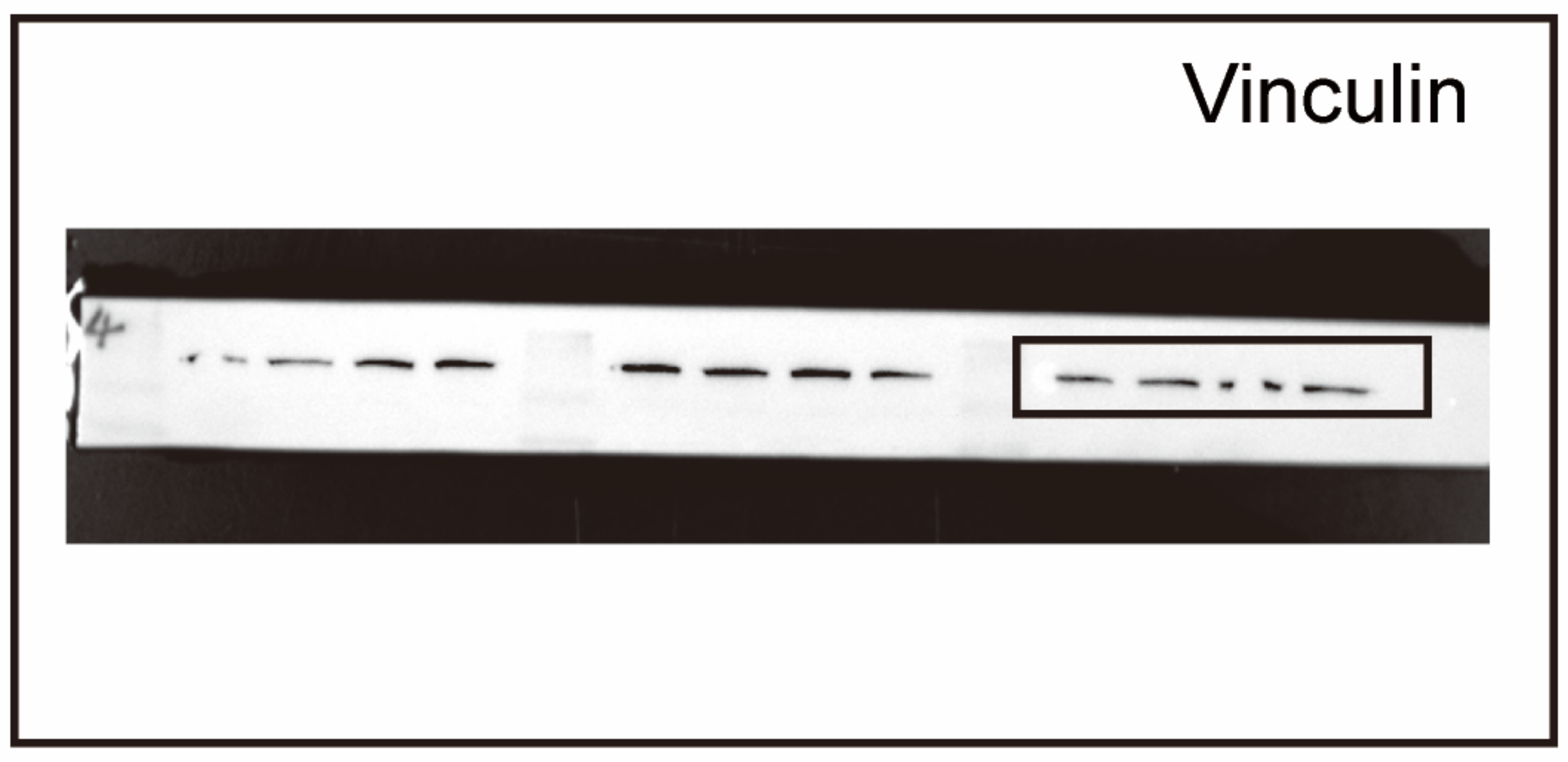

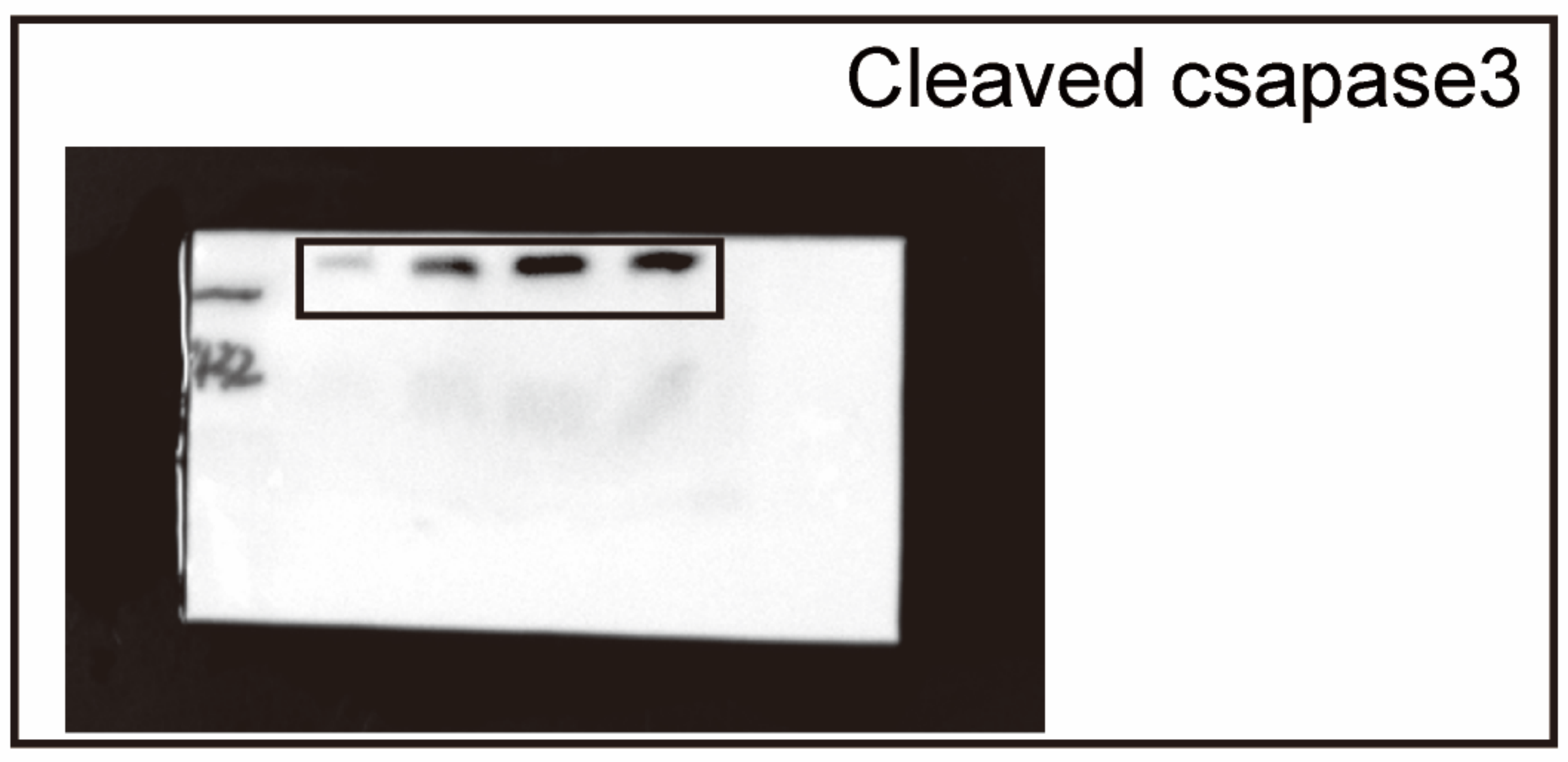

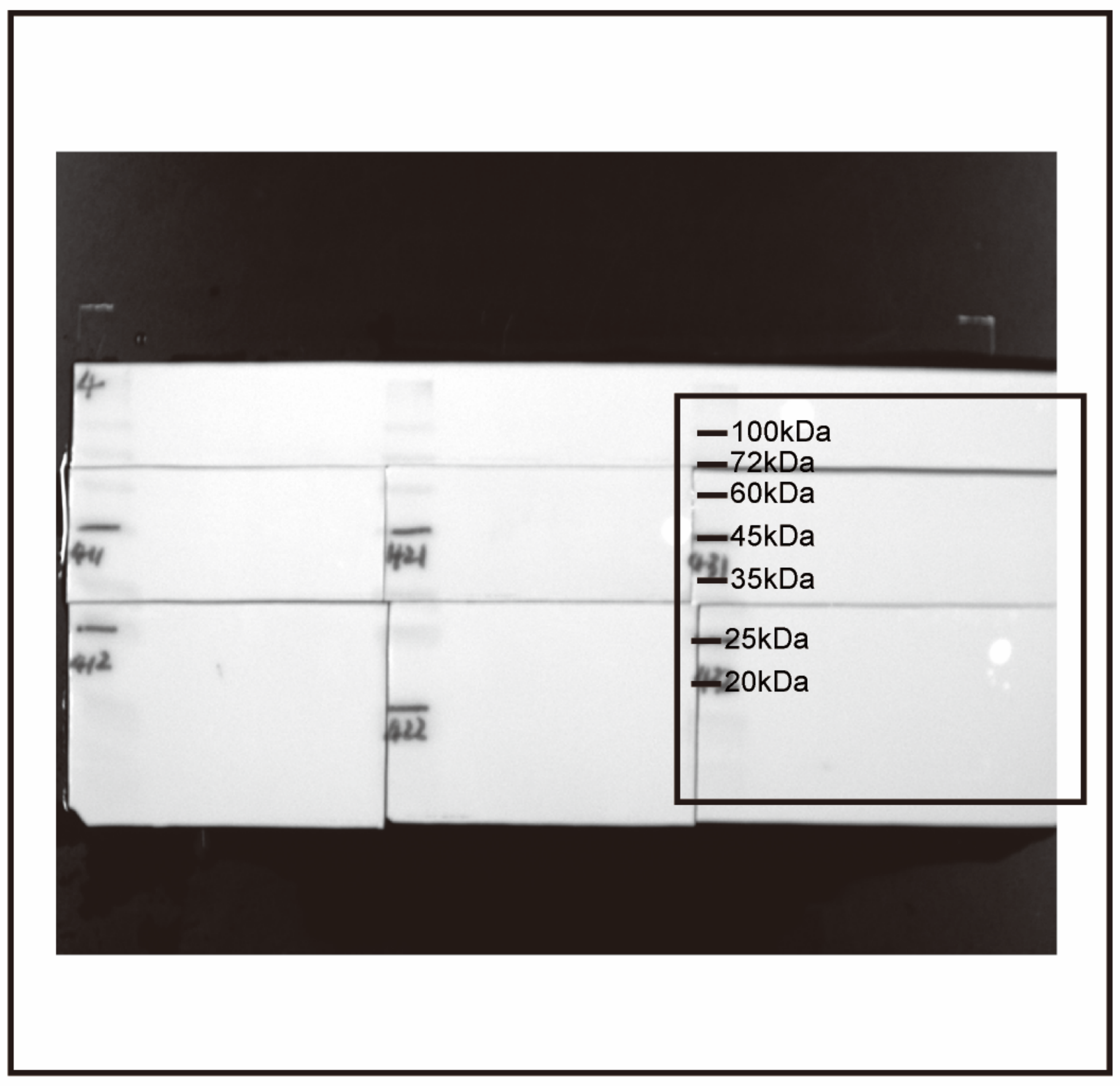

Figure	Merged	Stiched full image

Fig.S4E

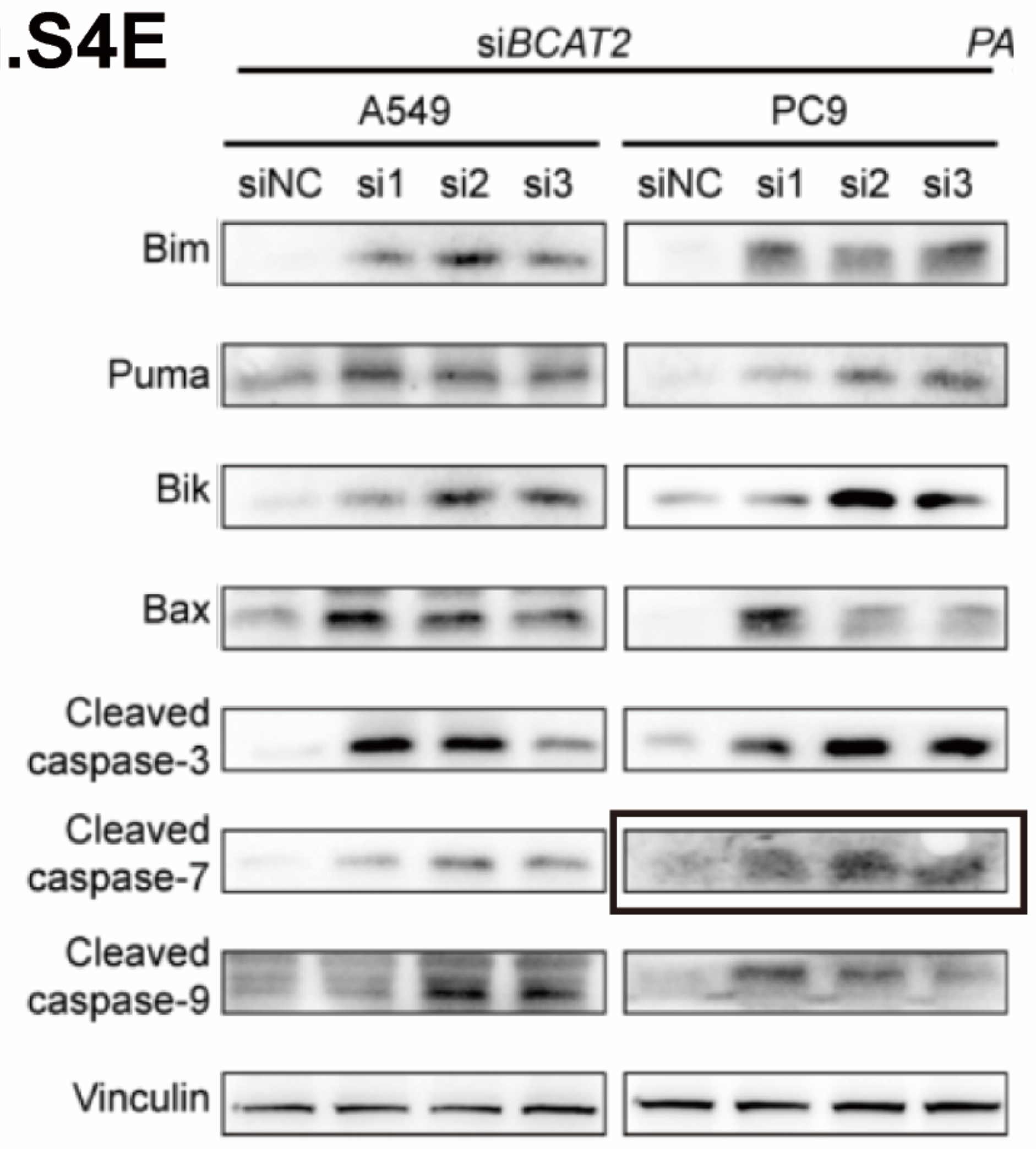

Vinculin

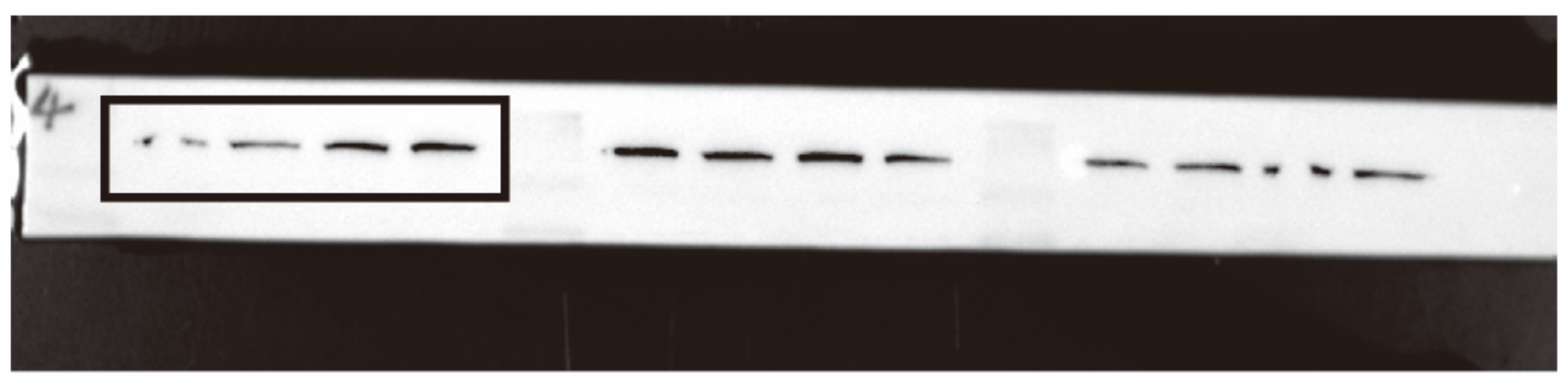

Cleaved caspase7

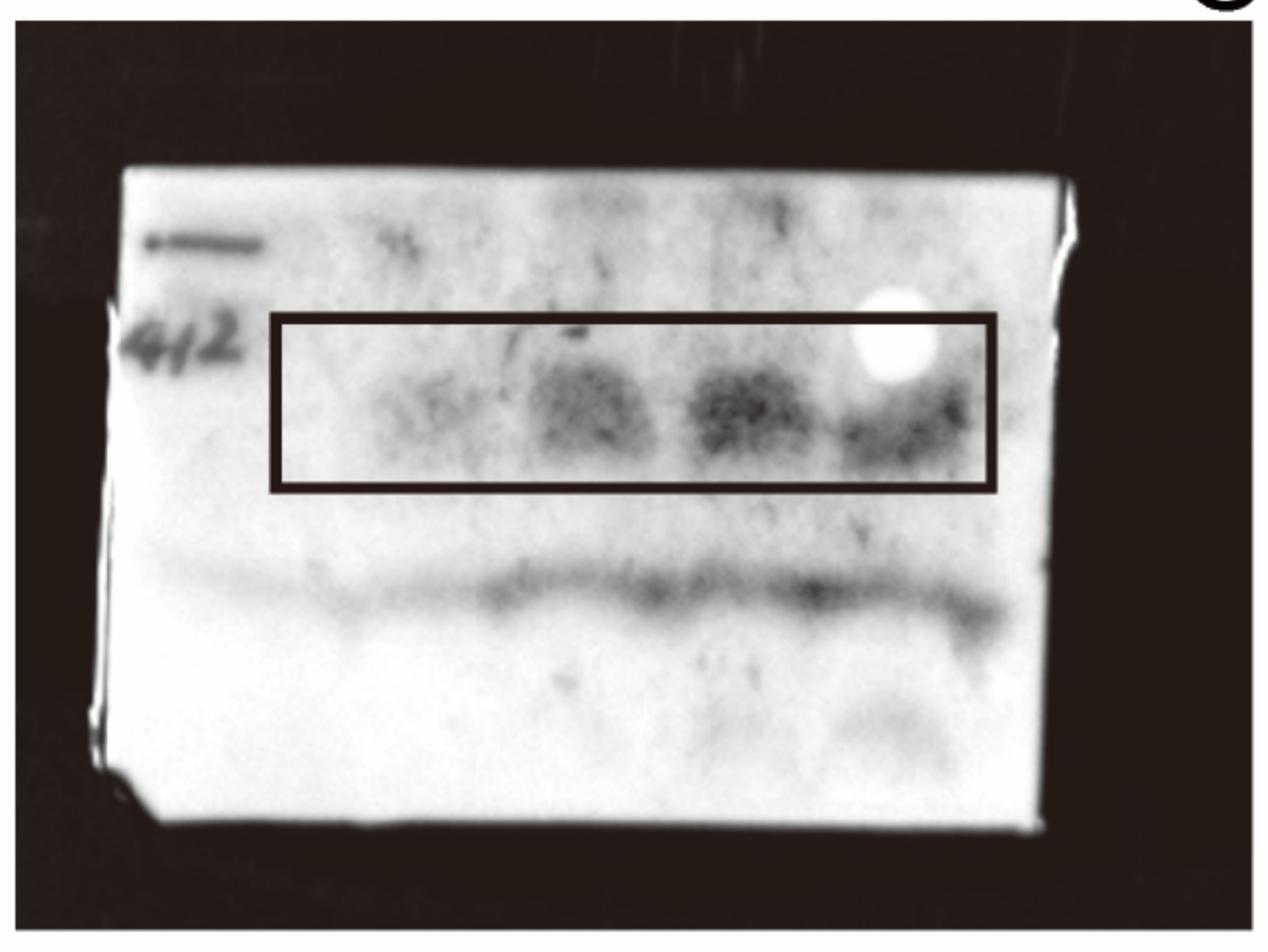

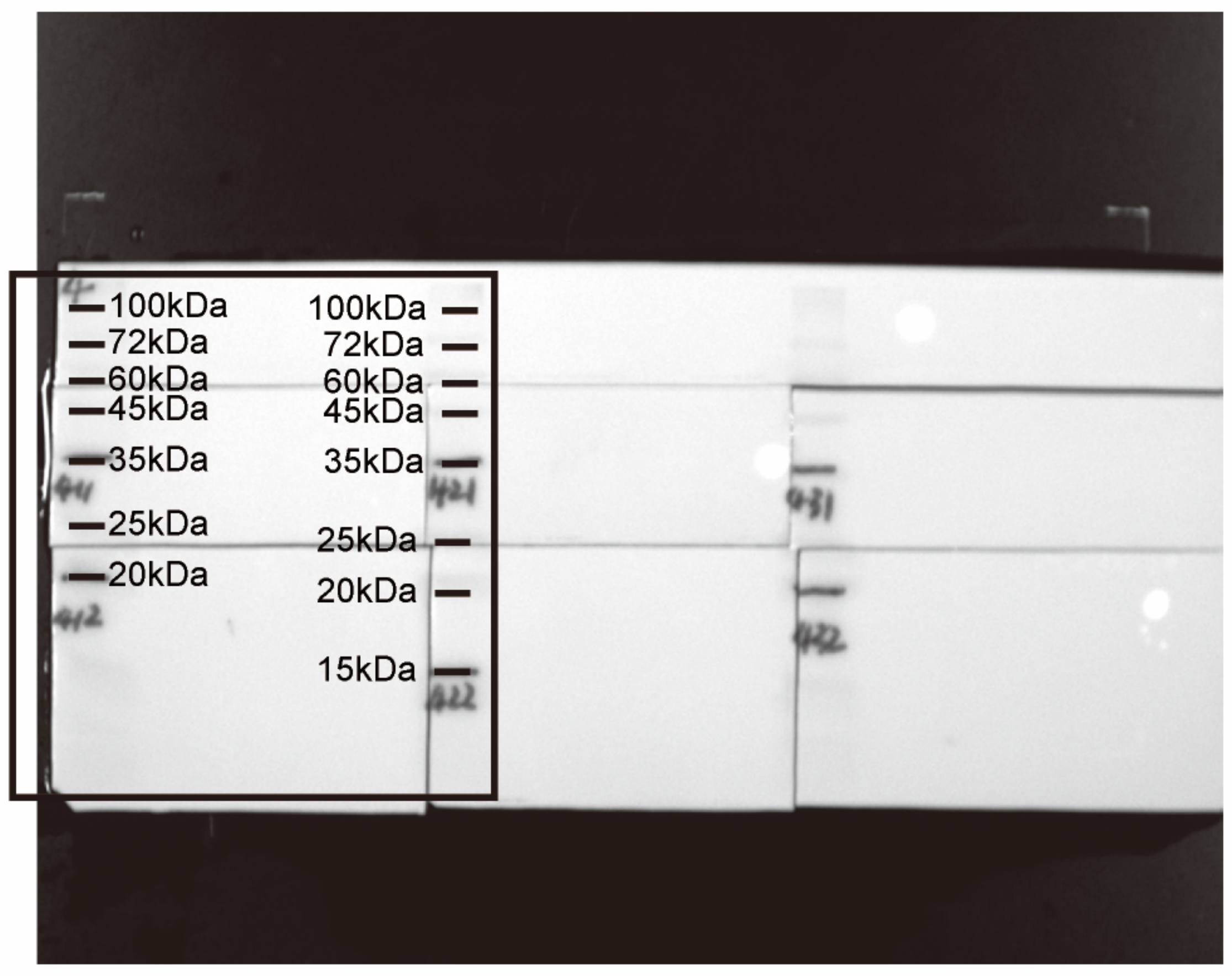

Fig.S4E

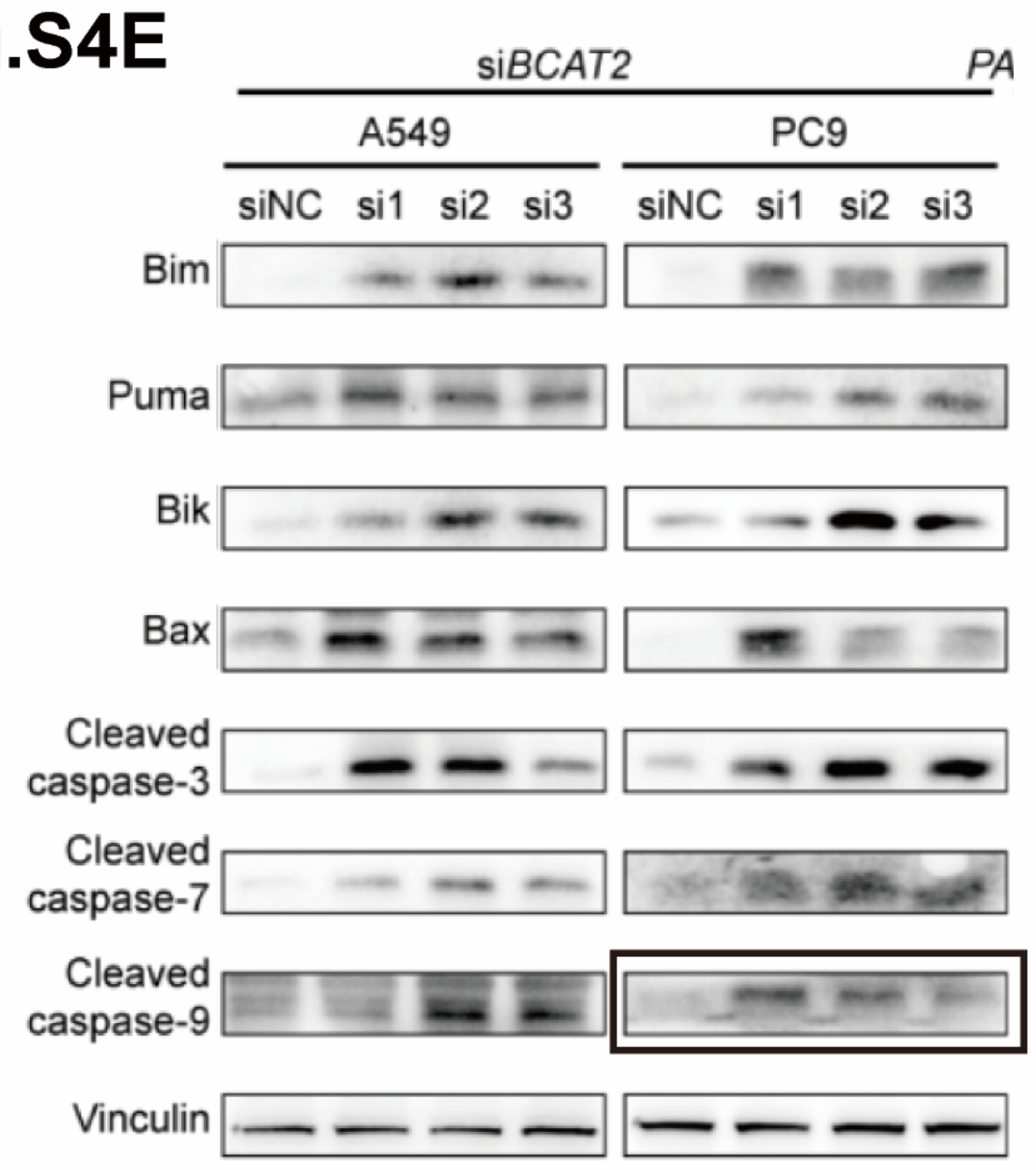

Vinculin

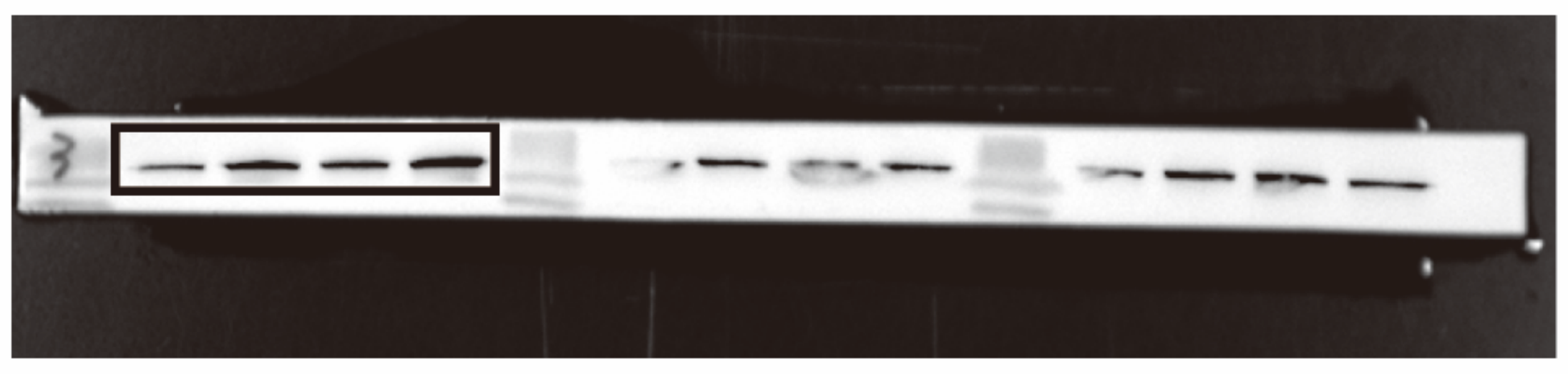

Cleaved caspase9

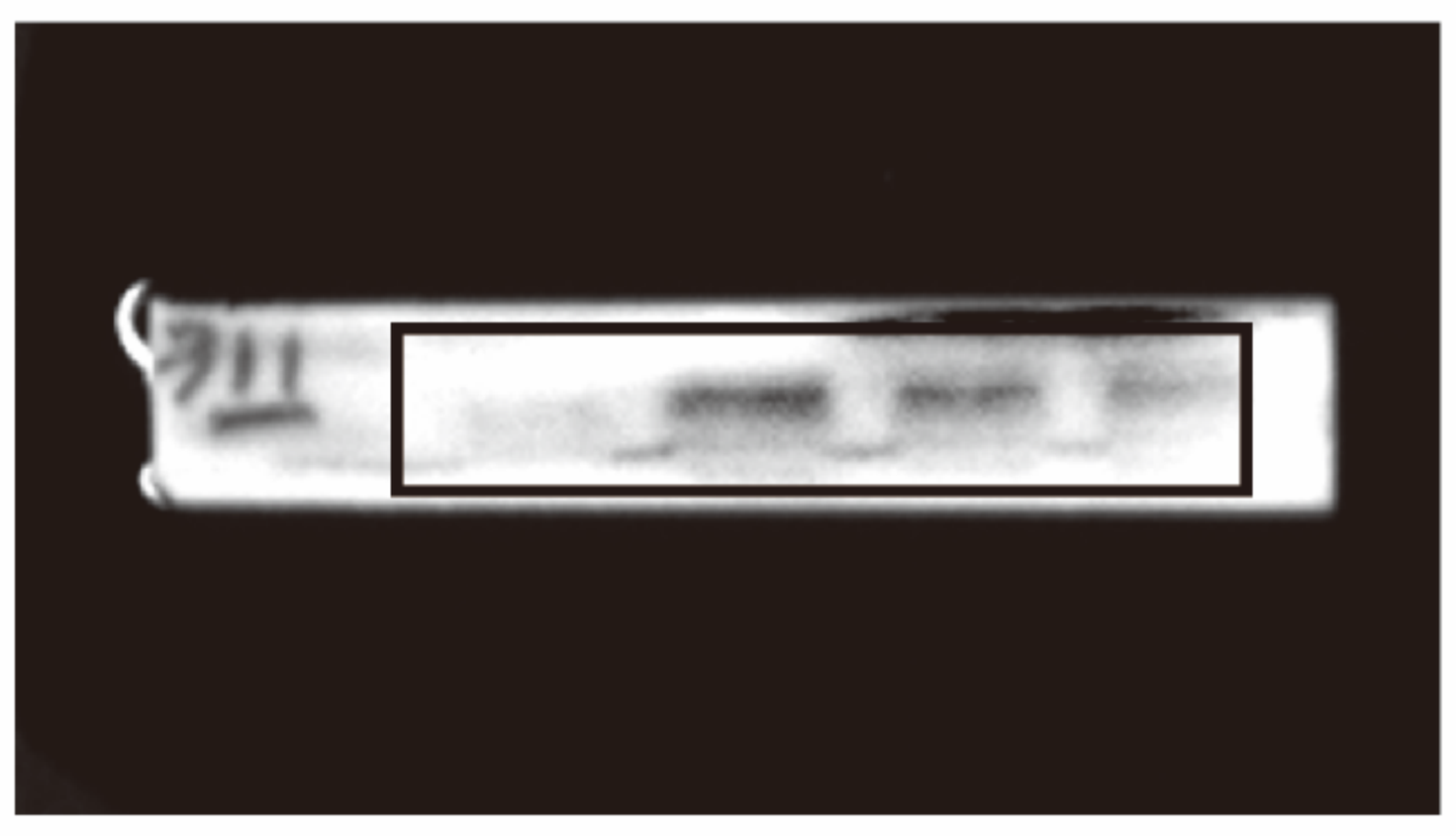

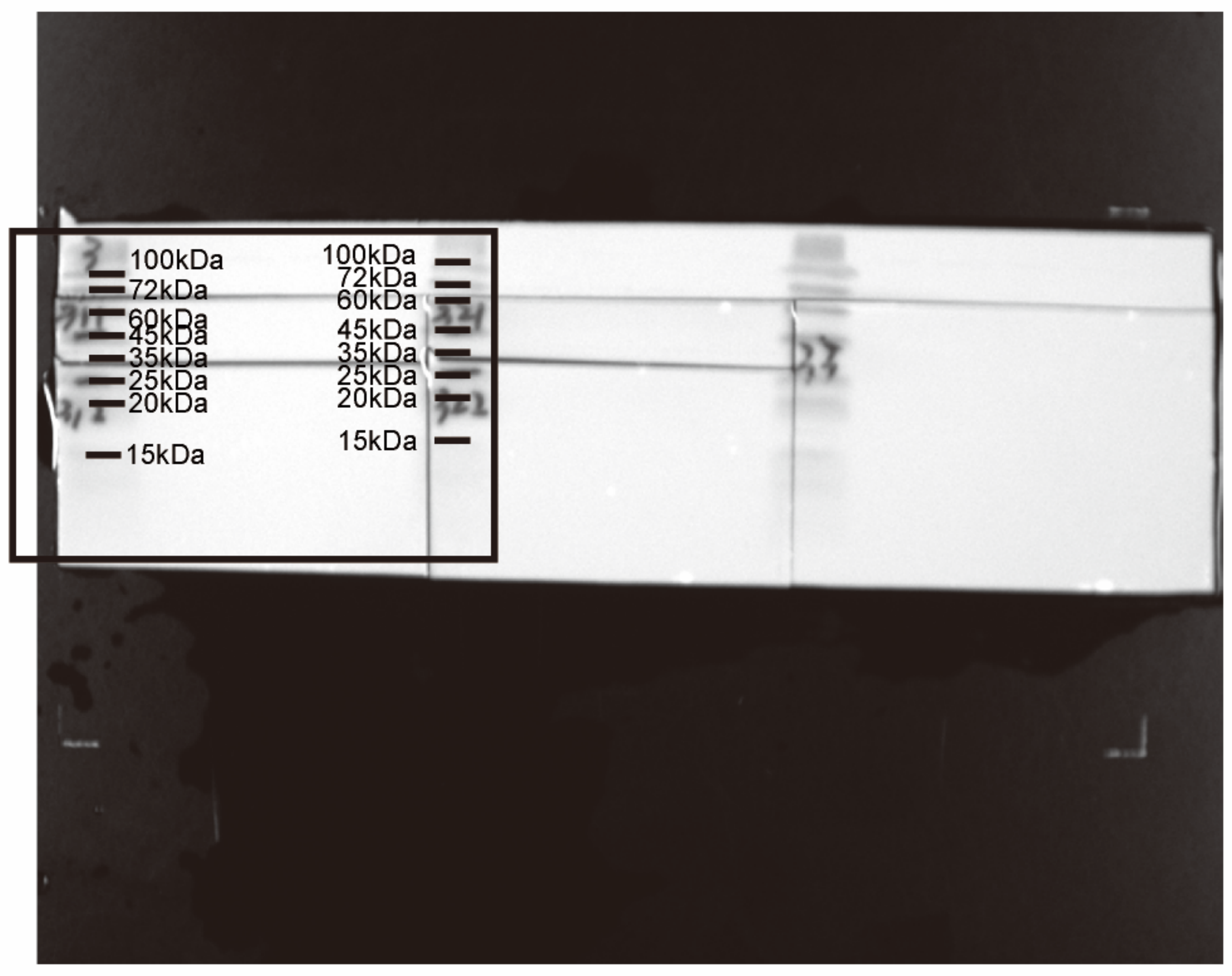

Fig.8K

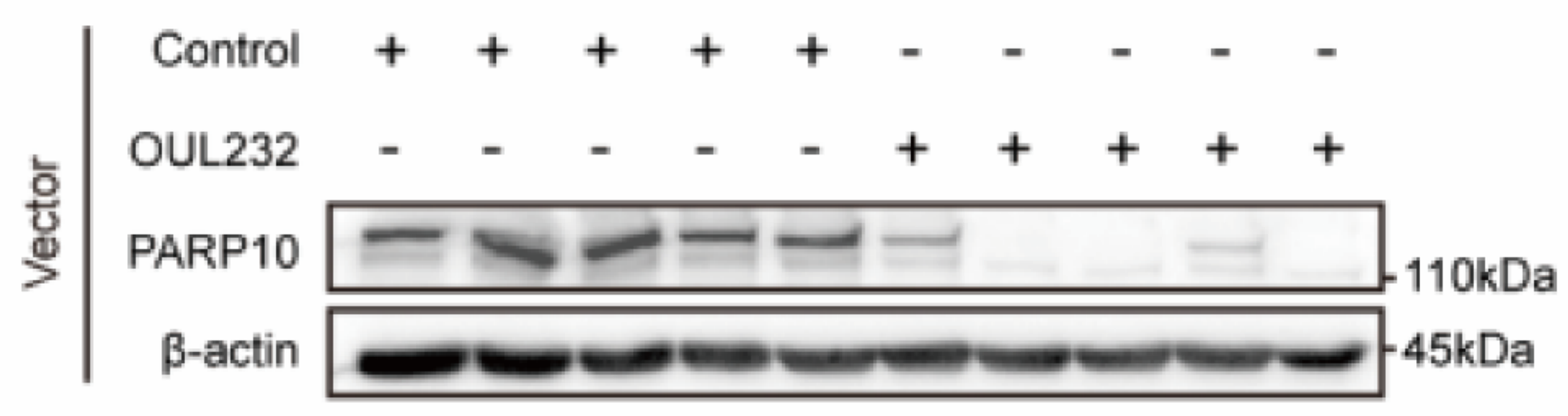

PARP10

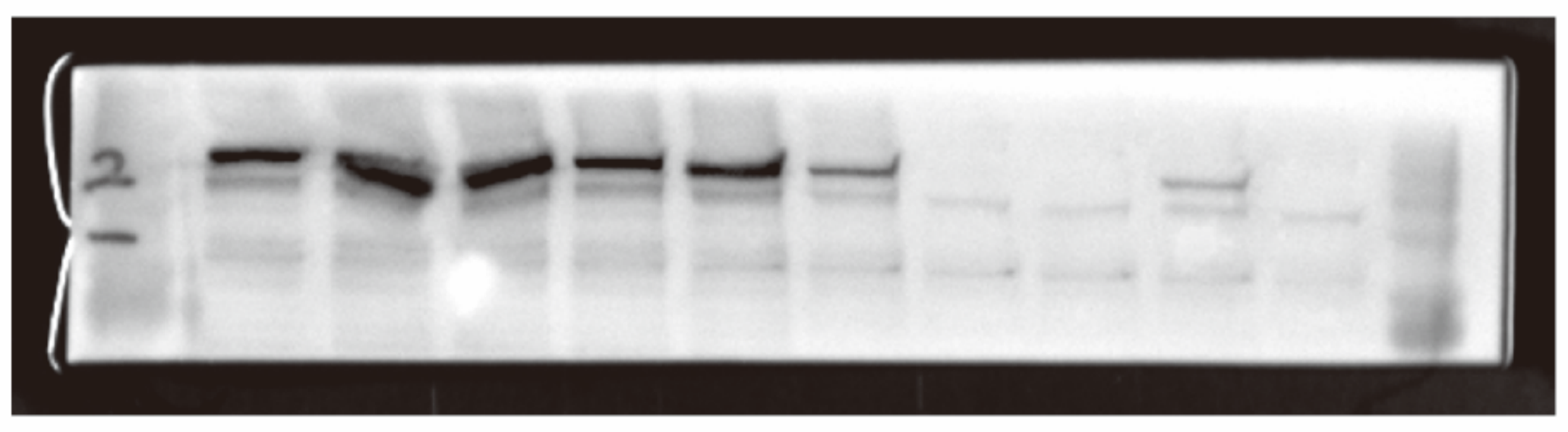

β-actin

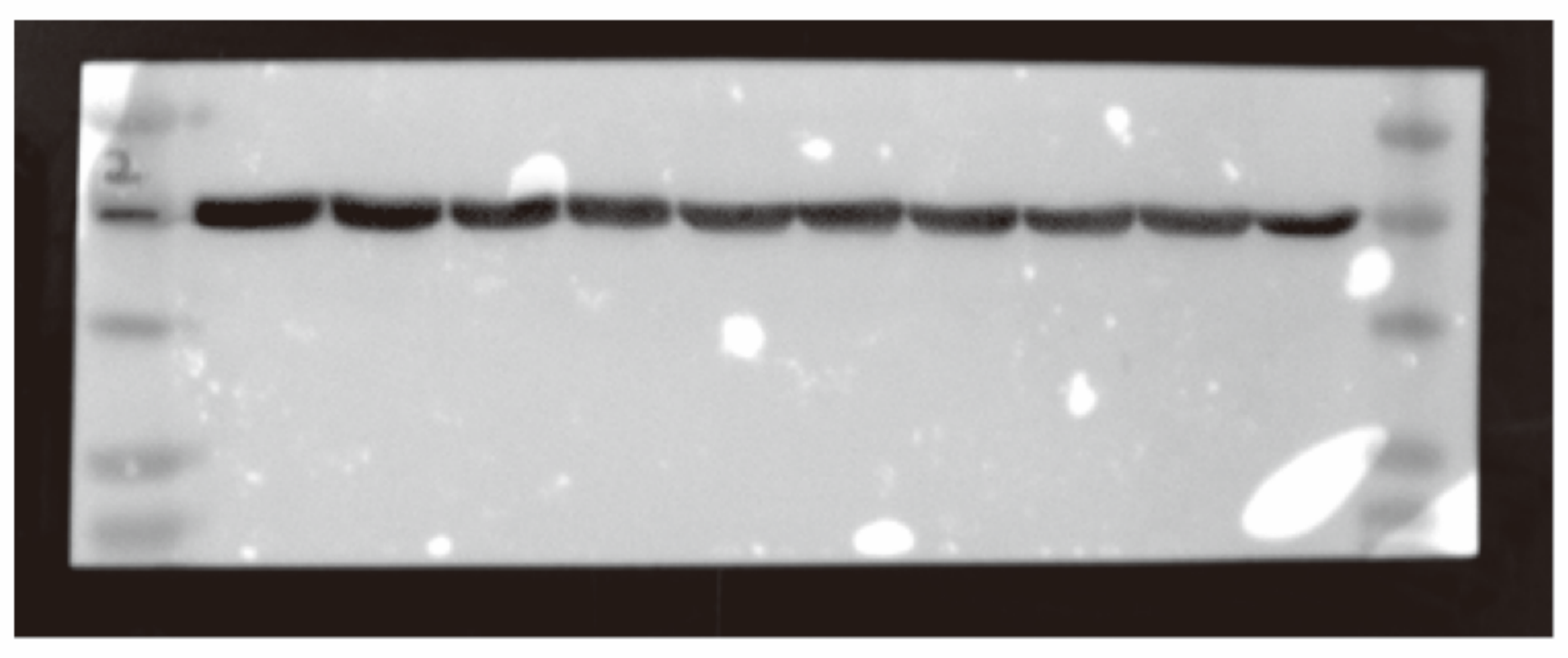

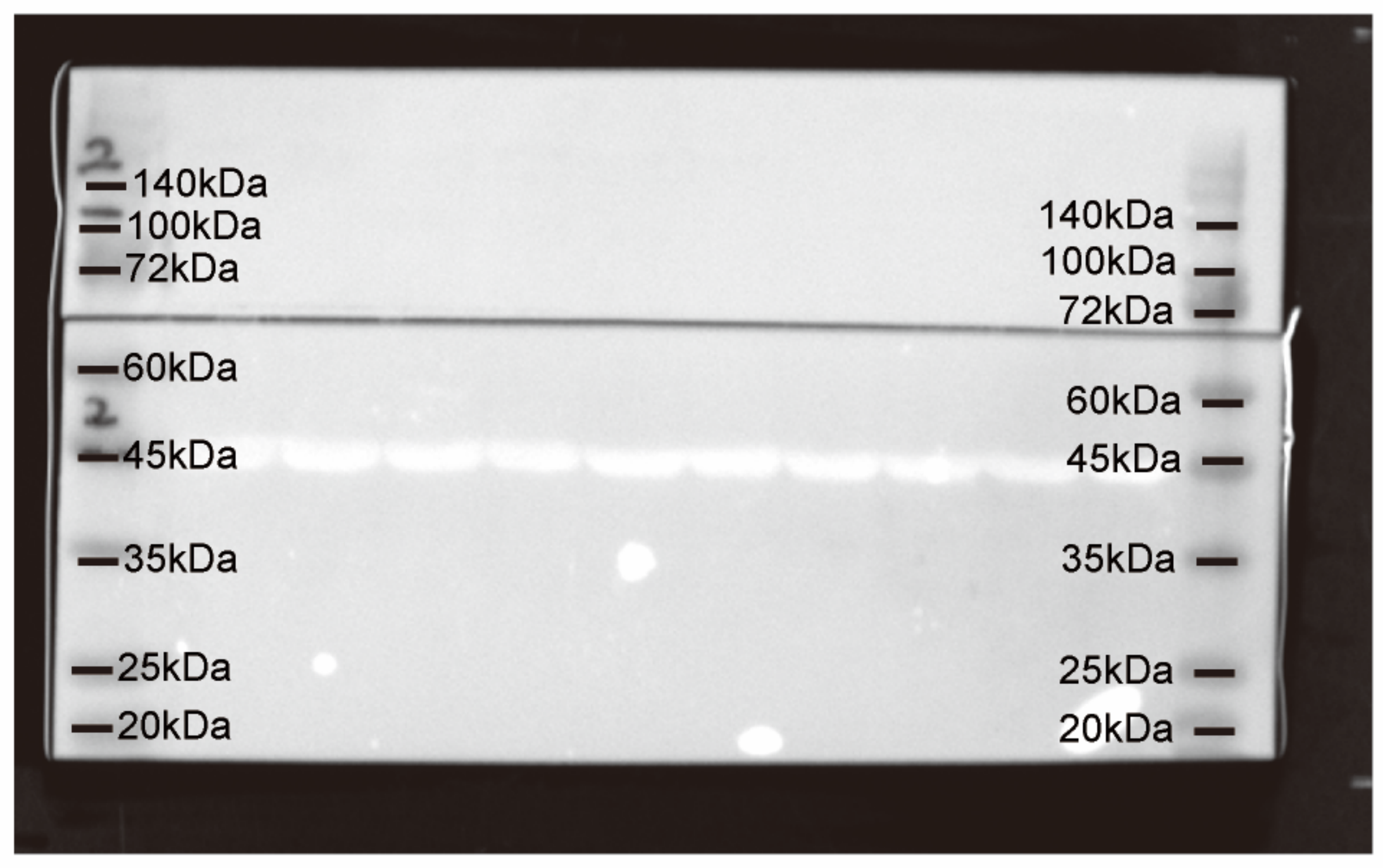

Fig.8K

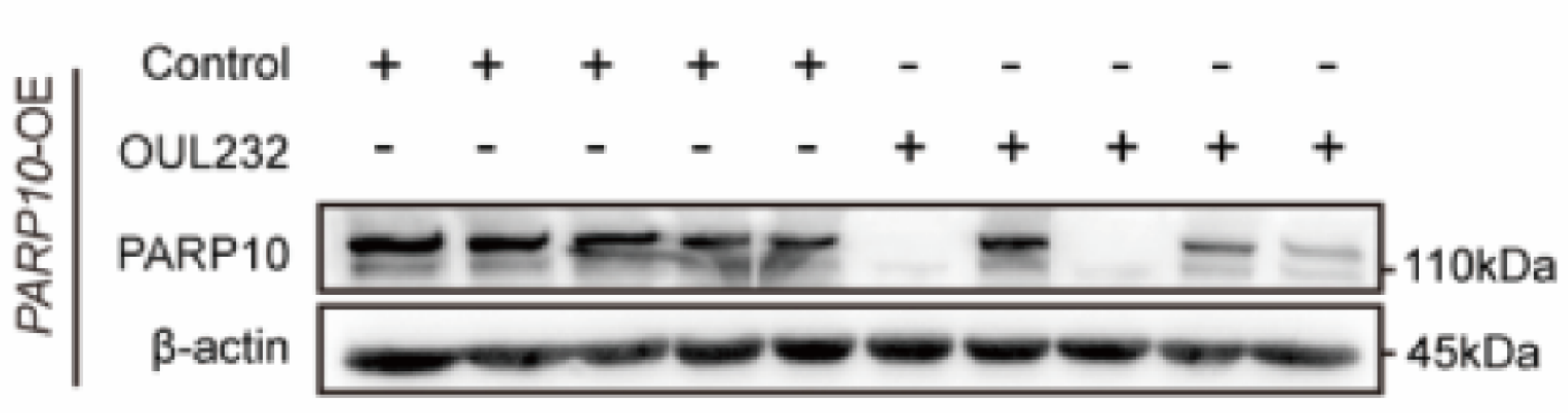

PARP10

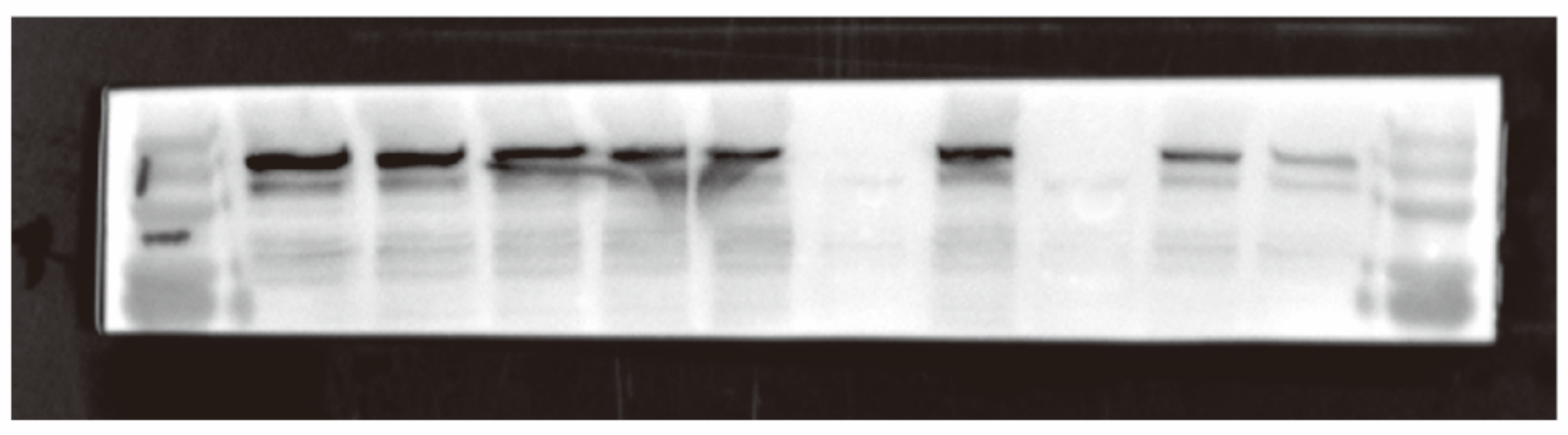

β-actin

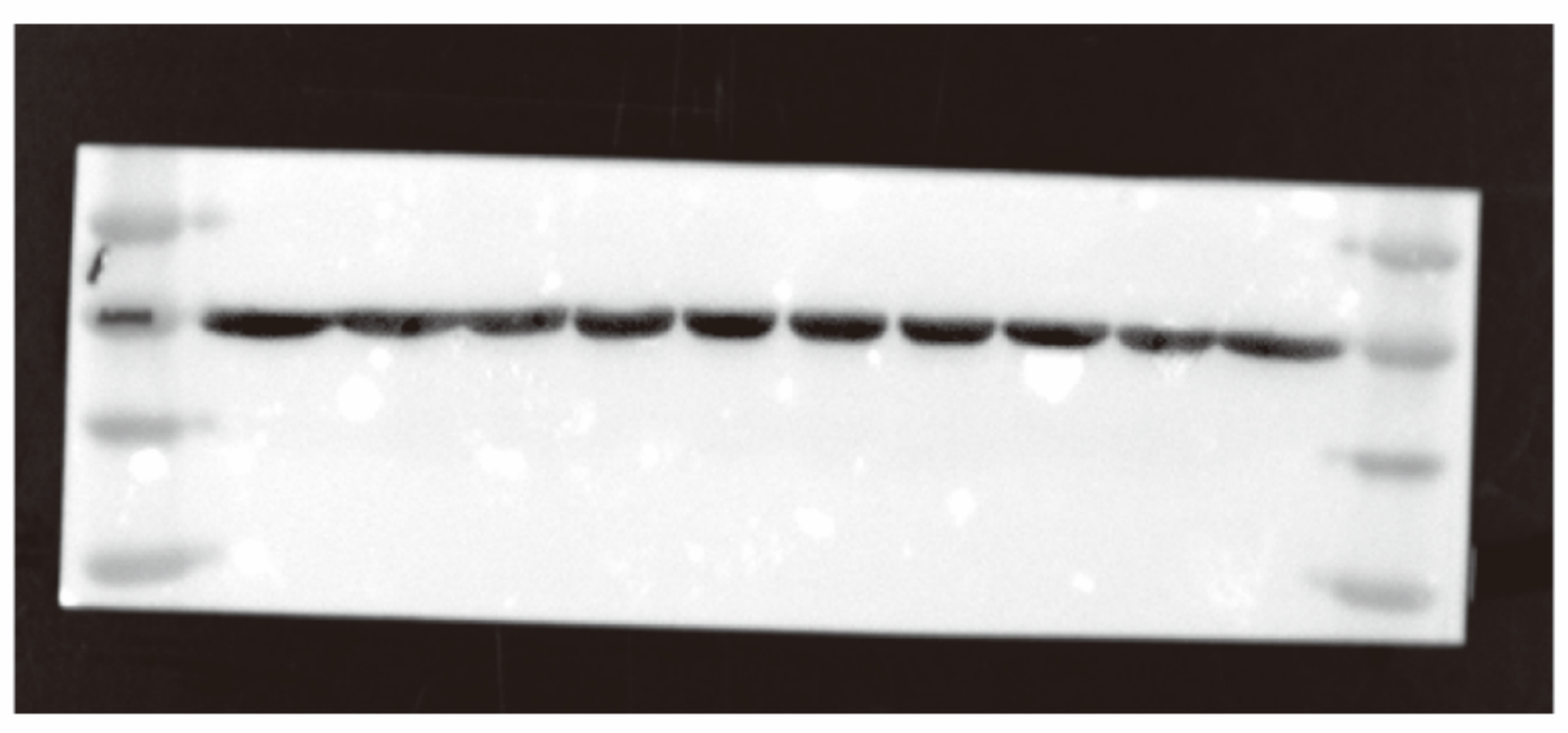

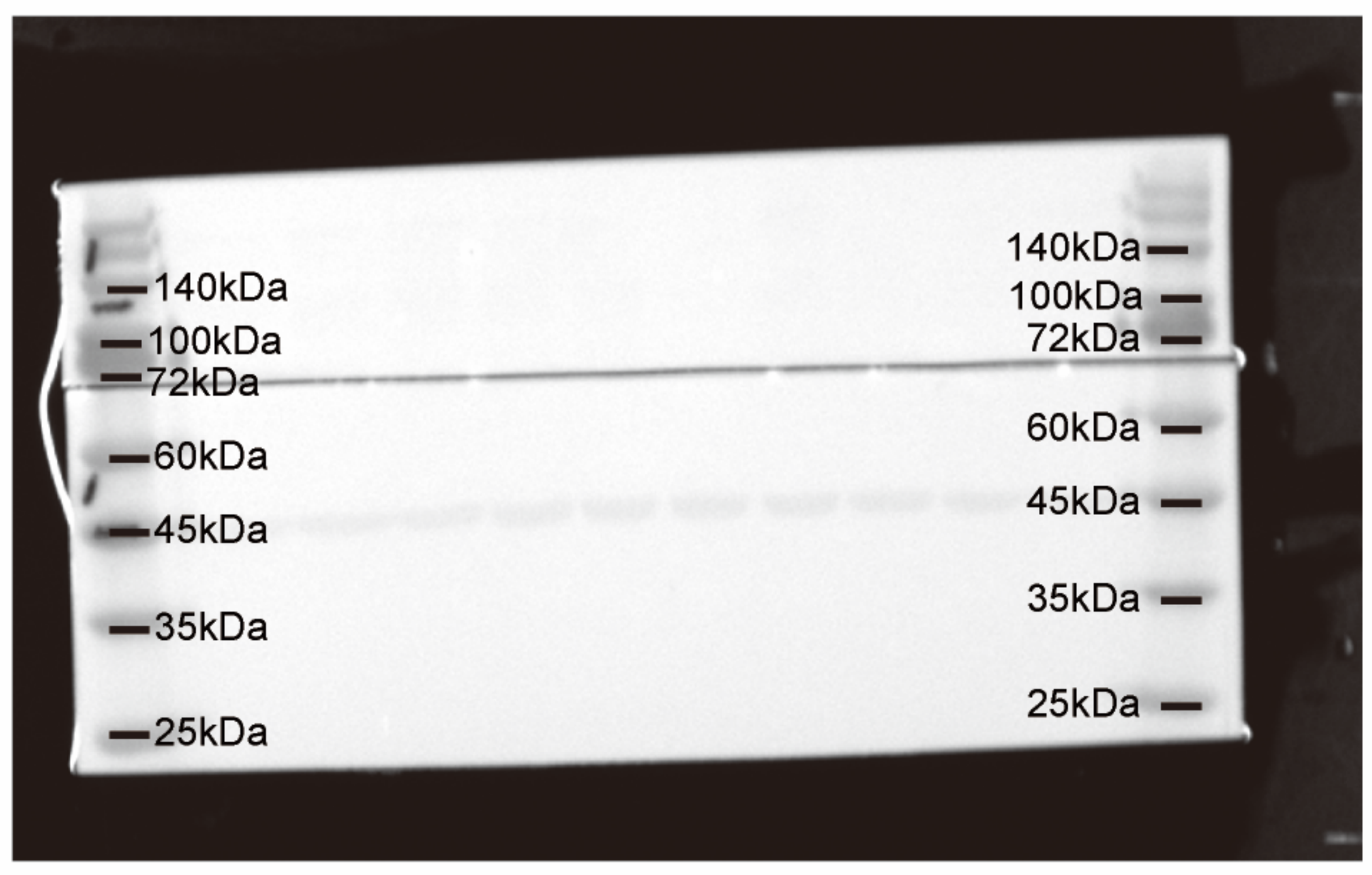

Supplement: Supplementary file 1 [file 41419_2026_8989_MOESM2_ESM.pdf]
