# Supplementary material for: Targeting the PARP10-BCAT2 axis disrupts branched-chain amino-acid metabolism to suppress bone metastasis in lung cancer
Source: Cell Death Dis. 2026 Jun 19;17(1):763. doi: 10.1038/s41419-026-08989-3 (PMC13526790; doi:10.1038/s41419-026-08989-3)
Supplement: Supplementary file 2 [file 41419_2026_8989_MOESM1_ESM.docx]

Supplementary Information

**Targeting the *PARP10*-*BCAT2* Axis Disrupts Branched-Chain Amino Acid Metabolism to Suppress Bone Metastasis in Lung Cancer**

*Yahan Qin, Ke Xue, Yujian Xu, Hongyu Pan, Wenjun Chai, Lei Sun, Xiaoli Liu, Jing Li, Yue Cao, Jing Li, Qian Liu*, Mingxia Yan**

**Supplementary Information includes:**

Fig. S1-S6

Table S1-S4

**Fig. S1. Effects of conditioned medium from *PARP10*-knockdown tumor cells on osteogenic differentiation and osteoblast recruitment**

A. Recruitment of MC3T3 cells and bone mesenchymal stem cells (BMSCs) by conditioned medium from *PARP10*-knockdown BM5 and PC9 cells. n=9–13 random fields per group. Scale bar, 200 μm.
B. ALP staining of MC3T3 cells after 7 days of culture in conditioned medium. ALP-positive area was quantified using ImageJ software. n=9–13 random fields per group.

**C-D.** Alizarin Red S staining of MC3T3 cells following 21 days of culture in conditioned medium **(C).** Quantification of Alizarin Red S-positive area using ImageJ software **(D).** Scale bar, 200 μm. n=9 random fields per independent experiment.

**E.** Western blotting analysis of early and late osteogenic differentiation marker proteins.

Data are presented as mean ± SD. Statistical significance was determined by one-way ANOVA with Dunnett's post-hoc test for comparisons to the control group, or two-way ANOVA with Tukey's post-hoc test for multi-factor grouped data.

**Fig. S2. *PARP10* knockdown enhances tumor cell chemotaxis toward branched-chain amino acids (Related to Figure 4)**

A. Transwell migration assay with supplementation of 400 μM individual leucine (Leu), isoleucine (Ile), or valine (Val). n=9–13 random fields per group. Scale bar, 200 μm.
B. Wound healing assays were performed in *PARP10*-knockdown BM5 and PC9 cells, as well as *PARP10*-overexpressing A549 cells, with or without 400 μM BCAA supplementation in the culture medium. Wound closure was detected at 0 and 24 h post-wounding, and the migratory area was quantified using ImageJ software and presented as the percentage of wound closure. n=5 independent experiments. Scale bar, 500 μm.

**C.** Wound healing assay treated with 400 μM individual leucine (Leu), isoleucine (Ile), or valine (Val). n=5 independent experiments. Scale bar, 200 μm.

Data are presented as mean ± SD. Statistical significance was determined by one-way ANOVA with Dunnett's post-hoc test for comparisons to the control group, or unpaired two-tailed Student's t-test for comparisons between two groups. *P < 0.05, **P < 0.01, ***P < 0.001, ****P < 0.0001.

**Fig. S3. Transcriptome sequencing analysis following *PARP10* knockdown in lung adenocarcinoma cells**

A-**D.** Functional enrichment analysis of differentially expressed genes (DEGs) in *PARP10*-knockdown LUAD cells based on RNA-seq data. **(A)** KEGG pathway enrichment analysis of all DEGs. **(B)** Separate KEGG enrichment analysis of upregulated and downregulated DEGs. **(C)** WikiPathways enrichment analysis of downregulated DEGs. **(D)** WikiPathways enrichment analysis of upregulated DEGs. FDR < 0.05 was set as the significance threshold for all enrichment analyses.
E-**H.** Gene set enrichment analysis (GSEA) in *PARP10*-knockdown BM5 cells. Transcriptomic profiling was performed by RNA-seq in PARP10-knockdown BM5 cells. Enrichment plots of representative pathways derived from the WikiPathways database are shown. **(E)** Cell cycle (KEGG: HSA04110). **(F)** Homologous DNA pairing and strand exchange (Reactome: R-HSA-5693579). **(G)** Mitotic G1 phase and G1/S transition (Reactome: R-HSA-453279). **(H)** DNA replication (KEGG: HSA03030). Normalized enrichment score (NES) and false discovery rate (FDR) are annotated for each pathway.

**Fig. S4. Tumor cells upregulate BCAT2 to enhance BCAA metabolism and rescue cells from mitochondrial apoptosis**

A. RT-qPCR detection of mRNA levels of BCAA catabolic genes in *PARP10*-knockdown BM5 cells. n=3 independent experiments.
B-**D.** Glycolysis stress test. Real-time extracellular acidification rate (ECAR) was measured in *PARP10*-knockdown or *PARP10*-overexpressing BM5 **(B)**, PC9 **(C),** and A549 **(D)** cells using a Seahorse XF Analyzer. Glucose (10 mM), oligomycin (10 μM), and 2-deoxyglucose (2-DG, 50 mM) were sequentially injected at the indicated time points. Glycolysis level, glycolytic capacity, and glycolytic reserve were calculated accordingly. n=3 independent experiments.
E. Western blotting analysis of mitochondrial apoptosis-related proteins in *BCAT2*-knockdown or *PARP10*-overexpressing BM5, PC9 and A549 cells.

Data are presented as mean ± SD. Statistical significance was determined by one-way ANOVA with Dunnett's post-hoc test for comparisons to the control group, or unpaired two-tailed Student's t-test for comparisons between two groups. *P < 0.05, **P < 0.01, ***P < 0.001, ****P < 0.0001.

**Fig. S5. PARP10 inhibitor OUL232 suppresses lung adenocarcinoma metastasis in vitro (Related to Figure 8)**

A. DMSO vehicle control groups with corresponding doses in OUL232 IC₅₀ assays of PC9, A549, BM5, *PARP10*-overexpressing and *PARP10*-knockdown cells. n=3 independent experiments.
B. IC₅₀ values of OUL232 after 48 h treatment in PC9 and BM5 cells, as well as *PARP10*-overexpressing and *PARP10*-knockdown cells. n=3 independent experiments.
C-**D.** Transwell migration **(C)** and Matrigel invasion **(D)** assays in A549, BM5, and PC9 cells treated with 20 μM OUL232. n=9–13 random fields per group. Scale bar, 200 μm.

**E.** Cell cycle analysis of A549, BM5 and PC9 cells after treatment with 20 μM OUL232 or DMSO vehicle control for 24 h. n=3 independent experiments.

**F.** Comet assay assessing DNA damage (quantified by tail DNA percentage) in A549, BM5 and PC9 cells after 1 h treatment with 200 μM OUL232 or DMSO. n=50 comets per group. Scale bar, 100 μm.

**G.** Mitochondrial membrane potential (ΔΨm) detected by JC-1 staining after 24 h treatment with 20 μM OUL232. JC-1 aggregates (red fluorescence) indicate high ΔΨm. The green fluorescence of JC-1 monomers was not used to assess membrane depolarization in GFP-expressing BM5 cells to avoid interference from endogenous GFP signals. Quantification of mean JC-1 aggregate fluorescence intensity is shown. n=5 independent experiments. Scale bar, 100 μm.

**H.** IC₅₀ values of paclitaxel (PTX) and camptothecin (CPT) after 24 h treatment in PC9 and BM5 cells. n=3 independent experiments.

**I.** Comet assay assessing DNA damage (quantified by tail DNA percentage) in BM5 and PC9 cells after 1 h treatment with 50 μM PTX or 25 μM CPT. n=50 comets per group. Scale bar, 100 μm.

**J.** BCAA concentrations in cell culture supernatant after 24 h treatment with 1 nM PTX or 0.5 nM CPT. n=3 independent experiments.

Data are presented as mean ± SD. Statistical significance was determined by unpaired two-tailed Student's t-test for comparisons between two groups. *P < 0.05, **P < 0.01, ***P < 0.001, ****P < 0.0001.

**Fig. S6. Histopathological assessment of OUL232 systemic toxicity in the LUAD bone metastasis model**

A. Schematic of the OUL232 administration regimen in mice. A bone metastasis model was established by intracardiac injection of tumor cells. Mice were treated with OUL232 via intraperitoneal injection every other day for 4 weeks. Bone metastasis was assessed post-treatment. Q2D (Quaque 2 dies, once every two days). 4w ( 4-week time point).
B. H&E staining showing organ damage in the heart, liver, spleen, lungs, left kidney, right kidney, and intestine of mice following intracardiac injection of *PARP10*-overexpressing A549 cells and subsequent 4-week treatment with OUL232 via intraperitoneal injection. Control mice received an equivalent volume of saline via intraperitoneal injection. Scale bar, 40 µm. The schematic diagram was created with BioRender.com.

|  | **Table S1:The relative expression of *PARP10* in tumor tissues compared with adjacent normal tissues across different pathological characteristics in 72 paired samples** | | | | | | | | | | |
| --- | --- | --- | --- | --- | --- | --- | --- | --- | --- | --- | --- |
|  | Clinicopathologic Parameters | | | Number of Paired Cases | | | Meadian of Differences | | | | |
|  |  |  |  |  |  |  | Meadian | | *p* Value | | |
|  | **Age(years)** | | |  | | |  | |  | | |
|  | ＜54 | | | 13 | | | 0.007642 | | 0.0002 | | |
|  | 55-64 | | | 25 | | | 0.0001642 | | 0.3525 | | |
|  | 65-74 | | | 21 | | | 0.0003868 | | 0.0701 | | |
|  | ＞75 | | | 13 | | | 0.0002663 | | 0.1909 | | |
|  | **Gender** | | |  | | |  | |  | | |
|  | Female | | | 28 | | | 0.0003732 | | 0.0048 | | |
|  | Male | | | 44 | | | 0.0003046 | | 0.0294 | | |
|  | **Histological type** | | |  | | |  | |  | | |
|  | Adenocarcinoma (LUAD) | | | 46 | | | 0.0004503 | | 0.0012 | | |
|  | Squamous cell carcinoma (LUSC) | | | 21 | | | 0.0001579 | | 0.412 | | |
|  | Adenosquamous carcinoma (LUAS) | | | 3 | | |  | |  | | |
|  | Small Cell Lung Cancer (SCLC) | | | 1 | | |  | |  | | |
|  | Large Cell Carcinoma | | | 1 | | |  | |  | | |
|  | **TNM Stage** | | |  | | |  | |  | | |
|  | Ⅰ | | | 40 | | | 0.0002911 | | 0.0918 | | |
|  | Ⅱ | | | 13 | | | 0.0009057 | | 0.0105 | | |
|  | Ⅲ | | | 14 | | | 0.001111 | | 0.0009 | | |
|  | Ⅴ | | | 3 | | |  | |  | | |
|  | Unknown | | | 2 | | |  | |  | | |
|  | **Maximum tumor diameter (cm)** | | |  | | |  | |  | | |
|  | ≤4 | | | 54 | | | 0.0004058 | | 0.0033 | | |
|  | ＞4 | | | 16 | | | 0.0002491 | | 0.025 | | |
|  | Unknown | | | 2 | | |  | |  | | |
|  | **Metastasis** | | |  | | |  | |  | | |
|  | No | | | 35 | | | 0.0002984 | | 0.0535 | | |
|  | Yes | | | 37 | | | 0.0005098 | | 0.0025 | | |
|  | **Tumor Grade** | | |  | | |  | |  | | |
|  | G1 (Well differentiated) | | | 3 | | | 0.001933 | | 0.25 | | |
|  | G2 (Moderately differentiated) | | | 41 | | | 0.0009057 | | 0.0003 | | |
|  | G3 (Poorly differentiated) | | | 26 | | | 0.0001949 | | 0.4374 | | |
|  | Unknown | | | 2 | | |  | |  | | |
|  | | **Table S2: Primer sequences used in this study** | | | | | | | | |  |
|  | | Gene | Main Accession No. | | Primer Sequence (5' → 3') | | | | | |  |
|  | | *PARP10* | NM_032789.5 | | Forward TCCCAGCAGGGTTAGGCTT | | | | | |  |
|  | |  |  | | Reverse CGCCATTGCAACCATTCCC | | | | | |  |
|  | | BCAT1 | NM_005504.7 | | Forward GGCAACTCTGCCGGTATTTG | | | | | |  |
|  | |  |  | | Reverse CAGTTCCACCTTTCCAGGCT | | | | | |  |
|  | | *BCAT2* | NM_001190.4 | | Forward CACTACTCCCTGCAGCTGTT | | | | | |  |
|  | |  |  | | Reverse TCCAGCAACTCCAGCTTGTC | | | | | |  |
|  | | MCCC1 | NM_020166.5 | | Forward GCTGCACAGGCTATCCATCC | | | | | |  |
|  | |  |  | | Reverse CACCATGATAACCCTCCACAAC | | | | | |  |
|  | | MLYCD | NM_012213.3 | | Forward CAGCAACATCCAGGCAATCG | | | | | |  |
|  | |  |  | | Reverse CAACTCCTTGACGACTCGCT | | | | | |  |
|  | | BCKDHB | NM_183050.4 | | Forward GCCCAGGAATCAAGGTGGTT | | | | | |  |
|  | |  |  | | Reverse GCCATGGAAGCTACCTCTCG | | | | | |  |
|  | | ALDH6A1 | NM_005589.4 | | Forward CAGGTCTTGCTCCGCTATCA | | | | | |  |
|  | |  |  | | Reverse GCATGCTCAACCACCTGAAG | | | | | |  |
|  | | MMUT | NM_000255.4 | | Forward AACAGGCCTACCAGTCAGTTC | | | | | |  |
|  | |  |  | | Reverse GGGCTGTTGCTGGTGTAGAA | | | | | |  |
|  | | HMGCLL1 | NM_019036.3 | | Forward TTTCAGCCCGATGTCTCTGG | | | | | |  |
|  | |  |  | | Reverse GTGTGATCAGCCATCTGTGG | | | | | |  |
|  | | AUH | NM_001698.3 | | Forward GGCTACAGCTCTGAGATGAAGA | | | | | |  |
|  | |  |  | | Reverse GCACCACAATTCCTCGGTTCT | | | | | |  |
|  | | ACADSB | NM_001609.4 | | Forward GATGGCAAATGTAGACCCTACC | | | | | |  |
|  | |  |  | | Reverse AAGGCCCGGAGTATCACGA | | | | | |  |
|  | | HIBCH | NM_014362.4 | | Forward GGTTGCACGGGAGTCATAACA | | | | | |  |
|  | |  |  | | Reverse CTTTTCAGCTTCCGAGATCACT | | | | | |  |
|  | | PCCA | NM_000282.4 | | Forward GCAAGAAGATGGGCATTAAGACA | | | | | |  |
|  | |  |  | | Reverse GCCAACACAGACAGCCTCAT | | | | | |  |
|  | | MCCC2 | NM_022132.5 | | Forward ACAGATCGCAGTGGTCATGG | | | | | |  |
|  | |  |  | | Reverse GTTGCCGCTTTAACCAAGGG | | | | | |  |
|  | | ACAT1 | NM_000019.4 | | Forward AAGGCAGGCAGTATTGGGTG | | | | | |  |
|  | |  |  | | Reverse ACATCAGTTAGCCCGTCTTTTAC | | | | | |  |
|  | | IVD | NM_002225.5 | | Forward CAGGAGATCGATCGCAGCAA | | | | | |  |
|  | |  |  | | Reverse TCACCACTGATCAGCTTCGG | | | | | |  |
|  | | ACAD8 | NM_014384.3 | | Forward ATCGACCCTTCCATGGGACT | | | | | |  |
|  | |  |  | | Reverse CTGGGAACAGCTCCTTCTGG | | | | | |  |
|  | | DLD | NM_000108.5 | | Forward GGACTTTCTGCAGTGCCTCT | | | | | |  |
|  | |  |  | | Reverse CAATGCAGACTGTCTTGAAGC | | | | | |  |
|  | | DBT | NM_001918.5 | | Forward GGGAAGCTGATTTGTGTTCGC | | | | | |  |
|  | |  |  | | Reverse TCCACGGAGAGCAGCAGTT | | | | | |  |
|  | | PCCB | NM_000532.5 | | Forward CCACCTCTGTTAACGAACGC | | | | | |  |
|  | |  |  | | Reverse GGCTGTTAGCTTTCCTCGCT | | | | | |  |
|  | | HIBADH | NM_152740.4 | | Forward GCTTTGCAGCGGTGTGTTCT | | | | | |  |
|  | |  |  | | Reverse ATTGATACTGGTGGGCAGCAT | | | | | |  |
|  | | BCKDHA | NM_000709.4 | | Forward CTACAAGAGCATGACACTGCTT | | | | | |  |
|  | |  |  | | Reverse CCCTCCTCACCATAGTTGGTC | | | | | |  |
|  | | ACSF3 | NM_174917.5 | | Forward ACACAGAGGAAGTGGTCTTCT | | | | | |  |
|  | |  |  | | Reverse CGTTAGCGCATAGGAAGGAGA | | | | | |  |
|  | | HSD17B10 | NM_004493.3 | | Forward CTTCGAGGGTCAGGTTGGAC | | | | | |  |
|  | |  |  | | Reverse TGGTCATCACCCGGATACCT | | | | | |  |
|  | | ECHS1 | NM_004092.4 | | Forward TGAGCTTGCCATGATGTGTGA | | | | | |  |
|  | |  |  | | Reverse AACAGGACAAATCTTGCTGACA | | | | | |  |
|  | | ACTB | NM_001101.5 | | Forward GTCATTCCAAATATGAGATGCGT | | | | | |  |
|  | |  |  | | Reverse GCATTACATAATTTACACGAAAGCA | | | | | |  |
|  | | PARP1 | NM_001618.1 | | Forward CCAAGCCAGTTCAGGACCTCAT | | | | | |  |
|  | |  |  | | Reverse GGATCTGCCTTTTGCTCAGCTTC | | | | | |  |
|  | | PARP2 | NM_005484.2 | | Forward GGTGGCTTGTTCAGGCAATCTC | | | | | |  |
|  | |  |  | | Reverse GGTGGCATAGTCCATCTGTAGC | | | | | |  |
|  | | PARP3 | NM_005485.1 | | Forward TCTCTGAGCAGGAGAAGACGGT | | | | | |  |
|  | |  |  | | Reverse TGTGGTTGCTGCCAGTCTGTTC | | | | | |  |
|  | | PARP4 | NM_006437.1 | | Forward CATGGCGCTTACCTGATGAGTC | | | | | |  |
|  | |  |  | | Reverse AACAGTGCCCAGGATGCTGAGT | | | | | |  |
|  | | PARP6 | NM_020214.1 | | Forward GCCTATGGCAAAGGCATCTACC | | | | | |  |
|  | |  |  | | Reverse TCTCTGGACCAGCTCATCCTTG | | | | | |  |
|  | | PARP8 | NM_024615.1 | | Forward ACCGTATGTGAACGGGAGCTGT | | | | | |  |
|  | |  |  | | Reverse TCCAACGCAGACCTACACATGG | | | | | |  |
|  | | PARP9 | NM_031458.1 | | Forward GGCAAAGAGGTCCAAGATGCTG | | | | | |  |
|  | |  |  | | Reverse GCCTCACACATCTCTTCCACGT | | | | | |  |
|  | | PARP11 | NM_020367.1 | | Forward GAATCTCACCACTGGAAAGCAGC | | | | | |  |
|  | |  |  | | Reverse CATTCTCCCAGTGTGGTGGCAT | | | | | |  |
|  | | PARP12 | NM_022750.2 | | Forward CTCTGTCACCAAACCTCCACAC | | | | | |  |
|  | |  |  | | Reverse GCTACTGCTGACAGTGGTCACA | | | | | |  |
|  | | PARP14 | NM_017554.1 | | Forward GACTGTCGCTATGTGCTTCACG | | | | | |  |
|  | |  |  | | Reverse GGACAAGCTCTCAGTGATCTCC | | | | | |  |
|  | | PARP15 | NM_152615.1 | | Forward GACAGATGCAGACTCAGTGCCA | | | | | |  |
|  | |  |  | | Reverse TGGAGTAGGTGTCCTTGGCAGA | | | | | |  |
|  | | PARP16 | NM_017851.1 | | Forward GCATTTCATGGTAGCCGCCTAG | | | | | |  |
|  | |  |  | | Reverse CAAGTCACTGGTGAGGTAGGTC | | | | | |  |
|  | | MYC | NM_002467.6 | | Forward CCTGGTGCTCCATGAGGAGAC | | | | | |  |
|  | |  |  | | Reverse CAGACTCTGACCTTTTGCCAGG | | | | | |  |
|  | | ChIP primer | - | | Forward CACAAGTTGCCTCGACCCTG | | | | | |  |
|  | |  |  | | Reverse TCCATTACCTCCTAATTAG | | | | | |  |
|  | | **Table S3: Target sequences of siRNAs used in this study** | | | | | | | | |  |
|  | | Target Gene | shRNA Name | | Target Sequence (5' → 3') | | | | | |  |
|  | | Control | shNC | | TTCTCCGAACGTGTCACGT | | | | | |  |
|  | | BCAT1 | siBCAT1-1 | | GCTCATCAGCTTTGCACTA | | | | | |  |
|  | | BCAT1 | siBCAT1-2 | | GGACCATCAGATCACTGAA | | | | | |  |
|  | | BCAT1 | siBCAT1-3 | | GTATGTAAGAGCCTGGAAA | | | | | |  |
|  | | *BCAT2* | si*BCAT2*-1 | | GGTCGGCAACTACAAGTTA | | | | | |  |
|  | | *BCAT2* | si*BCAT2*-2 | | GGCTCATCGAAGTGGACAA | | | | | |  |
|  | | *BCAT2* | si*BCAT2*-3 | | CGAATCCTGTACAAAGACA | | | | | |  |
|  | | *PARP10* | si*PARP10*-1 | | GGTAGAGGGATTATGACAA | | | | | |  |
|  | | *PARP10* | si*PARP10*-2 | | CTACCATGAGGACCTTCTT | | | | | |  |
|  | | *MYC* | si*MYC*_1 | | GAGGAGACATGGTGAACCA | | | | | |  |
|  | | *MYC* | si*MYC*_2 | | GGGTCAAGTTGGACAGTGT | | | | | |  |
|  | | *MYC* | si*MYC*_3 | | CGACGAGACCTTCATCAAA | | | | | |  |
| **Table S4: Antibodies used in this study** | | | | | | | | | |  |  |
| Antibodies | | | | | | SOURCE | | IDENTIFIER | |  |  |
| Bad (D24A9) Rabbit mAb | | | | | | Cell Signaling Technology | | 9239 | |  |  |
| Phospho-Bad (Ser112) (40A9) Rabbit mAb | | | | | | Cell Signaling Technology | | 5284 | |  |  |
| Bax (D2E11) Rabbit mAb | | | | | | Cell Signaling Technology | | 5023 | |  |  |
| Bik Antibody | | | | | | Cell Signaling Technology | | 4592 | |  |  |
| Bim (C34C5) Rabbit mAb | | | | | | Cell Signaling Technology | | 2933 | |  |  |
| BID Antibody | | | | | | Cell Signaling Technology | | 2002 | |  |  |
| Bak (D4E4) Rabbit mAb | | | | | | Cell Signaling Technology | | 12105 | |  |  |
| Puma (D30C10) Rabbit mAb | | | | | | Cell Signaling Technology | | 12450 | |  |  |
| Caspase-3 (D3R6Y) Rabbit mAb | | | | | | Cell Signaling Technology | | 14220 | |  |  |
| Cleaved Caspase-3 (Asp175) (5A1E) Rabbit mAb | | | | | | Cell Signaling Technology | | 9664 | |  |  |
| Caspase-9 (C9) Mouse mAb | | | | | | Cell Signaling Technology | | 9508 | |  |  |
| Cleaved Caspase-9 (Asp330) (E5Z7N) Rabbit mAb | | | | | | Cell Signaling Technology | | 52873 | |  |  |
| Caspase-7 (D2Q3L) Rabbit mAb | | | | | | Cell Signaling Technology | | 12827 | |  |  |
| Cleaved Caspase-7 (Asp198) (D6H1) Rabbit mAb | | | | | | Cell Signaling Technology | | 8438 | |  |  |
| Anti-rabbit IgG, HRP-linked Antibody | | | | | | Cell Signaling Technology | | 7074 | |  |  |
| Anti-mouse IgG, HRP-linked Antibody | | | | | | Cell Signaling Technology | | 7076 | |  |  |
| Phospho-Bcl-2 (Ser70) (5H2) Rabbit mAb | | | | | | Cell Signaling Technology | | 2827 | |  |  |
| Phospho-Bcl-2 (Thr56) Antibody | | | | | | Cell Signaling Technology | | 2875 | |  |  |
| Bcl-2 (D55G8) Rabbit mAb | | | | | | Cell Signaling Technology | | 4223 | |  |  |
| Bcl-xL (54H6) Rabbit mAb | | | | | | Cell Signaling Technology | | 2764 | |  |  |
| Mcl-1 (D35A5) Rabbit mAb | | | | | | Cell Signaling Technology | | 5453 | |  |  |
| Phospho-Histone H2A.X (Ser139) (20E3) Rabbit Monoclonal Antibody | | | | | | Cell Signaling Technology | | 9718 | |  |  |
| BCKDH-E1 alpha (E4T3D) Rabbit Monoclonal Antibody | | | | | | Cell Signaling Technology | | 90198 | |  |  |
| Phospho-BCKDH-E1 alpha (Ser293) (E2V6B) Rabbit Monoclonal Antibody | | | | | | Cell Signaling Technology | | 40368 | |  |  |
| c-MYC Polyclonal antibody | | | | | | Proteintech | | 10828-1-AP | |  |  |
| COXIV Rabbit Polyclonal antibody | | | | | | Proteintech | | 11242-1-AP | |  |  |
| CYTB Rabbit Polyclonal antibody | | | | | | Proteintech | | 55090-1-AP | |  |  |
| OGDH Rabbit Polyclonal antibody | | | | | | Proteintech | | 15212-1-AP | |  |  |
| NDUFS6 Rabbit Polyclonal antibody | | | | | | Proteintech | | 14417-1-AP | |  |  |
| Vinculin Monoclonal antibody | | | | | | Proteintech | | 66305-1-lg | |  |  |
| HRP-conjugated Beta Actin Monoclonal antibody | | | | | | Proteintech | | HRP-66009 | |  |  |
| Anti-IDH1 Rabbit Polyclonal antibody | | | | | | BBI Life Sciences Corporation | | D221821 | |  |  |
| Anti-IDH2 Rabbit Polyclonal antibody | | | | | | BBI Life Sciences Corporation | | D222530 | |  |  |
| Anti-SDHB Rabbit Polyclonal antibody | | | | | | BBI Life Sciences Corporation | | D162175 | |  |  |
| Anti-SDHA Rabbit Polyclonal antibody | | | | | | BBI Life Sciences Corporation | | D223104 | |  |  |
| Anti-NDUFA9 Rabbit Polyclonal antibody | | | | | | BBI Life Sciences Corporation | | D122741 | |  |  |
| Anti-FH Rabbit Polyclonal antibody | | | | | | BBI Life Sciences Corporation | | D222390 | |  |  |
| Anti-ATP5A1 Rabbit Polyclonal antibody | | | | | | BBI Life Sciences Corporation | | D154243 | |  |  |
| *PARP10* Rabbit Polyclonal Antibody | | | | | | Thermo Fisher Scientific | | PA5-88774 | |  |  |
| Anti-Osteoprotegerin | | | | | | abcam | | ab183910 | |  |  |
| *BCAT2* Rabbit Polyclonal Antibody | | | | | | Biodragon | | BD-PB2940 | |  |  |
| ALP (16L9) Rabbit Monoclonal Antibody | | | | | | Biodragon | | RM9432 | |  |  |
| Osteopontin (10W13) Rabbit Monoclonal Antibody | | | | | | Biodragon | | RM4342 | |  |  |
| Collagen I alpha 1 (15S16) Rabbit Monoclonal Antibody | | | | | | Biodragon | | RM0325 | |  |  |
